# DNA binding protein identification by combining pseudo amino acid composition and profile-based protein representation

**DOI:** 10.1038/srep15479

**Published:** 2015-10-20

**Authors:** Bin Liu, Shanyi Wang, Xiaolong Wang

**Affiliations:** 1 School of Computer Science and Technology, Harbin Institute of Technology Shenzhen Graduate School, Shenzhen, Guangdong, China; 2 Key Laboratory of Network Oriented Intelligent Computation, Harbin Institute of Technology Shenzhen Graduate School, Shenzhen, Guangdong, China

## Abstract

DNA-binding proteins play an important role in most cellular processes. Therefore, it is necessary to develop an efficient predictor for identifying DNA-binding proteins only based on the sequence information of proteins. The bottleneck for constructing a useful predictor is to find suitable features capturing the characteristics of DNA binding proteins. We applied PseAAC to DNA binding protein identification, and PseAAC was further improved by incorporating the evolutionary information by using profile-based protein representation. Finally, Combined with Support Vector Machines (SVMs), a predictor called **iDNAPro-PseAAC** was proposed. Experimental results on an updated benchmark dataset showed that **iDNAPro-PseAAC** outperformed some state-of-the-art approaches, and it can achieve stable performance on an independent dataset. By using an ensemble learning approach to incorporate more negative samples (non-DNA binding proteins) in the training process, the performance of **iDNAPro-PseAAC** was further improved. The web server of **iDNAPro-PseAAC** is available at http://bioinformatics.hitsz.edu.cn/iDNAPro-PseAAC/.

DNA-binding proteins have diverse functions in the cell, and play vital roles in various cellular processes, such as gene regulation, DNA replication, and repair^1^. Identification of DNA-binding proteins is one of the most important tasks in the annotation of protein functions. In recent years, DNA-binding proteins can be identified by several experimental techniques, including filter binding assays^2^, X-ray crystallography^3^ and NMR^4^. However, it is time-consuming and expensive to identify DNA-binding proteins by experimental approaches. Facing the avalanche of new protein sequences generated in the post-genomic and big data age^5^,^6^, it is highly desired to develop automated methods for rapidly and effectively identifying DNA-binding proteins basing on the protein sequence information alone.

The computational methods for DNA binding protein identification can be grouped into two categories: (i) methods based on structures (ii) methods based on sequences. The first type makes use of both the structural and sequential information of target proteins (see, e.g.,^7^,^8^,^9^,^10^). Although these methods show promising predictive performance, the structural information of proteins is not always available, particularly for the huge amount of proteins, which prevents the application of these methods. In contrast, the second type methods overcome this shortcoming by only requiring the sequence information as input for the prediction^11^,^12^,^13^,^14^,^15^,^16^,^17^,^18^,^19^,^20^.

A key to improve the performance of the sequence-based methods is to find suitable feature extraction algorithms that can capture the characteristics of DNA binding proteins and non DNA binding proteins. Motivated by the successful application of Chou’s pseudo amino acid composition (PseAAC) to many important tasks in the field of computational proteomics, here we are to propose a new approach for DNA binding protein identification called **iDNAPro-PseAAC**, which extends the classic PseAAC approach by incorporating the evolutionary information in the form of profile-based protein representation^21^. The **iDNAPro-PseAAC** has the following advantages compared with other currently available approaches: (i) It is able to incorporate the global or long range sequence-order effects by means of PseAAC. (ii) The evolutionary information imbedded in the profile-based protein representation is employed by **iDNAPro-PseAAC**. (iii) It considers the various physical-chemical properties of amino acids.

To establish a really useful statistical predictor for a protein system, we need to consider the following procedures: (i) Construct or select a valid benchmark dataset to train and test the predictor. (ii) Formulate the protein samples with an effective mathematical expression that can truly reflect their intrinsic correlation with the attribute to be predicted. (iii) Introduce or develop a powerful algorithm (or engine) to operate the prediction. (iv) Perform properly cross-validation tests to objectively evaluate the anticipated accuracy of the predictor. Below, we are going to describe how to build the new predictor according to the four procedures.

## Results

### The influence of *λ* and *ω* on the performance of iDNAPro-PseAAC

**Figure 1:**
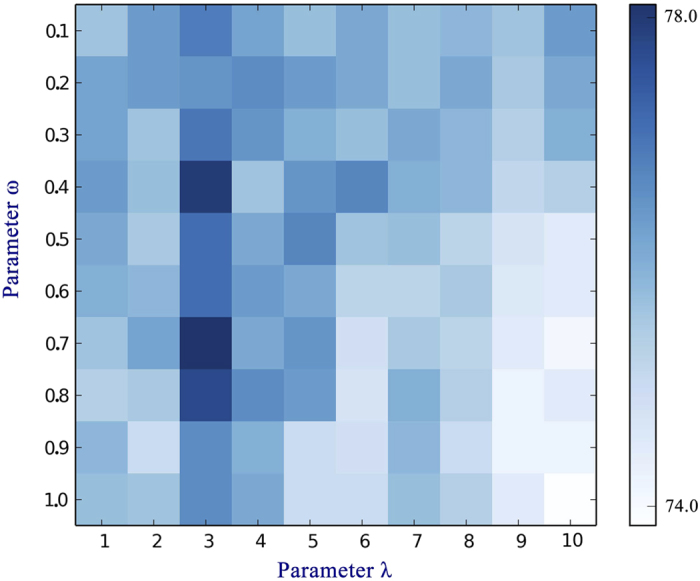
The performance of iDNAPro-PseAAC with different λ and ω combinations. iDNAPro-PseAAC achieves the best performance with *λ* = 3 and ω = 0.7.

There are two parameters *λ* and *ω* in **iDNAPro-PseAAC**, which would influence its performance (see method section). *λ* can be any integer between 1 and *L*-1, where *L* is the shortest length of sequences in the benchmark dataset. The range of *ω* is 0-1. The performance of **iDNAPro-PseAAC** with different *λ* and *ω* combinations is shown in Fig. 1 and Supplementary S1, from which we can see that **iDNAPro-PseAAC** achieves the best performance with *λ* = 3 and *ω* = 0.7. These parameter values are used in the following experiments.

### Discriminant Visualization

To further study the discriminant power of features, we calculate the discriminant weight vectors in the feature space. In the SVM training process, we can get the sequence-specific weights, which can be used to calculate the discriminant weight of each feature. The feature discriminant weight vector **W** can be calculated as following:


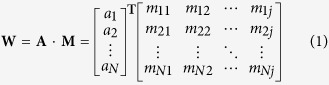


where the weight vector **A** of the training set with *N* samples obtained from the kernel-based training; **M** is the matrix of sequence representatives; *j* is the dimension of the feature vector. The element in **W** represents the discriminative power of the corresponding feature.

**Figure 2:**
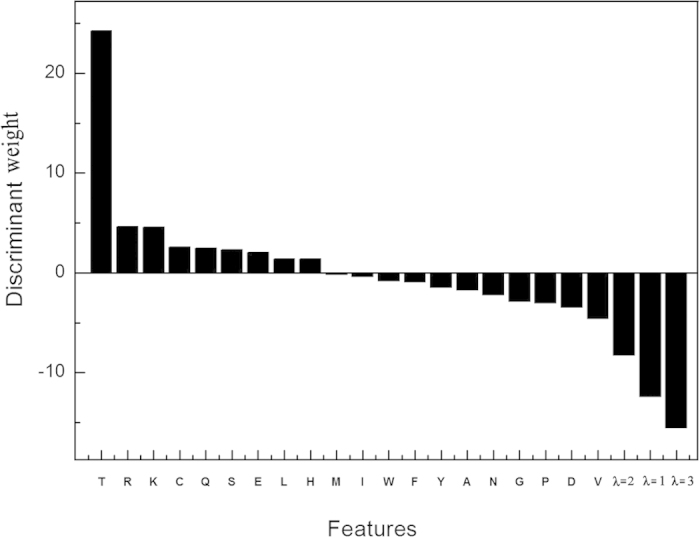
The discriminative weights of all the 23 features.

The discriminative weights of all the 23 features are shown in Fig. 2. We can see that 9 amino acids show positive values, while the other 11 amino acids show negative values. Interestingly, most of the amino acids with positive values, such as R, K, have been reported as important residues in DNA binding proteins, and they are redundant in DNA protein binding regions^22^. **iDNAPro-PseAAC** is able to capture this kind of features of DNA binding proteins, which could explain the reason for its better performance. Another interesting pattern is that all the three features capturing the sequence-order effects (*λ* = 1, 2, 3) show negative values, indicating that this kind of features is useful for representing the features of non DNA binding proteins.

### Results on the benchmark dataset

Table 1 shows the predictive results of **iDNAPro-PseAAC** on the benchmark dataset by using Jackknife test. For comparison, the results of four state-of-the-art methods are also listed, including DNAbinder (dimension 21)^23^, DNAbinder (dimension 400)^23^, DNA-Prot^24^ and iDNA-Prot^25^. The reason why we select these four methods is that they have public available software tools with reported optimized parameters. Their optimized results on the benchmark dataset can be easily obtained by using these tools and parameter settings.

**Figure 3:**
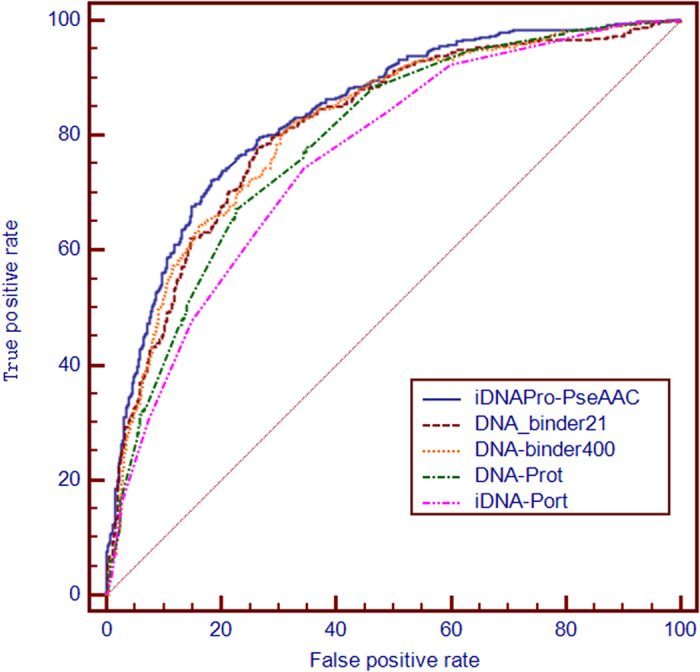
The ROC curves obtained by different methods on the benchmark dataset using the jackknife tests. The areas under the ROC curves or AUC are 0.839, 0.826, 0.814, 0.815, 0.789 and 0.761 for iDNAPro-PseAAC, DNAbinder (dimension 21), DNAbinder(dimension 400), DNA-Prot and iDNA-Prot, respectively. See the main text for further explanation.

From Table 1 we can see that **iDNAPro-PseAAC** achieves the best performance. In order to further study the performance of the proposed method, the ROC curve is employed to evaluate the performance of different methods. ROC curve is a graphical plot that illustrates the performance of a binary classifier system with its discrimination threshold varying. The horizontal coordinate is false-positive rate and the vertical is true-positive rate. The true-positive rate is also known as sensitivity in biomedical informatics, or recall in machine learning^5^,^26^. The false-positive rate is also known as the fall-out and can be calculated as 1 -specificity. The area under the curve (AUC) is the evaluation criteria for the classifier. Figure 3 shows the ROC curve of the five methods, from which we can see that **iDNAPro-PseAAC** outperforms other four approaches in terms of AUC.

### Performance comparison with other related computational predictors

**Figure 4:**
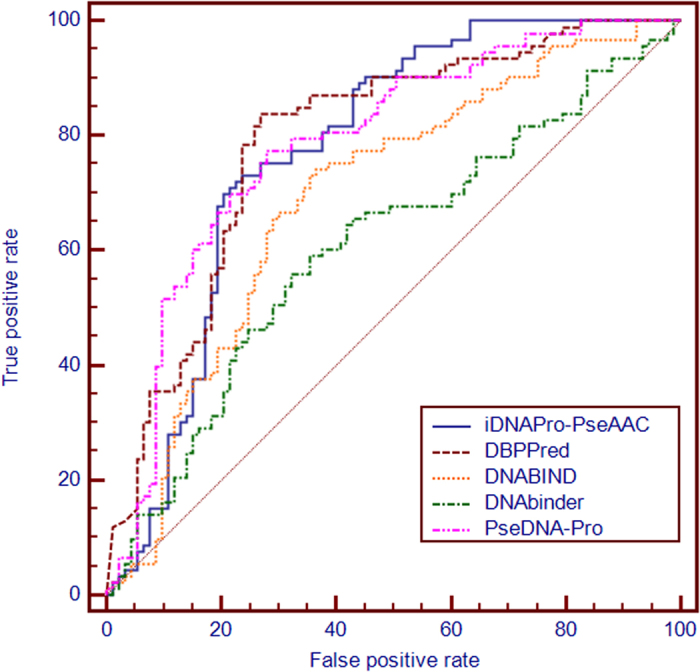
The ROC curves obtained by different methods on the independent dataset PDB186. The areas under the ROC curves or AUC are 0.775, 0.607, 0.694, and 0.791 for iDNAPro-PseAAC, DNAbinder, DNABIND and DBPPred, respectively. See the main text for further explanation.

To further evaluate the performance of **iDNAPro-PseAAC** and facilitate the comparison against previous predictors, an independent test dataset PDB186 constructed by Lou *et al.* is used^27^, where 93 proteins are DNA-binding proteins and 93 proteins are non-DNA-binding proteins. To avoid the homology bias, we use the NCBI’s BLASTCLUST^28^ to remove those proteins from the benchmark dataset that have more than 25% sequence identity to any protein within a same subset of the PDB186 dataset. The **iDNAPro-PseAAC** is re-trained on the resulting benchmark dataset, and then this model is used to predict the samples in the independent dataset. The results are shown in Table 2, and the ROC curves of various methods are plotted in Fig. 4. Compared with the results listed in Table 2, we can see that **iDNAPro-PseAAC** can achieve stable performance on the independent dataset, indicating that the proposed method is a useful tool for DNA binding protein identification. **iDNAPro-PseAAC** outperforms other approaches except for DBPPred. However, our method is more efficient than DBPPred. DBPPred uses 1486 features derived from predicted secondary structure, predicted relative solvent accessibility, and position specific scoring matrix. These features are calculated with the help of two software tools, including SPINE-X and Psi-Blast. All these tools require a time consuming multiple sequence alignment process^29^. Furthermore, these features contain several parameters, which should be optimized on a validate dataset. This requires additional running time for DBPPred and raises the risk of over-fitting problem caused by this parameter optimization process. In contrast, the 23 features used in **iDNAPro-PseAAC** can be easily generated only based on the protein sequences. Therefore, **iDNAPro-PseAAC** is more efficient than DBPPred, and avoids the risk of over-fitting.

### Influence of Negative Samples on the Predictive Performance

**Figure 5:**
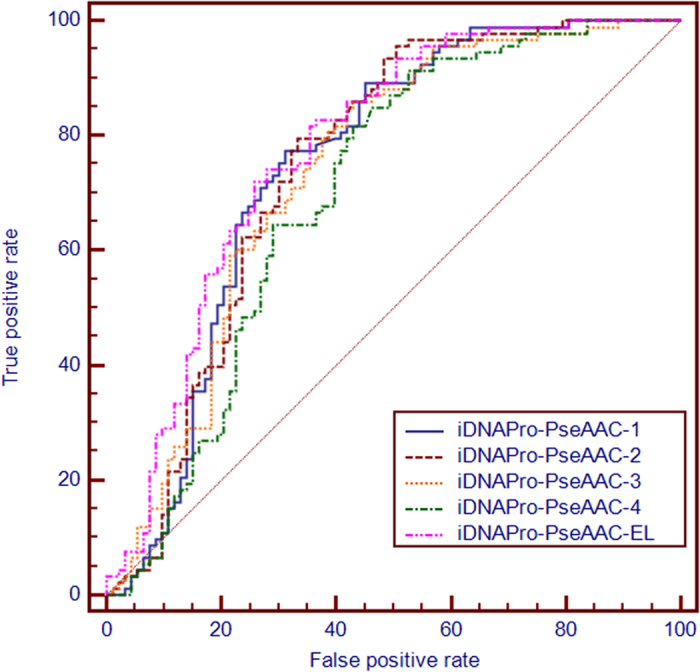
The ROC curves obtained by different iDNAPro-PseAAC predictors on the independent dataset PDB186. The areas under the ROC curves or AUC are 0.750, 0.750, 0.741, 0.702, 0.778 for **iDNAPro-PseAAC-1, iDNAPro-PseAAC-2, iDNAPro-PseAAC-3, iDNAPro-PseAAC-4, and iDNAPro-PseAAC-EL**, respectively.

In real world application, there are more non DNA binding proteins (negative samples) than the DNA binding proteins (positive samples)^30^. However, in order to avoid the classifier biased problem, a balanced benchmark dataset 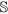 is used to construct **iDNAPro-PseAAC**. Therefore, it is interesting to explore the influence of different negative sets on the predictive performance of **iDNAPro-PseAAC**. In this regard, we conduct the following experiments. First we extend the size of the negative set 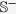 in the the benchmark dataset 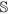 by selecting more non DNA binding proteins from PDB^31^. After removing the redundant proteins sharing more than 25% similarity with the independent dataset, we obtain 2059 negative samples, which were listed in Supplementary S2. The extended negative set is then randomly divided into 4 subsets. For each subset, its size is approximately equal to that of the positive set 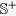 in the the benchmark dataset 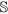. These four subsets are respectively combined with the positive set 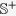, and four new datasets are generated. Four predictors of **iDNAPro-PseAAC** trained with these four datasets can be represented as **iDNAPro-PseAAC-1, iDNAPro-PseAAC-2, iDNAPro-PseAAC-3, and iDNAPro-PseAAC-4**, respectively. Their performance is then evaluated on the independent dataset. Table 3 shows the results of the four methods, and the corresponding ROC curves are plotted in Fig. 5, from which, we can see that the four predictors show similar performance, indicating that different subsets of negative samples don’t have significant impact on the performance of **iDNAPro-PseAAC**. Next, we investigate if these four predictors can be combined to further improve the performance. In this regard, we employ a simple ensemble learning approach to combine them^32^,^33^. For each test sample, it is predicted by the four predictors respectively, and the final class label of the test sample is assigned based on the average values of the four probability values calculated by the four predictors. The results of **iDNAPro-PseAAC-EL** (**iDNAPro-PseAAC** with the ensemble learning approach) are shown in Table 3 and Fig. 5. Performance improvement can be observed. This is because by using ensemble learning method, more negative samples are used to train **iDNAPro-PseAAC**, leading to a more accurate predictor.

## Discussion

Because of the importance of DNA binding protein identification, computational predictors only using the sequence information for DNA binding protein identification is highly desired. In this study, we proposed a method called **iDNAPro-PseAAC** for DNA binding protein identification, which combines the pseudo amino acid composition with profile-based protein representation. Experimental results show that it outperform other approaches in both benchmark dataset and independent dataset. Furthermore, the discriminative model can be analyzed to reveal the in-depth features of DNA binding proteins, which would benefit the researchers who want to investigate the characteristics of DNA binding proteins. Some recent studies have shown that DNA-binding proteins also regulate the microRNA targets, and involve in the noncoding RNA-protein-disease network 34,35,36,37,38,39. We believe that this predictor would be a high throughput tool for DNA binding protein investigation.

## Methods

### Benchmark Dataset

A reliable and stringent benchmark dataset is necessary to build and evaluate a statistical predictor. In this regard, an updated benchmark dataset for this study is constructed based on the latest version of Protein Data Bank (PDB)^31^, which can be formulated as:


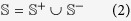


where 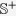 represents the subset containing DNA binding proteins (positive samples), 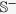 represents the subset containing non DNA binding proteins (negative samples), and the symbol ∪ is the “union” in the set theory. DNA-binding protein sequences are collected from the PDB by searching the mmCIF keyword of ‘DNA binding protein’, ‘protein-DNA complex’, and other key words with similar meaning. To construct a high quality and non-redundant benchmark dataset, the protein sequences obtained should be filtered by the following 2 criteria. (1) Proteins with length less than 50 AA were removed, which might be fragment. (2) To reduce redundancy and homology bias, the sequence similarity lower than 25% between any two proteins were cut off by using PISCES^40^. Finally, we obtained 525 DNA binding proteins for the subset of 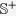. 550 non DNA binding proteins were randomly selected from the PDB according to the above criteria. The accession codes and sequences of the 525 positive and 550 negative samples are given in the Supplementary S3.

### Profile-based protein representation

Profile-based protein representation^21^ is an efficient approach to extract the evolutionary information from frequency profiles. Its main steps are as follows.

Given the protein sequence **P** consisting *L* amino acids as formulated as:


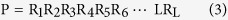


where R_1_ represents the 1^st^ residue, R_2_ represents the 2^nd^ residue and so forth. The frequency profile of sequence **P** generated by PSI-BLAST^28^ with default parameters can be represented as a matrix **M**:


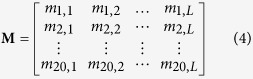


where 20 is the number of standard amino acids; *m*_*ij*_ is the target frequency representing the probability of amino acid *i* (*i* = 1, 2, …, 20) appearing in sequence position *j* (*j* = 1, 2, 3…, *L*) of protein **P** during evolutionary process. The *m*_*ij*_ is calculated as:


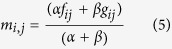


where *f*_*ij*_ represents the observed frequency of amino acid *i* in column *j*, *α* is the number of different amino acids in column *j*-1. *β* is a free parameter set to a constant value of 10, which is initially used by PSI-BLAST. *g*_*ij*_ is the pseudo-count for standard amino acid *i* in position *j*. It is calculated as follows:


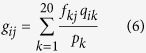


where *p*_*k*_ is the background frequency of amino acid *k*, *q*_*ik*_ is the score of amino acid *i* being aligned to amino acid *j* in BLOSUM62 substitution matrix, which is the default score matrix of PSI-BLAST.

For each column in **M**, the amino acids are sorted in descending order according to their frequency values. Thus the sorted matrix 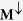 can be represented as:


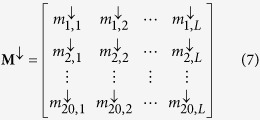


where


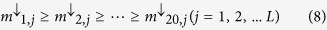


The profile-based protein representation **P′** of protein **P** can be generated by combining the most frequent amino acids in all the columns of **M**, and can be represented as:


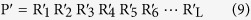


where 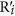 represents the most frequent amino acid in the *i*-th column of 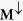, whose frequency value is 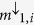.

### Pseudo amino acid composition (PseAAC)

One of the most important but also most difficult problems in computational biology and biomedicine is how to formulate a biological sequence with a discrete model or a vector, yet still keep considerable sequence order information. This is because all the existing operation engines, such as SVM (Support Vector Machine) and NN (Neural Network), can only handle vector but not sequence samples, as elaborated in^41^,^42^. However, a vector defined in a discrete model may completely lose all the sequence-order information. To avoid completely losing the sequence-order information for proteins, the pseudo amino acid composition or PseAAC was proposed^43^.

The PseAAC approach then performs on the profile-based protein representation **P′** (c.f. Eq. 9) to convert it into a fixed length feature vector by using PseAAC:


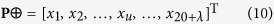


where T is transpose operator, *λ* is the distance parameter considering the sequence-order effects of residues in proteins. *x*_*u*_ can be calculated by


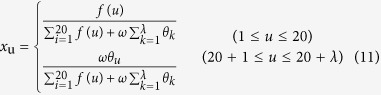


where *f*_*u*_ is the occurrence frequency of 20 standard amino acid in profile-based protein representation **P′**. *ω* is the weight factor for the sequence-order effect. *θ*_*k*_ is the sequence-order correlation factor, which can be calculated as:


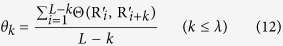


where 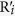 is the *i*-th amino acid in **P′**. *L* is the length of **P′**. *k* is the distance between two amino acids along **P′**. 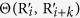 represents the scores calculated according to seven kinds of physical-chemical properties of amino acids (their values are listed in Supplementary S4, which can be calculated by:


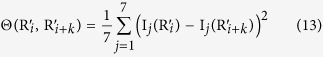


where 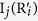 and 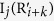 are the normalized physicochemical property values of amino acid 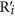 and 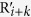 in property *j*, which can be calculated by the following equation:


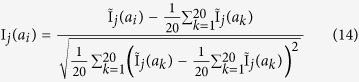


where 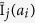 represents the raw physicochemical property value of amino acid *a*_*j*_ in property *j*. *a*_*k*_ (*k* = 1, 2, 3, 4, …, 20) represents the 20 standard amino acids.

### Support Vector Machine

In machine learning, support vectors are supervised learning models with associated learning algorithms^44^. For a given training samples, the basic mission of SVM is constructing a separating hyper-plane to maximize the margin of different samples in training set. An SVM model is a representation of examples as points in space, mapped so that the examples of the separate categories are divided by a clear gap.

In this study, we adopt the Lib-SVM package. The kernel function was set as Radial Basis Function(RBF) which can be defined as:


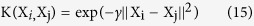


The two parameters *C* and γ were were optimized on the benchmark dataset by the grid tools in the LIBSVM. After optimizing, C is set as 8192 and γ is set as 8.0.

**Figure 6:**
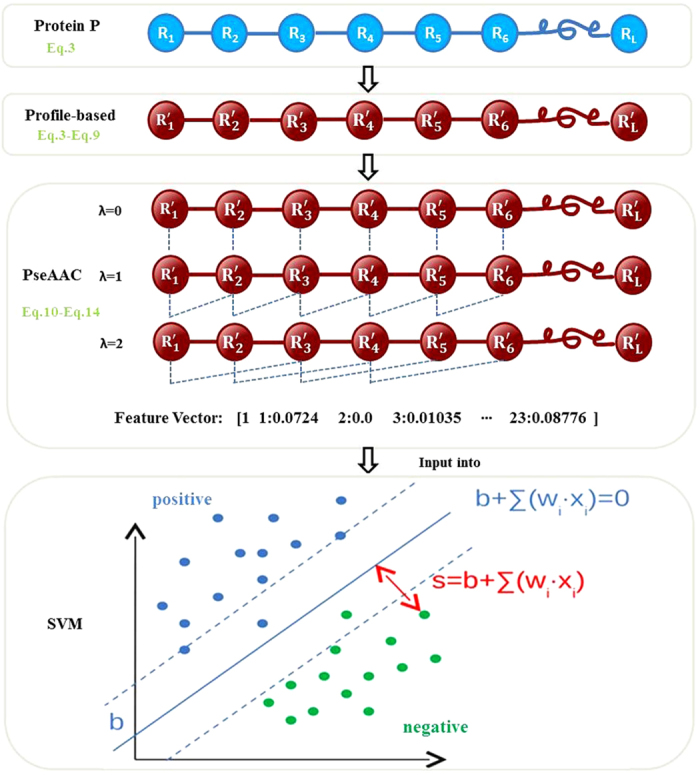
The flowchart of iDNAPro-PseAAC. First, a profile-based protein is generated based on the frequency profile. Pseudo amino acid composition is then performed on this profile-based protein to convert it into a fixed feature vector, which will be fed into SVM to train the classification model.

The flowchart of generating the feature vectors and constructing the SVM classifier for **iDNAPro-PseAAC** is shown in Fig. 6.

### Evaluation methodology

How to evaluate the performance of a new predictor is a key component. There are three cross-validation methods, which are often used: independent dataset, subsampling or K-fold(such as 5-fold, 7-fold, or 10-fold) test, and Jackknife test. However, there are considerable arbitrariness exists in the independent dataset test and the K-fold cross validation. Jackknife can make the least arbitrary and has been widely used in computational genomics and proteomics. In the jackknife test, each of the proteins sequence in the benchmark is singled out as an independent test sample in turn.

Also, four metrics called the sensitivity(Sn), specificity(Sp), accuracy(Acc), and Mathew’s correlation coefficient(MCC), are often used to measure the test quality of a predictor from different angles^45^.


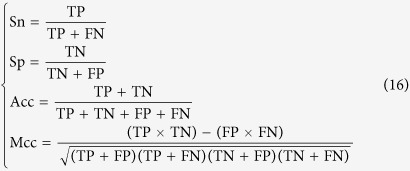


where TP represents the number of the true positive; TN, the number of the true negative; FP, the number of the false positive; FN, the number of the false negative; SN, the sensitivity; Sp, the specificity; Acc, the accuracy; MCC, the Mathew’s correlation coefficient.

In the study, we also use the metrics receiver operation characteristics (ROC) score. ROC curve is a graphical plot that illustrates the performance of a binary classifier system as its discrimination threshold is varied^46^. A score 1 denotes perfect separation of positive samples from negative ones, whereas a score of 0 indicates that none of the sequences selected by the algorithm is positive.

## Additional Information

**How to cite this article**: Liu, B. *et al.* DNA binding protein identification by combining pseudo amino acid composition and profile-based protein representation. *Sci. Rep.* **5**, 15479; doi: 10.1038/srep15479 (2015).

## Supplementary Material

Supplementary Information

## Figures and Tables

**Table 1:** A comparison of the jackknife test results by **iDNAPro-PseAAC** with the other methods on the benchmark dataset of Eq. 2 (cf. Supporting Information S3).

Method	Acc(%)	MCC	Sn(%)	Sp(%)	AUC(%)
iDNAPro-PseAAC	76.56	0.53	75.62	77.45	83.92
DNAbinder (dimension 21)^a^	73.95	0.48	68.57	79.09	81.40
DNAbinder (dimension 400)^b^	73.58	0.47	66.47	80.36	81.50
DNA-Prot^c^	72.55	0.44	82.67	59.76	78.90
iDNA-Prot^d^	75.40	0.50	83.81	64.73	76.10

**Table 2:** A comparison of the results^a^ obtained by **iDNAPro-PseAAC** and the other methods on the independent dataset PDB186.

Methods	Acc(%)	MCC	Sn(%)	Sp(%)	AUC(%)
iDNAPro-PseAAC	69.89	0.402	77.41	62.37	77.54
iDNA-Prot	67.20	0.344	67.70	66.70	N/A^b^
DNA-Prot	61.80	0.240	69.90	53.80	N/A
DNAbinder	60.80	0.216	57.00	64.50	60.70
DNABIND	67.70	0.355	66.70	68.80	69.40
DNA-Threader	59.70	0.279	23.70	95.70	N/A
DBPPred	76.90	0.538	79.60	74.20	79.10

^a^The results of iDNA-Prot^25^, DNA-Prot^24^, DNAbinder^23^, DNABIND^47^, DNA-Threader^48^, and DBPPred^27^ were obtained from^27^.

^b^N/A represents the unreported values.

**Table 3:** Results on independent dataset PDB186 achieved by **iDNAPro-PseAAC** trained with different datasets.

Methods	Acc(%)	MCC	Sn(%)	Sp(%)	AUC(%)
iDNAPro-PseAAC-1	69.89	0.406	79.57	60.22	75.00
iDNAPro-PseAAC-2	71.50	0.449	86.02	56.99	75.00
iDNAPro-PseAAC-3	69.89	0.409	81.72	58.06	74.10
iDNAPro-PseAAC-4	69.35	0.407	84.95	53.76	70.20
iDNAPro-PseAAC-EL	71.50	0.442	82.76	60.22	77.80
