# Supplementary material for: DNA binding protein identification by combining pseudo amino acid composition and profile-based protein representation
Source: Sci Rep. 2015 Oct 20;5:15479. doi: 10.1038/srep15479 (PMC4611492; doi:10.1038/srep15479)
Supplement: Supplementary Information [file srep15479-s1.pdf]

Supplementary Information for

DNA binding protein identification by combining pseudo amino acid composition and profile-based protein representation

Bin Liu, Shanyi Wang, Xiaolong Wang

Supplementary S1. Performance of iDNAPro-PseAAC with different λ and ω on the benchmark dataset.

Distance / Accuracy / Weight	1	2	3	4	5	6	7	8	9	10
0.1	75.35	75.91	76.28	75.81	75.44	75.72	75.44	75.53	75.35	75.91
0.2	75.81	75.91	76.00	76.09	75.91	75.72	75.44	75.72	75.26	75.72
0.3	75.81	75.35	76.37	76.00	75.63	75.44	75.72	75.53	75.16	75.63
0.4	75.91	75.44	77.12	75.35	76.00	76.19	75.63	75.53	74.98	75.16
0.5	75.72	75.26	76.47	75.72	76.19	75.35	75.44	75.07	74.70	74.51
0.6	75.63	75.53	76.47	75.91	75.72	75.07	75.07	75.26	74.60	74.51
0.7	75.35	75.81	77.21	75.72	76.00	74.79	75.26	75.07	74.51	74.23
0.8	75.16	75.26	76.93	76.09	75.91	74.70	75.63	75.16	74.33	74.51
0.9	75.53	74.88	76.09	75.63	74.88	74.79	75.53	74.88	74.33	74.33
1.0	75.44	75.35	76.09	75.72	74.88	74.88	75.44	75.16	74.51	74.05

Supplementary S2. The extended negative dataset contains 2059 non DNA-binding protein sequences.

(1)2059 non DNA-binding proteins
>1RQWA
ATFEIVNRCSYTVWAAASKGDAALDAGGRQLNSGESWTINVEPGTKGGKIWARTDCYFDDSGSGI
CKTGDCGGLLRCKRFGRPPTTLAEFSLNQYGKDYIDISNIKGFNVPMDFSPTTRGCRGVRCAADI
VGQCPAKLKAPGGGCNDACTVFQTSEYCCTTGKCGPTEYSRFFKRLCPDAFSYVLDKPTTVTCPG
SSNYRVTFCPTA
>1H2GB
SNMWVIGKSKAQDAKAIMVNGPQFGWYAPAYTYGIGLHGAGYDVTGNTPFAYPGLVFGHNGVISW
GSTAGLGDDVDIFAERLSAEKPGYYLHNGKWVKMLSREETITVKNGQAETFTVWRTVHGNILQTD
QTTQTAYAKSRAWDGKEVASLLAWTHQMKAKNWQEWTQQAAKQALTINWYYADVNGNIGYVHTGA
YPDRQSGHDPRLPVPGTGKWDWKGLLPFEMNPKVYNPQSGYIANWNNSPQKDYPASDLFAFLWGG
ADRVTEIDRLLEQKPRLTADQAWDVIRQTSRQDLNLRLFLPTLQAATSGLTQSDPRRQLVETLTR
WDGINLLNDDGKTWQQPGSAILNVWLTSMLKRTVVAAVPMPFDKWYSASGYETTQDGPTGSLNIS
VGAKILYEAVQGDKSPIPQAVDLFAGKPQQEVVLAALEDTWETLSKRYGNNVSNWKTPAMALTFR
ANNFFGVPQAAAEETRHQAEYQNRGTENDMIVFSPTTSDRPVLAWDVVAPGQSGFIAPDGTVDKH
YEDQLKMYENFGRKSLWLTKQDVEAHKESQEVLHVQR
>1A12A
RRSPPADAIPKSKKVKVSHRSHSTEPGLVLTLGQGDVGQLGLGENVMERKKPALVSIPEDVVQAE
AGGMHTVCLSKSGQVYSFGCNDEGALGRDTSVEGSEMVPGKVELQEKVVQVSAGDSHTAALTDDG
RVFLWGSFRDNNGVIGLLEPMKKSMVPVQVQLDVPVVKVASGNDHLVMLTADGDLYTLGCGEQGQ
LGRVPELFANRGGRQGLERLLVPKCVMLKSRGSRGHVRFQDAFCGAYFTFAISHEGHVYGFGLSN
YHQLGTPGTESCFIPQNLTSFKNSTKSWVGFSGGQHHTVCMDSEGKAYSLGRAEYGRLGLGEGAE
EKSIPTLISRLPAVSSVACGASVGYAVTKDGRVFAWGMGTNYQLGTGQDEDAWSPVEMMGKQLEN
RVVLSVSSGGQHTVLLVKDKEQS
>3INGA
GMKEIRIILMGTGNVGLNVLRIIDASNRRRSAFSIKVVGVSDSRSYASGRNLDISSIISNKEKTG
RISDRAFSGPEDLMGEAADLLVDCTPASRDGVREYSLYRMAFESGMNVVTANKSGLANKWHDIMD
SANQNSKYIRYEATVAGGVPLFSVLDYSILPSKVKRFRGIVSSTINYVIRNMANGRSLRDVVDDA
IKKGIAESNPQDDLNGLDAARKSVILVNHIFGTEYTLNDVEYSGVDERSYNANDRLVTEVYVDDR
RPVAVSRIISLNKDDFLMSIGMDGLGYQIETDSNGTVNVSDIYDGPYETAGAVVNDILLLSKVQK
>2RAUA
GMYEEWKIVKREAPILGNDQLIENIWKMKREDSPYDIISLHKVNLIGGGNDAVLILPGTWSSGEQ
LVTISWNGVHYTIPDYRKSIVLYLARNGFNVYTIDYRTHYVPPFLKDRQLSFTANWGWSTWISDI
KEVVSFIKRDSGQERIYLAGESFGGIAALNYSSLYWKNDIKGLILLDGGPTKHGIRPKFYTPEVN
SIEEMEAKGIYVIPSRGGPNNPIWSYALANPDMPSPDPKYKSISDFLMDSLYVTGSANPYDYPYS
KKEDMFPILASFDPYWPYRLSLERDLKFDYEGILVPTIAFVSERFGIQIFDSKILPSNSEIILLK
GYGHLDVYTGENSEKDVNSVVLKWLSQQR
>3N20A
MSMLKREDWYDLTRTTNWTPKYVTENELFPEEMSGARGISMEAWEKYDEPYKITYPEYVSIQREK
DSGAYSIKAALERDGFVDRADPGWVSTMQLHFGAIALEEYAASTAEARMARFAKAPGNRNMATFG
MMDENRHGQIQLYFPYANVKRSRKWDWAHKAIHTNEWAAIAARSFFDDMMMTRDSVAVSIMLTFA

FETGFVNMQFLGLAADAAEAGDHTFASLISSIQTDESRHAQQGGPSLKILVENGKKDEAQQMVDV
AIWRSWKLFSVLTGPIMDYYTPLESRNQSFKEFMLEWIVAQFERQLLDLGLDKPWYWDQFMQDLD
ETHHGMHLGVWYWRPTVWWDPAAGVSPEEREWLEEKYPGWNDTWGQCWDVITDNLVNGKPELTVP
ETLPTICNMCNLPIAHTPGNKWNVKDYQLEYEGRLYHFGSEADRWCFQIDPERYENHTNLVDRFL
KGEIQPADLAGALMYMSLEPGVMGDDAHDYEWVKAYQKKTNAA
>3N2BA
MHHHHHHSSGVDLGTENLYFQSNAMDYFNYQEDGQLWAEQVPLADLANQYGTPLYVYSRATLERH
WHAFDKSVGDYPHLICYAVKANSNLGVLNTLARLGSGFDIVSVGELERVLAAGGDPSKVVFSGVG
KTEAEMKRALQLKIKCFNVESEPELQRLNKVAGELGVKAPISLRINPDVDAKTHPYISTGLRDNK
FGITFDRAAQVYRLAHSLPNLDVHGIDCHIGSQLTALAPFIDATDRLLALIDSLKAEGIHIRHLD
VGGGLGVVYRDELPPQPSEYAKALLDRLERHRDLELIFEPGRAIAANAGVLVTKVEFLKHTEHKN
FAIIDAAMNDLIRPALYQAWQDIIPLRPRQGEAQTYDLVGPVCETSDFLGKDRDLVLQEGDLLAV
RSSGAYGFTMSSNYNTRPRVAEVMVDGNKTYLVRQREELSSLWALESVLPE
>1P0WA
TIKEMPQPKTFGELKNLPLLNTDKPVQALMKIADELGEIFKFEAPGRVTRYLSSQRLIKEACDES
RFDKNLSQALKFVRDFAGDGLFTSWTHEKNWKKAHNILLPSFSQQAMKGYHAMMVDIAVQLVQKW
ERLNADEHIEVPEDMTRLTLDTIGLCGFNYRFNSFYRDQPHPFITSMVRALDEAMNKLQRANPDD
PAYDENKRQFQEDIKVMNDLVDKIIADRKASGEQSDDLLTHMLNGKDPETGEPLDDENIRYQIIT
FLIAGHETTSGLLSFALYFLVKNPHVLQKAAEEAARVLVDPVPSYKQVKQLKYVGMVLNEALRLW
PTAPAFSLYAKEDTVLGGEYPLEKGDELMVLIPQLHRDKTIWGDDVEEFRPERFENPSAIPQHAF
KPWGNGQRACIGQQFALHEATLVLGMMLKHFDFEDHTNYELDIKETLTLKPEGFVVKAKSKKIPL
>1UJ0A
AGHMARRVRALYDFEAVEDNELTFKHGELITVLDDSDANWWQGENHRGTGLFPSNFVTTDLS
>1UJMA
AKIDNAVLPEGSLVLVTGANGFVASHVVEQLLEHGYKVRGTARSASKLANLQKRWDAKYPGRFET
AVVEDMLKQGAYDEVIKGAAGVAHIASVVSFSNKYDEVVTPAIGGTLNALRAAAATPSVKRFVLT
SSTVSALIPKPNVEGIYLDEKSWNLESIDKAKTLPESDPQKSLWVYAASKTEAELAAWKFMDENK
PHFTLNAVLPNYTIGTIFDPETQSGSTSGWMMSLFNGEVSPALALMPPQYYVSAVDIGLLHLGCL
VLPQIERRRVYGTAGTFDWNTVLATFRKLYPSKTFPADFPDQGQDLSKFDTAPSLEILKSLGRPG
WRSIEESIKDLVGSETA
>1PAHA
TVPWFPRTIQELDRFANQILSYGAELDADHPGFKDPVYRARRKQFADIAYNYRHGQPIPRVEYME
EEKKTWGTVFKTLKSLYKTHACYEYNHIFPLLEKYCGFHEDNIPQLEDVSQFLQTCTGFRLRPVA
GLLSSRDFLGGLAFRVFHCTQYIRHGSKPMYTPEPDICHELLGHVPLFSDRSFAQFSQEIGLASL
GAPDEYIEKLATIYWFTVEFGLCKQGDSIKAYGAGLLSSFGELQYCLSEKPKLLPLELEKTAIQN
YTVTEFQPLYYVAESFNDAKEKVRNFAATIPRPFSVRYDPYTQRIEVL
>1XKWA
ESTSATQPPGVTTLGKVPLKPRELPQSASVIDHERLEQQNLFSLDEAMQQATGVTVQPFQLLTTA
YYVRGFKVDSFELDGVPALLGNTASSPQDMAIYERVEILRGSNGLLHGTGNPAATVNLVRKRPQR
EFAASTTLSAGRWDRYRAEVDVGGPLSASGNVRGRAVAAYEDRDYFYDVADQGTRLLYGVTEFDL
SPDTLLTVGAQYQHIDSITNMAGVPMAKDGSNLGLSRDTYLDVDWDRFKWDTYRAFGSLEQQLGG
GWKGKVSAEYQEADSRLRYAGSFGAIDPQTGDGGQLMGAAYKFKSIQRSLDANLNGPVRLFGLTH
ELLGGVTYAQGETRQDTARFLNLPNTPVNVYRWDPHGVPRPQIGQYTSPGTTTTTQKGLYALGRI
KLAEPLTLVVGGRESWWDQDTPATRFKPGRQFTPYGGLIWDFARDWSWYVSYAEVYQPQADRQTW

NSEPLSPVEGKTYETGIKGELADGRLNLSLAAFRIDLENNPQEDPDHPGPPNNPFYISGGKVRSQ
GFELEGTGYLTPYWSLSAGYTYTSTEYLKDSQNDSGTRYSTFTPRHLLRLWSNYDLPWQDRRWSV
GGGLQAQSDYSVDYRGVSMRQGGYALVNMRLGYKIDEHWTAAVNVNNLFDRTYYQSLSNPNWNNR
YGEPRSFNVSLRGAF
>2EX4A
MGSSHHHHHHSSGLVPRGSTSEVIEDEKQFYSKAKTYWKQIPPTVDGMLGGYGHISSIDINSSRK
FLQRFLREGPNKTGTSCALDCGAGIGRITKRLLLPLFREVDMVDITEDFLVQAKTYLGEEGKRVR
NYFCCGLQDFTPEPDSYDVIWIQWVIGHLTDQHLAEFLRRCKGSLRPNGIIVIKDNMAQEGVILD
DVDSSVCRDLDVVRRIICSAGLSLLAEERQENLPDEIYHVYSFALR
>3ME7A
MSLGTYVPGDITLVDSYGNEFQLKNLKGKPIILSPIYTHCRAACPLITKSLLKVIPKLGTPGKDF
WVITFTFDPKDTLEDIKRFQKEYGIDGKGWKVVKAKTSEDLFKLLDAIDFRFMTAGNDFIHPNVV
VVLSPELQIKDYIYGVNYNYLEFVNALRLARGEGHHHHHH
>2NT3A
GSHMSKKILIVESDTALSATLRSALEGRGFTVDETTDGKGSVEQIRRDRPDLVVLAVDLSAGQNG
YLICGKLKKDDDLKNVPIVIIGNPDGFAQHRKLKAHADEAVAKPVDADQLVERAGALIGFPE
>1RFXA
SSMPLCPIDEAIDKKIKQDFNSLFPNAIKNIGLNCWTVSSRGKLASCPEGTAVLSCSCGSACGSW
DIREEKVCHCQCARIDWTAARCCKLQVAS
>1H0HB
SKGFFVDTTRCTACRGCQVACKQWHGNPATPTENTGFHQNPPDFNFHTYKLVRMHEQEIDGRIDW
LFFPDQCRHCIAPPCKATADMEDESAIIHDDATGCVLFTPKTKDLEDYESVISACPYDVPRKVAE
SNQMAKCDMCIDRITNGLRPACVTSCPTGAMNFGDLSEMEAMASARLAEIKAAYSDAKLCDPDDV
RVIFLTAHNPKLYHEYAVA
>1ZE3D
DLYFNPRFLADDPQAVADLSRFENGQELPPGTYRVDIYLNNGYMATRDVTFNTGDSEQGIVPCLT
RAQLASMGLNTASVAGMNLLADDACVPLTTMVQDATAHLDVGQQRLNLTIPQAFMSNRAR
>1J8MF
SKLLDNLRDTVRKFLTGSSSYDKAVEDFIKELQKSLISADVNVKLVFSLTNKIKERLKNEKPPTY
IERREWFIKIVYDELSNLFGGDKEPKVIPDKIPYVIMLVGVQGTGKTTTAGKLAYFYKKKGFKVG
LVGADVYRPAALEQLQQLGQQIGVPVYGEPGEKDVVGIAKRGVEKFLSEKMEIIIVDTAGRHGYG
EEAALLEEMKNIYEAIKPDEVTLVIDASIGQKAYDLASKFNQASKIGTIIITKMDGTAKGGGALS
AVAATGATIKFIGTGEKIDELEVFNPRRFVARLHHHH
>3K8GA
GSGAWKASVDPLGVVGSGADVYLYFPVAGNENLISRIIENHESKADIKKIVDRTTAVYGAFFARS
KEFRLFGSGSYPYAFTNLIFSRSDGWASTKTEHGITYYESEHTDVSIPAPHFSCVIFGSSKRERM
SKMLSRLVNPDRPQLPPRFEKECTSEGTSQTVALYIKNGGHFITKLLNFPQLNLPLGAMELYLTA
RRNEYLYTLSLQLGNAKINFPIQFLISRVLNAHIHVEGDRLIIEDGTISAERLASVISSLYSKKG
SS
>2B1LA
MQFYQADVLTQGKPVLLNVWATWCPTCRAEHQYLNQLSAQGIRVVGMNYKDDRQKAISWLKELGN
PYALSLFDGDGMLGLDLGVYGAPETFLIDGNGIIRYRHAGDLNPRVWEEEIKPLWEKYSKEAAQ
>2ZZ3A
GSHMRSRRVDVMDVMNRLILAMDLMNRDDALRVTGEVREYIDTVKIGYPLVLSEGMDIIAEFRKR

FGCRIIAAFKVADIPETNEKICRATFKAGADAIIVHGFPGADSVRACLNVAEEMGREVFLLTEMS
HPGAEMFIQGAADEIARMGVDLGVKNYVGPSTRPERLSRLREIIGQDSFLISPGVGAQGGDPGET
LRFADAIIVGRSIYLADNPAAAAAGIIESIKDLRIPEDPAANKARKEAELAAATAEQ
>3GY1A
MSLEPTIITDVLCYITKPDRHNLVVVKVETNKGIYGLGCATFQQRPKAVSLVVSEYLKPILIGRD
ANNIEDLWQMMMVNSYWRNGPILNNAISGVDMALWDIKGKLANMPLYQLFGGKSRDAIAAYTHAV
ADNLEDLYTEIDEIRKKGYQHIRCQLGFYGGNSSEFHTTDNPTQGSYFDQDEYMRTTVSMFSSLR
EKYGYKFHILHDVHERLFPNQAVQFAKDVEKYKPYFIEDILPPDQNEWLGQIRSQTSTPLATGEL
FNNPMEWKSLIANRQVDFIRCHVSQIGGITPALKLGSLCAAFGVRIAWHTPSDITPIGVAVNIHL
NINLHNAAIQENIEINDNTRCVFSGIPEAKNGFFYPIESPGIGVDIDENEIIKYPVEYRPHEWTQ
SRIPDGTIVTEGHHHHHH
>2P35A
QGHMAWSAQQYLKFEDERTRPARDLLAQVPLERVLNGYDLGCGPGNSTELLTDRYGVNVITGIDS
DDDMLEKAADRLPNTNFGKADLATWKPAQKADLLYANAVFQWVPDHLAVLSQLMDQLESGGVLAV
QMPDNLQEPTHIAMHETADGGPWKDAFSGGGLRRKPLPPPSDYFNALSPKSSRVDVWHTVYNHPM
KDADSIVEWVKGTGLRPYLAAAGEENREAFLADYTRRIAAAYPPMADGRLLLRFPRLFVVAVKK
>2OFXA
QGHMATNVTYQAHHVSRNKRGQVVGTRGGFRGCTVWLTGLSGAGKTTVSMALEEYLVCHGIPCYT
LDGDNIRQGLNKNLGFSPEDREENVRRIAEVAKLFADAGLVCITSFISPYTQDRNNARQIHEGAS
LPFFEVFVDAPLHVCEQRDVKGLYKKARAGEIKGFTGIDSEYEKPEAPELVLKTDSCDVNDCVQQ
VVELLQERDIVP
>2QSIA
GHMSGSLARAAARPNAPTLVDEATVDDFIAHSGKIVVLFFRGDAVRFPEAADLAVVLPELINAFP
GRLVAAEVAAEAERGLMARFGVAVCPSLAVVQPERTLGVIAKIQDWSSYLAQIGAMLAEVDQPGE
AELQSGS
>2J9FA
SSLDDKPQFPGASAEFIDKLEFIQPNVISGIPIYRVMDRQGQIINPSEDPHLPKEKVLKLYKSMT
LLNTMDRILYESQRQGRISFYMTNYGEEGTHVGSAAALDNTDLVFGQYREAGVLMYRDYPLELFM
AQCYGNISDLGKGRQMPVHYGCKERHFVTISSPLATQIPQAVGAAYAAKRANANRVVICYFGEGA
ASEGDAHAGFNFAATLECPIIFFCRNNGYAISTPTSEQYRGDGIAARGPGYGIMSIRVDGNDVFA
VYNATKEARRRAVAENQPFLIEAMTYRIGHHSTSDDSSAYRPVDEVNYWDKQDHPISRLRHYLLS
QGWWDEEQEKAWRKQSRRKVMEAFEQAERKPKPNPNLLFSDVYQEMPAQLRKQQESLARHLQTYG
EHYPLDHFDK
>3BB0A
MGSVTPIPLPKIDEPEEYNTNYILFWNHVGLELNRVTHTVGGPLTGPPLSARALGMLHLAIHDAY
FSICPPTDFTTFLSPDTENAAYRLPSPNGANDARQAVAGAALKMLSSLYMKPVEQPNPNPGANIS
DNAYAQLGLVLDRSVLEAPGGVDRESASFMFGEDVADVFFALLNDPRGASQEGYHPTPGRYKFDD
EPTHPVVLIPVDPNNPNGPKMPFRQYHAPFYGKTTKRFATQSEHFLADPPGLRSNADETAEYDDA
VRVAIAMGGAQALNSTKRSPWQTAQGLYWAYDGSNLIGTPPRFYNQIVRRIAVTYKKEEDLANSE
VNNADFARLFALVDVACTDAGIFSWKEKWEFEFWRPLSGVRDDGRPDHGDPFWLTLGAPATNTND
IPFKPPFPAYPSGHATFGGAVFQMVRRYYNGRVGTWKDDEPDNIAIDMMISEELNGVNRDLRQPY
DPTAPIEDQPGIVRTRIVRHFDSAWELMFENAISRIFLGVHWRFDAAAARDILIPTTTKDVYAVD
NNGATVFQNVEDIRYTTRGTREDREGLFPIGGVPLGIEIADEIFNNGLKPTPPEIQPMPQETPVQ
KPVGQQPVKGMWEEEQAPVVKEAP

>3H9CA
TQVAKKILVTCALPYANGSIHLGHMLEHIQADVWVRYQRMRGHEVNFICADDAHGTPIMLKAQQL
GITPEQMIGEMSQEHQTDFAGFNISYDNYHSTHSEENRQLSELIYSRLKENGFIKNRTISQLYDP
EKGMFLPDRFVKGTCPKCKSPDQYGDNCEVCGATYSPTELIEPKSVVSGATPVMRDSEHFFFDLP
SFSEMLQAWTRSGALQEQVANKMQEWFESGLQQWDISRDAPYFGFEIPNAPGKYFYVWLDAPIGY
MGSFKNLCDKRGDSVSFDEYWKKDSTAELYHFIGKDIVYFHSLFWPAMLEGSNFRKPSNLFVHGY
VTVNGAKMSKSRGTFIKASTWLNHFDADSLRYYYTAKLSSRIDDIDLNLEDFVQRVNADIVNKVV
NLASRNAGFINKRFDGVLASELADPQLYKTFTDAAEVIGEAWESREFGKAVREIMALADLANRYV
DEQAPWVVAKQEGRDADLQAICSMGINLFRVLMTYLKPVLPKLTERAEAFLNTELTWDGIQQPLL
GHKVNPFKALYNRIDMRQVEALVEASK
>2PRVA
GMIYSKVENFINENKQNAIFTEGASHENIGRIEENLQCDLPNSYKWFLEKYGAGGLFGVLVLGYN
FDHASVVNRTNEYKEHYGLTDGLVVIEDVDYFAYCLDTNKMKDGECPVVEWDRVIGYQDTVADSF
IEFFYNKIQEAKDDWDEDEDWDD
>1JLJA
MATEGMILTNHDHQIRVGVLTVSDSCFRNLAEDRSGINLKDLVQDPSLLGGTISAYKIVPDEIEE
IKETLIDWCDEKELNLILTTGGTGFAPRDVTPEATKEVIEREAPGMALAMLMGSLNVTPLGMLSR
PVCGIRGKTLIINLPGSKKGSQECFQFILPALPHAIDLLRDAIVKVKEVHDRSHHHHHH
>1XFIA
SESDSEMVPFPQLPMPIENNYRACTIPYRFPSDDPKKATPNEISWINVFANSIPSFKKRAESDIT
VPDAPARAEKFAERYAGILEDLKKDPESHGGPPDGILLCRLREQVLRELGFRDIFKKVKDEENAK
AISLFPQVVSLSDAIEDDGKRLENLVRGIFAGNIFDLGSAQLAEVFSRDGMSFLASCQNLVPRPW
VIDDLENFQAKWINKSWKKAVIFVDNSGADIILGILPFARELLRRGAQVVLAANELPSINDITCT
ELTEILSQLKDENGQLLGVDTSKLLIANSGNDLPVIDLSRVSQELAYLSSDADLVIVEGMGRGIE
TNLYAQFKCDSLKIGMVKHLEVAEFLGGRLYDCVFKFNEVQS
>1KZ1A
MFSGIKGPNPSDLKGPELRILIVHARGNLQAIEPLVKGAVETMIEKHDVKLENIDIESVPGSWEL
PQGIRASIARNTYDAVIGIGVLIKGSTMHFEYISEAVVHGLMRVGLDSGVPVILGLLTVLNEEQA
LYRAGLNGGHNHGNDWGSAAVEMGLKALY
>1E2XA
FADRMVIKAQSPAGFAEEYIIESIWNNRFPPGTILPAERELSELIGVTRTTLREVLQRLARDGWL
TIQHGKPTKVNNFWETSGLNILETLARLDHESVPQLIDNLLSVRTNISTIFIRTAFRQHPDKAQE
VLATANEVADHADAFAELDYNIFRGLAFASGNPIYGLILNGMKGLYTRIGRHYFANPEARSLALG
FYHKLSALCSEGAHDQVYETVRRYGHESGEIWHRMQKNLPGDLAIQGR
>2O7IA
MQVSLPREDTVYIGGALWGPATTWNLYAPQSTWGTDQFMYLPAFQYDLGRDAWIPVIAERYEFVD
DKTLRIYIRPEARWSDGVPITADDFVYALELTKELGIGPGGGWDTYIEYVKAVDTKVVEFKAKEE
NLNYFQFLSYSLGAQPMPKHVYERIRAQMNIKDWINDKPEEQVVSGPYKLYYYDPNIVVYQRVDD
WWGKDIFGLPRPKYLAHVIYKDNPSASLAFERGDIDWNGLFIPSVWELWEKKGLPVGTWYKKEPY
FIPDGVGFVYVNNTKPGLSDPAVRKAIAYAIPYNEMLKKAYFGYGSQAHPSMVIDLFEPYKQYID
YELAKKTFGTEDGRIPFDLDMANKILDEAGYKKGPDGVRVGPDGTKLGPYTISVPYGWTDWMMMC
EMIAKNLRSIGIDVKTEFPDFSVWADRMTKGTFDLIISWSVGPSFDHPFNIYRFVLDKRLSKPVG
EVTWAGDWERYDNDEVVELLDKAVSTLDPEVRKQAYFRIQQIIYRDMPSIPAFYTAHWYEYSTKY
WINWPSEDNPAWFRPSPWHADAWPTLFIISKKSDPQPVPSWLGTVDEGGIEIPTAKIFEDLQKAT

MHHHHHH
>1YU0A
MSTAVQFRGGTTAQHATFTGAAREITVDTDKNTVVVHDGATAGGFPLARHDLVKTAFIKADKSAV
AFTRTGNATASIKAGTIVEVNGKLVQFTADTAITMPALTAGTDYAIYVCDDGTVRADSNFSAPTG
YTSTTARKVGGFHYAPGSNAAAQAGGNTTAQINEYSLWDIKFRPAALDPRGMTLVAGAFWADIYL
LGVNHLTDGTSKYNVTIADGSASPKKSTKFGGDGSAAYSDGAWYNFAEVMTHHGKRLPNYNEFQA
LAFGTTEATSSGGTDVPTTGVNGTGATSAWNIFTSKWGVVQASGCLWTWGNEFGGVNGASEYTAN
TGGRGSVYAQPAAALFGGAWNGTSLSGSRAALWYSGPSFSFAFFGARGVCDHLILE
>2QNDA
ASRFHEQFIVREDLMGLAIGTHGANIQQARKVPGVTAIDLDEDTCTFHIYGEDQDAVKKARSFLE
FAEDVIQVPRNLVGKVIGKNGKLIQEIVDKSGVVRVRIEAENEKNVPQEEGMVPFVFVGTKDSIA
NATVLLDYHLNYLK
>3EOFA
GMMDTVKNRRTIRKYQQKDITPDLLNDLLETSFRASTMGGMQLYSVVVTRDAEKKEILSPAHFNQ
PMVKEAPVVLTFCADFRRFCKYCQERNAVPGYGNLMSFLNAAMDTLLVAQTFCTLAEEAGLGICY
LGTTTYNPQMIIDALHLPELVFPITTVTVGYPAESPKQVDRLPIEGIIHEESYHDYTAEDINRLY
AYKESLPENKLFIEENQKETLPQVFTDVRYTKKDNEFMSENLLKVLRRQGFMD
>2GRRA
GSHMSGIALSRLAQERKAWRKDHPFGFVAVPTKNPDGTMNLMNWECAIPGKKGTPWEGGLFKLRM
LFKDDYPSSPPKCKFEPPLFHPNVYPSGTVCLSILEEDKDWRPAITIKQILLGIQELLNEPNIQS
PAQAEAYTIYCQNRVEYEKRVRAQAKKFAPS
>3EJVA
MGSDKIHHHHHHENLYFQGMTMADETIILNVLGQYTRAHDRRDPDAMAALFAPEATIEIVDAVGG
ASRSISRLEGRDAIRVAVRQMMAPHGYRAWSQNVVNAPIIVIEGDHAVLDAQFMVFSILAAEVPD
GGWPTGTFGAQGRIVPIEAGQYRLTLRTVADGWVISAMRIEHRLPMAFG
>3GWBA
ELDGKAPSHRNLNVQTWSTAEGAKVLFVEARELPMFDLRLIFAAGSSQDGNAPGVALLTNAMLNE
GVAGKDVGAIAQGFEGLGADFGNGAYKDMAVASLRSLSAVDKREPALKLFAEVVGKPTFPADSLA
RIKNQMLAGFEYQKQNPGKLASLELMKRLYGTHPYAHASDGDAKSIPPITLAQLKAFHAKAYAAG
NVVIALVGDLSRSDAEAIAAQVSAALPKGPALAKIEQPAEPKASIGHIEFPSSQTSLMLAQLGID
RDDPDYAAVSLGNQILGGGGFGTRLMSEVREKRGLTYGVYSGFTPMQARGPFMINLQTRAEMSEG
TLKLVQDVFAEYLKNGPTQKELDDAKRELAGSFPLSTASNADIVGQLGAMGFYNLPLSYLEDFMR
QSQELTVEQVKAAMNKHLNVDKMVIVSAGPTVAQKPLEHHHHHH
>1GWTA
MQLTPTFYDNSCPNVSNIVRDTIVNELRSDPRIAASILRLHFHDCFVNGCDASILLDNTTSFRTE
KDAFGNANSARGFPVIDRMKAAVESACPRTVSCADLLTIAAQQSVTLAGGPSWRVPLGRRDSLQA
FLDLANANLPAPFFTLPQLKDSFRNVGLNRSSDLVALSGGHTFGKNQCRFIMDRLYNFSNTGLPD
PTLNTTYLQTLRGLCPLNGNLSALVDMDLRTPTIFDNKYYVNLEEQKGLIQSDQELFSSPNATDT
IPLVRSFANSTQTFFNAFVEAMDRMGNITPLTGTQGQIRLNCRVVNSNS
>1E39A
ADNLAEFHVQNQECDSCHTPDGELSNDSLTYENTQCVSCHGTLAEVAETTKHEHYNAHASHFPGE
VACTSCHSAHEKSMVYCDSCHSFDFNMPYAKKWLRDEPTIAELAKDKSERQAALASAPHDTVDVV
VVGSGGAGFSAAISATDSGAKVILIEKEPVIGGNAKLAAGGMNAAWTDQQKAKKITDSPELMFED
TMKGGQNINDPALVKVLSSHSKDSVDWMTAMGADLTDVGMMGGASVNRAHRPTGGAGVGAHVVQV

LYDNAVKRNIDLRMNTRGIEVLKDDKGTVKGILVKGMYKGYYWVKADAVILATGGFAKNNERVAK
LDPSLKGFISTNQPGAVGDGLDVAENAGGALKDMQYIQAAPTLSVKGGVMVTEAVRGNGAILVNR
EGKRFVNEITTRDKASAAILAQTGKSAYLIFDDSVRKSLSKIDKYIGLGVAPTADSLVKLGKMEG
IDGKALTETVARYNSLVSSGKDTDFERPNLPRALNEGNYYAIEVTPGVHHTMGGVMIDTKAEVMN
AKKQVIPGLYGAGEVTGGVHGANRLGGNAISDIITFGRLAGEEAAKYSKKN
>3E3UA
MAVVPIRIVGDPVLHTATTPVTVAADGSLPADLAQLIATMYDTMDAANGVGLAANQIGCSLRLFV
YDCAADRAMTARRRGVVINPVLETSEIPETMPDPDTDDEGCLSVPGESFPTGRAKWARVTGLDAD
GSPVSIEGTGLFARMLQHETGHLDGFLYLDRLIGRYARNAKRAVKSHGWGVPGLSWLPGEDPDPF
GH
>1E30A
GTLDTTWKEATLPQVKAMLEKDTGKVSGDTVTYSGKTVHVVAAAVLPGFPFPSFEVHDKKNPTLE
IPAGATVDVTFINTNKGFGHSFDITKKGPPYAVMPVIDPIVAGTGFSPVPKDGKFGYTNFTWHPT
AGTYYYVCQIPGHAATGQFGKIVVK
>3K2CA
MAHHHHHHMGTLEAQTQGPGSMAKEASGNVYFDVYANEESLGRIVMKLEDDIVPKTAKNFRTLCE
RPKGEGYKGSTFHRIIPGFMVQGGDYTAHNGTGGRSIYGEKFPDENFELKHTKEGILSMANCGAH
TNGSQFFITLGKTQWLDEKHVVFGEVVEGMDVVHKIAKYGSESGQVKKGYRIEIRDCGVLGSN
>2R1BA
GSSLRGGHAGTTYIFSKGGGQITYKWPPNDRPSTRADRLAIGFSTVQKEAVLVRVDSSSGLGDYL
ELHIHQGKIGVKFNVGTDDIAIEESNAIINDGKYHVVRFTRSGGNATLQVDSWPVIERYPAGNND
NERLAIARQRIPYRLGRVVDEWLLDKGRQLTIFNSQATIIIGGKEQGQPFQGQLSGLYYNGLKVL
NMAAENDANIAIVGNVRLVGEVPSS
>1I9YA
YDPIHEYVNHELRKRENEFSEHKNVKIFVASYNLNGCSATTKLENWLFPENTPLADIYVVGFQEI
VQLTPQQVISADPAKRREWESCVKRLLNGKCTSGPGYVQLRSGQLVGTALMIFCKESCLPSIKNV
EGTVKKTGLGGVSGNKGAVAIRFDYEDTGLCFITSHLAAGYTNYDERDHDYRTIASGLRFRRGRS
IFNHDYVVWFGDFNYRISLTYEEVVPCIAQGKLSYLFEYDQLNKQMLTGKVFPFFSELPITFPPT
YKFDIGTDIYDTSDKHRVPAWTDRILYRGELVPHSYQSVPLYYSDHRPIYATYEANIVKVDREKK
KILFEELYNQRKQEVRDASQTS
>1OAIA
PTLSPEQQEMLQAFSTQSGMNLEWSQKCLQDNNWDYTRSAQAFTHLKAKGEIPEVAFMK
>3C1JA
APAVADKADNAFMMICTALVLFMTIPGIALFYGGLIRGKNVLSMLTQVTVTFALVCILWVVYGYS
LAFGEGNNFFGNINWLMLKNIELTAVMGSIYQYIHVAFQGSAACITVGLIVGALAERIRFSAVLI
FVVVWLTLSYIPIAHMVWGGGLLASHGALDFAGGTVVHINAAIAGLVGAYLIGKRVGFGKEAFKP
HNLPMVFTGTAILYIGWFGANAGSAGTANEIAALAFVNTVVATAAAILGWIFGEWALRGKPSLLG
ACSGAIAGLVGVTPACGYIGVGGALIIGVVAGLAGLWGVTMLKRLLRVDDPCDVFGVHGVCGIVG
CIMTGIFAASSLGGVGFAEGVTMGHQLLVQLESIAITIVWSGVVAFIGYKLADLTVGLRVPEEQE
REGLDVNSHGENAYNADQAQQPAQADLEHHHHHH
>2C15A
MSFTPANRAYPYTRLRRNRRDDFSRRLVRENVLTVDDLILPVFVLDGVNQRESIPSMPGVERLSI
DQLLIEAEEWVALGIPALALFPVTPVEKKSLDAAEAYNPEGIAQRATRALRERFPELGIITDVAL
DPFTTHGQDGILDDDGYVLNDVSIDVLVRQALSHAEAGAQVVAPSDMMDGRIGAIREALESAGHT

NVRVMAYSAKYASAYYGPFRDAVGSASNLGKGNKATYQMDPANSDEALHEVAADLAEGADMVMVX
PGMPYLDIVRRVKDEFRAPTFVYQVSGEYAMHMGAIQNGWLAESVILESLTAFKRAGADGILTYF
AKQAAEQLRRGR
>1CHMA
QMPKTLRIRNGDKVRSTFSAQEYANRQARLRAHLAAENIDAAIFTSYHNINYYSDFLYCSFGRPY
ALVVTEDDVISISANIDGGQPWRRTVGTDNIVYTDWQRDNYFAAIQQALPKARRIGIEHDHLNLQ
NRDKLAARYPDAELVDVAAACMRMRMIKSAEEHVMIRHGARIADIGGAAVVEALGDQVPEYEVAL
HATQAMVRAIADTFEDVELMDTWTWFQSGINTDGAHNPVTTRKVNKGDILSLNCFPMIAGYYTAL
ERTLFLDHCSDDHLRLWQVNVEVHEAGLKLIKPGARCSDIARELNEIFLKHDVLQYRTFGYGHSF
GTLSHYYGREAGLELREDIDTVLEPGMVVSMEPMIMLPEGLPGAGGYREHDILIVNENGAENITK
FPYGPEKNIIR
>3CZVA
GSMSRLSWGYREHNGPIHWKEFFPIADGDQQSPIEIKTKEVKYDSSLRPLSIKYDPSSAKIISNS
GHSFNVDFDDTENKSVLRGGPLTGSYRLRQVHLHWGSADDHGSEHIVDGVSYAAELHVVHWNSDK
YPSFVEAAHEPDGLAVLGVFLQIGEPNSQLQKITDTLDSIKEKGKQTRFTNFDLLSLLPPSWDYW
TYPGSLTVPPLLESVTWIVLKQPINISSQQLAKFRSLLCTAEGEAAAFLVSNHRPPQPLKGRKVR
ASFH
>1CZYA
AMADLEQKVLEMEASTYDGVFIWKISDFPRKRQEAVAGRIPAIFSPAFYTSRYGYKMCLRIYLNG
DGTGRGTHLSLFFVVMKGPNDALLRWPFNQKVTLMLLDQNNREHVIDAFRPDVTSSSFQRPVNDM
NIASGCPLFCPVSKMEAKNSYVRDDAIFIKAIVDLTGL
>2V5IA
MVSVGDAAFRQEANKKFKYSVKLSDYSTLQDAVTDAVDGLLIDINYNFTDGESVDFXGKILTINC
KAKFIGDGALIFNNMGPGSVINQPFMESKTTPWVIFPWDADGKWITDAALVAATLKQSKIEGYQP
GVNDWVKFPGLEALLPQNVKDQHIAATLDIRSASRVEIRNAGGLMAAYLFRSCHHCKVIDSDSII
GGKDGIITFENLSGDWGLGNYVIGGRVHYGSGSGVQFLRNNGGESHNGGVIGVTSWRAGESGFKT
YQGSVGGGTARNYNLQFRDSVALSPVWDGFDLGSDPGMAPEPDRPGDLPVSEYPFHQLPNNHLVD
NILVMNSLGVGLGMDGSGGYVSNVTVQDCAGAGMLAHTYNRVFSNITVIDCNYLNFDSDQIIIIG
DCIVNGIRAAGIKPQPSNGLVISAPNSTISGLVGNVPPDKILVGNLLDPVLGQSRVIGFNSDTAE
LALRINKLSATLDSGALRSHLNGYAGSGSAWTELTALSGSTPNAVSLKVNRGDYKTTEIPISGTV
LPDEGVLDINTMSLYLDAGALWALIRLPDGSKTRMKLSV
>1YCLA
PSVESFELDHNAVVAPYVRHCGVHKVGTDGVVNKFDIRFCQPNKQAMKPDTIHTLEHLLAFTIRS
HAEKYDHFDIIDISPMGAQTGYYLVVSGEPTSAEIVDLLEDTMKEAVEITEIPAANEKQCGQAKL
HDLEGAKRLMRFWLSQDKEELLKVFG
>2G8FA
GSHMAKEEIIWESLSVDVGSQGNPGIVEYKGVDTKTGEVLFEREPIPIGTNNMGEFLAIVHGLRY
LKERNSRKPIYSDSQTAIKWVKDKKAKSTLVRNEETALIWKLVDEAEEWLNTHTYETPILKWQTD
KWGAIKADYGRK
>1G8KB
RTTLAYPATAVSVAKNLAANEPVSFTYPDTSSPCVAVKLGAPVPGGVGPDDDIVAYSVLCTHMGC
PTSYDSSSKTFSCPCHFTEFDAEKAGQMICGEATADLPRVLLRYDAASDALTAVGVDGLIYGRQA
NVI
>2NS9A

SLRLHAGVWGLKVRYEGSFEVSKTPEEVFEFLTDPKRFSRAFPGFKSVEVEDGSFTIELRLSLGP
LRGDARVRASFEDLEKPSKATVKGSGRGAGSTLDFTLRFAVEPSGGGSRVSWVFEGNVGGLAASM
GGRVLDSLARRMINDVISGVKRELGEA
>2V9MA
MQNITQSWFVQGMIKATTDAWLKGWDERNGGNLTLRLDDADIAPYHDNFHQQPRYIPLSQPMPLL
ANTPFIVTGSGKFFRNVQLDPMANLGIVKVDSDGAGYHILWGLFNEAVPTSELPAHFLSHCERIK
ATNGKDRVIMHCHATNLIALTYVLENDTAVFTRQLWEGSTECLVVFPDGVGILPWMVPGTDAIGQ
ATAQEMQKHSLVLWPFHGVFGSGPTLDETFGLIDTAEKSAQVLVKVYSMGGMKQTISREELIALG
KRFGVTPLASALAL
>2QIQA
AGFRKMAFPSGKVEGCMVQVTCGTTTLNGLWLDDTVYCPRHVICTAEDMLNPNYEDLLIRKSNHS
FLVQAGNVQLRVIGHSMQNCLLRLKVDTSNPKTPKYKFVRIQPGQTFSVLACYNGSPSGVYQCAM
RPNHTIKGSFLNGSCGSVGFNIDYDCVSFCYMHHMELPTGVHAGTDLEGKFYGPFVDRQTAQAAG
TDTTITLNVLAWLYAAVINGDRWFLNRFTTTLNDFNLVAMKYNYEPLTQDHVDILGPLSAQTGIA
VLDMCAALKELLQNGMNGRTILGSTILEDEFTPFDVVRQCS
>2Z25A
TAPSQVLKIRRPDDWHLHLRDGDMLKTVVPYTSEIYGRAIVMPNLAPPVTTVEAAVAYRQRILDA
VPAGHDFTPLMTCYLTDSLDPNELERGFNEGVFTAAKLYPANATVNSSHGVTSVDAIMPVLERME
KIGMPLLVHGEVTHADIDIFDREARFIESVMEPLRQRLTALKVVFEHITTKDAADYVRDGNERLA
ATITPQHLMFNRNHMLVGGVRPHLYCLPILKRNIHQQALRELVASGFNRVFLGTDSAPHARHRKE
SSCGCAGCFNAPTALGSYATVFEEMNALQHFEAFCSVNGPQFYGLPVNDTFIELVREEQQVAESI
ALTDDTLVPFLAGETVRWSVKQ
>3NZNA
SNAVNLFGQKDRGNHVSGVDRGKVIMYGLSTCVWCKKTKKLLTDLGVDFDYVYVDRLEGKEEEEA
VEEVRRFNPSVSFPTTIINDEKAIVGFKEKEIRESLGF
>1YPQA
RVANCSAPCPQDWIWHGENCYLFSSGSFNWEKSQEKCLSLDAKLLKINSTADLDFIQQAISYSSF
PFWMGLSRRNPSYPWLWEDGSPLMPHLFRVRGAVSQTYPSGTCAYIQRGAVYAENCILAAFSICQ
KKANL
>3ENUA
TIEVPVLTFVPVQVSAELENRGCWVKFFDKKNFQGDSLFLSGPATLPRLIGPFGYDWENKVRSVK
VGPRANLTIFDNHNYRDEDKFLDAGANVANLSKEMGFFDNFRSMVLNCI
>3APAA
GSARSSSYSGEYGSGGGKRFSHSGNQLDGPITALRVRVNTYYIVGLQVRYGKVWSDYVGGRNGDL
EEIFLHPGESVIQVSGKYKWYLKKLVFVTDKGRYLSFGKDSGTSFNAVPLHPNTVLRFISGRSGS
LIDAIGLHWDV
>2Q5XA
GIILTKVGYYTIPSMDDLAKITNEKGECIVSDFTIGRKGYGSIYFEGDVNLTNLNLDDIVHIRRK
EVVVYLDDNQKPPVGEGLNRKAEVTLDGVWPTDKTSRCLIKSPDRLADINYEGRLEAVSRKQGAQ
FKEYRPETGSWVFKVSHFAKYGLQD
>2Q66A
KVFGITGPVSTVGATAAENKLNDSLIQELKKEGSFETEQETANRVQVLKILQELAQRFVYEVSKK
KNMSDGMARDAGGKIFTYGSYRLGVHGPGSDIDTLVVVPKHVTREDFFTVFDSLLRERKELDEIA
PVPDAFVPIIKIKFSGISIALICARLDQPQVPLSLTLSDKNLLRNLDEKDLRALNGTRVTDEILE

LVPKPNVFRIALRAIKLWAQRRAVYANIFGFPGGVAWAMLVARICQLYPNACSAVILNRFFIILS
EWNWPQPVILKPIEDGPLQVRVWNPKIYAQDRSHRMPVITPAYPSMCATHNITESTKKVILQEFV
RGVQITNDIFSNKKSWANLFEKNDFFFRYKFYLEITAYTRGSDEQHLKWSGLVESKVRLLVMKLE
VLAGIKIAHPFTKPFESSYCCPTEDDYEMIQDKYGSHKTETALNALKLVTDENKEEESIKDAPKA
YLSTMYIGLDFNIENKKEKVDIHIPCTEFVNLCRSFNEDYGDHKVFNLALRFVKGYDLPDEVFDE
NEKRP
>1PC5A
MAFVVTDNCIKCKYTDCVEVCPVDCFYEGPNFLVIHPDECIDCALCEPECGAQAIFSEDEVPEDM
QEFIQLNAELAEVWPNITEKKDPLPDAEDWDGVKGKLQHLER
>2FW6A
MMSETAPLPSASSALEDKAASAPVVGIIMGSQSDWETMRHADALLTELEIPHETLIVSANRTPDR
LADYARTAAERGLNVIIAGAGGAAHLPGMCAAWTRLPVLGVPVESRALKGMDSLLSIVQMPGGVP
VGTLAIGASGAKNAALLAASILALYNPALAARLETWRALQTASVPNSPITEDK
>2QQRA
GHMQSITAGQKVISKHKNGRFYQCEVVRLTTETFYEVNFDDGSFSDNLYPEDIVSQDCLQFGPPA
EGEVVQVRWTDGQVYGAKFVASHPIQMYQVEFEDGSQLVVKRDDVYTLDEELP
>1BF6A
SFDPTGYTLAHEHLHIDLSGFKNNVDCRLDQYAFICQEMNDLMTRGVRNVIEMTNRYMGRNAQFM
LDVMRETGINVVACTGYYQDAFFPEHVATRSVQELAQEMVDEIEQGIDGTELKAGIIAEIGTSEG
KITPLEEKVFIAAALAHNQTGRPISTHTSFSTMGLEQLALLQAHGVDLSRVTVGHCDLKDNLDNI
LKMIDLGAYVQFDTIGKNSYYPDEKRIAMLHALRDRGLLNRVMLSMDITRRSHLKANGGYGYDYL
LTTFIPQLRQSGFSQADVDVMLRENPSQFFQ
>3AHYA
MHHHHHHMLPKDFQWGFATAAYQIEGAVDQDGRGPSIWDTFCAQPGKIADGSSGVTACDSYNRTA
EDIALLKSLGAKSYRFSISWSRIIPEGGRGDAVNQAGIDHYVKFVDDLLDAGITPFITLFHWDLP
EGLHQRYGGLLNRTEFPLDFENYARVMFRALPKVRNWITFNEPLCSAIPGYGSGTFAPGRQSTSE
PWTVGHNILVAHGRAVKAYRDDFKPASGDGQIGIVLNGDFTYPWDAADPADKEAAERRLEFFTAW
FADPIYLGDYPASMRKQLGDRLPTFTPEERALVHGSNDFYGMNHYTSNYIRHRSSPASADDTVGN
VDVLFTNKQGNCIGPETQSPWLRPCAAGFRDFLVWISKRYGYPPIYVTENGTSIKGESDLPKEKI
LEDDFRVKYYNEYIRAMVTAVELDGVNVKGYFAWSLMDNFEWADGYVTRFGVTYVDYENGQKRFP
KKSAKSLKPLFDELIAAA
>2AHEA
MALSMPLNGLKEEDKEPLIELFVKAGSDGESIGNCPFSQRLFMILWLKGVVFSVTTVDLKRKPAD
LQNLAPGTHPPFITFNSEVKTDVNKIEEFLEEVLCPPKYLKLSPKHPESNTAGMDIFAKFSAYIK
NSRPEANEALERGLLKTLQKLDEYLNSPLPDEIDENSMEDIKFSTRKFLDGNEMTLADCNLLPKL
HIVKVVAKKYRNFDIPKEMTGIWRYLTNAYSRDEFTNTCPSDKEVEIAYSDVAKRLPSKVPKGEF
QHTGGRY
>1OX3A
ADIVLNDLPFVDGPPAEGQSRISWIKNGEEILGADTQYGSEGSMNRPTVSVLRNVEVLDKNIGIL
KTSLETANSDIKTIQEAGYIPEAPRDGQAYVRKDGEWVLLSTFL
>1QZ0A
MRERPHTSGHHGAGEARATAPSTVSPYGPEARAELSSRLTTLRNTLAPATNDPRYLQACGGEKLN
RFRDIQCRRQTAVRADLNANYIQVGNTRTIACQYPLQSQLESHFRMLAENRTPVLAVLASSSEIA
NQRFGMPDYFRQSGTYGSITVESKMTQQVGLGDGIMADMYTLTIREAGQKTISVPVVHVGNWPDQ

TAVSSEVTKALASLVDQTAETKRNMYESKGSSAVADDSKLRPVIHCRAGVGRTAQLIGAMCMNDS
RNSQLSVEDMVSQMRVQRNGIMVQKDEQLDVLIKLAEGQGRPLLNS
>1LT4A
NGDRLYRADSRPPDEIKRSGGLMPRGHNEYFDRGTQMNINLYDHARGTQTGFVRYDDGYVSTKLS
LRSAHLAGQSILSGYSTYYIYVIATAPNMFNVNDVLGVYSPHPYEQEVSALGGIPYSQIYGWYRV
NFGVIDERLHRNREYRDRYYRNLNIAPAEDGYRLAGFPPDHQAWREEPWIHHAPQGCGNSSNSSR
TITRTITGDTCNEETQNLSTIYLREYQSKVKRQIFSDYQSEVDIYNRIRDEL
>1OCBA
YNGNPFEGVQLWANNYYRSEVHTLAIPQITDPALRAAASAVAEVPSFQWLDRNVTVDTLLVQTLS
EIREANQAGANPQYAAQIVVYDLPDRDCAAAASNGEWAIANNGVNNYKAYINRIREILISFSDVR
TILVIEPDSLANMVTNMNVPKCSGAASTYRELTIYALKQLDLPHVAMYMDAGHAGWLGWPANIQP
AAELFAKIYEDAGKPRAVRGLATNVANYNAWSVSSPPPYTSPNPNYDEKHYIEAFRPLLEARGFP
AQFIVDQGRSGKQPTGQKEWGHWCNAIGTGFGMRPTANTGHQYVDAFVWVKPGGECDGTSDTTAA
RYDYHCGLEDALKPAPEAGQWFNEYFIQLLRNANPPF
>3OCCA
MATPHINAEMGDFADVVLMPGDPLRAKFIAETFLQDVREVNNVRGMLGFTGTYKGRKISVMGHGM
GIPSCSIYAKELITDFGVKKIIRVGSCGAVRTDVKLRDVVIGMGACTDSKVNRMRFKDHDYAAIA
DFEMTRNAVDAAKAKGVNVRVGNLFSADLFYTPDPQMFDVMEKYGILGVEMEAAGIYGVAAEFGA
KALTICTVSDHIRTGEQTTAAERQTTFNDMIEIALESVLLGDNA
>1ZCJA
ASGQAKALQYAFFAEKSANKWSTPSGASWKTASAQPVSSVGVLGLGTMGRGIAISFARVGISVVA
VESDPKQLDAAKKIITFTLEKEASRAHQNGQASAKPKLRFSSSTKELSTVDLVVEAVFEDMNLKK
KVFAELSALCKPGAFLCTNTSALNVDDIASSTDRPQLVIGTHFFSPAHVMRLLEVIPSRYSSPTT
IATVMSLSKKIGKIGVVVGNCYGFVGNRMLAPYYNQGFFLLEEGSKPEDVDGVLEEFGFKMGPFR
VSDLAGLDVGWKIRKGQGLTGPSLPPGTPVRKRGNSRYSPLGDMLCEAGRFGQKTGKGWYQYDKP
LGRIHKPDPWLSTFLSQYREVHHIEQRTISKEEILERCLYSLINEAFRILEEGMAARPEHIDVIY
LHGYGWPRHKGGPMFYAASVGLPTVLEKLQKYYRQNPDIPQLEPSDYLRRLVAQGSPPLKEWQSL
AGPHGSKL
>1LVMA
GHHHHHHHGESLFKGPRDYNPISSTICHLTNESDGHTTSLYGIGFGPFIITNKHLFRRNNGTLLV
QSLHGVFKVKNTTTLQQHLIDGRDMIIIRMPKDFPPFPQKLKFREPQREERICLVTTNFQTKSMS
SMVSDTSCTFPSSDGIFWKHWIQTKDGQCGSPLVSTRDGFIVGIHSASNFTNTNNYFTSVPKNFM
ELLTNQEAQQWVSGWRLNADSVLWGGHKVFMDKP
>1E0WA
AESTLGAAAAQSGRYFGTAIASGRLSDSTYTSIAGREFNMVTAENEMKIDATEPQRGQFNFSSAD
RVYNWAVQNGKQVRGHTLAWHSQQPGWMQSLSGSALRQAMIDHINGVMAHYKGKIVQWDVVNEAF
ADGSSGARRDSNLQRSGNDWIEVAFRTARAADPSAKLCYNDYNVENWTWAKTQAMYNMVRDFKQR
GVPIDCVGFQSHFNSGSPYNSNFRTTLQNFAALGVDVAITELDIQGAPASTYANVTNDCLAVSRC
LGITVWGVRDSDSWRSEQTPLLFNNDGSKKAAYTAVLDALNGGDSSEPPADGG
>3G5JA
SNAMSVIKIEKALKLDKVIFVDVRTEGEYEEDHILNAINMPLFKNNEHNEVGTIYKMQGKHEAIQ
KGFDYVSYKLKDIYLQAAELALNYDNIVIYCARGGMRSGSIVNLLSSLGVNVYQLEGGYKAYRNF
VLEY
>3B7AA

MTMEQFLTSLDMIRSGCAPKFKLKTEDLDRLRVGDFNFPPSQDLMCYTKCVALMAGTVNKKGEFN
APKALAQLPHLVPPEMMEMSRKSVEACRDTHKQFKESCERVYQTAKCFSENADGQFMWP
>3B7CA
GMPTDDIVQLLKGQEEAWNRGDLDAYMQGYWQNEQLMLISNGKFRNGWDETLAAYKKNYPDKESL
GELKFTIKEIKMLSNYAAMVVGRWDLKRLKDTPTGVFTLLVEKIDDRWVITMDHSSD
>2AD6A
DADLDKQVNTAGAWPIATGGYYSQHNSPLAQINKSNVKNVKAAWSFSTGVLNGHEGAPLVIGDMM
YVHSAFPNNTYALNLNDPGKIVWQHKPKQDASTKAVMCCDVVDRGLAYGAGQIVKKQANGHLLAL
DAKTGKINWEVEVCDPKVGSTLTQAPFVAKDTVLMGCSGAELGVRGAVNAFDLKTGELKWRAFAT
GSDDSVRLAKDFNSANPHYGQFGLGTKTWEGDAWKIGGGTNWGWYAYDPKLNLFYYGSGNPAPWN
ETMRPGDNKWTMTIWGRDLDTGMAKWGYQKTPHDEWDFAGVNQMVLTDQPVNGKMTPLLSHIDRN
GILYTLNRENGNLIVAEKVDPAVNVFKKVDLKTGTPVRDPEFATRMDHKGTNICPSAMGFHNQGV
DSYDPESRTLYAGLNHICMDWEPFMLPYRAGQFFVGATLAMYPGPNGPTKKEMGQIRAFDLTTGK
AKWTKWEKFAAWGGTLYTKGGLVWYATLDGYLKALDNKDGKELWNFKMPSGGIGSPMTYSFKGKQ
YIGSMYGVGGWPGVGLVFDLTDPSAGLGAVGAFRELQNHTQMGGGLMVFSL
>1BCH1
AIEVKLANMEAEINTLKSKLELTNKLHAFSMGKKSGKKFFVTNHERMPFSKVKALCSELRGTVAI
PRNAEENKAIQEVAKTSAFLGITDEVTEGQFMYVTGGRLTYSNWKKDQPDDWYGHGLGGGEDCVH
IVDNGLWNDISCQASHTAVCEFPA
>1DXKA
SQKVEKTVIKNETGTISISQLNKNVWVHTELGSFNGEAVPSNGLVLNTSKGLVLVDSSWDDKLTK
ELIEMVEKKFQKRVTDVIITHAHADRIGGIKTLKERGIKAHSTALTAELAKKNGYEEPLGDLQTV
TNLKFGNMKVETFYPGKGHTEDNIVVWLPQYNILVGGSLVKSTSAKDLGNVADAYVNEWSTSIEN
VLKRYRNINAVVPGHGEVGDKGLLLHTLDLLK
>3M7OA
MNGVAAALLVWILTSPSSSDHGSENGWPKHTACNSGGLEVVYQSCDPLQDFGLSIDQCSKQIQSN
LNIRFGIILRQDIRKLFLDITLMAKGSSILNYSYPLCEEDQPKFSFCGRRKGEQIYYAGPVNNPG
LDVPQGEYQLLLELYNENRATVACANATVTSS
>3OYYA
MASMKTAQEFRAGQVANINGAPWVIQKAEFNKSGRNAAVVKMKLKNLLTGAGTETVFKADDKLEP
IILDRKEVTYSYFADPLYVFMDSEFNQYEIEKDDLEGVLTFIEDGMTDICEAVFYNDKVISVELP
TTIVRQIAYTEPAVRGDTSGKVMKTARLNNGAELQVSAFCEIGDSIEIDTRTGEYKSRVKA
>1FAZA
APADKPQVLASFTQTSASSQNAWLAANRNQSAWAAYEFDWSTDLCTQAPDNPFGFPFNTACARHD
FGYRNYKAAGSFDANKSRIDSAFYEDMKRVCTGYTGEKNTACNSTAWTYYQAVKIFG
>1ODZA
MRADVKPVTVKLVDSQATMETRSLFAFMQEQRRHSIMFGHQHETTQGLTITRTDGTQSDTFNAVG
DFAAVYGWDTLSIVAPKAEGDIVAQVKKAYARGGIITVSSHFDNPKTDTQKGVWPVGTSWDQTPA
VVDSLPGGAYNPVLNGYLDQVAEWANNLKDEQGRLIPVIFRLYHENTGSWFWWGDKQSTPEQYKQ
LFRYSVEYLRDVKGVRNFLYAYSPNNFWDVTEANYLERYPGDEWVDVLGFDTYGPVADNADWFRN
VVANAALVARMAEARGKIPVISGIGIRAPDIEAGLYDNQWYRKLISGLKADPDAREIAFLLVWRN
APQGVPGPNGTQVPHYWVPANRPENINNGTLEDFQAFYADEFTAFNRDIEQVYQRPTLIVK
>1UWLA
MTDNNNYRDVEIRAPRGNKLTAKSWLTEAPLRMLMNNLDPQVAENPKELVVYGGIGRAARNWECY

DKIVETLTRLEDDETLLVQSGKPVGVFKTHSNAPRVLIANSNLVPHWANWEHFNELDAKGLAMYG
QMTAGSWIYIGSQGIVQGTYETFVEAGRQHYGGSLKGKWVLTAGLGGMGGAQPLAATLAGACSLN
IESQQSRIDFRLETRYVDEQATDLDDALVRIAKYTAEGKAISIALHGNAAEILPELVKRGVRPDM
VTDQTSAHDPLNGYLPAGWTWEQYRDRAQTEPAAVVKAAKQSMAVHVQAMLDFQKQGVPTFDYGN
NIRQMAKEEGVANAFDFPGFVPAYIRPLFCRGVGPFRWAALSGEAEDIYKTDAKVKELIPDDAHL
HRWLDMARERISFQGLPARICWVGLGLRAKLGLAFNEMVRSGELSAPVVIGRDHLDSGSVSSPNR
ETEAMRDGSDAVSDWPLLNALLNTAGGATWVSLHHGGGVGMGFSQHSGMVIVCDGTDEAAERIAR
VLTNDPGTGVMRHADAGYDIAIDCAKEQGLDLPMITG
>1Z82A
MGSDKIHHHHHHMEMRFFVLGAGSWGTVFAQMLHENGEEVILWARRKEIVDLINVSHTSPYVEES
KITVRATNDLEEIKKEDILVIAIPVQYIREHLLRLPVKPSMVLNLSKGIEIKTGKRVSEIVEEIL
GCPYAVLSGPSHAEEVAKKLPTAVTLAGENSKELQKRISTEYFRVYTCEDVVGVEIAGALKNVIA
IAAGILDGFGGWDNAKAALETRGIYEIARFGMFFGADQKTFMGLAGIGDLMVTCNSRYSRNRRFG
ELIARGFNPLKLLESSNQVVEGAFTVKAVMKIAKENKIDMPISEEVYRVVYEGKPPLQSMRDLMR
RSLKDEFWAS
>3GZRA
GGEGTDAIQALIQAYFTAWNTNAPERFAEIFWPDGSWVNVVGMHWRGRDQIVFAHTAFLKTIFKD
CKQELVTIEARTIAPGSALAVVTLIQDAYVTPDGRQMPRAHDRLTLLAVEREGVWRFIHGHNTIV
NPDAANNDPVLRMKPA
>2A33A
MEIKGESMQKSKFRRICVFCGSSQGKKSSYQDAAVDLGNELVSRNIDLVYGGGSIGLMGLVSQAV
HDGGRHVIGIIPKTLMPRELTGETVGEVRAVADMHQRKAEMAKHSDAFIALPGGYGTLEELLEVI
TWAQLGIHDKPVGLLNVDGYYNSLLSFIDKAVEEGFISPTAREIIVSAPTAKELVKKLEEYAPCH
ERVATKLCWEMERIGYSSEE
>1VDWA
MSVKTWRKIAIDIIRDFDHNIMPLFGNPKASETISISPSGDETKVVDKVAENIIISKFKDLGVNV
VSEEIGRIDQGSDYTVVVDPLDGSYNFINGIPFFAVSVAIFHEKDPIYAFIYEPIVERLYEGIPG
KGSYLNGEKIKVRELAEKPSISFYTKGKGTKIIDKVKRTRTLGAIALELAYLARGALDAVVDIRN
YLRPTDIAAGVVIAREAGAIVKDLDGKDVEITFSATEKVNIIAANNEELLETILRSIEK
>2QKFA
MDIKINDITLGNNSPFVLFGGINVLESLDSTLQTCAHYVEVTRKLGIPYIFKASFDKANRSSIHS
YRGVGLEEGLKIFEKVKAEFGIPVITDVHEPHQCQPVAEVCDVIQLPAFLARQTDLVVAMAKTGN
VVNIKKPQFLSPSQMKNIVEKFHEAGNGKLILCERGSSFGYDNLVVDMLGFGVMKQTCGNLPVIF
DVTHSLQTRDAGSAASGGRRAQALDLALAGMATRLAGLFLESHPDPKLAKCDGPSALPLHLLEDF
LIRIKALDDLIKSQPILTIE
>2ZU1A
GPAFEFAVAMMKRNSSTVKTEYGEFTMLGIYDRWAVLPRHAKPGPTILMNDQEVGVLDAKELVDK
DGTNLELTLLKLNRNEKFRDIRGFLAKEEVEVNEAVLAINTSKFPNMYIPVGQVTEYGFLNLGGT
PTKRMLMYNFPTRAGQAGGVLMSTGKVLGIHVGGNGHQGFSAALLKHYFNDEQ
>2GJPA
HHMGTNGTMMQYFEWHLPNDGQHWNRLRDDASNLRNRGITAIWIPPAWKGTSQNDVGYGAYDLYD
LGEFNQKGTVRTKYGTRSQLESAIHALKNNGVQVYGDVVMNHKGGADATENVLAVEVNPNNRNQE
ISGDYTIEAWTKFDFPGRGNTYSDFKWRWYHFDGVDWDQSRQFQNRIYKFRGDGKAWDWEVDSEN
GNYDYLMYADVDMDHPEVVNELRRWGEWYTNTLNLDGFRIDAVKHIKYSFTRDWLTHVRNATGKE

MFAVAEFWKNDLGALENYLNKTNWNHSVFDVPLHYNLYNASNSGGNYDMAKLLNGTVVQKHPMHA
VTFVDNHDSQPGESLESFVQEWFKPLAYALILTREQGYPSVFYGDYYGIPTHSVPAMKAKIDPIL
EARQNFAYGTQHDYFDHHNIIGWTREGNTTHPNSGLATIMSDGPGGEKWMYVGQNKAGQVWHDIT
GNKPGTVTINADGWANFSVNGGSVSIWVKR
>3B5NC
GSIKFTKQSSVASTRNTLKMAQDAERAGMNTLGMLGHQSEQLNNVEGNLDLMKVQNKVADEKVAE
LKKLQ
>1HQGA
MSSKPKPIEIIGAPFSKGQPRGGVEKGPAALRKAGLVEKLKETEYNVRDHGDLAFVDVPNDSPFQ
IVKNPRSVGKANEQLAAVVAETQKNGTISVVLGGDHSMAIGSISGHARVHPDLCVIWVDAHTDIN
TPLTTSSGNLCGQPVAFLLKELKGKFPDVPGFSWVTPCISAKDIVYIGLRDVDPGEHYIIKTLGI
KYFSMTEVDKLGIGKVMEETFSYLLGRKKRPIHLSFDVDGLDPVFTPATGTPVVGGLSYREGLYI
TEEIYKTGLLSGLDIMEVNPTLGKTPEEVTRTVNTAVALTLSCFGTKREGNHKPETDYLKPPK
>2OQYA
MKITDLELHAVGIPRHTGFVNKHVIVKIHTDEGLTGIGEMSDFSHLPLYSVDLHDLKQGLLSILL
GQNPFDLMKINKELTDNFPETMYYYEKGSFIRNGIDNALHDLCAKYLDISVSDFLGGRVKEKIKV
CYPIFRHRFSEEVESNLDVVRQKLEQGFDVFRLYVGKNLDADEEFLSRVKEEFGSRVRIKSYDFS
HLLNWKDAHRAIKRLTKYDLGLEMIESPAPRNDFDGLYQLRLKTDYPISEHVWSFKQQQEMIKKD
AIDIFNISPVFIGGLTSAKKAAYAAEVASKDVVLGTTQELSVGTAAMAHLGCSLTNINHTSDPTG
PELYVGDVVKNRVTYKDGYLYAPDRSVKGLGIELDESLLAKYQVPDLSWDNVTVHQLQDRTADTK
S
>2OQAA
DVSFSLSGSSSTSYSKFIGALRKALPSNGTVYNITLLLSSASGASRYTLMKLSNYDGKAITVAID
VTNVYIMGYLVNSTSYFFNESDAKLASQYVFAGSTIVTLPYSGNYEKLQTAAGKIREKIPLGFPA
LDSAITTLFHYDSTAAAAAFLVIIQTTAESSRFKYIEGQIIMRISKNGVPSLATISLENEWSALS
KQIQLAQTNNGTFKTPVVIMDAGGQRVEIGNVGSKVVTKNIQLLLN
>3QD5A
GPGSMAATPLPPLRLAIACDDAGVSYKEALKAHLSDNPLVSSITDVGVTSTTDKTAYPHVAIQAA
QLIKDGKVDRALMICGTGLGVAISANKVPGIRAVTAHDTFSVERAILSNDAQVLCFGQRVIGIEL
AKRLAGEWLTYRFDQKSASAQKVQAISDYEKKFVEVN
>3D1RA
GMRRELAIEFSRVTESAALAGYKWLGRGDKNTADGAAVNAMRIMLNQVNIDGTIVIGEGEIAEAP
MLYIGEKVGTGRGDAVDIAVDPIEGTRMTAMGQANALAVLAVGDKGCFLNAPDMYMEKLIVGPGA
KGTIDLNLPLADNLRNVAAALGKPLSELTVTILAKPRHDAVIAEMQQLGVRVFAIPDGDVAASIL
TCMPDSEVDVLYGIGGAPEGVVSAAVIRALDGDMNGRLLARHDVKGDNEENRRIGEQELARCKAM
GIEAGKVLRLGDMARSDNVIFSATGITKGDLLEGISRKGNIATTETLLIRGKSRTIRRIQSIHYL
DRKDPEMQVHIL
>1RWIB
RPSWSPTQASGQTVLPFTGIDFRLSPSGVAVDSAGNVYVTSEGMYGRVVKLATGSTGTTVLPFNG
LYQPQGLAVDGAGTVYVTDFNNRVVTLAAGSNNQTVLPFDGLNYPEGLAVDTQGAVYVADRGNNR
VVKLAAGSKTQTVLPFTGLNDPDGVAVDNSGNVYVTDTDNNRVVKLEAESNNQVVLPFTDITAPW
GIAVDEAGTVYVTEHNTNQVVKLLAGSTTSTVLPFTGLNTPLAVAVDSDRTVYVADRGNDRVVKL
TSLEHHHHHH
>1TEJB

NSVNPCCDPQTCKPIEGKHCISGPCCENCYFLRSGTICQRARGDGNNDYCTGITPDCPRNRYNV

>3EWDA

MNILQEPIDFLKKEELKNIDLSQMSKKERYKIWKRIPKCELHCHLDLCFSADFFVSCIRKYNLQP
NLSDEEVLDYYLFAKGGKSLGEFVEKAIKVADIFHDYEVIEDLAKHAVFNKYKEGVVLMEFRYSP
TFVAFKYNLDIELIHQAIVKGIKEVVELLDHKIHVALMCIGTGHEAANIKASADFCLKHKADFVG
FDHGGHEVDLKEYKEIFDYVRESGVPLSVHAGEDVTLPNLNTLYSAIQVLKVERIGHGIRVAESQ
ELIDMVKEKNILLEVCPISNVLLKNAKSMDTHPIRQLYDAGVKVSVNSDDPGMFLTNINDDYEEL
YTHLNFTLEDFMKMNEWALEKSFMDSNIKDKIKNLYFKGEFEAYV

>1EW4A

MNDSEFHRLADQLWLTIEERLDDWDGDSDIDCEINGGVLTITFENGSKIIINRQEPLHQVWLATK
QGGYHFDLKGDEWICDRSGETFWDLLEQAATQQAGETVSFR

>1H6WA

LSYPNATESVYGLTRYSTNDEAIAGVNNESSITPAKFTVALNNVFETRVSTESSNGVIKISSLPQ
ALAGADDTTAMTPLKTQQLAVKLIAQIAPSKNAATESEQGVIQLATVAQARQGTLREGYAISPYT
FMNSTATEEYKGVIKLGTQSEVNSNNASVAVTGATLNGRGSTTSMRGVVKLTTTAGSQSGGDASS
ALAWNADVIHQRGGQTINGTLRINNTLTIASGGANITGTVNMTGGYIQGKRVVTQNEIDRTIPVG
AIMMWAADSLPSDAWRFCHGGTVSASDCPLYASRIGTRYGGSSSNPGLPDMR

>2CB9A

MARSQLSAAGEQHVIQLNQQGGKNLFCFPPISGFGIYFKDLALQLNHKAAVYGFHFIEEDSRIEQ
YVSRITEIQPEGPYVLLGYSAGGNLAFEVVQAMEQKGLEVSDFIIVDAYKKDQSITADTENDDSA
AYLPEAVRETVMQKKRCYQEYWAQLINEGRIKSNIHFIEAGIQTETSGAMVLQKWQDAAEEGYAE
YTGYGAHKDMLEGEFAEKNANIILNILDKINSDQKVLPNKHGSHHHHHH

>1GXJA

EKEMIERDMREYRGFSRAVRAVFEEKERFPGLVDVVSNLIEVDEKYSLAVSVLLGGTAQNIVVRN
VDTAKAIVEFLKQNEAGRVTILPLDLIDGSFNRISGLENERGFVGYAVDLVKFPSDLEVLGGFLF
GNSVVVETLDDAIRMKKKYRLNTRIATLDGELISGRGAITGGREERSSNVFERRIK

>1VLCA

MGSDKIHHHHHHMKIAVLPGDGIGPEVVREALKVLEVVEKKTGKTFEKVFGHIGGDAIDRFGEPL
PEETKKICLEADAIFLGSVGGPKWDDLPPEKRPEIGGLLALRKMLNLYANIRPIKVYRSLVHVSP
LKEKVIGSGVDLVTVRELSYGVYYGQPRGLDEEKGFDTMIYDRKTVERIARTAFEIAKNRRKKVT
SVDKANVLYSSMLWRKVVNEVAREYPDVELTHIYVDNAAMQLILKPSQFDVILTTNMFGDILSDE
SAALPGSLGLLPSASFGDKNLYEPAGGSAPDIAGKNIANPIAQILSLAMMLEHSFGMVEEARKIE
RAVELVIEEGYRTRDIAEDPEKAVSTSQMGDLICKKLEEIW

>2VLPB

MESKRNKPGKATGKGKPVGDKWLDDAGKDSGAPIPDRIADKLRDKEFKSFDDFAKAVWEEVSKDP
ELSKNLNPSNKSSVSKGYSPFTPKNQQVGGRKVYELHHDKPISQGGEVYDMDNIRVTTPKRHIDI
HRGK

>2Y88A

MPLILLPAVNVVEGRAVRLVQGKAGSQTEYGSAVDAALGWQRDGAEWIHLVDLDAAFGRGSNHEL
LAEVVGKLDVQVELSGGIRDDESLAAALATGCARVNVGTAALENPQWCARVIGEHGDQVAVGLDV
QIIDGEHRLRGRGWETDGGDLWDVLERLDSEGCSRFVVTDITKDGTLGGPNLDLLAGVADRTDAP
VIASGGVSSLDDLRAIATLTHRGVEGAIVGKALYARRFTLPQALAAVRD

>3DV9A

SNAMFKEAINNYLHTHGYESIDLKAVLFDMDGVLFDSMPNHAESWHKIMKRFGFGLSREEAYMHE

GRTGASTINIVSRRERGHDATEEEIKAIYQAKTEEFNKCPKAERMPGALEVLTKIKSEGLTPMVV
TGSGQTSLLDRLNHNFPGIFQANLMVTAFDVKYGKPNPEPYLMALKKGGFKPNEALVIENAPLGV
QAGVAAGIFTIAVNTGPLHDNVLLNEGANLLFHSMPDFNKNWETLQSALKQD
>2QRJA
MGHHHHHHHHHHSSGHIEGRHMAAVTLHLRAETKPLEARAALTPTTVKKLIAKGFKIYVEDSPQS
TFNINEYRQAGAIIVPAGSWKTAPRDRIIIGLKEMPETDTFPLVHEHIQFAHCYKDQAGWQNVLM
RFIKGHGTLYDLEFLENDQGRRVAAFGFYAGFAGAALGVRDWAFKQTHSDDEDLPAVSPYPNEKA
LVKDVTKDYKEALATGARKPTVLIIGALGRCGSGAIDLLHKVGIPDANILKWDIKETSRGGPFDE
IPQADIFINCIYLSKPIAPFTNMEKLNNPNRRLRTVVDVSADTTNPHNPIPIYTVATVFNKPTVL
VPTTAGPKLSVISIDHLPSLLPREASEFFSHDLLPSLELLPQRKTAPVWVRAKKLFDRHCARVKR
SSRL
>1GQGA
DTSSLIVEDAPDHVRPYVIRHYSHARAVTVDTQLYRFYVTGPSSGYAFTLMGTNAPHSDALGVLP
HIHQKHYENFYCNKGSFQLWAQSGNETQQTRVLSSGDYGSVPRNVTHTFQIQDPDTEMTGVIVPG
GFEDLFYYLGTNATDTTHTPYIPSSSDSSSTTGPDSSTISTLQSFDVYAELSFTPRTDTVNGTAP
ANTVWHTGANALASTAGDPYFIANGWGPKYLNSQYGYQIVAPFVTATQAQDTNYTLSTISMSTTP
STVTVPTWSFPGACAFQVQEGRVVVQIGDYAATELGSGDVAFIPGGVEFKYYSEAYFSKVLFVSS
GSDGLDQNLVNGGEEWSSVSFPADW
>3MSUA
SNAMEVMLMSKYATLKYADKNIEIELPVYSPSLGNDCIDVSSLVKHGIFTYDPGFMSTAACESKI
TYIDGGKGVLLHRGYPIEEWTQKSNYRTLCYALIYGELPTDEQVKSFRQEIINKMPVCEHVKAAI
AAMPQHTHPMSSLIAGVNVLAAEHIHNGQKESQDEVAKNIVAKIATIAAMAYRHNHGKKFLEPKM
EYGYAENFLYMMFADDESYKPDELHIKAMDTIFMLHADHEQNASTSTVRLSGSTGNSPYAAIIAG
ITALWGPAHGGANEAVLKMLSEIGSTENIDKYIAKAKDKDDPFRLMGFGHRVYKNTDPRATAMKK
NCEEILAKLGHSDNPLLTVAKKLEEIALQDEFFIERKLFSNVDFYSGIILKAMGIPEDMFTAIFA
LARTSGWISQWIEMVNDPAQKIGRPRQLYTGATNRNF
>1KTBA
LENGLARTPPMGWLAWERFRCNVNCREDPRQCISEMLFMEMADRIAEDGWRELGYKYINIDDCWA
AKQRDAEGRLVPDPERFPRGIKALADYVHARGLKLGIYGDLGRLTCGGYPGTTLDRVEQDAQTFA
EWGVDMLKLDGCYSSGKEQAQGYPQMARALNATGRPIVYSCSWPAYQGGLPPKVNYTLLGEICNL
WRNYDDIQDSWDSVLSIVDWFFTNQDVLQPFAGPGHWNDPDMLIIGNFGLSYEQSRSQMALWTIM
AAPLLMSTDLRTISPSAKKILQNRLMIQINQDPLGIQGRRIIKEGSHIEVFLRPLSQAASALVFF
SRRTDMPFRYTTSLAKLGFPMGAAYEVQDVYSGKIISGLKTGDNFTVIINPSGVVMWYLCPKALL
IQQQAPGGPSRLPLL
>2WGVA
MGSITENTSWNKEFSAEAVNGVFVLCKSSSKSCATNDLARASKEYLPASTFKIPNAIIGLETGVI
KNEHQVFKWDGKPRAMKQWERDLTLRGAIQVSATPVFQQIAREVGEVRMQKYLKKFSYGNQNISG
GIDKFWLEGQLRISAVNQVEFLESLYLNKLSASKENQLIVKEALVTEAAPEYLVHSKTGFSGVGT
ESNPGVAWWVGWVEKETEVYFFAFNMDIDNESKLPLRKSIPTKIMESEGIIGG
>3GBXA
SNAMLKREMNIADYDAELWQAMEQEKVRQEEHIELIASENYTSPRVMQAQGSQLTNKYAEGYPGK
RYYGGCEYVDVVEQLAIDRAKELFGADYANVQPHSGSQANFAVYTALLQPGDTVLGMNLAQGGHL
THGSPVNFSGKLYNIVPYGIDESGKIDYDEMAKLAKEHKPKMIIGGFSAYSGVVDWAKMREIADS
IGAYLFVDMAHVAGLIAAGVYPNPVPHAHVVTTTTHKTLAGPRGGLILAKGGDEELYKKLNSAVF

PSAQGGPLMHVIAGKAVALKEAMEPEFKVYQQQVAKNAKAMVEVFLNRGYKVVSGGTENHLFLLD
LVDKNLTGKEADAALGRANITVNKNSVPNDPKSPFVTSGIRIGSPAVTRRGFKEAEVKELAGWMC
DVLDNINDEATIERVKAKVLDICARFPVYA
>1E6WA
AAAVRSVKGLVAVITGGASGLGLSTAKRLVGQGATAVLLDVPNSEGETEAKKLGGNCIFAPANVT
SEKEVQAALTLAKEKFGRIDVAVNCAGIAVAIKTYHEKKNQVHTLEDFQRVINVNLIGTFNVIRL
VAGVMGQNEPDQGGQRGVIINTASVAAFEGQVGQAAYSASKGGIVGMTLPIARDLAPIGIRVVTI
APGLFATPLLTTLPDKVRNFLASQVPFPSRLGDPAEYAHLVQMVIENPFLNGEVIRLDGAIRMQP
>1E6FA
MKSNEHDDCQVTNPSTGHLFDLSSLSGRAGFTAAYSEKGLVYMSICGENENCPPGVGACFGQTRI
SVGKANKRLRYVDQVLQLVYKDGSPCPSKSGLSYKSVISFVCRPEAGPTNRPMLISLDKQTCTLF
FSWHTPLACEQAT
>3A75B
TTHFTVADRWGNVVSYTTTIEQLFGTGIMVPDYGVILNNELTDFDAIPGGANEVQPNKRPLSSMT
PTILFKDDKPVLTVGSPGGATIISSVLQTILYHIEYGMELKAAVEEPRIYTNSMSSYRYEDGVPK
DVLSKLNGMGHKFGTSPVDIGNVQSISIDHENGTFKGVADSSRNGAAIGINLKRK
>3FIRA
MKKIITLFGACALAFSMANADVNLYGPGGPHTALKDIANKYSEKTGVKVNVNFGPQATWFEKAKK
DADILFGASDQSALAIASDFGKDFNVSKIKPLYFREAIILTQKGNPLKIKGLKDLANKKVRIVVP
EGAGESNTSGTGVWEDMIGRTQDIKTIQNFRNNIVAFVPNSGSARKLFAQDQADAWITWIDWSKS
NPDIGTAVAIEKDLVVYRTFNVIAKEGASKETQDFIAYLSSKEAKEIFKKYGWREH
>1Q74A
MSETPRLLFVHAHPDDESLSNGATIAHYTSRGAQVHVVTCTLGEEGEVIGDRWAQLTADHADQLG
GYRIGELTAALRALGVSAPIYLGGAGRWRDSGMAGTDQRSQRRFVDADPRQTVGALVAIIRELRP
HVVVTYDPNGGYGHPDHVHTHTVTTAAVAAAGVGSGTADHPGDPWTVPKFYWTVLGLSALISGAR
ALVPDDLRPEWVLPRADEIAFGYSDDGIDAVVEADEQARAAKVAALAAHATQVVVGPTGRAAALS
NNLALPILADEHYVLAGGSAGARDERGWETDLLAGLGFTASGT
>2CWLA
MFLRIDRLQIELPMPKEQDPNAAAAVQALLGGRFGEMSTLMNYMYQSFNFRGKKALKPYYDLIAN
IATEELGHIELVAATINSLLAKNPGKDLEEGVDPESAPLGFAKDVRNAAHFIAGGANSLVMGAMG
EHWNGEYVFTSGNLILDLLHNFFLEVAARTHKLRVYEMTDNPVAREMIGYLLVRGGVHAAAYGKA
LESLTGVEMTKMLPIPKIDNSKIPEAKKYMDLGFHRNLYRFSPEDYRDLGLIWKGASPEDGTEVV
VVDGPPTGGPVFDAGHDAAEFAPEFHPGELYEIAKKLYEKAK
>3NK6A
SEFMTEPAIITNASDPAVQRIIDVTKHSRASIKTTLIEDTEPLMECIRAGVQFIEVYGSSGTPLD
PALLDLCRQREIPVRLIDVSIVNQLFKAERKAKVFGIARVPRPARLADIAERGGDVVVLDGVKIV
GNIGAIVRTSLALGAAGIVLVDSDLATIADRRLLRASRGYVFSLPVVLADREEAVSFLRDNDIAL
MVLDTDGDLGVKDLGDRADRMALVFGSEKGGPSGLFQEASAGTVSIPMLSSTESLNVSVSVGIAL
HERSARNFAVRRAAAQA
>2W87A
ALLLQEAQAGFCRVDGTIDNNHTGFTGSGFANTNNAQGAAVVWAIDATSSGRRTLTIRYANGGTA
NRNGSLVINGGSNGNYTVSLPTTGAWTTWQTATIDVDLVQGNNIVQLSATTAEGLPNIDSLSVVG
GTVRAGNCG
>2W8TA

TEAAAQPHALPADAPDIAPERDLLSKFDGLIAERQKLLDSGVTDPFAIVMEQVKSPTEAVIRGKD
TILLGTYNYMGMTFDPDVIAAGKEALEKFGSGTCGSRMLNGTFHDHMEVEQALRDFYGTTGAIVF
STGYMANLGIISTLAGKGEYVILDADSHASIYDGCQQGNAEIVRFRHNSVEDLDKRLGRLPKEPA
KLVVLEGVYSMLGDIAPLKEMVAVAKKHGAMVLVDEAHSMGFFGPNGRGVYEAQGLEGQIDFVVG
TFSKSVGTVGGFVVSNHPKFEAVRLACRPYIFTASLPPSVVATATTSIRKLMTAHEKRERLWSNA
RALHGGLKAMGFRLGTETCDSAIVAVMLEDQEQAAMMWQALLDGGLYVNMARPPATPAGTFLLRC
SICAEHTPAQIQTVLGMFQAAGRAVGVIGLEHHHHHH
>1Y0BA
SNAMEALKRKIEEEGVVLSDQVLKVDSFLNHQIDPLLMQRIGDEFASRFAKDGITKIVTIESSGI
APAVMTGLKLGVPVVFARKHKSLTLTDNLLTASVYSFTKQTESQIAVSGTHLSDQDHVLIIDDFL
ANGQAAHGLVSIVKQAGASIAGIGIVIEKSFQPGRDELVKLGYRVESLARIQSLEEGKVSFVQEV
HS
>3K3KA
MGSSHHHHHHSSGLVPRGSHMPSELTPEERSELKNSIAEFHTYQLDPGSCSSLHAQRIHAPPELV
WSIVRRFDKPQTYKHFIKSCSVEQNFEMRVGCTRDVIVISGLPANTSTERLDILDDERRVTGFSI
IGGEHRLTNYKSVTTVHRFEKENRIWTVVLESYVVDMPEGNSEDDTRMFADTVVKLNLQKLATVA
EAMARNSGDGSGSQVT
>3LIDA
MEKYQALLANNVENTAKEALHQLAYTGREYNNIQDQIETISDLLGHSQSLYDYLREPSKANLTIL
ENMWSSVARNQKLYKQIRFLDTSGTEKVRIKYDFKTSIAGPSLILRDKSAREYFKYAQSLDNEQI
SAWGIELERDKGELVYPLSPSLRILMPISVNDVRQGYLVLNVDIEYLSSLLNYSPVRDFHIELVK
HKGFYIASPDESRLYGDIIPERSQFNFSNMYPDIWPRVVSEQAGYSYSGEHLIAFSSIKFVSNEP
LHLIIDLSNEQLSKRATRDINDLIQESLEHHHHHH
>1PGTA
MPPYTVVYFPVRGRCAALRMLLADQGQSWKEEVVTVETWQEGSLKASCLYGQLPKFQDGDLTLYQ
SNTILRHLGRTLGLYGKDQQEAALVDMVNDGVEDLRCKYVSLIYTNYEAGKDDYVKALPGQLKPF
ETLLSQNQGGKTFIVGDQISFADYNLLDLLLIHEVLAPGCLDAFPLLSAYVGRLSARPKLKAFLA
SPEYVNLPINGNGKQ
>1ZLHB
NECVSKGFGCLPQSDCPQEARLSYGGCSTVCCDLSKLTGCKGKGGECNPLDRQCKELQAESASCG
KGQKCCVWLH
>3CIFA
MGSSHHHHHHSSGLVPRGSHMTATLGINGFGRIGRLVLRACMERNDITVVAINDPFMDVEYMAYL
LKYDSVHGNFNGTVEVSGKDLCINGKVVKVFQAKDPAEIPWGASGAQIVCESTGVFTTEEKASLH
LKGGAKKVIISAPPKDNVPMYVMGVNNTEYDPSKFNVISNASSTTNCLAPLAKIINDKFGIVEGL
MTTVHSLTANQLTVDGPSKGGKDWRAGRCAGNNIIPASTGAAKAVGKVIPALNGKLTGMAIRVPT
PDVSVVDLTCKLAKPASIEEIYQAVKEASNGPMKGIMGYTSDDVVSTDFIGCKYSSILDKNACIA
LNDSFVKLISWYDNESGYSNRLVDLAVYVASRGL
>2GAIA
MAKKVKKYIVVESPAKAKTIKSILGNEYEVFASMGHIIDLPKSKFGVDLEKDFEPEFAVIKGKEK
VVEKLKDLAKKGELLIASDMDREGEAIAWHIARVTNTLGRKNRIVFSEITPRVIREAVKNPREID
MKKVRAQLARRILDRIVGYSLSPVLWRNFKSNLSAGRVQSATLKLVCDREREILRFVPKKYHRIT
VNFDGLTAEIDVKEKKFFDAETLKEIQSIDELVVEEKKVSVKKFAPPEPFKTSTLQQEAYSKLGF
SVSKTMMIAQQLYEGVETKDGHIAFITYMRTDSTRVSDYAKEEARNLITEVFGEEYVGSKRERRK

SNAKIQDAHEAIRPTNVFMTPEEAGKYLNSDQKKLYELIWKRFLASQMKPSQYEETRFVLRTKDG
KYRFKGTVLKKIFDGYEKVWKTERNTGEFPFEEGESVKPVVVKIEEQETKPKPRYTEGSLVKEME
RLGIGRPSTYASTIKLLLNRGYIKKIRGYLYPTIVGSVVMDYLEKKYSDVVSVSFTAEMEKDLDE
VEQGKKTDKIVLREFYESFSSVFDRNDRIVVDFPTNQKCSCGKEMRLSFGKYGFYLKCECGKTRS
VKNDEIAVIDDGKIFLGRKDSESGSPDGRSVEGKGNLSEKRRKGKKGS
>2GAKA
PEFFSVRHLELAGDDPYSNVNCTKILQGDPEEIQKVKLEILTVQFKKRPRWTPHDYINMTRDCAS
FIRTRKYIVEPLTKEEVGFPIAYSIVVHHKIEMLDRLLRAIYMPQNFYCIHVDRKAEESFLAAVQ
GIASCFDNVFVASQLESVVYASWTRVKADLNCMKDLYRMNANWKYLINLCGMDFPIKTNLEIVRK
LKCSTGENNLETEKMPPNKEERWKKRYAVVDGKLTNTGIVKAPPPLKTPLFSGSAYFVVTREYVG
YVLENENIQKLMEWAQDTYSPDEFLWATIQRIPEVPGSFPSSNKYDLSDMNAIARFVKWQYFEGD
VSNGAPYPPCSGVHVRSVCVFGAGDLSWMLRQHHLFANKFDMDVDPFAIQCLDEHLRRKALENLE
H
>2RLCA
CTGLALETKDGLHLFGRNMDIEYSFNQSIIFIPRNFKCVNKSNKKELTTKYAVLGMGTIFDDYPT
FADGMNEKGLGCAGLNFPVYVSYSKEDIEGKTNIPVYNFLLWVLANFSSVEEVKEALKNANIVDI
PISENIPNTTLHWMISDITGKSIVVEQTKEKLNVFDNNIGVLTNSPTFDWHVANLNQYVGLRYNQ
VPEFKLGDQSLTALGQGTGLVGLPGDFTPASRFIRVAFLRDAMIKNDKDSIDLIEFFHILNNVAM
VRGSTRTVEEKSDLTQYTSCMCLEKGIYYYNTYENNQINAIDMNKENLDGNEIKTYKYNKTLSIN
HVN
>3DR3A
SNAMLNTLIVGASGYAGAELVTYVNRHPHMNITALTVSAQSNDAGKLISDLHPQLKGIVELPLQP
MSDISEFSPGVDVVFLATAHEVSHDLAPQFLEAGCVVFDLSGAFRVNDATFYEKYYGFTHQYPEL
LEQAAYGLAEWCGNKLKEANLIAVPGCYPTAAQLALKPLIDADLLDLNQWPVINATSGVSGAGRK
AAISNSFCEVSLQPYGVFTHRHQPEIATHLGADVIFTPHLGNFPRGILETITCRLKSGVTQAQVA
QALQQAYAHKPLVRLYDKGVPALKNVVGLPFCDIGFAVQGEHLIIVATEDNLLKGAAAQAVQCAN
IRFGYAETQSLI
>2CN3A
ISSQAVTSVPYKWDNVVIGGGGGFMPGIVFNETEKDLIYARAAIGGAYRWDPSTETWIPLLDHFQ
MDEYSYYGVESIATDPVDPNRVYIVAGMYTNDWLPNMGAILRSTDRGETWEKTILPFKMGGNMPG
RSMGERLAIDPNDNRILYLGTRCGNGLWRSTDYGVTWSKVESFPNPGTYIYDPNFDYTKDIIGVV
WVVFDKSSSTPGNPTKTIYVGVADKNESIYRSTDGGVTWKAVPGQPKGLLPHHGVLASNGMLYIT
YGDTCGPYDGNGKGQVWKFNTRTGEWIDITPIPYSSSDNRFCFAGLAVDRQNPDIIMVTSMNAWW
PDEYIFRSTDGGATWKNIWEWGMYPERILHYEIDISAAPWLDWGTEKQLPEINPKLGWMIGDIEI
DPFNSDRMMYVTGATIYGCDNLTDWDRGGKVKIEVKATGIEECAVLDLVSPPEGAPLVSAVGDLV
GFVHDDLKVGPKKMHVPSYSSGTGIDYAELVPNFMALVAKADLYDVKKISFSYDGGRNWFQPPNE
APNSVGGGSVAVAADAKSVIWTPENASPAVTTDNGNSWKVCTNLGMGAVVASDRVNGKKFYAFYN
GKFYISTDGGLTFTDTKAPQLPKSVNKIKAVPGKEGHVWLAAREGGLWRSTDGGYTFEKLSNVDT
AHVVGFGKAAPGQDYMAIYITGKIDNVLGFFRSDDAGKTWVRINDDEHGYGAVDTAITGDPRVYG
RVYIATNGRGIVYGEPASDEPV
>1XA3A
MHHHHHHGSTSLYKKAGSETLYIQGDHLPMPKFGPLAGLRVVFSGIEIAGPFAGQMFAEWGAEVI
WIENVAWADTIRVQPNYPQLSRRNLHALSLNIFKDEGREAFLKLMETTDIFIEASKGPAFARRGI
TDEVLWQHNPKLVIAHLSGFGQYGTEEYTNLPAYNTIAQAFSGYLIQNGDVDQPMPAFPYTADYF

SGLTATTAALAALHKVRETGKGESIDIAMYEVMLRMGQYFMMDYFNGGEMCPRMSKGKDPYYAGC
GLYKCADGYIVMELVGITQIEECFKDIGLAHLLGTPEIPEGTQLIHRIECPYGPLVEEKLDAWLA
THTIAEVKERFAELNIACAKVLTVPELESNPQYVARESITQWQTMDGRTCKGPNIMPKFKNNPGQ
IWRGMPSHGMDTAAILKNIGYSENDIQELVSKGLAKVEDSTHHHHHH
>1GPIA
QQAGTNTAENHPQLQSQQCTTSGGCKPLSTKVVLDSNWRWVHSTSGYTNCYTGNEWDTSLCPDGK
TCAANCALDGADYSGTYGITSTGTALTLKFVTGSNVGSRVYLMADDTHYQLLKLLNQEFTFDVDM
SNLPCGLNGALYLSAMDADGGMSKYPGNKAGAKYGTGYCDSQCPKDIKFINGEANVGNWTETGSN
TGTGSYGTCCSEMDIWEANNDAAAFTPHPCTTTGQTRCSGDDCARNTGLCDGDGCDFNSFRMGDK
TFLGKGMTVDTSKPFTVVTQFLTNDNTSTGTLSEIRRIYIQNGKVIQNSVANIPGVDPVNSITDN
FCAQQKTAFGDTNWFAQKGGLKQMGEALGNGMVLALSIWDDHAANMLWLDSDYPTDKDPSAPGVA
RGTCATTSGVPSDVESQVPNSQVVFSNIKFGDIGSTFSGTS
>1LRIA
TACTASQQTAAYKTLVSILSDASFNQCSTDSGYSMLTAKALPTTAQYKLMCASTACNTMIKKIVT
LNPPNCDLTVPTSGLVLNVYSYANGFSNKCSSL
>2QECA
GMSPTVLPATQADFPKIVDVLVEAFANDPTFLRWIPQPDPGSAKLRALFELQIEKQYAVAGNIDV
ARDSEGEIVGVALWDRPDGNHSAKDQAAMLPRLVSIFGIKAAQVAWTDLSSARFHPKFPHWYLYT
VATSSSARGTGVGSALLNHGIARAGDEAIYLEATSTRAAQLYNRLGFVPLGYIPSDDDGTPELAM
WKPPAMPTV
>1VAJA
MVFKIKDEWGEFLVRLARRAIEEYLKTGKEIEPPKDTPPELWEKMGVFVTLNRYNVPPQTALRGC
IGFPTPIYPLVEATIKAAIYSAVDDPRFPPVKLEEMDNLVVEVSVLTPPELIEGPPEERPRKIKV
GRDGLIVEKGIYSGLLLPQVPVEWGWDEEEFLAETCWKAGLPPDCWLDEDTKVYKFTAEIFEEEY
PRGPIKRKPLVLEHHHHHH
>1D7EA
VAFGSEDIENTLAKMDDGQLDGLAFGAIQLDGDGNILQYNAAEGDITGRDPKQVIGKNFFKDVAP
CTDSPEFYGKFKEGVASGNLNTMFEYTFDYQMTPTKVKVHMKKALSGDSYWVFVKRV
>1YNVX
DVSGTVCLSALPPEATDTLNLIASDGPFPYSQDGVVFQNRESVLPTQSYGYYHEYTVITPGARTR
GTRRIITGEATQEDYYTGDHYATFSLIDKTC
>1XH9A
GNAAAAKKGSEQESVKEFLAKAKEDFLKKWENPAQNTAHLDQFERIKTLGTGSFGRVMLVKHMET
GNHYAMKILDKQKVVKLKEIEHTLNEKRILQAVNFPFLVKLEFSFKDNSNLYMVMEYAPGGEMFS
HLRRIGRFSEPHARFYAAQIVLTFEYLHSLDLIYRDLKPENLMIDQQGYIKVTDFGLAKRVKGRT
WTLCGTPEYLAPEIILSKGYNKAVDWWALGVLIYEMAAGYPPFFADQPIQIYEKIVSGKVRFPSH
FSSDLKDLLRNLLQVDLTKRFGNLKNGVNDIKNHKWFATTDWIAIYQRKVEAPFIPKFKGPGDTS
NFDDYEEEEIRVSINEKCGKEFSEF
>3OIRA
SNADGLEGMDDPDATSKKVVPLGVEIYEINGPFFFGVADRLKGVLDVIEETPKVFILRMRRVPVI
DATGMHALWEFQESCEKRGTILLLSGVSDRLYGALNRFGFIEALGEERVFDHIDKALAYAKLLVE
TAEER
>2V4CA
ADYDLKFGMNAGTSSNEYKAAEMFAKEVKEKSQGKIEISLYPSSQLGDDRAMLKQLKDGSLDFTF

AESARFQLFYPEAAVFALPYVISNYNVAQKALFDTEFGKDLIKKMDKDLGVTLLSQAYNGTRQTT
SNRAINSIADMKGLKLRVPNAATNLAYAKYVGASPTPMAFSEVYLALQTNAVDGQENPLAAVQAQ
KFYEVQKFLAMTNHILNDQLYLVSNETYKELPEDLQKVVKDAAENAAKYHTKLFVDGEKDLVTFF
EKQGVKITHPDLVPFKESMKPYYAEFVKQTGQKGESALKQIEAINPHHH
>1JNRA
MVYYPKKYELYKADEVPTEVVETDILIIGGGFSGCGAAYEAAYWAKLGGLKVTLVEKAAVERSGA
VAQGLSAINTYIDLTGRSERQNTLEDYVRYVTLDMMGLAREDLVADYARHVDGTVHLFEKWGLPI
WKTPDGKYVREGQWQIMIHGESYKPIIAEAAKMAVGEENIYERVFIFELLKDNNDPNAVAGAVGF
SVREPKFYVFKAKAVILATGGATLLFRPRSTGEAAGRTWYAIFDTGSGYYMGLKAGAMLTQFEHR
FIPFRFKDGYGPVGAWFLFFKCKAKNAYGEEYIKTRAAELEKYKPYGAAQPIPTPLRNHQVMLEI
MDGNQPIYMHTEEALAELAGGDKKKLKHIYEEAFEDFLDMTVSQALLWACQNIDPQEQPSEAAPA
EPYIMGSHSGEAGFWVCGPEDLMPEEYAKLFPLKYNRMTTVKGLFAIGDCAGANPHKFSSGSFTE
GRIAAKAAVRFILEQKPNPEIDDAVVEELKKKAYAPMERFMQYKDLSTADDVNPEYILPWQGLVR
LQKIMDEYAAGIATIYKTNEKMLQRALELLAFLKEDLEKLAARDLHELMRAWELVHRVWTAEAHV
RHMLFRKETRWPGYYYRTDYPELNDEEWKCFVCSKYDAEKDEWTFEKVPYVQVIEWSF
>2QM0A
SNAMNTTVEKQQIITSNTEQWKMYSKLEGKEYQIHISKPKQPAPDSGYPVIYVLDGNAFFQTFHE
AVKIQSVRAEKTGVSPAIIVGVGYPIEGAFSGEERCYDFTPSVISKDAPLKPDGKPWPKTGGAHN
FFTFIEEELKPQIEKNFEIDKGKQTLFGHXLGGLFALHILFTNLNAFQNYFISSPSIWWNNKSVL
EKEENLIIELNNAKFETGVFLTVGSLEREHMVVGANELSERLLQVNHDKLKFKFYEAEGENHASV
VPTSLSKGLRFISYV
>2QM6A
MGSSHHHHHHSSGLVPRGSHMASAASYPPIKNTKVGLALSSHPLASEIGQKVLEEGGNAIDAAVA
IGFALAVVHPAAGNIGGGGFAVIHLANGENVALDFREKAPLKATKNMFLDKQGNVVPKLSEDGYL
AAGVPGTVAGMEAMLKKYGTKKLSQLIDPAIKLAENGYAISQRQAETLKEARERFLKYSSSKKYF
FKKGHLDYQEGDLFVQKDLAKTLNQIKTLGAKGFYQGQVAELIEKDMKKNGGIITKEDLASYNVK
WRKPVVGSYRGYKIISMSPPSSGGTHLIQILNVMENADLSALGYGASKNIHIAAEAMRQAYADRS
VYMGDADFVSVPVDKLINKAYAKKIFDTIQPDTVTPSSQIKPGMGQLHEGSN
>3GHJA
MGSSHHHHHHSSGRENLYFQGVPMNIKGLFEVAVKVKNLEKSSQFYTEILGFEAGLLDSARRWNF
LWVSGRAGMVVLQEEKENWQQQHFSFRVEKSEIEPLKKALESKGVSVHGPVNQEWMQAVSLYFAD
PNGHALEFTAL
>2Z58B
NTIRVIVSVDKAKFNPHEVLGIGGHIVYQFKLIPAVVVDVPANAVGKLKKMPWVEKVEFDHQAVL
L
>1CUHA
LPTSNPAQELEARQLGRTTRDDLINGNSASCADVIFIYARGSTETGNLGTLGPSIASNLESAFGK
DGVWIQGVGGAYRATLGDNALPRGTSSAAIREMLGLFQQANTKCPDATLIAGGYSQGAALAAASI
EDLDSAIRDKIAGTVLFGYTKNLQNRGRIPNYPADRTKVFCNTGDLVCTGSLIVAAPHLAYGPDA
EGPAPEFLIEKVRAVRGSA
>1OJQA
AETKNFTDLVEATKWGNSLIKSAKYSSKDKMAIYNYTKNSSPINTPLRSANGDVNKLSENIQEQV
RQLDSTISKSVTPDSVYVYRLLNLDYLSSITGFTREDLHMLQQTNNGQYNEALVSKLNNLMNSRI
YRENGYSSTQLVSGAALAGRPIELKLELPKGTKAAYIDSKELTAYPGQQEVLLPRGTEYAVGSVK

LSDNKRKIIITAVVFKK

>1NIJA

MNPIAVTLLTGFLGAGKTTLLRHILNEQHGYKIAVIENEFGEVSVDDQLIGDRATQIKTLTNGCI
CCSRSNELEDALLDLLDNLDKGNIQFDRLVIECTGMADPGPIIQTFFSHEVLCQRYLLDGVIALV
DAVHADEQMNQFTIAQSQVGYADRILLTKTDVAGEAEKLHERLARINARAPVYTVTHGDIDLGLL
FNTNGFMLEENVVSTKPRFHFIADKQNDISSIVVELDYPVDISEVSRVMENLLLESADKLLRYKG
MLWIDGEPNRLLFQGVQRLYSADWDRPWGDEKPHSTMVFIGIQLPEEEIRAAFAGLRK

>2O2GA

GMDRTLTHQPQEYAVSVSVGEVKLKGNLVIPNGATGIVLFAHGSGSSRYSPRNRYVAEVLQQAGL
ATLLIDLLTQEEEEIDLRTRHLRFDIGLLASRLVGATDWLTHNPDTQHLKVGYFGASTGGGAALV
AAAERPETVQAVVSRGGRPDLAPSALPHVKAPTLLIVGGYDLPVIAMNEDALEQLQTSKRLVIIP
RASHLFEEPGALTAVAQLASEWFMHYLR

>1O2DA

MGSDKIHHHHHHVWEFYMPTDVFFGEKILEKRGNIIDLLGKRALVVTGKSSSKKNGSLDDLKKLL
DETEISYEIFDEVEENPSFDNVMKAVERYRNDSFDFVVGLGGGSPMDFAKAVAVLLKEKDLSVED
LYDREKVKHWLPVVEIPTTAGTGSEVTPYSILTDPEGNKRGCTLMFPVYAFLDPRYTYSMSDELT
LSTGVDALSHAVEGYLSRKSTPPSDALAIEAMKIIHRNLPKAIEGNREARKKMFVASCLAGMVIA
QTGTTLAHALGYPLTTEKGIKHGKATGMVLPFVMEVMKEEIPEKVDTVNHIFGGSLLKFLKELGL
YEKVAVSSEELEKWVEKGSRAKHLKNTPGTFTPEKIRNIYREALGV

>3NJCA

MGHHHHHHSHMKSKFEASIDNLKEIEMNAYAYELIREIVLPDMLGQDYSSMMYWAGKHLARKFPL
ESWEEFPAFFEEAGWGTLTNVSAKKQELEFELEGPIISNRLKHQKEPCFQLEAGFIAEQIQLMND
QIAESYEQVKKRADKVVLTVKWDMKDPV

>3R0NA

MQDVRVQVLPEVRGQLGGTVELPCHLLPPVPGLYISLVTWQRPDAPANHQNVAAFHPKMGPSFPS
PKPGSERLSFVSAKQSTGQDTEAELQDATLALHGLTVEDEGNYTCEFATFPKGSVRGMTWLRV

>1FPZA

MKPPSSIQTSEFDSSDEEPIEDEQTPIHISWLSLSRVNCSQFLGLCALPGCKFKDVRRNVQKDTE
ELKSCGIQDIFVFCTRGELSKYRVPNLLDLYQQCGIITHHHPIADGGTPDIASCCEIMEELTTCL
KNYRKTLIHSYGGLGRSCLVAACLLLYLSDTISPEQAIDSLRDLRGSGAIQTIKQYNYLHEFRDK
LAAHLSSRDSQSRSVSR

>3LX4A

MGSSHHHHHHSQDPNSAAPAAEAPLSHVQQALAELAKPKDDPTRKHVCVQVAPAVRVAIAETLGL
APGATTPKQLAEGLRRLGFDEVFDTLFGADLTIMEEGSELLHRLTEHLEAHPHSDEPLPMFTSCC
PGWIAMLEKSYPDLIPYVSSCKSPQMMLAAMVKSYLAEKKGIAPKDMVMVSIMPCTRKQSEADRD
WFCVDADPTLRQLDHVITTVELGNIFKERGINLAELPEGEWDNPMGVGSGAGVLFGTTGGVMEAA
LRTAYELFTGTPLPRLSLSEVRGMDGIKETNITMVPAPGSKFEELLKHRAAARAEAAAHGTPGPL
AWDGGAGFTSEDGRGGITLRVAVANGLGNAKKLITKMQAGEAKYDFVEIMACPAGCVGGGGQPRS
TDKAITQKRQAALYNLDEKSTLRRSHENPSIRELYDTYLGEPLGHKAHELLHTHYVAGGVEEKDE
KK

>1SCTB

SKVAELANAVVSNADQKDLLRMSWGVLSVDMEGTGLMLMANLFKTSPSAKGKFARLGDVSAGKDN
SKLRGHSITLMYALQNFVDALDDVERLKCVVEKFAVNHINRQISADEFGEIVGPLRQTLKARMGN
YFDEDTVAAWASLVAVVQASL

>3M1MA
SSNFSSERIRYAKWFLEHGFNIIPIDPESKKPVLKEWQKYSHEMPSDEEKQRFLKMIEEGYNYAI
PGGQKGLVILDFESKEKLKAWIGESALEELCRKTLCTNTVHGGIHIYVLSNDIPPHKINPLFEEN
GKGIIDLQSYNSYVLGLGSCVNHLHCTTDKCPWKEQNYTTCYTLYNELKEISKVDLKSLLRFLAE
KGKRLGITLSKTAKEWLEGKKEEEDTVVEFEELRKELVKRDSGKPVEKIKEEICTKSPPKLIKEI
ICENKTYADVNIDRSRGDWHVILYLMKHGVTDPDKILELLPRDSKAKENEKWNTQKYFVITLSKA
WSVVKKYLEA
>3M12A
STHFDVIVVGAGSMGMAAGYQLAKQGVKTLLVDAFDPPHTNGSHHGDTRIIRHAYGEGREYVPLA
LRSQELWYELEKETHHKIFTKTGVLVFGPKGESAFVAETMEAAKEHSLTVDLLEGDEINKRWPGI
TVPENYNAIFEPNSGVLFSENCIRAYRELAEARGAKVLTHTRVEDFDISPDSVKIETANGSYTAD
KLIVSMGAWNSKLLSKLNLDIPLQPYRQVVGFFESDESKYSNDIDFPGFMVEVPNGIYYGFPSFG
GCGLRLGYHTFGQKIDPDTINREFGVYPEDESNLRAFLEEYMPGANGELKRGAVCMYTKTLDEHF
IIDLHPEHSNVVIAAGFSGHGFKFSSGVGEVLSQLALTGKTEHDISIFSINRPALKESLQKTTI
>2VR3A
MRGSHHHHHHGSGTDITNQLTNVTVGIDSGTTVYPHQAGYVKLNYGFSVPNSAVKGDTFKITVPK
ELNLNGVTSTAKVPPIMAGDQVLANGVIDSDGNVIYTFTDYVNTKCDVKATLTMPAYIDPENVKK
TGNVTLATGIGSTTANKTVLVDYEKYGKFYNLSIKGTIDQIDKTNNTYRQTIYVNPSGDNVIAPV
LTGNLKPNTDSNALIDQQNTSIKVYKVDNAADLSESYFVNPENFEDVTNSVNITFPNPNQYKVEF
NTPDDQITTPYIVVVNGHIDPNSKGDLALRSTLYGYNSNIIWRSMSWDNEVAFNNGSGSGDGIDC
PVVP
>3D0KA
SNAMKPADLTNADRIALELGHAGRNAIPYLDDDRNADRPFTLNTYRPYGYTPDRPVVVVQHGVLR
NGADYRDFWIPAADRHKLLIVAPTFSDEIWPGVESYNNGRAFTAAGNPRHVDGWTYALVARVLAN
IRAAEIADCEQVYLFGHSAGGQFVHRLMSSQPHAPFHAVTAANPGWYTLPTFEHRFPEGLDGVGL
TEDHLARLLAYPMTILAGDQDIATDDPNLPSEPAALRQGPHRYARARHYYEAGQRAAAQRGLPFG
WQLQVVPGIGHDGQAMSQVCASLWFDGRMPDAAELARLAGSQSA
>1ZHVA
APRIKLKILNGSYGIARLSASEAIPAWADGGGFVSITRTDDELSIVCLIDRIPQDVRVDPGWSCF
KFQGPFAFDETGIVLSVISPLSTNGIGIFVVSTFDGDHLLVRSNDLEKTADLLANAGHSLLLEHH
HHHH
>3H36A
SNAVELLQVDADLQAEIVGKYNADLQKAVQIEEKKASEIATEAVKEHVTAEYEERYAEHEEHDRI
MRDVAEILEQMEHAEVRRLITEDKVRPD
>1Z3EB
MEKEKVLEMTIEELDLSVRSYNCLKRAGINTVQELANKTEEDMMKVRNLGRKSLEEVKAKLEELG
LGLRKDDG
>2RB8A
MRLDAPSQIEVKDVTDTTALITWMPPSQPVDGFELTYGIKDVPGDRTTIDLTEDENQYSIGNLKP
DTEYEVSLISRRGDMSSNPAKETFTTGLAAALEHHHHHH
>2RBDA
GMGILSGNPQDEPLHYGEVFSTWTYLSTNNGLINGYRSFINHTGDEDLKNLIDEAIQAMQDENHQ
LEELLRSNGVGLPPAPPDRPAARLDDIPVGARFNDPEISATISMDVAKGLVTCSQIIGQSIREDV
ALMFSQFHMAKVQFGGKMLKLNKNKGWLIPPPLHSDRPIKE

>3OBIA
GMPHHQYVLTLSCPDRAGIVSAVSTFLFENGQNILDAQQYNDTESGHFFMRVVFNAAAKVIPLAS
LRTGFGVIAAKFTMGWHMRDRETRRKVMLLVSQSDHCLADILYRWRVGDLHMIPTAIVSNHPRET
FSGFDFGDIPFYHFPVNKDTRRQQEAAITALIAQTHTDLVVLARYMQILSDEMSARLAGRCINIH
HSFLPGFKGAKPYHQAFDRGVKLIGATAHYVTSALDEGPIIDQDVERISHRDTPADLVRKGRDIE
RRVLSRALHYHLDDRVILNGRKTVVFTD
>2BZUA
RIAISNSNRTRSVPSLTTIWSISPTPNCSIYETQDANLFLCLTKNGAHVLGTITIKGLKGALREM
HDNALSLKLPFDNQGNLLNCALESSTWRYQETNAVASNALTFMPNSTVYPRNKTAHPGNMLIQIS
PNITFSVVYNEINSGYAFTFKWSAEPGKPFHPPTAVFCYITEQGSHHHHHH
>1Q16A
MSKFLDRFRYFKQKGETFADGHGQLLNTNRDWEDGYRQRWQHDKIVRSTHGVNCTGSCSWKIYVK
NGLVTWETQQTDYPRTRPDLPNHEPRGCPRGASYSWYLYSANRLKYPMMRKRLMKMWREAKALHS
DPVEAWASIIEDADKAKSFKQARGRGGFVRSSWQEVNELIAASNVYTIKNYGPDRVAGFSPIPAM
SMVSYASGARYLSLIGGTCLSFYDWYCDLPPASPQTWGEQTDVPESADWYNSSYIIAWGSNVPQT
RTPDAHFFTEVRYKGTKTVAVTPDYAEIAKLCDLWLAPKQGTDAAMALAMGHVMLREFHLDNPSQ
YFTDYVRRYTDMPMLVMLEERDGYYAAGRMLRAADLVDALGQENNPEWKTVAFNTNGEMVAPNGS
IGFRWGEKGKWNLEQRDGKTGEETELQLSLLGSQDEIAEVGFPYFGGDGTEHFNKVELENVLLHK
LPVKRLQLADGSTALVTTVYDLTLANYGLERGLNDVNCATSYDDVKAYTPAWAEQITGVSRSQII
RIAREFADNADKTHGRSMIIVGAGLNHWYHLDMNYRGLINMLIFCGCVGQSGGGWAHYVGQEKLR
PQTGWQPLAFALDWQRPARHMNSTSYFYNHSSQWRYETVTAEELLSPMADKSRYTGHLIDFNVRA
ERMGWLPSAPQLGTNPLTIAGEAEKAGMNPVDYTVKSLKEGSIRFAAEQPENGKNHPRNLFIWRS
NLLGSSGKGHEFMLKYLLGTEHGIQGKDLGQQGGVKPEEVDWQDNGLEGKLDLVVTLDFRLSSTC
LYSDIILPTATWYEKDDMNTSDMHPFIHPLSAAVDPAWEAKSDWEIYKAIAKKFSEVCVGHLGKE
TDIVTLPIQHDSAAELAQPLDVKDWKKGECDLIPGKTAPHIMVVERDYPATYERFTSIGPLMEKI
GNGGKGIAWNTQSEMDLLRKLNYTKAEGPAKGQPMLNTAIDAAEMILTLAPETNGQVAVKAWAAL
SEFTGRDHTHLALNKEDEKIRFRDIQAQPRKIISSPTWSGLEDEHVSYNAGYTNVHELIPWRTLS
GRQQLYQDHQWMRDFGESLLVYRPPIDTRSVKEVIGQKSNGNQEKALNFLTPHQKWGIHSTYSDN
LLMLTLGRGGPVVWLSEADAKDLGIADNDWIEVFNSNGALTARAVVSQRVPAGMTMMYHAQERIV
NLPGSEITQQRGGIHNSVTRITPKPTHMIGGYAHLAYGFNYYGTVGSNRDEFVVVRKMKNIDWLD
GEGNDQVQESVK
>3CX5C
MAFRKSNVYLSLVNSYIIDSPQPSSINYWWNMGSLLGLCLVIQIVTGIFMAMHYSSNIELAFSSV
EHIMRDVHNGYILRYLHANGASFFFMVMFMHMAKGLYYGSYRSPRVTLWNVGVIIFILTIATAFL
GYCCVYGQMSHWGATVITNLFSAIPFVGNDIVSWLWGGFSVSNPTIQRFFALHYLVPFIIAAMVI
MHLMALHIHGSSNPLGITGNLDRIPMHSYFIFKDLVTVFLFMLILALFVFYSPNTLGHPDNYIPG
NPLVTPASIVPEWYLLPFYAILRSIPDKLLGVITMFAAILVLLVLPFTDRSVVRGNTFKVLSKFF
FFIFVFNFVLLGQIGACHVEVPYVLMGQIATFIYFAYFLIIVPVISTIENVLFYIGRVNK
>3P1GA
GSILAETHGTRPDLTDQPIPDADYTWYTDGSSFLQEGQRRAGAAVTTETEVIWARALPAGTSAQR
AELIALTQALKMAEGKKLNVYTDSRYAFATAHVHSEGREIKNKNEILALLKALFLPKRLSIIHCP
GHQKGNSAEARGNRMADQAAREAAMKAVLETSTLL
>3EA6A
QGDIGIDNLRNFYTKKDFVDLKDVKDNDTPIANQLQFSNESYDLISESKDFNKFSNFKGKKLDVF

GISYNGQCNTKYIYGGVTATNEYLDKSRNIPINIWINGNHKTISTNKVSTNKKFVTAQEIDVKLR
KYLQEEYNIYGHNGTKKGEEYGHKSKFYSGFNIGKVTFHLNNNDTFSYDLFYTGDDGLPKSFLKI
YEDNKTVESEKFHLDVDISYKETI
>1EZ6A
ATSTKKLHKEPATLIKAIDGDTVKLMYKGQPMVFRLLLVDIPETKHPKKGVEKYGPEAAAFTKKM
VENAKKIEVEFDKGQRTDKYGRGLAYIYADGKMVNEALVRQGLAKVAYVYKGNNTHEQLLRKAEA
QAKKEKLNIWSEDNADSGQ
>1LG7A
VDEMDTHDPHQLRYEKFFFTVKMTVRSNRPFRTYSDVAAAVSHWDHMYIGMAGKRPFYKILAFLG
SSNLKATPAVLADQGQPEYHAHCEGRAYLPHRMGKTPPMLNVPEHFRRPFNIGLYKGTVELTMTI
YDDESLEAAPMIWDHFNSSKFSDFREKALMFGLIVEKKASGAWVLDSVSHFK
>2Y0GA
MAHHHHHHGHHHQLVSKGEELFTGVVPILVELDGDVNGHKFSVSGEGEGDATYGKLTLKFICTTG
KLPVPWPTLVTTLXVQCFSRYPDHMKQHDFFKSAMPEGYVQERTIFFKDDGNYKTRAEVKFEGDT
LVNRIELKGIDFKEDGNILGHKLEYNYNSHNVYIMADKQKNGIKVNFKIRHNIEDGSVQLADHYQ
QNTPIGDGPVLLPDNHYLSTQSALSKDPNEKRDHMVLLEFVTAAGITLGMDELYK
>1K3UB
TTLLNPYFGEFGGMYVPQILMPALNQLEEAFVSAQKDPEFQAQFADLLKNYAGRPTALTKCQNIT
AGTRTTLYLKREDLLHGGAHKTNQVLGQALLAKRMGKSEIIAETGAGQHGVASALASALLGLKCR
IYMGAKDVERQSPNVFRMRLMGAEVIPVHSGSATLKDACNEALRDWSGSYETAHYMLGTAAGPHP
YPTIVREFQRMIGEETKAQILDKEGRLPDAVIACVGGGSNAIGMFADFINDTSVGLIGVEPGGHG
IETGEHGAPLKHGRVGIYFGMKAPMMQTADGQIEESYSISAGLDFPSVGPQHAYLNSIGRADYVS
ITDDEALEAFKTLCRHEGIIPALESSHALAHALKMMREQPEKEQLLVVNLSGRGDKDIFTVHDIL
KARGEI
>3NYSA
MIEFIDLKNQQARIKDKIDAGIQRVLRHGQYILGPEVTELEDRLADFVGAKYCISCANGTDALQI
VQMALGVGPGDEVITPGFTYVATAETVALLGAKPVYVDIDPRTYNLDPQLLEAAITPRTKAIIPV
SLYGQCADFDAINAIASKYGIPVIEDAAQSFGASYKGKRSCNLSTVACTSFFPSAPLGCYGDGGA
IFTNDDELATAIRQIARHGQDRRYHHIRVGVNSRLDTLQAAILLPKLEIFEEEIALRQKVAAEYD
LSLKQVGIGTPFIEVNNISVYAQYTVRMDNRESVQASLKAAGVPTAVHYPIPLNKQPAVADEKAK
LPVGDKAATQVMSLPMHPYLDTASIKIICAALTNLEHHHHHH
>2A6ZA
GSDASKLSSDYSLPDLINTRKVPNNWQTGEQASLEEGRIVLTSNQNSKGSLWLKQGFDLKDSFTM
EWTFRSVGYSGQTDGGISFWFVQDSNIPRDKQLYNGPVNYDGLQLLVDNNGPLGPTLRGQLNDGQ
KPVDKTKIYDQSFASCLMGYQDSSVPSTIRVTYDLEDDNLLKVQVDNKVCFQTRKVRFPSGSYRI
GVTAQNGAVNNNAESFEIFKMQFFNGV
>1G72B
YDGQNCKEPGNCWENKPGYPEKIAGSKYDPKHDPVELNKQEESIKAMDARNAKRIANAKSSGNFV
FDVK
>1ALHA
MPVLENRAAQGDITAPGGARRLTGDQTAALRDSLSDKPAKNIILLIGDGMGDSEITAARNYAEGA
GGFFKGIDALPLTGQYTHYALNKKTGKPDYVTDSAASATAWSTGVKTYNGALGVDIHEKDHPTIL
EMAKAAGLATGNVSTAELQDATPAALVAHVTSRKCYGPSATSEKCPGNALEKGGKGSITEQLLNA
RADVTLGGGAKTFAETATAGEWQGKTLREQAEARGYQLVSDAASLNSVTEANQQKPLLGLFADGN

MPVRWLGPKATYHGNIDKPAVTCTPNPQRNDSVPTLAQMTDKAIELLSKNEKGFFLQVEGASIDK
QDHAANPCGQIGETVDLDEAVQRALEFAKKEGNTLVIVTANHAHASQIVAPDTKAPGLTQALNTK
DGAVMVMSYGNSEEDSQEHTGSQLRIAAYGPHAANVVGLTDQTDLFYTMKAALGLK
>1B51A
ADVPAGVQLADKQTLVRNNGSEVQSLDPHKIEGVPESNVSRDLFEGLLISDVEGHPSPGVAEKWE
NKDFKVWTFHLRENAKWSDGTPVTAHDFVYSWQRLADPNTASPYASYLQYGHIANIDDIIAGKKP
ATDLGVKALDDHTFEVTLSEPVPYFYKLLVHPSVSPVPKSAVEKFGDKWTQPANIVTNGAYKLKN
WVVNERIVLERNPQYWDNAKTVINQVTYLPISSEVTDVNRYRSGEIDMTYNNMPIELFQKLKKEI
PNEVRVDPYLCTYYYEINNQKAPFNDVRVRTALKLALDRDIIVNKVKNQGDLPAYSYTPPYTDGA
KLVEPEWFKWSQQKRNEEAKKLLAEAGFTADKPLTFDLLYNTSDLHKKLAIAVASIWKKNLGVNV
NLENQEWKTFLDTRHQGTFDVARAGWCADYNEPTSFLNTMLSDSSNNTAHYKSPAFDKLIADTLK
VADDTQRSELYAKAEQQLDKDSAIVPVYYYVNARLVKPWVGGYTGKDPLDNIYVKNLYIIKH
>4FHZA
MHHHHHHSSGLVPRGSGMKETAAAKFERQHMDSPDLGTDDDDKAMADIMTRKLTFGRRGAAPGEA
TSLVVFLHGYGADGADLLGLAEPLAPHLPGTAFVAPDAPEPCRANGFGFQWFPIPWLDGSSETAA
AEGMAAAARDLDAFLDERLAEEGLPPEALALVGFSQGTMMALHVAPRRAEEIAGIVGFSGRLLAP
ERLAEEARSKPPVLLVHGDADPVVPFADMSLAGEALAEAGFTTYGHVMKGTGHGIAPDGLSVALA
FLKERLPDACGRTRAPPPPPLRSGC
>4FHRA
GTKFSKEQLRTFQMIHENFGRALSTYLSGRLRTFVDVEISIDQLTYEEFIRSVMIPSFIVIFTGD
VFEGSAIFEMRLDLFYTMLDIIMGGPGENPPNRPPTEIETSIMRKEVTNMLTLLAQAWSDFQYFI
PSIENVETNPQFVQIVPPNEIVLLVTASVSWGEFTSFINVCWPFSLLEPLLEKLSDR
>4FGZA
MTLIENLNSDKTFLENNQYTDEGVKVYEFIFGENYISSGGLEATKKILSDIELNENSKVLDIGSG
LGGGCMYINEKYGAHTHGIDICSNIVNMANERVSGNNKIIFEANDILTKEFPENNFDLIYSRDAI
LHLSLENKNKLFQKCYKWLKPTGTLLITDYCATEKENWDDEFKEYVKQRKYTLITVEEYADILTA
CNFKNVVSKDLSDYWNQLLEVEHKYLHENKEEFLKLFSEKKFISLDDGWSRKIKDSKRKMQRWGY
FKATKN
>4FFXA
GSHMAAGGDHGSPDSYRSPLASRYASPEMCFVFSDRYKFRTWRQLWLWLAEAEQTLGLPITDEQI
REMKSNLENIDFKMAAEEEKRLRHDVMAHVHTFGHCCPKAAGIIHLGATSCYVGDNTDLIIILRNA
LDLLLPKLARVISRLADFAKERASLPTLGFTHFQPAQLTTVGKRCCLWIQDLCMDLQNLKRVRDD
LRFRGVKGTTGTQASFLQLFEGDDHKVEQLDKMVTEKAGFKRAFIITGQTYTRKVDIEVLSVLAS
LGASVHKICTDIRLLANLKEMEEPFEKQQIGSSAMPYKRNPMRSERCCSLARHLMTLVMDPLQTA
SVQWFERTLDDSANRRICLAEAFLTADTILNTLQNISEGLVVYPKVIERRIRQELPFMATENIIM
AMVKAGGSRQDCHEKIRVLSQQAASVVKQEGGDNDLIERIQVDAYFSPIHSQLDHLLDPSSFTGR
ASQQVQRFLEEEVYPLLKPYESVMKVKAELCL
>4AVXA
SMGRGSGTFERLLDKATSQLLLETDWESILQICDLIRQGDTQAKYAVNSIKKKVNDKNPHVALYA
LEVMESVVKNCGQTVHDEVANKQTMEELKDLLKRQVEVNVRNKILYLIQAWAHAFRNEPKYKVVQ
DTYQIMKVEGHVFPEFKESDAMFAAERAPDWVDAEECHRCRVQFGVMTRKHHCRACGQIFCGKCS
SKYSTIPKFGIEKEVRVCEPCYEQLNRKAEG
>4FEIA
QGGPWTPAADWRDAGTHLDLLLDVPGVDAGTLALAEDGGQLTVSGERPGTEHLLRSERPSGRFVR

ELAFPEPVRPASGVASLAGGVLTVRFEKLRPTIDVTA

>4FF1A

MGGSHHHHHHRSESTVTEELKEGIDAVYPSLVGTADSKAEGIKNYFKLSFTLPEEQKSRTVGSEA
PLKDVAQALSSRARYELFTEKETANPAFNGEVIKRYKELMEHGEGIADILRSRLAKFLNTKDVGK
RFAQGTEANRWVGGKLLNIVEQDGDTFKYNEQLLQTAVLAGLQWRLTATSNTAIKDAKDVAAITG
IDQALLPEGLVEQFDTGMTLTEAVSSLAQKIESYWGLSRNPNAPLGYTKGIPTAMAAEILAAFVE
STDVVENIVDMSEIDPDNKKTIGLYTITELDSFDPINSFPTAIEEAVLVNPTEKMFFGDDIPPVA
NTQLRNPAVRNTPEQKAALKAEQATEFYVHTPMVQFYETLGKDRILELMGAGTLNKELLNDNHAK
SLEGKNRSVEDSYNQLFSVIEQVRAQSEDISTVPIHYAYNMTRVGRMQMLGKYNPQSAKLVREAI
LPTKATLDLSNQNNEDFSAFQLGLAQALDIKVHTMTREVMSDELTKLLEGNLKPAIDMMVEFNTT
GSLPENAVDVLNTALGDRKSFVALMALMEYSRYLVAEDKSAFVTPLYVEADGVTNGPINAMMLMT
GGLFTPDWIRNIAKGGLFIGSPNKTMNEHRSTADNNDLYQASTNALMESLGKLRSNYASNMPIQS
QIDSLLSLMDLFLPDINLGENGALELKRGIAKNPLTITIYGSGARGIAGKLVSSVTDAIYERMSD
VLKARAKDPNISAAMAMFGKQAASEAHAEELLARFLKDMETLTSTVPVKRKGVLELQSTGTGAKG
KINPKTYTIKGEQLKALQENMLHFFVEPLRNGITQTVGESLVYSTEQLQKATQIQSVVLEDMFKQ
RVQEKLAEKAKDPTWKKGDFLTQKELNDIQASLNNLAPMIETGSQTFYIAGSENAEVANQVLATN
LDDRMRVPMSIYAPAQAGVAGIPFMTIGTGDGMMMQTLSTMKGAPKNTLKIFDGMNIGLNDITDA
SRKANEAVYTSWQGNPIKNVYESYAKFMKNVDFSKLSPEALEAIGKSALEYDQRENATVDDIANA
ASLIERNLRNIALGVDIRHKVLDKVNLSIDQMAAVGAPYQNNGKIDLSNMTPEQQADELNKLFRE
ELEARKQKVAKAR

>4FE1A

MTISPPEREPKVRVVVDNDPVPTSFEKWAKPGHFDRTLARGPQTTTWIWNLHALAHDFDTHTSDL
EDISRKIFSAHFGHLAVVFIWLSGMYFHGAKFSNYEAWLADPTGIKPSAQVVWPIVGQGILNGDV
GGGFHGIQITSGLFQLWRASGITNEFQLYCTAIGGLVMAGLMLFAGWFHYHKRAPKLEWFQNVES
MLNHHLAGLLGLGSLAWAGHQIHVSLPINKLLDAGVAAKDIPLPHEFILNPSLMAELYPKVDWGF
FSGVIPFFTFNWAAYSDFLTFNGGLNPVTGGLWLSDTAHHHLAIAVLFIIAGHMYRTNWGIGHSL
KEILEAHKGPFTGAGHKGLYEVLTTSWHAQLAINLAMMGSLSIIVAQHMYAMPPYPYLATDYPTQ
LSLFTHHMWIGGFLVVGGAAHGAIFMVRDYDPAMNQNNVLDRVLRHRDAIISHLNWVCIFLGFHS
FGLYVHNDTMRAFGRPQDMFSDTGIQLQPVFAQWVQNLHTLAPGGTAPNAAATASVAFGGDVVAV
GGKVAMMPIVLGTADFMVHHIHAFTIHVTVLILLKGVLFARSSRLIPDKANLGFRFPCDGPGRGG
TCQVSGWDHVFLGLFWMYNCISVVIFHFSWKMQSDVWGTVAPDGTVSHITGGNFAQSAITINGWL
RDFLWAQASQVIGSYGSALSAYGLLFLGAHFIWAFSLMFLFSGRGYWQELIESIVWAHNKLKVAP
AIQPRALSIIQGRAVGVAHYLLGGIATTWAFFLARIISVG

>4AVPA

SMGPTSQRRGSLQLWQFLVALLDDPSNSHFIAWTGRGMEFKLIEPEEVARRWGIQKNRPAMNYDK
LSRSLRYYYEKGIMQKVAGERYVYKFVCDPEALFSMAFSDN

>4FD5A

MLDSKLNNIRFETISSKYYDDVIEHLRQTFFADEPLNKAVNLTRPGQGHPLLEQHSLSTLKDNVS
IMAISNDGDIAGVALNGILYGNTDIEKSREKLNEIQDESFKKIFKLLYEQNLKINLFKQFDVDKI
FEIRILSVDSRFRGKGLAKKLIEKSEELALDRGFQVMKTDATGAFSQRVVSSLGFITKCEINYTD
YLDENGEQIFVVDPPHEKLKIMCKVIN

>4F9KA

MSGLNDIFEAQKIEWHEHHHHHHENLYFQSHMEDESLKGCELYVQLHGIQQVLKDCIVHLCISKP
ERPMKFLREHFEKLEKEENRQILARQKSNS

>4F99B
GPGTRTGRLKKPFVKVEDMSQLYRPFYLQLTNMPFINYSIQKPCSPFDVDKPSSMQKQTQVKLRI
QTDGDKYGGTSIQLQLKEKKKKGYCECCLQKYEDLETHLLSEQHRNFAQSNQYQVVDDIVSKLVF
DFVEYEKDTPKKKR
>4F8CA
MAHHHHHHSSGLEVLFQGPPVSHSINNPSIQHVQDFATLSARSLRANVLLNSDDHSVPIHAKNPS
ELLEAIDNNISQTAQDWGVSIQEVEVILGSSKRIIEPVAGVTANTIMKLFLDNDIFSYSFEKGQS
LSLSQLQERLASLPAHKNFILRVNDGGLGHAYVIDFPATTNPSRDAFLYQSDLGEGVTREVRFED
WMTQKASHPISLDDINTHFIGIAQDQIDLAHIAKLFDVDGNVKMLRADHLISHKTSEFNFQLFEY
DLKNLENNMSIIKTH
>4F84A
MGSSHHHHHHSSGLVPRGSHMAAASAPVPGPGGASSTARGRIPAPATPYQEDIARYWNNEARPVN
LRLGDVDGLYHHHYGIGAVDHAALGDPGDGGYEARLIAELHRLESAQAEFLLDHLGPVGPGDTLV
DAGCGRGGSMVMAHQRFGCKVEGVTLSAAQAEFGNRRARELGIDDHVRSRVCNMLDTPFEKGTVA
ASWNNESSMYVDLHDVFAEHSRFLRVGGRYVTVTGCWNPRYGQPSKWVSQINAHFECNIHSRREY
LRAMADNRLVPQTVVDLTPETLPYWELRATSSLVTGIEEAFIESYRDGSFQYVLIAADRV
>4F6OA
EQAKAQLSNGYNNPNVNASNMYGPPQNMSLPPPQTQTIQGTDQPYQYSQCTGRRKALIIGINYIG
SKNQLRGCINDAHNIFNFLTNGYGYSSDDIVILTDDQNDLVRVPTRANMIRAMQWLVKDAQPNDS
LFLHYSGHGGQTEDLDGDEEDGMDDVIYPVDFETQGPIIDDEMHDIMVKPLQQGVRLTALFDSCH
SGTVLDLPYTYSTKGIIKEPNIWKDVGQDGLQAAISYATGNRAALIGSLGSIFKTVKGGMGNNVD
RERVRQIKFSAADVVMLSGSKDNQTSADAVEDGQNTGAMSHAFIKVMTLQPQQSYLSLLQNMRKE
LAGKYSQKPQLSSSHPIDVNLQFIM
>2LT5A
RPCKYKLKKSTNKFCVTCENQAPVHFVGVGSCGSGGSGIFLETSLSAGSDWLTFQKKHITNTRDV
DCDNIMSTNLFHCKDKNTFIYSRPEPVKAICKGIIASKNVLTTSEFYLSDCNVTS
>4F52B
GSMDVDTPSGTNSGAGKKRFEVKKWNAVALWAWDIVVDNCAICRNHIMDLCIECQANQASATSEE
CTVAWGVCNHAFHFHCISRWLKTRQVCPLDNREWEFQKYGH
>4F52E
GSMAVEELQSIIKRCQILEEQDFKEEDFGLFQLAGQRCIEEGHTDQLLEIIQNEKNKVIIKNMGW
NLVGPVVRCLLCKDKEDSKRKVYFLIFDLLVKLCNPKELLLGLLELIEEPSGKQISQSILLLLQP
LQTVIQKLHNKAYSIGLALSTLWNQLSLLPVPYSKEQIQMDDYGLCQCCKALIEFTKPFVEEVID
NKENSLENEKLKDELLKFCFKSLKCPLLTAQFFEQSEEGGNDPFRYFASEIIGFLSAIGHPFPKM
IFNHGRKKRTWNYLEFEEEENKQLADSMASLAYLVFVQGIHIDQLPMVLSPLYLLQFNMGHIEVF
LQRTEESVISKGLELLENSLLRIEDNSLLYQYLEIKSFLTVPQGLVKVMTLCPIETLRKKSLAML
QLYINKLDSQGKYTLFRCLLNTSNHSGVEAFIIQNIKNQIDMSLKRTRNNKWFTGPQLISLLDLV
LFLPEGAETDLLQNSDRIMASLNLLRYLVIKDNENDNQTGLWTELGNIENNFLKPLHIGLNMSKA
HYEAEIKNSQEAQKSKDLCSITVSGEEIPNMPPEMQLKVLHSALFTFDLIESVLARVEELIEIKT
KSTSEENIGIK
>4F3WA
GPGSMPDIDWKQLRDKATQVAAGAYAPYSRFPVGAAALVDDGRVVTGCNVENVSYGLALCAECGV
VCALHATGGGRLVALACVDGRGAPLMPCGRCRQLLFEHGGPELLVDHLAGPRRLGDLLPEPFHAD
LTGEP

>4F2ME
YPYDVPDYAGAQPARSPGLVPRGSRTANLNNGFYPVSSSEVGLVNKSVVLLPSFYTHTIVNITID
LGMKRSGYGQPIASTLSNITLPMQDNNTDVYCIRSDQFSVYVHSTCKSSLWDNIFKRNCTDVLDA
TAVIKTGTCPFSFDKLNNYLTFNKFCLSLSPVGANCKFDVAARTRTNEQVVRSLYVIYEEGDNIV
LVPRGSDYKDDDDK
>4F2ZA
MSSQVEHPAGGYKKLFETVEELSSPLTAHVTGRIPLWLTGSLLRCGPGLFEVGSEPFYHLFDGQA
LLHKFDFKEGHVTYHRRFIRTDAYVRAMTEKRIVITEFGTCAFPDPCKNIFSRFFSYFRGVEVTD
NALVNIYPVGEDYYACTETNFITKVNPETLETIKQVDLCNYVSVNGATAHPHIENDGTVYNIGNC
FGKNFSIAYNIVKIPPLQADKEDPISKSEIVVQFPCSDRFKPSYVHSFGLTPNYIVFVETPVKIN
LFKFLSSWSLWGANYMDCFESNETMGVWLHIADKKRKKYINNKYRTSPFNLFHHINTYEDHEFLI
VDLCCWKGFEFVYNYLYLANLRENWEEVKKNARKAPQPEVRRYVLPLNIDKADTGKNLVTLPNTT
ATAILCSDETIWLEPEVLFSGPRQAFEFPQINYQKYGGKPYTYAYGLGLNHFVPDRLCKLNVKTK
ETWVWQEPDSYPSEPIFVSHPDALEEDDGVVLSVVVSPGAGQKPAYLLILNAKDLSEVARAEVEI
NIPVTFHGLFKKS
>4F0VA
MGSSHHHHHHSSGENLYFEGSHMASMTGGQQMGRMDSLDQCIVNACKNSWDKSYLAGTPNKDNCS
GFVQSVAAELGVPMPRGNANAMVDGLEQSWTKLASGAEAAQKAAQGFLVIAGLKGRTYGHVAVVI
SGPLYRQKYPMCWCGSIAGAVGQSQGLKSVGQVWNRTDRDRLNYYVYSLASCSLPRAS
>4F02A
GPLGSMNPSAPSYPMASLYVGDLHPDVTEAMLYEKFSPAGPILSIRVCRDMITRRSLGYAYVNFQ
QPADAERALDTMNFDVIKGKPVRIMWSQRDPSLRKSGVGNIFIKNLDKSIDNKALYDTFSAFGNI
LSCKVVCDENGSKGYGFVHFETQEAAERAIEKMNGMLLNDRKVFVGRFKSRKEREAELGARAKEF
YPYDVPDYAGSSGRIVTD
>4ASUH
AEAAAAQAPAAGPGQMSFTFASPTQVFFNSANVRQVDVPTQTGAFGILAAHVPTLQVLRPGLVVV
HAEDGTTSKYFVSSGSVTVNADSSVQLLAEEAVTLDMLDLGAAKANLEKAQSELLGAADEATRAE
IQIRIEANEALVKALE
>4EY0A
HSNEKWFHGKLGAGRDGRHIAERLLTEYCIETGAPDGSFLVRESETFVGDYTLSFWRNGKVQHCR
IHSRQDAGTPKFFLTDNLVFDSLYDLITHYQQVPLRCNEFEMRLSEPVPQTNAHESKEWYHASLT
RAQAEHMLMRVPRDGAFLVRKRNEPNSYAISFRAEGKIKHCRVQQEGQTVMLGNSEFDSLVDLIS
YYEKHPLYRKMKLRYPINEEALEKIGTAEPDFGALFEGRNPGFYVEANPMP
>4ARZB
MSLEATDSKAMVLLMGVRRCGKSSICKVVFHNMQPLDTLYLESTSNPSLEHFSTLIDLAVMELPG
QLNYFEPSYDSERLFKSVGALVYVIDSQDEYINAITNLAMIIEYAYKVNPSINIEVLIHKVDGLS
EDFKVDAQRDIMQRTGEELLELGLDGVQVSFYLTSIFDHSIYEAFSRIVQKLIPELSFLENMLDN
LIQHSKIEKAFLFDVNSKIYVSTDSNPVDIQMYEVCSEFIDVTIDLFDLYKAPVLRNSQKSSDKD
NVINPRNELQNVSQLANGVIIYLRQMIRGLALVAIIRPNGTDMESCLTVADYNIDIFKKGLEDIW
ANARASQAKNSIEDDV
>4EVWA
MIVIPMAGMSSRFFKAGYTQPKYMLEAHGQTLFEHSVNSFAAYFASTPFLFIVRNVYDTAVFVRE
KATQLGIKQFYIAELHTETRGQAETVTLGLEELAKQGVDYQGSITVFNIDTFRPNFVFPDISQHS
DGYLEVFQGGGDNWSFAKPEHAGSTKVIQTAEKNPISDLCSTGLYHFNRKEDYLEAYREYVARPS

QEWERGELYIAPLYNELIQKGLNIHYHLIARHEVIFCGVPDEYTDFLRQPQPLEHHHHHH
>4EUWA
MAHHHHHHVDDDDKMSENLYFQSSKNKPHVKRPMNAFMVWAQAARRKLADQYPHLHNAELSKTLG
KLWRLLNESEKRPFVEEAERLRVQHKKDHPDYKYQPRRRKS
>4ETYA
SMQEGSLPDITIFPNSSLMISQGTFVTVVCSYSDKHDLYNMVRLEKDGSTFMEKSTEPYKTEDEF
EIGPVNETITGHYSCIYSKGITWSERSKTLELKVIKENVIQTPAPGPTSEHLG
>4ETPB
GASEIAALEKEIAALEKEIAALEKEISKQEKFYNDTYNTVCKELLRSRRLENSIIEQKGTMRVYA
YVMEQNLPENLLFDYENGVITQGLSEHVYKFNRVIPHLKVSEDCFFTQEYSVYHDMALNQKKNFN
LISLSTTPHGSLRESLIKFLAEKDTIYQKQYVITLQFVFLSDDEFSQDMLLDYSHNDKDSIKLKF
EKHSISLDSKLVIIENGLEDLPLNFSADEHPNLPHSGMGIIKVQFFPRDSKSDGNNDPVPVDFYF
IELNNLKSIEQFDKSIFKKESAETPIALVLKKLISDTKSFFLLNLNDSKNVNKLLTISEEVQTQL
AKRKKKLT
>4ESVA
MSELFSERIPPQSIEAEQAVLGAVFLDPAALVPASEILIPEDFYRAAHQKIFHAMLRVADRGEPV
DLVTVTAELAASEQLEEIGGVSYLSELADAVPTAANVEYYARIVEEKSVLRRLIRTATSIAQDGY
TREDEIDVLLDEADRKIMEVSQRKHSGAFKNIKDILVQTYDNIEMLHNRDGEITGIPTGFTELDR
MTSGFQRSDLIIVAARPSVGKTAFALNIAQNVATKTNENVAIFSLEMSAQQLVMRMLCAEGNINA
QNLRTGKLTPEDWGKLTMAMGSLSNAGIYIDDTPSIRVSDIRAKCRRLKQESGLGMIVIDYLQLI
QGSGRSKENRQQEVSEISRSLKALARELEVPVIALSQLSRSVEQRQDKRPMMSDIRESGSIEQDA
DIVAFLYRDDYYNKDSENKNIIEIIIAKQRNGPVGTVQLAFIKEYNKFVNLERRFDEAQIPPGA
>4ESEA
SNAMSKVLVLKSSILATSSQSNQLADFFVEQWQAAHAGDQITVRDLAAQPIPVLDGELVGALRPS
GTALTPRQQEALALSDELIAELQANDVIVIAAPMYNFNIPTQLKNYFDMIARAGVTFRYTEKGPE
GLVTGKRAIILTSRGGIHKDTPTDLVVPYLRLFLGFIGITDVEFVFAEGIAYGPEVATKAQADAK
TLLAQVVAA
>2LS8A
MVCPIDWRAFQSNCYFPLTDNKTWAESERNCSGMGAHLMTISTEAEQNFIIQFLDRRLSYFLGLR
DENAKGQWRWVDQTPFNPRRVFWHKNEPDNSQGENCVVLVYNQDKWAWNDVPCNFEASRICKIPG
TTLNAENLYFQSHHHHHHWSHPQFEK
>4ERRA
MGQIFTVQELKERAKVFAKPIGASYQGILDQLDLVHQAKGRDQIAASFELNKKINDYIAEHPTSG
RNQALTQLKEQVTSALGLEHHHHHH
>4EPAA
GAMGQTSQQDESTLVVTASKQSSRSASANNVSSTVVSAPELSDAGVTASDKLPRVLPGLNIENSG
NMLFSTISLRGVSSAQDFYNPAVTLYVDGVPQLSTNTIQALTDVQSVELLRGPQGTLYGKSAQGG
IINIVTQQPDSTPRGYIEGGVSSRDSYRSKFNLSGPIQDGLLYGSVTLLRQVDDGDMINPATGSD
DLGGTRASIGNVKLRLAPDDQPWEMGFAASRECTRATQDAYVGWNDIKGRKLSISDGSPDPYMRR
CTDSQTLSGKYTTDDWVFNLISAWQQQHYSRTFPSGSLIVNMPQRWNQDVQELRAATLGDARTVD
MVFGLYRQNTREKLNSAYDMPTMPYLSSTGYTTAETLAAYSDLTWHLTDRFDIGGGVRFSHDKSS
TQYHGSMLGNPFGDQGKSNDDQVLGQLSAGYMLTDDWRVYTRVAQGYKPSGYNIVPTAGLDAKPF
VAEKSINYELGTRYETADVTLQAATFYTHTKDMQLYSGPVRMQTLSNAGKADATGVELEAKWRFA
PGWSWDINGNVIRSEFTNDSELYHGNRVPFVPRYGAGSSVNGVIDTRYGALMPRLAVNLVGPHYF

DGDNQLRQGTYATLDSSLGWQATERMNISVYVDNLFDRRYRTYGYMNGSSAVAQVNMGRTVGINT
RIDFF
>4EPCA
GSTTSTKPSQPSKPSGGTNNKLTVSANRGVAQIKPTNNGLYTTVYDSKGHKTDQVQKTLSVTKTA
TLGNNKFYLVEDYNSGKKYGWVKQGDVVYNTAKAPVKVNQTYNVKAGSTLYTVPWGTPKQVASKV
SGTGNQTFKATKQQQIDKATYLYGTVNGKSGWISKYYLTTASKPSNPTKPSTNNQLTVTNNSGVA
QINAKNSGLYTTVYDTKGKTTNQIQRTLSVTKAATLGDKKFYLVGDYNTGTNYGWVKQDEVIYNT
AKSPVKINQTYNVKPGVKLHTVPWGTYNQVAGTVSGKGDQTFKATKQQQIDKATYLYGTVNGKSG
WISKYYLTA
>4EOZA
GSNMVKVPECRLADELGGLWENSRFTDCCLCVAGQEFQAHKAILAARSPVFSAMFEHEMEESKKN
RVEINDVEPEVFKEMMCFIYTGKAPNLDKMADDLLAAADKYALERLKVMCEDALCSNLSVENAAE
ILILADLHSADQLKT
>4EODA
MAVTDLSLTNSSLMPTLNPMIQQLALAIAASWQSLPLKPYQLPEDLGYVEGRLEGEKLVIENRCY
QTPQFRKMELELAKVGKGLDILHCVMFPEPLYGLPLFGCDIVAGPGGVSAAIADLSPTQSDRQLP
AAYQKSLAELGQPEFEQQRELPPWGEIFSEYCLFIRPSNVTEEERFVQRVVDFLQIHCHQSIVAE
PLSEAQTLEHRQGQIHYCQQQQKNDKTRRVLEKAFGEAWAERYMSQVLFDVIQ
>4EN6B
QTILPYPNGLYVINKGDGYMRTNDKDLIGTLLIESSTSGSIIQPRLRNTTRPLFNTSNPTIFSQE
YTEARLNDAFNIQLFNTSTTLFKFVEEAPTNKNISMKVYNTYEKYELINYQNGNIDDKAEYYLPS
LGKCEVSDAPSPQAPVVETPVDQDGFIQTGPNENIIVGVINPSENIEEISTPIPDDYTYNIPTSI
QNNACYVLFKVNTTGVYKITTKNNLPPLIIYEAIGSSNRNMNSNNLSNDNIKAIKYITGLNRSDA
KSYLIVSLFKDKNYYIRIPQISSSTTSQLIFKRELGNISDLADSTVNILDNLNTSGTHYYTRQSP
DVGNYISYQLTIPGDFNNIASSIFSFRTRNNQGIGTLYRLTESINGYNLITINNYSDLLNNVEPI
SLLNGATYIFRVKVTELNNYNIIFDAYRNS
>4EMOA
GSMAPPAGGAAAAASDLGSAAVLMAVHAAVRPLGAGPDAEAQLRRLQLSADPERPGRFRLELLGA
GPGAVNLEWPLESVSYTIRGPTQHELQPPPGGPGTLSMHFLNPQEAQRWAVLVRGATVEGQNGS
>4AQ1A
MASFTDVAPQYKDAIDFLVSTGATKGKTETKFGVYDEITRLDAAVILARVLKLDVGNAKDAGFTD
VPKDRAKYVNALVEAGVLNGKAPGKFGAYDPLTRVEMAKIIANAHKLKADDVKLPFTDVNDTWAP
YVKALYKYEVTKGKTPTSFGAYQNITRGDFAQFVYRAVNINAVPEIVEVTAVNSTTVKVTFNTQI
ADVDFTNFAIDNGLTVTKATLSRDKKSVEVVVNKPFTRNQEYTITATGIKNLKGETAKELTGKFV
WSVQDAVTVALNNSSLKVGEESGLTVKDQDGKDVVGAKVELTSSNTNIVVVSSGEVSVSAAKVTA
VKPGTADVTAKVTLPDGVVLTNTFKVTVTEVPVQVQNQGFTLVDNLSNAPQNTVAFNKAEKVTSM
FAGETKTVAMYDTKNGDPETKPVDFKDATVRSLNPIIATAAINGSELLVTANAGQSGKASFEVTF
KDNTKRTFTVDVKKEPVLQDIKVDATSVKLSDEAVGGGEVEGVNQKTIKVSAVDQYGKEIKFGTK
GKVTVTTNTEGLVIKNVNSDNTIDFDSGNSATDQFVVVATKDKIVNGKVEVKYFKNASDTTPTST
KTITVNVVNVKADATPVGLDIVAPSEIDVNAPNTASTADVDFINFESVEIYTLDSNGNRLKKVTP
TATTLVGTNDYVEVNGNVLQFKGNDELTLLTSSSTVNVDVTADGITKRIPVKYINSASVPASATV
ATSPVTVKLNSSDNDLTFEELIFGVIDPTQLVKDEDINEFIAVSKAAKNDGYLYNKPLVTVKDAS
GEVIPTGANVYGLNHDATNGNIWFDEEQAGLAKKFSDVHFDVDFSLANVVKTGSGTVSSSPSLSD
AIQLTNSGDAVSFTLVIKSIYVKGADKDDNNLLAAPVSVNVTVTKGS

>4EMZB
MGGKWSKSSVIGWPAVRERMRRAEPAADGVGAVSRDLEKHGAITSSNTAANNAACAWLEAQEEE
VGFPVTPQVPLRPMTYKAAVDLSHFLKEKGGLEGLIHSQRRQDILDLWIYHTQGYFPDWQNYTPG
PGVRYPLTFGWCYKLVPVEPDKVEEANKGENTSLLHPVSLHGMDDPEREVLEWRFDSRLAFHHVA
RELHPEYFKNC
>4EMKB
MDSSPNEFLNKVIGKKVLIRLSSGVDYKGILSCLDGYMNLALERTEEYVNGKKTNVYGDAFIRGN
NVLYVSALDD
>4EMCC
QKKRFLPQSVLIKREDEIAFDDFHLDARKVLNDLSATSENPFSSSPNTKKIKSKGKTLEVVPKKK
NKKII
>4ELLA
GEFNTIQQLMMILNSASDQPSENLISYFNNCTVNPKESILKRVKDIGYIFKEKFAKAVGQGCVEI
GSQRYKLGVRLYYRVMESMLKSEEERLSIQNFSKLLNDNIFHMSLLACALEVVMATYSRSTSQNL
DSGTDLSFPWILNVLNLKAFDFYKVIESFIKAEGNLTREMIKHLERCEHRIMESFAWLSDSPLFD
LIKQSKDREGPTDHLESACPLNLPLQNNHTAADMYLEPVRAPKKKGSTTRVNSTANAETQATSAF
QTQKPLKSTSLSLFYKKVYRLAYLRLNTLCERLLSEHPELEHIIWTLFQHTLQNEYELMRDRHLD
QIMMCSMYGICKVKNIDLKFKIIVTAYKDLPHAVQETFKRVLIKEEEYDSIIVFYNSVFMQRLKT
NILQYASTRPPTLAPIPHIPR
>3VRCA
ADLSPEEQIETRQAGYAFMAWNMGKIKANLEGEYNADQVRAAANVVAAIANSGMGALYGPGTDKN
VGAVKTRAKPELFQNLEDVGKLARDLGTAANALAAAAATGEANAVKSAFADVGAACKACHQKYRA
D
>3VR8A
MLRAVRALICRIGARRTLSVSSSRLDVSTSNIAQYKVIDHAYDVVIIGAGGAGLRAAMGLGEAGF
KTAVVTKMFPTRSHTTAAQGGINAALGSMNPDDWKWHFYDTAKGSDWLGDQNAMHYLTRNAVEAV
TELENFGMPFSRTPEGKIYQRSFGGQSNNYGKGGVAKRTCCVADRTGHSMLHTLYGNSLRCHCTF
FIEYFALDLLMDKGRCVGVIALCLEDGTIHRFRSKRTIVATGGYGRAYFSCTTAHMNTGDGTALA
TRAGIALEDLEFIQFHPTGIYGVGCLITEGSRGEGGFLVNSEGERFMERYAPKAKDLASRDVVSR
AETIEIMEGRGVGPEKDHIYLQLHHLPAEQLHQRLPGISETAKIFAGVDVTKEPIPVIPTVHYNM
GGIPTNYKAQVIKYTKEGGDKIVPGLYACGECACHSVHGANRLGANSLLDAVVFGRACSINIKEE
LKPDEKIPELPEGAGEESIANLDAVRYANGDVPTAELRLTMQKTMQKHAGVFRRGDILAEGVKKM
MDLSKELKRLKTTDRSLIWNSDLTESLELQNLMLNATQTIVAAENRKESRGAHARDDFPKREDEY
DYSKPIEGQTKRPFEKHWRKHTLTKQDPRTGHITLDYRPVIDKTLDPAEVDWIPPIIRSY
>4EJOA
SNAMAYDDIVSSMVLELRRGTLVMLVLSQLREPAYGYALVKSLADHGIPIEANTLYPLMRRLESQ
GLLASEWDNGGSKPRKYYRTTDEGLRVLREVEAQWHVLCDGVGKLLETNGEDREHAER
>4EJ7A
MGSSHHHHHHSSGRENLYFQGMSHIQRETSCSRPRLNSNLDADLYGYRWARDNVGQSGATIYRLY
GKPNAPELFLKHGKGSVANDVTDEMVRLNWLTAFMPLPTIKHFIRTPDDAWLLTTAIPGKTAFQV
LEEYPDSGENIVDALAVFLRRLHSIPVCNCPFNSDRVFRLAQAQSRMNNGLVDASDFDDERNGWP
VEQVWKEMHKLLPFSPDSVVTHGDFSLDNLIFDEGKLIGCIDVGRVGIADRYQDLAILWNCLGEF
SPSLQKRLFQKYGIDNPDMNKLQFHLMLDEFF
>4EIYA

MKTIIALSYIFCLVFADYKDDDDGAPPIMGSSVYITVELAIAVLAILGNVLVCWAVWLNSNLQNV
TNYFVVSLAAADIAVGVLAIPFAITISTGFCAACHGCLFIACFVLVLTQSSIFSLLAIAIDRYIA
IRIPLRYNGLVTGTRAKGIIAICWVLSFAIGLTPMLGWNNCGQPKEGKNHSQGCGEGQVACLFED
VVPMNYMVYFNFFACVLVPLLLMLGVYLRIFLAARRQLADLEDNWETLNDNLKVIEKADNAAQVK
DALTKMRAAALDAQKATPPKLEDKSPDSPEMKDFRHGFDILVGQIDDALKLANEGKVKEAQAAAE
QLKTTRNAYIQKYLERARSTLQKEVHAAKSLAIIVGLFALCWLPLHIINCFTFFCPDCSHAPLWL
MYLAIVLSHTNSVVNPFIYAYRIREFRQTFRKIIRSHVLRQQEPFKAHHHHHHHHHH
>2LRKA
AEELEEVVMGLIINSGQARSLAYAALKQAKQGDFAAAKAMMDQSRMALNEAHLVQTKLIEGDAGE
GKMKVSLVLVEAQLHLMTSMLARELITELIELHEKLKA
>4EIVA
MHHHHHHENLYFQGGTIYKQFTSRTLLNFFEVAALTDGETNESVAAVCKIAAKDPAIVGVSVRPA
FVRFIRQELVKSAPEVAGIKVCAAVNFPEGTGTPDTVSLEAVGALKDGADEIECLIDWRRMNENV
ADGESRIRLLVSEVKKVVGPKTLKVVLSGGELQGGDIISRAAVAALEGGADFLQTSSGLGATHAT
MFTVHLISIALREYMVRENERIRVEGINREGAAVRCIGIKIEVGDVHMAETADFLMQMIFENGPR
SIVRDKFRVGGGFNLLKELRDCYESWDSVGVSPDTSP
>4APMA
GSAMGSTPKDIWGRYMAKFDLAKSHGSGIYVDLGGTERVGATQHRMPTGKCPVMGKVINLGNNAD
FLNRISAENPQDRGLAFPDTAVAVTRNSNARNRAAAEKTEIILSPVSAADLVRWGYDGNDVANCA
EYAGNIIPASDTATKYRYPFVYDAKEEMCHILFTPMQYNRGSRYCDNDGSQDEGTSSLLCMEPMK
SGIDAHLYYGSSRVDKKWEENCPMYPVKDAIFGRGANGSCVAIESAFEEFTRDAEECSALMFENA
AADLEIDEEADNFDELKTLSDGLRNIKASKIAQALFSPIAKAGTSAKNSKGVGMNWANYDSNTGL
CRVIEETPNCLIIDAGSFAMTAVGSPLEQDAVPFPCDIVTNGYIEPRPRSRHRNTTPIFEVTTAL
SREALKCSKYVHEKYSESCGTYYYCSEEKPSSWAFWRNLDAAALVPR
>2LRJA
GSSISHSGNLYTAGQCTWYVYDKVGGEIGSTWGNANNWAAAAQGAGFTVNHTPSKGAILQSSEGP
FGHVAYVESVNSDGSVTISEMNYSGGPFSVSSRTISASEAGNYNYIHI
>4EFOA
GPLGSTSDILHRMVIHVFSLQQMTAHKIYIHSYNTATIFHELVYKQTKIISSNQELIYEGRRLVL
EPGRLAQHFPKTTEENPIFVVSLERPHRD
>4EFZA
GPGSMTVEGFFDPATCTISYLLFDSGSGECALIDSVLDYDPKSGRTRTASADQLIARVAALGARV
RWLLETHVHADHLSAAPYLKTRVGGEIAIGRHVTRVQDVFGKLFNAGPAFAHDGSQFDRLLDDGD
TLALGALSIRAMHTPGHTPACMTYVVTEAHAAHDARDAAAFVGDTLFMPDYGTARCDFPGGDARS
LYRSIRKVLSLPPATRLYMCHDYQPNGRAIQYASTVADELRENVHIREGVTEDDFVAMRTARDAT
LDMPVLMLPSVQVNMRAGRLPEPEDNGVRYLKIPLDAI
>4AOWA
MHHHHHHSSGVDLGTENLYFQSMMTEQMTLRGTLKGHNGWVTQIATTPQFPDMILSASRDKTIIM
WKLTRDETNYGIPQRALRGHSHFVSDVVISSDGQFALSGSWDGTLRLWDLTTGTTTRRFVGHTKD
VLSVAFSSDNRQIVSGSRDKTIKLWNTLGVCKYTVQDESHSEWVSCVRFSPNSSNPIIVSCGWDK
LVKVWNLANCKLKTNHIGHTGYLNTVTVSPDGSLCASGGKDGQAMLWDLNEGKHLYTLDGGDIIN
ALCFSPNRYWLCAATGPSIKIWDLEGKIIVDELKQEVISTSSKAEPPQCTSLAWSADGQTLFAGY
TDNLVRVWQVTIGTR
>4EFIA

MSSPDFSAGRELRTQGARIAGVVSCVPSKQVDNDYFVERFDASAVRDVVKMIGVNRRRWADAQTS
AGDLCRKAGEKLLAGLGWQADSIDALIFVSQTPNYRLPATAFVLQAELDLPASCLALDINLGCSG
YPQALWLGMNLIQTGAAKRVLLAVGDTISKMIDPTDRSTSLLFGDAGTMTALETSNGDAAAHFII
GADGKGARNLIVPSGGFKPYDAAADERMAGKSPECLFMDGGEIFNFTLNAVPKLVSRTLDIAGRD
KDSYDAFLFHQANLFMLKHLAKKAGLPAERVPVNIGEYGNTSCASIPLLITTELKDRLKEETLQL
GMFGFGVGYSWASAALAVGPLNIVDTIET
>4EEEA
MECVKTRSVNIHVPVKETSKVVLECRGDSYFRHFSYVYWIIGKNKTVDQLPPNSGYRERIYLFKK
PHRCENRPRADLILTNITDEMRNEKLTCVLIDPKDPLKESVILSKIWNCVYKI
>2LRDA
AMGKCSVLKKVACAAAIAGAVAACGGIDLPCVLAALKAAEGCASCFCEDHCHGVCKDLHLC
>4EEIA
MIKRYDVAEISKIWADENKYAKMLEVELAILEALEDRMVPKGTAAEIRARAQIRPERVDEIEKVT
KHDIIAFCTSIAEQFTAETGKFFHFGVTSSDIIDSALSLQIRDSMSYVIKDLEALCDSLLTKAEE
TKEIITMGRSHGMFAEPMSFGQKFLGAYVEFKRRLKDLKDFQKDGLTVQFSGAVGNYCILTTEDE
KKAADILGLPVEEVSTQVIPRDRIAKLISIHGLIASAIERLAVEIRHLHRSDVFEVYEGFSKGQK
GSSTMPHKKNPISTENLTGMARMLRSHVSIALENCVLWHERDISHSSAERFYLPDNFGIMVYALR
RMKNTIDNLVVQRDIIEDRVRSTSAYLSSFYLHFLVANTPFMREDCYKIVQQVAFDLKQGESFSK
KLQKVMHDEHNIILDIPEMDFEGIKKTYLKEIDHVFDRSVKARGENLY
>4EDFA
MFEIKKICCIGAGYVGGPTCSVIAHMCPEIRVTVVDVNESRINAWNSPTLPIYEPGLKEVVESCR
GKNLFFSTNIDDAIKEADLVFISVNTPTETYGMGKGRAADLKYIEACARRIVQNSNGYKIVTEKS
TVPVRAAESIRRIFDANTKPNLNLQVLSNPEFLAEGTAIKDLKNPDRVLIGGDETPEGQRAVQAL
CAVYEHWVPREKILTTNTWSSELSKLAANAFLAQRISSINSISALCEATGADVEEVATAIGMDQR
IGNKFLKASVGFGGSCFQKDVLNLVYLCEALNLPEVARYWQQVIDMNDYQRRRFASRIIDSLFNT
VTDKKIAILGFAFKKDTGDTRESSSIYISKYLMDEGAHLHIYDPKVPREQIVVDLSHPGVSEDDQ
VSRLVTISKDPYEACDGAHAVVICTEWDMFKELDYERIHKKMLKPAFIFDGRRVLDGLHNELQTI
GFQIETIGKKVSSKRIPYAPSGEIPKFSLQDPPNKKPKV
>3VQKA
MYYLGKELQKRSEELSRGFYELVYPPVDMYEEGGYLVVVADLAGFNKEKIKARVSGQNELIIEAE
REITEPGVKYLTQRPKYVRKVIRLPYNVAKDAEISGKYENGVLTIRIPIAGTSVIKIE
>4EBBA
PDPGFQERFFQQRLDHFNFERFGNKTFPQRFLVSDRFWVRGEGPIFFYTGNEGDVWAFANNSAFV
AELAAERGALLVFAEHRYYGKSLPFGAQSTQRGHTELLTVEQALADFAELLRALRRDLGAQDAPA
IAFGGSYGGMLSAYLRMKYPHLVAGALAASAPVLAVAGLGDSNQFFRDVTADFEGQSPKCTQGVR
EAFRQIKDLFLQGAYDTVRWEFGTCQPLSDEKDLTQLFMFARNAFTVLAMMDYPYPTDFLGPLPA
NPVKVGCDRLLSEAQRITGLRALAGLVYNASGSEHCYDIYRLYHSCADPTGCGTGPDARAWDYQA
CTEINLTFASNNVTDMFPDLPFTDELRQRYCLDTWGVWPRPDWLLTSFWGGDLRAASNIIFSNGN
LDPWAGGGIRRNLSASVIAVTIQGGAHHLDLRASHPEDPASVVEARKLEATIIGEWVKAARREQQ
PALRGGPRLSLENLYFQ
>4EAZA
DPNSMRSIASSKLWMLEFSAFLERQQDPDTYNKHLFVHISQSSPSYSDPYLETVDIRQIYDKFPE
KKGGLKELFERGPSNAFFLVKFWADLNTNIDDEGSAFYGVSSQYESPENMIITCSTKVCSFGKQV
VEKVETEYARYENGHYLYRIHRSPLCEYMINFIHKLKHLPEKYMMNSVLENFTILQVVTNRDTQE

TLLCIAYVFEVSASEHGAQHHIYRLVKE
>4EAGC
MESVAAESAPAPENEHSQETPESNSSVYTTFMKSHRCYDLIPTSSKLVVFDTSLQVKKAFFALVT
NGVRAAPLWDSKKQSFVGMLTITDFINILHRYYKSALVQIYELEEHKIETWREVYLQDSFKPLVC
ISPNASLFDAVSSLIRNKIHRLPVIDPESGNTLYILTHKRILKFLKLFITEFPKPEFMSKSLEEL
QIGTYANIAMVRTTTPVYVALGIFVQHRVSALPVVDEKGRVVDIYSKFDVINLAAEKTYNNLDVS
VTKALQHRSHYFEGVLKCYLHETLEAIINRLVEAEVHRLVVVDEHDVVKGIVSLSDILQALVLTG
GEKKP
>4E8JA
MKNNNVTEKELFYILDLFEHMKVTYWLDGGWGVDVLTGKQQREHRDIDIDFDAQHTQKVIQKLED
IGYKIEVHWMPSRMELKHEEYGYLDIHPINLNDDGSITQANPEGGNYVFQNDWFSETNYKDRKIP
CISKEAQLLFHSGYDLTETDHFDIKNLKSIT
>4E88A
MGGRALRVLVDMDGVLADVEGGLLRKFRARFPDQPFIALEDRRGYKACEQYGRLRPGLSEKARSI
AESKNFFFELEPLPGAVEAVKEMASLQNTDVFICTSPHKMFKYCPYEKYAWVEKYFGPDFLEQIV
LTRDKTVVSADLLIDDRPDITGAEPTPSWEHVLFTACHNQHLQLQPPRRRLHSWADDWKAILDSK
RPCGSLEHHHHHH
>4AN6A
DYTVHDTDGKPVLNNAGQYYILPAKQGKGGGLGLSNDDDGNCPLTVSQTPIDLPIGLPVRFSSRA
RISHITTALSLNIEFTIAPACAPKPARWRIFNEQSSEKGYTPVKISDDFSSAAPFQIKKFEEDYK
LVYCSKSESGERKCVDLGIKIDDEKNRRLVLKEGDPFKVKFKKVDEESSEEWSIV
>4E4TA
MAHHHHHHMGTLEAQTQGPGSMTATPDSVSPILPGAWLGMVGGGQLGRMFCFAAQSMGYRVAVLD
PDPASPAGAVADRHLRAAYDDEAALAELAGLCEAVSTEFENVPAASLDFLARTTFVAPAGRCVAV
AQDRIAEKRFIEASGVPVAPHVVIESAAALAALDDAALDAVLPGILKTARLGYDGKGQVRVSTAR
EARDAHAALGGVPCVLEKRLPLKYEVSALIARGADGRSAAFPLAQNVHHNGILALTIVPAPAADT
ARVEEAQQAAVRIADTLGYVGVLCVEFFVLEDGSFVANEMAPRPHNSGHYTVDACATSQFEQQVR
AMTRMPLGNPRQHSPAAMLNILGDVWFPNGAAAGAVTPPWDTVAAMPAAHLHLYGKEEARVGRKM
GHVNFTAEMRDDAVAAATACAQLLRVPLD
>4E51A
MAHHHHHHMGTLEAQTQGPGSMTEQKRKLEKLTGVKGMNDILPQDAGLWEFFEATVKSLLRAYGY
QNIRTPIVEHTPLFTRGIGEVTDIVEKEMYSFVDALNGENLTLRPENTAAVVRAAIEHNMLYDGP
KRLWYIGPMFRHERPQRGRYRQFHQVGVEALGFAGPDADAEIVMMCQRLWEDLGLTGIKLEINSL
GLAEERAAHRVELIKYLEQHADKLDDDAQRRLYTNPLRVLDTKNPALQEIVRNAPKLIDFLGDVS
RAHFEGLQRLLKANNVPFTINPRLVRGLDYYNLTVFEWVTDKLGAQGTVAAGGRYDPLIEQLGGK
PTAACGWAMGIERILELLKEEHLVPEQEGVDVYVVHQGDAAREQAFIVAERLRDTGLDVILHCSA
DGAGASFKSQMKRADASGAAFAVIFGEDEVTNGTASVKPLRGTGDDGEKSVQQSVPVESLTEFLI
NAMVATAEDGDD
>2LQOA
MVTAALTIYTTSWCGYCLRLKTALTANRIAYDEVDIEHNRAAAEFVGSVNGGNRTVPTVKFADGS
TLTNPSADEVKAKLVKIAGLEHHHHHH
>4E1YA
GSHPFDQAVVKDPTASYVDVKARRTFLQSGQLDDRLKAALPKEYDCTTEATPNPQQGEMVIPRRY
LSGNHGPVNPDYEPVVTLYRDFEKISATLGNLYVATGKPVYATCLLNMLDKWAKADALLNYDPKS

QSWYQVEWSAATAAFALSTMMAEPNVDTAQRERVVKWLNRVARHQTSFPGGDTSCCNNASYWRGQ
EATIIGVISKDDELFRWGLGRYVQAMGLINEDGSFVHEMTRHEQSLHYQNYAMLPLTMIAETASR
QGIDLYAYKENGRDIHSARKFVFAAVKNPDLIKKYASEPQDTRAFKPGRGDLNWIEYQRARFGFA
DELGFMTVPIFDPRTGGSGTLLAYKPQG
>4E1JA
MHHHHHHSSGVDLGTENLYFQSMMGGYILAIDQGTTSTRAIVFDGNQKIAGVGQKEFKQHFPKSG
WVEHDPEEIWQTVVSTVKEAIEKSGITANDIAAIGITNQRETVVVWDRETGKPIHNAIVWQDRRT
AAFCDKLKKKGLEKTFVKKTGLLLDPYFSGTKLNWLLSNVKGAQVRAAKGELCFGTIDTFLIWRL
TGGECFCTDATNASRTLLYNIAENAWDDELTEVLRVPKEMLPEVKDCAADFGVTDPSLFGAAIPI
LGVAGDQQAATIGQACFKPGMLKSTYGTGCFALLNTGKDMVRSKNRLLTTIAYRLDGETTYALEG
SIFVAGAAVQWLRDGLKVIKAAPDTGSLAESADPSQEVYLVPAFTGLGAPHWDPDARGAIFGMTR
NTGPAEFARAALEAVCYQTRDLLEAMHKDWRRNGNDTVLRVDGGMVASDWTMQRLSDLLDAPVDR
PVILETTALGVAWLAGSRAGVWPNQEAFAKSWARDRRFEPHMDEATRKVKLKGWRSAVKRTLIAA
>4E0VA
MNVFFMFSKPGKLADDRNPLEECFRETDYEEFLEIAKNGLSTTSNPKRVVIVGAGMSGLSAAYVL
ANAGHQVTVLEASERAGGQVKTYRNEKEGWYANLGPMRLPEKHRIVREYIRKFGLQLNEFSQENE
NAWYFIKNIRKRVGEVNKDPGVLDYPVKPSEVGKSAGQLYEESLQKAVEELRRTNCSYMLNKYDT
YSTKEYLLKEGNLSPGAVDMIGDLLNEDSGYYVSFIESLKHDDIFAYEKRFDEIVGGMDKLPTSM
YQAIQEKVHLNARVIKIQQDVKEVTVTYQTSEKETLSVTADYVIVCTTSRAARRIKFEPPLPPKK
AHALRSVHYRSGTKIFLTCTKKFWEDDGIHGGKSTTDLPSRFIYYPNHNFPNGVGVIIAYGIGDD
ANYFEALDFEDCGDIVINDLSLIHQLPKEEIQAICRPSMIQRWSLDKYAMGGITTFTPYQFQHFS
EALTAPVDRIYFAGEYTAQAHGWIASTIKSGPEGLDVNRASE
>4E0IA
MKAIDKMTDNPPQEGLSGRKIIYDEDGKPSRSCNTLLDFQYVTGKISNGLKNLSSNGKLAGTGAL
TGEASELMPGSRTYRKVDPPDVEQLGRSSWTLLHSVAASYPAQPTDQQKGEMKQFLNIFSHIYPC
NWSAKDFEKYIRENAPQVESREELGRWMCEAHNKVNKKLRKPKFDCNFWEKRWKDGWDE
>4DYLA
SMGFSSELCSPQGHGVLQQMQEAELRLLEGMRKWMAQRVKSDREYAGLLHHMSLQDSGGQSRAIS
PDSPISQSWAEITSQTEGLSRLLRQHAEDLNSGPLSKLSLLIRERQQLRKTYSEQWQQLQQELTK
THSQDIEKLKSQYRALARDSAQAKRKYQEASKDKDRDKAKDKYVRSLWKLFAHHNRYVLGVRAAQ
LHHQHHHQLLLPGLLRSLQDLHEEMACILKEILQEYLEISSLVQDEVVAIHREMAAAAARIQPEA
EYQGFLRQYGSAPDVPPCVTFDESLLEEGEPLEPGELQLNELTVESVQHTLTSVTDELAVATEMV
FRRQEMVTQLQQELRNEEENTHPRERVQLLGKRQVLQEALQGLQVALCSQAKLQAQQELLQTKLE
HLGPGEPPPVLLLQDD
>4DXRA
GPGGSGGVTEEQVHHIVKQALQRYSEDRIGLADYALESGGASVISTRCSETYETKTALLSLFGIP
LWYHSQSPRVILQPDVHPGNCWAFQGPQGFAVVRLSARIRPTAVTLEHVPKALSPNSTISSAPKD
FAIFGFDEDLQQEGTLLGKFTYDQDGEPIQTFHFQAPTMATYQVVELRILTNWGHPEYTCIYRFR
VHGEPAH
>3VP7A
INIFNATFKISHSGPFATINGLRLGSIPESVVPWKEINAALGQLILLLATINKNLKINLVDYELQ
PMGSFSKIKKRMVNSVEYNNSTTNAPGDWLILPVYYDENFNLGRIFRKETKFDKSLETTLEIISE
ITRQLSTIASSYSSQTLTTSQDESSMNNANDVENSTSILELPYIMNKDKINGLSVKLHGSSPNLE
WTTAMKFLLTNVKWLLAFSSNLLSK

>4DXDA
MHHHHHHLEFEQGFNHLATLKVIGVGGGGNNAVNRMIDHGMNNVEFIAINTDGQALNLSKAESKI
QIGEKLTRGLGAGANPEIGKKAAEESREQIEDAIQGADMVFVTSGMGGGTGTGAAPVVAKIAKEM
GALTVGVVTRPFSFEGRKRQTQAAAGVEAMKAAVDTLIVIPNDRLLDIVDKSTPMMEAFKEADNV
LRQGVQGISDLIAVSGEVNLDFADVKTIMSNQGSALMGIGVSSGENRAVEAAKKAISSPLLETSI
VGAQGVLMNITGGESLSLFEAQEAADIVQDAADEDVNMIFGTVINPELQDEIVVTVIATGFDDKP
TSHGRKSGSTGFGTSVNTSSNATSKDESFTSNSSNAQATDSVSERTHTTKEDDIPSFIRNREERR
SRRTRR
>4DX1A
MFNRPILFDIVSRGSTADLDGLLPFLLTHKKRLTDEEFREPSTGKTCLPKALLNLSNGRNDTIPV
LLDIAERTGNMREFINSPFRDIYYRGQTALHIAIERRCKHYVELLVAQGADVHAQARGRFFQPKD
EGGYFYFGELPLSLAACTNQPHIVNYLTENPHKKADMRRQDSRGNTVLHALVAIADNTRENTKFV
TKMYDLLLLKCARLFPDSNLEAVLNNDGLSPLMMAAKTGKIGIFQHIIRREVTDEAAAHHHHHH
>4AKKA
MRGSHHHHHHTDPHASSVPGRGSIEGRMNNMAGNTPEVVDWFARARRLQKQQLHQLAQQGTLAGQ
ISALVHMLQCERGASNIWLCSGGRLYAAECRAGAALVDEQLTRFYAALEPARDAASSALCWRIAC
AVWYLPQLAALRKRVRDREIAAEEATGQFSRIIRHLLNIVPQLNDSIDDPQIAGRMVALYSFMQG
KELAGQERALGALGFARGQFSDELRQQLVDRIDGQQPCFDSFQALAQPPQTALFAEQCQASLEIE
QLRRVACTRQPPADEGETALRWFCAQTQRLEQLRGVEELLIVDLLNAADALLEGEEPEAQLPPAD
WQEDSIALRLDKQLLPLVRQQAHELQQLSGQLASLKDALEERKLIEKAKSVLMTYQGMQEEQAWQ
ALRKMAMDKNQRMVEIARALLTVKALWRVTPKE
>4DW0A
GSSKKVGTLNRFTQALVIAYVIGYVFVYNKGYQDTDTVLSSVTTKVKGIALTKTSELGERIWDVA
DYIIPPQEDGSFFVLTNMIITTNQTQSKCAENPTPASTCTSHRDCKRGFNDARGDGVRTGRCVSY
SASVKTCEVLSWCPLEKIVDPPNPPLLADAERFTVLIKNNIRYPKFNFNKRNILPNINSSYLTHC
VFSRKTDPDCPIFRLGDIVGEAEEDFQIMAVRGGVMGVQIRWDCDLDMPQSWCVPRYTFRRLDNK
DPDNNVAPGYNFRFAKYYKNSDGTETRTLIKGYGIRFDVMVFGQAGKFNIIPTLLNIGAGLALLG
LVNVICDWIVLTFMK
>4DT4A
MGSSHHHHHHSSGLVPRGSHMSESVQSNSAVLVHFTLKLDDGTTAESTRNNGKPALFRLGDASLS
EGLEQHLLGLKVGDKTTFSLEPDAAFGVPSPDLIQYFSRREFMDAGEPEIGAIMLFTAMDGSEMP
GVIREINGDSITVDFNHPLAGQTVHFDIEVLEIDPALEA
>2LPIA
GSGNSHTTPWTNPGLAENFMNSFMQGLSSMPGFTASQLDDMSTIAQSMVQSIQSLAAQGRTSPNK
LQALNMRFASSMAEIAASEEGGGSLSTKTSSIASAMSNAFLQTTGVVNQPFINEITQLVSMFAQA
GMNDVSA
>4DPPA
MHHHHHHGLPIPNPLLGLDSTENLYFQGIDPFTAAVVPNFHLPMRSLEVKNRTNTDDIKALRVIT
AIKTPYLPDGRFDLEAYDDLVNIQIQNGAEGVIVGGTTGEGQLMSWDEHIMLIGHTVNCFGGSIK
VIGNTGSNSTREAIHATEQGFAVGMHAALHINPYYGKTSIEGLIAHFQSVLHMGPTIIYNVPGRT
GQDIPPRAIFKLSQNPNLAGVKECVGNKRVEEYTENGVVVWSGNDDECHDSRWDYGATGVISVTS
NLVPGLMRKLMFEGRNSSLNSKLLPLMAWLFHEPNPIGINTALAQLGVSRPVFRLPYVPLPLSKR
LEFVKLVKEIGREHFVGEKDVQALDDDDFILIGRY
>4DOTA

MRAPIPEPKPGDLIEIFRPFYRHWAIYVGDGYVVHLAPPSEVAGAGAASVMSALTDKAIVKKELL
YDVAGSDKYQVNNKHDDKYSPLPCSKIIQRAEELVGQEVLYKLTSENCEHFVNELRYGVARSDQV
RDLEHHHHHH
>4DOJA
LENPTNLEGKLADAEEEIILEGEDTQASLNWSVIVPALVIVLATVVWGIGFKDSFTNFASSALSA
VVDNLGWAFILFGTVFVFFIVVIAASKFGTIRLGRIDEAPEFRTVSWISMMFAAGMGIDLMFYGT
TEPLTFYRNGVPGHDEHNVGVAMSTTMFHWTLHPWAIYAIVGLAIAYSTFRVGRKQLLSSAFVPL
IGEKGAEGWLGKLIDILAIIATVFGTACSLGLGALQIGAGLSAANIIEDPSDWTIVGIVSVLTLA
FIFSAISGVGKGIQYLSNANMVLAALLAIFVFVVGPTVSILNLLPGSIGNYLSNFFQMAGRTAMS
ADGTAGEWLGSWTIFYWAWWISWSPFVGMFLARISRGRSIREFILGVLLVPAGVSTVWFSIFGGT
AIVFEQNGESIWGDGAAEEQLFGLLHALPGGQIMGIIAMILLGTFFITSADSASTVMGTMSQHGQ
LEANKWVTAAWGVATAAIGLTLLLSGGDNALSNLQNVTIVAATPFLFVVIGLMFALVKDLSNDVI
YLEYREQQRFNARLARERRVHNEHRKRELAAKRRRERKASGAGKRR
>4DMUB
APDCSQPLDVILLLDGSSSFPASYFDEMKSFAKAFISKANIGPRLTQVSVLQYGSITTIDVPWNV
VPEKAHLLSLVDVMQREGGPSQIGDALGFAVRYLTSEMHGARPGASKAVVILVTDVSVDSVDAAA
DAARSNRVTVFPIGIGDRYDAAQLRILAGPAGDSNVVKLQRIEDLPTMVTLGNSFLHKLCSG
>4DM3A
MSGADRSPNAGAAPDSAPGQAAVASAYQRFEPRAYLRNNYAPPRGDLCNPNGVGPWKLRCLAQTF
ATGEVSGRTLIDIGSGPTVYQLLSACSHFEDITMTDFLEVNRQELGRWLQEEPGAFNWSMYSQHA
CLIEGKGECWQDKERQLRARVKRVLPIDVHQPQPLGAGSPAPLPADALVSAFCLEAVSPDLASFQ
RALDHITTLLRPGGHLLLIGALEESWYLAGEARLTVVPVSEEEVREALVRSGYKVRDLRTYIMPA
HLQTGVDDVKGVFFAWAQKVGLEHHHHHH
>4DLFA
MGALRIDSHQHFWRYRAADYPWIGAGMGVLARDYLPDALHPLMHAQALGASIAVQARAGRDETAF
LLELACDEARIAAVVGWEDLRAPQLAERVAEWRGTKLRGFRHQLQDEADVRAFVDDADFARGVAW
LQANDYVYDVLVFERQLPDVQAFCARHDAHWLVLDHAGKPALAEFDRDDTALARWRAALRELAAL
PHVVCKLSGLVTEADWRRGLRASDLRHIEQCLDAALDAFGPQRLMFGSDWPVCLLAASYDEVASL
VERWAESRLSAAERSALWGGTAARCYALPEPADARLAENLYFQ
>4DJSA
LATRAIPELTKLLNDEDQVVVNKAAVMVHQLSKKEASRHAIMRSPQMVSAIVRTMQNTNDVETAR
CTAGTLHNLSHHREGLLAIFKSGGIPALVKMLGSPVDSVLFYAITTLHNLLLHQEGAKMAVRLAG
GLQKMVALLNKTNVKFLAITTDCLQILAYGNQESKLIIILASGGPQALVNIMRTYTYEKLLWTTSR
VLKVLSVCSSNKPAIVEAGGMQALGLHLTDPSQRLVQNCLWTLRNLSDAATKQEGMEGLLGTLVQ
LLGSDDINVVTCAAGILSNLTCNNYKNKMMVCQVGGIEALVRTVLRAGDREDITEPAICALRHLT
SRHQEAEMAQNAVRLHYGLPVVVKLLHPPSHWPLIKATVGLIRNLALCPANHAPLREQGAIPRLV
QLLVRAHQDTQRRTSMGGTQQQFVEGVRMEEIVEGCTGALHILARDVHNRIVIRGLNTIPLFVQL
LYSPIENIQRVAAGVLCELAQDKEAAEAIEAEGATAPLTELLHSRNEGVATYAAAVLFRMSED
>4DJTA
GPGSMERRELTYKICLIGDGGVGKTTYINRVLDGRFEKNYNATVGAVNHPVTFLDDQGNVIKFNV
WDTAGQEKKAVLKDVYYIGASGAILFFDVTSRITCQNLARWVKEFQAVVGNEAPIVVCANKIDIK
NRQKISKKLVMEVLKGKNYEYFEISAKTAHNFGLPFLHLARIFTGRPDLIFVSNVNLEPTEVNYD
YHSPEESKYIDYMEQASKMAPEE
>4DJBA

MGSSHHHHHHSQDPMIRCLRLKVEGALEQIFTMAGLNIRDLLRDILRRWRDENYLGMVEGAGMFI
EEIHPEGFSLYVHLDVRAVSLLEAIVQHLTEAIISSLAVEFDHATGGERVHLIDLHFEVLDNLLE
>4DIXA
GPSSSKSEENISLVYEIDGTEALGSCLRVRPCSNDAPDLSKCTIQWYRSSSDGSKKELISGATKS
VYAPEPFDVGRVLHADIIYDGHSLSLSTVGKIDPAAGLGSYVEALVRKHDVDFNVVVTQMSGEDH
TSESIHLFHVGKMRIKLCKGKTVIAKEYYSSAMQLCGVRGGGNAAAQALYWQAKKGVSFVIAFES
ERERNAAIMLARRFACDCNVTLAGPEDRTETGQSP
>4DIPA
YFQSMGALIPEPEVKIEVLQKPFICHRKTKGGDLMLVHYEGYLEKDGSLFHSTHKHNNGQPIWFT
LGILEALKGWDQGLKGMCVGEKRKLIIPPALGYGKEGKGKIPPESTLIFNIDLLEIRNGP
>4DIQA
LRRRYTMASGPQVDNTGGEPAWDSPLRRVLAELNRIPSSRRRAARLFEWLIAPMPPDHFYRRLWE
REAVLVRRQDHTYYQGLFSTADLDSMLRNEEVQFGQHLDAARYINGRRETLNPPGRALPAAAWSL
YQAGCSLRLLCPQAFSTTVWQFLAVLQEQFGSMAGSNVYLTPPNSQGFAPHYDDIEAFVLQLEGR
KLWRVYRPRAPTEELALTSSPNFSQDDLGEPVLQTVLEPGDLLYFPRGFIHQAECQDGVHSLHLT
LSTYQRNTWGDFLEAILPLAVQAAMEENVEFRRGLPRDFMDYMGAQHSDSKDPRRTAFMEKVRVL
VARLGHFAPVDAVADQRAKDFIHDSLPPVLTDRERALSVYGLPIRWEAGEPVNVGAQLTTETEVH
MLQDGIARLVGEGGHLFLYYTVENSRVYHLEEPKCLEIYPQQADAMELLLGSYPEFVRVGDLPCD
SVEDQLSLATTLYDKGLLLTKMPLALNAENLYFQ
>2LOYA
MLIYKDIFTDDELSSDSFPMKLVDDLVYEFKGKHVVRKEGEIVLAGSNPSAEEGAEDDGSDEHVE
RGIDIVLNHKLVEMNCYEDASMFKAYIKKFMKNVIDHMEKNNRDKADVDAFKKKIQGWVVSLLAK
DRFKNLAFFIGERAAEGAENGQVAIIEYRDVDGTEVPTLMLVKEAIIEEKCLEHHHHHH
>2LORA
MVNLGLSRVDDAVAAKHPGLGEYAACQSHAFMKGVFTFVTGTGMAFGLQMFIQRKFPYPLQWSLL
VAVVAGSVVSYGVTRVESEKCNNLWLFLETGQLPKDRSTDQRS
>2LONA
MSANRRWWVPPDDEDCVSEKLLRKTRESPLVPIGLGGCLVVAAYRIYRLRSRGSTKMSIHLIHTR
VAAQACAVGAIMLGAVYTMYSDYVKRMAQDAGEK
>4DG8A
GHMDSFFRKKAIVRMSQNSLLDLYAHPTVVARFSEMAALHPHREAIRDRFGSVDYRQLLDSAEQL
SDYLLEHYPQPGVCLGVYGEYSRESITCLLAILLSGHHYLYIDLKQPAAWNAELCRQVDCRLILD
CSTTPTPANGLPCVPVRHLPAAPASVARPCFAADQIAYINFSSGTTGRPKAIACTHAGITRLCLG
QSFLAFAPQMRFLVNSPLSFDAATLEIWGALLNGGCCVLNDLGPLDPGVLRQLIGERGADSAWLT
ASLFNTLVDLDPDCLGGLRQLLTGGDILSVPHVRRALLRHPRLHLVNGYGPTENTTFTCCHVVTD
DDLEEDDIPIGKAIAGTAVLLLDEHGQEIAEPDRAGEIVAFGAGLAQGYRNDAARTRASFVELPY
RGRLLRAYRTGDRARYDEQGRLRFIGRGDGQVKLNGYRLDLPALEQRFRRQPGILDCALLVRERN
GVKQLLCAWTGKADASPQALLRQLPTWQRPHACVRVEALPLTAHGKLDRAALLRRLEEPLERCAS
ALDPDQRGCAQLWSELLGCEVGAADQDFFLCGGNSLLALQLVALCQSAGAGANLGLADLQANSRL
DQFSRLLRSHGLAPERLLERAATPEQPLVLSRSAA
>4DEPB
EPLEADKCKEREEKIILVSSANEIDVRPCPLNPNEHKGTITWYKDDSKTPVSTEQASRIHQHKEK
LWFVPAKVEDSGHYYCVVRNSSYCLRIKISAKFVENEPNLCYNAQAIFKQKLPVAGDGGLVCPYM
EFFKNENNELPKLQWYKDCKPLLLDNIHFSGVKDRLIVMNVAEKHRGNYTCHASYTYLGKQYPIT

RVIEFITLEENKPTRPVIVSPANETMEVDLGSQIQLICNVTGQLSDIAYWKWNGSVIDEDDPVLG
EDYYSVENPANKRRSTLITVLNISEIESRFYKHPFTCFAKNTHGIDAAYIQLIYPVTNFQK
>2LOBA
HHHHHHHHHHSSGHIEGRHMENLYFQGIRKVLLLKEDHEGLGISITGGKEHGVPILISEIHPGQP
ADRCGGLHVGDAILAVNGVNLRDTKHKEAVTILSQQRGEIEFEVVYV
>4AFIA
GSPFYIKSSPSPQKRYQDTPGVEHIPVVQIDLSVPLKVPGLPMSDQYVKLEEAMAILFAVVARGT
TILAKHAWCGGNFLEVTEQILAKIPSENNKLTYSHGNYLFHYICQDRIVYLCITDDDFERSRAFS
FLNEVKKRFQTTYGSRAQTALPYAMNSEFSSVLAAQLKHHSEN
>4DCNC
GSRTVDLELELQIELLRETKRKYESVLQLGRALTAHLYSLLQTQHALGDAFADLSQKSPELQEEF
GYNAETQKLLCKNGETLLGAVNFFVSSINTLVTKTMEDTLMTVKQYEAARLEYDAYRTDLEELSL
GPRDAGTRGRLESAQATFQAHRDKYEKLRGDVAIKLKFLEENKIKVMHKQLLLFHNAVSAYFAGN
QKQ
>3VO1A
MVSTTETAEAEPVKKLEKVSKKQEEGLVTNKYKPKEPYVGRCLLNTRITGDQAPGETWHMVFSTE
GEVPYREGQSIGVIADGEDKNGKPHKLRLYSIASSALGDFGDSKTVSLCVKRLVYTNDQGEVVKG
VCSNFLCDLKPGAEVKITGPVGKEMLMPKDPNATIIMLATGTGIAPFRSFLWKMFFEEHEDYKYT
GLAWLFLGVPTSDTLLYKEELEKMKEMAPDNFRLDFAVSREQTNAAGEKMYIQTRMAEYKEELWE
LLKKDNTYVYMCGLKGMEKGIDDIMLDLAAKDGINWLDYKKQLKKSEQWNVEVY
>4DCXA
AAPDEITTAWPVNVGPLNPHLYTPNQMFAQSMVYEPLVKYQADGSVIPWLAKSWTHSEDGKTWTF
TLRDDVKFSNGEPFDAEAAAENFRAVLDNRQRHAWLELANQIVDVKALSKTELQITLKSAYYPFL
QELALPRPFRFIAPSQFKNHETMNGIKAPIGTGPWILQESKLNQYDVFVRNENYWGEKPAIKKIT
FNVIPDPTTRAVAFETGDIDLLYGNEGLLPLDTFARFSQNPAYHTQLSQPIETVMLALNTAKAPT
NELAVREALNYAVNKKSLIDNALYGTQQVADTLFAPSVPYANLGLKPSQYDPQKAKALLEKAGWT
LPAGKDIREKNGQPLRIELSFIGTDALSKSMAEIIQADMRQIGADVSLIGEEESSIYARQRDGRF
GMIFHRTWGAPYDPHAFLSSMRVPSHADFQAQQGLADKPLIDKEIGEVLATHDETQRQALYRDIL
TRLHDEAVYLPISYISMMVVSKPELGNIPYAPIATEIPFEQIKPVKP
>4DCKC
MALLRKSYSEPQLKGIVTKLYSRQGYHLQLQADGTIDGTKDEDSTYTLFNLIPVGLRVVAIQGVQ
TKLYLAMNSEGYLYTSELFTPECKFKESVFENYYVTYSSMIYRQQQSGRGWYLGLNKEGEIMKGN
HVKKNKPAAHFLPKPLKVAMYKEPSLHDLTEFSRSGSGTPTKSRSVSGVLNGGKSMSHNEST
>4DBLC
MSIVMQLQDVAESTRLGPLSGEVRAGEILHLVGPNGAGKSTLLARMAGMTSGKGSIQFAGQPLEA
WSATKLALHRAYLSQQQTPPFATPVWHYLTLHQHDKTRTELLNDVAGALALDDKLGRSTNQLSGG
EWQRVRLAAVVLQITPQANPAGQLLLLDQPMNSLDVAQQSALDKILSALSQQGLAIVMSSHDLNH
TLRHAHRAWLLKGGKMLASGRREEVLTPPNLAQAYGMNFRRLDIEGHRMLISTI
>4D97A
MRGSHHHHHHGMASMPLHHLTRFPRLEFIGAPTPLEYLPRLSDYLGREIYIKRDDVTPIAMGGNK
LRKLEFLVADALREGADTLITAGAIQSNHVRQTAAVAAKLGLHCVALLENPIGTTAENYLTNGNR
LLLDLFNTQIEMCDALTDPDAQLQTLATRIEAQGFRPYVIPVGGSSALGAMGYVESALEIAQQCE
EVVGLSSVVVASGSAGTHAGLAVGLEHLMPDVELIGVTVSRSVAEQKPKVIALQQAIAGQLALTA
TADIHLWDDYFAPGYGVPNDAGMEAVKLLASLEGVLLDPVYTGKAMAGLIDGISQKRFNDDGPIL

FIHTGGAPALFAYHPHVTYPE

>4D8QH

MSLRLPQNPNAGLFKQGYNSYSNADGQIIKSIAAIRELHQMCLTSMGPCGRNKIIVNHLGKIIIT
NDAATMLRELDIVHPAVKVLVMATEQQKIDMGDGTNLVMILAGELLNVSEKLISMGLSAVEIIQG
YNMARKFTLKELDEMVVGEITDKNDKNELLKMIKPVISSKKYGSEDILSELVSEAVSHVLPVAQQ
AGEIPYFNVDSIRVVKIMGGSLSNSTVIKGMVFNREPEGHVKSLSEDKKHKVAVFTCPLDIANTE
TKGTVLLHNAQEMLDFSKGEEKQIDAMMKEIADMGVECIVAGAGVGELALHYLNRYGILVLKVPS
KFELRRLCRVCGATPLPRLGAPTPEELGLVETVKTMEIGGDRVTVFKQEQGEISRTSTIILRGAT
QNNLDDIERAIDDGVAAVKGLMKPSGGKLLPGAGATEIELISRITKYGERTPGLLQLAIKQFAVA
FEVVPRTLAETAGLDVNEVLPNLYAAHNVTEPGAVKTDHLYKGVDIDGESDEGVKDIREENIYDM
LATKKFAINVATEAATTVLSIDQIIMAKKAGGPRAPQGPRPGNWDQED

>4D87A

MSNKYRVRKNVLHLTDTEKRDFVRTVLILKEKGIYDRYIAWHGAAGKFHTPPGSDRNAAHMSSAF
LPWHREYLLRFERDLQSINPEVTLPYWEWETDAQMQDPSQSQIWSADFMGGNGNPIKDFIVDTGP
FAAGRWTTIDEQGNPSGGLKRNFGATKEAPTLPTRDDVLNALKITQYDTPPWDMTSQNSFRNQLE
GFINGPQLHNRVHRWVGGQMGVVPTAPNDPVFFLHHANVDRIWAVWQIIHRNQNYQPMKNGPFGQ
NFRDPMYPWNTTPEDVMNHRKLGYVYDIELRKSKRSSHHHHHH

>3VF0B

HMLDPEEIRKRLEHTERQFRNRRKILIRGLPGDVTNQEVHDLLSDYELKYCFVDKYKGTAFVTLL
NGEQAEAAINAFHQSRLRERELSVQLQPTDALLCVANLPPSLTQQQFEELVRPFGSLERCFLVYS
ERTGQSKGYGFAEYMKKDSAARAKSDLLGKPLGPRTLYVHWTDAGQLTPALLHSRCLCVDRLPPG
FNDVDALCRALSAVHSPTFCQLACGQDGQLKGFAVLEYETAEMAEEAQQQADGLSLGGSHLRVSF
CAPGPPGRSMLAALIAAQATALNRG

>3VFDA

ESGAVPKRKDPLTHTSNSLPRSKTVMKTGSAGLSGHHRAPSYSGLSMVSGVKQGSGPAPTTHKGT
PKTNRTNKPSTPTTATRKKKDLKNFRNVDSNLANLIMNEIVDNGTAVKFDDIAGQDLAKQALQEI
VILPSLRPELFTGLRAPARGLLLFGPPGNGKTMLAKAVAAESNATFFNISAASLTSKYVGEGEKL
VRALFAVARELQPSIIFIDQVDSLLCERREGEHDASRRLKTEFLIEFDGVQSAGDDRVLVMGATN
RPQELDEAVLRRFIKRVYVSLPNEETRLLLLKNLLCKQGSPLTQKELAQLARMTDGYSGSDLTAL
AKDAALGPIRELKPEQVKNMSASEMRNIRLSDFTESLKKIKRSVSPQTLEAYIRWNKDFGDTTV

>2LO0A

SVDVAVSAGAGERASAEQKESYEPPKPAVGPSGESVVATEAFWDDLQGFLEQRLKDYDEANKLRV
LFKEAWRSSF

>3VE0I

YPYDVPDYAIEGRGARSMPLGVVTNSTLEVTEIDQLVCKDHLASTDQLKSVGLNLEGSGVSTDIP
SATKRWGFRSGVPPKVVSYEAGEWAENCYNLEIKKPDGSECLPPPPDGVRGFPRCRYVHKAQGTG
PCPGDYAFHKDGAFFLYDRLASTVIYRGVNFAEGVIAFLILAKPKETFLQSPPIREAVNYTENTS
SYYATSYLEYEIENFGAQHSTTLFKINNNTFVLLDRPHTPQFLFQLNDTIHLHQQLSNTTGKLIW
TLDANINADIGEWAFWENKKNLSEQLRGEELSFETLSL

>4AE2A

ETGHHHHHHSADEPMDFKINTDEIMTSLKSVNGQIESLISPDGSRKNPARNCRDLKFCHPELKSG
EYWVDPNQGCKLDAIKVFCNMETGETCISANPLNVPRKHWWTDSSAEKKHVWFGESMDGGFQFSY
GNPELPEDVLDVQLAFLRLLSSRASQQITYHCKNSIAYMDQASGNVKKALKLMGSNEGEFKAEGN
SKFTYTVLEDGCTKHTGEWSKTVFEYRTRKAVRLPIVDIAPYDIGGPDQEFGVDVGPVCFL

>3VDIA

MALFGTKDTTTAHSDYEIILEGGSSSWGQIKGRAKVNVPAALPLLPADCNIKIEAKPLDAQKGVV
RFTSQIESIVDSTKNKLVVEVDIANETKDRRIAVGEGEVSVGDFSHKFSFEGSVVNMYYYRSDAV
RRNVPNPVYMQGRQFHDIMMKVPLDNKDLIETWEGFQQSISGGGVNFGDWIREFWFIGPAYTAIN
EGGQRISPIQVNNFGVESGEKGPVGVSRWKFSHAGSGIVDSISRWAELFPVEQLNKPASIEGGFR
SDSQGIEVKVDGNLPGVSRDAGGGLRRILNHPLIPLVHHGMVGKFNDFTVDTQLKVVLPKGYKIR
YAAPQFRSQNLEEYRWSGGAYARWVEHVCKGGTGQFEVLYAQ

>3VDJA

YVEFEPSDKHIKEYLNKIQNSLSTEWSPCSVTCGNGIQVRIKPGSANKPKDELDYANDIEKKICK
MEKCPHHHHHHA

>4ADZA

GSHMTTTEAGASAPSPAVDGAVNQTARQAEADGTDIVTDHDRGVHGYHKQKAEHLKRLRRIEGQI
RGLQRMVDEDVYCIDILTQVSASTKALQSFALQLLEEHLRHCVADAALKGGTEIDAKVEEATKAI
GRLLRT

>3VBAA

MRSIIKGRVWKFGNNVDTDAILPARYLVYTKPEELAQFVMTGADPDFPKKVKPGDIIVGGKNFGC
GSSREHAPLGLKGAGISCVIAESFARIFYRNAINVGLPLIECKGISEKVNEGDELEVNLETGEIK
NLTTGEVLKGQKLPEFMMEILEAGGLMPYLKKKMAESQLEHHHHHH

>3V9OA

GPGSMTGTTMFAALLHPRLADCRRLYLRNHEVYMNIGAFEHEKRGEQRVVINVDLFVPLALTTPV
EDKLREVVDYDLMKQSVAQCVARGHIHLQETLCDAIAASLLAHDAVRAVRVSTEKPDAYPDCDAV
GVEVFRIKDEERA

>3V9BA

IPRFGVKTEQEDVLAKELEDVNKWGLHVFRIAELSGNRPLTVIMHTIFQERDLLKTFKIPVDTLI
TYLMTLEDHYHADVAYHNNIHAADVVQSTHVLLSTPALEAVFTDLEILAAIFASAIHDVDHPGVS
NQFLINTNSELALMYNDSSVLENHHLAVGFKLLQEENCDIFQNLTKKQRQSLRKMVIDIVLATDM
SKHMNLLADLKTMVETKKVTSSGVLLLDNYSDRIQVLQNMVHCADLSNPTKPLQLYRQWTDRIME
EFFRQGDRERERGMEISPMCDKHNASVEKSQVGFIDYIVHPLWETWADLVHPDAQDILDTLEDNR
EWYQSTIPQSPSPAPDDPEEGRQGQTEKFQFELTL

>2LNAA

MGHHHHHHSHMKRSGREITWKDFVNNYLSKGVVDRLEVVNKRFVRVTFTPGKTPVDGQYVWFNIG
SVDTFERNLETLQQELGIEGENRVPVVYIAESDG

>3V65B

TGEENCNVNNGGCAQKCQMIRGAVQCTCHTGYRLTEDGRTCQDVNECAEEGYCSQGCTNSEGAFQ
CWCEAGYELRPDRRSCKALGPEPVLLFANRIDIRQVLPHRSEYTLLLNNLENAIALDFHHRRELV
FWSDVTLDRILRANLNGSNVEEVVSTGLESPGGLAVDWVHDKLYWTDSGTSRIEVANLDGAHRKV
LLWQSLEKPRAIALHPMEGTIYWTDWGNTPRIEASSMDGSGRRIIADTHLFWPNGLTIDYAGRRM
YWVDAKHHVIERANLDGSHRKAVISQGLPHPFAITVFEDSLYWTDWHTKSINSANKFTGKNQEII
RNKLHFPMDIHTLHPQRQPAGKNRCGDNNGGCTHLCLPSGQNYTCACPTGFRKINSHACAQ

>3V53A

MGHHHHHHMKRKHIKSLIEKIPTAKPELFAYPLDWSIVDSILMERRIRPWINKKIIEYIGEEEAT
LVDFVCSKVMAHSSPQSILDDVAMVLDEEAEVFIVKMWRLLIYETEAKKIGLVK

>3V57B

MLDAFSRVVVNSDAKAAYVGGSDLQALKSFIADGNKRLDAVNSIVSNASCMVSDAVSGMICENPG

LISPGGNCYTNRRMAACLRDGEIILRYVSYALLAGDASVLEDRCLNGLKETYIALGVPTNSSIRA
VSIMKAQAVAFITNTATERKMSFAAGDCTSLASEVASYFDRVGAAIS

>3V43A

HMEPIPICSFCLGTKEQNREKKPEELISCADCGNSGHPSCLKFSPELTVRVKALRWQCIECKTCS
SCRDQGKNADNMLFCDSCDRGFHMECCDPPLTRMPKGMWICQICRPR

>4AC5C

CFEPPPATTTQTGFRGLSMGEVLHPATVKAKKERDAQYPPALAAVKAEGPPVSQVYKNVKVLGNL
TEAEFLRTMTAITEWVSPQEGCTYCHDENNLASEAKYPYVVARRMLEMTRAINTNWTQHVAQTGV
TCYTCHRGTPLPPYVRYLEPTLPLNNRETPTHVERVETRSGYVVRLAKYTAYSALNYDPFTMFLA
NDKRQVRVVPQTALPLVGVSRGKERRPLSDAYATFALMMSISDSLGTNCTFCHNAQTFESWGKKS
TPQRAIAWWGIRMVRDLNMNYLAPLNASLPASRLGRQGEAPQADCRTCHQGVTKPLFGASRLKDY
PELGPIKAAAK

>3V48A

GHMKLSLSPPPYADAPVVVLISGLGGSGSYWLPQLAVLEQEYQVVCYDQRGTGNNPDTLAEDYSI
AQMAAELHQALVAAGIEHYAVVGHALGALVGMQLALDYPASVTVLISVNGWLRINAHTRRCFQVR
ERLLYSGGAQAWVEAQPLFLYPADWMAARAPRLEAEDALALAHFQGKNNLLRRLNALKRADFSHH
ADRIRCPVQIICASDDLLVPTACSSELHAALPDSQKMVMPYGGHACNVTDPETFNALLLNGLASL
LHHREAAL

>3V2AR

MQSKVLLAVALWLCVETRAASVGLPSVSLDLPRLSIQKDILTIKANTTLQITCRGQRDLDWLWPN
NQSGSEQRVEVTECSDGLFCKTLTIPKVIGNDTGAYKCFYRETDLASVIYVYVQDYRSPFIASVS
DQHGVVYITENKNKTVVIPCLGSISNLNVSLCARYPEKRFVPDGNRISWDSKKGFTIPSYMISYA
GMVFCEAKINDESYQSIMYIVVVVGYRIYDVVLSPSHGIELSVGEKLVLNCTARTELNVGIDFNW
EYPSSKHQHKKLVNRDLKTQSGSEMKKFLSTLTIDGVTRSDQGLYTCAASSGLMTKKNSTFVRVH
EKPFVAFGSGMESLVEATVGERVRIPAKYLGYPPPEIKWYKNGIPLESNHTIKAGHVLTIMEVSE
RDTGNYTVILTNPISKEKQSHVVSLVVYVPPQIGEKSLISPVDSYQYGTTQTLTCTVYAIPPPHH
IHWYWQLEEECANEPSQAVSVTNPYPCEEWRSVEDFQGGNKIEVNKNQFALIEGKNKTVSTLVIQ
AANVSALYKCEAVNKVGRGERVISFHVTRGPEITLQPDMQPTEQESVSLWCTADRSTFENLTWYK
LGPQPLPIHVGELPTPVCKNLDTLWKLNATMFSNSTNDILIMELKNASLQDQGDYVCLAQDRKTK
KRHCVVRQLTVLERVAPTITGNLENQTTSIGESIEVSCTASGNPPPQIMWFKDNETLVEDSGIVL
KDGNRNLTIRRVRKEDEGLYTCQACSVLGCAKVEAFFIIEGAQEKTNLERTHHHHHH

>3J16C

MKLNISYPVNGSQKTFEIDDEHRIRVFFDKRIGQEVDGEAVGDEFKGYVFKISGGNDKQGFPMKQ
GVLLPTRIKLLLTKNVSCYRPRRDGERKRKSVRGAIVGPDLAVLALVIVKKGEQELEGLTDTTVP
KRLGPKRANNIRKFFGLSKEDDVRDFVIRREVTKGEKTYTKAPKIQRLVTPQRLQRKRHQRALKV
RNAQAQREAAAEYAQLLAKRLSERKAEKAEIRKRRASSLKA

>3VMGA

MANVDEAILKRVKGWAPYVDAKLGFRNHWYPVMFSKEINEGEPKTLKLLGENLLVNRIDGKLYCL
KDRCLHRGVQLSVKVECKTKSTITCWYHAWTYRWEDGVLCDILTNPTSAQIGRQKLKTYPVQEAK
GCVFIYLGDGDPPPLARDTPPNFLDDDMEILGKNQIIKSNWRLAVENGFDPSHIYIHKDSILVKD
NDLALPLGFAPGGDRKQQTRVVDDDVVGRKGVYDLIGEHGVPVFEGTIGGEVVREGAYGEKIVAN
DISIWLPGVLKVNPFPNPDMMQFEWYVPIDENTHYYFQTLGKPCANDEERKKYEQEFESKWKPMA
LEGFNNDDIWAREAMVDFYADDKGWVNEILFESDEAIVAWRKLASEHNQGIQTQAHVSGLEHHHH
HH

>3V33A
GGGTPKAPNLEPPLPEEEKEGSDLRPVVIDGSNVAMSHGNKEVFSCRGILLAVNWFLERGHTDIT
VFVPSWRKEQPRPDVPITDQHILRELEKKKILVFTPSRRVGGKRVVCYDDRFIVKLAYESDGIVV
SNDTYRDLQGERQEWKRFIEERLLMYSFVNDKFMPPDDPLGRHGPSLDNFLRKKPLTLEHRKQPC
PYGRKCTYGIKCRFFHPERPSCPQRSVA
>3V22V
MKRQKRDRLERAHQRGYQAGIAGRSKEMCPYQTLNQRSQWLGGWREAMADRVVMAHHHHHH
>4ABRL
MVALPTINQLVRKGREKVRKKSKVPALKGAPFRRGVCTVVRTVTPKKPNSALRKVAKVRLTSGYE
VTAYIPGEGHNLQEHSVVLIRGGRVKDLPGVRYHIVRGVYDAAGVKDRKKSRSKYGTKKPKEAAK
TAAKK
>3VM6A
MNHKVHHHHHHIEGRHMAVVKEVLEIAEKIKNMEIRGAGKIARSAAYALQLQAEKSKATNVDEFW
KEMKQAAKILFETRPTAVSLPNALRYVMHRGKIAYSSGADLEQLRFVIINAAKEFIHNSEKALER
IGEFGAKRIEDGDVIMTHSHSKAAISVMKTAWEQGKDIKVIVTETRPKWQGKITAKELASYGIPV
IYVVDSAARHYMKMTDKVVMGADSITVNGAVINKIGTALIALTAKEHRVWTMIAAETYKFHPETM
LGQLVEIEMRDPTEVIPEDELKTWPKNIEVWNPAFDVTPPEYVDVIITERGIIPPYAAIDILREE
FGWALKYTEPWED
>3J0TL
MKTFTAKPETVKRDWYVVDATGKTLGRLATELARRLRGKHKAEYTPHVDTGDYIIVLNADKVAVT
GNKRTDKVYYHHTGHIGGIKQATFEEMIARRPERVIEIAVKGMLPKGPLGRAMFRKLKVYAGNEH
NHAAQQPQVLDI
>3UXQD
MAVKKFKPYTPSRRFMTVADFSEITKTEPEKSLVKPLKKTGGRNNQGRITVRFRGGGHKRLYRII
DFKRWDKVGIPAKVAAIEYDPNRSARIALLHYVDGEKRYIIAPDGLQVGQQVVAGPDAPIQVGNA
LPLRFIPVGTVVHAVELEPKKGAKLARAAGTSAQIQGREGDYVILRLPSGELRKVHGECYATVGA
VGNADHKNIVLGKAGRSRWLGRRPHVRGAAMNPVDHPHGGGEGRAPRGRPPASPWGWQTKGLKTR
KRRKPSSRFIIARRKK
>3UX2A
SNARIMEEKALEVYDLIRTIRDPEKPNTLEELEVVSESCVEVQEINEEEYLVIIRFTPTVPHCSL
ATLIGLCLRVKLQRCLPFKHKLEIYISEGTHSTEEDINKQINDKERVAAAMENPNLREIVEQCVL
>3UW8A
MAETPNSDMSGATGGRSKRPKSNQDWWPSKLNLEILDQNARDVGPVEDDFDYAEEFQKLDLEAVK
SDLEELMTSSQDWWPADYGHYGPLFIRMAWHSAGTYRTADGRGGAAGGRQRFAPINSWPDNANLD
KARRLLLPIKQKYGQKISWADLMILAGNVAIESMGFKTFGYAGGREDAFEEDKAVNWGPEDEFET
QERFDEPGEIQEGLGASVMGLIYVNPEGPDGNPDPEASAKNIRQTFDRMAMNDKETAALIAGGHT
FGKVHGADDPEENLGPEPEAAPIEQQGLGWQNKNGNSKGGEMITTGIEGPWTQSPTEWDMGYINN
LLDYEWEPEKGPGGAWQWAPKSEELKNSVPDAHDPDEKQTPMMLTTDIALKRDPDYREVMETFQE
NPMEFGMNFAKAWYKLTHRDMGPPERFLGPEVPDEEMIWQDPLPDADYDLIGDEEIAELKEEILD
SDLSVSQLVKTAWASASTYRDSDKRGGANGARLRLEPQKNWEVNEPEQLETVLGTLENIQTEFND
SRSDGTQVSLADLIVLGGNAAVEQAAANAGYDVEIPFEPGRVDAGPEHTDAPSFDALKPKVDGVR
NYIQDDITRPAEEVLVDNADLLNLTASELTALIGGMRSIGANYQDTDLGVFTDEPETLTNDFFVN
LLDMGTEWEPAADSEHRYKGLDRDTGEVKWEATRIDLIFGSNDRLRAISEVYGSADAEKKLVHDF
VDTWSKVMKLDRFDLEHHHHHH

>3UW2A
MAHHHHHHMGTLEAQTQGPGSMISQSIFKAYDIRGVIGKTLDADVARSIGRAFGSEVRAQGGDAV
VVARDGRLSGPELVGALADGLRAAGVDVVDVGMVPTPVGYFAASVPLALSGGERRVDSCIVVTGS
HNPPDYNGFKMVLRGAAIYGDQIQGLYKRIVDARFETGSGSYEQYDVADQYVERIVGDIKLTRPL
KLVVDAGNGVAGPLATRLFKALGCELVELFTDIDGNFPNHHPDPAHPENLQDVIAKLKATDAEIG
FAFDGDGDRLGVVTKDGQIIYPDRQLMLFAEEVLSRNPGAQIIYDVKCTRNLARWVREKGGEPLM
WKTGHSLVKAKLRETGAPLAGEMSGHVFFKDRWYGFDDGLYTGARLLEILARVADPSALLNGLPN
AVSTPELQLKLEEGENVKLIDKLRADAKFDGADEVVTIDGLRVEYPDGFGLARSSNTTPVVVLRF
EATSDAALARIQDDFRRALKAAKPGANLPF
>3UV1A
DPIHYDKITEEINKAIDDAIAAIEQSETIDPMKVPDHADKFERHVGILDFKGELAMRNIEARGLK
QMKRQGDANVKGEEGIVKAHLLIGVHDDIVSMEYDLAYKLGDLHPTTHVISDIQDFVVALSLEIP
DEGNITMTSFEVRQFANVVNHIGGLSILDPIFGVLSDVLTAIFQDTVRKEMTKVLAPAFKRELEK
N
>4A9WA
MDSVDVVVIGGGQSGLSAGYFLRRSGLSYVILDAEASPGGAWQHAWHSLHLFSPAGWSSIPGWPM
PASQGPYPARAEVLAYLAQYEQKYALPVLRPIRVQRVSHFGERLRVVARDGRQWLARAVISATGT
WGEAYTPEYQGLESFAGIQLHSAHYSTPAPFAGMRVAIIGGGNSGAQILAEVSTVAETTWITQHE
PAFLADDVDGRVLFERATERWKAQQEGREPDLPPGGFGDIVMVPPVLDARARGVLAAVPPPARFS
PTGMQWADGTERAFDAVIWCTGFRPALSHLKGLDLVTPQGQVEVDGSGLRALAVPSVWLLGYGDW
NGMASATLIGVTRYAREAVRQVTAYCADHQDR
>3UV2A
SMQCQSTEDAMTVLTPLTEKDYEGLKRVLRSLQAHKMAWPFLEPVDPNDAPDYYGVIKEPMDLAT
MEERVQRRYYEKLTEFVADMTKIFDNCRYYNPSDSPFYQCAEVLESFFVQKLKGFKASRSH
>3UUMA
DMDLDSYQIALEEVLTWLLSAEDTFQEQDDISDDVEDVKEQFATHETFMMELSAHQSSVGSVLQA
GNQLMTQGTLSDEEEFEIQEQMTLLNARWEALRVESMERQSRLHDALMELQKKQLQQL
>3UTNX
MGSSHHHHHHSSGLVPRGSHMASMPLFDLISPKAFVKLVASEKVHRIVPVDATWYLPSWKLDNKV
DFLTKPRIPNSIFFDIDAISDKKSPYPHMFPTKKVFDDAMSNLGVQKDDILVVYDRVGNFSSPRC
AWTLGVMGHPKVYLLNNFNQYREFKYPLDSSKVAAFSPYPKSHYESSESFQDKEIVDYEEMFQLV
KSGELAKKFNAFDARSLGRFEGTEPEPRSDIPSGHIPGTQPLPYGSLLDPETKTYPEAGEAIHAT
LEKALKDFHCTLDPSKPTICSCGTGVSGVIIKTALELAGVPNVRLYDGSWTEWVLKSGPEWIAEN
RD
>4A9AA
MHHHHHHMSTTVEKIKAIEDEMARTQKNKATSFHLGQLKAKLAKLRRELLTSASSGSGGGAGIGF
DVARTGVASVGFVGFPSVGKSTLLSKLTGTESEAAEYEFTTLVTVPGVIRYKGAKIQMLDLPGII
DGAKDGRGRGKQVIAVARTCNLLFIILDVNKPLHHKQIIEKELEGVGIRLNKTPPDILIKKKEKG
GISITNTVPLTHLGNDEIRAVMSEYRINSAEIAFRCDATVDDLIDVLEASSRRYMPAIYVLNKID
SLSIEELELLYRIPNAVPISSGQDWNLDELLQVMWDRLNLVRIYTKPKGQIPDFTDPVVLRSDRC
SVKDFCNQIHKSLVDDFRNALVYGSSVKHQPQYVGLSHILEDEDVVTILKK
>4A91A
MTDTQYIGRFAPSPSGELHFGSLIAALGSYLQARARQGRWLVRIEDIDPPREVPGAAETILRQLE
HYGLHWDGDVLWQSQRHDAYREALAWLHEQGLSYYCTCTRARIQSIGGIYDGHCRVLHHGPDNAA

VRIRQQHPVTQFTDQLRGIIHADEKLAREDFIIHRRDGLFAYNLAVVVDDHFQGVTEIVRGADLI
EPTVRQISLYQLFGWKVPDYIHLPLALNPQGAKLSKQNHAPALPKGDPRPVLIAALQFLGQQAEA
HWQDFSVEQILQSAVKNWRLTAVPESAIVNSTFSNASC
>3UR1C
SQIGETLENIRSIEKLIQNIMRIARETNILALNATIEAARAGEAGKGFMIVANEVQNLSNETNEV
TKQIVEKAREILESSQRSLE
>4A8JC
MHHHHHHMGSVQRQDLVLFSDQSVLPAHFFQDSNSHNLFFITHQSCTQPLWMINALVETHVLGSP
SSLNESSSSMLPSSTRSHAVLASFIHEQNYFTNSLNKLKIPSNNYNVLDFLSDFIVNNIHNKPRD
KILSDVLAKFSAAIQNNPTDTIVIIEQPELLLSLVSGLTCSELNNKFITPLLRQCKVLIIVSNSD
IFNIDEYDASVHSSNLQNFYKSSFIKSMINLNLNPLKTGFAKDVTGSLHVCRGGAPIATSNTSLH
VVENEYLYLNEKESTKLFYR
>3UR1B
KEFEVLSFEIDEQALAFDVDNIEMVIEKSDITPVPKSRHFVEGVINLRGRIIPVVNLAKILGISF
DEQKMKSIIVARTKDVEVGFLVDRVLGVLRITENQLDLTNVSDKFGKKSKGLVKTDGRLIIYLDI
DKIIEEITV
>4A8JB
MASSSHNPVILLKRILSLTESSPFILCLDSIAQTSYKLIQEFVHQSKSKGNEYPIVYISFETVNK
PSYCTQFIDATQMDFVHLVKQIISYLPAATATQAKKHMVIIDSLNYISTEYITRFLSEIASPHCT
MVATYHKDIKDENRTVIPDWNNNYPDKLTLLQFMATTIVDIDVVLTGTLDTEEVSELLNEFRIPR
GLNNDIFQLRLVNKRKSGRSLEYDFIVNSNTHEYELLSTTKQEEESSSNGLETPEMLQGLTTFNL
GTSNKQKLAK
>3UPIA
SMSYTWTGALITPCAAEESKLPINPLSNSLLRHHNMVYATTSRSASLRQKKVTFDRLQVLDDHYR
DVLKEMKAKASTVKAKLLSIEEACKLTPPHSAKSKFGYGAKDVRNLSSRAVNHIRSVWEDLLEDT
ETPIDTTIMAKSEVFCVQPEKGGRKPARLIVFPDLGVRVCEKMALYDVVSTLPQAVMGSSYGFQY
SPKQRVEFLVNTWKSKKCPMGFSYDTRCFDSTVTESDIRVEESIYQCCDLAPEARQAIRSLTERL
YIGGPLTNSKGQNCGYRRCRASGVLTTSCGNTLTCYLKATAACRAAKLQDCTMLVNGDDLVVICE
SAGTQEDAAALRAFTEAMTRYSAPPGDPPQPEYDLELITSCSSNVSVAHDASGKRVYYLTRDPTT
PLARAAWETARHTPINSWLGNIIMYAPTLWARMILMTHFFSILLAQEQLGKALDCQIYGACYSIE
PLDLPQIIERLHGLSAFTLHSYSPGEINRVASCLRKLGVPPLRTWRHRARSVRAKLLSQGGRAAI
CGRYLFNWAVRTKLKLTPIPAASQLDLSGWFVAGYSGGDIYHSLSRARPRENLYFQGLEHHHHHH
>3UOQG
MPRRRVIGQRKILPDPKFGSELLAKFVNILMVDGKKSTAESIVYSALETLAQRSGKSELEAFEVA
LENVRPTVEVKSRRVGGSTYQVPVEVRPVRRNALAMRWIVEAARKRGDKSMALRLANELSDAAEN
KGTAVKKREDVHRMAEANKAFAHYRWLSLRSFSHQAGASSKQPALGYLN
>3VKFA
SQKLDDVDPLVTTNFGKIRGIKKELNNEILGPVIQFLGVPYAAPPTGEHRFQPPEPPSPWSDIRN
ATQFAPVCPQNIIDGRLPEVMLPVWFTNNLDVVSSYVQDQSEDCLYLNIYVPTEDVKRISKECAR
KPGKKICRKGDIRDSGGPKPVMVYIHGGSYMEGTGNLYDGSVLASYGNVIVITVNYRLGVLGFLS
TGDQAAKGNYGLLDLIQALRWTSENIGFFGGDPLRITVFGSGAGGSCVNLLTLSHYSEGLFQRAI
AQSGTALSSWAVSFQPAKYARILATKVGCNVSDTVELVECLQKKPYKELVDQDVQPARYHIAFGP
VIDGDVIPDDPQILMEQGEFLNYDIMLGVNQGEGLKFVENIVDSDDGVSASDFDFAVSNFVDNLY
GYPEGKDVLRETIKFMYTDWADRHNPETRRKTLLALFTDHQWVAPAVATADLHSNFGSPTYFYAF

YHHCQTDQVPAWADAAHGDEVPYVLGIPMIGPTELFPCNFSKNDVMLSAVVMTYWTNFAKTGDPN
QPVPQDTKFIHTKPNRFEEVAWTRYSQKDQLYLHIGLKPRVKEHYRANKVNLWLELVPHLHNLND
>4A7FB
ELDRAQERLATALQKLEEAEKAADESERGMKVIESRAQKDEEKMEIQEIQLKEAKHIAEDADRKY
EEVARKLVIIESDLERAEERAELSEGKCAELEEELKTVTNNLKSLEAQAEKYSQKEDKYEEEIKV
LSDKLK
>4A7KA
MYSKVFLKPHCEPEQPAALPLFQPQLVQGGRPDGYWVEAFPFRSDSSKCPNIIGYGLGTYDMKSD
IQMLVNPYATTNNQSSSWTPVPLAKLDFPVAMHYADITKNGFNDVIITDQYGSSMDDIWAYGGRV
SWLENPGELRDNWTMRTIGHSPGMHRLKAGHFTRTDRVQVVAVPIVVASSDLTTPADVIIFTAPD
DPRSEQLWQRDVVGTRHLVHEVAIVPAAETDGEMRFDQIILAGRDGVDCLWYDGARWQRHLVGTG
LPEERGDPYWGAGSAAVGRVGDDYAGYICSAEAFHGNTVSVYTKPAGSPTGIVRAEWTRHVLDVF
GPLNGKHTGSIHQVVCADIDGDGEDEFLVAMMGADPPDFQRTGVWCYKLVDRTNMKFSKTKVSSV
SAGRIATANFHSQGSEVDIATISYSVPGYFESPNPSINVFLSTGILAERLDEEVMLRVVRAGSTR
FKTEMEFLDVAGKKLTLVVLPPFARLDVERNVSGVKVMAGTVCWADENGKHERVPATRPFGCESM
IVSADYLESGEEGAILVLYKPSSTSGRPPFRSMDELVAHNLFPAYVPDSVRAMKFPWVRCADRPW
AHGRFKDLDFFNLIGFHVNFADDSAAVLAHVQLWTAGIGVSAGFHNHVEASFCEIHACIANGTGR
GGMRWATVPDANFNPDSPNLEDTELIVVPDMHEHGPLWRTRPDGHPLLRMNDTIDYPWHAWLAGA
GNPSPQAFDVWVAFEFPGFETFSTPPPPRVLEPGRYAIRFGDPHQTASLALQKNDATDGTPVLAL
LDLDGGPSPQAWNISHVPGTDMYEIAHAKTGSLVCARWPPVKNQRVAGTHSPAAMGLTSRWAVTK
NTKGQITFRLPEAPDHGPLFLSVSAIRHQQEADAIPVIVQGDSIELSAWSLVPAN
>3UMFA
MGSSHHHHHHSSGLVPRGSHMTDQKLAKAKVIFVLGGPGSGKGTQCEKLVQKFHFNHLSSGDLLR
AEVQSGSPKGKELKAMMERGELVPLEVVLALLKEAMIKLVDKNCHFLIDGYPRELDQGIKFEKEV
CPCLCVINFDVSEEVMRKRLLKRAETSNRVDDNEETIVKRFRTFNELTKPVIEHYKQQNKVITID
ASGTVDAIFDKVNHELQKFGVK
>2LLIA
KEAAPKCNNCSQRGHLKKDCPHIICSYCGATDDHYSRHCPKAIQCSKCDEVGHYRSQCPHKWKKV
QCTLCKSKKHSKERCPSIWRAYILVDDNEKAKPKVLPFHTIYCYNCGGKGHFGDDCKEK
>4A6SA
AWKGEVLANNEAGQVTSIIYNPGDVITIVAAGWASYGPTQKWGPQGDREHPDQGLICHDAFCGAL
VMKIGNSGTIPVNTGLFRWVAPNNVQGAITLIYNDVPGTYGNNSGSFSVNIGKDQS
>3UILA
EDPPACGSIVPRREWRALASECRERLTRPVRYVVVSHTAGSHCDTPASCAQQAQNVQSYHVRNLG
WCDVGYNFLIGEDGLVYEGRGWNIKGAHAGPTWNPISIGISFMGNYMNRVPPPRALRAAQNLLAC
GVALGALRSNYEVKGHRDVQPTLSPGDRLYEIIQTWSHYRA
>3UGJA
GLVPRGSHMMEILRGSPALSAFRINKLLARFQAANLQVHNIYAEYVHFADLNAPLNDSEQAQLTR
LLQYGPALSSHTPAGKLLLVTPRPGTISPWSSKATDIAHNCGLQQVDRLERGVAYYIEASTLTAE
QWRQVAAELHDRMMETVFSSLTDAEKLFIHHQPAPVSSVDLLGEGRQALIDANLRLGLALAEDEI
DYLQEAFTKLGRNPNDIELYMFAQANSEHCRHKIFNADWIIDGKPQPKSLFKMIKNTFETTPDYV
LSAYKDNAAVMEGSAVGRYFADHNTGRYDFHQEPAHILMKVETHNHPTAISPWPGAATGSGGEIR
DEGATGRGAKPKAGLVGFSVSNLRIPGFEQPWEEDFGKPERIVTALDIMTEGPLGGAAFNNEFGR
PALTGYFRTYEEKVNSHNGEELRGYHKPIMLAGGIGNIRADHVQKGEIVVGAKLIVLGGPAMNIG

LGGGAASSMASGQSDADLDFASVQRDNPEMERRCQEVIDRCWQLGDANPILFIHDVGAGGLSNAM
PELVSDGGRGGKFELRDILSDEPGMSPLEIWCNESQERYVLAVAADQLPLFDELCKRERAPYAVI
GDATEEQHLSLHDNHFDNQPIDLPLDVLLGKTPKMTRDVQTLKAKGDALNRADITIADAVKRVLH
LPTVAEKTFLVTIGDRTVTGMVARDQMVGPWQVPVADCAVTTASLDSYYGEAMSIGERAPVALLD
FAASARLAVGEALTNIAATQIGDIKRIKLSANWMAAAGHPGEDAGLYDAVKAVGEELCPQLGLTI
PVGKDSMSMKTRWQEGNEQREMTSPLSLVISAFARVEDVRHTLTPQLSTEDNALLLIDLGKGHNA
LGATALAQVYRQLGDKPADVRDVAQLKGFYDAMQALVAARKLLAWHDRSDGGLLVTLAEMAFAGH
CGVQVDIAALGDDHLAALFNEELGGVIQVRAEDRDAVEALLAQYGLADCVHYLGQALAGDRFVIT
ANDQTVFSESRTTLRVWWAETTWQMQRLRDNPQCADQEHEAKANDTDPGLNVKLSFDINEDIAAP
YIATGARPKVAVLREQGVNSHVEMAAAFHRAGFDAIDVHMSDLLGGRIGLGNFHALVACGGFSYG
DVLGAGEGWAKSILFNHRVRDEFETFFHRPQTLALGVCNGCQMMSNLRELIPGSELWPRFVRNHS
DRFEARFSLVEVTQSPSLLLQGMVGSQMPIAVSHGEGRVEVRDDAHLAALESKGLVALRYVDNFG
KVTETYPANPNGSPNGITAVTTENGRVTIMMPHPERVFRTVANSWHPENWGEDSPWMRIFRNARK
QLG
>3UGQA
MGSSHHHHHHSSGLVPRGSHMASATKNASSATPATMTSMVSQRQDLFMTDPLSPGSMFFLPNGAK
IFNKLIEFMKLQQKFKFGFNEVVTPLIYKKTLWEKSGHWENYADDMFKVETTDEEKEEYGLKPMN
CPGHCLIFGKKDRSYNELPLRFSDFSPLHRNEASGALSGLTRLRKFHQDDGHIFCTPSQVKSEIF
NSLKLIDIVYNKIFPFVKGGSGAESNYFINFSTRPDHFIGDLKVWNHAEQVLKEILEESGKPWKL
NPGDGAFYGPKLDIMVTDHLRKTHQVATIQLDFQLPERFDLKFKDQDNSYKRPIMIHRATFGSIE
RFMALLIDSNEGRWPFWLNPYQAVIIPVNTKNVQQLDMCTALQKKLRNELEADDMEPVPLNDWHF
NVDLDIRNEPVGYRIKSAILKNYSYLIIVGDEEVQLQKYNIRERDNRKSFEKLTMSQIWEKFIEL
EKNYK
>4A69C
GAMRQLAVIPPMLYDADQQRIKFINMNGLMADPMKVYKDRQVMNMWSEQEKETFREKFMQHPKNF
GLIASFLERKTVAECVLYYYLTKKNENYK
>4A6DA
MGSSEDQAYRLLNDYANGFMVSQVLFAACELGVFDLLAEAPGPLDVAAVAAGVRASAHGTELLLD
ICVSLKLLKVETRGGKAFYRNTELSSDYLTTVSPTSQCSMLKYMGRTSYRCWGHLADAVREGRNQ
YLETFGVPAEELFTAIYRSEGERLQFMQALQEVWSVNGRSVLTAFDLSVFPLMCDLGGGAGALAK
ECMSLYPGCKITVFDIPEVVWTAKQHFSFQEEEQIDFQEGDFFKDPLPEADLYILARVLHDWADG
KCSHLLERIYHTCKPGGGILVIESLLDEDRRGPLLTQLYSLNMLVQTEGQERTPTHYHMLLSSAG
FRDFQFKKTGAIYDAILARKGTHHHHHH
>3UEZE
GSHMEEVSEYCSHMIGSGHLQSLQRLIDSQMETSCQITFEFVDQEQLKDPVCYLKKAFLLVQDIM
EDTMRFRDNTPNAIAIVQLQELSLRLKSCFTKDYEEHDKACVRTFYETPLQLLEKVKNVFNETKN
LLDKDWNIFSKNCNNSFAECSSQ
>4A5VA
SSEPAKLDLSCVHSDNKGSRAPTIGEPVPDVSLEQCAAQCKAVDGCTHFTYNDDSKMCHVKEGKP
DLYDLTGGKTASRSCDRSCFEQHVSYEGAPDVMTAMVTSQSADCQAACAADPSCEIFTYNEHDQK
CTFKGRGFSAFKERGVLGVTSGPKQFCDEGG
>3UBBA
ERAGPVTWVMMIACVVVFIAMQILGDQEVMLWLAWPFDPTLKFEFWRYFTHALMHFSLMHILFNL
LWWWYLGGAVEKRLGSGKLIVITLISALLSGYVQQKFSGPWFGGLSGVVYALMGYVWLRGERDPQ

SGIYLQRGLIIFALIWIVAGWFDLFGMSMANGAHIAGLAVGLAMAFVDSLNA
>3UBRA
SDKTEPRNEVYKDKFKNQYNSWHDTAKSEELVDALEQDPNMVILWAGYAFAKDYKAPRGHMYAVT
DVRNTLRTGAPKNAEDGPLPMACWSCKSPDVPRLIEEQGEDGYFKGKWAKGGPEVTNTIGCSDCH
EKGSPKLRISRPYVDRALDAIGTPFSKASKQDKESMVCAQCHVEYYFEKKEDKKGFVKFPWDMGV
TVDQMEVYYDGIEFSDWTHALSKTPMLKAQHPEYETWKMGIHGKNNVSCVDCHMPKVTSPEGKKF
TDHKVGNPFDRFEETCATCHSQTKEFLVGVTNERKAKVKEMKLKAEEQLVKAHFEAAKAWELGAT
EAEMKPILTDIRHAQWRWDLAIASHGVAAHAPEEALRVLGTSVNKAADARVKLAQLLAKKGLTDP
VAIPDISTKAKAQAVLGMDMEKMNAEKEAFKKDMLPKWDAEAKKREATY
>4A53A
GAMGMSVADFYGSNVEVLLNNDSKARGVITNFDSSNSILQLRLANDSTKSIVTKDIKDLRILPKN
EIMPKNGTKSPSTNSTKLKSAETYSSKNKWSMDCDEEFDFAANLEKFDKKQVFAEFREKD
>3UA0A
MGHHHHHHMRVKTFVILCCALQYVAYTNANINDFDEDYFGSDVTVQSSNTTDEIIRDASGAVIEE
QITTKKMQRKNKNHGILGKNEKMIKTFVITTDSDGNESIVEEDVLMKTLSDGTVAQSYVAADAGA
YSQS
>3U9GA
GPLGMADPGVCCFITKILCAHGGRMTLEELLGEIRLPEAQLYELLETAGPDRFVLLETGGQAGIT
RSVVATTRARVCRRKYCQRPCDSLHLCKLNLLGRCHYAQSQRNLCKYSHDVLSEQNFQILKNHEL
SGLNQEELACLLVQSDPFFLPEICKSYKGEGRKQTCGQPQPCERLHICEHFTRGNCSYLNCLRSH
NLMDRKVLTIMREHGLSPDVVQNIQDICNNKHAR
>3U88C
SMDSRLQRIHAEIKNSLKIDNLDVNRCIEALDELASLQVTMQQAQKHTEMITTLKKIRRFKVSQV
IMEKSTMLYNKFKNMFLVGEGDSV
>3U6WA
GAMTTSESPDAYTESFGAHTIVKPAGPPRVGQPSWNPQRASSMPVNRYRPFAEEVEPIRLRNRTW
PDRVIDRAPLWCAVDLRDGNQALIDPMSPARKRRMFDLLVRMGYKEIEVGFPSASQTDFDFVREI
IEQGAIPDDVTIQVLTQCRPELIERTFQACSGAPRAIVHFYNSTSILQRRVVFRANRAEVQAIAT
DGARKCVEQAAKYPGTQWRFEYSPESYTGTELEYAKQVCDAVGEVIAPTPERPIIFNLPATVEMT
TPNVYADSIEWMSRNLANRESVILSLHPHNDRGTAVAAAELGFAAGADRIEGCLFGNGERTGNVC
LVTLGLNLFSRGVDPQIDFSNIDEIRRTVEYCNQLPVHERHPYGGDLVYTAFSGSHQDAINKGLD
AMKLDADAADCDVDDMLWQVPYLPIDPRDVGRTYEAV
>3U43A
MELKHSISDYTEAEFLEFVKKICRAEGATEEDDNKLVREFERLTEHPDGSDLIYYPRDDREDSPE
GIVKEIKEWRAANGKSGFKQGLEHHHHHH
>3VIQB
MEKSQLESRVHLLEQQKEQLESSLQDALAKLKNRDAKQTVQKHIDLLHTYNEIRDIALGMIGKVA
EHEKCTSVELFDRFGVNGSE
>3U3EA
MVLSEGEWQLVLHVWAKVEADVAGHGQDILIRLFKSHPETLEKFDRFKHLKTEAEMKASEDLKKH
GVTVLTALGAILKKKGHHEAELKPLAQSHATKHKIPIKYLEFISEAIIHVLHSRHPGDFGADAQG
AMNKALELFRKDIAAKYKELGYQG
>3U2GA
MGTYEIRGQVASGFGDQSWDASSFAGFYYDIDDNVSTETLTVSDLDGNVIPEGGLVYTTTIADVD

FEYYNPDAGWDQYPVMGFFAEEYIPINPDKADKIAKLVLDSDDKYTIRTGEMLDLGEGYAIEAKQ
VDVDGEKVWLEFTKDGEFVDDEIISVSTADDEANTWDVELDDIEDEDDVVVLKVHVNQVFQGAVD
SIAQIEGLWLIDYANAMTIESDDEFGNLDDVSIDGDTLKISNEDTFTLTRDSEEEIGEGMYFMIA
DTSSSDLRYYPYVEKTIGLEHHHHHH
>3U22A
GACDGILEGIYDSPAASDSNELGFIRTDPSTHSGTIYIDATDYRRWTFIDFHTQKVDSVNVTDSE
QKEPEEWDIAVHRYDVKTNAGAVLETGFTGFSALRNADAMPEGAYVEDVWTTAKIAIDMSGMMDG
NIVYMESYYNEELSKWLNVDKSNMPPTYTLSNKVYMVKLKDGTYAAVRLTNYMNASGVKGFMTID
YIYPFEL
>3U0CA
GSQAANDAANKLFSLTIADLTANQNINTTNAHSTSNILIPELKAPKSLNASSQLTLLIGNLIQIL
GEKSLTALTNKITAWKSQQQARQQKNLEFSDKINTLLSETEGLTRDYEKQINKLKNADSKIKDLE
NKINQIQTRLSELDPESPEKKKLSREEIQLTIKKDAAVKDRTLIEQKTLSIHSKLTDKSMQLEKE
IDSFSA
>3U0OA
MSENSIRLTQYSHGAGCGCKISPKVLETILHSEQAKFVDPNLLVGNETRDDAAVYDLGNGTSVIS
TTDFFMPIVDNPFDFGRIAATNAISDIFAMGGKPIMAIAILGWPINKLSPEIAREVTEGGRYACR
QAGIALAGGHSIDAPEPIFGLAVTGIVPTERVKKNSTAQAGCKLFLTKPLGIGVLTTAEKKSLLK
PEHQGLATEVMCRMNIAGASFANIEGVKAMTDVTGFGLLGHLSEMCQGAGVQARVDYEAIPKLPG
VEEYIKLGAVPGGTERNFASYGHLMGEMPREVRDLLCDPQTSGGLLLAVMPEAENEVKATAAEFG
IELTAIGELVPARGGRAMVEIR
>4A2NB
MNENLWKICFIVMFIIWVFVRKVYGTRAMKNKSKKKVRPNFEKSLVFLNFIGMVFLPLTAVFSSY
LDSFNINLPDSIRLFALIVTFLNIGLFTKIHKDLGNNWSAILEIKDGHKLVKEGIYKNIRHPMYA
HLWLWVITQGIILSNWVVLIFGIVAWAILYFIRVPKEEELLIEEFGDEYIEYMGKTGRLFPKVV
>3VIAA
GSMAFVKSGWLLRQSTILKRWKKNWFDLWSDGHLIYYDDQTRQNIEDKVHMPMDCINIRTGQECR
DTQPPDGKSKDCMLQIVCRDGKTISLCAESTDDCLAWKFTLQDSRTN
>4A2AA
MIDLSKTVFYTSIDIGSRYIKGLVLGKRDQEWEALAFSSVKSRGLDEGEIKDAIAFKESVNTLLK
ELEEQLQKSLRSDFVISFSSVSFEREDTVIERDFGEEKRSITLDILSEMQSEALEKLKENGKTPL
HIFSKRYLLDDERIVFNPLDMKASKIAIEYTSIVVPLKVYEMFYNFLQDTVKSPFQLKSSLVSTA
EGVLTTPEKDRGVVVVNLGYNFTGLIAYKNGVPIKISYVPVGMKHVIKDVSAVLDTSFEESERLI
ITHGNAVYNDLKEEEIQYRGLDGNTIKTTTAKKLSVIIHARLREIMSKSKKFFREVEAKIVEEGE
IGIPGGVVLTGGGAKIPRINELATEVFKSPVRTGCYANSDRPSIINADEVANDPSFAAAFGNVFA
VSENPYEETPVKSENPLKKIFRLFKELME
>4A25A
TTIHDVQTTGLTQDAVTGFDASSRLNAGLQEVLVDLTALHLQGKQAHWNIVGENWRDLHLQLDTL
VEAARGFSDDVAERMRAVGGVPDARPQTVAASRIGDVGPDEIDTRACVEAIVALVRHTVDTIRRV
HDPIDAEDPASADLLHAITLELEKQAWMIGSENRSPRRR
>3TWLA
MPELPEVEAARRAIEENCLGKKIKRVIIADDNKVIHGISPSDFQTSILGKTIISARRKGKNLWLE
LDSPPFPSFQFGMAGAIYIKGVAVTKYKRSAVKDSEEWPSKYSKFFVELDDGLELSFTDKRRFAK
VRLLANPTSVSPISELGPDALLEPMTVDEFAESLAKKKITIKPLLLDQGYISGIGNWIADEVLYQ

ARIHPLQTASSLSKEQCEALHTSIKEVIEKAVEVDADSSQFPSNWIFHNREKKPGKAFVDGKKID
FITAGGRTTAYVPELQKLYGKDAEKAAKVRPAKRGVKPKEDDGDHHHHHH
>3TV0A
GSSSMAEKTQKSVKIAPGAVVCVESEIRGDVTIGPRTVIHPKARIIAEAGPIVIGEGNLIEEQAL
IINAYPDNITPDTEDPEPKPMIIGTNNVFEVGCYSQAMKMGDNNVIESKAYVGRNVILTSGCIIG
ACCNLNTFEVIPENTVIYGADCLRRVQTERPQPQTLQLDFLMKILPNYHHLKKTMKGSSTPVKN
>3TURA
RRYTLNATALGLGGAATRQLTFQTSSPAHLTMPYVMPGDGEVVGVGEPVAIRFDENIADRGAAEK
AIKITTNPPVEGAFYWLNNREVRWRPEHFWKPGTAVDVAVNTYGVDLGEGMFGEDNVQTHFTIGD
EVIATADDNTKILTVRVNGEVVKSMPTSMGKDSTPTANGIYIVGSRYKHIIMDSSTYGVPVNSPN
GYRTDVDWATQISYSGVFVHSAPWSVGAQGHTNTSHGCLNVSPSNAQWFYDHVKRGDIVEVVNTV
GGTLPGIDGLGDWNIPWDQWRAGNAKA
>3TUNA
GPGSMKVEKVFFVTSPIYYVNAAPHIGHVYSTLITDVIGRYHRVKGERVFALTGTDEHGQKVAEA
AKQKQVSPYDFTTAVAGEFKKCFEQMDYSIDYFIRTTNEQHKAVVKELWTKLEQKGDIYLGRYEG
WYSISDESFLTPQNITDGVDKDGNPCKVSLESGHVVTWVSEENYMFRLSAFRERLLEWYHANPGC
IVPEFRRREVIRAVEKGLPDLSVSRARATLHNWAIPVPGNPDHCVYVWLDALTNYLTGSRLRVDE
SGKEVSLVDDFNELERFPADVHVIGKDILKFHAIYWPAFLLSAGLPLPKKIVAHGWWTKDRKKIS
KSLGNVFDPVEKAEEFGYDALKYFLLRESGFSDDGDYSDKNMIARLNGELADTLGNLVMRCTSAK
INVNGEWPSPAAYTEEDESLIQLIKDLPGTADHYYLIPDIQKAIIAVFDVLRAINAYVTDMAPWK
LVKTDPERLRTVLYITLEGVRVTTLLLSPILPRKSVVIFDMLGVPEVHRKGIENFEFGAVPPGTR
LGPAVEGEVLFSKRSTENTKST
>3TU5A
MCDEDETTALVCDNGSGLVKAGFAGDDAPRAVFPSIVGRPRHQGVMVGMGQKDSYVGDEAQSKRG
ILTLKYPIEHGIITNWDDMEKIWHHTFYNELRVAPEEHPTLLTEAPLNPKANREKMTQIMFETFN
VPAMYVAIQAVLSLYASGRTTGIVLDSGDGVTHNVPIYEGYALPHAIMRLDLAGRDLTDYLMKIL
TERGYSFVTTAEREIVRDIKEKLCYVALDFENEMATAASSSSLEKSYELPDGQVITIGNERFRCP
ETLFQPSFIGMESAGIHETTYNSIMKCDIDIRKDLYANNVMSGGTTMYPGIADRMQKEITALAPS
TMKIKIIAPPERKYSVWIGGSILASLSTFQQMWITKQEYDEAGPSIVHRKCF
>3TU3A
MHHHHHHSSGVDLGTENLYFQSNAMIDTWLAQWGLRLPSSNDATLRLQPAEGPELVMERLEGGWL
FVVELGLVPSGLPLGVILQLLQVNSPFSSLAPVKLAADDAGRLVLWAEARDGVDDVDALNRLHDR
LREGHSRLVPLLEPTGELVPAQIQTSALVFV
>3TT1A
MEVKREHWATRLGLILAMAGNAVGLGNFLRFPVQAAENGGGAFMIPYIIAFLLVGIPLMWIEWAM
GRYGGAQGHGTTPAIFYLLWRNRFAKILGVFGLWIPLVVAIYFVYIESWTLGFAIKFLVGLVPEP
PPNATDPDSILRPFKEFLYSYIGVPKGDEPILKPSLFAYIVFLITMFINVSILIRGISKGIERFA
KIAMPTLFILAVFLVIRVFLLETPNGTAADGLNFLWTPDFEKLKDPGVWIAAVGQIFFTLSLGFG
AIITYASYVRKDQDIVLSGLTAATLNEAAEVILGGSISIPAAVAFFGVANAVAIAKAGAFNLGFI
TLPAIFSQTAGGTFLGFLWFFLLFFAGLTSSIAIMQPMIAFLEDELKLSRKHAVLWTAAIVFFSA
HLVMFLNKSLDEMDFWAGTIGVVFFGLTELIIFFWIFGADKAWEEINRGGIIKVPRIYYYVMRYI
TPAFLAVLLVVWAREYIPKIMEETHWTVWITRFYIIGLFLFLTFLVFLAERRRNHESAGTLVPR
>4A0XA
MRGETLKLKKDKRREAIRQQIDSNPFITDHELSDLFQVSIQTIRLDRTYLNIPELRKRIKLVAEK

NYDQISSIEEQEFIGDLIQVNPNVKAQSILDITSDSVFHKTGIARGHVLFAQANSLCVALIKQPT
VLTHESSIQFIEKVKLNDTVRAEARVVNQTAKHYYVEVKSYVKHTLVFKGNFKMFYDKRG

>3TS9A

GHMDTRENPFKEKLLEIMASIQTYCQKSPMSDFGTQHYEQWAIQMEKKAAKDGNRKDRVCAEHLR
KYNEALQINDTIRMIDAYSHLETFYTDEKEKKFAVLNDSKKSLKLDETDEFLMNLFFDNKKMLKK
LAENPKYE

>3VHXB

GSLLFQPDQNAPPIRLRHRRSRSAGDRWVDHKPASNMQTETVMQPHVPHAITVSVANEKALAKCE
KYMLTHQELASDGEIETKLIKGDIYKTRGGGQSVQFTDIETLKQESPNGSRKRRS

>3TRTA

GGSKPDCTAAMRDVRQQYESVAAKNLQEAEEWYKSKFADLSEAANRNNDALRQAKQESTEYRRQV
QSLTMEVDALKG

>4A0EA

GGGSWVCRFYQGKHRGVEVELPHGRCVFGSDPLQSDIVLSDSEIAPVHLVLMVDEEGIRLTDSAE
PLLQEGLPVPLGTLLRAGSCLEVGFLLWTFVAVGQPLPETLQVPTQRKEPTDRLPRSR

>4A03A

TMAHHHHHHVTNSTDGRADGRLRVVVLGSTGSIGTQALQVIADNPDRFEVVGLAAGGAHLDTLLR
QRAQTGVTNIAVADEHAAQRVGDIPYHGSDAATRLVEQTEADVVLNALVGALGLRPTLAALKTGA
RLALANKESLVAGGSLVLRAARPGQIVPVDSEHSALAQCLRGGTPDEVAKLVLTASGGPFRGWSA
ADLEHVTPEQAGAHPTWSMGPMNTLNSASLVNKGLEVIETHLLFGIPYDRIDVVVHPQSIIHSMV
TFIDGSTIAQASPPDMKLPISLALGWPRRVSGAAAACDFHTASSWEFEPLDTDVFPAVELARQAG
VAGGCMTAVYNAANEEAAAAFLAGRIGFPAIVGIIADVLHAADQWAVEPATVDDVLDAQRWARER
AQRAVSGM

>3TO8A

MGHHHHHHDSPVLMVYGLDQSKMNCDRVFNVFCLYGNVEKVKFMKSKPGAAMVEMADGYAVDRAI
THLNNNFMFGQKLNVCVSKQPAIMPGQSYGLEDGSCSYKDFSESRNNRFSTPEQAAKNRIQHPSN
VLHFFNAPLEVTEENFFEICDELGVKRPSSVKVFSGKSERSSSGLLEWESKSDALETLGFLNHYQ
MKNPNGPYPYTLKLCFSTAQHAS

>3TNXA

MHHHHHHSSGLVPRGSGMKETAAAKFERQHMDSPDLGTDDDDKMDFSIVGYSQNDLTSTERLIQL
FESWMLKHNKIYKNIDEKIYRFEIFKDNLKYIDETNKKNNSYWLGLNVFADMSNDEFKEKYTGSI
AGNYTTTELSYEEVLNDGDVNIPEYVDWRQKGAVTPVKNQGSCGSAWAFSAVSTIESIIKIRTGN
LNEYSEQELLDCDRRSYGCNGGYPWSALQLVAQYGIHYRNTYPYEGVQRYCRSREKGPYAAKTDG
VRQVQPYNEGALLYSIANQPVSVVLEAAGKDFQLYRGGIFVGPCGNKVDHAVAAVGYGPNYILIR
NSWGTGWGENGYIRIKRGTGNSYGVCGLYTSSFYPVKN

>3ZZOA

ECCTSRELVEFKMDRGDCEAVRAIENYPNGCEVTICADGVAQLGAYCGQGPCNIFGCNCDGGCLS
GDWSQEFVRRNQQYGIQIIKVTRLPFWRPL

>3TMUA

MRSLLILVLCFLPLAALGKVFGRCELAAAMKRHGLDNYRGYSLGNWVCAAKFESNFNTQATNRNT
DGSTDYGILQINSRWWCNDGRTPGSRNLCNIPCSALLSSDITASVNCAKKIVSDGNGMNAWVAWR
NRCKGTDVQAWIRGCRL

>3TKLB

GPLGSTSSTSQADKEIQKMLDEYEQAIKRAQENIKKGEELEKKLDKLERQGKDLEDKYKTYEENL

EGFEKLLTDSEELSLSEINEKMKAFSKDSEKLTQLMEKHKGDEKTVQSLQREHHDIKAKLANLQV
LHDAHTGKKSYVNEKGNPVSSLKDAHLAINKDQEVVEHKGQFYLLQKGQWDAIKNDPAALEKAQK
DYSQSKHDLATIKMEALIHKLSLEMEKQLETINDLIMSTDPKENEEATKLLHKHNGLNLKLANLQ
DMLAVHR
>3TIKA
KGKLPPVYPVTVPILGHIIQFGKSPLGFMQECKRQLKSGIFTINIVGKRVTIVGDPHEHSRFFLP
RNEVLSPREVYSFMVPVFGEGVAYAAPYPRMREQLNFLAEELTIAKFQNFVPAIQHEVRKFMAAN
WDKDEGEINLLEDCSTMIINTACQCLFGEDLRKRLDARRFAQLLAKMESSLIPAAVFLPILLKLP
LPQSARCHEARTELQKILSEIIIARKEEEVNKDSSTSDLLSGLLSAVYRDGTPMSLHEVCGMIVA
AMFAGQHTSSITTTWSMLHLMHPANVKHLEALRKEIEEFPAQLNYNNVMDEMPFAERCARESIRR
DPPLLMLMRKVMADVKVGSYVVPKGDIIACSPLLSHHDEEAFPEPRRWDPERDEKVEGAFIGFGA
GVHKCIGQKFGLLQVKTILATAFRSYDFQLLRDEVPDPDYHTMVVGPTASQCRVKYIRRKAAAA
>3TGUB
SLKVAPKVAVSAAAERVKLCPGAEDLEITKLPNGLIIASLENFSPASRIGVFIKAGSRYETTANL
GTAHLLRLASPLTTKGASSFRITRGIEAVGGSLSVYSTREKMTYCVECLRDHVDTVMEYLLNVTT
APEFRPWEVTDLQPQLKVDKAVAFQSPQVGVLENLHAAAYKTALANPLYCPDYRIGKITSEQLHH
FVQNNFTSARMALVGIGVKHSDLKQVAEQFLNIRSGAGTSSAKATYWGGEIREQNGHSLVHAAVV
TEGAAVGSAEANAFSVLQHVLGAGPLIKRGSSVTSKLYQGVAKATTQPFDASAFNVNYSDSGLFG
FYTISQAAHAGEVIRAAMNQLKAAAQGGVTEEDVTKAKNQLKATYLMSVETAQGLLNEIGSEALL
SGTHTAPSVVAQKIDSVTSADVVNAAKKFVSGKKSMAASGDLGSTPFLDEL
>3ZXUB
MDFTSSSGVLDSERNTGSNDSDEPSSHSDVIETEELKLIKLQEHKNNLLRQRSELLDQLSQTRVV
EPRSVQLDDKLLLKLLRRNDNAVSDSSQSSNNPLPRVLPSLNIEQRKKYLDITLNDVTVTCEKDM
ILLRKGSFTASFRIAVENESIRSMAIDLNAFEVELQPIIQYAEDTQNVNVAMMAVVQFLRIKELH
EQMISKIVEASKFIRASNNTITLNDLEVSFHCYWNLPSPYPETLILTNKVQKILDFLIYQYGIQL
GVIKYGSTII
>2LHRA
SDDYVDEETYNLQKLLAPYHKAKTLERQVYELEKLQEKLPEKYKAEYKKKLDQTRVELADQVKSA
VTEFENVTPTNDQ
>3TEEA
QDINAQLTTWFSQRLAGFSDEVVVTLRSSPNLLPSCEQPAFSMTGSAKLWGNVNVVARCANEKRY
LQVNVQATGNYVAVAAPIARGGKLTPANVTLKRGRLDQLPPRTVLDIRQIQDAVSLRDLAPGQPV
QLTMIRQAWRVKAGQRVQVIANGEGFSVNAEGQAMNNAAVAQNARVRMTSGQIVSGTVDSDGNIL
INLDPNSSSVDKLAAALEHHHHHH
>3TDOA
MGRAHKETLDKLTNAAINKINLLNTSKVKYLVSSAFAGLYVGIGILLIFTIGGLLTDAGSPMTKI
VMGLSFAIALSLVIMTGTELFTGNNMVMSAGMLNKGVSIKDTSKIWAYSWVGNLIGALVLGIIFV
GTGLVDKGPVAEFFANTAASKASMPFTALFFRGILCNILVCVSVLCSFRTNSDTAKIIMIFLCLF
AFITSGFEHSVANMTIYSVSLFSPTISTVTIGGAIYNLVAVTLGNIVGGALFMGLGTYILGKEKL
NAAAENLY
>3TCJA
MSQFTLYKNKDKSSAKTYPYFVDVQSDLLDNLNTRLVIPLTPIELLDKKAPSHLCPTIHIDEGDF
IMLTQQMTSVPVKILSEPVNELSTFRNEIIAAIDFLITGI
>3ZXBA

FTCPECRPELCGDPGYCEYGTTKDACDCCPVCFQGPGGYCGGPEDVFGICADGFACVPLVGERDS
QDPEIVGTCVKIP
>3VGBA
TFAYKIDGNEVIFTLWAPYQKSVKLKVLEKGLYEMERDEKGYFTITLNNVKVRDRYKYVLDDASE
IPDPASRYQPEGVHGPSQIIQESKEFNNETFLKKEDLIIYEIHVGTFTPEGTFEGVIRKLDYLKD
LGITAIEIMPIAQFPGKRDWGYDGVYLYAVQNSYGGPEGFRKLVDEAHKKGLGVILDVVYNHVGP
EGNYMVKLGPYFSQKYKTPWGLTFNFDDAESDEVRKFILENVEYWIKEYNVDGFRLDAVHAIIDT
SPKHILEEIADVVHKYNRIVIAESDLNDPRVVNPKEKCGYNIDAQWVDDFHHSIHAYLTGERQGY
YTDFGNLDDIVKSYKDVFVYDGKYSNFRRKTHGEPVGELDGCNFVVYIQNHDQVGNRGKGERIIK
LVDRESYKIAAALYLLSPYIPMIFMGEEYGEENPFYFFSDFSDSKLIQGVREGRKKENGQDTDPQ
DESTFNASKLSWKIDEEIFSFYKILIKMRKELSIACDRRVNVVNGENWLIIKGREYFSLYVFSKS
SIEVKYSGTLLLSSNNSFPQHIEEGKYEFDKGFALYKL
>3ZX8A
MENDPRVRKFASDGAQWAIKWQKKGWSTLTSRQKQTARAAMGIKLSPVAQPVQKVTRLSAPVALA
YREVSTQPRVSTARDGITRSGSELITTLKKNTDTEPKYTTAVLNPSEPGTFNQLIKEAAQYEKYR
FTSLRFRYSPMSPSTTGGKVALAFDRDAAKPPPNDLASLYNIEGCVSSVPWTGFILTVPTDSTDR
FVADGISDPKLVDFGKLIMATYGQGAAQLGEVRVEYTVQLKNRTGSTSAQIGDFAGVKDGPRLVS
WSKTKGTAGWEHDCHFLGTGNFSLTLFYEKAPVSGLENADASDFSVLGEAAAGSVQWAGVKVAER
GQGVKMVTTEEQPKGKWQALRI
>3TAYA
GSLLDGPYQPTTFNPPTSYWILLAPTVEGVVIQGTNNIDRWLATILIEPNVQTTNRIYNLFGQQV
TLSVENTSQTQWKFIDVSKTTPTGNYTQHGSLFSTPKLYAVMKFSGRIYTYNGTTPNATTGYYST
TNYDTVNMTSFCDFYIIPRNQEEKCTEYINHGL
>3T60A
MHLKIVCLSDEVREMYKNHKTHHEGDSGLDLFIVKDEVLKPKSTTFVKLGIKAIALQYKSNYYYK
CEKSENKKKDDDKSNIVNTSFLLFPRSSISKTPLRLANSIGLIDAGYRGEIIAALDNTSDQEYHI
KKNDKLVQLVSFTGEPLSFELVEELDETSRGEGGFGSTSNNKYLEHHHHHH
>3T5VA
GSPLPSDVRPPHILVKTLDYIVDNLLTTLPESEGFLWDRMRSIRQDFTYQNYSGPEAVDCNERIV
RIHLLILHIMVKSNVEFSLQQELEQLHKSLITLSEIYDDVRSSGGTCPNEAEFRAYALLSKIRDP
QYDENIQRLPKHIFQDKLVQMALCFRRVISNSAYTERGFVKTENCLNFYARFFQLMQSPSLPLLM
GFFLQMHLTDIRFYALRALSHTLNKKHKPIPFIYLENMLLFNNRQEIIEFCNYYSIEIINGDAAD
LKTLQHYSHKLSETQPLKKTYLTCLERRLQKTTYKGLINGGEDNLASSVYVKDPKK
>3T63M
PAQDNSRFVIRDRNWHPKALTPDYKTSIARSPRQALVSIPQSISETTGPNFSHLGFGAHDHDLLL
NFNNGGLPIGERIIVAGRVVDQYGKPVPNTLVEMWQANAGGRYRHKNDRYLAPLDPNFGGVGRCL
TDSDGYYSFRTIKPGPAPWRNGPNDWRPAHIYFGISGPSIATKLITQLYFEGDPLIPMCPIVKSI
ANPEAVQQLIAKLDMNNANPMDCLAYRFDIVLRGQRKTHFENC
>3T6BA
MADTQYILPNDIGVSSLDCREAFRLLSPTERLYAYHLSRAAWYGGLAVLLQTSPEAPYIYALLSR
LFRAQDPDQLRQHALAEGLTEEEYQAFLVYAAGVYSNMGNYKSFGDTKFVPNLPKEKLERVILGS
EAAQQHPEEVRGLWQTCGELMFSLEPRLRHLGLGKEGITTYFSGNCTMEDAKLAQDFLDSQNLSA
YNTRLFKEVDGEGKPYYEVRLASVLGSEPSLDSEVTSKLKSYEFRGSPFQVTRGDYAPILQKVVE
QLEKAKAYAANSHQGQMLAQYIESFTQGSIEAHKRGSRFWIQDKGPIVESYIGFIESYRDPFGSR

GEFEGFVAVVNKAMSAKFERLVASAEQLLKELPWPPTFEKDKFLTPDFTSLDVLTFAGSGIPAGI
NIPNYDDLRQTEGFKNVSLGNVLAVAYATQREKLTFLEEDDKDLYILWKGPSFDVQVGLHALLGH
GSGKLFVQDEKGAFNFDQETVINPETGEQIQSWYRSGETWDSKFSTIASSYEECRAESVGLYLCL
HPQVLEIFGFEGADAEDVIYVNWLNMVRAGLLALEFYTPEAFNWRQAHMQARFVILRVLLEAGEG
LVTITPTTGSDGRPDARVRLDRSKIRSVGKPALERFLRRLQVLKSTGDVAGGRALYEGYATVTDA
PPECFLTLRDTVLLRKESRKLIVQPNTRLEGSDVQLLEYEASAAGLIRSFSERFPEDGPELEEIL
TQLATADARFW
>3T5AA
MGSSHHHHHHSSGLVPRGSHMSVRSLPAALRACARLQPHDPAFTFMDYEQDWDGVAITLTWSQLY
RRTLNVAQELSRCGSTGDRVVISAPQGLEYVVAFLGALQAGRIAVPLSVPQGGVTDERSDSVLSD
SSPVAILTTSSAVDDVVQHVARRPGESPPSIIEVDLLDLDAPNGYTFKEDEYPSTAYLQYTSGST
RTPAGVVMSHQNVRVNFEQLMSGYFADTDGIPPPNSALVSWLPFYHDMGLVIGICAPILGGYPAV
LTSPVSFLQRPARWMHLMASDFHAFSAAPNFAFELAARRTTDDDMAGRDLGNILTILSGSERVQA
ATIKRFADRFARFNLQERVIRPSYWLAEATVYVATSKPGQPPETVDFDTESLSAGHAKPCAGGGA
TSLISYMLPRSPIVRIVDSDTCIECPDGTVGEIWVHGDNVANGYWQKPDESERTFGGKIVTPSPG
TPEGPWLRTGDSGFVTDGKMFIIGR
>2LGDA
GSMALPIIVKWGGQEYSVTTLSEDDTVLDLKQFLKTLTGVLPERQKLLGLKVKGKPAENDVKLGA
LKLKPNTKIMMMGTREES
>3T1HR
MSTKNAKPKKEAQRRPSRKAKVKATLGEFDLRDYRNVEVLKRFLSETGKILPRRRTGLSGKEQRI
LAKTIKRARILGLLPFTEKLVRK
>3T1HQ
MPKKVLTGVVVSDKMQKTVTVLVERQFPHPLYGKVIKRSKKYLAHDPEEKYKLGDVVEIIESRPI
SKRKRFRVLRLVESGRMDLVEKYLIRRQNYQSLSKRGGKA
>3ZUIA
DSESDCTGSEPVDAFQAFSEGKEAYVLVRSTDPKARDCLKGEPAGEKQDNTLPVMMTFKNGTDWA
STDWTFTLDGAKVTATLGNLTQNREVVYDSQSHHCHVDKVEKEVPDYEMWMLDAGGLEVEVECCR
QKLEELASGRNQMYPHLKDC
>3SX6A
MRGSAHVVILGAGTGGMPAAYEMKEALGSGHEVTLISANDYFQFVPSNPWVGVGWKERDDIAFPI
RHYVERKGIHFIAQSAEQIDAEAQNITLADGNTVHYDYLMIATGPKLAFENVPGSDPHEGPVQSI
CTVDHAERAFAEYQALLREPGPIVIGAMAGASCFGPAYEYAMIVASDLKKRGMRDKIPSFTFITS
EPYIGHLGIQGVGDSKGILTKGLKEEGIEAYTNCKVTKVEDNKMYVTQVDEKGETIKEMVLPVKF
GMMIPAFKGVPAVAGVEGLCNPGGFVLVDEHQRSKKYANIFAAGIAIAIPPVETTPVPTGAPKTG
YMIESMVSAAVHNIKADLEGRKGEQTMGTWNAVAFADMGDRGAAFIALPQLKPRKVDVFAYGRWV
HLAKVAFEKYFIRKMKMGVSEPFYEKVLFKMMGITRLKEEDTHRKAS
>3SWMA
HHHHHHMGIQETDPLTQLSLPPGFRFYPTDEELMVQYLCRKAAGYDFSLQLIAEIDLYKFDPWVL
PNKALFGEKEWYFFSPRDRKYPNGSRPNRVAGSGYWKATGTDKIISTEGQRVGIKKALVFYIGKA
PKGTKTNWIMHEYRLIEPSRRNGSTKLDDWVLCRIYKKQSSAQK
>3STTA
GSMEKSMSPFVKKHFVLVHTAFHGAWCWYKIVALMRSSGHNVTALDLGASGINPKQALQIPNFSD
YLSPLMEFMASLPANEKIILVGHALGGLAISKAMETFPEKISVAVFLSGLMPGPNIDATTVCTKA

GSAVLGQLDNCVTYENGPTNPPTTLIAGPKFLATNVYHLSPIEDLALATALVRPLYLYLAEDISK
EVVLSSKRYGSVKRVFIVATENDALKKEFLKLMIEKNPPDEVKEIEGSDHVTMMSKPQQLFTTLL
SIANKYK
>3SS3A
GSHMVAAGDNKIKQGLLPSLEDLLFYTIAEGQEKIPVHKFITALKSTGLRTSDPRLKECMDMLRL
TLQTTSDGVMLDKDLFKKCVQSNIVLLTQAFRRKFVIPDFMSFTSHIDELYESAKKQSGGKVADY
IPQLAKFSPDLWGVSVCTVDGQRHSIGDTKVPFCLQSCVKPLKYAIAVNDLGTEYVHRYVGKEPS
GLRFNKLFLNEDDKPHNPMVNAGAIVVTSLIKQGVNNAEKFDYVMQFLNKMAGNEYVGFSNATFQ
SERESGDRNFAIGYYLKEKKCFPEGTDMVGILDFYFQLCSIEVTCESASVMAATLANGGFCPITG
ERVLSPEAVRNTLSLMHSCGMYDFSGQFAFHVGLPAKSGVAGGILLVVPNVMGMMCWSPPLDKMG
NSVKGIHFCHDLVSLCNFHNYDNLRHFAKKLDPRREGGDQRHSFGPLDYESLQQELALKDTVWKK
VSPESSDDTSTTVVYRMESLGERS
>3SQRA
MKYFTVFTALTALFAQASASAIPAVRSTLTPRQNTTASCANSATSRSCWGEYSIDTNWYDVTPTG
VTREYWLSVENSTITPDGYTRSAMTFNGTVPGPAIIADWGDNLIIHVTNNLEHNGTSIHWHGIRQ
LGSLEYDGVPGVTQCPIAPGDTLTYKFQVTQYGTTWYHSHFSLQYGDGLFGPLIINGPATADYDE
DVGVIFLQDWAHESVFEIWDTARLGAPPALENTLMNGTNTFDCSASTDPNCVGGGKKFELTFVEG
TKYRLRLINVGIDSHFEFAIDNHTLTVIANDLVPIVPYTTDTLLIGIGQRYDVIVEANAAADNYW
IRGNWGTTCSTNNEAANATGILRYDSSSIANPTSVGTTPRGTCEDEPVASLVPHLALDVGGYSLV
DEQVSSAFTNYFTWTINSSSLLLDWSSPTTLKIFNNETIFPTEYNVVALEQTNANEEWVVYVIED
LTGFGIWHPIHLHGHDFFIVAQETDVFNSDESPAKFNLVNPPRRDVAALPGNGYLAIAFKLDNPG
SWLLHCHIAWHASEGLAMQFVESQSSIAVKMTDTAIFEDTCANWNAYTPTQLFAEDDSGI
>3SO6A
MEGMVFSLKYLGMTLVERPKGEELSAAAVKRIVATAKASGKKLQKVTLKVSPRGIILTDSLTSQL
IENVSIYRISYCTADKMHDKVFAYIAQSQQNESLECHAFLCTKRKVAQAVTLTVAQAFKVAFEFW
QVSLVPR
>3SMHA
SRNNPFYFPSRRFSTRYGNQNGRIRVLQRFDQRSRQFQNLQNHRIVQIEAKPNTLVLPKHADADN
ILVIQQGQATVTVANGNNRKSFNLDEGHALRIPSGFISYILNRHDNQNLRVAKISMPVNTPGQFE
DFFPASSRDQSSYLQGFSRNTLEAAFNAEFNEIRRVLLEENAGGEQEERGQRRWSTRSSENNEGV
IVKVSKEHVEELTKHAKSVSKKGSEEEGDITNPINLREGEPDLSNNFGKLFEVKPDKKNPQLQDL
DMMLTCVEIKEGALVLPHFNSKAMVIVVVNKGTGNLELVAVRKEQQQRGRREEEEDEDEEEEGSN
REVRRYTARLKEGDVFIMPAAHPVAINASSELHLLGFGINAENNHRIFLAGDKDNVIDQIEKQAK
DLAFPGSGEQVEKLIKNQKESHFVSARP
>3B18A
MSHTDLTPCTRVLASSGTVPIAEELLARVLEPYSCKGCRYLIDAQYSATEDSVLAYGNFTIGESA
YIRSTGHFNAVELILCFNQLAYSAFAPAVLNEEIRVLRGWSIDDYCQHQLSSMLIRKASSRFRKP
LNPQKFSARLLCRDLQVIERTWRYLKVPCVIEFWDENGGAASGEIELAALNIP
>3ZSCA
SLNDKPVGFASVPTADLPEGTVGGLGGEIVFVRTAEELEKYTTAEGKYVIVVDGTIVFEPKREIK
VLSDKTIVGINDAKIVGGGLVIKDAQNVIIRNIHFEGFYMEDDPRGKKYDFDYINVENSHHIWID
HITFVNGNDGAVDIKKYSNYITVSWNKFVDHDKVSLVGSSDKEDPEQAGQAYKVTYHHNYFKNLI
QRMPRIRFGMAHVFNNFYSMGLRTGVSGNVFPIYGVASAMGAKVHVEGNYFMGYGAVMAEAGIAF
LPTRIMGPVEGYLTLGEGDAKNEFYYCKEPEVRPVEEGKPALDPREYYDYTLDPVQDVPKIVVDG

AGAGKLVFEELNTAQ
>2LENA
MQLKPMEINPEMLNKVLYRLGVAGQWRFVDVLGLEEESLGSVPAPACALLLLFPLTAQHENFRKK
QIEELKGQEVSPKVYFMKQTIGNSCGTIGLIHAVANNQDKLGFEDGSVLKQFLSETEKMSPEDRA
KCFEKNEAIQAAHDAVAQEGQCRVDDKVNFHFILFNNVDGHLYELDGRMPFPVNHGASSEDTLLK
DAAKVCREFTEREQGEVRFSAVALCKAALEHHHHHH
>2LELA
VDMSNVVKTYDLQDGSKVHVFKDGKMGMENKFGKSMNMPEGKVMETRDGTKIIMKGNEIFRLDEA
LRKGHSEGG
>3SGFY
MAAKIRRDDEVIVLTGKDKGKRGKVKNVLSSGKVIVEGINLVKKHQKPVPALNQPGGIVEKEAAI
QVSNVAIFNAATGKADRVGFRFEDGKKVRFFKSNSETIK
>3ZQDA
GRKLLTYQVKQGDTLNSIAADFRISTAALLQANPSLQAGLTAGQSIVIPGLPDPYTIPYHIAVSI
GAKTLTLSLNNRVMKTYPIAVGKILTQTPTGEFYIINRQRNPGGPFGAYWLSLSKQHYGIHGTNN
PASIGKAVSKGCIRMHNKDVIELASIVPNGTRVTINRGSHHHHHH
>3SCIE
RVVPSGDVVRFPNITNLCPFGEVFNATKFPSVYAWERKKISNCVADYSVLYNSTFFSTFKCYGVS
ATKLNDLCFSNVYADSFVVKGDDVRQIAPGQTGVIADYNYKLPDDFMGCVLAWNTRNIDATSTGN
YNYKYRFLRHGKLRPFERDISNVPFSPDGKPCTPPAFNCYWPLNDYGFYTTTGIGYQPYRVVVLS
FELLNAPATVCGPKLSTDLIKNQCVNFHHHHHH
>2YMAA
GSIGSNSIDLITKYEPIFLGSGIYFLRPFNTDERDKLMVTDNAMSNWDEITETYYQKFGNAINKM
LSLRLVSLPNGHILQPGDSCVWLAEVVDMKDRFQTTLSLNILNSQRAEIFFNKTFTFNEDNGNFL
SYKIGDHGESTELGQITHSNKADINTAEIRS
>3SBSA
MNTVPFTSAPIEVTIGIDQYSFNVKENQPFHGIKDIPIGHVHVIHFQHADNSSMRYGYWFDCRMG
NFYIQYDPKDGLYKMMEERDGAKFENIVHNFKERQMMVSYPKIDEDDTWYNLTEFVQMDKIRKIV
RKDENQFSYVDSSMTTVQENELLKSSLQKAGSKMEAKNEDDPAHSLNYTVINFKSREAIRPGHEM
EDFLDKSYYLNTVMLQGIFKNSSNYFGELQFAFLNAMFFGNYGSSLQWHAMIELICSSATVPKHM
LDKLDEILYYQIKTLPEQYSDILLNERVWNICLYSSFQKNSLHNTEKIMENKYPELLGKDNEDDA
LIYGISDEERDDEDDEHNPTIVGGLYYQRPLEHHHHHH
>2YJGA
MANIEIPYGKSKLAFDLPDERIQGILRSKAGSYKVNMSEEDIVKRALENPIGTKRLQDLAEGKKN
IVIITSDHTRPVPSRITLPLLLDEIRKKNKSANVKILIATGFHRGTTLQEMKAKFGEDLVENEQF
VVHDSRNSENMELIGTLPSGGKLEINKLAVEADLLVAEGFIEPHFFAGFSGGRKSILPGIASVQC
ILANHCSEFIKNPYARTGVLENNPIHRDMIYAAKKANLAFILNVVIDSSHKIVNAFAGHSEKAHL
KGCEFVSEIATVNAKPADIVITSNGGYPLDQNIYQSVKGMTAGEAACKDGGVIIIAAECADGHGG
EGFYRWFKESKDPQDVMNKILSRGRDETLPDQWEAQILARILINHKVIMVTDSKNYEYVKDMFMT
PAKDLGEALKIAESIVNNDSKINVIPDGVSVIVREKASWSHPQFEK
>3S4EA
ASQVGVIKPWLLLGSQDAAHDLDTLKKNKVTHILNVAYGVENAFLSDFTYKSISILDLPETNILS
YFPECFEFIEEAKRKDGVVLVHSNAGVSRAAAIVIGFLMNSEQTSFTSAFSLVKNARPSICPNSG
FMEQLRTYQEGKES

>3S44A

MKTITLYLDPASLPALNQLMDFTQNNEDKTHPRIFGLSRFKIPDNIITQYQNIHFVELKDNRPTE
ALFTILDQYPGNIELNIHLNIAHSVQLIRPILAYRFKHLDRVSIQQLNLYDDGSDEYVDLEKEEN
KDISAEIKQAEKQLSHYLLTGKIKFDNPTIARYVWQSAFPVKYHFLSTDYFEKAEFLQPLKEYLA
ENYQKMDWTAYQQLTPEQQAFYLTLVGFNDEVKQSLEVQQAKFIFTGTTTWEGNTDVREYYAQQQ
LNLLNHFTQAEGDLFIGDHYKIYFKGHPRGGEINDYILNNAKNITNIPANISFEVLMMTGLLPDK
VGGVASSLYFSLPKEKISHIIFTSNKQVKSKEDALNNPYVKVMRRLGIIDESQVIFWDSLKQLGG
GLEHHHHHH

>3S2SA

MGSSHHHHHHSSGLVPRGSHMASMTGGQQMGRGSMSKALISIDYTYDFVADDGKLTAGKPAQAIS
KAIAQVTQKAYDNGDYIFFTIDGHDEGDDFHPETKLFPPHNIKGTSGRDLYGALADFYQKHENDK
RVFWMDKRHYSAFSGTDLDIRLRERRVDTVVLTGVLTDICVLHTAIDAYNLGYQIEVVQSAVASL
SQENHQFALNHLQNVLGATIIE

>3S2QA

GSHMRKQQRMVVVRAEGGGGINPEIRKNEDKVVDSVVVTELSKNITPYCRCWRSGTFPLCDGSHV
KHNKANGDNVGPLLLKKQ

>3S0PA

ATKKAVAVLKGNSNVEGVVTLSQDDDGPTTVNVRITGLAPGLHGFHLHEYGDTTNGCMSTGAHFN
PNKLTHGAPGDEIRHAGDLGNIVANADGVAEVTLVDNQIPLTGPNSVVGRALVVHELEDDLGKGG
HELSLTTGNAGGRLACGVVGLTPI

>3AYQA

FAGGTVSQRCLSCICKMESGCRNVGCKMDMGSLSCGYFQIKEAYWIDCGRPGSSWKSCAASSYCA
SLCVQNYMKRYAKWAGCPLRCEGFAREHNGGPRGCKKGSTIGYWNRLQKISGCHGVQ

>3RZIA

MNWTVDIPIDQLPSLPPLPTDLRTRLDAALAKPAAQQPTWPADQALAMRTVLESVPPVTVPSEIV
RLQEQLAQVAKGEAFLLQGGDCAETFMDNTEPHIRGNVRALLQMAVVLTYGASMPVVKVARIAGQ
YAKPRSADIDALGLRSYRGDMINGFAPDAAAREHDPSRLVRAYANASAAMNLVRALTSSGLASLH
LVHDWNREFVRTSPAGARYEALATEIDRGLRFMSACGVADRNLQTAEIYASHEALVLDYERAMLR
LSDGDDGEPQLFDLSAHTVWIGERTRQIDGAHIAFAQVIANPVGVKLGPNMTPELAVEYVERLDP
HNKPGRLTLVSRMGNHKVRDLLPPIVEKVQATGHQVIWQCDPMHGNTHESSTGFKTRHFDRIVDE
VQGFFEVHRALGTHPGGIHVEITGENVTECLGGAQDISETDLAGRYETACDPRLNTQQSLELAFL
VAEMLRD

>3RQOA

RAPAPATPHAPDHSPAPNSPTLTRPPEGPKFPRVKNWELGSITYDTLCAQSQQDGPCTPRRCLGS
LVLPRKLQTRPSPGPPPAEQLLSQARDFINQYYSSIKRSGSQAHEERLQEVEAEVASTGTYHLRE
SELVFGAKQAWRNAPRCVGRIQWGKLQVFDARDCSSAQEMFTYICNHIKYATNRGNLRSAITVFP
QRAPGRGDFRIWNSQLVRYAGYRQQDGSVRGDPANVEITELCIQHGWTPGNGRFDVLPLLLQAPD
EAPELFVLPPELVLEVPLEHPTLEWFAALGLRWYALPAVSNMLLEIGGLEFSAAPFSGWYMSTEI
GTRNLCDPHRYNILEDVAVCMDLDTRTTSSLWKDKAAVEINLAVLHSFQLAKVTIVDHHAATVSF
MKHLDNEQKARGGCPADWAWIVPPISGSLTPVFHQEMVNYILSPAFRYQPDPW

>3RO9A

KVPVVGIVAALLPEMGIGFQGNLPWRLAKEMKYFREVTTLTNDNSKQNVVIMGRKTWESIPQKFR
PLPKRINVVVSRSFDGELRKVEDGIYHSNSLRNCLTALQSSLANENKIERIYIIGGGEIYRQSMD
LADHWLITKIMPLPETTIPQMDTFLQKQELEQRFYDNSDKLVDFLPSSIQLEGRLTSQEWNGELV

KGLPVQEKGYQFYFTLYTKKLEHHHHHHHH
>3RMJA
GIDPFTMTQTNRVIIFDTTLRDGEQSPGAAMTKEEKIRVARQLEKLGVDIIEAGFAAASPGDFEA
VNAIAKTITKSTVCSLSRAIERDIRQAGEAVAPAPKKRIHTFIATSPIHMEYKLKMKPKQVIEAA
VKAVKIAREYTDDVEFSCEDALRSEIDFLAEICGAVIEAGATTINIPDTVGYSIPYKTEEFFREL
IAKTPNGGKVVWSAHCHNDLGLAVANSLAALKGGARQVECTVNGLGERAGNASVEEIVMALKVRH
DLFGLETGIDTTQIVPSSKLVSTITGYPVQPNKAIVGANAFSHESGIHQDGVLKHRETYEIMSAE
SVGWATNRLSLGKLSGRNAFKTKLADLGIELESEEALNAAFARFK
>3AXXA
MEGNTILKIVLICTILAGLFGQVVPVYAENTTYQTPTGIYYEVRGDTIYMINVTSGEETPIHLFG
VNWFGFETPNHVVHGLWKRNWEDMLLQIKSLGFNAIRLPFCTESVKPGTQPIGIDYSKNPDLRGL
DSLQIMEKIIKKAGDLGIFVLLDYHRIGCTHIEPLWYTEDFSEEDFINTWIEVAKRFGKYWNVIG
ADLKNEPHSVTSPPAAYTDGTGATWGMGNPATDWNLAAERIGKAILKVAPHWLIFVEGTQFTNPK
TDSSYKWGYNAWWGGNLMAVKDYPVNLPRNKLVYSPHVYGPDVYNQPYFGPAKGFPDNLPDIWYH
HFGYVKLELGYSVVIGEFGGKYGHGGDPRDVIWQNKLVDWMIENKFCDFFYWSWNPDSGDTGGIL
QDDWTTIWEDKYNNLKRLMDSCSKSSSSTQSVIRSTTPTKSNTSKKICGPAILIILAVFSLLLRR
APR
>3RKLA
MSKITINIKDNTIEYGHKEFVLSNLQEDIKNLAEIVYQLAKLIEKLSQYEEEVDTELYNLLHEYA
IYLAGATSMFIDSENKHHHHHH
>3RIKA
ARPCIPKSFGYSSVVCVCNATYCDSFDPPTFPALGTFSRYESTRSGRRMELSMGPIQANHTGTGL
LLTLQPEQKFQKVKGFGGAMTDAAALNILALSPPAQNLLLKSYFSEEGIGYNIIRVPMASCDFSI
RTYTYADTPDDFQLHNFSLPEEDTKLKIPLIHRALQLAQRPVSLLASPWTSPTWLKTNGAVNGKG
SLKGQPGDIYHQTWARYFVKFLDAYAEHKLQFWAVTAENEPSAGLLSGYPFQCLGFTPEHQRDFI
ARDLGPTLANSTHHNVRLLMLDDQRLLLPHWAKVVLTDPEAAKYVHGIAVHWYLDFLAPAKATLG
ETHRLFPNTMLFASEACVGSKFWEQSVRLGSWDRGMQYSHSIITNLLYHVVGWTDWNLALNPEGG
PNWVRNFVDSPIIVDITKDTFYKQPMFYHLGHFSKFIPEGSQRVGLVASQKNDLDAVALMHPDGS
AVVVVLNRSSKDVPLTIKDPAVGFLETISPGYSIHTYLWHRQ
>3RFYA
GSPGISGGGGGILLVANPVIPDVSVLISGPPIKDPEALLRYALPIDNKAIREVQKPLEDITDSLK
IAGVKALDSVERNVRQASRTLQQGKSIIVAGFAESKKDHGNEMIEKLEAGMQDMLKIVEDRKRDA
VAPKQKEILKYVGGIEEDMVDGFPYEVPEEYRNMPLLKGRASVDMKVKIKDNPNIEDCVFRIVLD
GYNAPVTAGNFVDLVERHFYDGMEIQRSDGFVVQTGDPEGPAEGFIDPSTEKTRTVPLEIMVTGE
KTPFYGSTLEELGLYKAQVVIPFNAFGTMAMAREEFENDSGSSQVFWLLKESELTPSNSNILDGR
YAVFGYVTDNEDFLADLKVGDVIESIQVVSGLENLANPSYKIAG
>2YFKA
METFKEYIEKLDKLEFEKMYENDFFLTWEKTRDELEAVFTVADTLRYLRENNISTKIFDSGLGIS
LFRDNSTRTRFSFASACNLLGLEVQDLDEGKSQISHGETVRETANMISFMADIIGIRDDMYIGKG
NAYMHEVSESVQEGYKDGVLEQRPTLVNLQCDIDHPTQAMADALHLIHEFGGIENLKGKKVAMTW
AYSPSYGKPLSVPQGIVGLMTRLGMDVVLAHPEGYEIMPEVEEVAKKNAAEFGGNFTKTNSMAEA
FKDADVVYPKSWAPFAAMEKRTELYGNGDQAGIDQLEQELLSQNKKHKDWECTEELMKTTKDGKA
LYMHCLPADITGVSCEEGEVEASVFDRYRVELYKEASYKPYVIAAMIFLSKVKNPQKTLTDLADK
ATPREVKDPNSSSVDKLAAALEHHHHHH

>2LBTA
GQAPPGPPASGPCADLQSAINAVTGGPIAFGNDGASLIPAAYEILNRVADKLKACPDARVTINGY
TDNTGSEGINIPLSAQRAKIVADYLVARGVAGDHIATVGLGSVNPIASNATPEGRAKNRRVEIVV
NHHHHHH
>3AXDA
MVSMKDFSGAELYTLEEYQYGKFEARMKMAAASGTVSSMFLYQNGSEIADGRPWVEVDIEVLGKS
PGSFQSNIITGKAGAQKTSEKHHAVSPAADQAFHTYGLEWTPNYVRWTVDGQEVRKTEGGQVSNL
TGTQGLRFNLWSSESAAWVGQFDESKLPLFQFINWVKVYKYTPGQGEGGSDFTLDWTDNFDTFDG
SRWGKGDYTFDGNRVDLTDKNIYSRDGMLILALTRKGQESFNGQVPRDDEPAPL
>2YEVB
MQRSFAALGLWGLSLAQEAHRVAITHPGGSFNQEVAFLFPWVYFFSFLIFLVVAGSLAYVTWKFR
ARPEDQEEPPQIHGNDRLEVVWTLIPLAIVFVLFGLTAKALIQVNRPIPGAMKVEVTGYQFWWDF
HYPELGLRNSNELVLPAGVPVELEITSKDVIHSFWVPGLAGKRDAIPGQTTRISFEPKEPGLYYG
FCAELCGASHARMLFRVVVLPKEEFDRFVEAAKASPAPVADERGQQVFQQNCAACHGVARSMPPA
VIGPELGLWGNRTSLGAGIVENTPENLKAWIRDPAGMKPGVKMPGFPQLSEEDLDALVRYLEGLK
VEGFDFGALPKF
>2YEVA
MAITAKPKAGVWAVLWDLLTTVDHKKIGLMYTATAFFAFALAGVFSLLIRTQLAVPNNQFLTGEQ
YNQILTLHGATMLFFFIIQAGLTGFGNFVVPLMLGARDVALPRVNAFSYWAFLGAIVLALMSYFF
PGGAPSVGWTFYYPFSAQSESGVDFYLAAILLLGFSSLLGNANFVATIYNLRAQGMSLWKMPIYV
WSVFAASVLNLFSLAGLTAATLLVLLERKIGLSWFNPAVGGDPVLFQQFFWFYSHPTVYVMLLPY
LGILAEVASTFARKPLFGYRQMVWAQMGIVVLGTMVWAHHMFTVGESTLFQIAFAFFTALIAVPT
GVKLFNIIGTLWGGKLQMKTPLYWVLGFIFNFLLGGITGVMLSMTPLDYQFHDSYFVVAHFHNVL
MAGSGFGAFAGLYYWWPKMTGRMYDERLGRLHFWLFLVGYLLTFLPQYALGYLGMPRRYYTYNAD
IAGWPELNLLSTIGAYILGLGGLVWIYTMWKSLRSGPKAPDNPWGGYTLEWLTASPPKAHNFDVK
LPTEFPSERPLYDWKKKGVELKPEDPAHIHLPNSSFWPFYSAATLFAFFVAVAALPVPNVWMWVF
LALFAYGLVRWALEDEYSHPVEHHTVTGKSNAWMGMAWFIVSEVGLFAILIAGYLYLRLSGAATP
PEERPALWLALLNTFLLVSSSFTVHFAHHDLRRGRFNPFRFGLLVTIILGVLFFLVQSWEFYQFY
HHSSWQENLWTAAFFTIVGLHGLHVVIGGFGLILAYLQALRGKITLHNHGTLEAASMYWHLVDAV
WLVIVTIFYVW
>3RBUA
RSGLNDIFEAQKIEWHEGSGSGSENLYFQGRSKSSNEATNITPKHNMKAFLDELKAENIKKFLYN
FTQIPHLAGTEQNFQLAKQIQSQWKEFGLDSVELAHYDVLLSYPNKTHPNYISIINEDGNEIFNT
SLFEPPPPGYENVSDIVPPFSAFSPQGMPEGDLVYVNYARTEDFFKLERDMKINCSGKIVIARYG
KVFRGNKVKNAQLAGAKGVILYSDPADYFAPGVKSYPDGWNLPGGGVQRGNILNLNGAGDPLTPG
YPANEYAYRRGIAEAVGLPSIPVHPIGYYDAQKLLEKMGGSAPPDSSWRGSLKVPYNVGPGFTGN
FSTQKVKMHIHSTNEVTRIYNVIGTLRGAVEPDRYVILGGHRDSWVFGGIDPQSGAAVVHEIVRS
FGTLKKEGWRPRRTILFASWDAEEFGLLGSTEWAEENSRLLQERGVAYINADSSIEGNYTLRVDC
TPLMYSLVHNLTKELKSPDEGFEGKSLYESWTKKSPSPEFSGMPRISKLGSGNDFEVFFQRLGIA
SGRARYTKNWETNKFSGYPLYHSVYETYELVEKFYDPMFKYHLTVAQVRGGMVFELANSIVLPFD
CRDYAVVLRKYADKIYSISMKHPQEMKTYSVSFDSLFSAVKNFTEIASKFSERLQDFDKSNPIVL
RMMNDQLMFLERAFIDPLGLPDRPFYRHVIYAPSSHNKYAGESFPGIYDALFDIESKVDPSKAWG
EVKRQIYVAAFTVQAAAETLSEVA
>2YDQA

GSVGPKTGEENQVLVPNLNPTPENLEVVGDGFKITSSINLVGEEEADENAVNALREFLTANNIEI
NSENDPNSTTLIIGEVDDDIPELDEALNGTTAENLKEEGYALVSNDGKIAIEGKDGDGTFYGVQT
FKQLVKESNIPEVNITDYPTVSARGIVEGFYGTPWTHQDRLDQIKFYGENKLNTYIYAPKDDPYH
REKWREPYPESEMQRMQELINASAENKVDFVFGISPGIDIRFDGDAGEEDFNHLITKAESLYDMG
VRSFAIYWDNIQDKSAAKHAQVLNRFNEEFVKAKGDVKPLITVPTEYDTGAMVSNGQPRAYTRIF
AETVDPSIEVMWTGPGVVTNEIPLSDAQLISGIYDRNMAVWWNYPVTDYFKGKLALGPMHGLDKG
LNQYVDFFTVNPMEHAELSKISIHTAADYSWNMDNYDYDKAWNRAIDMLYGDLAEDMKVFANHST
RMDNKTWAKSGREDAPELRAKMDELWNKLSSKEDASALIEELYGEFARMEEACNNLKANLPEVAL
EECSRQLDELITLAQGDKASLDMIVAQLNEDTEAYESAKEIAQNKLNTALSSFAVISEKVAQSFI
QEALS

>3R6TA

MGDTKEQRILRYVQQNAKPGDPQSVLEAIDTYCTQKEWAMNVGDAKGQIMDAVIREYSPSLVLEL
GAYCGYSAVRMARLLQPGARLLTMEINPDCAAITQQMLNFAGLQDKVTILNGASQDLIPQLKKKY
DVDTLDMVFLDHWKDRYLPDTLLLEKCGLLRKGTVLLADNVIVPGTPDFLAYVRGSSSFECTHYS
SYLEYMKVVDGLEKAIYQGPSSPDKS

>3R3JA

LHNYGYTSTKSVDNQIEELREKVVSKNKNEPEFLQAFEEVLSCLKPVFKKDNVYIGVLENIAEPE
RVIQFRVPWINDKGEHKMNRGFRVQYNSVLGPYKGGLRFHPAVNLSVIKFLGFEQIFKNSLTTLP
MGGGKGGSDFDPKGKSENEILKFCQSFMTNLFRYIGPNTDVPAGDIGVGGREIGYLFGQYKKLKN
SFEGVLTGKNIKWGGSNIRAEATGYGVVYFAENVLKDLNDNLENKKCLVSGSGNVAQYLVEKLIE
KGAIVLTMSDSNGYILEPNGFTKEQLNYIMDIKNNQRLRLKEYLKYSKTAKYFENQKPWNIPCDI
AFPCATQNEINENDADLFIQNKCKMIVEGANMPTHIKALHKLKQNNIILCPSKAANAGGVAVSGL
EMSQNSMRLQWTHQETDMKLQNIMKSIYEQCHNTSKIYLNESDLVAGANIAGFLKVADSFLEQGG
L

>3R18A

APSYPEYTREEVGRHRSPEERVWVTHGTDVFDVTDFVELHPGGPDKILLAAGGALEPFWALYAVH
GEPHVLELLQQYKVGELSPDEAPAAPDAQDPFAGDPPRHPGLRVNSQKPFNAEPPAELLAERFLT
PNELFFTRNHLPVPAVEPSSYRLRVDGPGGGTLSLSLAELRSRFPKHEVTATLQCAGNRRSEMSR
VRPVKGLPWDIGAISTARWGGARLRDVLLHAGFPEELQGEWHVCFEGLDADPGGAPYGASIPYGR
ALSPAADVLLAYEMNGTELPRDHGFPVRVVVPGVVGARSVKWLRRVAVSPDESPSHWQQNDNKGF
SPCVDWDTVDYRTAPAIQELPVQSAVTQPRPGAAVPPGELTVKGYAWSGGGREVVRVDVSLDGGR
TWKVARLMGDKAPPGRAWAWALWELTVPVEAGTELEIVCKAVDSSYNVQPDSVAPIWNLMGVLST
AWHRVRVSVQD

>3QVSA

MKVWLVGAYGIVSTTAMVGARAIERGIAPKIGLVSELPHFEGIEKYAPFSFEFGGHEIRLLSNAY
EAAKEHWELNRHFDREILEAVKSDLEGIVARKGTALNCGSGIKELGDIKTLEGEGLSLAEMVSRI
EEDIKSFADDETVVINVASTEPLPNYSEEYHGSLEGFERMIDEDRKEYASASMLYAYAALKLGLP
YANFTPSPGSAIPALKELAEKKGVPHAGNDGKTGETLVKTTLAPMFAYRNMEVVGWMSYNILGDY
DGKVLSARDNKESKVLSKDKVLEKMLGYSPYSITEIQYFPSLVDNKTAFDFVHFKGFLGKLMKFY
FIWDAIDAIVAAPLILDIARFLLFAKKKGVKGVVKEMAFFFKSPMDTNVINTHEQFVVLKEWYSN
LK

>3QSYA

MAWPKVQPEVNIGVVGHVDHGKTTLVQAITGIWTSKHSEELKRGMTIKLGYAETNIGVCESCKKP
EAYVTEPSCKSCGSDDEPKFLRRISFIDAPGHEVLMATMLSGAALMDGAILVVAANEPFPQPQTR

EHFVALGIIGVKNLIIVQNKVDVVSKEEALSQYRQIKQFTKGTWAENVPIIPVSALHKINIDSLI
EGIEEYIKTPYRDLSQKPVMLVIRSFDVNKPGTQFNELKGGVIGGSIIQGLFKVDQEIKVLPGLR
VEKQGKVSYEPIFTKISSIRFGDEEFKEAKPGGLVAIGTYLDPSLTKADNLLGSIITLADAEVPV
LWNIRIKYNLLERVVGAKEMLKVDPIRAKETLMLSVGSSTTLGIVTSVKKDEIEVELRRPVAVWS
NNIRTVISRQIAGRWRMIGWGLVEI
>3AUKA
MAASRANDAPIVLLHGFTGWGREEMFGFKYWGGVRGDIEQWLNDNGYRTYTLAVGPLSSNWDRAC
EAYAQLVGGTVDYGAAHAAKHGHARFGRTYLGLLPELKRGGRIHIIAHSQGGQTARMLVSLLENG
SQEEREYAKAHNVSLSPLFEGGHHFVLSVTTIATPHDGTTLVNMVDFTDRFFDLQKAVLEAAAVA
SNVPYTSQVYDFKLDQWGLRRQPGESFDHYFERLKRSPVWTSTDTARYDLSVSGAEKLNQWVQAS
PNTYYLSFATERTYRGALTGNYYPELGMNAFSAVVCAPFLGSYRNPTLGIDDRWLENDGIVNTVS
MNGPKRGSSDRIVPYDGALKKGVWNDMGTYNVDHLEIIGVDPNPSFDIRAFYLRLAEQLASLQP
>3QMXA
MRGSHHHHHHGSAVSAKIEIYTWSTCPFCMRALALLKRKGVEFQEYCIDGDNEAREAMAARANGK
RSLPQIFIDDQHIGGCDDIYALDGAGKLDPLLHS
>3QLIA
HHHHHHSSGLVPRGSMDIRALYDEKLTTPEEAVSSIASGSHLSMGMFAAEPPALLKALADRATRG
DIGDLRVYYFETAKIAGDTILRYELNNRIKPYSMFVTAVERALIRRGIEDGGRKVVNYVPSNFHQ
APRLLAEEIGIDTFMHTVSPMDCHGYFSLGVGNDYSSRIARSARRFIVEVNRYMPRVQGEAAAIH
ISEVDAIVENHVPLIEMPVRSAIPEYTSISHIIADLVPDGACLQMGVGALPNLVCGVLKDRNDLG
IHTEVLNPGLVDLIRRGVVTNQRKTLDRGRSVFTFAMGQQEMYEYLNDHPAIFSRPVDYVNDPHI
IAQNDNVVSINATLQIDLTGACNSEHMLGHQYSASGGQLDFVRGAYASKGGRSIIATPSTAAKGT
VSRIIPRIDGPVTTPRIDTHYIVTEFGAVNLKGLSSTERALRIIELAHPDFRDELTQAAKKMHLI
>2L97A
MEGLGFAIPANDAINIIEQLEKNGKVTRPALGIQMVNLSNVSTSDIRRLNIPSNVTSGVIVRSVQ
SNMPANGHLEKYDVITKVDDKEIASSTDLQSALYNHSIGDTIKITYYRNGKEETTSIKLNKLEHH
HHHH
>3QKGA
GPVPTPPDNIQVQENFNISRIYGKWYNLAIGSTSPWLKKIMDRMTVSTLVLGEGATEAEISMTST
RWRKGVCEETSGAYEKTDTDGKFLYHKSKWNITMESYVVHTNYDEYAIFLTKKFSRHHGPTITAK
LYGRAPQLRETLLQDFRVVAQGVGIPEDSIFTMADRGECVPGEQEPEPILIPRSAWSHPQFEK
>3QJJA
HHHHMRIEVKLLPLKDNPILPFNYNYEVYSQILEKVNSIEPTIAKLLSSPHGFWTFSRIIVRKRK
ILPDKGIEILSDDVSLYISSSNEDIIRAIAEAVEKSPEFKIGELSFLVGDIKAIKVKELGKENVF
STLSPIVVRTVKFEGNKLRHWDLYPHDELFMDRLRKVMILRYSEVMGETPKDRDFTIEVLKFKPT
RLMVGSSYIRGSLMVFRYAGSEEIARFGYENGFGEKTGLGFGMVKLIE
>2Y6YA
GAMTIGRAKVYATLSKIFYHLFYDEAIPKDCREIIEKFGEIDFNLRSVLVRELRGSVLIKDMPQS
LAEVYESVMKDFYERYGFQASELHADHIAVELAFMSKLVEREISLAQQMKEEELYKIRAAQHRFI
KAHLQPLVKNLPSAPLLNFVRDFVREDAKYLYSSLVGEKNEGADNN
>3QDDA
HMPEETQTQDQPMEEEEVETFAFQAEIAQLMSLIINTFYSNKEIFLRELISNSSDALDKIRYESL
TDPSKLDSGKELHINLIPNKQDRTLTIVDTGIGMTKADLINNLGTIAKSGTKAFMEALQAGADIS
MIGQFGVGFYSAYLVAEKVTVITKHNDDEQYAWESSAGGSFTVRTDTGEPMGRGTKVILHLKEDQ

TEYLEERRIKEIVKKHSQFIGYPITLFVEKERDKEVSDDEAE

>3QBDA

HHHHHHMTDIGAPVTVQVAVDPPYPVVIGTGLLDELEDLLADRHKVAVVHQPGLAETAEEIRKRL
AGKGVDAHRIEIPDAEAGKDLPVVGFIWEVLGRIGIGRKDALVSLGGGAATDVAGFAAATWLRGV
SIVHLPTTLLGMVDAAVGGKTGINTDAGKNLVGAFHQPLAVLVDLATLQTLPRDEMICGMAEVVK
AGFIADPVILDLIEADPQAALDPAGDVLPELIRRAITVKAEVVAADEKESELREILNYGHTLGHA
IERRERYRWRHGAAVSVGLVFAAELARLAGRLDDATAQRHRTILSSLGLPVSYDPDALPQLLEIM
AGDKKTRAGVLRFVVLDGLAKPGRMVGPDPGLLVTAYAGVCAP

>3QB4A

MKRQGKRPSKNLKARCSRKALHVNFKDMGWDDWIIAPLEYEAFHCEGLCEFPLASHLEPTNHAVI
QTLMNSMDPESTPPTCCVPTRLSPISILFIDSANNVVYKQYEDMVVESCGCR

>2L8BA

TSGIHVLDELSVRALSRDIMKQNRVTVHPEKSVPRTAGYSDAVSVLAQDRPSLAIVSGQGGAAGQ
RERVAELVMMAREQGREVQIIAADRRSQMNMKQDERLSGELITGRRQLLEGMAFTPGSTVIVDQG
EKLSLKETLTLLDGAARHNVQVLITDSGQRTGTGSALMAMKDAGVNTYRWQGGEQRPAT

>2V2GA

MGITLGEVFPNFEADSTIGKLKFHDWLGNSWGVLFSHPRDFTPVSTTELGRVIQLEGDFKKRGVK
LIALSCDNVADHKEWSEDVKCLSGVKGDMPYPIIADETRELAVKLGMVDPDERTSTGMPLTCRAV
FIIGPDKKLKLSILYPATTGRNFSEILRVIDSLQLTAQKKVATPADWQPGDRCMVVPGVSAEEAK
TLFPNMEVKAVPSGKGYLRYTPQPKSMGGSRSHHHHHH

>3D33A

GANTTETPVKDVELDGRWDDPIRSAATNCPITVFTDGYLLTLKNASPDRDMTIRITDMAKGGVVY
ENDIPEVQSAYITISIANFPAEEYKLEITGTPSGHLTGYFTKE

>2J9OA

MPAPEAPTSTLPPERPLTNLQQQIQQLVSRQPNLTAGLYFFNLDSGASLNVGGDQVFPAASTIKF
PILVAFFKAVDEGRVTLQERLTMRPDLIAPEAGTLQYQKPNSQYAALEVAELMITISDNTATNMI
IDRLGGAAELNQQFQEWGLENTVINNPEPDMKGTNTTSPRDLATLMLKIGQGEILSPRSRDRLLD
IMRRTVTNTLLPAGLGKGATIAHKTGDIGIVVGDAGMVDMPNGQRYVAAMMVKRPYNDPRGSELI
RQVSRMVYQAFEKLSPPEQKLISEEDLNSAVDHHHHHH

>2WI8A

MGSKNESTASKASGTASEKKKIEYLDKTYEVTVPTDKIAITGSVESMEDAKLLDVHPQGAISFSG
KFPDMFKDITDKAEPTGEKMEPNIEKILEMKPDVILASTKFPEKTLQKISTAGTTIPVSHISSNW
KENMMLLAQLTGKEKKAKKIIADYEQDLKETKTKINDKAKDSKALVIRIRQGNIYIYPEQVYFNS
TLYGDLGLKAPNEVKAAKAQELISLEKLSEMNPDHIFVQFSDDENADKPDALKDLEKNPIWKSLK
AVKEDHVYVNSVDPLAQGGTAWSKVRFLKAAAEKLTQNKLAAALEHHHHHH

>4JDNA

SLGLLKAFNNFPITNKIQCNGLFTPSNIETLLGGTEIGKFTVTPKSSGSMFLVSADIIASRMEGG
VVLALVREGDSKPCAISYGYSSGVPNLCSLRTSITNTGLTPTTYSLRVGGLESGVVWVNALSNGN
DILGITNTSNVSFLEVIPQTNA

>2PR7A

GMRGLIVDYAGVLDGTDEDQRRWRNLLAAAKKNGVGTVILSNDPGGLGAAPIRELETNGVVDKVL
LSGELGVEKPEEAAFQAAADAIDLPMRDCVLVDDSILNVRGAVEAGLVGVYYQQFDRAVVEIVGL
FGLEGEF

>3G8YA

GYQPEKHAVVKSDRGDGRLLSTYAIVHEMLKDTHPQYAYRSGMSAQEFTQWQDGVRAAMVEIMKF
PEIKRQPSPVCVKTEKKEGYILEKWEFYPFPKSVSTFLVLKPEHLKGAVPGVLCIPGSGRTKEGL
VGEPGICDKLTEDYNNPKVSMALNMVKEGYVAVAVDNAAAGEASDLECYDKGWNYDYDVVSRFLL
ELGWSWLGYTSYLDMQVLNWMKAQSYIRKDRIVISGFSLGTEPMMVLGVLDKDIYAFVYNDFLCQ
TQERAVVMTKPDKENRRPFPNSIRHLIPGYWRYFNFPDVVASLAPRPIIFTEGGLDRDFRLVQSA
YAASGKPENAEFHHYPKFADKAVRKDVEHLDEGLDSKTYFEAVNVDPPSHYFKNELVIPWLRKVL
K
>3MC9A
MLETRATTAKQLVQAPEQPAPQPEVAPPTTEQPAPAPAPGTTPGTENFNTPNATPETTEPRVLVS
EVLVRPQSGQLTPELETQVYNVIRTQPGRTTTRSQLQEDINAIFGTGFFSNVQASPEDTPLGVRV
SFIVQPNPVLSKVEIQANPGTNVPSVLPQATADEIFRAQYGKILNLRDLQEGIKELTKRYQDQGY
VLANVVGAPQVSENGVVTLQVAEGVVENISVRFRNKEGQDVNEQGQPIRGRTQDYIITREVELKP
GQVFNRNTVQKDLQRVFGTGLFEDVNVSLDPGTDPTKVNVVVNVVERSLEHHHHHH
>4EIRA
HTIFSSLEVNGVNQGLGEGVRVPTYNGPIEDVTSASIACNGSPNTVASTSKVITVQAGTNVTAIW
RYMLSTTGDSPADVMDSSHKGPTIAYLKKVDNAATASGVGNGWFKIQQDGMDSSGVWGTERVING
KGRHSIKIPECIAPGQYLLRAEMIALHAASNYPGAQFYMECAQLNVVGGTGAKTPSTVSFPGAYS
GSDPGVKISIYWPPVTAYTVPGPSVFTC
>3O1IC
GSGSDEKICAIYPHLKDSYWLSVNYGMVSEAEKQGVNLRVLEAGGYPNKSRQEQQLALCTQWGAN
AIILGTVDPHAYEHNLKSWVGNTPVFATVNQLDLDEEQSTLLKGEVGVDWYWMGYEAGKYLAERH
PKGSGKTNIALLLGPRTRGGTKPVTTGFYEAIKNSDIHIVDSFWADNDKELQRNLVQRVIDMGNI
DYIVGSAVAIEAAISELRSADKTHDIGLVSVYLSHGVYRGLLRNKVLFAPTDKMVQQGRLSVMQA
AHYLRHQPYEKQASPIIKPLTPKTLHDDTIEESLSPSEYRPTFS
>4KQ9A
GGGSAAGKKVVYSTFGAQIPFFNRIGEGAKAQATVRRLDFDISTSEIDPGKQIDSIDNAVAQQPD
GLIVSPIDGSALVPTIKGAVEDGVPVILLADGLSEDVGQLSFVGSDFAEIGRLKATYIADRLGDG
GTVAMVNGTRGMSFVEEQGEAAREVFEERGIEIVDDVYTKAITPDEGLTATQNILTRHSDVGAIY
YSGDDGALGGIRAIAARNIAPGKIMVVGTDANEGALAAVRAGTMALTVSQCAYEQGGIAIDVMAD
YLETGKKPDRRIFTPVIEIDTETIDRVMSGAAWERCENH
>3F47A
MKIAILGAGCYRTHAAAGITNFMRACEVAKEVGKPEIALTHSSITYGAELLHLVPDVKEVIVSDP
CFAEEPGLVVIDEFDPKEVMEAHLSGNPESIMPKIREVVKAKAKELPKPPKACIHLVHPEDVGLK
VTSDDREAVEGADIVITWLPKGNKQPDIIKKFADAIPEGAIVTHACTIPTTKFAKIFKDLGREDL
NITSYHPGCVPEMKGQVYIAEGYASEEAVNKLYEIGKIARGKAFKMPANLIGPVCDMCSAVTATV
YAGLLAYRDAVTKILGAPADFAQMMADEALTQIHNLMKEKGIANMEEALDPAALLGTADSMCFGP
LAEILPTALKVLEKHKVVEEEGKTKCEIMSQKE
>1GKPA
PLLIKNGEIITADSRYKADIYAEGETITRIGQNLEAPPGTEVIDATGKYVFPGFIDPHVHIYLPF
MATFAKDTHETGSKAALMGGTTTYIEMCCPSRNDDALEGYQLWKSKAEGNSYCDYTFHMAVSKFD
EKTEGQLREIVADGISSFKIFLSYKNFFGVDDGEMYQTLRLAKELGVIVTAHCENAELVGRLQQK
LLSEGKTGPEWHEPSRPEAVEAEGTARFATFLETTGATGYVVHLSCKPALDAAMAAKARGVPIYI
ESVIPHFLLDKTYAERGGVEAMKYIMSPPLRDKRNQKVLWDALAQGFIDTVGTDHCPFDTEQKLL
GKEAFTAIPNGIPAIEDRVNLLYTYGVSRGRLDIHRFVDAASTKAAKLFGLFPRKGTIAVGSDAD

LVVYDPQYRGTISVKTQHVNNDYNGFEGFEIDGRPSVVTVRGKVAVRDGQFVGEKGWGKLLRREP
MYF
>2GK9A
SMHFVQQKVKVFRAADPLVGVFLWGVAHSINELSQVPPPVMLLPDDFKASSKIKVNNHLFHRENL
PSHFKFKEYCPQVFRNLRDRFGIDDQDYLVSLTRNPPSESEGSDGRFLISYDRTLVIKEVSSEDI
ADMHSNLSNYHQYIVKCHGNTLLPQFLGMYRVSVDNEDSYMLVMRNMFSHRLPVHRKYDLKGSLV
SREASDKEKVKELPTLRDMDFLNKNQKVYIGEEEKKIFLEKLKRDVEFLVQLKIMDYSLLLGIHD
IIRGSEPEEEAPVREDESEVDGDCSLTGPPALVGSYGTSPEGIGGYIHSHRPLGPGEFESFIDVY
AIRSAEGAPQKEVYFMGLIDILTQYDAKKKAAHAAKTVKHGAGAEISTVHPEQYAKRFLDFITNI
FA
>3JTWA
GMARKVILFIAMSIDNYIADDQGAVDWLEKNVHGTESDDSYEKMYSKIDTVIMGRTTYEQVTQKL
SPEKYVYADRQTYIVTSHLGEDTDKIKYWKQSPVELVKRIQKEKGKDVWIVGGAKIIDPLVQANL
IDTYILTTVPIFLGSGIRLFDRLEEQVPVRLIDVYQKNELVYSIYQRG
>3CNEA
MAKKVAVLAVNPVNGCGLFQYLEAFFENGISYKVFAVSDTKEIKTNSGMVLIVDDVIANLKGHED
EFDALVFSCGDAVPVFQQYANQPYNVDLMEVIKTFGEKGKMMIGHCAGAMMFDFTGITKGKKVAV
HPLAKPAIQNGIATDEKSEIDGNFFTAQDENTIWTMLPKVIEALK
>3OY2A
MKLIIVGAHSSVPSGYGRVMRAIVPRISKAHEVIVFGIHAFGRSVHANIEEFDAQTAEHVRGLNE
QGFYYSGLSEFIDVHKPDIVMIYNDPIVIGNYLLAMGKCSHRTKIVLYVDLVSKNIRENLWWIFS
HPKVVGVMAMSKCWISDICNYGCKVPINIVSHFVDTKTIYDARKLVGLSEYNDDVLFLNMNRNTA
RKRLDIYVLAAARFISKYPDAKVRFLCNSHHESKFDLHSIALRELVASGVDNVFTHLNKIMINRT
VLTDERVDMMYNACDVIVNCSSGEGFGLCSAEGAVLGKPLIISAVGGADDYFSGDCVYKIKPSAW
ISVDDRDGIGGIEGIIDVDDLVEAFTFFKDEKNRKEYGKRVQDFVKTKPTWDDISSDIIDFFNSL
LRVESRETPGNEEHPLEHHHHHH
>3HH1A
SNAHKGTLYVVATPLGNLDDMTFRAVNTLRNAGAIACEDTRRTSILLKHFGIEGKRLVSYHSFNE
ERAVRQVIELLEEGSDVALVTDAGTPAISDPGYTMASAAHAAGLPVVPVPGA
>1Z2NX
GPLGSMTTKQTVSLFIWLPESKQKTLFISTKNHTQFELNNIIFDVTLSTELPDKEPNAIITKRTH
PVGKMADEMRKYEKDHPKVLFLESSAIHDMMSSREEINALLIKNNIPIPNSFSVKSKEEVIQLLQ
SKQLILPFIVKPENAQGTFNAHQMKIVLEQEGIDDIHFPCLCQHYINHNNKIVKVFCIGNTLKWQ
TRTSLPNVHRCGIKSVDFNNQHLEDILSWPEGVIDKQDIIENSANRFGSKILEDPILLNLTSEAE
MRDLAYKVRCALGVQLCGIDFIKENEQGNPLVVDVNVFPSYGGKVDFDWFVEKVALCYTEVAKI
>3GREA
MGHHHHHHGEGDVESIEKFLSTFKILPPLRDYKEFGPIQEIVRSPNMGNLRGKLIATLMENEPNS
ITSSAVSPGETPYLITGSDQGVIKIWNLKEIIVGEVYSSSLTYDCSSTVTQITMIPNFDAFAVSS
KDGQIIVLKVNHYQQESEVKFLNCECIRKINLKNFGKNEYAVRMRAFVNEEKSLLVALTNLSRVI
IFDIRTLERLQIIENSPRHGAVSSICIDEECCVLILGTTRGIIDIWDIRFNVLIRSWSFGDHAPI
THVEVCQFYGKNSVIVVGGSSKTFLTIWNFVKGHCQYAFINSDEQPSMEHFLPIEKGLEELNFCG
IRSLNALSTISVSNDKILLTDEATSSIVMFSLNELSSSKAVISPSRFSDVFIPTQVTANLTMLLR
KMKRTSTHSVDDSLYHHDIINSISTCEVDETPLLVACDNSGLIGIFQ
>1EZGA

QCTGGADCTSCTGACTGCGNCPNAVTCTNSQHCVKANTCTGSTDCNTAQTCTNSKDCFEANTCTD
STNCYKATACTNSSGCPGH
>2WNVB
ATQKIAFSATRTINVPLRRDQTIRFDHVITNMNNNYEPRSGKFTCKVPGLYYFTYHASSRGNLCV
NLMRGRERAQKVVTFCDYAYNTFQVTTGGMVLKLEQGENVFLQATDKNSLLGMEGANSIFSGFLL
FPDMEA
>4IQNA
SNAMDISLTNLIELVKKVNRNKVPTPMSAEEISRLRVRKYRDPQNTETTELPESLKALLAYDRDL
LSNYNMPVIETLQKSIDNEGVIHSYSPDEEAYYGVGMDSSGIDIEDLMPVWSNDPRLPALIRIDH
VGDQAIFIYITERDANGEYPIARMERNEFWLAESSLVEYLYNIISGAKDIGFTEEDLHLPQWKAQ
QKMNEQRDAALLDLEDYHEAFWAKLDALVD
>2PA7A
GHMENKVINFKKIIDSRGSLVAIEENKNIPFSIKRVYYIFDTKGEEPRGFHAHKKLEQVLVCLNG
SCRVILDDGNIIQEITLDSPAVGLYVGPAVWHEMHDFSSDCVMMVLASDYYDETDYIRQYDNFKK
YIAKINLEKEG
>3HL6A
MDIETIVNEFETRAGTLLRYYTGLLERSKVQPCCFKLYNDPFDMVYVMMNSKLFSHVYIKDCKVR
QSFELASPKHTEGLIRSIEGHYVGYELHDGKQLSISDMMASQLFEDEYFMYGLQTYAESNNSDVF
KCLENGFDTDTLEGIQSSNTDVIANIEMLYQLATGINEPVPELVEGLKLVTEFVQDENATQEDYK
ALERKLNDLKASYYSLSKLAAALEHHHHHH
>3P06A
NGVELSAVGVLLPVLMDSGRRISGGAFMAVKGDLSEHIKNPKNTRIAQTVAGGTIYGLSEMVNID
EAEKLPIKGAITVLPVVQATATSILVPDNQPQLAFNSWEAAACAADTLESQQTPFLMVTGAVESG
NLSPNLLAVQKQLLVAKPAGIGLAANSDRALKVVTLEQLRQVVGDKPWRKPMVTFSSGKNVAQA
>3KP8A
SPLAVGLAAHLRQIGGTMYGAYWCPHCQDQKELFGAAFDQVPYVECSPNGPGTPQAQECTEAGIT
SYPTWIINGRTYTGVRSLEALAVASGYPLEEGRLEHHHHHH
>3HG9A
MSLTSSAELAEVDTLARSLLLYRSRLAEYAHANPGFSGSPADSALGLPAWFRKPVRLQGYIAAGT
SYAFIASPPAGLAAAVDTGTESDLVGVRRNGQLVTRRLGATAIALPAPIPEGAVVAVKEGHHHHH
H
>3H0UA
MSLTASYETIKARLDGTVLSATFNAPPMNLIGPEVVRDLVALLEELAHPTAPRVVIFDSADADFF
FPHVDMTKVPEYTAEAAKAGGPGDASLGMLFRKLSQLPAVTIAKLRGRARGAGSEFLLACDMRFA
SRENAILGQPEVGIGAPPGAGAIQHLTRLLGRGRALEAVLTSSDFDADLAERYGWVNRAVPDAEL
DEFVAGIAARMSGFPRDALIAAKSAINAISLPAPAEVRADAALFQQLVRGEKVQQRTAELFKQGF
QTRGATELDLGDALGHLKAVDEGHHHHHH
>3ZBOA
GSHMASMDFKTVMQELEALGKERTKKIYISNGAHEPVFGVATGAMKPIAKKIKLNQELAEELYAT
GNYDAMYFAGIIADPKAMSESDFDRWIDGAYFYMLSDYVVAVTLSESNIAQDVADKWIASGDELK
MSAGWSCYCWLLGNRKDNAFSESKISDMLEMVKDTIHHSPERTKSAMNNFLNTVAISYVPLHEKA
VEIAKEVGIVEVKRDNKKSSLLNASESIQKELDRGRLGFKRKYVRC
>3DDEA
GMSIIDLTKLEQKVATMWDSILTNSPFIHEVLDGKATKALYAIYMTETYHYTKHNAKNQALVGIM

GKDLPGKYLSFCFHHAHEEAGHELMALSDIASIGFDREDVLSSKPLPATETLIAYLYWISATGNP
VQRLGYSYWAENVYGYIDPVLKAIQSTLDLTPQSMKFFIAHSKIDAKHAEEVNEMLHEVCKTQED
VDSVVAVMENSLVLTARILDDVWKEYQLFQSGASDRYAFLRDNA
>1GWMA
MNVRATYTVIFKNASGLPNGYDNWGWGCTLSYYGGAMIINPQEGKYGAVSLKRNSGSFRGGSLRF
DMKNEGKVKILVENSEADEKFEVETISPSDEYVTYILDVDFDLPFDRIDFQDAPGNGDRIWIKNL
VHSTGSADDFVDPINLEHHHHHH
>3HXLA
MAAGTFTAQNKVRPGVYINFKSEPQAAGTLGERGIVSMPLILSWGEPGKMITIEAGDDVFPKLGY
SIMDAQLRLINEALKRAKTLLLYRLNAGTKAAVTVGNLTVTAKWGGARGNDITLVIQENIDDETK
FDVSTLVDGAELDKQTVSDIAGLAANDWVIFSGTGALTETAGAPLINGSDGAVTNQAYIDYLAAV
EIFDFNTIALPSTDDALKATFTAFAKRLRDDEGKKIQVVLENYPAADYEGVISVKNGVVLADGTI
LTAAQATAWVAGATAGARVNESLTYQGYDEAVDVAPRYTNAQIIAALQAGEFLFTASDNQALVEQ
DINTLTSFTADKGKQFAKNRVIRVLDGINNDFVRIFSKFYIGKVSNNADGRNLLKSECINYMNTL
QDIDAIKNFDGQTDLTVQSGNDVDAVYIEAYAWPVDSIEKIYVRVRIKLEHHHHHH
>3DCPA
MKRDGHTHTEFCPHGTHDDVEEMVLKAIELDFDEYSIVEHAPLSSEFMKNTAGDKEAVTTASMAM
SDLPYYFKKMNHIKKKYASDLLIHIGFEVDYLIGYEDFTRDFLNEYGPQTDDGVLSLHFLEGQGG
FRSIDFSAEDYNEGIVQFYGGFEQAQLAYLEGVKQSIEADLGLFKPRRMGHISLCQKFQQFFGED
TSDFSEEVMEKFRVILALVKKRDYELDFNTAGLFKPLCGETYPPKKIVTLASELQIPFVYGSDSH
GVQDIGRGYSTYCQKLEHHHHHH
>4H14A
VIGDLKCTTVSINDVDTGAPSISTDTVDVTNGLGTYYVLDRVYLNTTLLLNGYYPTSGSTYRNMA
LKGTLLLSRLWFKPPFLSDFINGIFAKVKNTKVIKKGVMYSEFPAITIGSTFVNTSYSVVVQPHT
TNLDNKLQGLLEISVCQYTMCEYPHTICHPKLGNKRVELWHWDTGVVSCLYKRNFTYDVNADYLY
FHFYQEGGTFYAYFTDTGVVTKFLFNVYLGTVLSHYYVLPLTCSSAMTLEYWVTPLTSKQYLLAF
NQDGVIFNAVDCKSDFMSEIKCKTHHHHHH
>1O57A
MKFRRSGRLVDLTNYLLTHPHELIPLTFFSERYESAKSSISEDLTIIKQTFEQQGIGTLLTVPGA
AGGVKYIPKMKQAEAEEFVQTLGQSLANPERILPGGYVYLTDILGKPSVLSKVGKLFASVFAERE
IDVVMTVATKGIPLAYAAASYLNVPVVIVRKDNKVTEGSTVSINYVSGSSNRIQTMSLAKRSMKT
GSNVLIIDDFMKAGGTINGMINLLDEFNANVAGIGVLVEAEGVDERLVDEYMSLLTLSTINMKEK
SIEIQNGNFLRFFKDNLLKNGETESHHHHHH
>3EZ0A
GMSTSPADTARYNRFVADLFGMMAYGELSAFERFSADARYSPTLHDRAVLGRIAVVEFRHYELVS
ARLEAMGIDAEDAMLPFQAAVDYFHSRTRPADWYESLMKAYVIDTVSADFYRAISRYVDAGTRDV
IEQIQASDETTEVLRERLRSALADDPRLASRLALWGRRLLGEALTQAQRVSYEHAFLGSLIGGED
SAAAKELVSGLIAGLAEKHSKRMTQLGLTG
>4DOGA
GSHMNSVTVSHAPYTITYHNDWEPVMSQLVEFYNEVASWLLRDETSPIPDKFFIQLKQPLRNKRV
CVCGIDPYPKDGTGVPFESPNFTKKSIKEIASSISRLTGVIDYKGYNLNIIDGVIPWNYYLSCKL
GETKSHAIYWDKISKLLLQHITKHVSVLYCLGKTDFSNIRAKLESPVTTIVGYHPAARDRQFEKD
RSFEIINVLLELDNKAPINWAQGFIY
>2XTCA

MSITSDEVNFLVYRYLQESGFSHSAFTFGIESHISQSNINGTLVPPAALISILQKGLQYVEAEIS
INEDGTVFDGRPIESLSLIDAVMPD
>2I1SA
MKKTFEKVYHLKLSIKGITPQIWRRIQVPENYTFLDLHKAIQAVMDWEDYHLHEFEMVNPKTGML
DKIGAEGDDFDAFGGPLVSEKKAKLSDYFTLENKEALYTYDFGDNWQVKVRLEKILPRKEGVEYP
ICTAGKRAAVPEDSGGVWGYEEMLEVLKDSEHEEYEDTVLWLGDDFDPEYFDPKDVSF
>3PVHA
SASEFNILNDGPPKETYVVDDAGVLSRVTKSDLKKLLSDLEYRKKLRLNFITVRKLTSKADAFEY
ADQVLEKWYPSIEEGNNKGIVVLITSQKEGAITGGPAFIEAVGENILDATVSENLPVLATDEKYN
EAVYSSAKRLVAAIDGQPDPGGP
>1TIQA
MSVKMKKCSREDLQTLQQLSIETFNDTFKEQNSPENMKAYLESAFNTEQLEKELSNMSSQFFFIY
FDHEIAGYVKVNIDDAQSEEMGAESLEIERIYIKNSFQKHGLGKHLLNKAIEIALERNKKNIWLG
VWEKNENAIAFYKKMGFVQTGAHSFYMGDEEQTDLIMAKTLILEHHHHHH
>3BRVA
AMAPAKKSEELVAEAHNLCTLLENAIQDTVREQDQSFTALDWSWLQTE
>4KVHA
SNAMDRVATARAYYRALDEHDYDLLSDVLAPDFVHDRPDRTIEGRERFVRFMREERPQTDTSHPI
ATIYTGASTVAVEGRLLNSDGAEITQFVDVFAFEDGVIGRIRTHTPEP
>4JERA
MSTTIQYNSNYADYSISSYLREWANNFGDIDQAPAETKDRGSFSGSSTLFSGTQYAIGSSHSNPE
GMIAEGDLKYSFMPQHTFHGQIDTLQFGKDLATNAGGPSAGKHLEKIDITFNELDLSGEFDSGKS
MTENHQGDMHKSVRGLMKGNPDPMLEVMKAKGINVDTAFKDLSIASQYPDSGYMSDAPMVDTV
>3BA3A
GMDISLLKQVVQSTNKIALSTAVNNEADVKIVNFVWYEAQPDTLYFSSVKTSPALKVYDQNPDIA
FITIPNDGTAGNPYLRAQHVKLQRSTKTMTDLLPQYLETVPNYQQVWDAIGSTLVVFELKLTDLF
VDAGVGGEKQTLTFN
>2ZAHA
NISYTEGAKPGAISAPVAISRRVAGMKPRFVRSEGSVKIVHREFIASVLPSNDLTVNNGDVNIGK
YRVNPSNNALFTWLQGQAQLYDMYRFTRLRFTYIPTTGSTSTGRVSILWDRDSQDPLPIDRAAIS
SYAHYADSAPWAENVLVVPCDNTWRYMNDTNAVDRKLVDFGQFLFATYSGAGATAHGDLYVEYAV
EFKDPQPIAGMVCMFDRLVSFSEVGSTIKGVNYIADRDVITTGGNIGVNINIPGTYLVTIVLNAT
SIGSLTFTGNSKLVGNSLNVTSSGASALTFTLNSTGVPNSSNSSFSVGTVVALTRVRMTITRCSP
ETAYLA
>1JQ5A
MAAERVFISPAKYVQGKNVITKIANYLEGIGNKTVVIADEIVWKIAGHTIVNELKKGNIAAEEVV
FSGEASRNEVERIANIARKAEAAIVIGVGGGKTLDTAKAVADELDAYIVIVPTAASTDAPTSALS
VIYSDDGVFESYRFYKKNPDLVLVDTKIIANAPPRLLASGIADALATWVEARSVIKSGGKTMAGG
IPTIAAEAIAEKCEQTLFKYGKLAYESVKAKVVTPALEAVVEANTLLSGLGFESGGLAAAHAIHN
GFTALEGEIHHLTHGEKVAFGTLVQLALEEHSQQEIERYIELYLCLDLPVTLEDIKLKDASREDI
LKVAKAATAEGETIHNAFNVTADDVADAIFAADQYAKAYKEKHRK
>1P9YA
GSHMQVSVETTQGLGRRVTITIAADSIETAVKSELVNVAKKVRIDGLRKGKVPMNIVAQRYGASV
RQDVLGDLMSRNFIDAIIKEKINPAGAPTYVPGEYKLGEDFTYSVEFEVYPEVELQ

>3EYEA
MSLSSPNILLTRIDNRLVHGQVGVTWTSTIGANLLVVVDDVVANDDIQQKLMGITAETYGFGIRF
FTIEKTINVIGKAAPHQKIFLICRTPQTVRKLVEGGIDLKDVNVGNMHFSEGKKQISSKVYVDDQ
DLTDLRFIKQRGVNVFIQDVPGDQKEQIPDEGHHHHHH
>3RKCA
SRPFSVLRANDVLWLSLTAAEYDQTTYGSSTNPMYVSDTVTFVNVATGAQGVSRSLDWSKVTLDG
RPLTTIQQYSKTFFVLPLRGKLSFWEAGTTKAGYPYNYNTTASDQILIENAPGHRVCISTYTTNL
GSGPVSISAVGVLAPHSA
>3DUZA
AEHCNAQMKTGPYKIKNLDITPPKETLQKDVEITIVETDYNENVIIGYKGYYQAYAYNGGSLDPN
TRVEETMKTLNVGKEDLLMWSIRQQCEVGEELIDRWGSDSDDCFRDNEGRGQWVKGKELVKRQNN
NHFAHHTCNKSWRCGISTSKMYSRLECQDDTDECQVYILDAEGNPINVTVDTVLHRDGVSMILKQ
KSTFTTRQIKAACLLIKDDKNNPESVTREHCLIDNDIYDLSKNTWNCKFNRCIKRKVEHRVKKRP
PTWRHNVRAKYTEGDTATKGDLMHIQEELMYENDLLKMNIELMHAHINKLNNMLHDLIVSVAKVD
ERLIGNLMNNSVSSTFLSDDTFLLMPCTNPPAHTSNCYNNSIYKEGRWVANTDSSQCIDFSNYKE
LAIDDDVEFWIPTIGNTTYHDSWKDASGWSFIAQQKSNLITTMENTKFGGVGTSLSDITSMAEGE
LAAKLTSFMFGHVVNFVIILIVILDYKDDDDK
>3LZQA
GGEVPIGDPKELNGMEIAAVYLQPIEMEPRGIDLAASLADIHLEADIHALKNNPNGFPEGFWMPY
LTIAYELKNTDTGAIKRGTLMPMVADDGPHYGANIAMEKDKKGGFGVGNYELTFYISNPEKQGFG
RHVDEETGVGKWFEPFKVDYKFKYTGTPK
>2GKPA
SNAMTFNQEQDYWAGYKANERALIIQTWSGFGRYAPDHLYPPHILPLDTDNETLGTTVLQALANS
RTFVYDSPEDQDFFDTEKIRQRYEDWVAKLCGNLGYKTRRALFKNMMSVDIWLHNGCLKISPSRH
VKLEAWDAIDADDVILSLDNSPEEIGAGLKLALSRCR
>3FO8D
AVDRDTAKNSSPIAGNIEYTISTPGSNYAVGDKITVKYVSDDIETEGKITEVDADGKIKKINIPT
AKIIAKAKEVGEYPTLGSNWTAEISSSSSGLAAVITLGKIITDSGILLAEIENAEAAMTAVDFQA
NLKKYGIPGVVALYPGELGDKIEIEIVSKADYAKGASALLPIYPGGGTRASTAKAVFGYGPQTDS
QYAIIVRRNDAIVQSVVLSTKRGGKDIYDSNIYIDDFFAKGGSEYIFATAQNWPEGFSGILTLSG
GLSSNAEVTAGDLMEAWDFFADR
>3CL6A
MSVDYPRDLIGYGSNPPHPHWPGKARIALSFVLNYEEGGERNILHGDKESEAFLSEMVSAQPLQG
ERNMSMESLYEYGSRAGVWRILKLFKAFDIPLTIFAVAMAAQRHPDVIRAMVAAGHEICSHGYRW
IDYQYMDEAQEREHMLEAIRILTELTGERPLGWYTGRTGPNTRRLVMEEGGFLYDCDTYDDDLPY
WEPNNPTGKPHLVIPYTLDTNDMRFTQVQGFNKGDDFFEYLKDAFDVLYAEGAEAPKMLSIGLHC
RLIGRPARLAALQRFIEYAKSHEQVWFTRRVDIARHWHATHPYTGAAK
>1Q5YA
GTQGFAVLSYVYEHEKRDLASRIVSTQHHHHDLSVATLHVHINHDDCLEIAVLKGDMGDVQHFAD
DVIAQRGVRHGHLQCLPKED
>2XWSA
GMRRGLVIVGHGSQLNHYREVMELHRKRIEESGAFDEVKIAFAARKRRPMPDEAIREMNCDIIYV
VPLFISYGLHVTEDLPDLLGFPRGRGIKEGEFEGKKVVICEPIGEDYFVTYAILNSVFRIGRDGK
GEE

>2VBKA
DPDQFGPDLIEQLAQSGKYSQDNTKGDAMIGVKQPLPKAVLRTQHDKNKEAISILDFGVIDDGVT
DNYQAIQNAIDAVASLPSGGELFIPASNQAVGYIVGSTLLIPGGVNIRGVGKASQLRAKSGLTGS
VLRLSYDSDTIGRYLRNIRVTGNNTCNGIDTNITAEDSVIRQVYGWVFDNVMVNEVETAYLMQGL
WHSKFIACQAGTCRVGLHFLGQCVSVSVSSCHFSRGNYSADESFGIRIQPQTYAWSSEAVRSEAI
ILDSETMCIGFKNAVYVHDCLDLHMEQLDLDYCGSTGVVIENVNGGFSFSNSWIAADADGTEQFT
GIYFRTPTSTQSHKIVSGVHINTANKNTAANNQSIAIEQSAIFVFVSGCTLTGDEWAVNIVDINE
CVSFDKCIFNKPLRYLRSGGVSVTDCYLAGITEVQKPEGRYNTYRGCSGVPSVNGIINVPVAVGA
TSGSAAIPNPGNLTYRVRSLFGDPASSGDKVSVSGVTINVTRPSPVGVALPSMVEYLAI
>2X3MA
GMSAFDEFNEGFGLDVSDTPEELAFETESAIEEIESETSPGDQPKGSEPEEIRVWAEEKARKAVE
EGREVTNWADWIMGWRTPNASEKKMEFMYWYTRTYLEEAKDIRPDIADALARGMAGLAFGRTDWV
ASMLDPQIMRHIYTDPEVARIYSETRDMLRRVSDYYISLTTMELGKVADIIAEAKAKGENPEVVA
REIAEAVPRLSPKSLYFNLYYIGRSIGDNYVLEVARVLSKMRRR
>3H8DE
GSSSGGGSSSSGTSSAFSSYFNNKVGIPQEHVDHDDFDANQLLNKINE
>2P8IA
GMTFRDTSAIASWHAHVYFDASSRDAAWTLREQIEAHWSGKLQLGRFHERPVGPHPMWSYQLAFT
QEQFADLVGWLTLNHGALDIFLHPNTGDALRDHRDAAVWIGHSHELVLSALN
>2IMZA
CLAEGTRIFDPVTGTTHRIEDVVDGRKPIHVVAAAKDGTLHARPVVSWFDQGTRDVIGLRIAGGA
ILWATPDHKVLTEYGWRAAGELRKGDRVAQPRRFDGFGDSAPIPARVQALADALDDKFLHDMLAE
ELRYSVIREVLPTRRARTFDLEVEELHTLVAEGVVVHN
>2IG8A
MTAVRRIRAAALPDLPDASWSNALLVGEELVMSGMTAHPATRQAAERGAALDAHAQALVVLGKVK
ALLEAAGGHVGNLYKLNVYVTRIADKDAIGRARQEFFAGQGTFPASTLVEVSGLVFPELLVEIDA
WARLDIDLANCDEA
>3K5JA
GMDYNQTVLSHLQKFWKHHDIKGFTWTLGRIVEELPDFQVFQVIPNHEDEPWVYVSSGIGQFLGQ
EFFIISPFETPEHIETLAMLASASMHYPDQFQLGKTVNIGRPWVEQSSFRHFLISLPYPYGQELE
YMDNVRFFWLLPITQTERLFLNTHSVEELETKFDEAGIDYLDINRASTVWQAG
>2DY0A
GSSGSSGMTATAQQLEYLKNSIKSIQDYPKPGILFRDVTSLLEDPKAYALSIDLLVERYKNAGIT
KVVGTEARGFLFGAPVALGLGVGFVPVRKPGKLPRETISETYDLEYGTDQLEIHVDAIKPGDKVL
VVDDLLATGGTIEATVKLIRRLGGEVADAAFIINLFDLGGEQRLEKQGITSYSLVPFPGH
>3SNYA
MPTKAVTFYEDINYGGASVSLQPGNYTLSQLNTAKIPNDWMTSLKVPSGWTVDVYENDNFTGTKW
TYTSDTPWVGNDANDKMRSVKIYSTTNTGGDT
>3MAHA
SLDTEKDCIKAVAAKDGITVIKVKSSNKLLSWHFMRKLFEIFEFYQEPVDMVATSEVGVSLTIDN
DKNLPDIVRALSDIGDVTVDKDMVIICIVGDMEWDNVGFEARIINALKGVPVRMISYGGSNYNVS
VLVKAEDKKKALIALSNKLFNSRATKA
>4AQOA
GGTISNNKAPIAKVTGPSTGAVGRNIEFSGKDSKDEDGKIVSYDWDFGDGATSRGKNSVHAYKKA

GTYNVTLKVTDDKGATATESFTIEIKN
>2ZBLA
MKWFNTLSHNRWLEQETDRIFNFGKNAVVPTGFGWLGNKGQIKEEMGTHLWITARMLHVYSVAAS
MGRPGAYDLVDHGIKAMNGALRDKKYGGWYACVNDQGVVDASKQGYQHFFALLGAASAVTTGHPE
ARKLLDYTIEVIEKYFWSEEEQMCLESWDEAFSQTEDYRGGNANMHAVEAFLIVYDVTHDKKWLD
RALRIASVIIHDVARNGDYRVNEHFDSQWNPIRDYNKDNPAHRFRAYGGTPGAWIEWGRLMLHLH
AALEARFETPPAWLLEDAKGLFHATIRDAWAPDGADGFVYSVDWDGKPIVRERVRWPIVEAMGTA
YALYTLTDDSQYEEWYQKWWDYCIKYLMDYENGSWWQELDADNKVTTKVWDGKQDIYHLLHCLVI
PRLPLAPGLAPAVAAGLLDINAKLEHHHHHH
>2CDUA
MKVIVVGCTHAGTFAVKQTIADHPDADVTAYEMNDNISFLSCGIALYLGKEIKNNDPRGLFYSSP
EELSNLGANVQMRHQVTNVDPETKTIKVKDLITNEEKTEAYDKLIMTTGSKPTVPPIPGIDSSRV
YLCKNYNDAKKLFEEAPKAKTITIIGSGYIGAELAEAYSNQNYNVNLIDGHERVLYKYFDKEFTD
ILAKDYEAHGVNLVLGSKVAAFEEVDDEIITKTLDGKEIKSDIAILCIGFRPNTELLKGKVAMLD
NGAIITDEYMHSSNRDIFAAGDSAAVHYNPTNSNAYIPLATNAVRQGRLVGLNLTEDKVKDMGTQ
SSSGLKLYGRTYVSTGINTALAKANNLKVSEVIIADNYRPEFMLSTDEVLMSLVYDPKTRVILGG
ALSSMHDVSQSANVLSVCIQNKNTIDDLAMVDMLFQPQFDRPFNYLNILGQAAQAQADKAHK
>1XE7A
MSANVQEAANAAIEPASFVKVPMPEPPSSLQQLINDWQLIKHREGGYFKETDRSPYTMEVEKPVN
GGSGNTEMVTRNQSTLIYYLLTPDSPIGKFHKNINRIIHILQRGKGQYVLVYPDGQVKSFKVGFD
YKNGEVSQWVVPGGVFKASFLLPNEEFDNGFLISEVVVPGFDFEDHTFLKGEDELKHLVGPEKAA
ELAFLAHH
>2EFJA
MELQEVLHMNGGEGDTSYAKNSSYNLFLIRVKPVLEQCIQELLRANLPNINKCFKVGDLGCASGP
NTFSTVRDIVQSIDKVGQEKKNELERPTIQIFLNDLFQNDFNSVFKLLPSFYRNLEKENGRKIGS
CLIGAMPGSFYSRLFPEESMHFLHSCYCLHWLSQVPSGLVTELGISVNKGCIYSSKASRPPIQKA
YLDQFTKDFTTFLRIHSEELISRGRMLLTFICKEDEFDHPNSMDLLEMSINDLVIEGHLEEEKLD
SFNVPIYAPSTEEVKRIVEEEGSFEILYLETFNAPYDAGFSIDDDYQGRSHSPVSCDEHARAAHV
ASVVRSIYEPILASHFGEAILPDLSHRIAKNAAKVLRSGKGFYDSVIISLAKKPEKADM
>3GWRA
GMSEPVFPTPEAAEDAFYAAFEARSLDDMMAVWARDDHVACIHPLAAPLNGRAAVAAGWRSMFGA
AGRFRLQVKAVHEIRQADHVIRIVDEFLTIGDETAPRPAILATNVYRREADGWRMVLHHASPLQV
GAKAGADTPPVVFH
>1K04A
LSSPADSYNEGVKLQPQEISPPPTANLDRSNDKVYENVTGLVKAVIEMSSKIQPAPPEEYVPMVK
EVGLALRTLLATVDETIPLLPASTHREIEMAQKLLNSDLGELINKMKLAQQYVMTSLQQEYKKQM
LTAAHALAVDAKNLLDVIDQARLKMLGQTRPH
>3E4WA
MHHHHHHMSGGLTPDQAIDAIRGTGGAQPGCRALHAKGTLYRGTFTATRDAVMLSAAPHLDGSTV
PALIRFSNGSGNPKQRDGAPGVRGMAVKFTLPDGSTTDVSAQTARLLVSSTPEGFIDLLKAMRPG
LTTPLRLATHLLTHPRLLGALPLLREANRIPASYATTEYHGLHAFRWIAADGSARFVRYHLVPTA
AEEYLSASDARGKDPDFLTDELAARLQDGPVRFDFRVQIAGPTDSTVDPSSAWQSTQIVTVGTVT
ITGPDTEREHGGDIVVFDPMRVTDGIEPSDDPVLRFRTLVYSASVKLRTGVDRGAQAPPV
>3FCNA

GMGMEHKTYEADLFVWCQQQADGLRALSRSRRDLPDDLDLEHIAEEIEDMGRSELREATSLVRQI
CVRVIMAMSAPEAPDRARWRSEVVSWHNLLLDTITPGMIDRIDIGVIWRRAVSEAKAALIEINVA
PQAGLSFQAPLPADHFLDEDFDYDATVARLGPTA
>3LFTA
SNAKIGVLQFVSHPSLDLIYKGIQDGLAEEGYKDDQVKIDFMNSEGDQSKVATMSKQLVANGNDL
VVGIATPAAQGLASATKDLPVIMAAITDPIGANLVKDLKKPGGNVTGVSDHNPAQQQVELIKALT
PNVKTIGALYSSSEDNSKTQVEEFKAYAEKAGLTVETFAVPSTNEIASTVTVMTSKVDAIWVPID
NTIASGFPTVVSSNQSSKKPIYPSATAMVEVGGLASVVIDQHDLGVATGKMIVQVLKGAKPADTP
VNVFSTGKSVINKKIAQELGITIPESVLKEAGQVI
>3N17A
ANNLGSKLLVGYWHNFDNGTGIIKLKDVSPKWDVINVSFGETGGDRSTVEFSPVYGTDADFKSDI
SYLKSKGKKVVLSIGGQNGVVLLPDNAAKDRFINSIQSLIDKYGFDGIDIDLQSGIYLNGNDTNF
KNPTTPQIVNLISAIRTISDHYGPDFLLSMAPETAYVQGGYSAYGSIWGAYLPIIYGVKDKLTYI
HVQHFNAGSGIGMDGNNYNQGTADYEVAMADMLLHGFPVGGNANNIFPALRSDQVMIGLPAAPAA
APSGGYISPTEMKKALNYIIKGVPFGGKYKLSNQSGYPAFRGLMSWSINWDAKNNFEFSNNYRTY
FDGLSLQK
>3CVOA
GMDDQSGDQMRPELTMPPAEAEALRMAYEEAEVILEYGSGGSTVVAAELPGKHVTSVESDRAWAR
MMKAWLAANPPAEGTEVNIVWTDIGPTGDWGHPVSDAKWRSYPDYPLAVWRTEGFRHPDVVLVDG
RFRVGCALATAFSITRPVTLLFDDYSQRRWQHQVEEFLGAPLMIGRLAAFQVEPQPIPPGSLMQL
IRTMTSP
>4KEFA
GSRSGVAVADESLTAFNDLKLGKKYKFILFGLNDAKTEIVVKETSTDPSYDAFLEKLPENDCLYA
IYDFEYEINGNEGKRSDIVFFTWSPDTAPVRSKMVYASSKDALRRALNGVSTDVQGTDFSEVSYD
SVLERVSRGAGSH
>2Q4XA
MEKRGVIDTWIDKHRSIYTAATRHAFVVSIRDGSVDLSSFRTWLGQDYLFVRRFVPFVASVLIRA
CKDSGESSDMEVVLGGIASLNDEIEWFKREGSKWDVDFSTVVPQRANQEYGRFLEDLMSSEVKYP
VIMTAFWAIEAVYQESFAHCLEDGNKTPVELTGACHRWGNDGFKQYCSSVKNIAERCLENASGEV
LGEAEDVLVRVLELEVAFWEMSRGGQ
>1MUWA
SYQPTPEDRFTFGLWTVGWQGRDPFGDATRPALDPVETVQRLAELGAHGVTFHDDDLIPFGSSDT
ERESHIKRFRQALDATGMTVPMATTNLFTHPVFKDGGFTANDRDVRRYALRKTIRNIDLAVELGA
KTYVAWGGREGAESGAAKDVRVALDRMKEAFDLLGEYVTSQGYDIRFAIEPKPNEPRGDILLPTV
GHALAFIERLERPELYGVNPEVGHEQMAGLNFPHGIAQALWAGKLFHIDLNGQSGIKYDQDLRFG
AGDLRAAFWLVDLLESAGYEGPRHFDFKPPRTEDIDGVWASAAGCMRNYLILKERAAAFRADPEV
QEALRASRLDELAQPTAADGVQELLADRTAFEDFDVDAAAARGMAFERLDQLAMDHLLGAR
>3KS6A
GMTRIASHRGGTLEFGDSTPHGFTATAAMALEEVEFDLHPTADGAIVVHHDPTLDATTDMTGAIV
DMTLAKVKTATIRYGAGSHPMTLEELCALYVDSHVNFRCEIKPGVDGLPYEGFVALVIAGLERHS
MLERTTFSSFLLASMDELWKATTRPRLWLVSPSVLQQLGPGAVIETAIAHSIHEIGVHIDTADAG
LMAQVQAAGLDFGCWAAHTPSQITKALDLGVKVFTTDRPTLAIALRTEHRMEASV
>3DXPA
GMSSNVSHFEGTRPVADQQRFDTEALEAWMRQHVEGFAGPLSVEQFKGGQSNPTFKLVTPGQTYV

MRAKPGPKSKLLPSAHAIEREYRVMDALAGTDVPVAKMYALCEDESVIGRAFYIMEFVSGRVLWD
QSLPGMSPAERTAIYDEMNRVIAAMHTVDYQAIGLGDYGKPGNYFQRQIERWTKQYKLSETESIP
AMDSLMDWLPQHIPQEDADLTSIVHGDYRLDNLMFHPTEPRVLAVLDWELSTLGHPMGDFGYHCM
SWHIAPGQFRGIAGLDHAALGIPDEASYRKLYEQRTGRPITGDWNFYLAFSMFRIAGILQGIMKR
VVDGTASSAQALDAGKRARPMAEMGWEYAKKAKQ
>1XQAA
AMGIKHLNLTVADVVAAREFLEKYFGLTCSGTRGNAFAVMRDNDGFILTLMKGKEVQYPKTFHVG
FPQESEEQVDKINQRLKEDGFLVEPPKHAHAYTFYVEAPGGFTIEVMC
>2PFZA
TKWDLPTAYPASNLHVENLTQFVKDVDSLSGGKLKITLHNNASLYKAPEIKRAVQGNQAQIGEIL
LTNFANEDPVYELDGLPFLATGYDASFKLYQAQKPFLEKKLASQGMMLLYSVAWPPQGIFANRDI
KQVSDMKGLKWRAYSPVTAKIAELVGAQPVTVQQAELAQAMATGVIDSYMSSGSTGFDTKTYEYI
KKFYDTEAWLPKNAVLVNKKAFDALDPATQQALKKAGAQAEERGWKLSQEKNSWYKEQLAKNGMA
IIAPTAELKSGLTEVGKRMLDDWLKKAGADGQAMIDAYRKQ
>2VHLA
MAESLLIKDIAIVTENEVIKNGYVGINDGKISTVSTERPKEPYSKEIQAPADSVLLPGMIDIHIH
GGYGADTMDASFSTLDIMSSRLPEEGTTSFLATTITQEHGNISQALVNAREWKAAEESSLLGAEL
LGIHLEGPFVSPKRAGAQPKEWIRPSDVELFKKWQQEAGGLIKIVTLAPEEDQHFELIRHLKDES
IIASMGHTDADSALLSDAAKAGASHMTHLYNAMSPFHHREPGVIGTALAHDGFVTELIADGIHSH
PLAAKLAFLAKGSSKLILITDSMRAKGLKDGVYEFGGQSVTVRGRTALLSDGTLAGSILKMNEGA
RHMREFTNCSWTDIANITSENAAKQLGIFDRKGSVTVGKDADLVIVSSDCEVILTICRGNIAFIS
KEADQI
>3C8LA
GMARKRLIIEMGMGIDQHGQEPTIAASRAVRNAIAHNALPGVWEVAGLSHPNEMIIEVQVAVPYP
EQVREEEVLAVLPFGRKTLTVESGGMIVQGRAIPELNDKNDEMLIAIAAVTVLIENE
>4F11A
WARGAPRPPPSSPPLSIMGLMPLTKEVAKGSIGRGVLPAVELAIEQIRNESLLRPYFLDLRLYDT
ECDNAKGLKAFYDAIKYGPNHLMVFGGVCPSVTSIIAESLQGWNLVQLSFAATTPVLADKKKYPY
FFRTVPSDNAVNPAILKLLKHYQWKRVGTLTQDVQRFSEVRNDLTGVLYGEDIEISDTESFSNDP
CTSVKKLKGNDVRIILGQFDQNMAAKVFCCAYEENMYGSKYQWIIPGWYEPSWWEQVHTEANSSR
CLRKNLLAAMEGYIGVDFEPLSSKQIKTISGKTPQQYEREYNNKRSGVGPSKFHGYAYDGIWVIA
KTLQRAMETLHASSRHQRIQDFNYTDHTLGRIILNAMNETNFFGVTGQVVFRNGERMGTIKFTQF
QDSREVKVGEYNAVADTLEIINDTIRFQGSEPPKDDYKDDDDK
>4G68A
MAHHHHHHVDDDDKMCSSNNLSKSNTSNSSKTSSSSKKMCSSNNLSKSNTSNSSKTSSSSKKITL
TFWNLFTGEPAKTKVKEIIDQWNKENPNVQIVESVTENDAYKTKIKAAIAANEAPDIFQTWAGGF
SQPFVEAGKVLQLDSYLNDGTKDQLLPGSFDNVTYNGKIYGIPFDQQASVLYINKELFDKYNVKV
PTTFSELIDAIKTFKSKGVTPFALGEKDEWPGMWYYDMIALREGGVQLTRDALNGKASFDNQAFT
DAAQKLQDMVNAGAFDSGFMGLTRDEATAEFNQGKAAMYFGGNFDAAAFVSDPSSLVKGKIEAVR
FPTIEGGKGDPTEYIGGTVGALMVSANSKYKDEAVRAAKYLAKQLSDMDYLIATGLPAWKYDNID
QSKVDPLEIQIMNNIVANAKGSVPAWDIYLSGDAAQTHKDLVAQLFAKQITPEEYSKQMQQKING
K
>2EB4A
MFDKHTHTLIAQRLDQAEKQREQIRAISLDYPEITIEDAYAVQREWVRLKIAEGRTLKGHKIGLT

SKAMQASSQISEPDYGALLDDMFFHDGSDIPTDRFIVPRIEVELAFVLAKPLRGPNCTLFDVYNA
TDYVIPALELIDARCHNIDPETQRPRKVFDTISDNAANAGVILGGRPIKPDELDLRWISALMYRN
GVIEETGVAAGVLNHPANGVAWLANKLAPYDVQLEAGQIILGGSFTRPVPARKGDTFHVDYGNMG
SISCRFV
>4A0DA
GSSPDKKWLGTPIEEMRRMPRCGIRLPLLRPSANHTVTIRVDLLRAGEVPKPFPTHYKDLWDNKH
VKMPCSEQNLYPVEDENGERTAGSRWELIQTALLNKFTRPQNLKDAILKYNVAYSKKWDFTALID
FWDKVLEEAEAQHLYQSILPDMVKIALCLPNICTQPIPLLAAAMNHSITMSQEQIASLLANAFFC
TFPRRNAKMKSEYSSYPDINFNRLFEGRSSRKPEKLKTLFCYFRRVTAAAPTGLVTFTRQSLEDF
PEWERCEKPLTRLHVTYEGTIEENGQGMLQVDFANRFVGGGVTSAGLVQEEIRFLINPELIISRL
FTEVLDHNECLIITGTEQYSEYTGYAETYRWSRSHEDGSERDDWQRRCTEIVAIDALHFRRYLDQ
FVPEKMRRELNKAYCGFLRPGVSSENLSAVATGNWGCGAFGGDARLKALIQILAAAAAERDVVYF
TFGDSELMRDIYSMHIFLTERKLTVGDVYKLLLRYYNEECRNCSTPGPDIKLYPFIYHAVESCAE
TADHSGQRTGT
>2R19A
AVTGDTDQPIHIESDQQSLDMQGNVVTFTGNVIVTQGTIKINADKVVVTRPGGEQGKEVIDGYGK
PATFYQMQDNGKPVEGHASQMHYELAKDFVVLTGNAYLQQVDSNIKGDKITYLVKEQKMQAFSDK
GKRVTTVLVPSQLQDKNNKGQTPAQKKGN
>2O3OA
GSHMATGKEYEVIKNDVEHDMKADHITYEGLNKEATEGYRITANQKSFSKEEIEALKDQKPLMDM
PSDDHKVTSLKMKFANPIALSKKDIEDDAQALVSSKIQDGEKYKLWKVDKSKKEIIFFQTYEGHY
IYQKTDNPSNMIGQVVLHLNGKNEVVSYDQTTLETFKQIQKESLITEMDAVELLYYQNQLKEYST
VKSCKFGYVAQYPLTSTQVLAPVWRITVEYEKKVNGEKKTVQEYFTVNALESTILDTDQ
>1GG4A
MISVTLSQLTDILNGELQGADITLDAVTTDTRKLTPGCLFVALKGERFDAHDFADQAKAGGAGAL
LVSRPLDIDLPQLIVKDTRLAFGELAAWVRQQVPARVVALTGSSGKTSVKEMTAAILSQCGNTLY
TAGNLNNDIGVPMTLLRLTPEYDYAVIELGANHQGEIAWTVSLTRPEAALVNNLAAAHLEGFGSL
AGVAKAKGEIFSGLPENGIAIMNADNNDWLNWQSVIGSRKVWRFSPNAANSDFTATNIHVTSHGT
EFTLQTPTGSVDVLLPLPGRHNIANALAAAALSMSVGATLDAIKAGLANLKAVPGRLFPIQLAEN
QLLLDDSYNANVGSMTAAVQVLAEMPGYRVLVVGDMAELGAESEACHVQVGEAAKAAGIDRVLSV
GKQSHAISTASGVGEHFADKTALITRLKLLIAEQQVITILVKGSRSAAMEEVVRALQENGTC
>4GYXA
GPPGPPGPRGQPGVMGFPGPPGPPGPCCGGV
>1OAOC
MTDFDKIFEGAIPEGKEPVALFREVYHGAITATSYAEILLNQAIRTYGPDHPVGYPDTAYYLPVI
RCFSGEEVKKLGDLPPILNRKRAQVSPVLNFENARLAGEATWYAAEIIEALRYLKYKPDEPLLPP
PWTGFIGDPVVRRFGIKMVDWTIPGEAIILGRAKDSKALAKIVKELMGMGFMLFICDEAVEQLLE
ENVKLGIDYIAYPLGNFTQIVHAANYALRAGMMFGGVTPGAREEQRDYQRRRIRAFVLYLGEHDM
VKTAAAFGAIFTGFPVITDQPLPEDKQIPDWFFSVEDYDKIVQIAMETRGIKLTKIKLDLPINFG
PAFEGESIRKGDMYVEMGGNRTPAFELVRTVSESEITDGKIEVIGPDIDQIPEGSKLPLGILVDI
YGRKMQADFEGVLERRIHDFINYGEGLWHTGQRNINWLRVSKDAVAKGFRFKNYGEILVAKMKEE
FPAIVDRVQVTIFTDEAKVKEYMEVAREKYKERDDRMRGLTDETVDTFYSCVLCQSFAPNHVCIV
TPERVGLCGAVSWLDAKASYEINHAGPNQPIPKEGEIDPIKGIWKSVNDYLYTASNRNLEQVCLY
TLMENPMTSCGCFEAIMAILPECNGIMITTRDHAGMTPSGMTFSTLAGMIGGGTQTPGFMGIGRT

YIVSKKFISADGGIARIVWMPKSLKDFLHDEFVRRSVEEGLGEDFIDKIADETIGTTVDEILPYL
EEKGHPALTMDPIM
>4A5SA
SRKTYTLTDYLKNTYRLKLYSLRWISDHEYLYKQENNILVFNAEYGNSSVFLENSTFDEFGHSIN
DYSISPDGQFILLEYNYVKQWRHSYTASYDIYDLNKRQLITEERIPNNTQWVTWSPVGHKLAYVW
NNDIYVKIEPNLPSYRITWTGKEDIIYNGITDWVYEEEVFSAYSALWWSPNGTFLAYAQFNDTEV
PLIEYSFYSDESLQYPKTVRVPYPKAGAVNPTVKFFVVNTDSLSSVTNATSIQITAPASMLIGDH
YLCDVTWATQERISLQWLRRIQNYSVMDICDYDESSGRWNCLVARQHIEMSTTGWVGRFRPSEPH
FTLDGNSFYKIISNEEGYRHICYFQIDKKDCTFITKGTWEVIGIEALTSDYLYYISNEYKGMPGG
RNLYKIQLIDYTKVTCLSCELNPERCQYYSVSFSKEAKYYQLRCSGPGLPLYTLHSSVNDKGLRV
LEDNSALDKMLQNVQMPSKKLDFIILNETKFWYQMILPPHFDKSKKYPLLLDVYAGPCSQKADTV
FRLNWATYLASTENIIVASFDGRGSGYQGDKIMHAINRRLGTFEVEDQIEAARQFSKMGFVDNKR
IAIWGWSYGGYVTSMVLGSGSGVFKCGIAVAPVSRWEYYDSVYTERYMGLPTPEDNLDHYRNSTV
MSRAENFKQVEYLLIHGTADDNVHFQQSAQISKALVDVGVDFQAMWYTDEDHGIASSTAHQHIYT
HMSHFIKQCFSLPAAASWSHPQFEK
>3T3LA
GTLGHPGSLDETTYERLAEETLDSLAEFFEDLADKPYTFEDYDVSFGSGVLTVKLGGDLGTYVIN
KQTPNKAIWLSSPSSGPKRYDWTGKNWVYSHDGVSLHELLAAELTKALKTKLDLSSLAYSGKDA
>1VF7A
AESSGKSEAPPPAQTPEVGIVTLEAQTVTLNTELPGRTNAFRIAEVRPQVNGIILKRLFKEGSDV
KAGQQLYQIDPATYEADYQSAQANLASTQEQAQRYKLLVADQAVSKQQYADANAAYLQSKAAVEQ
ARINLRYTKVLSPISGRIGRSAVTEGALVTNGQANAMATVQQLDPIYVDVTQPSTALLRLRRELA
SGQLERAGDNAAKVSLKLEDGSQYPLEGRLEFSEVSVDEGTGSVTIRAVFPNPNNELLPGMFVHA
QLQEGVKQKAILAPQQGVTRDLKGQATALVVNAQNKVELRVIKADRVIGDKWLVTEGLNAGDKII
TEGLQFVQPGVEVKTVPAKNVASAQKADAAPAKTDSKGHHHHHH
>1JMSA
NSSPSPVPGSQNVPAPAVKKISQYACQRRTTLNNYNQLFTDALDILAENDELRENEGSCLAFMRA
SSVLKSLPFPITSMKDTEGIPCLGDKVKSIIEGIIEDGESSEAKAVLNDERYKSFKLFTSVFGVG
LKTAEKWFRMGFRTLSKIQSDKSLRFTQMQKAGFLYYEDLVSCVNRPEAEAVSMLVKEAVVTFLP
DALVTMTGGFRRGKMTGHDVDFLITSPEATEDEEQQLLHKVTDFWKQQGLLLYCDILESTFEKFK
QPSRKVDALDHFQKCFLILKLDHGRVHSEKSGQQEGKGWKAIRVDLVMCPYDRRAFALLGWTGSR
QFERDLRRYATHERKMMLDNHALYDRTKRVFLEAESEEEIFAHLGLDYIEPWERNA
>3KLQA
GAKDSTVQTSISVENVLERAGDSTPFSVALESIDAMKTIEEITIAGSGKASFSPLTFTTVGQYTY
RVYQKPSQNKDYQADTTVFDVLVYVTYDEDGTLVAKVISRRAGDEEKSAITFKPKRLVKPIPPRQ
PDFPKTPLPLA
>2APLA
MKSTEKKELSHFRLKLETYLNEHFPEMSGNNPFITARSDEALTAYCDAVAQGFSHPEAESMASEV
LYQGLHFSRYDTLVSVLEREFEQELPSPLPERLAPILLKNKAIQSVFAKYDLTDDFEASPEYEHL
YTELTGTIVLLIESNHLPTIGGGNDTV
>4AMWA
GSTDNPDGIDYKTYDYVGVWGFSPLSNTNWFAAGSSTPGGITDWTATMNVNFDRIDNPSITVQHP
VQVQVTSYNNNSYRVRFNPDGPIRDVTRGPILKQQLDWIRTQELSEGCDPGMTFTSEGFLTFETK
DLSVIIYGNFKTRVTRKSDGKVIMENDEVGTASSGNKCRGLMFVDRLYGNAIASVNKNFRNDAVK

QEGFYGAGEVNCKYQDTYILERTGIAMTNYNYDNLNYNQWDLRPPHHDGALNPDYYIPMYYAAPW
LIVNGCAGTSEQYSYGWFMDNVSQSYMNTGDTTWNSGQEDLAYMGAQYGPFDQHFVYGAGGGMEC
VVTAFSLLQGKEFENQVLNKRSVMPPKYVFGFFQGVFGTSSLLRAHMPAGENNISVEEIVEGYQN
NNFPFEGLAVDVDMQDNLRVFTTKGEFWTANRVGTGGDPNNRSVFEWAHDKGLVCQTNITCFLRN
DNEGQDYEVNQTLRERQLYTKNDSLTGTDFGMTDDGPSDAYIGHLDYGGGVECDALFPDWGRPDV
AEWWGNNYKKLFSIGLDFVWQDMTVPAMMPHKIGDDINVKPDGNWPNADDPSNGQYNWKTYHPQV
LVTDMRYENHGREPMVTQRNIHAYTLCESTRKEGIVENADTLTKFRRSYIISRGGYIGNQHFGGM
WVGDNSTTSNYIQMMIANNINMNMSCLPLVGSDIGGFTSYDNENQRTPCTGDLMVRYVQAGCLLP
WFRNHYDRWIESKDHGKDYQELYMYPNEMDTLRKFVEFRYRWQEVLYTAMYQNAAFGKPIIKAAS
MYNNDSNVRRAQNDHFLLGGHDGYRILCAPVVWENSTERELYLPVLTQWYKFGPDFDTKPLEGAM
NGGDRIYNYPVPQSESPIFVREGAILPTRYTLNGENKSLNTYTDEDPLVFEVFPLGNNRADGMCY
LDDGGVTTNAEDNGKFSVVKVAAEQDGGTETITFTNDCYEYVFGGPFYVRVRGAQSPSNIHVSSG
AGSQDMKVSSATSRAALFNDGENGDFWVDQETDSLWLKLPNVVLPDAVITIT
>3ZRIA
MRGSHHHHHHTDPIRIELPTLIAKLNAQSKLALEQAASLCIERQHPEVTLEHYLDVLLDNPLSDV
RLVLKQAGLEVDQVKQAIASTYSREQVLDTYPAFSPLLVELLQEAWLLSSTELEQAELRSGAIFL
AALTRADRYLSFKLISLFEGINRENLKKHFAMILSDSAETT
>3BHDA
MGSSHHHHHHSSGLVPRGSMAQGLIEVERKFLPGPGTEERLQELGGTLEYRVTFRDTYYDTPELS
LMQADHWLRRREDSGWELKCPGAAGVLGPHTEYKELTAEPTIVAQLCKVLRADGLGAGDVAAVLG
PLGLQEVASFVTKRSAWKLVLLGADEEEPQLRVDLDTADFGYAVGEVEALVHEEAEVPTALEKIH
RLSSMLGVPAQETAPAKLIVYLQRFRPQDYQRLLEVNSS
>4DQAA
GNDLLEPKVYFESKEYNFSVEDEMDVMTFDLVSRLSSATSSQVDVSYSVAEPSVVDEYNAKYGTN
YEMLDVSQVKLSSTTSSISSGKLYADNIEVELSGLEALKAGNSYVLPMRVHSSSVSTLSGTNIAY
FFFSKPLKITKAGNFSNHYISVKFPVGTFFSSFTYEALINVDYFLDNNTIMGTEGVMILRIGDAG
GGITPKDYLEVAGGQNYRVTKPLLTNRWYHVALTYDQPTGKTGIYVNGEKWAGSDWGIDGFDPNS
DMGFYIGRIYGFKWGERPFHGKMSEVRVWSVARTENQLKQNMLGVDPASEGLALYYKLDGSETQE
GGVIKDATGRINGTTNGITIKTLDAPIAIN
>1TWDA
MALLEICCYSMECALTAQQNGADRVELCAAPKEGGLTPSLGVLKSVRQRVTIPVHPIIRPRGGDF
CYSDGEFAAILEDVRTVRELGFPGLVTGVLDVDGNVDMPRMEKIMAAAGPLAVTFHRAFDMCANP
LYTLNNLAELGIARVLTSGQKSDALQGLSKIMELIAHRDAPIIMAGAGVRAENLHHFLDAGVLEV
HSSAGAWQASPMRYRNQGLSMSSDEHADEYSRYIVDGAAVAEMKGIIERHQAKLEHHHHHH
>1RA0A
GSSMANNALQTIINARLPGEEGLWQIHLQDGKISAIDAQSGVMPITENSLDAEQGLVIPPFVEPH
IHLDTTQTAGQPNWNQSGTLFEGIERWAERKALLTHDDVKQRAWQTLKWQIANGIQHVRTHVDVS
DATLTALKAMLEVKQEVAPWIDLQIVAFPQEGILSYPNGEALLEEALRLGADVVGAIPHFEFTRE
YGVESLHKTFALAQKYDRLIDVHCDEIDDEQSRFVETVAALAHHEGMGARVTASHTTAMHSYNGA
YTSRLFRLLKMSGINFVANPLVNIHLQGRFDTYPKRRGITRVKEMLESGINVCFGHDGVFDPWYP
LGTANMLQVLHMGLHVCQLMGYGQINDGLNLITHHSARTLNLQDYGIAAGNSANLIILPAENGFD
ALRRQVPVRYSVRGGKVIASTQPAQTTVYLEQPEAIDYKR
>2VPAA
MSYYHHHHHHLESTSLYKKAGMSDFYDPRERDPSVSRRPQNRQSDEWIRELLLRGTIARVATLWQ

GEDGAAFPFITPLAYAYRPEQGDLVYHTNVVGRLRANAGQGHPATLEVSEIGQFLPSNSPLELSV
QYRSVMVFGTARVLAGEDARAALTTLSERVFPGLKVGETTRPISEDDLKRTSVYSLSIDRWSGKE
NWAEQAIQEEDWPALGPEWLG
>2HEWF
GSHMSSSPAKDPPIQRLRGAVTRCEDGQLFISSYKNEYQTMEVQNNSVVIKCDGLYIIYLKGSFF
QEVKIDLHFREDHNPISIPMLNDGRRIVFTVVASLAFKDKVYLTVNAPDTLCEHLQINDGELIVV
QLTPGYCAPEGSYHSTVNQVPL
>3C8NA
MGSSHHHHHHSSGLVPRGSHMAELKLGYKASAEQFAPRELVELAVAAEAHGMDSATVSDHFQPWR
HQGGHAPFSLSWMTAVGERTNRLLLGTSVLTPTFRYNPAVIAQAFATMGCLYPNRVFLGVGTGEA
LNEIATGYEGAWPEFKERFARLRESVGLMRQLWSGDRVDFDGDYYRLKGASIYDVPDGGVPVYIA
AGGPAVAKYAGRAGDGFICTSGKGEELYTEKLMPAVREGAAAADRSVDGIDKMIEIKISYDPDPE
LAMNNTRFWAPLSLTAEQKHSIDDPIEMEKAADALPIEQIAKRWIVASDPDEAVEKVGQYVTWGL
NHLVFHAPGHDQRRFLELFQSDLAPRLRRLG
>2MPRA
VDFHGYARSGIGWTGSGGEQQCFQATGAQSKYRLGNECETYAELKLGQEVWKEGDKSFYFDTNVA
YSVNQQNDWESTDPAFREANVQGKNLIEWLPGSTIWAGKRFYQRHDVHMIDFYYWDISGPGAGIE
NIDLGFGKLSLAATRSTEAGGSYTFSSQNIYDEVKDTANDVFDVRLAGLQTNPDGVLELGVDYGR
ANTTDGYKLADGASKDGWMFTAEHTQSMLKGYNKFVVQYATDAMTTQGKGQARGSDGSSSFTEEL
SDGTKINYANKVINNNGNMWRILDHGAISLGDKWDLMYVGMYQNIDWDNNLGTEWWTVGVRPMYK
WTPIMSTLLEVGYDNVKSQQTGDRNNQYKITLAQQWQAGDSIWSRPAIRIFATYAKWDEKWGYIK
DGDNISRYAAATNSGISTNSRGDSDEWTFGAQMEIWW
>3K3CA
GAMAAEMDWDKTVGAAEDVRRIFEHIPAILVGLEGPDHRFVAVNAAYRGFSPLLDTVGQPAREVY
PELEGQQIYEMLDRVYQTGEPQSGSEWRLQTDYDGSGVEERYFDFVVTPRRRADGSIEGVQLIVD
DVTSRVRARQAAEARVEELSERYRNVRD
>3LKMA
MGGHHHHHHGENLYFQGISSETGEMGILWEFDPIINKWIRLSMKLKVERKPFAEGALREAYHTVS
LGVGTDENYPLGTTTKLFPPIEMISPISKNNEAMTQLKNGTKFVLKLYKKEAEQQASRELYFEDV
KMQMVCRDWGNKFNQKKPPKKIEFLMSWVVELIDRSPSSNGQPILCSIEPLLVGEFKKNNSNYGA
VLTNRSTPQAFSHFTYELSNKQMIVVDIQGVDDLYTDPQIHTPDGKGFGLGNLGKAGINKFITTH
KCNAVCALLDLDVKLGGVLSGNNKKQLQQGTMVMPDILPELMPSDNT
>2XDGA
SMLREDESACLQAAEEMPQTTLGCPATWDGLLCWPTAGSGEWVTLPCPDFFSHFSSESGAVKRDC
TITGWSEPFPPYPVACPVPLELLAEEE
>1OF8A
MSESPMFAANGMPKVNQGAEEDVRILGYDPLASPALLQVQIPATPTSLETAKRGRREAIDIITGK
DDRVLVIVGPCSIHDLEAAQEYALRLKKLSDELKGDLSIIMRAYLEKPRTTVGWKGLINDPDVNN
TFNINKGLQSARQLFVNLTNIGLPIGSEMLDTISPQYLADLVSFGAIGARTTESQLHRELASGLS
FPVGFKNGTDGTLNVAVDACQAAAHSHHFMGVTKHGVAAITTTKGNEHCFVILRGGKKGTNYDAK
SVAEAKAQLPAGSNGLMIDYSHGNSNKDFRNQPKVNDVVCEQIANGENAITGVMIESNINEGNQG
IPAEGKAGLKYGVSITDACIGWETTEDVLRKLAAAVRQRREVNKK
>1BT3A
APIQAPEISKCVVPPADLPPGAVVDNCCPPVASNIVDYKLPAVTTMKVRPAAHTMDKDAIAKFAK

AVELMKALPADDPRNFYQQALVHCAYCNGGYDQVNFPDQEIQVHNSWLFFPFHRWYLYFYERILG
KLIGDPSFGLPFWNWDNPGGMVLPDFLNDSTSSLYDSNRNQSHLPPVVVDLGYNGADTDVTDQQR
ITDNLALMYKQMVTNAGTAELFLGKAYRAGDAPSPGAGSIETSPHIPIHRWVGDPRNTNNEDMGN
FYSAGRDIAFYCHHSNVDRMWTIWQQLAGKPRKRDYTDSDWLNATFLFYDENGQAVKVRIGDSLD
NQKMGYKYAKTPLPWLDSKP
>3BYWA
SNAPVNQVQSSVSWPQNGSLNSVSAPLMSYTPISFDAKIPVASVDKLRKDQDLILGTLPANSEDA
GARGLFVRANDDGLQITSHGELVLDLSKRELAQLPADATIAISATEDETTAGIEGDDSTTETVER
DVRPIIMGIYTELESNAAADLLNAGLNAHVEINSRFTSSPTLAKYAS
>3L6BA
MDAQYDISFADVEKAHINIRDSIHLTPVLTSSILNQLTGRNLFFKCELFQKTGSFKIRGALNAVR
SLVPDALERKPKAVVTHSSGNHGQALTYAAKLEGIPAYIVVPQTAPDCKKLAIQAYGASIVYCEP
SDESRENVAKRVTEETEGIMVHPNQEPAVIAGQGTIALEVLNQVPLVDALVVPVGGGGMLAGIAI
TVKALKPSVKVYAAEPSNADDCYQSKLKGKLMPNLYPPETIADGVKSSIGLNTWPIIRDLVDDIF
TVTEDEIKCATQLVWERMKLLIEPTAGVGVAAVLSQHFQTVSPEVKNICIVLSGGNVDLTSSITW
VKQAERPASYQSVSVHHHHHH
>4HPVA
GSHMRNINVQLNPLSDIEKLQVELVERKGLGHPDYIADAVAEEASRKLSLYYLKKYGVILHHNLD
KTLVVGGQATPRFKGGDIIQPIYIIVAGRATTEVKTESGIDQIPVGTIIIESVKEWIRNNFRYLD
AERHVIVDYKIGKGSSDLVGIFEASKRVPLSNDTSFGVGFAPLTKLEKLVYETERHLNSKQFKAK
LPEVGEDIKVMGLRRGNEVDLTIAMATISELIEDVNHYINVKEQVRNQILDLASKIAPGYNVRVY
VNTGDKIDKNILYLTVTGTSAEHGDDGMTGRGNRGVGLITPMRPMSLEATAGKNPVNHVGKLYNV
LANLIANKIAQEVKDVKFSQVQVLGQIGRPIDDPLIANVDVITYDGKLTDETKNEISGIVDEMLS
SFNKLTELILEGKATLF
>4A57A
MTDSSSLRGVDADTEKRINVGKKHLQTLRNLETRCHDSLQALVVIDAGSSSTRTNVFLAKTRSCP
NKGRSIDPDSIQLIGAGKRFAGLRVVLEEWLDTYAGKDWESRPVDARLLFQYVPQMHEGAKKLMQ
LLEEDTVAILDSQLNEKQKVQVKALGIPVMLCSTAGVRDFHEWYRDALFVLLRHLINNPSPAHGY
KFFTNPFWTRPITGAEEGLFAFITLNHLSRRLGEDPARCMIDEYGVKQCRNDLAGVVEVGGASAQ
IVFPLQEGTVLPSSVRAVNLQRERLLPERYPSADVVSVSFMQLGMASSAGLFLKELCSNDEFLQG
GICSNPCLFKGFQQSCSAGEVEVRPDGSASVNEDVRKNRLKPLATYCSVNNPEISFKVTNEMQCR
ENSIDPTKPLAERMKIENCSIIKGTGNFDKCVSQVESILVAPKLPLPANIEAASSGFESVDQVFR
FASSTAPMIVTGGGMLAAINTLKDHRLLRSDFSGDVEELAEAAREFCSSEVIIRTDGPVIQLPNA
RGEQKLNSLNFDLCKTMALTVSLLRHMAAGENQPSFIKWEKSIAGPDGKPLADLGWQVGVILHHV
LFTEEWGRNAYEAGYSHNLEHHHHHH
>2J7QA
MKIVRASRDQSAPVYGPRAGSQCMSNCFTFLHTCYLMGIDPVLDTTSLDAVLDSGARLDAIADEK
VKRQALTDHPYRLGTEIPTVIETPAGITGHALSRPFNGTAETQDLGGYKCLGILDFLTYARGKPL
PVYIIVTVGVHTRGVIVARGATYVFDPHTTDLSAEAAVYVCDDFTEAISALSFFTEMIGDFYYDA
VLVYFTRCRTTLISPSELLVQIMDQYKDPDIDASVMS
>2E0AA
GPVPREVEHFSRYSPSPLSMKQLLDFGSENACERTSFAFLRQELPVRLANILKEIDILPTQLVNT
SSVQLVKSWYIQSLMDLVEFHEKSPDDQKALSDFVDTLIKVRNRHHNVVPTMAQGIIEYKDACTV
DPVTNQNLQYFLDRFYMNRISTRMLMNQHILIFSDSQTGNPSHIGSIDPNCDVVAVVQDAFECSR

MLCDQYYLSSPELKLTQVNGKFPDQPIHIVYVPSHLHHMLFELFKNAMRATVEHQENQPSLTPIE
VIVVLGKEDLTIKISDRGGGVPLRIIDRLFSYTYSTAPTPVMDNSRNAPLAGFGYGLPISRLYAK
YFQGDLNLYSLSGYGTDAIIYLKALSSESIEKLPVFNKSAFKHYQMSSEADDWCIPSREPKNLAK
EVAM
>2O5NA
EVVRPEVNRTGTVDICQGPMELIFSVSRTSSGATGERISLKNTLSIVSMENGGKPGTYEWSFPAN
ESWPEIQFLLQNREFVSKYYADVVQTPGELVVEYRCPVPQFNCTITHRWKGETIMSFDGAIQTIR
SVTSEYTTKNEDTLVKYIRGLNVTLLTDNAKSIEHRWTEICKKLKDADRPDDNQYTLEDDILEDD
IEMDIVQCQMTTQVPLKYHMTVWSAGRDSRAIALSADYYTDIEVASYLPVNRSQILNTTCEITSS
SGWTVRLRFSEEMVAASKARQAQKRPLLPVEPHGFMSDEHGPAFVQRTINDSRLTLVPR
>3QVLA
SVRIQVINPNTSLAMTETIGAAARAVAAPGTEILAVCPRAGVPSIEGHFDEAIAAVGVLEQIRAG
REQGVDGHVIASFGDPGLLAARELAQGPVIGIAEAAMHMATMVATRFSIVTTLPRTLIIARHLLH
QYGFHQHCAALHAIDLPVLALEDGSGLAQEKVRERCIRALKEDGSGAIVLGSGGMATLAQQLTRE
LRVPVIDGVSAAVKMVESLVALGLATSKHGDLAFPEKKALSGQFQSLNPF
>1RWRA
QGLVPQGQTQVLQGGNKVPVVNIADPNSGGVSHNKFQQFNVANPGVVFNNGLTDGVSRIGGALTK
NPNLTRQASAILAEVTDTSPSRLAGTLEVYGKGADLIIANPNGISVNGLSTLNASNLTLTTGRPS
VNGGRIGLDVQQGTVTIERGGVNATGLGYFDVVARLVKLQGAVSSKQGKPLADIAVVAGANRYDH
ATRRATPIAAGARGAAAGAYAIDGTAAGAMYGKHITLVSSDSGLGVRQLGSLSSPSAITVSSQGE
IALGDATVQRGPLSLKGAGVVSAGKLASGGGAVNVAGGGAV
>1HDHA
MSKRPNFLVIVADDLGFSDIGAFGGEIATPNLDALAIAGLRLTDFHTASTXSPTRSMLLTGTDHH
IAGIGTMAEALTPELEGKPGYEGHLNERVVALPELLREAGYQTLMAGKWHLGLKPEQTPHARGFE
RSFSLLPGAANHYGFEPPYDESTPRILKGTPALYVEDERYLDTLPEGFYSSDAFGDKLLQYLKER
DQSRPFFAYLPFSAPHWPLQAPREIVEKYRGRYDAGPEALRQERLARLKELGLVEADVEAHPVLA
LTREWEALEDEERAKSARAMEVYAAMVERMDWNIGRVVDYLRRQGELDNTFVLFMSDNGAEGALL
EAFPKFGPDLLGFLDRHYDNSLENIGRANSYVWYGPRWAQAATAPSRLYKAFTTQGGIRVPALVR
YPRLSRQGAISHAFATVMDVTPTLLDLAGVRHPGKRWRGREIAEPRGRSWLGWLSGETEAAHDEN
TVTGWELFGMRAIRQGDWKAVYLPAPVGPATWQLYDLARDPGEIHDLADSQPGKLAELIEHWKRY
VSETGVVEGASPFLVR
>2WNPF
SCATGPRNCKDLLDRGYFLSGWHTIYLPDCRPLTVLCDMDTDGGGWTVFQRRMDGSVDFYRDWAA
YKQGFGSQLGEFWLGNDNIHALTAQGSSELRVDLVDFEGNHQFAKYKSFKVADEAEKYKLVLGAF
VGGSAGNSLTGHNNNFFSTKDQDNDVSSSNCAEKFQGAWWYADCHASNLNGLYLMGPHESFANGI
NWSAAKGYKYSYKVSEMKVRPA
>1JF3A
GLSAAQRQVVASTWKDIAGADNGAGVGKECLSKFISAHPEMAAVFGFSGASDPGVAELGAKVLAQ
IGVAVSHLGDEGKMVAEMKAVGVRHKGYGNKHIKAEYFEPLGASLLSAMEHRIGGKMNAAAKDAW
AAAYGDISGALISGLQS
>3ESMA
MSLHVTADAPGAAQGGYSVVTFRVPTESETAATTAMTVTLPNVRSARTEPMPGWTARVDRNDKSE
AVSVTWTADPGNPGVQPGQFQRFVVSIGPLPSAETVSFPAEQTYSDGRVVAWNQPPAANXSEPEH
PAPTLTLATAPGDTEGHHHHHH

>2PLGA
MSLTMVSEVQPVSPASLDAPLENAVEIIETVISSLHQGDAPLVGQTDSGKIWMFRYGSAEVFVQL
SGHTEEDFLTIWSPVLPLPVADELALYRKLLTLNWLTTFEAHFAIAEEQVQVVASRTLGGITAGE
ISRLITIVATLADDYDDALRAEFKGEGHHHHHH
>3LGEE
QAYQGPATGDDDDWDEDWDGPKSSSYFKDSE
>2RINA
AEPESCGTVRFSDVGWTDITATTATATTILEALGYETDVKVLSVPVTYTSLKNKDIDVFLGNWMP
TMEADIAPYREDKSVETVRENLAGAKYTLATNAKGAELGIKDFKDIAAHKDELDGKIYGIEPGND
GNRLIIDMVEKGTFDLKGFEVVESSEQGMLAQVARAEKSGDPIVFLGWEPHPMNANFKLTYLSGG
DDVFGPNYGGATVHTNVRAGYTTECPNVDKLLQNLSFSLQMENEIMGKILNDGEDPEKAAAAWLK
DNPQSIEPWLSGVATKDGGDGLAAVKAALGLEHHHHHH
>3N1MC
GSTERPVAGPYITFTDAVNETTIMLKWMYIPASNNNTPIHGFYIYYRPTDSDNDSDYKKDMVEGD
KYWHSISHLQPETSYDIKMQCFNEGGESEFSNVMICETKARKSSGQ
>4F2DA
MTIFDNYEVWFVIGSQHLYGPETLRQVTQHAEHVVNALNTEAKLPCKLVLKPLGTTPDEITAICR
DANYDDRCAGLVVWLHTFSPAKMWINGLTMLNKPLLQFHTQFNAALPWDSIDMDFMNLNQTAHGG
REFGFIGARMRQQHAVVTGHWQDKQAHERIGSWMRQAVSKQDTRHLKVCRFGDNMREVAVTDGDK
VAAQIKFGFSVNTWAVGDLVQVVNSISDGDVNALVDEYESCYTMTPATQIHGEKRQNVLEAARIE
LGMKRFLEQGGFHAFTTTFEDLHGLKQLPGLAVQRLMQQGYGFAGEGDWKTAALLRIMKVMSTGL
QGGTSFMEDYTYHFEKGNDLVLGSHMLEVCPSIAVEEKPILDVQHLGIGGKDDPARLIFNTQTGP
AIVASLIDLGDRYRLLVNCIDTVKTPHSLPKLPVANALWKAQPDLPTASEAWILAGGAHHTVFSH
ALNLNDMRQFAEMHDIEITVIDNDTRLPAFKDALRWNEVYYGFRR
>3MD9A
MAERIVTIGGDVTEIAYALGAGDEIVARDSTSQQPQAAQKLPDVGYMRTLNAEGILAMKPTMLLV
SELAQPSLVLTQIASSGVNVVTVPGQTTPESVAMKINAVATALHQTEKGQKLIEDYQQRLAAVNK
TPLPVKVLFVMSHGGLTPMAAGQNTAADAMIRAAGGSNAMQGFSRYRPLSQEGVIASAPDLLLIT
TDGVKALGSSENIWKLPGMALTPAGKHKRLLVVDDMALLGFGLETPQVLAQLREKMEQMQ
>3OD9A
MADGFFKQLTLPSGQVVTVSEGRGEPASTGSYDVRLYSGANPQFPLDQFIDGKVLPRDGSIKELK
LLDLNGDKQPELIVVVESAGSGSYLSADAFTLNPQEGLDSFNHVEGLAPNEDVIQALKTPRDLEH
HHHHH
>1GY7A
MSLDFNTLAQNFTQFYYNQFDTDRSQLGNLYRNESMLTFETSQLQGAKDIVEKLVSLPFQKVQHR
ITTLDAQPASPYGDVLVMITGDLLIDEEQNPQRFSQVFHLIPDGNSYYVFNDIFRLNYSA
>3D2OA
MNAIADVQSSRDLRNLPINQVGIKDLRFPITLKTAEGTQSTVARLTMTVYLPAEQKGTHMSRFVA
LMEQHTEVLDFAQLHRLTAEMVALLDSRAGKISVSFPFFRKKTAPVSGIRSLLDYDVSLTGEMKD
GAYGHSMKVMIPVTSLCPCSKEISQYGAHNQRSHVTVSLTSDAEVGIEEVIDYVETQASCQLYGL
LKRPDEKYVTEKAYENPKFVEDMVRDVATSLIADKRIKSFVVESENFESIHNHSAYAYIAYP
>1PBWA
MEADVEQQALTLPDLAEQFAPPDIAPPLLIKLVEAIEKKGLECSTLYRTQSSSNLAELRQLLDCD
TPSVDLEMIDVHVLADAFKRYLLDLPNPVIPAAVYSEMISLAPEVQSSEEYIQLLKKLIRSPSIP

HQYWLTLQYLLKHFFKLSQTSSKNLLNARVLSEIFSPMLFRFSAASSDNTENLIKVIEILISTEW
NERQPAPALPPKPPKPTTVAN
>2WW5A
NETEVAKTSQDTTTASSSSEQNQSSNKTQTSAEVQTNAAAYWDGDYYVKDDGSKAQSEWIFDNYY
KAWFYINSDGRYSQNEWHGNYYLKSGGYMAQNEWIYDSNYKSWFYLKSDGAYAHQEWQLIGNKWY
YFKKWGYMAKSQWQGSYFLNGQGAMIQNEWLYDPAYSAYFYLKSDGTYANQEWQKVGGKWYYFKK
WGYMARNEWQGNYYLTGSGAMATDEVIMDGARYIFAASGELKEKKDLNVGWVHRDGKRYFFNNRE
EQVGTEHAKKIIDISEHNGRINDWKKVIDENEVDGVIVRLGYSGKEDKELAHNIKELNRLGIPYG
VYLYTYAENETDAENDAKQTIELIKKYNMNLSYPIYYDVENWEYVNKSKRAPSDTDTWVKIINKY
MDTMKQAGYQNVYVYSYRSLLQTRLKHPDILKHVNWVAAYTNALEWENPYYSGEKGWQYTSSEYM
KGIQGRVDVSVWY
>2FBAA
AYPSFEAYSNYKVDRTDLETFLDKQKEVSLYYLLQNIAYPEGQFNNGVPGTVIASPSTSNPDYYY
QWTRDSAITFLTVLSELEDNNFNTTLAKAVEYYINTSYNLQRTSNPSGSFDDENHKGLGEPKFNT
DGSAYTGAWGRPQNDGPALRAYAISRYLNDVNSLNEGKLVLTDSGDINFSSTEDIYKNIIKPDLE
YVIGYWDSTGFDLWEENQGRHFFTSLVQQKALAYAVDIAKSFDDGDFANTLSSTASTLESYLSGS
DGGFVNTDVNHIVENPDLLQQNSRQGLDSATYIGPLLTHDIGESSSTPFDVDNEYVLQSYYLLLE
DNKDRYSVNSAYSAGAAIGRYPEDVYNGDGSSEGNPWFLATAYAAQVPYKLAYDAKSASNDITIN
KINYDFFNKYIVDLSTINSAYQSSDSVTIKSGSDEFNTVADNLVTFGDSFLQVILDHINDDGSLN
EQLNRYTGYSTGAYSLTWSSGALLEAIRLRNKVKALA
>4JFHE
SQTIHQWPATLVQPVGSPLSLECTVEGTSNPNLYWYRQAAGRGPQLLFYWGPFGQISSEVPQNLS
ASRPQDRQFILSSKKLLLSDSGFYLCAWSETGLGMGGWQFGEGSRLTVLEDLKNVFPPEVAVFEP
SEAEISHTQKATLVCLATGFYPDHVELSWWVNGKEVHSGVCTDPQPLKEQPALNDSRYALSSRLR
VSATFWQDPRNHFRCQVQFYGLSENDEWTQDRAKPVTQIVSAEAWGRAD
>3CWNA
MGSSHHHHHHSSGLVPRGSHMTDKLTSLRQYTTVVADTGDIAAMKLYQPQDATTNPSLILNAAQI
PEYRKLIDDAVAWAKQQSNDRAQQIVDATDKLAVNIGLEILKLVPGRISTEVDARLSYDTEASIA
KAKRLIKLYNDAGISNDRILIKLASTWQGIRAAEQLEKEGINCNLTLLFSFAQARACAEAGVFLI
SPYVGRILDWYKANTDKKEYAPAEDPGVVSVSEIYQYYKEHGYETVVMGASFRNIGEILELAGCD
RLTIAPTLLKELAESEGAIERKLSYTGEVKARPARITESEFLWQHNQDPMAVDKLAEGIRKFAID
QEKLEKMIGDLL
>1EG2A
MANRSHHNAGHRAMNALRKSGQKHSSESQLGSSEIGTTRHVYDVCDCLDTLAKLPDDSVQLIICD
PPYNIMLADWDDHMDYIGWAKRWLAEAERVLSPTGSIAIFGGLQYQGEAGSGDLISIISHMRQNS
KMLLANLIIWNYPNGMSAQRFFANRHEEIAWFAKTKKYFFDLDAVREPYDEETKAAYMKDKRLNP
ESVEKGRNPTNVWRMSRLNGNSLERVGHPTQKPAAVIERLVRALSHPGSTVLDFFAGSGVTARVA
IQEGRNSICTDAAPVFKEYYQKQLTFLQDDGLIDKARSYEIVEGAANFGAALQRGDVAS
>1NWWA
MTSKIEQPRWASKDSAAGAASTPDEKIVLEFMDALTSNDAAKLIEYFAEDTMYQNMPLPPAYGRD
AVEQTLAGLFTVMSIDAVETFHIGSSNGLVYTERVDVLRALPTGKSYNLSILGVFQLTEGKITGW
RDYFDLREFEEAVDLPLRG
>1EYBA
MGHHHHHHHHHHSSGHIDDDDKHMGSMAELKYISGFGNECSSEDPRCPGSLPEGQNNPQVCPYNL

YAEQLSGSAFTCPRSTNKRSWLYRILPSVSHKPFESIDEGHVTHNWDEVDPDPNQLRWKPFEIPK
ASQKKVDFVSGLHTLCGAGDIKSNNGLAIHIFLCNTSMENRCFYNSDGDFLIVPQKGNLLIYTEF
GKMLVQPNEICVIQRGMRFSIDVFEETRGYILEVYGVHFELPDLGPIGANGLANPRDFLIPIAWY
EDRQVPGGYTVINKYQGKLFAAKQDVSPFNVVAWHGNYTPYKYNLKNFMVINSVAFDHADPSIFT
VLTAKSVRPGVAIADFVIFPPRWGVADKTFRPPYYHRNCMSEFMGLIRGHYEAKQGGFLPGGGSL
HSTMTPHGPDADCFEKASKVKLAPERIADGTMAFMFESSLSLAVTKWGLKASRCLDENYHKCWEP
LKSHFTPNSRNPAEPN
>1F60B
KPAKPAAKSIVTLDVKPWDDETNLEEMVANVKAIEMEGLTWGAHQFIPIGFGIKKLQINCVVEDD
KVSLDDLQQSIEEDEDHVQSTDIAAMQKL
>3VOQA
GAMATVQDMLSSHHYKSFKVSMIHRLRFTTDVQLGISGDKVEIDPVTNQKASTKFWIKQKPISID
SDLLCACDLAEEKSPSHAIFKLTYLSNHDYKHLYFESDAATVNEIVLKVNYILESRASTA
>4ESQA
GHQPVAEERLSALLLNSSEVNAVMGSSSMQPGKPITSMDSSPVTVSLPDCQGALYTSQDPVYAGT
GYTAINGLISSEPGDNYEHWVNQAVVAFPTADKARAFVQTSADKWKNCAGKTVTVTNKAKTYRWT
FADVKGSPPTITVIDTQEGAEGWECQRAMSVANNVVVDVNACGYQITNQAGQIAAKIVDKVNKE
>2QLWA
MRGSHHHHHHGMASMTGGQQMGRDLYDDDDKDRWGSGDMTLEKHAFKMQLNPGMEAEYRKRHDEI
WPELVDLLHQSGASDYSIHLDRETNTLFGVLTRPKDHTMASLPDHPVMKKWWAHMADIMATNPDN
SPVQSDLVTLFHMP
>3DA7A
SGRTWREADINYTSGFRNSDRILYSSDWLIYKTTDHYQTFTKIRCAQVINTFDGVADYLQTYHKL
PDNYITKSEAQALGWVASKGNLADVAPGKSIGGDIFSNREGKLPGK
>3DEEA
GMQPETSAQYQHRFSQAIRGGEAADGLPQDRLNVYIRLIRNNIHSFIDRCYTETRQYFDSKEWSR
LKEGFVRDARAQTPYFQEIPGEFLQYCQSLPLSDGILALMDFEYTQLLAEVAQIPDIPDIHYSND
SKYTPSPAAFIRQYRYDVTHDLQEAETALLIWRNAEDDVMYQTLDGFDMMLLEIMGSSALSFDTL
AQTLVEFMPKADNWKNILLGKWSGWIEQRIIIPSLSAISENMEGNSPSQNHLSA
>3DORA
SLVCKNALQDLSFLEHLLQVKYAPKTWKEQYLGWDLVQSSVSAQQKLRTQENPSTSFCQQVLADF
IGGLNDFHAGVTFFAIESAYLPYTVQKSSDGRFYFVDIMTFSSEIRVGDELLEVDGAPVQDVLAT
LYGSNHKGTAAEESAALRTLFSRMASLGHKVPSGRTTLKIRRPFGTTREVRVKWRYVPEGVGDLA
TIAPSIRAPQLQKSMRSFFPKKDDAFHRSSSLFYSPMVPHFWAELRNHYATSGLKSGYNIGSTDG
FLPVIGPVIWESEGLFRAYISSVTDGDGKSHKVGFLRIPTYSWQDMEDFDPSGPPPWEEFAKIIQ
VFSSNTEALIIDQTNNPGGSVLYLYALLSMLTDRPLELPKHRMILTQDEVVDALDWLTLLENVDT
NVESRLALGDNMEGYTVDLQVAEYLKSFGRQVLNCWSKGDIELSTPIPLFGFEKIHPHPRVQYSK
PICVLINEQDFSCADFFPVVLKDNDRALIVGTRTAGAGGFVFNVQFPNRTGIKTCSLTGSLAVRE
HGAFIENIGVEPHIDLPFTANDIRYKGYSEYLDKVKKLVCQLINNDGTIILAEDGSFHHHHHH
>2GDMA
GALTESQAALVKSSWEEFNANIPKHTHRFFILVLEIAPAAKDLFSFLKGTSEVPQNNPELQAHAG
KVFKLVYEAAIQLEVTGVVVTDATLKNLGSVHVSKGVADAHFPVVKEAILKTIKEVVGAKWSEEL
NSAWTIAYDELAIVIKKEMDDAA
>4AAJA

MGSSHHHHHHSSGLVPRGSHMFVKICGIKSLEELEIVEKHADATGVVVNSNSKRRIPLEKAREII
ENSAIPVFLVSTMVGFSEWAMAIERTGAQYIQVHSNALPQTIDTLKKEFGVFVMKAFRVPTISKN
PEEDANRLLSEISRYNADMVLLDTGAGSGKLHDLRVSSLVARKIPVIVAGGLNAENVEEVIKVVK
PYGVDVSSGVEKYGIKDPKLVEEFVRRAKNVVW

>3K40A

MEAPEFKDFAKTMVDFIAEYLENIRERRVLPEVKPGYLKPLIPDAAPEKPEKWQDVMQDIERVIM
PGVTHWHSPKFHAYFPTANSYPAIVADMLSGAIACIGFTWIASPACTELEVVMMDWLGKMLELPA
EFLACSGGKGGGVIQGTASESTLVALLGAKAKKLKEVKELHPEWDEHTILGKLVGYCSDQAHSSV
ERAGLLGGVKLRSVQSENHRMRGAALEKAIEQDVAEGLIPFYAVVTLGTTNSCAFDYLDECGPVG
NKHNLWIHVDAAYAGSAFICPEYRHLMKGIESADSFNFNPHKWMLVNFDCSAMWLKDPSWVVNAF
NVDPLYLKHDMQGSAPDYRHWQIPLGRRFRALKLWFVLRLYGVENLQAHIRRHCNFAKQFGDLCV
ADSRFELAAEINMGLVCFRLKGSNERNEALLKRINGRGHIHLVPAKIKDVYFLRMAICSRFTQSE
DMEYSWKEVSAAADEMEQEQ

>3CIHA

MSLILLGALSLASSTFAQTWIWYPGDYEIWLGNQMNNRRTERGAFFPPFWKTDSHYVVVEFSKVL
NLSEPEEVFIAAEGTYNVKLDGKLQFGMPETLLLPAGKHSLNIKVWNQATPPTIYVKGKTVNSDS
SWRVTYEDKEWIDESGKASDTSATIYMDAGCWNFDGATQRPSQFSLMREPQQPVAKTEQPEGGIL
YDFGKETFGFITLKNLSGKGKIDLYYGESPEEAKDKAYCETLDKLLLEPGQITDLAIRSTSPLHH
SDNEYTLENSKAFRYVYITHEPEVQIGEVSMQYEYLPEEYRGNFRCNDEELNCIWEVGAYTMHLT
TREFFIDGIKRDRWVWSGDAIQSYLMNYYLFFDSESVKRTIWLLRGKDPVTSHSNTIMDYTFYWF
LSVYDYYMYSGDRHFVNQLYPRMQTMMDYVLGRTNKNGMVEGMSGDWVFVDWADGYLDKKGELSF
EQVLFCRSLETMALCADLVGDKDGQQKYEKLASALKAKLEPTFWNNQKQAFVHNCVDGRQSDAVT
RYANMFSVFFDYLNADKQQAIKQSVLLNDEILKITTPYMRFYELEALCALGEQETVMKEMKAYWG
GMLKAGATSFWEKYNPEESGTQHLAMYGRPYGKSLCHAWGASPIYLLGKYYLGVKPTKEGYKEFA
VSPVLGGLKWMEGTVPTPNGDIHVYMDNKTIKVKATEGKGYLTIQSRRQPKANMGTVEKVSEGVW
RLWIDSPEERIVTYRLEGHHHHHH

>3LHOA

GMHTDVNALFAALWQDYIKMTPSAAKIHQLLGHGAPIINDHIALRTFNIAKVNLSVLAKHFTSIG
YVDSGDYKFEQKKLIAKHFEHPDPKQPKVFISELLVEEFSPEVQKSIHGLIDQVDIAATTADNFI
YSGRHWDVDKATYQALLAESEYAAWVAALGYRANHFTVSINDLPEFERIEDVNQALKQAGFVLNS
SGGEVKGSPEVLLEQSSTMADKVVVNFTDGDVEIPSCFYEFARRYPMANGQLYTGFVAASADKIF
ESTNAMM

>1Z70X

EANAPGPVPGERQLAHSKMVPIPAGVFTMGTDDPQIKQDGEAPARRVTIDAFYMDAYEVSNTEFE
KFVNSTGYLTEAEKFGDSFVFEGMLSEQVKTNIQQAVAAAPWWLPVKGANWRHPEGPDSTILHRP
DHPVLHVSWNDAVAYCTWAGKRLPTEAEWEYSCRGGLHNRLFPWGNKLQPKGQHYANIWQGEFPV
TNTGEDGFQGTAPVDAFPPNGYGLYNIVGNAWEWTSDWWTVHHSVEETLNPKGPPSGKDRVKKGG
SYMCHRSYCYRYRCAARSQNTPDSSASNLGFRCAADRLPTMDRGSHHHHHH

>3NVOA

GHMEAIKGSDVNVPDAVFAWLLDGRGGVKPLEDNDVIDSQHPCWLHLNYTHPDSARWLASTPLLP
NNVRDALAGESSRPRVSRMGEGTLITLRCINGSTDERPDQLVAMRLYMDERFIVSTRQRKVLALD
DVVSDLQEGTGPVDCGGWLVDVCDALTDHASEFIEELHDKIIDLEDNLLDQQIPPRGFLALLRKQ
LIVMRRYMAPQRDVYARLASERLPWMSDDHRRRMQDIADRLGRGLDEIDACIARTGIMADEIAQV
MQES

>1VR4A
MIVTTTSGIQGKEIIEYIDIVNGEAIMGANIVRDLFASVRDVVGGRAGSYESKLKEARDIAMDEM
KELAKQKGANAIVGVDVDYEVVRDGMLMVAVSGTAVRI
>4E3YA
DTLESIDNCAVGCPTGGSSNVSIVRHAYTLNNNSTTKFANWVAYHITKDTPASGKTRNWKTDPAL
NPADTLAPADYTGANAALKVDRGHQAPLASLAGVSDWESLNYLSNITPQKSDLNQGAWARLEDQE
RKLIDRADISSVYTVTGPLYERDMGKLPGTQKAHTIPSAYWKVIFINNSPAVNHYAAFLFDQNTP
KGADFCQFRVTVDEIEKRTGLIIWAGLPDDVQASLKSKPGVLPELMGCKN
>2UXYA
MRHGDISSSNDTVGVAVVNYKMPRLHTAAEVLDNARKIAEMIVGMKQGLPGMDLVVFPEYSLQGI
MYDPAEMMETAVAIPGEETEIFSRACRKANVWGVFSLTGERHEEHPRKAPYNTLVLIDNNGEIVQ
KYRKIIPWCPIEGWYPGGQTYVSEGPKGMKISLIICDDGNYPEIWRDCAMKGAELIVRCQGYMYP
AKDQQVMMAKAMAWANNCYVAVANAAGFDGVYSYFGHSAIIGFDGRTLGECGEEEMGIQYAQLSL
SQIRDARANDQSQNHLFKILHRGYSGLQASGDGDRGLAECPFEFYRTWVTDAEKARENVERLTRS
TTGVAQCPVGRLPYEG
>2CWRA
GPTTPVPVSGSLEVKVNDWGSGAEYDVTLNLDGQYDWTVKVKLAPGATVGSFWSANKQEGNGYVI
FTPVSWNKGPTATFGFIVNGPQGDKVEEITLEINGQVI
>2II2A
GPLGSYGDAIPEVKAILEAKNEEELVTFTSRWSAEERKELRTQFQDTTGLEFIAFLKKCIKNGPY
EDVMALGWDCNISARVNVIKKAMKNVNDFRAIHDVVLIATPDERLKLAQAYKEKTGNDLLQDFVD
QIPLTSAASYLCHLAIRENRTPRGSVASDAEVLKHNLIDADEPDHEAVVRLIITSTADEYKEINH
RFEVLTGKSVQEAIETRYADKENARGLCIAHYYNLAPARAVAYAFHSAVETQNDDMAYEQAARIT
GLFHDLHKFAWVHYACWGVMRDDILSRFQSKEANKVNFRDACLMFWKLAK
>3ZITA
MKKIEVYAQPDCPPCVIVKEFLKHNNVAYEEFDVKKDAAARNRLLYDYDSYSTPTVVIDGEVVAG
FQIEKLQQLLNIE
>1JX6A
VLNGYWGYQEFLDEFPEQRNLTNALSEAVRAQPVPLSKPTQRPIKISVVYPGQQVSDYWVRNIAS
FEKRLYKLNINYQLNQVFTRPNADIKQQSLSLMEALKSKSDYLIFTLDTTRHRKFVEHVLDSTNT
KLILQNITTPVREWDKHQPFLYVGFDHAEGSRELATEFGKFFPKHTYYSVLYFSEGYISDVRGDT
FIHQVNRDNNFELQSAYYTKATKQSGYDAAKASLAKHPDVDFIYACSTDVALGAVDALAELGRED
IMINGWGGGSAELDAIQKGDLDITVMRMNDDTGIAMAEAIKWDLEDKPVPTVYSGDFEIVTKADS
PERIEALKKRAFRYSDN
>1VPBA
MGSDKIHHHHHHMITDENKKLAQWAMDYALKNGCQAAKVLLYSSSNTSFELRDAKMDRLQQASEG
GLSLSLYVDGRYGSISTNRLNRKELETFIKNGIDSTRYLAKDEARVLADPSRYYKGGKPDLKLYD
AKFASLNPDDKIEMAKAVAEEALGKDERIISVGSSYGDGEDFAYRLISNGFEGETKSTWYSLSAD
ITIRGEGEARPSAYWYESSLYMNDLIKKGIGQKALERVLRKLGQKKVQSGKYTMVVDPMNSSRLL
SPMISALNGSALQQKNSFLLNKLNEKIASDRLTLTDEPHLVKASGARYFDNEGIATERRSIFDKG
VLNTYFIDTYNAKKMGVDPTISGSSILVMETGDKNLDGLIAGVEKGILVTGFNGGNNNSSTGDFS
YGIEGFLIENGKLTQPVSEMNVTGNLITLWNSLVATGNDPRLNSSWRIPSLVFEGVDFSGL
>2ZUXA
AARQMEALNRGLVAVKTDGGIFVSWRFLGTENASVLFNVYRDGQKLNAAPVKTTNYVDKNGSAGS

TYTVRAVVNGTEQPASEKASVWAQPYHSVPLDKPAGGTTPKGESYTYSANDASVGDVDGDGQYEL
ILKWDPSNSKDNSQDGYTGDVLIDAYKLDGTKLWRINLGKNIRAGAHYTQFMVYDLDGDGKAEVA
MKTADGTKDGTGKVIGNANADYRNEQGRVLSGPEYLTVFQGSTGKELVTANFEPARGNVSDWGDS
YGNRVDRFLAGIAYLDGQRPSLIMTRGYYAKTMLVAYNFRDGKLSKLWTLDSSKSGNEAFAGQGN
HNLSIADVDGDGKDEIIFGSMAVDHDGKGMYSTGLGHGDALHTGDLDPGRPGLEVFQVHEDKNAK
YGLSFRDAATGKILWGVYAGKDVGRGMAADIDPRYPGQEVWANGSLYSAKGVKIGSGVPSSTNFG
IWWDGDLLREQLDSNRIDKWDYQNGVSKNMLTASGAAANNGTKATPTLQADLLGDWREEVVWRTE
DSSALRIYTTTIPTEHRLYTLMHDPVYRLGIAWQNIAYNQPPHTSFFLGDGMAEQPKPNMYTPLE
HHHHHH
>2BS2B
MGRMLTIRVFKYDPQSAVSKPHFQEYKIEEAPSMTIFIVLNMIRETYDPDLNFDFVCRAGICGSC
GMMINGRPSLACRTLTKDFEDGVITLLPLPAFKLIKDLSVDTGNWFNGMSQRVESWIHAQKEHDI
SKLEERIEPEVAQEVFELDRCIECGCCIAACGTKIMREDFVGAAGLNRVVRFMIDPHDERTDEDY
YELIGDDDGVFGCMTLLACHDVCPKNLPLQSKIAYLRRKMVSVNMS
>3ZE3A
GHHHHHHELANNTTGFTRIIKAAGYSWKGLRAAWINEAAFRQEGVAVLLCVVIAAWLDVDAVTRV
LLISSVMLVMIVELLNSAIEAVVDRIGSEYHELSGRAKDLGSAAVLIAIIDAVITWAILLWSHFG
>1W66A
GAMAGSIRSKLSAIDVRQLGTVDYRTAWQLQRELADARVAGGADTLLLLEHPAVYTAGRRTETHE
RPIDGTPVVDTDRGGKITWHGPGQLVGYPIIGLAEPLDVVNYVRRLEESLIQVCADLGLHAGRVD
GRSGVWLPGRPARKVAAIGVRVSRATTLHGFALNCDCDLAAFTAIVPCGISDAAVTSLSAELGRT
VTVDEVRATVAAAVCAALDGVLPVGDRVPSHAVPSPL
>4HZ4A
MVMITLHYLKQSCSHRIVWLLEALGLDYELKIYDRLEGTGFAPEELKAQHPLGKAPVLQDGDLVL
AEGNAIIQHLLDRYDTENRFTPAHKTDAYSNYVYWLAISASMFSANLLALVSKKGDLGDFAQYTN
AQVGLYFSHVEKSLEGKTWIVGEQLTGADFALSFPLQWGLNYVNKADYPNITRYLEQIETHPAYL
KANEKTDGGLDLSRFAENLYFQ
>3NUFA
GMTPLDANVELPTEVKAMIEQSSDAQAATALVNYVIKLAAAAEIHFTDLQLQVLTNHLIEMLGRS
KSGEQLPAVDPTMFAEVSQKSLDLADQVVQHIGHLEVAEKYVLSIHFEAAQDKI
>1EFDN
AGIDPNRIVALEWLPVELLLALGIVPYGVADTINYRLWVSEPPLPDSVIDVGLRTEPNLELLTEM
KPSFMVWSAGYGPSPEMLARIAPGRGFNFSDGKQPLAMARKSLTEMADLLNLQSAAETHLAQYED
FIRSMKPRFVKRGARPLLLTTLIDPRHMLVFGPNSLFQEILDEYGIPNAWQGETNFWGSTAVSID
RLAAYKDVDVLCFDHDNSKDMDALMATPLWQAMPFVRAGRFQRVPAVWFYGATLSAMHFVRVLDN
AIGGKA
>2ZQ5A
MTRRPDRKDVATVDELHASATKLVGLDDFGTDDDNYREALGVLLDAYQGEAGLTVLGSKMNRFFL
RGALVARLLSQSAWKQYPEHVDVAIKRPIFVTGLVRTGTTALHRLLGADPAHQGLHMWLAEYPQP
RPPRETWESNPLYRQLDADFTQHHAENPGYTGLHFMAAYELEECWQLLRQSLHSVSYEALAHVPS
YADWLSRQDWTPSYCRHRRNLQLIGLNDAEKRWVLKNPSHLFALDALMATYPDALVVQTHRPVET
IMASMCSLAQHTTEGWSTKFVGAQIGADAMDTWSRGLERFNAARAKYDSAQFYDVDYHDLIADPL
GTVADIYRHFGLTLSDEARQAMTTVHAESQSGARAPKHSYSLADYGLTVEMVKERFAGL
>2O30A

MPSEAKYTWDQELNEINIQFPVTGDADSSAIKIRMVGKKICVKNQGEIVIDGELLHEVDVSSLWW
VINGDVVDVNVTKKRNEWWDSLLVGSESVDVQKLAENKHADMSMLDAEAREVVEKMMHNTSGKDS
E
>2CO3A
GSQKSVDIVFSSPQDLTVSLIPVSGLKAGKNAPSAKIAKLVVNSTTLKEFGVRGISNNVVDSTGT
AWRVAGKNTGKEIGVGLSSDSLRRSDSTEKWNGVNWMTFNSNDTLDIVLTGPAQNVTADTYPITL
DVVGYQP
>4GNRA
GSVEEKTIKIGFNFEESGSLAAYGTAEQKGAQLAVDEINAAGGIDGKQIEVVDKDNKSETAEAAS
VTTNLVTQSKVSAVVGPATSGATAAAVANATKAGVPLISPSATQDGLTKGQDYLFIGTFQDSFQG
KIISNYVSEKLNAKKVVLYTDNASDYAKGIAKSFRESYKGEIVADETFVAGDTDFQAALTKMKGK
DFDAIVVPGYYNEAGKIVNQARGMGIDKPIVGGDGFNGEEFVQQATAEKASNIYFISGFSTTVEV
SAKAKAFLDAYRAKYNEEPSTFAALAYDSVHLVANAAKGAKNSGEIKDNLAKTKDFEGVTGQTSF
DADHNTVKTAYMMTMNNGKVEAAEVVKP
>1F00I
ASITEIKADKTTAVANGQDAITYTVKVMKGDKPVSNQEVTFTTTLGKLSNSTEKTDTNGYAKVTL
TSTTPGKSLVSARVSDVAVDVKAPEVEFFTTLTIDDGNIEIVGTGVKGKLPTVWLQYGQVNLKAS
GGNGKYTWRSANPAIASVDASSGQVTLKEKGTTTISVISSDNQTATYTIATPNSLIVPNMSKRVT
YNDAVNTCKNFGGKLPSSQNELENVFKAWGAANKYEYYKSSQTIISWVQQTAQDAKSGVASTYDL
VKQNPLNNIKASESNAYATCVK
>2FD6U
SLRCMQCKTNGDCRVEECALGQDLCRTTIVRLWEEGEELELVEKSCTHSEKTNRTLSYRTGLKIT
SLTEVVCGLDLCNQGNSGRAVTYSRSRYLECISCGSSDMSCERGRHQSLQCRSPEEQCLDVVTHW
IQEGEEGRPKDDRHLRGCGYLPGCPGSNGFHNNDTFHFLKCCNTTKCNEGPILELENLPQNGRQC
YSCKGNSTHGCSSEETFLIDCRGPMNQCLVATGTHEPKNQSYMVRGCATASMCQHAHLGDAFSMN
HIDVSCCTKSGCNHPD
>2VPBB
AMAAKVVYVFSTEMANKAAEAVLKGQVETIVSFHI
>3VENA
HHHHHHMRVLGLNGWPRDFHDASAALLVDGRIAAFAEEERLTRKKHGYNTAPVQAAAFCLAQAGL
TVDDLDAVAFGWDLPAMYRERLGGWPHSDSEALDILLPRDVFPRRTDPPLHFVQHHLAHAASAYY
FSGEDRGAVLIVDGQGEEECVTLAHAEGGKITVLDTVPGAWSLGFFYEHVSEYTGLGGDNPGKLM
GLAAHGTTVDETLSAFAFDSDGYRLNLIDPQARDPEDWDEYSVTERAWFAHLERIYRLPPNEFVR
RYDPAKGRVVRDTRRDPYEYRDLAATAQAALERAVFGLADSVLARTGERTLFVAGGVGLNATMNG
KLLTRSTVDKMFVPPVASDIGVSLGAAAAVAVELGDRIAPMGDTAAWGPEFSPDQVRAALDRTGL
AYREPANLEREVAALIASGKVVGWAQGRGEVGPRALGQRSLLGSAHSPTMRDHINLRVKDREWWR
PFAPSMLRSVSDQVLEVDADFPYMIMTTKVRAAYAERLPSVVHEDWSTRPQTVTEASNPRYHRML
TELGDLVGDPVCLNTSFNDRGEPIVSSPADALLTFSRLPIDALAVGPYLVTKDLRH
>3VL1A
GSHMTKTITVAHIQYDFKAVLEENDENDDEFYINVDKNLNEIKEHKIVVLGNSRGVDAGKGNTFE
KVGSHLYKARLDGHDFLFNTIIRDGSKMLKRADYTAVDTAKLQMRRFILGTTEGDIKVLDSNFNL
QREIDQAHVSEITKLKFFPSGEALISSSQDMQLKIWSVKDGSNPRTLIGHRATVTDIAIIDRGRN
VLSASLDGTIRLWECGTGTTIHTFNRKENPHDGVNSIALFVGTDRQLHEISTSKKNNLEFGTYGK
YVIAGHVSGVITVHNVFSKEQTIQLPSKFTCSCNSLTVDGNNANYIYAGYENGMLAQWDLRSPEC

PVGEFLINEGTPINNVYFAAGALFVSSGFDTSIKLDIISDPESERPAIEFETPTFLVSNDDAVSQ
FCYVSDDESNGEVLEVGKNNFCALYNLSNP
>2O34A
SLPMQHVHTSPVRDYRNRCARREGETVFQVVVEETDLRVTALAELATPMAAYVGELRAQLKVWME
FQPAFRHSLVPVEVPEGAPEVVRRMAHGARLVGVGPFAAVAGTIAQMVAERFVDVSPELIVENGG
DLYLYSERDRVVGILPDPASGDMVGILVRAGTAPVSLCGSSARIGHSLSLGDGDLAVVRARDASL
ADAAATAFGNMLRRADDVAAVTERAAQLASIGIEGVYAQCGGRIGIWGDMELAVA
>2R8EA
MSKAGASLATCYGPVSADVMAKAENIRLLILDVDGVLSDGLIYMGNNGEELKAFNVRDGYGIRCA
LTSDIEVAIITGRKAKLVEDRCATLGITHLYQGQSNKLIAFSDLLEKLAIAPENVAYVGDDLIDW
PVMEKVGLSVAVADAHPLLIPRADYVTRIAGGRGAVREVCDLLLLAQGKLDEAKGQSI
>1KQFA
MDVSRRQFFKICAGGMAGTTVAALGFAPKQALAQARNYKLLRAKEIRNTCTYCSVGCGLLMYSLG
DGAKNAREAIYHIEGDPDHPVSRGALCPKGAGLLDYVNSENRLRYPEYRAPGSDKWQRISWEEAF
SRIAKLMKADRDANFIEKNEQGVTVNRWLSTGMLCASGASNETGMLTQKFARSLGMLAVDNQARV
CHGPTVASLAPTFGRGAMTNHWVDIKNANVVMVMGGNAAEAHPVGFRWAMEAKNNNDATLIVVDP
RFTRTASVADIYAPIRSGTDITFLSGVLRYLIENNKINAEYVKHYTNASLLVRDDFAFEDGLFSG
YDAEKRQYDKSSWNYQLDENGYAKRDETLTHPRCVWNLLKEHVSRYTPDVVENICGTPKADFLKV
CEVLASTSAPDRTTTFLYALGWTQHTVGAQNIRTMAMIQLLLGNMGMAGGGVNALRGHSNIQGLT
DLGLLSTSLPGYLTLPSEKQVDLQSYLEANTPKATLADQVNYWSNYPKFFVSLMKSFYGDAAQKE
NNWGYDWLPKWDQTYDVIKYFNMMDEGKVTGYFCQGFNPVASFPDKNKVVSCLSKLKYMVVIDPL
VTETSTFWQNHGESNDVDPASIQTEVFRLPSTCFAEEDGSIANSGRWLQWHWKGQDAPGEARNDG
EILAGIYHHLRELYQSEGGKGVEPLMKMSWNYKQPHEPQSDEVAKENNGYALEDLYDANGVLIAK
KGQLLSSFAHLRDDGTTASSCWIYTGSWTEQGNQMANRDNSDPSGLGNTLGWAWAWPLNRRVLYN
RASADINGKPWDPKRMLIQWNGSKWTGNDIPDFGNAAPGTPTGPFIMQPEGMGRLFAINKMAEGP
FPEHYEPIETPLGTNPLHPNVVSNPVVRLYEQDALRMGKKEQFPYVGTTYRLTEHFHTWTKHALL
NAIAQPEQFVEISETLAAAKGINNGDRVTVSSKRGFIRAVAVVTRRLKPLNVNGQQVETVGIPIH
WGFEGVARKGYIANTLTPNVGDANSQTPEYKAFLVNIEKA
>3SJRA
SNAMVMDDDITVQPIRGVQPRPAGSHEPFAVPSRAGQHGKRPDGEDSADISLSQGAQAAALLFSA
AMDQISRLAELDIEPVRLPESELTGDSHSQHLLLGMEILMELYRQQHPDWTAPAIRQAFAPLARA
GLERGYQEACQVLRQLNVYTPAVAGQLQGLLLLTQRLFEERLQIA
>3DNZA
ITGTSTVGVGRGVLGDQKNINTTYSTYYYLQDNTRGNGIFTYDAKYRTTLPGSLWADADNQFFAS
YDAPAVDAHYYAGVTYDYYKNVHNRLSYDGNNAAIRSSVHYSQGYNNAFWNGSQMVYGDGDGQTF
IPLSGGIDVVAHELTHAVTDYTAGLIYQNESGAINEAISDIFGTLVEFYANKNPDWEIGEDVYTP
GISGDSLRSMSDPAKYGDPDHYSKRYTGTQDNGGVHINSGIINKAAYLISQGGTHYGVSVVGIGR
DKLGKIFYRALTQYLTPTSNFSQLRAAAVQSATDLYGSTSQEVASVKQAFDAVGVK
>3CWRA
GMVEQRNRGRPAVPDAVVRESIVGAAQRLLSSGGAAAMTMEGVASEAGIAKKTLYRFASGRADLI
GLLVESWIAPIFPGFEADPQDAAAALERIVYDIAQAVLSREAVSLFRMLASDADLRNRFLPAYNA
NGIERSRRELARWLDQQASAGRLPLPIPAERVADLLLSAVIAEPLRQITLGLREPLPAWDIAPRV
ADAVRLIAPGRER
>1QOYA

GSPGISGGGGGILDSMAEIVADKTVEVVKNAIETADGALDLYNKYLDQVIPWQTFDETIKELSRF
KQEYSQAASVLVGDIKTLLMDSQDKYFEATQTVYEWCGVATQLLAAYILLFDEYNEKKASAQKDI
LIKVLDDGITKLNEAQKSLLVSSQSFNNASGKLLALDSQLTNDFSEKSSYFQSQVDKIRKEAYAG
AAAGVVVGPFGLIISYSIAAGVVEGKLIPELKNKLKSVQNFFTTLSNTVKQANKDIDAAKLKLTT
EIAAIGEIKTETETTRFYVDYDDLMLSLLKEAAKKMINTCNEYQKRHGKKTLFEVPEV
>2H1VA
MSRKKMGLLVMAYGTPYKEEDIERYYTHIRRGRKPEPEMLQDLKDRYEAIGGISPLAQITEQQAH
NLEQHLNEIQDEITFKAYIGLAHIEPFIEDAVAEMHKDGITEAVSIVLAPHFSTFSVQSYNKRAK
EEAEKLGGLTITSVESWYDEPKFVTYWVDRVKETYASMPEDERENAMLIVSAHSLPEKIKEFGDP
YPDQLHESAKLIAEGAGVSEYAVGWQSEGNTPDPWLGPDVQDLTRDLFEQKGYQAFVYVPVGFVA
DHLEVLYDNDYECKVVTDDIGASYYRPEMPNAKPEFIDALATVVLKKLGR
>3RUIA
GSDPLKIADQSVDLNLKLMKWRILPDLNLDIIKNTKVLLLGAGTLGCYVSRALIAWGVRKITFVD
NGTVSYSNPVRQALYNFEDCGKPKAELAAASLKRIFPLMDATGVKLSIPMIGHKLVNEEAQHKDF
DRLRALIKEHDIIFLLVDSRESRWLPSLLSNIENKTVINAALGFDSYLVMRHGNRDEQSSKQLGC
YFCHDVVAPTDSLTDRTLDQMSTVTRPGVAMMASSLAVELMTSLLQTKYSGSETTVLGDIPHQIR
GFLHNFSILKLETPAYEHCPACSPKVIEAFTDLGWEFVKKALEHPLYLEEISGLSVIKQEVERLG
NDVFEWEDDESDEIA
>3A9LA
MAQTDTYPNIEALENAETVGVAYNIEVKRQNPSMIYFSPHAGGIEVGTTELIYRVVELTGGSLYL
FQGLLPSGNSRLHVTSTHFDEPMAVCMLSKHTDAVSFHGYKDDYNKNTLVGGLNTELRNLIVSKL
NSKGIAAEVATDRFTATDPDNIVNRCASGKGVQLEISSAQRRAFFQNNDWSKANRGNVTQEFLDY
AEAIKEAEAEYYGLEHHHHHH
>3MCRA
GMTSNGQQGKPNLPEKDNLPRELGTQRINSPIARMGMFGAKTTGDTSGYGRLRVYRHVPAAAQRP
YSDPSDPRTAYFDEVADALERSLKEIGTPYDTAISRVVVDRGEITFHVQREHLLDVATRLRDDPA
LRFELCLGVTGVHYPEDEGNELHAVYALRSITHNYEIRLEVSCPDSDPHIPSIVSVYPTNDWHER
EAWDFFGIIFDGHPALTR
>3DMBA
GMADPKELQDKFWKALKSDRTVMLGLDGVEDGHARPMTAQIEGDSGGPIWFFTSKDNALIAMLGQ
GRRVIGAFSSKGHDLFASISGSLREDTDPAVVDRLWNPYVAAWYEGGKDDPKLALLRLDADHAQI
WLNGSSLLAGIKVLLGV
>2I0KA
STGPVAPLPTPPNFPNDIALFQQAYQNWSKEIMLDATWVCSPKTPQDVVRLANWAHEHDYKIRPR
GAMAGWTPLTVEKGANVEKVILADTMTHLNGITVNTGGPVATVTAGAGASIEAIVTELQKHDLGW
ANLPAPGVLSIGGALAVNAHGAALPAVGQTTLPGHTYGSLSNLVTELTAVVWNGTTYALETYQRN
DPRITPLLTNLGRCFLTSVTMQAGPNFRQRCQSYTDIPWRELFAPKGADGRTFEKFVAESGGAEA
IWYPFTEKPWMKVWTVSPTKPDSSNEVGSLGSAGSLVGKPPQAREVSGPYNYIFSDNLPEPITDM
IGAINAGNPGIAPLFGPAMYEITKLGLAATNANDIWGWSKDVQFYIKATTLRLTEGGGAVVTSRA
NIATVINDFTEWFHERIEFYRAKGEFPLNGPVEIRCCGLDQAADVKVPSVGPPTISATRPRPDHP
DWDVAIWLNVLGVPGTPGMFEFYREMEQWMRSHYNNDDATFRPEWSKGWAFGPDPYTDNDIVTNK
MRATYIEGVPTTENWDTARARYNQIDPHRVFTNGFMDKLLP
>3Q64A
MTERSVVHSTFIIERLYPAPPSKVFFALGNADAKRRWFTDPDNPMPGRFEMDFRVGGKEVNAGGP

KDGPIHVYTATYQDIVPDQRIVYSYDMLFGETRISVSLATIQLFAEGEGTRLVLTEQGAFLDGHD
TPSTREHGTGVLLDLLDAFLDKTTLEHHHHHH
>1XKPA
XQFRGESVQIVSGTLQSIADMAEEVTFVFSERKELSLDKRKLSDSQARVSDVEEQVNQYLSKVPE
LEQKQNVSELLSLLSNSPNISLSQLKAYLEGKSEEPSEQFKMLCGLRDALKGRPELAHLSHLVEQ
ALVSMAEEQGETIVLGARITPEAYRESQSGVNPLQPLRDTYRDAVMGYQGIYAIWSDLQKRFPNG
DIDSVILFLQKALSADLQSQQSGSGREKLGIVISDLQKLKEFGSVSDQVKG
>3TIPA
GPHMIAPGHRDEFDPKLPTGEKEEVPGKPGIKNPETGDVVRPPVDSVTKYGPVKGDSIVEKEEIP
FEKERKFNPDLAPGTEKVTREGQKGEKTITTPTLKNPLTGEIISKGESKEEITKDPINELTEYGP
ET
>1KPTA
LGINCRGSSQCGLSGGNLMVRIRDQACGNQGQTWCPGERRAKVCGTGNSISAYVQSTNNCISGTE
ACRHLTNLVNHGCRVCGSDPLYAGNDVSRGQLTVNYVNSC
>4EBJA
MGSSHHHHHHSSGRENLYFQGVQHTIARWVDRLREEYADAVAILLKGSYARGDAATWSDIDFDVL
VSTQDVEDYRTWIEPVGDRLVHISAAVEWVTGWERDTVDPSSWSYGLPTQETTRLMWAINDETRR
RLDRPYKTHPAAEPEVEDTVEALGKIRNAIARGDDLGVYQSAQTVAKLVPTLLIPINPPVTVSHA
RQAIEAILAFPRVPVGFAADWLTCLGLVEERSARSTAAAAERMVRGVLEMLPTDPDLLGEDIARL
MNAGLLEKYVQQ
>1LXJA
MPKIFCLADVCMVPIGTDSASISDFVALIEKKIRESPLKSTLHSAGTTIEGPWDDVMGLIGEIHE
YGHEKGYVRVHTDIRVGTRTDKHQTAQDKIDVVLKKISQ
>3TWDA
ASRLERVYQSEQAEKLLLAGVMLRDPARFDLRGTLTHGRDVEIDTNVIIEGNVTLGHRVKIGTGC
VIKNSVIGDDCEISPYTVVEDANLAAACTIGPFARLRPGAELLEGAHVGNFVEMKKARLGKGSKA
GHLTYLGDAEIGDNVNIGAGTITCNYDGANKFKTIIGDDVFVGSDTQLVAPVTVGKGATIAAGTT
VTRNVGENALAISRVPQTQKEGWRRPA
>2AUKA
GSHMAAAESSIQVKNKGSIKLSNVKSVVNSSGKLVITSRNTELKLIDEFGRTKESYKVPYGAVLA
KGDGEQVAGGETVANWDPHTMPVITEVSGFVRFTDMIDGQTITRQTDELTGLSSLVVLDSAERTA
GGKDLRPALKIVDAQGNDVLIPGTDMPAQYFLPGKAIVQLEDGVQISSGDTLARIPQESG
>3KF8B
SSKIILIPSNIPQEFPEASISNPERLRILAQVKDFIPHESTIVIDKVPTITSEQSTYINICIFNL
LEACSSRVLVPGTLVNIDAFYDGESINPVDIYEVNGANFTMENIQLIDEMNNSIGKFN
>4F9ZA
SSDGPGAAQEPTWLTDVPAAMEFIAATEVAVIGFFQDLEIPAVPILHSMVQKFPGVSFGISTDSE
VLTHYNITGNTICLFRLVDNEQLNLEDEDIESIDATKLSRFIEINSLHMVTEYNPVTVIGLFNSV
IQIHLLLIMNKASPEYEENMHRYQKAAKLFQGKILFILVDSGMKENGKVISFFKLKESQLPALAI
YQTLDDEWDTLPTAEVSVEHVQNFCDGFLSGK
>3VGPA
GPDLTEDWKEALEWMRTSLEEQNYLNPYEKPEYSVMSWWDYGNWILYVSKKAVVANNFQAGAVDA
AKFFTAKSEDEAIKIAKKRGVRYVVTADEITMKDANNTKFPAIMRIAGYNVDLMTEGEILNFFNH
TVLYRLHMENAENLTHFRLVKEFGDVKIFEVVGS

>4GEYA
ANVRLQHHHHHHHLEAEAFSSESKWMTGDWGGTRTELLDKGYDFTLDYVGEVAGNLHGGYNDDKT
ARYSDQFALGAHLDLQKILGWHDAEFKLAITERSGRNLSNDRISDPRAGQFSSVQEVWGRGQTWR
LTQMWIKQKYFDGALDVKFGRFGEGEDFNSFPCDFQNLAFCGSQVGNWVGGIWYNWPVSQWALRV
KYNITPAFFVQVGAFEQNPSNLETGNGFKLSGSGTKGAIMPMEAVWSPKVNGLPGEYRLGYYYST
AKADDVYDDVNGNPQALTGEAFKSHSSKHGWWVVAQQQVTAHGGDVNRGLSLFANFTVHDKATNV
VDNYQQVGLVYKGAFDARPKDDIGFGVARIHVNDDVKKRAELLNAQSGINDYDNPGFVPLQRTEY
NAELYYGFHVTNWLTVRPNLQYIKSPGGVDEVDNALVAGLKIQSSF
>4GB5A
SNAMDAETDRAEIIELFGRYADIADLKEFTDLPRRVHTDPLTIDFESVTGMPPMTVPLSDYGAAL
RASFGAFSATHHAITGHVVTIDSDRATIHAHVRAEHWLPAEVAGDGPDRWLVVGFYDNEAVRTAD
GWRLSSVKLTASYQENAHLARAAAAGQAG
>3LDCA
VPATRILLLVLAVIIYGTAGFHFIEGESWTVSLYWTFVTIATVGYGDYSPHTPLGMYFTCTLIVL
GIGTFAVAVERLLEFLI
>2R6JA
SGHGMEENGMKSKILIFGGTGYIGNHMVKGSLKLGHPTYVFTRPNSSKTTLLDEFQSLGAIIVKG
ELDEHEKLVELMKKVDVVISALAFPQILDQFKILEAIKVAGNIKRFLPSDFGVEEDRINALPPFE
ALIERKRMIRRAIEEANIPYTYVSANCFASYFINYLLRPYDPKDEITVYGTGEAKFAMNYEQDIG
LYTIKVATDPRALNRVVIYRPSTNIITQLELISRWEKKIGKKFKKIHVPEEEIVALTKELPEPEN
IPIAILHCLFIDGATMSYDFKENDVEASTLYPELKFTTIDELLDIFVHDPPPPASAAF
>4DN7A
MHHHHHHSSGVDLGTENLYFQSMQTEQVSLKKRAESAAEKKAAFGEDFELEKYEEGSKVSKPIED
LQSLDEESKKTLLQVGVIPSEEGRSGSFLVLDNAVSHSTLKDKNVELMSTHKAMEKYEWLKDYSW
KLVQVDADKYTAKTYLEDADGYFIRVPAGKKTSMPVQTCLMLGSKKAAQTVHNIIIVEEGATLDI
ITGCTTKKGVEEGLHLGISEMYIKKGGTLNFTMIHNWAEQIGVRPRTVVSVEEGGTYVSNYICLK
PVRSVQTYPTVRLEGEGAVTRLNTIAIAHPGSELDLGSKAIFNAPGTRAELISRTITIGGRLIAR
GEMIGNAKGAKGHLECKGLVLTDKGSQLAIPILEANVDDIELTHEAAVGKIAKDQVEYLMARGLT
EDEAVGMIIRGFLDVGIRGIPEELKEEIENTIAQTALGM
>2DWKA
GSSGSSGMANERMNLMNMAKLSIKGLIESALNLGRTLDSDYAPLQQFFVVMEHCLKHGLKAKKTF
LGQNKSFWGPLELVEKLVPEAAEITASVKDLPGLKTPVGRGRAWLRLALMQKKLSEYMKALINKK
ELLSEFYEVNALMMEEEGAIIAGLLVGLNVIDANFCMKGEDLDSQVGVID
>3B5OA
GMEFNHLTKQLNQLLAQDYVAFSITENPVVQMLSQASFAQIAYVMQQYSIFPKELVGFTELARRK
ALGAGWNGVAQELQENIDEEMGSTTGGISHYTLLADGLEEGLGVAVKNTMPSVATSKLLRTVLSL
FDRQVDYVLGATYAIEATSIPELTLIVKLVEWLHEGAIPKDLQYFFSKHLDEWEIEHEAGLRTSV
AAYIQPEEFGEFAAGFRAMIDAMQVWWQELAQEAISSEVVLSTAIAQHH
>3B34A
MGSSHHHHHHSSGENLYFQGHMTQQPQAKYRHDYRAPDYQITDIDLTFDLDAQKTVVTAVSQAVR
HGASDAPLRLNGEDLKLVSVHINDEPWTAWKEEEGALVISNLPERFTLKIINEISPAANTALEGL
YQSGDALCTQCEAEGFRHITYYLDRPDVLARFTTKIIADKIKYPFLLSNGNRVAQGELENGRHWV
QWQDPFPKPCYLFALVAGDFDVLRDTFTTRSGREVALELYVDRGNLDRAPWAMTSLKNSMKWDEE
RFGLEYDLDIYMIVAVDFFNMGAMENKGLNIFNSKYVLARTDTATDKDYLDIERVIGHEYFHNWT

GNRVTCRDWFQLSLKEGLTVFRDQEFSSDLGSRAVNRINNVRTMRGLQFAEDASPMAHPIRPDMV
IEMNNFYTLTVYEKGAEVIRMIHTLLGEENFQKGMQLYFERHDGSAATCDDFVQAMEDASNVDLS
HFRRWYSQSGTPIVTVKDDYNPETEQYTLTISQRTPATPDQAEKQPLHIPFAIELYDNEGKVIPL
QKGGHPVNSVLNVTQAEQTFVFDNVYFQPVPALLCEFSAPVKLEYKWSDQQLTFLMRHARNDFSR
WDAAQSLLATYIKLNVARHQQGQPLSLPVHVADAFRAVLLDEKIDPALAAEILTLPSVNEMAELF
DIIDPIAIAEVREALTRTLATELADELLAIYNANYQSEYRVEHEDIAKRTLRNACLRFLAFGETH
LADVLVSKQFHEANNMTDALAALSAAVAAQLPCRDALMQEYDDKWHQNGLVMDKWFILQATSPAA
NVLETVRGLLQHRSFTMSNPNRIRSLIGAFAGSNPAAFHAEDGSGYLFLVEMLTDLNSRNPQVAS
RLIEPLIRLKRYDAKRQEKMRAALEQLKGLENLSGDLYEKITKALA
>3DMAA
MLTKVIAQAHIDHFTKWFERADKIVIVSHVSPDGDAIGSSLGLYHFLDSQDKIVNVIVPNAFPDF
LKWMPGSKDILLYDRYQEFADKLIMEADVICCLDFNALKRIDEMSDIVAASPGRKIMIDHHLYPE
DFCRITISHPEISSTSELVFRLICRMGYFSDISKEGAECIYTGMMTDTGGFTYNSNNREIYFIIS
ELLSKGIDKDDIYRKVYNTYSESRLRLMGYVLSNMKVYKDYNSALISLTKEEQGKFDYIKGDSEG
FVNIPLSIKNVCFSCFLREDTEKKMIKISLRSVGKFPCNRLAAEFFNGGGHLNASGGEFYGTMEE
AVKVFEQALEKYKPLLKE
>1O75A
CGSSSHETSYGYATLSYADYWAGELGQSRDVLLAGNAEADRAGDLDAGMFDAVSRATHGHGAFRQ
QFQYAVEVLGEKVLSKQETEDSRGRKKWEYETDPSVTKMVRASASFQDLGEDGEIKFEAVEGAVA
LADRASSFMVDSEEYKITNVKVHGMKFVPVAVPHELKGIAKEKFHFVEDSRVTENTNGLKTMLTE
DSFSARKVSSMESPHDLVVDTVGTGYHSRFGSDAEASVMLKRADGSELSHREFIDYVMNFNTVRY
DYYGDDASYTNLMASYGTKHSADSWWKTGRVPRISCGINYGFDRFKGSGPGYYRLTLIANGYRDV
VADVRFLPKYEGNIDIGLKGKVLTIGGADAETLMDAAVDVFADGQPKLVSDQAVSLGQNVLSADF
TPGTEYTVEVRFKEFGSVRAKVVAQ
>3P9ZA
SNAMREIVWVHSQRIAPYKTLILNEFCYYPLELDPTPFNALIFTSKNAVFSLLETLKNSPKLKML
QNIPAYALSEPTAKTLQDHHFKVAFMGEKAHGKEFVQEIFPLLEKKSVLYLRAKEIVSSLDTILL
EHGIDFKQAVVYENKLKHLTLSEQNALKPKEKSILIFTAISHAKAFLHYFEFLENYTAISIGNTT
ALYLQEQGIPSYIAKKPSLEACLELALSLRIKEC
>3DMYA
MQQLEEALKQLAQGSGSSQALTQVRRWDSACQKLPDANLALISVAGEYAAELANQALDRNLNVMM
FSDNVTLEDEIQLKTRAREKGLLVMGPDCGTSMIAGTPLAFANVMPEGNIGVIGASGTGIQELCS
QIALAGEGITHAIGLGGRDLSREVGGISALTALEMLSADEKSEVLAFVSKPPAEAVRLKIVNAMK
ATGKPTVALFLGYTPAVARDENVWFASSLDEAARLACLLSRVTARRNAIAPVSSGFICGLYTGGT
LAAEAAGLLAGHLGVEADDTHQHGMMLDADSHQIIDLGDDFYTVGRPHPMIDPTLRNQLIADLGA
KPQVRVLLLDVVIGFGATADPAASLVSAWQKACAARLDNQPLYAIATVTGTERDPQCRSQQIATL
EDAGIAVVSSLPEATLLAAALIHPLSPAAQQHTPSLLENVAVINIGLRSFALELQSASKPVVHYQ
WSPVAGGNKKLARLLERLQGHPHHH
>3AHNA
MKFSEFRYERPNIEKLKASFQQALQSFQKASNAEEQNEAMKEINQLRNDFSTMAQICYIRHTIDT
NDEFYKQEQDFFDEVEPIVKGLVNDYYRALVSSPFRSQLEGKWGKQLFALAEAELKTYSPDIVED
LQLENKLTSEYTKLVASAKIFFEGEERTLAQLQPFVESPDRDMRKRASEARFTFFQEHEEKFDEI
YDQLVKVRTAIAQKLGFKNFVELGYARLGRTDYNAEMVAKFRKQVEKHIVPIAVKLRERQRERIG
VEKLKYYDEAFVFPTGNPMPKGDANWIIENGKKMYEELSPETGEFFRYMIEHELMDLVAKKGKAS

GGYCTYIENYKAPFIFSNFTGTSGDIDVLTHEAGHAFQVYESRHYEIPEYNWPTLEACEIHSMSM
EFFTWPWMKLFFKEDAEKYQFYHLSDALLFLPYGVAVDEFQHFVYENPNATPAERKQAWRAIERK
YMPTKDYDGNDYLERGGFWQRQSHIYTTAFYYIDYTLAQICAFQFWKRSRENYKEAWNDYLTLCR
QGGSKPFTELVRVANLISPFEDGCVQSVVGGIEGWLNSVDDQSL
>3CP7A
QNPADSPHIGKVFFSTNQGDFVCSANIVASANQSTVATAGHCLHDGNGGQFARNFVFAPAYDYGE
SEHGVWAAEELVTSAEWANRGDFEHDYAFAVLETKGGTTVQQQVGTASPIAFNQPRGQYYSAYGY
PAAAPFNGQELHSCHGTATNDPMGSSTQGIPCNMTGGSSGGPWFLGNGTGGAQNSTNSYGYTFLP
NVMFGPYFGSGAQQNYNYASTTN
>1Y43B
QSEEYCASAWVGIDGDTCETAILQTGVDFCYEDGQTSYDAWYEWYPDYAYDFSDITISEGDSIKV
TVEATSKSSGSATVENLTTGQSVTHTFSGNVEGDLCETNAEWIVEDFESGDSLVAFADFGSVTFT
NAEATSGGSTVGPSDATVMDIEQDGSVLTETSVSGDSVTVTYV
>3D1PA
MWKAVMNAWNGTESQSKNVSNIQSYSFEDMKRIVGKHDPNVVLVDVREPSEYSIVHIPASINVPY
RSHPDAFALDPLEFEKQIGIPKPDSAKELIFYCASGKRGGEAQKVASSHGYSNTSLYPGSMNDWV
SHGGDKLDL
>2O1QA
GMLKSKIKEEYVQMDQVDWKPFPAAFSTGGIRWKLLHVSPEMGSWTAIFDCPAGSSFAAHVHVGP
GEYFLTKGKMDVRGGKAAGGDTAIAPGYGYESANARHDKTEFPVASEFYMSFLGPLTFVKPDGSP
IAVIGWEDAQGAWAA
>1Z67A
SNAMGLFDEVVGAFLKGDAGKYQAILSWVEEQGGIQVLLEKLQSGGLGAILSTWLSNQQRNQSVS
GEQLESALGTNAVSDLGQKLGVDTSTASSLLAEQLPKIIDALSPQGEVSAQANNDLLSAGMELLK
GKLFR
>3DZAA
GATDSATAAPAAAATTQVQKEAADVLQVAVQGANAMRDIQFARLALFHGQPDSAKKLTDDAAALL
AADDASWAKFVKTDAKAKMIADRYVIINASIALSEDYVATPEKESAIQSANEKLAKGDQKGAIDT
LRLAGIGVIENQYLMPLNQTRKAVAQSQELLKAGKYYEANLVLKGAEEGIVVDSEMLVAGN
>2H21A
SLSPAVQTFWKWLQEEGVITAKTPVKASVVTEGLGLVALKDISRNDVILQVPKRLWINPDAVAAS
EIGRVCSELKPWLSVILFLIRERSREDSVWKHYFGILPQETDSTIYWSEEELQELQGSQLLKTTV
SVKEYVKNECLKLEQEIILPNKRLFPDPVTLDDFFWAFGILRSRAFSRLRNENLVVVPMADLINH
SAGVTTEDHAYEVKGAAGLFSWDYLFSLKSPLSVKAGEQVYIQYDLNKSNAELALDYGFIEPNEN
RHAYTLTLEISESDPFFDDKLDVAESNGFAQTAYFDIFYNRTLPPGLLPYLRLVALGGTDAFLLE
SLFRDTIWGHLELSVSRDNEELLCKAVREACKSALAGYHTTIEQDRELKEGNLDSRLAIAVGIRE
GEKMVLQQIDGIFEQKELELDQLEYYQERRLKDLGLCGENGDILENLYFQ
>2OAJA
NKNKIFSLAETNKYGMSSKPIAAAFDFTQNLLAIATVTGEVHIYGQQQVEVVIKLEDRSAIKEMR
FVKGIYLVVINAKDTVYVLSLYSQKVLTTVFVPGKITSIDTDASLDWMLIGLQNGSMIVYDIDRD
QLSSFKLDNLQKSSFFPAARLSPIVSIQWNPRDIGTVLISYEYVTLTYSLVENEIKQSFIYELPP
FAPGGDFSEKTNEKRTPKVIQSLYHPNSLHIITIHEDNSLVFWDANSGHMIMARTVFETEINVPQ
PDYIRDSSTNAAKISKVYWMCENNPEYTSLLISHKSISRGDNQSLTMIDLGYTPRYSITSYEGMK
NYYANPKQMKIFPLPTNVPIVNILPIPRQSPYFAGCHNPGLILLILGNGEIETMLYPSGIFTDKA

SLFPQNLSWLRPLATTSMAASVPNKLWLGALSAAQNKDYLLKGGVRTKRQKLPAEYGTAFITGHS
NGSVRIYDASHGDIQDNASFEVNLSRTLNKAKELAVDKISFAAETLELAVSIETGDVVLFKYEVN
QFYSVENRPESGDLEMNFRRFSLNNTNGVLVDVRDRAPTGVRQGFMPSTAVHANKGKTSAINNSN
IGFVGIAYAAGSLMLIDRRGPAIIYMENIREISGAQSACVTCIEFVIMEYGDDGYSSILMVCGTD
MGEVITYKILPASGGKFDVQLMDITNVTSKGPIHKIDAFSKETKSSCLATIPKMQNLSKGLCIPG
IVLITGFDDIRLITLGKSKSTHKGFKYPLAATGLSYISTVEKNNDRKNLTVIITLEINGHLRVFT
IPDFKEQMSEHIPFPIAAKYITESSVLRNGDIAIRVSEFQASLFSTVKEQDTLAPVSDTLYINGI
RIPYRPQVNSLQWARGTVYCTPAQLNELLGGVNRPASKYKESIIAEGSFSERSSDDN
>3OXPA
NSAMLKTLLTSDVIQVVSQAKDWRDAIAISCQPLIDNGAVEARYVEAIYRSHEAIGPYYVVGPGI
AMPHARPEDGVNRLSLALTVITEGVTFNAEGNDPVKLLIVLAATDSNSHIEAISQLAQLFDTASD
VQALLNAKTPQDILSVIARY
>4JN3A
GMSENSSVRHGLTSAQHEVWLAQQLDPRGAHYRTGSCLEIDGPLDHAVLSRALRLTVAGTETLCS
RFLTDEEGRPYRAYCPPAPEGSAAVEDPDGVPYTPVLLRHIDLSGHEDPEGEAQRWMDRDRATPL
PLDRPGLSSHALFTLGGGRHLYYLGVHHIVIDGTSMALFYERLAEVYRALRDGRAVPAAAFGDTD
RMVAGEEAYRASARYERDRAYWTGLFTDRPEPVSLTGRGGGRALAPTVRSLGLPPERTEVLGRAA
EATGAHWARVVIAGVAAFLHRTTGARDVVVSVPVTGRYGANARITPGMVSNRLPLRLAVRPGESF
ARVVETVSEAMSGLLAHSRFRGEDLDRELGGAGVSGPTVNVMPYIRPVDFGGPVGLMRSISSGPT
TDLNIVLTGTPESGLRVDFEGNPQVYGGQDLTVLQERFVRFLAELAADPAATVDEVALLT
>2F01A
AEAGITGTWYNQLGSTFIVTAGADGALTGTYESAVGNAESRYVLTGRYDSAPATDGSGTALGWTV
AWKNNYRNAHSATTWSGQYVGGAEARINTQWLLTSGTTEANAWKSTLVGHDTFTKVKPSAAS
>1M0KA
MLELLPTAVEGVSQAQITGRPEWIWLALGTALMGLGTLYFLVKGMGVSDPDAKKFYAITTLVPAI
AFTMYLSMLLGYGLTMVPFGGEQNPIYWARYADWLFTTPLLLLDLALLVDADQGTILALVGADGI
MIGTGLVGALTKVYSYRFVWWAISTAAMLYILYVLFFGFTSKAESMRPEVASTFKVLRNVTVVLW
SAYPVVWLIGSEGAGIVPLNIETLLFMVLDVSAKVGFGLILLRSRAIFGEAEAPEPSAGDGAAAT
SD
>2ESSA
GMSEENKIGTYQFVAEPFHVDFNGRLTMGVLGNHLLNCAGFHASDRGFGIATLNEDNYTWVLSRL
AIELDEMPYQYEKFSVQTWVENVYRLFTDRNFAVIDKDGKKIGYARSVWAMINLNTRKPADLLAL
HGGSIVDYICDEPCPIEKPSRIKVTSNQPVATLTAKYSDIDINGHVNSIRYIEHILDLFPIELYQ
TKRIRRFEMAYVAESYFGDELSFFCDEVSENEFHVEVKKNGSEVVCRSKVIFE
>3A16A
MGSSHHHHHHSSGLVPRGSHMESAIGEHLQCPRTLTRRVPDTYTPPFPMWVGRADDALQQVVMGY
LGVQFRDEDQRPAALQAMRDIVAGFDLPDGPAHHDLTHHIDNQGYENLIVVGYWKDVSSQHRWST
STPIASWWESEDRLSDGLGFFREIVAPRAEQFETLYAFQEDLPGVGAVMDGISGEINEHGYWGSM
RERFPISQTDWMQASGELRVIAGDPAVGGRVVVRGHDNIALIRSGQDWADAEADERSLYLDEILP
TLQSGMDFLRDNGPAVGCYSNRFVRNIDIDGNFLDLSYNIGHWASLDQLERWSESHPTHLRIFTT
FFRVAAGLSKLRLYHEVSVFDAADQLYEYINCHPGTGMLRDAVTIAEH
>3UA3A
MASMSNRTYADNLFPQQVAEQHEEQMSSGSSPKSNSPSRSISSVEAANSRIHIGWMATTLDVAEN
LDRHVATFCTRLGEFKYNFVVYPIGGVVRAFWTPNGSAENHPPVIDLPDVQLRNDLWESYVVGKI

SPWIDCDSSDPAFASLSEEHLLKELSYICYLGLQTMAIELTRISSPRTAAILKKWIWTRNSRFTV
WVQLPSAIEKCKDYDAFTIEHVDLWTIWADFRKNCGNFSGVYFQVALTISSELPDELTELKLVDR
WKAEPLAAFVIESGLFISGRNGEASIPSAHINLLKHLWTTDALRIVLRATTDTFKYNTSIKSEYS
QALRHAVRNVNYRSRPDVGEGSNDSTHYLNVIEYKDVLQAPLQPLSENLDSGVYNTFEQDQIKYD
VYGEAVVGALKDLGADGRKTVVIYLLGGGRGPIGTKILKSEREYNNTFRQGQESLKVKLYIVEKN
PNAIVTLKYMNVRTWKRRVTIIESDMRSLPGIAKDRGFEQPDIIVSELLGSFGDNELSPECLDGV
TGFLKPTTISIPQKYTSYVKPIMSTHIHQTIKAQSIPYLSRAIPSHGRGEPELDEDEMWIQKYPQ
GHVRNNMDQIYVVYLSKYIPLAETTKPVFTFEHPNFMNSSNERSDSIEFVMDRNADLMGFAGYFD
LQLYKTVMLSIEPSTHTPGMVSWFPAVIPLRDQLRVGEGDRISLKIDRKVDNTGVWYEWHVEKKK
TNGESVSTPIQNPNGESYYMRMLEHHHHHH

>2HO2A

GSDLPAGWMRVQDTSGTYYWHIPTGTTQWEPPGRASPS

>1W7CA

ASAECVSNENVEIEAPKTNIWTSLAKEEVQEVLDLLHSTYNITEVTKADFFSNYVLWIETLKPNK
TEALTYLDEDGDLPPRNARTVVYFGEGEEGYFEELKVGPLPVSDETTIEPLSFYNTNGKSKLPFE
VGHLDRIKSAAKSSFLNKNLNTTIMRDVLEGLIGVPYEDMGCHSAAPQLHDPATGATVDYGTCNI
NTENDAENLVPTGFFFKFDMTGRDVSQWKMLEYIYNNKVYTSAEELYEAMQKDDFVTLPKIDVDN
LDWTVIQRNDSAPVRHLDDRKSPRLVEPEGRRWAYDGDEEYFSWMDWGFYTSWSRDTGISFYDIT
FKGERIVYELSLQELIAEYGSDDPFNQHTFYSDISYGVGNRFSLVPGYDCPSTAGYFTTDTFEYD
EFYNRTLSYCVFENQEDYSLLRHTGASYSAITQNPTLNVRFISTIGNYDYNFLYKFFLDGTLEVS
VRAAGYIQAGYWNPETSAPYGLKIHDVLSGSFHDHVLNYKVDLDVGGTKNRASQYVMKDVDVEYP
WAPGTVYNTKQIAREVFENEDFNGINWPENGQGILLIESAEETNSFGNPRAYNIMPGGGGVHRIV
KNSRSGPETQNWARSNLFLTKHKDTELRSSTALNTNALYDPPVNFNAFLDDESLDGEDIVAWVNL
GLHHLPNSNDLPNTIFSTAHASFMLTPFNYFDSENSRDTTQQVFYTYDDETEESNWEFYGNDWSS
CGVEVAEPNFEDYTYGRGTRINKKMTNSDEVY

>3A72A

SSPTSLTNVTIFSPPSDYIVPRTLYPRNEQLPNGDLLATWENYSPEPPAVYFPIYRSKDHGKTWN
EISRVHDTVNGYGLRYQPFLYSLPERVGSFKKGTLLLAGSSIPTDLSSTDIVLYASQDDGMTWDF
VSHIAAGGEARPNNGLTPVWEPFLLANKGKLICYYSDQRDNATYGQTMVHQVTNDLKNWGPVVED
VTYPTYTDRPGMPVVTKLPNGQYFYVYEYGSFFGTETYSFPLYYRLSSDPENIASAPGQRLVVSS
GTQPTSSPYAVWTPYGGENGTIIVSSGTQGTLFINKALGEGEWTEIPCPEEHGYTRALRVLSEDG
GRYLVVNSAGVLLGENNRVSVSVMDLKEVL

>3CBZA

GSHMNIITVTLNMEKYNFLGISIVGQSNERGDGGIYIGSIMKGGAVAADGRIEPGDMLLQVNDMN
FENMSNDDAVRVLRDIVHKPGPIVLTVAKSGGGSGNEVWIDGP

>2QR4A

MSLSDQEFDEKYLELSEELKQSEKHKGTLDQGASQFLNAIEFVLRVYRQTEVIYVYAHLKNDQDT
GNTDYQALYARASSLFSKVSEAVSWFEPEILQLSDDQIWQYFKEEPKLEVYRHYIQQIVDNRAHV
LSAEQESLLAGAGEIFDASSDTFAVLNNADLVFPTIEGENGEIVQLSHGVYGQLLESTDRRVREA
AFKGLYSVYEQFRNTFASTLGTHIKGHNFKAKVRNYSSAREASLSNNHIPESVYDTLVDVVNKHL
PLLHRYMELRKRLLEVEKLHMYDLYTPVLGEAPITFTYEEAKEKALEALKPMGEEYMAIVEKAFS
ERWIDVVENKGKRSGAYSSGSYDTNPYILLNWHDTLDQLFTLVHEMGHSVHSYFTRSNQPYVYGD
YSIFLAEIASTTNENILTEYLLETEKDPRVRAYVLNHYLDGFKGTVFRQTQFAEFEHFMHTEDEK
GVPLTSEYLSDSYGKLNAKYYGPAVEEDPEIKFEWSRIPHFYYNYYVFQYSTGFSAASALAKKIL

NQEPEALENYLAYLKAGNSDYPVEVMKKAGVDMTQAAYIEDAMSMFEQRLNELEELIDREGHHHH
HH
>2XFVA
ALEEVVRYLGPHNEIPLTLTRDSETGHFLLKHFLPILQQYHDTGNINETNPDSFPTDEERNKLLA
HYGIAVNTDDRGELWIELEKCLQLLNMLNLFGLFQDAFEFEEPETDQDEEDPSHSKLPEN
>2XSKA
MGSSQITFNTTQQGDMYTIIPEVTLTQSCLCRVQILSLREGSSGQSQTKQEKTLSLPANQPIALT
KLSLNISPDDRVKIVVTVSDGQSLHLSQQWPPSSEKSLEHHHHHH
>4H5BA
METALLTLDTLAKYLQEKEVQLDIEENGGQRFIRMGWRFEMGDAAVLVSVNDGPNNTSRLEITCV
TQKTYADRRAEVAMMLNDRNRERAFARSIDQEGNVWLEYVGFYPTLAEMPQETFDTLFGGVLMHF
QDDYAALEGYVPQEGMQIQQPQA
>2NRJA
SLSEIEQTNNGDTALSANEARMKETLQKAGLFAKSMNAYSYMLIKNPDVNFEGITINGYVDLPGR
IVQDQKNARAHAVTWDTKVKKQLLDTLNGIVEYDTTFDNYYETMVEAINTGDGETLKEGITDLRG
EIQQNQKYAQQLIEELTKLRDSIGHDVRAFGSNKELLQSILKNQGADVDADQKRLEEVLGSVNYY
KQLESDGFNVMKGAILGLPIIGGIIVGVARDNLGKLEPLLAELRQTVDYKVTLNRVVGVAYSNIN
EMHKALDDAINALTYMSTQWHDLDSQYSGVLGHIENAAQKADQNKFKFLKPNLNAAKDSWKTLRT
DAVTLKEGIKELKVETVTPQK
>4FIBA
MKVGSQVIINTSHMKGMKGAEATVTGAYDTTAYVVSYTPTNGGQRVDHHKWVIQEEIKDAGDKTL
QPGDQVILEASHMKGMKGATAEIDSAEKTTVYMVDYTSTTSGEKVKNHKWVTEDELLEHHHHHH
>4D9IA
MSVFSLKIDIADNKFFNGETSPLFSQSQAKLARQFHQKIAGYRPTPLCALDDLANLFGVKKILVK
DESKRFGLNAFKMLGGAYAIAQLLCEKYHLDIETLSFEHLKNAIGEKMTFATTTDGNHGRGVAWA
AQQLGQNAVIYMPKGSAQERVDAILNLGAECIVTDMNYDDTVRLTMQHAQQHGWEVVQDTAWEGY
TKIPTWIMQGYATLADEAVEQMREMGVTPTHVLLQAGVGAMAGGVLGYLVDVYSPQNLHSIIVEP
DKADCIYRSGVKGDIVNVGGDMATIMAGLACGEPNPLGWEILRNCATQFISCQDSVAALGMRVLG
NPYGNDPRIISGESGAVGLGVLAAVHYHPQRQSLMEKLALNKDAVVLVISTEGDTDVKHYREVVW
EGKHAVAP
>2CJGA
MAAVVKSVALAGRPTTPDRVHEVLGRSMLVDGLDIVLDLTRSGGSYLVDAITGRRYLDMFTFVAS
SALGMNPPALVDDREFHAELMQAALNKPSNSDVYSVAMARFVETFARVLGDPALPHLFFVEGGAL
AVENALKAAFDWKSRHNQAHGIDPALGTQVLHLRGAFHGRSGYTLSLTNTKPTITARFPKFDWPR
IDAPYMRPGLDEPAMAALEAEALRQARAAFETRPHDIACFVAEPIQGEGGDRHFRPEFFAAMREL
CDEFDALLIFDEVQTGCGLTGTAWAYQQLDVAPDIVAFGKKTQVCGVMAGRRVDEVADNVFAVPS
RLNSTWGGNLTDMVRARRILEVIEAEGLFERAVQHGKYLRARLDELAADFPAVVLDPRGRGLMCA
FSLPTTADRDELIRQLWQRAVIVLPAGADTVRFRPPLTVSTAEIDAAIAAVRSALPVVT
>3RONA
MENLNHCPLEDIKVNPWKTPQSTARVITLRVEDPNEINNLLSINEIDNPNYILQAIMLANAFQNA
LVPTSTDFGDALRFSMPKGLEIANTITPMGAVVSYVDQNVTQTNNQVSVMINKVLEVLKTVLGVA
LSGSVIDQLTAAVTNTFTNLNTQKNEAWIFWGKETANQTNYTYNVLFAIQNAQTGGVMYCVPVGF
EIKVSAVKEQVLFFTIQDSASYNVNIQSLKFAQPLVSSSQYPIADLTSAINGTL
>1AVGI

AEGDDCSIEKAMGDFKPEEFFNGTWYLAHGPGVTSPAVCQKFTTSGSKGFTQIVEIGYNKFESNV
KFQCNQVDNKNGEQYSFKCKSSDNTEFEADFTFISVSYDNFALVCRSITFTSQPKEDRYLVFERT
KSDTDPDAKEIC
>4ENEA
MRRRQLIRQLLERDKTPLAILFMAAVVGTLVGLAAVAFDKGVAWLQNQRMGALVHTADNYPLLLT
VAFLCSAVLAMFGYFLVRKYAPEAGGSGIPEIEGALEDQRPVRWWRVLPVKFFGGLGTLGGGMVL
GREGPTVQIGGNIGRMVLDIFRLKGDEARHTLLATGAAAGLAAAFNAPLAGILFIIEEMRPQFRY
TLISIKAVFIGVIMSTIMYRIFNHEVALIDVGKLSDAPLNTLWLYLILGIIFGIFGPIFNKWVLG
MQDLLHRVHGGNITKWVLMGGAIGGLCGLLGFVAPATSGGGFNLIPIATAGNFSMGMLVFIFVAR
VITTLLCFSSGAPGGIFAPMLALGTVLGTAFGMVAVELFPQYHLEAGTFAIAGMGALLAASIRAP
LTGIILVLEMTDNYQLILPMIITGLGATLLAQFTGGKPLYSAILARTLAKQEAEQK
>3IO3A
MDLELEPTLESIVQHDSLKWIFVGGKGGVGKTTTSSSVAVQLALAQPNEQFLLISTDPAHNLSDA
FCQKFGKDARKVEGLPNLSCMEIDPEAAMSDLQQQASQYNNDPNDPLKSMMSDMTGSIPGIDEAL
SFMEVLKHIKNQKVLEGEDNSNAISYKTIIFDTAPTGHTLRFLQLPSTLEKLLSKFKDLSGKLGP
MLSMMGGGQQQDIFEKLNEVQKNVSEVNEQFTNPELTTFICVCISEFLSLYETERMIQELMSYNM
DVNSIVVNQLLFAEGDDHSCKRCESRWKMQKKYLDQMGELYEDYHLVKMPLLGCEIRGVENLKKF
SKFLLKPYDPKADSDIVFDLEEK
>3KSNA
GDAASDLKSRLDKVSSFHASFTQKVTDGSGAAVQEGQGDLWVKRPNLFNWHMTQPDESILVSDGK
TLWFYNPFVEQATATWLKDATGNTPFMLIARNQSSDWQQYNIKQNGDDFVLTPKASNGNLKQFTI
NVGRDGTIHQFSAVEQDDQRSSYQLKSQQNGAVDAAKFTFTPPQGVTVDDQRK
>3NYCA
HHHHHHPIEADYLVIGAGIAGASTGYWLSAHGRVVVLEREAQPGYHSTGRSAAHYTVAYGTPQVR
ALTAASRAFFDNPPAGFCEHPLLSPRPEMVVDFSDDPEELRRQYESGKALVPQMRLLDAEQACSI
VPVLRRDKVFGATYDPTGADIDTDALHQGYLRGIRRNQGQVLCNHEALEIRRVDGAWEVRCDAGS
YRAAVLVNAAGAWCDAIAGLAGVRPLGLQPKRRSAFIFAPPPGIDCHDWPMLVSLDESFYLKPDA
GMLLGSPANADPVEAHDVQPEQLDIATGMYLIEEATTLTIRRPEHTWAGLRSFVADGDLVAGYAA
NAEGFFWVAAQGGYGIQTSAAMGEASAALIRHQPLPAHLREHGLDEAMLSPRRLSP
>3ZN6A
MGVFDRIRGALGRGLDVFRGDLPQVQPPAPQPAPAPAITPAAVQVGGWGFAWIDNEDFSPTGLAW
RSGEYFALAQMKTPETAHFRIAAQERRLRIYLRGQKVVNGRNLSDPDSRTVNLPFLMQTPQGAPT
LPSTYHPDVAVWAKVGSTWQPCVITAINYSTGDVTFTEPAGVTASDGIEIYYVHGDGQFRLRVAR
DAGGVDDSAATVFNQSFSTMHSVDQNNVETMIAWPQQVELVPGTRLVLEVFTTQVPMVWNERSGH
YIQIAAMGRRIEVLDKGGLQRLAELEARGGL
>3PUIA
MVDCNYSVACNVMVPMRDGVRLAVDLYRPDADGPVPVLLVRNPYDKFDVFAWSTQSTNWLEFVRD
GYAVVIQDTRGLFASEGEFVPHVDDEADAEDTLSWILEQAWCDGNVGMFGVSYLGVTQWQAAVSG
VGGLKAIAPSMASADLYRAPWYGPGGALSVEALLGWSALIGTGLITSRSDARPEDAADFVQLAAI
LNDVAGAASVTPLAEQPLLGRLIPWVIDQVVDHPDNDESWQSISLFERLGGLATPALITAGWYDG
FVGESLRTFVAVKDNADARLVVGPWSHSNLTGRNADRKFGIAATYPIQEATTMHKAFFDRHLRGE
TDALAGVPKVRLFVMGIDEWRDETDWPLPDTAYTPFYLGGSGAANTSTGGGTLSTSISGTESADT
YLYDPADPVPSLGGTLLFHNGDNGPADQRPIHDRDDVLCYSTEVLTDPVEVTGTVSARLFVSSSA
VDTDFTAKLVDVFPDGRAIALCDGIVRMRYRETLVNPTLIEAGEIYEVAIDMLATSNVFLPGHRI

MVQVSSSNFPKYDRNSNTGGVIAREQLEEMCTAVNRIHRGPEHPSHIVLPIIKRKLAAALEHHHH
HH
>1YLIA
SANFTDKNGRQSKGVLLLRTLAMPSDTNANGDIFGGWIMSQMDMGGAILAKEIAHGRVVTVAVES
MNFIKPISVGDVVCCYGQCLKVGRSSIKIKVEVWVKKVASEPIGERYCVTDAVFTFVAVDNNGRS
RTIPRENNQELEKALALISEQPL
>2AHFA
MWQQAIGDALGITARNLKKFGDRFPHVSDGSNKYVLNDNTDWTDGFWSGILWLCYEYTGDEQYRE
GAVRTVASFRERLDRFENLDHHNIGFLYSLSAKAQWIVEKDESARKLALDAADVLMRRWRADAGI
IQAWGPKGDPENGGRIIIDCLLNLPLLLWAGEQTGDPEYRRVAEAHALKSRRFLVRGDDSSYHTF
YFDPENGNAIRGGTHQGNTDGSTWTRGQAWGIYGFALNSRYLGNADLLETAKRMARHFLARVPED
GVVYWDFEVPQEPSSYRDSSASAITACGLLEIASQLDESDPERQRFIDAAKTTVTALRDGYAERD
DGEAEGFIRRGSYHVRGGISPDDYTIWGDYYYLEALLRLERGVTGYWYERGR
>2YYYA
MPAKVLINGYGSIGKRVADAVSMQDDMEVIGVTKTKPDFEARLAVEKGYKLFVAIPDNERVKLFE
DAGIPVEGTILDIIEDADIVVDGAPKKIGKQNLENIYKPHKVKAILQGGEKAKDVEDNFNALWSY
NRCYGKDYVRVVSCNTTGLCRILYAINSIADIKKARIVLVRRAADPNDDKTGPVNAITPNPVTVP
SHHGPDVVSVVPEFEGKILTSAVIVPTTLMHMHTLMVEVDGDVSRDDILEAIKKTPRIITVRAED
GFSSTAKIIEYGRDLGRLRYDINELVVWEESINVLENEIFLMQAVHQESIVIPENIDCIRAMLQM
EEDNFKSIEKTNKAMGIQ
>4DOIA
MSSSNACASPSPFPAVTKLHVDSVTFVPSVKSPASSNPLFLGGAGVRGLDIQGKFVIFTVIGVYL
EGNAVPSLSVKWKGKTTEELTESIPFFREIVTGAFEKFIKVTMKLPLTGQQYSEKVTENCVAIWK
QLGLYTDCEAKAVEKFLEIFKEETFPPGSSILFALSPTGSLTVAFSKDDSIPETGIAVIENKLLA
EAVLESIIGKNGVSPGTRLSVAERLSQLMMKNKDEKEVSDHSVEEKLAKEN
>4DOXA
MSKSSMSTPNIAFPAITQEQMSSIKVDPTSNLLPSQEQLKSVSTLMVAAKVPAASVTTVALELVN
FCYDNGSSAYTTVTGPSSIPEISLAQLASIVKASGTSLRKFCRYFAPIIWNLRTDKMAPANWEAS
GYKPSAKFAAFDFFDGVENPAAMQPPSGLTRSPTQEERIANATNKQVHLFQAAAQDNNFASNSAF
ITKGQISGSTPTIQFLPPPETSTTRHHHHHH
>4GYTA
GMSLDNDSLHLPKYDDFVQSISVLALTMSGSELHGIMCGYLCAGADSQGEAYIRALLNNKKDEQS
RNALLSMFSVFSISQQQMNNFDFEFEMLLPDDDESLVTRAQAFSEWCEGFTQGLTIAGVGMEQFY
EEESQDALQHLMEFAELDCESLEVGEEDERALMEVSEYTRMAVLRLHSDLVLHERELGDSGTTH
>1MSPA
AQSVPPGDINTQPSQKIVFNAPYDDKHTYHIKITNAGGRRIGWAIKTTNMRRLSVDPPCGVLDPK
EKVLMAVSCDTFNAATEDLNNDRITIEWTNTPDGAAKQFRREWFQGDGMVRRKNLPIEYNL
>1U19A
XMNGTEGPNFYVPFSNKTGVVRSPFEAPQYYLAEPWQFSMLAAYMFLLIMLGFPINFLTLYVTVQ
HKKLRTPLNYILLNLAVADLFMVFGGFTTTLYTSLHGYFVFGPTGCNLEGFFATLGGEIALWSLV
VLAIERYVVVCKPMSNFRFGENHAIMGVAFTWVMALACAAPPLVGWSRYIPEGMQCSCGIDYYTP
HEETNNESFVIYMFVVHFIIPLIVIFFCYGQLVFTVKEAAAQQQESATTQKAEKEVTRMVIIMVI
AFLICWLPYAGVAFYIFTHQGSDFGPIFMTIPAFFAKTSAVYNPVIYIMMNKQFRNCMVTTLCCG
KNPLGDDEASTTVSKTETSQVAPA

>2YMOA
GSAMGNLTCDFNDVYKLEFHPNQQTSVTKLCNLTPNVLEKVTIKCGSDKLNYNLYPPTCFEEVYA
SRNMMHLKKIKEFVIGSSMFMRRSLTPNKINEVSFRIPPNMMPEKPIYCFCENKKTITINGSNGN
PSSKKDIINRGIVEIIIPSLNEKVKGCDFTTSESTIFSKGYSINEISNKSSNNQQDIVCTVKAHA
NDLIGFKCPSNYSVEPHDCFVSAFNLSGKNENLENKLKLTNIIMDHYNNTFYSRLPSLISDNWKF
FCVCSKDNEKKLVFTVEASISSAAALVPR
>1A9XA
MPKRTDIKSILILGAGPIVIGQACEFDYSGAQACKALREEGYRVINVNSNPATIMTDPEMADATY
IEPIHWEVVRKIIEKERPDAVLPTMGGQTALNCALELERQGVLEEFGVTMIGATADAIDKAEDRR
RFDVAMKKIGLETARSGIAHTMEEALAVAADVGFPCIIRPSFTMGGSGGGIAYNREEFEEICARG
LDLSPTKELLIDESLIGWKEYEMEVVRDKNDNCIIVCSIENFDAMGIHTGDSITVAPAQTLTDKE
YQIMRNASMAVLREIGVETGGSNVQFAVNPKNGRLIVIEMNPRVSRSSALASKATGFPIAKVAAK
LAVGYTLDELMNDITGGRTPASFEPSIDYVVTKIPRFNFEKFAGANDRLTTQMKSVGEVMAIGRT
QQESLQKALRGLEVGATGFDPKVSLDDPEALTKIRRELKDAGADRIWYIADAFRAGLSVDGVFNL
TNIDRWFLVQIEELVRLEEKVAEVGITGLNADFLRQLKRKGFADARLAKLAGVREAEIRKLRDQY
DLHPVYKRVDTCAAEFATDTAYMYSTYEEECEANPSTDREKIMVLGGGPNRIGQGIEFDYCCVHA
SLALREDGYETIMVNCNPETVSTDYDTSDRLYFEPVTLEDVLEIVRIEKPKGVIVQYGGQTPLKL
ARALEAAGVPVIGTSPDAIDRAEDRERFQHAVERLKLKQPANATVTAIEMAVEKAKEIGYPLVVR
ASYVLGGRAMEIVYDEADLRRYFQTAVSVSNDAPVLLDHFLDDAVEVDVDAICDGEMVLIGGIME
HIEQAGVHSGDSACSLPAYTLSQEIQDVMRQQVQKLAFELQVRGLMNVQFAVKNNEVYLIEVNPR
AARTVPFVSKATGVPLAKVAARVMAGKSLAEQGVTKEVIPPYYSVKEVVLPFNKFPGVDPLLGPE
MRSTGEVMGVGRTFAEAFAKAQLGSNSTMKKHGRALLSVREGDKERVVDLAAKLLKQGFELDATH
GTAIVLGEAGINPRLVNKVHEGRPHIQDRIKNGEYTYIINTTSGRRAIEDSRVIRRSALQYKVHY
DTTLNGGFATAMALNADATEKVISVQEMHAQIK
>3BJQA
GMSQMSPGQARVVDPILSTHARGYRQSTLIGKKLFPVAPVAQYGGKILTFGKEAFRLYNTKRAPG
ANTKRIDFGYEGDPYSIVPSALEAKVPRELMRDASQVPGIDLGARSVNTVLRIMALAHEHECAQI
ALDPAKYNADHKVKLVGSARWTSPDSDPTKDVETAKEAIADSIGMEPNRLMLSRKALSACKYHPK
LIERVKYTRAESITIDMLKALWEVEEIVVGTARVATGANDSFGDVWGPDVWLGYVSDNPDPSVEE
PSFGYTYQIEGHPLVEVPYWDNNAKSWIYGVSDDNTPALSGMLAGYLIEDAGLPAA
>3LLPA
MTANGTAEAVQIQFGLINCGNKYLTAEAFGFKVNASASSLKKKQIWTLEQPPDEAGSAAVCLRSH
LGRYLAADKDGNVTCEREVPGPDCRFLIVAHDDGRWSLQSEAHRRYFGGTEDRLSCFAQTVSPAE
KWSVHIAMHPQVNIYSVTRKRYAHLSARPADEIAVDRDVPWGVDSLITLAFQDQRYSVQTADHRF
LRHDGRLVARPEPATGYTLEFRSGKVAFRDCEGRYLAPSGPSGTLKAGKATKVGKDELFALEQSC
AQVVLQAANERNVSTRQGMDLSANQDEETDQETFQLEIDRDTKKCAFRTHTGKYWTLTATGGVQS
TASSKNASCYFDIEWRDRRITLRASNGKFVTSKKNGQLAASVETAGDSELFLMKLINRPIIVFRG
EHGFIGCRKVTGTLDANRSSYDVFQLEFNDGAYNIKDSTGKYWTVGSDSAVTSSGDTPVDFFFEF
CDYNKVAIKVGGRYLKGDHAGVLKASAETVDPASLWEY
>3U99A
DGRGLSRAIPQNAEYTAECGSCHMAYPANLLPADKWRAITANLENHFGDNASLDPQVTARIEEYL
VQHAAQNGKVMKNSTPLLAGAGPQKITEQAFFIRKHDEIPRRMVQDNPKVGSFSQCSNCHNLAEK
GIFDEDTVNIPGFGRWDD
>2IN5A

MTHSQQSMVDTFRASLFDNQDITVADQQIQALPYSTMYLRLNEGQRIFVVLGYIEQEQSKWLSQD
NAMLVTHNGRLLKTVKLNNNLLEVTNSGQDPLRNALAIKDGSRWTRDILWSEDNHFRSATLSSTF
SFAGLETLNIAGRNVLCNVWQEEVTSTRPEKQWQNTFWVDSATGQVRQSRQMLGAGVIPVEMTFL
KPAPLEHHHHHH
>4G4SO
MLFKQWNDLPEPKHLLDLPEISKNLQSLEVCPVPKVEFPQDLDVPQYSTAVITTKIMNPLFPKNL
LQLTSIGEIKTTLTVKSPSLPQSSGKHSWNYDENFPNEVDPDQKNDTADETVYGFSFPIYSFGKT
LLFSMEENFISISPIFGNMISRSIISQLAQFSPDIIVIGTSDKIASMKVMTENECTLQPPEFITG
FIGSVLTQLIVGPSKGLKFKCLVAPSEGPNGFEKLSLSDMGSLVDLCGQWLGFEPSRYSEECYRL
WRCDSAAIGAQSGLYI
>1UUNA
GLDNELSLVDGQDRTLTVQQWDTFLNGVFPLDRNRLTREWFHSGRAKYIVAGPGADEFEGTLELG
YQIGFPWSLGVGINFSYTTPNILIDDGDITRPPFGLNSVITPNLFPGVSISADLGNGPGIQEVAT
FSVDVSGAEGGVAVSNAHGTVTGAAGGVLLRPFARLIASTGDSVTTYGEPWNMN
>1ZJCA
GSHMTNYKEKLQQYAELLVKVGMNVQPKQPVFIRSSVETLELTHLIVEEAYHCGASDVRVVYSDP
TLKRLKFENESVEHFANHEIKSYDVEARMDYVKRGAANLALISEDPDLMDGIDSQKLQAFQQQNA
RAFKGYMESVQKNQFPWVVAAFPSKAWAKRVYPELSVEEAYIKFIDEVFDIVRIDGNDPVENWRQ
HIANLSVYAQKLQQKNYHALHYVSEGTDLTVGLAKNHIWEDATSYVNGKEQAFIANIPTEEVFTA
PDRNRVDGYVTNKLPLSYNGTIIDQFKLMFKDGEIIDFSAEKGEAVLKDLINTDEGSRRLGEVAL
VPDDSPISNRNTIFYNTLFDENAACHLAIGSAYAFNIQGGTEMTVEEKIASGLNDSNVHVDFMIG
SSDLTIYGIFEDGSKELVFENGNWASTF
>2C71A
MPANKLVALTFDDGPDNVLTARVLDKLDKYNVKATFMVVGQRVNDSTAAIIRRMVNSGHEIGNHS
WSYSGMANMSPDQIRKSIADTNAVIQKYAGTTPKFFRPPNLETSPTLFNNVDLVFVGGLTANDWI
PSTTAEQRAAAVINGVRDGTIILLHDVQPEPHPTPEALDIIIPTLKSRGYEFVTLTELFTLKGVP
IDPSVKRMYNSVPLEHHHHHH
>4EE6A
GSMSESADLTELYSIIEKTAQVVDVTASHDKVWPILNAFQDVIADSVISFRASTGSSADDLDCRF
TMLPKGLDPYARALEHGLTPKTDHPVGSLLKEVHENLPITSCGVDFGVAGGFTKTWSFPSAEKLG
KVSELVKLPSIPDAVAANRDFFEKWGIADMVSTVGIDYSKRTMNLYFGGGVGDRVPAGVFEEKGV
RAILGELGLAAPSEELLKFCERSFVIYVTLSWDSPKINRFTYSVMTPEPLGLPVDLAPTFERLIK
SAPYDTEGRNYVYGIASTPKGEYHKIASYYQWQKRVEKLLRSDG
>4E6XA
ERLSTLIHQRMQEAKVPALSVSVTIKGVRQRFVYGVADVASQKANTLDTVYELGSMSKAFTGLVV
QILIQEGRLRQGDDIITYLPEMRLNYQGKPASLTVADFLYHTSGLPFSTLARLENPMPGSAVAQQ
LRNENLLFAPGAKFSYASANYDVLGAVIENVTGKTFTEVIAERLTQPLGMSATVAVKGDEIIVNK
ASGYKLGFGKPVLFHAPLARNHVPAAYIHSTLPDMEIWIDAWLHRKALPATLREAMSNSWRGNSD
VPLAADNRILYASGWFIDQNQGPYISHGGQNPNFSSCIALRPDQQIGIVALANMNSNLILQLCAD
IDNYLRIGKYA
>1JUVA
MIKLVFRYSPTKTVDGFNELAFGLGDGLPWGRVKKDLQNFKARTEGTIMIMGAKTFQSLPTLLPG
RSHIVVCDLARDYPVTKDGDLAHFYITWEQYITYISGGEIQVSSPNAPFETMLDQNSKVSVIGGP
ALLYAALPYADEVVVSRIVKRHRVNSTVQLDASFLDDISKREMVETHWYKIDEVTTLTESVYK

>4GQ2M
RMNELKHAVVPIDLQSFCLEGTLALWVPALENDSEDDSEAIETADDNEKLFKKECVAYDAGVYTS
NKSKGSQTLRWSIFQNRTLTIFDVSLNSKKEPLSKFNVKIHFPSNVMKDGVAFSFSEHSDTTIIY
AITHARVLYYIRLSKTWFQLPDARLDDDWCLCYRPISFLNQKPDLMAAISTSEICVSFFNGGLTK
IILNPKDASHYEQHIDDSSYLFSLKKYLSLQAFKADYRSPNTIISMIFLSTYNVLVMLSLDYKLK
VLDLSTNQCVETIELSQTILPLQSFPYLTSDHTTNSFIALYYPDNSHGSFSIYKLNANAHSFKLN
VVIEKGIIPPSLPDDEFIPWMLSDFQLISSEGSQSKFLLIIAWKSNLNTVIQKCNLSLDQDESFS
CVWSHSLDSFSLIEKTFFDVPTNMSSGDISEIWLQHIFAHNTSIESIQVALLSFQNSSSQVSKNK
LDKFGALTISELKNAVLSSIVSTIQIEPNSDLTGYDYYEYKRLLYNEWERFAKLVAYLDHFGDEI
LSINFDPSNAVTYINYANKVAFIRDPYLIESFDEEPLTKLISSLETDDPSLIEGYQILDLGRSLH
SCMSFSTLSEIRYSLRELVQDLPSYSLFDTLWVFYDKHIYPNVDPDYISTLIDTLVSLENPMRDI
DSLIQRLRSFDIYNHSAQSPSLFLCASVARVLDSILKKFQVSIEGFIFLLSLITSQQDYELQSKF
AGCDKLFLSLLEDWRLVSFLLENSALLLEKFEEEDVDSTNCNLNTMEALASVNTALQFFSALNYS
ECFSESQISPLHATVISSLSAIFIRDDTENDLVTELVEKLFLFKQYNACMQLIGWLNSDPIAVYL
KALIYLKSKEAVKAVRCFKTTSLVLYSHTSQFAVLREFQEIAEKYHHQNLLSCYYLHLSKKLFEE
SAYIDALEFSLLADASKETDDEDLSIAITHETLKTACAAG
>2VK8A
MSEITLGKYLFERLKQVNVNTVFGLPGDFNLSLLDKIYEVEGMRWAGNANELNAAYAADGYARIK
GMSCIITTFGVGELSALNGIAGSYAEHVGVLHVVGVPSISAQAKQLLLHHTLGNGDFTVFHRMSA
NISETTAMITDIATAPAEIDRCIRTTYVTQRPVYLGLPANLVDLNVPAKLLQTPIDMSLKPNDAE
SEKEVIDTILVLDKDAKNPVILADACCSRHDVKAETKKLIDLTQFPAFVTPMGKGSIDEQHPRYG
GVYVGTLSKPEVKEAVESADLILSVGALLSDFNTGSFSYSYKTKNIVEFHSDHMKIRNATFPGVQ
MKFVLQKLLTTIADAAKGYKPVAVPARTPANAAVPASTPLKQEWMWNQLGNFLQEGDVVIAETGT
SAFGINQTTFPNNTYGISQVLWGSIGFTTGATLGAAFAAEEIDPKKRVILFIGDGSLQLTVQEIS
TMIRWGLKPYLFVLNNDGYTIQKLIHGPKAQYNEIQGWDHLSLLPTFGAKDYETHRVATTGEWDK
LTQDKSFNDNSKIRMIEVMLPVFDAPQNLVEQAKLTAATNAKQ
>4GXWA
MVKGTPGNVPAARTGIEITAAHRAFFHALPKVELHCHLLGAVRHDTFVALAQRSGAPIERAEIDA
FYARGEKPVGVLHVLRALDRYLLTRPDDLRRIAYEYLEDAAAHNVRHAEFFWNPTGTVRVSGIPY
ADAQAAIVTGMRDAARDFGIGARLIPSIDREQDPDEAVAIVDWMKANRADEVAGIGIDYRENDRP
PELFWKAYRDARAAGFRTTAHAGEFGMPWRNVETAVDLLHVDRVDHGYTIVDNPELCARYAERGI
VFTVVPTNSYYLRTLPPDQWAERHPMRKMPGLGLKIHPNTDDPTLHKVNPSEAWELMFSHFGFTI
ADLKQFMLNGIDGAWVDDDTKAAWRAAWAPEFDMLADTLAADKLAAALEHHHHHH
>1JLYA
XAGLPVIMCLKSNNHQKYLRYQSDNIQQYGLLQFSADKILDPLAQFEVEPSKTYDGLVHIKSRYT
NKYLVRWSPNHYWITASANEPDENKSNWACTLFKPLYVEEGNMKKVRLLHVQLGHYTQNYTVGGS
FVSYLFAESSQIDTGSKDVFHVIDWKSIFQFPKGYVTFKGNNGKYLGVITINQLPCLQFGYDNLN
DPKVAHQMFVTSNGTICIKSNYMNKFWRLSTDDWILVDGNDPRETNEAAALFRSDVHDFNVISLL
NMQKTWFIKRFTSGKPGFINCMNAATQNVDETAILEIIELGQNN
>1TE2A
GHMSTPRQILAAIFDMDGLLIDSEPLWDRAELDVMASLGVDISRRNELPDTLGLRIDMVVDLWYA
RQPWNGPSRQEVVERVIARAISLVEETRPLLPGVREAVALCKEQGLLVGLASASPLHMLEKVLTM
FDLRDSFDALASAEKLPYSKPHPQVYLDCAAKLGVDPLTCVALEDSVNGMIASKAARMRSIVVPA
PEAQNDPRFVLANVKLSSLTELTAKDLLGGS

>1MKFA
LTLGLAPALSTHSSGVSTQSVDLSQIKRGDEIQAHCLTPAETEVTECAGILKDVLSKNLHELQGL
CNVKNKMGVPWVSVEELGQEIITGRLPFPSVGGTPVNDLVRVLVVAESNTPEETPEEEFYAYVEL
QTELYTFGLSDDNVVFTSDYMTVWMIDIPKSYVDVGMLTRATFLEQWPGAKVTVMIPYSSTFTWC
GELGAISEESAPQPSLSARSPVCKNSARYSTSKFCEVDGCTAETGMEKMSLLTPFGGPPQQAKMN
TCPCYYKYSVSPLPAMDHLILADLAGLDSLTSPVYVMAAYFDSTHENPVRPSSKLYHCALQMTSH
DGVWTSTSSEQCPIRLVEGQSQNVLQVRVAPTSMPNLVGVSLMLEGQQYRLEYFGDH
>1EI9A
DPPAPLPLVIWHGMGDSCCNPLSMGAIKKMVEKKIPGIHVLSLEIGKTLREDVENSFFLNVNSQV
TTVCQILAKDPKLQQGYNAMGFSQGGQFLRAVAQRCPSPPMVNLISVGGQHQGVFGLPRCPGESS
HICDFIRKTLNAGAYNKAIQERLVQAEYWHDPIREDIYRNHSIFLADINQERGVNESYKKNLMAL
KKFVMVKFLNDTIVDPVDSEWFGFYRSGQAKETIPLQESTLYTQDRLGLKAMDKAGQLVFLALEG
DHLQLSEEWFYAHIIPFLE
>3NO2A
GSSPQHLLVGGSGWNKIAIINKDTKEIVWEYPLEKGWECNSVAATKAGEILFSYSKGAKMITRDG
RELWNIAAPAGCEMQTARILPDGNALVAWCGHPSTILEVNMKGEVLSKTEFETGIERPHAQFRQI
NKNKKGNYLVPLFATSEVREIAPNGQLLNSVKLSGTPFSSAFLDNGDCLVACGDAHCFVQLNLES
NRIVRRVNANDIEGVQLFFVAQLFPLQNGGLYICNWQGHDREAGKGKHPQLVEIDSEGKVVWQLN
DKVKFGMISTICPIRE
>4KQCA
SNADPYIAVVSKGFQHKFWVTVRDGAEAAAKQNGVKISFVGPETESDSKIQQDLLDSEINKNPDA
IAFAAVTGDFTEQIKRIKEKNIPLIGFDSGILPDQAQGAVLATASTDNRAAAAIVADKMFEALKT
RIAAFTSDNKAKIAVLQLDNSDTGIGRAEGFVKRFTELADGDAATAGKYALQVIVPTTQNEADIA
NEVNALRGKSVLGIYLSNEAMARGFLVVYKSAEAGAANTIVGDAQDGGDLVVMGFDSGKPQLDAI
RNGIIQGSVTQDPYSIGFQAVTLAYKASKGESVSNIDTGAKWYDKTNIDDPEIAKLLYE
>2OGBA
STIEYNEILEWVNSLQPARVTRWGGMISTPDAVLQAVIKRSLVESGCPASIVNELIENAHERSWP
QGLATLETRQMNRRYYENYVAKRIPGKQAVVVMACENQHMGDDMVQEPGLVMIFAHGVEEI
>1U5XA
GSKKHSVLHLVPVNITSKADSDVTEVMWQPVLRRGRGLEAQGDIVRVWDTGIYLLYSQVLFHDVT
FTMGQVVSREGQGRRETLFRCIRSMPSDPDRAYNSCYSAGVFHLHQGDIITVKIPRANAKLSLSP
HGTFLGFVKL
>1L9XA
MGSSHHHHHHSSGLVPRGSHMRPHGDTAKKPIIGILMQKCRNKVMKNYGRYYIAASYVKYLESAG
ARVVPVRLDLTEKDYEILFKSINGILFPGGSVDLRRSDYAKVAKIFYNLSIQSFDDGDYFPVWGT
CLGFEELSLLISGECLLTATDTVDVAMPLNFTGGQLHSRMFQNFPTELLLSLAVEPLTANFHKWS
LSVKNFTMNEKLKKFFNVLTTNTDGKIEFISTMEGYKYPVYGVQWHPEKAPYEWKNLDGISHAPN
AVKTAFYLAEFFVNEARKNNHHFKSESEEEKALIYQFSPIYTGNISSFQQCYIFD
>3PNRB
MGHHHHHHHHHHSSGHIEGRHMGDEKCGKSLKLGNISNQTNQETITQSLSVGEILCIDLEGNAGT
GYLWVLLGIHKDEPIINPENFPTKLTKKSFFSEEISVTQPKKYKIDEHDSSKNVNREIESPEQKE
SDSKPKKPQMQLLGGPDRMRSVIKGHKPGKYYIVYSYYRPFSPTSGANTKIIYVTVQ
>1FN9A
MEVCLPNGHQVVDLINNAFEGRVSIYSAQEGWDKTISAQPDMMVCGGAVVCMHCLGVVGSLQRKL

KHLPHHRCNQQIRHQDYVDVQFADRVTAHWKRGMLSFVAQMHEMMNDVSPDDLDRVRTEGGSLVE
LNWLQVDPNSMFRSIHSSWTDPLQVVDDLDTKLDQYWTALNLMIDSSDLIPNFMMRDPSHAFNGV
KLGGDARQTQFSRTFDSRSSLEWGVMVYDYSELEHDPSKGRAYRKELVTPARDFGHFGLSHYSRA
TTPILGKMPAVFSGMLTGNCKMYPFIKGTAKLKTVRKLVEAVNHAWGVEKIRYALGPGGMTGWYN
RTMQQAPIVLTPAALTMFPDTIKFGDLNYPVMIGDPMILG
>1XCRA
GSACAEFSFHVPSLEELAGVMQKGLKDNFADVQVSVVDCPDLTKEPFTFPVKGICGKTRIAEVGG
VPYLLPLVNQKKVYDLNKIAKEIKLPGAFILGAGAGPFQTLGFNSEFMPVIQTESEHKPPVNGSY
FAHVNPADGGCLLEKYSEKCHDFQCALLANLFASEGQPGKVIEVKAKRRTGPLNFVTCMRETLEK
HYGNKPIGMGGTFIIQKGKVKSHIMPAEFSSCPLNSDEEVNKWLHFYEMKAPLVCLPVFVSRDPG
FDLRLEHTHFFSRHGEGGHYHYDTTPDIVEYLGYFLPAEFLYRIDQPKETHSIGRD
>3O4PA
MEIPVIEPLFTKVTEDIPGAEGPVFDKNGDFYIVAPEVEVNGKPAGEILRIDLKTGKKTVICKPE
VNGYGGIPAGCQCDRDANQLFVADMRLGLLVVQTDGTFEEIAKKDSEGRRMQGCNDCAFDYEGNL
WITAPAGEVAPADYTRSMQEKFGSIYCFTTDGQMIQVDTAFQFPNGIAVRHMNDGRPYQLIVAET
PTKKLWSYDIKGPAKIENKKVWGHIPGTHEGGADGMDFDEDNNLLVANWGSSHIEVFGPDGGQPK
MRIRCPFEKPSNLHFKPQTKTIFVTEHENNAVWKFEWQRNGKKQYCETLKFGIF
>2NLVA
GMDKLVKYQELVKKLLTNYASDDVSDQDVEVQLILDTERNHYQWMNVGWQGLNRIYRCVIHFDIK
DGKIWLQQNLTDRNPAEELVMMGVPREDIVLGLQAPYKRQYTDYGVA
>3H6RA
MASLEDGTYRLRAVTTHNPDPGVGGEYATVEGARQPVKAEPSTPPFSEQQIWQVTRNSDGQYTIK
YQGLNAPFEYGFSYDQLEPNAPVIAGDPKEYILQLVPSTADVYIIRAPIQRVGVDVEVGVQGNTL
VYKFFPVDGSGGDRPAWRFTRE
>1BRTA
PFITVGQENSTSIDLYYEDHGTGQPVVLIHGFPLSGHSWERQSAALLDAGYRVITYDRRGFGQSS
QPTTGYDYDTFAADLNTVLETLDLQDAVLVGFSTGTGEVARYVSSYGTARIAKVAFLASLEPFLL
KTDDNPDGAAPQEFFDGIVAAVKADRYAFYTGFFNDFYNLDENLGTRISEEAVRNSWNTAASGGF
FAAAAAPTTWYTDFRADIPRIDVPALILHGTGDRTLPIENTARVFHKALPSAEYVEVEGAPHGLL
WTHAEEVNTALLAFLAK
>4I8IA
GNGCHANNDTIKVLAIGNSFSQDAVEQYLHELGEAEGITMIIGNMFIGGCSLERHVQNIRNNAPA
YAYRKVEKDGEKTETRSMTIEKALADEKWDYISVQQASPLSGIYDSYKASLPELVNYIRERIGKE
TVLMMHQTWAYATNANHTGFKNYDQNQMKMYTSIVDAVKKAANLVGIKKIIPSGTAIQNARTSFI
GDHMNRDGYHLDLTIGRYTAACTWFEALTHRNVTENPYSPEGIDPIHKKAAQMAAHNAILYPDKV
TELTELKKIAD
>3D8UA
SNAYSIALIIPSLFEKACAHFLPSFQQALNKAGYQLLLGYSDYSIEQEEKLLSTFLESRPAGVVL
FGSEHSQRTHQLLEASNTPVLEIAELSSKASYLNIGVDHFEVGKACTRHLIEQGFKNVGFIGARG
NHSTLQRQLHGWQSAMIENYLTPDHFLTTHEAPSSQLGAEGLAKLLLRDSSLNALVCSHEEIAIG
ALFECHRRVLKVPTDIAIICLEGSSMGEHAYPSLTSAEFDYERMGTKAAEKLLHAIKGEPEERPT
SMGFKLKRRASTAIN
>2WP7A
MEPPNLYPVKLYVYDLSKGLARRLSPIMLGKQLEGIWHTSIVVHKDEFFFGSSGISSCTPGGTLL

GPPDSVVDVGNTEVTEEIFLEYLSSLGESLFRGEAYNLFEHNCNTFSNEVAQFLTGRKIPSYITD
LPSEVLSTPFGQALRPFLDSIQIQPPGGNSVGRPNGQS
>1YQ5A
GGVTDALSLMYSTSTGGPASIAANALTDFDLSGALTVNSVGTGLTKSAAGIQLAAGKSGLYQITM
TVKNNTVTTGNYLLRVKYGSSDFVVACPASSLTAGGTISLLIYCNVLGVVSLDVLKFSLCNDGAA
LSNYIINITAAKIN
>1YKDA
VTEVEQKLQIVHQTLSMLDSHGFENILQEMLQSITLKTGELLGADRTTIFLLDEEKQELWSIVAA
GEGDRSLEIRIPADKGIAGEVATFKQVVNIPFDFYHDPRSIFAQKQEKITGYRTYTMLALPLLSE
QGRLVAVVQLLNKLKPYSPPDALLAERIDNQGFTSADEQLFQEFAPSIRLILESSRSFYIATQKQ
RAAAAMMKAVKSLSQSSLDLEDTLKRVMDEAKELMNADRSTLWLIDRDRHELWTKITQDNGSTKE
LRVPIGKGFAGIVAASGQKLNIPFDLYDHPDSATAKQIDQQNGYRTCSLLCMPVFNGDQELIGVT
QLVNKKKTGEFPPYNPETWPIAPECFQASFDRNDEEFMEAFNIQAGVALQNAQLFATVKQQEQGS
RSHHHHHH
>1K94A
SVYTYFSAVAGQDGEVDAEELQRCLTQSGINGTYSPFSLETCRIMIAMLDRDHTGKMGFNAFKEL
WAALNAWKENFMTVDQDGSGTVEHHELRQAIGLMGYRLSPQTLTTIVKRYSKNGRIFFDDYVACC
VKLRALTDFFRKRDHLQQGSANFIYDDFLQGTMAI
>4G9FD
KITQTQPGMFVQEKEAVTLDCTYDTSDQSYGLFWYKQPSSGEMIFLIYQGSYDEQNATEGRYSLN
FQKARKSANLVISASQLGDSAMYFCAMRDLRDNFNKFYFGSGTKLNVKPNIQNPDPAVYQLRDSK
SSDKSVCLFTDFDSQTNVSQSKDSDVYITDKCVLDMRSMDFKSNSAVAWSNKSDFACANAFNNSI
IPEDTFFPS
>3BT3A
SLENERLIKMSRFSERGYVVRENGPVYFTKDMDKTVKWFEEILGWSGDIVARDDEGFGDYGCVFD
YPSEVAVAHLTPFRGFHLFKGEPIKGVAGFMMIEGIDALHKYVKENGWDQISDIYTQPWGARECS
ITTTDGCILRFFESIQEG
>1G6HA
MRDTMEILRTENIVKYFGEFKALDGVSISVNKGDVTLIIGPNGSGKSTLINVITGFLKADEGRVY
FENKDITNKEPAELYHYGIVRTFQTPQPLKEMTVLENLLIGEICPGESPLNSLFYKKWIPKEEEM
VEKAFKILEFLKLSHLYDRKAGELSGGQMKLVEIGRALMTNPKMIVMDEPIAGVAPGLAHDIFNH
VLELKAKGITFLIIEHRLDIVLNYIDHLYVMFNGQIIAEGRGEEEIKNVLSDPKVVEIYIGE
>3L39A
MGSDKIHHHHHHMKNSFFSKFTPKEPKFFPLLKQLSDVLSASSVLLVESMEHDLPTERADYYKQI
KDMEREGDRLTHLIFDELSTTFITPFDREDIHDLASCMDDVIDGINSSAKRIVIYNPRPISESGK
ELSRLIHEEAINIGKAMDELETFRKNPKPLRDYCTQLHDIENQADDVYELFITKLFEEEKDCIEL
IKIKEIMHELEKTTDAAEHVGKILKNLIVKYS
>3FFRA
GMNNKIYFTPGPSELYPTVRQHMITALDEKIGVISHRSKKFEEVYKTASDNLKTLLELPSNYEVL
FLASATEIWERIIQNCVEKKSFHCVNGSFSKRFYEFAGELGREAYKEEAAFGKGFYPADITVPAD
AEIICLTHNETSSGVSMPVEDINTFRDKNKDALIFVDAVSSLPYPKFDWTKIDSVFFSVQKCFGL
PAGLGVWILNDRVIEKSKALLAKRKSIGTYHTIPSMLEKARVNQTPETPNAMNIFLLGKVTGDML
QISADGIRKQTEEKAALINTYIESSKVFSFGVEDAKLRSMTTIVANTTMLPGEINKILEPFDMAV
GAGYGSKKETQIRIANFPAHSLEQVHKLVQTLKEKIG

>3OYVA
GSDDDNPTVDPANIDYTPENASSWHNYMRNVAALLKTDATNLYNAWNSSYKGGESYASLFKAHSG
SPYASALSCVEEIVDKCAEIANEVGTAKIGDPYNLYKAGNTEEALYAVESWYSWHSRDDYTNNIY
SIRNAYYGSLDGNINANSLSTVIAGANSSLDTKIKNAIQKAAKAIQDIPQPFRNHIPSNETVAAM
DACAELESILKNDLKSYIANNSNNINTDAVLNPVVTQYVDAVVVPTYKSLKEKNDALYNAVIVLA
DNPSNSAFETACDAWITAREPWEKSEAFLFGPVDEMGLDPNMDSWPLDQNAIVQILNSQSWSDLE
WSEGDDEAAVESAQNVRGFHTLEFLLYKNGEPRKVQ
>1SWVA
MDRMKIEAVIFAWAGTTVDYGCFAPLEVFMEIFHKRGVAITAEEARKPMGLLKIDHVRALTEMPR
IASEWNRVFRQLPTEADIQEMYEEFEEILFAILPRYASPINGVKEVIASLRERGIKIGSTTGYTR
EMMDIVAKEAALQGYKPDFLVTPDDVPAGRPYPWMCYKNAMELGVYPMNHMIKVGDTVSDMKEGR
NAGMWTVGVILGSSELGLTEEEVENMDSVELREKIEVVRNRFVENGAHFTIETMQELESVMEHIE
KQELIIS
>3TUFA
GPAMSPESKNAVQMQSEKSASDSGEVATEKAPAKQDTKEKSGTETEKGKEDGTKGTKDSSADKET
SAEASEKGTVVTETADDDLFTTYRLDLEDARSKEREELNAIVSSDDATAKEKSEAYDKMTALSEV
EGTEKQLETLIKTQGYEDALVNAEGDKINITVKSDKHSKSKATAIIDLVAKEIKTMKDVAVTFEP
SK
>1R4PA
REFTIDFSTQQSYVSSLNSIRTEISTPLEHISQGTTSVSVINHTPPGSYFAVDIRGLDVYQARFD
HLRLIIEQNNLYVAGFVNTATNTFYRFSDFTHISVPGVTTVSMTTDSSYTTLQRVAALERSGMQI
SRHSLVSSYLALMEFSGNTMTRDASRAVLRFVTVTAEALRFRQIQREFRQALSETAPVYTMTPGD
VDLTLNWGRISNVLPEYRGEDGVRVGRISFNNISAILGTVAVILNCHHQGARSVRAVNEESQPEC
QITGDRPVIKINNTLWESNTAAAFLNRKSQFLYTTGK
>2WZPR
MLEANVYDNFNPNYYNISDFSMPNGKKEKRGLPIPKARCQVINYELWETGYLYTSSATLTVSVEV
GDIVQILFPEVVPIEEALGKKKKLNLDMVYLVTDVDESNKATLKNYFWAMIESLDVPNAITKTTN
FAIIDYLIDPNKNNLMSYGYFFNSSIFAGKATINRKAETSSAHDVAKRIFSKVQFQPTTTIQHAP
SETDPRNLLFINFASRNWNRKRITTRVDIKQSVTMDTETIVERSAYNFAVVFVKNKATDDYTDPP
KMYIAKNNGDVIDYSTYHGDGTDLPDVRTAKTLFYDRDDHGNPPELSTIKVEISPSTIVTRLIFN
QNELLPLYVNDLVDIWYEGKLYSGYIADRVKTEFNDRLIFVESGDKPNVI
>4ACJA
GNEPSDLLEAEQIEKLAKHLPPRTIGYPWNLAFSTSKHGMSIKTLYRAMQDQDSPMLLVIKDSDG
QIFGALASEPFKVSEGFYGTGETFLFTFYPEFEAYKWTGDNLFFIKGDMDSLAFGGGSGEFGLWL
DGDLYHGRNHSCKTFGNPMLSMKEDFFVQDIEIWSFE
>3GNFB
GPLGMATEEAIIRIPPYHYIHVLDQNSNVSRVEVGPKTYIRQDNERVLFAPVRMVTVPPRHYCIV
ANPVSRDAQSSVLFDVTGQVRLRHADQEIRLAQDPFPLYPGELLEKDITPLQVVLPNTALHLKAL
LDFEDKNGDKVMAGDEWLFEGPGTYIPQKEVEVVEIIQATVIKQNQALRLRARKECFDRDGKERV
TGEEWLVRSVGAYLPAVFEEVLDLVDAVILTEKTALHLRARQNFKDLRGVAHRTGEEWLVTVQDT
EAHVPDVYEEVLGVVPITTLGPRHYCVILDPMGPDGKNQLGQKRVVKGEKSFFLQPGERLERGIQ
DVYVLSEQQGLLLKALQPLEEGEGEERVAHQAGDRWLIRGPLEYVPSAKVEVVEERQAIPLD
>4JX2A
GHTNNTNNKGLSASINNGVGSSSSNNTYVTPQAFWNLYFDFTGDETPGYPKGKINISQTLFQSEM

KKNSSLAQQNEGQLILFINSTLYIYNSDRQLKLKQLMRTAPNSGFTEMTAISHIGPALMYLAKIK
ENGDASWKSQMENLLKDIQAVKVINAQTPNNWLEQVNAPAWKPHLTTIHNMIDYACSMAGNYMSD
VLNEKLSFDMASLQNDFLNGNKTYPIPYNNVMIGTFMLTALQSMDQLHSKISQLKIDWPHAKVII
RFVAGSNVSAGVSKGSNWLVPFVQALSNNKLATDRIYITPYAAVKPSLGAQELTQADYNYYNNTV
WGARHNRRIIANEVFTNITSIFLPDRPAIPGDYTYSKPPKIEDFLMRLKFSLAEPTEMLSNTVGF
WMAGELAEKNWNYNKISIPGITTGFPEGISTYPNNNPVIQR
>3DHAA
GRISMTVKKLYFIPAGRCMLDHSSVNSALTPGKLLNLPVWCYLLETEEGPILVDTGMPESAVNNE
GLFNGTFVEGQILPKMTEEDRIVNILKRVGYEPDDLLYIISSHLHFDHAGGNGAFTNTPIIVQRT
EYEAALHREEYMKECILPHLNYKIIEGDYEVVPGVQLLYTPGHSPGHQSLFIETEQSGSVLLTID
ASYTKENFEDEVPFAGFDPELALSSIKRLKEVVKKEKPIIFFGHDIEQEKSCRVFPEYI
>3K2IA
SMSATLILEPPGRCCWNEPVRIAVRGLAPEQRVTLRASLRDEKGALFRAHARYCADACGELDLER
APALGGSFAGLEPMGLLWALEPEKPFWRFLKRDVQIPFVVELEVLDGHDPEPGRLLCQAQHERHF
LPPGVWRQSVRAGRVRATLFLPPGPGPFPGIIDIFGIGGGLLEYRASLLAGHGFATLALAYYNFE
DLPNNMDNISLEYFEEAVCYMLQHPQVKGPGIGLLGISLGADICLSMASFLKNVSATVSINGSGI
SGNTAINYKHSSIPPLGYDLRRIKVAFSGLVDIVDIRNALVGGYKNPSMIPIEKAQGPILLIVGQ
DDHNWRSELYAQTVSERLQAHGKEKPQIICYPGTGHYIEPPYFPLCPASLHRLLNKHVIWGGEPR
AHSKAQEDAWKQILAFFCKHLGGTQKTAVPKL
>4G8AA
RSPWDYKDDDDKLAAANSSIPESWEPCVEVVPNITYQCMELNFYKIPDNLPFSTKNLDLSFNPLR
HLGSYSFFSFPELQVLDLSRCEIQTIEDGAYQSLSHLSTLILTGNPIQSLALGAFSGLSSLQKLV
AVETNLASLENFPIGHLKTLKELNVAHNLIQSFKLPEYFSNLTNLEHLDLSSNKIQSIYCTDLRV
LHQMPLLNLSLDLSLNPMNFIQPGAFKEIRLHKLTLRNNFDSLNVMKTCIQGLAGLEVHRLVLGE
FRNEGNLEKFDKSALEGLCNLTIEEFRLAYLDYYLDGIIDLFNCLTNVSSFSLVSVTIERVKDFS
YNFGWQHLELVNCKFGQFPTLKLKSLKRLTFTSNKGGNAFSEVDLPSLEFLDLSRNGLSFKGCCS
QSDFGTISLKYLDLSFNGVITMSSNFLGLEQLEHLDFQHSNLKQMSEFSVFLSLRNLIYLDISHT
HTRVAFNGIFNGLSSLEVLKMAGNSFQENFLPDIFTELRNLTFLDLSQCQLEQLSPTAFNSLSSL
QVLNMSHNNFFSLDTFPYKCLNSLQVLDYSLNHIMTSKKQELQHFPSSLAFLNLTQNDFACTCEH
QSFLQWIKDQRQLLVEVERMECATPSDKQGMPVLSLNITCQMTGHHHHHH
>2CWSA
MPAAAPGKNFDLSHWKLQLPDANTTEISSANLGLGYTSQYFYTDTDGAMTFWAPTTGGTTANSSY
PRSELREMLDPSNSKVNWGWQGTHTMKLSGKTVQLPSSGKIIVAQIHGIMDDGTNAPPLVKAVFQ
DGQLDMQVKQNSDGTGSDVHNYFTGIKLGDLYNMEIRVTDGVAYVTMNGDTRSVDFVGKDAGWKN
LKYYFKAGNYVQDNTSTGGSAIAKLYSLSVSHSNLEHHHHHH
>3HRGA
GMIDFTKSKQYTLSIRLSTDGFSFSIYNPINDNSQSLFEKEVDTSLSLTANLKNVFHESDFLSYS
YKRVNIMIASKRFTMIPLELFEEEQAELLFYHNHQKRENEIVMYNILKKNNVVIIFGIDKSTYTF
LNEQYPEARFYSQSTPLIEYFSIKSRLGNSKKMYASVRKDAIDIYCFERGQLLLANSFECMQTED
RIYYLLYVWKQLEFNQERDELHLTGTLSDKETLMNELKKFILQVFIMNPANNIDMQALLTCE
>3GA4A
MATASHNIDDILQLKDDTGVITVTADNYPLLSRGVPGYFNILYITMRGTNSNGMSCQLCHDFEKT
YHAVADVIRSQAPQSLNLFFTVDVNEVPQLVKDLKLQNVPHLVVYPPAESNKQSQFEWKTSPFYQ
YSLVPENAENTLQFGDFLAKILNISITVPQAFNVQEEFHHHHHHHHHH

>3PQHA
GSFIPKIATATDSSEEVDSEKVIISNNKQTYASFDPNGNISVYNTQGMKIDMTPNSIVLTDAGGG
KLTLQGGTMTYKGGTVNLNGLTITPDGRMTDSGGIGLHTHTHPVRGVETGGSTVTSDKPNGG
>1QMYA
MELTLYNGEKKTFYSRPNNHDNAWLNAILQLFRYVEEPFFDWVYSSPENLTLEAIKQLEDLTGLE
LHEGGPPALVIWNIKHLLHTGIGTASRPSEVCVVDGTDMSLADFHAGIFLKGQEHAVFACVTSNG
WYAIDDEDFYPWTPDPSDVLVFVPYDQEPLNGEWKAK
>2BJIA
MADPWQECMDYAVTLAGQAGEVVREALKNEMNIMVKSSPADLVTATDQKVEKMLITSIKEKYPSH
SFIGEESVAAGEKSILTDNPTWIIDPIDGTTNFVHGFPFVAVSIGFVVNKKMEFGIVYSCLEDKM
YTGRKGKGAFCNGQKLQVSHQEDITKSLLVTELGSSRTPETVRIILSNIERLLCLPIHGIRGVGT
AALNMCLVAAGAADAYYEMGIHCWDVAGAGIIVTEAGGVLLDVTGGPFDLMSRRVIASSNKTLAE
RIAKEIQIIPLQRDDED
>3I57A
MQKVHVQYIDGETDQMLRQDDLDGYTDETIPYSTAEGIKKFEGDGYELFKDNFPAGEKFDNDDTN
DQFYTVIFKHHRENVDPNHSSADGTKGTKTLTETVHYKYANGTKAAEDQTAQVTFTRNGVLDDVT
GIVAWGKWNEASQSYKALTSPTIAGYAPSEAVVKRSSNSDAEQGPTLTVIYTADA
>1U7LA
MATALYTANDFILISLPQNAQPVTAPGSKTDSWFNETLIGGRAFVSDFKIPEFKIGSLDTLIVES
EELSKVDNQIGASIGKIIEILQGLNETSTNAYRTLPINNMPVPEYLENFQWQTRKFKLDKSIKDL
ITLISNESSQLDADVRATYANYNSAKTNLAAAERKKTGDLSVRSLHDIVKPEDFVLNSEHLTTVL
VAVPKSLKSDFEKSYETLSKNVVPASASVIAEDAEYVLFNVHLFKKNVQEFTTAAREKKFIPREF
NYSEELIDQLKKEHDSAASLEQSLRVQLVRLAKTAYVDVFINWFHIKALRVYVESVLRYGLPPHF
NIKIIAVPPKNLSKCKSELIDAFGFLGGNAFMKDKKGKINKQDTSLHQYASLVDTEYEPFVMYII
NL
>2IGSA
GHMAEINIYQNPGQSLANIYKGFARQCNPGFVFPEAQTIEAWDIPLRLHPEFIPGGDISKADQQY
STLLAQEIANGVTIGFRMVNEKERVCNVEILPLLTSMAQNLDRIKARFGSGYLDRFKGSPNVYPT
DVGFSTDASGGISQESGLLVSYGVNLRTLTPGTWQAMTLPEDIKALVGPGVGLRLDAPNFSDVFN
TIKSGLRYTTAVTLLLAYFAAIGS
>3MGAA
SNAMKEALATGSEAWWRTKTGPEWIREKDGNYRVTFWWRDPQGNETHSPIRRVWVYITGVTDHHQ
NAQPQTMARIAGTDVWRWSTALSANWRGSYCFIPTERDDVFAAFAPGETPDRNVLREGWRQLLPQ
AIADPLNSQSWRGGRGHAVSALEMPDAPLQPGWDRPETPYSPPLMMQWHSERLGNSRRVWILTTG
DEAPEERPLAILLDGQFWAENMPVWPALASLTHQRLLPGAVYLLIDAIDTQHRSQELPCNADFWL
AVQQELLPQVRAVTPFSDDAGRTVVAGQSFGGLSALYAGLNWPTRFGCVLSQSGSFWWPHRITPP
EGEVITRLKTGALCARGLRIVLEAGVREPIVFQANQALYAQLNTSQQSIFWRQVDGGHDALCWRG
GLTQGLMLLWQPLIDTL
>2PKEA
GMTPIAQRDGQAIQLVGFDGDDTLWKSEDYYRTAEADFEAILSGYLDLGDSRMQQHLLAVERRNL
KIFGYGAKGMTLSMIETAIELTEARIEARDIQRIVEIGRATLQHPVEVIAGVREAVAAIAADYAV
VLITKGDLFHQEQKIEQSGLSDLFPRIEVVSEKDPQTYARVLSEFDLPAERFVMIGNSLRSDVEP
VLAIGGWGIYTPYAVTWAHEQDHGVAADEPRLREVPDPSGWPAAVRALDAQAGRQQ
>1F32A

QFLFSMSTGPFICTVKDNQVFVANLPWTMLEGDDIQVGKEFAARVEDCTNVKHDMAPTCTKPPPF
CGPQDMKMFNFVGCSVLGNKLFIDQKYVRDLTAKDHAEVQTFREKIAAFEEQQENQPPSSGMPHG
AVPAGGLSPPPPPSFCTVQ
>1QF8A
MSSSEEVSWISWFCGLRGNEFFCEVDEDYIQDKFNLTGLNEQVPHYRQALDMILDLEPDEELEDN
PNQSDLIEQAAEMLYGLIHARYILTNRGIAQMLEKYQQGDFGYCPRVYCENQPMLPIGLSDIPGE
AMVKLYCPKCMDVYTPKSSRHHHTDGAYFGTGFPHMLFMVHPEYRPKRPANQ
>3IX1A
ALETVEVMLDWYPNAVHTFLYVAIENGYFAEEGLDVDIVFPTNPTDPIQLTASGAIPLALSYQPD
VILARSKDLPVVSVASVVRSPLNHVMFLAEQDFDSPADLVGLTVGYPGIPVNEPILKTMVEAAGG
DYEQVHLMDVGFELGASIVSGRADAVVGTYINHEYPVLKHEGHDISYFNPVDYGVPEYDELVLIS
NEAYVEESGEVLAAFWRAALKGYEWMVENPDEALNVLLTNQDEANFPLIQEVEEESLSILLEKME
NPNGPFGGQDAESWEEVISWLDAHDWLEQPVVAEDAFSSITD
>2D42A
AIINLLRELEIYGMQYANSHQYTYGSSYSDDTNPIRIAGLDARIPDPIVTDPVNHIVLDRRIITN
TTSNSLEGVFSFSNAYTSRTSSQTRDGVTAGTNITGKYFANLFFEQVGLSGRIAFEGAVTNENKY
TLDATQDFRDSQTIRVPPFHRATGVYTLEQGAFEKMTVLECVVSGNGIIRYYRTLPDNSYTEIVQ
RVNIIDVLQANGTPGFTISKEQNRAYFTGEGTISGQIGLQTFIDVVIEPLPGHA
>3FVSA
MAKQLQARRLDGIDYNPWVEFVKLASEHDVVNLGQGFPDFPPPDFAVEAFQHAVSGDFMLNQYTK
TFGYPPLTKILASFFGELLGQEIDPLRNVLVTVGGYGALFTAFQALVDEGDEVIIIEPFFDCYEP
MTMMAGGRPVFVSLKPGPIQNGELGSSSNWQLDPMELAGKFTSRTKALVLNTPNNPLGKVFSREE
LELVASLCQQHDVVCITDEVYQWMVYDGHQHISIASLPGMWERTLTIGSAGKTFSATGWKVGWVL
GPDHIMKHLRTVHQNSVFHCPTQSQAAVAESFEREQLLFRQPSSYFVQFPQAMQRCRDHMIRSLQ
SVGLKPIIPQGSYFLITDISDFKRKMPDLPGAVDEPYDRRFVKWMIKNKGLVAIPVSIFYSVPHQ
KHFDHYIRFCFVKDEATLQAMDEKLRKWKVEL
>1RYPL
TTTLAFRFQGGIIVAVDSRATAGNWVASQTVKRVIEINPFLLGTMAGGAADCQFWETWLGSQCRL
HELREKERISVAAASKILSNLVYQYKGAGLSMGTMICGYTRKEGPTIYYVDSDGTRLKGDIFCVG
SGQTFAYGVLDSNYKWDLSVEDALYLGKRSILAAAHRDAYSGGSVNLYHVTEDGWIYHGNHDVGE
LFWKVKEEEGSFNNVIG
>3VXJA
ANDTILPLNNIQGDILVGMKKQKERFVFFQVNDATSFKTALKTYVPERITSAAILISDPSQQPLA
FVNLGFSNTGLQALGITDDLGDAQFPDGQFADAANLGDDLSQWVAPFTGTTIHGVFLIGSDQDDF
LDQFTDDISSTFGSSITQVQALSGSARPGDQAGHEHFGFLDGISQPSVTGWETTVFPGQAVVPPG
IILTGRDGDTGTRPSWALDGSFMAFRHFQQKVPEFNAYTLANAIPANSAGNLTQQEGAEFLGARM
FGRWKSGAPIDLAPTADDPALGADPQRNNNFDYSDTLTDETRCPFGAHVRKTNPRQDLGGPVDTF
HAMRSSIPYGPETSDAELASGVTAQDRGLLFVEYQSIIGNGFRFQQINWANNANFPFSKPITPGI
EPIIGQTTPRTVGGLDPLNQNETFTVPLFVIPKGGEYFFLPSISALTATIAA
>1JOVA
MKTTLLKTLTPELHLVQHNDIPVLHLKHAVGTAKISLQGAQLISWKPQNAKQDVLWLSEVEPFKN
GNAIRGGVPICYPWFGGVKQPAHGTARIRLWQLSHYYISVHKVRLEFELFSDLNIIEAKVSMVFT
DKCHLTFTHYGEESAQAALHTYFNIGDINQVEVQGLPETCFNSLNQQQENVPSPRHISENVDCIY
SAENMQNQILDKSFNRTIALHHHNASQFVLWNPWHKKTSGMSETGYQKMLCLETARIHHLLEFGE

SLSVEISLKG

>1UUQA

MVAESNSAVAPTANVATSPAHEHFVRVNGGHFELQGKPYVITGVNMWYAAYLGAPNEVGDRDRLA
KELDNLKAIGVNNLRVLAVSEKSEINSAVKPAVTNGFGNYDETLLQGLDYLLVELAKRDMTVVLY
FNNFWQWSGGMTQYMAWIEGEPVQDPNVTNEWEAFMAKSASFYRSEKAQQEYRKTLEKIITRVNS
INGKAYVDDATIMSWQLANEPRPGNSQTTAEEKQIYIDWVHAAAAYIKTLDAHHLVSSGSEGEMG
SVNDMQVFIDAHATPDIDYLTYHMWIRNWSWFDKTKPAETWPSAWEKAQNYMRAHIDVAKQLNKP
LVLEEFGLDRDMGSYAMDSTTEYRDNYFRGVFELMLASLEQGEPSAGYNIWAWNGYGRTTRANYW
WQEGDDFMGDPPQEEQGMYGVFDTDTSTIAIMKEFNARFQPKLEHHHHHH

>2FSUA

MRGSHHHHHHGSGSMGMTLETAFMLPVQDAQHSFRRLLKAMSEPGVIVALHQLKRGWQPLNIATT
SVLLTLADNDTPVWLSTPLNNDIVNQSLRFHTNAPLVSQPEQATFAVTDEAISSEQLNALSTGTA
VAPEAGATLILQVASLSGGRMLRLTGAGIAEERMIAPQLPECILHELTERPHPFPLGIDLILTCG
ERLLAIPRTTHVEVC

>3OV5A

MTSYTYQATPMDGTLKTMLERWAADSNMQLSYNLPSDYTLIGPVSAISTTSVQQAATELSAVYAA
QGVSVSVSANKLLVQPVPVS

>2HLJA

GMPALITYRTTVQEDWVDYNGHLRDAFYLLIFSYATDALMDRIGLDADSRGQSGNSLFTLEAHIN
YLHEVKLGTEVWVQTQILGFDRKRLHVYHSLHRAGFDEVLAASEQMLLHVDLAGPQSAPFGHTTV
CRLNHLVEQQEGAQAPQYMGRTIKLPA

>3TTGA

MGSSHHHHHHSSGRENLYFQGMLTEVSDTRIAHKKFGLFYPSVSRPSIFVEGEDRKNFLQGIASQ
DILKQDEKSLSYSFFLNPKARILFDAWCGNFEDKIALFPPAGTREEFVNHLKKYLFFRTKAKITD
MSDHFREIRLVGPETISVLLSLFDNNFSGSSFRMLKNGGYVLIHPTSFQHNLDVGLQADLFIPID
QFETTQKSLEDFTSNKGGVLLDESSYLAYLTEKGIPLFPSELNDSFFPAEAGLDSVGVSYNKGCY
VGQEPVTRLKFQGHLNRSLAGFRLEGGPFPKMEFPVTLFNPKDGNEAGILTRTSSSDILGSGIGL
GYIKRNFSENGTELLLPDAQLVRVHSLPFV

>2FEXA

MTRIAIALAQDFADWEPALLAAAARSYLGVEIVHATPDGMPVTSMGGLKVTPDTSYDALDPVDID
ALVIPGGLSWEKGTAADLGGLVKRFRDRDRLVAGICAAASALGGTGVLNDVAHTGNALASHKAYP
AYRGEAHYRDQPRAVSDGGVVTAAGSAPVSFAVEILKSLGLFGPEAEAELQIFAAEHR

>2ZZVA

MKRVSRRAFLRRLGVGVAATAAFSPLAVAQARRYRWRIQTAWDAGTVGYSLFQKFTERVKELTDG
QLEVQPFPAGAVVGTFDMFDAVKTGVLDGMNPFTLYWAGRMPVTAFLSSYALGLDRPDQWETWFY
SLGGLDIARRAFAEQGLFYVGPVQHDLNIIHSKKPIRRFEDFKGVKLRVPGGMIAEVFAAAGAST
VLLPGGEVYPALERGVIDAADFVGPAVNYNLGFHQVAKYIIMGPPETPAIHQPVDLMDFTINLNR
WRSLPKPLQERFIAAVHEYSWIHYAGIQKANLEAWPKYRQAGVEVIRLSNEDVRKFRRLAIPIWF
KWAKMDKYSREAFASQLEYMKGIGYVTDEELKGLSL

>2XVYA

GHGAPKAQKTGILLVAFGTSVEEARPALDKMGDRVRAAHPDIPVRWAYTAKMIRAKLRAEGIAAP
SPAEALAGMAEEGFTHVAVQSLHTIPGEEFHGLLETAHAFQGLPKGLTRVSVGLPLIGTTADAEA
VAEALVASLPADRKPGEPVVFMGHGTPHPADICYPGLQYYLWRLDPDLLVGTVEGSPSFDNVMAE
LDVRKAKRVWLMPLMAVAGDHARNDMAGDEDDSWTSQLARRGIEAKPVLHGTAESDAVAAIWLRH

LDDALARLN
>2ERVA
ADVSAAVGATGQSGMTYRLGLSWDWDKSWWQTSTGRLTGYWDAGYTYWEGGDEGAGKHSLSFAPV
FVYEFAGDSIKPFIEAGIGVAAFSGTRVGDQNLGSSLNFEDRIGAGLKFANGQSVGVRAIHYSNA
GLKQPNDGIESYSLFYKIPI
>4DUIA
MRGSHHHHHHGSDLGKKLLEAARAGQDDEVRILMANGADVNATDASGLTPLHLAATYGHLEIVEV
LLKHGADVNAIDIMGSTPLHLAALIGHLEIVEVLLKHGADVNAVDTWGDTPLHLAAIMGHLEIVE
VLLKHGADVNAQDKFGKTAFDISIDNGNEDLAEILQKLN
>2UWAA
AYVQGPPSPGYYPSSQITSLGFDQGYTNLWGPQHQRVDQGSLTIWLDSTSGSGFKSINRYRSGYF
GANIKLQSGYTAGVITSFYLSNNQDYPGKHDEIDIEFLGTIPGKPYTLQTNVFIEGSGDYNIIGR
EMRIHLWFDPTQDYHNYAIYWTPSEIIFFVDDVPIRRYPRKSDATFPLRPLWVYGSVWDASSWAT
ENGKYKADYRYQPFVGKYEDFKLGSCTVEAASSCNPASVSPYGQLSQQQVAAMEWVQKNYMVYNY
CDDPTRDHTLTPEC
>1OI7A
MILVNRETRVLVQGITGREGQFHTKQMLTYGTKIVAGVTPGKGGMEVLGVPVYDTVKEAVAHHEV
DASIIFVPAPAAADAALEAAHAGIPLIVLITEGIPTLDMVRAVEEIKALGSRLIGGNCPGIISAE
ETKIGIMPGHVFKRGRVGIISRSGTLTYEAAAALSQAGLGTTTTVGIGGDPVIGTTFKDLLPLFN
EDPETEAVVLIGEIGGSDEEEAAAWVKDHMKKPVVGFIGGRSAPKGKRMGHAGAIIMGNVGTPES
KLRAFAEAGIPVADTIDEIVELVKKALG
>3KS7A
GAGGHKNLPAKGDLHIPVFENVNVRFSPDTYPDNYNEADGTGVYHLVNGRIILKKITLPEYKRNV
SVSLKVTLASNGDRWDKSGSCFVLPKSSAINLLTIARDGMKFPSVDSLKLEKMVGIVPGKDYLPT
VELMRFMTPFGIGHYSNNNDSLSSKRRPVYIPKWESNVTWQQDITDLYPLLEGEAYVGIYIDTWT
SEGYLVNADIDVKESRLACDVLPKRHVEPLMNTVYYMGQSYPDIFARRDVSTDFTVPKGAKNIRL
KYIVTGHGGHSGGDEFVQKRNIISVDGKEVLNFIPWRDDCASFRRFNPATGVWLIKRLASYIGEK
GYTEKEVEEPLASSDLSRSNWCPGSDVVPEEAVIGTLAPGKHTFTVSIPEAQAVDGNKLNHWLVS
AYLVWEE
>1YQHA
SNAMSQQVTMSFSVVPQAKTKDVYSVVDKAIEVVQQSGVRYEVGAMETTLEGELDVLLDVVKRAQ
QACVDAGAEEVITSIKIHYRPSTGVTIDEKVWKYRDEYAKPEAI
>4K6JA
GPLGSGRPELYTVVQHVKHFNDVVEFGENQEFTDDIEYLLSGLKSTQPLNTRCLSVISLATKCAM
PSFRMHLRAHGMVAMVFKTLDDSQHHQNLSLCTAALMYILSRDRLNMDLDRASLDLMIRLLELEQ
DASSAKLLNEKDMNKIKEKIRRLCETVHNKHLDLENITTGHLAMETLLSLTSKRAGDWFKEELRL
LGGLDHIVDKVKECVDHLSRDEDEEKLVASLWGAERCLRVLESVTVHNPENQSYLIAYKDSQLIV
SSAKALQHCEELIQQYNRAEDSICLADSKPLPHQNVTNHVGKAVEDCMRAIIGVLLNLTNDNEWG
STKTGEQDGLIGTALNCVLQVPKYLPQEQRFDIRVLGLGLLINLVEYSARNRHCLVNMETSCSFD
SSICSGEGDDSLRIGGQVHAVQALVQLFLERERAAQLAESKTDELIKDAPTTQHDKSGEWQETSG
EIQWVSTEKTDGTEEKHKKEEEDEELDLNKALQHAGKHMEDCIVASYTALLLGCLCQESPINVTT
VREYLPEGDFSIMTEMLKKFLSFMNLTCAVGTTGQKSISRVIEYLEHC
>3TC8A
GKETGNMPEIKKQPIASAVPDFNADSAYAYVANQVAFGPRVPNTAAHKACGDYLASELKRFGAKV

YQQEAILTAYDGTKLEARNIIGSFDPENSKRVLLFAHWDSRPYSDHDPDPSKHRTPLDGADDGGS
GVGALLEIARQIGQKAPGIGIDIIFFDAEDYGTPEFVTDYTPDSWCLGTQFWAKNPHVPNYTAEY
GILLDMVGGKNATFFKEQQSLRAAAPIVEMVWSAARDLGYGKYFINAAGGAITDDHQYVISGRNI
PSIDIINYDPESKTGFASYWHTQKDNMENIDRETLKAAGQTVLEVIYNR
>2WE3A
GAMGSGIPMEACPHIRYAFQNDKLLLQQASVGRLTLVNKTTILLRPMKTTTVDLGLYARPPEGHG
LMLWGSTSRPVTSHVGIIDPGYTGELRLILQNQRRYNSTLRPSELKIHLAAFRYATPQMEEDKGP
INHPQYPGDVGLDVSLPKDLALFPHQTVSVTLTVPPPSIPHHRPTIFGRSGLAMQGILVKPCRWR
RGGVDVSLTNFSDQTVFLNKYRRFCQLVYLHKHHLTSFYSPHSDAGVLGPRSLFRWASCTFEEVP
SLAM
>3DYJA
GIDPFTGIDPFTGTQACITAASAVSGIIADLDTTIMFATAGTLNREGAETFADHREGILKTAKVL
VEDTKVLVQNAAGSQEKLAQAAQSSVATITRLADVVKLGAASLGAEDPETQVVLINAVKDVAKAL
GDLISATKAAAGKVGDDPAVWQLKNSAKVMVTNVTSLLKTVKAVEDEATKGTRALEATTEHIRQE
LAVFCSPEPPAKTSTPEDFIRMTKGITMATAKAVAAGNSCRQEDVIATANLSRRAIADMLRACKE
AAFHPEVAPDVRLRALHYGRECANGYLELLDHVLLTLQKPNPDLKQQLTGHSKRVAGSVTELIQA
AEAMKGT
>2OIZA
REVLTGGHSVSAPQENRIYVMDSVFMHLTESRVHVYDYTNGKFLGMVPTAFNGHVQVSNDGKKIY
TMTTYHERITRGKRSDVVEVWDADKLTFEKEISLPPKRVQGLNYDGLFRQTTDGKFIVLQNASPA
TSIGIVDVAKGDYVEDVTAAAGCWSVIPQPNRPRSFMTICGDGGLLTINLGEDGKVASQSRSKQM
FSVKDDPIFIAPALDKDKAHFVSYYGNVYSADFSGDEVKVDGPWSLLNDEDKAKNWVPGGYNLVG
LHRASGRMYVFMHPDGKEGTHKFPAAEIWVMDTKTKQRVARIPGRDALSMTIDQQRNLMLTLDGG
NVNVYDISQPEPKLLRTIEGAAEASLQVQFHPVGGT
>2ZZJA
TRSFYNDGHLNGWDYVRKENQGTVSEVSNVVFKGTSALKMTQTYTPGYTGRYHSEVDHNRGYQRG
EEQFYGFAFRLSEDWQFQPQSYNIAQFIANRPGAGCGGDDWMPSTMIWIQNNQLYSRYVNGHYRQ
PNCGRNIVTRPNLATVSAGAWHRVVLQIKWASDNTGYFKIWFDGAKVHEEYNVATTVDDDSVFQF
RVGLYANSWHDDGHMTGTQGFRQVWYDEVAVGTTFADVDPDQA
>1Q8DA
ERPNCLSLQDSCKTNYICRSRLADFFTNCQPESRSVSNCLKENYADCLLAYSGLIGTVMTPNYVD
SSSLSVAPWCDCSNSGNDLEDCLKFLNFFKDNTCLKNAIQAFG
>1T1JA
NLYFQGHMRKIFLACPYSHADAEVVEQRFRACNEVAATIVRAGHVVFSQVSMSHPINLCLAELDR
AAIGRLWAPVDAFYMDHLEELIVLDLPGWRDSAGIRREMEFFEAGGQRVSLWSEVEHEFR
>3JUMA
MGSSHHHHHHSSGLVPRGSHMSDVESLENTSENRAQVAARQHNRKIVEQYMHTRGEARLKRHLLF
TEDGVGGLWTTDSGQPIAIRGREKLGEHAVWSLQCFPDWVWTDIQIFETQDPNWFWVECRGEGAI
VFPGYPRGQYRNHFLHSFRFENGLIKEQREFMNPCEQFRSLGIEVPEVRRDGLPS
>1XDYA
DLLSWFKGNDRPPAPAGKALEFSKPAAWQNNLPLTPADKVSGYNNFYEFGLDKADPAANAGSLKT
DPWTLKISGEVAKPLTLDHDDLTRRFPLEERIYRMRCVEAWSMVVPWIGFPLHKLLALAEPTSNA
KYVAFETIYAPEQMPGQQDRFIGGGLKYPYVEGLRLDEAMHPLTLMTVGVYGKALPPQNGAPVRL
IVPWKYGFKGIKSIVSIKLTRERPPTTWNLAAPDEYGFYANVNPYVDHPRWSQATERFIGSGGIL

DVQRQPTLLFNGYADQVASLYRGLDLRENFLEHHHHHH
>3C2QA
SNANIPEIENANLKPALKDSVLPDGFYSTTNHPTHVKVNDEWIEVANPKMDAVIVVYPEEKRAET
KVIRKVKKGDFVLIGHNGIRVMPPEKSREAGQLFEFMNSEVSSEKPKEAIIKRIAKEMHEIREEY
KKTGTGGIAIVGGPAIIHTGGGPALAKMVELGYIQAILAGNALATHDIESALYGTSLGVNIKTAK
PVTGGHKHHIYAINAINDAGNIKNAVESGVLKEGIMYQCIKNNIPYVLAGSIRDDGPIPDVITDS
MVAQDKMRTTVMDKKMVIMLSTLLHSVATGNLMPSYIKTVCVDIQPSTVTKLMDRGTSQAIGVVT
DVGVFLVLLLKELERLELQE
>3QP4A
GSHMRPLPAGLTASQQWTLLEWIHMAGHIETENELKAFLDQVLSQAPSERLLLALGRLNNQNQIQ
RLERVLNVSYPSDWLDQYMKENYAQHDPILRIHLGQGPVMWEERFNRAKGAEEKRFIAEATQNGM
GSGITFSAASERNNIGSILSIAGREPGRNAALVAMLNCLTPHLHQAAIRVAN
>1NYCA
GSMYQLQFINLVYDTTKLTHLEQTNINLFIGNWSNHQLQKSICIRHGDDTSHNQYHILFIDTAHQ
RIKFSSFDNEEIIYILDYDDTQHILMQTSSKQGIGTSRPIVYERLV
>3A6FA
MSKSVFVGELTWKEYEARVAAGDCVLMLPVGALEQHGHHMCMNVDVLLPTAVCKRVAERIGALVM
PGLQYGYKSQQKSGGGNHFPGTTSLDGATLTGTVQDIIRELARHGARRLVLMNGHYENSMFIVEG
IDLALRELRYAGIQDFKVVVLSYWDFVKDPAVIQQLYPEGFLGFDIEHGGVFETSLMLALYPDLV
DLDRVVDHPPATFPPYDVFPVDPARTPAPGTLSSAKTASREKGELILEVCVQGIADAIREEFPPT
>2ZF9A
MTHHHHHHAMGPAAGQAYDAGNLDVASSPVKPTLSITKKTLTAAEAPNAKVTMELSVEGAADKYA
ATGLHIQFDPKLKLIPDEDGALATAGRAARLLELKKAEADTDNSFFTATGSSTNNGKDGVLWSFV
LQVPADAQPGDKYDVQVAYQSRTTNEDLFTNVKKDEEGLLMQAWTFTQGIEQGYIQVESTTSLE
>3QQZA
SNASNHAASFQNYHATIDGKEIAGITNNISSLTWSAQSNTLFSTINKPAAIVEMTTNGDLIRTIP
LDFVKDLETIEYIGDNQFVISDERDYAIYVISLTPNSEVKILKKIKIPLQESPTNCGFEGLAYSR
QDHTFWFFKEKNPIEVYKVNGLLSSNELHISKDKALQRQFTLDDVSGAEFNQQKNTLLVLSHESR
ALQEVTLVGEVIGEMSLTKGSRGLSHNIKQAEGVAMDASGNIYIVSEPNRFYRFTPQSSH
>1KF6C
TTKRKPYVRPMTSTWWKKLPFYRFYMLREGTAVPAVWFSIELIFGLFALKNGPEAWAGFVDFLQN
PVIVIINLITLAAALLHTKTWFELAPKAANIIVKDEKMGPEPIIKSLWAVTVVATIVILFVALYW
>4HSPA
GAPRELTWSQLIPAGAPPAPAPLPIHDLANALSEAGPAASQQSPNAPVVKALDGIEAKLPGYIVP
LEISEAGLVTEFLLVPYYGACIHVPPPPSNQIVYVKTAKGVQMDELYQPFWVEGTFKVENASSEL
AAAGYRMQASKVTPYEYEGG
>4I66A
SNASAFRFGQLALGDRWDIYPQALSRMSREIDKRTSIEAAREPAAVTLSSPTLHETPFLYLAGDR
EFAIPPEPEVEALRRHLTFGGFLLIDSAEGALGGAFDRSVRRLLQAVFPAPAPGLEIVSGEHVVF
KSFYLLERPLGRLALSPVMEGILRDGRLMVAYVQNDLGGAFARDDFGNFQLACVPDGERQRELAF
RMLVNLVMYALCLD
>2RFEE
LSPSNSRTPSPKSLPSYLNGVMPPTQSFAPDPKYVSSKAL
>2FELA

MSLSPFEHPFLSGLFGDSEIIELFSAKADIDAMIRFETALAQAEAEASIFADDEAEAIVSGLSEF
AADMSALRHGVAKDGVVVPELIRQMRAAVAGQAADKVHFGATSQDVIDTSLMLRLKMAAEIIATR
LGHLIDTLGDLASRDGHKPLTGYTRMQAAIGITVADRAAGWIAPLERHLLRLETFAQNGFALQFG
GAAGTLEKLGDNAGAVRADLAKRLGLADRPQWHNQRDGIAEFANLLSLVTGTLGKFGQDIALMAE
IGSEIRLSGGGGSSAMPHKQNPVNAETLVTLARFNAVQISALHQSLVQEQERSGAGWMLEWLTLP
QMVTATGTSLLVAERLAAQIDRLGADESHHHHHH
>4JHMA
MSLEDCRISRVELYALADENAPPIPWADNQEPLLYTNNIVRLFTEDGTEGLGATMSYTENFFDRC
IIESLRTIVPGLIGKNPLMTQELNNWLGARCTWGGLPAKSPIDIAAWDIKGKKAGMPLYMLLGGA
RTKIKSYASTPMFDTVEEYFPYIDDCIEHGFTAIKLHCYCVYDKDVALVEAVEAKYGTSGIRFML
DTAGFYTPEQAMKMAKWMERHNWEWLEAPVSDYDFKTYQRLVANTDLEISSHGNCLLTLQEVTHA
LSTGMWSDVRQDATVCGGITQLNKCFAIAAGHSKNLEIQSMGYTLTQAANLHVALAHDNCNFFEQ
FYPYEAFELASKTQIRTDKEGYVHAPAGNGLGVEMDWDAVKEASFASYVFEEGHHHHHH
>3S8GA
MHHHHHHHAVRASEISRVYEAYPEKKATLYFLVLGFLALIVGSLFGPFQALNYGNVDAYPLLKRL
LPFVQSYYQGLTLHGVLNAIVFTQLFAQAIMVYLPARELNMRPNMGLMWLSWWMAFIGLVVFALP
LLANEATVLYTFYPPLKGHWAFYLGASVFVLSTWVSIYIVLDLWRRWKAANPGKVTPLVTYMAVV
FWLMWFLASLGLVLEAVLFLLPWSFGLVEGVDPLVARTLFWWTGHPIVYFWLLPAYAIIYTILPK
QAGGKLVSDPMARLAFLLFLLLSTPVGFHHQFADPGIDPTWKMIHSVLTLFVAVPSLMTAFTVAA
SLEFAGRLRGGRGLFGWIRALPWDNPAFVAPVLGLLGFIPGGAGGIVNASFTLDYVVHNTAWVPG
HFHLQVASLVTLTAMGSLYWLLPNLTGKPISDAQRRLGLAVVWLWFLGMMIMAVGLHWAGLLNVP
RRAYIAQVPDAYPHAAVPMVFNVLAGIVLLVALLLFIYGLFSVLLSRERKPELAEAPLPFAEVIS
GPEDRRLVLAMDRIGFWFAVAAILVVLAYGPTLVQLFGHLNPVPGWRLW
>2EABA
MVIASVEDGGDGDTSKDDWLWYKQPASQTDATATAGGNYGNPDNNRWQQTTLPFGNGKIGGTVWG
EVSRERVTFNEETLWTGGPGSSTSYNGGNNETKGQNGATLRALNKQLANGAETVNPGNLTGGENA
AEQGNYLNWGDIYLDYGFNDTTVTEYRRDLNLSKGKADVTFKHDGVTYTREYFASNPDNVMVARL
TASKAGKLNFNVSMPTNTNYSKTGETTTVKGDTLTVKGALGNNGLLYNSQIKVVLDNGEGTLSEG
SDGASLKVSDAKAVTLYIAAATDYKQKYPSYRTGETAAEVNTRVAKVVQDAANKGYTAVKKAHID
DHSAIYDRVKIDLGQSGHSSDGAVATDALLKAYQRGSATTAQKRELETLVYKYGRYLTIGSSREN
SQLPSNLQGIWSVTAGDNAHGNTPWGSDFHMNVNLQMNYWPTYSANMGELAEPLIEYVEGLVKPG
RVTAKVYAGAETTNPETTPIGEGEGYMAHTENTAYGWTAPGQSFSWGWSPAAVPWILQNVYEAYE
YSGDPALLDRVYALLKEESHFYVNYMLHKAGSSSGDRLTTGVAYSPEQGPLGTDGNTYESSLVWQ
MLNDAIEAAKAKGDPDGLVGNTTDCSADNWAKNDSGNFTDANANRSWSCAKSLLKPIEVGDSGQI
KEWYFEGALGKKKDGSTISGYQADNQHRHMSHLLGLFPGDLITIDNSEYMDAAKTSLRYRCFKGN
VLQSNTGWAIGQRINSWARTGDGNTTYQLVELQLKNAMYANLFDYHAPFQIDGNFGNTSGVDEML
LQSNSTFTDTAGKKYVNYTNILPALPDAWAGGSVSGLVARGNFTVGTTWKNGKATEVRLTSNKGK
QAAVKITAGGAQNYEVKNGDTAVNAKVVTNADGASLLVFDTTAGTTYTITKKAS
>2YIZA
SNHTYRVIEIVGTSPDGVDAAIQGGLARAAQTMRALDWFEVQSIRGHLVDGAVAHFQVTMKVGFR
LEDS
>2UVKA
SVLPETPVPFKSGTGAIDNDTVYIGLGSAGTAWYKLDTQAKDKKWTALAAFPGGPRDQATSAFID
GNLYVFGGIGKNSEGLTQVFNDVHKYNPKTNSWVKLMSHAPMGMAGHVTFVHNGKAYVTGGVNQN

IFNGYFEDLNEAGKDSTAIDKINAHYFDKKAEDYFFNKFLLSFDPSTQQWSYAGESPWYGTAGAA
VVNKGDKTWLINGEAKPGLRTDAVFELDFTGNNLKWNKLAPVSSPDGVAGGFAGISNDSLIFAGG
AGFKGSRENYQNGKNYAHEGLKKSYSTDIHLWHNGKWDKSGELSQGRAYGVSLPWNNSLLIIGGE
TAGGKAVTDSVLITVKDNKVTVQNLEHHHHHH

>1ERZA

TRQMILAVGQQGPIARAETREQVVVRLLDMLTKAASRGANFIVFPELALTTFFPRWHFTDEAELD
SFYETEMPGPVVRPLFEKAAELGIGFNLGYAELVVEGGVKRRFNTSILVDKSGKIVGKYRKIHLP
GHKEYEAYRPFQHLEKRYFEPGDLGFPVYDVDAAKMGMFICNDRRWPEAWRVMGLRGAEIICGGY
NTPTHNPPVPQHDHLTSFHHLLSMQAGSYQNGAWSAAAGKVGMEENCMLLGHSCIVAPTGEIVAL
TTTLEDEVITAAVDLDRCRELREHIFNFKQHRQPQHYGLIAEL

>2YHCA

DNPPNEIYATAQQKLQDGNWRQAITQLEALDNRYPFGPYSQQVQLDLIYAYYKNADLPLAQAAID
RFIRLNPTHPNIDYVMYMRGLTNMALDDSALQGFFGVDRSDRDPQQARAAFSDFSKLVRGYPNSQ
YTTDATKRLVFLKDRLAKYEYSVAEYYTERGAWVAVVNRVEGMLRDYPDTQATRDALPLMENAYR
QMQMNAQAEKVAKIIAANSSNTLEHHHHHH

>3BN8A

MGSDKIHHHHHHMSEAKELIKKMCDLQNSNEEIQKEMAGWSGVVQYKLDGYYFYVEYKSDGTCEF
KEGVHSSPTFTVVAPPDFWLAVLKGQEDPVSGFMMGKYRIEGNIMEAQRLAGVIKKFQGKFEL

>1PM4A

LRIPNIATYTGTIQGKGEVCIIGNKEGKTRGGELYAVLHSTNVNADMTLILLRNVGGNGWGEIKR
NDIDKPLKYEDYYTSGLSWIWKIKNNSSETSNYSLDATVHDDKEDSDVLTKCPV

>3DNHA

GHMLDVAPPVITPRGTKIEPSAGAPFEAVRVARDVLHTSRTAALATLDPVSGYPYTTATNIGIEP
DGTPFFFAAGLTLHARNMETDARISVTLAPFGKGDALTLPRLTLVGRADRIGPDEVPLAIARYIA
RYPKAKLYLSLPDTRLYRLRTEGVQINGGPARNASNITPADLRTDLSGAEELMAAAESEATRLNA
IKGEASRLAVLAGAKTGRWKITSIDPDGIDLASASDLARLWFAERVETLKQFEKALAQLLKGS

>3HR0A

GSQQGKFDTKGIESTDEAKMSFLVTLNNVEVCSENISTLKKTLESDCTKLFSQGIGGEQAQAKFD
SCLSDLAAVSNKFRDLLQEGLTELNSTAIKPQVQPWINSFFSVSHNIEEEEFNDYEANDPWVQQF
ILNLEQQMAEFKASLSPVIYDSLTGLMTSLVAVELEKVVLKSTFNRLGGLQFDKELRSLIAYLTT
VTTWTIRDKFARLSQMATILNLERVTEILDYWGPNSGPLTWRLTPAEVRQVLALRIDFRSEDIKR
LRL

>3ATSA

TLPAVISRWLSSVLPGGAAPEVTVESGVDSTGMSSETIILTARWQQDGRSIQQKLVARVAPAAED
VPVFPTYRLDHQFEVIRLVGELTDVPVPRVRWIETTGDVLGTPFFLMDYVEGVVPPDVMPYTFGD
NWFADAPAERQRQLQDATVAALATLHSIPNAQNTFSFLTQGRTSDTTLHRHFNWVRSWYDFAVEG
IGRSPLLERTFEWLQSHWPDDAAAREPVLLWGDARVGNVLYRDFQPVAVLDWEMVALGPRELDVA
WMIFAHRVFQELAGLATLPGLPEVMREDDVRATYQALTGVELGDLHWFYVYSGVMWACVFMRTGA
RRVHFGEIEKPDDVESLFYHAGLMKHLLGEEH

>3UB6A

GSKVMQKDVLAQLMEHLETGQYKKREKTLAYMTKILEQGIHEYYKSFDNDTARKMALDYFKRIND
DKGMIYMVVVDKNGVVLFDPVNPKTVGQSGLDAQSVDGVYYVRGYLEAAKKGGGYTYYKMPKYDG
GVPEKKFAYSHYDEVSQMVIAATSYYTDINTENKAIKEGVNKVFNENTTRL

>3HWWA

MSVSAFNRRWAAVILEALTRHGVRHICIAPGSRSTLLTLAAAENSAFIHHTHFDERGLGHLALGL
AKVSKQPVAVIVTSGTAVANLYPALIEAGLTGEKLILLTADRPPELIDCGANQAIRQPGMFASHP
THSISLPRPTQDIPARWLVSTIDHALGTLHAGGVHINCPFAEPLYGEMDDTGLSWQQRLGDWWQD
DKPWLREAPRLESEKQRDWFFWRQKRGVVVAGRMSAEEGKKVALWAQTLGWPLIGDVLSQTGQPL
PCADLWLGNAKATSELQQAQIVVQLGSSLTGKRLLQWQASCEPEEYWIVDDIEGRLDPAHHRGRR
LIANIADWLELHPAEKRQPWCVEIPRLAEQAMQAVIARRDAFGEAQLAHRICDYLPEQGQLFVGN
SLVVRLIDALSQLPAGYPVYSNRGASGIDGLLSTAAGVQRASGKPTLAIVGDLSALYDLNALALL
RQVSAPLVLIVVNNNGGQIFSLLPTPQSERERFYLMPQNVHFEHAAAMFELKYHRPQNWQELETA
FADAWRTPTTTVIEMVVNDTDGAQTLQQLLAQVSHL

>2YGBA

MAHHHHHHSSGLEVLFQGPNNTIINSLIGGDDSIKRSNVFAVDSQIPTLYMPQYISLSGVMTNDG
PDNQAIASFEIRDQYITALNHLVLSLELPEVKGMGRFGYVPYVGYKCINHVSISSCNGVIWEIEG
EELYNNCINNTIALKHSGYSSELNDISIGLTPNDTIKEPSTVYVYIKTPFDVEDTFSSLKLSDSK
ITVTVTFNPVSDIVIRDSSFDFETFNKEFVYVPELSFIGYMVKNVQIKPSFIEKPRRVIGQINQP
TATVTEVHAATSLSVYTKPYYGNTDNKFISYPGYSQDEKDYIDAYVSRLLDDLVIVSDGPPTGYP
ESAEIVEVPEDGIVSIQDADVYVKIDNVPDNMSVYLHTNLLMFGTRKNSFIYNISKKFSAITGTY
SDATKRTIFAHISHSINIIDTSIPVSLWTSQRNVYNGDNRSAESKAKDLFINDPFIKGIDFKNKT
DIISRLEVRFGNDVLYSENGPISRIYNELLTKSNNGTRTLTFNFTPKIFFRPTTITANVSRGKDK
LSVRVVYSTMDVNHPIYYVQKQLVVVCNDLYKVSYDQGVSITKIMGDNN

>3QXFA

ACTWPAWEQFKKDYISQEGRVIDPSDARKITTSEGQSYGMFSALAANDRAAFDNILDWTQNNLAQ
GSLKERLPAWLWGKKENSKWEVLDSNSASDGDVWMAWSLLEAGRLWKEQRYTDIGSALLKRIARE
EVVTVPGLGSMLLPGKVGFAEDNSWRFNPSYLPPTLAQYFTRFGAPWTTLRETNQRLLLETAPKG
FSPDWVRYEKDKGWQLKAEKTLISSYDAIRVYMWVGMMPDSDPQKARMLNRFKPMATFTEKNGYP
PEKVDVATGKAQGKGPVGFSAAMLPFLQNRDAQAVQRQRVADNFPGSDAYYNYVLTLFGQGWDQH
RFRFSTKGELLPDWGQECANSHLEHHHHHH

>1LYQA

AHPELKSSVPQADSAVAAPEKIQLNFSENLTVKFSGAKLTMTGMKGMSSHSPMPVAAKVAPGADP
KSMVIIPREPLPAGTYRVDWRAVSSDTHPITGNYTFTVK

>3CNYA

GMSSKAEKDIKWGIAPIGWRNDDIPSIGKDNNLQQLLSDIVVAGFQGTEVGGFFPGPEKLNYELK
LRNLEIAGQWFSSYIIRDGIEKASEAFEKHCQYLKAINAPVAVVSEQTYTIQRSDTANIFKDKPY
FTDKEWDEVCKGLNHYGEIAAKYGLKVAYHHHMGTGIQTKEETDRLMANTDPKLVGLLYDTGHIA
VSDGDYMALLNAHIDRVVHVHFKDVRRSKEEECRAKGLTFQGSFLNGMFTVPGDGDLDFKPVYDK
LIANNYKGWIVVEAEQDPSKANPLEMAQIAHRYIKQHLIEN

>2X9ZA

AKPKIDKDFKGKANPDTPRVDKDTPVNHQVGDVVEYEIVTKIPALANYATANWSDRMTEGLAFNK
GTVKVTVDDVALEAGDYALTEVATGFDLKLTDAGLAKVNDQNAEKTVKITYSATLNDKAIVEVPE
SNDVTFNYGNNPDHGNTPKPNKPNENGDLTLTKTWVDATGAPIPAGAEATFDLVNAQAGKVVQTV
TLTTDKNTVTVNGLDKNTEYKFVERSIKGYSADYQEITTAGEIAVKNWKDENPKPLDPTEPKVVT
YG

>2OIZD

AGGGGSSSGADHISLNPDLANEDEVNSCDYWRHCAVDGFLCSCCGGTTTTCPPGSTPSPISWIGT
CHNPHDGKDYLISYHDCCGKTACGRCQCNTQTRERPGYEFFLHNDVNWCMANENSTFHCTTSVLV

GLAKN
>3QQ2A
MAESNALDKRLGELRLRADAGGPWARTFSERQQISNRHARAYDQTVSGLEIGLDRGWSASGGRWY
AGGLLGYTYADRTYPGDGGGKVKGLHVGGYAAYVGDGGYYLDTVLRLGRYDQQYNIAGTDGGRVT
ADYRTSGAAWSLEGGRRFELPNDWFAEPQAEVMLWRTSGKRYRASNGLRVKVDANTATLGRLGLR
FGRRIALAGGNIVQPYARLGWTQEFKSTGDVRTNGIGHAGAGRHGRVELGAGVDAALGKGHNLYA
SYEYAAGDRINIPWSFHAGYRYSF
>1JIWI
SSLILLSASDLAGQWTLQQDEAPAICHLELRDSEVAEASGYDLGGDTACLTRWLPSEPRAWRPTP
AGIALLERGGLTLMLLGRQGEGDYRVQKGDGGQLVLRRATP
>1MTYB
ERRRGLTDPEMAAVILKALPEAPLDGNNKMGYFVTPRWKRLTEYEALTVYAQPNADWIAGGLDWG
DWTQKFHGGRPSWGNETTELRTVDWFKHRDPLRRWHAPYVKDKAEEWRYTDRFLQGYSADGQIRA
MNPTWRDEFINRYWGAFLFNEYGLFNAHSQGAREALSDVTRVSLAFWGFDKIDIAQMIQLERGFL
AKIVPGFDESTAVPKAEWTNGEVYKSARLAVEGLWQEVFDWNESAFSVHAVYDALFGQFVRREFF
QRLAPRFGDNLTPFFINQAQTYFQIAKQGVQDLYYNCLGDDPEFSDYNRTVMRNWTGKWLEPTIA
ALRDFMGLFAKLPAGTTDKEEITASLYRVVDDWIEDYASRIDFKADRDQIVKAVLAGLK
>1XR4A
SNAMKETVTMLNQQYVVPEGLQPYQGVTANSPWLASETEKRRRKICDSLEEAIRRSGLKNGMTIS
FHHAFRGGDKVVNMVMAKLAEMGFRDLTLASSSLIDAHWPLIEHIKNGVVRQIYTSGLRGKLGEE
ISAGLMENPVQIHSHGGRVKLIQSGELNIDVAFLGVPCCDEFGNANGFSGKSRCGSLGYAQVDAQ
YAKCVVLLTEEWVEFPNYPASIAQDQVDLIVQVDEVGDPEKITAGAIRLSSNPRELLIARQAANV
IEHSGYFCDGFSLQTGTGGASLAVTRFLEDKMRRHNITASFGLGGITGTMVDLHEKGLIKALLDT
QSFDGDAARSLAQNPHHIEISTNQYANPASKGAACERLNVVMLSALEIDVNFNVNVMTGSNGVLR
GASGGHSDTAAGADLTIITAPLVRGRIPCVVEKVLTTVTPGASVDVLVTDHGIAVNPARQDLLDN
LRAAGVALMTIEQLQQRAEQLTGKPQPIEFTDRVVAVVRYRDGSVIDVIRQVKG
>3EO7A
GMPEIHQSIAQHYHERTKYDPETIASKSQRLDWAKQPVPFKEYKIGSAIDLKPYLQETPEVFVND
TNGQWWQRLSRLLFRSYGLTARMPSMGNTVYLRAAPSAGGLYPAEVYVVSRGTPLLSPGLYNYQC
RTHSLIHYWESDVWQSLQEACFWHPALESTQLAIIVTAVFYRSAWRYEDRAYRRICLDTGHLLGN
IELSAAITDYRPHLIGGFIDEAVNDLLYIDPLQEGAIAVLPLADLLDIQQNISPGCTALPSATET
NYPQVPDGELLKYFHHHTQISASITGKLNLPTVIQEKSLEDKYNFPFCLKISTVSAPIYWGENLS
DLEITMHKRRSTRAYNGEELTFDELKALLDFTYQPQNYIDQSLDNSPDYFDLNLIETFIAVCGVQ
GLEAGCYYYAPKAQELRQIRFKNFRRELHFLCLGQELGRDAAAVIFHTSDLKSAIAQYGDRVYRY
LHMDAGHLGQRLNLAAIQLNLGVSGIGGFFDDQVNEVLGIPNDEAVIYITTLGRPR
>2OU6A
GMPTFNPELHAQTLNSERAYFVQPDADPAFTPHIGALVEMLTYARLTTLQAVEGLPEDQLWATAP
GFANSIGTLLAHIAAVERVYHVLSFQGRDVTPEDDGAAYWGLTMGKEGTAPARLPTLDELRAELA
DARAETLRVFAAKDDAWLAEPLGPGWANQHWAWFHVMEDEVNHRGQLRLLRQVLAPEEGG
>3IB5A
GESSKSGYQTTGENNSSDYQGIIEDGEYKTSKSRGVGISQNSDNLLNLKSFEAGLTTISKDHFST
KSYIFQEGQYLNKATIQDWLGRKSSSNPEGLNPSDNGKKEANKRNPIYVQQIEEQDYMKQNNGKL
ELAGMTIGIGMNQKDYYQKEQYGATYSTTISKEKRIEEGKIAAKKVLARVRQKVGNNVPIVIAMF
AQAPNDSLVGGYFYSYTVSKSGTDIGSWTETNIKSYVLPATEDNKLPNDNDSTSFDNFQKEVKNF

FPNISNVTGQGQYKDKTLQGLHITITTQFYSETEITSFTQYVAQAAKSYLPSGIPVDIKINGSDG
ETQSFVSTTGGNGGYYTHVFGSY
>4JQSA
GQVPEGYPANYAKAPRFKALIYYTQHAEEAHVQFAEQATTFFKKLNYGDGFVLDITTDFSKYPYE
KLKEYNVIIMLNTSPNTKAERDAFEQYMENGGGWVGFHAAAYNDKNTHWPWFVKFLGGGVFYCNN
WPPQPVLVEVDNEEHPVTKNLPASFVAPASEWYQWTPSPRQNKDVEVLLSLSPKNYPLGIKDVVN
FGDFPIVWSNKNYRMIYLNMGHGDEEFIDGTQNLLLVNAFRWVVSKDKSGNPFLK
>3F0PA
MKLAPYILELLTSVNRTNGTADLLVPLLRELAKGRPVSRTTLAGILDWPAERVAAVLEQATSTEY
DKDGNIIGYGLTLRETSYVFEIDDRRLYAWCALDTLIFPALIGRTARVSSHCAATGAPVSLTVSP
SEIQAVEPAGMAVSLVLPQEAADVRQSFCCHVHFFASVPTAEDWASKHQGLEGLAIVSVHEAFGL
GQEFNRHLLQTMSSRTP
>4AQNA
MSDTMVVNGSGGVPAFLFSGSTLSSYRPNFEANSITIALPHYVDLPGRSNFKLMYIMGFPIDTEM
EKDSEYSNKIRQESKISKTEGTVSYEQKITVETGQEKDGVKVYRVMVLEGTIAESIEHLDKKENE
DILNNNRNRIVLADNTVINFDNISQLKEFLRRSVNIVDHDIFSSNGFEGFNPTSHFPSNPSSDYF
NSTGVTFGSGVDLGQRSKQDLLNDGVPQYIADRLDGYYMLRGKEAYDKVRTAPLTLSDNEAHLLS
NIYIDKFSHKIEGLFNDANIGLRFSDLPLRTRTALVSIGYQKGFKLSRTAPTVWNKVIAKDWNGL
VNAFNNIVDGMSDRRKREGALVQKDIDSGLLK
>4BQQA
MSYYHHHHHHDYDIPTTENLYFQGAMDTYAGAYDRQSRERENSSAASPATQRSANEDKAADLQRE
VERDGGRFRFVGHFSEAPGTSAFGTAERPEFERILNECRAGRLNMIIVYDVSRFSRLKVMDAIPI
VSELLALGVTIVSTQEGVFRQGNVMDLIHLIMRLDASHKESSLKSAKILDTKNLQRELGGYVGGK
APYGFELVSETKEITRNGRMVNVVINKLAHSTTPLTGPFEFEPDVIRWWWREIKTHKHLPFKPGS
QAAIHPGSITGLCKRMDADAVPTRGETIGKKTASSAWDPATVMRILRDPRIAGFAAEVIYKKKPD
GTPTTKIEGYRIQRDPITLRPVELDCGPIIEPAEWYELQAWLDGRGRGKGLSRGQAILSAMDKLY
CECGAV
>2GWNA
SNAMKILLRNALITNEGKTFPGSVMIDGAFISRIIEGELPADDNLSADEVIECSGLRLFPGCIDD
QVHFREPGLTHKATIASESRAAVAGGVTSFMDMPNTNPPTTMWERLLEKRQIGADTAWANYGFFF
GGTNDNIDEIKRVDKHLVPGLKLFLGSSTGNMLVDNKETLEKIFGECDLLIATHCEKEEIIRANK
EHYKAKYGNDLDIHFHPLIRSEEACYRSSAEAVELAERMNARLHILHLSTEKELSLFRNDIPTAQ
KRITSEVCVHHLWFSDTDYGRLGNRIKWNPAIKKESDREALRAAVRNGRIDIIATDHAPHLLREK
EGSCLQAASGGPLVQHSLLALLELCNQGIFSIEEIVSKTAHIPATLFAIEKRGYIRPGYYADLVL
VDPSSPHTVSADNILSLCGWSPFEGFTFSHSVAYTFVNGCLAYAKGRLAESRPTVHPLFFNR
>3CTPA
SLANIREIAKRAGISIATVSRHLNNTGYVSEDAREKIQKVVDELNYTPNALARAMFTKNSKTIGL
MVPNISNPFFNQMASVIEEYAKNKGYTLFLCNTDDDKEKEKTYLEVLQSHRVAGIIASRSQCEDE
YANIDIPVVAFENHILDNIITISSDNYNGGRMAFDHLYEKGCRKILHIKGPEVFEATELRYKGFL
DGARAKDLEIDFIEFQHDFQVKMLEEDINSMKDIVNYDGIFVFNDIAAATVMRALKKRGVSIPQE
VQIIGFDNSFIGELLYPSLTTINQPIEALAYTIIELLIKIINGEGVLIEDYIMEVKLIERETTIS
LKDEG
>1QQP2
DKKTEETTLLEDRILTTRNGHTTSTTQSSVGVTYGYATAEDFVSGPNTSGLETRVVQAERFFKTH

LFDWVTSDSFGRCHLLELPTDHKGVYGSLTDSYAYMRNGWDVEVTAVGNQFNGGCLLVAMVPELC
SIQKRELYQLTLFPHQFINPRTNMTAHITVPFVGVNRYDQYKVHKPWTLVVMVVAPLTVNTEGAP
QIKVYANIAPTNVHVAGEFPSKE
>3L1WA
MKIATYNVRVDTEYDQDWQWSFRKEAVCQLINFHDWSLCCIQEVRPNQVRDLKAYTTFTCLSAER
EGDGQGEGLAILYNEQKVQAIDTGYFWLSETPQQPSIHPEAGCPRIALWGLFKETTQNTPFLVIN
VHLDHISAHARLAGMTVILEELHDKIAQYPTLLMGDFNAESGEEVHQLVQKKFQDSKNLATHYGP
RGTFQNFTYTKPWAELEEIDYIYVKGWQVQQTASLTDSIDGRFPSDHFPLEAEVAGENLYFQ
>2AQWA
MHFKTKLKNRRSEVNTCLCIGLDPDEDDIKNFMKNEEQNGYKNIKNNMNSNNNGIENIIKIGKEI
LLTDGENIQNLSEEDKFFYFFNHFCFYIINNTKEYALVYKMNFAFYIPYGSVGINALKNVFDYLN
SMNIPTMLDMKINDIGNTVKNYRKFIFEYLKSDSCTINVYMGTNMLKDICFDYEKNKYYSAYVLI
KTTNKDSFIFQNELSINDKQAYIVMADETQKMATELKIEQNNEFIGFVVGSNAFEEMKIIRNKFP
DSYILSPGIGAQNGDLYKTLKNGYNKDYEKLLINVGRAITKSPDPKKSSESYYNQIIQIFKDIEN
GDNIEQV
>2H0QA
RCLFVCRHGERMDVVFGKYWLSQCFDAKGRYIRTNLNMPHSLPQRSGGFRDYEKDAPITVFGCMQ
ARLVGEALLESNTVIDHVYCSPSLRCVQTAHNILKGLQQDNHLKIRVEPGLFEWTKWVAGSTLPA
WIPPSELAAANLSVDTTYRPHIPVSKLAISESYDTYINRSFQVTKEIISECKSKGNNILIVAHAS
SLEACTCQLQGLSPQNSKDFVQMVRKIPYLGFCSCEELGETGIWQLTDPPILPLTHGPTGGFNWR
E
>3ISMC
MAKRKAEDTQSDKMATAEKVAQNDYTIGLVDPVKDYQKLIETRVQVDEIVDDDVTKENFDRTAAA
ARDVIWRLLFDEAGTSQSNTEKASQLLEEYRGDACFYDPTPYNEWIVKLRDEVLKKELLDFWRDV
LVKKQLGPCWSRDSDLFDSDDTPPLEFYAHAGCTAPFAASLKVRAALEEQASLDQDGPATPTTPG
ELSADDAAALSGEFEATLTKENPLEEYRTLMKRFVLTKIIVPDSVHQASVKKIAAAAREIIWKLL
FDGTPSAEDQNKAAELLQEYKGDAGFYGPDDYNSWIFNLRDEVLTKELLDFWRDKMVKMELGPSC
ARDSDYYDNEDPLPFEFYEKAGCKAPFEGPVNDD
>3QE7A
MTRRAIGVSERPPLLQTIPLSLQHLFAMFGATVLVPVLFHINPATVLLFNGIGTLLYLFICKGKI
PAYLGSSFAFISPVLLLLPLGYEVALGGFIMCGVLFCLVSFIVKKAGTGWLDVLFPPAAMGAIVA
VIGLELAGVAAGMAGLLPAEGQTPDSKTIIISITTLAVTVLGSVLFRGFLAIIPILIGVLVGYAL
SFAMGIVDTTPIINAHWFALPTLYTPRFEWFAILTILPAALVVIAEHVGHLVVTANIVKKDLLRD
PGLHRSMFANGLSTVISGFFGSTPNTTYGENIGVMAITRVYSTWVIGGAAIFAILLSCVGKLAAA
IQMIPLPVMGGVSLLLYGVIGASGIRVLIESKVDYNKAQNLILTSVILIIGVSGAKVNIGAAELK
GMALATIVGIGLSLIFKLISVLRPEEVVLDAEDADITDK
>3VJZA
HMTALTLPEDIRQQEPSALLYTLVSAYLEHTAQTGDESLSCLSDDQHTLTAFCYLDSQVEEGGFV
QLIASGYGEYIFRNPLADSLRRWKIKAVPKVLDKAKALYEQHGKTIETLADGGADIPSLRKQFPE
FEEWDGAYYEAAEQDLPLLAEHIQSNWETFAHIGQA
>1Z6OM
TQCNVNPVQIPKDWITMHRSCRNSMRQQIQMEVGASLQYLAMGAHFSKDVVNRPGFAQLFFDAAS
EEREHAMKLIEYLLMRGELTNDVSSLLQVRPPTRSSWKGGVEALEHALSMESDVTKSIRNVIKAC
EDDSEFNDYHLVDYLTGDFLEEQYKGQRDLAGKASTLKKLMDRHEALGEFIFDKKLLGIDV

>4E9JA

SHMENSGGNAFVPAGNQQEAHWTINLKDADIREFIDQISEITGETFVVDPRVKGQVSVVSKAQLS
LSEVYQLFLSVMSTHGFTVVAQGDQARIVPNAEAKTEAGGGQSAPDRLETRVIQVQQSPVSELIP
LIRPLVPQYGHLAAVPSANALIISDRSANIARIEDVIRQLDQKGSHDYSVINLRYGWVMDAAEVL
NNAMSRGQAKGAAGAQVIADARTNRLIILGPPQARAKLVQLAQSLDTPTAR

>3PZDA

EFDTPTQQLIQDIKENCLNSDVVEQIYKRNPILRYTHHPLHSPLLPLPYGDINLNLLKDKGYTTL
QDEAIKIFNSLQQLESMSDPIPIIQGILQTGHDLRPLRDELYCQLIKQTNKVPHPGSVGNLYSWQ
ILTCLSCTFLPSRGILKYLKFHLKRIREQFPGSEMEKYALFTYESLKKTKCREFVPSRDEIEALI
HRQEMTSTVYCHGGGSCKITINSHTTAGEVVEKLIRGLAMEDSRNMFALFEYNGHVDKAIESRTV
VADVLAKFEKLAATSEVGDLPWKFYFKLYCFLDTDNVPKDSVEFAFMFEQAHEAVIHGHHPAPEE
NLQVLAALRLQYLQGDYTLHAAIPPLEEVYSLQRLKARISQSTKTQMLDMWIKEEVSSARASIID
KWRKFQGMNQEQAMAKYMALIKEWPGYGSTLFDVECKEGGFPQELWLGVSADAVSVYKRGEGRPL
EVFQYEHILSFGAPLANTYKIVVDERELLFETSEVVDVAKLMKAYISMIVKKRYST

>1QYNA

MSEQNNTEMTFQIQRIYTKDISFEAPNAPHVFQKDWQPEVKLDLDTASSQLADDVYEVVLRVTVT
ASLGEETAFLCEVQQGGIFSIAGIEGTQMAHCLGAYCPNILFPYARECITSMVSRGTFPQLNLAP
VNFDALFMNYLQQQAGEGTEEHQ

>3CA8A

MNITPFPTLSPATIDAINVIGQWLAQDDFSGEVPYQADCVILAGNAVMPTIDAACKIARDQQIPL
LISGGIGHSTTFLYSAIAQHPHYNTIRTTGRAEATILADIAHQFWHIPHEKIWIEDQSTNCGENA
RFSIALLNQAVERVHTAIVVQDPTMQRRTMATFRRMTGDNPDAPRWLSYPGFVPQLGNNADSVIF
INQLQGLWPVERYLSLLTGELPRLRDDSDGYGPRGRDFIVHVDFPAEVIHAWQTLKHDAVLIEAM
ESRSLR

>1EL6A

MSLLNNKAGVISRLADFLGFRPKTGDIDVMNRQSVGSVTISQLAKGFYEPNIESAINDVHNFSIK
DVGTIITNKTGVSPEGVSQTDYWAFSGTVTDDSLPPGSPITVLVFGLPVSATTGMTAIEFVAKVR
VALQEAIASFTAINSYKDHPTDGSKLEVTYLDNQKHVLSTYSTYGITISQEIISESKPGYGTWNL
LGAQTVTLDNQQTPTVFYHFERTA

>1SDDA

AKLRQFYVAAQSIRWNYRPESTHLSSKPFETSFKKIVYREYEAYFQKEKPQSRTSGLLGPTLYAE
VGDIMKVHFKNKAHKPLSIHAQGIKYSKFSEGASYSDHTLPMEKMDDAVAPGQEYTYEWIISEHS
GPTHDDPPCLTHIYYSYVNLVEDFNSGLIGPLLICKKGTLTEDGTQKMFEKQHVLMFAVFDESKS
WNQTSSLMYTVNGYVNGTMPDITVCAHDHISWHLIGMSSGPELFSIHFNGQVLEQNHHKISAITL
VSATSTTANMTVSPEGRWTIASLIPRHFQAGMQAYIDIKNCAKKTR

>2W9YA

GAMSVASLPECVKNFFPTEQLEFSSSITADEKPVLHEVFQKHSCFSQCGEMIDEVSKKHPELGKR
LATVLEGNKKRLDGLSPAAVEYAKKLIHMVTTTLCSLTVGKPIDDADAKRLHQEFQSLSSEDQAA
LRKNNPDIKF

>2Z2NA

MEFKLQELNLTNQDTGPYGITVSDKGKVWITQHKANMISCINLDGKITEYPLPTPDAKVMCLTIS
SDGEVWFTENAANKIGRITKKGIIKEYTLPNPDSAPYGITEGPNGDIWFTEMNGNRIGRITDDGK
IREYELPNKGSYPSFITLGSDNALWFTENQNNAIGRITESGDITEFKIPTPASGPVGITKGNDDA
LWFVEIIGNKIGRITTSGEITEFKIPTPNARPHAITAGAGIDLWFTEWGANKIGRLTSNNIIEEY

PIQIKSAEPHGICFDGETIWFAMECDKIGKLTLIKDNME
>2Q83A
GEEGNSSELPLSAEDAKKLTELAENVLQGWDVQAEKIDVIQGNQMALVWKVHTDSGAVCLKRIHR
PEKKALFSIFAQDYLAKKGMNVPGILPNKKGSLYSKHGSFLFVVYDWIEGRPFELTVKQDLEFIM
KGLADFHTASVGYQPPNGVPIFTKLGRWPNHYTKRCKQMETWKLMAEAEKEDPFSQLYLQEIDGF
IEDGLRIKDRLLQSTYVPWTEQLKKSPNLCHQDYGTGNTLLGENEQIWVIDLDTVSFDLPIRDLR
KMIIPLLDTTGVWDDETFNVMLNAYESRAPLTEEQKQVMFIDMLFPYELYDVIREKYVRKSALPK
EELESAFEYERIKANALRQLI
>2YF4A
GIDPFTMSDLPCPPTNAERLHEFHRAIGAATPERPTPPPPELLRLRQTLLDEESAEVRAEIDHLL
ARQAAGEALSAGDLAPLAHELADLLYVTYGALDQLGIDADAVFAEVHRANLSKASGPRRADGKQL
KPEGWRPADVRGVIERLQHAPADD
>1PMHX
ESSVNPVVLDFEDGTVMSFGEAWGDSLKCIKKVSVSQDLQRPGNKYALRLDVEFNPNNGWDQGDL
GTWIGGVVEGQFDFTGYKSVEFEMFIPYDEFSKSQGGFAYKVVINDGWKELGSEFNITANAGKKV
KINGKDYTVIHKAFAIPEDFRTKKRAQLVFQFAGQNSNYKGPIYLDNVRIRPEDA
>1Q0PA
GEQQKRKIVLDPSGSMNIYLVLDGSDSIGASNFTGAKKSLVNLIEKVASYGVKPRYGLVTYATYP
KIWVKVSEADSSNADWVTKQLNEINYEDHKLKSGTNTKKALQAVYSMMSWPDDVPPEGWNRTRHV
IILMTDGLHNMGGDPITVIDEIRDLLYIGKDRKNPREDYLDVYVFGVGPLVNQVNINALASKKDN
EQHVFKVKDMENLEDVFYQMIDESQSLS
>2R2ZA
SNADEVENLYTQVADNEYLVQGRMLIDEFNEVFETDLHMSDVDTMAGYLITALGTIPDEGEKPSF
EVGNIKLTAEEMEGTRLLVLRVHFYDEE
>3H9MA
MSLSFTPLHTTSEAFIEKALPWLEDRYFHIAYLNPNGYTAYPQGAFRHYLAFGSEAAIHVSDATR
VFETWNEIKKGYTNEWIFVFASYDGKNSVEQLHTSKEAGIAFAAATFFIPEHVWEIQPDGILIHK
GSGSSLVTEIQHAEPSTPVQQSDIFVKQVVSKESYFNAFDELQQIIAQGDAYEINYCIPFTAKGN
ISPAATYQRLNKKTPMPFSVYYKFNTEYILSASPERFIKKTGDTIISQPIKGTSKRGKSKAEDEM
LKQQLGTSEKEQSENTMIVDLVRNDLSRTAVAGSVCVPELSGLYTFPNVHQLISTVQSTIDPACS
SIDVIQQAFPMGSMTGAPKVNVMKFIDRIESMARGPFSGTVGYMDPHDNFDFNVLIRSIFYNSAT
QELFMEAGSAITSYAKAETEYEECLLKITPMIHILNNQEGHHHHHH
>3BQ9A
MSLASISPQGSMSLLSQLEIERLKASSNSQLYKLFRNCCLAVLNAGSHTDSSADIYDSYKDFEVN
IIRRERGIKLELIEPPEEAFVDGEVIVGIRELLESVLRDILFTGERYSETDLEHADSATLTHVVF
DILRNARTLRPQEEPNMVVCWGGHSINEIEYKYTKDVGYHIGLRGLNICTGCGPGAMKGPMKGAT
IGHAKQRVEGGRYLGLTEPGIIAAEPPNPIVNELVILPDIEKRLEAFVRCAHGIVIFPGGAGTAE
ELLYLLGILMHPDNQRQSLPVILTGPASSRDYFEALDEFIGATIGDEARQLYKIIIDDPAAVAQH
MHAGMAAVKQYRRDSGDAYYFNWTLKINEEFQRPFSPTHENVAALNLHPDQPKERLAADLRRAFS
AIVAGNVKDEGIRQIRKNGVFTIHGEQSLMKRLDELLRAFVEQGRMKLPGSVYNPCYKVITDEGH
HHHHH
>2IQIA
SLSLGDQKPATIYAPTVRVTPNPAWPQVSWQLLVAKPSAARIIDSPRINVRPTPGELQVYHGAGW
AQPATDMLEDSVVRAFEDSGKIAAVARIGAGIRSDYKLAIDVRRFESDYAGQSLPAATIELNAKL

LHSSDQRVVASRTFTVARPSSSTDTAAVAAAFEQALTQVTTELVGWTLITGQQDSQTLPRAL
>2O8SA
MAPCLENVACVNLSAMPPAPPSPQPVSHKQAATLCRQGRTCALKSGSNESGGSVTSTYTSYRLIS
QDIGKSLERVSKQPDVARETEYYREKIGSVKSIDDFMADTRLYNYALKAHGLEDMAYAKAFIRKV
LTEGASDKNAFANKLSDNRYAELAKSLDFAGLGAAATATEAAKSGVIGNYARQTLEQEAGDDNNG
VRLALYFERKAPTIKSGLDFLADDALAQVFRTTFNLPDAFAAADVDKQAALIEKSINIKDLQDPE
KVGKLLERFTIMWEMQNPSTTYDPLAVFGSSSGYGISPDLLISINSLKLGGKAAALEHHHHHH
>2A4XA
MSARISLFAVVVEDMAKSLEFYRKLGVEIPAEADSAPHTEAVLDGGIRLAWDTVETVRSYDPEWQ
APTGGHRFAIAFEFPDTASVDKKYAELVDAGYEGHLKPWNAVWGQRYAIVKDPDGNVVDLFAPLP
LEHHHHHH
>4DT5A
GYSCRAVGVDGRAVTDIQGTCHAKATGAGAMASGTSEPGSTSTATATGRGATARSTSTGRGTATT
TATGTASATSNAIGQGTATTTATGSAGGRATGSATTSSSASQPTQTQTITGPGFQTAKSFARNTA
TTTVTASHHHHHH
>3NREA
GMTIYTLSHGSLKLDVSDQGGVIEGFWRDTTPLLRPGKKSGVATDASCFPLVPFANRVSGNRFVW
QGREYQLQPNVEWDAHYLHGDGWLGEWQCVSHSDDSLCLVYEHRSGVYHYRVSQAFHLTADTLTV
TLSVTNQGAETLPFGTGWHPYFPLSPQTRIQAQASGYWLEREQWLAGEFCEQLPQELDFNQPAPL
PRQWVNNGFAGWNGQARIEQPQEGYAIIMETTPPAPCYFIFVSDPAFDKGYAFDFFCLEPMSHAP
DDHHRPEGGDLIALAPGESTTSEMSLRVEWL
>1QD6C
FTLYPYDTNYLIYTQTSDLNKEAIASYDWAENARKDEVKFQLSLAFPLWRGILGPNSVLGASYTQ
KSWWQLSNSEESSPFRETNYEPQLFLGFATDYRFAGWTLRDVEMGYNHDSNGRSDPTSRSWNRLY
TRLMAENGNWLVEVKPWYVVGNTDDNPDITKYMGYYQLKIGYHLGDAVLSAKGQYNWNTGYGGAE
LGLSYPITKHVRLYTQVYSGYGESLIDYNFNQTRVGVGVMLNDLF
>1V0AA
MASAVGEKMLDDFEGVLNWGSYSGEGAKVSTKIVSGKTGNGMEVSYTGTTDGYWGTVYSLPDGDW
SKWLKISFDIKSVDGSANEIRFMIAEKSINGVGDGEHWVYSITPDSSWKTIEIPFSSFRRRLDYQ
PPGQDMSGTLDLDNIDSIHFMYANNKSGKFVVDNIKLIGALEHHHHHH
>3GN6A
GMTGLSQSQASPMQIQPGNAAFNPWTDAALDTIRDVNQALTLYAEMRVVPAHHDAFLAAIDTVSA
KLRVLPGFLSLALKQMSGDSTMVKNYPETYKGVLATAYLDGVAAGTQPYFYNLFVRFADGRAARA
AGFEALFETHIHPLLHAMAPRGGDGPELLAYRAVLQSVVAGDRHAIYRGAEEIRSFLRRPVELPE
RETVTVENHVMVPEDKHAAWEPQVAILLQVAQDTFEPQDEPSGVGLPGARDNRYYRKALSTEILR
NAHADGGLRAYIMHGVWESVWDHENSHLDPRFLAAAGPVGAAAVVGPVEPFYLTRRLVVAD
>4GQ4A
GGSSSMGLKAAQKTLFPLRSIDDVVRLFAAELGREEPDLVLLSLVLGFVEHFLAVNRVGLTYFPV
ADLSIIAALYARFTAQIRGAVDLSLYPREGGVSSRELVKKVSDVIWNSLSRSYFKDRAHIQSLFS
FITGTKLDSSGVAFAVVGACQALGLRDVHLALSEDHAWVVFGPNGEQTAEVTWHGKGNEDRRGQT
VNAGVAERSWLYLKGSYMRCDRKMEVAFMVCAINPSIDLHTDSLELLQLQQKLLWLLYDLGHLER
YPMALGNLADLEELEPTPGRPDPLTLYHKGIASAKTYYRDEHIYPYMYLAGYHCRNRNVREALQA
WADTATVIQDYNYCREDEEIYKEFFEVANDVIPNLLKEAASLLEAGSQGSALQDPECFAHLLRFY
DGICKWEEGSPTPVLHVGWATFLVQSLGRFEGQVRQKVRIVSVPAPAASPPPEGPVLTFQSEKMK

GMKELLVATKINSSAIKLQLTAQSQVQMKKQKVS

>3AI7A

MTSPVIGTPWKKLNAPVSEEALEGVDKYWRVANYLSIGQIYLRSNPLMKEPFTREDVKHRLVGHW
GTTPGLNFLIGHINRFIADHGQNTVIIMGPGHGGPAGTSQSYLDGTYTETFPKITKDEAGLQKFF
RQFSYPGGIPSHFAPETPGSIHEGGELGYALSHAYGAIMDNPSLFVPAIVGDGEAETGPLATGWQ
SNKLVNPRTDGIVLPILHLNGYKIANPTILSRISDEELHEFFHGMGYEPYEFVAGFDDEDHMSIH
RRFAELWETIWDEICDIKATAQTDNVHRPFYPMLIFRTPKGWTCPKYIDGKKTEGSWRSHQVPLA
SARDTEAHFEVLKNWLESYKPEELFDANGAVKDDVLAFMPKGELRIGANPNANGGVIRNDLKLPN
LEDYEVKEVAEYGHGWGQLEATRTLGAYTRDIIKNNPRDFRIFGPDETASNRLQASYEVTNKQWD
AGYISDEVDEHMHVSGQVVEQLSEHQMEGFLEAYLLTGRHGIWSSYESFVHVIDSMLNQHAKWLE
ATVREIPWRKPIASMNLLVSSHVWRQDHNGFSHQDPGVTSVLLNKCFHNDHVIGIYFATDANMLL
AIAEKCYKSTNKINAIIAGKQPAATWLTLDEARAELEKGAAAWDWASTAKNNDEAEVVLAAAGDV
PTQEIMAASDKLKELGVKFKVVNVADLLSLQSAKENDEALTDEEFADIFTADKPVLFAYHSYAHD
VRGLIYDRPNHDNFNVHGYEEEGSTTTPYDMVRVNRIDRYELTAEALRMIDADKYADKIDELEKF
RDEAFQFAVDNGYDHPDYTDWVYSGVNTDKKGAVTATAATAGDNEHHHHHH

>2F1NA

MAHHHHHHVGTDLTDFRVATWNLQGASATTESKWNINVRQLISGENAVDILAVQEAGSPPSTAVD
TGRVIPSPGIPVRELIWNLSTNSRPQQVYIYFSAVDALGGRVNLALVSNRRADEVFVLSPVRQGG
RPLLGIRIGNDAFFTAHAIAMRNNDAPALVEEVYNFFRDSRDPVHQALNWMILGDFNREPADLEM
NLTVPVRRASEIISPAAATQTSQRTLDYAVAGNSVAFRPSPLQAGIVYGARRTQISSDHFPVGVS
RR

>3TVJI

GSGEVTCEPGTTFKDKCNTCRCGSDGKSAVCTKLWCNQ

>1EP3B

MSQLQEMMTVVSQREVAYNIFEMVLKGTLVDEMDLPGQFLHLAVPNGAMLLRRPISISSWDKRAK
TCTILYRIGDETTGTYKLSKLESGAKVDVMGPLGNGFPVAEVTSTDKILIIGGGIGVPPLYELAK
QLEKTGCQMTILLGFASENVKILENEFSNLKNVTLKIATDDGSYGTKGHVGMLMNEIDFEVDALY
TCGAPAMLKAVAKKYDQLERLYISMESRMACGIGACYACVEHDKEDESHALKVCEDGPVFLGKQL
SL

>2HJNA

MSPVLTTPKRHAPPPEQLQNVTDFNYTPSHQKPFLQPQAGTTVTTHQDIKQIVEMTLGSEGVLNQ
AVKLPRGEDENEWLAVHCVDFYNQINMLYGSITEFCSPQTCPRMIATNEYEYLWAFQKGQPPVSV
SAPKYVECLMRWCQDQFDDESLFPSKVTGTFPEGFIQRVIQPILRRLFRVYAHIYCHHFNEILEL
NLQTVLNTSFRHFCLFAQEFELLRPADFGPLLELVMELRDR

>1Z0WA

DYKLFITEGYEVGRVNGLAVIGESAGIVLPIIAEVTPSMSKSEGRVIATGRLQEIAREAVMNVSA
IIKKYTGRDISNMDVHIQFVGTYEGVEGDSASISIATAVISAIEGIPVDQSVAMTGSLSVKGEVL
PVGGVTQKIEAAIQAGLKKVIIPKDNIDDVLLDAEHEGKIEVIPVSRINEVLEHVLEDGKKKNRL
MSKFKELELAAV

>4F55A

GGGRTNVPNGYSQNDIQLMANAVYGESRGEPYLGQVAVAAVILNRVTSASFPNTVSGVIFEPRAF
TAVADGQIYLTPNETAKKAVLDAINGWDPTGNALYYFNPDTATSKWIWTRPQIKKIGKHIFCK

>2PNWA

MSLNTPFSIDEVSFRDLPGWGQDDPRKLFPAMATILSHLRNAKPYRTGALGITAAELVSLLELAE

RGQVNSPEQARQFFETNSVPFRISPAQGKSGFVTAFYEPELEVSATPDDVWRYPIYRRPPELVDI
DNDNRPDGFDPSYAFGKADEEGISYFPDRRAIDEGCLRGRGLEIAWARSKVDLFFVHVQGAARLV
FPDGAIKRITYAAKAGHVFSPIGRLLLDRGELDPKTISMQTIRQWLADHPDEVDGVLWHNRSYIF
FREADVAGLDMGPIAAAKVPLVAGRALAVDRLIHTFGLPFFIHAPTLTHLDDGKPFARLMLALDT
GSAIVGPARGDIFTGSGFEAGELAGTVRNEADFYILLPRIAAERYRREGHHHHHH
>2OWLA
MLWFKNLMVYRLSREISLRAEEMEKQLASMAFTPCGSQDMAKMGWVPPMGSHSDALTHVANGQIV
ICARKEEKILPSPVIKQALEAKIAKLEAEQARKLKKTEKDSLKDEVLHSLLPRAFSRFSQTMMWI
DTVNGLIMVDCASAKKAEDTLALLRKSLGSLPVVPLSMENPIELTLTEWVRSGSAAQGFQLLDEA
ELKSLLEDGGVIRAKKQDLTSEEITNHIEAGKVVTKLALDWQQRIQFVMCDDGSLKRLKFCDELR
DQNEDIDREDFAQRFDADFILMTGELAALIQNLIEGLGGEAQR
>2Z72A
MGNTATEFDGPYVITPISGQSTAYWICDNRLKTTSIEKLQVNRPEHCGDLPETKLSSEIKQIMPD
TYLGIKKVVALSDVHGQYDVLLTLLKKQKIIDSDGNWAFGEGHMVMTGDIFDRGHQVNEVLWFMY
QLDQQARDAGGMVHLLMGNHEQMVLGGDLRYVHQRYDIATTLINRPYNKLYSADTEIGQWLRSKN
TIIKINDVLYMHGGISSEWISRELTLDKANALYRANVDASKKSLKADDLLNFLFFGNGPTWYRGY
FSETFTEAELDTILQHFNVNHIVVGHTSQERVLGLFHNKVIAVDSSIKVGKSGELLLLENNRLIR
GLYDGTRETLQENSLNQ
>1WUIS
LMGPRRPSVVYLHNAECTGCSESVLRAFEPYIDTLILDTLSLDYHETIMAAAGDAAEAALEQAVN
SPHGFIAVVEGGIPTAANGIYGKVANHTMLDICSRILPKAQAVIAYGTCATFGGVQAAKPNPTGA
KGVNDALKHLGVKAINIAGCPPNPYNLVGTIVYYLKNKAAPELDSLNRPTMFFGQTVHEQCPRLP
HFDAGEFAPSFESEEARKGWCLYELGCKGPVTMNNCPKIKFNQTNWPVDAGHPCIGCSEPDFWDA
MTPFYQN
>2Q22A
GMSMPNHPNLTTADAKKILNKFNCLDIAPILKPSEKESVRRALILITKLSDYQILGICADTADEG
LLAMKTYSHALGYEVPIDLPVVEGPVYIKLNGKNGLCYLDSYAGHHRGVLVSCQSYYEGGINEMY
GHLPLDLFV
>4HR9A
PNSEDKNFPRTVMVNLNIHNRNTNTNPKRSSDYYDRSTSPWNLHRNEDPERYPSVIWEAKCRHLG
CINADGNVDYHMNSVPIQQEILVLRREPPHSPNSFRLEKILVSVGCTCVTPIVHHVA
>2GA1A
GMNKKTQLLEVIAALPEELVDQALNYVQMLQNPIQITPGVCGGQARIRNTRIPVWTLVAYRQQGA
PDKELLANYPGLTAEDLSAAWHYYEQNPEQIDREIAQDDLV
>1WVGA
SIDKNFWQGKRVFVTGHTGFKGSWLSLWLTEMGAIVKGYALDAPTVPSLFEIVRLNDLMESHIGD
IRDFEKLRSSIAEFKPEIVFHMAAQPLVRLSYEQPIKTYSTNVMGTVHLLETVKQVGNIKAVVNI
TSDKCYDNREWVWGYRENEPMGGYDPYSNSKGCAELVASAFRNSFFNPANYEQHGVGLASVRAGN
VIGGGDWAKDRLIPDILRSFENNQQVIIRNPYSIRPWQHVLEPLSGYIVVAQRLYTEGAKFSEGW
NFGPRDEDAKTVEFIVDKMVTLWGDDASWLLDGENHPHEAHYLKLDCSKANMQLGWHPRWGLTET
LSRIVKWHKAWIRGEDMLICSKREISDYMSATTR
>2YFRA
MDVKQVEKKDSVDKTNAEENKDSSVKPAENATKAELKGQVKDIVEESGVDTSKLTNDQINELNKI
NFSKEAKSGTQLTYNDFKKIAKTLIEQDARYAIPFFNASKIKNMPAAKTLDAQSGKVEDLEIWDS

WPVQDAKTGYVSNWNGYQLVIGMMGVPNVNDNHIYLLYNKYGDNDFNHWKNAGPIFGLGTPVIQQ
WSGSATLNKDGSIQLYYTKVDTSDNNTNHQKLASATVYLNLEKDQDKISIAHVDNDHIVFEGDGY
HYQTYDQWKETNKGADNIAMRDAHVIDDDNGNRYLVFEASTGTENYQGDDQIYQWLNYGGTNKDN
LGDFFQILSNSDIKDRAKWSNAAIGIIKLNDDVKNPSVAKVYSPLISAPMVSDEIERPDVVKLGN
KYYLFAATRLNRGSNDDAWMATNKAVGDNVAMIGYVSDNLTHGYVPLNESGVVLTASVPANWRTA
TYSYYAVPVEGRDDQLLITSYITNRGEVAGKGMHATWAPSFLLQINPDNTTTVLAKMTNQGDWIW
DDSSENPDMMGVLEKDAPNSAALPGEWGKPVDWDLIGGYNLKPHQHHHHHH
>2IVFC
MKAKRVPGGKELLLDLDAPIWAGAESTTFEMFPTPLVMVKEVSPFLALSEGHGVIKRLDVAALHN
GSMIALRLKWASEKHDKIVDLNSFVDGVGAMFPVARGAQAVTMGATGRPVNAWYWKANANEPMEI
VAEGFSAVRRMKDKAGSDLKAVAQHRNGEWNVILCRSMATGDGLAKLQAGGSSKIAFAVWSGGNA
ERSGRKSYSGEFVDFEILK
>3AXGA
MNTTPVHALTDIDGGIAVDPAPRLAGPPVFGGPGNDAFDLAPVRSTGREMLRFDFPGVSIGAAHY
EEGPTGATVIHIPAGARTAVDARGGAVGLSGGYDFNHAICLAGGASYGLEAGAGVSGALLERLEY
RTGFAEAQLVSSAVIYDFSARSTAVYPDKALGRAALEFAVPGEFPQGRAGAGMSASAGKVDWDRT
EITGQGAAFRRLGDVRILAVVVPNPVGVIMDRAGTVVRGNYDAQTGVRRHPVFDYQEAFAEQVPP
VTEAGNTTISAIVTNVRMSPVELNQFAKQVHSSMHRGIQPFHTDMDGDTLFAVTTDEIDLPTTPG
SSRGRLSVNATALGAIASEVMWDAVLEAGK
>1SBXA
GSHMFMPSDRSTERCETVLEGETISCFVVGGEKRLCLPQILNSVLRDFSLQQINAVCDELHIYCS
RCTADQLEILKVMGILPFSAPSCGLITKTDAERLCNALLYG
>3QEKA
SDPKIVNIGAVLSTKKHEQIFREAVNQANKRHFTRKIQLQATSVTHRPNAIQMALSVCEDLISSQ
VYAILVSHPPAPTDHLTPTPISYTAGFYRIPVIGLTTRMSIYSDKSIHLSFLRTVPPYSHQALVW
FEMMRLFNWNHVILIVSDDHEGRAAQKKLETLLEGKESKSKKRNYENLDQLSYDNKRGPKADKVL
QFEPGTKNLTALLLEAKELEARVIILSASEDDATAVYKSAAMLDMTGAGYVWLVGEREISGSALR
YAPDGIIGLQLINGKNESAHISDAVAVVAQAIHELFEMENITDPPRGCVGNTNIWKTGPLFKRVL
MSSKYPDGVTGRIEFNEDGDRKFAQYSIMNLQNRKLVQVGIFNGSYIIQNDRKIIWPGG
>3KU3A
PGDQICIGYHANNSTEKVDTILERNVTVTHAKDILEKTHNGKLCKLNGIPPLELGDCSIAGWLLG
NPECDRLLSVPEWSYIMEKENPRDGLCYPGSFNDYEELKHLLSSVKHFEKVKILPKDRWTQHTTT
GGSRACAVSGNPSFFRNMVWLTEKGSNYPVAKGSYNNTSGEQMLIIWGVHHPNDETEQRTLYQNV
GTYVSVGTSTLNKRSTPEIATRPKVNGQGGRMEFSWTLLDMWDTINFESTGNLIAPEYGFKISKR
GSSGIMKTEGTLENCETKCQTPLGAINTTLPFHNVHPLTIGECPKYVKSEKLVLATGLRNVPQIE
SR
>1DS1A
MTSVDCTAYGPELRALAARLPRTPRADLYAFLDAAHTAAASLPGALATALDTFNAEGSEDGHLLL
RGLPVEADADLPTTPSSTPAPEDRSLLTMEAMLGLVGRRLGLHTGYRELRSGTVYHDVYPSPGAH
HLSSETSETLLEFHTEMAYHRLQPNYVMLACSRADHERTAATLVASVRKALPLLDERTRARLLDR
RMPCCVDVAFRGGVDDPGAIAQVKPLYGDADDPFLGYDRELLAPEDPADKEAVAALSKALDEVTE
AVYLEPGDLLIVDNFRTTHARTPFSPRWDGKDRWLHRVYIRTDRNGQLSGGERAGDVVAFTPRG
>1QFTA
NQPDWADEAANGAHQDAWKSLKADVENVYYMVKATYKNDPVWGNDFTCVGVMANDVNEDEKSIQA

EFLFMNNADTNMQFATEKVTAVKMYGYNRENAFRYETEDGQVFTDVIAYSDDNCDVIYVPGTDGN
EEGYELWTTDYDNIPANCLNKFNEYAVGRETRDVFTSACLEIAAA
>3W06A
GPMGVVEEAHNVKVIGSGEATIVLGHGFGTDQSVWKHLVPHLVDDYRVVLYDNMGAGTTNPDYFD
FDRYSNLEGYSFDLIAILEDLKIESCIFVGHSVSAMIGVLASLNRPDLFSKIVMISASPRYVNDV
DYQGGFEQEDLNQLFEAIRSNYKAWCLGFAPLAVGGDMDSIAVQEFSRTLFNMRPDIALSVGQTI
FQSDMRQILPFVTVPCHILQSVKDLAVPVVVSEYLHANLGCESVVEVIPSDGHLPQLSSPDSVIP
VILRHIRNDIAM
>2QTSA
STLHGISHIFSYERLSLKRVVWALCFMGSLALLALVCTNRIQYYFLYPHVTKLDEVAATRLTFPA
VTFCNLNEFRFSRVTKNDLYHAGELLALLNNRYEIPDTQTADEKQLEILQDKANFRNFKPKPFNM
LEFYDRAGHDIREMLLSCFFRGEQCSPEDFKVVFTRYGKCYTFNAGQDGKPRLITMKGGTGNGLE
IMLDIQQDEYLPVWGETDETSFEAGIKVQIHSQDEPPLIDQLGFGVAPGFQTFVSCQEQRLIYLP
PPWGDCKATTGDSEFYDTYSITACRIDCETRYLVENCNCRMVHMPGDAPYCTPEQYKECADPALD
FLVEKDNEYCVCEMPCNVTRYGKELSMVKIPSKASAKYLAKKYNKSEQYIGENILVLDIFFEALN
YETIEQKKAYEVAGLLGDIGGQMGLFIGASILTVLELFDYAYEVIKHR
>2PQRC
GSHMQKGQVGIFSFQNNYADSATTFRILAHLDEQRYPLPNGAAEKNLPSLFEGFKATVSIIQQR
>2HOXA
KMTWTMKAAEEEAEAVANINCSEHGRAFLDGIISEGSPKCECNTCYTGPDCSEKIQGCSADVASGD
GLFLEEYWKQHKEASAVLVSPWHRMSYFFNPVSNFISFELEKTIKELHEVVGNAAAKDRYIVFGV
GVTQLIHGLVISLSPNMTATPDAPESKVVAHAPFYPVFREQTKYFDKKGYVWAGNAANYVNVSNP
EQYIEMVTSPNNPEGLLRHAVIKGCKSIYDMVYYWPHYTPIKYKADEDILLFTMSKFTGHSGSRF
GWALIKDESVYNNLLNYMTKNTEGTPRETQLRSLKVLKEVVAMVKTQKGTMRDLNTFGFKKLRER
WVNITALLDQSDRFSYQELPQSEYCNYFRRMRPPSPSYAWVKCEWEEDKDCYQTFQNGRINTQNG
VGFEASSRYVRLSLIKTQDDFDQLMYYLKDMVKAKRK
>3HLZA
GMQGKKFISPGAWFSMNYPSDWNEFEDGEGSFLFYNPDVWTGNFRISAFKGNASYGKDAIRQELK
ENDSASLVKIGTWDCAYSKEMFQEEGTYYTSHLWITGTGNIAFECSFTVPKGGSAKEAEEVIATL
EARKEGEKYPAELIPVRLSEIYQINEGYEWVVSTVKQELKKDFQGVEEDLEKIQQVIDSGKISPK
KKDEWLAIGITVCAILTNEVEGMEWKTLIDGNREVPVLEYQGRTIDPMKIAWSKVKAGQPCNIAE
AYQSAIDHH
>2WH7A
MGSSHHHHHHSSGLVPRGSHNAVNIVMRQPTTPNFSSALNITSANEGGSAMQIRGVEKALGTLKI
THENPSVDKEYDKNAAALSIDIVKKQKGGKGTAAQGIYINSTSGTTGKLLRIRNLNDDKFYVKPD
GGFYAKETSQIDGNLKLKDPIANDHAATKAYVDGEVEKLKALLAAKQM
>2V0XA
AKSVVSHSLTTLGVEVSKPPPQHDKIEASEPSFPLHESILKVVEEEWQQIDRQLPSVACRYPVSS
IEAARILSVPKVDDEILGFISEATPAAATQASSTESCDKHLDLALCRSYEAAASALQIAAHTAFV
AKSLQADISQAAQIINSDPSDAQQALRILNRTYDAASYLCDAAFDEVRMSACAMGSSTMGRRYLW
LKDCKISPASKNKLTVAPFKGGTLFGGEVHKVIKKRGNKQ
>4FUSA
MGSSHHHHHHSSGLVPRGSHMASSAEEYNERFMEMWNKIHDPANGYFSADGGPYHSVETLIVEAP
DHGHESTSEAYSYFLLLEAYYGKVTGDWSKLRNAWAKMEEHIIPTQEMQPTNNFYNPSKPASYAA

EHAQPSGYPSQLEFGVPVGEDPISAKLAQTYGSWDVYGMHWLLDMDNIYGYGNLGDGVSTPSYIN
TFQRGEQESVWETVTHPSWESFKWGGPNGFLPLFTKDNNYSRQWRYTNAPDADARAVQVMYWAYQ
WIKEQGKDPEQEVPGLMAKAAKMGDYLRLAMFDKYFKKMGTQDKNAQGGKGYESAHYLMSWYYAW
GGAADANAGWAFRIGSSHVHFGYQNPIAAMALSEFDPLKPRTPGATEDWATGLKRSMEFYTWLQS
AEGGIAGGATNSWDGSYKPHPQDRADATFYGMVYDENPVYHDPGSGTWFGWQAWSMQRVAEYYYL
KGDAQAKQLMDKWAPWVLSNINWLEDGSFEIPATLEWTGKPEKWDPANPKANTNLHVSVVDHGQD
LGIAAGVAKALMFYAAAAEKYDTPQNEAKEASKKLLDAMWTHFKTPKGLAAPEKRGDYARFFDKV
YVPGEFNGSMANGDAINSESTFLSMRSFYLDDPMFKQVEDALNSGEDPVFTYHRFWAQTEAATAY
ANYAALFEGDNPCDEGCAPTAQPLSVSTRVNKAVSITLKGTDSDG
>2QZBA
SLAANPWNWFGSSTKVSEQGVGELTASTPLQEQAIADALDGDYRLRSGMKTANGNVVRFFEVMKG
DNVAMVINGDQGTISRIDVLDSDIPADTGVKIGTPFSDLYSKAFGNCQKADGDDNRAVECKAEGS
QHISYQFSGEWRGPEGLMPSDDTLKNWKVSKIIWRR
>3GIWA
GMGGAALPDNGWPADRIDTESAHSARIYDYIIGGKDYYPADKEAGDAMSREWPALPVHMRANRDW
MNRAVAHLAKEAGIRQFLDIGTGIPTSPNLHEIAQSVAPESRVVYVDNDPIVLTLSQGLLASTPE
GRTAYVEADMLDPASILDAPELRDTLDLTRPVALTVIAIVHFVLDEDDAVGIVRRLLEPLPSGSY
LAMSIGTAEFAPQEVGRVAREYAARNMPMRLRTHAEAEEFFEGLELVEPGIVQVHKWHPDAATAD
GIRDEDIAMYGAVARKP
>3B79A
SNAMKDPLLNSLIYVSRYYGLANSPEALVNGLPLSDGKLTPFLLPRAAERAGLVAKENRAELEKI
SSLILPAILVLKGGDSCVLNSINMETREAEVTTLESGMVPISIPLEDLLEQYTGRYFLVKKQFR
>4I6MA
MTLNRKCVVIHNGSHRTVAGFSNVELPQCIIPSSYIKRTDEGGEAEFIFGTYNMIDAAAEKRNGD
EVYTLVDSQGLPYNWDALEMQWRYLYDTQLKVSPEELPLVITMPATNGKPDMAILERYYELAFDK
LNVPVFQIVIEPLAIALSMGKSSAFVIDIGASGCNVTPIIDGIVVKNAVVRSKFGGDFLDFQVHE
RLAPLIKEENDMENMADEQKRSTDVWYEASTWIQQFKSTMLQVSEKDLFELERYYKEQADIYAKQ
QEQLKQMDQQLQYTALTGSPNNPLVQKKNFLFKPLNKTLTLDLKECYQFAEYLFKPQLISDKFSP
EDGLGPLMAKSVKKAGASINSMKANTSTNPNGLGTSHINTNVGDNNSTASSSNISPEQVYSLLLT
NVIITGSTSLIEGMEQRIIKELSIRFPQYKLTTFANQVMMDRKIQGWLGALTMANLPSWSLGKWY
SKEDYETLKRDRKQSQATNATN
>4JPQA
GQSGKSLSVKKVMCTASPEGEAVPSLLDGNGIEFQPLDVVNWKDYPYKPEVSFRIAHTGREILLH
YKVKEASVRAVASGDNGRVWEDACVEFFVSPEGDDRYYNFECNCAGRLLIQGGAVNERRPTASQE
VLGMVKRWSSLAGEPFEERLGECSWELVMVIPVSAFFQHSVGSLDGKTMKGNFYKCGDKLQTPHF
LSWSPIGLERPMFHCPAFFGTLSFE
>4F2EA
GAMGQKAQQKNGYQEIRVEVMGGYTPELIVLKKSVPARIVFDRKDPSPCLDQIVFPDFGVHANLP
MGEEYVVEITPEQAGEFSFACGMNMMHGKMIVE
>4IEFA
GPLGSQPAERGRNPQVRLLSAEQSMSKVQFRMDNLQFTGVQTSKGVAQVPTFTEGVNISEKGTPI
LPILSRSLAVSETRAMKVEVVSSKFIEKKDVLIAPSKGVISRAENPDQIPYVYGQSYNEDKFFPG
EIATLSDPFILRDVRGQVVNFAPLQYNPVTKTLRIYTEIVVAVSETAEAGQNTISLVKNSTFTGF
EDIYKSVFMNYEATR

>1Z3XA
PEFMVTTEPALADLQEQLYNGNEKSQLAAMSTLSTAGTEGYHLLQEFLKDSATFSPPPAPWIRGQ
AYRLLFHSPEASVQAFLQQHYPQGVIPLRSDRGVDYQELAKLLVAEKFEAADRLTTQKLCELAGP
LAQKRRWLYFTEVEQLPIPDLQTIDQLWLAFSLGRFGYSVQRQLWLGCGQNWDRLWEKIGWRQGK
RWPRYPNEFIWDLSAPRGHLPLTNQLRGVQVLNALLNHPAWTA
>2J8BA
MLQCYNCPNPTADCKTAVNCSSDFDACLITKAGLQVYNKCWKFEHCNFNDVTTRLRENELTYYCC
KKDLCNFNEQLENG
>3F0DA
MAHHHHHHMGTLEAQTQGPGSMDFRIGQGYDVHQLVPGRPLIIGGVTIPYERGLLGHSDADVLLH
AITDALFGAAALGDIGRHFSDTDPRFKGADSRALLRECASRVAQAGFAIRNVDSTIIAQAPKLAP
HIDAMRANIAADLDLPLDRVNVKAKTNEKLGYLGRGEGIEAQAAALVVREAAA
>3DANA
MDPSSKPLREIPGSYGIPFFQPIKDRLEYFYGTGGRDEYFRSRMQKYQSTVFRANMPPGPFVSSN
PKVIVLLDAKSFPILFDVSKVEKKDLFTGTYMPSTKLTGGYRVLSYLDPSEPRHAQLKNLLFFML
KNSSNRVIPQFETTYTELFEGLEAELAKNGKAAFNDVGEQAAFRFLGRAYFNSNPEETKLGTSAP
TLISSWVLFNLAPTLDLGLPWFLQEPLLHTFRLPAFLIKSTYNKLYDYFQSVATPVMEQAEKLGV
PKDEAVHNILFAVCFNTFGGVKILFPNTLKWIGLAGENLHTQLAEEIRGAIKSYGDGNVTLEAIE
QMPLTKSVVYESLRIEPPVPPQYGKAKSNFTIESHDATFEVKKGEMLFGYQPFATKDPKVFDRPE
EYVPDRFVGDGEALLKYVWWSNGPETESPTVENKQCAGKDFVVLITRLFVIELFRRYDSFEIELG
ESPLGAAVTLTFLKRASI
>2WQKA
MPTFLLVNDDGYFSPGINALREALKSLGRVVVVAPDRNLSGVGHSLTFTEPLKMRKIDTDFYTVI
DGTPADCVHLGYRVILEEKKPDLVLSGINEGPNLGEDITYSGTVSGAMEGRILGIPSIAFSAFGR
ENIMFEEIAKVCVDIVKKVLNEGIPEDTYLNVNIPNLRYEEIKGIKVTRQGKRAYKERVFKYIDP
YGKPFYWIAAEEFGWHAEEGTDYWAVLNGYVSVTPLHLDLTNYKVMKSIKYLEDSP
>2V73A
GMASIKGEVDEIANYGNLKITKEEERVNITGDLEKFSSLEEGTIVTRFNMNDTSIQSLIGLSDGN
KANNYFSLYVSGGKVGYELRRQEGNGDFNVHHSADVTFNRGINTLALKIEKGIGAKIFLNGSLVK
TVSDPNIKFLNAINLNSGFIGKTDRANGYNEYLFRGNIDFMNIYDKPVSDNYLLRKTGETK
>1NOWA
AKPGPALWPLPLSVKMTPNLLHLAPENFYISHSPNSTAGPSCTLLEEAFRRYHGYIFGFYKWHHE
PAEFQAKTQVQQLLVSITLQSECDAFPNISSDESYTLLVKEPVAVLKANRVWGALRGLETFSQLV
YQDSYGTFTINESTIIDSPRFSHRGILIDTSRHYLPVKIILKTLDAMAFNKFNVLHWHIVDDQSF
PYQSITFPELSNKGSYSLSHVYTPNDVRMVIEYARLRGIRVLPEFDTPGHTLSWGKGQKDLLTPC
YSRQNKLDSFGPINPTLNTTYSFLTTFFKEISEVFPDQFIHLGGDEVEFKCWESNPKIQDFMRQK
GFGTDFKKLESFYIQKVLDIIATINKGSIVWQEVFDDKAKLAPGTIVEVWKDSAYPEELSRVTAS
GFPVILSAPWYLDLISYGQDWRKYYKVEPLDFGGTQKQKQLFIGGEACLWGEYVDATNLTPRLWP
RASAVGERLWSSKDVRDMDDAYDRLTRHRCRMVERGIAAQPLYAGYCNHENM
>3FEGA
GSSHHHHHHSSGLVPRGSRRRASSLSRDAERRAYQWCREYLGGAWRRVQPEELRVYPVSGGLSNL
LFRCSLPDHLPSVGEEPREVLLRLYGAILQGVDSLVLESVMFAILAERSLGPQLYGVFPEGRLEQ
YIPSRPLKTQELREPVLSAAIATKMAQFHGMEMPFTKEPHWLFGTMERYLKQIQDLPPTGLPEMN
LLEMYSLKDEMGNLRKLLESTPSPVVFCHNDIQEGNILLLSEPENADSLMLVDFEYSSYNYRGFD

IGNHFCEWVYDYTHEEWPFYKARPTDYPTQEQQLHFIRHYLAEAKKGETLSQEEQRKLEEDLLVE
VSRYALASHFFWGLWSILQASMSTIEFGYLDYAQSRFQFYFQQKGQLTSVHSSS
>1R75A
MAHHHHHHMGSRISKEAAPVTFKNGKPTVKGTKTYPMFSNILYRIADTEARRWAFYNDSKELIIH
VAVLFDYDSQIVPLGDTTAFRIDDPDEGNEDDFGKYLCEVDVRPLETQMFVEGSVTGWRVDTLEA
RTAEDERGYRL
>4KGDA
MVMKQTKQTNILAGAAVIKVLEAWGVDHLYGIPGGSINSIMDALSAERDRIHYIQVRHEEVGAMA
AAADAKLTGKIGVCFGSAGPGGTHLMNGLYDAREDHVPVLALIGQFGTTGMNMDTFQEMNENPIY
ADVADYNVTAVNAATLPHVIDEAIRRAYAHQGVAVVQIPVDLPWQQIPAEDWYASANSYQTPLLP
EPDVQAVTRLTQTLLAAERPLIYYGIGARKAGKELEQLSKTLKIPLMSTYPAKGIVADRYPAYLG
SANRVAQKPANEALAQADVVLFVGNNYPFAEVSKAFKNTRYFLQIDIDPAKLGKRHKTDIAVLAD
AQKTLAAILAQVSERESTPWWQANLANVKNWRAYLASLEDKQEGPLQAYQVLRAVNKIAEPDAIY
SIDVGDINLNANRHLKLTPSNRHITSNLFATMGVGIPGAIAAKLNYPERQVFNLAGDGGASMTMQ
DLATQVQYHLPVINVVFTNCQYGFIKDEQEDTNQNDFIGVEFNDIDFSKIADGVHMQAFRVNKIE
QLPDVFEQAKAIAQHEPVLIDAVITGDRPLPAEKLRLDSAMSSAADIEAFKQRYEAQDLQPLSTY
LKQFGLDDLQHQIGQGGF
>2VECA
MHHHHHHGTSLYKKAGSENLYFQGITTRTARQCGQADYGWLQARYTFSFGHYFDPKLLGYASLRV
LNQEVLAPGAAFQPRTYPKVDILNVILDGEAEYRDSEGNHVQASAGEALLLSTQPGVSYSEHNLS
KDKPLTRMQLWLDACPQRENPLIQKLALNMGKQQLIASPEGAMGSLQLRQQVWLHHIVLDKGESA
NFQLHGPRAYLQSIHGKFHALTHHEEKAALTCGDGAFIRDEANITLVADSPLRALLIDLPV
>2BLNA
MKTVVFAYHDMGCLGIEALLAAGYEISAIFTHTDNPGEKAFYGSVARLAAERGIPVYAPDNVNHP
LWVERIAQLSPDVIFSFYYRHLIYDEILQLAPAGAFNLHGSLLPKYRGRAPLNWVLVNGETETGV
TLHRMVKRADAGAIVAQLRIAIAPDDIAITLHHKLCHAARQLLEQTLPAIKHGNILEIAQRENEA
TCFGRRTPDDSFLEWHKPASVLHNMVRAVADPWPGAFSYVGNQKFTVWSSRVHPHASKAQPGSVI
SVAPLLIACGDGALEIVTGQAGDGITMQGSQLAQTLGLVQGSRLN
>1HYOA
GSMSFIPVAEDSDFPIQNLPYGVFSTQSNPKPRIGVAIGDQILDLSVIKHLFTGPALSKHQHVFD
ETTLNNFMGLGQAAWKEARASLQNLLSASQARLRDDKELRQRAFTSQASATMHLPATIGDYTDFY
SSRQHATNVGIMFRGKENALLPNWLHLPVGYHGRASSIVVSGTPIRRPMGQMRPDNSKPPVYGAC
RLLDMELEMAFFVGPGNRFGEPIPISKAHEHIFGMVLMNDWSARDIQQWEYVPLGPFLGKSFGTT
ISPWVVPMDALMPFVVPNPKQDPKPLPYLCHSQPYTFDINLSVSLKGEGMSQAATICRSNFKHMY
WTMLQQLTHHSVNGCNLRPGDLLASGTISGSDPESFGSMLELSWKGTKAIDVGQGQTRTFLLDGD
EVIITGHCQGDGYRVGFGQCAGKVLPALSPA
>3QBXA
MPRYLGLMSGTSLDGMDIVLIEQGDRTTLLASHYLPMPAGLREDILALCVPGPDEIARAAEVEQR
WVALAAQGVRELLLQQQMSPDEVRAIGSHGQTIRHEPARHFTVQIGNPALLAELTGIDVVADFRR
RDVAAGGQGAPLVPAFHQALFGDDDTSRAVLNIGGFSNVSLLSPGKPVRGFDCGPGNVLMDAWIH
HQRGEHFDRDGAWAASGQVNHALLASLLADEFFAARGPKSTGRERFNLPWLQEHLARHPALPAAD
IQATLLELSARSISESLLDAQPDCEEVLVCGGGAFNTALMKRLAMLMPEARVASTDEYGIPPAWM
EGMAFAWLAHRFLERLPGNCPDVTGALGPRTLGALYPAGSHHHHHH
>2HY5A

MKFALQINEGPYQHQASDSAYQFAKAALEKGHEIFRVFFYHDGVNNSTRLTTPPQDDRHIVNRWA
ELAEQYELDMVVCVAAAQRRGIVDEGEASRNGKDATNIHPKFRISGLGQLVEAAIQADRLVVFGD
>1MQSA
GKSASMAVEEIASRKDISLRDMQISAILKMLFLNKDLNNNDNITTITDDIFNQQEIIWKVLILDI
KSTATISSVLRVNDLLKAGITVHSLIKQDRSPLPDVPAIYFVSPTKENIDIIVNDLKSDKYSEFY
INFTSSLPRNLLEDLAQQVSITGKSDKIKQVYDQYLDFIVTEPELFSLEISNAYLTLNDPKTTEE
EITGLCANIADGLFNTVLTINSIPIIRAAKGGPAEIIAEKLGTKLRDFVINTNSSSTSTLQGNDS
LERGVLIILDRNIDFASMFSHSWIYQCMVFDIFKLSRNTVTIPLESKENGTDNTTAKPLATKKYD
IEPNDFFWMENSHLPFPEAAENVEAALNTYKEEAAEITRKTGVTNISDLDPNSNNDTVQIQEVVK
KLPELTAKKNTIDTHMNIFAALLSQLESKSLDTFFEVEQDPGSTKTRSRFLDILKDGKTNNLEDK
LRSFIVLYLTSTTGLPKDFVQNVENYFKENDYDINALKYVYKLREFMQLSNMSLQNKSLEDGSDS
AFKPSNLTLSGIYGLTEGKLQGGVGSLISGIKKLLPEKKTIPITNVVDAIMDPLNSSQKNLETTD
SYLYIDPKITRGSHTRKPKRQSYNKSLVFVVGGGNYLEYQNLQEWAHSQLHNPKKVMYGSTAITT
PAEFLNEISRLGASNSSNNDA
>1I0RA
MDVEAFYKISYGLYIVTSESNGRKCGQIANTVFQLTSKPVQIAVCLNKENDTHNAVKESGAFGVS
VLELETPMEFIGRFGFRKSSEFEKFDGVEYKTGKTGVPLVTQHAVAVIEAKVVKECDVGTHTLFV
GEAVDAEVLKDAEVLTYADYHLMKKGKTPRTATVYFESK
>3RLKA
GHHHHHHSKPDPVIPDPPIDPPPGTGKYTCPFAIWSLEEVYEPPTKNRPWPIYNAVELQPREFDV
ALKDLLGNTKWRDWDSRLSYTTFRGCRGNGYIDLDATYLATDQAMRDQKYDIREGKKPGAFGNIE
RFIYLKSINAYCSLSDIAAYHADGVIVGFWRDPSSGGAIPFDFTKFDKTKCPIQAVIVVPRA
>2BU3A
FCLTLRRRYTMGHHHHHHHHHHSSGHIEGRHMKLEQTLTLSPNLIGFNSNEGEKLLLTSRSREDF
FPLSMQFVTQVNQAYCGVASIIMVLNSLGINAPETAQYSPYRVFTQDNFFSNEKTKAVIAPEVVA
RQGMTLDELGRLIASYGVKVKVNHASDTNIEDFRKQVAENLKQDGNFVIVNYLRKEIGQERGGHI
SPLAAYNEQTDRFLIMDVSRYKYPPVWVKTTDLWKAMNTVDSVSQKTRGFVFVSKTQDD
>4IOXA
MGSSHHHHHHSSGLVPRGSHMTGDDRPVLTKSAGERFLLYRPSTTTNSGLMAPDLYVYVDPAFTA
NTRASGTGVAVVGRYRDDYIIFALEHFFLRALTGSAPADIARCVVHSLTQVLALHPGAFRGVRVA
VEGNSSQDSAVAIATHVHTEMHRLLASEGADAGSGPELLFYHCEPPGSAVLYPFFLLNKQKTPAF
EHFIKKFNSGGVMASQEIVSATVRLQTDPVEYLLEQLNNLTETVSPNTDVRTYSGKRNGASDDLM
VAVIMAIYLAAQAGPPHTFAPITRVS
>3E5TA
MRGSHHHHHHGIHMNSLIKENMRMMVVMEGSVNGYQFKCTGEGDGNPYMGTQTMRIKVVEGGPLP
FAFDILATSFMYGSKTFIKHTKGIPDFFKQSFPEGFTWERVTRYEDGGVFTVMQDTSLEDGCLVY
HAKVTGTNFPSNGAVMQKKTKGWEPNTEMLYPADGGLRGYSQMALNVDGGGYLSCSFETTYRSKK
TVENFKMPGFHFVDHRLERLEESDKEMFVVQHEHAVAKFCDLPSKLGRL
>3ZSSA
MGSSHHHHHHSSGLVPRGSHMPATHHSSATSAERPTVVGRIPVLDVRPVVQRGRRPAKAVTGESF
EVSATVFREGHDAVGANVVLRDPRGRPGPWTPMRELAPGTDRWGATVTAGETGTWSYTVEAWGDP
VTTWRHHARIKIPAGLDTDLVLEEGARLYERAAADVPGREDRRELLAAVDALRDESRPAASRLAA
ALTPQVDAVLARHPLRDLVTSSDPLPLLVERERALYGAWYEFFPRSEGTPHTPHGTFRTAARRLP
AIAAMGFDVVYLPPIHPIGTTHRKGRNNTLSATGDDVGVPWAIGSPEGGHDSIHPALGTLDDFDH

FVTEAGKLGLEIALDFALQCSPDHPWVHKHPEWFHHRPDGTIAHAENPPKKYQDIYPIAFDADPD
GLATETVRILRHWMDHGVRIFRVDNPHTKPVAFWERVIADINGTDPDVIFLAEAFTRPAMMATLA
QIGFQQSYTYFTWRNTKQELTEYLTELSGEAASYMRPNFFANTPDILHAYLQHGGRPAFEVRAVL
AATLSPTWGIYSGYELCENTPLREGSEEYLDSEKYQLKPRDWTRAAREGTTIAPLVTRLNTIRRE
NPALRQLRDLHFHPTDKEEVIAYSKRQGSNTVLVVVNLDPRHTQEATVSLDMPQLGLDWHESVPV
RDELTGETYHWGRANYVRLEPGRTPAHVCTVLRPSHPQIGGSHTT
>4J5TA
AEFMEEYQKFTNESLLWAPYRSNCYFGMRPRYVHESPLIMGIMWFNSLSQDGLHSLRHFATPQDK
LQKYGWEVYDPRIGGKEVFIDEKNNLNLTVYFVKSKNGENWSVRVQGEPLDPKRPSTASVVLYFS
QNGGEIDGKSSLAMIGHDGPNDMKFFGYSKELGEYHLTVKDNFGHYFKNPEYETMEVAPGSDCSK
TSHLSLQIPDKEVWKARDVFQSLVSDSIRDILEKEETKQRPADLIPSVLTIRNLYNFNPGNFHYI
QKTFDLTKKDGFQFDITYNKLGTTQSISTREQVTELITWSLNEINARFDKQFSFGEGPDSIESVE
VKRRFALETLSNLLGGIGYFYGNQLIDRETEFDESQFTEIKLLNAKEEGPFELFTSVPSRGFFPR
GFYWDEGFHLLQIMEYDFDLAFEILASWFEMIEDDSGWIAREIILGNEARSKVPQEFQVQNPNIA
NPPTLLLAFSEMLSRAIENIGDFNSDSYHQVMFNSRTAKFMTNNLEANPGLLTEYAKKIYPKLLK
HYNWFRKSQTGLIDEYEEILEDEGIWDKIHKNEVYRWVGRTFTHCLPSGMDDYPRAQPPDVAELN
VDALAWVGVMTRSMKQIAHVLKLTQDEQRYAQIEQEVVENLDLLHWSENDNCYCDISIDPEDDEI
REFVCHEGYVSVLPFALKLIPKNSPKLEKVVALMSDPEKIFSDYGLLSLSRQDDYFGKDENYWRG
PIWMNINYLCLDAMRYYYPEVILDVAGEASNAKKLYQSLKINLSNNIYKVWEEQGYCYENYSPID
GHGTGAEHFTGWTALVVNILGRFRSHHHHHH
>1K5NA
GSHSMRYFHTSVSRPGRGEPRFITVGYVDDTLFVRFDSDAASPREEPRAPWIEQEGPEYWDRETQ
ICKAKAQTDREDLRTLLRYYNQSEAGSHTLQNMYGCDVGPDGRLLRGYHQHAYDGKDYIALNEDL
SSWTAADTAAQITQRKWEAARVAEQLRAYLEGECVEWLRRYLENGKETLQRADPPKTHVTHHPIS
DHEATLRCWALGFYPAEITLTWQRDGEDQTQDTELVETRPAGDRTFQKWAAVVVPSGEEQRYTCH
VQHEGLPKPLTLRWEP
>1LMLA
VVRDVNWGALRIAVSTEDLTDPAYHCARVGQHVKDHAGAIVTCTAEDILTNEKRDILVKHLIPQA
VQLHTERLKVQQVQGKWKVTDMVGDICGDFKVPQAHITEGFSNTDFVMYVASVPSEEGVLAWATT
CQTFSDGHPAVGVINIPAANIASRYDQLVTRVVTHEMAHALGFSGPFFEDARIVANVPNVRGKNF
DVPVINSSTAVAKAREQYGCDTLEYLEVEDQGGAGSAGSHIKMRNAQDELMAPAAAAGYYTALTM
AIFQDLGFYQADFSKAEVMPWGQNAGCAFLTNKCMEQSVTQWPAMFCNESEDAIRCPTSRLSLGA
CGVTRHPGLPPYWQYFTDPSLAGVSAFMDYCPVVVPYSDGSCTQRASEAHASLLPFNVFSDAARC
IDGAFRPKATDGIVKSYAGLCANVQCDTATRTYSVQVHGSNDYTNCTPGLRVELSTVSNAFEGGG
YITCPPYVEVCQGNVQAAKDGGN
>3OT9A
GSHMASNKYKRIFLVVMDSVGIGEAPDAEQFGDLGSDTIGHIAEHMNGLQMPNMVKLGLGNIREM
KGISKVEKPLGYYTKMQEKSTGKDTMTGHWEIMGLYIDTPFQVFPEGFPKELLDELEEKTGRKII
GNKPASGTEILDELGQEQMETGSLIVYTSADSVLQIAAHEEVVPLDELYKICKIARELTLDEKYM
VGRVIARPFVGEPGNFTRTPNRHDYALKPFGRTVMNELKDSDYDVIAIGKISDIYDGEGVTESLR
TKSNMDGMDKLVDTLNMDFTGLSFLNLVDFDALFGHRRDPQGYGEALQEYDARLPEVFAKLKEDD
LLLITADHGNDPIHPGTDHTREYVPLLAYSPSMKEGGQELPLRQTFADIGATVAENFGVKMPEYG
TSFLNELKK
>3SKVA

MGSSHHHHHHSSGLVPRGSHMTTQNTARARADRSVSPTDPALTYRGAVSLQDRDGWLAPWRAPHE
DAYLYFPKGSVGRLAQTSGVRLHLRTDSPWLAVRYEAVGPKPKPGEPQPPAEPALLDVLVDGELA
RTVELKLDADAELHVDGLPAGDKLVELWLPTLLQFRLAEVRLEAGATLEKDTSSKPHWIHYGDSI
CHGRGAASPSRTWLALAARAEGLDLQSLSFAADGSHLQPMFARLIRDLPADLISLRVGTSNFMDG
DGFVDFPANLVGFVQIIRERHPLTPIVLGSSVYSPFWDELPADDKPTVADYREQVVKVAELLRKH
GDQNVHYLDGMRVWGPERGMELYLEKPDKYPTHPNAVGHEIFAESSRREMAALGVLPVRG
>3NOJA
MNTLIGKTGIVVRNIQRAELDSIDALGRLGVATVHEAQNRKGLLSSKMRPIQQGTSLAGSAVTVL
VAPGDNWMFHVAVEQCRPGDVLVVSPSSPCTDGYFGDLLATSLQARGVRALIVDAGVRDTQTLRD
MGFAVWARAINAQGTVKETLGSVNLPVICGGQLINPGDIVVADDDGVVVVRRDECESTLVAAAER
AGLEEEKRLRLAAGELGLDIYKMRERLEAKGLRYVDNIEDLEG
>4INWA
SPEIMKDLSINFGKALDTCKKELDLPDSINEDFYKFWKEDYEITNRLTGCAIKCLSEKLEMVDAD
GKLHHGNAREFAMKHGADDAMAKQLVDLIHGCEKSIPPNDDRCMEVLSIAMCFKKEIHNLKWAPN
MEVVVGEVLA
>1YM3A
MAHHHHHHGPNTNPVAAWKALKEGNERFVAGRPQHPSQSVDHRAGLAAGQKPTAVIFGCADSRVA
AEIIFDQGLGDMFVVRTAGHVIDSAVLGSIEYAVTVLNVPLIVVLGHDSCGAVNAALAAINDGTL
PGGYVRDVVERVAPSVLLGRRDGLSRVDEFEQRHVHETVAILMARSSAISERIAGGSLAIVGVTY
QLDDGRAVLRDHIGNIGEEV
>3RBYA
HMPALLKRLLFQVGPHPNERTFTLSSVSTDGHYISLRPFVKPSGDELSFPFEWAFAGTNETVKAN
DQGNGVVTQDFNFWLDTNVYLNVPNTHRGEVNTTWKNWDSGCVEETGAVYPFGADKESVSFREMW
QPVDPSREDLVIVSPNNEKFSSNARSIVLKVTDEAYDGLVIVIGRWIQGFLSQKNNNTIEGLNFI
RLLEKDSGKSEFLLSYGKEVNKIPQSYENLKKGSTVTSNGLNWEVIEYHA
>2TRCP
EGQATHTGPKGVINDWRKFKLESEDGDSIPPSKKEILRQMSSPQSRDDKDSKERMSRKMSIQEYE
LIHQDKEDEGCLRKYRRQCMQDMHQKLSFGPRYGFVYELETGEQFLETIEKEQKVTTIVVNIYED
GVRGCDALNSSLECLAAEYPMVKFCKIRASNTGAGDRFSSDVLPTLLVYKGGELISNFISVAEQF
AEDFFAADVESFLNEYGLLPER
>2HNUA
VRTCLPCGPGGKGRCFGPSICCGDELGCFVGTAEALRCQEENYLPSPCQSGQKPCGSGGRCAAAG
ICCSPDGCHEDPACDP
>1VJNA
MGSDKIHHHHHHMKITWFGHACFALEMEGKTIVTDPFDESVGYPIPNVTADVVTESHQHFDHNAH
HLVKGNFRVIDRPGAYTVNGVKIKGVETFHDPSHGRERGKNIVFVFEGEGIKVCHLGDLGHVLTP
AQVEEIGEIDVLLVPVGGTYTIGPKEAKEVADLLNAKVIIPMHYKTKYLKFNLLPVDDFLKLFDS
YERVGNILELFEKPKERKVVVMEVQ
>2IXDA
MSGLHILAFGAHADDVEIGMAGTIAKYTKQGYEVGICDLTEADLSSNGTIELRKEEAKVAARIMG
VKTRLNLAMPDRGLYMKEEYIREIVKVIRTYKPKLVFAPYYEDRHPDHANCAKLVEEAIFSAGIR
KYMPELSPHRVESFYNYMINGFHKPNFCIDISEYLSIKVEALEAYESQFSTGSDGVKTPLTEGYV
ETVIAREKMFGKEVGVLYAEGFMSKKPVLLHADLLGGCKLGHHHHHH
>4IUMA

GYNPPGDGACGYRCLAFMNGATVVSAGCSSDLWCDDELAYRVFQLSPTFTVTIPGGRVCPNAKYA
MICDKQHWRVKRAKGVGLCLDESCFRGICNCQRMSGPPPAPVSAAVLDHILEAATFGNVRVVTPE
GQGSSGHHHHHH

>3PZ7A

GPHMSSTCTKVLYFTDRSLTPFMVNIPKRLEEVTLKDFKAAIDREGNHRYHFKAMDPEFGTVKEE
IFHDDDAIPGWEGKIVAWVEEDHGEN

>2Q88A

RDENKLEELKEQGFARIAIANEPPFTAVGADGKVSGAAPDVAREIFKRLGVADVVASISEYGAMI
PGLQAGRHDAITAGLFMKPERCAAVAYSQPILCDAEAFALKKGNPLGLKSYKDIADNPDAKIGAP
GGGTEEKLALEAGVPRDRVIVVPDGQSGLKMLQDGRIDVYSLPVLSINDLVSKANDPNVEVLAPV
EGAPVYCDGAAFRKGDEALRDAFDVELAKLKESGEFAKIIEPYGFSAKAAMSTTREKLCAAK

>3OYZA

MTERRHDREFVRTFFTSPTAVEGEDDSAKMLRRAAGLRGMQAPDVWVPDNEDATAPSMRDEGAEN
IVEVISEQGAEFPGEIHPRMVWHRDSPETRYQGFQHMLDITDPERGAVEHIHGFVIPEVGGIDDW
KKADEFFTIVEHEHGLDEGSLAMSVIIESGEAELAMGDLRDEMGKPTNNLERLFLLVDGEVDYTK
DMRAMTPTGELPAWPELRHNTSRGASAAGCVAVDGPYDDIRDVEGYRERMTDNQAKGMLGIWSLT
PGQVVEANTSPLPPKTGSWLLDADGEEVELASEDGVEAYDGDRLSLEATDGGYELRVGGDARELT
ADELREELLGLTSYVPSMDDIVDSMEEFEAAKEAGRGAIAMTQSATLRIGGTEIDIEKDRMWDEA
TYQAAMTPISLFQDVYENRPDQHEELEERYGAGVVERAMEVGL

>1JKXA

MNIVVLISGNGSNLQAIIDACKTNKIKGTVRAVFSNKADAFGLERARQAGIATHTLIASAFDSRE
AYDRELIHEIDMYAPDVVVLAGFMRILSPAFVSHYAGRLLNIHPSLLPKYPGLHTHRQALENGDE
EHGTSVHFVTDELDGGPVILQAKVPVFAGDSEDDITARVQTQEHAIYPLVISWFADGRLKMHENA
AWLDGQRLPPQGYAADE

>2PSPA

EKPAACRCSRQDPKNRVNCGFPGITSDQCFTSGCCFDSQVPGVPWCFKPLPAQESEECVMQVSAR
KNCGYPGISPEDCAARNCCFSDTIPEVPWCFFPMSVEDCHY

>3FGRB

CSALIKLLPGGHDLLVAHNTWNSYQNMLRIIKKYRLQFREGPQEEYPLVAGNNLVFSSYPGTIFS
GDDFYILGSGLVTLETTIGNKNPALWKYVQPQGCVLEWIRNVVANRLALDGATWADVFKRFNSGT
YNNQWMIVDYKAFLPNGPSPGSRVLTILEQIPGMVVVADKTAELYKTTYWASYNIPYFETVFNAS
GLQALVAQYGDWFSYTKNPRAKIFQRDQSLVEDMDAMVRLMRYNDFLHDPLSLCEACNPKPNAEN
AISARSDLNPANGSYPFQALHQRAHGGIDVKVTSFTLAKYMSMLAASGPTWDQCPPFQWSKSPFH
SMLHMGQPDLWMFSPIRVPWDGRGSHHHHHHG

>3EKIA

MLKKLKNFILFSSIFSPIAFAISCSNTGVVKQEDVSVSQGQWDKSITFGVSEAWLNKKKGGEKVN
KEVINTFLENFKKEFNKLKNANDKTKNFDDVDFKVTPIQDFTVLLNNLSTDNPELDFGINASGKL
VEFLKNNPGIITPALETTTNSFVFDKEKDKFYVDGTDSDPLVKIAKEINKIFVETPYASWTDENH
KWNGNVYQSVYDPTVQANFYRGMIWIKGNDETLAKIKKAWNDKDWNTFRNFGILHGKDNSSSKFK
LEETILKNHFQNKFTTLNEDRSAHPNAYKQKSADTLGTLDDFHIAFSEEGSFAWTHNKSATKPFE
TKANEKMEALIVTNPIPYDVGVFRKSVNQLEQNLIVQTFINLAKNKQDTYGPLLGYNGYKKIDNF
QKEIVEVYEKAIK

>1XS0A

QDDLTISSLAKGETTKAAFNQMVQGHKLPAWVMKGGTYTPAQTVTLGDETYQVMSACKPHDCGSQ

RIAVMWSEKSNQMTGLFSTIDEKTSQEKLTWLNVNDALSIDGKTVLFAALTGSLENHPDGFNFRS
HHHHHH
>2YWIA
GHMEERVLGMPAVESNMFPLGKQAPPFALTNVIDGNVVRLEDVKSDAATVIMFICNHCPFVKHVQ
HELVRLANDYMPKGVSFVAINSNDAEQYPEDSPENMKKVAEELGYPFPYLYDETQEVAKAYDAAC
TPDFYIFDRDLKCVYRGQLDDSRPNNGIPVTGESIRAALDALLEGRPVPEKQKPSIGCSIKWKPS
A
>3O3MB
MEAILSKMKEVVENPNAAVKKYKSETGKKAIGCFPVYCPEEIIHAAGMLPVGIWGGQTELDLAKQ
YFPAFACSIMQSCLEYGLKGAYDELSGVIIPGMCDTLICLGQNWKSAVPHIKYISLVHPQNRKLE
AGVKYLISEYKGVKRELEEICGYEIEEAKIHESIEVYNEHRKTMRDFVEVAYKHSNTIKPSIRSL
VIKSGFFMRKEEHTELVKDLIAKLNAMPEEVCSGKKVLLTGILADSKDILDILEDNNISVVADDL
AQETRQFRTDVPAGDDALERLARQWSNIEGCSLAYDPKKKRGSLIVDEVKKKDIDGVIFCMMKFC
DPEEYDYPLVRKDIEDSGIPTLYVEIDQQTQNNEQARTRIQTFAEMMSLASAWSHPQFEK
>3I4ZA
GSHGGSMKAANASSAEAYRVLSRAFRFDNEDQKLWWHSTAPMFAKMLETANYTTPCQYQYLITYK
ECVIPSLGCYPTNSAPRWLSILTRYGTPFELSLNCSNSIVRYTFEPINQHTGTDKDPFNTHAIWE
SLQHLLPLEKSIDLEWFRHFKHDLTLNSEESAFLAHNDRLVGGTIRTQNKLALDLKDGRFALKTY
IYPALKAVVTGKTIHELVFGSVRRLAVREPRILPPLNMLEEYIRSRGSKSTASPRLVSCDLTSPA
KSRIKIYLLEQMVSLEAMEDLWTLGGRRRDASTLEGLSLVRELWDLIQLSPGLKSYPAPYLPLGV
IPDERLPLMANFTLHQNDPVPEPQVYFTTFGMNDMAVADALTTFFERRGWSEMARTYETTLKSYY
PHADHDKLNYLHAYISFSYRDRTPYLSVYLQSFETGDWAVANLSESKVKCQDAACQPTALPPDLS
KTGVYYSGLH
>2B06A
MSRSQLTILTNICLIEDLETQRVVMQYRAPENNRWSGYAFPGGHVENDEAFAESVIREIYEETGL
TIQNPQLVGIKNWPLDTGGRYIVICYKATEFSGTLQSSEEGEVSWVQKDQIPNLNLAYDMLPLME
MMEAPDKSEFFYPRRTEDDWEKKIF
>3M0ZA
SNAMKLTPNFYRDRVCLNVLAGSKDNAREIYDAAEGHVLVGVLSKNYPDVASAVVDMRDYAKLID
NALSVGLGAGDPNQSAMVSEISRQVQPQHVNQVFTGVATSRALLGQNETVVNGLVSPTGTPGMVK
ISTGPLSSGAADGIVPLETAIALLKDMGGSSIKYFPMGGLKHRAEFEAVAKACAAHDFWLEPTGG
IDLENYSEILKIALDAGVSKIIPHIYSSIIDKASGNTRPADVRQLLEMTKQLVK
>1VH4A
SLMAGLPNSSNALQQWHHLFEAEGTKRSPQAQQHLQQLLRTGLPTRKHENWKYTPLEGLINSQFV
SIAGEISPQQRDALALTLDSVRLVFVDGRYVPALSDATEGSGYEVSINDDRQGLPDAIQAEVFLH
LTESLAQSVTHIAVKRGQRPAKPLLLMHITQGVAGEEVNTAHYRHHLDLAEGAEATVIEHFVSLN
DARHFTGARFTINVAANAHLQHIKLAFENPLSHHFAHNDLLLAEDATAFSHSFLLGGAVLRHNTS
TQLNGENSTLRINSLAMPVKNEVCDTRTWLEHNKGFCNSRQLHKTIVSDKGRAVFNGLINVAQHA
IKTDGQMTNNNLLMGKLAEVDTKPQLEIYADDVKCSHGATVGRIDDEQIFYLRSRGINQQDAQQM
IIYAFAAELTEALRDEGLKQQVLARIGQRLPGGAREGGSHHHHHH
>4HD5A
MRKYAAIALCTSAILAGCNTSNVSQEPKKEKKVQEVAIQKEALQEQGKISYTPITHESTNTSIHI
TDLKDSLNEVQYKIWRTADGKERAKSFSSKEKEKQFTIPFDIKEFEGKRGEFQIEATGMKEDGKT
IPLTKSIITFEQKVPVLMYHAIDDYHGQGIKDLFVSPANFEAQMKHLKDNGYTLLTFERWGDINK

VNKPIFVTFDDGMKNNMNAFRVLQKLKDDTFKPAATEYMIVDNVDVEGALSTSEIKEMVDSGIFS
VQSHTATHADLPKITNYEEELKGSKEKLEKITGKPVIAIAYXFGHVDDKVVTETKKYYQFATTTK
PGQFITKGEPDELLKMKRVRIHHTTTVEQFASSIK
>3MVUA
GMSEPYGKAFSLMRAEAEPAWRAYTHHAFVEGLKAGTLPREAFLHYLQQDYVFLIHFSRAWALAV
VKSETHSEMLAAVGTVNALVAEEMQLHIGICEASGISQEALFATRERAENLAYTRFVLEAGYSGD
LLDLLAALAPCVMGYGEIGKRLTAEATSTLYGDWIDTYGGDDYQAACKAVGTLLDDALERRLGAE
FTSSPRWSRLCQTFHTATELEVGFWQMGLTP
>3RC9A
MGSSHHHHHHENLYFQGHMENPANANPIRVGVIGCADIAWRRALPALEAEPLTEVTAIASRRWDR
AKRFTERFGGEPVEGYPALLERDDVDAVYVPLPAVLHAEWIDRALRAGKHVLAEAPLTTDRPQAE
RLFAVARERGLLLMENFMFLHHPQHRQVADMLDEGVIGEIRSFAASFTIPPKPQGDIRYQADVGG
GALLDIGVYPIRAAGLFLGADLEFVGAVLRHERDRDVVVGGNALLTTRQGVTAQLTFGMEHAYTN
NYEFRGSTGRLWMNRVFTPPATYQPVVHIERQDHAEQFVLPAHDQFAKSIRAFAQAVLSGEHPRE
WSEDSLRQASLVDAVRTGARDIYFP
>3RM3A
MGSSHHHHHHSSGLVPRGSHMSEQYPVLSGAEPFYAENGPVGVLLVHGFTGTPHSMRPLAEAYAK
AGYTVCLPRLKGHGTHYEDMERTTFHDWVASVEEGYGWLKQRCQTIFVTGLSMGGTLTLYLAEHH
PDICGIVPINAAVDIPAIAAGMTGGGELPRYLDSIGSDLKNPDVKELAYEKTPTASLLQLARLMA
QTKAKLDRIVCPALIFVSDEDHVVPPGNADIIFQGISSTEKEIVRLRNSYHVATLDYDQPMIIER
SLEFFAKHAG
>1VZMA
AAKELTLAQTESLREVCETNMACDEMADAQGIVAAYQAFYGPIPF
>2NMMA
MGHHHHHHSHMAVADLALIPDVDIDSDGVFKYVLIRVHSAPRSGAPAAESKEIVRGYKWAEYHAD
IYDKVSGDMQKQGCDCECLGGGRISHQSQDKKIHVYGYSMAYGPAQHAISTEKIKAKYPDYEVTW
ANDGY
>2DDRA
EVSTTQNDTLKVMTHNVYMLSTNLYPNWGQTERADLIGAADYIKNQDVVILNEVFDNSASDRLLG
NLKKEYPNQTAVLGRSSGSEWDKTLGNYSSSTPEDGGVAIVSKWPIAEKIQYVFAKGCGPDNLSN
KGFVYTKIKKNDRFVHVIGTHLQAEDSMCGKTSPASVRTNQLKEIQDFIKNKNIPNNEYVLIGGD
MNVNKINAENNNDSEYASMFKTLNASVPSYTGHTATWDATTNSIAKYNFPDSPAEYLDYIIASKD
HANPSYIENKVLQPKSPQWTVTSWFQKYTYNDYSDHYPVEATISMK
>3RFRA
MKKLVKLAAFGAAAAVAATLGAIAPASAHGEKSQQAFLRMRTLNWYDVQWSKTTVNVNEEMILSG
KVHVFSAWPQAVANPRVSFLNAGEPGPVLVRTAQFIGEQFAPRSVSLEIGKDYAFSINLRGRRAG
RWHVHAQINVEGGGPIIGPGQWIEIKGDMKDFTDPVTLLDGSTVDLENYGISRIYAWHLPWLAVG
AAWILFWFIRKGIIASYVRVAEGRPDDVIGDDDRRIGAIVLALTILATIVGYAVTNSTFPRTIPL
QAGLQKPLTPIETEGTVGVGKEQVTTELNGGVYKVPGRELTINVKVKNGTSQPVRLGEYTAAGLR
FLNPTVFTQKPDFPDYLLADRGLSNDDVIAPGESKEIVVKIQDARWDIERLSDLAYDTDSQVGGL
LFFFTPDGKRFAAEIGGPVIPKFVAGDMP
>1H8PA
DQDEGVSTEPTQDGPAELPEDEECVFPFVYRNRKHFDCTVHGSLFPWCSLDADYVGRWKYCAQRD
YAKCVFPFIYGGKKYETCTKIGSMWMSWCSLSPNYDKDRAWKYC

>3N91A
GSDNEFPDFDYQTVYFANQYGLRTIELGESEFVDNTLDNQHKMVIKAAWGGGYTNRNNVVINFKV
DESLCDNLYFKDTDQPLVPMPASYYTLASDRIAIPKGQIMAGVEVQLTDDFFADEKSISENYVIP
LLMTNVQGADSILQGKPVVENPVLTNAGDWSILPQNFVLYAVKYVNPWHGEYLRRGIDHATVAGT
SKDIIRHEQFVENDEVVNISTKSMKDNLLTLKTKDESGKDISYTVRLSFAEDGSCTVHSGSQNVV
VSGSGKFVSKGEKNSLGGKDRNAIYLDYTVNLTDNNIQLATKDTLVLRTRNVYGGKSLEVVRK
>2PKFA
GTEDLYFQSHMTIAVTGSIATDHLMRFPGRFSEQLLPEHLHKVSLSFLVDDLVMHRGGVAGNMAF
AIGVLGGEVALVGAAGADFADYRDWLKARGVNCDHVLISETAHTARFTCTTDVDMAQIASFYPGA
MSEARNIKLADVVSAIGKPELVIIGANDPEAMFLHTEECRKLGLAFAADPSQQLARLSGEEIRRL
VNGAAYLFTNDYEWDLLLSKTGWSEADVMAQIDLRVTTLGPKGVDLVEPDGTTIHVGVVPETSQT
DPTGVGDAFRAGFLTGRSAGLGLERSAQLGSLVAVLVLESTGTQEWQWDYEAAASRLAGAYGEHA
AAEIVAVLA
>4G2SA
GSSPGPYIVRLLNSSLNGCEFPLLTGRTLFVVGQSDALTASGQLPDIPADSFFIPLDHGGVNFEI
QVDTDATEIILHELKEGNSESRSVQLNTPIQVGELLILIRPESEPW
>1T8SA
MNNKGSGLTPAQALDKLDALYEQSVVALRNAIGNYITSGELPDENARKQGLFVYPSLTVTWDGST
TNPPKTRAFGRFTHAGSYTTTITRPTLFRSYLNEQLTLLYQDYGAHISVQPSQHEIPYPYVIDGS
ELTLDRSMSAGLTRYFPTTELAQIGDETADGIYHPTEFSPLSHFDARRVDFSLARLRHYTGTPVE
HFQPFVLFTNYTRYVDEFVRWGCSQILDPDSPYIALSCAGGNWITAETEAPEEAISDLAWKKHQM
PAWHLITADGQGITLVNIGVGPSNAKTICDHLAVLRPDVWLMIGHCGGLRESQAIGDYVLAHAYL
RDDHVLDAVLPPDIPIPSIAEVQRALYDATKLVSGRPGEEVKQRLRTGTVVTTDDRNWELRYSAS
ALRFNLSRAVAIDMESATIAAQGYRFRVPYGTLLCVSDKPLHGEIKLPGQANRFYEGAISEHLQI
GIRAIDLLRAEGDRLHSRKLRTFNEPPFR
>2XD7A
DGPGDGFTILSSKSLVLGQKLSLTQSDISHIGSMRVEGIVHPTTAEIDLKEDIGKALEKAGGKEF
LETVKELRKSQGPLEVAEAAVSQSSGLAAKFVIHCHIPQWGSDKCEEQLEETIKNCLSAAEDKKL
KSVAFPPFPSGRNCFPKQTAAQVTLKAISAHFDDSSASSLKNVYFLLFDSESIGIYVQEMAKL
>3PE9A
MVKLTAPKSNVVAYGNEFLKITATASDSDGKISRVDFLVDGEVIGSDREAPYEYEWKAVEGNHEI
SVIAYDDDDAASTPDSVKIFVKQARLEHHHHHH
>2RKNA
AIDLCGMSQDELNECKPAVSKENPTSPSQPCCTALQHADFACLCGYKNSPWLGSFGVDPELASAL
PKQCGLANAPTC
>1JNIA
DAPAVGKDLTQAAENIPPAFHNAPRQGELPALNYVNQPPMVPHSVANYQVTKNVNQCLNCHSPEN
SRLSGATRISPTHFMDRDGKVGSSSSPRRYFCLQCHVSQANVDPIVPNDFKPMKGYGN
>1T61A
SVDHGFLVTRHSQTTDDPQCPPGTKILYHGYSLLYVQGNERAHGQDLGTAGSCLRKFSTMPFLFC
NINNVCNFASRNDYSYWLSTPEPMPMSMAPITGENIRPFISRCAVCEAPAMVMAVHSQTIQIPQC
PTGWSSLWIGYSFVMHTSAGAEGSGQALASPGSCLEEFRSAPFIECHGRGTCNYYANAYSFWLAT
IERSEMFKKPTPSTLKAGELRTHVSRCQVCMRRT
>4J32A

MGHHHHHHLYFQGMNRPSFNEAWLAFRKVNHSVADVGSIIGGNVGKNITGGYFQNACPIRMSYVL
NATGFPIARNSPYAKVSGADNKFYIYRVNDMIDYLTHTMGKPDLIVNNPKQSDFIGKKGIIVVKG
HGWSNARGHVTLWNGSICSDQCHLLNDPDNGPFVPEVGTLWILP
>3U52C
MSIEIKTNSVEPIRHTYGHIARRFGDKPATRYQEASYDIEAKTNFHYRPQWDSEHTLNDPTRTAI
RMEDWCAVSDPRQFYYGAYVGNRAKMQESAETSFGFCEKRNLLTRLSEETQKQLLRLLVPLRHVE
LGANMNNAKIAGDATATTVSQMHIYTGMDRLGIGQYLSRIALMIDGSTGAALDESKAYWMDDEMW
QPMRKLVEDTLVVDDWFELTLVQNILIDGMMYPLVYDKMDQWFESQGAEDVSMLTEFMRDWYKES
LRWTNAMMKAVAGESETNRELLQKWIDHWEPQAYEALKPLAEASVGIDGLNEARAELSARLKKFE
LQSRGVSA
>1WC2A
NQKCSGNPRRYNGKSCASTTNYHDSHKGACGCGPASGDAQFGWNAGSFVAAASQMYFDSGNKGWC
GQHCGQCIKLTTTGGYVPGQGGPVREGLSKTFMITNLCPNIYPNQDWCNQGSQYGGHNKYGYELH
LDLENGRSQVTGMGWNNPETTWEVVNCDSEHNHDHRTPSNSMYGQCQCAHQ
>2CW9A
GSSGSSGDESDNAFIRASRALTDKVTDLLGGLFSKTEMSEVLTEILRVDPAFDKDRFLKQCENDI
IPNVLEAMISGELDILKDWCYEATYSQLAHPIQQAKALGLQFHSRILDIDNVDLAMGKMVEQGPV
LIITFQAQLVMVVRNPKGEVVEGDPDKVLRMLYVWALCRDQDELNPYAAWRLLDISASSTEQIL
>1OFLA
EVVASNETLYQVVKEVKPGGLVQIADGTYKDVQLIVSNSGKSGLPITIKALNPGKVFFTGDAKVE
LRGEHLILEGIWFKDGNRAIQAWKSHGPGLVAIYGSYNRITACVFDCFDEANSAYITTSLTEDGK
VPQHCRIDHCSFTDKITFDQVINLNNTARAIKDGSVGGPAMYHRVDHCFFSNPQKPGNAGGGIRI
GYYRNDIGRCLVDSNLFMRQDSEAEIITSKSQENVYYGNTYLNCQGTMNFRHGDHQVAINNFYIG
NDQRFGYGGMFVWGSRHVIACNYFELSETIKSRGNAALYLNPGAMASEHALAFDMLIANNAFINV
NGYAIHFNPLDERRKEYCAANRLKFETPHQLMLKGNLFFKDKPYVYPFFKDDYFIAGKNSWTGNV
ALGVEKGIPVNISANRSAYKPVKIKDIQPIEGIALDLNALISKGITGKPLSWDEVRPYWLKEMPG
TYALTARLSADRAAKFKAVIKRNKEH
>2OAFA
GMQGAGRLMQPRPDSAFVHDVRVTWGDCDPAKIAYTGHLPRFALEAIDAWWSEYHGPGGWYHLEL
DTNVGTPFVRLEMDFKSPVTPRHILKCHTWPTRLGTKSITFRVDGVQDGVTCFVGAFTCVFTIAD
QFKSQPAPDHLRALIEPHIPA
>3ML3A
MGSSHHHHHHSSGLVPRGSHMSGTVLINNINAPFLPDPVIVTGNMTLEKNGHVILNNSSSNVGQT
YVQKGNWHGKGGILSLGAVLGNDNSKTDRLEIAGHASGITYVAVTNEGGSGDKTLEGVQIISTDS
SDKNAFIQKGRIVAGSYDYRLKQGTASGLNTNKWYLTSQMDNQESKQMSNQESTQMSSR
>3IFNP
DAEFRHDSGYEVHHQKLVFFAEDVGSNKGAIIGLMVGGVV
>3IT3A
MVGYSSKLIFVSMITRHGDRAPFANIENANYSWGTELSELTPIGMNQEYNLGLQLRKRYIDKFGL
LPEHYVDQSIYVLSSHTNRTVVSAQSLLMGLYPAGTGPLIGDGDPAIKDRFQPIPIMTLSADSRL
IQFPYEQYLAVLKKYVYNSPEWQNKTKEAAPNFAKWQQILGNRISGLNDVITVGDVLIVAQAHGK
PLPKGLSQEDADQIIALTDWGLAQQFKSQKVSYIMGGKLTNRMIEDLNNAVNGKSKYKMTYYSGH
ALTLLEVMGTLGVPLDTAPGYASNLEMELYKDGDIYTVKLRYNGKYVKLPIMDKNNSCSLDALNK
YMQSINEKFQKHHHHHH

>1PYAA
SELDAKLNKLGVDRIAISPYKQWTRGYMEPGNIGNGYVTGLKVDAGVRDKSDDDVLDGIVSYDRA
ETKNAYIGQINMTTAS
>1G3KA
TTIVSVRRNGQVVVGGDGQVSLGNTVMKGNARKVRRLYNGKVLAGFAGGTADAFTLFELFERKLE
MHQGHLLKSAVELAKDWRTDRALRKLEAMLIVADEKESLIITGIGDVVQPEEDQILAIGSGGNYA
LSAARALVENTELSAHEIVEKSLRIAGDICVFTNTNFTIEELPN
>3OR1A
MAKHPTPMLDELEKGPWPSFVSDIKQECDNRAKNPKGLDYQIPAECPDDLLGILELSFHEGETHW
KHGGIVGVFGYGGGVIGRYCDQPEMFPGVAHFHTVRLAQPAAKYYTAEYLEAICDVWDLRGSGLT
NMHGSTGDIVLLGTQTPQLEEIFFEMTHNLNTDLGGSGSNLRTPESCLGISRCEFACYDTQLMCY
QLTQDYQDELHRPAFPYKFKFKFDGCPNGCVASMARSDFAVIGTWKDDIKIDQEAVKAYVGGEFK
PNAGAHAGRDWGKFDIEAEVVGLCPTGCMTYESGTLSIDNKNCTRCMHCINTMPRALKIGDERGA
SILVGAKAPVLDGAQMGSLLIPFIAAEEPFDEVKEVIENIWEWWMEEGKNRERLGETMKRVGFQK
LLEVTGTKAVPQHVSEPRHNPYIFFKEEEVPGGWSRDISDYRKRHMR
>3AH7A
MPLVTFLPHEKFCPEGLTVEVKPGTNILELAHDHHIEMESACGGVKACTTCHCIVRKGFDSLEEA
DELEEDMLDKAWGLEAQSRLGCQVFVADEDLTIEIPKYSLNHAAEAPH
>3R5TA
QQNVWPRTFQNADGSITTIPSQPKRILSTAVTVTGTLLAIDAPVIASAATTQSTFFEQWRKLAEL
RQVKKLWPAGSVDLESVYVEQPDLIVVSMIGADSARDQIPLLQAIAPTILVDYSDQTWQSLAQQL
GLATGLEEQAERTIHNFEQWTKQVRDVLDLPKGRANIVSYHGPGVVNAVAKAQSAHAQLLQSVGV
VLEEPDPAWQAGSIVHRDFLRIHYEHLTQLQAETTFLITMTDQQAQAFLHDPILKNLPSIQRKQV
YGLGENSFRIDLFSAREIINSLLRRFAGEQAQSLVMPLEHHHHHH
>2RJ2A
GSHMFYFLSKRRRNLLRNPCGEEDLEGWSDVEHGGDGWKVEELPGDGNVEFTQDDSVKKYFASSF
EWCRKAQVIDLQAEGYWEELLDTTQPAIVVKDWYSGRTDAGSLYELTVRLLSENEDVLAEFATGQ
VAVPEDGSWMEISHTFIDYGPGVRFVRFEHGGQDSVYWKGWFGARVTNSSVWVEP
>4IGBB
PTTENLYFQGAMALEEIKNGTDISTLDIRKFNLNINNVSVLSKSQSVDQFHLSNPHYEYLSGGAY
PGEMENFTLKVDKSKKQDQVFENPLSLKFTNIGTVNGKQVDAYLNFNKVTLHYLNTAQAESEMNS
AQKSTVEFFSISELWESNAFEIGNVPYVDANHDYIMNKAFWIDADVTAEIRYADGTETDLKLVMK
PTDIDAIDANNLKETFYVKNYQNDVNLRLMNNANVLVQEEASDRTSWIATQITGGSYNENNVSGL
ALRSNSNSMNFGYSSTETCSAVFGLYIEKIDPRPVLEVDPAEIPAKDGQDVTYKATFKVPVPGKD
ILAAPSSIEMVQKFDERLDYKELKVESGGVTLQEGRDYTIEKTGQTVTVKMTPEYLKGNSSSDII
ITYKTATNKKVEEKGSEKIDNTVTLHVDNLSAPSNQVSTALLYEK
>3TJ1A
MGSSHHHHHHSSGLVPRGSHMAMMAFENTSKRPPQDFVAPIDQKKRKVQFSDSTGLVTLQPEEIK
DEVFSAAMYSRFVKSALDDLDKNDSTQIGIIANQVALPSKNPERINDKNLNILLDILSSNINRIE
SSRGTFLIQSIINFEKWWELPPHTLSKYIYFIKILCSSIPKWWQDVSMILVSCFILPIKQTVCHH
DMLKYFLRMIPSSMGFIDTYLAKFFPNKNDTRRKLVNYTSNLLKLRGYCSELGFQIWSLLIEKII
SIDVELQNELDELDDDVDDDDLEEVDLEDDDDLDDDSGDDDDENCGNSNEELRSGAADGSQSDSE
DMDIIEGMDGTEEYNVELTQGIKELSTKLDSILTLVSTHVEEQVTPESLESGEGVGVFNTLTTLF
KTHVLPTYYTRSIQYIMFHVSQQQLELMDSFLVTLIDISFAVNEAAEKKIKSLQYLGSYIARAKK

LSRTQIIFVASYLTSWLNRYVIEREEEVDQRGGMERFKHFYAAFQALCYIFCFRHNIFRDTDGNW
ECELDKFFQRMVISKFNPLKFCNENVMLMFARIAQQESVAYCFSIIENNNNERLRGIIGKADSDK
KENSAQANTTSSSWSLATRQQFIDLQSYFPYDPLFLKNYKILMKEYYIEWSEASGEYESDGSDD
>2BKFA
GAMEPQVTLNVTFKNEIQSFLVSDPENTTWADIEAMVKVSFDLNTIQIKYLDEENEEVSINSQGE
YEEALKMAVKQGNQLQMQVHEG
>4G6VA
MGATDRTPPSNAILSNSNSDNNSTQGSQSGTVTKTPNPEATGSLSGKPTQIPPLSDEVTTRSLIR
ENQSAVTLANKGYDVVQNPEVLGPKNPDYTINGQVFDNYAPATGNVRNIATTISNKVSSGQASNI
VVNLADSSASPAAIEAQINSYPIPGLGKVIVIDKLGNITIIKPKGN
>3BEMA
MGSDKIHHHHHHMAEFTHLVNERRSASNFLSGHPITKEDLNEMFELVALAPSAFNLQHTKYVTVL
DQDVKEKLKQAANGQYKVVSSSAVLLVLGDKQAYQQAADIYEGLKVLGILNKQEYDHMVQDTVSF
YENRGEQFKRDEAIRNASLSAMMFMLSAAAAGWDTCPMIGFDAEAVKRILNIDDQFEVVMMITIG
KEKTESRRPRGYRKPVNEFVEYM
>3FD3A
RVTLNIATNADSLGTWFLDAVSKFTGGSDYLVNIAVDDQDHTVEWLRGGRVLAAVTAHDKPVQGC
RVTPLGVLRYHATASPDFMARHFADGVTPAALARAPGLTFNQKDRLQASWIRTALGEDVSYPTHW
LPSTDGFVKASLAGMGWGLNPVQLVAEHLAAGRLVELMPGTPLDIPLYWQVNRLAAERLAGLTAN
MVGTARVVLMPVG
>1YNFA
MGSSHHHHHHGSNAWEVNFDGLVGLTHHYAGLSFGNEASTRHRFQVSNPRLAAKQGLLKMKALAD
AGFPQAVIPPHERPFIPVLRQLGFSGSDEQVLEKVARQAPHWLSSVSSASPMWVANAATIAPSAD
TLDGKVHLTVANLNNKFHRSLEAPVTESLLKAIFNDEEKFSVHSALPQVALLGDEGAANHNRLGG
HYGEPGMQLFVYGREEGNDTRPSRYPARQTREASEAVARLNQVNPQQVIFAQQNPDVIDQGVFHN
DVIAVSNRQVLFCHQQAFARQSQLLANLRARVNGFMAIEVPATQVSVSDTVSTYLFNSQLLSRDD
GSMMLVLPQECREHAGVWGYLNELLAADNPISELKVFDLRESMANGGGPACLRLRVVLTEEERRA
VNPAVMMNDTLFNALNDWVDRYYRDRLTAADLADPQLLREGREALDVLSQLLNLGSVYPFQREGG
GNG
>1O70A
AAAADTTVTQFLQSFKENAENGALRKFYEVIMDNGGAVLDDINSLTEVTILAPSNEAWNSSNINN
VLRDRNKMRQILNMHIIKDRLNVDKIRQKNANLIAQVPTVNNNTFLYFNVRGEGSDTVITVEGGG
VNATVIQADVAQTNGYVHIIDHVLGVPYTTVLGKLESDPMMSDTYKMGKFSHFNDQLNNTQRRFT
YFVPRDKGWQKTELDYPSAHKKLFMADFSYHSKSILERHLAISDKEYTMKDLVKFSQESGSVILP
TFRDSLSIRVEEEAGRYVIIWNYKKINVYRPDVECTNGIIHVIDYPLLEEKDVVVAGGSAAAAA
>4IAUA
MSTAKVTLVTSGGSSQDFTSEQTNITTDFARVRVTKGMWIFYQQANYNDASGGGSLWIKLDESSH
LMDLPFTPRSFRPVKTFQVGATLYKHVNFGGKELDLPNSNPRIDIGGVSSALISQGQWRLYEQYD
YAGPSTRRGPGVYVNAGALGVANDALKSMEREF
>3A2ZA
MSKGTTSQDAPFGTLLGYAPGGVAIYSSDYSSLDPQEYEDDAVFRSYIDDEYMGHKWQCVEFARR
FLFLNYGVVFTDVGMAWEIFSLRFLREVVNDNILPLQAFPNGSPRAPVAGALLIWDKGGEFKDTG
HVAIITQLHGNKVRIAEQNVIHSPLPQGQQWTRELEMVVENGCYTLKDTFDDTTILGWMIQTEDT
EY

>3V7NA
GPGSMNYISTRGAGIGERHTFSDILLGGLAKDGGLYLPSEYPQVSADELARWRTLPYADLAFEIL
SKFCDDIAAADLRAITRRTYTADVYRHARRGGNAADITPLTTLGTENGAPVSLLELSNGPTLAFK
DMAMQLLGNLFEYTLAKHGETLNILGATSGDTGSAAEYAMRGKEGVRVFMLSPHKKMSAFQTAQM
YSLQDPNIFNLAVNGVFDDCQDIVKAVSNDHAFKAQQKIGTVNSINWARVVAQVVYYFKGYFAAT
RSNDERVSFTVPSGNFGNVCAGHIARMMGLPIEKLVVATNENDVLDEFFRTGAYRVRSAQDTYHT
SSPSMDISKASNFERFVFDLLGRDPARVVQLFRDVEQKGGFDLAASGDFARVAEFGFVSGRSTHA
DRIATIRDVFERYRTMIDTHTADGLKVAREHLRPGVPMVVLETAQPIKFGESIREALGQEPSRPA
AFDGLEALPQRFEVVDANAQQVKDFIAAHTGA
>1OSYA
XSATSLTFQLAYLVKKIDFDYTPNWGRGTPSSYIDNLTFPKVLTDKKYSYRVVVNGSDLGVESNF
AVTPSGGQTINFLQYNKGYGVADTKTIQVFVVIPDTGNSEEYIIAEWKKT
>3LYEA
GSHMAEDEPFSGAKKLRHLLENTDELIVCPGVYDGLSARTAMELGFKSLYMTGAGTTASRLGQPD
LAIAQLHDMRDNADMIANLDPFGPPLIADMDTGYGGPIMVARTVEHYIRSGVAGAHLEDQILTKR
CGHLSGKKVVSRDEYLVRIRAAVATKRRLRSDFVLIARTDALQSLGYEECIERLRAARDEGADVG
LLEGFRSKEQAAAAVAALAPWPLLLNSVENGHSPLITVEEAKAMGFRIMIFSFATLAPAYAAIRE
TLVRLRDHGVVGTPDGITPVRLFEVCGLQDAMEVDNGAGGKAFSEGV
>4JJPA
SNAMSNYKIPTLTIAGSDSSGGAGIQADLKTFSAIGTYGMSVITAITAQNTKGVFAVEDLNKKII
KKQIEAVFEDIPPRAVKIGMVSSPEIILEIVENLKKYNPKYLVVDPVMISKSGYYLLKPEAKENL
IKYLIPLAYIITPNIPEAEEITGIKIHNVDDMKRVGEEILQLGPKFVLMKGGHLDGEAVDILVGK
NIFKVYKSERIDKKNTHGTGCTLSSAITSYLALGYEITEAVNLSKIYITEAIKRSFDIGHGVGPV
HHFYKFE
>2ODAA
MPLPTFPALLFGLSGCLVDFGAQAATSDTPDDEHAQLTPGAQNALKALRDQGMPCAWIDELPEAL
STPLAAPVNDWMIAAPRPTAGWPQPDACWMALMALNVSQLEGCVLISGDPRLLQSGLNAGLWTIG
LASCGPLCGLSPSQWQALNNAEREQRRAQATLKLYSLGVHSVIDHLGELESCLADIALRRSKGEK
P
>3MT5A
MGRKHIVVCGHITLESVSNFLKDFLHKDRDDVNVEIVFLHNISPNLELEALFKRHFTQVEFYQGS
VLNPHDLARVKIESADACLILANKYCADPDAEDASNIMRVISIKNYHPKIRIITQMLQYHNKAHL
LNIPSWNWKEGDDAICLAELKLGFIAQSCLAQGLSTMLANLFSMRSFIKIEEDTWQKYYLEGVSN
EMYTEYLSSAFVGLSFPTVCELCFVKLKLLMIAIEYKSANRESRILINPGNHLKIQEGTLGFFIA
SDAKEVKRAFFYCKACHDDITDPKRIKKCGCKRLEDEQPSTLSPKKKQRNGGMRNSPNTSPKLMR
HDPLLIPGNDQIDNMDSNVKKYDSTGMFHWCAPKEIEKVILTRSEAAMTVLSGHVVVCIFGDVSS
ALIGLRNLVMPLRASNFHYHELKHIVFVGSIEYLKREWETLHNFPKVSILPGTPLSRADLRAVNI
NLCDMCVILSANQNNIDDTSLQDKECILASLNIKSMQFDDSIGVLQANSQGFTPPGMDRSSPDNS
PVHGMLRQPSITTGVNIPIITELVNDTNVQFLDQDDDDDPDTELYLTQPFACGTAFAVSVLDSLM
SATYFNDNILTLIRTLVTGGATPELEALIAEENALRGGYSTPQTLANRDRCRVAQLALLDGPFAD
LGDGGCYGDLFCKALKTYNMLCFGIYRLRDAHLSTPSQCTKRYVITNPPYEFELVPTDLIFCLMQ
FDSNSLEVLFQ
>3ER7A
GMMNTTTLDRYFDLFDASRTDEKAFDDLISLFSDEITFVLNGQEQHGIDAWKQFVRMVFTANQDI

KHMYAGWVPSETGDTMETRWAVCGKSADGSVFTQDGTDIARLNADGKIVYLANVPDDTAMFNQYN
D
>1V2BA
GKPKTDTDFQTYNGDGFKLQIPSKWNPNKEVEYPGQVLRFEDNFDATSNVIVAITPTDKKSITDF
GSPEQFLSQVDYLLGRQAYSGKTDSEGGFESDAVAIANVLETSTAEVGGKQYYYLSILTRTADGN
EGGKHQLVTATVNDGKLYICKAQAGDKRWFKGAKKFVENTATSFSLA
>3HL1A
GMPPVWTLPRLYQHFQGAIDLELWTIPYYLTVLYSIKDPTTVPYRLIQAAVYQEMLHAQLVSNIA
NAYGYSPTLSAPEYVGTAVPHIDFDLDTPNPTSIFTPYSAELGPLDLTRVNTMCLIEYPEWRTQR
EPDLADDVTDYGSIGEFYDALRVGMEQLRGHVRGNQKQMDEFGPFYQNSPPLTVTESGDAGFLQA
LTLVDIIVDQGEGQTQPVETIPTEFQNTADGFQDAWPHFQRFDFIRRMPNWPGVYTGVTDPPAGS
PGAEAQARLIADFAGFLDILNGMFSGGGAPPAFGVQMAKLGGDILSCWKLGAVPRYS
>3QS2A
MAHHHHHHVDDDDKMADVTAQAVATWSATAKKDTTSKLVVTPLGSLAFQYAEGIKGFNSQKGLFD
VAIEGDSTATAFKLTSRLITNTLTQLDTSGSTLNVGVDYNGTAVEKTGDTVMIDTANGVLGGNLS
PLANGYNASNRTTAQDGFTFSIISGTTNGTTAVTDYSTLPEGIWSGDVSVQFDATWTS
>2PV2A
TELNLSHILIPLPENPTSDQVNEAESQARAIVDQARNGADFGKLAIAHSADQQALNGGQMGWGRI
QELPGIFAQALSTAKKGDIVGPIRSGVGFHILKVNDLR
>2WVXA
KDWTQYVNPLMGSQSTFELSTGNTYPAIARPWGMNFWTPQTGKMGDGWQYTYTANKIRGFKQTHQ
PSPWINDYGQFSIMPIVGQPVFDEEKRASWFAHKGEVATPYYYKVYLAEHDIVTEMTPTERAVLF
RFTFPENDHSYVVVDAFDKGSYIKIIPEENKIIGYTTRNSGGVPENFKNYFIIEFDKPFTYKATV
ENGNLQENVAEQTTDHAGAIIGFKTRKGEQVNARIASSFISFEQAAANMNELGKDNIEQLAQKGK
DAWNQVLGKIEVEGGNLDQYRTFYSCLYRSLLFPRKFYELDANGQPIHYSPYNGQVLPGYMFTDT
GFWDTFRCLFPLLNLMYPSVNKEMQEGLINTYLESGFFPEWASPGHRGCMVGNNSASILVDAYMK
GVKVDDIKTLYEGLIHGTENVHPEVSSTGRLGYEYYNKLGYVPYDVKINENAARTLEYAYDDWCI
YRLAKELKRPKKEISLFAKRAMNYKNLFDKESKLMRGRNEDGTFQSPFSPLKWGDAFTEGNSWHY
TWSVFHDPQGLIDLMGGKEMFVTMMDSVFAVPPIFDDSYYGQVIHEIREMTVMNMGNYAHGNQPI
QHMIYLYDYAGQPWKAQYWLRQVMDRMYTPGPDGYCGDEDNGQTSAWYVFSALGFYPVCPGTDEY
VMGTPLFKKATLHFENGNSLVIDAPNNSTENFYIDSMSFNGADHTKNYLRHEDLFKGGTIKVDMS
NRPNLNRGTKEEDMPYSFSKELEHHHHHH
>3D5KA
CSLIPDYQRPEAPVAAAYPQGQAYGQNTGAAAVPAADIGWREFFRDPQLQQLIGVALENNRDLRV
AALNVEAFRAQYRIQRADLFPRIGVDGSGTRQRLPGDLSTTGSPAISSQYGVTLGTTAWELDLFG
RLRSLRDQALEQYLATEQAQRSAQTTLVASVATAYLTLKADQAQLQLTKDTLGTYQKSFDLTQRS
YDVGVASALDLRQAQTAVEGARATLAQYTRLVAQDQNALVLLLGSGIPANLPQGLGLDQTLLTEV
PAGLPSDLLQRRPDILEAEHQLMAANASIGAARAAFFPSISLTANAGTMSRQLSGLFDAGSGSWL
FQPSINLPIFTAGSLRASLDYAKIQKDINVAQYEKAIQTAFQEVADGLAARGTFTEQLQAQRDLV
KASDEYYQLADKRYRTGVDNYLTLLDAQRSLFTAQQQLITDRLNQLTSEVNLYKALGGGWNQQTV
TQQQTAKKEDPQAHHHHHH
>2AJ7A
MGSDKIHHHHHHMKVIETKYSGKLEVAEDRLIAFDQGIPAFEDEKEFVLLPFAAGTPYYTLQSTK
TVDLAFIIVNPFSFFPEYRVKLPEATIAQLNITNENDVAIFSLLTVKEPFSETTVNLQAPIVINA

NKQMGKQLVLGDTAYNRKQPLFQKELVLAKEAK
>1LKTA
ANVVVSNPRPIFTESRSFKAVANGKIYIGQIDTDPVNPANQIPVYIENEDGSHVQITQPLIINAA
GKIVYNGQLVKIVTVQGHSMAIYDANGSQVDYIANVLKY
>1L6RA
GSHMIRLAAIDVDGNLTDRDRLISTKAIESIRSAEKKGLTVSLLSGNVIPVVYALKIFLGINGPV
FGENGGIMFDNDGSIKKFFSNEGTNKFLEEMSKRTSMRSILTNRWREASTGFDIDPEDVDYVRKE
AESRGFVIFYSGYSWHLMNRGEDKAFAVNKLKEMYSLEYDEILVIGDSNNDMPMFQLPVRKACPA
NATDNIKAVSDFVSDYSYGEEIGQIFKHFELM
>2JBYA
MGSHHHHHHSQDPMMSRLKTAVYDYLNDVDITECTEMDLLCQLSNCCDFINETYAKNYDTLYDIM
ERDILSYNIVNIKNTLTFALRDASPSVKLATLTLLASVIKKLNKIQHTDAAMFSEVIDGIVAEEQ
QVIGFIQKKCKYNTT
>4KSNA
GNSDGQLDTHLADLYLLKYDTGLGVYESFICKYLEDSNDYIASHPQKLSLDEMPRPLESETVSLR
QLIVSVLPSRPSI
>3BF7A
MKLNIRAQTAQNQHNNSPIVLVHGLFGSLDNLGVLARDLVNDHNIIQVDVRNHGLSPREPVMNYP
AMAQDLVDTLDALQIDKATFIGHSMGGKAVMALTALAPDRIDKLVAIDIAPVDYHVRRHDEIFAA
INAVSESDAQTRQQAAAIMRQHLNEEGVIQFLLKSFVDGEWRFNVPVLWDQYPHIVGWEKIPAWD
HPALFIPGGNSPYVSEQYRDDLLAQFPQARAHVIAGAGHWVHAEKPDAVLRAIRRYLNDH
>3HHSA
ADIFDSFELLYDRPGEPMINTKGEDKVLFELTEQFLTPEYANNGLELNNRFGDEEEVSRKIILKN
LDKIPEFPKAKQLPNDADFSLFLPSHQEMANEVIDVLMSVTENQLQELLSTCVYARINLNPQLFN
YCYTVAIMHRRDTGKVRVQNYAEIFPAKFLDSQVFTQAREAAAVIPKTIPRTPIIIPRDYTATDL
EEEHRLAYWREDLGINLHHWHWHLVYPFSASDEKIVAKDRRGELFFYMHQQIIARYNCERLCNSL
KRVKKFSDWREPIPEAYYPKLDSLTSARGWPPRQAGMRWQDLKRPVDGLNVTIDDMERYRRNIEE
AIATGNVILPDKSTKKLDIDMLGNMMEASVLSPNRDLYGSIHNNMHSFSAYMHDPEHRYLESFGV
IADEATTMRDPFFYRVHAWVDDIFQSFKEAPHNVRPYSRSQLENPGVQVTSVAVESAGGQQNVLN
TFWMQSDVNLSKGLDFSDRGPVYARFTHLNHRPFRYVIKANNTASARRTTVRIFIAPKTDERNLP
WALSDQRKMFIEMDRFVVPLSAGENTITRQSTESSLTIPFEQTFRDLSIQGSDPRRSELAAFNYC
GCGWPQHMLVPKGTVGGVAYQLFVMLSNYELDKIEQPDGRELSCVEASMFCGLKDKKYPDARPMG
YPFDRPSNSATNIEDFSAMSNMGLQDIVIKLSDVTEPNPRNPPA
>2AUWA
GHMNEYFFPKLTAVEALAPYRLRTTWSTGEVLEVDVGDILRKIPDLAPILDPEAFARVHIAEWEG
SVEWFDTEFGRDNVYAWAKEQAGEVSHEMFGDWMHRNNLSLTTAAEALGISRRMVSYYRTAHKII
PRTIWLACLGWEATRPETKTLPRTLPAAYAKGVSASLSGS
>2Z0BA
GSSGSSGPSQVAFEIRGTLLPGEVFAICGSCDALGNWNPQNAVALLPENDTGESMLWKATIVLSR
GVSVQYRYFKGYFLEPKTIGGPCQVIVHKWETHLQPRSITPLESEIIIDDGQFGIHNGVESGPSS
G
>1K4ZA
MPPRKELVGNKWFIENYENETESLVIDANKDESIFIGKCSQVLVQIKGKVNAISLSETESCSVVL
DSSISGMDVIKSNKFGIQVNHSLPQISIDKSDGGNIYLSKESLNTEIYTSCSTAINVNLPIGEDD

DYVEFPIPEQMKHSFADGKFKSAVFEHAG

>2QIPA

SNAMQSDHKEKIAILVDVQNVYYTCREAYRSNFDYNQFWYVATQEKEVVSAKAYAIASNDPKQRQ
FHHILRGVGFEVMLKPYIQRRDGSAKGDWDVGITLDAIEIAPDVDRVILVSGDGDFSLLVERIQQ
RYNKKVTVYGVPRLTSQTLIDCADNFVAIDDDFLL

>2FVVA

MHHHHHHSSGVDLGTENLYFQSMMKLKSNQTRTYDGDGYKKRAACLCFRSESEEEVLLVSSSRHP
DRWIVPGGGMEPEEEPSVAAVREVCEEAGVKGTLGRLVGIFENQERKHRTYVYVLIVTEVLEDWE
DSVNIGRKREWFKIEDAIKVLQYHKPVQASYFETLRQGYSANNGTPVVATTYSVSAQSSMSGIR

>3RWXA

GNNDDDGTNKAQEIAGKYEGYSIGNCAMFTDYVMGEKSVATIVPNEDGTINVTYDSGSGEFKLNN
IKVTSKTFEGSGQVELSMNDKPAGAKDFTLTGSIDEQQKLTLKVNVPSVMGGLTIEFIQGTLPIS
YHVSGTYNKEANLSVSVGSTTYPDITDCKVSIKRSSDDTVELTLKGLSNLNSSQTGRAMNLGDFT
VTDVKVTSTDNSIFKIEGSINTTDTNNTPITGTLSGTVSNSETNITFTFKPGAMPIDITAMFKGK
K

>2QGYA

MSLNNQDISIGKLSRLKIWITDNHLSDDQWSNTKKFIIIKITTEDGIEGWGEAFSINFREKGIAI
IIKELFREISNIPNLSIKSFYNKISLLSDGHRGLDFSSATSAIEIALWDISGKLKNLPLNSLLTK
SPKPNVPIYATCWSDLKKDTNDYLRQIEKFYGKKYGGIKIYPMLDSLSISIQFVEKVREIVGDEL
PLMLDLAVPEDLDQTKSFLKEVSSFNPYWIEEPVDGENISLLTEIKNTFNMKVVTGEKQSGLVHF
RELISRNAADIFNPDISGMGGLIDIIEISNEASNNGIFISPHCWNSMSVSASAMLHVCSSIPNSE
KAEIFPDYINFSKKFCELPFDIIDNKAHINKSAGLGIVIHEDILSELSIYSLDEKSNDEGHHHHH
H

>1ITHA

GLTAAQIKAIQDHWFLNIKGCLQAAADSIFFKYLTAYPGDLAFFHKFSSVPLYGLRSNPAYKAQT
LTVINYLDKVVDALGGNAGALMKAKVPSHDAMGITPKHFGQLLKLVGGVFQEEFSADPTTVAAWG
DAAGVLVAAMK

>1FTRA

MEINGVEIEDTFAEAFEAKMARVLITAASHKWAMIAVKEATGFGTSVIMCPAEAGIDCGYVPPEE
TPDGRPGVTIMIGHNDEDELKEQLLDRIGQCVMTAPTASAFDAMPEAEKEDEDRVGYKLSFFGDG
YQEEDELDGRKVWKIPVVEGEFIVEDSFGITTGVAGGNFYIMAESQPAGLQAAEAAVDAIKGVEG
AYAPFPGGIVASASKVGSKQYDFLPASTNDAYCPTVEDNELPEGVKCVYEIVINGLNEEAVKEAM
RVGIEAACQQPGVVKISAGNFGGKLGQYEIHLHDLF

>1WLGA

GLDVAISQNGFFRLVDSNGSVFYSRNGQFKLDENRNLVNMQGMQLTGYPATGTPPTIQQGANPAP
ITIPNTLMAAKSTTTASMQINLNSTDPVPSKTPFSVSDADSYNKKGTVTVYDSQGNAHDMNVYFV
KTKDNEWAVYTHDSSDPAATAPTTASTTLKFNENGILESGGTVNITTGTINGATAATFSLSFLNS
MQQNTGANNIVATNQNGYKPGDLVSYQINNDGTVVGNYSNEQEQVLGQIVLANFANNEGLASQGD
NVWAATQASGVALLGTAGSGNFGKLTNGALEASNVDLSK

>2Y26A

GLAGRGVIYIPKDCQANRYLGTLNIRDMISDFKGVQYEKWITAGLVMPTFKIVIRLPANAFTGLT
WVMSFDAYNRITSRITASADPVYTLSVPHWLIHHKLGTFSCEIDYGELCGHAMWFKSTTFESPRL
HFTCLTGNNKELAADWQAVVELYAELEEATSFLGKPTLVFDPGVFNGKFQFLTCPPIFFDLTAVT
ALRSAGLTLGQVPMVGTTKVYNLNSTLVSCVLGMGGTVRGRVHICAPIFYSIVLWVVSEWNGTTM

DWNELFKYPGVYVEEDGSFEVKIRSPYHRTPARLLADQSQRDMSSLNFYAIAGPIAPSGETAQLP
IVVQIDEIVRPDLSLPSFEDDYFVWVDFSEFTLDKEEIEIGSRFFDFTSNTCRVSMGENPFAAMI
ACHGLHSGVLDLKLQWSLNTEFGKSSGSVTITKLVGDKAMGLDGPSHVFAIQKLEGTTELLVGNF
AGANPNTRFSLYSRWMAIKLDQAKSIKVLRVLCKPRPGFSFYGRTSFPV
>3SC7X
MLNPKVAYMVWMTCLGLTLPSQAQSNDYRPSYHFTPDQYWMNEPNGLIKIGSTWHLFFQHNPTAN
VWGNICWGHATSTDLMHWAHKPTAIADENGVEAFTGTAYYDPNNTSGLGDSANPPYLAWFTGYTT
SSQTQDQRLAFSVDNGATWTKFQGNPIISTSQEAPHDITGGLESRDPKVFFHRQSGNWIMVLAHG
GQDKLSFWTSADTINWTWQSDLKSTSINGLSSDITGWEVPDMFELPVEGTEETTWVVMMTPAEGS
PAGGNGVLAITGSFDGKSFTADPVDASTMWLDNGRDFDGALSWVNVPASDGRRIIAAVMNSYGSN
PPTTTWKGMLSFPRTLSLKKVGTQQHFVQQPITELDTISTSLQILANQTITPGQTLLSSIRGTAL
DVRVAFYPDAGSVLSLAVRKGASEQTVIKYTQSDATLSVDRTESGDISYDPAAGGVHTAKLEEDG
TGLVSIRVLVDTCSVEVFGGQGEAVISDLIFPSDSSDGLALEVTGGNAVLQSVDVRSVSLE
>3DSMA
ASGLFITNEGNFQYSNATLSYYDPATCEVENEVFYRANGFKLGDVAQSMVIRDGIGWIVVNNSHV
IFAIDINTFKEVGRITGFTSPRYIHFLSDEKAYVTQIWDYRIFIINPKTYEITGYIECPDMDMES
GSTEQMVQYGKYVYVNCWSYQNRILKIDTETDKVVDELTIGIQPTSLVMDKYNKMWTITDGGYEG
SPYGYEAPSLYRIDAETFTVEKQFKFKLGDWPSEVQLNGTRDTLYWINNDIWRMPVEADRVPVRP
FLEFRDTKYYGLTVNPNNGEVYVADAIDYQQQGIVYRYSPQGKLIDEFYVGIIPGAFCWKLEHHH
HHH
>3D1BA
GSHMNPLASLTTDKNDLYINWLKSLSFFQTNSSCAEALVKVIPHYHNKLIDFSQVLQLVFSASEK
FPIQENQPLPEQLMFLSNLEKQTPFAKAVGSSIYKLVTGKNLSLDFASQILKEASILEH
>4MT2A
XMDPNCSCATDGSCSCAGSCKCKQCKCTSCKKSCCSCCPVGCAKCSQGCICKEASDKCSCCA
>1JNDA
ASNLVCYYDSSSYTREGLGKLLNPDLEIALQFCSHLVYGYAGLRGENLQAYSMNENLDIYKHQFS
EVTSLKRKYPHLKVLLSVGGDHDIDPDHPNKYIDLLEGEKVRQIGFIRSAYELVKTYGFDGLDLA
YQFPKNKPRKVHGDLGLAWKSIKKLFTGDFIVDPHAALHKEQFTALVRDVKDSLRADGFLLSLTV
LPNVNSTWYFDIPALNGLVDFVNLATFDFLTPARNPEEADYSAPIYHPDGSKDRLAHLNADFQVE
YWLSQGFPSNKINLGVATYGNAWKLTKDSGLEGVPVVPETSGPAPEGFQSQKPGLLSYAEICGKL
SNPQNQFLKGNESPLRRVSDPTKRFGGIAYRPVDGQITEGIWVSYDDPDSASNKAAYARVKNLGG
VALFDLSYDDFRGQCSGDKYPILRAIKYRL
>2YH6A
GDTASLLVENGRGNTLWPQVVSVLQAKNYTITQRDDAGQTLTTDWVQWNRLDEDEQYRGRYQISV
KPQGYQQAVTVKLLNLEQAGKPVADAASMQRYSTEMMNVISAGLDKS
>3SDBA
SMNFYSAYQHGFVRVAACTHHTTIGDPAANAASVLDMARACHDDGAALAVFPELTLSGYSIEDVL
LQDSLLDAVEDALLDLVTESADLLPVLVVGAPLRHRHRIYNTAVVIHRGAVLGVVPKSYLPTYRE
FYERRQMAPGDGERGTIRIGGADVAFGTDLLFAASDLPGFVLHVEIAEDMFVPMPPSAEAALAGA
TVLANLSGSPITIGRAEDRRLLARSASARCLAAYVYAAAGEGESTTDLAWDGQTMIWENGALLAE
SERFPKGVRRSVADVDTELLRSERLRMGTFDDNRRHHRELTESFRRIDFALDPPAGDIGLLREVE
RFPFVPADPQRLQQDCYEAYNIQVSGLEQRLRALDYPKVVIGVSGGLDSTHALIVATHAMDREGR
PRSDILAFALPGFATGEHTKNNAIKLARALGVTFSEIDIGDTARLMLHTIGHPYSVGEKVYDVTF

ENVQAGLRTDYLFRIANQRGGIVLGTGDLSELALGWSTYGVGDQMSHYNVNAGVPKTLIQHLIRW
VISAGEFGEKVGEVLQSVLDTEITPELIPTGEEELQSSEAKVGPFALQDFSLFQVLRYGFRPSKI
AFLAWHAWNDAERGNWPPGFPKSERPSYSLAEIRHWLQIFVQRFYSFSQFKRSALPNGPKVSHGG
ALSPRGDWRAPSDMSARIWLDQIDREVPKG
>2QQ4A
MSVLDELYREILLDHYQSPRNFGVLPQATKQAGGMNPSCGDQVEVMVLLEGDTIADIRFQGQGCA
ISTASASLMTEAVKGKKVAEALELSRKFQAMVVEGAPPDPTLGDLLALQGVAKLPARVKCATLAW
HALEEALR
>1IZMA
GSMLISHSDMNQQLKSAGIGFNATELHGFLSGLLCGGLKDQSWLPLLYQFSNDNHAYPTGLVQPV
TELYEQISQTLSDVEGFTFELGLTEDENVFTQADSLSDWANQFLLGIGLAQPELAKEKGEIGEAV
DDLQDICQLGYDEDDNEEELAEALEEIIEYVRTIAMLFYSHFNEGEIESKPVLH
>1NQJA
GGPGNEKLKEKENNDSSDKATVIPNFNTTMQGSLLGDDSRDYYSFEVKEEGEVNIELDKKDEFGV
TWTLHPESNINDRITYGQVDGNKVSNKVKLRPGKYYLLVYKYSGSGNYELRVNK
>2X0QA
MSRTTPPHPAEIVAHLQPEIWNKVNRLLVRKAISEYAHEWLLEPQRLGPGETPGFERFRLTLADG
AQYDFDAQVMAMRHWRIPPESIVKTVAGVPAPLDALQFVIEIRDKLGLPVDRLPIYMDEITSTLH
GSAYKHGRTTLGAAALARADYQTIETSMIEGHPSFVANNGRLGFDAEDYHGYAPEAATPVRLMWL
AVHKDNAHFSCLSDMDYDSLMSEELGESAVTDFAARLREQGLHPADYYFMPAHPWQWFNKLSLAF
APYVAQRKIVCLGYGEEQYLAQQSIRTFFNISRPGKRYVKTSLSILNMGFMRGLSPYYMAGTPAI
NEYIHDLISADPWLRANGFRILREVASMGFRNYYYEAAIDTDTPYKKMFSALWRENPLTLIAPGQ
NLMTMAALLHVDPQGRALLPELIQASGLDAGTWLERYVDAYLTPLIHCFYAHDLVFMPHGENVIL
VIQDGVPVRAFMKDIAEESSILNPQVRLPQAAQRLAADVPEAYKLLTIFVDVFEGYFRHLTQILV
ETELMPEHDFWRLVAGRIAAYQQAHPQRLDKYRRYDLFAPDMIHSCLNRLQLANNLQMVNLADPI
GSFQMAPNLPNPIACFRPSWLGSGEALQTLTAA
>3H6JA
MNTYFDIPHRLVGKALYESYYDHFGQMDILSDGSLYLIYRRATEHVGGSDGRVVFSKLEGGIWSA
PTIVAQAGGQDFRDVAGGTMPSGRIVAASTVYETGEVKVYVSDDSGVTWVHKFTLARGGADYNFA
HGKSFQVGARYVIPLYAATGVNYELKWLESSDGGETWGEGSTIYSGNTPYNETSYLPVGDGVILA
VARVGSGAGGALRQFISLDDGGTWTDQGNVTAQNGDSTDILVAPSLSYIYSEGGTPHVVLLYTNR
TTHFCYYRTILLARAVAGSSGWTERVPAYSAPAASGYTSQVVLGGRRILGNLFRETSSTTSGAYQ
FEVYLGGVPDFESDWFSVSSNSLYTLSHGLQRSPRRVVVEFARSSSPSTWNIVMPSYFNDGGHKG
SGAQVEVGSLNIRLGTGAAVWGTGYFGGIDNSATTRLATGYYRVRAWI
>3IBYA
MTHALLIGNPNCGKTTLFNALTNANQRVGNWPGVTVEKKTGEFLLGEHLIEITDLPGVYSLVANA
EGISQDEQIAAQSVIDLEYDCIINVIDACHLERHLYLTSQLFELGKPVVVALNMMDIAEHRGISI
DTEKLESLLGCSVIPIQAHKNIGIPALQQSLLHCSQKIKPLKLSLSVAAQQILNDLENQLISKGY
KNSFAYYFSRRLAEGDTLIGEKAFTESLLIKLQETEQNLDVLLADARYQKIHEIVTLVQKK
>3FHHA
TETMTVTATGNARSSFEAPMMVSVIDTSAPENQTATSATDLLRHVPGITLDGTGRTNGQDINMRG
YDHRGVLVLVDGIRQGTDTGHLNGTFLDPALIKRVEIVRGPSALLYGSGALGGVISYDTVDAKDL
LQEGQSSGFRVFGTGGTGDHSLGLGASAFGRTENLDGIVAWSSRDRGDLRQSNGETAPNDESINN
MLAKGTWQIDSAQSLSGLVRYYNNDAREPKNPQTVEASESSNPMVDRSTIQRDAQLSYKLAPQGN

DWLNADAKIYWSEVRINAQNTGSSGEYREQITKGARLENRSTLFADSFASHLLTYGGEYYRQEQG
SHHHHHHHPGGATTGFPQAKIDFSSGWLQDEITLRDLPITLLGGTRYDSYRGSSDGYKDVDADKW
SSRAGMTINPTNWLMLFGSYAQAFRAPTMGEMYNDSKHFSIGRFYTNYWVPNPNLRPETNETQEY
GFGLRFDDLMLSNDALEFKASYFDTKAKDYISTTVDFAAATTMSYNVPNAKIWGWDVMTKYTTDL
FSLDVAYNRTRGKDTDTGEYISSINPDTVTSTLNIPIAHSGFSVGWVGTFADRSTHISSSYSKQP
GYGVNDFYVSYQGQQALKGMTTTLVLGNAFDKEYWSPQGIPQDGRNGKIFVSYQW
>2I71A
MGSSHHHHHHSSGRENLYFQGMASIVFSTIGNPKGYQKVTYEIDGEKFESNVSVLALRDLLKVDK
TVVILGISVADVYNCKYADYRSCKECIIQNSKNDLGISESYVVAPNVYQKFKGKPDHYFTYIYYH
SLRILEKEGINEVFIDTTHGINYMGVLAKEAIQLAVSAYAAKSEKEVKVSLYNSDPVGKDVSDTV
KLHEIEAIKISPLSGLKYVTYQILNKDKNFFNKIFSDSVNAIPRFATALDNGLFIYLSEKDSSLH
LKRLEDDLSKDPLLTPSENEINVVYKDMKYALSHALFYVISRFSGNVDLDTLRHYAETYADKVTR
AIIENEVDKIEKYQMGSERKLLGEYMKVEGKGFDKRILYAHGGLPYAGTYVYKEKDKVYVTYGDK
IDEIERQIGS
>3BRKX
MSEKRVQPLARDAMAYVLAGGRGSRLKELTDRRAKPAVYFGGKARIIDFALSNALNSGIRRIGVA
TQYKAHSLIRHLQRGWDFFRPERNESFDILPASQRVSETQWYEGTADAVYQNIDIIEPYAPEYMV
ILAGDHIYKMDYEYMLQQHVDSGADVTIGCLEVPRMEATGFGVMHVNEKDEIIDFIEKPADPPGI
PGNEGFALASMGIYVFHTKFLMEAVRRDAADPTSSRDFGKDIIPYIVEHGKAVAHRFADSCVRSD
FEHEPYWRDVGTIDAYWQANIDLTDVVPDLDIYDKSWPIWTYAEITPPAKFVHDDEDRRGSAVSS
VVSGDCIISGAALNRSLLFTGVRANSYSRLENAVVLPSVKIGRHAQLSNVVIDHGVVIPEGLIVG
EDPELDAKRFRRTESGICLITQSMIDKLDL
>1VI1A
MSLRIAVDAMGGDHAPKAVIDGVIKGIEAFDDLHITLVGDKTTIESHLTTTSDRITVLHADEVIE
PTDEPVRAVRRKKNSSMVLMAQEVAENRADACISAGNTGALMTAGLFIVGRIKGIDRPALAPTLP
TVSGDGFLLLDVGANVDAKPEHLVQYAIMGSVYSQQVRGVTSPRVGLLNVGTEDKKGNELTKQTF
QILKETANINFIGNVEARDLLDDVADVVVTDGFTGNVTLKTLEGSALSIFKMMRDVMTSTLTSKL
AAAVLKPKLKEMKMKMEYSNYGGASLFGLKAPVIKAHGSSDSNAVFRAIRQAREMVSQNVAALIQ
EEVKEEKTDEEGGSHHHHHH
>4J05A
GPMLADLDHFGKNYKHDEEAQRNQKPWMLTWPQIKLVLLAGVGFFLDAYDLFIINQVAPMLAQVY
FPKTGLPAQRQDLMKAAANIGCVVGQVMFGVLGDSFGRKFVYGKELILIIVATIFQMSAPSHWDG
NRVLTWITICRVFLGIGIGGDYPMSATVVSDRANIHRRGTLLCFIFANQGWGSFVGSLVTIVTIS
GFKHRLKSGHTHDVDKAWRILIGLSLIPAFGTLYQRLTLPESRKFELTRDAASSSTVAIDKKDHD
ATHEVKDAPESEKSSPKVTPADAIDDDRHGVIASKKAHWQEFVAYFSTWNHFRNLLGSMLGWFLV
DIAFYGINLNQSVVLAQIGFAGKTGDVYDKLFQLATGNIIVTALGFLPGYYFTLFLIDIVGRKKL
QFMGFIMSGLFLAILAGEIDHIGKGPLLACFTFMQFFFNFGANTTTFIVAAELFPTRIRASAHGI
SAAAGKCGAILSSLVFNQLKAKIGTSAVLWIFFSTCILGFISTFLIDETMGVDPDEKDLEERRAR
GEIPGGLVPR
>1QTFA
KEYSAEEIRKLKQKFEVPPTDKELYTHITDNARSPYNSVGTVFVKGSTLATGVLIGKNTIVTNYH
VAREAAKNPSNIIFTPAQNRDAEKNEFPTPYGKFEAEEIKESPYGQGLDLAIIKLKPNEKGESAG
DLIQPANIPDHIDIAKGDKYSLLGYPYNYSAYSLYQSQIEMFNDSQYFGYTEVGNSGSGIFNLKG
ELIGIHSGKGGQHNLPIGVFFNRKISSLYSVDNTFGDTLGNDLKKRAKLDK

>3LMBA
MNASLTPDQVSKKLKQFFSDHLPISQFMGLEIESYDGDTLILTAPLEPNINDKQTAFGGSLYNAA
VMACWGMVYLKTQEENIACNQVVTEGNMKYIAPVYGRIRAICHAPDEEELANFFDHFERKGKARI
SLEAAIYNDACVMKIEPETKPSVKFNGQYAILKNQ
>4I4OA
VNFPNIPAEGVQFRLRARDTGYVIYSRTENPPLVWQYNGPPYDDQLFTLIYGTGPRKNLYAIKSV
PNGRVLFSRTSASPYVGNIAGDGTYNDNWFQFIQDDNDPNSFRIYNLASDTVLYSRTTADPKFGN
FTGAKYDDQLWHFELV
>1N62A
MAKAHIELTINGHPVEALVEPRTLLIHFIREQQNLTGAHIGCDTSHCGACTVDLDGMSVKSCTMF
AVQANGASITTIEGMAAPDGTLSALQEGFRMMHGLQCGYCTPGMIMRSHRLLQENPSPTEAEIRF
GIGGNLCRCTGYQNIVKAIQYAAAKINGVPFEEAAE
>4FWWA
VKYVVPSFSAGGLVQAMVTYEGDRNESAVFVAIRNRLHVLGPDLKSVQSLATGPAGDPGCQTCAA
CGPGPHGPPGDTDTKVLVLDPALPALVSCGSSLQGRCFLHDLEPQGTAVHLAAPACLFSAHHNRP
DDCPDCVASPLGTRVTVVEQGQASYFYVASSLDAAVAASFSPRSVSIRRLKADASGFAPGFVALS
VLPKHLVSYSIEYVHSFHTGAFVYFLTVQPASVTDDPSALHTRLARLSATEPELGDYRELVLDCR
FAPKLVPRGSPEGGQPYPVLQVAHSAPVGAQLATELSIAEGQEVLFGVFVTGKDGGPGVGPNSVV
CAFPIDLLDTLIDEGVERCCESPVHPGLRRGLDFFQSPSFCPNPPGLEALSPNTSCRHFPLLVSS
SFSRVDLFNGLLGPVQVTALYVTRLDNVTVAHMGTMDGRILQVELVRSLNYLLYVSNFSLGDSGQ
PVQRDVSRLGDHLLFASGDQVFQVPIQGPGCRHFLTCGRCLRAWHFMGCGWCGNMCGQQKECPGS
WQQDHCP
>3B47A
NAIMDLQTRNTRGLSTLVVRDIGELMMAGDMAVIERYVADVRGKGAVLDLRIYDAAGRPAGKKQD
APDGEVQAALTSGATAEKRHKVDGRHVLSFIVPLANEVRCQSCHEQGARFNGAMLLTTSLEEGYA
GARN
>2FWHA
ATHTAQTQTHLNFTQIKTVDELNQALVEAKGKPVMLDLYADWCVACKEFEKYTFSDPQVQKALAD
TVLLQANVTANDAQDVALLKHLNVLGLPTILFFDGQGQEHPQARVTGFMDAETFSAHLRDRQPHH
HHHH
>2H26A
EHAFQGPTSFHVIQTSSFTNSTWAQTQGSGWLDDLQIHGWDSDSGTAIFLKPWSKGNFSDKEVAE
LEEIFRVYIFGFAREVQDFAGDFQMKYPFEIQGIAGCELHSGGAIVSFLRGALGGLDFLSVKNAS
CVPSPEGGSRAQKFCALIIQYQGIMETVRILLYETCPRYLLGVLNAGKADLQRQVKPEAWLSSGP
SPGPGRLQLVCHVSGFYPKPVWVMWMRGEQEQQGTQLGDILPNANWTWYLRATLDVADGEAAGLS
CRVKHSSLEGQDIILYWRNPIXXXXX
>2QYCA
GMTMFLHVVMMEFDDGIDAGFFRTVDEYVARMKRECDGLLLYHFGENVAARSQGYTHATSSAFVD
AAAHDAYQVCPAHVAMKAFMGPRIKRVVVYDGEVPAIG
>3K9TA
GMEEINKYIQNSSETGGEIYNLIEELFPICRSITGNGVRKTMDIIRKHIPLEIHEVKSGTKVFDW
TVPKEWNIKDAYVRNSKGEKVIDFKENNLHVMSYSVPVHKTMTLDELKPYLHTIPGNKDRIPYLT
SYYKENWGFSLTQNKFDELCDDDYEVVIDSSLEDGSLTYGEYYIRGELEEEILLTTYTCHPSMCN
DNLSGVALITFIAKALSKLKTKYSYRFLFAPETIGSITWLSRNEDKLKNIKMGLVATCVGDAGIK

NYKRTKFGDAEIDKIVEKVLMHCGSEYYVADFFPWGSDERQFSSPGINLSVGSLMRSCYGFDGYH
TSADNLCYMNKDGLADSYKTYLEVIYTIENNRTYLNLNPKCEPQLGKRGIYRMIGGGSDYPFDEF
AMFWVLNMSDGKNSLLDIAYKSGMEFRRIKYAADALYRVELLKLV
>2B0TA
MAKIIWTRTDEAPLLATYSLKPVVEAFAATAGIEVETRDISLAGRILAQFPERLTEDQKVGNALA
ELGELAKTPEANIIKLPNISASVPQLKAAIKELQDQGYDIPELPDNATTDEEKDILARYNAVKGS
AVNPVLREGNSDRRAPIAVKNFVKKFPHRMGEWSADSKTNVATMDANDFRHNEKSIILDAADEVQ
IKHIAADGTETILKDSLKLLEGEVLDGTVLSAKALDAFLLEQVARAKAEGILFSAHLKATMMKVS
DPIIFGHVVRAYFADVFAQYGEQLLAAGLNGENGLAAILSGLESLDNGEEIKAAFEKGLEDGPDL
AMVNSARGITNLHVPSDVIVDASMPAMIRTSGHMWNKDDQEQDTLAIIPDSSYAGVYQTVIEDCR
KNGAFDPTTMGTVPNVGLMAQKAEEYGSHDKTFRIEADGVVQVVSSNGDVLIEHDVEANDIWRAC
QVKDAPIQDWVKLAVTRSRLSGMPAVFWLDPERAHDRNLASLVEKYLADHDTEGLDIQILSPVEA
TQLSIDRIRRGEDTISVTGNVLRDYNTDLFPILELGTSAKMLSVVPLMAGGGLFETGAGGSAPKH
VQQVQEENHLRWDSLGEFLALAESFRHELNNNGNTKAGVLADALDKATEKLLNEEKSPSRKVGEI
DNRGSHFWLTKFWADELAAQTEDADLAATFAPVAEALNTGAADIDAALLAVQGGATDLGGYYSPN
EEKLTNIMRPVAQFNEIVDALKK
>3SD2A
GANSNDIHLLKDSRSNPMGIPIQPTYEKCAILSNILNVSFGRAKDYAIITVTNKATGEIVHSKTY
HNTSIVMIDMSSCEKGEYTIHIILNDCLLEGTFTVQ
>1M2DA
AEFKHVFVCVQDRPPGHPQGSCAQRGSREVFQAFMEKIQTDPQLFMTTVITPTGCMNASMMGPVV
VVYPDGVWYGQVKPEDVDEIVEKHLKGGEPVERLVISKGKPPGMF
>3K1TA
GMMVPHLTTALTGPLLTLEKRLLDNMPRIEHWFRSQWQEYGAPFYASVDLRNAGFKLAPVDTNLF
PGGFNNLNPDFLPLCIQAAMVAVEKICPDARRLLLIPENHTRNTFYLRNVHALTHILRQAGLEVR
IGSIAPEITAPTFLETHDGHSILLEPVRRKANRLELDNFDSCAILLNNDLSGGIPDILQGLEQSL
IPPLHAGWATRRKSNHFTAYDRVVEEFAPLIDIDPWLLNPYFDTCGGLDFHARLGEEQLAEKVDS
LLAKIRRKYAEYGVKQEPFVIVKADAGTYGMGIMTVKSADDVRDLNRKQRNKMSVVKEGLKVSEV
ILQEGVYTFEHLKDAVAEPVIYMMDHFVVGGFYRVHTSRGADENLNAPGMHFEPLTFETPCSTPD
CAGAPDAAPNRFYAYGVVARLALLAATIELQETDPDLLDERT
>2W2EA
MPDIENQAADGQAEIKPEDAPYITNAYKPAYARWGFGSDSVRNHFIAMSGEFVGTFLFLWSAFVI
AQIANQAPETPDGGSNPAQLIMISFGFGFGVMVGVFITYRVSGGNLNPAVTLALVLARAIPPFRG
ILMAFTQIVAGMAAAGAASAMTPGEIAFANALGGGASRTRGLFLEAFGTAILCLTVLMLAVEKHR
ATWFAPFVIGIALLIAHLICIYYTGAGLNPARSFGPAVAARSFPNYHWIYWLGPILGAFLAYSIW
QMWKWLNYQTTNPGQDSDA
>1ZELA
GAMVVSPAGADRRIPTWASRVVSGLARDRPVVVTKEDLTQRLTEAGCGRDPDSAIRELRRIGWLV
QLPVKGTWAFIPPGEAAISDPYLPLRSWLARDQNAGFMLAGASAAWHLGYLDRQPDGRIPIWLPP
AKRLPDGLASYVSVVRIPWNAADTALLAPRPALLVRRRLDLVAWATGLPALGPEALLVQIATRPA
SFGPWADLVPHLDDLVADCSDERLERLLSGRPTSAWQRASYLLDSGGEPARGQALLAKRHTEVMP
VTRFTTAHSRDRGESVWAPEYQLVDELVVPLLRVIGKA
>2JCQA
MNQIDLNVTCRYAGVFHVEKNGRYSISRTEAADLCQAFNSTLPTMDQMKLALSKGFETCRYGFIE

GNVVIPRIHPNAICAANHTGVYILVTSNTSHYDTYCFNASAPPEEDCTSVTDLPNSFDGPVTITI
VNRDGTRYSKKGEYRTHQEDIDAS
>3SXUB
MGTSRRDWQLQQLGITQWSLRRPGALQGEIAIAIPAHVRLVMVANDLPALTDPLVSDVLRALTVS
PDQVLQLTPEKIAMLPQGSHCNSWRLGTDEPLSLEGAQVASPALTDLRANPTARAALWQQICTYE
HDFFPRND
>3CMGA
MSLRQDILLNNNWNFRFSHQVQGDTRRVDLPHTWNAQDALAGKIDYKRGIGNYEKALYIRPEWKG
KRLFLRFDGVNSIADVFINRKHIGEHRGGYGAFIFEITDLVKYGEKNSVLVRANNGEQLDIMPLV
GDFNFYGGIYRDVHLLITDETCISPLDYASPGVYLVQEVVSPQEAKVCAKVNLSNRAADGTAELQ
VLVTDGTKVICKESRNVSLKQGADILEQLPLLIQKPRLWNGCEDPFMYQVSISLHKDGKQIDSVT
QPLGLRYYHTDPDKGFFLNGKHLPLHGVCRHQDRAEVGNALRPQHHEEDVALMREMGVNAIRLAH
YPQATYMYDLMDKHGIVTWAEIPFVGPGGYADKGFVDQASFRENGKQQLIELIRQHYNHPSICFW
GLFNELKEVGDNPVEYVKELNALAKQEDPTRPTTSASNQDGNLNFITENIAWNRYDGWYGSTPKT
LATFLDRTHKKHPELRIGISEYGAGASIYHQQDSLKQPSASGWWHPENWQTYYHMENWKIIAERP
FVWGTFVWNMFDFGAAHRTEGDRPGINDKGLVTFDRKVRKDAFYFYKANWNKQEPMIYLAEKRCR
LRYQPEQTFMAFTTAPEAELFVNGVSCGKQKADTYSTVVWKNVKLTSGENIIRVTTPGKKPLTDE
VTVEYKEDREGHHHHHH
>3OAKC
DPFTHMSDKIDEMYDIFGDGHDYDWALEIEN
>2GU9A
MQYATLELNNAFKVLFSLRQVQAAEMVIAPGDREGGPDNRHRGADQWLFVVDGAGEAIVDGHTQA
LQAGSLIAIERGQAHEIRNTGDTPLKTVNFYHPPAYDAQGEPLPAGEG
>2W56A
MSKLTFTASSLPVSKKLHKLLSKQLTAHLLSSEALTTSRYLVFNFRDKSYSADEGGFHPVEMAIC
QTSTGEWSIEYITDFAYMGNYYPELERNLDFDFRVGQFFVAYRGWLPMQGSRDAKELYRLWESNF
LAYVDMDAYNEIAITAQ
>2HY7A
MGVSPAAPASGIRRPCYLVLSSHDFRTPRRANIHFITDQLALRGTTRFFSLRYSRLSRMKGDMRL
PLDDTANTVVSHNGVDCYLWRTTVHPFNTRRSWLRPVEDAMFRWYAAHPPKQLLDWMRESDVIVF
ESGIAVAFIELAKRVNPAAKLVYRASDGLSTINVASYIEREFDRVAPTLDVIALVSPAMAAEVVS
RDNVFHVGHGVDHNLDQLGDPSPYAEGIHAVAVGSMLFDPEFFVVASKAFPQVTFHVIGSGMGRH
PGYGDNVIVYGEMKHAQTIGYIKHARFGIAPYASEQVPVYLADSSMKLLQYDFFGLPAVCPNAVV
GPYKSRFGYTPGNADSVIAAITQALEAPRVRYRQCLNWSDTTDRVLDPRAYPETRLYPHPPTAAP
QLSSEAALSHHHHHHH
>2BKRA
MDPVVLSYMDSLLRQSDVSLLDPPSWLNDHIIGFAFEYFANSQFHDSSDHVSFISPEVTQFIKCT
SNPAEIAMFLEPLDLPNKRVVFLAINDNSNQAAGGSHWSLLVYLQDKNSFFHYDSHSRSNSVHAK
QVAEKLEAFLGRKGDKLAFVEEKAPAQQNSYDCGMYVICNTEALCQNFFRQQTESLLQLLTPAYI
TKKRGEWKDLIATLAKK
>2C1VA
ETEAIDNGALREEAKGVFEAIPEKMTAIKQTEDNPEGVPLTAEKIELGKVLFFDPRMSSSGLISC
QTCHNVGLGGVDGLPTSIGHGWQKGPRNAPTMLNAIFNAAQFWDGRAADLAEQAKGPVQAGVEMS
NTPDQVVKTINSMPEYVEAFKAAFPEEADPVTFDNFAAAIEQFEATLITPNSAFDRFLAGDDAAM

TDQEKRGLQAFMETGCTACHYGVNFGGQDYHPFGLIAKPGAEVLPAGDTGRFEVTRTTDDEYVFR
AAPLRNVALTAPYFHSGVVWELAEAVKIMSSAQIGTELTDQQAEDITAFLGTLTGEQPVIDHPIL
PVRTGTTPLPTPM
>2HO0A
GSHMYEYPVFSHVQAGMFSPELRTFTKGDAERWVSTTKKASDSAFWLEVEGNSMTTPTGSKTSFP
DGMLILVDPEQAVEPGDFCIARLGGDEFTFKKLIRDSGQVFLQPLNPQYPMIPCNESCSVVGKVI
ASQ
>2JH1A
VGPEAYGEASHSHSPASGRYIQQMLDQRCQEIAAELCQSGLRKMCVPSSRIVARNAVGITHQNTL
QWRCFDTASLLESNQENNGVNCVDDCGHTIPCPGGVHRQNSNHATRHEILSKLVEEGVQRFCSPY
QASANKYCNDKFPGTIARRSKGFGNNVEVAWRCYEKASLLYSVYAECASNCGTTWYCPGGRRGTS
TELDKRHYTEEEGIRQAIGSVDSPCSEVEVCLPKDENPPLCLDESGQISRT
>3AA0B
MSDQQLDCALDLMRRLPPQQIEKNLSDLIDLVPSLCEDLLSSVDQPLKIARDKVVGKDYLLCDYN
RDGDSYRSPWSNKYDPPLEDGAMPSARLRKLEVEANNAFDQYRDLYFEGGVSSVYLWDLDHGFAG
VILIKKAGDGSKKIKGCWDSIHVVEVQEKSSGRTAHYKLTSTVMLWLQTNKTGSGTMNLGGSLTR
QMEKDETVSDSSPHIANIGRLVEDMENKIRSTLNEIYFGKTKDIVNGLR
>3FM2A
GMSHSLKDFLEACETLGTLRLIVTSSAAVLEARGKIEKLFYAELAKGKYANMHTEGFEFHLNMEK
ITQVKFETGEAKRGNFTTYAIRFLDEKQESALSLFLQWGKPGEYEPGQVEAWHTLKEKYGEVWEP
LPVQL
>3GS9A
GMNSDIIVADFWKNNEEILTDFDKDSFCESWTENEMWSIEFKVAQTPKNAHCYSFLDYESSVYFR
GQEFVVKQLSHDAVGKTLSKDIRAPHIYYTCQDGRQDDAITGSFTLEQCLTHIFKTDNRGFSWEI
IDPSNILEKVQQENFGNNNYLTLIDQLLDDYGVVVIPDNRHLVFKPREIYGAKTENFIRYKYNTD
EASFDIDTLSLKTKIKGYGKVDSNGNNYFSPITYTSPEVEKWGIRWQEPVSDERYTVAGNMQRRL
KLELQDYPATTGSVILKNDYECEKGDYVLFIYEPLGIDYDVQIVAYKKYPFTIKAPEITLSNNKK
SIVSIMAQLAKVLKGAK
>4DUQA
MMSSSLEQALAVLVTTFHKYSSQEGDKFKLSKGEMKELLHKELPSFVGEKVDEEGLKKLMGSLDE
NSDQQVDFQEYAVFLALITVMSNDFFQGCPDRP
>3SNOA
GMALEPQIKSAPTPVILIVEPYGGSIRQQNPNLPMVFWDDAALTRGDGIFETLLIRDGHACNVRR
HGERFKASAALLGLPEPILEDWEKATQMGIESWYSHPNAGEASCTWTLSRGRSSTGLASGWLTIT
PVSSDKLAQREHGVSVMTSSRGYSIDTGLPGIGKATRGELSKVERTPAPWLTVGAKTLAYAANMA
ALRYAKSNGFDDVIFTDGDRVLEGATSTVVSFKGDKIRTPSPGGDILPGTTQAALFAHATEKGWR
CKEKDLSIDDLFGADSVWLVSSVRGPVRVTRLDGHKLRKPDNEKEIKALITKALG
>2GR8A
ASWSHPQFEKSGGGGGLVPRGSKRADAGTASALAASQLPQATMPGKSMVAIAGSSYQGQNGLAIG
VSRISDNGKVIIRLSGTTNSQGKTGVAAGVGYQW
>2WB7A
MNATINDDDIDDVKKALDHATQAAHKAAAELTAKLRSDFVEYGNGGTAGQVLIHIYGPGLIYGFS
AFPVQIRLEIPNQPVPFNKVHITEVTAYVIDENNRTYWTRVWNSSTFRQGGYIADTLDLVTVMKA
PDPLVYQIRDAIVTGQISRELYDKIWNTSTTHFEIRVIVKGYQEAWKTDSSVSNQSSCPSDGHWY

EDACWVHDKDIDFTLKAETTTAWGHVTGTNDVATIDGGMLGSLPIKFLQSLDLSGKWVLYQNKYA
GALSDFIIITAASPVHVLNSTAMYKFLITPNPGYFQPANPKISDEYRFVTLRVIEGGRMELADTT
TGHIGDLTEPTFFGLTAHYTDAPGTLDYHALGLVYAYVERDDGVKIPIWLAAEPMISVLSNTYTV
MKDQDVKNLIDLYKKKDREKINATTKAMINSLQEKIDEAEQLLAKAKGMNNENAIEYAQGAIDEY
KAAINDLQKAAQQDDYQMFLNYLNAAKKHEMAGDYYVNAARKALNGDLEQAKIDAEKAKEYSNLA
KEYEPG
>3PF0A
GDDNNAAEVDRQVAQDSAEPKTGENAAAGDSSSTNKNAEKIVAVDISAETEKTYLTHVANDMVIP
AYADAAKQSDLLHDLAQKHCQKAPVSGDELQALRDQWLVLAQAWASAEMVNFGPATASMSNLYIN
YYPDERGLVHGGVADLITANPALTAEQLANESAVVQGIPGLEEALYANDSLDAGQCAYVMSASSA
LGTRLKDIEKNWQQNAIKLLAIDKTAESDQGLNQWFNSLLSLVETMKSNAIEQPLGLSGKAKGHL
PAATAGQSRAIINAKLATLNKAMTDPVLTAILGSNNENTVADTLSTALADTTALLAQMPEDLATA
DKATQQELYDHLTNITRLIKSQLIPTLGIRVGFNSTDGD
>2NPTA
SMALGPFPAMENQVLVIRIKIPNSGAVDWTVHSGPQLLFRDVLDVIGQVLPEATTTAFEYEDEDG
DRITVRSDEEMKAMLSYYYSTVMEQQVNGQLIEPLQIFPRA
>2BDRA
MRTLMIEPLTKEAFAQFGDVIETDGSDHFMINNGSTMRFHKLATVETAEPEDKAIISIFRADAQD
MPLTVRMLERHPLGSQAFIPLLGNPFLIVVAPVGDAPVSGLVRAFRSNGRQGVNYHRGVWHHPVL
TIEKRDDFLVVDRSGSGNNCDEHYFTEEQMLILNPHQLEHHHHHH
>1IUQA
ASHSRKFLDVRSEEELLSCIKKETEAGKLPPNVAAGMEELYQNYRNAVIESGNPKADEIVLSNMT
VALDRILLDVEDPFVFSSHHKAIREPFDYYIFGQNYIRPLIDFGNSFVGNLSLFKDIEEKLQQGH
NVVLISNHQTEADPAIISLLLEKTNPYIAENTIFVAGDRVLADPLCKPFSIGRNLICVYSKKHMF
DIPELTETKRKANTRSLKEMALLLRGGSQLIWIAPSGGRDRPDPSTGEWYPAPFDASSVDNMRRL
IQHSDVPGHLFPLALLCHDIMPPPSQVEIEIGEKRVIAFNGAGLSVAPEISFEEIAATHKNPEEV
REAYSKALFDSVAMQYNVLKTAISGKQGLGASTADVSLSQPW
>2W5QA
SEDDLTKVLNYTKQRQTEPNPEYYGVAKKKNIIKIHLESFQTFLINKKVNGKEVTPFLNKLSSGK
EQFTYFPNFFHQTGQGKTSDSEFTMDNSLYGLPQGSAFSLKGDNTYQSLPAILDQKQGYKSDVMH
GDYKTFWNRDQVYKHFGIDKFYDATYYDMSDKNVVNLGLKDKIFFKDSANYQAKMKSPFYSHLIT
LTNHYPFTLDEKDATIEKSNTGDATVDGYIQTARYLDEALEEYINDLKKKGLYDNSVIMIYGDHY
GISENHNNAMEKLLGEKITPAKFTDLNRTGFWIKIPGKSGGINNEYAGQVDVMPTILHLAGIDTK
NYLMFGTDLFSKGHNQVVPFRNGDFITKDYKYVNGKIYSNKNNELITTQPADFEKNKKQVEKDLE
MSDNVLNGDLFRFYKNPDFKKVNPSKYKYETGPK
>3NSWA
GSHMEYCPKMLSEIRQEDINDVETVAYVTVTGKTARSYNLQYWRLYDVPKTAPSQWPSFGTLRDD
CGNIQLTADTDYVLGCKSGNQDCFVKLHDGLSQKEKDLLKE
>3F79A
AANRELQASLNLLQEDQNAGRQVQMNMLPVTPWSIEGLEFSHRIIPSLYLSGDFVDYFRVDERRV
AFYLADVSGHGASSAFVTVLLKFMTTRLLYESRRNGTLPEFKPSEVLAHINRGLINTKLGKHVTM
LGGVIDLEKNSLTYSIGGHLPLPVLFVEGQAGYLEGRGLPVGLFDDATYDDRVMELPPSFSLSLF
SDGILDVLPGATLKEKEASLPEQVAAAGGTLDGLRQVFGLANLAEMPDDIALLVLSRNLA
>3C5PA

SNAMTNIIKIRASVFIPMSWTEAKMDMETGQVIQFEGDSREFTPHAVNTMRSRVEQEVVVDFYKQ
EVFSYANTGITTEKVISPDGSVNKRTGKASTENIVCTDIVWNSGGVQFKMSASASNPLNVYAPPV
DYVLNVCVKKDGSIDVQGEHDGFPCFEFYKQVDFGPFEKIYTHDFRETGDTAAALGGNMDYSFTK
RL
>3NQIA
GMDSGESGPQQWAGVVKVNDRMGYVTFTDAAGTELIPTNTIPVTLNARMAYIYCQVDEGQDLSTN
PKSIKITLLADPTGIDATAITTPKVGESGDVTTNAPVGSLSFVSGYSTVAPFQFSENTIVLPVLY
RVKNVTTTEDIKNELAKHTFTLVCYTDDIKSGDTILKLYLRYKVEDEPAAIAERATRTSSFKAYE
ISQILREYTLKSGQTKPAKITIVAQQNEYNNKLEDTSTIEKVYEIEYKTAE
>1JYHA
MNYEIKQEEKRTVAGFHLVGPWEQTVKKGFEQLMMWVDSKNIVPKEWVAVYYDNPDETPAEKLRC
DTVVTVPGYFTLPENSEGVILTEITGGQYAVAVARVVGDDFAKPWYQFFNSLLQDSAYEMLPKPC
FEVYLNNGAEDGYWDIEMYVAVQPKHH
>1H97A
TLTKHEQDILLKELGPHVDTPAHIVETGLGAYHALFTAHPQYISHFSRLEGHTIENVMQSEGIKH
YARTLTEAIVHMLKEISNDAEVKKIAAQYGKDHTSRKVTKDEFMSGEPIFTKYFQNLVKDAEGKA
AVEKFLKHVFPMMAAEI
>3GRAA
MSLAPYRVDFILLEHFSMASFTVAMDVLVTANLLRADSFQFTPLSLDGDRVLSDLGLELVATELS
AAALKELDLLVVCGGLRTPLKYPELDRLLNDCAAHGMALGGLWNGAWFLGRAGVLDDYGCSIHPE
QRASLSERSPQTRITPASFTLDRDRLSAASPNGAMELMLGLVRRLYGDGLAEGVEEILSFSGARE
GHHHHHH
>1QV9A
MTVAKAIFIKCGNLGTSMMMDMLLDERADREDVEFRVVGTSVKMDPECVEAAVEMALDIAEDFEP
DFIVYGGPNPAAPGPSKAREMLADSEYPAVIIGDAPGLKVKDEMEEQGLGYILVKPDAMLGARRE
FLDPVEMAIYNADLMKVLAATGVFRVVQEAFDELIEKAKEDEISENDLPKLVIDRNTLLEREEFE
NPYAMVKAMAALEIAENVADVSVEGCFVEQDKERYVPIVASAHEMMRKAAELADEARELEKSNDA
VLRTPHAPDGKVLSKRKFMEDPE
>3HTRA
SNAMTPETNETLKLIGSDKVQGTAVYGPDGEKIGSIERVMIEKVSGRVSYAVLSFGGFLGIGDDH
YPLPWPALKYNVELGGYQVMVTVDQLERAPKYGPGSEWDWRGARKVDDYYGVALT
>1ZZ1A
MAIGYVWNTLYGWVDTGTGSLAAANLTARMQPISHHLAHPDTKRRFHELVCASGQIEHLTPIAAV
AATDADILRAHSAAHLENMKRVSNLPTGGDTGDGITMMGNGGLEIARLSAGGAVELTRRVATGEL
SAGYALVNPPGHHAPHNAAMGFCIFNNTSVAAGYARAVLGMERVAILDWDVHHGNGTQDIWWNDP
SVLTISLHQHLCFPPDSGYSTERGAGNGHGYNINVPLPPGSGNAAYLHAMDQVVLPALRAYRPQL
IIVGSGFDASMLDPLARMMVTADGFRQMARRTIDCAADICDGRIVFVQEGGYSPHYLPFCGLAVI
EELTGVRSLPDPYHEFLAGMGGNTLLDAERAAIEEIVPLLADIR
>2GUFA
QDTSPDTLVVTANRFEQPRSTVLAPTTVVTRQDIDRWQSTSVNDVLRRLPGVDITQNGGSGQLSS
IFIRGTNASHVLVLIDGVRLNLAGVSGSADLSQFPIALVQRVEYIRGPRSAVYGSDAIGGVVNII
TTRDEPGTEISAGWGSNSYQNYDVSTQQQLGDKTRVTLLGDYAHTHGYDVVAYGNTGTQAQTDND
GFLSKTLYGALEHNFTDAWSGFVRGYGYDNRTNYDAYYSPGSPLLDTRKLYSQSWDAGLRYNGEL
IKSQLITSYSHSKDYNYDPHYGRYDSSATLDEMKQYTVQWANNVIVGHGSIGAGVDWQKQTTTPG

TGYVEDGYDQRNTGIYLTGLQQVGDFTFEGAARSDDNSQFGRHGTWQTSAGWEFIEGYRFIASYG
TSYKAPNLGQLYGFYGNPNLDPEKSKQWEGAFEGLTAGVNWRISGYRNDVSDLIDYDDHTLKYYN
EGKARIKGVEATANFDTGPLTHTVSYDYVDARNAITDTPLLRRAKQQVKYQLDWQLYDFDWGITY
QYLGTRYDKDYSSYPYQTVKMGGVSLWDLAVAYPVTSHLTVRGKIANLFDKDYETVYGYQTAGRE
YTLSGSYTF
>4GJZA
STVPRLHRPSLQHFREQFLVPGRPVILKGVADHWPCMQKWSLEYIQEIAGCRTVPVEVGSRYTDE
EWSQTLMTVNEFISKYIVNEPRDVGYLAQHQLFDQIPELKQDISIPDYCSLGDGEEEEITINAWF
GPQGTISPLHQDPQQNFLVQVMGRKYIRLYSPQESGALYPHDTHLLHNTSQVDVENPDLEKFPKF
AKAPFLSCILSPGEILFIPVKYWHYVRALDLSFSVSFWWS
>3A57A
FELPSVPFPAPGSDEILFVVRDTTFNTNAPVNVEVSDFWTNRNVKRKPYKDVYGQSVFTTSGTKW
LTSYMTVNINDKDYTMAAVSGYKHGHSAVFVKSDQVQLQHSYDSVASFVGEDEDSIPSKMYLDET
PEYFVNVEAYESGSGNILVMCISNKESFFECKHQQ
>2Y7LA
RKTITGVFNSFDSLTWTRSVEYVYKGPETPTWNAVLGWSLNSTTADPGDTFTLILPCVFKFITTQ
TSVDLTADGVSYATCDFNAGEEFTTFSSLSCTVNSVSVSYARVSGTVKLPITFNVGGTGSSVDLA
DSKCFTAGKNTVTFMDGDTKISTTVDFDASPVSPSGYITSSRIIPSLNKLSSLFVVPQCENGYTS
GIMGFVASNGATIDCSNVNIGISKGLNDWNFPVSSESFSYTKTCTSTSITVEFQNVPAGYRPFVD
AYISAENIDKYTLTYANEYTCENGNTVVDPFTLTWWGYKNSEADSDGDVIVV
>2W39A
ATYHLEDNWVGSAFLSTFTHEAIADPTHGRVNYVDQATALAKNLTYASGDTLILRADHTTTLSPS
GPGRNSVRIRSIKTYTTHVAVFDVRHMPQGCGTWPAAWETDEGDWPNGGEVDIIEGVNDQSPNAM
TLHTGANCAMPASRTMTGHATNNNCDVNTDGNTGCGVQAPTANSYGPSFNANGGGWYAMERTNSF
IKVWFFPRNAGNVPNDIASGPATINTDNWGTPTAFFPNTNCDIGSHFDANNIIINLTFCGDWAGQ
ASIFNGAGCPGSCVDYVNNNPSAFANAYWDIASVRVYQ
>1O8BA
MTQDELKKAVGWAALQYVQPGTIVGVGTGSTAAHFIDALGTMKGQIEGAVSSSDASTEKLKSLGI
HVFDLNEVDSLGIYVDGADEINGHMQMIKGGGAALTREKIIASVAEKFICIADASKQVDILGKFP
LPVEVIPMARSAVARQLVKLGGRPEYRQGVVTDNGNVILDVHGMEILDPIAMENAINAIPGVVTV
GLFANRGADVALIGTPDGVKTIVK
>2CARA
GSMAASLVGKKIVFVTGNAKKLEEVVQILGDKFPCTLVAQKIDLPEYQGEPDEISIQKCQEAVRQ
VQGPVLVEDTCLCFNALGGLPGPYIKWFLEKLKPEGLHQLLAGFEDKSAYALCTFALSTGDPSQP
VRLFRGRTSGRIVAPRGCQDFGWDPCFQPDGYEQTYAEMPKAEKNAVSHRFRALLELQEYFGSLA
A
>3A77A
GAMENPLKRLLVPGEEWEFEVTAFYRGRQVFQQTISCPEGLRLVGSEVGDRTLPGWPVTLPDPGM
SLTDRGVMSYVRHVLSCLGGGLALWRAGQWLWAQRLGHCHTYWAVSEELLPNSGHGPDGEVPKDK
EGGVFDLGPFIVDLITFTEGSGRSPRYALWFCVGESWPQDQPWTKRLVMVKVVPTCLRALVEMAR
VGGASSLENTVDLHISNSHPLSLTSDQYKAYLQDLVEGMDFQGPGES
>3DR5A
MSNAFEYLRTYVESTTETDAAVARAREDAAEFGLPAPDEMTGQLLTTLAATTNGNGSTGAIAITP
AAGLVGLYILNGLADNTTLTCIDPESEHQRQAKALFREAGYSPSRVRFLLSRPLDVMSRLANDSY

QLVFGQVSPMDLKALVDAAWPLLRRGGALVLADALLDGTIADQTRKDRDTQAARDADEYIRSIEG
AHVARLPLGAGLTVVTKALEHHHHHH
>2CH5A
PQFMAAIYGGVEGGGTRSEVLLVSEDGKILAEADGLSTNHWLIGTDKCVERINEMVNRAKRKAGV
DPLVPLRSLGLSLSGGDQEDAGRILIEELRDRFPYLSESYLITTDAAGSIATATPDGGVVLISGT
GSNCRLINPDGSESGCGGWGHMMGDEGSAYWIAHQAVKIVFDSIDNLEAAPHDIGYVKQAMFHYF
QVPDRLGILTHLYRDFDKCRFAGFCRKIAEGAQQGDPLSRYIFRKAGEMLGRHIVAVLPEIDPVL
FQGKIGLPILCVGSVWKSWELLKEGFLLALTQGREIQAQNFFSSFTLMKLRHSSALGGASLGARH
IGHLLPMDYSANAIAFYSYTFS
>3CTZA
MPPKVTSELLRQLRQAMRNSEYVTEPIQAYIIPSGDAHQSEYIAPCDCRRAFVSGFDGSAGTAII
TEEHAAMWTDGRYFLQAAKQMDSNWTLMKMGLKDTPTQEDWLVSVLPEGSRVGVDPLIIPTDYWK
KMAKVLRSAGHHLIPVKENLVDKIWTDRPERPCKPLLTLGLDYTGISWKDKVADLRLKMAERNVM
WFVVTALDEIAWLFNLRGSDVEHNPVFFSYAIIGLETIMLFIDGDRIDAPSVKEHLLLDLGLEAE
YRIQVHPYKSILSELKALCADLSPREKVWVSDKASYAVSETIPKDHRCCMPYTPICIAKAVKNSA
ESEGMRRAHIKDAVALCELFNWLEKEVPKGGVTEISAADKAEEFRRQQADFVDLSFPTISSTGPT
GAIIHYAPVPETNRTLSLDEVYLIDSGAQYKDGTTDVTRTMHFETPTAYEKECFTYVLKGHIAVS
AAVFPTGTKGHLLDSFARSALWDSGLDYLHGTGHGVGSFLNVHEGPCGISYKTFSDEPLEAGMIV
TDEPGYYEDGAFGIRIENVVLVVPVKTKYNFNNRGSLTLEPLTLVPIQTKMIDVDSLTDKECDWL
NNYHLTCRDVIGKELQKQGRQEALEWLIRETQPISKQH
>1T77A
GPVSLSTPAQLVAPSVVVKGTLSVTSSELYFEVDEEDPNFKKIDPKILAYTEGLHGKWLFTEIRS
IFSRRYLLQNTALEIFMANRVAVMFNFPDPATVKKVVNFLPRVGVGTSFGLPQTRRISLASPRQL
FKASNMTQRWQHREISNFEYLMFLNTIAGRSYNDLNQYPVFPWVITNYESEELDLTLPTNFRDLS
KPIGALNPKRAAFFAERYESWEDDQVPKFHYGTHYSTASFVLAWLLRIEPFTTYFLNLQGGKFDH
ADRTFSSISRAWRNSQRDTSDIKELIPEFYYLPEMFVNFNNYNLGVMDDGTVVSDVELPPWAKTS
EEFVHINRLALESEFVSCQLHQWIDLIFGYKQQGPEAVRALNVFYYLTYEGAVNLNSITDPVLRE
AVEAQIRSFGQTPSQLLIEPHPPR
>3NKUA
GHMMSVNEEQFGSLYSDERDKPLLSPTAQKKFEEYQNKLANLSKIIRENEGNEVSPWQEWENGLR
QIYKEMIYDAFDALGVEMPKDMEVHFAGSLAKAQATEYSDLDAFVIVKNDEDIKKVKPVFDALNN
LCQRIFTASNQIYPDPIGINPSRLIGTPDDLFGMLKDGMVADVEATAMSILTSKPVLPRYELGEE
LRDKIKQEPSFSNMVSAK
>2D4XA
MSDDPIAASQAVVLSQAQAQNSQYALARTFATQKVSLEESVLSQVTTAIQTAQEKIVYAGNGTLS
DDDRASLATDLQGIRDQLMNLANSTDGNGRYIFAGYKTEAAPFDQATGGYHGGEKSVTQQVDSAR
TMVIGHTGAQIFNSITSNAVPEPDGSDSEKNLFVMLDTAIAALKTPVEGNNVEKEKAAAAIDKTN
RGLKNSLNNVLTVRAELGTQLSELSTLDSLGSDRALGQKLQMSNLVDVDWNSV
>2W68A
AAMALFDYNATGDTEFDSPAKQGWMQDNTNNGSGVLTNADGMPAWLVQGIGGRAQWTYSLSTNQH
AQASSFGWRMTTEMKVLSGGMITNYYANGTQRVLPIISLDSSGNLVVEFEGQTGRTVLATGTAAT
EYHKFELVFLPGSNPSASFYFDGKLIRDNIQPTASKQNMIVWGNGSSNTDGVAAYRDIKFEIQGD
>2JK9A
SMQELQGLDYCKPTRLDLLLDMPPVSYDVQLLHSWNNNDRSLNVFVKEDDKLIFHRHPVAQSTDA

IRGKVGYTRGLHVWQITWAMRQRGTHAVVGVATADAPLHSVGYTTLVGNNHESWGWDLGRNRLYH
DGKNQPSKTYPAFLEPDETFIVPDSFLVALDMDDGTLSFIVDGQYMGVAFRGLKGKKLYPVVSAV
WGHCEIRMRYLNGLDPE
>2F6EA
YYFEPNTAIGANGYKIIDNKNFYFRNGLPQIGVFKGPNGFEYFAPANTDANNIEGQAIRYQNRFL
HLLGNIYYFGNNSKAVTGWQTINGNMYYFMPDTAMAAAGGLFEIDGVIYFFGVDGVKAPGIY
>3GP6A
MNADEWMTTFRENIAQTWQQPEHYDLYIPAITWHARFAYDKEKTDRYNERPWGGGFGLSRWDEKG
NWHGLYAMAFKDSWNKWEPIAGYGWESTWRPLADENFHLGLGFTAGVTARDNWNYIPLPVLLPLA
SVGYGPVTFQMTYIPGTYNNGNVYFAWMRFQFL
>1L8WA
MRGSHHHHHHGSSQVADKDDPTNKFYQSVIQLGNGFLDVFTSFGGLVAEAFGFKSDPKKSDVKTY
FTTVAAKLEKTKTDLNSLPKEKSDISSTTGKPDSTGSVGTAVEGAIKEVSELLDKLVKAVKTAEG
ASSGTAAIGEVVADADAAKVADKASVKGIAKGIKEIVEAAGGSEKLKAVAAAKGENNKGAGKLFG
KAGAAAHGDSEAASKAAGAVSAVSGEQILSAIVTAADAAEQDGKKPEEAKNPIAAAIGDKDGGAE
FGQDEMKKDDQIAAAIALRGMAKDGKFAVKDGEKEKAEGAIKGAAESAVRKVLGAITGLIGDAVS
SGLRKVGDSVKAASKETPPALNK
>3EF2A
MSLRRGIYHIENAGVPSAIDLKDGSSSDGTPIVGWQFTPDTINWHQLWLAEPIPNVADTFTLCNL
FSGTYMDLYNGSSEAGTAVNGWQGTAFTTNPHQLWTIKKSSDGTSYKIQNYGSKTFVDLVNGDSS
DGAKIAGWTGTWDEGNPHQKWYFNRMSVSSAEAQAAIARNPHIHGTYRGYILDGEYLVLPNATFT
QIWKDSGLPGSKWREQIYDCDDFAIAMKAAVGKWGADSWKANGFAIFCGVMLGVNKAGDAAHAYN
FTLTKDHADIVFFEPQNGGYLNDIGYDSYMAFY
>2FREA
MTNSNNRQSEYPVDPLFLDRWSPRAFDGSPMPKEHLLTILDAAHWAPSASNHQPWRFVYAHKDSE
DWPLFVELLMEGNQKWAKNASVLLFVISRDHTISHEGEKKPSATHSFDAGAAWFSLAMQAHLLGY
HAHGMGGIFKDRIVEKLDIPDGFKVEAGVAIGTLTDKSILPDDLAEREVPSKRVPLADVAFEGRF
TGKAD
>2PRSA
AVVASLKPVGFIASAIADGVTETEVLLPDGASEHDYSLRPSDVKRLQNADLVVWVGPEMEAFMQK
PVSKLPGAKQVTIAQLEDVKPLLMKSIHGDDDDHDHAEKSDEDHHHGDFNMHLWLSPEIARATAV
AIHGKLVELMPQSRAKLDANLKDFEAQLASTETQVGNELAPLKGKGYFVFHDAYGYFEKQFGLTP
LGHFTVNPEIQPGAQRLHEIRTQLVEQKATCVFAEPQFRPAVVESVARGTSVRMGTLDPLGTNIK
LGKTSYSEFLSQLANQYASCLKGD
>3M9VA
GHMPPWTARQDSTTGLYAPVTPAGRVLLDRLAAHLPRIRSTAAEHDRDGTFPTDTFDALRKDGLM
GATVPAELGGLGVDRLYDVAVALLAVARADASTALALHMQLSRGLTLGYEWRHGDERARTLAERI
LRGMVAGDAVVCSGIKDHHTAVTTLRPDGAGGWLLSGRKTLVSMAPVGTHFVINARTDGTDGPPR
LASPVVTRDTPGFTVLDNWDGLGMRASGTVDIVFDDCPIPADHVLMRDPVGARNDAVLAGQTVSS
VSVLGVYVGVAQAAYDTAVAALERRPEPPQAAALTLVAEIDSRLYALRATAGSALTAADALSADL
SGDMDERGRQMMRHFQCAKLAVNRLAPEIVSDCLSLVGGASYTAGHPLARLLRDVQAGRFMQPYA
YVDAVDFLSAQALGIERDNNYMSTWAKRSGGNGKSADAAGPRRPTPTSR
>1PSWA
MKILVIGPSWVGDMMMSQSLYRTLQARYPQAIIDVMAPAWCRPLLSRMPEVNEAIPMPLGHGALE

IGERRKLGHSLREKRYDRAYVLPNSFKSALVPLFAGIPHRTGWRGEMRYGLLNDVRVLDKEAWPL
MVERYIALAYDKGIMRTAQDLPQPLLWPQLQVSEGEKSYTCNQFSLSSERPMIGFCPGAEFGPAK
RWPHYHYAELAKQLIDEGYQVVLFGSAKDHEAGNEILAALNTEQQAWCRNLAGETQLDQAVILIA
ACKAIVTNDSGLMHVAAALNRPLVALYGPSSPDFTPPLSHKARVIRLITGYHKVRKGDAAEGYHQ
SLIDITPQRVLEELNALLLQEEA
>3C8GA
SNAMATLTEDDVLEQLDAQDNLFSFMKTAHSILLQGIRQFLPSLFVDNDEEIVEYAVKPLLAQSG
PLDDIDVALRLIYALGKMDKWLYADITHFSQYWHYLNEQDETPGFADDITWDFISNVNSITRNAT
LYDALKAMKFADFAVWSEARFSGMVKTALTLAVTTTLKELTP
>4FMHA
GSHMLEGGVEVLSVVTGEDSITQIELYLNPRMGVNSPDLPTTSNWYTYTYDLQPKGSSPDQPIKE
NLPAYSVARVSLPMLNEDITCDTLQMWEAISVKTEVVGISSLINVHYWDMKRVHDYGAGIPVSGV
NYHMFAIGGEPLDLQGLVLDYQTQYPKTTNGGPITIETVLGRKMTPKNQGLDPQAKAKLDKDGNY
PIEVWCPDPSKNENSRYYGSIQTGSQTPTVLQFSNTLTTVLLDENGVGPLCKGDGLFISCADIVG
FLFKTSGKMALHGLPRYFNVTLRKRWVKN
>3MVNA
MHHHHHHSSGVDLGTENLYFQSNAQRRLEVKGVVNNITVYDDFAHHPTAITATIDALRAKVGQQR
ILAVLEPRSNTMKMGVHKHELATSLQDADSVFIYQPPTIEWQVSEVLANLAQPAISADDVDELVM
RIVQQAKPNDHILIMSNGAFGGIHQKLLTALAN
>3CJ1A
MAHHHHHHVGTGSNDDDDKSPDPTQDVREPPALKYGIVLDAGSSHTSMFVYKWPADKENDTGIVG
QHSSCDVQGGGISSYANDPSKAGQSLVRCLEQALRDVPRDRHASTPLYLGATAGMRLLNLTSPEA
TARVLEAVTQTLTQYPFDFRGARILSGQDEGVFGWVTANYLLENFIKYGWVGRWIRPRKGTLGAM
DLGGASTQITFETTSPSEDPGNEVHLRLYGQHYRVYTHSFLCYGRDQILLRLLASALQIHRFHPC
WPKGYSTQVLLQEVYQSPCTMGQRPRAFNGSAIVSLSGTSNATLCRDLVSRLFNISSCPFSQCSF
NGVFQPPVAGNFIAFSAFYYTVDFLTTVMGLPVGTLKQLEEATEITCNQTWTELQARVPGQKTRL
ADYCAVAMFIHQLLSRGYHFDERSFREVVFQKKAADTAVGWALGYMLNLTNLIPADLPGLRKGTH
F
>3OT1A
SNAMEQGMSKRILVPVAHGSEEMETVIIVDTLVRAGFQVTMAAVGDKLQVQGSRGVWLTAEQTLE
ACSAEAFDALALPGGVGGAQAFADSTALLALIDAFSQQGKLVAAICATPALVFAKQQKFVGARMT
CHPNFFDHIPSERLSRQRVCYYATQHLLTSQGPGTALEFALAMIALLAGVELAQHVAAPMVLHPQ
QLTELSGFIDAQS
>4DVCA
SNAAQFKEGEHYQVLKTPASSSPVVSEFFSFYCPHCNTFEPIIAQLKQQLPEGAKFQKNHVSFMG
GNMGQAMSKAYATMIALEVEDKMVPVMFNRIHTLRKPPKDEQELRQIFLDEGIDAAKFDAAYNGF
AVDSMVHRFDKQFQDSGLTGVPAVVVNNRYLVQGQSAKSLDEYFDLVNYLLTLK
>2Z5EA
MEDTPLVISKQKTEVVCGVPTQVVCTAFSSHILVVVTQFGKMGTLVSLEPSSVASDVSKPVLTTK
VLLGQDEPLIHVFAKNLVAFVSQEAGNRAVLLAVAVKDKSMEGLKALREVIRVCQVW
>1G5AA
SPNSQYLKTRILDIYTPEQRAGIEKSEDWRQFSRRMDTHFPKLMNELDSVYGNNEALLPMLEMLL
AQAWQSYSQRNSSLKDIDIARENNPDWILSNKQVGGVCYVDLFAGDLKGLKDKIPYFQELGLTYL
HLMPLFKCPEGKSDGGYAVSSYRDVNPALGTIGDLREVIAALHEAGISAVVDFIFNHTSNEHEWA

QRCAAGDPLFDNFYYIFPDRRMPDQYDRTLREIFPDQHPGGFSQLEDGRWVWTTFNSFQWDLNYS
NPWVFRAMAGEMLFLANLGVDILRMDAVAFIWKQMGTSCENLPQAHALIRAFNAVMRIAAPAVFF
KSEAIVHPDQVVQYIGQDECQIGYNPLQMALLWNTLATREVNLLHQALTYRHNLPEHTAWVNYVR
SHDDIGWTFADEDAAYLGISGYDHRQFLNRFFVNRFDGSFARGVPFQYNPSTGDCRVSGTAAALV
GLAQDDPHAVDRIKLLYSIALSTGGLPLIYLGDEVGTLNDDDWSQDSNKSDDSRWAHRPRYNEAL
YAQRNDPSTAAGQIYQDLRHMIAVRQSNPRFDGGRLVTFNTNNKHIIGYIRNNALLAFGNFSEYP
QTVTAHTLQAMPFKAHDLIGGKTVSLNQDLTLQPYQVMWLEIA
>1YQSA
ADLPAPDDTGLQAVLHTALSQGAPGAMVRVDDNGTIHQLSEGVADRATGRAITTTDRFRVGSVTK
SFSAVVLLQLVDEGKLDLDASVNTYLPGLLPDDRITVRQVMSHRSGLYDYTNDMFAQTVPGFESV
RNKVFSYQDLITLSLKHGVTNAPGAAYSYSNTNFVVAGMLIEKLTGHSVATEYQNRIFTPLNLTD
TFYVHPDTVIPGTHANGYLTPDEAGGALVDSTEQTVSWAQSAGAVISSTQDLDTFFSALMSGQLM
SAAQLAQMQQWTTVNSTQGYGLGLRRRDLSCGISVYGHTGTVQGYYTYAFASKDGKRSVTALANT
SNNVNVLNTMARTLESAFCGKPTT
>1P3CA
VVIGDDGRTKVANTRVAPYNSIAYITFGGSSCTGTLIAPNKILTNGHCVYNTASRSYSAKGSVYP
GMNDSTAVNGSANMTEFYVPSGYINTGASQYDFAVIKTDTNIGNTVGYRSIRQVTNLTGTTIKIS
GYPGDKMRSTGKVSQWEMSGSVTREDTNLAYYTIDTFSGNSGSAMLDQNQQIVGVHNAGYSNGTI
NGGPKATAAFVEFINYAKAQ
>2H5NA
MGLGRQSLNIMTFSGQELTAIIKMAKSMVMADGKIKPAEIAVMTREFMRFGILQDQVDLLLKASD
SIEASQAVALIARMDEERKKYVASYLGVIMASDGDIDDNELALWTLISTLCGLPTMTVMEAINNM
KNL
>3H3IA
GACDNDTEPGGTAVEKMAGDWWVTVNAFIDGKEVEDPFGAGHLQMSTYNTASNSETEMWLDDLGN
FWEYKLKVNVNYAARTFSTTGFVDNVTYESKVKITDGKVLEKAATTPSGMPADSIVYMVQFDDDE
DGLTYKVSGFRRTGFPADDF
>3GHAA
MNNKTEQGNDAVSGQPSIKGQPVLGKDDAPVTVVEFGDYKCPSCKVFNSDIFPKIQKDFIDKGDV
KFSFVNVMFHGKGSRLAALASEEVWKEDPDSFWDFHEKLFEKQPDTEQEWVTPGLLGDLAKSTTK
IKPETLKENLDKETFASQVEKDSDLNQKMNIQATPTIYVNDKVIKNFADYDEIKETIEKELKGKL
EHHHHHH
>1JZTA
MSTLKVVSSKLAAEIDKELMGPQIGFTLQQLMELAGFSVAQAVCRQFPLRGKTETEKGKHVFVIA
GPGNNGGDGLVCARHLKLFGYNPVVFYPKRSERTEFYKQLVHQLNFFKVPVLSQDEGNWLEYLKP
EKTLCIVDAIFGFSFKPPMREPFKGIVEELCKVQNIIPIVSVDVPTGWDVDKGPISQPSINPAVL
VSLTVPKPCSSHIRENQTTHYVGGRFIPRDFANKFGFEPFGYESTDQILKL
>3ACHA
MRGSHHHHHHRAVVEAPVEHAPIGKATLPSTFEDSTRQGWAWDATSGVQSALTIKDANESKAISW
EVKYPEVKPVDGWASAPRIMLGNVNTTRGNNKYLTFDFYLKPTQASKGSLTISLAFAPPSLGFWA
QATGDVNIPLSSLSKMKKTTDGLYHFQVKYDLDKINDGKVLTANTVLRDITIVVADGNSDFAGTM
YLDNIRFE
>3ER6A
MSLTNKKNLRVVALAPTGRYFASIISSLEILETAAEFAEFQGFMTHVVTPNNRPLIGRGGISVQP

TAQWQSFDFTNILIIGSIGDPLESLDKIDPALFDWIRELHLKGSKIVAIDTGIFVVAKAGLLQQN
KAVMHSYFAHLFGELFPEIMLMTEQKALIDGNVYLSSGPYSHSSVMLEIVEEYFGKHTRNLGNQF
LSTIESEGHHHHHH
>3BO6A
GPPQMSATNEDLKTNFHSLHNQMRQMPMSHFREALDAPDYSGMRQSGFFAMSQGFQLESHGGDVF
MHAHRENPQCKGDFAGDKFHISVQREQVPQAFQALSGLLFSVDSPIDKWKVTDMERVDQQSRVAV
GAQFTLYVKPDQENSQYSASSLHNTRQFIECLESRLSESGLMPGQYPESDVHPENWKYVSYRNEL
RSGRDGGEMQSQALREEPFYRLMAE
>2UVOA
ERCGEQGSNMECPNNLCCSQYGYCGMGGDYCGKGCQNGACWTSKRCGSQAGGATCTNNQCCSQYG
YCGFGAEYCGAGCQGGPCRADIKCGSQAGGKLCPNNLCCSQWGFCGLGSEFCGGGCQSGACSTDK
PCGKDAGGRVCTNNYCCSKWGSCGIGPGYCGAGCQSGGCDG
>3S6FA
GMTQRSLADIQFQTTLEGVTPAQLGGFFEGWPNPPTPETLWRILDRAAVFVLARTPDGQVIGFVN
ALSDGILAASIPLLEVQAGWRSLGLGSELMRRVLTELGDLYMVDLSCDDDVVPFYERLGLKRANA
MFLRRYDNQAGIPAE
>4ADIA
EEAFTYLCTAPGCATQTPVPVRLAGVRFESKIVDGGCFAPWDLEATGACICEIPTDVSCEGLGAW
VPTAPCARIWNGTQRACTFWAVNAYSSGGYAQLASYFNPGGSYYKQYHPTACEVEPAFGHSDAAC
WGFPTDTVMSVFALASYVQHPHKTVRVKFHTETRTVWQLSVAGVSCNVTTEHPFCNTPHGQLEVQ
VPPDPGDLVEYIMNYTGNQQSRWGLGSPNCHGPDWASPVCQRHSPDCSRLVGATPERPRLRLVDA
DDPLLRTAPGPGEVWVTPVIGSQARKCGLHIRAGPYGHATVEMPEWIHAHTTSDPWHPPGPLGLK
FKTVRPVALPRALAPPRNVRVTGCYQCGTPALVEGLAPGGGNCHLTVNGEDVGAFPPGKFVTAAL
LNTPPPYQVSCGGESDRASARVIDPAAQSFTGVVYGTHTTAVSETRFEDDDDKAGWSHPQFEKGG
GSGGGSGGGSWSHPQFEK
>4A4JA
AQTINLQLEGMDCTSCASSIERAIAKVPGVQSCQVNFALEQAVVSYHGETTPQILTDAVERAGYH
ARVL
>3QUFA
MGSSHHHHHHSSGRENLYFQGHGTAGGGKTKISFYSYFKDNQIGEVVKGFEKKNPDITLDVQYGQ
DPAQYISTLQTRLAGGKPPTIFNLTMDNRTDVMKSGAALDISGEDFLDGIDDTNFALFQQDGKTY
GMPVSAWVGAFFYNKDILKKAGYDKFPKTWDEFIEMGKKINSNGSTAFLEDFNTQIAGSFTGLLA
SYYGEQGKSGDLDADIWSGKSTFTKDWTPVFKRWEAAAKAGVIPQKSVGLSADQVKQEFVSGNLG
VMRSGPWDLPDLQKSDIDFGVAPFPAYSKEDGQWINGGPDQGFAIASRASDKEKAAAKKFLAYLN
SEEGLEAFTSAAGTLSLSSKYNAEPPAELKDVVDNYFKQNKFYWVNWPKSPTVMSTEGIAQQQKI
VQGQISAKDAAKALDAKWATLKGS
>3NVWC
DTVGRPLPHLAAAMQASGEAVYCDDIPRYENELFLRLVTSTRAHAKIKSIDVSEAQKVPGFVCFL
SADDIPGSNETGLFNDETVFAKDTVTCVGHIIGAVVADTPEHAERAAHVVKVTYEDLPAIITIED
AIKNNSFYGSELKIEKGDLKKGFSEADNVVSGELYIGGQDHFYLETHCTIAIPKGEEGEMELFVS
TQNAMKTQSFVAKMLGVPVNRILVRVKRMGGGFGGKETRSTLVSVAVALAAYKTGHPVRCMLDRN
EDMLITGGRHPFLARYKVGFMKTGTIVALEVDHYSNAGNSRDLSHSIMERALFHMDNCYKIPNIR
GTGRLCKTNLSSNTAFRGFGGPQALFIAENWMSEVAVTCGLPAEEVRWKNMYKEGDLTHFNQRLE
GFSVPRCWDECLKSSQYYARKSEVDKFNKENCWKKRGLCIIPTKFGISFTVPFLNQAGALIHVYT

DGSVLVSHGGTEMGQGLHTKMVQVASKALKIPISKIYISETSTNTVPNSSPTAASVSTDIYGQAV
YEACQTILKRLEPFKKKNPDGSWEDWVMAAYQDRVSLSTTGFYRTPNLGYSFETNSGNAFHYFTY
GVACSEVEIDCLTGDHKNLRTDIVMDVGSSLNPAIDIGQVEGAFVQGLGLFTLEELHYSPEGSLH
TRGPSTYKIPAFGSIPTEFRVSLLRDCPNKKAIYASKAVGEPPLFLGASVFFAIKDAIRAARAQH
TNNNTKELFRLDSPATPEKIRNACVDKFTTLCVTGAPGNCK
>3EA0A
SNAKRVFGFVSAKGGDGGSCIAANFAFALSQEPDIHVLAVDISLPFGDLDMYLSGNTHSQDLADI
SNASDRLDKSLLDTMVQHISPSLDLIPSPATFEKIVNIEPERVSDLIHIAASFYDYIIVDFGASI
DHVGVWVLEHLDELCIVTTPSLQSLRRAGQLLKLCKEFEKPISRIEIILNRADTNSRITSDEIEK
VIGRPISKRIPQDEDAMQESLLSGQSVLKVAPKSQLSKTIVDWALHLNGV
>1B5EA
MISDSMTVEEIRLHLGLALKEKDFVVDKTGVKTIEIIGASFVADEPFIFGALNDEYIQRELEWYK
SKSLFVKDIPGETPKIWQQVASSKGEINSNYGWAIWSEDNYAQYDMCLAELGQNPDSRRGIMIYT
RPSMQFDYNKDGMSDFMCTNTVQYLIRDKKINAVVNMRSNDVVFGFRNDYAWQKYVLDKLVSDLN
AGDSTRQYKAGSIIWNVGSLHVYSRHFYLVDHWWKTGETHISKKDYVGKYA
>2P51A
GMNSNFSYPALGVDGISSQISPIRDVWSTNLQQEMNLIMSLIERYPVVSMDTEFPGVVARPLGVF
KSSDDYHYQTLRANVDSLKIIQIGLALSDEEGNAPVEACTWQFNFTFNLQDDMYAPESIELLTKS
GIDFKKHQEVGIEPADFAELLIGSGLVLQEEVTWITFHSGYDFAYLLKAMTQIPLPAEYEEFYKI
LCIYFPKNYDIKYIMKSVLNNSKGLQDIADDLQIHRIGPQHQAGSDALLTARIFFEIRSRYFDGS
IDSRMLNQLYGLGSTGSVLWHNNSSTPQIQFRDLPGAHPSPTPSNAGIPTTLTNTSSAPNFANST
FRFPPRVV
>3FPWA
GAGAMSSRKKPSRRTRVLVGGAALAVLGAGVVGTVAANAADTTEATPAAAPVAARGGELTQSTHL
TLEAATKAARAAVEAAEKDGRHVSVAVVDRNGNTLVTLRGDGAGPQSYESAERKAFTAVSWNAPT
SELAKRLAQAPTLKDIPGTLFLAGGTPVTAKGAPVAGIGVAGAPSGDLDEQYARAGAAVLGH
>1Y43A
EEYSSNWAGAVLIGDGYTKVTGEFTVPSVSAGSSGSSGY
>1HI9A
MKLYMSVDMEGISGLPDDTFVDSGKRNYERGRLIMTEEANYCIAEAFNSGCTEVLVNDSHSKMNN
LMVEKLHPEADLISGDVKPFSMVEGLDDTFRGALFLGYHARASTPGVMSHSMIFGVRHFYINDRP
VGELGLNAYVAGYYDVPVLMVAGDDRAAKEAEELIPNVTTAAVKQTISRSAVKCLSPAKRGRLLT
EKTAFALQNKDKVKPLTPPDRPVLSIEFANYGQAEWANLMPGTEIKTGTTTVQFQAKDMLEAYQA
MLVMTELAMRTSFC
>4KV7A
SNAAPKSLDATPVDAQLLKFGMSTALSGPAAELGINMRHGILAAFDEAKAKNHLPSKTLKLIALD
DGYEPARTAPNMHRLTDEHEVLAVVGNVGTPTAITAIPIAQQTKTPFFGAFTGASALRKTESVEF
VINYRASYAEETAAMVDALVAKGIKPEEIGFFTQNDSYGDDGFFGGLAAIRRHQSVKVSSLPHGR
YRRNTSQVEDGLADLLMHQPLPKAVIMVGTYEPCSKLIRMARMNNFNPQFLAVSFVGADALQRSL
GDLANGIVATQVVPHFDSDLPLVREYRDAMRDYDPELPLSFVSLEGYIVGRILVKAVTSIKGEIS
RSSIAAALEQLGQFDIGLGAPLTLGPNDHQASSKVWPVLIGADSSQSLAWEELLSE
>1LSHB
KYVPQRKPQTSRRHTPASSSSSSSSSSSSSSSSSSSSDSDMTVSAESFEKHSKPKVVIVLRAVRAD
GKQQGLQTTLYYGLTSNGLPKAKIVAVELSDLSVWKLCAKFRLSAHMKAKAAIGWGKNCQQYRAM

LEASTGNLQSHPAARVDIKWGRLPSSLQRAKNALLENGAPVIASKLEMEIMPKANQKHQVSVILA
AMTPRRMNIIVKLPKVTYFQQGILLPFTFPSPRFWDRPEGSQSDSLPAQIASAFSGIVQDPVASA
CELNEQSLTTFNGAFFNYDMPESCYHVLAQECSSRPPFIVLIKLDSERRISLELQLDDK
>3F95A
MVNSDSHPLFVRSLAKNMTWQLADTSTQKVLASGASATSGDKQSLLMQSVNLSYQEDGRGFNWRA
QAALSLSYLEPTPLDSKFSTGYLELKMRIDKAPEQGANLQVMCSESNCLRDIDFSSFSQLMADKS
WHTLAIPLHCDDSDQAEQPITDALRITSQNLSLAIADVALTIKPSDDSISLTCAKLEHHHHHH
>3FILA
MQYKLILNGKTLKGVLTIEAVDAATAEKVFKQYANDLGVDGEWTYDDATKTFTVTE
>2GUDA
XSLTHRKFGGSGGSPFSGLSSIAVRSGSYLDAIIIDGVHHGGSGGNLSPTFTFGSGEYISNMTIR
SGDYIDNISFETNMGRRFGPYGGSGGSANTLSNVKVIQINGSAGDYLDSLDIYYEQY
>3P0YA
RKVCNGIGIGEFKDSLSINATNIKHFKNCTSISGDLHILPVAFRGDSFTHTPPLDPQELDILKTV
KEITGFLLIQAWPENRTDLHAFENLEIIRGRTKQHGQFSLAVVSLNITSLGLRSLKEISDGDVII
SGNKNLCYANTINWKKLFGTSGQKTKIISNRGENSCKATGQVCHALCSPEGCWGPEPRDCVSCRN
VSRGRECVDKGNSHHHHHH
>3VE2A
GSKDQGGYGFAMRLKRRNWYPGAEESEVKLNESDWEATGLPTKPKELPKRQKSVIEKVETDGDSD
IYSSPYLTPSNHQNGSAGNGVNQPKNQATGHENFQYVYSGWFYKHAASEKDFSNKKIKSGDDGYI
FYHGEKPSRQLPASGKVIYKGVWHFVTDTKKGQDFREIIQPSKKQGDRYSGFSGDGSEEYSNKNE
STLKDDHEGYGFTSNLEVDFGNKKLTGKLIRNNASLNNNTNNDKHTTQYYSLDAQITGNRFNGTA
TATDKKENETKLHPFVSDSSSLSGGFFGPQGEELGFRFLSDDQKVAVVGSAKTKDKLENGAAASG
STGAAASGGAAGTSSENSKLTTVLDAVELTLNDKKIKNLDNFSNAAQLVVDGIMIPLLPKDSESG
NTQADKGKNGGTEFTRKFEHTPESDKKDAQAGTQTNGAQTASNTAGDTNGKTKTYEVEVCCSNLN
YLKYGMLTRKNSKSAMQAGGNSSQADAKTEQVEQSMFLQGERTDEKEIPTDQNVVYRGSWYGHIA
NGTSWSGNASDKEGGNRAEFTVNFADKKITGKLTAENRQAQTFTIEGMIQGNGFEGTAKTAESGF
DLDQKNTTRTPKAYITDAKVKGGFYGPKAEELGGWFAYPGDKQTEKATATSSDGNSASSATVVFG
AKRQQPVQ
>4A27A
SMEMRAVVLAGFGGLNKLRLFRKAMPEPQDGELKIRVKACGLNFIDLMVRQGNIDNPPKTPLVPG
FECSGIVEALGDSVKGYEIGDRVMAFVNYNAWAEVVCTPVEFVYKIPDDMSFSEAAAFPMNFVTA
YVMLFEVANLREGMSVLVHSAGGGVGQAVAQLCSTVPNVTVFGTASTFKHEAIKDSVTHLFDRNA
DYVQEVKRISAEGVDIVLDCLCGDNTGKGLSLLKPLGTYILYGSSNMVTGETKSFFSFAKSWWQV
EKVNPIKLYEENKVIAGFSLLNLLFKQGRAGLIRGVVEKLIGLYNQKKIKPVVDSLWALEEVKEA
MQRIHDRGNIGKLILDVEKTPTPL
>3MMHA
MHALHFSASDKAALYREVLPQIESVVADETDWVANLANTAAVLKEAFGWFWVGFYLVDTRSDELV
LAPFQGPLACTRIPFGRGVCGQAWAKGGTVVVGDVDAHPDHIACSSLSRSEIVVPLFSDGRCIGV
LDADSEHLAQFDETDALYLGELAKILEKRFEASRQAV
>2IZWA
ARKKGKSASQVIVLKEKSRKKRQKSRGQQPTRQVTPVSAPAAMGTQITYRGPQVVTQYGDITPAK
NSGSLVRVTSSATAGTEVSGTVLFNVRNATELPWLSGQGSRYSKYRVRYAHFTWEPIVGSNTNGE
VAMAMLYDVADVTSITIERLMQTRGGTWGPIWSPTRKRLSYDPEHASLPWYLSGVSSGAAAGNIQ

TPFQIAWAAQSSLVSTTLGRIMAEYLVELTDPVDVTINQ
>3DXTA
METMKSKANCAQNPNCNIMIFHPTKEEFNDFDKYIAYMESQGAHRAGLAKIIPPKEWKARETYDN
ISEILIATPLQQVASGRAGVFTQYHKKKKAMTVGEYRHLANSKKYQTPPHQNFEDLERKYWKNRI
YNSPIYGADISGSLFDENTKQWNLGHLGTIQDLLEKECGVVIEGVNTPYLYFGMWKTTFAWHTED
MDLYSINYLHLGEPKTWYVVPPEHGQRLERLARELFPGSSRGCGAFLRHKVALISPTVLKENGIP
FNRITQEAGEFMVTFPYGYHAGFNHGFNCAEAINFATPRWIDYGKMASQCSCGEARVTFSMDAFV
RILQPERYDLWKRGQDRAVVDHMEPRVPA
>2OL5A
MYIPKHFAVNDPDVAYQVIEENSFATLVSMHQRELFATHLPLLLDREKTCLYGHFARSNPQWNDI
QHQTVLAIFHGPHCYISPSWYETNQAVPTWNYVAVHVYGNVELINDQGEVMQSLHDMVEKYEAPG
SRYQLSEVDAGMLSGMNKGIQAFKIIIKRIEGKAKLSQNHPAHRQERIIKQLEQMPFENEKRIAS
LMKKQRQ
>4HTGA
CVAVEQKTRTAIIRIGTRGSPLALAQAYETREKLKKKHPELVEDGAIHIEIIKTTGDKILSQPLA
DIGGKGLFTKEIDEALINGHIDIAVHSMKDVPTYLPEKTILPCNLPREDVRDAFICLTAATLAEL
PAGSVVGTASLRRKSQILHKYPALHVEENFRGNVQTRLSKLQGGKVQATLLALAGLKRLSMTENV
ASILSLDEMLPAVAQGAIGIACRTDDDKMATYLASLNHEETRLAISCERAFLETLDGSCRTPIAG
YASKDEEGNCIFRGLVASPDGTKVLETSRKGPYVYEDMVKMGKDAGQELLSRAGPGFFGN
>4I0XA
SIDEVGALSKFAASLADQMRAGSNSLDRDVQSLFGVWKGSAADAYRSGWDEMQDGATKVWNALTD
IASTLGSNAAAFHAQETSTASSITSTQAD
>1KJNA
MKTESTGKALMVLGCPESPVQIPLAIYTSHKLKKKGFRVTVTANPAALRLVQVADPEGIYTDEMV
DLESCINELAEGDYEFLAGFVPNDAAAAYLVTFAGILNTETLAIIFDRDADVLEELVNEIMETLD
AEIIAARAHHNPAPLRVRIDRFMEEKP
>3VGIA
MSKKKFVIVSILTILLVQAIYFVEKYHTSEDKSTSNTSSTPPQTTLSTTKVLKIRYPDDGEWPGA
PIDKDGDGNPEFYIEINLWNILNATGFAEMTYNLTSGVLHYVQQLDNIVLRDRSNWVHGYPEIFY
GNKPWNANYATDGPIPLPSKVSNLTDFYLTISYKLEPKNGLPINFAIESWLTREAWRTTGINSDE
QEVMIWIYYDGLQPAGSKVKEIVVPIIVNGTPVNATFEVWKANIGWEYVAFRIKTPIKEGTVTIP
YGAFISVAANISSLPNYTELYLEDVEIGTEFGTPSTTSAHLEWWITNITLTPLDRPLIS
>3GYCA
GAKSEMAFAGKGEISPRAITMWDFSWLERRWPGAGYEDWDQVLDELSERGYNAIRIDAYPHLIAE
NPMKKWLLKEVWNQQDWGSPDMNEVQVQPNLNLFLSKCKERDIKVGLSSWYRLDVDEVCLKLDTP
EKLADCWLTILRSIEEDGLLDTILYVDLCNEWPGDSWAPFFAKTYPNVGWGNWYKEESLRWMKTS
LEKMRQVYPDMPFLYSFDHGDVKKYEEVDCSFLDLYEHHIWMAQQNGGEFYKLVGYGYNRFLPDD
YKNVVKNAERVYRERPGYWQKLLTDKIELMASVARKNRRPLVTTECWGLVDYKDWPLLKWDWVKD
LCELGTITAARTGMWVGVATSNFCGPQFAGMWRDVEWHKRLTSIIRSSPLDESLTKNNEVAAKLL
KRL
>3FO5A
SMRPQPGDGERRYREASARKKIRLDRKYIVSCKQTEVPLSVPWDPSNQVYLSYNNVSSLKMLVAK
DNWVLSSEISQVRLYTLEDDKFLSFHMEMVVHVDAAQAFLLLSDLRQRPEWDKHYRSVELVQQVD
EDDAIYHVTSPALGGHTKPQDFVILASRRKPCDNGDPYVIALRSVTLPTHRETPEYRRGETLCSG

FCLWREGDQLTKVSYYNQATPGVLNYVTTNVAGLSSEFYTTFKACEQFLLDNRNDLAPSLQTL
>2P26A
QECTKFKVSSCRECIESGPGCTWCQKLNFTGPGDPDSIRCDTRPQLLMRGCAADDIMDPTSLAET
QEDHNGGQKQLSPQKVTLYLRPGQAAAFNVTFRRAKLSSRVFLDHNALPDTLKVTYDSFCSNGVT
HRNQPRGDCDGVQINVPITFQVKVTATECIQEQSFVIRALGFTDIVTVQVLPQCECRCRDQSRDR
SLCHGKGFLECGICRCDTGYIGKNCECQTQGRSSQELEGSCRKDNNSIICSGLGDCVCGQCLCHT
SDVPGKLIYGQYCEHHHHHH
>4HNOA
GSHMIKIGAHMPISKGFDRVPQDTVNIGGNSFQIFPHNARSWSAKLPSDEAATKFKREMKKHGID
WENAFCHSGYLINLASPKDDIWQKSVELLKKEVEICRKLGIRYLNIHPGSHLGTGEEEGIDRIVR
GLNEVLNNTEGVVILLENVSQKGGNIGYKLEQLKKIRDLVDQRDRVAITYDTCHGFDSGYDITKK
EGVEALLNEIESLFGLERLKMIHLNDSKYPLGAAKDRHERIGSGFIGEEGFAVFFSFKEIQEVPW
ILETPGGNEEHAEDIKKVFEIIEKFGIE
>3HRZA
ALYTLITPAVLRTDTEEQILVEAHGDSTPKQLDIFVHDFPRKQKTLFQTRVDMNPAGGMLVTPTI
EIPAKEVSTDSRQNQYVVVQVTGPQVRLEKVVLLSYQSSFLFIQTDKGIYTPGSPVLYRVFSMDH
NTSKMNKTVIVEFQTPEGILVSSNSVDLNFFWPYNLPDLVSLGTWRIVAKYEHSPENYTAYFDVR
KYVLPSFEVRLQPSEKFFYIDGNENFHVSITARYLYGEEVEGVAFVLFGVKIDDAKKSIPDSLTR
IPIIDGDGKATLKRDTFRSRFPNLNELVGHTLYASVTVMTESGSDMVVTEQSGIHIVASPYQIHF
TKTPKYFKPGMPYELTVYVTNPDGSPAAHVPVVSEAFHSMGTTLSDGTAKLILNIPLNAQSLPIT
VRTNHGDLPRERQATKSMTAIAYQTQGGSGNYLHVAITSTEIKPGDNLPVNFNVKGNANSLKQIK
YFTYLILNKGKIFKVGRQPRRDGQNLVTMNLHITPDLIPSFRFVAYYQVGNNEIVADSVWVDVKD
TCMGTLVVKGDNLIQMPGAAMKIKLEGDPGARVGLVAVDKAVYVLNDKYKISQAKIWDTIEKSDF
GCTAGSGQNNLGVFEDAGLALTTSTNLNTKQRSAAKCPQPAN
>3VSSA
QSGLQDGPEPTIHTQQAYAPEDDFTAKWTRADARQLQRMSDPTAPSRENSMPASVTMPTVPQDFP
DMSNEQVWVWDTWPLTDEDANQYSVNGWEIIFSLVADRNLGFDDRHVFAKIGYFYRPAGVPAAER
PENGGWTYGGLVFKEGVTGQIFEDQSFSHQTQWSGSARVSKNGEIKLFFTDVAFYRNSDGTNIKP
YDPRIALSVGKVKANKKGVTLTGFNKVTDLLQADGTYYQTGAQNEFFNFRDPFTFEDPAHPGETF
MVFEGNSAMQRETATCNEADLGYRQGDPYAETVDDVNASGATYQIGNVGLAKAKNKQLTEWEFLP
PILSANCVTDQTERPQIYFKDGKSYLFTISHRGTFAAGLDGPEGVYGFVGDGIRSDYQPLNGGSG
LALGNPTNLNFLGGQPFAPDFNQHPGHFQAYSHYVMPGGLVQSFIDTIGTHDDFVRGGTLAPTVK
MDIGVGGDPTKTAVDYSYGSEGLGGWADIPANKHLFTNGKF
>3RFRC
MSSTTSTAAGAAAEVESVVDLRGMWIGLAVLNVFYLIVRIYEQVFGWRAGLDSFAPEFQTYWMSI
LWTEIPLELVSGLGLAGYLWKTRDRNVDAVAPREEMRRLVVLVQWLVVYGIAIYWGASFFTEQDG
AWHMTVIRDTDFTPSHIIEFYMSYPIYSVIAVGAFFYAKTRIPYFAHGYSLAFLIVAIGPFMIIP
NVGLNEWGHTFWFMEELFVAPLHWGFVFFGWMALGVFGVVLQILGRIHALIGKEGVALLTE
>4EG0A
GPGSMSGIDPKRFGKVAVLFGGESAEREVSLTSGRLVLQGLRDAGIDAHPFDPAERPLSALKDEG
FVRAFNALHGGYGENGQIQGALDFYGIRYTGSGVLGSALGLDKFRTKLVWQQTGVPTPPFETVMR
GDDYAARATDIVAKLGLPLFVKPASEGSSVAVLKVKTADALPAALSEAATHDKIVIVEKSIEGGG
EYTACIAGDLDLPLIKIVPAGEFYDYHAKYVANDTQYLIPCGLPAEQETELKRIARRAFDVLGCT
DWGRADFMLDAAGNAYFLEVNTAPGMTDHSLPPKAARSIGIGYSELVVKVLSLTLND

>3VL9A

GPLGSASLQRRSDFCGQWDTATAGDFTLYNDLWGESAGTGSQCTGVDSYSGDTIAWHTSWSWSGG
SSSVKSYVNAALTFTPTQLNCISSIPTTWKWSYSGSSIVADVAYDTFLAETASGSSKYEIMVWLA
ALGGAGPISSTGSTIATPTIAGVNWKLYSGPNGDTTVYSFVADSTTESFSGDLNDFFTYLVDNEG
VSDELYLTTLEAGTEPFTGSNAKLTVSEYSISIE

>4DD5A

MGVMNMREVVIASAARTAVGSFGGAFKSVSAVELGVTAAKEAIKRANITPDMIDESLLGGVLTAG
LGQNIARQIALGAGIPVEKPAMTINIVCGSGLRSVSMASQLIALGDADIMLVGGAENMSMSPYLV
PSARYGARMGDAAFVDSMIKDGLSDIFNNYHMGITAENIAEQWNITREEQDELALASQNKAEKAQ
AEGKFDEEIVPVVIKGRKGDTVVDKDEYIKPGTTMEKLAKLRPAFKKDGTVTAGNASGINDGAAM
LVVMAKEKAEELGIEPLATIVSYGTAGVDPKIMGYGPVPATKKALEAANMTIEDIDLVEANEAFA
AQSVAVIRDLNIDMNKVNVNGGAIAIGHPIGCSGARILTTLLYEMKRRDAKTGLATLCIGGGMGT
TLIVKR

>1NP6A

AGKTMIPLLAFAAWSGTGKTTLLKKLIPALCARGIRPGLIKHTHHDMDVDKPGKDSYELRKAGAA
QTIVASQQRWALMTETPDEEELDLQFLASRMDTSKLDLILVEGFKHEEIAKIVLFRDGAGHRPEE
LVIDRHVIAVASDVPLNLDVALLDINDVEGLADFVVEWMQKQNG

>2Q3ZA

MAEELVLERCDLELETNGRDHHTADLCREKLVVRRGQPFWLTLHFEGRNYQASVDSLTFSVVTGP
APSQEAGTKARFPLRDAVEEGDWTATVVDQQDCTLSLQLTTPANAPIGLYRLSLEASTGYQGSSF
VLGHFILLFNAWCPADAVYLDSEEERQEYVLTQQGFIYQGSAKFIKNIPWNFGQFQDGILDICLI
LLDVNPKFLKNAGRDCSRRSSPVYVGRVGSGMVNCNDDQGVLLGRWDNNYGDGVSPMSWIGSVDI
LRRWKNHGCQRVKYGQCWVFAAVACTVLRCLGIPTRVVTNYNSAHDQNSNLLIEYFRNEFGEIQG
DKSEMIWNFHCWVESWMTRPDLQPGYEGWQALDPTPQEKSEGTYCCGPVPVRAIKEGDLSTKYDA
PFVFAEVNADVVDWIQQDDGSVHKSINRSLIVGLKISTKSVGRDEREDITHTYKYPEGSSEEREA
FTRANHLNKLAEKEETGMAMRIRVGQSMNMGSDFDVFAHITNNTAEEYVCRLLLCARTVSYNGIL
GPECGTKYLLNLTLEPFSEKSVPLCILYEKYRDCLTESNLIKVRALLVEPVINSYLLAERDLYLE
NPEIKIRILGEPKQKRKLVAEVSLQNPLPVALEGCTFTVEGAGLTEEQKTVEIPDPVEAGEEVKV
RMDLVPLHMGLHKLVVNFESDKLKAVKGFRNVIIGPA

>2XFRA

MEVNVKGNYVQVYVMLPLDAVSVNNRFEKGDELRAQLRKLVEAGVDGVMVDVWWGLVEGKGPKAY
DWSAYKQLFELVQKAGLKLQAIMSFHQCGGNVGDAVNIPIPQWVRDVGTRDPDIFYTDGHGTRNI
EYLTLGVDNQPLFHGRSAVQMYADYMTSFRENMKEFLDAGVIVDIEVGLGPAGEMRYPSYPQSHG
WSFPGIGEFICYDKYLQADFKAAAAAVGHPEWEFPNDVGQYNDTPERTQFFRDNGTYLSEKGRFF
LAWYSNNLIKHGDRILDEANKVFLGYKVQLAIKISGIHWWYKVPSHAAELTAGYYNLHDRDGYRT
IARMLKRHRASINFTCAEMRDSEQSSQAMSAPEELVQQVLSAGWREGLNVACENALPRYDPTAYN
TILRNARPHGINQSGPPEHKLFGFTYLRLSNQLVEGQNYANFKTFVDRMHANLPRDPYVDPMAPL
PRSGPEISIEMILQAAQPKLQPFPFQEHTDLPVGPTGGMGGQAEGPTCGMGGQVKGPTGGMGGQA
EDPTSGIGGELPATM

>1T2DA

MAPKAKIVLVGSGMIGGVMATLIVQKNLGDVVLFDIVKNMPHGKALDTSHTNVMAYSNCKVSGSN
TYDDLAGADVVIVTAGFTKAPGKSDKEWNRDDLLPLNNKIMIEIGGHIKKNCPNAFIIVVTNPVD
VMVQLLHQHSGVPKNKIIGLGGVLDTSRLKYYISQKLNVCPRDVNAHIVGAHGNKMVLLKRYITV
GGIPLQEFINNKLISDAELEAIFDRTVNTALEIVNLHASPYVAPAAAIIEMAESYLKDLKKVLIC

STLLEGQYGHSDIFGGTPVVLGANGVEQVIELQLNSEEKAKFDEAIAETKRMKALAHHHHHH
>3AQ2A
GSHMTVPTWQVRDLRRILRVSELSQHLRQARTDFRSTLSQLVYFNRSVVNPNEYDDEYLLSDQRL
TYVYVDEVTAQLCGLNRLLPSNSPAFGTVATAMPPWLLDPQEMNAILQQSCGQGGFVNYHHGPST
NGFFLAILMSQLFIRIRTDVIRGQGYGWYARQGNYVEEGEDNEGIENEEEEEETREFQLSDLIHY
PIVALGSCHLTR
>3OSXA
MRGSHHHHHHGSEGMQFDRGYLSPYFINKPESGSVELENPYILLVDKKISNIRELLPVLEGVAKA
SKPLVIIAEDVEGEALATLVVNNMRGIVKVASVKAPGFGDRRKAMLQDIATLTNGTVISEEIGLE
LEKATLEDLGQAKRVVINKDTTTIIDGVGEEGAIAARVTQIRQQIEESTSDYDREKLQERVAKLA
GGVKLN
>2FHZA
MTNKLFEHTVLYDSGDAFFELKGNASMKLSPKAAIEVCNEAAKKGLWILGIDGGHWLNPGFRIDS
SASWTYDMPEEYKSKIPENNRLAIENIKDDIENGYTAFIITLKM
>2YK4A
EPVNLIFCYTILQMKVAERIMAQHPGERFYVVLMSENRNEKYDYYFNQIKDKAEWAYFFHLPYGL
NKSFNFIPTMAELKVKAMLLPKVKRIYLASLEKVSIAAFLSTYPDAEIKTFDDGTINLIQSSSYL
GDEFSVNGTIKRNFARMMIGDWSIAKTRNASDEHYTIFKGLKNIMDDGRRKMTYLPLFDASELKA
GDETGGTVRILLGSPDKEMKEISEKAAKNFNIQYVAPHPRQTYGLSGVTTLNSPYVIEDYILREI
KKNPHTRYEIYTFFSGAALTMKDFPNVHVYALKPASLPEDYWLKPVYALFTQSGIPILTFDDKLV
PR
>3COVA
AMAIPAFHPGELNVYSAPGDVADVSRALRLTGRRVMLVPTMGALHEGHLALVRAAKRVPGSVVVV
SIFVNPMQFGAGGDLDAYPRTPDDDLAQLRAEGVEIAFTPTTAAMYPDGLRTTVQPGPLAAELEG
GPRPTHFAGVLTVVLKLLQIVRPDRVFFGEKDYQQLVLIRQLVADFNLDVAVVGVPTVREADGLA
MSSRNRYLDPAQRAAAVALSAALTAAAHAATAGAQAALDAARAVLDAAPGVAVDYLELRDIGLGP
MPLNGSGRLLVAARLGTTRLLDNIAIEIGTFAGTDRPDGYR
>2JKHL
RKLCSLDNGDCDQFCHEEQNSVVCSCARGYTLADNGKACIPTGPYPCGKQTLERR
>2PXXA
GSGYREVEYWDQRYQGAADSAPYDWFGDFSSFRALLEPELRPEDRILVLGCGNSALSYELFLGGF
PNVTSVDYSSVVVAAMQACYAHVPQLRWETMDVRKLDFPSASFDVVLEKGTLDALLAGERDPWTV
SSEGVHTVDQVLSEVSRVLVPGGRFISMTSAAPHFRTRHYAQAYYGWSLRHATYGSGFHFHLYLM
HKGGKLSVAQLALGAQILSP
>2E6FA
MMCLKLNLLDHVFANPFMNAAGVLCSTEEDLRCMTASSSGALVSKSCTSAPRDGNPEPRYMAFPL
GSINSMGLPNLGFDFYLKYASDLHDYSKKPLFLSISGLSVEENVAMVRRLAPVAQEKGVLLELNL
SCPNVPGKPQVAYDFEAMRTYLQQVSLAYGLPFGVKMPPYFDIAHFDTAAAVLNEFPLVKFVTCV
NSVGNGLVIDAESESVVIKPKQGFGGLGGKYILPTALANVNAFYRRCPDKLVFGCGGVYSGEDAF
LHILAGASMVQVGTALQEEGPGIFTRLEDELLEIMARKGYRTLEEFRGRVKTIE
>1V18B
LPDADTLLHFATESTPDGFSCSSSLSALSLDEPFIQKDVELRIMPPV
>1WSWA
MAKALIVYGSTTGNTEYTAETIARELADAGYEVDSRDAASVEAGGLFEGFDLVLLGCSTWGDDCI

ELQDDFIPLFDSLEETGAQGRKVACFGCGDSSYEYFCGAVDAIEEKLKNLGAEIVQDGLRIDGDP
RAARDDIVGWAHDVRGAI
>2RA9A
GQHTLKQFAADSALTTTTPLCSEVPLFDINALGDWTYLGTSLPAKFAKLFASILHCIDDEYFLIT
PVEKVRVQVEDAPLLIVDFERAQPHSLLNVSTSIGTLHHNVDIKQMKLTDDSVYLPLERGLWGKL
GRACYYNFVNEFNLSDLNEQ
>1GWYA
ALAGTIIAGASLTFQVLDKVLEELGKVSRKIAVGIDNESGGTWTALNAYFRSGTTDVILPEFVPN
TKALLYSGRKDTGPVATGAVAAFAYYMSSGNTLGVMFSVPFDYNWYSNWWDVKIYSGKRRADQGM
YEDLYYGNPYRGDNGWHEKNLGYGLRMKGIMTSAGEAKMQIKISR
>1PAQA
MSVNSIYTDREEIDSEFEDEDFEKEGIATVERAMENNHDLDTALLELNTLRMSMNVTYHEVRIAT
ITALLRRVYHFIATQTLGPKDAVVKVFNQWGLLFKRQAFDEEEYIDLMNIIMEKIVEQSFDKPDL
ILFSALVSLYDNDIIEEDVIYKWWDNVSTDPRYDEVKKLTVKWVEWLQNADEESSSEEE
>1YOVA
MKLMAQLGKLLKEQKYDRQLRLWGDHGQEALESAHVCLINATATGTEILKNLVLPGIGSFTIIDG
NQVSGEDAGNNFFLQRSSIGKNRAEAAMEFLQELNSDVSGSFVEESPENLLDNDPSFFCRFTVVV
ATQLPESTSLRLADVLWNSQIPLLICRTYGLVGYMRIIIKEHPVIESHPDNALEDLRLDKPFPEL
REHFQSYDLDHMEKKDHSHTPWIVIIAKYLAQWYSETNGRIPKTYKEKEDFRDLIRQGILKNENG
APEDEENFEEAIKNVNTALNTTQIPSSIEDIFNDDRCINITKQTPSFWILARALKEFVAKEGQGN
LPVRGTIPDMIADSGKYIKLQNVYREKAKKDAAAVGNHVAKLLQSIGQAPESISEKELKLLCSNS
AFLRVVRCRSLAEEYGLDTINKDEIISSMDNPDNEIVLYLMLRAVDRFHKQQGRYPGVSNYQVEE
DIGKLKSCLTGFLQEYGLSVMVKDDYVHEFCRYGAAEPHTIAAFLGGAAAQEVIKIITKQFVIFN
NTYIYSGMSQTSATFQL
>3FO3A
EPGENLKPVDAMQCFDCHTQIEDMHTVGKHATVNCVHCHDATEHVETASSRRMGERPVTRMDLEA
CATCHTAQFNSFVEVRHESHPRLEKATPTSRSPMFDKLIAGHGFAFEHAEPRSHAFMLVDHFVVD
RAYGGRFQFKNWQKVTDGMGAVRGAWTVLTDADPESSDQRRFLSQTATAANPVCLNCKTQDHILD
WAYMGDEHEAAKWSRTSEVVEFARDLNHPLNCFMCHDPHSAGPRVVRDGLINAVVDRGLGTYPHD
PVKSEQQGMTKVTFQRGREDFRAIGLLDTADSNVMCAQCHVEYNCNPGYQLSDGSRVGMDDRRAN
HFFWANVFDYKEAAQEIDFFDFRHATTGAALPKLQHPEAETFWGSVHERNGVACADCHMPKVQLE
NGKVYTSHSQRTPRDMMGQACLNCHAEWTEDQALYAIDYIKNYTHGKIVKSEYWLAKMIDLFPVA
KRAGVSEDVLNQARELHYDAHLYWEWWTAENSVGFHNPDQARESLMTSISKSKEAVSLLNDAIDA
QVASR
>2O5HA
SNAMRKLNNHDVHKRYQDRLEEDVEFTINYELPLSCLWSTIKDFSSDFEEKTEAFFILFKELLRR
GHLKLQRDGQIIGHTPEEWEQIFREVWPEYEIEPNPLPGYAPFDIGMWLTVEAPAYAVWIDPEDG
SEYWAG
>2XHGA
MGGSRSRKSDQGIIAGNVPLTPIQKWFFGKNFTNTGHWNQSSVLYRPEGFDPKVIQSVMDKIIEH
HDALRMVYQHENGNVVQHNRGLGGQLYDFFSYNLTAQPDVQQAIEAETQRLHSSMNLQEGPLVKV
ALFQTLHGDHLFLAIHHLVVDGISWRILFEDLATGYAQALAGQAISLPEKTDSFQSWSQWLQEYA
NEADLLSEIPYWESLESQAKNVSLPKDYEVTDCKQKSVRNMRIRLHPEETEQLLKHANQAYQTEI
NDLLLAALGLAFAEWSKLAQIVIHLEGHGREDIIEQANVARTVGWFTSQYPVLLDLKQTAPLSDY

IKLTKENMRKIPRKGIGYDILKHVTLPENRGSLSFRVQPEVTFNYLGQFDADMRTELFTRSPYSG
GNTLGADGKNNLSPESEVYTALNITGLIEGGELVLTFSYSSEQYREESIQQLSQSYQKHLLAIIA
HCLQSHHHHHH
>4AC7B
MSNNNYIVPGEYRVAEGEIEINAGREKTTIRVSNTGDRPIQVGSHIHFVEVNKELLFDRAEGIGR
RLNIPSGTAARFEPGEEMEVELTELGGNREVFGISDLTNGSVDNKELILQRAKELGYKGVE
>3PFEA
GMFKPQGLYDYICQQWQEEILPSLCDYIKIPNKSPHFDAKWEEHGYMEQAVNHIANWCKSHAPKG
MTLEIVRLKNRTPLLFMEIPGQIDDTVLLYGHLDKQPEMSGWSDDLHPWKPVLKNGLLYGRGGAD
DGYSAYASLTAIRALEQQGLPYPRCILIIEACEESGSYDLPFYIELLKERIGKPSLVICLDSGAG
NYEQLWMTTSLRGNLVGKLTVELINEGVHSGSASGIVADSFRVARQLISRIEDENTGEIKLPQLY
CDIPDERIKQAKQCAEILGEQVYSEFPWIDSAKPVIQDKQQLILNRTWRPALTVTGADGFPAIAD
AGNVMRPVTSLKLSMRLPPLVDPEAASVAMEKALTQNPPYNAKVDFKIQNGGSKGWNAPLLSDWL
AKAASEASMTYYDKPAAYMGEGGTIPFMSMLGEQFPKAQFMITGVLGPHSNAHGPNEFLHLDMVK
KLTSCVSYVLYSFSQKK
>1Y5HA
GIDPFTMTTARDIMNAGVTCVGEHETLTAAAQYMREHDIGALPICGDDDRLHGMLTDRDIVIKGL
AAGLDPNTATAGELARDSIYYVDANASIQEMLNVMEEHQVRRVPVISEHRLVGIVTEADIARHLP
EHA
>3V7PA
MRIIKPFAILTPQTIIQDKAVAFDKKIEAIDTVENLIKKYPNAAVEHDENSLLLPGFANPHLHLE
FSANKATLQYGDFIPWLYSVIRHREDLLPLCDGACLEQTLSSIIQTGTTAIGAISSYGEDLQACI
DSALKVVYFNEVIGSNAATADVMYASFLERFHQSKKHENERFKAAVAIHSPYSVHYILAKRALDI
AKKYGSLVSVHFMESRAEREWLDKGSGEFAKFFKEFLNQTRPVNDTKSFLELFKELHTLFVHMVW
ANEEEIQTIASYNAHIIHCPISNRLLGNGVLDLEKIKSIPYAIATDGLSSNYSLNMYEELKAALF
VHPNKEATTFAKELIIRATKAGYDALGFEGGEIAVGKDADMQLIDLPEGLTNVEDLYLHVILHTT
KPKKVYIQGEEHVREAENLYFQSHHHHHHWSHPQFEK
>2AXCA
MRGSRSGHGNGGGNSNSGGGSNSSVAAPMAFGFPALAAPGAGTLGISVSGEALSAAIADIFAALK
GPFKFSAWGIALYGILPSEIAKDDPNMMSKIVTSLPAETVTNVQVSTLPLDQATVSVTKRVTDVV
KDTRQHIAVVAGVPMSVPVVNAKPTRTPGVFHASFPGVPSLTVSTVKGLPVSTTLPRGITEDKGR
TAVPAGFTFGGGSHEAVIRFPKESGQKPVYVSVTDVLTPAQVKQRQDEEKRLQQEWNDAHPVEVA
ERRS
>3TMPA
GSHMGAGYNSEDEYEAAAARIEAMDPATVEQQEHWFEKALRDKKGFIIKQMKEDGACLFRAVADQ
VYGDQDMHEVVRKHCMDYLMKNADYFSNYVTEDFTTYINRKRKNNCHGNHIEMQAMAEMYNRPVE
VYQYSTGTSAVEPINTFHGIHQNEDEPIRVSYHRNIHYNSVVNPNKATIGVGLG
>1R31A
MSLDSRLPAFRNLSPAARLDHIGQLLGLSHDDVSLLANAGALPMDIANGMIENVIGTFELPYAVA
SNFQINGRDVLVPLVVEEPSIVAAASYMAKLARANGGFTTSSSAPLMHAQVQIVGIQDPLNARLS
LLRRKDEIIELANRKDQLLNSLGGGCRDIEVHTFADTPRGPMLVAHLIVDVRDAMGANTVNTMAE
AVAPLMEAITGGQVRLRILSNLADLRLARAQVRITPQQLETAEFSGEAVIEGILDAYAFAAVDPY
RAATHNKGIMNGIDPLIVATGNDWRAVEAGAHAYACRSGHYGSLTTWEKDNNGHLVGTLEMPMPV
GLVGGATKTHPLAQLSLRILGVKTAQALAEIAVAVGLAQNLGAMRALATEGIQRGHMALHARNIA

VVAGARGDEVDWVARQLVEYHDVRADRAVALLKQKRGQ
>2IAFA
IGIGPSSSHTVGPMLAANAFLQLLEQKNLFDKTQRVKVELYGSLALTGKGHGTDKAILNGLENKA
PETVDPASMIPRMHEILDSNLLNLAGKKEIPFHEATDFLFLQKELLPKHSNGMRFSAFDGNANLL
IEQVYYSIGGGFITTEEDFDK
>2V78A
MVDVIALGEPLIQFNSFNPGPLRFVNYFEKHVAGSELNFCIAVVRNHLSCSLIARVGNDEFGKNI
IEYSRAQGIDTSHIKVDNESFTGIYFIQRGYPIPMKSELVYYRKGSAGSRLSPEDINENYVRNSR
LVHSTGITLAISDNAKEAVIKAFELAKSRSLDTNIRPKLWSSLEKAKETILSILKKYDIEVLITD
PDDTKILLDVTDPDEAYRKYKELGVKVLLYKLGSKGAIAYKDNVKAFKDAYKVPVEDPTGAGDAM
AGTFVSLYLQGKDIEYSLAHGIAASTLVITVRGDNELTPTLEDAERFLNEFKT
>4I6MD
MDPQTLITKANKVSYYGNPTSKESWRYDWYQPSKVSSNVQQPQQQLGDMENNLEKYPFRYKTWLR
NQEDEKNLQRESCEDILDLKEFDRRILKKSLMTSHTKGDTSKATGAPSANQGDEALSVDDIRGAV
GNSEAIPGLSAGVNNDNTKESKDVKMN
>3C8IA
PKQVGNAQHLYDNYDLVPAMIAEVNPRDMVVMALVNTNVDPTLPPRWALATRNITAIPGIEGDTR
KVGTRIPAVAVTGQRSVGNQDSWDQISPMPIAWATPDSSVIARAESTIPSEQWTTLSKNLNKLDQ
VRETKFDLLEL
>3VUSA
QPWPHNGFVAISWHNVEDEAADQRFMSVRTSALREQFAWLRENGYQPVSIAQIREAHRGGKPLPE
KAVVLTFDDGYQSFYTRVFPILQAFQWPAVWAPVGSWVDTPADKQVKFGDELVDREYFATWQQVR
EVARSRLVELASHTWNSHYGIQANATGSLLPVYVNRAYFTDHARYETAAEYRERIRLDAVKMTEY
LRTKVEVNPHVFVWPYGEANGIAIEELKKLGYDMFFTLESGLANASQLDSIPRVLIANNPSLKEF
AQQIITVQ
>4AVAA
MDGIAELTGARVEDLAGMDVFQGCPAEGLVSLAASVQPLRAAAGQVLLRQGEPAVSFLLISSGSA
EVSHVGDDGVAIIARALPGMIVGEIALLRDSPRSATVTTIEPLTGWTGGRGAFATMVHIPGVGER
LLRTARQRLAAFVSPIPVRLADGTQLMLRPVLPGDRERTVHGHIQFSGETLYRRFMSARVPSPAL
MHYLSEVDYVDHFVWVVTDGSDPVADARFVRDETDPTVAEIAFTVADAYQGRGIGSFLIGALSVA
ARVDGVERFAARMLSDNVPMRTIMDRYGAVWQREDVGVITTMIDVPGPGELSLGREMVDQINRVA
RQVIEAVG
>3RPCA
SNAMTQYTHIRNATGKLTIKNTTFLIDPFLAPKDTYPGFEGTFNYQQRMPMVDLPLSMDDLLSNV
TAVVVTHTHLDHWDDTAINSIPKSLPIFVQNTADKELITSQGFIDVRIIFESLEFNGITLRKTGG
SHGTVEMYANPVLAPLAGDAMGVIFEAADEPTVYLVGDTVWTSDVEKALLRFDPNVIIMNTGYAQ
ILGFEDSIIMGTKDIGRMVVRKPEAKIIAVHMDTVNHTATSRKDVRKFIKGNNIESHVAVPEDGE
TITL
>4AC1X
DLPRLIVYFQTTHDSSNRPISMLPLITEKGIALTHLIVCSFHINQGGVVHLNDFPPDDPHFYTLW
NETITMKQAGVKVMGMVGGAAPGSFNTQTLDSPDSATFEHYYGQLRDAIVNFQLEGMDLDVEQPM
SQQGIDRLIARLRADFGPDFLITLAPVASALEDSSNLSGFSYTALQQTQGNDIDWYNTQFYSGFG
SMADTSDYDRIVANGFAPAKVVAGQLTTPEGAGWIPTSSLNNTIVSLVSEYGQIGGVMGWEYFNS
LPGGTAEPWEWAQIVTVILRPGL

>2FP8A
SLALSSPILKEILIEAPSYAPNSFTFDSTNKGFYTSVQDGRVIKYEGPNSGFVDFAYASPYWNKA
FCENSTDAEKRPLCGRTYDISYNLQNNQLYIVDCYYHLSVVGSEGGHATQLATSVDGVPFKWLYA
VTVDQRTGIVYFTDVSTLYDDRGVQQIMDTSDKTGRLIKYDPSTKETTLLLKELHVPGGAEVSAD
SSFVLVAEFLSHQIVKYWLEGPKKGTAEVLVKIPNPGNIKRNADGHFWVSSSEELDGNMHGRVDP
KGIKFDEFGNILEVIPLPPPFAGEHFEQIQEHDGLLYIGTLFHGSVGILVYDKKGNSFVSSH
>4GDHA
GSHMVKVCLFVADGTDEIEFSAPWGIFKRAEIPIDSVYVGENKDRLVKMSRDVEMYANRSYKEIP
SADDFAKQYDIAIIPGGGLGAKTLSTTPFVQQVVKEFYKKPNKWIGMICAGTLTAKTSGLPNKQI
TGHPSVRGQLEEGGYKYLDQPVVLEENLITSQGPGTAMLFGLKLLEQVASKDKYNAVYKSLSMP
>3ZW5A
MHHHHHHSSGVDLGTENLYFQSMLIRRLDHIVMTVKSIKDTTMFYSKILGMEVMTFKEDRKALCF
GDQKFNLHEVGKEFEPKAAHPVPGSLDICLITEVPLEEMIQHLKACDVPIEEGPVPRTGAKGPIM
SIYFRDPDRNLIEVSNY
>3P02A
GDEWTDEQFKQLISFKTQPGGWGVTDVHVRYANSAKYTYNLPVLVSGSTDNTDDRLVSFSLRDDT
LDILNFEKFGNRPELYFRELPQKYYSFPKELTIPAGQSHALLPIEFSLDGLDDSQKWALPLKVCE
DANGTYAVNPRKYYRTAVLRPILFNEFSGRFSGSSLLGTMAGESDIKFSSTEIKLNVVTDSIVFF
YAGQRTEDYEDRINYKVFLQFTGDKVDSKKDLYKMKIWAENEKLKFNSYSTPTYKVSSEMDATKT
YLKHTYIVISDIDFDFVDYTSVPNYEIEYNMKGGLSVSRDLDTRKPDEDQGSDSKWW
>1ZA7A
RVVQPVIVEPIASGQGRAIKAWTGYSVSKWTASCAAAEAKVTSAITISLPNELSSERNKQLKVGR
VLLWLGLLPSVSGTVKSCVTETQTTAAASFQVALAVADNSKDVVAAMYPEAFKGITLEQLAADLT
IYLYSSAALTEGDVIVHLEVEHVRPTFDDSFTPVY
>2XOCA
GAMDLLELSDVDSESSDISQPYVVCRQCPEYRRQAAQPPHCPAPEGEPGAPQALGDAPSTSVSLT
TAVQDYVCPLQGSHALCTCCFQPMPDRRVEREQDPRVAPQQCAVCLQPFCHLYWGCTRTGCYGCL
APFCELNLGDKCLDGVLNNNSYESDILKNYLATRGLTWKNMLTESLVALQRGVFLLSDYRVTGDT
VLCYCCGLRSFRELTYQYRQNIPASELPVAVTSRPDCYWGRNCRTQVKAHHAMKFNHICEQTRFK
N
>3N2NA
SMACYGGFDLYFILDKSGSVLHHWNEIYYFVEQLAHKFISPQLRMSFIVFSTRGTTLMKLTEDRE
QIRQGLEELQKVLPGGDTYMHEGFERASEQIYYENRQGYRTASVIIALTDGELHEDLFFYSEREA
NRSRDLGAIVYAVGVKDFNETQLARIADSKDHVFPVNDGFQALQGIIHSILKKSC
>4F1VA
MDINGGGATLPQALYQTSGVLTAGFAQYIGVGSGNGKAAFLNNDYTKFQAGVTNKNVHWAGSDSK
LSATELSTYASAKQPTWGKLIQVPSVGTSVAIPFNKSGSAAVDLSVQELCGVFSGRINTWDGISG
SGRTGPIVVVYRSESSGTTELFTRFLNAKCNAETGNFAVTTTFGTSFSGGLPAGAVAATGSQGVM
TALAAGDGRITYMSPDFAAPTLAGLDDATKVARVGKNVATNTQGVSPAAANVSAAIGAVPVPAAA
DRSNPDAWVPVFGPDNTAGVQPYPTSGYPILGFTNLIFSQCYADATQTTQVRDFFTKHYGASNNN
DAAITANAFVPLPTAWKATVRASFLTASNALSIGNTNVCNGIGRPLLEAAHHHHHH
>3R4IA
GMRALTPAEVLFDGEVPPAVLPACDHYAGSEKLMLKSLALQQQLGPVFDITLDCEDGAQVGREAQ
HAELVASLLGSEHDRFGRVGVRIHDFDHAHWRDDVRLILRAAKRAPAYITLPKIRHVHDAAEMVA

FIEATRRELGIAQPVPVQLLVETHGALTRVFDLAALPGVEALSFGLMDFVSAHDGAIPDTAMRSP
GQFDHPLVRRAKLEISAACHAYGKVPSHNVSTEVRDMSVVANDAARARNEFGYTRMWSIHPAQIE
AIVAAFAPRDEEITTATEILLAAQSAQWGPTRYHDTLHDRASYRYYWSVLRRAQATGRAVPQDAA
PLFTKVGTNVQAAS
>1JMUA
GNASSIVQTINVTGDGNVFKPSAETSSTAVPSLSLSPGMLN
>2IMRA
SLLRFSAVSRHHRGASIDPMTFSEATTPDALTPDAHTPRLLTCDVLYTGMGGAQSPGGVVVVGET
VAAAGHPDELRRQYPHAAEERAGAVIAPPPVNAHTHLDMSAYEFQALPYFQWIPEVVIRGRHLRG
VAAAQAGADTLTRLGAGGVGDIVWAPEVMDALLAREDLSGTLYFEVLNPFPDKADEVFAAARTHL
ERWRRLERPGLRLGLSPHTPFTVSHRLMRLLSDYAAGEGLPLQIHVAEHPTELEMFRTGGGPLWD
NRMPALYPHTLAEVIGREPGPDLTPVRYLDELGVLAARPTLVHMVNVTPDDIARVARAGCAVVTC
PRSNHHLECGTFDWPAFAAAGVEVALGTDSVASGETLNVREEVTFARQLYPGLDPRVLVRAAVKG
GQRVVGGRTPFLRRGETWQEGFRWELSRDL
>1V0WA
ADSATPHLDAVEQTLRQVSPGLEGDVWERTSGNKLDGSAADPSDWLLQTPGCWGDDKCADRVGTK
RLLAKMTENIGNATRTVDISTLAPFPNGAFQDAIVAGLKESAAKGNKLKVRILVGAAPVYHMNVI
PSKYRDELTAKLGKAAENITLNVASMTTSKTAFSWNHSKILVVDGQSALTGGINSWKDDYLDTTH
PVSDVDLALTGPAAGSAGRYLDTLWTWTCQNKSNIASVWFAASGNAGCMPTMHKDTNPKASPATG
NVPVIAVGGLGVGIKDVDPKSTFRPDLPTASDTKCVVGLHDNTNADRDYDTVNPEESALRALVAS
AKGHIEISQQDLNATCPPLPRYDIRLYDALAAKMAAGVKVRIVVSDPANRGAVGSGGYSQIKSLS
EISDTLRNRLANITGGQQAAKTAMCSNLQLATFRSSPNGKWADGHPYAQHHKLVSVDSSTFYIGS
KNLYPSWLQDFGYIVESPEAAKQLDAKLLDPQWKYSQETATVDYARGICNA
>3BWZA
MAPTSSIEIVLDKTTASVGEIVTASINIKNITNFSGCQLNMKYDPAVLQPVTSSGVAYTKSTMPG
AGTILNSDFNLRQVADNDLEKGILNFSKAYVSLDDYRTAAAPEQTGTVAVVKFKVLKEETSSISF
EDTTSVPNAIDGTVLFDWNGDRIQSGYSVIQPAVINLDMIKASLEHHHHHH
>2XZIA
INDPAKSAAPYHDEFPLFRSANMASPDKLSTGIGFHSFRIPAVVRTTTGRILAFAEGRRHTNQDF
GDINLVYKRTKTTANNGASPSDWEPLREVVGSGAGTWGNPTPVVDDDNTIYLFLSWNGATYSQNG
KDVLPDGTVTKKIDSTWEGRRHLYLTESRDDGNTWSKPVDLTKELTPDGWAWDAVGPGNGIRLTT
GELVIPAMGRNIIGRGAPGNRTWSVQRLSGAGAEGTIVQTPDGKLYRNDRPSQKGYRMVARGTLE
GFGAFAPDAGLPDPACQGSVLRYNSDAPARTIFLNSASGTSRRAMRVRISYDADAKKFNYGRKLE
DAKVSGAGHEGGYSSMTKTGDYKIGALVESDFFNDGTGKNSYRAIIWRRFNLSWILNGPNN
>1POIB
DYTNYTNKEMQAVTIAKQIKNGQVVTVGTGLPLIGASVAKRVYAPDCHIIVESGLMDCSPVEVPR
SVGDLRFMAHCGCIWPNVRFVGFEINEYLHKANRLIAFIGGAQIDPYGNVNSTSIGDYHHPKTRF
TGSGGANGIATYSNTIIMMQHEKRRFMNKIDYVTSPGWIDGPGGRERLGLPGDVGPQLVVTDKGI
LKFDEKTKRMYLAAYYPTSSPEDVLENTGFDLDVSKAVELEAPDPAVIKLIREEIDPGQAFIQVP
>3SFKA
MGHHHHHHAENLYFQGAPFESYNPEFFLYDIFLKFCLKYIDGEICHDLFLLLGKYNILPYDTSND
SIYACTNIKHLDFINPFGVAAGFDKNGVCIDSILKLGFSFIEIGTITPRGQTGNAKPRIFRDVES
RSIINSCGFNNMGCDKVTENLILFRKRQEEDKLLSKHIVGVSIGKNKDTVNIVDDLKYCINKIGR
YADYIAINVSSPNTPGLRDNQEAGKLKNIILSVKEEIDNLEKNNIMNDEFLWFNTTKKKPLVFVK

LAPDLNQEQKKEIADVLLETNIDGMIISNTTTQINDIKSFENKKGGVSGAKLKDISTKFICEMYN
YTNKQIPIIASGGIFSGLDALEKIEAGASVCQLYSCLVFNGMKSAVQIKRELNHLLYQRGYYNLK
EAIGRKHSKS
>2ZIHA
GSMSTLQRRRVNRADSGDTSSIHSSANNTKGDKIANIAVDGDDDNGTNKKIAYDPEESKLRDNIN
IPTLTLMEEVLLMGLRDREGYLSFWNDSISYALRGCIIIELALRGKIRILDDSARKRFDLSERLI
EVIDSSKTGEVLLDETLQLMKNDEPLSISNWIDLLSGETWNLLKINYQLKQVRERLAKGLVDKGV
LRTEMKNFFLFDMATHPIADASCKEAIKRRVLSVLVSRNMELSYNEYFPETTSFKIIRTLALICG
SYGANVLENVLTTLEYEKRDKAISRAEEIMAQFSQYPFDLEKETELGVSVNLNKEVKEEIENNPG
HDLQLEVIAGVFEVFSRMDMLL
>1DYOA
KPEEPDAGYYYHDTFEGSVGQWTARGPAEVLLSGRTAYKGSESLLVRNRTAAWNGAQRALNPRTF
VPGNTYCFSVVASFIEGASSTTFCMKLQYVDGSGTQRYDTIDMKTVGPNQWVHLYNPQYRIPSDA
TDMYVYVETADDTINFYIDEAIGAVAGTVI
>3LMEA
MSLKIIAPTDKTITPSGTWSIGARAGDFVFIGGMHGTDRVTGKMVDGDEARIRRMFDNMLAAAEA
AGATKADAVRLTVFVTDVAKYRPVVNKVQKDIWGDGPYPPRTVLQVPALDQGDIAEIDGTFYAPA
EGHHHHHH
>3GDWA
SNANVGVFVLMHGDSTASSMLKTAQELLGTSIGTAMNMPLTMEVQTMYEQLRNQVITQKESLNNG
ILLLTDMGSLNSFGNMLFEETGIRTKAITMTSTMIVLEAIRMASVGRSLEDIYQNIQLSFESVVR
EQFRSSLQK
>1RJUV
HECQCQCGSCKNNEQCQKSCSCPTGCNSDDKCPCGN
>4I3MA
GSHMGLFNAHAVAQQRADRIATLLQSFADGQLDTAVGEAPAPGYERHYDSLRALQRQLREQRAEL
QQVESLEAGLAEMSRQHEAGWIDQTIPAERLEGRAARIAKGVNELVAAHIAVKMKVVSVVTAYGQ
GNFEPLMDRLPGKKAQITEAIDGVRERLRGAAEATSAQLATAAYN
>3M6NA
MGSSHHHHHHSQDPNSMSAVQPFIRTNIGSTLRIIEEPQRDVYWIHMHADLAINPGRACFSTRLV
DDITGYQTNLGQRLNTAGVLAPHVVLASDSDVFNLGGDLALFCQLIREGDRARLLDYAQRCVRGV
HAFHVGLGARAHSIALVQGNALGGGFEAALSCHTIIAEEGVMMGLPEVLFDLFPGMGAYSFMCQR
ISAHLAQKIMLEGNLYSAEQLLGMGLVDRVVPRGQGVAAVEQVIRESKRTPHAWAAMQQVREMTT
AVPLEEMMRITEIWVDTAMQLGEKSLRTMDRLVRAQSRRSGLDAG
>3SOYA
SNAATSTVKQEITEGINRYLYSIDKADPTLGKQLFYVSPETSFIHPRGHERGWSQIAENFYGTTM
GKTFSKRTLKLDAPPAIHVYGNAAVAEFDWHFTAVRRDNGQTQHTTGRESQVWAKIPNTGWRIVH
VHYSGPAKTGVGEGY
>1LQVA
SQDASDGLQRLHMLQISYFRDPYHVWYQGNASLGGHLTHVLEGPDTNTTIIQLQPLQEPESWART
QSGLQSYLLQFHGLVRLVHQERTLAFPLTIRCFLGCELPPEGSRAHVFFEVAVNGSSFVSFRPER
ALWQADTQVTSGVVTFTLQQLNAYNRTRYELREFLEDTCVQYVQKHISAENTKGSQTSRSYTS
>3GODA
GSFTMDDISPSELKTILHSKRANLYYLQHCRVLVNGGRVEYVTDEGRHSHYWNIPIANTTSLLLG

TGTSITQAAMRELARAGVLVGFCGGGGTPLFSANEVDVEVSWLTPQSEYRPTEYLQRWVGFWFDE
EKRLVAARHFQRARLERIRHSWLEDRVLRDAGFAVDATALAVAVEDSARALEQAPNHEHLLTEEA
RLSKRLFKLAAQATRYGEFVRAKRGSGGDPANRFLDHGNYLAYGLAATATWVLGIPHGLAVLHGK
TRRGGLVFDVADLIKDSLILPQAFLSAMRGDEEQDFRQACLDNLSRAQALDFMIDTLKDVAQRST
VSA

>3CR3A
LLTIDTTIEWLGKFNEKIQENKAYLSELDGPIGDGDHGANMARGMSETMKALEVSNFGNVSEIFK
KVAMTLMSKVGGASGPLYGSAFLAMSKTAIETLDTSELIYAGLEAIQKRGKAQVGEKTMVDIWSA
FLNDLQTDSASKDNLEKVVKASAGLLATKGRASYLGERSIGHIDPGTQSSAYLFETLLEVVA

>2QT1A
MGSSHHHHHHSSGLVPRGSKTFIIGISGVTNSGKTTLAKNLQKHLPNCSVISQDDFFKPESEIET
DKNGFLQYDVLEALNMEKMMSAISCWMESARHSVVSTDQESAEEIPILIIEGFLLFNYKPLDTIW
NRSYFLTIPYEECKRRRSTRVYQPPDSPGYFDGHVWPMYLKYRQEMQDITWEVVYLDGTKSEEDL
FLQVYEDLIQEL

>1JMUB
PGGVPWIAIGDETSVTSPGALRRMTSKDIPETAIINTDNSSGAVPSESALVPYNDEPLVVVTEHA
IANFTKAEMALEFNREFLDKLRVLSVSPKYSDLLTYVDCYVGVSARQALNNFQKQVPVITPTRQT
MYVDSIQAALKALEKWEIDLRVAQTLLPTNVPIGEVSCPMQSVVKLLDDQLPDDSLIRRYPKEAA
VALAKRNGGIQWMDVSEGTVMNEAVNAVAASALAPSASAPPLEEKSKLTEQAMDLVTAAEPEIIA
SLVPVPAPVFAIPPKPADYNVRTLKIDEATWLRMIPKTMGTLFQIQVTDNTGTNWHFNLRGGTRV
VNLDQIAPMRFVLDLGGKSYKETSWDPNGKKVGFIVFQSKIPFELWTAASQIGQATVVNYVQLYA
EDSSFTAQSIIATTSLAYNYEPEQLNKTDPEMNYYLLATFIDSAAITPTNMTQPDVWDALLTMSP
LSAGEVTVKGAVVSEVVPAELIGSYTPESLNASLPNDAARCMIDRASKIAEAIKIDDDAGPDEYS
PNSVPIQGQLAISQLETGYGVRIFNPKGILSKIASRAMQAFIGDPSTIITQAAPVLSDKNNWIAL
AQGVKTSLRTKSLSAGVKTAVSKLSSSESIQNWTQGFLDKVSTHFPAPKPDCPTNGDGSEPSARR
VKRDSYAGVVKRGYTR

>2EZ2A
MNYPAEPFRIKSVETVSMIPRDERLKKMQEAGYNTFLLNSKDIYIDLLTDSGTNAMSDKQWAGMM
MGDEAYAGSENFYHLERTVQELFGFKHIVPTHQGRGAENLLSQLAIKPGQYVAGNMYFTTTRYHQ
EKNGAVFVDIVRDEAHDAGLNIAFKGDIDLKKLQKLIDEKGAENIAYICLAVTVNLAGGQPVSMA
NMRAVRELTEAHGIKVFYDATRCVENAYFIKEQEQGFENKSIAEIVHEMFSYADGCTMSGKKDCL
VNIGGFLCMNDDEMFSSAKELVVVYEGMPSYGGLAGRDMEAMAIGLREAMQYEYIEHRVKQVRYL
GDKLKAAGVPIVEPVGGHAVFLDARRFCEHLTQDEFPAQSLAASIYVETGVRSMERGIISAGRNN
VTGEHHRPKLETVRLTIPRRVYTYAHMDVVADGIIKLYQHKEDIRGLKFIYEPKQLRFFTARFDY
I

>2QWUA
MIMSEMITRQQVTSGETIHVRTDPTACIGSHPNCRMFIDSLTIAGEKLDKNIVAIDGGEDVTKAD
SATAAASVIRMSITPGSINPTISITLGVLIKSNVRTKIEEKVSSILQASATDMKIKLGNSNKKQE
YKTDEAWGIMIDLSNLELYPISAKAFSISIEPTELMGVSKDGMRYHIISIDGLTTSQGSLPVCCA
ASTDKGVAKIGYIAAA

>3DI5A
GMYQTIEGFLQSWTYETESTQKMLDVLTDESLSQEIAPGHWTLGRVAWHIVTAIPVILSGTGLKF
EGETKDYPVPTSAKTIADGYRKVNTAFVEALQSEWTDKDLTTINDFFGRPMPNSIFLMTLINHQN
HHRGQMTVLMRQAGLTVPGVYGPAKEEWATAGMEAPKM

>2ZPMA
GIPIEPVLENVQPNSAASKAGLQAGDRIVKVDGQPLTQWVTFVMLVRDNPGKSLALEIERQGSPL
SLTLIPESKPGNGKAIGFVGIEPKVI
>2BWRA
SVVVISQALPVPTRIPGVADLVGFGNGGVYIIRNSLLIQVVKVINNFGYDAGGWRVEKHVRLLAD
TTGDNQSDVVGFGENGVWISTNNGNNTFVDPPKMVLANFAYAAGGWRVEKHIRFMADLRKTGRAD
IVGFGDGGIYISRNNGGGQFAPAQLALNNFGYAQGWRLDRHLRFLADVTGDGLLDVVGFGENQVY
IARNSGNGTFQPAQAVVNNFCIGAGGWTISAHPRVVADLTGDRKADILGFGVAGVYTSLNNGNGT
FGAVNLVLKDFGVNSGWRVEKHVRCVSSLTNKKVGDIIGFGDAGVYVALNNGNGTFGPVKRVIDN
FGYNQGWRVDKHPRFVVDLTGDGCADIVGFGENSVWACMNKGDGTFGPIMKLIDDMTVSKGWTLQ
KTVRYAANLYL
>1IQZA
PKYTIVDKETCIACGACGAAAPDIYDYDEDGIAYVTLDDNQGIVEVPDILIDDMMDAFEGCPTDS
IKVADEPFDGDPNKFE
>1M6IA
EEVPQDKAPSHVPFLLIGGGTAAFAAARSIRARDPGARVLIVSEDPELPYMRPPLSKELWFSDDP
NVTKTLRFKQWNGKERSIYFQPPSFYVSAQDLPHIENGGVAVLTGKKVVQLDVRDNMVKLNDGSQ
ITYEKCLIATGGTPRSLSAIDRAGAEVKSRTTLFRKIGDFRSLEKISREVKSITIIGGGFLGSEL
ACALGRKARALGTEVIQLFPEKGNMGKILPEYLSNWTMEKVRREGVKVMPNAIVQSVGVSSGKLL
IKLKDGRKVETDHIVAAVGLEPNVELAKTGGLEIDSDFGGFRVNAELQARSNIWVAGDAACFYDI
KLGRRRVEHHDHAVVSGRLAGENMTGAAKPYWHQSMFWSDLGPDVGYEAIGLVDSSLPTVGVFAK
ATAQDNPKSATEQSGTGIRSESETESEASEITIPPSTPAVPQAPVQGEDYGKGVIFYLRDKVVVG
IVLWNIFNRMPIARKIIKDGEQHEDLNEVAKLFNIHED
>1R6WA
SHMRSAQVYRWQIPMDAGVVLRDRRLKTRDGLYVCLREGEREGWGEISPLPGFSQETWEEAQSVL
LAWVNNWLAGDCELPQMPSVAFGVSCALAELTDTLPQAANYRAAPLCNGDPDDLILKLADMPGEK
VAKVRVGLYEAVRDGMVVNLLLEAIPDLHLRLDANRAWTPLKGQQFAKYVNPDYRDRIAFLEEPC
KTRDDSRAFARETGIAIAWDESLREPDFAFVAEEGVRAVVIKPTLTGSLEKVREQVQAAHALGLT
AVISSSIESSLGLTQLARIAAWLTPDTIPGLDTLDLMQAQQVRRWPGSTLPVVEVDALERLL
>1XIPA
GASSLKDEVPTETSEDFGFKFLGQKQILPSFNEKLPFASLQNLDISNSKSLFVAASGSKAVVGEL
QLLRDHITSDSTPLTFKWEKEIPDVIFVCFHGDQVLVSTRNALYSLDLEELSEFRTVTSFEKPVF
QLKNVNNTLVILNSVNDLSALDLRTKSTKQLAQNVTSFDVTNSQLAVLLKDRSFQSFAWRNGEME
KQFEFSLPSELEELPVEEYSPLSVTILSPQDFLAVFGNVISETDDEVSYDQKMYIIKHIDGSASF
QETFDITPPFGQIVRFPYMYKVTLSGLIEPDANVNVLASSCSSEVSIWDSKQVIEPSQDSERAVL
PISEETDKDTNPIGVAVDVVTSGTILEPCSGVDTIERLPLVYILNNEGSLQIVGLFHVAAIKS
>3VV1A
MDYKDDDDKAAAGSMIGGGIGISFRNEFFNPQTPVNIPVQGFSNGARLRLVLLPTSADSRFHINL
RTPDDIVLHFNARFDEGAVVNNSTSGGGWQSEDRHANPFQQNKIYTLEFVSNGGIISIFVNGAHF
ADFVERTPSHGVHLIEIEGGVHVHSAHVSH
>2GBLA
MTSAEMTSPNNNSEHQAIAKMRTMIEGFDDISHGGLPIGRSTLVSGTSGTGKTLFSIQFLYNGII
EFDEPGVFVTFEETPQDIIKNARSFGWDLAKLVDEGKLFILDASPDPEGQEVVGGFDLSALIERI
NYAIQKYRARRVSIDSVTSVFQQYDASSVVRRELFRLVARLKQIGATTVMTTERIEEYGPIARYG

VEEFVSDNVVILRNVLEGERRRRTLEILKLRGTSHMKGEYPFTITDHGINIFPLGAMRLTQRSSN
VRVSSGVVRLDEMCGGGFFKDSIILATGATGTGKTLLVSRFVENACANKERAILFAYEESRAQLL
RNAYSWGMDFEEMERQNLLKIVCAYPESAGLEDHLQIIKSEINDFKPARIAIDSLSALARGVSNN
AFRQFVIGVTGYAKQEEITGLFTNTSDQFMGAHSITDSHISTITDTIILLQYVEIRGEMSRAINV
FKMRGSWHDKAIREFMISDKGPDIKDSFRNFERIISGSPTRITVDEKSELSRIVRGVQEKGPES
>3PIUA
MRMLSRNATFNSHGQDSSYFLGWQEYEKNPYHEVHNTNGIIQMGLAENQLCFDLLESWLAKNPEA
AAFKKNGESIFAELALFQDYHGLPAFKKAMVDFMAEIRGNKVTFDPNHLVLTAGATSANETFIFC
LADPGEAVLIPTPYYPGFDRDLKWRTGVEIVPIHCTSSNGFQITETALEEAYQEAEKRNLRVKGV
LVTNPSNPLGTTMTRNELYLLLSFVEDKGIHLISDEIYSGTAFSSPSFISVMEVLKDRNCDENSE
VWQRVHVVYSLSKDLGLPGFRVGAIYSNDDMVVAAATKMSSFGLVSSQTQHLLSAMLSDKKLTKN
YIAENHKRLKQRQKKLVSGLQKSGISCLNGNAGLFCWVDMRHLLRSNTFEAEMELWKKIVYEVHL
NISPGSSCHCTEPGWFRVCFANLPERTLDLAMQRLKAFVGEYYNV
>3U8VA
SGHTAHVDEAVKHAEEAVAHGKEGHTDQLLEHAKESLTHAKAASEAGGNTHVGHGIKHLEDAIKH
GEEGHVGVATKHAQEAIEHLRASEHKSH
>3VSVA
MEYHVAKTGSDEGKGTLKDPFLTINKAASVAMAGDTIIVHEGVYREWVKPKYKGLSDKRRITYKA
AEGEKVVIKGSERIQSWQRVEGNVWRCQLPNSFFGEFNPYKEEVFGDWLLTVNEKKHLGDVYLNG
MSFYEVTNYEDLFNPQLRTEVLDHWTQKIVPIKNAEQTKYVWYAEVDREKTTIYANFQGADPNEE
FVEINVRRSCFYPVETGIDYITVKGFEMAHAATPWAPPTADQPGLIGPNWSKGWIIEDNIIHDAK
CSAISIGKEATTGNNYRSIRKDKPGYQYQLEAVFNAKRNGWSKEKIGSHIIRNNTIYDCGQNAIV
GHLGGVFSEIYNNHIYNIALKREFYGHEIAGIKLHAAIDVQIHHNRIHDCSLGLWLDWEAQGTRV
SKNLFYNNNRDVFVEVSHGPYLVDHNILSSEYAIDNMSQGGAYINNLIAGKMNQRKVLNRSTQYH
LPHSTEVAGFAFVYGGDDRFYNNIFIGKEGLENVGTSHYNNCTTSLEEYIEKVNEVPGDLGEFER
VEQPVYINKNAYFNGAEPFEKEKDNLVKKDFDPKLAIIDEGDEVYLSLQLPDEFENIVGDIHSTK
TLERVRIVDAEYESPDGKELVLDTDYLDAKKPENSSIGPIALLKKGNNYIKVW
>3SGGA
GAGVVVPKYTPSTENPGGPGEEPGDGLIDPAEPLDRGFMHLKGRELKSLNSISITGLNDGEKVIL
STLAGLAARVTGDQVYINEGGPSSVWLKQMQNKYGIPVNTYNALAPLVQHYVETGVIKGYIVYTP
YSEGQSHSINVATSLCGLLRGIAVPESLVDKVKAMGVTTELMDVRSYDEKWLYENYKDQLDKSLA
ADMKPEIFHHLRDYITMTNAFAFYDYNARRDWSWRTSILKDLDKGAYCFGYYDLDEWGMVNNASQ
LGVSMLPTDQAANLATLSSIYDTTGLKQRPATKEVVTEENVHYVTFLVSDGDNIAFNLWGQQGYM
DHDLHGQFPLGYTISPSLYDLAPAALRWYYENSKEGDYFVAGPSGSSYIFPSKMSDADLDDYLAK
LNEYVDKSGLNICNILDQKIMDNPKVYNKYLAQPNIDAIFYTGYGEKGDGRIKFSDNGKPVIEQR
SVLWEGIDGGSNRGEESTVISQINSRSANPHSADGYTFVFVHCWTKNQQSIKTVIDGLNDNVRVV
PVDQFVQLVKQNLGPK
>4DVEA
MHHHHHHHHAMTNNQKVKTLTYSAFMTAFIIILGFLPGIPIGFIPVPIILQNMGIMMAGGLLGPK
YGTISVGAFLALALIGLPVLTGGNGGAASFLGPSGGYRIAWLFTPFLIGFFLKKLKITTSQNWFG
ELIIVLLFGVIFVDFVGAIWLSFQSNIPLLTSLISNLVFIPGDCIKAILTVVIVRRLRKQGGFEL
YFR
>2Q4MA
MEQPYVYAYPQGSGPSGAPTPQAGGVVVDPKYCAPYPIDMAIVRKMMSLTDGNFVITDVNGNLLF

KVKEPVFGLHDKRVLLDGSGTPVVTLREKMVSMHDRWQVFRGGSTDQRDLLYTVKRSSMLQLKTK
LDVFLGHNKDEKRCDFRVKGSWLERSCVVYAGESDAIVAQMHRKHTVQSVFLGKDNFSVTVYPNV
DYAFIASLVVILDDVNREDRAA
>2G0WA
MGSDKIHHHHHHMTNANGNLKKCPITISSYTLGTEVSFPKRVKVAAENGFDGIGLRAENYVDALA
AGLTDEDMLRILDEHNMKVTEVEYITQWGTAEDRTAEQQKKEQTTFHMARLFGVKHINCGLLEKI
PEEQIIVALGELCDRAEELIIGLEFMPYSGVADLQAAWRVAEACGRDNAQLICDTWHWARANQTA
ESIKNVPADRIVSIQLCDVHETPYKELREESLHDRLAPGEGYGDTVGFAKILKEHGVNPRVMGVE
VISDSMVATGLEYAALKVYNATKKVLDEAWPEISPR
>2NXVA
MKPVPTYVQDKDESTLMFSVCSLVRDQAKYDRLLESFERFGFTPDKAEFLAADNREGNQFHGFSW
HKQMLPRCKGRYVIFCHEDVELVDRGYDDLVAAIEALEEADPKWLVAGVAGSPWRPLNHSVTAQA
LHISDVFGNDRRRGNVPCRVESLDECFLLMRRLKPVLNSYDMQGFHYYGADLCLQAEFLGGRAYA
IDFHLHHYGRAIADENFHRLRQEMAQKYRRWFPGRILHCVTGRVALGGGWYEAR
>3L6IA
HHHSHMNQLTQYTITEQEINQSLAKHNNFSKDIGLPGVADAHIVLTNLTSQIGREEPNKVTLTGD
ANLDMNSLFGSQKATMKLKLKALPVFDKEKGAIFLKEMEVVDATVQPEKMQTVMQTLLPYLNQAL
RNYFNQQPAYVLREDGSQGEAMAKKLAKGIEVKPGEIVIPFTDLEHHHHHH
>1IARB
FKVLQEPTCVSDYMSISTCEWKMNGPTNCSTELRLLYQLVFLLSEAHTCIPENNGGAGCVCHLLM
DDVVSADNYTLDLWAGQQLLWKGSFKPSEHVKPRAPGNLTVHTNVSDTLLLTWSNPYPPDNYLYN
HLTYAVNIWSENDPADFRIYNVTYLEPSLRIAASTLKSGISYRARVRAWAQAYNTTWSEWSPSTK
WHNSYREPFEQH
>4FR9A
GGDDDDTGYLPPSQAIQDALKKLYPNATAIKWEQKGVYYVADCQADGREKEVWFDANANWLMTET
ELNSINNLPPAVLTAFMESSYNNWVVDDVVILEYPNEPSTEFVVTVEQGKKVDLYFSEGGGLLHE
KDVTNGDDTHWPRV
>1OXWA
MHHHHHHAMAQLGEMVTVLSIDGGGIRGIIPATILEFLEGQLQEMDNNADARLADYFDVIGGTST
GGLLTAMISTPNENNRPFAAAKEIVPFYFEHGPQIFNPSGQILGPKYDGKYLMQVLQEKLGETRV
HQALTEVVISSFDIKTNKPVIFTKSNLANSPELDAKMYDISYSTAAAPTYFPPHYFVTNTSNGDE
YEFNLVDGAVATVADPALLSISVATRLAQKDPAFASIRSLNYKKMLLLSLGTGTTSEFDKTYTAK
EAATWTAVHWMLVIQKMTDAASSYMTDYYLSTAFQALDSKNNYLRVQENALTGTTTEMDDASEAN
MELLVQVGENLLKKPVSEDNPETYEEALKRFAKLLSDRKKLRANKASY
>1JB0K
MVLATLPDTTWTPSVGLVVILCNLFAIALGRYAIQSRGKGPGLPIALPALFEGFGLPELLATTSF
GHLLAAGVVSGLQYAGAL
>4ELNA
MGSSHHHHHHSSGLVPRGSHMGLCTSKPSVVGSPVAGSPEHYLTHTAEQTTPSTPSSPEAPMSPS
LHGLAALGSPRASSSPRPLSPLVELNTSDLIKQKKQLWQRVQHDGAQFRSTPEERKQFKTALITL
WGEQYRPERQQRWNGMMQRMAQMKWNHPELKYMATEDLVALQAWTTDDYEVVQDVLEKEARPTAH
GLAFAKCIISALHSLPEEYSYQGTVFTGEDQLPDWVSERYQERSITTDRRFFAASETKNASWQGM
AVEWESNSTTGKRISMFSERPNEQEVLFPPGTRFQVTRIEENETHPRLKIYQSQIA
>3Q1CA

SHMASSWDEMSCAEKLLKVLSFGLWNPTYSRSERQSFQELLTVLEPVYPLPNELGRVSARFSDGS
SLRISVTNSESIEAEIRTPDNEKITVLLESNEQNRLLQSLPIDRHMPYIQVHRALSEMDLTDTTS
MRNLLGFTSKLSTTLIPHNAQTDPLSGPTPFSSIFMDTCRGLGNAKLSLNGVDIPANAQMLLRDA
LGLKDTHSSPSRNVIDHGISRHDAEQIARESSGSDNQKAEVVEFLCHPEAATAICSAFYQSFNVP
ALTLTHERISKASEYNAERSLDTPNACINISISQSSDGNIYVTSHTGVLIMAPEDRPNEMGMLTN
RTSYEVPQGVKCTIDEMVRALQPRYAASETYLQNT
>2OXGB
MRGSHHHHHHGSSTVDELTAAFTGGAATGEGGLTLTAPEIAENGNTVPIEVKAPGAVAIMLLAAG
NPEPAVATFNFGPAAADQRAATRIRLAQTQDVIALAKMADGSVVKAQTTVKVTIGGCGG
>2WE5A
MGKKMVVALGGNAILSNDASAHAQQQALVQTSAYLVHLIKQGHRLIVSHGNGPQVGNLLLQQQAA
DSEKNPAMPLDTCVAMTQGSIGYWLSNALNQELNKAGIKKQVATVLTQVVVDPADEAFKNPTKPI
GPFLTEAEAKEAMQAGAIFKEDAGRGWRKVVPSPKPIDIHEAETINTLIKNDIITISCGGGGIPV
VGQELKGVEAVIDKDFASEKLAELVDADALVILTGVDYVCINYGKPDEKQLTNVTVAELEEYKQA
GHFAPGSMLPKIEAAIQFVESQPNKQAIITSLENLGSMSGDEIVGTVVTK
>3UC2A
MGSDKIHHHHHHENLYFQGEQVQRFGDLDVHYNVFNSSFLQPNVASAVGLVRSKAQGVINVVPME
KGKPVEAAVTGSAKDLTGKVIPLEFRRVSEEGAIYNLAQFPISQRETLVFTIKVEAKGEPAQTFS
FNKEIFPDE
>2VTWA
TSSVAAFTSGTIGLSSPTGNFVSSSNNPFNGSYFLQQINTMGMLTTSLYVKVDTTTMGTRPTGAV
NENARYFTVWVSSFLTQCNPSNIGQGTLEPSNISMTSFEPARNPISPPVFNMNQNIPYYASRFGV
LESYRPIFTGSLNTGSIDVRMQVTPVLATNNTTYNLIAFTFQCASAGLFNPTVNGTVAIGPVVHT
CPAARAPVTV
>2XSUA
MHHHHHHNRQQIDALVKQMNVDTAKGPVDERIQQVVVRLLGDLFQAIEDLDIQPSEVWKGLEYLT
DAGQANELGLLAGGLGLEHYLDLRADEADAKAGITGGTPRTIEGPLYVAGAPESVGFARMDDGSE
SDKVDTLIIEGTVTDTEGNIIEGAKVEVWHANSLGNYSFFDKSQSDFNLRRTILTDVNGKYVALT
TMPVGYGCPPEGTTQALLNKLGRHGNRPSHVHYFVSAPGYRKLTTQFNIEGDEYLWDDFAFATRD
GLVATATDVTDEAEIARRELDKPFKHITFNVELVKEAEAAPSSEVERRRASA
>3EDOA
GMAKKTLILYYSWSGETKKMAEKINSEIKDSELKEVKVSEGTFDADMYKTSDIALDQIQGNKDFP
EIQLDNIDYNNYDLILIGSPVWSGYPATPIKTLLDQMKNYRGEVASFFTSAGTNHKAYVSHFNEW
ADGLNVIGVARDDSEVDKWSK
>2FH1A
MDDDGTGQKQIWRIEGSNKVPVDPATYGQFYGGDSYIILYNYRHGGRQGQIIYNWQGAQSTQDEV
AASAILTAQLDEELGGTPVQSRVVQGKEPAHLMSLFGGKPMIIYKGGTSREGGQTAPASTRLFQV
RANSAGATRAVEVLPKAGALNSNDAFVLKTPSAAYLWVGTGASEAEKTGAQELLRVLRAQPVQVA
EGSEPDGFWEALGGKAAYRTSPRLKDKKMDAHPPRLFACSNKIGRFVIEEVPGELMQEDLATDDV
MLLDTWDQVFVWVGKDSQEEEKTEALTSAKRYIETDPANRDRRTPITVVKQGFEPPSFVGWFLGW
DDDYWSVDPLDRAMAELAA
>1HUXA
MSIYTLGIDVGSTASKCIILKDGKEIVAKSLVAVGTGTSGPARSISEVLENAHMKKEDMAFTLAT
GYGRNSLEGIADKQMSELSCHAMGASFIWPNVHTVIDIGGQDVKVIHVENGTMTNFQMNDKCAAG

TGRFLDVMANILEVKVSDLAELGAKSTKRVAISSTCTVFAESEVISQLSKGTDKIDIIAGIHRSV
ASRVIGLANRVGIVKDVVMTGGVAQNYGVRGALEEGLGVEIKTSPLAQYNGALGAALYAYKKAAK
SAWSHPQFEK
>1V5VA
MIQMVKRVHIFDWHKEHARKIEEFAGWEMPIWYSSIKEEHLAVRNAVGIFDVSHMGEIVFRGKDA
LKFLQYVTTNDISKPPAISGTYTLVLNERGAIKDETLVFNMGNNEYLMICDSDAFEKLYAWFTYL
KRTIEQFTKLDLEIELKTYDIAMFAVQGPKARDLAKDLFGIDINEMWWFQARWVELDGIKMLLSR
SGYTGENGFEVYIEDANPYHPDESKRGEPEKALHVWERILEEGKKYGIKPCGLGARDTLRLEAGY
TLYGNETKELQLLSTDIDEVTPLQANLEFAIYWDKDFIGKDALLKQKERGVGRKLVHFKMIDKGI
PREGYKVYANGEMIGEVTSGTLSPLLNVGIGIAFVKEEYAKPGIEIEVEIRGQRKKAVTVTPPFY
DPKKYGLFRET
>3VATA
MMSYYHHHHHHDYDIPTTENLYFQGAMGRARKEAVQAAARELLKFVNRSPSPFHAVAECRSRLLQ
AGFHELKETESWDIKPESKYFLTRNSSTIIAFAVGGQYVPGNGFSLIGAHTDSPCLRVKRRSRRS
QVGFQQVGVETYGGGIWSTWFDRDLTLAGRVIVKCPTSGRLEQRLVHVDRPILRIPHLAIHLQRN
VNENFGPNMEMHLVPILATSIQEELEKGTPEPGPLNATDERHHSVLTSLLCAHLGLSPEDILEME
LCLADTQPAVLGGAYEEFIFAPRLDNLHSCFCALQALIDSCSAPASLAADPHVRMIALYDNEEVG
SESAQGAQSLLTELVLRRISASPQHLTAFEEAIPKSYMISADMAHAVHPNYLDKHEENHRPLFHK
GPVIKVNSKQRYASNAVSEALIREVASSVGVPLQDLMVRNDSPCGTTIGPILASRLGLRVLDLGS
PQLAMHSIRETACTTGVLQTITLFKGFFELFPSLSRSLLVD
>4IQ0A
SNAMLKLGVIGTGAISHHFIEAAHTSGEYQLVAIYSRKLETAATFASRYQNIQLFDQLEVFFKSS
FDLVYIASPNSLHFAQAKAALSAGKHVILEKPAVSQPQEWFDLIQTAEKNNCFIFEAARNYHEKA
FTTIKNFLADKQVLGADFNYAKYSSKMPDLLAGQTPNVFSDRFAGGALMDLGIYPLYAAVRLFGK
ANDATYHAQQLDNSIDLNGDGILFYPDYQVHIKAGKNITSNLPCEIYTTDGTLTLNTIEHIRSAI
FTDHQGNQVQLPIQQAPHTMTEEVAAFAHMIQQPDLNLYQTWLYDAGSVHELLYTMRQTAGIRFE
AEK
>2QISA
GSSHHHHHHSSGRENLYFQGHMNGDQNSDVYAQEKQDFVQHFSQIVRVLTEDEMGHPEIGDAIAR
LKEVLEYNAIGGKYNRGLTVVVAFRELVEPRKQDADSLQRAWTVGWCVELLQAFFLVADDIMDSS
LTRRGQICWYQKPGVGLDAINDANLLEACIYRLLKLYCREQPYYLNLIELFLQSSYQTEIGQTLD
LLTAPQGNVDLVRFTEKRYKSIVKYKSAFYSFYLPIAAAMYMAGIDGEKEHANAKKILLEMGEFF
QIQDDYLDLFGDPSVTGKIGTDIQDNKCSWLVVQCLQRATPEQYQILKENYGQKEAEKVARVKAL
YEELDLPAVFLQYEEDSYSHIMALIEQYAAPLPPAVFLGLARKIYKRRK
>4HHRA
MRGSHHHHHHGSMKVITSLISSILLKFIHKDFHEIYARMSLLDRFLLLIVHGVDKMVPWHKLPVF
LGLTYLEVRRHLHQQYNLLNVGQTPTGIRFDPANYPYRTADGKFNDPFNEGVGSQNSFFGRNCPP
VDQKSKLRRPDPMVVATKLLGRKKFIDTGKQFNMIAASWIQFMIHDWIDHLEDTHQIELVAPKEV
ASKCPLSSFRFLKTKEVPTGFFEIKTGSQNIRTPWWDSSVIYGSNSKTLDRVRTYKDGKLKISEE
TGLLLHDEDGLAISGDIRNSWAGVSALQALFIKEHNAVCDALKDEDDDLEDEDLYRYARLVTSAV
VAKIHTIDWTVQLLKTDTLLAGMRANWYGLLGKKFKDSFGHAGSSILGGVVGMKKPQNHGVPYSL
TEDFTSVYRMHSLLPDQLHILDIDDVPGTNKSLPLIQEISMRDLIGRKGEETMSHIGFTKLMVSM
GHQASGALELMNYPMWLRDIVPHDPNGQARPDHVDLAALEIYRDRERSVPRYNEFRRSMFMIPIT
KWEDLTEDEEAIEVLDDVYDGDVEELDLLVGLMAEKKIKGFAISETAFYIFLIMATRRLEADRFF

TSDFNETIYTKKGLEWVNTTESLKDVIDRHYPDMTDKWMNSESAFSVWDSPPLTKNPIPLYLRIP
SR
>3MWCA
MSLTESARIDGVSLYEIVIPMKIPFQISSGTCYTRRSLVVEIREGDLFGYGESAPFEEPFYLGET
LETTKVILKNHLLPMILGKEPLSIEEFNHLIKNGIRGNHFARCGVENAYWDLIAKKNKISLKAMI
EKKMKNLGVKQEYLASNNYIESGAALGIPEDGRIETLIHQVEESLQEGYRRIKIKIKPGWDVEPL
QETRRAVGDHFPLWTDANSSFELDQWETFKAMDAAKCLFHEQPLHYEALLDLKELGERIETPICL
DESLISSRVAEFVAKLGISNIWNIKIQRVGGLLEAIKIYKIATDNGIKLWGGTMPESGLGARFLI
SLASFRGFVFPADVAASEKWYGKGNDLVENTMTDGKIYVPDEPGASFDMTLSHLEALGKKIWESQ
RGEGHHHHHH
>2PNQA
ASTPQKFYLTPPQVNSILKANEYSFKVPEFDGKNVSSILGFDSNRLPANAPIEDRRSATTCLQTR
GMLLGVFDGHAGCACSQAVSERLFYYIAVSLLPHETLLEIENAVESGRALLPILQWHKHPNDYFS
KEASKLYFNGLRTYWQELIDLNTGESADIDVKEALINAFKRLDNDISLEAQVGDPNSFLNYLVLR
VAFSGATACVAHVDGVDLHVANTGDSRAMLGVQEEDGSWSAVTLSNDHNAQNERELQRLKLEHPK
NEAKSVVKQDRLLGLLMPFRAFGDVKFKWSIDLQKRVIESGPDQLNDNEYTKFIPPNYHTPPYLT
AEPEVTYHRLRPQDKFLVLATDGLWETMHRQDVVRIVGEYLTGMHHQQPIAVGGYKVTLGQMHGL
LTERRAKMSSVFEDQNAATHLIRHAVGNNEFGAVDHERLSKMLSLPEELARMYRDDITIIVVQFN
SHVVGAYQNQEQ
>1YQGA
MNVYFLGGGNMAAAVAGGLVKQGGYRIYIANRGAEKRERLEKELGVETSATLPELHSDDVLILAV
KPQDMEAACKNIRTNGALVLSVAAGLSVGTLSRYLGGTRRIVRVMPNTPGKIGLGVSGMYAEAEV
SETDRRIADRIMKSVGLTVWLDDEEKMHGITGISGSGPAYVFYLLDALQNAAIRQGFDMAEARAL
SLATFKGAVALAEQTGEDFEKLQKNVTSKGGTTHEAVEAFRRHRVAEAISEGVCACVRRSQEMER
QYQ
>3T4LA
MDDANKIRREEVLVSMCDQRARMLQDQFSVSVNHVHALAILVSTFHYHKNPSAIDQETFAEYTAR
TAFERPLLSGVAYAEKVVNFEREMFERQHNWVIKTMDRGEPSPVRDEYAPVIFSQDSVSYLESLD
MMSGEEDRENILRARETGKAVLTSPFRLLETHHLGVVLTFPVYKSSLPENPTVEERIAATAGYLG
GAFDVESLVENLLGQLAGNQAIVVHVYDITNASDPLVMYGNQDEEADRSLSHESKLDFGDPFRKH
KMICRYHQKA
>1NJHA
SNAMKAIIKEDVQASLERYADRPVYIHLETTTGSYSAHLNEKNMTVVAYIRNAKVTYHQAKIKGN
GPYRVGLKTEEGWIYAEGLTEYTVDEENRLLMAGHLPGGKLAISLQISEKPFTV
>2WLUA
MTNTLVENIYASVTHNISKKEASKNEKTKAVLNQAVADLSVAASIVHQVHWYMRGPGFLYLHPKM
DELLDSLNANLDEVSERLITIGGAPYSTLAEFSKHSKLDEAKGTYDKTVAQHLARLVEVYLYLSS
LYQVGLDITDEEGDAGTNDLFTAAKTEAEKTIWMLQAERGQGPAL
>3T9OA
MAIKKTTEIDAILLNLNKAIDAHYQWLVSMFHSVVARDASKPEITDNHSYGLCQFGRWIDHLGPL
DNDELPYVRLMDSAHQHMHNCGRELMLAIVENHWQDAHFDAFQEGLLSFTAALTDYKIYLLTLEH
HHHHH
>3HZ7A
MITIDALGQVCPIPVIRAKKALAELGEAGGVVTVLVDNDISRQNLQKMAEGMGYQSEYLEKDNGV

IEVTIVAGEGCAVELEHHHHHH
>3H6PC
MSQIMYNYPAMMAHAGDMAGYAGTLQSLGADIASEQAVLSSAWQGDTGITYQGWQTQWNQALEDL
VRAYQSMSGTHESNTMAMLARDGAEAAKWGG
>4EO0A
MDNWESITKSYYTGFAISKTVESKDKDGKPVRKEVITQADLTTACNDAKASAQNVFNQIKLTLSG
TWPNSQFRLVTGDTCVYNGSPGEKTESWSIRAQVEGDIQRSVPDHHHHHH
>2QW5A
GMTKLPATSDIYISFFMFTTNLQPDNLDYRRIVVAHIKKLQRFGYSGFEFPIAPGLPENYAQDLE
NYTNLRHYLDSEGLENVKISTNVGATRTFDPSSNYPEQRQEALEYLKSRVDITAALGGEIMMGPI
VIPYGVFPTTDFNEPIWSDELQEHLKVRYANAQPILDKLGEYAEIKKVKLAIEPITHWETPGPNK
LSQLIEFLKGVKSKQVGVVIDSAHEILDGEGPEIFKTQVEYLAQQGRLHYVQVSPPDRGALHTSW
LPWKSFLTPIVKVYDGPIAVEIFNAIPAFTNSLRLTRRKFWIPDEDPPNQYPNAYDIADEAIKVT
RKELKKIGSK
>3VHJA
MVKNNLGVAVIGSKQYAVNLLWGSSQDTETTNQALNKSLTLMSSKLYSVIGRFQGEQFAVGDKNI
GHKRGQVTLLSAIDFDGSSFCGLFPADNELWLVIGVDKDGMVHFDKSFHSKDDAKKFFFDHVAYG
YPWDRTYSPSDVGVGESRSISELSLIKGKKLKEKGSHHHHHH
>3AMLA
MVTVVEEVDHLPIYDLDPKLEEFKDHFNYRIKRYLDQKCLIEKHEGGLEEFSKGYLKFGINTVDG
ATIYREWAPAAQEAQLIGEFNNWNGAKHKMEKDKFGIWSIKISHVNGKPAIPHNSKVKFRFRHGG
GAWVDRIPAWIRYATFDASKFGAPYDGVHWDPPACERYVFKHPRPPKPDAPRIYEAHVGMSGEEP
EVSTYREFADNVLPRIRANNYNTVQLMAIMEHSYYASFGYHVTNFFAVSSRSGTPEDLKYLVDKA
HSLGLRVLMDVVHSHASNNVTDGLNGYDVGQNTHESYFHTGDRGYHKLWDSRLFNYANWEVLRFL
LSNLRYWMDEFMFDGFRFDGVTSMLYHHHGINKGFTGNYKEYFSLDTDVDAIVYMMLANHLMHKL
LPEATIVAEDVSGMPVLCRPVDEGGVGFDFRLAMAIPDRWIDYLKNKEDRKWSMSEIVQTLTNRR
YTEKCIAYAESHDQSIVGDKTIAFLLMDKEMYTGMSDLQPASPTINRGIALQKMIHFITMALGGD
GYLNFMGNEFGHPEWIDFPREGNNWSYDKCRRQWSLVDTDHLRYKYMNAFDQAMNALEEEFSFLS
SSKQIVSDMNEKDKVIVFERGDLVFVFNFHPNKTYKGYKVGCDLPGKYRVALDSDALVFGGHGRV
GHDVDHFTSPEGMPGVPETNFNNRPNSFKVLSPPRTCVAYYRVDEDREELRRGGAVASGKIVTEY
IDVEATSGETISGGWKGSEKDDCGKKGMKFVFRSSDEDCK
>4E2UA
SQHMAFARDTEVYYENDTVPHMESIEEMYSKYASMNGELPFDNGYAVPLDNVFVYTLDIASGEIK
KTRASYIYREKVEKLIEIKLSSGYSLKVTPSHPVLLFRDGLQWVPAAEVKPGDVVVGVRNGELEF
HEVSSVRIIDYNNWVYDLVIPETHNFIAPNGLVLHNAQ
>1KXOA
DVYHDGACPEVKPVDNFDWSQYHGKWWQVAAYPDHITKYGKCGWAEYTPEGKSVKVSRYSVIHGK
EYFSEGTAYPVGDSKIGKIYHSYTIGGVTQEGVFNVLSTDNKNYIIGYFCSYDEDKKGHMDLVWV
LSRSMVLTGEAKTAVENYLIGSPVVDSQKLVYSDFSEAACKVNNSNWSHPQFEK
>3R41A
GHMPDLADLFPGFGSEWINTSSGRIFARVGGDGPPLLLLHGFPQTHVMWHRVAPKLAERFKVIVA
DLPGYGWSDMPESDEQHTPYTKRAMAKQLIEAMEQLGHVHFALAGHDRGARVSYRLALDSPGRLS
KLAVLDILPTYEYWQRMNRAYALKIYHWSFLAQPAPLPENLLGGDPDFYVKAKLASWTRAGDLSA
FDPRAVEHYRIAFADPMRRHVMCEDYRAGAYADFEHDKIDVEAGNKIPVPMLALWGASGIAQSAA

TPLDVWRKWASDVQGAPIESGNFLPEEAPDQTAEALVRFFSAAPGS

>3FIAA

MGHHHHHHSHVAQFPTPFGGSLDTWAITVEERAKHDQQFHSLKPISGFITGDQARNFFFQSGLPQ
PVLAQIWALADMNNDGRMDQVEFSIAMKLIKLKLQGYQLPSALPPVMKQQPVAISS

>1EAYC

MSQSPRRIILSRLKAGEVDLLEEELGHLTTLTDVVKGADSLSAILPGDIAEDDITAVLCFVIEAD
QITFETVEV

>2Z16A

GSSGSSGMSLLTEVETYVLSIIPSGPLKAEIAQKLEDVFAGKNTDLEALMEWLKTRPILSPLTKG
ILGFVFTLTVPSERGLQRRRFVQNALNGNGDPNNMDRAVKLYKKLKREITFHGAKEVALSYSTGA
LASCMGLIYNRMGTVTTEVAFGLVCATCEQIADSQ

>3BQXA

MSLQQVAVITLGIGDLEASARFYGEGFGWAPVFRNPEIIFYQMNGFVLATWLVQNLQEDVGVAVT
SRPGSMALAHNVRAETEVAPLMERLVAAGGQLLRPADAPPHGGLRGYVADPDGHIWEIAFNPVWP
IGADGSVTFAAKEGHHHHHH

>3CS3A

MSLKRRQTNIIGVYLADYGGSFYGELLEGIKKGLALFDYEMIVCSGKKSHLFIPEKMVDGAIILD
WTFPTKEIEKFAERGHSIVVLDRTTEHRNIRQVLLDNRGGATQAIEQFVNVGSKKVLLLSGPEKG
YDSQERLAVSTRELTRFGIPYEIIQGDFTEPSGYAAAKKILSQPQTEPVDVFAFNDEMAIGVYKY
VAETNYQMGKDIRIIGFDNSELGAFVQPRLATIAYSKHRWGMVAAEKIIHLMRGEAAESEHIYTR
FIEGESFPSEGHHHHHH

>4E3EA

MSAKTNPGNFFEDFRLGQTIVHATPRTITEGDVALYTSLYGSRFALTSSTPFAQSLGLERAPIDS
LLVFHIVFGKTVPDISLNAIANLGYAGGRFGAVVYPGDTLSTTSKVIGLRQNKDGKTGVVYVHSV
GVNQWDEVVLEYIRWVMVRKRDPNAPAPETVVPDLPDSVPVTDLTVPYTVSAANYNLAHAGSNYL
WDDYEVGEKIDHVDGVTIEEAEHMQATRLYQNTARVHFNLHVEREGRFGRRIVYGGHIISLARSL
SFNGLANALSIAAINSGRHTNPSFAGDTIYAWSEILAKMAIPGRTDIGALRVRTVATKDRPCHDF
PYRDAEGNYDPAVVLDFDYTVLMPRRG

>1MG2D

APQFFNIIDGSPLNFDDAMEEGRDTEAVKHFLETGENVYNEDPEILPEAEELYAGMCSGCHGHYA
EGKIGPGLNDAYWTYPGNETDVGLFSTLYGGATGQMGPMWGSLTLDEMLRTMAWVRHLYTGDPKD
ASWLTDEQKAGFTPFQPKSSGEDQS

>3LHIA

GMFVWHEYENAAEAAQSLADAVADALQGALDEKGGAVLAVSGGRSPIAFFNALSQKDLDWKNVGI
TLADERIVPTNHADSNTGLVREYLLKNKAAAAVWIPMVEDGKTETELHPDAVVDYALKHYKQPDV
LILGMGNDGHTASIFPKAPQFQTAIDGSAGVALVHTTPVTAPHERISMTLDAIAHTGHVFLAIQG
EEKKAVFDQAAQGENREYPISLVLNHQGVNCHVFYAE

>2CAYA

MAHHHHHHMEYWHYVETTSSGQPLLREGEKDIFIDQSVGLYHGKSKILQRQRGRIFLTSQRIIYI
DDAKPTQNSLGLELDDLAYVNYSSGFLTRSPRLILFFKDPSSSTEFVQLSFRKSDGVLFSQATER
ALENILTEKNKHIFN

>3K8WA

TQNSLSTSLQRLSSGLRINSAKDDAAGLAISDRMTAQIKGLTQAQRNANDGISLAQTAEGALGEI
SNNLQRIRELAVQASNGTNTQTDRDALQAEVTQLQSEIQRVAEQTSFNGQKLLDGSFNGVQFQIG

ANAGETIGVSKIMNAQTASLGGSLTRTTSTIDATDLTKYDTAMAAGDLTINGVDVGKIDAASTAQ
ERAAQLTEAINRVSSQTNVGASYDKTTGQVTLTSNAAIAVAGAANDATVAGWANNATTGTATTTT
GINSLTVSSFTNAQQTITQIDNALKDINTARADLGAVQNRFTSTVANLQSMTENLSSALEHHHHH
H
>2G38A
MSFVITNPEALTVAATEVRRIRDRAIQSDAQVAPMTTAVRPPAADLVSEKAATFLVEYARKYRQT
IAAAAVVLEEFAHALTTGADKYATAEADNIKTFS
>4HVMA
SNAMTSLSSRPGLRRASFLQRGAWRWLREAPPAAAFAARGLLGSGRIDDDRLAAAADEVLDAFPL
LRVNFVDDDGLWMRTRENADALVRSDLRGHPDPQARCVELLRADRDRPTDPERDPLVRLHLVRLS
ETDVVLGVVAHQMLLDARSRYMVLGAVWQAYYGRFRPAQYRDFAEVADFHPLDRETVRVARHRWW
SRRLPALPVRGGDGGPVGPPETSRLRVPGSRWQALTEPGGPLGGNGSLAMAALTAWWLWTQGAGT
GTDTGAGTGTGKDSLYLSTEVDLRDHLQLGSVVGPLTDRVVFGVDLTGLREPSFRDLMSRTQAGF
LDAVVHYLPYHDVVDLAVDLGVVTPPRVAARWDVAVHLCRNAPSSSLTRGERTLAELGVSIELFR
EADLIGGDTRSATDTWDGTDTWDGTTTDLSVGELGEDMVIVLDQRRTHPAGGGSALLDGLDAAMA
QAVADPSAPLPHSTVDTTTQSTVDKDTVDRNTAHENEE
>2D7VA
MSFGGKSMSEHSAIVTWKRKDSEAFTDNQYSRAHTWEFDGGSKILASASPHVVPVPLSVEANVDP
EEAFVAALSSCHMLVFLSIAAKQRYLVESYTDNAVGILGKNSKGKTSVTKVVLRPQVVFSGTSKP
TLQQLEKMHHLAHENCFIANSVETEVVTEIIA
>2CW6A
TLPKRVKIVEVGPRDGLQNEKNIVSTPVKIKLIDMLSEAGLSVIETTSFVSPKWVPQMGDHTEVL
KGIQKFPGINYPVLTPNLKGFEAAVAAGAKEVVIFGAASELFTKKNINCSIEESFQRFDAILKAA
QSANISVRGYVSCALGCPYEGKISPAKVAEVTKKFYSMGCYEISLGDTIGVGTPGIMKDMLSAVM
QEVPLAALAVHCHDTYGQALANTLMALQMGVSVVDSSVAGLGGCPYAQGASGNLATEDLVYMLEG
LGIHTGVNLQKLLEAGNFICQALNRKTSSKVAQATCKL
>1QWYA
AETTNTQQAHTQMSTQSQDVSYGTYYTIDSNGDYHHTPDGNWNQAMFDNKEYSYTFVDAQGHTHY
FYNCYPKNANANGSGQTYVNPATAGDNNDYTASQSQQHINQYGYQSNVGPDASYYSHSNNNQAYN
SHDGNGKVNYPNGTSNQNGGSASKATASGHAKDASWLTSRKQLQPYGQYHGGGAHYGVDYAMPEN
SPVYSLTDGTVVQAGWSNYGGGNQVTIKEANSNNYQWYMHNNRLTVSAGDKVKAGDQIAYSGSTG
NSTAPHVHFQRMSGGIGNQYAVDPTSYLQSR
>3AMRA
KLSDPYHFTVNAAAETEPVDTAGDAADDPAIWLDPKTPQNSKLITTNKKSGLVVYSLDGKMLHSY
NTGKLNNVDIRYDFPLNGKKVDIAAASNRSEGKNTIEIYAIDGKNGTLQSMTDPDHPIATAINEV
YGFTLYHSQKTGKYYAMVTGKEGEFEQYELKADKNGYISGKKVRAFKMNSQTEGMAADDEYGRLY
IAEEDEAIWKFSAEPDGGSNGTVIDRADGRHLTRDIEGLTIYYAADGKGYLMASSQGNSSYAIYD
RQGKNKYVADFRITDGPETDGTSDTDGIDVLGFGLGPEYPFGIFVAQDGENIDHGQKANQNFKIV
PWERIADQIGFRPLANEQVDPRKLTDRSGK
>4DX5A
MPNFFIDRPIFAWVIAIIIMLAGGLAILKLPVAQYPTIAPPAVTISASYPGADAKTVQDTVTQVI
EQNMNGIDNLMYMSSNSDSTGTVQITLTFESGTDADIAQVQVQNKLQLAMPLLPQEVQQQGVSVE
KSSSSFLMVVGVINTDGTMTQEDISDYVAANMKDAISRTSGVGDVQLFGSQYAMRIWMNPNELNK
FQLTPVDVITAIKAQNAQVAAGQLGGTPPVKGQQLNASIIAQTRLTSTEEFGKILLKVNQDGSRV

LLRDVAKIELGGENYDIIAEFNGQPASGLGIKLATGANALDTAAAIRAELAKMEPFFPSGLKIVY
PYDTTPFVKISIHEVVKTLVEAIILVFLVMYLFLQNFRATLIPTIAVPVVLLGTFAVLAAFGFSI
NTLTMFGMVLAIGLLVDDAIVVVENVERVMAEEGLPPKEATRKSMGQIQGALVGIAMVLSAVFVP
MAFFGGSTGAIYRQFSITIVSAMALSVLVALILTPALCATMLKPIAKGDHGEGKKGFFGWFNRMF
EKSTHHYTDSVGGILRSTGRYLVLYLIIVVGMAYLFVRLPSSFLPDEDQGVFMTMVQLPAGATQE
RTQKVLNEVTHYYLTKEKNNVESVFAVNGFGFAGRGQNTGIAFVSLKDWADRPGEENKVEAITMR
ATRAFSQIKDAMVFAFNLPAIVELGTATGFDFELIDQAGLGHEKLTQARNQLLAEAAKHPDMLTS
VRPNGLEDTPQFKIDIDQEKAQALGVSINDINTTLGAAWGGSYVNDFIDRGRVKKVYVMSEAKYR
MLPDDIGDWYVRAADGQMVPFSAFSSSRWEYGSPRLERYNGLPSMEILGQAAPGKSTGEAMELME
QLASKLPTGVGYDWTGMSYQERLSGNQAPSLYAISLIVVFLCLAALYESWSIPFSVMLVVPLGVI
GALLAATFRGLTNDVYFQVGLLTTIGLSAKNAILIVEFAKDLMDKEGKGLIEATLDAVRMRLRPI
LMTSLAFILGVMPLVISTGAGSGAQNAVGTGVMGGMVTATVLAIFFVPVFFVVVRRRFSRKNEDI
EHSHTVDHHLEHHHHHH
>4A6RA
MQKQRTTSQWRELDAAHHLHPFTDTASLNQAGARVMTRGEGVYLWDSEGNKIIDGMAGLWCVNVG
YGRKDFAEAARRQMEELPFYNTFFKTTHPAVVELSSLLAEVTPAGFDRVFYTNSGSESVDTMIRM
VRRYWDVQGKPEKKTLIGRWNGYHGSTIGGASLGGMKYMHEQGDLPIPGMAHIEQPWWYKHGKDM
TPDEFGVVAARWLEEKILEIGADKVAAFVGEPIQGAGGVIVPPATYWPEIERICRKYDVLLVADE
VICGFGRTGEWFGHQHFGFQPDLFTAAKGLSSGYLPIGAVFVGKRVAEGLIAGGDFNHGFTYSGH
PVCAAVAHANVAALRDEGIVQRVKDDIGPYMQKRWRETFSRFEHVDDVRGVGMVQAFTLVKNKAK
RELFPDFGEIGTLCRDIFFRNNLIMRACGDHIVSAPPLVMTRAEVDEMLAVAERCLEEFEQTLKA
RGLA
>1TUKA
ACQASQLAVCASAILSGAKPSGECCGNLRAQQGCFCQYAKDPTYGQYIRSPHARDTLTSCGLAVP
HC
>4KF8A
SLLPKDISQLLSLVSATINGVDNPWSKDQISYFRTLLKVLFVVLRGTKHSNNAAPQKPTAESPVA
VTQLVLTTLDRVVARSFRNLAALVHEPDAATTPEDLALITAILQACLSVPGIEQCQLQVLNIMSS
HNVLQVATSLFSWSDRLAEKGDPIYGELALLLLLELSALPALAEQLACDGLLGHLTSANLAGFMR
RANVSPFTDNAGAARCYAIWAKGILPLLLNILGALGATIAPEVAFVLNQFPNLLRSSVDRLEAPG
LSRTVPLSSRDAPGAGPHYFVALVALSEVHSLALLTRVLAALRSGNARDIPEVVWDSGAVLENVE
FWLASRKVLRERLLPLNPREAEWRGMKASEGSGCETKLEEKAVGLLEGIRDVLAEEEE
>1FSGA
GSHMASKPIEDYGKGKGRIEPMYIPDNTFYNADDFLVPPHCKPYIDKILLPGGLVKDRVEKLAYD
IHRTYFGEELHIICILKGSRGFFNLLIDYLATIQKYSGRESSVPPFFEHYVRLKSYQNDNSTGQL
TVLSDDLSIFRDKHVLIVEDIVDTGFTLTEFGERLKAVGPKSMRIATLVEKRTDRSNSLKGDFVG
FSIEDVWIVGCCYDFNEMFRDFDHVAVLSDAARKKFEK
>2GUJA
MALKAQNTISGKEGRLFLDGEEMAHIKTFEANVEKNKSEVNIMGRRMTGHKTTGANGTGTATFYK
VTSKFVLLMMDYVKKGSDPYFTLQAVLDDQSSGRGTERVTLYDVNFDSAKIASLDVDSEALEEEV
PFTFEDFDVPEKLSDTFLEHHHHHH
>1O13A
MGSDKIHHHHHHMIIAIPVSENRGKDSPISEHFGRAPYFAFVKVKNNAIADISVEENPLAQDHVH
GAVPNFVKEKGAELVIVRGIGRRAIAAFEAMGVKVIKGASGTVEEVVNQYLSGQLKDSDYEVHDH

HHHEHH
>3NBCA
SITPGTYNITNVAYTNRLIDLTGSNPAENTLIIGHHLNKTPSGYGNQQWTLVQLPHTTIYTMQAV
NPQSYVRVRDDNLVDGAALVGSQQPTPVSIESAGNSGQFRIKIPNLGLALTLPSDANSTPIVLGE
VDETSTNQLWAFESVSAV
>1VK3A
MGSDKIHHHHHHMKLRYLNILKEKLGREPTFVELQAFSVMWSEHCGYSHTKKYIRRLPKTGFEGN
AGVVNLDDYYSVAFKIESHNHPSAIEPYNGAATGVGGIIRDVLAMGARPTAIFDSLHMSRIIDGI
IEGIADYGNSIGVPTVGGELRISSLYAHNPLVNVLAAGVVRNDMLVDSKASRPGQVIVIFGGATG
RDGIHGASFASEDLTGDKATKLSIQVGDPFAEKMLIEAFLEMVEEGLVEGAQDLGAGGVLSATSE
LVAKGNLGAIVHLDRVPLREPDMEPWEILISESQERMAVVTSPQKASRILEIARKHLLFGDVVAE
VIEEPVYRVMYRNDLVMEVPVQLLANAPEEDIVEYTPGKIPEFKRVEFEEVNAREVFEQYDHMVG
TDTVVPPGFGAAVMRIKRDGGYSLVTHSRADLALQDTYWGTLIAVLESVRKTLSVGAEPLAITNC
VNYGDPDVDPVGLSAMMTALKNACEFSGVPVASGNASLYNTYQGKPIPPTLVVGMLGKVNPQKVA
KPKPSKVFAVGWNDFELEREKELWRAIRKLSEEGAFILSSSQLLTRTHVETFREYGLKIEVKLPE
VRPAHQMVLVFSERTPVVDVPVKEIGTLSR
>3BZWA
MSLEQASVTNDFSENKQGCIQHPWQGKKVGYIGDSITDPNCYGDNIKKYWDFLKEWLGITPFVYG
ISGRQWDDVPRQAEKLKKEHGGEVDAILVFMGTNDYNSSVPIGEWFTEQEEQVLSAHGEMKKMVT
RKKRTPVMTQDTYRGRINIGITQLKKLFPDKQIVLLTPLHRSLANFGDKNVQPDESYQNGCGEYI
DAYVQAIKEAGNIWGIPVIDFNAVTGMNPMVEEQLIYFYDAGYDRLHPDTKGQERMARTLMYQLL
ALPVAFEGHHHHHH
>1NEPA
EPVKFKDCGSWVGVIKEVNVSPCPTQPCKLHRGQSYSVNVTFTSNTQSQSSKAVVHGIVMGIPVP
FPIPESDGCKSGIRCPIEKDKTYNYVNKLPVKNEYPSIKVVVEWELTDDKNQRFFCWQIPIEVEA
>1H2SB
GAVFIFVGALTVLFGAIAYGEVTAAAATGDAAAVQEAAVSAILGLIILLGINLGLVAATL
>3IUPA
GMHSALQLRSRIKSSGELELSLDSIDTPHPGPDEVLIRIEASPLNPSDLGLLFGAADMSTAKASG
TAERPIVTARVPEGAMRSMAGRLDASMPVGNEGAGVVVEAGSSPAAQALMGKTVAAIGGAMYSQY
RCIPADQCLVLPEGATPADGASSFVNPLTALGMVETMRLEGHSALVHTAAASNLGQMLNQICLKD
GIKLVNIVRKQEQADLLKAQGAVHVCNAASPTFMQDLTEALVSTGATIAFDATGGGKLGGQILTC
MEAALNKSAREYSRYGSTTHKQVYLYGGLDTSPTEFNRNFGMAWGMGGWLLFPFLQKIGRERANA
LKQRVVAELKTTFASHYSKEISLAEVLDLDMIAVYNKRATGEKYLINPNKGLAG
>1WUBA
MKWNLDPSHTSIDFKVRHMGIASVRGSLKVLSGSVETDEAGRPIQVEAVIDAASIATGEPQRDGH
LRSADFLHAEQYPEIRFVSTQIEPLGGNRYRIQGNLTIRDITKPVTLEAEVSAPIKDPWGMQRVA
ASASGQINRKDWNLTWNQVLELGALLVGEEVKFNLEVEAVAPAPVAAQ
>4EL6A
GADSCKYCLQLYDETYERGSYIEVYKSVGSLSPPWTPGSVCVPFVNDTKRERPYWYLFDNVNYTG
RITGLGHGTCIDDFTKSGFKGISSIKRCIQTKDGKVECINQ
>2Q82A
MDFITDMSKNQRLELQNRLAQYETSLMVMSHNGDVPVITGFNVMRVTTMLDALKVELPAVAVLGD
DAQDLAYVFGARPLAVGVNIIRVVDVPGQQPSALVDAELGALHEVSMVRVLNDIADEQLVKANM

>3GKEA
MATFVRNAWYVAALPEELSEKPLGRTILDTPLALYRQPDGVVAALLDICPHRFAPLSDGILVNGH
LQCPYHGLEFDGGGQCVHNPHGNGARPASLNVRSFPVVERDALIWIWPGDPALADPGAIPDFGCR
VDPAYRTVGGYGHVDCNYKLLVDNLMDLGHAQYVHRANAQTDAFDRLEREVIVGDGEIQALMKIP
GGTPSVLMAKFLRGANTPVDAWNDIRWNKVSAMLNFIAVAPEGTPKEQSIHSRGTHILTPETEAS
CHYFFGSSRNFGIDDPEMDGVLRSWQAQALVKEDKVVVEAIERRRAYVEANGIRPAMLSCDEAAV
RVSREIEKLEQLEAARLEHHHHHH
>1WPXB
XMNQAIDFAQASIDSYKKHGILEDVIHDTSFQPSGILAVEYSSSAPVAMGNTLPTEKARSKPQFQ
FTFNKQMQKSVPQANAYVPQDDDLFTLVMTDPDAPSKTDHKWSEFCHLVECDLKLLNEATHETSG
ATEFFASEFNTKGSNTLIEYMGPAPPKGSGPHRYVFLLYKQPKGVDSSKFSKIKDRPNWGYGTPA
TGVGKWAKENNLQLVASNFFYAETK
>2CSGA
SNAMTTPFTHETLPADPKAAIRQMKQALRAQIGDVQAVFDRLSATIAARVAEINDLKAQGQPVWP
IIPFSELAMGNISDATRAEVKRRGCAVIKGHFPREQALAWDQSMLDYLDKNHFDEVYKGPGDNFF
GTLSASRPEIYPVYWSQAQMQARQSEEMALAQSFLNRLWQVEHDGKRWFNPDISIIYPDRIRRRP
PGTTSKGLGAHTDSGALERWLLPAYQQVFASVFNGNVEQYDPWNAAHRTDVEEYTVDNTTKCSVF
RTFQGWTALSDMLPGQGLLHVVPIPEAMAYILLRPLLDDVPEDELCGVAPGRVLPISEQWHPLLM
AALTSIPPLEAGDSVWWHCDVIHSVAPVENQQGWGNVMYIPAAPMCEKNLAYARKVKAALETGAS
PGDFPREDYETTWEGRFTLRDLNIHGKRALGIDV
>3KG7A
SNASGQQVHRLLGNKLELASTGQTIYHQDINLNNHPWIGDHRVYDTPVIPGVSYIAMTLAAVGVP
AAVEDINFQQPLFLAESNTTRETQLMLHTADNVGKQFVEVFSRDGAKQEEWQQHASMSVSENPPP
PPTLSVDIPALCEQLRPLDTDTLTEIYASISLVYGPMLQAVRQAWIGEETSLLEIEVPKALAFQL
AGEPIHPVLIDACTRLTPDLFDFSSDSGVFWAPWRVKEMTLSHPTPSRFYAYVEEPSRVNEQLQT
RSYDIQLLDETGQAFGRINGFTVKRAPSQLFLK
>2I0MA
MRNQFDLELHELEQSFLGLGQLVLETASKALLALASKDKEMAELIINKDHAINQGQSAIELTCAR
LLALQQPQVSDLRFVISIMSSCSDLERMGDHMAGIAKAVLQLKENQLAPDEEQLHQMGKLSLSML
ADLLVAFPLHQASKAISIAQKDEQIDQYYYALSKEIIGLMKDQETSIPNGTQYLYIIGHLERFAD
YIANICERLVYLETGELVDLN
>3IAXB
MPGFNYGGKGDGTGWSSERGSGPEPGGGSHGNSGGHDRGDSSNVGNESVTVMKPGDSYNTPWGKV
IINAAGQPTMNGTVMTADNSSMVPYGRGFTRVLNSLVNNPVSLEHHHHHH
>1D2SA
PPAVHLSNGPGQEPIAVMTFDLTKITKTSSSFEVRTWDPEGVIFYGDTNPKDDWFMLGLRDGRPE
IQLHNHWAQLTVGAGPRLDDGRWHQVEVKMEGDSVLLEVDGEEVLRLRQVSGHPIMRIALGGLLF
PASNLRLPLVPALDGCLRRDSWLDKQAEISASAPTSLRSC
>4A0GA
GSSHHHHHHSSGLVPRGSHMKSTSVSPFHLPLNHPTYLIWSANTSLGKTLVSTGIAASFLLQQPS
SSATKLLYLKPIQTGFPSDSDSRFVFSKLDSLSLRRQIPISISNSVLHSSLPAAKSLGLNVEVSE
SGMCSLNFRDEKTVTGAPELLCKTLYAWEAAISPHLAAERENATVEDSVVLQMIEKCLKEEMECG
VKSEKSDLLCLVETAGGVASPGPSGTLQCDLYRPFRLPGILVGDGRLGGISGTIAAYESLKLRGY
DIAAVVFEDHGLVNEVPLTSYLRNKVPVLVLPPVPKDPSDDLIEWFVESDGVFKALKETMVLANL

ERLERLNGMAKLAGEVFWWPFTQHKLVHQETVTVIDSRCGENFSIYKASDNSSLSQQFDACASWW
TQGPDPTFQAELAREMGYTAARFGHVMFPENVYEPALKCAELLLDGVGKGWASRVYFSDNGSTAI
EIALKMAFRKFCVDHNFCEATEEEKHIVVKVIALRGSYHGDTLGAMEAQAPSPYTGFLQQPWYTG
RGLFLDPPTVFLSNGSWNISLPESFSEIAPEYGTFTSRDEIFDKSRDASTLARIYSAYLSKHLQE
HSGVRQSAHVGALIIEPVIHGAGGMHMVDPLFQRVLVNECRNRKIPVIFDEVFTGFWRLGVETTT
ELLGCKPDIACFAKLLTGGMVPLAVTLATDAVFDSFSGDSKLKALLHGHSYSAHAMGCATAAKAI
QWFKDPETNHNITSQGKTLRELWDEELVQQISSHSAVQRVVVIGTLFALELKADASNSGYASLYA
KSLLIMLREDGIFTRPLGNVIYLMCGPCTSPEICRRLLTKLYKRLGEFNRT
>1SU8A
MAKQNLKSTDRAVQQMLDKAKREGIQTVWDRYEAMKPQCGFGETGLCCRHCLQGPCRINPFGDEP
KVGICGATAEVIVARGLDRSIAAGAAGHSGHAKHLAHTLKKAVQGKAASYMIKDRTKLHSIAKRL
GIPTEGQKDEDIALEVAKAALADFHEKDTPVLWVTTVLPPSRVKVLSAHGLIPAGIDHEIAEIMH
RTSMGCDADAQNLLLGGLRCSLADLAGCYMGTDLADILFGTPAPVVTESNLGVLKADAVNVAVHG
HNPVLSDIIVSVSKEMENEARAAGATGINVVGICCTGNEVLMRHGIPACTHSVSQEMAMITGALD
AMILDYQCIQPSVATIAECTGTTVITTMEMSKITGATHVNFAEEAAVENAKQILRLAIDTFKRRK
GKPVEIPNIKTKVVAGFSTEAIINALSKLNANDPLKPLIDNVVNGNIRGVCLFAGCNNVKVPQDQ
NFTTIARKLLKQNVLVVATGCGAGALMRHGFMDPANVDELCGDGLKAVLTAIGEANGLGGPLPPV
LHMGSCVDNSRAVALVAALANRLGVDLDRLPVVASAAEAMHEKAVAIGTWAVTIGLPTHIGVLPP
ITGSLPVTQILTSSVKDITGGYFIVELDPETAADKLLAAINERRAGLGLPW
>4AKFA
GSMKLAEIMTKSRKLKRNLLEISKTEAGQYSVSAPEHKGLVLSGGGAKGISYLGMIQALQERGKI
KNLTHVSGASAGAMTASILAVGMDIKDIKKLIEGLDITKLLDNSGVGFRARGDRFRNILDVIYMM
QMKKHLESVQQPIPPEQQMNYGILKQKIALYEDKLSRAGIVINNVDDIINLTKSVKDLEKLDKAL
NSIPTELKGAKGEQLENPRLTLGDLGRLRELLPEENKHLIKNLSVVVTNQTKHELERYSEDTTPQ
QSIAQVVQWSGAHPVLFVPGRNAKGEYIADGGILDNMPEIEGLDREEVLCVKAEAGTAFEDRVNK
AKQSAMEAISWFKARMDSLVEATIGGKWLHATSSVLNREKVYYNIDNMIYINTGEVTTTNTSPTP
EQRARAVKNGYDQTMQLLDSHKQTFDHPLMAILYIGHDKLKDALIDEKSEKEIFEASAHAQAILH
LQEQIVKEMNDGDYSSVQNYLDQIEDILTVDAKMDDIQKEKAFALCIKQVNFLSEGKLETYLNKV
EAEAKAAAEPSWATKILNLLWAPIEWVVSLFKGPAQDFKVEVQPEPVKVSTSENQET
>3EF8A
GMTDTNLVEMRAIERMMFDYSYHLDMNHPEELAALFVEDCEVSYAPNFGATGRDAYKKTLEGIGT
FFRGTSHHNSNICIDFVSETEANVRSVVLAIHRYTKERPDGILYGQYFDTVVKVDGQWKFKRREL
RTTMTTDYHVRAANPIGRAE
>1PIWA
MSYPEKFEGIAIQSHEDWKNPKKTKYDPKPFYDHDIDIKIEACGVCGSDIHCAAGHWGNMKMPLV
VGHEIVGKVVKLGPKSNSGLKVGQRVGVGAQVFSCLECDRCKNDNEPYCTKFVTTYSQPYEDGYV
SQGGYANYVRVHEHFVVPIPENIPSHLAAPLLCGGLTVYSPLVRNGCGPGKKVGIVGLGGIGSMG
TLISKAMGAETYVISRSSRKREDAMKMGADHYIATLEEGDWGEKYFDTFDLIVVCASSLTDIDFN
IMPKAMKVGGRIVSISIPEQHEMLSLKPYGLKAVSISYSALGSIKELNQLLKLVSEKDIKIWVET
LPVGEAGVHEAFERMEKGDVRYRFTLVGYDKEFSD
>4J4HA
MALLTPDDLININMQLQKADSAVQEVTGLDIKGICKALYGTFSSSEKVGIVPVTSGNGIIGNFSA
SLHAITQYFGFDSFVTDMPDVSGYYEAVQNGAEIILMADDRTFLAHNLKNGKMANNQPCTGIIYA
EIASRYLKADSKDVLVVGLGKVGFPGAEHLVQKDFRVYGYDADETLLERATSNLGIIPFDPANPK

KFSIIFEATPCANTIPEAVLSENCVLSTPGIPCAISEELRDKYEVQLIAEPLGIGTASMLYSVL
>1QWRA
SNAMTQSPIFLTPVFKEKIWGGTALRDRFGYSIPSESTGECWAISAHPKGPSTVANGPYKGKTLI
ELWEEHREVFGGVEGDRFPLLTKLLDVKEDTSIKVHPDDYYAGENEEGELGKTECWYIIDCKENA
EIIYGHTARSKTELVTMINSGDWEGLLRRIKIKPGDFYYVPSGTLHALCKGALVLETQQNSDATY
RVYDYDRLDSNGSPRELHFAKAVNAATVPHVDGYIDESTESRKGITIKTFVQGEYFSVYKWDING
EAEMAQDESFLICSVIEGSGLLKYEDKTCPLKKGDHFILPAQMPDFTIKGTCTLIVSHI
>2GX5A
MGSSHHHHHHMALLQKTRIINSMLQAAAGKPVNFKEMAETLRDVIDSNIFVVSRRGKLLGYSINQ
QIENDRMKKMLEDRQFPEEYTKNLFNVPETSSNLDINSEYTAFPVENRDLFQAGLTTIVPIIGGG
ERLGTLILSRLQDQFNDDDLILAEYGATVVGMEILREKAE
>2Q2RA
APAGSHMNIKELSLHELCEELKTPAWNAPLTFVGDVGGTSARMGFVREGKNDSVHACVTRYSMKR
KDITEIIEFFNEIIELMPASVMKRVKAGVINVPGPVTGGAVGGPFNNLKGIARLSDYPKALFPPG
HSAILNDLEAGGFGVLAVSDAHVFSEYFGVMWEGTQWRTCEQEPAGSVIGRGRCLVLAPGTGLGS
SLIYYNPPMNQHIVVPLELGSQTLPMRKDIDYIQTLHAELKLFPNYENMVSGAGLEFHYRQVVRG
SRPPCSAGEIAKLASEGDANACKAMKKYHEYLMRVGSEASMALLPLTIVLVGDNIVNNAFFYRNP
QNLKEMHHEALNHEMERFGFQSRVSYLRQKKLLNLNLMGCYRCGLDLS
>3SXOA
MGSSHHHHHHSSGLVPRGSHMPQSDSVTVTLCSPTEDDWPGMFLLAAASFTDFIGPESATAWRTL
VPTDGAVVVRDGAGPGSEVVGMALYMDLRLTVPGEVVLPTAGLSFVAVAPTHRRRGLLRAMCAEL
HRRIADSGYPVAALHASEGGIYGRFGYGPATTLHELTVDRRFARFHADAPGGGLGGSSVRLVRPT
EHRGEFEAIYERWRQQVPGGLLRPQVLWDELLAECKAAPGGDRESFALLHPDGYALYRVDRTDLK
LARVSELRAVTADAHCALWRALIGLDSMERISIITHPQDPLPHLLTDTRLARTTWRQDGLWLRIM
NVPAALEARGYAHEVGEFSTVLEVSDGGRFALKIGDGRARCTPTDAAAEIEMDRDVLGSLYLGAH
RASTLAAANRLRTKDSQLLRRLDAAFASDVPVQTAFEF
>2PH0A
MTMTLNELLATNPDGTLEDIAGKYNTSLFAVVEALPTAQCTLATGDRFDQVWDTIATWGEVTLIS
HTADAILEFKSELPTGTHRHGYFNLRGKNGLSGHIRATSCQHIAFIERKFMGMDTASVVFFNANG
AAMFKIFLGRDSHRQLLSAQVDAFRALASELQPEQVLEHHHHHH
>1G73A
AVPIAQKSEPHSLSSEALMRRAVSLVTDSTSTDLSQTTYALIEAITEYTKAVYTLTSLYRQYTSL
LGKMNSEEEDEVWQVIIGARAEMTSKHQEYLKLETTWMTAVGLSEMAAEAAYQTGADQASITARN
HIQLVKLQVEEVHQLSRKAETKLAEAQIEELR
>2B97A
AVCPTGLFSNPLCCATNVLDLIGVDCKTPTIAVDTGAIFQAHCASKGSKPLCCVAPVADQALLCQ
KAIGTF
>1Q9JA
MFPGSVIRKLSHSEEVFAQYEVFTSMTIQLRGVIDVDALSDAFDALLETHPVLASHLEQSSDGGW
NLVADDLLHSGICVIDGTAATNGSPSGNAELRLDQSVSLLHLQLILREGGAELTLYLHHCMADGH
HGAVLVDELFSRYTDAVTTGDPGPITPQPTPLSMEAVLAQRGIRKQGLSGAERFMSVMYAYEIPA
TETPAVLAHPGLPQAVPVTRLWLSKQQTSDLMAFGREHRLSLNAVVAAAILLTEWQLRNTPHVPI
PYVYPVDLRFVLAPPVAPTEATNLLGAASYLAEIGPNTDIVDLASDIVATLRADLANGVIQQSGL
HFGTAFEGTPPGLPPLVFCTDATSFPTMRTPPGLEIEDIKGQFYCSISVPLDLYSCAVYAGQLII

EHHGHIAEPGKSLEAIRSLLCTVPSEYGWIME
>1RYP1
QFNPYGDNGGTILGIAGEDFAVLAGDTRNITDYSINSRYEPKVFDCGDNIVMSANGFAADGDALV
KRFKNSVKWYHFDHNDKKLSINSAARNIQHLLYGKRFFPYYVHTIIAGLDEDGKGAVYSFDPVGS
YEREQCRAGGAAASLIMPFLDNQVNFKNQYEPGTNGKVKKPLKYLSVEEVIKLVRDSFTSATERH
IQVGDGLEILIVTKDGVRKEFYELKRD
>3FBIA
MASNDPGNEVSSLYPPPPPYVKFFTQSNLEKLPKYKEKKAASAKQTAPNNSNGGSEEEITCALDY
LIPPPMPKNQQYRAFGSIW
>4DMTA
XGPPGPPGPPGPRGQPGVMGFPGPPGPPGPPX
>3ZXKA
MPRQASTFTNPVLWEDHPALEVFRVGSVFYYSSSTFAYSPGAPVLKSYDLVHWTPVTHSVPRLNF
GSNYDLPSGTPGAYVKGIWASTLRYRRSNDRFYWYGCVEGRTYLWTSPGGNALANNGEVPPSAWN
WQHTATIDNCYYDAGLLIDDDDTMYIAYGNPTINVAQLSPDGTRQVRVQQRVYAHPQGQTVEGAR
MYKIRGNYYILVTRPADAEYVLRSTTGSPFGPYEARTLVSRIQGPLANAGFAHQGGIVDAPDGTW
HYVAFMDAYPGGRIPVVAPLRWTADGWPEVVTDSQGRWGTSYPIPVRGAKNATEGLASTDLDEFR
GTRFSEHWEWNHNPDTSKFTLLGGNEGGLILRTATVTGDLFAARNTLTRRIAGPKASGIFRLDVR
GMRDGDRAGAVLFRDRAAYIGVWKQGNEARIVMVDDLRLNEDGWRTASTGRVAANGPVIDTNAQQ
DIWLRIDADITPAFGTNTERTTTFYYSIDGGRTYTRLGPAFAMTNSWRYFTGYRFGVFNFSTKSL
GGEVKVKGFKMNMILEHHHHHH
>2NXPA
GSVAVEDQPDVSAVLSAYNQQGDPTMYEEYYSGLKHFIECSLDCHRAELSQLFYPLFVHMYLELV
YNQHENEAKSFFEKFHGDQECYYQDDLRVLSSLTKKEHMKGNETMLDFRTSKFVLRISRDSYQLL
KRHLQEKQNNQIWNIVQEHLYIDIFD
>2GRRB
STGEPAPVLSSPPPADVSTFLAFPSPEKLLRLGPKSSVLIAQQTDTSDPEKVVSAFLKVSSVFKD
EATVRMAVQDAVDALMQKAFNSSSFNSNTFLTRLLVHMGLLKSEDKVKAIANLYGPLMALNHMVQ
QDYFPKALAPLLLAFVTKPNSALESCSFARHSLLQTLYKV
>2WGKA
MAMETGLIFHPYMRPGRSARQTFDWGIKSAVQADSVGIDSMMISEHASQIWENIPNPELLIAAAA
LQTKNIKFAPMAHLLPHQHPAKLATMIGWLSQILEGRYFLGIGAGAYPQASYMHGIRNAGQSNTA
TGGEETKNLNDMVRESLFIMEKIWKREPFFHEGKYWDAGYPEELEGEEGDEQHKLADFSPWGGKA
PEIAVTGFSYNSPSMRLAGERNFKPVSIFSGLDALKRHWEVYSEAAIEAGHTPDRSRHAVSHTVF
CADTDKEAKRLVMEGPIGYCFERYLIPIWRRFGMMDGYAKDAGIDPVDADLEFLVDNVFLVGSPD
TVTEKINALFEATGGWGTLQVEAHDYYDDPAPWFQSLELISKEVAPKILLPKR
>3SK2A
GSHMTDLAGPTITPNLQLVYVSNVERSTDFYRFIFKKEPVFVTPRYVAFPSSGDALFAIWSGGEE
PVAEIPRFSEIGIMLPTGEDVDKLFNEWTKQKSHQIIVIKEPYTDVFGRTFLISDPDGHIIRVCP
LD
>1E29A
VELTESTRTIPLDEAGGTTTLTARQFTNGQKIFVDTCTQCHLQGKTKTNNNVSLGLADLAGAEPR
RDNVLALVEFLKNPKSYDGEDDYSELHPNISRPDIYPEMRNYTEDDIFDVAGYTLIAPKLDERWG
GTIYF

>4F7HA
SSIMTSENHLNNSDKEVDEVDAALSDLEITLEGGKTSTILGDITSIPELADYIKVFKPKKLTLKG
YKQYWCTFKDTSISCYKSKEESSGTPAHQMNLRGCEVTPDVNISGQKFNIKLLIPVAEGMNEIWL
RCDNEKQYAHWMAACRLASKGKTMADSSYNLEVQNILSFLKMQ
>1EXTA
MDSVCPQGKYIHPQNNSICCTKCHKGTYLYNDCPGPGQDTDCRECESGSFTASENHLRHCLSCSK
CRKEMGQVEISSCTVDRDTVCGCRKNQYRHYWSENLFQCFNCSLCLNGTVHLSCQEKQNTVCTCH
AGFFLRENECVSCSNCKKSLECTKLCLPQIEN
>2X7QA
MAHHHHHHGHHHQLPTLKVAYIPEHFSTPLFFAQQQGYYKAHDLSIEFVKVPEGSGRLINLLNSN
EVDIAIGLTEAFIADIAKGNENIHVLDTYVKSPLLWAVSTGSNRDDVTDAKQLKRIGVSRIGSGS
YVMSFVLAHQLGVPSFDQFQVLSNFKNLRDSVNLKDGVEGSDAFMWEYFTSKKYYDNHEIKQIDQ
IYTPWSSWVVATSSDSLQAKSDVIKNFIDAVNQGIQYYNEHVDEAIEYISSNLDYSAEDAKEWTK
TVEFNSRIGKTPLDWDTIVVKTKDTLKLAGVLAESDDVILKRLNSNVKKTNLQLDGDLEAA
>1L1LA
MSEEISLSAEFIDRVKASVKPHWGKLGWVTYKRTYARWLPEKGRSENWDETVKRVVEGNINLDPR
LQDSPSLELKQSLTEEAERLYKLIYGLGATPSGRNLWISGTDYQRRTGDSLNNCWFVAIRPQKYG
DSKIVPSYLGKQEKAVSMPFSFLFDELMKGGGVGFSVARSNISQIPRVDFAIDLQLVVDETSESY
DASVKVGAVGKNELVQDADSIYYRLPDTREGWVLANALLIDLHFAQTNPDRKQKLILDLSDIRPY
GAEIHGFGGTASGPMPLISMLLDVNEVLNNKAGGRLTAVDAADICNLIGKAVVAGNVRRSAELAL
GSNDDQDFISMKQDQEKLMHHRWASNNSVAVDSAFSGYQPIAAGIRENGEPGIVNLDLSKNYGRI
VDGYQAGIDGDVEGTNPCGEISLANGEPCNLFEVFPLIAEEQGWDLQEVFALAARYAKRVTFSPY
DWEISREIIQKNRRIGISMSGIQDWLLTRLGNRVVTGFKDDFDPETHEAIKVPVYDKRAIKMVDQ
LYKAVVKADQDYSKTLGCNESIKHTTVKPSGTVAKLAGASEGMHFHYGAYLIQRIRFQDSDPLLP
ALKACGYRTEADIYTENTTCVEFPIKAVGADNPNFASAGTVSIAEQFATQAFLQTYWSDNAVSCT
ITFQDSEGDQVESLLRQYRFITKSTSLLPYFGGSLQQAPKEPIDKETYEKRSQEITGNVEEVFSQ
LNSDVKDLELVDQTDCEGGACPIK
>2I7RA
SNAMNLNQLDIIVSNVPQVCADLEHILDKKADYANDGFAQFTIGSHCLMLSQNHLVPLENFQSGI
IIHIEVEDVDQNYKRLNELGIKVLHGPTVTDWGTESLLVQGPAGLVLDFYRMK
>3SCYA
GAQPTDPSTTDSELTMLVGTYTSGNSKGIYTFRFNEETGESLPLSDAEVANPSYLIPSADGKFVY
SVNEFSKDQAAVSAFAFDKEKGTLHLLNTQKTMGADPCYLTTNGKNIVTANYSGGSITVFPIGQD
GALLPASDVIEFKGSGPDKERQTMPHLHCVRITPDGKYLLADDLGTDQIHKFNINPNANADNKEK
FLTKGTPEAFKVAPGSGPRHLIFNSDGKFAYLINEIGGTVIAFRYADGMLDEIQTVAADTVNAQG
SGDIHLSPDGKYLYASNRLKADGVAIFKVDETNGTLTKVGYQLTGIHPRNFIITPNGKYLLVACR
DTNVIQIFERDQATGLLTDIKKDIKVDKPVCLKFVD
>3O12A
MGSSHHHHHHSSGRENLYFQGMVESKNTELSQGTWLNKPKSVFQEAGKVTLETDEKTDFWRETFY
GFTRDSGHFLGVETGSAFTAQVRVQGSYESLYDQAGIMVRIDDGHWLKAGIEISDGHAMLSSVLT
NGKSDWSTAVYGGNARDFWLRVTVEKGVLRIQVSSDKKTWPLVRLAPFPTSDHYLVGPMACTPER
GGLKVTFSEWSLTAPLGKALHDLSGS
>1EDMB
VDGDQCESNPCLNGGSCKDDINSYECWCPFGFEGKNCEL

>2QL8A
GMQDERWNHPLYTTTAINDEELEGHAYIPGGLKVQTSSPMNDHPGTNPEQLLGLSLSTCLEATLE
AVEKEHGLPHTGAVRVKVAFIGARAEYQFLVHAQVMVKGVDFDTAKAFTNEIENRCPVSKLLKNS
GNYTIETVTDFKD
>1U5HA
MNLRAAGPGWLFCPADAPEAFAAAAAAADVVILDLEDGVAEAQKPAARNALRDTPLDPERTVVRI
NAGGTADQARDLEALAGTAYTTVMLPKAESAAQVIELAPRDVIALVETARGAVCAAEIAAADPTV
GMMWGAEDLIATLGGSSSRRADGAYRDVARHVRSTILLAASAFGRLALDAVHLDILDVEGLQEEA
RDAAAVGFDVTVCIHPSQIPVVRKAYAASHEKLAWARRVLAASRSERGAFAFEGQMVDSPVLTHA
ETMLRRAGEATSE
>3CJYA
GMTEAFPALVRQDDARYAITVGPDLAVGPPGHAYLFGGASMALALDVAAETVGRPVVQGSLQFVS
FTPLGSVLDLTVEVLQSGRTLAQARVAGTVDGRLVFHSGISLGMREGFSARQWALAPPVPQPDNC
PPCTTLPAQDDNARYLEGIEVREAGGPEVPSGRTRLWLRRKDGAPLDAASLAMFADFLPIALGRA
TGCSGGGNSLDNSLRITGAAAPGWCLCDMIIPSSASGFAQGQVTLWDQSGRLLATGAQSLLLKG
>3MAYA
GASDPCAASEVARTVGSVAKSMGDYLDSHPETNQVMTAVLQQQVGPGSVASLKAHFEANPKVASD
LHALSQPLTDLSTRCSLPISGLQAIGLMQAVQGARR
>2VRSA
ILQTTVDGNSTAISNLKSDISSNGLAITDLQDRVKSLESTASHGLSFSPPLSVADGVVSLDMDPY
FCSQRVSLTSYSAEAQLMQFRWMARGTNGSSDTIDMTVNAHCHGRRTDYMMSSTGNLTVTSNVVL
LTFDLSDITHIPSDLARLVPSAGFQAASFPVDVSFTRDSATHAYQAYGVYSSSRVFTITFPTGGD
GTANIRSLTVRTGIDT
>2HA9A
AMDIRQVTETIAMIEEQNFDIRTITMGISLLDCIDPDINRAAEKIYQKITTKAANLVAVGDEIAA
ELGIPIVNKRVSVTPISLIGAATDATDYVVLAKALDKAAKEIGVDFIGGFSALVQKGYQKGDEIL
INSIPRALAETDKVCSSVNIGSTKSGINMTAVADMGRIIKETANLSDMGVAKLVVFANAVEDNPF
MAGAFHGVGEADVIINVGVSGPGVVKRALEKVRGQSFDVVAETVKKTAFKITRIGQLVGQMASER
LGVEFGIVDLSLAPTPAVGDSVARVLEEMGLETVGTHGTTAALALLNDQVKKGGVMACNQVGGLS
GAFIPVSEDEGMIAAVQNGSLNLEKLEAMTAICSVGLDMIAIPEDTPAETIAAMIADEAAIGVIN
MKTTAVRIIPKGKEGDMIEFGGLLGTAPVMKVNGASSVDFISRGGQIPAPIHSFKN
>1RYPK
MDIILGIRVQDSVILASSKAVTRGISVLKDSDDKTRQLSPHTLMSFAGEAGDTVQFAEYIQANIQ
LYSIREDYELSPQAVSSFVRQELAKSIRSRRPYQVNVLIGGYDKKKNKPELYQIDYLGTKVELPY
GAHGYSGFYTFSLLDHHYRPDMTTEEGLDLLKLCVQELEKRMPMDFKGVIVKIVDKDGIRQVDDF
QAQ
>4DOYA
MGSSHHHHHHSSGLVPRGSHMTLSPEKQHVRPRDAADNDPVAVARGLAEKWRATAVERDRAGGSA
TAEREDLRASGLLSLLVPREYGGWGADWPTAIEVVREIAAADGSLGHLFGYHLTNAPMIELIGSQ
EQEEHLYTQIAQNNWWTGNASSENNSHVLDWKVSATPTEDGGYVLNGTKHFCSGAKGSDLLFVFG
VVQDDSPQQGAIIAAAIPTSRAGVTPNDDWAAIGMRQTDSGSTDFHNVKVEPDEVLGAPNAFVLA
FIQSERGSLFAPIAQLIFANVYLGIAHGALDAAREYTRTQARPWTPAGIQQATEDPYTIRSYGEF
TIALQGADAAAREAAHLLQTVWDKGDALTPEDRGELMVKVSGVKALATNAALNISSGVFEVIGAR
GTHPRYGFDRFWRNVRTHSLHDPVSYKIADVGKHTLNGQYPIPGFTS

>1UHVA
MIKVRVPDFSDKKFSDRWRYCVGTGRLGLALQKEYIETLKYVKENIDFKYIRGHGLLCDDVGIYR
EDVVGDEVKPFYNFTYIDRIFDSFLEIGIRPFVEIGFMPKKLASGTQTVFYWEGNVTPPKDYEKW
SDLVKAVLHHFISRYGIEEVLKWPFEIWNEPNLKEFWKDADEKEYFKLYKVTAKAIKEVNENLKV
GGPAICGGADYWIEDFLNFCYEENVPVDFVSRHAYTSKQGEYTPHLIYQEIMPSEYMLNEFKTVR
EIIKNSHFPNLPFHITEYNTSYSPQNPVHDTPFNAAYIARILSEGGDYVDSFSYWTFSDVFEERD
VPRSQFHGGFGLVALNMIPKPTFYTFKFFNAMGEEMLYRDEHMLVTRRDDGSVALIAWNEVMDKT
ENPDEDYEVEIPVRFRDVFIKRQLIDEEHGNPWGTWIHMGRPRYPSKEQVNTLREVAKPEIMTSQ
PVANDGYLNLKFKLGKNAVVLYELTERIDESSTYIGLDDSKINGY
>3LEDA
MGSSHHHHHHSSGRENLYFQGVRPAVIAATGLYTPPDSVSNAELVEAFNTYVANFNAANKARIEA
GEIEPLQPSSSEFIEKASGIKSRYVVAKPGIVDPDVMRPIIPERSNDELSILAEMAVTAAEQAIE
RWGKPRERIGAVLCACSNMQRAYPAMAIEVQNALGLGGFAFDMNVACSSATFGLKTAADFVGGGS
VDAVLMVNPEICSGHLNFRDRDSHFIFGDVATAAIVERADDAQGGWSILGTKLKTQFSNNIRNNA
GFLNRAWPEGRDKADKLFVQQGRKVFKEVVPLVSEMIIEHAREIGIDPHGLKRMWLHQANINMNE
IIGRKVLGRDPTRDENVIILDDYANTSSAGSIIAFHKHQDDMAQGDLGLICSFGAGYSAGTVFVQ
KR
>1QQP3
GIFPVACSDGYGGLVTTDPKTADPVYGKVFNPPRNQLPGRFTNLLDVAEACPTFLRFEGGVPYVT
TKTDSDRVLAQFDMSLAAKHMSNTFLAGLAQYYTQYSGTINLHFMFTGPTDAKARYMVAYAPPGM
EPPKTPEAAAHCIHAEWDTGLNSKFTFSIPYLSAADYTYTASDVAETTNVQGWVCLFQITHGKAD
GDALVVLASAGKDFELRLPVDARAE
>2UW1A
MQVTHSMPPQKLEIFKSLDDWARNNVLIHLKSVEKSWQPQDYLPDPVSDGFEEQVRELRERAKEI
PDDYFVVLVGDMITEEALPTYMSMLNRCDGIKDETGAEPSAWAMWTRAWTAEENRHGDLLNKYLY
LSGRVDMRKIEKTIQYLIGSGMDIKSENSPYLGFIYTSFQERATFISHANTAKLAQHYGDKKLAH
ICGSIASDEKRHATAYTKIVEKLAEIDPDTTVIAFADMMRKKITMPAHLMYDGSDELLFKHFTAV
AQRLGVYSALDYCDILEFLVDKWNVERLTGLSDEGRKAQEYVCELGPKIRRLEERAQGRAKEAPT
MPFSWIFDRQVKL
>2FE8A
MEVKTIKVFTTVDNTNLHTQLVDMSMTYGQQFGPTYLDGADVTKIKPHVNHEGKTFFVLPSDDTL
RSEAFEYYHTLDESFLGRYMSALNHTKKWKFPQVGGLTSIKWADNNCYLSSVLLALQQLEVKFNA
PALQEAYYRARAGDAANFCALILAYSNKTVGELGDVRETMTHLLQHANLESAKRVLNVVCKHCGQ
KTTTLTGVEAVMYMGTLSYDNLKTGVSIPCVCGRDATQYLVQQESSFVMMSAPPAEYKLQQGTFL
CANEYTGNYQCGHYTHITAKETLYRIDGAHLTKMSEYKGPVTDVFYKETSYTTTI
>3ETVA
GAMGMNGIDDLLNINDRIKQVQNERNELASKLQNLKQSLASNDTGGGSGGGSDSSDLLQREAILA
NELNILDNLKTFLNLIKEVKTNLNILELENCYYSLQSLRKKMRNNAAYLKQSFNFQQSISTYVDT
LHLELVSTLYKILTNGFWKITENSIQFTPTVEWGKDKVHIEYDTFMDFVAQQYFPKGSLDNQAWF
ILDMTSADSQEQVRAKLNTIMKEYMNLSRIVSMIKNSIFISGKEISYENEKNILVFSKSSSHGQH
CVSTVLTSFEAVCDFMLDGLAFRDRKTLSYELGPLFNTEFTKFVKNNASIILESLDSPLKNLVSV
INNKLTRLVAKSEVTNWTHSGKEIQDLLMN
>4EZIA
GALEHEKLVNYIALGEFSRETAEIALKKMPPLDTLTVHYDLQLYKINYKTQSPDGNLTIASGLVA

MPIHPVGQVGIISYQHGTRFERNDVPSRNNEKNYIYLAAYGNSAGYMTVMPDYLGLGDNELTLHP
YVQAETLASSSIDMLFAAKELANRLHYPISDKLYLAGYSEGGFSTIVMFEMLAKEYPDLPVSAVA
PGSAPYGWEETMHFVMLEPGPRATAYLAYFFYSLQTYKSYWSGFDEIFAPPYNTLIPELMDGYHA
VDEILQALPQDPLLIFQPKFSNGIISKTDRNTEILKINFNHYDFKPTAPLLLVGTKGDRDVPYAG
AEMAYHSFRKYSDFVWIKSVSDALDHVQAHPFVLKEQVDFFKQFERQEAMNK
>4AXOA
MGQIIEEKISGTKDTVDFVRNKDISGITSIKLPTVKVSESDRLDTGNPSDVVYTKDLFTLEESPR
LGCGMMEMKETTFDWTLNYDEIDYVIDGTLDIIIDGRKVSASSGELIFIPKGSKIQFSVPDYARF
IYVTYPADWASQNLEHHHHHH
>3H9WA
MTKAIPWKINWQTMAFEYIGPQIEALLGWPQGSWKSVEDWATRMHPEDQEWVVNFCVKQSECGVD
HEADYRALHRDGHYVWIRDVVHVVRDDSGEVEALIGFMFDISLEHHHHHH
>1THTA
MNNQCKTIAHVLRVNNGQELHVWETPPKENVPFKNNTILIASGFARRMDHFAGLAEYLSTNGFHV
FRYDSLHHVGLSSGSIDEFTMTTGKNSLCTVYHWLQTKGTQNIGLIAASLSARVAYEVISDLELS
FLITAVGVVNLRDTLEKALGFDYLSLPIDELPNDLDFEGHKLGSEVFVRDCFEHHWDTLDSTLDK
VANTSVPLIAFTANNDDWVKQEEVYDMLAHIRTGHCKLYSLLGSSHDLGENLVVLRNFYQSVTKA
AIAMDGGSLEIDVDFIEPDFEQLTIATVNERRLKAEIENRTPEMA
>4G55A
GSPEFMAQILPIRFQEHLQLQNLGINPANIGFSTLTMESDKFICIREKVGEQAQVVIIDMNDPSN
PIRRPISADSAIMNPASKVIALKAGKTLQIFNIEMKSKMKAHTMTDDVTFWKWISLNTVALVTDN
AVYHWSMEGESQPVKMFDRHSSLAGCQIINYRTDAKQKWLLLTGISAQQNRVVGAMQLYSVDRKV
SQPIEGHAASFAQFKMEGNAEESTLFCFAVRGQAGGKLHIIEVGTPPTGNQPFPKKAVDVFFPPE
AQNDFPVAMQISEKHDVVFLITKYGYIHLYDLETGTCIYMNRISGETIFVTAPHEATAGIIGVNR
KGQVLSVCVEEENIIPYITNVLQNPDLALRMAVRNNLAGAEELF
>2EW0A
MTKQYLTHRCLIAPPEMADDFFANTVIYLARHDEEGAQGIIINRPAGIQIKELLNDLDIDADNVN
PHEVLQGGPLRPEAGFVLHTGQPTWHSSIAVGENVCITTSKDILDAIAHNEGVGRYQIALGYASW
GKNQLEDEIARGDWLICDADMDLIFNLPYDDRWDAAYKKIGVDRTWLASEIGHALEHHHHHH
>1SEFA
MSLMGYKNNRVGYQKELLTSRAVIKKDNYAIIPHDGLVQNAVPGFENVDISILGSPKLGATFVDY
IATFHKNGQQTTGFGGDGIQTLVYVIDGRLRVSDGQETHELEAGGYAYFTPEMKMYLANAQEADT
EVFLYKKRYQPLAGHQPYKVVGSIHDQQPEEYEGMTDVLLWSLLPKEFDFDMNMHILSFEPGASH
AYIETHVQEHGAYLISGQGMYNLDNEWYPVEKGDYIFMSAYVPQAAYAVGREEPLMYVYSKDANR
EPELEGGSHHHHHH
>1JB0L
AEELVKPYNGDPFVGHLSTPISDSGLVKTFIGNLPAYRQGLSPILRGLEVGMAHGYFLIGPWVKL
GPLRDSDVANLGGLISGIALILVATACLAAYGLVSFQKGGSSSDPLKTSEGWSQFTAGFFVGAMG
SAFVAFFLLENFLVVDGIMTGLFN
>4IILA
MSGSHHHHHHSSGIEGRGRLIKHRPAVQDERAVRIAVFVPGFRHDSPVYAMLCDGVERAVTQERA
TGRSIGLDIIEAGPNQALWREKLAHLAAEQRYRLIVSSNPALPHVLEPILRQFPLQRFLVLDAYA
PQEHSLITFRYNQWEQAYLAGHLSALVSASAMRFANADKKIGLIAGQSYPVMTQTIIPAFLAGAR
AVDPAFEVDVRVVGNWYDAAKSADLARILFHEGVDVMMPICGGANQGVLAAARELGFYVSWFDDN

GYARAPGYVVGSSVMEQERLAYEQTLRCIRGELPSAGAWTLGVKDGYVRFIEEDPLYLQTVPEPI
RVRQSALLRRIQSGELTLPVR
>4ASMB
HHHHHHQYDWDNVPIPANAGAGKTWKLQTAASDDFNYTFNPTNNVVDFGPNGNMKWYNKYHNRPN
GQPNNFEGPGPTKWMQNHVAVSGGNLNIWASRIPGATKSFTGSNNTPISRPETRAGCITNKTRVK
YPVFVEARVKVMNSTLASDIWLLSPDDTQEIDIMECYGGPGNDNRNSYFASKIHLSHHVFIRPPN
FKDYQPADLNSWWGKNGVTQWGGKTIRIGVNWVSPTRLEYFVDGQMVRILDNDAVQTRLADGTWQ
YTYPAGVTSTGVNGQLIKENGYQKMNIASSLSDAKNKSNISVIDPFNYLNNGRKFSKEMDIIINV
EDQSWQAEAYRSPNAAEMANFYDNNLLVDWIRVYKPVN
>1FM0E
MAETKIVVGPQPFSVGEEYPWLAERDEDGAVVTFTGKVRNHNLGDSVNALTLEHYPGMTEKALAE
IVDEARNRWPLGRVTVIHRIGELWPGDEIVFVGVTSAHRSSAFEAGQFIMDYLKTRAPFWKREAT
PEGDRWVEARESDQQAAKRW
>2Y6HA
MLVANINGGFESTPAGVVTDLAEGVEGWDLNVGSSVTNPPVFEVLETSDAPEGNKVLAVTVNGVG
NNPFNIQATALPVNVRPGVTYTYTIRARAEQDGAVVSFTVGNQSFDEYGRLHHQQITTEWQPFTF
EFTVSDQETVIRAPIHFGYAANVGNTIYIDGLAIVDL
>3ZBDA
MAHHHHHHMSSKQFKILVNEDYQVNVPSLPIRDVLQEIKYCYRNGFEGYVFVPEYCRDLVDCDRK
DHYVIGVLGNGVSDLKPVLLTEPSVMLQGFIVRANCNGVLEDFDLKIA
>3ATVA
GSFTMAKAAAIGIDLGTTYSCVGVFQHGKVEIIANDQGNRTTPSYVAFTDTERLIGDAAKNQVAL
NPQNTVFDAKRLIGRKFGDPVVQSDMKHWPFQVINDGDKPKVQVSYKGETKAFYPEEISSMVLTK
MKEIAEAYLGYPVTNAVITVPAYFNDSQRQATKDAGVIAGLNVLRIINEPTAAAIAYGLDRTGKG
ERNVLIFDLGGGTFDVSILTIDDGIFEVKATAGDTHLGGEDFDNRLVNHFVEEFKRKHKKDISQN
KRAVRRLRTACERAKRTLSSSTQASLEIDSLFEGIDFYTSITRARFEELCSDLFRSTLEPVEKAL
RDAKLDKAQIHDLVLVGGSTRIPKVQKLLQDFFNGRDLNKSINPDEAVAYGAAVQAAILMGDKSE
NV
>2C3VA
GSHMASGDATDITIYYKTGWTHPHIHYSLNQGAWTTLPGVPLTKSEYEGYVKVTIEAEEGSQLRA
AFNNGSGQWDNNQGRDYDFSSGVHTLADGRILSGTPK
>3CIJA
GHMNVKLKVFHAGSLTEPMKAFKRAFEEKHPNVEVQTEAAGSAATIRKVTELGRKADVIATADYT
LIQKMMYPEFANWTIMFAKNQIVLAYRNDSRYADEINSQNWYEILKRPDVRFGFSNPNDDPCGYR
SLMAIQLAELYYNDPTIFDELVAKNSNLRFSEDNGSYVLRMPSSERIEINKSKIMIRSMEMELIH
LVESGELDYFFIYKSVAKQHGFNFVELPVEIDLSSPDYAELYSKVKVVLANGKEVTGKPIVYGIT
IPKNAENRELAVEFVKLVISEEGQEILRELGQEPL
>3OC8A
GSHMEIYPHIKVYEGTLSRLKPGGAMIAVLEYDVNELSKHGYTNLWDVQFKVLVGVPHAETGVIY
DPVYEETVKPYQPSNNLTGKKLYNVSTNDMHNGYKWSNTMFSNSNYKTQILLTKGDGSGVKLYSK
AYSENFK
>4EA9A
GHMGAASASLAIGGVVIIGGGGHAKVVIESLRACGETVAAIVDADPTRRAVLGVPVVGDDLALPM
LREQGLSRLFVAIGDNRLRQKLGRKARDHGFSLVNAIHPSAVVSPSVRLGEGVAVMAGVAINADS

WIGDLAIINTGAVVDHDCRLGAACHLGPASALAGGVSVGERAFLGVGARVIPGVTIGADTIVGAG
GVVVRDLPDSVLAIGVPAKIKGDRS
>3ARCA
MTTTLQRRESANLWERFCNWVTSTDNRLYVGWFGVIMIPTLLAATICFVIAFIAAPPVDIDGIRE
PVSGSLLYGNNIITGAVVPSSNAIGLHFYPIWEAASLDEWLYNGGPYQLIIFHFLLGASCYMGRQ
WELSYRLGMRPWICVAYSAPLASAFAVFLIYPIGQGSFSDGMPLGISGTFNFMIVFQAEHNILMH
PFHQLGVAGVFGGALFCAMHGSLVTSSLIRETTETESANYGYKFGQEEETYNIVAAHGYFGRLIF
QYASFNNSRSLHFFLAAWPVVGVWFAALGISTMAFNLNGFNFNHSVIDAKGNVINTWADIINRAN
LGMEVMHERNAHNFPLDLA
>3V6OA
IDVNINISCETDGYLTKMTCRWSTSTIQSLAESTLQLRYHRSSLYCSDIPSIHPISEPKDCYLQS
DGFYECIFQPIFLLSGYTMWIRINHSLGSLDSPPTCVLPDSVVKPLPPSSVKAEITINIGLLKIS
WEKPVFPENNLQFQIRYGLSGKEVQWKMYEVYDAKSKSVSLPVPDLCAVYAVQVRCKRLDGLGYW
SNWSNPAYTVV
>3FETA
SNAMKFLTVSDDMNFLRQVNTLVAGKGDMDSVIIGEGDAKGLGSKVLYRAKKGTPFDAVSEGILK
IAGNYDYIAIGSTEVGREIAGYLSFKTGFYTATEIFSLEFNGQKAHTKRFFYGGKTVIEEESDAR
ILTVAPGVIEAKDLGTTPEIRDLEIGQSRIKITKFV
>1M1LA
ASLFPPGLHAIYGECRRLYPDQPNPLQVTAIVKYWLGGPDPLDYVSMYRNVGSPSANIPEHWHYI
SFGLSDLYGDNRVHEFTGTDGPSGFGFELTFRLKRETGESAPPTWPAELMQGLARYVFQSENTFC
SGDHVSWHSPLDNSESRIQHMLLTEDPQMQPVQTPFGVVTFLQIVGVCTEELHSAQQWNGQGILE
LLRTVPIAGGPWLITDMRRGETIFEIDPHLQERVDKGIETD
>3CG6A
GSTARMQGAGKALHELLLSAQRQGCLTAGVYESAKVLNVDPDNVTFCVLAADEEDEGDIALQIHF
TLIQAFCCENDIDIVRVGDVQRLAAIVGSDEEGGAPGDLHCILISNPNEDTWKDPALEKLSLFCE
ESRSFNDWVPSITLPE
>2GS5A
MFADRLFNAMERNEPAPGMVLVAAPSMESEDFARSVILIIEHSEYATFGVNLASRSDVAVFNVIP
EWVPCVTKPQALYIGGPLNQQSVVGVGVTAQGVDAARVDNLTRLANRLVMVNLGADPEEIKPLVS
GMRLFAGHAEWAPGQLAQEIENGDWFVAPALPSDVTAPGSVDVWGDVMRRQPMPLPLYSTFPVNV
GEN
>1ZVTA
SEPVTIVLSQMGWVRSAKGHDIDAPGLNYKAGDSFKAAVKGKSNQPVVFVDSTGRSYAIDPITLP
SARGQGEPLTGKLTLPPGATVDHMLMESDDQKLLMASDAGYGFVCTFNDLVARNRAGKALITLPE
NAHVMPPVVIEDASDMLLAITQAGRMLMFPVSDLPQLSKGKGNKIINIPSAEAARGEDGLAQLYV
LPPQSTLTIHVGKRKIKLRPEELQKVTGERGRRGTLMRGLQRIDRVEIDSPRRASSGDSEE
>3ZQUA
MSGPERITLAMTGASGAQYGLRLLDCLVQEEREVHFLISKAAQLVMATETDVALPAKPQAMQAFL
TEYCGAAAGQIRVFGQNDWMAPPASGSSAPNAMVICPCSTGTLSAVATGACNNLIERAADVALKE
RRPLVLVPREAPFSSIHLENMLKLSNLGAVILPAAPGFYHQPQSVEDLVDFVVARILNTLGIPQD
MLPRWGEQHLVSDE
>2Y9WA
SDKKSLMPLVGIPGEIKNRLNILDFVKNDKFFTLYVRALQVLQARDQSDYSSFFQLGGIHGLPYT

EWAKAQPQLHLYKANYCTHGTVLFPTWHRAYESTWEQTLWEAAGTVAQRFTTSDQAEWIQAAKDL
RQPFWDWGYWPNDPDFIGLPDQVIRDKQVEITDYNGTKIEVENPILHYKFHPIEPTFEGDFAQWQ
TTMRYPDVQKQENIEGMIAGIKAAAPGFREWTFNMLTKNYTWELFSNHGAVVGAHANSLEMVHNT
VHFLIGRDPTLDPLVPGHMGSVPHAAFDPIFWMHHCNVDRLLALWQTMNYDVYVSEGMNREATMG
LIPGQVLTEDSPLEPFYTKNQDPWQSDDLEDWETLGFSYPDFDPVKGKSKEEKSVYINDWVHKHY
G
>3KZSA
GAQKKTQKTYIPWSNGKLVVSEEGRYLKHENGTPFFWLGETGWLLPERLNRDEAEYYLEQCKRRG
YNVIQVQTLNNVPSMNIYGQYSMTDGYNFKNINQKGVYGYWDHMDYIIRTAAKKGLYIGMVCIWG
SPVSHGEMNVDQAKAYGKFLAERYKDEPNIIWFIGGDIRGDVKTAEWEALATSIKAIDKNHLMTF
HPRGRTTSATWFNNAPWLDFNMFQSGHRRYGQRFGDGDYPIEENTEEDNWRFVERSMAMKPMKPV
IDGEPIYEEIPHGLHDENELLWKDYDVRRYAYWSVFAGSFGHTYGHNSIMQFIKPGVGGAYGAKK
PWYDALNDPGYNQMKYLKNLMLTFPFFERVPDQSVIAGQNGERYDRAIATRGNDYLMVYNYTGRP
MEVDFSKISGAKKNAWWYTTKDGKLEYIGEFDNGVHKFQHDSGYSSGNDHVLIVVDSSKDYVKKD
CYQIDTHE
>3ORUA
GMTSFDRPFEAARPDGENPSAHETLAEGGRLRPEATYTIPARQGRAIRMAQGEALMVINRDGSQI
GDFWAFVEGDCGEYLSMEHLRPTLRRVSPRPGDVLVSNRRRPILTLLEDSSPGVHDTLVASCDVH
RYAQLGHEGYHDNCTDNLRMALGALGLRPTTVPCPLNLWMNTPVVEGGAMEWRPPVSRRGDHVLF
RAELDVVVVISCCPMDLLPINGEEAQPRALDVRLRPRPA
>3NFTA
GSALTVRDWPALEALAKTMPADAGARAMTDDDLRAAGVDRRVPEQKLGAAIDEFASLRLPDRIDG
RFVDGRRANLTVFDDARVAVRGHARAQRNLLERLETELLGGTLDTAGDEGGIQPDPILQGLVDVI
GQGKSDIDAYATIVEGLTKYFQSVADVMSKLQDYISAKDDKNMKIDGGKIKALIQQVIDHLPTMQ
LPKGADIARWRKELGDAVSISDSGVVTINPDKLIKMRDSLPPDGTVWDTARYQAWNTAFSGQKDN
IQNDVQTLVEKYSHQNSNFDNLVKVLSGAISTLTDTAKSYLQI
>3LATA
VSSQKTSSLPKYTPKVNSSINNYIRKKNMKAPRIEEDYTSYFPKYGYRNGVGRPEGIVVHDTAND
NSTIDGEIAFMKRNYTNAFVHAFVDGNRIIETAPTDYLSWGAGPYGNQRFINVEIVHTHDYDSFA
RSMNNYADYAATQLQYYNLKPDSAENDGRGTVWTHAAISNFLGGTDHADPHQYLRSHNYSYAELY
DLIYEKYLIKTKQVAPWG
>3N6XA
GMDTAKTKPFDEMFLQDEVIRPIYAEYAAWLQDVPHQQLESKRQEAELLFRRVGITFNVYGEDAG
AERLIPFDVVPRILSASEWARLSDGAIQRVKALNMFLHDVYHDQEIIKAGIVPSSILANAQYRPE
MFGVDVPGGVYAHIAGVDLVRTGENDFYVLEDNLRTPSGVSYMLENRKMMMRLFPELFRRYPVAP
VEHYPQVLLNNLRAVAQAGVHEPTVVLLTPGAYNSAYFEHAFIAQQMGIELVEGQDLFVRNNAVY
MRTTEGPKRVDVIYRRIDDDFIDPLSFRPDSMLGVPGLLSVYRNGGVTLANAVGTGVADDKDTYI
YVPEMIRFYLGEEPILSNVPTYQLSKADDLKYVLDNLAELVVKEVQGSGGYGMLVGPAASKQELE
DFRQRILANPANYIAQPTLALSTCPTLVETGIAPRHVDLRPFVLSGKTVSLVPGALCRVALREGS
LVVNSSQGGGTKDTWILKD
>3AYFA
MEVNRTVSPNIQTGRKTTNSFLKSILIFTILISSTVLLVGGYWIFKEMAPRPKEVRSESGEVLMT
KETIIGGQAVFQKYGLMDYGTVLGHGSYMGPDYTAEALKVYTEGMQDYKAKERYNKPFADLTDDE
KSIIREQVIKEMRKNRYNPVTDVLVLTDAQVYGLEKVRDYYRDVFTNGDGWGLKKGLIKESDMPK

ANRAWVADSDQIQQIADFFFWTAWLSSTLRIGDEITYTNNWPYYEDAGNTMSFSAVWWSGASVTI
LILFIGIILYVFYRYQLSMQEAYAEGKFPVIDLRRQPLTPSQVKAGKYFVVVSALFFVQTMFGAL
LAHYYTEPDSFFGINWIYDILPFNIAKGYHLQLAIFWIATAWLGMGIFIAPLVGGQEPKKQGLLV
DLLFWALVVLVGGSMIGQWLGVNGYLGNEWFLLGHQGWEYIELGRIWQIILVVGMLLWLFIVFRG
VKRGLKRESDKGGLIHLLFYSAIAVPFFYIFAFFIQPDTNFTMADFWRWWIIHLWVEGIFEVFAV
VVIGFLLVQLRLVTKKSTVRALYFQFTILLGSGVIGIGHHYYYNGSPEVWIALGAVFSALEVIPL
TLLILEAYEQYKMMRDGGANFPYKATFWFLISTAIWNLVGAGVFGFLINLPAVSYFEHGQFLTPA
HGHAAMMGVYGMFAIAVLLYSLRNIVKPEAWNDKWLKFSCWMLNIGLAGMVVITLLPVGILQMKE
AFIHGYWASRSPSFLQQDVVQNLLLVRAVPDTIFLIGVVALLVFAIKALFHLRKPTHGEGEELPV
ANHWMKDRLKNSLEHHHHHH

>3A07A

SAQFASVTIRNAQTGRLLDSNYNGNVYTLPANGGNYQRWTGPGDGTVRNAQTGRCLDSNYDGAVY
TLPCNGGSYQKWLFYSNGYIQNVETGRVLDSNYNGNVYTLPANGGNYQKWYTG

>1YD7A

AHHHHHHGSNKRFPFPVGEPDFIQGDEAIARAAILAGCRFYAGYPITPASEIFEAMALYMPLVDG
VVIQMEDEIASIAAAIGASWAGAKAMTATSGPGFSLMQENIGYAVMTETPVVIVDVQRSGPSTGQ
PTLPAQGDIMQAIWGTHGDHSLIVLSPSTVQEAFDFTIRAFNLSEKYRTPVILLTDAEVGHMRER
VYIPNPDEIEIINRKLPRNEEEAKLPFGDPHGDGVPPMPIFGKGYRTYVTGLTHDEKGRPRTVDR
EVHERLIKRIVEKIEKNKKDIFTYETYELEDAEIGVVATGIVARSALRAVKMLREEGIKAGLLKI
ETIWPFDFELIERIAERVDKLYVPEMNLGQLYHLIKEGANGKAEVKLISKIGGEVHTPMEIFEFI
RREFK

>1WTJA

MSASHADQPTQTVSYPQLIDLLRRIFVVHGTSPEVADVLAENCASAQRDGSHSHGIFRIPGYLSS
LASGWVDGKAVPVVEDVGAAFVRVDACNGFAQPALAAARSLLIDKARSAGVAILAIRGSHHFAAL
WPDVEPFAEQGLVALSMVNSMTCVVPHGARQPLFGTNPIAFGAPRAGGEPIVFDLATSAIAHGDV
QIAAREGRLLPAGMGVDRDGLPTQEPRAILDGGALLPFGGHKGSALSMMVELLAAGLTGGNFSFE
FDWSKHPGAQTPWTGQLLIVIDPDKGAGQHFAQRSEELVRQLHGVGQERLPGDRRYLERARSMAH
GIVIAQADLERLQELAGH

>1EXSA

VEVTPIMTELDTQKVAGTWHTVAMAVSDVSLLDAKSSPLKAYVEGLKPTPEGDLEILLQKRENDK
CAQEVLLAKKTDIPAVFKINALDENQLFLLDTDYDSHLLLCMENSASPEHSLVCQSLARTLEVDD
QIREKFEDALKTLSVPMRILPAQLEEQCRV

>3VPZA

SLHSSAQFDPILVADIGGTNARFALITAFDAAKNEFVIEYNHTFPSADFGSLQNATRHYLSTVPH
IKPVRACLAVAGPIKAGQVHLTNLGWHFSVSEFKQAFSFLQLEVINDFAAFAYAAPYLDSNQNVV
IKAGQADENSNIAVMGPGTGFGAACLVRTAQSSAVLSSEGGHISLAAVTDLDAKLLIELRKEHPH
VSLETVFSGPGIAHLYKAMAAVNGITAKHLDAAQISNLANTGECEVCDATLNQFCDWLGSAAGDL
ALAYGALGGLFIGGGILPRMQSRLLESRFVERFSQKGIMSQYNGQVPVTLVTQDNIPLIGAAACL
HNSKQE

>2W3QA

PLGSMPFHAEPLKPSDEIDMDLGHSVAAQKFKEIREVLEGNRYWARKVTSEEPEFMAEQVKGQAP
NFLWIGCADSRVPEVTIMARKPGDVFVQRNVANQFKPEDDSSQALLNYAIMNVGVTHVMVVGHTG
CGGCIAAFDQPLPTEENPGGTPLVRYLEPIIRLKHSLPEGSDVNDLIKENVKMAVKNVVNSPTIQ
GAWEQARKGEFREVFVHGWLYDLSTGNIVDLNVTQGPHPFVDDRVPRA

>3EJKA
GMDIMLNTADISAAAILLPVEGAQLSELRQIPAEGGPVLHMLRLDSPQFSQFGEIYFSEVLPRRV
KAWKRHSLMTQLFAVPVGCIHVVLYDGREKSPTSGRLAQVTLGRPDNYRLLRIPPQVWYGFAATG
DTPALVANCTDIPHRQGESERAPQDAPFIPFSWAGADLSGTPVM
>4F7UP
MVLIMHVSPPEHGLLYTANNIKLKLGDKVVGEGTVYIAQNTLSWQPTELAEGISIEWKQVSLHGI
SSNPRKCIYFMLDHKVEWNGVYGDVDEQFGEVTECWLMPEDIATVDTMYSAMTTCQALHHHHHH
>1Z2WA
GSPEFGTRDRMLVLVLGDLHIPHRCNSLPAKFKKLLVPGKIQHILCTGNLCTKESYDYLKTLAGD
VHIVRGDFDENLNYPEQKVVTVGQFKIGLIHGHQVIPWGDMASLALLQRQFDVDILISGHTHKFE
AFEHENKFYINPGSATGAYNALETNIIPSFVLMDIQASTVVTYVYQLIGDDVKVERIEYKKS
>4EQBA
SNANSRDSQKLVIYNWGDYIDPELLTQFTEETGIQVQYETFDSNEAMYTKIKQGGTTYDIAIPSE
YMINKMKDEDLLVPLDYSKIEGIENIGPEFLNQSFDPGNKFSIPYFWGTLGIVYNETMVDEAPEH
WDDLWKLEYKNSIMLFDGAREVLGLGLNSLGYSLNSKDPQQLEETVDKLYKLTPNIKAIVADEMK
GYMIQNNVAIGVTFSGEASQMLEKNENLRYVVPTEASNLWFDNMVIPKTVKNQDSAYAFINFMLK
PENALQNAEYVGYSTPNLPAKELLPEETKEDKAFYPDVETMKHLEVYEKFDHKWTGKYSDLFLQF
KMYRK
>1U5UA
MTWKNFGFEIFGEKYGQEELEKRIKDEHTPPPDSPVFGGLKLKLKKEKFKTLFTLGTTLKGFRRA
THTVGTGGIGEITIVNDPKFPEHEFFTAGRTFPARLRHANLKYPDDAGADARSFSIKFADSDSDG
PLDIVMNTGEANIFWNSPSLEDFVPVEEGDAAEEYVYKNPYYYYNLVEALRRAPDTFAHLYYYSQ
VTMPFKAKDGKVRYCRYRALPGDVDIKEEDESGRLTEEEQRKIWIFSRHENEKRPDDYLRKEYVE
RLQKGPVNYRLQIQIHEASPDDTATIFHAGILWDKETHPWFDLAKVSIKTPLSPDVLEKTAFNIA
NQPASLGLLEAKSPEDYNSIGELRVAVYTWVQHLRKLKIGSLVPAGQNA
>2VK2A
APLTVGFSQVGSESGWRAAETNVAKSEAEKRGITLKIADGQQKQENQIKAVRSFVAQGVDAIFIA
PVVATGWEPVLKEAKDAEIPVFLLDRSIDVKDKSLYMTTVTADNILEGKLIGDWLVKEVNGKPCN
VVELQGTVGASVAIDRKKGFAEAIKNAPNIKIIRSQSGDFTRSKGKEVMESFIKAENNGKNICMV
YAHNDDMVIGAIQAIKEAGLKPGKDILTGSIDGVPDIYKAMMDGEANASVELTPNMAGPAFDALE
KYKKDGTMPEKLTLTKSTLYLPDTAKEELEKKKNMGYLEHHHHHHH
>3V5UA
MVILGVGYFLLGLILLYYGSDWFVLGSERIARHFNVSNFVIGATVMAIGTSLPEILTSAYASYMH
APGISIGNAIGSCICNIGLVLGLSAIISPIIVDKNLQKNILVYLLFVIFAAVIGIDGFSWIDGVV
LLILFIIYLRWTVKNGSAEIEENNDKNNPSVVFSLVLLIIGLIGVLVGAELFVDGAKKIALALDI
SDKVIGFTLVAFGTSLPELMVSLAAAKRNLGGMVLGNVIGSNIADIGGALAVGSLFMHLPAENVQ
MAVLVIMSLLLYLFAKYSKIGRWQGILFLALYIIAIASLRMGGGSLVPRGSRSHHHHHHH
>3G3SA
GMAEQMRRVARLFGDWPETIIWTCLEGTMGDIYVDDSQSPQSALALYGRQSFFGFLAGQPHRDLL
KICEGKNIILVPQNQAWSDLIEEVYGDGVRFFTRYATKKDTEFDLGHLQKLVDDLPESFDMKLID
RNLYETCLVEEWSRDLVGNYIDVEQFLDLGLGCVILHKGQVVSGASSYASYSAGIEIEVDTREDY
RGLGLAKACAAQLILACLDRGLYPSWDAHTLTSLKLAEKLGYELDKAYQAYEWR
>4H2DA
GSFTMPSPQLLVLFGSQTGTAQDVSERLGREARRRRLGCRVQALDSYPVVNLINEPLVIFVCATT

GQGDPPDNMKNFWRFIFRKNLPSTALCQMDFAVLGLGDSSYAKFNFVAKKLHRRLLQLGGSALLP
VCLGDDQHELGPDAAVDPWLRDLWDRVLGLYPPPP
>2AG4A
HMSSFSWDNCDEGKDPAVIRSLTLEPDPIVVPGNVTLSVVGSTSVPLSSPLKVDLVLEKEVAGLW
IKIPCTDYIGSCTFEHFCDVLDMLIPTGEPCPEPLRTYGLPCHCPFKEGTYSLPKSEFVVPDLEL
PSWLTTGNYRIESVLSSSGKRLGCIKIAASLKGI
>3IFRA
MSLAQGRQVIGLDIGTTSTIAILVRLPDTVVAVASRPTTLSSPHPGWAEEDPAQWWDNARAVLAE
LKTTAGESDWRPGGICVTGMLPAVVLLDDRGAVLRPSIQQSDGRCGDEVAELRAEVDSEAFLART
GNGVTQQLVTAKLRWIERHEPAVFGAIATVCGSYDYINMLLTGERVVDRNWALEGGFIDLASGTV
EADLVALAHIPPSAVPPAHPTHRVLGAVTAEAAALTGLPTGLPVYGGAADHIASALAAGITRPGD
VLLKFGGAGDIIVASATAKSDPRLYLDYHLVPGLYAPNGCMAATGSALNWLAKLLAPEAGEAAHA
QLDALAAEVPAGADGLVCLPYFLGEKTPIHDPFASGTFTGLSLSHTRGHLWRALLEAVALAFRHH
VAVLDDIGHAPQRFFASDGGTRSRVWMGIMADVLQRPVQLLANPLGSAVGAAWVAAIGGGDDLGW
DDVTALVRTGEKITPDPAKAEVYDRLYRDFSALYATLHPFFHRSREGHHHHHH
>3DQYA
TWTYILRQGDLPPGEMQRYEGGPEPVMVCNVDGEFFAVQDTCTHGDWALSDGYLDGDIVECTLHF
GKFCVRTGKVKALPACKPIKVFPIKVEGDEVHVDLDNGELK
>4IPIA
GAMQNNNEFKIGNRSVGYNHEPLIICEIGINHEGSLKTAFEMVDAAYNAGAEVVKHQTHIVEDEM
SDEAKQVIPGNADVSIYEIMERCALNEEDEIKLKEYVESKGMIFISTPFSRAAALRLQRMDIPAY
KIGSGECNNYPLIKLVASFGKPIILSTGMNSIESIKKSVEIIREAGVPYALLHCTNIYPTPYEDV
RLGGMNDLSEAFPDAIIGLSDHTLDNYACLGAVALGGSILERHFTDRMDRPGPDIVCSMNPDTFK
ELKQGAHALKLARGGKKDTIIAGEKPTKDFAFASVVADKDIKKGELLSGDNLWVKAPGNGDFSVN
EYETLFGKVAACNIRKGAQIKKTDIE
>4ASCA
MFLQDLIFMISEEGAVAYDPAANECYCASLSSQVPKNHVSLVTKENQVFVAGGLFYNEDNKEDPM
SAYFLQFDHLDSEWLGMPPLPSPRCLFGLGEALNSIYVVGGREIKDGERCLDSVMCYDRLSFKWG
ESDPLPYVVYGHTVLSHMDLVYVIGGKGSDRKCLNKMCVYDPKKFEWKELAPMQTARSLFGATVH
DGRIIVAAGVTDTGLTSSAEVYSITDNKWAPFEAFPQERSSLSLVSLVGTLYAIGGFATLETESG
ELVPTELNDIWRYNEEEKKWEGVLREIAYAAGATFLPVRLNVLRLTKMAENLYFQ
>3ISYA
GMENQEVVLSIDAIQEPEQIKFNMSLKNQSERAIEFQFSTGQKFELVVYDSEHKERYRYSKEKMF
TQAFQNLTLESGETYDFSDVWKEVPEPGTYEVKVTFKGRAENLKQVQAVQQFEVK
>2EHZA
MSKQAAVIELGYMGISVKDPDAWKSFATDMLGLQVLDEGEKDRFYLRMDYWHHRIVVHHNGQDDL
EYLGWRVAGKPEFEALGQKLIDAGYKIRICDKVEAQERMVLGLMKTEDPGGNPTEIFWGPRIDMS
NPFHPGRPLHGKFVTGDQGLGHCIVRQTDVAEAHKFYSLLGFRGDVEYRIPLPNGMTAELSFMHC
NARDHSIAFGAMPAAKRLNHLMLEYTHMEDLGYTHQQFVKNEIDIALQLGIHANDKALTFYGATP
SGWLIEPGWRGATAIDEAEYYVGDIFGHGVEATGYGLDVKLS
>2WLRA
MASAELAKPLTLDQLQQQNGKAIDTRPSAFYNGWPQTLNGPSGHELAALNLSASWLDKMSTEQLN
AWIKQHNLKTDAPVALYGNDKDVDAVKTRLQKAGLTHISILSDALSEPSRLQKLPHFEQLVYPQW
LHDLQQGKEVTAKPAGDWKVIEAAWGAPKLYLISHIPGADYIDTNEVESEPLWNKVSDEQLKAML

AKHGIRHDTTVILYGRDVYAAARVAQIMLYAGVKDVRLLDGGWQTWSDAGLPVERGTPPKVKAEP
DFGVKIPAQPQLMLDMEQARGLLHRQDASLVSIRSWPEFIGTTSGYSYIKPKGEIAGARWGHAGS
DSTHMEDFHNPDGTMRSADDITAMWKAWNIKPEQQVSFYCGTGWRASETFMYARAMGWKNVSVYD
GGWYEWSSDPKNPVATGERGPDSSKLEHHHHHH
>3UAFA
KTSCLMATGVLKCPTDPEAVKKVHIDLWDAAAAAAESDDLMGRTWSDRNGNFQVTGCASDFGPIN
TPDPYLYIQHNCPHRDSNATNPIQIDVIPLFLPSIVRLGNVYLDRYLEDYHH
>1PBYC
MNALVGCTTSFDPGWEVDAFGAVSNLCQPMEADLYGCADPCWWPAQVADTLNTYPNWSAGADDVM
QDWRKLQSVFPETK
>3VLDA
MGSSHHHHHHSSGLVPRGSHMSEKETNYVENLLTQLENELNEDNLPEDINTLLRKCSLNLVTVVS
LPDMDVKPLLATIKRFLTSNVSYDSLNYDYLLDVVDKLVPMADFDDVLEVYSAEDLVKALRSEID
PLKVAACRVIENSQPKGLFATSNIIDILLDILFDEKVENDKLITAIEKALERLSTDELIRRRLFD
NNLPYLVSVKGRMETVSFVRLIDFLTIEFQFISGPEFKDIIFCFTKEEILKSVEDILVFIELVNY
YTKFLLEIRNQDKYWALRHVKKILPVFAQLFEDTENYPDVRAFSTNCLLQLFAEVSRIEEDEYSL
FKTMDKDSLKIGSEAKLITEWLELINPQYLVKYHKDVVENYFHVSGYSIGMLRNLSADEECFNAI
RNKFSAEIVLRLPYLEQMQVVETLTRYEYTSKFLLNEMPKVMGSLIGDGSAGAIIDLETVHYRNS
ALRNLLDKGEEKLSVWYEPLLREYSKAVNGKNYSTGSETKIADCR
>2HIQA
MTDPALRAATLLQLHFAFNGPFGDAMAEQLKPLAESINQEPGFLWKVWTESEKNHEAGGIYLFTD
EKSALAYLEKHTARLKNLGVEEVVAKVFDVNEPLSQINQAKLAGLCGR
>3UAUA
MGHHHHHHHHHHSSGHIDDDDKHMCGNSIDEKTVKKYENQLNQTVKQEIASLSQDSGIKIEFSDF
KCNADGDFIACLSPNFKTLAKDNNDEYQELFQAKNIKIRSNEIYKGETNTSISIKEYYNDLFKNQ
KSIQSNLVFEDFKLGEKVVSDINASLFQQDPKISSFINKLSSDSYTLSFDNSINKQENNYLDNLD
IKFYNAKLNFNTNLNINLKEDLLNYLDSKGIKFNTQTLAMDEQAINELLNMVNYEQASDFSNTIQ
KYIIILNNFKIDSTLKTEGVFSSYIATAKENLQTLKAQSQNEEQALIFDKALAILNNITQNDDYKL
NLDLKFKNIPVSDYSTQGIDSIEKLSINNQDATEALKIILPFIMFSMLMGGASF
>3D40A
MGSSHHHHHHSSGLVPRGSHMTPDFLAIKVGGSLFSRKDEPGSLDDDAVTRFARNFARLAETYRG
RMVLISGGGAFGHGAIRDHDSTHAFSLAGLTEATFEVKKRWAEKLRGIGVDAFPLQLAAMCTLRN
GIPQLRSEVLRDVLDHGALPVLAGDALFDEHGKLWAFSSDRVPEVLLPMVEGRLRVVTLTDVDGI
VTDGAGGDTILPEVDARSPEQAYAALWGSSEWDATGAMHTKLDALVTCARRGAECFIMRGDPGSD
LEFLTAPFSSWPAHVRSTRITTTASA
>3EJNA
GGNLEEMNIDPDNATQTHPKLLLTQICMNAFKRGTDGMYATKKVIQADGESADQYYKWTRGSFGY
YDNLRNVQKMGEEAERVNAPVYTALTKFFRAYYFYELTLRFGDIPYSQALKGEKEEIYTPEYDAQ
EDVFAGILQELREADEILANDASVIDGDIIYNGNSTQWRKLINSFRLKVLMTLSNHTTVGNINIA
SEFKNIATNSPLMNSLADNGQLVYLDQQGNRYPQFNAQWSGYYMDDTFIQRMRERRDPRLFIFSA
QTNKGKTEGKPIDDFSSYEGGDPAAPYSDAIIKVSEGTISPINDRFRTDPIVEPTMLMGYAELQQ
ILAEAVVRGWISGNAQTYYEKGIRASFSFYETHAKDYAGYLNENAVAQYLKEPLVDFTQASGTEE
QIERIIMQKYLVTFYQGNWDSFYEQLRTGYPDFRRPAGTEIPKRWMYPQGEYDNNGTNVETAITR
QFGAGNDKINQATWWQKKS

>3HVNA
GPLGSRKSSHLILSSIVSLALVGVTPLSVLADSKQDINQYFQSLTYEPQEILTNEGEYIDNPPAT
TGMLENGRFVVLRREKKNITNNSADIAVIDAKAANIYPGALLRADQNLLDNNPTLISIARGDLTL
SLNLPGLANGDSHTVVNSPTRSTVRTGVNNLLSKWNNTYAGEYGNTQAELQYDETMAYSMSQLKT
KFGTSFEKIAVPLDINFDAVNSGEKQVQIVNFKQIYYTVSVDEPESPSKLFAEGTTVEDLKRNGI
TDEVPPVYVSSVSYGRSMFIKLETSSRSTQVQAAFKAAIKGVDISGNAEYQDILKNTSFSAYIFG
GDAGSAATVVSGNIETLKKIIEEGARYGKLNLGVPISYSTNFVKDNRPAQILSNSEYIETTSTVH
NSSALTLDHSGAYVAKYNITWEEVSYNEAGEEVWEPKAWDKNGVNLTSHWSETIQIPGNARNLHV
NIQECTGLAWEWWRTVYDKDLPLVGQRKITIWGTTLYPQYADEVIELERPHRD
>3MKCA
MSLALNPAVAPIKSIEFIPVNYQASNWSQNTVVVKVTDENGVYGLGEADGSPDAILAYANIETEH
KWLTNITEKAIGRLPIEINAIWDAMYDATQWQGMRGLGMFALSGIDMALYDLAGKQLGVPAYQLL
GGTNKDKVHPYLTLYPAIPVDASLDVAIKGYAPLLEKAKAHNIRAVKVCVPIKADWSTKEVAYYL
RELRGILGHDTDMMVDYLYRFTDWYEVARLLNSIEDLELYFAEATLQHDDLSGHAKLVENTRSRI
CGAEMSTTRFEAEEWITKGKVHLLQSDYNRCGGLTELRRITEMATANNVQVMPHNWKTGITSAAA
IHYQFAVGNAPYFEYVHPEFCDGELRKYLVTPEAELVDGGFAKPTAPGLGIDLNQEFLASLEGHH
HHHH
>4ID2A
GCGGKKGSSDNTSTLAMIDSVDAHGLQRMQTSKSETDFKFKGKDYHSLVSRTPDDNLPHVTNELG
DTYVDNKIVLHLTRGNETVLNKTFTKNDFSSVVDANFLSKSILEGIVYDKTTPQGIVYAASVCYP
QTDLYMPLSITITADGKMSIQKVDILEEDYDDEAPN
>1YUMA
MGSSHHHHHHSSGLVPRGSHMGKRIGLFGGTFDPVHIGHMRSAVEMAEQFALDELRLLPNARPPH
RETPQVSAAQRLAMVERAVAGVERLTVDPRELQRDKPSYTIDTLESVRAELAADDQLFMLIGWDA
FCGLPTWHRWEALLDHCHIVVLQRPDADSEPPESLRDLLAARSVADPQALKGPGGQITFVWQTPL
AVSATQIRALLGAGRSVRFLVPDAVLNYIEAHHLYRAPHLEHHHHHH
>3D02A
GAAEKTVVNISKVDGMPWFNRMGEGVVQAGKEFNLNASQVGPSSTDAPQQVKIIEDLIARKVDAI
TIVPNDANVLEPVFKKARDAGIVVLTNESPGQPSANWDVEIIDNEKFAAEYVEHMAKRMGGKGGY
VIYVGSLTVPQHNLWADLLVKYQKEHYPDMHEVTRRMPVAESVDDSRRTTLDLMKTYPDLKAVVS
FGSNGPIGAGRAVKEKRAKNKVAVYGMMIPSQAASLIKSGDITEGITYDPATAGYALAAVASTLL
NGKTIEPGFELKELGKAEVDSDKHIIRFHKVLLVNKDNIDSLY
>2HBOA
GMSDDLTDAQTAAIPEGFSQLNWSRGFGRQIGPLFEHREGPGQARLAFRVEEHHTNGLGNCHGGM
LMSFADMAWGRIISLQKSYSWVTVRLMCDFLSGAKLGDWVEGEGELISEEDMLFTVRGRIWAGER
TLITGTGVFKALSARKPRPGELAYKEEA
>3TEWA
EVKQENRLLNESESSSQGLLGYYFSDLNFQAPMVVTSSTTGDLSIPSSELENIPSENQYFQSAIW
SGFIKVKKSDEYTFATSADNHVTMWVDDQEVINKASNSNKIRLEKGRLYQIKIQYQRENPTEKGL
DFKLYWTDSQNKKEVISSDNLQLPELKQKSSNSRKKRSTSAGPTVPDRDNDGIPDSLEVEGYTVD
VKNKRTFLSPWISNIHEKKGLTKYKSSPEKWSTASDPYSDFEKVTGRIDKNVSPEARHPLVAAYP
IVHVDMENIILSKNEDQSTQNTDSQTRTISKNTSTSRTHTSEPGSNSNSSTVAIDHSLSLAGERT
WAETMGLNTADTARLNANIRYVNTGTAPIYNVLPTTSLVLGKNQTLATIKAKENQLSQILAPNNY
YPSKNLAPIALNAQDDFSSTPITMNYNQFLELEKTKQLRLDTDQVYGNIATYNFENGRVRVDTGS

NWSEVLPQIQETTARIIFNGKDLNLVERRIAAVNPSDPLETTKPDMTLKEALKIAFGFNEPNGNL
QYQGKDITEFDFNFDQQTSQNIKNQLAELNATNIYTVLDKIKLNAKMNILIRDKRFHYDRNNIAV
GADESVVKEAHREVINSSTEGLLLNIDKDIRKILSGYIVEIEDTEGLKEVINDRYDMLNISSLRQ
DGKTFIDFKKYNDKLPLYISNPNYKVNVYAVTKENTIINPSENGDTSTNGIKKILIFSKKGYEIG
>3B9OA
MTKKIHINAFEMNCVGHIAHGLWRHPENQRHRYTDLNYWTELAQLLEKGKFDALFLADVVGIYDV
YRQSRDTAVREAVQIPVNDPLMLISAMAYVTKHLAFAVTFSTTYEHPYGHARRMSTLDHLTKGRI
AWNVVTSHLPSADKNFGIKKILEHDERYDLADEYLEVCYKLWEGSWEDNAVIRDIENNIYTDPSK
VHEINHSGKYFEVPGPHLCEPSPQRTPVIYQAGMSERGREFAAKHAECVFLGGKDVETLKFFVDD
IRKRAKKYGRNPDHIKMFAGICVIVGKTHDEAMEKLNSFQKYWSLEGHLAHYGGGTGYDLSKYSS
NDYIGSISVGEIINNMSKLDGKWFKLSVGTPKKVADEMQYLVEEAGIDGFNLVQYVSPGTFVDFI
ELVVPELQKRGLYRVDYEEGTYREKLFGKGNYRLPDDHIAARYRNISSNV
>2HFTA
SGTTNTVAAYNLTWKSTNFKTILEWEPKPVNQVYTVQISTKSGDWKSKCFYTTDTECDLTDEIVK
DVKQTYLARVFSYPAGNVESTGSAGEPLYENSPEFTPYLETNLGQPTIQSFEQVGTKVNVTVEDE
RTLVRRNNTFLSLRDVFGKDLIYTLYYWKSSSQEKGEFRSGKKTAKTNTNEFLIDVDKGENYCFS
VQAVIPSRTVNRKSTDSPVECMG
>1NQKA
MSLNMFWFLPTHGDGHYLGTEEGSRPVDHGYLQQIAQAADRLGYTGVLIPTGRSCEDAWLVAASM
IPVTQRLKFLVALRPSVTSPTVAARQAATLDRLSNGRALFNLVTGSDPQELAGDGVFLDHSERYE
ASAEFTQVWRRLLQRETVDFNGKHIHVRGAKLLFPAIQQPYPPLYFGGSSDVAQELAAEQVDLYL
TWGEPPELVKEKIEQVRAKAAAHGRKIRFGIRLHVIVRETNDEAWQAAERLISHLDDETIAKAQA
AFARTDSVGQQRMAALHNGKRDNLEISPNLWAGVGLVRGGAGTALVGDGPTVAARINEYAALGID
SFVLSGYPHLEEAYRVGELLFPLLDVAIPEIPQPQPLNPQGEAVANDFIPRKVAQS
>4ATEA
HHHHHHAQLPSPTNGKKWEKVEQLSDEFNGNSIDTNKWYDYHPFWEGRAPSNFKKGNAFVSDGFL
NLRSTLRKEPSSVQDPFKDIWVDAAAAVSKTKAQPGYYYEARFKASSLSMTSSFWFRVGQFSEID
VIEHIGNPSKENRQDDLPYQYHVNTHYYGKHAGLQPLGTEYKMPGRGRDNFYTYGFWWKSPNELL
FYFNGKQVMRIVPRVPLDEELRMIFDTEVFPFATAGVANIGLPKPENLRDNSKNTMKVDWVRVYK
LVDGTA
>4HVKA
MAYFDYTSAKPVDERILEAMLPYMTESFGNPSSVHSYGFKAREAVQEAREKVAKLVNGGGGTVVF
TSGATEANNLAIIGYAMRNARKGKHILVSAVEHMSVINPAKFLQKQGFEVEYIPVGKYGEVDVSF
IDQKLRDDTILVSVQHANNEIGTIQPVEEISEVLAGKAALHIDATASVGQIEVDVEKIGADMLTI
SSNDIYGPKGVGALWIRKEAKLQPVILGGGQENGLRSGSENVPSIVGFGKAAEITAMEWREEAER
LRRLRDRIIDNVLKIEESYLNGHPEKRLPNNVNVRFSYIEGESIVLSLDMAGIQASTGSACSSKT
LQPSHVLMACGLKHEEAHGTLLLTLGRYNTDEDVDRLLEVLPGVIERLRSMSPLYRR
>2VO8A
QIGELKTTVGNSTIKVNDEVQVGSAFEAILGIEGLNGDTEVYSAEYLFEYNAEAFILNEITSFND
SLFVKSKEVEPGKVRILVASLGNEIEKDSDLVKVNLTPKISSELEVLGLTTALVGAGDGNTHDLE
LSSKEVKINEEAS
>3LAGA
GMTVAAKSEIQIDNDEVRVTEWRLPPGSATGHHTHGMDYVVVPMADGEMTIVAPDGTRSLAQLKT
GRSYARKAGVQHDVRNESTAEIVFLEIELKAGS

>2VS0A
MAMIKMSPEEIRAKSQSYGQGSDQIRQILSDLTRAQGEIAANWEGQAFSRFEEQFQQLSPKVEKF
AQLLEEIKQQLNSTADAVQEQDQQLSNNFGLQ
>4JOQA
SNAQEKVGTIGIAIPSATHGFMGGLNFHAQDTIKRLQEVYPQLDFVLATAGNAGKMVNDIEDMVA
TRNISALVVLPFESEPLTSPVQAVKEAGIWVTVVDRGLSVEGIEDLYVAGDNPGFGRVAGEYFAQ
HLESGKKIVVLRGIPTTLDNERVEAFTAAIEGSGIEVLDMQHGNWNRDDAFNVMQDFLSKYPQID
AVWAADDDMAIGAMEAIAQAGRTEEMWVMGGAGMKEIIRRIADGDPQLPANVTYPPAQISTAIEL
TALKLVSSTPVSGRFIIGSQLVTPENAEQFYFPDSPF
>3GOHA
GMEQHQVWAYQTKTHSVTLNSVDIPALAADDILVQNQAIGINPVDWKFIKANPINWSNGHVPGVD
GAGVIVKVGAKVDSKMLGRRVAYHTSLKRHGSFAEFTVLNTDRVMTLPDNLSFERAAALPCPLLT
AWQAFEKIPLTKQREVLIVGFGAVNNLLTQMLNNAGYVVDLVSASLSQALAAKRGVRHLYREPSQ
VTQKYFAIFDAVNSQNAAALVPSLKANGHIICIQDRIPAPIDPAFTRTISYHEIALGALHDFGDR
QDWQILMQQGEALLTLIAQGKMEIAAPDIFRFEQMIEALDHSEQTKLKTVLTLNE
>2XXPA
MGLTNRLNATSNYSEYSLSVAVLADSEIENVTQLTSVTAPTGTDNENIQKLLADIKSSQNTDLTV
NQSSSYLAAYKSLIAGETKAIVLNSVFENIIELEYPDYASKIKKIYTKGFTKKVEAPKTSKNQSF
NIYVSGIDTYGPISSVSRSDVNILMTVNRDTKKILLTTTPRDAYVPIADGGNNQKDKLTHAGIYG
VDSSIHTLENLYGVDINYYVRLNFTSFLKMIDLLGGVDVHNDQEFSALHGKFHFPVGNVHLDSEQ
ALGFVRERYSLADGDRDRGRNQQKVIVAILQKLTSTEALKNYSTIINSLQDSIQTNVPLETMINL
VNAQLESGGNYKVNSQDLKGTGRMDLPSYAMPDSNLYVMEIDDSSLAVVKAAIQDVMEGRKLAAA
LEHHHHHH
>1EEXA
MRSKRFEALAKRPVNQDGFVKEWIEEGFIAMESPNDPKPSIKIVNGAVTELDGKPVSDFDLIDHF
IARYGINLNRAEEVMAMDSVKLANMLCDPNVKRSEIVPLTTAMTPAKIVEVVSHMNVVEMMMAMQ
KMRARRTPSQQAHVTNVKDNPVQIAADAAEGAWRGFDEQETTVAVARYAPFNAIALLVGSQVGRP
GVLTQCSLEEATELKLGMLGHTCYAETISVYGTEPVFTDGDDTPWSKGFLASSYASRGLKMRFTS
GSGSEVQMGYAEGKSMLYLEARCIYITKAAGVQGLQNGSVSCIGVPSAVPSGIRAVLAENLICSS
LDLECASSNDQTFTHSDMRRTARLLMQFLPGTDFISSGYSAVPNYDNMFAGSNEDAEDFDDYNVI
QRDLKVDGGLRPVREEDVIAIRNKAARALQAVFAGMGLPPITDEEVEAATYAHGSKDMPERNIVE
DIKFAQEIINKNRNGLEVVKALAQGGFTDVAQDMLNIQKAKLTGDYLHTSAIIVGDGQVLSAVND
VNDYAGPATGYRLQGERWEEIKNIPGALDPNEID
>1NBWB
MSLSPPGVRLFYDPRGHHAGAINELCWGLEEQGVPCQTITYDGGGDAAALGALAARSSPLRVGIG
LSASGEIALTHAQLPADAPLATGHVTDSDDQLRTLGANAGQLVKVLPLSERN
>3E4BA
MAGLPDQRLANEALKRGDTVTAQQNYQQLAELGYSEAQVGLADIQVGTRDPAQIKQAEATYRAAA
DTSPRAQARLGRLLAAKPGATEAEHHEAESLLKKAFANGEGNTLIPLAMLYLQYPHSFPNVNAQQ
QISQWQAAGYPEAGLAQVLLYRTQGTYDQHLDDVERICKAALNTTDICYVELATVYQKKQPEQQ
AELLKQMEAGVSRGTVTAQRVDSVARVLGDATLGTPDEKTAQALLEKIAPGYPASWVSLAQLLYD
FPELGDVEQMMKYLDNGRAADQPRAELLLGKLYYEGKWVPADAKAAEAHFEKAVGREVAADYYLG
QIYRRGYLGKVYPQKALDHLLTAARNGQNSADFAIAQLFSQGKGTKPDPLNAYVFSQLAKAQDTP
EANDLATQLEAPLTPAQRAEGQRLVQQELAARGTLAQSTLQLHALQEEDGEESLLEHHHHHH

>4FDZA
MKNDLIRPNVLSVKIISNVSPEMAKKLELEPHHKSLGLITADCDDVTYTALDEATKAAEVDVVYA
RSMYAGAGNASTKLAGEVIGILAGPSPAEVRSGLNATLDFIDSGVGFVSANEDDSICYYAQCVSR
TGSYLSKTAGIREGEALAYLVAPPLEAMYALDAALKAADVEMCEFFAPPTETNFAGALLTGSQSA
CKAACDAFAEAVQSVASNPLGFLEHHHHHH
>3DK9A
ACRQEPQPQGPPPAAGAVASYDYLVIGGGSGGLASARRAAELGARAAVVESHKLGGTCVNVGCVP
KKVMWNTAVHSEFMHDHADYGFPSCEGKFNWRVIKEKRDAYVSRLNAIYQNNLTKSHIEIIRGHA
AFTSDPKPTIEVSGKKYTAPHILIATGGMPSTPHESQIPGASLGITSDGFFQLEELPGRSVIVGA
GYIAVEMAGILSALGSKTSLMIRHDKVLRSFDSMISTNCTEELENAGVEVLKFSQVKEVKKTLSG
LEVSMVTAVPGRLPVMTMIPDVDCLLWAIGRVPNTKDLSLNKLGIQTDDKGHIIVDEFQNTNVKG
IYAVGDVCGKALLTPVAIAAGRKLAHRLFEYKEDSKLDYNNIPTVVFSHPPIGTVGLTEDEAIHK
YGIENVKTYSTSFTPMYHAVTKRKTKCVMKMVCANKEEKVVGIHMQGLGCDEMLQGFAVAVKMGA
TKADFDNTVAIHPTSSEELVTLR
>1AYOA
EFPFALEVQTLPQTCDGPKAHTSFQISLSVSYIGSRPASNMAIVDVKMVSGFIPLKPTVKMLERS
NVSRTEVSNNHVLIYLDKVTNETLTLTFTVLQDIPVRDLKPAIVKVYDYYETDEFAVAEYSAPCS
>3U9WA
IVDTCSLASPASVCRTKHLHLRCSVDFTRRTLTGTAALTVQSQEDNLRSLVLDTKDLTIEKVVIN
GQEVKYALGERQSYKGSPMEISLPIALSKNQEIVIEISFETSPKSSALQWLTPEQTSGKEHPYLF
SQCQAIHCRAILPCQDTPSVKLTYTAEVSVPKELVALMSAIRDGETPDPEDPSRKIYKFIQKVPI
PCYLIALVVGALESRQIGPRTLVWSEKEQVEKSAYEFSETESMLKIAEDLGGPYVWGQYDLLVLP
PSFPYGGMENPCLTFVTPTLLAGDKSLSNVIAHEISHSWTGNLVTNKTWDHFWLNEGHTVYLERH
ICGRLFGEKFRHFNALGGWGELQNSVKTFGETHPFTKLVVDLTDIDPDVAYSSVPYEKGFALLFY
LEQLLGGPEIFLGFLKAYVEKFSYKSITTDDWKDFLYSYFKDKVDVLNQVDWNAWLYSPGLPPIK
PNYDMTLTNACIALSQRWITAKEDDLNSFNATDLKDLSSHQLNEFLAQTLQRAPLPLGHIKRMQE
VYNFNAINNSEIRFRWLRLCIQSKWEDAIPLALKMATEQGRMKFTRPLFKDLAAFDKSHDQAVRT
YQEHKASMHPVTAMLVGKDLKVD
>4ILKA
MGSSHHHHHHSSGLVPRGSHMKSILIEKPNQLSIIEREIPTPSAGEVRVKVKLAGICGSDSHIYR
GHNPFAKYPRVIGHEFFGVIDAVGEGVESARVGERVAVDPVVSCGHCYPCSIGKPNVCTTLAVLG
VHADGGFSEYAVVPAKNAWKIPEAVADQYAVMIEPFTIAANVTGHGQPTENDTVLVYGAGPIGLT
IVQVLKGVYNVKNVIVADRIDERLEKAKESGADWAINNSQTPLGESFAEKGIKPTLIIDAACHPS
ILKEAVTLASPAARIVLMGFSSEPSEVIQQGITGKELSIFSSRLNANKFPVVIDWLSKGLIKPEK
LITHTFDFQHVADAISLFELDQKHCCKVLLTFSE
>2UYTA
MTFRNCVAVDLGASSGRVMLARYERECRSLTLREIHRFNNGLHSQNGYVTWDVDSLESAIRLGLN
KVCAAGIAIDSIGIDTWGVDFVLLDQQGQRVGLPVAYRDSRTNGLMAQAQQQLGKRDIYQRSGIQ
FLPFNTLYQLRALTEQQPELIPHIAHALLMPDYFSYRLTGKMNWEYTNATTTQLVNINSDDWDES
LLAWSGANKAWFGRPTHPGNVIGHWICPQGNEIPVVAVASHDTASAVIASPLNGSRAAYLSSGTW
SLMGFESQTPFTNDTALAANITNEGGAEGRYRVLKNIMGLWLLQRVLQERQINDLPALIAATQAL
PACRFIINPNDDRFINPDEMCSEIQAACREMAQPIPESDAELARCIFDSLALLYADVLHELAQLR
GEDFSQLHIVGGGCQNTLLNQLCADACGIRVIAGPVEASTLGNIGIQLMTLDELNNVDDFRQVVS
TTANLTTFTPNPDSEIAHYVALIHSTRQTKELCA

>1LUCA
MKFGNFLLTYQPPELSQTEVMKRLVNLGKASEGCGFDTVWLLEHHFTEFGLLGNPYVAAAHLLGA
TETLNVGTAAIVLPTAHPVRQAEDVNLLDQMSKGRFRFGICRGLYDKDFRVFGTDMDNSRALMDC
WYDLMKEGFNEGYIAADNEHIKFPKIQLNPSAYTQGGAPVYVVAESASTTEWAAERGLPMILSWI
INTHEKKAQLDLYNEVATEHGYDVTKIDHCLSYITSVDHDSNRAKDICRNFLGHWYDSYVNATKI
FDDSDQTKGYDFNKGQWRDFVLKGHKDTNRRIDYSYEINPVGTPEECIAIIQQDIDATGIDNICC
GFEANGSEEEIIASMKLFQSDVMPYLKEKQ
>2RH0A
GFEERSGVVPCGTPWGQWYQTLEEVFIEVQVPPGTRAQDIQCGLQSRHVALAVGGREILKGKLFD
STIADEGTWTLEDRKMVRIVLTKTKRDAANCWTSLLESEYAADPWVQDQMQRKLTLERFQKENPG
FDFSGAEISGNYTKGGPDFSNLGNDGT
>1GMXA
MDQFECINVADAHQKLQEKEAVLVDIRDPQSFAMGHAVQAFHLTNDTLGAFMRDNDFDTPVMVMC
YHGNSSKGAAQYLLQQGYDVVYSIDGGFEAWQRQFPAEVAYGA
>1N13A
MNAEINPLHAYFKLPNTVSLVAGSSEGETPLNAFDGALLNAGIGNVNLIRIS
>3ONDA
GSHMALLVEKTTSGREYKVKDMSQADFGRLEIELAEVEMPGLMASRSEFGPSQPFKGAKITGSLH
MTIQTAVLIETLTALGAEVRWCSCNIFSTQDHAAAAIARDSAAVFAWKGETLQEYWWCTERALDW
GPGGGPDLIVDDGGDTTLLIHEGVKAEEIYEKSGQFPDPDSTDNAEFKIVLSIIKEGLKTDPKRY
HKMKDRVVGVSEETTTGVKRLYQMQANGTLLFPAINVNDSVTKSKFDNLYGCRHSLPDGLMRATD
VMIAGKVAVVAGYGDVGKGCAAALKQAGARVIVTEIDPICALQATMEGLQVLTLEDVVSEADIFV
TTTGNKDIIMLDHMKKMKNNAIVCNIGHFDNEIDMLGLETHPGVKRITIKPQTDRWVFPETNTGI
IILAEGRLMNLGCATGHPSFVMSCSFTNQVIAQLELWNEKSSGKYEKKVYVLPKHLDEKVAALHL
EKLGAKLTKLSKDQADYISVPVEGPYKPFHYRY
>2VPBA
GHSSSDPVYPCGICTNEVNDDQDAILCEASCQKWFHRICTGMTETAYGLLTAEASAVWGCDTCMA
>1DTDB
DESFLCYQPDQVCCFICRGAAPLPSEGECNPHPTAPWCREGAVEWVPYSTGQCRTTCIPYV
>1JHJA
ATPNKTPPGADPKQLERTGTVREIGSQAVWSLSSCKPGFGVDQLRDDNLETYWQSDGSQPHLVNI
QFRRKTTVKTLCIYADYKSDESYTPSKISVRVGNNFHNLQEIRQLELVEPSGWIHVPLTDNHKKP
TRTFMIQIAVLANHQNGRDTHMRQIKIYTPVEESSIGKFPR
>1Z6MA
SNAMDISVIDATKVNTETGLHIGESNAPVKMIEFINVRCPYCRKWFEESEELLAQSVKSGKVERI
IKLFDKEKESLQRGNVMHHYIDYSAPEQALSALHKMFATQDEWGNLTLEEVATYAEKNLGLKEQK
DATLVSAVIAEANAAHIQFVPTIIIGEYIFDESVTEEELRGYIEK
>3CP5A
TESGTAAQDPEALAAEIGPVKQVSLGEQIDAALAQQGEQLFNTYCTACHRLDERFIGPALRDVTK
RRGPVYIMNVMLNPNGMIQRHPVMKQLVQEYGTMMTDMALSEEQARAILEYLRQVAENQ
>4GDZA
GQESSTQRRADRKAQRDAERARLKAEQQAADAVSYDDAVAALKAQQFVLEANQVMFRNGQTAFVT
SNTNFVLVNQGRGTVQVAFNTVYPGPNGIGGVTVDGTVSDIKTSTDKRGNINCSFSIQGIGISAQ
IFLTLTNGDNNATVTINPNFNSNTMTLSGSLLPLNQSNIFKGRSW

>3TUFB
GPAMQSVSNDEVKDQLADNGGNSAYDNNDDAVEVGKSMENVAMPVVDSENVSVVKKFYETDAAKE
EKEAALVTYNNTYSLSKGIDLAEKDGKDFDVSASLSGTVVKAEKDPVLGYVVEVEHADGLSTVYQ
SLSEVSVEQGDKVKQNQVIGKSGKNLYSEDSGNHVHFEIRKDGVAMNPLNFMDKPVSSIEKAATQ
ETEESIQQSSEKKDGSTEKGTEEKSGEKKDDSTDKSGSKESSTTEDTEQS
>1KZLA
MFTGLVEAIGVVKDVQGTIDNGFAMKIEAPQILDDCHTGDSIAVNGTCLTVTDFDRYHFTVGIAP
ESLRLTNLGQCKAGDPVNLERAVLSSTRMGGHFVQGHVDTVAEIVEKKQDGEAIDFTFRPRDPFV
LKYIVYKGYIALDGTSLTITHVDDSTFSIMMISYTQSKVIMAKKNVGDLVNVEVDQIGKYTEKLV
EAHIADWIKKTQA
>1FX2A
NNNRAPKEPTDPVTLIFTDIESSTALWAAHPDLMPDAVAAHHRMVRSLIGRYKCYEVKTVGDSFM
IASKSPFAAVQLAQELQLCFLHHDWGTNALDDSYREFEEQRAEGECEYTPPTAHMDPEVYSRLWN
GLRVRVGIHTGLCDIRHDEVTKGYDYYGRTPNMAARTESVANGGQVLMTHAAYMSLSAEDRKQID
VTALGDVALRGVSDPVKMYQLNTVPSRNFAALRLDREYFD
>3RK6A
GSHMASMTGGQQMGRGSTLSEYVQDFLNHLTEQPGSFETEIEQFAETLNGCVTTDDALQELVELI
YQQATSIPNFSYMGARLCNYLSHHLTISPQSGNFRQLLLQRCRTEYEVKDQAAKGDEVTRKRFHA
FVLFLGELYLNLEIKGTNGQVTRADILQVGLRELLNALFSNPMDDNLICAVKLLKLTGSVLEDAW
KEKGKMDMEEIIQRIENVVLDANCSRDVKQMLLKLVELR
>2GKEA
MQFSKMHGLGNDFVVVDGVTQNVFFTPETIRRLANRHCGIGFDQLLIVEAPYDPELDFHYRIFNA
DGSEVSQCGNGARCFARFVTLKGLTNKKDISVSTQKGNMVLTVKDDNQIRVNMGEPIWEPAKIPF
TANKFEKNYILRTDIQTVLCGAVSMGNPHCVVQVDDIQTANVEQLGPLLESHERFPERVNAGFMQ
IINKEHIKLRVYERGAGETQACGSGACAAVAVGIMQGLLNNNVQVDLPGGSLMIEWNGVGHPLYM
TGEATHIYDGFITL
>1VHZA
MSLSKSLQKPTILNVETVARSRLFTVESVDLEFSNGVRRVYERMRPTNREAVMIVPIVDDHLILI
REYAVGTESYELGFSKGLIDPGESVYEAANRELKEEVGFGANDLTFLKKLSMAPSYFSSKMNIVV
AQDLYPESLEGDEPEPLPQVRWPLAHMMDLLEDPDFNEARNVSALFLVREWLKGQGRVEGGSHHH
HHH
>1RK6A
MRGSHHHHHHGSMSQPDATPFDYILSGGTVIDGTNAPGRLADVGVRGDRIAAVGDLSASSARRRI
DVAGKVVSPGFIDSHTHDDNYLLKHRDMTPKISQGVTTVVTGNCGISLAPLAHANPPAPLDLLDE
GGSFRFARFSDYLEALRAAPPAVNAACMVGHSTLRAAVMPDLRREATADEIQAMQALADDALASG
AIGISTGAFYPPAAHASTEEIIEVCRPLITHGGVYATHMRDEGEHIVQALEETFRIGRELDVPVV
ISHHKVMGKLNFGRSKETLALIEAAMASQDVSLDAYPYVAGSTMLKQDRVLLAGRTLITWCKPYP
ELSGRDLEEIAAERGKSKYDVVPELQPAGAIYFMMDEPDVQRILAFGPTMIGSDGLPHDERPHPR
LWGTFPRVLGHYSRDLGLFPLETAVWKMTGLTAAKFGLAERGQVQPGYYADLVVFDPATVADSAT
FEHPTERAAGIHSVYVNGAAVWEDQSFTGQHAGRVLNRAGA
>3FFVA
MDDLTAQALKDFTARYCDAWHEEHKSWPLSEELYGVPSPCIISTTEDAVYWQPQPFTGEQNVNAV
ERAFDIVIQPTIHTFYTTQFAGDMHAQFGDIKLTLLQTWSEDDFRRVQENLIGHLVTQKRLKLPP
TLFIATLEEELEVISVCNLSGEVCKETLGTRKRTHLASNLAEFLNQLKPLL

>7ODCA
MSSFTKDEFDCHILDEGFTAKDILDQKINEVSSSDDKDAFYVADLGDILKKHLRWLKALPRVTPF
YAVKCNDSRAIVSTLAAIGTGFDCASKTEIQLVQGLGVPAERVIYANPCKQVSQIKYAASNGVQM
MTFDSEIELM
>4AFKA
ANSGEAPKNFGLDVKITGESENDRDLGTAPGGTLNDIGIDLRPWAFGQWGDWSAYFMGQAVAATD
TIETDTLQSDTDDGNNSRNDGREPDKSYLAAREFWVDYAGLTAYPGEHLRFGRQRLREDSGQWQD
TNIEALNWSFETTLLNAHAGVAQRFSEYRTDLDELAPEDKDRTHVFGDISTQWAPHHRIGVRIHH
ADDSGHLRRPGEEVDNLDKTYTGQLTWLGIEATGDAYNYRSSMPLNYWASATWLTGDRDNLTTTT
VDDRRIATGKQSGDVNAFGVDLGLRWNIDEQWKAGVGYARGSGGGKDGEEQFQQTGLESNRSNFT
GTRSRVHRFGEAFRGELSNLQAATLFGSWQLREDYDASLVYHKFWRVDDDSDIGTSGINAALQPG
EKDIGQELDLVVTKYFKQGLLPASMSQYVDEPSALIRFRGGLFKPGDAYGPGTDSTMHRAFVDFI
WRF
>3P2YA
MAHHHHHHMGTLEAQTQGPGSMTLIGVPRESAEGERRVALVPKVVEKLSARGLEVVVESAAGAGA
LFSDADYERAGATIGDPWPADVVVKVNPPTSDEISQLKPGSVLIGFLAPRTQPELASRLRIADVT
AFAMESIPRISRAQTMDALSSQANVAGYKAVLLGASLSTRFVPMLTTAAGTVKPASALVLGVGVA
GLQALATAKRLGAKTTGYDVRPEVAEQVRSVGAQWLDLGIDAAGEGGYARELSEAERAQQQQALE
DAITKFDIVITTALVPGRPAPRLVTAAAATGMQPGSVVVDLAGETGGNCELTEPGRTIVHHGVTI
TSPLNLPATMPEHASELYAKNVTALLDLLLTDDGVAPDFTDEIVAASCITRTEGDI
>3NQNA
GMILSAEQSFTLRHPHGQAAALAFVREPAAALAGVRFLRGLDSDGEQVWGELLVTVPLLGEVDLP
FRSEIVRTPQGAELRPLTLTGERAWVAVSGQATAAEGGEMAFAFQFQAHLATPEAEGWGGAAFEK
MVQAAAGRTLERVAKALPEGLAAGLPPA
>2P0NA
SNAMNPFETKSVTFAEPIEMLYACHGKVRRFCGQVAMLSDYIAENGCNQIVLQTIRQIAQYFNVA
APLHHEDEEENFFPLLLQYAPQAQESVDELLRQHIGLHDNWAAVSAEFAKLEADNAYVPDEEAFK
RFVAGYDVHLAIEEPLFDMGNTFIPKEKLTEIGEIMAARRRK
>2VSGA
THFGVKYELWQPECELTAELRKTAGVAKMKVNSDLNSFKTLELTKMKLLTFAAKFPESKEALTLR
ALEAALNTDLRALRDNIANGIDRAVRATAYASEAAGALFSGIQTLHDATDGTTYCLSASGQGSNG
NAAMASQGCKPLALPELLTEDSYNTDVISDKGFPKISPLTNAQGQGKSGECGLFQAASGAQATNT
GVQFSGGSRINLGLGAIVASAAQQPTRPDLSDFSGTARNQADTLYGKAHASITELLQLAQGPKPG
QTEVETMKLLAQKTAALDSIKFQLAASTGKKTSDYKEDENLKTEYFGKTESNIEALWNKVKEEKV
KGADPEDPSKESKISDLNTEEQLQRVLDYYAVA
>3VZ9D
GYEREDDGVPSAAYVTQLYYKISRIDWDYEVEPARIKGIHYGPDIAQPINMDSSHHSRCFISDYL
WSLVPTAW
>2OLTA
GMPVNSILGVFAKSPIKPLQEHMDKVYDCASLLVPFFEATITGNWDDAVQIRKQISLAEKQGDSL
KREIRLTLPSGLFMPVERTDLLELLTQQDKIANKAKDISGRVIGRQLLIPQALQVPFIAYLQRCI
DAVGLAQQVINELDDLLEAGFRGREVDFVAKMINELDIIEEDTDDLQIQLRRQLFALESELNPVD
VMFLYKTIEWVGGLADLAERVGSRLELMLARV
>4IE5A

MGSSHHHHHHSSGLVPRGSHMTPKDDEFYQQWQLKYPKLILREASSVSEELHKEVQEAFLTLHKH
GCLFRDLVRIQGKDLLTPVSRILIGNPGCTYKYLNTRLFTVPWPVKGSNIKHTEAEIAAACETFL
KLNDYLQIETIQALEELAAKEKANEDAVPLCMSADFPRVGMGSSYNGQDEVDIKSRAAYNVTLLN
FMDPQKMPYLKEEPYFGMGKMAVSWHHDENLVDRSAVAVYSYSCEGPEEESEDDSHLEGRDPDIW
HVGFKISWDIETPGLAIPLHQGDCYFMLDDLNATHQHCVLAGSQPRFSSTHRVAECSTGTLDYIL
QRCQLALQNVCDDVDNDDVSLKSFEPAVLKQGEEIHNEVEFEWLRQFWFQGNRYRKCTDWWCQPM
AQLEALWKKMEGVTNAVLHEVKREGLPVEQRNEILTAILASLTARQNLRREWHARCQSRIARTLP
ADQKPECRPYWEKDDASMPLPFDLTDIVSELRGQLLEAKP

>1L4DB

NNSQLVVSVAGTVEGTNQDISLKFFEIDLTSRPASKPFATDSGAMPHKLEKADLLKAIQEQLIAN
VHSNDDYFEVIDFASDATITDRNGKVYFADKDGSVTLPTQPVQEFLLSGHVRVRPYK

>1KMIZ

MMQPSIKPADEHSAGDIIARIGSLTRMLRDSLRELGLDQAIAEAAEAIPDARDRLYYVVQMTAQA
AERALNSVEASQPHQDQMEKSAKALTQRWDDWFADPIDLADARELVTDTRQFLADVPAHTSFTNA
QLLKIMMAQDFQDLTGQVIKRMMDVIQEIERQLLMVLLENIPEQESRPKRENQSLLNGPQVDTSK
AGVVASQDQVDDLLDSLGF

>3GE2A

SNAAQQVQQPVAQQQVQQPAQQNTNTANAGGNQNQAAPVQNQPVAQPTDIDGTYTGQDDGDRITL
VVTGTTGTWTELESDGDQKVKQVTFDSANQRMIIGDDVKIYTVNGNQIVVDDMDRDPSDQIVLTK

>2ICUA

GSSGSSGMCGRFAQSQTREDYLALLAEDIERDIPYDPEPIGRYNVAPGTKVLLLSERDEHLHLDP
VFWGYAPGWWDKPPLINARVETAATSRMFKPLWQHGRAICFADGWFEWKKEGDKKQPFFIYRADG
QPIFMAAIGSTPFERGDEAEGFLIVTAAADQGLVDIHDRRPLVLSPEAAREWMRQEISGKEASEI
AASGCVPANQFSWHPVSRAVGNVKNQGAELIQPV

>1SCFA

MKKTQTWILTCIYLQLLLFNPLVKTEGICRNRVTNNVKDVTKLVANLPKDYMITLKYVPGMDVLP
SHCWISEMVVQLSDSLTDLLDKFSNISEGLSNYSIIDKLVNIVDDLVECVKENSSKDLKKSFKSP
EPRLFTPEEFFRIFNRSIDAFKDFVVASETSDCVVSSTLSPEKDSRVSVTKPFMLPPVAASSLRN
DSSSSNRKAKNPPGDSSLHWAAMALPALFSLIIGFAFGALYWKKRQPSLTRAVENIQINEEDNEI
SMLQEKEREFQEV

>3DO6A

GMKPIKEIADQLELKDDILYPYGHYIAKIDHRFLKSLENHEDGKLILVTAVTPTPAGEGKTTTSI
GLSMSLNRIGKKSIVTLREPSLGPTLGLKGGATGGGRSRVLPSDEINLHFTGDMHAVASAHNLLA
AVLDSHIKHGNELKIDITRVFWKRTMDMNDRALRSIVIGLGGSANGFPREDSFIITAASEVMAIL
ALSENMKDLKERLGKIIVALDADRKIVRISDLGIQGAMAVLLKDAINPNLVQTTEGTPALIHCGP
FANIAHGTNSIIATKMAMKLSEYTVTEAGFGADLGAEKFIDFVSRVGGFYPNAAVLVATVRALKY
HGGANLKNIHEENLEALKEGFKNLRVHVENLRKFNLPVVVALNRFSTDTEKEIAYVVKECEKLGV
RVAVSEVFKKGSEGGVELAKAVAEAAKDVEPAYLYEMNDPVEKKIEILAKEIYRAGRVEFSDTAK
NALKFIKKHGFDELPVIVAKTPKSISHDPSLRGAPEGYTFVVSDLFVSAGAGFVVALSGDINLMP
GLPKKPNALNMDVDDSGNIVGVS

>3B9WA

SAVAPAEINEARLVAQYNYSINILAMLLVGFGFLMVFVRRYGFSATTGTYLVVATGLPLYILLRA
NGIFGHALTPHSVDAVIYAEFAVATGLIAMGAVLGRLRVFQYALLALFIVPVYLLNEWLVLDNAS
GLTEGFQDSAGSIAIHAFGAYFGLGVSIALTTAAQRAQPIESDATSDRFSMLGSMVLWLFWPSFA

TAIVPFEQMPQTIVNTLLALCGATLATYFLSALFHKGKASIVDMANAALAGGVAIGSVCNIVGPV
GAFVIGLLGGAISVVGFVFIQPMLESKAKTIDTCGVHNLHGLPGLLGGFSAILIVPGIAVAQLTG
IGITLALALIGGVIAGALIKLTGTTKQAYEDSHEFIHLAGPEDEHKAERLVLEAKTEIQGLKNRI
DAAVLSAKSEGHHHHHH
>3CUZA
TEQATTTDELAFTRPYGEQEKQILTAEAVEFLTELVTHFTPQRNKLLAARIQQQQDIDNGTLPDF
ISETASIRDADWKIRGIPADLEDRRVEITGPVERKMVINALNANVKVFMADFEDSLAPDWNKVID
GQINLRDAVNGTISYTNEAGKIYQLKPNPAVLICRVRGLHLPEKHVTWRGEAIPGSLFDFALYFF
HNYQALLAKGSGPYFYLPKTQSWQEAAWWSEVFSYAEDRFNLPRGTIKATLLIETLPAVFQMDEI
LHALRDHIVGLNCGRWDYIFSYIKTLKNYPDRVLPDRQAVTMDKPFLNAYSRLLIKTCHKRGAFA
MGGMAAFIPSKDEEHNNQVLNKVKADKSLEANNGHDGTWIAHPGLADTAMAVFNDILGSRKNQLE
VMREQDAPITADQLLAPCDGERTEEGMRANIRVAVQYIEAWISGNGCVPIYGLMEDAATAEISRT
SIWQWIHHQKTLSNGKPVTKALFRQMLGEEMKVIASELGEERFSQGRFDDAARLMEQITTSDELI
DFLTLPGYRLLA
>4EYSA
SNAMVSTIGIVSLSSGIIGEDFVKHEVDLGIQRLKDLGLNPIFLPHSLKGLDFIKDHPEARAEDL
IHAFSDDSIDMILCAIGGDDTYRLLPYLFENDQLQKVIKQKIFLGFSDTTMNHLMLHKLGIKTFY
GQSFLADICELDKEMLAYSLHYFKELIETGRISEIRPSDVWYEERTDFSPTALGTPRVSHTNTGF
DLLQGSAQFEGKILGGCLESLYDIFDNSRYADSTELCQKYKLFPDLSDWEGKILLLETSEEKPKP
EDFKKMLLTLKDTGIFAVINGLLVGKPMDETFHDDYKEALLDIIDSNIPIVYNLNVGHATPRAIV
PFGVHAHVDAQEQVILFDYNK
>3AVRA
GPGYQDPNSQIIPSMSVSIYPSSAEVLKACRNLGKNGLSNSSILLDKCPPPRPPSSPYPPLPKDK
LNPPTPSIYLENKRDAFFPPLHQFCTNPNNPVTVIRGLAGALKLDLGLFSTKTLVEANNEHMVEV
RTQLLQPADENWDPTGTKKIWHCESNRSHTTIAKYAQYQASSFQESLREENEKRSHHKDHSDSES
TSSDNSGRRRKGPFKTIKFGTNIDLSDDKKWKLQLHELTKLPAFVRVVSAGNLLSHVGHTILGMN
TVQLYMKVPGSRTPGHQENNNFCSVNINIGPGDCEWFVVPEGYWGVLNDFCEKNNLNFLMGSWWP
NLEDLYEANVPVYRFIQRPGDLVWINAGTVHWVQAIGWCNNIAWNVGPLTACQYKLAVERYEWNK
LQSVKSIVPMVHLSWNMARNIKVSDPKLFEMIKYCLLRTLKQCQTLREALIAAGKEIIWHGRTKE
EPAHYCSICEVEVFDLLFVTNESNSRKTYIVHCQDCARKTSGNLENFVVLEQYKMEDLMQVYDQF
TLAPPLPSASS
>3C9UA
MGSHHHHHHDITSLYKKAGSAAAVLEENLYFQGSFTMRLKELGEFGLIDLIKKTLESKVIGDDTA
PVEYCSKKLLLTTDVLNEGVHFLRSYIPEAVGWKAISVNVSDVIANGGLPKWALISLNLPEDLEV
SYVERFYIGVKRACEFYKCEVVGGNISKSEKIGISVFLVGETERFVGRDGARLGDSVFVSGTLGD
SRAGLELLLMEKEEYEPFELALIQRHLRPTARIDYVKHIQKYANASMDISDGLVADANHLAQRSG
VKIEILSEKLPLSNELKMYCEKYGKNPIEYALFGGEDYQLLFTHPKERWNPFLDMTEIGRVEEGE
GVFVDGKKVEPKGWKHF
>1RTTA
MSLSDDIKVLGISGSLRSGSYNSAALQEAIGLVPPGMSIELADISGIPLYNEDVYALGFPPAVER
FREQIRAADALLFATPEYNYSMAGVLKNAIDWASRPPEQPFSGKPAAILGASAGRFGTARAQYHL
RQTLVFLDVHPLNKPEVMISSAQNAFDAQGRLLDDKARELIQQQLQALQLWVREGGSHHHHHH
>3LYDA
SNAMPSIRYPSTEFPALTGFTVPIPETWQPDPTMGTQFAARPHTPPQGFTPNIIGTVRRAATGAL

HNQRTELDQRATQLPDYAERGRTETTVDGFPAYHIEYAYRHHGTITIAQMITLVEVSHPHAVDII
QLTATCAGDQTADYWDTFRLMHADLTVQPHG
>3W4SA
MKCLVVGHVVRDIVKKGNKVLERLGGGAYYSALALSRFCDVEILTSFSNLPEEWIKELESMAKLQ
VVPSETTTTYELTYLDGNRRRLKLLERASPIEELPDGEYDVLLMNPVAREVPPALVTSALKKFPF
VAVDIQGFIRSSSPGEIQYQPIDGSFLKGVKILHADLGEYQYLQGFSPEFVDVLLLSNGPEPGKA
FLHGREYTFEPVHVGVDESTGAGDVFLGAFTGFYSQCPFVQALKRAAAFTALFLKNRSVDFSMDD
VNELAMKVEVKRV
>1T0BA
SNAMTTPIRVVVWNEFRHEKKDEQVRAIYPEGMHTVIASYLAEAGFDAATAVLDEPEHGLTDEVL
DRCDVLVWWGHIAHDEVKDEVVERVHRRVLEGMGLIVLHSGHFSKIFKKLMGTTCNLKWREADEK
ERLWVVAPGHPIVEGIGPYIELEQEEMYGEFFDIPEPDETIFISWFEGGEVFRSGCTFTRGKGKI
FYFRPGHETYPTYHHPDVLKVIANAVRWAAPVNRGEIVFGNVKPLEPIKAKQGGVTQ
>4K1CA
GPSSPMDATTPLLTVANSHPARNPKHTAWRAAVYDLQYILKASPLNFLLVFVPLGLIWGHFQLSH
TLTFLFNFLAIIPLAAILANATEELADKAGNTIGGLLNATFGNAVELIVSIIALKKGQVRIVQAS
MLGSLLSNLLLVLGLCFIFGGYNRVQQTFNQTAAQTMSSLLAIACASLLIPAAFRATLPHGKEDH
FIDGKILELSRGTSIVILIVYVLFLYFQLGSHHALFEQQEEETDEVMSTISRNPHHSLSVKSSLV
ILLGTTVIISFCADFLVGTIDNVVESTGLSKTFIGLIVIPIVGNAAEHVTSVLVAMKDKMDLALG
VAIGSSLQVALFVTPFMVLVGWMIDVPMTLNFSTFETATLFIAVFLSNYLILDGESNWLEGVMSL
AMYILIAMAFFYYPDEKTLDSIGNSLGLVPR
>3BVFA
MGSSHHHHHHSQDPMLSKDIIKLLNEQVNKEMNSSNLYMSMSSWCYTHSLDGAGLFLFDHAAEEY
EHAKKLIIFLNENNVPVQLTSISAPEHKFEGLTQIFQKAYEHEQHISESINNIVDHAIKSKDHAT
FNFLQWYVAEQHEEEVLFKDILDKIELIGNENHGLYLADQYVKGIAKSRKS
>3HZPA
GMSSKEEILSILEAFASTERMGSFFLDNATADFLFIRPSGNPLDAKGFENMWSSGDLVLESAEIT
KVHKFELLGSNAAICVFTLGSKFTYKGTQNDDLPTVTSIFKKIDEKWKVAWMQRSSGQSDMTLWN
E
>3I16A
GMLESTKQFLKKYNINDRVLKLYETAMNDIQNQFKILDDIREFNQLKVLNAFQEERISEAHFTNS
SGYGYGDIGRDSLDAVYARVFNTESALVRPHFVNGTHALGAALFGNLRPGNTMLSVCGEPYDTLH
DVIGITENSNMGSLKEFGINYKQVDLKEDGKPNLEEIEKVLKEDESITLVHIQRSTGYGWRRALL
IEDIKSIVDCVKNIRKDIICFVDNCYGEFMDTKEPTDVGADLIAGSLIKNIGGGIAPTGGYLAGT
KDCIEKTSYRLTVPGIGGECGSTFGVVRSMYQGLFLAPHISMEALKGAILCSRIMELAGFEVMPK
YDEKRSDIIQSIKFNDKDKLIEFCKGIQTGSPIDSFVSCEPWDMPGYTDQVIMAAGAFIQGSSIE
LSADAPIREPYIAYLQGGLTFDHAKIGILIALSRIVK
>1UYNX
DGVRIFNSLAATVYADSTAAHADMQGRRLKAVSDGLDHNGTGLRVIAQTQQDGGTWEQGGVEGKM
RGSTQTVGIAAKTGENTTAAATLGMGRSTWSENSANAKTDSISLFAGIRHDAGDIGYLKGLFSYG
RYKNSISRSTGADEHAEGSVNGTLMQLGALGGVNVPFAATGDLTVEGGLRYDLLKQDAFAEKGSA
LGWSGNSLTEGTLVGLAGLKLSQPLSDKAVLFATAGVERDLNGRDYTVTGGFTGATAATGKTGAR
NMPHTRLVAGLGADVEFGNGWNGLARYSYAGSKQYGNHSGRVGVGYRF
>2HFNA

AGHMSLYRLIYSSQGIPNLQPQDLKDILESSQRNNPANGITGLLCYSKPAFLQVLEGECEQVNET
YHRIVQDERHHSPQIIECMPIRRRNFEVWSMQAITVNDLSTEQVKTLVLKYSGFTTLRPSAMDPE
QCLNFLLDIAKIYELSDNFFLDL
>3DFUA
GMQAPRLRVGIFDDGSSTVNMAEKLDSVGHYVTVLHAPEDIRDFELVVIDAHGVEGYVEKLSAFA
RRGQMFLHTSLTHGITVMDPLETSGGIVMSAHPIGQDRWVASALDELGETIVGLLVGELGGSIVE
IADDKRAQLAAALTYAGFLSTLQRDASYFLDEFLGDPDVTSDIVMDSAQQFQALPSLDEVIAQYD
SINNPGRQRLFRDLARRQAEISRAQDIELWAIQKEDR
>4IX1A
MTHWSADYGWRGKVGLISTPVIENAHVELARVAPEGVGVYQTFPYVPNFRVDATNIKRAVEQLET
SAAALGSAGVDIVGQVGTPFSFAGGTGLEWAEDISTKLEKASGKPVALMGLSIVEALQERGYKTV
AISSTYYSRELSERYTQFLEAGGIRVLTIKNWVDQKRFPDEESVDGRNLWYPASYAYKSAREVAA
EAPEADCIIMSGAAVHTMDIIAPLEADLGKPVISSDSAFFWKILSLLGVRETSGGWGSLLDSL
>3GNJA
SNAMSLEKLDTNTFEQLIYDEGKACLVMFSRKNCHVCQKVTPVLEELRLNYEESFGFYYVDVEEE
KTLFQRFSLKGVPQILYFKDGEYKGKMAGDVEDDEVEQMIADVLED
>1R7AA
MKNKVQLITYADRLGDGTIKSMTDILRTRFDGVYDGVHILPFFTPFDGADAGFDPIDHTKVDERL
GSWDDVAELSKTHNIMVDAIVNHMSWESKQFQDVLAKGEESEYYPMFLTMSSVFPNGATEEDLAG
IYRPRPGLPFTHYKFAGKTRLVWVSFTPQQVDIDTDSDKGWEYLMSIFDQMAASHVSYIRLDAVG
YGAKEAGTSCFMTPKTFKLISRLREEGVKRGLEILIEVHSYYKKQVEIASKVDRVYDFALPPLLL
HALSTGHVEPVAHWTDIRPNNAVTVLDTHDGIGVIDIGSDQLDRSLKGLVPDEDVDNLVNTIHAN
THGESQAATGAAASNLDLYQVNSTYYSALGCNDQHYIAARAVQFFLPGVPQVYYVGALAGKNDME
LLRKTNNGRDINRHYYSTAEIDENLKRPVVKALNALAKFRNELDAFDGTFSYTTDDDTSISFTWR
GETSQATLTFEPKRGLGVDNTTPVAMLEWEDSAGDHRSDDLIANPPVVA
>2HY5C
MSILHTVNKSPFERNSLESCLKFATEGASVLLFEDGIYAALAGTRVESQVTEALGKLKLYVLGPD
LKARGFSDERVIPGISVVDYAGFVDLTTECDTVQAWL
>2Z5BA
GSHMLVKTISRTIESESGFLQPTLDVIATLPADDRSKKIPISLVVGFKQEASLNSSSSLSCYYYA
IPLMRDRHINLKSGGSNVVGIPLLDTKDDRIRDMARHMATIISERFNRPCYVTWSSLPSEDPSML
VANHLYILKKCLDLLKTELGE
>4INDA
MSVTTLGQSFPANAKVKYYYKLSEKQDLDAFVNSIFVGSYKLKQISYLLYGNTKIVSAPVVPLGP
NASIIIDDELQEGLYLIRIKVYNTNSFSVTVTPFFNNNNTMTYSIGANSEFEIYDIFTKEQGNIY
YIQLPPGLAILEFSLERVFEKGNRINIPKIIHTSGNGYISFRLRKGTYAIKMPYSYNNTTSTTFT
NFQFGTISTSVATIPLVISSIPANGSGSGTFLVYLKITGDYEDVKFSVTYGGGLGVPFTFGLEVE
EINELVENTNFVTQSVTLSGSQVTQSILNVQGSGSHLRLKYASVSGLTTAVTQCQLQATNLNRST
TYSTVWDFIAGGSSTPPSWDIREINSIQLVANGGSSTSSVTITLILVYEQIAGELSHHHHHH
>2Q3TA
SELRPSGDSGSSDVDAEISDGFSPLDTSHRDVADEGSLLRRAEMYQDYMKQVPIPTNRGSLIPFT
SWVGLSISMKQLYGQPLHYLTNVLLQRWDQSRFGTDSEEQRLDSIIHPTKAEATIWLVEEIHRLT
PSHLHMALLWRSDPMYHSFIDPIFPEK
>2Y0NE

GAMGIQESEPEVTSFFPEPDDVESLLITPFLPVVAFGRPLPKLAPQNFELPWLDER
>3OC9A
GPGSMTYQPVDITTNTIPVTKEHYYRGLELISQGKTALITPAGGQGSRLGFEHPKGMFVLPFEIP
KSIFQMTSERLLRLQELASEYSHQKNVMIHWFLMTNEETIEEINNYFKEHQYFGLSSEQIHCFPQ
GMLPVVDFNGKILYEKKDKPYMAPNGHGGLFKALKDNGILEFMNEKGIKYSVAHNVDNILCKDVD
PNMIGYMDLLQSEICIKIVKKGFKEEKVGVLVKEQERIKVVEYTELTDELNKQLSNGEFIYNCGH
ISINGYSTSFLEKAAEYQLPYHIAKKKVPFVNEQGIVIHPSENNGIKKEIFFFDVFPLATKVSIF
EIQRFIEFSALKNSLNESFDNVNTVKRDWYRLNIYYLKKAGAIVDDSKSPICEISFRKSFEEEGL
KEFKGKTIQLPFILQ
>2GNPA
SNANFDTNMFKLENYVKEKYSLESLEIIPNEFDDTPTILSERISQVAAGVLRNLIDDNMKIGFSW
GKSLSNLVDLIHSKSVRNVHFYPLAGGPSHIHAKYHVNTLIYEMSRKFHGECTFMNATIVQENKL
LADGILQSRYFENLKNSWKDLDIAVVGIGDFSNKGKHQWLDMLTEDDFKELTKVKTVGEICCRFF
DSKGKEVYENLQERTIAISLEDLKNIPQSLAVAYGDTKVSSILSVLRANLVNHLITDKNTILKVL
EEDGDL
>1R1HA
GICKSSDCIKSAARLIQNMDATTEPCTDFFKYACGGWLKRNVIPETSSRYGNFDILRDELEVVLK
DVLQEPKTEDIVAVQKAKALYRSCINESAIDSRGGEPLLKLLPDIYGWPVATENWEQKYGASWTA
EKAIAQLNSKYGKKVLINLFVGTDDKNSVNHVIHIDQPRLGLPSRDYYECTGIYKEACTAYVDFM
ISVARLIRQEERLPIDENQLALEMNKVMELEKEIANATAKPEDRNDPMLLYNKMTLAQIQNNFSL
EINGKPFSWLNFTNEIMSTVNISITNEEDVVVYAPEYLTKLKPILTKYSARDLQNLMSWRFIMDL
VSSLSRTYKESRNAFRKALYGTTSETATWRRCANYVNGNMENAVGRLYVEAAFAGESKHVVEDLI
AQIREVFIQTLDDLTWMDAETKKRAEEKALAIKERIGYPDDIVSNDNKLNNEYLELNYKEDEYFE
NIIQNLKFSQSKQLKKLREKVDKDEWISGAAVVNAFYSSGRNQIVFPAGILQPPFFSAQQSNSLN
YGGIGMVIGHEITHGFDDNGRNFNKDGDLVDWWTQQSASNFKEQSQCMVYQYGNFSWDLAGGQHL
NGINTLGENIADNGGLGQAYRAYQNYIKKNGEEKLLPGLDLNHKQLFFLNFAQVWCGTYRPEYAV
NSIKTDVHSPGNFRIIGTLQNSAEFSEAFHCRKNSYMNPEKKCRVW
>3RF3A
MPVFHTRTIESILEPVAQQISHLVIMHEEGEVDGKAIPDLTAPVAAVQAAVSNLVRVGKETVQTT
EDQILKRDMPPAFIKVENACTKLVQAAQMLQSDPYSVPARDYLIDGSRGILSGTSDLLLTFDEAE
VRKIIRVCKGILEYLTVAEVVETMEDLVTYTKNLGPGMTKMAKMIDERQQELTHQEHRVMLVNSM
NTVKELLPVLISAMKIFVTTKNSKNQGIEEALKNRNFTVEKMSAEINEIIRVLQLTSWDEDAW
>1J1TA
DNSNGSTIPSSITSGSIFDLEGDNPNPLVDDSTLVFVPLEAQHITPNGNGWRHEYKVKESLRVAM
TQTYEVFEATVKVEMSDGGKTIISQHHASDTGTISKVYVSDTDESGFNDSVANNGIFDVYVRLRN
TSGNEEKFALGTMTSGETFNLRVVNNYGDVEVTAFGNSFGIPVEDDSQSYFKFGNYLQSQDPYTL
DKCGEAGNSNSFKNCFEDLGITESKVTMTNVTYTRETN
>1YPXA
MNQVAPFYADHVGSILRTKGIKDAREKFQSGEITALELRKIENTEIKYIVEKQKEVGLKSITDGE
FRRAWWHFDFLENLDGVEGYDAAGGIQFSKVQTKSHSVKITGPIDFTTHPFIEDFIFLKEAVGDN
HVAKQTIPSPAMLHYRGDIEYQPYLDDAEKFANDLATAYQKAIQAFYDAGCRYLQLDDTSWSYLC
SDEQREVVRQRGFDPETLQETYKNLINEAIKHKPADMVITMHICRGNFRSTWIAEGGYGPVAETL
FGKLNIDGFFLEYDNERSGDFAPLKYVTRPDLKIVLGLITSKTGELEDEAAIKARIEEASEIVPL
SQLRLSPQCGFASTEEGNILTEEEQWDKLRYVVRLANDIWGELEHHHHHH

>2R85A
MKVRIATYASHSALQILKGAKDEGFETIAFGSSKVKPLYTKYFPVADYFIEEKYPEEELLNLNAV
VVPTGSFVAHLGIELVENMKVPYFGNKRVLRWESDRNLERKWLKKAGIRVPEVYEDPDDIEKPVI
VKPHGAKGGKGYFLAKDPEDFWRKAEKFLGIKRKEDLKNIQIQEYVLGVPVYPHYFYSKVREELE
LMSIDRRYESNVDAIGRIPAKDQLEFDMDITYTVIGNIPIVLRESLLMDVIEAGERVVKAAEELM
GGLWGPFCLEGVFTPDLEFVVFEISARIVAGTNIFVNGSPYTWLRYDRPVSTGRRIAMEIREAIE
NDMLEKVLT
>1OGOX
HGTTANTHCGADFCTWWHDSGEINTQTPVQPGNVRQSHKYSVQVSLAGTNNFHDSFVYESIPRNG
NGRIYAPTDPPNSNTLDSSVDDGISIEPSIGLNMAWSQFEYSHDVDVKILATDGSSLGSPSDVVI
RPVSISYAISQSDDGGIVIRVPADANGRKFSVEFKTDLYTFLSDGNEYVTSGGSVVGVEPTNALV
IFASPFLPSGMIPHMTPDNTQTMTPGPINNGDWGAKSILYFPPGVYWMNQDQSGNSGKLGSNHIR
LNSNTYWVYLAPGAYVKGAIEYFTKQNFYATGHGILSGENYVYQANAGDNYIAVKSDSTSLRMWW
HNNLGGGQTWYCVGPTINAPPFNTMDFNGNSGISSQISDYKQVGAFFFQTDGPEIYPNSVVHDVF
WHVNDDAIKIYYSGASVSRATIWKCHNDPIIQMGWTSRDISGVTIDTLNVIHTRYIKSETVVPSA
IIGASPFYASGMSPDSRKSISMTVSNVVCEGLCPSLFRITPLQNYKNFVVKNVAFPDGLQTNSIG
TGESIIPAASGLTMGLAISAWTIGGQKVTMENFQANSLGQFNIDGSYWGEWQIS
>1CLVI
CIPKWNRCGPKMDGVPCCEPYTCTSDYYGNCS
>3EMNX
MRGSHHHHHHGSMAVPPTYADLGKSARDVFTKGYGFGLIKLDLKTKSENGLEFTSSGSANTETTK
VNGSLETKYRWTEYGLTFTEKWNTDNTLGTEITVEDQLARGLKLTFDSSFSPNTGKKNAKIKTGY
KREHINLGCDVDFDIAGPSIRGALVLGYEGWLAGYQMNFETSKSRVTQSNFAVGYKTDEFQLHTN
VNDGTEFGGSIYQKVNKKLETAVNLAWTAGNSNTRFGIAAKYQVDPDACFSAKVNNSSLIGLGYT
QTLKPGIKLTLSALLDGKNVNAGGHKLGLGLEFQA
>3AG3F
ASGGGVPTDEEQATGLEREVMLAARKGQDPYNILAPKATSGTKEDPNLVPSITNKRIVGCICEED
NSTVIWFWLHKGEAQRCPSCGTHYKLVPHQLAH
>4AQ4A
GSHMVTTIPFWHSMEGELGKEVDSLAQRFNAENPDYKIVPTYKGNYEQNLSAGIAAFRTGNAPAI
LQVYEVGTATMMASKAIKPVYDVFKEAGIQFDESQFVPTVSGYYSDSKTGHLLSQPFNSSTPVLY
YNKDAFKKAGLDPEQPPKTWQDLADYAAKLKASGMKCGYASGWQGWIQLENFSAWNGLPFASKNN
GFDGTDAVLEFNKPEQVKHIAMLEEMNKKGDFSYVGRKDESTEKFYNGDCAMTTASSGSLANIRE
YAKFNYGVGMMPYDADAKDAPQNAIIGGASLWVMQGKDKETYTGVAKFLDFLAKPENAAEWHQKT
GYLPITKAAYDLTREQGFYEKNPGADTATRQMLNKPPLPFTKGLRLGNMPQIRVIVDEELESVWT
GKKTPQQALDTAVERGNQLLRRFEKSTKS
>1Y8XB
GSSQLPQNIQFSPSAKLQEVLDYLTNSASLQMKSPAITATLEGKNRTLYMQSVTSIEERTRPNLS
KTLKELGLVDGQELAVADVTTPQTVLFKLHFTS
>3EH1A
QPESLRPVNLTQERNILPMTPVWAPVPNLNADLKKLNCSPDSFRCTLTNIPQTQALLNKAKLPLG
LLLHPFRDLTQLPVITSNTIVRCRSCRTYINPFVSFIDQRRWKCNLCYRVNDVPEEFMYNPLTRS
YGEPHKRPEVQNSTVEFIASSDYMLRPPQPAVYLFVLDVSHNAVEAGYLTILCQSLLENLDKLPG
DSRTRIGFMTFDSTIHFYNLQEGLSQPQMLIVSDIDDVFLPTPDSLLVNLYESKELIKDLLNALP

NMFTNTRETHSALGPALQAAFKLMSPTGGRVSVFQTQLPSLGAGLLQSREDPNQRSSTKVVQHLG
PATDFYKKLALDCSGQQTAVDLFLLSSQYSDLASLACMSKYSAGCIYYYPSFHYTHNPSQAEKLQ
KDLKRYLTRKIGFEAVMRIRCTKGLSMHTFHGNFFVRSTDLLSLANINPDAGFAVQLSIEESLTD
TSLVCFQTALLYTSSKGERRIRVHTLCLPVVSSLADVYAGVDVQAAICLLANMAVDRSVSSSLSD
ARDALVNAVVDSLSAYGSTVSNLQHSALMAPSSLKLFPLYVLALLKQKAFRTGTSTRLDDRVYAM
CQIKSQPLVHLMKMIHPNLYRIDRLTDEGAVHVNDRIVPQPPLQKLSAEKLTREGAFLMDCGSVF
YIWVGKGCDNNFIEDVLGYTNFASIPQKMTHLPELDTLSSERARSFITWLRDSRPLSPILHIVKD
ESPAKAEFFQHLIEDRTEAAFSYYEFLLHVQQQICK

>2XVEA

MATRIAILGAGPSGMAQLRAFQSAQEKGAEIPELVCFEKQADWGGQWNYTWRTGLDENGEPVHSS
MYRYLWSNGPKECLEFADYTFDEHFGKPIASYPPREVLWDYIKGRVEKAGVRKYIRFNTAVRHVE
FNEDSQTFTVTVQDHTTDTIYSEEFDYVVCCTGHFSTPYVPEFEGFEKFGGRILHAHDFRDALEF
KDKTVLLVGSSYSAEDIGSQCYKYGAKKLISCYRTAPMGYKWPENWDERPNLVRVDTENAYFADG
SSEKVDAIILCTGYIHHFPFLNDDLRLVTNNRLWPLNLYKGVVWEDNPKFFYIGMQDQWYSFNMF
DAQAWYARDVIMGRLPLPSKEEMKADSMAWREKELTLVTAEEMYTYQGDYIQNLIDMTDYPSFDI
PATNKTFLEWKHHKKENIMTFRDHSYRSLMTGTMAPKHHTPWIDALDDSLEAYLSDKSEIPVAKE
ALEHHHHHH

>3BFMA

GMSETITFPPLMTGEAAGPGQDPFDLACQKAELGVDAGLVVYELGTDVLRAALVLAPEVPLAKAM
AMLPVCGVGFQNALGALAPPEVAVHLDWNGALRINGARCGRLRIAASTDDPDTQPDWLVVGLDLP
LWPEGDGGETPDETALYAEGCADVAAPRLLESWARHCLHWINRWDEGELETIHGEWRGLAHGMGE
ARTEAGRSGTFLGVDEDFGMLLRDETTTHLIPLTTVLVQD

>2HEUA

MHHHHHHLEVLFQGPSSTVTIEYFNQKKEMTKTLEEITRDFEKENPKIKVKVVNVPNAGEVLKTR
VLAGDVPDVVNIYPQSIELQEWAKAGVFEDLSNKDYLKRVKNGYAEKYAVNEKVYNVPFTANAYG
IYYNKDKFEELGLKVPETWDEFEQLVKDIVAKGQTPFGIAGADAWTLNGYNQLAFATATGGGKEA
NQYLRYSQPNAIKLSDPIMKDDIKVMDILRINGSKQKNWEGAGYTDVIGAFARGDVLMTPNGSWA
ITAINEQKPNFKIGTFMIPGKEKGQSLTVGAGDLAWSISATTKHPKEANAFVEYMTRPEVMQKYY
DVDGSPTAIEGVKQAGEDSPLAGMTEYAFTDRHLVWLQQYWTSEADFHTLTMNYVLTGDKQGMVN
DLNAFFNPMKM

>3I53A

MGKRAAHIGLRALADLATPMAVRVAATLRVADHIAAGHRTAAEIASAAGAHADSLDRLLRHLVAV
GLFTRDGQGVYGLTEFGEQLRDDHAAGKRKWLDMNSAVGRGDLGFVELAHSIRTGQPAYPVRYGT
SFWEDLGSDPVLSASFDTLMSHHLELDYTGIAAKYDWAALGHVVDVGGGSGGLLSALLTAHEDLS
GTVLDLQGPASAAHRRFLDTGLSGRAQVVVGSFFDPLPAGAGGYVLSAVLHDWDDLSAVAILRRC
AEAAGSGGVVLVIEAVAGDEHAGTGMDLRMLTYFGGKERSLAELGELAAQAGLAVRAAHPISYVS
IVEMTAL

>1GV9A

SQFVGSDGMGGDAAAPGAAGTQAELPHRRFEYKYSFKGPHLVQSDGTVPFWAHAGNAIPSADQIR
IAPSLKSQRGSVWTKTKAAFENWEVEVTFRVTGRGRIGADGLAIWYTENQGLDGPVFGSADMWNG
VGIFFDSFDNDGKKNNPAIVVVGNNGQINYDHQNDGATQALASCQRDFRNKPYPVRAKITYYQKT
LTVMINNGFTPDKNDYEFCAKVENMVIPTQGHFGISAATGGLADDHDVLSFLTFQLTEPGKEPPT

>3H6EA

MSLSTGATIVIDLGKTLSKVSLWDLDGRMLDRQVRPSIPLEIDGIRRLDAPDTGRWLLDVLSRYA

DHPVTTIVPVGHGAGIAALTDGRLAFPPLDYEQSIPEAVMADYRSQRDPFARTGSPALPDGLNIG
SQLWWLDQLHPDVMANATLLPWAQYWAWFLTGRAVSEVTSLGCHSDLWDPQDGDFSPMAKRLGWA
ARFAPIVRAGDTVGALLPAIAERTGLSPDVQVLAGLHDSNAALLAARGFAEIADNEATVLSTGTW
FIAMRLPATPVDTATLPEARDCLVNVDVHGRPVPSARFMGGREIETLIEIDTRRVDIKPDQPALL
AAVPEVLRHGRMILPTLMRGFGPYPHGRFAWINRPEDWFERRAAACLYAALVADTALDLIGSTGR
ILVEGRFAEADVFVRALASLRPDCAVYTANAHNDVSFGALRLIDPGLRPQGELVRIEPLDTGSWA
DLDTYRNRWQAEVEAAKVEEGHHHHHH
>2E6MA
KSVLEDNLPFLEFPGSIVYSYEASDCSFLSEDISMRLSDGDVVGFDMEWPPIYKPGKRSRVAVIQ
LCVSESKCYLFHISSMSVFPQGLKMLLENKSIKKAGVGIEGDQWKLLRDFDVKLESFVELTDVAN
EKLKCAETWSLNGLVKHVLGKQLLKDKSIRCSNWSNFPLTEDQKLYAATDAYAGLIIYQKLGNLG
DTVQVFALNKAEE
>1WWBX
VHFAPTITFLESPTSDHHWCIPFTVKGNPKPALQWFYNGAILNESKYICTKIHVTNHTEYHGCLQ
LDNPTHMNNGDYTLIAKNEYGKDEKQISAHFMGWPGID
>2AOTA
MASSMRSLFSDHGKYVESFRRFLNHSTEHQCMQEFMDKKLPGIIGRIGDTKSEIKILSIGGGAGE
IDLQILSKVQAQYPGVCINNEVVEPSAEQIAKYKELVAKTSNLENVKFAWHKETSSEYQSRMLEK
KELQKWDFIHMIQMLYYVKDIPATLKFFHSLLGTNAKMLIIVVSGSSGWDKLWKKYGSRFPQDDL
CQYITSDDLTQMLDNLGLKYECYDLLSTMDISDCFIDGNENGDLLWDFLTETCNFNATAPPDLRA
ELGKDLQEPEFSAKKEGKVLFNNTLSFIVIEA
>4G1OA
MDSSRTIGLYFDSALPSSNLLAFPIVLQDIGDGKKQIAPQYRIQRLDSWTDSKEDSVFITTYGFI
FQVGNEEVTVGMISDNPKHELLSAAMLCLGSVPNVGDLVELARACLTMVVTCKKSATDTERMVFS
VVQAPQVLQSCRVVANKYSSVNAVKHVKAPEKIPGSGTLEYKVNFVSLTVVPRKDVYKIPTAALK
VSGSSLYNLALNVTIDVEVDPKSPLVKSLSKSDSGYYANLFLHIGLMSTVDKKGKKVTFDKLERK
IRRLDLSVGLSDVLGPSVLVKARGARTRLLAPFFSSSGTACYPISNASPQVAKILWSQTARLRSV
KVIIQAGTQRAVAVTADHEVTSTKIEKRHTIAKYNPFKK
>3CI0I
GAMSNQHVLEEKTVAGWVAENQTALLYLMTRGQRAVRQQGESDMAGSRWYWRTTPLSTGNALLQA
VDIEVSLHEDFSSVIQSRRAWFSA
>3IH6A
SNAYTVEPVQDGERSVTLRRAGGTPLVAAMYHLPAAGSPDFVGLDLAATILADTPSSRLYHALVP
TKLASGVFGFTMDQLDPGLAMFGAQLQPGMDQDKALQTLTATLESLSSKPFSQEELERARSKWLT
AWQQTYADPEKVGVALSEAIASGDWRLFFLQRDRVREAKLDDVQRAAVAYLVRSNRTEGRYIPTE
KP
>2FT0A
MGSSHHHHHHGSPVRASIEPLTWENAFFGVNSAIVRITSEAPLLTPDALAPWSRVQAKIAASNTG
ELDALQQLGFSLVEGEVDLALPVNNVSDSGAVVAQETDIPALRQLASAAFAQSRFRAPWYAPDAS
GRFYAQWIENAVRGTFDHQCLILRAASGDIRGYVSLRELNATDARIGLLAGRGAGAELMQTALNW
AYARGKTTLRVATQMGNTAALKRYIQSGANVESTAYWLYR
>1W9ZA
MLSETELRALKKLSTTTSRVVGDSTLALPSNVKLSKGEVEKIAVTKKEMFDELAQCNLPTIELIT
REHTFNGDVIRFAAWLFLMNGQKLMIANNVAVRMGMQYATNLAGNNVKITYVTSNNVVKLGHIAA

GVLANPYSNKGSGLFITYEHNLISNQIETGKVCVLFITSLSTTASSTNSFAYSACSVPIEDWDFN
MIKLTAETSCASLTAMTNLVNSLVPGERTRPVGLYVDIPGVTVTTSASSGSLPLTTIPAVTPLIF
SAYTKQVEEVGVINTLYALSYLP

>1GCIA

AQSVPWGISRVQAPAAHNRGLTGSGVKVAVLDTGISTHPDLNIRGGASFVPGEPSTQDGNGHGTH
VAGTIAALNNSIGVLGVAPSAELYAVKVLGASGSGSVSSIAQGLEWAGNNGMHVANLSLGSPSPS
ATLEQAVNSATSRGVLVVAASGNSGAGSISYPARYANAMAVGATDQNNNRASFSQYGAGLDIVAP
GVNVQSTYPGSTYASLNGTSMATPHVAGAAALVKQKNPSWSNVQIRNHLKNTATSLGSTNLYGSG
LVNAEAATR

>3FUCA

MQNGYTYEDYQDTAKWLLSHTEQRPQVAVICGSGLGGLVNKLTQAQTFDYSEIPNFPESTVPGHA
GRLVFGILNGRACVMMQGRFHMYEGYPFWKVTFPVRVFRLLGVETLVVTNAAGGLNPNFEVGDIM
LIRDHINLPGFSGENPLRGPNEERFGVRFPAMSDAYDRDMRQKAHSTWKQMGEQRELQEGTYVML
GGPNFETVAECRLLRNLGADAVGMSTVPEVIVARHCGLRVFGFSLITNKVIMDYESQGKANHEEV
LEAGKQAAQKLEQFVSLLMASIPV

>3G2EA

MSLKYQLRFGGEGGQGVITAGEILAEAAIKEGRQAFKASTYTSQVRGGPTKVDIIIDDKEILFPY
AVEGEVDFMLSTADKGYKGFRGGVKEGGIIVVEPNLVHPESEDYKKWQIFEIPIITIAKDEVGNV
ATQSVVALAIAAYMSKCIDLDVLKETMLHMVPAKTRDANAKAFDLGVKYATQAKPHEGHHHHHH

>4E2XA

GHMSHLADVSPPTACRVCGGGVQEFLDLGRQPLSDRFRKPDELDDEFTYRLAVGRCDSCEMVQLT
EEVPRDLMFHEVYPYHSSGSSVMREHFAMLARDFLATELTGPDPFIVEIGCNDGIMLRTIQEAGV
RHLGFEPSSGVAAKAREKGIRVRTDFFEKATADDVRRTEGPANVIYAANTLCHIPYVQSVLEGVD
ALLAPDGVFVFEDPYLGDIVAKTSFDQIFDEHFFLFSATSVQGMAQRCGFELVDVQRLPVHGGEV
RYTLARQGSRTPSAAVAQLLAAEREQELSDMATLRAFAGNVVKIRDELTALLHRLRAEGRSVVGY
GATAKSATVTNFCGIGPDLVHSVYDTTPDKQNRLTPGAHIPVRPASAFSDPYPDYALLFAWNHAE
EIMAKEQEFHQAGGRWILYVPEVHIR

>3GZAA

GAQQQELPVPKPHQLKWHEAEMGAVFHYDLHVFDGIRYGQGNNRINPIEDYNIFNPTELNTDQWV
QAAKAAGCKFAVLTATHETGFGLWQSDVNPYCLKAVKWRDGKGDIVRDFVNSCRKYGLQPGIYIG
IRWNSLLGIHNFKAEGEGAFARNRQAWYKRLCEKMVTELCTRYGDLYMIWFDGGADDPRADGPDV
EPIVNKYQPNCLFYHNIDRADFRWGGSETGTVEYPCWSTFPVPCSHHKRIESSIDQLELLKHGDK
NGRYWVPAMADTPLRGANGRHEWFWEPDDENNIYPLNTLMDKYEKSVGRNATLILGLTPDPTGLI
PAGDAQRLKEMGDEINRRFSSPIARISGQKKSLTLKLGKEQSVNYCIIQENIKNGERIRQYQIEA
KVNGKWQTVCKGESVGHKRIEKFEPVEATALRLTVSESIALPDIINFSAYSVK

>4EADA

LFLAQEIIRKKRDGHALSDEEIRFFINGIRDNTISEGQIAALAMTIFFHDMTMPERVSLTMAMRD
SGTVLDWKSLHLNGPIVDKHSTGGVGDVTSLMLGPMVAACGGYIPMISGRGLGHTGGTLDKLESI
PGFDIFPDDNRFREIIKDVGVAIIGQTSSLAPADKRFYATRDITATVDSIPLITASILAKKLAEG
LDALVMDVKVGSGAFMPTYELSEALAEAIVGVANGAGVRTTALLTDMNQVLASSAGNAVEVREAV
QFLTGEYRNPRLFDVTMALCVEMLISGKLAKDDAEARAKLQAVLDNGKAAEVFGRMVAAQKGPTD
FVENYAKYLPTAMLTKAVYADTEGFVSEMDTRALGMAVVAMGGGRRQASDTIDYSVGFTDMARLG
DQVDGQRPLAVIHAKDENNWQEAAKAVKAAIKLADKAPESTPTVYRRISE

>3NZMA

KSPDPFCPGCLSFGTEILTVEYGPLPIGKIVSEEINCSVYSVDPEGRVYTQAIAQWHDRGEQEVL
EYELEDGSVIRATSDHRFLTTDYQLLAIEEIFARQLDLLTLENIKQTEEALDNHRLPFPLLDAGT
IKMVKVIGRRSLGVQRIFDIGLPQDHNFLLANGAIAAN
>1ROCA
GASIVSLLGIKVLNNPAKFTDPYEFEITFECLESLKHDLEWKLTYVGSSRSLDHDQELDSILVGP
VPVGVNKFVFSADPPSAELIPASELVSVTVILLSCSYDGREFVRVGYYVNNEYDEEELRENPPAK
VQVDHIVRNILAEKPRVTRFNIVWD
>1U8VA
MLMTAEQYIESLRKLNTRVYMFGEKIENWVDHPMIRPSINCVRMTYELAQDPQYADLMTTKSNLI
GKTINRFANLHQSTDDLRKKVKMQRLLGQKTASCFQRCVGMDAFNAVFSTTYEIDQKYGTNYHKN
FTEYLKYIQENDLIVDGAMTDPKGDRGLAPSAQKDPDLFLRIVEKREDGIVVRGAKAHQTGSINS
HEHIIMPTIAMTEADKDYAVSFACPSDADGLFMIYGRQSCDTRKMEEGADIDLGNKQFGGQEALV
VFDNVFIPNDRIFLCQEYDFAGMMVERFAGYHRQSYGGCKVGVGDVVIGAAALAADYNGAQKASH
VKDKLIEMTHLNETLYCCGIACSAEGYPTAAGNYQIDLLLANVCKQNITRFPYEIVRLAEDIAGG
LMVTMPSEADFKSETVVGRDGETIGDFCNKFFAAAPTCTTEERMRVLRFLENICLGASAVGYRTE
SMHGAGSPQAQRIMIARQGNINAKKELAKAIAGIK
>3ES4A
GMTMPIFNISDDVDLVPAMPAEGRDGGSYRRQIWQDDVENGTIVAVWMAEPGIYNYAGRDLEETF
VVVEGEALYSQADADPVKIGPGSIVSIAKGVPSRLEILSSFRKLATVIPKP
>1Y28A
MRETHLRSILHTIPDAMIVIDGHGIIQLFSTAAERLFGWSELEAIGQNVNILMPEPDRSRHDSYI
SRYRTTSDPHIIGIGAIVTGKRRDGTTFPMHLSIGEMQSGGEPYFTGFVRDLTEHQQTQARLQEL
Q
>4IJAA
MGSSHHHHHHSSGENLYFQGPMNDNEKRVLREIYNHHNISRTQISKNLEINKATISSILNKLKYK
SLVNEVGEGDSTKSGGRKPILLKVNHLYGYFISLDLTYSSVEVMYNYFDGNVIKHESYDLPDEKV
SSILSIIKKHIDIQEKLDTYNGLLGVSVSIHGVVDNEQHVTYLPFHETEGISIAKKIKEITNVPV
VVENEANLSALYERNFNHNLSYNNLIALSIHKGIGAGLIINNQLYRGANGEAGEIGKTLVSKVSD
NVEIFHKIEDIFSQEALLHNLSNQLNEKMTLSKLIQFYNEKNPVVVEEMEQFINKIAVLIHNLNT
QFNPNAIYINCPLFNEMPEILEAIKNQFKQYSRNEIQIKLTSNVKFATLLGGTLAIIQKVLQIND
IYLDIKA
>3ELFA
MPIATPEVYAEMLGQAKQNSYAFPAINCTSSETVNAAIKGFADAGSDGIIQFSTGGAEFGSGLGV
KDMVTGAVALAEFTHVIAAKYPVNVALHTDHCPKDKLDSYVRPLLAISAQRVSKGGNPLFQSHMW
DGSAVPIDENLAIAQELLKAAAAAKIILEIEIGVVGGEEDGVANEINEKLYTSPEDFEKTIEALG
AGEHGKYLLAATFGNVHGVYKPGNVKLRPDILAQGQQVAAAKLGLPADAKPFDFVFHGGSGSLKS
EIEEALRYGVVKMNVDTDTQYAFTRPIAGHMFTNYDGVLKVDGEVGVKKVYDPRSYLKKAEASMS
QRVVQACNDLHCAGKSLTHHHHHH
>2BL2A
MMDYLITQNGGMVFAVLAMATATIFSGIGSAKGVGMTGEAAAALTTSQPEKFGQALILQLLPGTQ
GLYGFVIAFLIFINLGSDMSVVQGLNFLGASLPIAFTGLFSGIAQGKVAAAGIQILAKKPEHATK
GIIFAAMVETYAILGFVISFLLVLNA
>2V3GA
MASNYNSGLKIGAWVGTQPSESAIKSFQELQGRKLDIVHQFINWSTDFSWVRPYADAVYNNGSIL

MITWEPWEYNTVDIKNGKADAYITRMAQDMKAYGKEIWLRPLHEANGDWYPWAIGYSSRVNTNET
YIAAFRHIVDIFRANGATNVKWVFNVNCDNVGNGTSYLGHYPGDNYVDYTSIDGYNWGTTQSWGS
QWQSFDQVFSRAYQALASINKPIIIAEFASAEIGGNKARWITEAYNSIRTSYNKVIAAVWFHENK
ETDWRINSSPEALAAYREAIGAG
>3B23C
SDQGDVAEPKMHKTAPPFDFEAIPEEYLDDES
>4AO6A
MRGSHHHHHHGSMRHQMSWNGKDERKLSVQERGFSLEVDGRTVPGVYWSPAEGSSDRLVLLGHGG
TTHKKVEYIEQVAKLLVGRGISAMAIDGPGHGERASVQAGREPTDVVGLDAFPRMWHEGGGTAAV
IADWAAALDFIEAEEGPRPTGWWGLSMGTMMGLPVTASDKRIKVALLGLMGVEGVNGEDLVRLAP
QVTCPVRYLLQWDDELVSLQSGLELFGKLGTKQKTLHVNPGKHSAVPTWEMFAGTVDYLDQRLK
>2DE3A
MTSRVDPANPGSELDSAIRDTLTYSNSPVPNALLTASESGFLDAAGIELDVLSGQQGTVHFTYDQ
PAYTRFGGEIPPLLSEGLRAPGRTRLLGITPLLGRQGFFVRDDSPITAAADLAGRRIGVSASAIR
ILRGQLGDYLELDPWRQTLVALGSWEARALLHTLEHGELGVDDVELVPISSPGVDVPAEQLEESA
TVKGADLFPDVARGQAAVLASGDVDALYSWLPWAGELQATGARPVVDLGLDERNAYASVWTVSSG
LVRQRPGLVQRLVDAAVDAGLWARDHSDAVTSLHAANLGVSTGAVGQGFGADFQQRLVPRLDHDA
LALLERTQQFLLTNNLLQEPVALDQWAAPEFLNNSLNRHR
>1XSZA
GASHPEIEKAQREIIEAFNAKPKNGINKIKEICEQYKISPNEEIAEFFHQQRKNLDLEAVGDYLS
SPEAENQQVLKAFTSQMNFNGQSFVEGLRTFLKTFKLPGEAQKIDRLVQSFSGAYFQQNPDVVSN
ADAAYLLAFQTIMLNTDLHNPSIPEKNKMTVDGLKRNLRGGNNGGDFDAKFLEELYSEIKAKPFE
LNFVKTSPGYELTSTTLNKDSTFKKLDSFLHSTDVNINTVFPGIGDNVKTTVDQPKSWLSFFTGY
KGTITLTDNKTSAQATIQVYTPNIFSKWLFGEQPRVIIQPGQTKESIDLAAKAAADFSSPVKNFK
ATYDYEVGDLIKAYDNQKKLITIERNLALKA
>3O8MA
MVRLGPKKPPARKGSMADVPANLMEQIHGLETLFTVSSEKMRSIVKHFISELDKGLSKKGGNIPM
IPGWVVEYPTGKETGDFLALDLGGTNLRVVLVKLGGNHDFDTTQNKYRLPDHLRTGTSEQLWSFI
AKCLKEFVDEWYPDGVSEPLPLGFTFSYPASQKKINSGVLQRWTKGFDIEGVEGHDVVPMLQEQI
EKLNIPINVVALINDTTGTLVASLYTDPQTKMGIIIGTGVNGAYYDVVSGIEKLEGLLPEDIGPD
SPMAINCEYGSFDNEHLVLPRTKYDVIIDEESPRPGQQAFEKMTSGYYLGEIMRLVLLDLYDSGF
IFKDQDISKLKEAYVMDTSYPSKIEDDPFENLEDTDDLFKTNLNIETTVVERKLIRKLAELVGTR
AARLTVCGVSAICDKRGYKTAHIAADGSVFNRYPGYKEKAAQALKDIYNWDVEKMEDHPIQLVAA
EDGSGVGAAIIACLTQKRLAAGKSVGIKGE
>3LY1A
GETQPESAAFTAPSTDNPIRINFNENPLGMSPKAQAAARDAVVKANRYAKNEILMLGNKLAAHHQ
VEAPSILLTAGSSEGIRAAIEAYASLEAQLVIPELTYGDGEHFAKIAGMKVTKVKMLDNWAFDIE
GLKAAVAAYSGPSIVYLVNPNNPTGTITPADVIEPWIASKPANTMFIVDEAYAEFVNDPRFRSIS
PMITQGAENIILLKTFSKIHAMAGMRVGYAVAHPTVIALMGRYVAGEKINFSGVDAALASMNDSA
FITYSKKSNDVSRQILLKALEDLKLPYLPSEGNFVFHQLVVPLKDYQTHMADAGVLIGRAFPPAD
NWCRISLGTPQEMQWVADTMREFRKKSWI
>3L0FA
MKTPITEAIAAADTQGRFLSNTELQAVDGRFKRAVASMEAARALTNNAQSLIDGAAQAVYQKFPY
TTTMQGSQYASTPEGKAKCARDIGYYLRMVTYCLVAGGTGPMDEYLIAGLSEINSTFDLSPSWYI

EALKYIKANHGLTGQAAVEANAYIDYAINALS
>3M4WA
TPASGALLQQMNLASQSLNYELSFISINKQGVESLRYRHARLDNRPLAQLLQMDGPRREVVQRGN
EISYFEPGLEPFTLNGDYIVDSLPSLIYTDFKRLSPYYDFISVGRTRIADRLCEVIRVVARDGTR
YSYIVWMDTESKLPMRVDLLDRDGETLEQFRVIAFNVNQDISSSMQTLAKANLPPLLSVPVGEKA
KFSWTPTWLPQGFSEVSSSRRPLPTMDNMPIESRLYSDGLFSFSVNVNRATPSSTDQMLRTGRRT
VSTSVRDNAEITIVGELPPQTAKRIAENIKFGAAQ
>4GHKA
MAHHHHHHMGTLEAQTQGPGSMDIDQYMTDVGRRARRASRSIARASTAAKNAALEAVARAIERDA
GALKAANARDVARAKDKGLDAAFVDRLTLSDKALKTMVEGLRQVATLPDPIGEMSNLKYRPSGIQ
VGQMRVPLGVIGIIYESRPNVTIDAAALCLKSGNATILRGGSEALESNTALAKLIGEGLAEAGLP
QDTVQVVETADRAAVGRLITMTEYVDVIVPRGGKSLIERLINEARVPMIKHLDGICHVYVDDRAS
VTKALTVCDNAKTHRYGTCNTMETLLVARGIAPAVLSPLGRLYREKGVELRVDADARAVLEAAGV
GPLVDATDEDWRTEYLAPVLAIKIVDGIDAAIEHINEYGSHHTDAIVTEDHDRAMRFLREVDSAS
VMVNASTRFADGFEFGLGAEIGISNDKLHARGPVGLEGLTSLKYVVLGHGEGRQ
>1LO7A
MARSITMQQRIEFGDCDPAGIVWYPNYHRWLDAASRNYFIKCGLPPWRQTVVERGIVGTPIVSCN
ASFVCTASYDDVLTIETCIKEWRRKSFVQRHSVSRTTPGGDVQLVMRADEIRVFAMNDGERLRAI
EVPADYIELCS
>2FGQX
QSSVTLFGIVDTNVAYVNKDAAGDSRYGLGTSGASTSRLGLRGTEDLGGGLKAGFWLEGEIFGDD
GNASGFNFKRRSTVSLSGNFGEVRLGRDLVPTSQKLTSYDLFSATGIGPFMGFRNWAAGQGADDN
GIRANNLISYYTPNFGGFNAGFGYAFDEKQTIGTADSVGRYIGGYVAYDNGPLSASLGLAQQKTA
VGGLATDRDEITLGASYNFGVAKLSGLLQQTKFKRDIGGDIKTNSYMLGASAPVGGVGEVKLQYA
LYDQKAIDSKAHQITLGYVHNLSKRTALYGNLAFLKNKDASTLGLQAKGVYAGGVQAGESQTGVQ
VGIRHAF
>4HK1A
GDPMFEARLGQATILKKILDAIKDLLNEATFDCSDSGIQLQAMDNSHVSLVSLTLRSDGFDKFRC
DRNLSMGMNLGSMAKILKCANNEDNVTMKAQDNADTVTIMFESANQEKVSDYEMKLMNLDQEHLG
IPETDFSCVVRMPAMEFARICRDLAQFSESVVICCTKEGVKFSASGDVGTANIKLAQTGSVDKEE
EAVIIEMQEPVTLTFACRYLNAFTKATPLSTQVQLSMCADVPLVVEYAIKDLGHIRYYLAPKIED
NET
>3NKZA
SNAMERHQHLLSEYQQILTLSEQMLVLATEGNWDALVDLEMTYLKAVESTANITISSCSSLMLQD
LLREKLRAILDNEIEIKRLLQLRLDRLSDLVGQSTKQQAVNNTYGQFPDHALLLGETQ
>1X91A
GAMDSSEMSTICDKTLNPSFCLKFLNTKFASANLQALAKTTLDSTQARATQTLKKLQSIIDGGVD
PRSKLAYRSCVDEYESAIGNLEEAFEHLASGDGMGMNMKVSAALDGADTCLDDVKRLRSVDSSVV
NNSKTIKNLCGIALVISNMLPRN
>2O4TA
GAHVSRVEKLPKDYQIVYKEIQKYLFKVGPVELNEGIGLLSEILGFFEEGAAAGKGVLDVTGTDV
AAFCDALIGDSKTYADLYQESIQQHVDKAMKNMKD
>4HZOA
SITSSLDVRPEIKQAVTVRPGMCGPGSLFVGQLGDWTWETVSAQCDTDVFAARDASGNPTYLAFY

YFRVRGGRELHPGSLTFGDRLTVTSGCYDQGTESVLTLHRIDRAGSDDAQRPLDLHEFYERPRDG
SLYVENFNRWVTRSAPGSNEDLVKSSPPGFRNDGLPQLPAAYSPRAVYREARTAHTFRALDEPGF
RLLPDTVEVEHPVDIVRDVNGVGLLYFASYFSMVDKAALALWRRLGRSDRAFLRRVVVDQQMCYL
GNADLDSVLTLGARVRVSTETPGEELVDVVISDRDSGRVIAVSTLHTQHDAHDPKGEA
>4H3TA
SNAMEPEMHFDLSSEPWLPVRFRDGRRSEVSLRDIFVLAHTIVGFDVDFPTLEPALLRLVLALAY
RILRGPKDDAEWGRLWEADRFSEDAIDDYFARWRHRFDLFSKEFPFFQVADLEPAGKGGVKTANS
LVAYAPSGNNVPVFTPITDRTELALSPAEAARWLVERHAFGSASDKTGAKGNPKVKGGKDTPAIG
YLAWIGFVAPVGQTLRETLLLNLVPWQYRNLIRGGEDDVPAWERDPLGPTRVMRAPDGVCDLFTW
QGRRIRLFPERRGDAIVVPRVLICAGDEVDRRAARDVDPHVGWRMESRRGAEVSYVPLRARPGQQ
VWRGLSSVLALGAEEQRAGVLSFVEGLQSRGIALVSLLVTSAKFGNMSTTLDDLAYDRLDTPLAV
LNQEDPAAATVAIDAVTFAAHAAQALGYVAEARYLSYDLSFHEESKRHRVPEGKAALAKAARSAL
AEEIYGRLDAPYRHFLTGLANIDDLERPRAEWAALVEAVARDLASRELAQLAPAQAFAGVAGEDR
FRRMLARARNEFSPSDSPEKGAA
>2V6XB
GSHMLQSTPQNLVSNAPIAETAMGIAEPIGAGSEFHGNPDDDLQARLNTLKKQT
>3BY9A
MRFQYQALLNEHQSQLDRFSSHIVATLDKYAHIPHLISKDKELVDALLSAQNSAQIDITNRYLEQ
VNEVIQAADTYLIDRFGNTIASSNWNLDRSFIGRNFAWRPYFYLSIAGQKSQYFALGSTSGQRGY
YYAYPVIYAAEILGVIVVKMDLSAIEQGWQNKSSYFVATDDHQVVFMSSQPAWLFHSVADLSPAQ
LNDIRQSQQYLDSPIPSLGWQGDLQAEQSEWRKPEKHWLQDDYIVSSRPLPELALTIRVLSPKIE
>1BYKA
SDKVVAIIVTRLDSLSENLAVQTMLPAFYEQGYDPIMMESQFSPQLVAEHLGVLKRRNIDGVVLF
GFTGITEEMLAHWQSSLVLLARDAKGFASVCYDDEGAIKILMQRLYDQGHRNISYLGVPHSDVTT
GKRRHEAYLAFCKAHKLHPVAALPGLAMKQGYENVAKVITPETTALLCATDTLALGASKYLQEQR
IDTLQLASVGNTPLMKFLHPEIVTVDPGYAEAGRQAACQLIAQVTGRSEPQQIIIPATLS
>3AFOA
LQSGSKFVKIKPVNNLRSSSSADFVSPPNSKLQSLIWQNPLQNVYITKKPWTPSTREAMVEFITH
LHESYPEVNVIVQPDVAEEISQDFKSPLENDPNRPHILYTGPEQDIVNRTDLLVTLGGDGTILHG
VSMFGNTQVPPVLAFALGTLGFLSPFDFKEHKKVFQEVISSRAKCLHRTRLECHLKKKDSNSSIV
THAMNDIFLHRGNSPHLTNLDIFIDGEFLTRTTADGVALATPTGSTAYSLSAGGSIVSPLVPAIL
MTPICPRSLSFRPLILPHSSHIRIKIGSKLNQKPVNSVVKLSVDGIPQQDLDVGDEIYVINEVGT
IYIDGTQLPTTRKTENDFNNSKKPKRSGIYCVAKTENDWIRGINELLGFNSSFRLTKRQTDND
>3LWXA
GQSLRSFQKQNEDNDKRQQILRSINVNVSSSEAETKYNELIKEAFLVNENGEKVEGDAFATDVVK
AATEHQYPVFVANVDGQPKYIMALHGAGLWGPLWGYISVDSDKNTIYGADFSHQGETPGLGAEIS
KPVFSNEFKGKKIFMSGEFKSVAVVKPGKSVAGQDYVDGISGGTITSKGVDEMLFNSLSGYVKFL
TSQN
>2OB9A
GHMSQTLKQLAMAKMAGFRHKTVVVPEWEGVKVVLREPSGEAWLRWQEVVKGGGDDENVSVSEKA
HRNLCADVVLFIDVLCDTDKQPVFSVDEEEQVREIYGPVHSRLLKQALDLINNADEAREKSQPPA
>3RXYA
SNAMAGLSTAELVDIALEMAEMRTLPADSAVYVESTDLKRVMMGIDIGPAELLLARQLGCDGVIA
HHPAGGSATLNFPEVLTRHVELMVEHGVPATAARDAIQGLLTRSLLRAQSANHDHTPSVARLLEM

PFLNIHLPLDEVGRRIMVKTIQEAVEPLGDEARVQDAIDALMTLPEFAGAATRIMVPVGAVDQPL
GKIAVVHGAGTNGGYAVARAYFDHGVRTVLYIHIAPEEAERLRREGGGNLIVTGHIASDLVGINR
YVQALEERGVEVVRMSGL
>3QZBA
MGSDKIHHHHHHMKLSDFIKTEDFKKEKHVPVIEAPEKVKKDEKVQIVVTVGKEIPHPNTTEHHI
RWIKVFFQPDGDPYVYEVGRYEFNAHGESVQGPNIGAVYTEPTVTTVVKLNRSGTIIALSYCNIH
GLWESSQKITVEE
>2PYXA
GMMQKPITEIIIVGGGTAGWITAGLLAAEHNVDKGVLAHSPKLNITLIESPDVATIGVGEGTWPS
MRSTLSKIGIDENDFIRQCDASFKQGSRFINWCKDPQSNVADSYLHPFSLPHGHQELDLCPYWLP
HAEQVSFAEAVCSQQVLTQLGLAPKSIVTAQYHFQNNYGYHLNAAKFSQLLTEHCTQKLGVTHIR
DHVSQIINNQHGDIEKLITKQNGEISGQLFIDCTGAKSLLLGEHLQVPFLSQKSVLFNDRALAIQ
VPYSDANSPIASCTHSTAQPNGWIWDIGLPTRKGVGYVYSSSHTNDIDAQKTLFNYLGVDGAAAD
KLEPRQLAINPGYRAKCWQNNCIAIGMAAGFIEPLEASALALIEWTASTLAQQLPPNRMVMDTIS
ARVNERYQQHWQQIIDFLKLHYVISQRQEDRYWRDHRESNSIPDSLQAMLELWRYQTPSQQDISY
KEALFPAASFQYVLYGMSFNTQLPTHVKPSMQQLAQRLFNDNQQRTQALSKNLPTNRELLDKVAQ
YGFPKL
>3C8WA
GMDKYLSANSLEGVIDNEFSMPAPRWLNTYPAGPYRFINREFFIIAYETDPDLLQAILPPDMELL
EPVVKFEFIRMPDSTGFGDYTESGQVVPVRYKGEEGGFTISMFLDCHAPIAGGREIWGFPKKLAK
PKLFVEEDTLIGILKYGSIDIAIATMGYKHRPLDAEKVLESVKKPVFLLKNIPNVDGTPLVNQLT
KTYLTDITVKGAWTGPGSLELHPHALAPISNLYIKKIVSVSHFITDLTLPYGKVVADYLA
>2IDBA
MDAMKYNDLRDFLTLLEQQGELKRITLPVDPHLEITEIADRTLRAGGPALLFENPKGYSMPVLCN
LFGTPKRVAMGMGQEDVSALREVGKLLAFLKEPEPPKGFRDLFDKLPQFKQVLNMPTKRLRGAPC
QQKIVSGDDVDLNRIPIMTCWPEDAAPLITWGLTVTRGPHKERQNLGIYRQQLIGKNKLIMRWLS
HRGGALDYQEWCAAHPGERFPVSVALGADPATILGAVTPVPDTLSEYAFAGLLRGTKTEVVKCIS
NDLEVPASAEIVLEGYIEQGETAPEGPYGDHTGYYNEVDSFPVFTVTHITQREDAIYHSTYTGRP
PDEPAVLGVALNEVFVPILQKQFPEIVDFYLPPEGCSYRLAVVTIKKQYAGHAKRVMMGVWSFLR
QFMYTKFVIVCDDDVNARDWNDVIWAITTRMDPARDTVLVENTPIDYLDFASPVSGLGSKMGLDA
TNKWPGETQREWGRPIKKDPDVVAHIDAIWDELAIFNNGKSALEHHHHHH
>3CRJA
SNAMAGPSDRTFSDQTEEIMQATYRALREHGYADLTIQRIADEYGKSTAAVHYYYDTKDDLLAAF
LDYLLERFVDSIHDVETTDPEARLNLLLDELLVKPQENPDLSVALLEMRSQAPYKEAFSDRFRQN
DEYVRYMLKAVINHGIDEGVFTDVDAEHVTRSLLTIIDGARTRAVMLDDTEELETARQTASEYAD
AMLQ
>2A15A
MTQTTQSPALIASQSSWRCVQAHDREGWLALMADDVVIEDPIGKSVTNPDGSGIKGKEAVGAFFD
THIAANRLTVTCEETFPSSSPDEIAHILVLHSEFDGGFTSEVRGVFTYRVNKAGLITNMRGYWNL
DMMTFGNQE
>2X6WA
DPDQFRAIIESPEGAGHVGYQYRRNTGSTMRMVSDVLDERVSLWDFHCDPSGNVIQPGPNVDSRQ
YLQAAIDYVSSNGGGTITIPAGYTWYLGSYGVGGIAGHSGIIQLRSNVNLNIEGRIHLSPFFDLK
PFQVFVGFDNGDPASSGNLENCHIYGHGVVDFGGYEFGASSQLRNGVAFGRSYNCSVTGITFQNG

DVTWAITLGWNGYGSNCYVRKCRFINLVNSSVNADHSTVYVNCPYSGVESCYFSMSSSFARNIAC
SVQLHQHDTFYRGSTVNGYCRGAYVVMHAAEAAGAGSYAYNMQVENNIAVIYGQFVILGSDVTAT
VSGHLNDVIVSGNIVSIGERAAFSAPFGAFIDIGPDNSGASNVQDIQRVLVTGNSFYAPANITDS
AAITLRANLNGCTFIANNFDCRYMVYNAPGTTSPVVQNLVWDKSNVIGGTHANQRAGQNLFDMQF
ASVVNSTIEVQLSCEDLSMFSCILFPASCQLSYSKITVDSAWTKSMSNTAVFEGNQQAGANVYVS
YPATVNLTSYNTQGAVPFFSTDTNYAWVTSAYSLSINENLDFSPPATYTNKANGQLVGVGYNEIG
GVRSVSVRLMLQRQV
>4ECOA
GGEQFTVKDNALTDDAIVPIKLSRTAEYIKDYLALKEIWDALNGKNWSQQGFGTQPGANWNFNKE
LDMWGAQPGVSLNSNGRVTGLSLEGFGASGRVPDAIGQLTELEVLALGSHGEKVNERLFGPKGIS
ANMSDEQKQKMRMHYQKTFVDYDPREDFSDLIKDCINSDPQQKSIKKSSRITLKDTQIGQLSNNI
TFVSKAVMRLTKLRQFYMGNSPFVAENICEAWENENSEYAQQYKTEDLKWDNLKDLTDVEVYNCP
NLTKLPTFLKALPEMQLINVACNRGISGEQLKDDWQALADAPVGEKIQIIYIGYNNLKTFPVETS
LQKMKKLGMLECLYNQLEGKLPAFGSEIKLASLNLAYNQITEIPANFCGFTEQVENLSFAHNKLK
YIPNIFDAKSVSVMSAIDFSYNEIGSVDGKNFDPLDPTPFKGINVSSINLSNNQISKFPKELFST
GSPLSSINLMGNMLTEIPKNSLKDENENFKNTYLLTSIDLRFNKLTKLSDDFRATTLPYLVGIDL
SYNSFSKFPTQPLNSSTLKGFGIRNQRDAQGNRTLREWPEGITLCPSLTQLQIGSNDIRKVNEKI
TPNISVLDIKDNPNISIDLSYVCPYIEAGMYMLFYDKTQDIRGCDALDIKR
>2EI9A
HMDIRPRLRIGQINLGGAEDATRELPSIARDLGLDIVLVQEQYSMVGFLAQCGAHPKAGVYIRNR
VLPCAVLHHLSSTHITVVHIGGWDLYMVSAYFQYSDPIDPYLHRLGNILDRLRGARVVICADTNA
HSPLWHSLPRHYVGRGQEVADRRAKMEDFIGARRLVVHNADGHLPTFSTANGESYVDVTLSTRGV
RVSEWRVTNESSSDHRLIVFGVGGGTTGERDEDEEARSDLRPGEP
>2IDLA
SNAMIQAVFERAEDGELRSAEITGHAESGEYGLDVVCASVSTLAINFINSIEKFAGYEPILELNE
DEGGYLMVEIPKDLPSHQREMTQLFFESFFLGMANLSENYSEFVQTRVITEN
>4GZRA
MTINYQFGDVDAHGAMIRAQAGLLEAEHQAIVRDVLAAGDFWGGAGSVACQEFITQLGRNFQVIY
EQANAHGQKVQAAGNNMAQTDSAVGSSWATHHHHHH
>3QSQA
MGATPMTFGRSIPEPGEQFRVLLTVGPPMAPNTANSQNWVNKTIVPPENQYTVKIGIDLEHYTTM
QGFTPVESVSWYTADFQPSDEPSPIPGLYARVNNTKKADVYGVQQFKSSHTNNRHQITSVFLVRV
TTSFQVINYTSYFIRGAESGSNVSNLKIRDQTYHTPLQFTQGKWYLLTSTVMHDGPTSSGWVWMN
QELTNNIAYRVDPGMMYLITPPPAASQLYFELHTVLPQGGHHHHHH
>4I1FA
SIYNSFYVYCKGPCQRVQPGKLRVQCSTCRQATLTLTQGPSCWDDVLIPNRMSGECQSPHCPGTS
AEFFFKCGAHPTSDKETPVALHLIATNSRNITCITCTDVRSPVLVFQCNSRHVICLDCFHLYCVT
RLNDRQFVHDPQLGYSLPCVAGCPNSLIKELHHFRILGEEQYNRYQQYGAEECVLQMGGVLCPRP
GCGAGLLPEPDQRKVTCEGGNGLGCGFAFCRECKEAYHEGECSAVFEASGTTTQAYRVDERAAEQ
ARWEAASKETIKKTTKPCPRCHVPVEKNGGCMHMKCPQPQCRLEWCWNCGCEWNRVCMGDHWFDV
>1FYEA
MELLLLSNSTLPGKAWLEHALPLIANQLNGRRSAVFIPFAGVTQTWDEYTDKTAEVLAPLGVNVT
GIHRVADPLAAIEKAEIIIVGGGNTFQLLKESRERGLLAPMADRVKRGALYIGWSAGANLACPTI
RTTNDMPIVDPNGFDALDLFPLQINPHFTNALPEGHKGETREQRIRELLVVAPELTVIGLPEGNW

IQVSNGQAVLGGPNTTWVFKAGEEAVALEAGHRF

>2DDXA

LDGVLVPESGILVSVGQDVDSVNDYASALGTIPAGVTNYVGIVNLDGLNSDADAGAGRNNIAELA
NAYPTSALVVGVSMNGEVDAVASGRYNANIDTLLNTLAGYDRPVYLRWAYEVDGPWNGHSPSGIV
TSFQYVHDRIIALGHQAKISLVWQVASYCPTPGGQLDQWWPGSEYVDWVGLSYFAPQDCNWDRVN
EAAQFARSKGKPLFLNESTPQRYQVADLTYSADPAKGTNRQSKTSQQLWDEWFAPYFQFMSDNSD
IVKGFTYINADWDSQWRWAAPYNEGYWGDSRVQANALIKSNWQQEIAKGQYINHSETLFETLGYG
STHHHHHH

>1CQYA

TPVMQTIVVKNVPTTIGDTVYITGNRAELGSWDTKQYPIQLYYDSHSNDWRGNVVLPAERNIEFK
AFIKSKDGTVKSWQTIQQSWNPVPLKTTSHTSSW

>2Q0SA

MAKRILCFGDSLTWGWVPVEDGAPTERFAPDVRWTGVLAQQLGADFEVIEEGLSARTTNIDDPTD
PRLNGASYLPSCLATHLPLDLVIIMLGTNDTKAYFRRTPLDIALGMSVLVTQVLTSAGGVGTTYP
APKVLVVSPPPLAPMPHPWFQLIFEGGEQKTTELARVYSALASFMKVPFFDAGSVISTDGVDGIH
FTEANNRDLGVALAEQVRSLL

>2GZ6A

MGKNLQALAQLYKNALLNDVLPFWENHSLDSEGGYFTCLDRQGKVYDTDKFIWLQNRQVWTFSML
CNQLEKRENWLKIARNGAKFLAQHGRDDEGNWYFALTRGGEPLVQPYNIFSDCFAAMAFSQYALA
SGEEWAKDVAMQAYNNVLRRKDNPKGKYTKTYPGTRPMKALAVPMILANLTLEMEWLLPQETLEN
VLAATVQEVMGDFLDQEQGLMYENVAPDGSHIDCFEGRLINPGHGIEAMWFIMDIARRKNDSKTI
NQAVDVVLNILNFAWDNEYGGLYYFMDAAGHPPQQLEWDQKLWWVHLESLVALAMGYRLTGRDAC
WAWYQKMHDYSWQHFADPEYGEWFGYLNRRGEVLLNLKGGKWKGCFHVPRAMYLCWQQFEALS

>2EX2A

MANVDEYITQLPAGANLALMVQKVGASAPAIDYHSQQMALPASTQKVITALAALIQLGPDFRFTT
TLETKGNVENGVLKGDLVARFGADPTLKRQDIRNMVATLKKSGVNQIDGNVLIDTSIFASHDKAP
GWPWNDMTQCFSAPPAAAIVDRNCFSVSLYSAPKPGDMAFIRVASYYPVTMFSQVRTLPRGSAEA
QYCELDVVPGDLNRFTLTGCLPQRSEPLPLAFAVQDGASYAGAILKYELKQAGITWSGTLLRQTQ
VNEPGTVVASKQSAPLHDLLKIMLKKSDNMIADTVFRMIGHARFNVPGTWRAGSDAVRQILRQQA
GVDIGNTIIADGSGLSRHNLIAPATMMQVLQYIAQHDNELNFISMLPLAGYDGSLQYRAGLHQAG
VDGKVSAKTGSLQGVYNLAGFITTASGQRMAFVQYLSGYAVEPADQRNRRIPLVRFESRLYKDIY
QNN

>3GNZP

AVINHDAVPVWPQPEPADATQALAVRFKPQLDVVNGCQPYPAVDPQGNTSGGLKPSGSQAAACRD
MSKAQVYSRSGTYNGYYAIMYSWYMPKDSPSTGIGHRHDWENVVVWLDNAASANIVALSASAHSG
YKKSFPADKSYLDGITAKISYKSTWPLDHELGFTTSAGKQQPLIQWEQMTQAARDALESTDFGNA
NVPFKSNFQDKLVKAFFQ

>3LEWA

GLDTIPTTYVDAGSVFGKTGDAEKVLNGGWNYLMETFNSYANPGYGAMLRANDAMGSDVVLNSKY
GFRTHNEFSAIYGKGGTNTLSWLLAYRVINDCNGVLDNIDAAEGTQADRNRIKGQALALRGFLYL
HLASCYSFAIDKDPDAVCAPIYTQSTDETIAAEGKPASSVSEVYAQSINDLEEALELIPETYVRD
AKHKIDNEVVLGILSRACLYARQWEKAKTYSDKLLAKDNYLMTESEYKAGFNSVDNKEWIWGHAQ
TNDQSNASYQFHYLDTTTKGSYYYSFNVDPYFRDLFEDGDYRKEMLFWATDPGADVESAAYVWMR
NSKFRFRDIENQLGDIVLMRVAEIYLINAEAKAHLNDPDAINKLNDLKTARGAKTIHTNLSQQDL

LETIWLERRKELWGEGFSLIDIIRNQQTVVRNAYPEGPIDYIYTDENGQTHTLKKKTQGHRFFNF
PDKSAFCPNSKYYLYRITDSEELANKNLYKDHPKLSIYTK
>3KG9A
SNATAKNLHPLLGEKLNLARIENQHHFQSYLTAESPAYLSQHQVFNKVLFPATGYLEIAAAVGKN
LLTTGEQVVVSDVTIVRGLVIPETDIKTVQTVISTLENNSYKLEIFSTSEGDNQQANQWTLHAEG
KIFLDSTTNTKAKIDLEQYQRECSQVIDIQQHYQQFKSRGIDYGNSFQGIKQLWKGQGKALGKIA
LPEEIAGQATDYQLHPALLDAALQILGHAIGNTETDDKAYLPVGIDKLKQYRQTITQVWAIVEIP
ENTLKGSIKLVDNQGSLLAEIEGLRVTATTADALLK
>1TUWA
MAYRALMVLRMDPADAEHVAAAFAEHDTTELPLEIGVRRRVLFRFHDLYMHLIEADDDIMERLYQ
ARSHPLFQEVNERVGQYLTPYAQDWEELKDSKAEVFYSWTAPDS
>1P1XA
HMTDLKASSLRALKLMDLTTLNDDDTDEKVIALCHQAKTPVGNTAAICIYPRFIPIARKTLKEQG
TPEIRIATVTNFPHGNDDIDIALAETRAAIAYGADEVDVVFPYRALMAGNEQVGFDLVKACKEAC
AAANVLLKVIIETGELKDEALIRKASEISIKAGADFIKTSTGKVAVNATPESARIMMEVIRDMGV
EKTVGFKPAGGVRTAEDAQKYLAIADELFGADWADARHYRFGASSLLASLLKALGHGDGKSASSY
>4EAEA
SNAEAFSFSPSGFKVSTVESILGGDVTTTYLSSSKSFQKDFEALTLFINQYKVEHVINPTKEVSA
SNPESYLANKNGYVITLDISIKNNSKKDKMYKADQISLLGASKSVGGSLDNFIPSGFHLIGSSSD
PYNFTAGKTARGLLTFTMDEATYNDLAKDSQIGVPDPSRFDSSSTKGSSQDNVVAPFPIK
>3GR5A
GSHMSSLEKRLGKNEYFIITKSSPVRAILNDFAANYSIPVFISSSVNDDFSGEIKNEKPVKVLEK
LSKLYHLTWYYDENILYIYKTNEISRSIITPTYLDIDSLLKYLSDTISVNKNSCNVRKITTFNSI
EVRGVPECIKYITSLSESLDKEAQSK
>3SUMA
SNWLIKWDDKFQNDTLSISEFKCSAALAKLGPDPKHPPTKLGEVLNFPHFVAAPEAQTECGSCWK
LRYKGNHAFVTVVDRVEEANLFVGGTDLVKNLTTFNGAPEGYDWGTAQLFSAYQVDGSCCQQNTG
KQCGDP
>2QP2A
SMSNDKTGKSLEQENSERDVEIRDRNYFRKLSLFDDTVIAGAEMIGTSYDVFGKYCNVGSCMNSL
FDERKINASEDNFKKVTILGKTLKVPYYIDCYSVGDLKYTNASGESIESYQSNISSKSRIKGNYL
FFSASLKVDFDTDSLTDFENAFSRIQYTYDLYILKSSAEALKEFLKESVKTALDKADTEEDMNDL
FNTWGSHFLSGVVMGGCAQYSSSTNKYTSNLTNSFDVVAAASFAGFIGLSARTGNSFMEDIKKFR
SASNIKTHAIGGDLSRFDPFGGATSADQPSAEEIAAAKKAFEDWKASVPNAPELVNFADSNPLTG
IWELCSDRTQKAKLKKHFETVWAPAESAKRRVHADYIDEIIIGINNTNTPPEGYIGLKSTKDENL
NSKGNICLFMHKAKYDPNIDNKDCITELKFITVRDKSPEGDWVKIPQDIYISPNQYLYLCYLPAK
YSAEKAIKDIQLLCSSCGSSMILPYGYNDVLDERGERANATEDDNVHYLIYSAGWK
>2I2LA
MNTAYRVWDGEQMHYWDDEGLSLIIKSNGDWTLKRLYTDVLVPVVDSTNRNAALMWGAKVRGKFI
YDRSIVKITSDDKESSDVCEVKFSDGVFQVDVSKISADYDVTAVGWVEYATIEVIGDVYQNPELL
EGVKLEHHHHHH
>1PG6A
LEHHHHHHMVDNRMRFTIDQNMQFPLVEIDLEHGGSVYLQQGSMVYHTENVTLNTKLNGKGSGLGK
LVGAIGRSMVSGESMFITQAMSNGDGKLALAPNTPGQIVALELGEKQYRLNDGAFLALDGSAQYK

MERQNIGKALFGGQGGLFVMTTEGLGTLLANSFGSIKKITLDGGTMTIDNAHVVAWSRELDYDIH
LENGFMQSIGTGEGVVNTFRGHGEIYIQSLNLEQFAGTLKRYLPTSSN
>1ZATA
KEQLASMNAIANVKATYSINGETFQIPSSDIMSWLTYNDGKVDLDTEQVRQYVTDLGTKYNTSTN
DTKFKSTKRGEVTVPVGTYSWTIQTDSETEALKKAILAGQDFTRSPIVQGGTTADHPLIEDTYIE
VDLENQHMWYYKDGKVALETDIVSGKPTTPTPAGVFYVWNKEEDATLKGTNDDGTPYESPVNYWM
PIDWTGVGIHDSDWQPEYGGDLWKTRGSHGCINTPPSVMKELFGMVEKGTPVLVF
>2EPLX
MATFLGLSSKQEKALVRLDKYLNLGEIAVSLVTDSATSIKVEGRQGYYQVSYKQPHQLYRALALL
SAALRSGQDEVQIEEEAAYEDLAYMADCSRNAVLNLSSAKKMIEVLALMGYSTFELYMEDTYEIE
NQPYFGYFRGRYTVAELQEIEDYAADFDMSFVPCIQTLAHLSAFVKWGIKEVQELRDVEDILLIG
EEKVYDLIEGMFQTMAHLHTRKINIGMDEAHLVGLGRYLIKHGFQNRSLLMCQHLERVLDIADKY
GFNCQMWSDMFFKLMSADGQYDRDVEIPEETRVYLDRLKERVTLVYWDYYQDSEEKYNRNFQNHH
KISQDIAFAGGAWKWIGFTPHNHFSRLVAIEANKACRKNQVKEVIVTGWGDNGGETSQFSVLPAL
QIWAELAYRNDLKKVSEHFLVSTGLDFDDFMKIDLANLLPDLPDNLSGINPNRYVLYQDVLCPLL
EQHIRPEKDKQHFASSAQQLGEISKRAGEYAYIFETQAQLNALLALKISITSGIQKAYRNGDKEH
LSALAEKDFPQLYQMVEDFSDQFSRQWQQENKIFGLDTIDIRFGGLLKRIKRAQERLEQFISGQI
DCVEELEQEILPFNDFYKDQGLTATTANQWHLIATASTIYTT
>1L3PA
IPAGELQIIDKIDAAFKVAATAAATAPADDKFTVFEAAFNKAIKETTGGAYDTYKCIPSLEAAVK
QAYAATVAAAPQVKYAVFEAALTKAITAMSEVQKVSQ
>1YHTA
NCCVKGNSIYPQKTSTKQTGLMLDIARHFYSPEVIKSFIDTISLSGGNFLHLHFSDHENYAIESH
LLNQRAENAVQGKDGIYINPYTGKPFLSYRQLDDIKAYAKAKGIELIPELDSPNHMTAIFKLVQK
DRGVKYLQGLKSRQVDDEIDITNADSITFMQSLMSEVIDIFGDTSQHFHIGGDEFGYSVESNHEF
ITYANKLSYFLEKKGLKTRMWNDGLIKNTFEQINPNIEITYWSYDGDTQDKNEAAERRDMRVSLP
ELLAKGFTVLNYNSYYLYIVPKASPTFSQDAAFAAKDVIKNWDLGVWDGRNTKNRVQNTHEIAGA
ALSIWGEDAKALKDETIQKNTKSLLEAVIHKTNGDEHHHHHH
>4BC3A
GAMAEHAPRRCCLGWDFSTQQVKVVAVDAELNVFYEESVHFDRDLPEFGTQGGVHVHKDGLTVTS
PVLMWVQALDIILEKMKASGFDFSQVLALSGAGQQHGSIYWKAGAQQALTSLSPDLRLHQQLQDC
FSISDCPVWMDSSTTAQCRQLEAAVGGAQALSCLTGSRAYERFTGNQIAKIYQQNPEAYSHTERI
SLVSSFAASLFLGSYSPIDYSDGSGMNLLQIQDKVWSQACLGACAPHLEEKLSPPVPSCSVVGAI
SSYYVQRYGFPPGCKVVAFTGDNPASLAGMRLEEGDIAVSLGTSDTLFLWLQEPMPALEGHIFCN
PVDSQHYMALLCFKNGSLMREKIRNESVSRSWSDFSKALQSTEMGNGGNLGFYFDVMEITPEIIG
RHRFNTENHKVAAFPGDVEVRALIEGQFMAKRIHAEGLGYRVMSKTKILATGGASHNREILQVLA
DVFDAPVYVIDTANSACVGSAYRAFHGLAGGTDVPFSEVVKLAPNPRLAATPSPGASQVYEALLP
QYAKLEQRILSQTRGPPE
>2FI1A
MKGMKYHDYIWDLGGTLLDNYETSTAAFVETLALYGITQDHDSVYQALKVSTPFAIETFAPNLEN
FLEKYKENEARELEHPILFEGVSDLLEDISNQGGRHFLVSHRNDQVLEILEKTSIAAYFTEVVTS
SSGFKRKPNPESMLYLREKYQISSGLVIGDRPIDIEAGQAAGLDTHLFTSIVNLRQVLDI
>2HJEA
GSKQQTSALIHNIFDSHFAAIQIHHDSNSKSEVIRDFYTDRDTDVLNFFFLSIDQSDPSHTPEFR

FLTDHKGIIWDDGNAHFYGVNDLILDSLANRVSFSNNWYYINVMTSIGSRHMLVRRVPILDPSTG
EVLGFSFNAVVLDNNFALMEKLKSESNVDNVVLVANSVPLANSLIGDEPYNVADVLQRKSSDKRL
DKLLVIETPIVVNAVTTELCLLTVQD
>3MW6A
SNAMTQETALGAALKSAVQTMSKKKQTEMIADHIYGKYDVFKRFKPLALGIDQDLIAALPQYDAA
LIARVLANHCRRPRYLKALARGGKRFDLNNRFKGEVTPEEQAIAQNHPFVQQALQQQSAQAAAET
LSVEAEAAESSAAE
>2XEDA
MGSSHHHHHHSSGLEVLFQGPAMGIRRIGLVVPSSNVTVETEMPALLSRHPGAEFSFHSTRMRMH
TVSPEGLAAMNAQRERCVLEIADAAPEVILYACLVAVMVGGPGEHHRVESAVAEQLATGGSQALV
RSSAGALVEGLRALDAQRVALVTPYMRPLAEKVVAYLEAEGFTISDWRALEVADNTEVGCIPGEQ
VMAAARSLDLSEVDALVISCAVQMPSLPLVETAEREFGIPVLSAATAGAYSILRSLDLPVAVPGA
GRLLRQDSAVTAS
>1G8KA
GCPNDRITLPPANAQRTNMTCHFCIVGCGYHVYKWPELEEGGRAPEQNALGLDFRKQLPPLAVTL
TPAMTNVVTEHDGARYDIMVVPDKACVVNSGLSSTRGGKMASYMYTPTGDGKERLSAPRLYAADE
WVDTTWDHAMALYAGLIKKTLDKDGPQGVFFSCFDHGGAGGGFENTWGTGKLMFSAIQTPMVRIH
NRPAYNSECHATREMGIGELNNAYEDAQLADVIWSIGNNPYESQTNYFLNHWLPNLQGATTSKKK
ERFPNENFPQARIIFVDPRETPSVAIARHVAGNDRVLHLAIEPGTDTALFNGLFTYVVEQGWIDK
PFIEAHTKGFDDAVKTNRLSLDECSNITGVPVDMLKRAAEWSYKPKASGQAPRTMHAYEKGIIWG
NDNYVIQSALLDLVIATHNVGRRGTGCVRMGGHQEGYTRPPYPGDKKIYIDQELIKGKGRIMTWW
GCNNFQTSNNAQALREAILQRSAIVKQAMQKARGATTEEMVDVIYEATQNGGLFVTSINLYPTKL
AEAAHLMLPAAHPGEMNLTSMNGERRIRLSEKFMDPPGTAMADCLIAARIANALRDMYQKDGKAE
MAAQFEGFDWKTEEDAFNDGFRRAGQPGAPAIDSQGGSTGHLVTYDRLRKSGNNGVQLPVVSWDE
SKGLVGTEMLYTEGKFDTDDGKAHFKPAPWNGLPATVQQQKDKYRFWLNNGRNNEVWQTAYHDQY
NSLMQERYPMAYIEMNPDDCKQLDVTGGDIVEVYNDFGSTFAMVYPVAEIKRGQTFMLFGYVNGI
QGDVTTDWTDRDIIPYYKGTWGDIRKVGSMSEFKRTVSFKSRRFG
>1TOAA
SYYHHHHHHDYDIPTTENLYFQGAMGSFGSKDAAADGKPLVVTTIGMIADAVKNIAQGDVHLKGL
MGPGVDPHLYTATAGDVEWLGNADLILYNGLHLETKMGEVFSKLRGSRLVVAVSETIPVSQRLSL
EEAEFDPHVWFDVKLWSYSVKAVYESLCKLLPGKTREFTQRYQAYQQQLDKLDAYVRRKAQSLPA
ERRVLVTAHDAFGYFSRAYGFEVKGLQGVSTASEASAHDMQELAAFIAQRKLPAIFIESSIPHKN
VEALRDAVQARGHVVQIGGELFSDAMGDAGTSEGTYVGMVTHNIDTIVAALAR
>3EOIA
SLSRTVHHQQTAEITQQAADFIRYMNAINDYLYQHPERRAAGGQLTSAQLGLPATKNVSHLISQQ
RVFVWAKEKPGLMGALLEQSGDSALLARVENGRLLDTHGRRISITLPAVIPDQVIIWMN
>1XQRA
GSSHHHHHHSSGLVPRGSHMRGQRGEVEQMKSCLRVLSQPMPPTAGEAEQAADQQEREGALELLA
DLCENMDNAADFCQLSGMHLLVGRYLEAGAAGLRWRAAQLIGTCSQNVAAIQEQVLGLGALRKLL
RLLDRDACDTVRVKALFAISCLVREQEAGLLQFLRLDGFSVLMRAMQQQVQKLKVKSAFLLQNLL
VGHPEHKGTLCSMGMVQQLVALVRTEHSPFHEHVLGALCSLVTDFPQGVRECREPELGLEELLRH
RCQLLQQHEEYQEELEFCEKLLQTCFSSPADDSMDR
>2GTRA
AYRYRDIVVRKQDGFTHILLSTKSSENNSLNPEVMREVQSALSTAAADDSKLVLLSAVGSVFCCG

LDFIYFIRRLTDDRKRESTKMAEAIRNFVNTFIQFKKPIIVAVNGPAIGLGASILPLCDVVWANE
KAWFQTPYTTFGQSPDGCSTVMFPKIMGGASANEMLLSGRKLTAQEACGKGLVSQVFWPGTFTQE
VMVRIKELASCNPVVLEESKALVRCNMKMELEQANERECEVLKKIWGSAQGMDSMLKYLQRKIDE
F
>3GF8A
GASCDSFNEDLPECRLSVKFKYDYNMEFADAFHAQVDKVELYVFDKNGKYLFKQAEEGSALSTGN
YLMEVELPVGQYQFMAWAGARDSYDITSLTPGVSTLTDLKLKLKREASLIINKRMETLWYGEVIN
VNFDGTVHQTETINLIRDTKIVRFGFQSYTGSWTLDMNDYDYEIIESNGHLGHDNSLLDDDVLSF
RPYYMEQKDPATAYVDMNTMRLMEDRKTRLVLTEKASGKRVFDINLIDYLAMTNAEGKNLSTQEY
LDRQSNYHIIFFLSESWLAVQIVVNGWVHRIQEENQ
>1BCPB
STPGIVIPPQEQITQHGSPYGRCANKTRALTVAELRGSGDLQEYLRHVTRGWSIFALYDGTYLGG
EYGGVIKDGTPGGAFDLKTTFCIMTTRNTGQPATDHYYSNVTATRLLSSTNSRLCAVFVRSGQPV
IGACTSPYDGKYWSMYSRLRKMLYLIYVAGISVRVHVSKEEQYYDYEDATFETYALTGISICNPG
SSLC
>3OTNA
GQPTGTMTTDSKLTSKESALALTNSAYLKNTVFNKMTPGWGCNTILLLEYMTGKATSENSQSNYK
DFQDLLVSDRSLYIEDWWQDCYAGIANCNLALQKLGEFENLDASLVNGYMAEVKFMRALYYFYLV
RIFGDVPKITTVQSELGELQVSRAPVKEIYDEIIIPDLLEAEQSDLAFSDHTGRVSMGAVKALLA
DVYLTYAGYPLQGGKSYYAESAKRSLEVIKSNEYTLFTDYESLRLPSQNNKGEFIYQVQFSLNKR
HNESVRIFLPSRSGISAYDLEYGSLIPTKEFVESFEKGDKRTEEKQYFFTNYKGHPSKFSPGAAE
LEFMDLNGYYIYKFFDQVAVDNTAKSDLNWSVYRYTDVLLMYAEAQVNADGTPNQQSIDIVNQIR
GRAGLAPFKQTNASAFLEEVWDQRYFDLCYENKMWFDMLRTRKIRDDKSGEYVDFIGYKTNWGKV
YTETQLLFPIPLSERQANPNLTQNQGY
>4JGLA
GGAKKNVQDAEGQAEAGGNAPSGYLMPAISANNFCGDFTTMTPDYGYLMPEKGLFLKMHDIRGAY
GINIYTYVMDGDNIQCTPGHFVMIVPRGGDKLEITIKKSSMKNTPSFTFIPTPDCENSAYVATEK
VAGKYYYLCGDAEARYKFEDLFEDERCAEFKNLVDNYGK
>2ZEXA
GAHMVNMVSNPGFEDGLDSWQDWQQDMSAVPEAAHNGALGLKIGGGKAAGGGQDIPLKPNTTYIL
GAWAKFDSKPAGTFDVVVQYHLKDANNTYVQHILNFNETDWTYKQLLFTTPDVFGSTPQLALWKG
DTSKANLYVDDVYLVEV
>3PPMA
MGSSHHHHHHSSGLVPRGSHMASRWTGRQKARGAATRARQKQRASLETMDKAVQRFRLQNPDLDS
EALLTLPLLQLVQKLQSGELSPEAVFFTYLGKAWEVNKGTNCVTSYLTDCETQLSQAPRQGLLYG
VPVSLKECFSYKGHDSTLGLSLNEGMPSESDCVVVQVLKLQGAVPFVHTNVPQSMFSYDCSNPLF
GQTMNPWKSSKSPGGSSGGEGALIGSGGSPLGLGTDIGGSIRFPSAFCGICGLKPTGNRLSKSGL
KGCVYGQTAVQLSLGPMARDVESLALCLKALLCEHLFTLDPTVPPLPFREEVYRSSRPLRVGYYE
TDNYTMPSPAMRRALIETKQRLEAAGHTLIPFLPNNIPYALEVLSTGGLFSDGGRSFLQNFKGDF
VDPCLGDLILILRLPSWFKRLLSLLLKPLFPRLAAFLNNMRPRSAEKLWKLQHEIEMYRQSVIAQ
WKAMNLDVLLTPMLGPALDLNTPGRATGAVSYTMLYNCLDFPAGVVPVTTVTAEDDAQMELYKGY
FGDIWDIILKKAMKNSVGLPVAVQCVALPWQEELCLRFMREVEQLMTPQKQPS
>3EC3A
GPLGSPPSKEILTLKQVQEFLKDGDDVVILGVFQGVGDPGYLQYQDAANTLREDYKFHHTFSTEI

AKFLKVSLGKLVLMQPEKFQSKYEPRMHVMDVQGSTEASAIKDYVVKHALPLVGHRKTSNDAKRY
SKRPLVVVYYSVDFSFDYRTATQFWRNKVLEVAKDFPEYTFAIADEEDYATEVKDLGLSESGGDV
NAAILDESGKKFAMEPEEFDSDALREFVMAFKKGKLKPVIKSQPVPKNNKGAAAS
>2AHDA
MKIGIMSDTHDHLPNIRKAIEIFNDENVETVIHCGDFVSLFVIKEFENLNANIIATYGNNDGERC
KLKEWLKDINEENIIDDFISVEIDDLKFFITHGHHQSVLEMAIKSGLYDVVIYGHTHERVFEEVD
DVLVINPGECCGYLTGIPTIGILDTEKKEYREIVL
>1MN8A
AMGQTVTTPLSLTLGHWKDVERIAHNQSVDVKKRRWVTFCSAEWPTFNVGWPRDGTFNRDLITQV
KIKVFSPGPHGHPDQVPYIVTWEALAFDPPPWVKP
>2ISBA
MGSDKIHHHHHHMVMEYELRTPLVKDQILKLKVGDVVYITGEIFTARDEAHARALEWMEEGKELP
FSFDKGVVYHCGPLVKKNDEWRVVSAGPTTSARMNPFTPKILEKVECMGIIGKGGMSEEVVEAMR
GKAAYFAFTGGAGALAAMSIKKVKGVVWEDLGMPEAVWLLEVERFGPCIVAIDAHGNSLYRR
>3MUQA
SNAAEHVRLATTTSTYHSGLLDYLLPQFEKDTGYKVDVIAAGTGKALKMGENGDVDLVMTHAPKA
EGTFVEKGYGVLPRKLMYNDFVIVGPKADPAKIKDDESVLDVFKEIANKNATFISRGDDSGTHKK
EMGFWAQTKIEPNFGGYRSVGQGMGPTLNMASEMQGYTMSDRGTWLAYQNKLDLEILFQGDEKLF
NPYQVILVNPERYPTINYQGAKAFSDWLVNPRGQELINGFRL
>1ZMAA
SNAMEQFLDNIKDLEVTTVVRAQEALDKKETATFFIGRKTCPYCRKFAGTLSGVVAETKAHIYFI
NSEEPSQLNDLQAFRSRYGIPTVPGFVHITDGQINVRCDSSMSAQEIKDFAGL
>2V8QA
GSMAWHLGIRSQSRPNDIMAEVCRAIKQLDYEWKVVNPYYLRVRRKNPVTSTFSKMSLQLYQVDS
RTYLLDFRSIDDEITEAKSGTATPQRSGSISNYRSCQRSDSDAEAQGKPSEVSLTSSVTSLDSSP
VDVAPRPGSHTIEFFEMCANLIKILAQ
>4A1RA
GHAKSVPSRYSLVFDADRQVNAAAGAQPAPIKIRVLLLRSDAEFMDADFFSLQNDAKSVLGNSLL
DSDQFFLTPGQTGKKLGGQSALDARYIGVIAEYQNLDGKTWRISLPLPEPTETNFYKVWQFSPDE
LEAHIVAGVSGLRPVKKVD
>1R6JA
GAMDPRTITMHKDSTGHVGFIFKNGKITSIVKDSSAARNGLLTEHNICEINGQNVIGLKDSQIAD
ILSTSGTVVTITIMPAF
>1N62C
MIPGSFDYHRPKSIADAVALLTKLGEDARPLAGGHSLIPIMKTRLATPEHLVDLRDIGDLVGIRE
EGTDVVIGAMTTQHALIGSDFLAAKLPIIRETSLLIADPQIRYMGTIGGNAANGDPGNDMPALMQ
CLGAAYELTGPEGARIVAARDYYQGAYFTAIEPGELLTAIRIPVPPTGHGYAYEKLKRKIGDYAT
AAAAVVLTMSGGKCVTASIGLTNVANTPLWAEEAGKVLVGTALDKPALDKAVALAEAITAPASDG
RGPAEYRTKMAGVMLRRAVERAKARAKN
>3TOVA
SNAMELDYKRIVVTFLMHLGDVILTTPFLEVLRKAAPHSHITYVIDEKLQQVMEYNPNIDELIVV
DKKGRHNSISGLNEVAREINAKGKTDIVINLHPNERTSYLAWKIHAPITTGMSHFLFRPFMTKYT
RLDRKTRHAADMYINVLEQLGVTDTSNSGLHIEICEEWRCQAQEFYSSHGLTDTDILIGFNIGSA
VPEKRWPAERFAHVADYFGRLGYKTVFFGGPMDLEMVQPVVEQMETKPIVATGKFQLGPLAAAMN

RCNLLITNDSGPMHVGISQGVPIVALYGPSNPFFYGPYQAHAIVLETMDSYEIGKSMKKIIKEGN
YKGLSVISEEQVIKAAETLLLESK
>3ND1A
MGSSHHHHHHSSGLVPRGSHMIELSLIGIGTGNPRHITGQAVDAMNAADLILIPLKGADKSDLAG
LRRQICAAHLTNPATKVIDFALPVRDASNPSYRKGVDDWHDAIAETWLSEITAHVPGLEGRVALL
VWGDPSLYDSTLRIAERLKSRLPLTTKVIPGITAIQALCAAHAIPLNDIGAPVVITTGRQLRDHG
WPAGTETVVAMLDGECSFQSLPPDGLTIFWGACVAMPEEVLIRGPVAEVTDEILQARADLRARHG
WVMDIYLLRRNVPAA
>4I79A
MGSSHHHHHHSSGLVPRGSMEEIYAKFVSQKISKTRWRPLPPGSLQTAETFATGSWDNEENYISL
WSIGDFGNLDSDGGFEGDHQLLCDIRHHGDVMDLQFFDQERIVAASSTGCVTVFLHHPNNQTLSV
NQQWTTAHYHTGPGSPSYSSAPCTGVVCNNPEIVTVGEDGRINLFRADHKEAVRTIDNADSSTLH
AVTFLRTPEILTVNSIGQLKIWDFRQQGNEPSQILSLTGDRVPLHCVDRHPNQQHVVATGGQDGM
LSIWDVRQGTMPVSLLKAHEAEMWEVHFHPSNPEHLFTCSEDGSLWHWDASTDVPEKSSLFHQGG
RSSTFLSHSISNQANVHQSVISSWLSTDPAKDRIEITSLLPSRSLSVNTLDVLGPCLVCGTDAEA
IYVTRHLFS
>1I5PA
MNNVLNSGRTTICDAYNVVAHDPFSFEHKSLDTIQKEWMEWKRTDHSLYVAPVVGTVSSFLLKKV
GSLIGKRILSELWGIIFPSGSTNLMQDILRETEQFLNQRLNTDTLARVNAELIGLQANIREFNQQ
VDNFLNPTQNPVPLSITSSVNTMQQLFLNRLPQFQIQGYQLLLLPLFAQAANMHLSFIRDVILNA
DEWGISAATLRTYRDYLRNYTRDYSNYCINTYQTAFRGLNTRLHDMLEFRTYMFLNVFEYVSIWS
LFKYQSLMVSSGANLYASGSGPQQTQSFTAQNWPFLYSLFQVNSNYILSGISGTRLSITFPNIGG
LPGSTTTHSLNSARVNYSGGVSSGLIGATNLNHNFNCSTVLPPLSTPFVRSWLDSGTDREGVATS
TNWQTESFQTTLSLRCGAFSARGNSNYFPDYFIRNISGVPLVIRNEDLTRPLHYNQIRNIESPSG
TPGGARAYLVSVHNRKNNIYAANENGTMIHLAPEDYTGFTISPIHATQVNNQTRTFISEKFGNQG
DSLRFEQSNTTARYTLRGNGNSYNLYLRVSSIGNSTIRVTINGRVYTVSNVNTTTNNDGVNDNGA
RFSDINIGNIVASDNTNVTLDINVTLNSGTPFDLMNIMFVPTNLPPLY
>2R3SA
GMSTPSPALFFNTVNAYQRSAAIKAAVELNVFTAISQGIESSQSLAQKCQTSERGMRMLCDYLVI
IGFMTKQAEGYRLTSDSAMFLDRQSKFYVGDAIEFLLSPMITNGFNDLTAAVLKGGTAISSEGTL
SPEHPVWVQFAKAMSPMMANPAQLIAQLVNENKIEPLKVLDISASHGLFGIAVAQHNPNAEIFGV
DWASVLEVAKENARIQGVASRYHTIAGSAFEVDYGNDYDLVLLPNFLHHFDVATCEQLLRKIKTA
LAVEGKVIVFDFIPNSDRITPPDAAAFSLVMLATTPNGDAYTFAEYESMFSNAGFSHSQLHSLPT
TQQQVIVAYK
>1OIHA
MSNAALATAPHALELDVHPVAGRIGAEIRGVKLSPDLDAATVEAIQAALVRHKVIFFRGQTHLDD
QSQEGFAKLLGEPVAHPTVPVVDGTRYLLQLDGAQGQRANSWHTDVTFVEAYPKASILRSVVAPA
SGGDTVWANTAAAYQELPEPLRELADKLWAVHSNEYDYASLKPDIDPAKLERHRKVFTSTVYETE
HPVVRVHPISGERALQLGHFVKRIKGYSLADSQHLFAVLQGHVTRLENTVRWRWEAGDVAIWDNR
ATQHYAVDDYGTQPRIVRRVTLAGEVPVGVDGQLSRTTRKG
>3LKBA
MSLGQQQVTLFWSGAITGPTSDAGAPYGAAVEDYCKWANERKLVPGVVFNCVVRDDQYNNANTQR
FFEEAVDRFKIPVFLSYATGANLQLKPLIQELRIPTIPASMHIELIDPPNNDYIFLPTTSYSEQV
VALLEYIAREKKGAKVALVVHPSPFGRAPVEDARKAARELGLQIVDVQEVGSGNLDNTALLKRFE

QAGVEYVVHQNVAGPVANILKDAKRLGLKMRHLGAHYTGGPDLIALAGDAAEGFLWATSFYMAHE
DTPGIRLQKEIGRKYGRPENFIESVNYTNGMLAAAIAVEAIRRAQERFKRITNETVYQAIVGMNG
PNAFKPGFAVSTKQGVEIDFTKSEHTGAEGLRILEAKGGRFVPVTEPFTSALFRKVHYGEGHHHH
HH
>2QSQA
AKLTIESTPFNVAEGKEVLLLVHNLPQHLFGYSWYKGERVDGNRQIIGYVIGTQQATPGPAYSGR
EIIYPNASLLIQNIIQNDAGFYTLHVIKSDLVNEEATGQFRVYPEL
>2QMQA
MGSDKIHHHHHHTHSVETPYGSVTFTVYGTPKPKRPAIFTYHDVGLNYKSCFQPLFRFGDMQEII
QNFVRVHVDAPGMEEGAPVFPLGYQYPSLDQLADMIPCILQYLNFSTIIGVGVGAGAYILSRYAL
NHPDTVEGLVLINIDPNAKGWMDWAAHKLTGLTSSIPDMILGHLFSQEELSGNSELIQKYRGIIQ
HAPNLENIELYWNSYNNRRDLNFERGGETTLKCPVMLVVGDQAPHEDAVVECNSKLDPTQTSFLK
MADSGGQPQLTQPGKLTEAFKYFLQG
>2GJ2A
GSHMATFQTDADFLLVGDDTSRYEEVMKTFDTVEAVRKSDLDDRVYMVCLKQGSTFVLNGGIEEL
RLLTGDSTLEIQPMIVPTTE
>1YT8A
MSLSQIAVRTFHDIRAALLARRELALLDVREEDPFAQAHPLFAANLPLSRLELEIHARVPRRDTP
ITVYDDGEGLAPVAAQRLHDLGYSDVALLDGGLSGWRNAGGELFRDVNVPSKAFGELVEAERHTP
SLAAEEVQALLDARAEAVILDARRFDEYQTMSIPGGISVPGAELVLRVAELAPDPRTRVIVNCAG
RTRSIIGTQSLLNAGIPNPVAALRNGTIGWTLAGQQLEHGQTRRFGAISQDTRKAAAQRARAVAD
RAGVERLDLAGLAQWQDEHDRTTYLLDVRTPEEYEAGHLPGSRSTPGGQLVQETDHVASVRGARL
VLVDDDGVRANMSASWLAQMGWQVAVLDGLSEADFSERGAWSAPLPRQPRADTIDPTTLADWLGE
PGTRVLDFTASANYAKRHIPGAAWVLRSQLKQALERLGTAERYVLTCGSSLLARFAVAEVQALSG
KPVFLLDGGTSAWVAAGLPTEDGESLLASPRIDRYRRPYEGTDNPREAMQGYLDWEFGLVEQLGR
DGTHGFFVIEGGSHHHHHH
>2G84A
GHMNDALHIGLPPFLVQANNEPRVLAAPEARMGYVLELVRANIAADGGPFAAAVFERDSGLLIAA
GTNRVVPGRCSAAHAEILALSLAQAKLDTHDLSADGLPACELVTSAEPCVMCFGAVIWSGVRSLV
CAARSDDVEAIGFDEGPRPENWMGGLEARGITVTTGLLRDAACALLREYNACNGVIYNARCGVHK
GS
>2RGYA
MSLATQQLGIIGLFVPTFFGSYYGTILKQTDLELRAVHRHVVVATGCGESTPREQALEAVRFLIG
RDCDGVVVISHDLHDEDLDELHRMHPKMVFLNRAFDALPDASFCPDHRRGGELAAATLIEHGHRK
LAVISGPFTASDNVERLDGFFDELARHGIARDSVPLIESDFSPEGGYAATCQLLESKAPFTGLFC
ANDTMAVSALARFQQLGISVPGDVSVIGYDDDYSAAYAAPALTSVHIPTAELTQNAVRWLINQCY
GTKWEIFREFPVTVSMRASVAREGHHHHHH
>3SZVA
GHHHHHHHENLYFQGLEDLVEDSHASLELRNFYFNRDFRQSGARDNADEWAQGFLLRLESGFSEG
TVGFGVDAIGLLGFKLDSGSGSGGTGLLPADGSAGGSQDDYAKLGLTAKARVSNSLLKVGALHFK
SPLVSANDTRLLPELFRGALLDVQEIDGLTLRGAHLDRNKLNSSSDYQVFSANRIGGRSDAFDFA
GGDYRLTPALTASLHQGRLKDIYRQTFAGLVHTLDLGGQRSLKSDLRFARASEDGGFRELDNRAF
GALFSLRLGAHAVAAGYQRISGDDPYPYIAGSDPYLVNFIQIGDFGNVDERSWQLRYDYDFGALG
LPGLSFMSRYVSGDNVARGAANDGKEWERNTDLGYVVQSGPLKNLGVKWRNATVRSNFANDLDEN

RLILSYSLALW
>2IUYA
MRPLKVALVNIPLRVPGSDAWISVPPQGYGGIQWVVANLMDGLLELGHEVFLLGAPGSPAGRPGL
TVVPAGEPEEIERWLRTADVDVVHDHSGGVIGPAGLPPGTAFISSHHFTTRPVNPVGCTYSSRAQ
RAHCGGGDDAPVIPIPVDPARYRSAADQVAKEDFLLFMGRVSPHKGALEAAAFAHACGRRLVLAG
PAWEPEYFDEITRRYGSTVEPIGEVGGERRLDLLASAHAVLAMSQAVTGPWGGIWCEPGATVVSE
AAVSGTPVVGTGNGCLAEIVPSVGEVVGYGTDFAPDEARRTLAGLPASDEVRRAAVRLWGHVTIA
ERYVEQYRRLLAGATWK
>3QY3A
MGSSHHHHHHSSGRENLYFQGHMADRQLLHTAHIPVRWGDMDSYGHVNNTLYFQYLEEARVAWFE
TLGIDLEGAAEGPVVLQSLHTYLKPVVHPATVVVELYAGRLGTSSLVLEHRLHTLEDPQGTYGEG
HCKLVWVRHAENRSTPVPDSIRAAIAGS
>2W3YA
MLNEKAIQDVDLYFQNIHGPEGRLASNETFDIVPGLSKDGAVQYQTYQFNEAPKHLQKQVKAGRI
LMERFVAVASAAVNKKAPSNKEKYHYDIWKEVSNQLIPAFFTDPIKGEQNLNTTVKGVEVAKSVI
QFAGNVIAGNVTGFATFLQNFGNGLSAEMNKTQANYNYLYAYSTHDLFQDTSGNVFYKPRFLIYG
THFKQEQKKIATSCASYQEVNLEFGVDTVGGTFRIEEYFSNETFKKKVDNFLDKYEGKAIDDADS
YFDDIFNGVKPNKNYVYHHHHHH
>3EZXA
ANQEIFDKLRDAIVNQNVAGTPELCKEALAAGVPALDIITKGLSVGMKIVGDKFEAAEIFLPQIM
MSGKAMSNAMEVLTPELEKNKKEGEEAGLAITFVAEGDIHDIGHRLVTTMLGANGFQIVDLGVDV
LNENVVEEAAKHKGEKVLLVGSALMTTSMLGQKDLMDRLNEEKLRDSVKCMFGGAPVSDKWIEEI
GADATAENAAEAAKVALEVM
>4JR9A
GSHMSHSSAPERATGAVITDWRPEDPAFWQQRGQRIASRNLWISVPCLLLAFCVWMLFSAVAVNL
PKVGFNFTTDQLFMLTALPSVSGALLRVPYSFMVPIFGGRRWTAFSTGILIIPCVWLGFAVQDTS
TPYSVFIIISLLCGFAGANFASSMANISFFFPKQKQGGALGLNGGLGNMGVSVMQLVAPLVVSLS
IFAVFGSQGVKQPDGTELYLANASWIWVPFLAIFTIAAWFGMNDLATSKASIKEQLPVLKRGHLW
IMSLLYLATFGSFIGFSAGFAMLSKTQFPDVQILQYAFFGPFIGALARSAGGALSDRLGGTRVTL
VNFILMAIFSGLLFLTLPTDGQGGSFMAFFAVFLALFLTAGLGSGSTFQMISVIFRKLTMDRVKA
EGGSDERAMREAATDTAAALGFISAIGAIGGFFIPKAFGSSLALTGSPVGAMKVFLIFYIACVVI
TWAVYGRHSKK
>1LZLA
MTTFPTLDPELAAALTMLPKVDFADLPNARATYDALIGAMLADLSFDGVSLRELSAPGLDGDPEV
KIRFVTPDNTAGPVPVLLWIHGGGFAIGTAESSDPFCVEVARELGFAVANVEYRLAPETTFPGPV
NDCYAALLYIHAHAEELGIDPSRIAVGGQSAGGGLAAGTVLKARDEGVVPVAFQFLEIPELDDRL
ETVSMTNFVDTPLWHRPNAILSWKYYLGESYSGPEDPDVSIYAAPSRATDLTGLPPTYLSTMELD
PLRDEGIEYALRLLQAGVSVELHSFPGTFHGSALVATAAVSERGAAEALTAIRRGLRSLSPVS
>2GAGC
MANNTLIETPLRHSPAEHLDTVMDAASVAGRVELREIAFTTQISLRCAPGTQAHAALAAATGAGL
PAKVGEVAGEAQGTAVLWLAPDEFLATSAENTELGGVLSAALGDAPGQVVDLSANRSVLELTGPD
APLVLRKSCPADLHPRAFAVNQAIVTSVANIPVLLWRTGEQAWRIMPRASFTEHTVHWLVDAMSE
FASEAVILEHHHHHH
>1CFRA

MDIISKSGEGNKYTINSAIAFVAYASHIDINTTEFSKVLSGLRDFINDEAIRLGGKISDGSFNKC
NGDWYEWLIGIRAIEFFLESETNFIVVKMPNATSFDVMSIYKSCLSEFIYDLRSKLSLNNVNLIT
SNPDFSIIDIRGRREELKSMLKDISFSNISLSTISEIDNLYKNFIDYAELEHIKSFLSVKTTFRP
DRRLQLAHEGSLMKALYTHLQTRTWTINPTGIRYYAAATSIGNADVIGLKTVATHSITDVKSLPQ
SAVDEIFKINSVLDVDSCLSHILSS
>3ONQA
SNAMNSEQADILDLLSGHTDDTTIERLAFECLLTNMTDDRVVSLMNILGWQGDFNCFAIGGVPSA
SLASTSLAIRKAVRDLGGEHVVIGTYGTFLLALACQMGAVTPEVTCTAVMPAFSEDEPLYLSPVR
SGVAGASHALRETMFSLQAAPALSTPSRPLRADELLPERALLGDDYAREELYRNVYQVLRGENPD
DPTYLTVSTFLKYGSSLENTAKELNVHPNTVRYRLKRAAETTGWDATDPRDAYVLTTALAIGRMR
DR
>3POPA
GSHMTASVPPFTVGREDPRYIELSHSDNHRFVVEPEEFFLPATPDDVVASLQKAVTEGRGVACRS
GGHCGQDFVGTPRRDLVLDLHNLHAIGPAADGAGVRVGSGATVDQVQKALFRRWNAALPLGACSA
VGMGGLVAGGGYGPLSRQLGLVVDHLHAVEVAVVDESRTVRLVTARADDTGDLGELFWAHTGGGG
GNFGVVTAYEFRSPEHLATEPVGLPRAAGRLHVQKVVFPWAMIDETSFVTVMRRFFEWHERHSEP
GSPESSLFATFFVNHVSSGVLQLMVQQDADVDPEGEILARFVASLTEGTGVVGIPRGGVMSWLTG
TRYMSQADCGDVMGARSASKSAYHRAAPTDEQLSVLHRHLHADHPGQASYVMFNSYGGEINRRGP
SDAAVPQRDSVVKSSWFSAWQDAELDELHLGWLRGLYEEFFAGTGGVPVTGGRTDGCYINYPDAD
LLDPARNRSGEPWHHLYYKDNYARLRSAKRAWDPLNTFHHSMSIGL
>3IGZB
MSALLLKPHKDLPRRTVLIVVMDGLGIGPEDDYDAVHMASTPFMDAHRRDNRHFRCVRAHGTAVG
LPTDADMGNSEVGHNALGAGRVALQGASLVDDAIKSGEIYTGEGYRYLHGAFSKEGSTLHLIGLL
SDGGVHSRDNQIYSIIEHAVKDGAKRIRVHALYDGRDVPDGSSFRFTDELEAVLAKVRQNGCDAA
IASGGGRMFVTMDRYDADWSIVERGWRAQVLGDARHFHSAKEAITTFREEDPKVTDQYYPPFIVV
DEQDKPLGTIEDGDAVLCVNFRGDRVIEMTRAFEDEDFNKFDRVRVPKVRYAGMMRYDGDLGIPN
NFLVPPPKLTRVSEEYLCGSGLNIFACSETQKFGHVTYFWNGNRSGKIDEKHETFKEVPSDRVQF
NEKPRMQSAAITEAAIEALKSGMYNVVRINFPNGDMVGHTGDLKATITGVEAVDESLAKLKDAVD
SVNGVYIVTADHGNSDDMAQRDKKGKPMKDGNGNVLPLTSHTLSPVPVFIGGAGLDPRVAMRTDL
PAAGLANVTATFINLLGFEAPEDYEPSLIYVEKLEHHHHHH
>3GUUA
MRVSLRSITSLLAAATAAVLAAPAAETLDRRAALPNPYDDPFYTTPSNIGTFAKGQVIQSRKVPT
DIGNANNAASFQLQYRTTNTQNEAVADVATVWIPAKPASPPKIFSYQVYEDATALDCAPSYSYLT
GLDQPNKVTAVLDTPIIIGWALQQGYYVVSSDHEGFKAAFIAGYEEGMAILDGIRALKNYQNLPS
DSKVALEGYSGGAHATVWATSLAESYAPELNIVGASHGGTPVSAKDTFTFLNGGPFAGFALAGVS
GLSLAHPDMESFIEARLNAKGQRTLKQIRGRGFCLPQVVLTYPFLNVFSLVNDTNLLNEAPIASI
LKQETVVQAEASYTVSVPKFPRFIWHAIPDEIVPYQPAATYVKEQCAKGANINFSPYPIAEHLTA
EIFGLVPSLWFIKQAFDGTTPKVICGTPIPAIAGITTPSADQVLGSDLANQLRSLDGKQSAFGKP
FGPITPP
>4HWMA
GGCVQVDRYEDVVKAPAPAGLAGFWQTKGPQSAMMSPDAIASLIVTKEGDTFDCRQWQRVIAQPG
KLMNRDSEIYNVTASLDIYPVEREGNTISYDRMTLSRVERLTPECEKAWAKARATGPVSAPASTR
>2VOVA
GLDTLDRDGDGSTADADCNDFAPTIHPGAAEATLDGVDSNCDGRDSGVAEVVETFKNPGTYSSPV

INFKIASPPGPGTPIYGPPRDFSGYNKSYSLAIGKTSYYDPTTGTKWNDDTITPVSDGQDIWRGX
THTGKWSFFNGKAGDKITLSVQRDAQEASLKGAHPGFILFWRPEGGPLFWAGTQDLDEGQTALPA
DSDTVIGHVIVQHADWTLQGLPPKADHTAPAGVDTELYPMKPDSYTMYYVDSGYDADKYVASKKL
IMHPTAFKGLALNDGTAGAFTKSITLPKTGYYMLYVANVLEVDDWSVDADGKLTTTGEVWEVPAK
GCWVNITISKP

>1UKKA

MPVRKAKAVWEGGLRQGKGVMELQSQAFQGPYSYPSRFEEGEGTNPEELIAAAHAGCFSMALAAS
LEREGFPPKRVSTEARVHLEVVDGKPTLTRIELLTEAEVPGISSEKFLEIAEAAKEGCPVSRALA
GVKEVVLTARLV

>3CHHA

MREEQPHLATTWAARGWVEEEGIGSATLGRLVRAWPRRAAVVNKADILDEWADYDTLVPDYPLEI
VPFAEHPLFLAAEPHQRQRVLTGMWIGYNERVIATEQLIAEPAFDLVMHGVFPGSDDPLIRKSVQ
QAIVDESFHTYMHMLAIDRTRELRKISERPPQPELVTYRRLRRVLADMPEQWERDIAVLVWGAVA
ETCINALLALLARDATIQPMHSLITTLHLRDETAHGSIVVEVVRELYARMNEQQRRALVRCLPIA
LEAFAEQDLSALLLELNAAGIRGAEEIVGDLRSTAGGTRLVRDFSGARKMVEQLGLDDAVDFDFP
ERPDWSPHTPR

>2QNGA

MGSSHHHHHHSSGRENLYFQGHMNGLNKGIRKVELAVKWDPSPPGDPATDLDIVAATFLAGDAYG
KPAYVVHFDSRSPDGTIYLNRDSKDGKGFGWDEVMTLELNRLDSRYARVVVGVVIQQRDAHRTFV
GVLNPGLRMREGYTVLAEDDFGGVLGSTAATVGEFVRDDSGEWTFHPGIHGYDSDPATFARVMGG
RQDS

>1ZU0A

RSELTIVPDFYPTMVRNFNPYLATNLRTTTDFIYEPLVVFNEMKGNTPVFRLAESYKMADDLMSV
TFDIRKGVKWSDGEAFTADDVVYSFGLLKAKPELDQRGINKWVTSVEKVDEYKVRFRLSEANSNV
PYEISLIPIVAEHVWKDVKDPTTFTNENPVGTGPFTVIDTFTPQLYIQCRNPNYWDAANLEVDCL
RVPQIANNDQLLGKIVNSELDWTSSFVPDIDRTYAAANPNHHYWYPAAGTQAFMVNFKNPDPAKK
EALDNVDFRRAFSMALDRQTIIDIAFYGSGTVNDFASGLGYAFEAWSDEATHKKYKGFNTYDVEG
SKKLLAKAGFKDVNGDGFVETPSGKSFELLIQSPNGWTDFNNTVQLAVEQLQEVGIKAKARTPEF
AVYNQAMLEGTYDVAYTNYFHGADPFTYWNSGYNSALQSGDGMPRFAMHYFTDKKLDGLLDSFYK
TADKNEQLAIAHGIQKIIAENQVTIPVMSGAWMYQYNTTRFTGWWSEENPKGRPSVWAGIPERLL
HVLDLKPVK

>3BW6A

GAMAMRIYYIGVFRSGGEKALELSEVKDLSQFGFFERSSVGQFMTFFAETVASRTGAGQRQSIEE
GNYIGHVYARSEGICGVLITDKEYPVRPAYTLLNKILDEYLVAHPKEEWADVTETNDALKMKQLD
TYISKYQDPSQADA

>2Q01A

MSLARPLSFHEDRLFPSDPATRSYARGLYALVKDLPIISPHGHTDPSWFATNAPFQDATDLLLAP
DHYLFRMLYSQGVSLDALKVRSKAGVPDTDPREAWRVFASHFYLFRGTPSWVWLNHVFSQVFGFT
EFLEASNADDYFDRITAALATDAFRPRALFDRFNIETLATTEGPHESLQHHAAIRESGWGGHVIT
AYRPDAVIDFEDERSPRAFERFAETSGQDVYSWKSYLEAHRLRRQAFIDAGATSSDHGHPTAATA
DLSDVEAEALFNSLVKGDVTPEKAELFRAQMLTEMAKMSLDDGLVMQIHPGSHRNHNVGLLNSHG
RDKGADIPMRTEYVDALKPLLTRLGNDPRLSIILFTLDETTYSRELAPLAGHYPVLKLGPSWWFH
DSPEGMMRFREQVTETAGFYNTVGFNDDTRAFLSIPARHDVARRVDSAFLARMVAEHRMDLVEAE
ELIVDLTYNLPKKAYKLDQRPDWARPATLRAAAEEGHHHHHH

>3IGQA
AQDMVSPPPPIADEPLTVNTGIYLIECYSLDDKAETFKVNAFLSLSWKDRRLAFDPVRSGVRVKT
YEPEAIWIPEIRFVNVENARDADVVDISVSPDGTVQYLERFSARVLSPLDGRRTESDSQTLHIYL
IVRSVDTRNIVLAVDLEKVGKNDDVFLTGWDIESFTAVVKPANFALEDRLESKLDYQLRISRQGG
HHHHHH
>1F0XA
MSSMTTTDNKAFLNELARLVGSSHLLTDPAKTARYRKGFRSGQGDALAVVFPGSLLELWRVLKAC
VTADKIILMQAANTGLTEGSTPNGNDYDRDVVIISTLRLDKLHVLGKGEQVLAYPGTTLYSLEKA
LKPLGREPHSVIGSSCIGASVIGGICNNSGGSLVQRGPAYTEMSLFARINEDGKLTLVNHLGIDL
GETPEQILSKLDDDRIKDDDVRHDGRHAHDYDYVHRVRDIEADTPARYNADPDRLFESSGCAGKL
AVFAVRLDTFEAEKNQQVFYIGTNQPEVLTEIRRHILANFENLPVAGEYMHRDIYDIAEKYGKDT
FLMIDKLGTDKMPFFFNLKGRTDAMLEKVKFFRPHFTDRAMQKFGHLFPSHLPPRMKNWRDKYEH
HLLLKMAGDGVGEAKSWLVDYFKQAEGDFFVCTPEEGSKAFLHRFAAAGAAIRYQAVHSDEVEDI
LALDIALRRNDTEWYEHLPPEIDSQLVHKLYYGHFMCYVFHQDYIVKKGVDVHALKEQMLELLQQ
RGAQYPAEHNVGHLYKAPETLQKFYRENDPTNSMNPGIGKTSKRKNWQEVE
>3IT4B
TMLCVLTTDAAAEPAALERALRRAAAATFDRLDIDGSCSTNDTVLLLSSGASEIPPAQADLDEAV
LRVCDDLCAQLQADAEGVTKRVTVTVTGAATEDDALVAARQIARDSLVKTALFGSDPNWGRVLAA
VGMAPITLDPDRISVSFNGAAVCVHGVGAPGAREVDLSDADIDITVDLGVGDGQARIRTTDLSHA
YVEENSAYSS
>2CLPA
MHHHHHHSSGVDLGTENLYFQSMARPATVLGAMEMGRRMDAPTSAAVTRAFLERGHTEIDTAFVY
SEGQSETILGGLGLRLGGSDCRVKIDTKAIPLFGNSLKPDSLRFQLETSLKRLQCPRVDLFYLHM
PDHSTPVEETLRACHQLHQEGKFVELGLSNYAAWEVAEICTLCKSNGWILPTVYQGMYNAITRQV
ETELFPCLRHFGLRFYAFNPLAGGLLTGKYKYEDKDGKQPVGRFFGNTWAEMYRNRYWKEHHFEG
IALVEKALQAAYGASAPSMTSATLRWMYHHSQLQGAHGDAVILGMSSLEQLEQNLAAAEEGPLEP
AVVDAFNQAWHLVAHECPNYFR
>2WC1A
MAKIGLFFGSDTGTTRKIAKQIKDMFDDEVMAKPLNVNRADVADFMAYDFLILGTPTLGDGQLPG
LSANAASESWEEFLPRIADQDFSGKTIALFGLGDQVTYPLEFVNALFFLHEFFSDRGANVVGRWP
AKGYGFEDSLAVVEGEFLGLALDQDNQAALTPERLKGWLSLIAADFGLVLPA
>3JYZA
GIDPFTVRTRVSEGLVLAEPAKLMISTDGSASTADLTRATTTWNQQSNNLGASSKYVTSVLMDAG
NTGVITITYVADQVGLPTAGNTLILSPYINDGNTRTALATAVAAGTRGTIDWACTSASNATATAQ
GFTGMAAGSVPQEFAPAQCR
>2WDCA
MASWSHPQFEKGALEDPRSLYDLPPYGDATLLYFSDLHGQAFPHYFMEPPNLIAPKPLMGRPGYL
TGEAILRYYGVERGTPLAYLLSYVDFVELARTFGPIGGMGALTALIRDQKARVEAEGGKALVLDG
GDTWTNSGLSLLTRGEAVVRWQNLVGVDHMVSHWEWTLGRERVEELLGLFRGEFLSYNIVDDLFG
DPLFPAYRIHRVGPYALAVVGASYPYVKVSHPESFTEGLSFALDERRLQEAVDKARAEGANAVVL
LSHNGMQLDAALAERIRGIDLILSGHTHDLTPRPWRVGKTWIVAGSAAGKALMRVDLKLWKGGIA
NLRVRVLPVLAEHLPKAEDVEAFLKAQLAPHQDHLFTPLAVSETLLYKRDTLYSTWDQLVGEAVK
AIYPEVEVVFSPAVRWGTTILPGQAITWDHLYAYTGFTYPELYLFYLRGAQIKAVLEDIASNVFT
SDPFYQQGGDVSRVFGLRYVLDPDAPTGERVREVEVGGRPLDPNRRYLAAAYGGRLQRVGEAKPG

YEPRPIYEVLAEYLRSVGRVRVRPEPNVKVIGRNYRLPEVTG
>3SXWA
MMMYKEPFGVKVDFETGIIEGAKKSVRRLSDMEGYFVDERAWKELVEKEDPVVYEVYAVEQEEKE
GDLNFSTHVIYPGKVGKEFFFAKGHFHAKLDRAQVAVALKGKGGVLMQTPEGDAKWISSEPGTVI
YSPPYWAARAVNIGDEPFIMLVIYPADAGHDYGTIAEKGFSKIVIEENGEVKVVDNPRWKKGSLE
HHHHHH
>2HCFA
GMSRTLVLFDIDGTLLKVESMNRRVLADALIEVYGTEGSTGSHDFSGKMDGAIIYEVLSNVGLER
AEIADKFDKAKETYIALFRERARREDITLLEGVRELLDALSSRSDVLLGLLTGNFEASGRHKLKL
PGIDHYFPFGAFADDALDRNELPHIALERARRMTGANYSPSQIVIIGDTEHDIRCARELDARSIA
VATGNFTMEELARHKPGTLFKNFAETDEVLASILTPKHS
>4BF3A
GPLGSKNHTLYDGQSNGEAKVKKIEFSEFTVKIKNKNNSNNWADLGDLVVRKEEDGIETGLNVGK
GDSDTFAGYTATFFSLEESEVNNFIKAMTEGGSFKTSLYYGYKDEQSNANGIQNKEIITKIEKID
DFEYITFLGDKIKDSGDKVVEYAILLEDLKKNLK
>4HATC
GAMEGILDFSNDLDIALLDQVVSTFYQGSGVQQKQAQEILTKFQDNPDAWQKADQILQFSTNPQS
KFIALSILDKLITRKWKLLPNDHRIGIRNFVVGMIISMCQDDEVFKTQKNLINKSDLTLVQILKQ
EWPQNWPEFIPELIGSSSSSVNVCENNMIVLKLLSEEVFDFSAEQMTQAKALHLKNSMSKEFEQI
FKLCFQVLEQGASSSLIVATLESLLRYLHWIPYRYIYETNILELLSTKFMTSPDTRAITLKCLTE
VSNLKIPQDNDLIKRQTVLFFQNTLQQIATSVMPVTADLKATYANANGNDQSFLQDLAMFLTTYL
ARNRALLESDESLRELLLNAHQYLIQLSKIEERELFKTTLDYWHNLVADLFYEPLKKHIYEEICS
QLRLVIIENMVRPEEVLVVENDEGEIVREFVKESDTIQLYKSEREVLVYLTHLNVIDTEEIMISK
LARQIDGSEWSWHNINTLSWAIGSISGTMSEDTEKRFVVTVIKDLLDLCVKKRGKDNKAVVASDI
MYVVGQYPRFLKAHWNFLRTVILKLFEFMHETHEGVQDMACDTFIKIVQKCKYHFVIQQPRESEP
FIQTIIRDIQKTTADLQPQQVHTFYKACGIIISEERSVAERNRLLSDLMQLPNMAWDTIVEQSTA
NPTLLLDSETVKIIANIIKTNVAVCTSMGADFYPQLGHIYYNMLQLYRAVSSMISAQVAAEGLIA
TKTPKVRGLRTIKKEILKLVETYISKARNLDDVVKVLVEPLLNAVLEDYMNNVPDARDAEVLNCM
TTVVEKVGHMIPQGVILILQSVFECTLDMINKDFTEYPEHRVEFYKLLKVINEKSFAAFLELPPA
AFKLFVDAICWAFKHNNRDVEVNGLQIALDLVKNIERMGNVPFANEFHKNYFFIFVSETFFVLTD
SDHKSGFSKQALLLMKLISLVYDNKISVPLYQEAEVPQGTSNQVYLSQYLANMLSNAFPHLTSEQ
IASFLSALTKQCKDLVVFKGTLRDFLVQIKEVGGDPTDYLFAEDKENA
>3UPLA
MTTNVALVGLARDLAARAETGKPIRIGLIGAGEMGTDIVTQVARMQGIEVGALSARRLPNTFKAI
RTAYGDEENAREATTESAMTRAIEAGKIAVTDDNDLILSNPLIDVIIDATGIPEVGAETGIAAIR
NGKHLVMMNVEADVTIGPYLKAQADKQGVIYSLGAGDEPSSCMELIEFVSALGYEVVSAGKGKNN
PLNFDATPDDYRQEADRRNMNVRLLVEFIDGSKTMVEMAAIANATGLVPDIAGMHGPRASIDQLS
HTLIPQAEGGVLSKSGVVDYSIGKGVSPGVFVVAKMDHPRLNERLEDLKIGKGPYFTFHRPYHLT
SLEVPLTVARVVLHGKTDMVPLPKPVAEVCAVAKKDMQPGEHLDAIGQYCYRSWIMTVPEARAAK
AIPCGLLQNGTVIAPIKKGELITYANAAPQPGSRIAELRALQDAMLGQLEHHHHHH
>1BYIA
SKRYFVTGTDTEVGKTVASCALLQAAKAAGYRTAGYKPVASGSEKTPEGLRNSDALALQRNSSLQ
LDYATVNPYTFAEPTSPHIISAQEGRPIESLVMSAGLRALEQQADWVLVEGAGGWFTPLSDTFTF
ADWVTQEQLPVILVVGVKLGCINHAMLTAQVIQHAGLTLAGWVANDVTPPGKRHAEYMTTLTRMI

PAPLLGEIPWLAENPENAATGKYINLALL
>2PS1A
MPIMLEDYQKNFLELAIECQALRFGSFKLKSGRESPYFFNLGLFNTGKLLSNLATAYAIAIIQSD
LKFDVIFGPAYKGIPLAAIVCVKLAEIGGSKFQNIQYAFNRKEAKDHGEGGIIVGSALENKRILI
IDDVMTAGTAINEAFEIISNAKGQVVGSIIALDRQEVVSTDDKEGLSATQTVSKKYGIPVLSIVS
LIHIITYLEGRITAEEKSKIEQYLQTYGASA
>3H4XA
MGSSHHHHHHSSGLVPRGSHMEPAATTYGTSTSVGVHNAYEKEKYRYFADALDSGAALLELDLWS
NALGRSWRVSHSNPLGNNSNCEGAANASELRTKSRDQDFAGCLSDMRAWHDAHPGHRPILLKIEM
KDGFNAKGGRGPAEFDALIRQKLGDAVYGPGDLTGGHATADEAVRAGGWPSRADLAGKFLFELIP
GTVEEKNPFDKLWTDVEYAGHLKDLAAQGKLAQSTAFPAVHGAAPGDPRERYADPALRPWFVVFD
GDAATYLNGSIDTSWYDTRHYLLIMTDAHNVPPVIDGTHPTEAEALARVRQLAAAHASFATADWY
PLPSVLKTVVPRGA
>3IOXA
AANEADYQAKLTAYQTELARVQKANADAKAAYEAAVAANNAANAALTAENTAIKKRNADAKADYE
AKLAKYQADLAKYQKDLADYPVKLKAYEDEQTSIKAALAELEKHKNEDGNLTEPSAQNLVYDLEP
NANLSLTTDGKFLKASAVDDAFSKSTSKAKYDQKILQLDDLDITNLEQSNDVASSMELYGNFGDK
AGWSTTVSNNSQVKWGSVLLERGQSATATYTNLQNSYYNGKKISKIVYKYTVDPKSKFQGQKVWL
GIFTDPTLGVFASAYTGQVEKNTSIFIKNEFTFYDEDGKPINFDNALLSVASLNREHNSIEMAKD
YSGKFVKISGSSIGEKNGMIYATDTLNFKQGEGGSRWTMYKNSQAGSGWDSSDAPNSWYGAGAIK
MSGPNNYVTVGATSATNVMPVSDMPVVPGKDNTDGKKPNIWYSLNGKIRAVNVPKVTKEKPTPPV
KPTAPTKPTYETEKPLKPAPVAPNYEKEPTPPTRLEHHHHHH
>1E8NA
MLSFQYPDVYRDETAIQDYHGHKVCDPYAWLEDPDSEQTKAFVEAQNKITVPFLEQCPIRGLYKE
RMTELYDYPKYSCHFKKGKRYFYFYNTGLQNQRVLYVQDSLEGEARVFLDPNILSDDGTVALRGY
AFSEDGEYFAYGLSASGSDWVTIKFMKVDGAKELPDVLERVKFSCMAWTHDGKGMFYNAYPQQDG
KSDGTETSTNLHQKLYYHVLGTDQSEDILCAEFPDEPKWMGGAELSDDGRYVLLSIREGCDPVNR
LWYCDLQQESNGITGILKWVKLIDNFEGEYDYVTNEGTVFTFKTNRHSPNYRLINIDFTDPEESK
WKVLVPEHEKDVLEWVACVRSNFLVLCYLHDVKNTLQLHDLATGALLKIFPLEVGSVVGYSGQKK
DTEIFYQFTSFLSPGIIYHCDLTKEELEPRVFREVTVKGIDASDYQTVQIFYPSKDGTKIPMFIV
HKKGIKLDGSHPAFLYGYGGFNISITPNYSVSRLIFVRHMGGVLAVANIRGGGEYGETWHKGGIL
ANKQNCFDDFQCAAEYLIKEGYTSPKRLTINGGANGGLLVATCANQRPDLFGCVIAQVGVMDMLK
FHKYTIGHAWTTDYGCSDSKQHFEWLIKYSPLHNVKLPEADDIQYPSMLLLTADHDDRVVPLHSL
KFIATLQYIVGRSRKQNNPLLIHVDTKAGHGAGKPTAKVIEEVSDMFAFIARCLNIDWIP
>3IC8A
MSELILHHYPTSLFAEKARLMLGFKGVNWRSVTIPSIMPKPDLTALTGGYRKTPVLQIGADIYCD
TALMARRLEQEKASPAFYPQGQEFAVAGLAAWADSVLFLHAVSLVFQPESMAVRFAKVPPDAAKA
FIADRSMLFNGGTASRPPVEQVKHQWPTFMSRLESQLSHGGDFLFGAPSIADFSVAHTLWFLKQT
PVTAPFVDDYPSVSVWLDRVLGFGHGSLSDLSSAAAIEIASNATPAPLPDETFIDPNGFKAGDKV
AIAAVDYGVEAVEGELMFTGREELILRREDNRAGVVHVHFPRLGFRVEKR
>3SUKA
SGAVQLRFDNTYDNASGSMNTVACSTGANGLSQRFPTFGSVPTFPHIGASSDIGGFNSPACGNCY
TISFTFQGVTRSINLVAIDHAGNGFNVAQAAMDELTNGNAVALGTIDVQSQQVARSVCGL
>1O7FA

GSPGIPMVAAHAAHSQSSAEWIACLDKRPLERSSEDVDIIFTRLKGVKAFEKFHPNLLRQICLCG
YYENLEKGITLFRQGDIGTNWYAVLAGSLDVKVSETSSHQDAVTICTLGIGTAFGESILDNTPRH
ATIVTRESSELLRIEQEDFKALWEKYRQYMAGLLAPPYGVMETGSNNDRIPDKENVPSEKILRAG
KILRIAILSRAPHMIRDRKYHLKTYRQCCVGTELVDWMIQQTSCVHSRTQAVGMWQVLLEDGVLN
HVDQERHFQDKYLFYRFLDDEREDAPLPTEEEKKECDEELQDTMLLLSQMGPDAHMRMILRKPPG
QRTVDDLEIIYDELLHIKALSHLSTTVKRELAGVLIFESHAKGGTVLFNQGEEGTSWYIILKGSV
NVVIYGKGVVCTLHEGDDFGKLALVNDAPRAASIVLREDNCHFLRVDKEDFNRILRDVEANTVRL
KEHDQDVLVLEKVP
>4FCZA
MISILRRGLLVLLAAFPLLALAVQTPHEVVQSTTNELLGDLKANKEQYKSNPNAFYDSLNRILGP
VVDADGISRSIMTVKYSRKATPEQMQRFQENFKRSLMQFYGNALLEYNNQGITVDPAKADDGKRA
SVGMKVTGNNGAVYPVQYTLENIGGEWKVRNVIVNGINIGKLFRDQFADAMQRNGNDLDKTIDGW
AGEVAKAKQAADNSPEKSVKLEHHHHHH
>4GI3C
SEDDGSASPESQEMSYTELPCPSICPLIYAPVCVEDSNQDFYLFVNECEVRKCGCEAGFVYTFVP
REMCKATTSLCPMQTKSS
>3PNAA
GRRRRGAISAEVYTEEDAASYVRKVIPKDYKTMAALAKAIEKNVLFSHLDDNERSDIFDAMFPVS
FIAGETVIQQGDEGDNFYVIDQGEMDVYVNNEWATSVGEGGSFGELALIYGTPRAATVKAKTNVK
LWGIDRDSYRRILMGSTLRKRKMY
>3GONA
MIAVKTCGKLYWAGEYAILEPGQLALIKDIPIYMRAEIAFSDSYRIYSDMFDFAVDLRPNPDYSL
IQETIALMGDFLAVRGQNLRPFSLAIYGKMEREGKKFGLGSSGSVVVLVVKALLALYNLSVDQNL
LFKLTSAVLLKRGDNGSMGDLACIAAEDLVLYQSFDRQKVAAWLEEENLATVLERDWGFSISQVK
PTLECDFLVGWTKEVAVSSHMVQQIKQNINQNFLTSSKETVVSLVEALEQGKSEKIIEQVEVASK
LLEGLSTDIYTPLLRQLKEASQDLQAVAKSSGAGGGDCGIALSFDAQSTKTLKNRWADLGIELLY
QERIGHDDKS
>4FSFA
HHHHHHARSVRHIAIPAHRGLITDRNGEPLAVSTPVTTLWANPKELMTAKERWPQLAAALGQDTK
LFADRIEQNAEREFIYLVRGLTPEQGEGVIALKVPGVYSIEEFRRFYPAGEVVAHAVGFTDVDDR
GREGIELAFDEWLAGVPGKRQVLKDRRGRVIKDVQVTKNAKPGKTLALSIDLRLQYLAHRELRNA
LLENGAKAGSLVIMDVKTGEILAMTNQPTYNPNNRRNLQPAAMRNRAMIDVFEPGSTVKPFSMSA
ALASGRWKPSDIVDVYPGTLQIGRYTIRDVSRNSRQLDLTGILIKSSNVGISKIAFDIGAESIYS
VMQQVGLGQDTGLGFPGERVGNLPNHRKWPKAETATLAYGYGLSVTAIQLAHAYAALANDGKSVP
LSMTRVDRVPDGVQVISPEVASTVQGMLQQVVEAQGGVFRAQVPGYHAAGKSGTARKVSVGTKGY
RENAYRSLFAGFAPATDPRIAMVVVIDEPSKAGYFGGLVSAPVFSKVMAGALRLMNVPPDNLPTA
TEQQQVNAAPAKGGRG
>1TD6A
MHHHHHHGGGGGMINKPNQFVNHLSALKKHFASYKELREAFNDYHKHNGDELTTFFLHQFDKVME
LVKQKDFKTAQSRCEEELAAPYLPKPLVSFFQSLLQLVNHDLLEQQNAALASLPAAKIIELVLQD
YPNKLNMIHYLLPKTKAFVKPHLLQRLQFVLTDSELLELKRFSFFQALNQIPGFQGEQVEYFNSK
LKQKFTLTLGEFEIAQQPDAKAYFEQLITQIQQLFLKEPVNAEFANEIIDAFLVSYFPLHPPVPL
AQLAAKIYEYVSQIVLNEAVNLKDELIKLIVHTLYEQLDRPVGDEN
>2PIFA

MNAFDRARQSAIAAAREARGTYRNGLVTPTAGVAPGMTQANLIALPRDWAYDFLLYAQRNPKACP
ILDVSDAGSPTTLLAEGSDLRTDIPMYRIWRDGKLAEEVSDATQAWAEHDDMVAFLIGCSFTFET
PLQEAGIEVRHITDGCNVPMYRTNRACRPAGRLHGEMVVSMRPIPADRVAEASAISGRYPSVHGA
PVHIGEPGRLGINDLSRPDFGDAVSIKPGEVPVFWACGVTPQAAVMASGVPFAITHSPGYMFITD
VPDSTYHVLEHHHHHH
>3LWTX
SMTGPIVYVQNADGIFFKLAEGKGTNDAVIHLANQDQGVRVLGAEEFPVQGEVVKIASLMGFIKL
KLNRYAIIANTVEETGRFNGHVFYRVLQHSIVSTKFNSRIDSEEAEYIKLLELHLKNSTFYFSYT
YDLTNSLQRNEKVGPAASWKTADERFFWNHYLTEDLRNFAHQDPRIDSFIQPVIYGYAKTVDAVL
NATPIVLGLITRRSIFRAGTRYFRRGVDKDGNVGNFNETEQILLAENPESEKIHVFSFLQTRGSV
PIYWAEINNLKYKPNLVLGENSLDATKKHFDQQKELYGDNYLVNLVNQKGHELPVKEGYESVVHA
LNDPKIHYVYFDFHHECRKMQWHRVKLLIDHLEKLGLSNEDFFHKVIDSNGNTVEIVNEQHSVVR
TNCMDCLDRTNVVQSVLAQWVLQKEFESADVVATGSTWEDNAPLLTSYQNLWADNADAVSVAYSG
TGALKTDFTRTGKRTRLGAFNDFLNSASRYYQNNWTDGPRQDSYDLFLGG
>1YUEA
MAKINELLRESTTTNSNSIGRPNLVALTRATTKLIYSDIVATQRTNQPVAAFYGIKYLNPDNEFT
FKTGATYAGEAGYVDREQITELTEESKLTLNKGDLFKYNNIVYKVLEDTPFATIEESDLELALQI
AIVLLKVRLFSDAASTSKFESSDSEIADARFQINKWQTAVKSRKLKTGITVELAQDLEANGFDAP
NFLEDLLATEMADEINKDILQSLITVSKRYKVTGITDSGFIDLSYASAPEAGRSLYRMVCEMVSH
IQKESTYTATFCVASARAAAILAASGWLKHKPEDDKYLSQNAYGFLANGLPLYCDTNSPLDYVIV
GVVENIGEKEIVGSIFYAPYTEGLDLDDPEHVGAFKVVVDPESLQPSIGLLVRYALSANPYTVAK
DEKEARIIDGGDMDKMAGRSDLSVLLGVKLPKIIIDE
>3HXJA
MLRGGVNDCKIKWEFLIGNSIDSSPILAKNGTIYLGSSNKNLYAINTDGSVKWFFKSGEIIECRP
SIGKDGTIYFGSDKVYAINPDGTEKWRFDTKKAIVSDFTIFEDILYVTSMDGHLYAINTDGTEKW
RFKTKKAIYATPIVSEDGTIYVGSNDNYLYAINPDGTEKWRFKTNDAITSAASIGKDGTIYFGSD
KVYAINPDGTEKWNFYAGYWTVTRPAISEDGTIYVTSLDGHLYAINPDGTEKWRFKTGKRIESSP
VIGNTDTIYFGSYDGHLYAINPDGTEKWNFETGSWIIATPVIDENGTIYFGTRNGKFYALFNLEH
HHHHH
>1SO4A
MSLPMLQVALDNQTMDSAYETTRLIAEEVDIIEVGTILCVGEGVRAVRDLKALYPHKIVLADAAI
ADAGKILSRMCFEANADWVTVICCADINTAKGALDVAKEFNGDVQIELTGYWTWEQAQQWRDAGI
GQVVYHRSRDAQAAGVAWGEADITAIKRLSDMGFKVTVTGGLALEDLPLFKGIPIHVFIAGRSIR
DAASPVEAARQFKRSIAELWG
>2HHCA
MTKERFVISRRRTGFGDCLWSLASAWSYAQRTGRTLVIDWRGSCYVEQPFSNAFPAFFEPVEDIA
GVPVICDDRVNQLSFPGPFFPRWWNRPSIDCINRPDEQIFRERDELTELFQAREDSEANTIVCDA
CLMWRCSEEAERLIFRNIKLRSEIRARIDALYEEHFSGHSIIGVHVRHGNGEDIMEHAPYWADSE
LALHQVCMAIRKAKALSYPKPVKVFLCTDSAQVLDQVSGLFPDVFAVPKRFQADRAGPLHSAEMG
IEGGASALIDMYLLARCATVIRFPPTSAFTRYARLLVPRIIEFDLSNPGHLTMIDNPYEHFAASH
HHHHH
>1JHSA
MNNKEVELYGGAITTVVPPGFIDASTLREVPDTQAVYVNSRRDEEEFEDGLATNESIIVDLLETV
DKSDLKEAWQFHVEDLTELNGTTKWEALQEDTVQQGTKFTGLVMEVANKWGKPDLAQTVVIGVAL

IRLTQFDTDVVISINVPLTKEEASQASNKELPARCHAVYQLLQEMVRKFHVVDTSLFA
>1QFJA
TTLSCKVTSVEAITDTVYRVRIVPDAAFSFRAGQYLMVVMDERDKRPFSMASTPDEKGFIELHIG
ASEINLYAKAVMDRILKDHQIVVDIPHGEAWLRDDEERPMILIAGGTGFSYARSILLTALARNPN
RDITIYWGGREEQHLYDLCELEALSLKHPGLQVVPVVEQPEAGWRGRTGTVLTAVLQDHGTLAEH
DIYIAGRFEMAKIARDLFCSERNAREDRLFGDAFAFI
>2HQBA
GGGGGGMVGLLVEDTIDDQGWNRKAYEGLLNIHSNLDVDVVLEEGVNSEQKAHRRIKELVDGGVN
LIFGHGHAFAEYFSTIHNQYPDVHFVSFNGEVKGENITSLHFEGYAMGYFGGMVAASMSETHKVG
VIAAFPWQPEVEGFVDGAKYMNESEAFVRYVGEWTDADKALELFQELQKEQVDVFYPAGDGYHVP
VVEAIKDQGDFAIGYVGDQADLGGSTILTSTVQHVDDLYVLVAKRFQEGKLESGNLYYDFQDGVV
SLGEFSSVVPDEVREQITDAISTYIQTGQFPHEEER
>1YARA
MQQGQMAYSRAITVFSPDGRLFQVEYAREAVKKGSTALGMKFANGVLLISDKKVRSRLIEQNSIE
KIQLIDDYVAAVTSGLVADARVLVDFARISAQQEKVTYGSLVNIENLVKRVADQMQQYTQYGGVR
PYGVSLIFAGIDQIGPRLFDCDPAGTINEYKATAIGSGKDAVVSFLEREYKENLPEKEAVTLGIK
ALKSSLEEGEELKAPEIASITVGNKYRIYDQEEVKKFL
>4GMOA
MGKTRRNRVRNRTDPIAKPVKPPTDPELAKLREDKILPVLKDLKSPDAKSRTTAAGAIANIVQDA
KCRKLLLREQVVHIVLTETLTDNNIDSRAAGWEILKVLAQEEEADFCVHLYRLDVLTAIEHAAKA
VLETLTTSEPPFSKLLKAQQRLVWDITGSLLVLIGLLALARDEIHEAVATKQTILRLLFRLISAD
IAPQDIYEEAISCLTTLSEDNLKVGQAITDDQETHVYDVLLKLATGTDPRAVMACGVLHNVFTSL
QWMDHSPGKDGACDAILIPTLTRALEHVVPGGAKFNGDARYANITLLALVTLASIGTDFQETLVK
GNQGSRESPISAADEEWNGFDDADGDAMDVDQKSSSGEDQEEDYEEIDVKEDDEDDDDDSITSEM
QADMERVVGADGTDDGDLEDLPTLRELIQTAVPQLIRLSNLPIDSDESLTIQSHALSALNNISWT
ISCLEFANGENANIHNAWYPTAKKIWRKTILPILEADSADLKLATQVTSLAWAVARVLHGETPTD
GNPHRKFISLYHSSKQQAGGNSNSIEEPEDPFQGLGVKCIGVVGSLAHDPAPIEVNREVGVFLVT
LLRQSNNVPPAEIVEALNQLFDIYGDEELACDKEVFWKDGFLKHLEEFLPKMRTLTKGIDKRTQP
ELRTRADEALLNLGRFVQYKKKHAPKGSHHHHHH
>3GZBA
GMFASLVIPVSAQANSGEMPQEQQLAVKYMDALTEHDYKTLITFYNRDSIFFDKTANRKYTGGRF
IIDFLERAHQGVLEYDFNIEHMYNAGSLVVMIGNYHFKGPGEQFGKPGKIIDVAIPAVTSLKLDM
LNRRVTEHVDLIDYQTMSDQLAMQ
>3POJA
MVECSGNLFTQRTGTITSPDYPNPYPKSSECSYTIDLEEGFMVTLQFEDIFDIEDHPEVPCPYDY
IKIKAGSKVWGPFCGEKSPEPISTQSHSIQILFRSDNSGENRGWRLSYRA
>3CGMA
MKVGQDKVVTIRYTLQVEGEVLDQGELSYLHGHRNLIPGLEEALEGREEGEAFQAHVPAEKAYGP
HDPEGVQVVPLSAFPEDAEVVPGAQFYAQDMEGNPMPLTVVAVEGEEVTVDFNHPLAGKDLDFQV
EVVKVREATPEELLHGHAHPSGHHHHHH
>3QRAA
MEGESSISIGYAQSRVKEDGYKLDKNPRGFNLKYRYEFNNDWGVIGSFAQTRRGFEESVDGFKLI
DGDFKYYSVTAGPVFRINEYVSLYGLLGAGHGKAKFSSIFGQSESRSKTSLAYGAGLQFNPHPNF
VIDASYEYSKLDDVKVGTWMLGAGYRF

>4EXKA

SNAAKIEEGKLVIWINGDKGYNGLAEVGKKFEKDTGIKVTVEHPDKLEEKFPQVAATGDGPDIIF
WAHDRFGGYAQSGLLAEITPAAAFQDKLYPFTWDAVRYNGKLIAYPIAVEALSLIYNKDLLPNPP
KTWEEIPALDKELKAKGKSALMFNLQEPYFTWPLIAADGGYAFKYAAGKYDIKDVGVDNAGAKAG
LTFLVDLIKNKHMNADTDYSIAEAAFNKGETAMTINGPWAWSNIDTSAVNYGVTVLPTFKGQPSK
PFVGVLSAGINAASPNKELAKEFLENYLLTDEGLEAVNKDKPLGAVALKSYEEELAKDPRIAATM
ENAQKGEIMPNIPQMSAFWYAVRTAVINAASGRQTVDAALAAAQTNAAANTPSPDLTVMSIDKSV
LSPGESATITTIVKDIDGNPVNEVHINKTVARENLKGLWDYGPLKKENVPGKYTQVITYRGHSNE
RIDISFKYAMSFTKEISIRGRLSLSSVKNNIG

>2Q7WA

MFENITAAPADPILGLADLFRADERPGKINLGIGVYKDETGKTPVLTSVKKAEQYLLENETTKNY
LGIDGIPEFGRCTQELLFGKGSALINDKRARTAQTPGGTGALRVAADFLAKNTSVKRVWVSNPSW
PNHKSVFNSAGLEVREYAYYDAENHTLDFDALINSLNEAQAGDVVLFHGCCHNPTGIDPTLEQWQ
TLAQLSVEKGWLPLFDFAYQGFARGLEEDAEGLRAFAAMHKELIVASSYSKNFGLYNERVGACTL
VAADSETVDRAFSQMKAAIRANYSNPPAHGASVVATILSNDALRAIWEQELTDMRQRIQRMRQLF
VNTLQEKGANRDFSFIIKQNGMFSFSGLTKEQVLRLREEFGVYAVASGRVNVAGMTPDNMAPLCE
AIVAVL

>3EPOA

MNIQSTIKAVAETISTGPIPGSRKVYQAGELFPELRVPFREVAVHPSANEPPVTIYDPSGPYSDP
AIQIDIEKGLPRTREALVVARGDVEEVADPRQVKPEDNGFAQGKHLAPEFPDTGRKIYRAKPGKL
VTQLEYARAGIITAEMEYVAIRENLRREQDRPCVRDGEDFGASIPDFVTPEFVRQEIARGRAIIP
ANINHGELEPMAIGRNFLVKINANIGNSAVLSTVADEVDKLVWATRWGADTVMDLSTGRNIHNIR
DWIIRNSSVPIGTVPIYQALEKVNGVAEDLNWEVFRDTLIEQCEQGVDYFTIHAGVRLPFIPMTA
KRVTGIVSRGGSIMAKWCLAHHKENFLYERFDEICEIMRAYDVSFSLGDGLRPGSTADANDEAQF
SELRTLGELTKVAWKHGVQVMIEGPGHVAMHKIKANMDEQLKHCHEAPFYTLGPLTTDIAPGYDH
ITSAIGAAMIGWFGTAMLCYVTPKEHLGLPDRDDVKTGVITYKLAAHAADLAKGHPGAAMWDDAI
SRARFEFRWEDQFNLGLDPETARKFHDETLPKEAHKTAHFCSMCGPKFCSMKISQEVRDFAAGKA
PNSAELGMAEMSEKFREQGSEIYLKTE

>2XSGA

APEPPSADYASLVDVFVGTEGDFGNDMPAAQAPNGLAKVNPRTTPGRNNTGYDYAQSKISGFTHT
NLDGVGGSGGGGDLLVVPTSGSYTARPGTGTYAHPFSHDDEDAGPGFYSVGLGNVAGTDGAITGA
PGTIEAEVAAATRSGVHRYAFPAGSTPSLVVDLETNNTSRRSSSVQVETRADGTVELSGQVTGYF
YNAAYTLYYTARTLQPATVQTWGDDDRLVDATAQDGVDTGAILTFDPADAGEIGLQVTLSPVSVE
QARIDQQVELGDLSFDAIRDRTRAEWNATLGRVAIDASTATDPTGELQRLFYTHLYRMFAMPMNA
TSTSGTYRGVDGAVHAAQGFTYYDSWATWDDFRKFSVIAYIDPALYRDMVQSLVYLFADAEATGT
GGGLGGFVHSVPTVRWERSSVVVADAIAKGFDGFDRLDEAYPALQRLVGQYSADELRRGYVAGNP
GASVQRGYDQYGLSVIADELGLTEEAETLREQASWPIEKLTKPGAWTAADGTQVGLLTPRAADGS
WQSADHAKFEAAGLYQGTLWQYHWYDAYDMDALVEAMGGHEAARLGMRHMFGEHAPDDGKAMLHS
NANEIDLQAPYLFNYTGEPSLTQKWARAIYTKETWNRYIATGSSSAVPSGGGEFTPPLKTKVYRL
DPRGMLPTMDNDAGTMSTMFVAAAVGLFPVTAGSSQFQVGSPFFDSTTITYDDGSAFTVTADGVS
EDAFYVQSATLDGATFGNTWVDYATVVGGADLAFRMGEQPSDWGTDTAPAFSMSTATDE

>2VVPA

MSGMRVYLGADHAGYELKQRIIEHLKQTGHEPIDCGALRYDADDDYPAFCIAAATRTVADPGSLG
IVLGGSGNGEQIAANKVPGARCALAWSVQTAALAREHNNAQLIGIGGRMHTVAEALAIVDAFVTT

PWSKAQRHQRRIDILAEYERTHEAPPVPGAPA
>2XQUA
MESNLTTAASVIAAALAVGIGSIGPGLGQGQAAGQAVEGIARQPEAEGKIRGTLLLSLAFMEALT
IYGLVVALVLLFANPFV
>3S1SA
MGGKGLNNCVFSYLPSYGDDEVSVYHPICEAALNQALVNTGLDSTYEVVHHELVGSIEADFVIKN
KQTKKYLLIVEVKRTKSQVSSTRYRLQAQSYVREANIKVEQHYYCLTNLEIIDFFKHDPNKPVVS
QQIIEPSPIVVGNFSDTVSEFYNRLVEAFQNIIDISVNDAGTYKSSTANLVDILENRKDNSTSWH
QALVVAGYEYIRGVLRGQNVEVPTRDAIYFKSRPGRLLEEGRKIDFNVLFSEPEPNTNDNDIWNV
NLLSSLNDLGRRILTGDELAELIHDIATRGRGHEGVVPTDIELGKVLSIISQHILGRPLTEDEVI
SDPAAGSGNLLATVSAGFNNVMPRQIWANDIETLFLELLSIRLGLLFPQLVSSNNAPTITGEDVC
SLNPEDFANVSVVVMNPPYVSGVTDPAIKRKFAHKIIQLTGNRPQTLFGQIGVEALFLELVTELV
QDGTVISAIMPKQYLTAQGNESKAFREFLVGNFGLEHIFLYPREGLFEEVIKDTVVFVGRKGSSV
EEIEVLDSFTPLEQVDLHNLKRALSNSSNEQIIQPMGMELRKEKREELENRVTVGWRHITSNGRV
AEEWITNNLESHCIRLVASDYDLRRGRVGNKGASDLLFINSKKKLWDLLDESVPRDWLYPALRKV
NEINTPIFNEDATPVRFLCPPNSAYQDGTGESIILDKILDVYVDFQVYKSKQKKFEKSKEELKEI
LYKESDFYSSEHTVFIPRALRRSARAFINEQKVFCSTNALEVFGGNSEEMWLLLSWLSSVFAQLQ
FEAMAKDQEGERKLEKKSIQNLYIPNLGDIDDVLKQDLIEEVREIHFFDLCRPRVRKLDLLWAKV
FWSGNEMSKTKEAAELLEDLVFERYPEGSQIGE
>2RK9A
MSLTLRVVPELYCFDINVSQSFFVDVLGFEVKYERPDEEFVYLTLDGVDVMLEGIAGKSRKWLSG
DLEFPLGSGVNFQWDVIDIEPLYQRVNESAADSIYLALESKSYQCGDSIATQKQFMVQTPDGYLF
RFCQDIHEGHHHHHH
>3GKMA
SNAMTDAVLELPAATFDLPLSLSGGTQTTLRAHAGHWLVIYFYPKDSTPGSTTEGLDFNALLPEF
DKAGAKILGVSRDSVKSHDNFSAKQGFAFPLVSDGDEALCRAFDVIKEKNMYGKQVLGIERSTFL
LSPEGQVVQAWRKVKVAGHADAVLAALKAHAKQ
>2HBVA
MKKPRIDMHSHFFPRISEQEAAKFDANHAPWLQVSAKGDTGSIMMGKNNFRPVYQALWDPAFRIE
EMDAQGVDVQVTCATPVMFGYTWEANKAAQWAERMNDFALEFAAHNPQRIKVLAQVPLQDLDLAC
KEASRAVAAGHLGIQIGNHLGDKDLDDATLEAFLTHCANEDIPILVHPWDMMGGQRMKKWMLPWL
VAMPAETQLAILSLILSGAFERIPKSLKICFGHGGGSFAFLLGRVDNAWRHRDIVREDCPRPPSE
YVDRFFVDSAVFNPGALELLVSVMGEDRVMLGSDYPFPLGEQKIGGLVLSSNLGESAKDKIISGN
ASKFFNINV
>3B64A
PVIQTFVSTPLDHHKRENLAQVYRAVTRDVLGKPEDLVMMTFHDSTPMHFFGSTDPVACVRVEAL
GGYGPSEPEKVTSIVTAAITKECGIVADRIFVLYFSPLHCGWNGTNF
>3RWNA
GPKVPEFGSSRIDALEYATTRKKSEVVYSGVSVTIPTAPTNLVSLLKTLTPSSGTLAPFFDTVNN
KMVVFNENKTLFFKLSIVGTWPSGTANRSMQLTFSGSVPDTLVSSRNSATTTDNILLATFFSVDK
DGFLATNGSTLTIQSNGASFTATTIKIIAEQ
>1ZK5A
AVSFIGSTENDVGPSQGSYSSTHAMDNLPFVYNTGHNIGYQNANVWRISGGFCVGLDGKVDLPVV
GSLDGQSIYGLTEEVGLLIWMGDTNYSRGTAMSGNSWENVFSGWCVGNYVSTQGLSVHVRPVILK

RNSSAQYSVQKTSIGSIRMRPYNGSSAGSVQTTVNFSLNPFTLNDT
>4K4KA
GGENTPQPTDGRVALEATSGIRMNTRAYDKTWEAGDAIGIYMLNGDATDGNGNRKYTTAQTAENG
SFTAAEGQTIYFPVDASQRDFVAYYPYRETLADGNVYTVDVSVQTPQKDIDLMGAAKVEGKDKTD
PKVAFVFTHKLVKLDITIKADGTSLTDADLAGTTVSISNQQTAATYNVVTGGDATVTTGTTKEIV
LHTDGLKAEGIVLPAASTAGMALTFTVPGLEGQAFHWDVNSAAQSKAFVAGSKYLYTITISKAGV
EVSSKVEDWTPGNGGGETGNAE
>3LFRA
ADLQVRDIMVPRSQMISIKATQTPREFLPAVIDAAHSRYPVIGESHDDVLGVLLAKDLLPLILKA
DGDSDDVKKLLRPATFVPESKRLNVLLREFRANHNHMAIVIDEYGGVAGLVTIEDVLEQIVGDIE
DEHDVE
>1NKZA
MNQGKIWTVVNPAIGIPALLGSVTVIAILVHLAILSHTTWFPAYWQGGVKKAA
>3BYQA
GMSLIEIRKRTLIVETTYHENGPAPAQPLKLAASCAVIRNPYAGRYEPDLMPFMAELRSLGTLLA
TELVDTLGKDNIEVYSKAAIVGVDGEMEHGAVWHEAGGWAMRSVLGEPKAMVPAVKAVATAGYRM
MVPVHYIHASYVRSHFNSIEIGIQDAPRPREILFALVMGTGARVHARLGGLTKEAVSVHDGQR
>2H7MA
MTGLLDGKRILVSGIITDSSIAFHIARVAQEQGAQLVLTGFDRLRLIQRITDRLPAKAPLLELDV
QNEEHLASLAGRVTEAIGAGNKLDGVVHSIGFMPQTGMGINPFFDAPYADVSKGIHISAYSYASM
AKALLPIMNPGGSIVGMDFDPSRAMPAYNWMTVAKSALESVNRFVAREAGKYGVRSNLVAAGPIR
TLAMSAIVGGALGEEAGAQIQLLEEGWDQRAPIGWNMKDATPVAKTVCALLSDWLPATTGDIIYA
DGGAHTQLL
>3GMGA
MSLQASGNTDQAALESAQARLAALSILVGAVGATGPGVMITIDDPGPGVAPEVMIDVINELRAAG
AEAIQINDAHRSVRVGVDTWVVGVPGSLTVDTKVLSPPYSILAIGDPPTLAAAMNIPGGAQDGVK
RVGGRMVVQQADRVDVTALRQPKQHQYAQPVKEGHHHHHH
>3HSUA
EAEAEFNSINACLAAADVEFHEEDSEGWDMDGTAFNLRVDYDPAAIAIPRSTEDIAAAVQCGLDA
GVQISAKGGGHSYGSYGFGGEDGHLMLELDRMYRVSVDDNNVATIQGGARLGYTALELLDQGNRA
LSHGTAPAVGVGGHVLGGGYGFATHTHGLTLDWLIGATVVLADASIVHVSETENADLFWALRGGG
GGFAIVSEFEFNTFEAPEIITTYQVTTTWNRKQHVAGLKALQDWAQNTMPRELSMRLEINANALN
WEGNFFGNAKDLKKILQPIMKKAGGKSTISKLVETDWYGQINTYLYGADLNITYNYDVHEYFYAN
SLTAPRLSDEAIQAFVDYKFDNSSVRPGRGWWIQWDFHGGKNSALAAVSNDETAYAHRDQLWLWQ
FYDSIYDYENNTSPYPESGFEFMQGFVATIEDTLPEDRKGKYFNYADTTLTKEEAQKLYWRGNLE
KLQAIKAKYDPEDVFGNVVSVEPIAYLEQKLISEEDLNSAVDHHHHHH
>3Q6VA
MSEKNLTLTHFKGPLYIVEDKEYVQENSMVYIGTDGITIIGATWTPETAETLYKEIRKVSPLPIN
EVINTNYHTDRAGGNAYWKTLGAKIVATQMTYDLQKSQWGSIVNFTRQGNNKYPNLEKSLPDTVF
PGDFNLQNGSIRAMYLGEAHTKDGIFVYFPAERVLYGNCILKENLGNMSFANRTEYPKTLEKLKG
LIEQGELKVDSIIAGHDTPIHDVGLIDHYLTLLEKAPK
>3DB2A
GMYNPVGVAAIGLGRWAYVMADAYTKSEKLKLVTCYSRTEDKREKFGKRYNCAGDATMEALLARE
DVEMVIITVPNDKHAEVIEQCARSGKHIYVEKPISVSLDHAQRIDQVIKETGVKFLCGHSSRRLG

ALRKMKEMIDTKEIGEVSSIEAVFSNERGLELKKGNWRGEPATAPGGPLTQLGVHQIDNLQFLLG
PVARVFNFGKPMYTEVENITVNQTLLEFEDGKQAYLGTNWACPGVFSINVYGTKANLFYQLDFSW
WSNSDVTDEHSTLIKREFASMSDDPDNRILRDVKVDFESVDHLRVEVEEVADVIRNGGETEIGAE
ASLRNLAVVLAAVKSVHEKRPVEIAEIIG
>1J7XA
DPSVTHVLHQLCDILANNYAFSERIPTLLQHLPNLDYSTVISEEDIAAKLNYELQSLTEDPRLVL
KSKTDTLVMPGDSIQAENIPEDEAMLQALVNTVFKVSILPGNIGYLRFDQFADVSVIAKLAPFIV
NTVWEPITITENLIIDLRYNVGGSSTAVPLLLSYFLDPETKIHLFTLHNRQQNSTDEVYSHPKVL
GKPYGSKKGVYVLTSHQTATAAEEFAYLMQSLSRATIIGEITSGNLMHSKVFPFGDTQLSVTVPI
INFIDSNGDYWLGGGVVPDAIVLADEALDKAKEIIAFHPPLA
>1VSGA
AAEKGFKQAFWQPLCQVSEELDDQPKGALFTLQAAASKIQKMRDAALRASIYAEINHGTNRAKAA
VIVANHYAMKADSGLEALKQTLSSQEVTATATASYLKGRIDEYLNLLLQTKESGTSGCMMDTSGT
NTVTKAGGTIGGVPCKLQLSPIQPKRPAATYLGKAGYVGLTRQADAANNFHDNDAECRLASGHNT
NGLGKSGQLSAAVTMAAGYVTVANSQTAVTVQALDALQEASGAAHQPWIDAWKAKKALTGAETAE
FRNETAGIAGKTGVTKLVEEALLKKKDSEASEIQTELKKYFSGHENEQWTAIEKLISEQPVAQNL
VGDNQPTKLGELEGNAKLTTILAYYRMETAGKFEVLTQK
>3VK5A
AGAGAMNASPQLDHHTELHAAPPLWRPGRVLARLREHQPGPVHIIDPFKVPVTEAVEKAAELTRL
GFAAVLLASTDYESFESHMEPYVAAVKAATPLPVVLHFPPRPGAGFPVVRGADALLLPALLGSGD
DYFVWKSFLETLAAFPGRIPREEWPELLLTVALTFGEDPRTGDLLGTVPVSTASTEEIDRYLHVA
RAFGFHMVYLYSRNEHVPPEVVRHFRKGLGPDQVLFVSGNVRSGRQVTEYLDSGADYVGFAGALE
QPDWRSALAEIAGRRPAAPARPGSGR
>2GS8A
SNAMDPNVITVTSYANIAIIKYWGKENQAKMIPSTSSISLTLENMFTTTSVSFLPDTATSDQFYI
NGILQNDEEHTKISAIIDQFRQPGQAFVKMETQNNMPTAAGLSSSSSGLSALVKACDQLFDTQLD
QKALAQKAKFASGSSSRSFFGPVAAWDKDSGAIYKVETDLKMAMIMLVLNAAKKPISSREGMKLC
RDTSTTFDQWVEQSAIDYQHMLTYLKTNNFEKVGQLTEANALAMHATTKTANPPFSYLTKESYQA
MEAVKELRQEGFACYFTMDAGPNVKVLCLEKDLAQLAERLGKNYRIIVSKTKDLPDV
>3AWUB
MPEITRRRALTAAAAVAATASAAVTLAAPAASAAGHHEPAAPESFDEVYKGRRIQGRPAGGGAHH
HEHGGGYEVFVDGVQLHVMRNADGSWISVVSHYDPVPTPRAAARAAVDELQGAPLLPFPANLEHH
HHHH
>2Y7EA
MHHHHHHEPLILTAAITGAETTRADQPNLPITPEEQAKEAKACFEAGARVIHLHIREDDGRPSQR
LDRFQEAISAIREVVPEIIIQISTGGAVGESFDKRLAPLALKPEMATLNAGTLNFGDDIFINHPA
DIIRLAEAFKQYNVVPEVEVYESGMVDAVARLIKKGIITQNPLHIQFVLGVPGGMSGKPKNLMYM
MEHLKEEIPTATWAVAGIGRWHIPTSLIAMVTGGHIRCGFEDNIFYHKGVIAESNAQLVARLARI
AKEIGRPLATPEQAREILALNK
>3D3OA
SNALFSSRDILEVLQDIHMETGETVAIATKNDIYLQYIQIIESVHALRFHVDENAIRPLTMSSNG
WMLMSTMNDKAIDNTVRRANTITQKDGIRFEVDDMMARIRQVREQGYASAEHIPFVGGGTICVLL
PMTIQGQPVTMGLGGALDRIKQNYDRYLELLLNGVQQLKKSDSFHQPI
>3H3LA

GAGTQTAQTQALDSDGIPTGGEWITMFDGKTLNGWRGYCRQDVPLGWVVEDGSITYKGSDNKADT
GFGDLIYDKKFKNFVFEIEWKIDKAGNSGIFYTAQEIEGTPIYYSSPEYQLLDNENMPDAWEGCD
GNRQAGAVYDMIMPDPQPVKPYGNWNKTRIVVYNQRVIHYMNDVKILEFQFGTPVWRALVDHSKF
SKFSTSPEKCPEAYDLMLQCGKQPGYIGMQDHGYGVCFRNIRIKEL
>3NOWA
NAKTSTKVKQMMDLTFDLATPIDKRRAAANNLVVLAKEQTGAELLYKDHCIAKVASLTKVEKDQD
IYVNMVHLVAALCENSVERTKGVLTELGVPWFMRVLDQKHENCVSTAQFCLQTILNALSGLKNKP
DSKPDKELCTRNNREIDTLLTCLVYSITDRTISGAARDGVIELITRNVHYTALEWAERLVEIRGL
CRLLDVCSELEDYKYESAMDITGSSSTIASVCLARIYENMYYDEAKARFTDQIDEYIKDKLLAPD
MESKVRVTVAITALLNGPLDVGNQVVAREGILQMILAMATTDDELQQRVACECLIAASSKKDKAK
ALCEQGVDILKRLYHSKNDGIRVRALVGLCKLGSYGGQDAAIRPFGDGAALKLAEACRRFLIKPG
KDKDIRRWAADGLAYLTLDAECKEKLIEDKASIHALMDLARGGNQSCLYGVVTTFVNLCNAYEKQ
EMLPEMIELAKFAKQHIPEEHELDDVDFINKRITVLANEGITTALCALAKTESHNSQELIARVLN
AVCGLKELRGKVVQEGGVKALLRMALEGTEKGKRHATQALARIGITINPEVSFSGQRSLDVIRPL
LNLLQQDCTALENFESLMALTNLASMNESVRQRIIKEQGVSKIEYYLMEDHLYLTRAAAQCLCNL
VMSEDVIKMFEGNNDRVKFLALLCEDEDEETATACAGALAIITSVSVKCCEKILAIASWLDILHT
LIANPSPAVQHRGIVIILNMINAGEEIAKKLFETDIMELLSGLGQLPDDTRAKAREVATQCLAAA
ERYRIIERSDNAEIPDVFAENSKISEIIDD
>1OW1A
GLVLPHTEFQPAPKQDSSPHLTSQRPVDMVQLLKKYPIVWQGLLALKNDTAAVQLHFVSGNNVLA
HRSLPLSEGGPPLRIAQRMRLEATQLEGVARRMTVETDYCLLLALPCGRDQEDVVSQTESLKAAF
ITYLQAKQAAGIINVPNPGSNQPAYVLQIFPPCEFSESHLSRLAPDLLASISNISPHLMIVIASV
>1W0NA
WQFTPNTGGNTITKVEAENMKIGGTYAGKISAPFDGVALYANADYVSYSQYFANSTHNISVRGAS
SNAGTAKVDLVIGGVTVGSFNFTGKTPTVQTLSNITHATGDQEIKLALTSDDGTWDAYVDFIEFS
L
>2BHWA
RKSATTKKVASSGSPWYGPDRVKYLGPFSGESPSYLTGEFPGDYGWDTAGLSADPETFSKNRELE
VIHSRWAMLGALGSVFPELLSRNGVKFGEAVWFKAGSQIFSEGGLDYLGNPSLVHAQSILAIWAT
QVILMGAVEGYRIAGGPLGEVVDPLYPGGSFDPLGLADDPEAFAELKVKELKNGRLAMFSMFGFF
VQAIVTGKGPLENLADHLADPVNNNAWSYATNFVPGK
>1X6ZA
ALEGTEFARSEGASALASVNPLKTTVEEALSRGWSVKSGTGTEDATKKEVPLGVAADANKLGTIA
LKPDPADGTADITLTFTMGGAGPKNKGKIITLTRTAADGLWKCTSDQDEQFIPKGCSR
>3C02A
MHMLFYKSYVREFIGEFLGTFVLMFLGEGATANFHTTGLSGDWYKLCLGWGLAVFFGILVSAKLS
GAHLNLAVSIGLSSINKFDLKKIPVYFFAQLLGAFVGTSTVYGLYHGFISNSKIPQFAWETSRNP
SISLTGAFFNELILTGILLLVILVVVDENICGKFHILKLSSVVGLIILCIGITFGGNTGFALNPS
RDLGSRFLSLIAYGKDTFTKDNFYFWVPLVAPCVGSVVFCQFYDKVICPLVDLANNEKDGVDL
>2RHMA
GMQTPALIIVTGHPATGKTTLSQALATGLRLPLLSKDAFKEVMFDGLGWSDREWSRRVGATAIMM
LYHTAATILQSGQSLIMESNFRVDLDTERMQNLHTIAPFTPIQIRCVASGDVLVERILSRIAQGA
RHPGHCDDRSPADLELVRSRGDIPPLPLGGPLLTVDTTFPEQIDMNAIVQWVRQHLQSGTAFL
>4FA8A

QAVTAFLGERVTLTSYWRRVSLGPEIEVSWFKLGPGEEQVLIGRMHHDVIFIEWPFRGFFDIHRS
ANTFFLVVTAANISHDGNYLCRMKLGETEVTKQEHLSVVKPLTLSVHSERSQFPDFSVLTVTCTV
NAFPHPHVQWLMPEGVEPAPTAANGGVMKEKDGSLSVAVDLSLPKPWHLPVTCVGKNDKEEAHGV
YVSGYLSQ
>3E8OA
GMSPQSMLTSPQHPRRTTMVISHGTLSASAEHAAHLRQLLVHIAQATRQEDGCLLYLVSEDLSQP
GHFLITEHWDNLGAMHTHLALPGVTQAIDALKHLNVTDLKITAYEAGEAINIMG
>4IG1A
CGGRARVREYSRAELVIGTLCRVRVYSKRPAAEVHAALEEVFTLLQQQEMVLSANRDDSALAALN
AQAGSAPVVVDRSLYALLERALFFAEKSGGAFNPALGAVVKLWNIGFDRAAVPDPDALKEALTRC
DFRQVHLRAGVSVGAPHTVQLAQAGMQLDLGAIAKGFLADKIVQLLTAHALDSALVDLGGNIFAL
GLKYGDVRSAAAQRLEWNVGIRDPHGTGQKPALVVSVRDCSVVTSGAYERFFERDGVRYHHIIDP
VTGFPAHTDVDSVSIFAPRSTDADALATACFVLGYEKSCALLREFPGVDALFIFPDKRVRASAGI
VDRVRVLDARFVLER
>3PVTA
MRSTQEERFEQRIAQETAIEPQDWMPDAYRKTLIRQIGQHAHSEIVGMLPEGNWITRAPTLRRKA
ILLAKVQDEAGHGLYLYSAAETLGCAREDIYQKMLDGRMKYSSIFNYPTLSWADIGVIGWLVDGA
AIVNQVALCRTSYGPYARAMVKICKEESFHQRQGFEACMALAQGSEAQKQMLQDAINRFWWPALM
MFGPNDDNSPNSARSLTWKIKRFTNDELRQRFVDNTVPQVEMLGMTVPDPDLHFDTESGHYRFGE
IDWQEFNEVINGRGICNQERLDAKRKAWEEGTWVREAALAHAQKQHARKVA
>3SG0A
SNAMQQTKTLIVALATMLAGVTAAQAEIKIGITMSASGPGAALGQPQSKTVAALPKEIGGEKVTY
FALDDESDPTKAAQNARKLLSEEKVDVLIGSSLTPVSLPLIDIAAEAKTPLMTMAAAAILVAPMD
ERRKWVYKVVPNDDIMAEAIGKYIAKTGAKKVGYIGFSDAYGEGYYKVLAAAAPKLGFELTTHEV
YARSDASVTGQVLKIIATKPDAVFIASAGTPAVLPQKALRERGFKGAIYQTHGVATEEFIKLGGK
DVEGAIFAGEAFSGAEDMPADSPFRKVKARFVDAYKAANGGAAPTIFGVHLWDSMTLVENAIPAA
LKAAKPGTPEFRAAIRDQIEKSKDLALNNGLSNMTPDNHNGYDERSAFLIEIRDGAFRLKQ
>2BZ4A
MRGSHHHHHHGSMKRAVITGLGIVSSIGNNQQEVLASLREGRSGITFSQELKDSGMRSHVWGNVK
LDTTGLIDRKVVRFMSDASIYAFLSMEQAIADAGLSPEAYQNNPRVGLIAGSGGGSPRFQVFGAD
AMRGPRGLKAVGPYVVTKAMASGVSACLATPFKIHGVNYSISSACATSAHCIGNAVEQIQLGKQD
IVFAGGGEELCWEMACEFDAMGALSTKYNDTPEKASRTYDAHRDGFVIAGGGGMVVVEELEHALA
RGAHIYAEIVGYGATSDGADMVAPSGEGAVRCMKMAMHGVDTPIDYLNSQGTSTPVGDVKELAAI
REVFGDKSPAISATKAMTGHSLGAAGVQEAIYSLLMLEHGFIAPSINIEELDEQAAGLNIVTETT
DRELTTVMSNSFGFGGTNATLVMRKLKD
>2VVEA
SFQEQTTKSRDVNSFQIPLRDGVRELLPEDASRNRASIKSPVDIWIGGENMTALNGIVDGGRKFE
AGQEFQINTFGSVNYWVSDEEIRVFKEYSARAKYAQNEGRTALEANNVPFFDIDVPPELDGVPFS
LKARVRHKSKGVDGLGDYTSISVKPAFYITEGDETTDTLIKYTSYGSTGSHSGYDFDDNTLDVMV
TLSAGVHRVFPVETELDYDAVQEVQHDWYDESFTTFIEVYSDDPLLTVKGYAQILMERT
>3DVOA
PFTYSIEATRNLATTERCIQDIRNAPVRNRSTQFQLAQQNMLAYTFGEVIPGFASAGINGMDYRD
VIGRPVENAVTEGTHFFRDDFRVDSNAKAKVAGDIFEIVSSAVMWNCAARWNSLMVGEGWRSQPR
YSRPTLSPSPRRQVAVLNLPRSFDWVSLLVPESQEVIEEFRAGLRKDGLGLPTSTPDLAVVVLPE

EFQNDEMWREEIAGLTRPNQILLSGAYQRLQGRVQPGEISLAVAFKRSLRSDRLYQPLYEANVMQ
LLLEGKLGAPKVEFEVHTLAPEGTNAFVTYEAASLYGLAEGRSAVHRAIRELYVPPTAADLARRF
FAFLNERMELVNG
>4FP5D
GASQFFKDNCNRTTASLVEGVELTKYISDINNNTDGMYVVSSTGGVWRISRAKDYPDNVMTAEMR
KIAMAAVLAGMRVNMCASPASSPNVIWAIELEA
>1YPYA
GAAASIQTTVNTLSERISSKLEQEANASAQTKCDIEIGNFYIRQNHGCNLTVKNMCSADADAQLD
AVLSAATETYSGLTPEQKAYVPAMFTAALNIQTSVNTVVRDFENYVKQTCNSSAVVDNKLKIQNV
IIDECYGAPGSPTNLEFINTGSSKGNCAIKALMQLTTKATTQIAPKQVAGTGVQ
>2RBKA
MTKALFFDIDGTLVSFETHRIPSSTIEALEAAHAKGLKIFIATGRPKAIINNLSELQDRNLIDGY
ITMNGAYCFVGEEVIYKSAIPQEEVKAMAAFCEKKGVPCIFVEEHNISVCQPNEMVKKIFYDFLH
VNVIPTVSFEEASNKEVIQMTPFITEEEEKEVLPSIPTCEIGRWYPAFADVTAKGDTKQKGIDEI
IRHFGIKLEETMSFGDGGNDISMLRHAAIGVAMGQAKEDVKAAADYVTAPIDEDGISKAMKHFGI
I
>3ECFA
GMATEKYHEILKKYFLSFETGDFSQVQFSCNLEFLSPISGNTLKGTEEVIPFLKGVTTRVAEVNI
MSTTVEYPRASGVWQMRTTKGTLYTLHNFFRLDEEGIVYVWPMFDPKAVMENPDALIQWLTGKDY
>1XCLA
SSSAASPLFAPGEDCGPAWRAAPAAYDTSDTHLQILGKPVMERWETPYMHSLAAAAASRGGRVLE
VGFGMAIAASRVQQAPIKEHWIIECNDGVFQRLQNWALKQPHKVVPLKGLWEEVAPTLPDGHFDG
ILYDTYPLSEETWHTHQFNFIKTHAFRLLKPGGILTYCNLTSWGELMKSKYTDITAMFEETQVPA
LLEAGFQRENICTEVMALVPPADCRYYAFPQMITPLVTKH
>1K8KC
MAYHSFLVEPISCHAWNKDRTQIAICPNNHEVHIYEKSGNKWVQVHELKEHNGQVTGVDWAPDSN
RIVTCGTDRNAYVWTLKGRTWKPTLVILRINRAARCVRWAPNEKKFAVGSGSRVISICYFEQEND
WWVCKHIKKPIRSTVLSLDWHPNSVLLAAGSCDFKCRIFSAYIKEVEERPAPTPWGSKMPFGELM
FESSSSCGWVHGVCFSANGSRVAWVSHDSTVCLADADKKMAVATLASETLPLLAVTFITESSLVA
AGHDCFPVLFTYDSAAGKLSFGGRLDVPKQSSQRGLTARERFQNLDKKASSEGSAAAGAGLDSLH
KNSVSQISVLSGGKAKCSQFCTTGMDGGMSIWDVRSLESALKDLKIV
>1D4OA
MEISGTHTEINLDNAIDMIREANSIIITPGYGLCAAKAQYPIADLVKMLSEQGKKVRFGIHPVAG
RMPGQLNVLLAEAGVPYDIVLEMDEINHDFPDTDLVLVIGANDTVNSAAQEDPNSIIAGMPVLEV
WKSKQVIVMKRSLGVGYAAVDNPIFYKPNTAMLLGDAKKTCDALQAKVRESYQK
>2PNLA
GKFSRALKNRLESANYEEVELPPPSKGVIVPVVHTVKSAPGEAFGSLAIIIPGEYPELLDANQQV
LSHFANDTGSVWGIGEDIPFEGDNMCYTALPLKEIKRNGNIVVEKIFAGPIMGPSAQLGLSLLVN
DIEDGVPRMVFTGEIADDEETIIPICGVDIAAIAAHEQGLPLIGNQPGVDEEVRNTSLAAHLIQT
GTLPVQRA
>4AK4A
GKAFDDGVFTGIREINLSYNKETAIGDFQVVYDLNGSPYVGENHKSFITGFTPVKISLDFPSEYI
MEVSGYTGKVSGYVVVRSLTFKTNKKTYGPYGVTSGTPFSLPIENGLIVGFKGSIGYWLDYFSMY
LSL

>3PFBA
MGSSHHHHHHSSGRENLYFQGMATITLERDGLQLVGTREEPFGEIYDMAIIFHGFTANRNTSLLR
EIANSLRDENIASVRFDFNGHGDSDGKFENMTVLNEIEDANAILNYVKTDPHVRNIYLVGHAQGG
VVASMLAGLYPDLIKKVVLLAPAATLKGDALEGNTQGVTYNPDHIPDRLPFKDLTLGGFYLRIAQ
QLPIYEVSAQFTKPVCLIHGTDDTVVSPNASKKYDQIYQNSTLHLIEGADHCFSDSYQKNAVNLT
TDFLQNNNAF
>3P6DA
QQMGRGSMCDAFVGTWKLVSSENFDDYMKEVGVGFATRKVAGMAKPNMIISVNGDVITIKSESTF
KNTEISFILGQEFDEVTADDRKVKSTITLDGGVLVHVQKWDGKSTTIKRKREDDKLVVECVMKGV
TSTRVYERA
>4A02A
HGYVASPGSRAFFGSSAGGNLNTNVGRAQWEPQSIEAPKNTFITGKLASAGVSGFEPLDEQTATR
WHKTNITTGPLDITWNLTAQHRTASWDYYITKNGWNPNQPLDIKNFDKIASIDGKQEVPNKVVKQ
TINIPTDRKGYHVIYAVWGIGDTVNAFYQAIDVNIQ
>3TG2A
MAIPKIASYPLPVSLPTNKVDWRIDASRAVLLIHNMQEYFVHYFDSQAEPIPSLIKHIQQLKAHA
KQAGIPVVYTAQPANQDPAERALLSDFWGPGLSEETAIIAPLAPESGDVQLTKWRYSAFKKSPLL
DWLRETGRDQLIITGVYAHIGILSTALDAFMFDIQPFVIGDGVADFSLSDHEFSLRYISGRTGAV
KSTQQACLEIAAQHSKLTGLLEHHHHHH
>3C8OA
MHYVTPDLCDAYPELVQVVEPMFSNFGGRDSFGGEIVTIKCFEDNSLVKEQVDKDGKGKVLVVDG
GGSLRRALLGDMLAEKAAKNGWEGIVVYGCIRDVDVIAQTDLGVQALASHPLKTDKRGIGDLNVA
VTFGGVTFRPGEFVYADNNGIIVSPQALKMPE
>2BBDA
GEIYTETLQQTYAWTAGTNIPIKIPRNNFIRKIRVQLIGSISNSGTAAVTLPSAPFPYNLVQTFN
LSYEGSKTLYSVSGTGLGILMYYTTKGQNPAYPAPGTSVPASGSVNLNVMWEFDLARFPATMVQN
IILSILTGQAPSGVSINASFYITITYERVTAQEILSEGGLGADGEMPLATVLPKVIEIPTFNVPA
SSAPIHVAYLQPGQIYKRQLVYVINSTSGINNTDPTEYELKIVRGVPTDKIKVSWAALQAENQAE
YQVAPYSGASAIIDFRKYFNGDLDLTHAPSDSIEYDLALQNQDNVYSLYVSYVLPYYDQLAALPA
QVAAIVQQYVARQKRRIKRHHHHHH
>2G40A
MGSDKIHHHHHHMTTIPSTAEAKLEMLTTINRAIAGSRPEALPPYPVPAPLSRAEILHQFEDRIL
DYGAAYTHVSAAELPGAIAKALGNARRVIVPAGIPAPWLTVGMDVLRDEPPLSHAELDRADAVLT
GCAVAISETGTIILDHRADQGRRALSLIPDFHICVVREDQIVQTVREGVEAVAASVREGRPLTWL
SGGSATSDIELVRVEGVHGPRRLQVIVVG
>3ONOA
SNAMKIALMMENSQAAKNAMVAGELNSVAGGLGHDVFNVGMTDENDHHLTYIHLGIMASILLNSK
AVDFVVTGCGTGQGALMSCNLHPGVVCGYCLEPSDAFLFNQINNGNAISLAFAKGFGWAGELNVR
YIFEKAFTGKRGEGYPIERAAPQQANAAILNNVKAAVAKDVVEGLRAIDQELVKTAVGSTQFQEC
FFAHCQVPEIAEYVKSLLD
>3E0EA
MNYKISELMPNLSGTINAEVVTAYPKKEFSRKDGTKGQLKSLFLKDDTGSIRGTLWNELADFEVK
KGDIAEVSGYVKQGYSGLEISVDNIGIIEKSL
>4DLHA

MSPIPLPVTDTDDAWRARIAAHRADKDEFLATHDQSPIPPADRGAFDGLRYFDIDASFRVAARYQ
PARDPEAVELETTRGPPAEYTRAAVLGFDLGDSHHTLTAFRVEGESSLFVPFTDETTDDGRTYEH
GRYLDVDPAGADGGDEVALDFNLAYNPFCAYGGSFSCALPPADNHVPAAITAGERVDADLEHHHH
HH
>2E2OA
MMIIVGVDAGGTKTKAVAYDCEGNFIGEGSSGPGNYHNVGLTRAIENIKEAVKIAAKGEADVVGM
GVAGLDSKFDWENFTPLASLIAPKVIIQHDGVIALFAETLGEPGVVVIAGTGSVVEGYNGKEFLR
VGGRGWLLSDDGSAYWVGRKALRKVLKMMDGLENKTILYNKVLKTINVKDLDELVMWSYTSSCQI
DLVASIAKAVDEAANEGDTVAMDILKQGAELLASQAVYLARKIGTNKVYLKGGMFRSNIYHKFFT
LYLEKEGIISDLGKRSPEIGAVILAYKEVGCDIKKLISD
>4JDUA
SNAVKRKGVEVMIALDISNSMLAQDVQPSRLEKAKRLISKLVDGMENDKVGMIVFAGDAFTQLPI
TSDYISAKMFLESISPSLISKQGTAIGAAINLAARSFTPQEGVGRAIVVITDGENHEGGAVAAAK
SSSSSGIQVNVLGVGLPDGAPIPIEGSNDFRRDREGNVIVTRLNEAMCQEIAKEGNGIYIRVDNS
NSAQKAINQEINKMAKSDVE
>1P3DA
MKHSHEEIRKIIPEMRRVQQIHFIGIGGAGMSGIAEILLNEGYQISGSDIADGVVTQRLAQAGAK
IYIGHAEEHIEGASVVVVSSAIKDDNPELVTSKQKRIPVIQRAQMLAEIMRFRHGIAVAGTHGKT
TTTAMISMIYTQAKLDPTFVNGGLVKSAGKNAHLGASRYLIAEADESDASFLHLQPMVSVVTNME
PDHMDTYEGDFEKMKATYVKFLHNLPFYGLAVMCADDPVLMELVPKVGRQVITYGFSEQADYRIE
DYEQTGFQGHYTVICPNNERINVLLNVPGKHNALNATAALAVAKEEGIANEAILEALADFQGAGR
RFDQLGEFIRPNGKVRLVDDYGHHPTEVGVTIKAAREGWGDKRIVMIFQPHRYSRTRDLFDDFVQ
VLSQVDALIMLDVYAAGEAPIVGADSKSLCRSIRNLGKVDPILVSDTSQLGDVLDQIIQDGDLIL
AQGAGSVSKISRGLAESWKN
>3NE5A
HHHHHHMGIEWIIRRSVANRFLVLMGALFLSIWGTWTIINTPVDALPDLSDVQVIIKTSYPGQAP
QIVENQVTYPLTTTMLSVPGAKTVRGFSQFGDSYVYVIFEDGTDPYWARSRVLEYLNQVQGKLPA
GVSAELGPDATGVGWIYEYALVDRSGKHDLADLRSLQDWFLKYELKTIPDVAEVASVGGVVKEYQ
VVIDPQRLAQYGISLAEVKSALDASNQEAGGSSIELAEAEYMVRASGYLQTLDDFNHIVLKASEN
GVPVYLRDVAKVQIGPEMRRGIAELNGEGEVAGGVVILRSGKNAREVIAAVKDKLETLKSSLPEG
VEIVTTYDRSQLIDRAIDNLSGKLLEEFIVVAVVCALFLWHVRSALVAIISLPLGLCIAFIVMHF
QGLNANIMSLGGIAIAVGAMVDAAIVMIENAHKRLEEWQHQHPDATLDNKTRWQVITDASVEVGP
ALFISLLIITLSFIPIFTLEGQEGRLFGPLAFTKTYAMAGAALLAIVVIPILMGYWIRGKIPPES
SNPLNRFLIRVYHPLLLKVLHWPKTTLLVAALSVLTVLWPLNKVGGEFLPQINEGDLLYMPSTLP
GISAAEAASMLQKTDKLIMSVPEVARVFGKTGKAETATDSAPLEMVETTIQLKPQEQWRPGMTMD
KIIEELDNTVRLPGLANLWVPPIRNRIDMLSTGIKSPIGIKVSGTVLADIDAMAEQIEEVARTVP
GVASALAERLEGGRYINVEINREKAARYGMTVADVQLFVTSAVGGAMVGETVEGIARYPINLRYP
QSWRDSPQALRQLPILTPMKQQITLADVADIKVSTGPSMLKTENARPTSWIYIDARDRDMVSVVH
DLQKAIAEKVQLKPGTSVAFSGQFELLERANHKLKLMVPMTLMIIFVLLYLAFRRVGEALLIISS
VPFALVGGIWLLWWMGFHLSVATGTGFIALAGVAAEFGVVMLMYLRHAIEAVPSLNNPQTFSEQK
LDEALYHGAVLRVRPKAMTVAVIIAGLLPILWGTGAGSEVMSRIAAPMIGGMITAPLLSLFIIPA
AYKLMWLHRHRVRK
>7A3HA
DNDSVVEEHGQLSISNGELVNERGEQVQLKGMSSHGLQWYGQFVNYESMKWLRDDWGINVFRAAM

YTSSGGYIDDPSVKEKVKEAVEAAIDLDIYVIIDWHILSDNDPNIYKEEAKDFFDEMSELYGDYP
NVIYEIANEPNGSDVTWGNQIKPYAEEVIPIIRNNDPNNIIIVGTGTWSQDVHHAADNQLADPNV
MYAFHFYAGTHGQNLRDQVDYALDQGAAIFVSEWGTSAATGDGGVFLDEAQVWIDFMDERNLSWA
NWSLTHKDESSAALMPGANPTGGWTEAELSPSGTFVREKIRES
>2XGFA
TGNITGGSGNFANLNSTIESLKTDIMSSYPIGAPIPWPSDSVPAGFALMEGQTFDKSAYPKLAVA
YPSGVIPDMRGQTIKGKPSGRAVLSAEADGVKAHSHSASASSTDLGTKTTSSFDYGTKGTNSTGG
HTHSGSGSTSTNGEHSHYIEAWNGTGVGGNKMSSYAISYRAGGSNTNAAGNHSHTFSFGTSSAGD
HSHSVGIGAHTHTVAIGSHGHTITVNSTGNTENTVKNIAFNYIVRLA
>3K25A
MYNISFTPDRPLTYHLEDDQSLARLSLVPGRGGLVTEWTVQGQPILYFDRERFQDPSLSVRGGIP
ILFPICGNLPQDQFNHAGKSYRLKQHGFARDLPWEVIGQQTQDNARLDLRLSHNDATLEAFPFAF
ELVFSYQLQGHSLRIEQRIANLGDQRMPFSLGFHPYFFCREKLGITLAIPANDYLDQKTGDCHGY
DGQLNLTSPELDLAFTQISQPRAHFIDPDRNLKIEVSFSELYQTLVLWTVAGKDYLCLEPWSGPR
NALNSGEQLAWVEPYSSRSAWVNFQVSTE
>3TIJA
GPAVPRMSLFMSLIGMAVLLGIAVLLSSNRKAINLRTVGGAFAIQFSLGAFILYVPWGQELLRGF
SDAVSNVINYGNDGTSFLFGGLVSGKMFEVFGGGGFIFAFRVLPTLIFFSALISVLYYLGVMQWV
IRILGGGLQKALGTSRAESMSAAANIFVGQTEAPLVVRPFVPKMTQSELFAVMCGGLASIAGGVL
AGYASMGVKIEYLVAASFMAAPGGLLFAKLMMPETEKPQDNEDITLDGGDDKPANVIDAAAGGAS
AGLQLALNVGAMLIAFIGLIALINGMLGGIGGWFGMPELKLEMLLGWLFAPLAFLIGVPWNEATV
AGEFIGLKTVANEFVAYSQFAPYLTEAAPVVLSEKTKAIISFALCGFANLSSIAILLGGLGSLAP
KRRGDIARMGVKAVIAGTLSNLMAATIAGFFLSF
>3EB8A
GSIYSPHETLAEKHSEKKLMDSFSPSLSQDKMDGEFAHANIDGISIRLCLNKGICSVFYLDGDKI
QSTQLSSKEYNNLLSSLPPKQFNLGKVHTITAPVSGNFKTHKPAPEVIETAINCCTSIIPNDDYF
HVKDTDFNSVWHDIYRDIRASDSNSTKIYFNNIEIPLKLIADLINELGINEFIDSKKELQMLSYN
QVNKIINSNFPQQDLCFQTEKLLFTSLFQDPAFISALTSAFWQSLHITSSSVEHIYAQIMSENIE
NRLNFMPEQRVINNCGHIIKINAVVPKNDTAISASGGRAYEVSSSILPSHITCNGVGINKIETSY
LVHAGTLPSSEGLRNAIPPESRQVSFAIISPDV
>3BONA
MQFVNKQFNYKDPVNGVDIAYIKIPNVGQMQPVKAFKIHNKIWVIPERDTFTNPEEGDLNPPPEA
KQVPVSYYDSTYLSTDNEKDNYLKGVTKLFERIYSTDLGRMLLTSIVRGIPFWGGSTIDTELKVI
DTNCINVIQPDGSYRSEELNLVIIGPSADIIQFECKSFGHEVLNLTRNGYGSTQYIRFSPDFTFG
FEESLEVDTNPLLGAGKFATDPAVTLAHELIHAGHRLYGIAINPNRVFKVNTNAYYEMSGLEVSF
EELRTFGGHDAKFIDSLQENEFRLYYYNKFKDIASTLNKAKSIVGTTASLQYMKNVFKEKYLLSE
DTSGKFSVDKLKFDKLYKMLTEIYTEDNFVKFFKVLNRKTYLNFDKAVFKINIVPKVNYTIYDGF
NLRNTNLAANFNGQNTEINNMNFTKLKNFTGLFEF
>2MBRA
HSLKPWNTFGIDHNAQHIVCAEDEQQLLNAWQYATAEGQPVLILGEGSNVLFLEDYRGTVIINRI
KGIEIHDEPDAWYLHVGAGENWHRLVKYTLQEGMPGLENLALIPGCVGSSPIQNIGAYGVELQRV
CAYVDSVELATGKQVRLTAKECRFGYRDSIFKHEYQDRFAIVAVGLRLPKEWQPVLTYGDLTRLD
PTTVTPQQVFNAVCHMRTTKLPDPKVNGNAGSFFKNPVVSAETAKALLSQFPTAPNYPQADGSVK
LAAGWLIDQCQLKGMQIGGAAVHRQQALVLINEDNAKSEDVVQLAHHVRQKVGEKFNVWLEPEVR

FIGASGEVSAVETIS
>3V0RA
MDTASCPVTTEGDYVWKISEFYGRKPEGTYYNSLGFNIKATNGGTLDFTCSAQADKLEDHKWYSC
GENSFMDFSFDSDRSGLLLKQKVSDDITYVATATLPNYCRAGGNGPKDFVCQGVADAYITLVTLP
KSS
>1SDIA
GAKNYYDITLALAGICQSARLVQQLAHQGHCDADALHVSLNSIIDMNPSSTLAVFGGSEANLRVG
LETLLGVLNASSRQGLNAELTRYTLSLMVLERKLSSAKGALDTLGNRINGLQRQLEHFDLQSETL
MSAMAAIYVDVISPLGPRIQVTGSPAVLQSPQVQAKVRATLLAGIRAAVLWHQVGGGRLQLMFSR
NRLTTQAKQILAHLTPEL
>3BCYA
GSHMVQTVTTEDGETVKVFEDLQGFETFIANETEDDDFDHLHCKLNYYPPFVLHESHEDPEKISD
AANSHSKKFVRHLHQHIEKHLLKDIKQAVRKPELKFHEKSKEETFDKITWHYGEETEYHGRPFKI
DVQVVCTHEDAMVFVDYKTHPVGAN
>3JQ1A
GTEDTFWKDETDFNLALTSCYTPLKNALNGGYYGTRGVMLRIARADEVDFRNDISDVYTVNRFTN
SNTNSLTQGMFYQFYNALYRTNSIMQKLEEKKEQFSTDFQNSVKGECLFIRGFYLFQLAKEFKDA
PLRLTASQSPSTFPLAKSSQADIWAQAKEDLKTAASLLPITNKIGKPTQGAAYAALGKIYVYEEN
WQEAINVLEPLTQNPYTYKLVEDFNWNFDDTHENNAESIFELLIEDVGGTDLWGDGENINSTQSN
TRPKEYAAAEVGGWYEANPTQQIMDIFWKEKDKDGNFDYRARCSVAWDYEGCTYYQRPFREVFAQ
DKWKTYWILKYQNWKTQKDEPAPPKSFINERAIRYADVLLMLAEAYMNKGALDTSIGYINQIRRR
ANLNDYSGPITKEGVFEDLVHQRAIEFFVEGERFYDLRRWGLLEQTLKTCDDTRYKNYQTGKSDN
INKFNYFPIPAKELDTNPLCTPSEGW
>4JHDC
MAHHHHHHVQRPLPKDVSLHSALMEAIHSSGGREKLRKVAEQTSEGRPKKPSYVEAESERSALLA
AIRGHSGTLSLRKVSSLASEELQSFRNAALGAPGLDKPQQEDLGLPPPPALPPTPAPAPQAPSAS
VTVSRFSTGTPSNSVNARQALMDAIRSGTGAARLRKVPLLV
>3M1XA
MAHHHHHHMGTLEAGQQGPGSMSKLTVVASPLAPEAVGAYSQAIICNGMVYCSGQIGLDRKTGDF
AGKTIEEQSKQVMTNLKYVLEEAGSSMDKVVKTTCLLADIKDFGVFNGIYAEAFGNHKPARACFA
AAALPKGALVEVECIATL
>3I7AA
GMSNEHQLLVGLLKKLKDDALILPTLPEVAMRVQEVVGRPDSSLKQVAEIIGQDAAISARIIKVA
NSALYSRGVPAENINSAVTRIGLTQIKSIATSVAMEQLFISTNEMVWEVMDEVWRTSIDVTAAAC
SLLQIYNKKHPGSGLNYDTLTLAGLVHNIGALPVLTEAEAHPEMFTTIEHLRSLVRKMQGPIGRA
VLKSWDFAPEVMEVVERWADLPYLGDHVSYLDFIRAAAFYTGELRAGNELEQRLDVFVKRGLPVS
PEDLGSDAFLDSYHSIKASYE
>3ZUXA
MVAASMNILSKISSFIGKTFSLWAALFAAAAFFAPDTFKWAGPYIPWLLGIIMFGMGLTLKPSDF
DILFKHPKVVIIGVIAQFAIMPATAWCLSKLLNLPAEIAVGVILVGCCPGGTASNVMTYLARGNV
ALSVAVTSVSTLTSPLLTPAIFLMLAGEMLEIQAAGMLMSIVKMVLLPIVLGLIVHKVLGSKTEK
LTDALPLVSVAAIVLIIGAVVGASKGKIMESGLLIFAVVVLHNGIGYLLGFFAAKWTGLPYDAQK
ALTIEVGMQNSGLAAALAAAHFAAAPVVAVPGALFSVWHNISGSLLATYWAAKAGKHKKPLDRAG
SENLYFQ

>1GND-_
MDEEYDVIVLGTGLTECILSGIMSVNGKKVLHMDRNPYYGGESSSITPLEELYKRFQLLEGPPET
MGRGRDWNVDLIPKFLMANGQLVKMLLYTEVTRYLDFKVVEGSFVYKGGKIYKVPSTETEALASN
LMGMFEKRRFRKFLVFVANFDENDPKTFEGVDPQNTSMRDVYRKFDLGQDVIDFTGHALALYRTD
DYLDQPCLETINRIKLYSESLARYGKSPYLYPLYGLGELPQGFARLSAIYGGTYMLNKPVDDIIM
ENGKVVGVKSEGEVARCKQLICDPSYVPDRVRKAGQVIRIICILSHPIKNTNDANSCQIIIPQNQ
VNRKSDIYVCMISYAHNVAAQGKYIAIASTTVETTDPEKEVEPALELLEPIDQKFVAISDLYEPI
DDGSESQVFCSCSYDATTHFETTCNDIKDIYKRMAGSAFDFENMKRKQNDVFGEADQ
>1PHP-_
MNKKTIRDVDVRGKRVFCRVDFNVPMEQGAITDDTRIRAALPTIRYLIEHGAKVILASHLGRPKG
KVVEELRLDAVAKRLGELLERPVAKTNEAVGDEVKAAVDRLNEGDVLLLENVRFYPGEEKNDPEL
AKAFAELADLYVNDAFGAAHRAHASTEGIAHYLPAVAGFLMEKELEVLGKALSNPDRPFTAIIGG
AKVKDKIGVIDNLLEKVDNLIIGGGLAYTFVKALGHDVGKSLLEEDKIELAKSFMEKAKEKGVRF
YMPVDVVVADRFANDANTKVVPIDAIPADWSALDIGPKTRELYRDVIRESKLVVWNGPMGVFEMD
AFAHGTKAIAEALAEALDTYSVIGGGDSAAAVEKFGLADKMDHISTGGGASLEFMEGKQLPGVVA
LEDK
>1LKI-_
SPLPITPVNATCAIRHPCHGNLMNQIKNQLAQLNGSANALFISYYTAQGEPFPNNVEKLCAPNMT
DFPSFHGNGTEKTKLVELYRMVAYLSASLTNITRDQKVLNPTAVSLQVKLNATIDVMRGLLSNVL
CRLCNKYRVGHVDVPPVPDHSDKEAFQRKKLGCQLLGTYKQVISVVVQAF
>1MRP-_
DITVYNGQHKEAATAVAKAFEQETGIKVTLNSGKSEQLAGQLKEEGDKTPADVFYTEQTATFADL
SEAGLLAPISEQTIQQTAQKGVPLAPKKDWIALSGRSRVVVYDHTKLSEKDMEKSVLDYATPKWK
GKIGYVSTSGAFLEQVVALSKMKGDKVALNWLKGLKENGKLYAKNSVALQAVENGEVPAALINNY
YWYNLAKEKGVENLKSRLYFVRHQDPGALVSYSGAAVLKASKNQAEAQKFVDFLASKKGQEALVA
ARAEYPLRADVVSPFNLEPYEKLEAPVVSATTAQDKEHAIKLIEEAGLK
>1MAI-_
MHGLQDDPDLQALLKGSQLLKVKSSSWRRERFYKLQEDCKTIWQESRKVMRSPESQLFSIEDIQE
VRMGHRTEGLEKFARDIPEDRCFSIVFKDQRNTLDLIAPSPADAQHWVQGLRKIIHHSGSMDQRQ
K
>1A8E-_
DKTVRWCAVSEHEATKCQSFRDHMKSVIPSDGPSVACVKKASYLDCIRAIAANEADAVTLDAGLV
YDAYLAPNNLKPVVAEFYGSKEDPQTFYYAVAVVKKDSGFQMNQLRGKKSCHTGLGRSAGWNIPI
GLLYCDLPEPRKPLEKAVANFFSGSCAPCADGTDFPQLCQLCPGCGCSTLNQYFGYSGAFKCLKD
GAGDVAFVKHSTIFENLANKADRDQYELLCLDNTRKPVDEYKDCHLAQVPSHTVVARSMGGKEDL
IWELLNQAQEHFGKDKSKEFQLFSSPHGKDLLFKDSAHGFLKVPPRMDAKMYLGYEYVTAIRNLR
EGTC
>1CZJ-_
ETFEIPESVTMSPKQFEGYTPKKGDVTFNHASHMDIACQQCHHTVPDTYTIESCMTEGCHDNIKE
RTEISSVYRTFHTTKDSEKSCVGCHRELKRQGPSDAPLACNSCHVQ
>1A53-_
PRYLKGWLKDVVQLSLRRPSFRASRQRPIISLNERILEFNKRNITAIIAEYKRKSPSGLDVERDP
IEYSKFMERYAVGLSILTEEKYFNGSYETLRKIASSVSIPILMKDFIVKESQIDDAYNLGADTVL
LIVKILTERELESLLEYARSYGMEPLIEINDENDLDIALRIGARFIGINSRDLETLEINKENQRK

LISMIPSNVVKVAESGISERNEIEELRKLGVNAFLIGSSLMRNPEKIKEFIL
>1BR9-_
CSCSPVHPQQAFCNADVVIRAKAVSEKEVDSGNDIYGNPIKRIQYEIKQIKMFKGPEKDIEFIYT
APSSAVCGVSLDVGGKKEYLIAGKAEGDGKMHITLCDFIVPWDTLSTTQKKSLNHRYQMGCECKI
TRCPMIPCYISSPDECLWMDWVTEKNINGHQAKFFACIKRSDGSCAWYRGAAPPKQEFLDIEDP
>1FIT-_
MSFRFGQHLIKPSVVFLKTELSFALVNRKPVVPGHVLVCPLRPVERFHDLRPDEVADLFQTTQRV
GTVVEKHFHGTSLTFSXQDGPEAGQTVKHVHVHVLPRKAGDFHRNDSIYEELQKHDKEDFPASWR
SEEEXAAEAAALRVYFQ
>1BDB-_
MKLKGEAVLITGGASGLGRALVDRFVAEGAKVAVLDKSAERLAELETDHGDNVLGIVGDVRSLED
QKQAASRCVARFGKIDTLIPNAGIWDYSTALVDLPEESLDAAFDEVFHINVKGYIHAVKACLPAL
VASRGNVIFTISNAGFYPNGGGPLYTAAKHAIVGLVRELAFELAPYVRVNGVGSGGINSDLRGPS
SLGMGSKAISTVPLADMLKSVLPIGRMPEVEEYTGAYVFFATRGDAAPATGALLNYDGGLGVRGF
FSGAGGNDLLEQLNIHP
>1RCB-_
HKCDITLQEIIKTLNSLTEQKTLCTELTVTDIFAASKNTTEKETFCRAATVLRQFYSHHEKDTRC
LGATAQQFHRHKQLIRFLKRLDRNLWGLAGLNSCPVKEANQSTLENFLERLKTIMREKYSKCSS
>1COT-_
QDGDAAKGEKEFNKCKACHMIQAPDGTDIIKGGKTGPNLYGVVGRKIASEEGFKYGEGILEVAEK
NPDLTWTEADLIEYVTDPKPWLVKMTDDKGAKTKMTFKMGKNQADVVAFLAQNSPDAGGDGEAA
>1CSH-_
STNLKDVLASLIPKEQARIKTFRQQHGNTAVGQITVDMSYGGMRGMKGLIYETSVLDPDEGIRFR
GFSIPECQKLLPKAGGGEEPLPEGLFWLLVTGQIPTPEQVSWVSKEWAKRAALPSHVVTMLDNFP
TNLHPMSQLSAAITALNSESNFARAYAEGINRTKYWEFVYEDAMDLIAKLPCVAAKIYRNLYRAG
SSIGAIDSKLDWSHNFTNMLGYTDPQFTELMRLYLTIHSDHEGGNVSAHTSHLVGSALSDPYLSF
AAAMNGLAGPLHGLANQEVLLWLSQLQKDLGADASDEKLRDYIWNTLNSGRVVPGYGHAVLRKTD
PRYTCQREFALKHLPSDPMFKLVAQLYKIVPNVLLEQGKAKNPWPNVDAHSGVLLQYYGMTEMNY
YTVLFGVSRALGVLAQLIWSRALGFPLERPKSMSTAGLEKLSAGG
>1A8P-_
MSNLNVERVLSVHHWNDTLFSFKTTRNPSLRFENGQFVMIGLEVDGRPLMRAYSIASPNYEEHLE
FFSIKVQNGPLTSRLQHLKEGDELMVSRKPTGTLVTSDLLPGKHLYMLSTGTGLAPFMSLIQDPE
VYERFEKVVLIHGVRQVNELAYQQFITEHLPQSEYFGEAVKEKLIYYPTVTRESFHNQGRLTDLM
RSGKLFEDIGLPPINPQDDRAMICGSPSMLDESCEVLDGFGLKISPRMGEPGDYLIERAFVEK
>1BG2-_
MADLAECNIKVMCRFRPLNESEVNRGDKYIAKFQGEDTVVIASKPYAFDRVFQSSTSQEQVYNDC
AKKIVKDVLEGYNGTIFAYGQTSSGKTHTMEGKLHDPEGMGIIPRIVQDIFNYIYSMDENLEFHI
KVSYFEIYLDKIRDLLDVSKTNLSVHEDKNRVPYVKGCTERFVCSPDEVMDTIDEGKSNRHVAVT
NMNEHSSRSHSIFLINVKQENTQTEQKLSGKLYLVDLAGSEKVSKTGAEGAVLDEAKNINKSLSA
LGNVISALAEGSTYVPYRDSKMTRILQDSLGGNCRTTIVICCSPSSYNESETKSTLLFGQRAKTI
>1FMK-_
MVTTFVALYDYESRTETDLSFKKGERLQIVNNTEGDWWLAHSLSTGQTGYIPSNYVAPSDSIQAE
EWYFGKITRRESERLLLNAENPRGTFLVRESETTKGAYCLSVSDFDNAKGLNVKHYKIRKLDSGG
FYITSRTQFNSLQQLVAYYSKHADGLCHRLTTVCPTSKPQTQGLAKDAWEIPRESLRLEVKLGQG

CFGEVWMGTWNGTTRVAIKTLKPGTMSPEAFLQEAQVMKKLRHEKLVQLYAVVSEEPIYIVTEYM
SKGSLLDFLKGETGKYLRLPQLVDMAAQIASGMAYVERMNYVHRDLRAANILVGENLVCKVADFG
LARLIEDNEYTARQGAKFPIKWTAPEAALYGRFTIKSDVWSFGILLTELTTKGRVPYPGMVNREV
LDQVERGYRMPCPPECPESLHDLMCQCWRKEPEERPTFEYLQAFLEDYFTSTEPQXQPGENL
>1NDH-_
STPAITLENPDIKYPLRLIDKEVVNHDTRRFRFALPSPEHILGLPVGQHIYLSARIDGNLVIRPY
TPVSSDDDKGFVDLVIKVYFKDTHPKFPAGGKMSQYLESMKIGDTIEFRGPNGLLVYQGKGKFAI
RPDKKSSPVIKTVKSVGMIAGGTGITPMLQVIRAIMKDPDDHTVCHLLFANQTEKDILLRPELEE
LRNEHSARFKLWYTVDRAPEAWDYSQGFVNEEMIRDHLPPPEEEPLVLMCGPPPMIQYACLPNLE
RVGHPKERCFAF
>1CSN-_
MSGQNNVVGVHYKVGRRIGEGSFGVIFEGTNLLNNQQVAIKFEPRRSDAPQLRDEYRTYKLLAGC
TGIPNVYYFGQEGLHNVLVIDLLGPSLEDLLDLCGRKFSVKTVAMAAKQMLARVQSIHEKSLVYR
DIKPDNFLIGRPNSKNANMIYVVDFGMVKFYRDPVTKQHIPYREKKNLSGTARYMSINTHLGREQ
SRRDDLEALGHVFMYFLRGSLPWQGLKAATNKQKYERIGEKKQSTPLRELCAGFPEEFYKYMHYA
RNLAFDATPDYDYLQGLFSKVLERLNTTEDENFDWNLL
>1OYC-_
MSFVKDFKPQALGDTNLFKPIKIGNNELLHRAVIPPLTRMRALHPGNIPNRDWAVEYYTQRAQRP
GTMIITEGAFISPQAGGYDNAPGVWSEEQMVEWTKIFNAIHEKKSFVWVQLWVLGWAAFPDNLAR
DGLRYDSASDNVFMDAEQEAKAKKANNPQHSLTKDEIKQYIKEYVQAAKNSIAAGADGVEIHSAN
GYLLNQFLDPHSNTRTDEYGGSIENRARFTLEVVDALVEAIGHEKVGLRLSPYGVFNSMSGGAET
GIVAQYAYVAGELEKRAKAGKRLAFVHLVEPRVTNPFLTEGEGEYEGGSNDFVYSIWKGPVIRAG
NFALHPEVVREEVKDKRTLIGYGRFFISNPDLVDRLEKGLPLNKYDRDTFYQMSAHGYIDYPTYE
EALKLGWDKK
>1LFO-_
XMNFSGKYQVQSQENFEPFMKAMGLPEDLIQKGKDIKGVSEIVHEGKKVKLTITYGSKVIHNEFT
LGEEXELETMTGEKVKAVVKMEGDNKMVTTFKGIKSVTEFNGDTITNTMTLGDIVYKRVSKRI
>1CIY-_
IETGYTPIDISLSLTQFLLSEFVPGAGFVLGLVDIIWGIFGPSQWDAFLVQIEQLINQRIEEFAR
NQAISRLEGLSNLYQIYAESFREWEADPTNPALREEMRIQFNDMNSALTTAIPLLAVQNYQVPLL
SVYVQAANLHLSVLRDVSVFGQRWGFDAATINSRYNDLTRLIGNYTDYAVRWYNTGLERVWGPDS
RDWVRYNQFRRELTLTVLDIVALFSNYDSRRYPIRTVSQLTREIYTNPVLENFDGSFRGMAQRIE
QNIRQPHLMDILNSITIYTDVHRGFNYWSGHQITASPVGFSGPEFAFPLFGNAGNAAPPVLVSLT
GLGIFRTLSSPLYRRIILGSGPNNQELFVLDGTEFSFASLTTNLPSTIYRQRGTVDSLDVIPPQD
NSVPPRAGFSHRLSHVTMLSQAAGAVYTLRAPTFSWQHRSAEFNNIIPSSQITQIPLTKSTNLGS
GTSVVKGPGFTGGDILRRTSPGQISTLRVNITAPLSQRYRVRIRYASTTNLQFHTSIDGRPINQG
NFSATMSSGSNLQSGSFRTVGFTTPFNFSNGSSVFTLSAHVFNSGNEVYIDRIEFVPAEVTFEAE
YDLER
>1GKY-_
XSRPIVISGPSGTGKSTLLKKLFAEYPDSFGFSVSSTTRTPRAGEVNGKDYNFVSVDEFKSMIKN
NEFIEWAQFSGNYYGSTVASVKQVSKSGKTCILDIDMQGVKSVKAIPELNARFLFIAPPSVEDLK
KRLEGRGTETEESINKRLSAAQAELAYAETGAHDKVIVNDDLDKAYKELKDFIFAEK
>1AXN-_
SASIWVGHRGTVRDYPDFSPSVDAEAIQKAIRGIGTDEKMLISILTERSNAQRQLIVKEYQAAYG

KELKDDLKGDLSGHFEHLMVALVTPPAVFDAKQLKKSMKGAGTNEDALIEILTTRTSRQMKDISQ
AYYTVYKKSLGDDISSETSGDFRKALLTLADGRRDESLKVDEHLAKQDAQILYKAGENRWGTDED
KFTEILCLRSFPQLKLTFDEYRNISQKDIVDSIKGELSGHFEDLLLAIVNCVRNTPAFLAERLHR
ALKGIGTDEFTLNRIMVSRSEIDLLDIRTEFKKHYGYSLYSAIKSDTSGDYEITLLKICGGDD
>1FDS-_
ARTVVLITGCSSGIGLHLAVRLASDPSQSFKVYATLRDLKTQGRLWEAARALACPPGSLETLQLD
VRDSKSVAAARERVTEGRVDVLVCNAGLGLLGPLEALGEDAVASVLDVNVVGTVRMLQAFLPDMK
RRGSGRVLVTGSVGGLMGLPFNDVYCASKFALEGLCESLAVLLLPFGVHLSLIECGPVHTAFMEK
VLGSPEEVLDRTDIHTFHRFYQYLAHSKQVFREAAQNPEEVAEVFLTALRAPKPTLRYFTTERFL
PLLRMRLDDPSGSNYVTAMHREVFGDVPAKAEAGAEAGGGRGPGAEDEAGRSAVGDPELGDPPAA
PQ
>1DRW-_
MHDANIRVAIAGAGGRMGRQLIQAALALEGVQLGAALEREGSSLLGSDAGELAGAGKTGVTVQSS
LDAVKDDFDVFIDFTRPEGTLNHLAFCRQHGKGMVIGTTGFDEAGKQAIRDAAADIAIVFAANFS
VGVNVMLKLLEKAAKVMGDYTDIEIIEAHHRHKVDAPSGTALAMGEAIAHALDKDLKDCAVYSRE
GHTGERVPGTIGFATVRAGDIVGEHTAMFADIGERLEITHKASSRMTFANGAVRSALWLSGKESG
LFDMRDVLDLNNL
>1IDO-_
GCPQEIFADSDIAFLIDGSGSIIPHDFRRMKEFVSTVMEQLKKSKTLFSLMQYSEEFRIHFTFKE
FQNNPNPRSLVKPITQLLGRTHTATGIRKVVRELFNITNGARKNAFKILVVITDGEKFGDPLGYE
DVIPEADREGVIRYVIGVGDAFRSEKSRQELNTIASKPPRDHVFQVNNFEALKTIQNQLREK
>1FRB-_
ATFVELSTKAKMPIVGLGTWKSPPNQVKEAVKAAIDAGYRHIDCAYAYCNENEVGEAIQEKIKEK
AVQREDLFIVSKLWPTCFEKKLLKEAFQKTLTDLKLDYLDLYLIHWPQGLQPGKELFPKDDQGRI
LTSKTTFLEAWEGMEELVDQGLVKALGVSNFNHFQIERLLNKPGLKHKPVTNQVECHPYLTQEKL
IQYCHSKGISVTAYSPLGSPDRPSAKPEDPSLLEDPKIKEIAAKHEKTSAQVLIRFHIQRNVVVI
PKSVTPSRIQENIQVFDFQLSDEEMATILSFNRNWRACLLPETVNMEEYPYDAEY
>1LAM-_
TKGLVLGIYSKEKEEDEPQFTSAGENFNKLVSGKLREILNISGPPLKAGKTRTFYGLHEDFPSVV
VVGLGKKTAGIDEQENWHEGKENIRAAVAAGCRQIQDLEIPSVEVDPCGDAQAAAEGAVLGLYEY
DDLKQKRKVVVSAKLHGSEDQEAWQRGVLFASGQNLARRLMETPANEMTPTKFAEIVEENLKSAS
IKTDVFIRPKSWIEEQEMGSFLSVAKGSEEPPVFLEIHYKGSPNASEPPLVFVGKGITFDSGGIS
IKAAANMDLMRADMGGAATICSAIVSAAKLDLPINIVGLAPLCENMPSGKANKPGDVVRARNGKT
IQVDNTDAEGRLILADALCYAHTFNPKVIINAATLTGAMDIALGSGATGVFTNSSWLWNKLFEAS
IETGDRVWRMPLFEHYTRQVIDCQLADVNNIGKYRSAGACTAAAFLKEFVTHPKWAHLDIAGVMT
NKDEVPYLRKGMAGRPTRTLIEFLFRFSQ
>1CPT-_
MDARATIPEHIARTVILPQGYADDEVIYPAFKWLRDEQPLAMAHIEGYDPMWIATKHADVMQIGK
QPGLFSNAEGSEILYDQNNEAFMRSISGGCPHVIDSLTSMDPPTHTAYRGLTLNWFQPASIRKLE
ENIRRIAQASVQRLLDFDGECDFMTDCALYYPLHVVMTALGVPEDDEPLMLKLTQDFFGVEAARR
FHETIATFYDYFNGFTVDRRSCPKDDVMSLLANSKLDGNYIDDKYINAYYVAIATAGHDTTSSSS
GGAIIGLSRNPEQLALAKSDPALIPRLVDEAVRWTAPVKSFMRTALADTEVRGQNIKRGDRIMLS
YPSANRDEEVFSNPDEFDITRFPNRHLGFGWGAHMCLGQHLAKLEMKIFFEELLPKLKSVELSGP
PRLVATNFVGGPKNVPIRFTKA

>1HCZ-_
YPIFAQQNYENPREATGRIVCANCHLASKPVDIEVPQAVLPDTVFEAVVKIPYDMQLKQVLANGK
KGALNVGAVLILPEGFELAPPDRISPEMKEKIGNLSFQNYRPNKKNILVIGPVPGQKYSEITFPI
LAPDPATNKDVHFLKYPIYVGGNRGRGQIYPDGSKSNNTVYNATAGGIISKILRKEKGGYEITIV
DASNERQVIDIIPRGLELLVSEGESIKLDQPLTSNPNVGGFGQGDAEIVLQDPLRVQ
>1AYL-_
MRVNNGLTPQELEAYGISDVHDIVYNPSYDLLYQEELDPSLTGYERGVLTNLGAVAVDTGIFTGR
SPKDKYIVRDDTTRDTFWWADKGKGKNDNKPLSPETWQHLKGLVTRQLSGKRLFVVDAFCGANPD
TRLSVRFITEVAWQAHFVKNMFIRPSDEELAGFKPDFIVMNGAKCTNPQWKEQGLNSENFVAFNL
TERMQLIGGTWYGGEMKKGMFSMMNYLLPLKGIASMHCSANVGEKGDVAVFFGLSGTGKTTLSTD
PKRRLIGDDEHGWDDDGVFNFEGGCYAKTIKLSKEAEPEIYNAIRRDALLENVTVREDGTIDFDD
GSKTENTRVSYPIYHIDNIVKPVSKAGHATKVIFLTADAFGVLPPVSRLTADQTQYHFLSGFTAK
LAGTERGITEPTPTFSACFGAAFLSLHPTQYAEVLVKRMQAAGAQAYLVNTGWNGTGKRISIKDT
RAIIDAILNGSLDNAETFTLPMFNLAIPTELPGVDTKILDPRNTYASPEQWQEKAETLAKLFIDN
FDKYTDTPAGAALVAAGPKLS
>1A6O-A
MSKARVYADVNVLRPKEYWDYEALTVQWGEQDDYEVVRKVGRGKYSEVFEGINVNNNEKCIIKIL
KPVKKKKIKREIKILQNLCGGPNIVKLLDIVRDQHSKTPSLIFEYVNNTDFKVLYPTLTDYDIRY
YIYELLKALDYCHSQGIMHRDVKPHNVMIDHELRKLRLIDWGLAEFYHPGKEYNVRVASRYFKGP
ELLVDLQDYDYSLDMWSLGCMFAGMIFRKEPFFYGHDNHDQLVKIAKVLGTDGLNVYLNKYRIEL
DPQLEALVGRHSRKPWLKFMNADNQHLVSPEAIDFLDKLLRYDHQERLTALEAMTHPYFQQVRAA
ENSRTRA
>1GOX-_
XMEITNVNEYEAIAKQKLPKMVYDYYASGAEDQWTLAENRNAFSRILFRPRILIDVTNIDMTTTI
LGFKISMPIMIAPTAMQKMAHPEGEYATARAASAAGTIMTLSSWATSSVEEVASTGPGIRFFQLY
VYKDRNVVAQLVRRAERAGFKAIALTVDTPRLGRREADIKNRFVLPPFLTLKNFEGIDLGKMDKA
NDSGGLSSYVAGQIDRSLSWKDVAWLQTITSLPILVKGVITAEDARLAVQHGAAGIIVSNHGARQ
LDYVPATIMALEEVVKAAQGRIPVFLDGGVRRGTDVFKALALGAAGVFIGRPVVFSLAAEGEAGV
KKVLQMMRDEFELTMALSGCRSLKEISRSHIAADWDPSSRAVARL
>1OAA-_
ADGLGCAVCVLTGASRGFGRALAPQLARLLSPGSVMLVSARSESMLRQLKEELGAQQPDLKVVLA
AADLGTEAGVQRLLSAVRELPRPEGLQRLLLINNAATLGDVSKGFLNVNDLAEVNNYWALNLTSM
LCLTSGTLNAFQDSPGLSKTVVNISSLCALQPYKGWGLYCAGKAARDMLYQVLAAEEPSVRVLSY
APGPLDNDMQQLARETSKDPELRSKLQKLKSDGALVDCGTSAQKLLGLLQKDTFQSGAHVDFYD
>1A17-_
RDEPPADGALKRAEELKTQANDYFKAKDYENAIKFYSQAIELNPSNAIYYGNRSLAYLRTECYGY
ALGDATRAIELDKKYIKGYYRRAASNMALGKFRAALRDYETVVKVKPHDKDAKMKYQECNKIVKQ
KAFERAIAGDEHKRSVVDSLDIESMTIEDEYSGPKL
>1PBE-_
MKTQVAIIGAGPSGLLLGQLLHKAGIDNVILERQTPDYVLGRIRAGVLEQGMVDLLREAGVDRRM
ARDGLVHEGVEIAFAGQRRRIDLKRLSGGKTVTVYGQTEVTRDLMEAREACGATTVYQAAEVRLH
DLQGERPYVTFERDGERLRLDCDYIAGCDGFHGISRQSIPAERLKVFERVYPFGWLGLLADTPPV
SHELIYANHPRGFALCSQRSATRSRYYVQVPLTEKVEDWSDERFWTELKARLPAEVAEKLVTGPS
LEKSIAPLRSFVVEPMQHGRLFLAGDAAHIVPPTGAKGLNLAASDVSTLYRLLLKAYREGRGELL

ERYSAICLRRIWKAERFSWWMTSVLHRFPDTDAFSQRIQQTELEYYLGSEAGLATIAENYVGLPY
EEIE
>1SKF-_
VTKPTIAAVGGYAMNNGTGTTLYTKAADTRRSTGSTTKIMTAKVVLAQSNLNLDAKVTIQKAYSD
YVVANNASQAHLIVGDKVTVRQLLYGLMLPSGCDAAYALADKYGSGSTRAARVKSFIGKMNTAAT
NLGLHNTHFDSFDGIGNGANYSTPRDLTKIASSAMKNSTFRTVVKTKAYTAKTVTKTGSIRTMDT
WKNTNGLLSSYSGAIGVKTGSGPEAKYCLVFAATRGGKTVIGTVLASTSIPARESDATKIMNYGF
AL
>1AF7-_
SVLLQMTQRLALSDAHFRRICQLIYQRAGIVLADHKRDMVYNRLVRRLRALGLDDFGRYLSMLEA
NQNSAEWQAFINALTTNLTAFFREAHHFPILAEHARRRHGEYRVWSAAASTGEEPYSIAITLADA
LGMAPGRWKVFASDIDTEVLEKARSGIYRLSELKTLSPQQLQRYFMRGTGPHEGLVRVRQELANY
VEFSSVNLLEKQYNVPGPFDAIFCRNVMIYFDKTTQEDILRRFVPLLKPDGLLFAGHSENFSNLV
REFSLRGQTVYALS
>1AJ2-_
MKLFAQGTSLDLSHPHVMGILNVTPDSFSDGGTHNSLIDAVKHANLMINAGATIIDVGGESTRPG
AAEVSVEEELQRVIPVVEAIAQRFEVWISVDTSKPEVIRESAKVGAHIINDIRSLSEPGALEAAA
ETGLPVCLMHMQGNPKTMQEAPKYDDVFAEVNRYFIEQIARCEQAGIAKEKLLLDPGFGFGKNLS
HNYSLLARLAEFHHFNLPLLVGMSRKSMIGQLLNVGPSERLSGSLACAVIAAMQGAHIIRVHDVK
ETVEAMRVVEATLSAKENKRYE
>153L-_
RTDCYGNVNRIDTTGASCKTAKPEGLSYCGVSASKKIAERDLQAMDRYKTIIKKVGEKLCVEPAV
IAGIISRESHAGKVLKNGWGDRGNGFGLMQVDKRSHKPQGTWNGEVHITQGTTILINFIKTIQKK
FPSWTKDQQLKGGISAYNAGAGNVRSYARMDIGTTHDDYANDVVARAQYYKQHGY
>1A1X-_
GSAGEDVGAPPDHLWVHQEGIYRDEYQRTWVAVVEEETSFLRARVQQIQVPLGDAARPSHLLTSQ
LPLMWQLYPEERYMDNNSRLWQIQHHLMVRGVQELLLKLLPDD
>1A44-_
PVDLSKWSGPLSLQEVDERPQHPLQVKYGGAEVDELGKVLTPTQVKNRPTSITWDGLDPGKLYTL
VLTDPDAPSRKDPKYREWHHFLVVNMKGNNISSGTVLSDYVGSGPPKGTGLHRYVWLVYEQEGPL
KCDEPILSNRSGDHRGKFKVASFRKKYELGAPVAGTCYQAEWDDYVPKLYEQLSG
>1A48-_
XSITKTELDGILPLVARGKVRDIYEVDAGTLLFVATDRISAYDVIMENSIPEKGILLTKLSEFWF
KFLSNDVRNHLVDIAPGKTIFDYLPAKLSEPKYKTQLEDRSLLVHKHKLIPLEVIVRGYITGSAW
KEYVKTGTVHGLKQPQGLKESQEFPEPIFTPSTKAEQGEHDENISPAQAAELVGEDLSRRVAELA
VKLYSKCKDYAKEKGIIIADTKFEFGIDEKTNEIILVDEVLTPDSSRFWNGASYKVGESQDSYDK
QFLRDWLTANKLNGVNGVKMPQDIVDRTRAKYIEAYETLTGSKWSH
>1A6Q-_
MGAFLDKPKMEKHNAQGQGNGLRYGLSSMQGWRVEMEDAHTAVIGLPSGLESWSFFAVYDGHAGS
QVAKYCCEHLLDHITNNQDFKGSAGAPSVENVKNGIRTGFLEIDEHMRVMSEKKHGADRSGSTAV
GVLISPQHTYFINCGDSRGLLCRNRKVHFFTQDHKPSNPLEKERIQNAGGSVMIQRVNGSLAVSR
ALGDFDYKCVHGKGPTEQLVSPEPEVHDIERSEEDDQFIILACDGIWDVMGNEELCDFVRSRLEV
TDDLEKVCNEVVDTCLYKGSRDNMSVILICFPNAPKVSPEAVKKEAELDKYLECRVEEIIKKQGE
GVPDLVHVMRTLASENIPSLPPGGELASKRNVIEAVYNRLNPYKNDDTDSTSTDDMW

>1A8L-_
MGLISDADKKVIKEEFFSKMVNPVKLIVFVRKDHCQYCDQLKQLVQELSELTDKLSYEIVDFDTP
EGKELAKRYRIDRAPATTITQDGKDFGVRYFGLPAGHEFAAFLEDIVDVSREETNLMDETKQAIR
NIDQDVRILVFVTPTCPYCPLAVRMAHKFAIENTKAGKGKILGDMVEAIEYPEWADQYNVMAVPK
IVIQVNGEDRVEFEGAYPEKMFLEKLLSALS
>1A8Y-_
EEGLDFPEYDGVDRVINVNAKNYKNVFKKYEVLALLYHEPPEDDKASQRQFEMEELILELAAQVL
EDKGVGFGLVDSEKDAAVAKKLGLTEEDSIYVFKEDEVIEYDGEFSADTLVEFLLDVLEDPVELI
EGERELQAFENIEDEIKLIGYFKNKDSEHYKAFKEAAEEFHPYIPFFATFDSKVAKKLTLKLNEI
DFYEAFMEEPVTIPDKPNSEEEIVNFVEEHRRSTLRKLKPESMYETWEDDMDGIHIVAFAEEADP
DGYEFLEILKSVAQDNTDNPDLSIIWIDPDDFPLLVPYWEKTFDIDLSAPQIGVVNVTDADSVWM
EMDDEEDLPSAEELEDWLEDVLEGEINTEDDDDEDDDDDDDD
>1ABE-_
ENLKLGFLVKQPEEPWFQTEWKFADKAGKDLGFEVIKIAVPDGEKTLNAIDSLAASGAKGFVICT
PDPKLGSAIVAKARGYDMKVIAVDDQFVNAKGKPMDTVPLVMMAATKIGERQGQELYKEMQKRGW
DVKESAVMAITANELDTARRRTTGSMDALKAAGFPEKQIYQVPTKSNDIPGAFDAANSMLVQHPE
VKHWLIVGMNDSTVLGGVRATEGQGFKAADIIGIGINGVDAVSELSKAQATGFYGSLLPSPDVHG
YKSSEMLYNWVAKDVEPPKFTEVTDVVLITRDNFKEELEKKGLGGK
>1AC5-_
LPSSEEYKVAYELLPGLSEVPDPSNIPQMHAGHIPLRSEDADEQDSSDLEYFFWKFTNNDSNGNV
DRPLIIWLNGGPGCSSMDGALVESGPFRVNSDGKLYLNEGSWISKGDLLFIDQPTGTGFSVEQNK
DEGKIDKNKFDEDLEDVTKHFMDFLENYFKIFPEDLTRKIILSGESYAGQYIPFFANAILNHNKF
SKIDGDTYDLKALLIGNGWIDPNTQSLSYLPFAMEKKLIDESNPNFKHLTNAHENCQNLINSAST
DEAAHFSYQECENILNLLLSYTRESSQKGTADCLNMYNFNLKDSYPSCGMNWPKDISFVSKFFST
PGVIDSLHLDSDKIDHWKECTNSVGTKLSNPISKPSIHLLPGLLESGIEIVLFNGDKDLICNNKG
VLDTIDNLKWGGIKGFSDDAVSFDWIHKSKSTDDSEEFSGYVKYDRNLTFVSVYNASHMVPFDKS
LVSRGIVDIYSNDVMIIDNNGKNVMITT
>1AHO-_
VKDGYIVDDVNCTYFCGRNAYCNEECTKLKGESGYCQWASPYGNACYCYKLPDHVRTKGPGRCH
>1AIR-_
ATDTGGYAATAGGNVTGAVSKTATSMQDIVNIIDAARLDANGKKVKGGAYPLVITYTGNEDSLIN
AAAANICGQWSKDPRGVEIKEFTKGITIIGANGSSANFGIWIKKSSDVVVQNMRIGYLPGGAKDG
DMIRVDDSPNVWVDHNELFAANHECDGTPDNDTTFESAVDIKGASNTVTVSYNYIHGVKKVGLDG
SSSSDTGRNITYHHNYYNDVNARLPLQRGGLVHAYNNLYTNITGSGLNVRQNGQALIENNWFEKA
INPVTSRYDGKNFGTWVLKGNNITKPADFSTYSITWTADTKPYVNADSWTSTGTFPTVAYNYSPV
SAQCVKDKLPGYAGVGKNLATLTSTACK
>1AJJ-_
PCSAFEFHCLSGECIHSSWRCDGGPDCKDKSDEENCA
>1AL3-_
MKLQQLRYIVEVVNHNLNVSSTAEGLYTSQPGISKQVRMLEDELGIQIFARSGKHLTQVTPAGQE
IIRIAREVLSKVDAIKSVAGEHTWPDKGSLYVATTHTQARYALPGVIKGFIERYPRVSLHMHQGS
PTQIAEAVSKGNADFAIATEALHLYDDLVMLPCYHWNRSIVVTPEHPLATKGSVSIEELAQYPLV
TYTFGFTGRSELDTAFNRAGLTPRIVFTATDADVIKTYVRLGLGVGVIASMAVDPVSDPDLVKLD
ANGIFSHSTTKIGFRRSTFLRSYMYDFIQRFAPHLTRDVVDTAVALRSNEDIEAMFKDIKLPEK

>1ALU-_
MAPVPPGEDSKDVAAPHRQPLTSSERIDKQIRYILDGISALRKETCNKSNMCESSKEALAENNLN
LPKMAEKDGCFQSGFNEETCLVKIITGLLEFEVYLEYLQNRFESSEEQARAVQMSTKVLIQFLQK
KAKNLDAITTPDPTTNASLLTKLQAQNQWLQDMTTHLILRSFKEFLQSSLRALRQM
>1AMF-_
DEGKITVFAAASLTNAMQDIATQFKKEKGVDVVSSFASSSTLARQIEAGAPADLFISADQKWMDY
AVDKKAIDTATRQTLLGNSLVVVAPKASVQKDFTIDSKTNWTSLLNGGRLAVGDPEHVPAGIYAK
EALQKLGAWDTLSPKLAPAEDVRGALALVERNEAPLGIVYGSDAVASKGVKVVATFPEDSHKKVE
YPVAVVEGHNNATVKAFYDYLKGPQAAEIFKRYGFTIK
>1AMK-_
MSAKPQPIAAANWKCNGTTASIEKLVQVFNEHTISHDVQCVVAPTFVHIPLVQAKLRNPKYVISA
ENAIAKSGAFTGEVSMPILKDIGVHWVILGHSERRTYYGETDEIVAQKVSEACKQGFMVIACIGE
TLQQREANQTAKVVLSQTSAIAAKLTKDAWNQVVLAYEPVWAIGTGKVATPEQAQEVHLLLRKWV
SENIGTDVAAKLRILYGGSVNAANAATLYAKPDINGFLVGGASLKPEFRDIIDATR
>1AMM-_
GKITFYEDRGFQGHCYECSSDCPNLQPYFSRCNSIRVDSGCWMLYERPNYQGHQYFLRRGDYPDY
QQWMGFNDSIRSCRLIPQHTGTFRMRIYERDDFRGQMSEITDDCPSLQDRFHLTEVHSLNVLEGS
WVLYEMPSYRGRQYLLRPGEYRRYLDWGAMNAKVGSLRRVMDFY
>1AMP-_
MPPITQQATVTAWLPQVDASQITGTISSLESFTNRFYTTTSGAQASDWIASEWQALSASLPNASV
KQVSHSGYNQKSVVMTITGSEAPDEWIVIGGHLDSTIGSHTNEQSVAPGADDDASGIAAVTEVIR
VLSENNFQPKRSIAFMAYAAEEVGLRGSQDLANQYKSEGKNVVSALQLDMTNYKGSAQDVVFITD
YTDSNFTQYLTQLMDEYLPSLTYGFDTCGYACSDHASWHNAGYPAAMPFESKFNDYNPRIHTTQD
TLANSDPTGSHAKKFTQLGLAYAIEMGSATG
>1AMX-_
MRGSHHHHHHGSITSGNKSTNVTVHKSEAGTSSVFYYKTGDMLPEDTTHVRWFLNINNEKSYVSK
DITIKDQIQGGQQLDLSTLNINVTGTHSNYYSGQSAITDFEKAFPGSKITVDNTKNTIDVTIPQG
YGSYNSFSINYKTKITNEQQKEFVNNSQAWYQEHGKEEVNGKSFNHTVHN
>1AOA-_
EGICALGGTSELSSEGTQHSYSEEEKYAFVNWINKALENDPDCRHVIPMNPNTDDLFKAVGDGIV
LCKMINLSVPDTIDERAINKKKLTPFIIQENLNLALNSASAIGCHVVNIGAEDLRAGKPHLVLGL
LWQIIKIGLFADIELSRNEALAALLRDGETLEELMKLSPEELLLRWANFHLENSGWQKINNFSAD
IKDSKAYFHLLNQIAPKGQKEGEPRIDINMSGFNETDDLKRAESMLQQADKLGCRQFVTPADVVS
GNPKLNLAFVANLFN
>1AOL-_
QVYNITWEVTNGDRETVWAISGNHPLWTWWPVLTPDLCMLALSGPPHWGLEYQAPYSSPPGPPCC
SGSSGSSAGCSRDCDEPLTSLTPRCNTAWNRLKLDQVTHKSSEGFYVCPGSHRPREAKSCGGPDS
FYCASWGCETTGRVYWKPSSSWDYITVDNNLTTSQAVQVCKDNKWCNPLAIQFTNAGKQVTSWTT
GHYWGLRLYVSGRDPGLTFGIRLRYQNLGPRVP
>1AQP-_
KETAAAKFERQHMDSSTSAASSSNYCNQMMKSRNLTKDRCKPVNTFVHESLADVQAVCSQKNVAC
KNGQTNCYQSYSTMSITDCRETGSSKYPNCAYKTTQANKHIIVACEGNPYVPVHFDASV
>1ARB-_
GVSGSCNIDVVCPEGDGRRDIIRAVGAYSKSGTLACTGSLVNNTANDRKMYFLTAHHCGMGTAST

AASIVVYWNYQNSTCRAPNTPASGANGDGSMSQTQSGSTVKATYATSDFTLLELNNAANPAFNLF
WAGWDRRDQNYPGAIAIHHPNVAEKRISNSTSPTSFVAWGGGAGTTHLNVQWQPSGGVTEPGSSG
SPIYSPEKRVLGQLHGGPSSCSATGTNRSDQYGRVFTSWTGGGAAASRLSDWLDPASTGAQFIDG
LDSGGGTP
>1ARU-_
QGPGGGGGSVTCPGGQSTSNSQCCVWFDVLDDLQTNFYQGSKCESPVRKILRIVFHDAIGFSPAL
TAAGQFGGGGADGSIIAHSNIELAFPANGGLTDTIEALRAVGINHGVSFGDLIQFATAVGMSNCP
GSPRLEFLTGRSNSSQPSPPSLIPGPGNTVTAILDRMGDAGFSPDEVVDLLAAHSLASQEGLNSA
IFRSPLDSTPQVFDTQFYIETLLKGTTQPGPSLGFAEELSPFPGEFRMRSDALLARDSRTACRWQ
SMTSSNEVMGQRYRAAMAKMSVLGFDRNALTDCSDVIPSAVSNNAAPVIPGGLTVDDIEVSCPSE
PFPEIATASGPLPSLAPAP
>1ASH-_
ANKTRELCMKSLEHAKVDTSNEARQDGIDLYKHMFENYPPLRKYFKSREEYTAEDVQNDPFFAKQ
GQKILLACHVLCATYDDRETFNAYTRELLDRHARDHVHMPPEVWTDFWKLFEEYLGKKTTLDEPT
KQAWHEIGREFAKEINKHGR
>1AT0-_
CFTPESTALLESGVRKPLGELSIGDRVLSXTANGQAVYSEVILFXDRNLEQXQNFVQLHTDGGAV
LTVTPAHLVSVWQPESQKLTFVFADRIEEKNQVLVRDVETGELRPQRVVKVGSVRSKGVVAPLTR
EGTIVVNSVAASCYA
>1AUK-_
RPPNIVLIFADDLGYGDLGCYGHPSSTTPNLDQLAAGGLRFTDFYVPVSLXTPSRAALLTGRLPV
RMGMYPGVLVPSSRGGLPLEEVTVAEVLAARGYLTGMAGKWHLGVGPEGAFLPPHQGFHRFLGIP
YSHDQGPCQNLTCFPPATPCDGGCDQGLVPIPLLANLSVEAQPPWLPGLEARYMAFAHDLMADAQ
RQDRPFFLYYASHHTHYPQFSGQSFAERSGRGPFGDSLMELDAAVGTLMTAIGDLGLLEETLVIF
TADNGPETMRMSRGGCSGLLRCGKGTTYEGGVREPALAFWPGHIAPGVTHELASSLDLLPTLAAL
AGAPLPNVTLDGFDLSPLLLGTGKSPRQSLFFYPSYPDEVRGVFAVRTGKYKAHFFTQGSAHSDT
TADPACHASSSLTAHEPPLLYDLSKDPGENYNLLGGVAGATPEVLQALKQLQLLKAQLDAAVTFG
PSQVARGEDPALQICCHPGCTPRPACCHCPDPHA
>1AV4-_
MTPSTIQTASPFRLASAGEISEVQGILRTAGLLGPEKRIAYLGVLDPARGAGSEAEDRRFRVFIH
DVSGARPQEVTVSVTNGTVISAVELDTAATGELPVLEEEFEVVEQLLATDERWLKALAARNLDVS
KVRVAPLSAGVFEYAEERGRRILRGLAFVQDFPEDSAWAHPVDGLVAYVDVVSKEVTRVIDTGVF
PVPAEHGNYTDPELTGPLRTTQKPISITQPEGPSFTVTGGNHIEWEKWSLDVGFDVREGVVLHNI
AFRDGDRLRPIINRASIAEMVVPYGDPSPIRSWQNYFDTGEYLVGQYANSLELGCDCLGDITYLS
PVISDAFGNPREIRNGICMHEEDWGILAKHSDLWSGINYTRRNRRMVISFFTTIGNXDYGFYWYL
YLDGTIEFEAKATGVVFTSAFPEGGSDNISQLAPGLGAPFHQHIFSARLDMAIDGFTNRVEEEDV
VRQTMGPGNERGNAFSRKRTVLTRESEAVREADARTGRTWIISNPESKNRLNEPVGYKLHAHNQP
TLLADPGSSIARRAAFATKDLWVTRYADDERYPTGDFVNQHSGGAGLPSYIAQDRDIDGQDIVVW
HTFGLTHFPRVEDWPIMPVDTVGFKLRPEGFFDRSPVLDVPANPSQSGSHCHG
>1AZ9-A
SEISRQEFQRRRQALVEQMQPGSAALIFAAPEVTRSADSEYPYRQNSDFWYFTGFNEPEAVLVLI
KSDDTHNHSVLFNRVRDLTAEIWFGRRLGQDAAPEKLGVDRALAFSEINQQLYQLLNGLDVVYHA
QGEYAYADVIVNSALEKLRKGSRQNLTAPATMIDWRPVVHEMRLFKSPEEIAVLRRAGEITAMAH
TRAMEKCRPGMFEYHLEGEIHHEFNRHGARYPSYNTIVGSGENGCILHYTENEXEMRDGDLVLID

AGCEYKGYAGDITRTFPVNGKFTQAQREIYDIVLESLETSLRLYRPGTSILEVTGEVVRIMVSGL
VKLGILKGDVDELIAQNAHRPFFMHGLSHWLGLDVHDVGVYGQDRSRILEPGMVLTVEPGLYIAP
DAEVPEQYRGIGIRIEDDIVITETGNENLTASVVKKPEEIEALMVAARKQ
>1B5L-_
CYLSRKLMLDARENLKLLDRMNRLSPHSCLQDRKDFGLPQEMVEGDQLQKDQAFPVLYEMLQQSF
NLFYTEHSSAAWDTTLLEQLCTGLQQQLDHLDTCRGQVMGEEDSELGNMDPIVTVKKYFQGIYDY
LQEKGYSDCAWEIVRVEMMRALTVSTTLQKRLTKMGGDLNSP
>1B6A-_
MAGVEEVAASGSHLNGDLDPDDREEGAASTAEEAAKKKRRKKKKSKGPSAAGEQEPDKESGASVD
EVARQLERSALEDKERDEDDEDGDGDGDGATGKKKKKKKKKRGPKVQTDPPSVPICDLYPNGVFP
KGQECEYPPTQDGRTAAWRTTSEEKKALDQASEEIWNDFREAAEAHRQVRKYVMSWIKPGMTMIE
ICEKLEDCSRKLIKENGLNAGLAFPTGCSLNNCAAHYTPNAGDTTVLQYDDICKIDFGTHISGRI
IDCAFTVTFNPKYDTLLKAVKDATNTGIKCAGIDVRLCDVGEAIQEVMESYEVEIDGKTYQVKPI
RNLNGHSIGQYRIHAGKTVPIVKGGEATRMEEGEVYAIETFGSTGKGVVHDDMECSHYMKNFDVG
HVPIRLPRTKHLLNVINENFGTLAFCRRWLDRLGESKYLMALKNLCDLGIVDPYPPLCDIKGSYT
AQFEHTILLRPTCKEVVSRGDDY
>1BA3-_
MEDAKNIKKGPAPFYPLEDGTAGEQLHKAMKRYALVPGTIAFTDAHIEVNITYAEYFEMSVRLAE
AMKRYGLNTNHRIVVCSENSLQFFMPVLGALFIGVAVAPANDIYNERELLNSMNISQPTVVFVSK
KGLQKILNVQKKLPIIQKIIIMDSKTDYQGFQSMYTFVTSHLPPGFNEYDFVPESFDRDKTIALI
MNSSGSTGLPKGVALPHRTACVRFSHARDPIFGNQIIPDTAILSVVPFHHGFGMFTTLGYLICGF
RVVLMYRFEEELFLRSLQDYKIQSALLVPTLFSFFAKSTLIDKYDLSNLHEIASGGAPLSKEVGE
AVAKRFHLPGIRQGYGLTETTSAILITPEGDDKPGAVGKVVPFFEAKVVDLDTGKTLGVNQRGEL
CVRGPMIMSGYVNNPEATNALIDKDGWLHSGDIAYWDEDEHFFIVDRLKSLIKYKGYQVAPAELE
SILLQHPNIFDAGVAGLPDDDAGELPAAVVVLEHGKTMTEKEIVDYVASQVTTAKKLRGGVVFVD
EVPKGLTGKLDARKIREILIKAKKGGKSKL
>1BB9-_
MGSSHHHHHHSSGLVPRGSHMATVNGAVEGSTTTGRLDLPPGFMFKVQAQHDYTATDTDELQLKA
GDVVLVIPFQNPEEQDEGWLMGVKESDWNQHKELEKCRGVFPENFTERVQ
>1BDO-_
EISGHIVRSPMVGTFYRTPSPDAKAFIEVGQKVNVGDTLCIVEAMKMMNQIEADKSGTVKAILVE
SGQPVEFDEPLVVIE
>1BEA-_
SAGTSCVPGWAIPHNPLPSCRWYVTSRTCGIGPRLPWPELKRRCCRELADIPAYCRCTALSILMD
GAIPPGPDAQLEGRLEDLPGCPREVQRGFAATLVTEAECNLATISGVAECPWILGGGTMPSK
>1BFD-_
MASVHGTTYELLRRQGIDTVFGNPGSNELPFLKDFPEDFRYILALQEACVVGIADGYAQASRKPA
FINLHSAAGTGNAMGALSNAWNSHSPLIVTAGQQTRAMIGVEALLTNVDAANLPRPLVKWSYEPA
SAAEVPHAMSRAIHMASMAPQGPVYLSVPYDDWDKDADPQSHHLFDRHVSSSVRLNDQDLDILVK
ALNSASNPAIVLGPDVDAANANADCVMLAERLKAPVWVAPSAPRCPFPTRHPCFRGLMPAGIAAI
SQLLEGHDVVLVIGAPVFRYHQYDPGQYLKPGTRLISVTCDPLEAARAPMGDAIVADIGAMASAL
ANLVEESSRQLPTAAPEPAKVDQDAGRLHPETVFDTLNDMAPENAIYLNESTSTTAQMWQRLNMR
NPGSYYFCAAGGLGFALPAAIGVQLAEPERQVIAVIGDGSANYSISALWTAAQYNIPTIFVIMNN
GTYGALRWFAGVLEAENVPGLDVPGIDFRALAKGYGVQALKADNLEQLKGSLQEALSAKGPVLIE

VSTVSPVK
>1BG6-_
MIESKTYAVLGLGNGGHAFAAYLALKGQSVLAWDIDAQRIKEIQDRGAIIAEGPGLAGTAHPDLL
TSDIGLAVKDADVILIVVPAIHHASIAANIASYISEGQLIILNPGATGGALEFRKILRENGAPEV
TIGETSSMLFTCRSERPGQVTVNAIKGAMDFACLPAAKAGWALEQIGSVLPQYVAVENVLHTSLT
NVNAVMHPLPTLLNAARCESGTPFQYYLEGITPSVGSLAEKVDAERIAIAKAFDLNVPSVCEWYK
ESYGQSPATIYEAVQGNPAYRGIAGPINLNTRYFFEDVSTGLVPLSELGRAVNVPTPLIDAVLDL
ISSLIDTDFRKEGRTLEKLGLSGLTAAGIRSAVE
>1BGC-_
TPLGPARSLPQSFLLKCLEQVRKIQADGAELQERLCAAHKLCHPEELMLLRHSLGIPQAPLSSCS
SQSLQLRGCLNQLHGGLFLYQGLLQALAGISPELAPTLDTLQLDVTDFATNIWLQMEDLGAAPAV
QPTQGAMPTFTSAFQRRAGGVLVASQLHRFLELAYRGLRYLAEP
>1BHE-_
SDSRTVSEPKTPSSCTTLKADSSTATSTIQKALNNCDQGKAVRLSAGSTSVFLSGPLSLPSGVSL
LIDKGVTLRAVNNAKSFENAPSSCGVVDKNGKGCDAFITAVSTTNSGIYGPGTIDGQGGVKLQDK
KVSWWELAADAKVKKLKQNTPRLIQINKSKNFTLYNVSLINSPNFHVVFSDGDGFTAWKTTIKTP
STARNTDGIDPMSSKNITIAYSNIATGDDNVAIKAYKGRAETRNISILHNDFGTGHGMSIGSETM
GVYNVTVDDLKMNGTTNGLRIKSDKSAAGVVNGVRYSNVVMKNVAKPIVIDTVYEKKEGSNVPDW
SDITFKDVTSETKGVVVLNGENAKKPIEVTMKNVKLTSDSTWQIKNVNVKK
>1BHP-_
KSCCKSTLGRNCYNLCRARGAQKLCANVCRCKLTSGLSCPKDFPK
>1BK0-_
MGSVSKANVPKIDVSPLFGDDQAAKMRVAQQIDAASRDTGFFYAVNHGINVQRLSQKTKEFHMSI
TPEEKWDLAIRAYNKEHQDQVRAGYYLSIPGKKAVESFCYLNPNFTPDHPRIQAKTPTHEVNVWP
DETKHPGFQDFAEQYYWDVFGLSSALLKGYALALGKEENFFARHFKPDDTLASVVLIRYPYLDPY
PEAAIKTAADGTKLSFEWHEDVSLITVLYQSNVQNLQVETAAGYQDIEADDTGYLINCGSYMAHL
TNNYYKAPIHRVKWVNAERQSLPFFVNLGYDSVIDPFDPREPNGKSDREPLSYGDYLQNGLVSLI
NKNGQT
>1BOB-_
MSANDFKPETWTSSANEALRVSIVGENAVQFSPLFTYPIYGDSEKIYGYKDLIIHLAFDSVTFKP
YVNVKYSAKLGDDNIVDVEKKLLSFLPKDDVIVRDEAKWVDCFAEERKTHNLSDVFEKVSEYSLN
GEEFVVYKSSLVDDFARRMHRRVQIFSLLFIEAANYIDETDPSWQIYWLLNKKTKELIGFVTTYK
YWHYLGAKSFDEDIDKKFRAKISQFLIFPPYQNKGHGSCLYEAIIQSWLEDKSITEITVEDPNEA
FDDLRDRNDIQRLRKLGYDAVFQKHSDLSDEFLESSRKSLKLEERQFNRLVEMLLLLNNS
>1BPI-_
RPDFCLEPPYTGPCKARIIRYFYNAKAGLCQTFVYGGCRAKRNNFKSAEDCMRTCGGA
>1BQK-_
ADFEVHMLNKGKDGAMVFEPASLKVAPGDTVTFIPTDKGHNVETIKGMIPDGAEAFKSKINENYK
VTFTAPGVYGVKCTPHYGMGMVGVVQVGDAPANLEAVKGAKNPKKAQERLDAALAALGN
>1BS9-_
SCPAIHVFGARETTASPGYGSSSTVVNGVLSAYPGSTAEAINYPACGGQSSCGGASYSSSVAQGI
AAVASAVNSFNSQCPSTKIVLVGYSQGGEIMDVALCGGGDPNQGYTNTAVQLSSSAVNMVKAAIF
MGDPMFRAGLSYEVGTCAAGGFDQRPAGFSCPSAAKIKSYCDASDPYCCNGSNAATHQGYGSEYG
SQALAFVKSKLG

>1BTN-_
MEGFLNRKHEWEAHNKKASSRSWHNVYCVINNQEMGFYKDAKSAASGIPYHSEVPVSLKEAICEV
ALDYKKKKHVFKLRLSDGNEYLFQAKDDEEMNTWIQAISSA
>1BV1-_
GVFNYETETTSVIPAARLFKAFILDGDNLFPKVAPQAISSVENIEGNGGPGTIKKISFPEGLPFK
YVKDRVDEVDHTNFKYNYSVIEGGPIGDTLEKISNEIKIVATPDGGSILKISNKYHTKGDHEVKA
EQVKASKEMGETLLRAVESYLLAHSDAYN
>1BX7-_
TQGNTCGGETCSAAQVCLKGKCVCNEVHCRIRCKYGLKKDENGCEYPCSCAKASQ
>1C52-_
QADGAKIYAQCAGCHQQNGQGIPGAFPPLAGHVAEILAKEGGREYLILVLLYGLQGQIEVKGMKY
NGVMSSFAQLKDEEIAAVLNHIATAWGDAKKVKGFKPFTAEEVKKLRAKKLTPQQVLAERKKLGL
K
>1CA1-_
WDGKIDGTGTHAMIVTQGVSILENDLSKNEPESVRKNLEILKENMHELQLGSTYPDYDKNAYDLY
QDHFWDPDTDNNFSKDNSWYLAYSIPDTGESQIRKFSALARYEWQRGNYKQATFYLGEAMHYFGD
IDTPYHPANVTAVDSAGHVKFETFAEERKEQYKINTVGCKTNEDFYADILKNKDFNAWSKEYARG
FAKTGKSIYYSHASMSHSWDDWDYAAKVTLANSQKGTAGYIYRFLHDVSEGNDPSVGKNVKELVA
YISTSGEKDAGTDDYMYFGIKTKDGKTQEWEMDNPGNDFMTGSKDTYTFKLKDENLKIDDIQNMW
IRKRKYTAFPDAYKPENIKVIANGKVVVDKDINEWISGNSTYNIK
>1CEC-_
MVSFKAGINLGGWISQYQVFSKEHFDTFITEKDIETIAEAGFDHVRLPFDYPIIESDDNVGEYKE
DGLSYIDRCLEWCKKYNLGLVLDMHHAPGYRFQDFKTSTLFEDPNQQKRFVDIWRFLAKRYINER
EHIAFELLNEVVEPDSTRWNKLMLECIKAIREIDSTMWLYIGGNNYNSPDELKNLADIDDDYIVY
NFHFYNPFFFTHQKAHWSESAMAYNRTVKYPGQYEGIEEFVKNNPKYSFMMELNNLKLNKELLRK
DLKPAIEFREKKKCKLYCGEFGVIAIADLESRIKWHEDYISLLEEYDIGGAVWNYKKMDFEIYNE
DRKPVSQELVNILARRKT
>1CEM-_
AGVPFNTKYPYGPTSIADNQSEVTAMLKAEWEDWKSKRITSNGAGGYKRVQRDASTNYDTVSEGM
GYGLLLAVCFNEQALFDDLYRYVKSHFNGNGLMHWHIDANNNVTSHDGGDGAATDADEDIALALI
FADKQWGSSGAINYGQEARTLINNLYNHCVEHGSYVLKPGDRWGGSSVTNPSYFAPAWYKVYAQY
TGDTRWNQVADKCYQIVEEVKKYNNGTGLVPDWCTASGTPASGQSYDYKYDATRYGWRTAVDYSW
FGDQRAKANCDMLTKFFARDGAKGIVDGYTIQGSKISNNHNASFIGPVAAASMTGYDLNFAKELY
RETVAVKDSEYYGYYGNSLRLLTLLYITGNFPNPLSDL
>1CFB-_
IVQDVPNAPKLTGITCQADKAEIHWEQQGDNRSPILHYTIQFNTSFTPASWDAAYEKVPNTDSSF
VVQMSPWANYTFRVIAFNKIGASPPSAHSDSCTTQPDVPFKNPDNVVGQGTEPNNLVISWTPMPE
IEHNAPNFHYYVSWKRDIPAAAWENNNIFDWRQNNIVIADQPTFVKYLIKVVAINDRGESNVAAE
EVVGYSGEDR
>1CHD-_
LKAGPLLSSEKLIAIGASTGGTEAIRHVLQPLPLSSPAVIITQHMPPGFTRSFAERLNKLCQISV
KEAEDGERVLPGHAYIAPGDKHMELARSGANYQIKIHDGPPVNRHRPSVDVLFHSVAKHAGRNAV
GVILTGMGNDGAAGMLAMYQAGAWTIAQNEASCVVFGMPREAINMGGVSEVVDLSQVSQQMLAKI
SAGQAIRI

>1CLC-_
TMITNSRGSVDLQPSLTGVFPSGLIETKVSAAKITENYQFDSRIRLNSIGFIPNHSKKATIAANC
STFYVVKEDGTIVYTGTATSMFDNDTKETVYIADFSSVNEEGTYYLAVPGVGKSVNFKIAMNVYE
DAFKTAMLGMYLLRCGTSVSATYNGIHYSHGPCHTNDAYLDYINGQHTKKDSTKGWHDAGDYNKY
VVNAGITVGSMFLAWEHFKDQLEPVALEIPEKNNSIPDFLDELKYEIDWILTMQYPDGSGRVAHK
VSTRNFGGFIMPENEHDERFFVPWSSAATADFVAMTAMAARIFRPYDPQYAEKCINAAKVSYEFL
KNNPANVFANQSGFSTGEYATVSDADDRLWAAAEMWETLGDEEYLRDFENRAAQFSKKIEADFDW
DNVANLGMFTYLLSERPGKNPALVQSIKDSLLSTADSIVRTSQNHGYGRTLGTTYYWGCNGTVVR
QTMILQVANKISPNNDYVNAALDAISHVFGRNYYNRSYVTGLGINPPMNPHDRRSGADGIWEPWP
GYLVGGGWPGPKDWVDIQDSYQTNEIAINWNAALIYALAGFVNYNSPQNEVLYGDVNDDGKVNST
DLTLLKRYVLKAVSTLPSSKAEKNADVNRDGRVNSSDVTILSRYLIRVIEKLPI
>1CNV-_
DISSTEIAVYWGQREDGLLRDTCKTNNYKIVFISFLDKFGCEIRKPELELEGVCGPSVGNPCSFL
ESQIKECQRMGVKVFLALGGPKGTYSACSADYAKDLAEYLHTYFLSERREGPLGKVALDGIHFDI
QKPVDELNWDNLLEELYQIKDVYQSTFLLSAAPGCLSPDEYLDNAIQTRHFDYIFVRFYNDRSCQ
YSTGNIQRIRNAWLSWTKSVYPRDKNLFLELPASQATAPGGGYIPPSALIGQVLPYLPDLQTRYA
GIALWNRQADKETGYSTNIIRYLNATAMPFTSNLLKYPS
>1CPO-_
XEPGSGIGYPYDNNTLPYVAPGPTDSRAPCPALNALANHGYIPHDGRAISRETLQNAFLNHMGIA
NSVIELALTNAFVVCEYVTGSDCGDSLVNLTLLAEPHAFEHDHSFSRKDYKQGVANSNDFIDNRN
FDAETFQTSLDVVAGKTHFDYADMNEIRLQRESLSNELDFPGWFTESKPIQNVESGFIFALVSDF
NLPDNDENPLVRIDWWKYWFTNESFPYHLGWHPPSPAREIEFVTSASSAVLAASVTSTPSSLPSG
AIGPGAEAVPLSFASTMTPFLLATNAPYYAQDPTLGPND
>1CPQ-_
ADTKEVLEAREAYFKSLGGSMKAMTGVAKAFDAEAAKVEAAKLEKILATDVAPLFPAGTSSTDLP
GQTEAKAAIWANMDDFGAKGKAMHEAGGAVIAAANAGDGAAFGAALQKLGGTCKACHDDYREED
>1CTJ-_
EADLALGKAVFDGNCAACHAGGGNNVIPDHTLQKAAIEQFLDGGFNIEAIVYQIENGKGAMPAWD
GRLDEDEIAGVAAYVYDQAAGNKW
>1CTT-_
MHPRFQTAFAQLADNLQSALEPILADKYFPALLTGEQVSSLKSATGLDEDALAFALLPLAAACAR
TPLSNFNVGAIARGVSGTWYFGANMEFIGATMQQTVHAEQSAISHAWLSGEKALAAITVNYTPCG
HCRQFMNELNSGLDLRIHLPGREAHALRDYLPDAFGPKDLEIKTLLMDEQDHGYALTGDALSQAA
IAAANRSHMPYSKSPSGVALECKDGRIFSGSYAENAAFNPTLPPLQGALILLNLKGYDYPDIQRA
VLAEKADAPLIQWDATSATLKALGCHSIDRVLLA
>1CV8-_
YNEQYVNKLENFKIRETQGNNGWCAGYTMSALLNATYNTNKYHAEAVMRFLHPNLQGQQFQFTGL
TPREMIYFGQTQGRSPQLLNRMTTYNEVDNLTKNNKGIAILGSRVESRNGMHAGHAMAVVGNAKL
NNGQEVIIIWNPWDNGFMTQDAKNNVIPVSNGDHYQWYSSIYGY
>1CVL-_
ADTYAATRYPVILVHGLAGTDKFANVVDYWYGIQSDLQSHGAKVYYVANLSGFQSDDGPNGRGEQL
LAYVKQVLAATGATKVNLIGHSQGGLTSRYVAAVAPQLVASVTTIGTPHRGSEFADFVQDVLKTD
PTGLSSTVIAAFVNVFGTLVSSSHNTDQDALAALRTLTTAQTATYNRNFPSAGLGAPGSCQTGAA
TETVGGSQHLLYSWGGTAIQPTSTVLGVTGATDTSTGTLDVANVTDPSTLALLATGAVMINRASG

QNDGLVSRCSSLFGQVISTSYHWNHLDEINQLLGVRGANAEDPVAVIRTHVNRLKLQGV
>1CYO-_
SKAVKYYTLEEIQKHNNSKSTWLILHYKVYDLTKFLEEHPGGEEVLREQAGGDATENFEDVGHST
DARELSKTFIIGELHPDDRSKITKPSES
>1DDT-_
GADDVVDSSKSFVMENFSSYHGTKPGYVDSIQKGIQKPKSGTQGNYDDDWKGFYSTDNKYDAAGY
SVDNENPLSGKAGGVVKVTYPGLTKVLALKVDNAETIKKELGLSLTEPLMEQVGTEEFIKRFGDG
ASRVVLSLPFAEGSSSVEYINNWEQAKALSVELEINFETRGKRGQDAMYEYMAQACAGNRVRRSV
GSSLSCINLDWDVIRDKTKTKIESLKEHGPIKNKMSESPNKTVSEEKAKQYLEEFHQTALEHPEL
SELKTVTGTNPVFAGANYAAWAVNVAQVIDSETADNLEKTTAALSILPGIGSVMGIADGAVHHNT
EEIVAQSIALSSLMVAQAIPLVGELVDIGFAAYNFVESIINLFQVVHNSYNRPAYSPGHKTQPFL
HDGYAVSWNTVEDSIIRTGFQGESGHDIKITAENTPLPIAGVLLPTIPGKLDVNKSKTHISVNGR
KIRMRCRAIDGDVTFCRPKSPVYVGNGVHANLHVAFHRSSSEKIHSNEISSDSIGVLGYQKTVDH
TKVNSKLSLFFEIKS
>1DFX-_
PKHLEVYKCTHCGNIVEVLHGGGAELVCCGEPMKHMVEGSTDGAMEKHVPVIEKVDGGYLIKVGS
VPHPMEEKHWIEWIELLADGRSYTKFLKPGDAPEAFFAIDASKVTAREYCNLHGHWKAEN
>1DHN-_
MQDTIFLKGMRFYGYHGALSAENEIGQIFKVDVTLKVDLSEAGRTDNVIDTVHYGEVFEEVKSIM
EGKAVNLLEHLAERIANRINSQYNRVMETKVRITKENPPIPGHYDGVGIEIVRENK
>1DHR-_
MAASGEARRVLVYGGRGALGSRCVQAFRARNWWVASIDVVENEEASASVIVKMTDSFTEQADQVT
AEVGKLLGDQKVDAILCVAGGWAGGNAKSKSLFKNCDLMWKQSIWTSTISSHLATKHLKEGGLLT
LAGAKAALDGTPGMIGYGMAKGAVHQLCQSLAGKNSGMPSGAAAIAVLPVTLDTPMNRKSMPEAD
FSSWTPLEFLVETFHDWITGNKRPNSGSLIQVVTTDGKTELTPAYF
>1DIN-_
MLTEGISIQSYDGHTFGALVGSPAKAPAPVIVIAQEIFGVNAFMRETVSWLVDQGYAAVCPDLYA
RQAPGTALDPQDERQREQAYKLWQAFDMEAGVGDLEAAIRYARHQPYSNGKVGLVGYXLGGALAF
LVAAKGYVDRAVGYYGVGLEKQLNKVPEVKHPALFHMGGQDHFVPAPSRQLITEGFGANPLLQVH
WYEEAGHSFARTSSSGYVASAAALANERTLDFLAPLQSKKP
>1DOI-_
PTVEYLNYEVVDDNGWDMYDDDVFGEASDMDLDDEDYGSLEVNEGEYILEAAEAQGYDWPFSCRA
GACANCAAIVLEGDIDMDMQQILSDEEVEDKNVRLTCIGSPDADEVKIVYNAKHLDYLQNRVI
>1DUN-_
MLAYQGTQIKEKRDEDAGFDLCVPYDIMIPVSDTKIIPTDVKIQVPPNSFGWVTGKSSMAKQGLL
INGGIIDEGYTGEIQVICTNIGKSNIKLIEGQKFAQLIILQHHSNSRQPWDENKISQRGDKGFGS
TGVF
>1DXY-_
MKIIAYGARVDEIQYFKQWAKDTGNTLEYHTEFLDENTVEWAKGFDGINSLQTTPYAAGVFEKMH
AYGIKFLTIRNVGTDNIDMTAMKQYGIRLSNVPAYSPAAIAEFALTDTLYLLRNMGKVQAQLQAG
DYEKAGTFIGKELGQQTVGVMGTGHIGQVAIKLFKGFGAKVIAYDPYPMKGDHPDFDYVSLEDLF
KQSDVIDLHVPGIEQNTHIINEAAFNLMKPGAIVINTARPNLIDTQAMLSNLKSGKLAGVGIDTY
EYETEDLLNLAKHGSFKDPLWDELLGMPNVVLSPHIAYYTETAVHNMVYFSLQHLVDFLTKGETS
TEVTGPAK

>1EAF-_
IPPIPPVDFAKYGEIEEVPMTRLMQIGATNLHRSWLNVPHVTQFESADITELEAFRVAQKAVAKK
AGVKLTVLPLLLKACAYLLKELPDFNSSLAPSGQALIRKKYVHIGFAVDTPDGLLVPVIRNVDQK
SLLQLAAEAAELAEKARSKKLGADAMQGACFTISSLGHIGGTAFTPIVNAPEVAILGVSKASMQP
VWDGKAFQPRLMLPLSLSYDHRVINGAAAARFTKRLGDLLADIRAILL
>1ECY-_
AESVQPLEKIAPYPQAEKGMKRQVIQLTPQEDESTLKVELLIGQTLEVDCNLHRLGGKLENKTLE
GWGYDYYVFDKVSSPVSTMMACPDGKKEKKFVTAYLGDAGMLRYNSKLPIVVYTPDNVDVKYRVW
KAEEKIDNAVVR
>1EDG-_
MYDASLIPNLQIPQKNIPNNDGMNFVKGLRLGWNLGNTFDAFNGTNITNELDYETSWSGIKTTKQ
MIDAIKQKGFNTVRIPVSWHPHVSGSDYKISDVWMNRVQEVVNYCIDNKMYVILNTHHDVDKVKG
YFPSSQYMASSKKYITSVWAQIAARFANYDEHLIFEGMNEPRLVGHANEWWPELTNSDVVDSINC
INQLNQDFVNTVRATGGKNASRYLMCPGYVASPDGATNDYFRMPNDISGNNNKIIVSVHAYCPWN
FAGLAMADGGTNAWNINDSKDQSEVTWFMDNIYNKYTSRGIPVIIGECGAVDKNNLKTRVEYMSY
YVAQAKARGILCILWDNNNFSGTGELFGFFDRRSCQFKFPEIIDGMVKYAFGLIN
>1ESC-_
APADPVPTVFFGDSYTANFGIAPVTNQDSERGWCFQAKENYPAVATRSLADKGITLDVQADVSCG
GALIHHFWEKQELPFGAGELPPQQDALKQDTQLTVGSLGGNTLGFNRILKQCSDELRKPSLLPGD
PVDGDEPAAKCGEFFGTGDGKQWLDDQFERVGAELEELLDRIGYFAPDAKRVLVGYPRLVPEDTT
KCLTAAPGQTQLPFADIPQDALPVLDQIQKRLNDAMKKAAADGGADFVDLYAGTGANTACDGADR
GIGGLLEDSQLELLGTKIPWYAHPNDKGRDIQAKQVADKIEEILNR
>1FUA-_
MERNKLARQIIDTCLEMTRLGLNQGTAGNVSVRYQDGMLITPTGIPYEKLTESHIVFIDGNGKHE
EGKLPSSEWRFHMAAYQSRPDANAVVHNHAVHCTAVSILNRSIPAIHYMIAAAGGNSIPCAPYAT
FGTRELSEHVALALKNRKATLLQHHGLIACEVNLEKALWLAHEVEVLAQLYLTTLAITDPVPVLS
DEEIAVVLEKFKTYGLRIEE
>1FUS-_
XSATTCGSTNYSASQVRAAANAACQYYQNDDTAGSSTYPHTYNNYEGFDFPVDGPYQEFPIKSGG
VYTGGSPGADRVVINTNCEYAGAITHTGASGNNFVGCSGTN
>1G3P-_
AETVESCLAKSHTENSFTNVXKDDKTLDRYANYEGCLWNATGVVVCTGDETQCYGTWVPIGLAIP
ENEGGGSEGGGSEGGGSEGGGTKPPEYGDTPIPGYTYINPLDGTYPPGTEQNPANPNPSLEESQP
LNTFMFQNNRFRNRQGALTVYTGTVTQGTDPVKTYYQYTPVSSKAMYDAYWNGKFRDCAFHSGFN
EDIFVCEYQGQSSDLPQPPVNA
>1GCA-_
ADTRIGVTIYKYDDNFMSVVRKAIEKDGKSAPDVQLLMNDSQNDQSKQNDQIDVLLAKGVKALAI
NLVDPAAAGTVIEKARGQNVPVVFFNKEPSRKALDSYDKAYYVGTDSKESGVIQGDLIAKHWQAN
QGWDLNKDGKIQYVLLKGEPGHPDAEARTTYVVKELNDKGIQTEQLALDTAMWDTAQAKDKMDAW
LSGPNANKIEVVIANNDAMAMGAVEALKAHNKSSIPVFGVDALPEALALVKSGAMAGTVLNDANN
QAKATFDLAKNLAEGKGAADGTSWKIENKIVRVPYVGVDKDNLSEFTQK
>1GEN-_
ELYGASPDIDLGTGPTPTLGPVTPEICKQDIVFDGIAQIRGEIFFFKDRFIWRTVTPRDKPMGPL
LVATFWPELPEKIDAVYEAPQEEKAVFFAGNEYWIYSASTLERGYPKPLTSLGLPPDVQRVDAAF

NWSKNKKTYIFAGDKFWRYNEVKKKMDPGFPKLIADAWNAIPDNLDAVVDLQGGGHSYFFKGAYY
LKLENQSLKSVKFGSIKSDWLGC
>1GOF-_
ASAPIGSAISRNNWAVTCDSAQSGNECNKAIDGNKDTFWHTFYGANGDPKPPHTYTIDMKTTQNV
NGLSMLPRQDGNQNGWIGRHEVYLSSDGTNWGSPVASGSWFADSTTKYSNFETRPARYVRLVAIT
EANGQPWTSIAEINVFQASSYTAPQPGLGRWGPTIDLPIVPAAAAIEPTSGRVLMWSSYRNDAFG
GSPGGITLTSSWDPSTGIVSDRTVTVTKHDMFCPGISMDGNGQIVVTGGNDAKKTSLYDSSSDSW
IPGPDMQVARGYQSSATMSDGRVFTIGGSWSGGVFEKNGEVYSPSSKTWTSLPNAKVNPMLTADK
QGLYRSDNHAWLFGWKKGSVFQAGPSTAMNWYYTSGSGDVKSAGKRQSNRGVAPDAMCGNAVMYD
AVKGKILTFGGSPDYQDSDATTNAHIITLGEPGTSPNTVFASNGLYFARTFHTSVVLPDGSTFIT
GGQRRGIPFEDSTPVFTPEIYVPEQDTFYKQNPNSIVRVYHSISLLLPDGRVFNGGGGLCGDCTT
NHFDAQIFTPNYLYNSNGNLATRPKITRTSTQSVKVGGRITISTDSSISKASLIRYGTATHTVNT
DQRRIPLTLTNNGGNSYSFQVPSDSGVALPGYWMLFVMNSAGVPSVASTIRVTQ
>1GPR-_
MIAEPLQNEIGEEVFVSPITGEIHPITDVPDQVFSGKMMGDGFAILPSEGIVVSPVRGKILNVFP
TKHAIGLQSDGGREILIHFGIDTVSLKGEGFTSFVSEGDRVEPGQKLLEVDLDAVKPNVPSLMTP
IVFTNLAEGETVSIKASGSVNREQEDIVKIEK
>1GSA-_
MIKLGIVMDPIANINIKKDSSFAMLLEAQRRGYELHYMEMGDLYLINGEARAHTRTLNVKQNYEE
WFSFVGEQDLPLADLDVILMRKDPPFDTEFIYATYILERAEEKGTLIVNKPQSLRDCNEKLFTAW
FSDLTPETLVTRNKAQLKAFWEKHSDIILKPLDGMGGASIFRVKEGDPNLGVIAETLTEHGTRYC
MAQNYLPAIKDGDKRVLVVDGEPVPYCLARIPQGGETRGNLAAGGRGEPRPLTESDWKIARQIGP
TLKEKGLIFVGLDIIGDRLTEINVTSPTCIREIEAEFPVSITGMLMDAIEARLQQQ
>1GYM-_
ASSVNELENWSKWMQPIPDSIPLARISIPGTHDSGTFKLQNPIKQVWGMTQEYDFRYQMDHGARI
FDIRGRLTDDNTIVLHHGPLYLYVTLHEFINEAKQFLKDNPSETIIMSLKKEYEDMKGAEDSFSS
TFEKKYFVDPIFLKTEGNIKLGDARGKIVLLKRYSGSNEPGGYNNFYWPDNETFTTTVNQNANVT
VQDKYKVSYDEKVKSIKDTMDETMNNSEDLNHLYINFTSLSSGGTAWNSPYYYASYINPEIANYI
KQKNPARVGWVIQDYINEKWSPLLYQEVIRANKSLIKE
>1HFC-_
VLTEGNPRWEQTHLTYRIENYTPDLPRADVDHAIEKAFQLWSNVTPLTFTKVSEGQADIMISFVR
GDHRDNSPFDGPGGNLAHAFQPGPGIGGDAHFDEDERWTNNFREYNLHRVAAHELGHSLGLSHST
DIGALMYPSYTFSGDVQLAQDDIDGIQAIYGRSQNPVQP
>1HKA-_
TVAYIAIGSNLASPLEQVNAALKALGDIPESHILTVSSFYRTPPLGPQDQPDYLNAAVALETSLA
PEELLNHTQRIELQQGRVRKAERWGPRTLDLDIMLFGNEVINTERLTVPHYDMKNRGFMLWPLFE
IAPELVFPDGEMLRQILHTRAFDKLNKW
>1HOE-_
DTTVSEPAPSCVTLYQSWRYSQADNGCAETVTVKVVYEDDTEGLCYAVAPGQITTVGDGYIGSHG
HARYLARCL
>1HTP-_
SNVLDGLKYAPSHEWVKHEGSVATIGITDHAQDHLGEVVFVELPEPGVSVTKGKGFGAVESVKAT
SDVNSPISGEVIEVNTGLTGKPGLINSSPYEDGWMIKIKPTSPDELESLLGAKEYTKFCEEEDAA
H

>1HXN-_
HRNSTQHGHESTRCDPDLVLSAMVSDNHGATYVFSGSHYWRLDTNRDGWHSWPIAHQWPQGPSTV
DAAFSWEDKLYLIQDTKVYVFLTKGGYTLVNGYPKRLEKELGSPPVISLEAVDAAFVCPGSSRLH
IMAGRRLWWLDLKSGAQATWTELPWPHEKVDGALCMEKPLGPNSCSTSGPNLYLIHGPNLYCYRH
VDKLNAAKNLPQPQRVSRLLGCTH
>1HYP-_
ALITRPSCPDLSICLNILGGSLGTVDDCCALIGGLGDIEAIVCLCIQLRALGILNLNRNLQLILN
SCGRSYPSNATCPRT
>1IAE-_
AAILGDEYLWSGGVIPYTFAGVSGADQSAILSGMQELEEKTCIRFVPRTTESDYVEIFTSGSGCW
SYVGRISGAQQVSLQANGCVYHGTIIHELMHAIGFYHEHTRMDRDNYVTINYQNVDPSMTSNFDI
DTYSRYVGEDYQYYSIMHYGKYSFSIQWGVLETIVPLQNGIDLTDPYDKAHMLQTDANQINNLYT
NECSL
>1INP-_
MSDILQELLRVSEKAANIARACRQQETLFQLLIEEKKEGEKNKKFAVDFKTLADVLVQEVIKENM
ENKFPGLGKKIFGEESNELTNDLGEKIIMRLGPTEEETVALLSKVLNGNKLASEALAKVVHQDVF
FSDPALDSVEINIPQDILGIWVDPIDSTYQYIKGSADITPNQGIFPSGLQCVTVLIGVYDIQTGV
PLMGVINQPFVSQDLHTRRWKGQCYWGLSYLGTNIHSLLPPVSTRSNSEAQSQGTQNPSSEGSCR
FSVVISTSEKETIKGALSHVCGERIFRAAGAGYKSLCVILGLADIYIFSEDTTFKWDSCAAHAIL
RAMGGGMVDLKECLERNPDTGLDLPQLVYHVGNEGAAGVDQWANKGGLIAYRSEKQLETFLSRLL
QHLAPVATHT
>1JDW-_
MLRVRCLRGGSRGAEAVHYIGSRLGRTLTGWVQRTFQSTQAATASSRNSCAADDKATEPLPKDCP
VSSYNEWDPLEEVIVGRAENACVPPFTIEVKANTYEKYWPFYQKQGGHYFPKDHLKKAVAEIEEM
CNILKTEGVTVRRPDPIDWSLKYKTPDFESTGLYSAMPRDILIVVGNEIIEAPMAWRSRFFEYRA
YRSIIKDYFHRGAKWTTAPKPTMADELYNQDYPIHSVEDRHKLAAQGKFVTTEFEPCFDAADFIR
AGRDIFAQRSQVTNYLGIEWMRRHLAPDYRVHIISFKDPNPMHIDATFNIIGPGIVLSNPDRPCH
QIDLFKKAGWTIITPPTPIIPDDHPLWMSSKWLSMNVLMLDEKRVMVDANEVPIQKMFEKLGITT
IKVNIRNANSLGGGFHCWTCDVRRRGTLQSYLD
>1JER-_
MQSTVHIVGDNTGWSVPSSPNFYSQWAAGKTFRVGDSLQFNFPANAHNVHEMETKQSFDACNFVN
SDNDVERTSPVIERLDELGMHYFVCTVGTHCSNGQKLSINVVAANATVSMPPPSSSPPSSVMPPP
VMPPPSPS
>1KLO-_
CPCPGGSSCAIVPKTKEVVCTHCPTGTAGKRCELCDDGYFGDPLGSNGPVRLCRPCQCNDNIDPN
AVGNCNRLTGECLKCIYNTAGFYCDRCKEGFFGNPLAPNPADKCKACACNPYGTVQQQSSCNPVT
GQCQCLPHVSGRDCGTCDPGYYNLQSGQGCER
>1KOE-_
QPVLHLVALNTPLSGGMRGIRGADFQCFQQARAVGLSGTFRAFLSSRLQDLYSIVRRADRGSVPI
VNLKDEVLSPSWDSLFSGSQGQLQPGARIFSFDGRDVLRHPAWPQKSVWHGSDPSGRRLMESYCE
TWRTETTGATGQASSLLSGRLLEQKAASCHNSYIVLCIENSF
>1KPF-_
XADEIAKAQVARPGGDTIFGKIIRKEIPAKIIFEDDRCLAFHDISPQAPTHFLVIPKKHISQISV
AEDDDESLLGHLMIVGKKCAADLGLNKGYRMVVNEGSDGGQSVYHVHLHVLGGRQMHWPPG

>1KTE-_
AQAFVNSKIQPGKVVVFIKPTCPFCRKTQELLSQLPFKEGLLEFVDITATSDTNEIQDYLQQLTG
ARTVPRVFIGKECIGGCTDLESMHKRGELLTRLQQVGAVK
>1KUH-_
TVTVTYDPSNAPSFQQEIANAAQIWNSSVRNVQLRAGGNADFSYYEGNDSRGSYAQTDGHGRGYI
FLDYQQNQQYDSTRVTAHETGHVLGLPDHYQGPCSELMSGGGPGPSCTNPYPNAQERSRVNALWA
NG
>1LBU-_
DGCYTWSGTLSEGSSGEAVRQLQIRVAGYPGTGAQLAIDGQFGPATKAAVQRFQSAYGLAADGIA
GPATFNKIYQLQDDDCTPVNFTYAELNRCNSDWSGGKVSAATARANALVTMWKLQAMRHAMGDKP
ITVNGGFRSVTCNSNVGGASNSRHMYGHAADLGAGSQGFCALAQAARNHGFTEILGPGYPGHNDH
THVAGGDGRFWSAPSCGI
>1LCL-_
MSLLPVPYTEAASLSTGSTVTIKGRPLVCFLNEPYLQVDFHTEMKEESDIVFHFQVCFGRRVVMN
SREYGAWKQQVESKNMPFQDGQEFELSISVLPDKYQVMVNGQSSYTFDHRIKPEAVKMVQVWRDI
SLTKFNVSYLKR
>1LED-_
XNTVNFTYPDFWSYSLKNGTEITFLGDATRIPGALQLTKTDANGNPVRSSAGQASYSEPVFLWDS
TGKAASFYTSFTFLLKNYGAPTADGLAFFLAPVDSSVKDYGGFLGLFRHETAADPSKNQVVAVEF
DTWINKDWNDPPYPHIGIDVNSIVSVATTRWENDDAYGSSIATAHITYDARSKILTVLLSYEHGR
DYILSHVVDLAKVLPQKVRIGFSAGVGYDEVTYILSWHFFSTLDGTNK
>1LIT-_
QEAQTELPQARISCPEGTNAYRSYCYYFNEDRETWVDADLYCQNMNSGNLVSVLTQAEGAFVASL
IKESGTDDFNVWIGLHDPKKNRRWHWSSGSLVSYKSWGIGAPSSVNPGYCVSLTSSTGFQKWKDV
PCEDKFSFVCKFKN
>1LST-_
ALPQTVRIGTDTTYAPFSSKDAKGEFIGFDIDLGNEMCKRMQVKCTWVASDFDALIPSLKAKKID
AIISSLSITDKRQQEIAFSDKLYAADSRLIAAKGSPIQPTLESLKGKHVGVLQGSTQEAYANDNW
RTKGVDVVAYANQDLIYSDLTAGRLDAALQDEVAASEGFLKQPAGKEYAFAGPSVKDKKYFGDGT
GVGLRKDDTELKAAFDKALTELRQDGTYDKMAKKYFDFNVYGDK
>1LTM-_
EPQHNVMQMGGDFANNPNAQQFIDKMVNKHGFDRQQLQEILSQAKRLDSVLRLMDNQAPTTSVKP
PSGPNGAWLRYRKKFITPDNVQNGVVFWNQYEDALNRAWQVYGVPPEIIVGIIGVETRWGRVMGK
TRILDALATLSFNYPRRAEYFSGELETFLLMARDEQDDPLNLKGSFAGAMGYGQFMPSSYKQYAV
DFSGDGHINLWDPVDAIGSVANYFKAHGWVKGDQVAVMANGQAPGLPNGFKTKYSISQLAAAGLT
PQQPLGNHQQASLLRLDVGTGYQYWYGLPNFYTITRYNHSTHYAMAVWQLGQAVALARVQ
>1MAT-_
MAISIKTPEDIEKMRVAGRLAAEVLEMIEPYVKPGVSTGELDRICNDYIVNEQHAVSACLGYHGY
PKSVCISINEVVCHGIPDDAKLLKDGDIVNIDVTVIKDGFHGDTSKMFIVGKPTIMGERLCRITQ
ESLYLALRMVKPGINLREIGAAIQKFVEAEGFSVVREYCGHGIGRGFHEEPQVLHYDSRETNVVL
KPGMTFTIEPMVNAGKKEIRTMKDGWTVKTKDRSLSAQYEHTIVVTDNGCEILTLRKDDTIPAII
SHDE
>1MAZ-_
MSMAMSQSNRELVVDFLSYKLSQKGYSWSQFSDVEENRTEAPEGTESEMETPSAINGNPSWHLAD

SPAVNGATGHSSSLDAREVIPMAAVKQALREAGDEFELRYRRAFSDLTSQLHITPGTAYQSFEQV
VNELFRDGVNWGRIVAFFSFGGALCVESVDKEMQVLVSRIAAWMATYLNDHLEPWIQENGGWDTF
VELYGNNAAAESRKGQERLEHHHHHH
>1MBA-_
XSLSAAEADLAGKSWAPVFANKNANGLDFLVALFEKFPDSANFFADFKGKSVADIKASPKLRDVS
SRIFTRLNEFVNNAANAGKMSAMLSQFAKEHVGFGVGSAQFENVRSMFPGFVASVAAPPAGADAA
WTKLFGLIIDALKAAGA
>1MLA-_
MTQFAFVFPGQGSQTVGMLADMAASYPIVEETFAEASAALGYDLWALTQQGPAEELNKTWQTQPA
LLTASVALYRVWQQQGGKAPAMMAGHSLGEYSALVCAGVIDFADAVRLVEMRGKFMQEAVPEGTG
AMAAIIGLDDASIAKACEEAAEGQVVSPVNFNSPGQVVIAGHKEAVERAGAACKAAGAKRALPLP
VSVPSHCALMKPAADKLAVELAKITFNAPTVPVVNNVDVKCETNGDAIRDALVRQLYNPVQWTKS
VEYMAAQGVEHLYEVGPGKVLTGLTKRIVDTLTASALNEPSAMAAALEL
>1MOQ-_
DAGDKGIYRHYMQKEIYEQPNAIKNTLTGRISHGQVDLSELGPNADELLSKVEHIQILACGTSYN
SGMVSRYWFESLAGIPCDVEIASEFRYRKSAVRRNSLMITLSQSGETADTLAGLRLSKELGYLGS
LAICNVPGSSLVRESDLALMTNAGTEIGVASTKAFTTQLTVLLMLVAKLSRLKGLDASIEHDIVH
GLQALPSRIEQMLSQDKRIEALAEDFSDKHHALFLGRGDQYPIALEGALKLKEISYIHAEAYAAG
ELKHGPLALIDADMPVIVVAPNNELLEKLKSNIEEVRARGGQLYVFADQDAGFVSSDNMHIIEMP
HVEEVIAPIFYTVPLQLLAYHVALIKGTDVDQPRNLAKSVTVE
>1MPP-_
AEGDGSVDTPGLYDFDLEEYAIPVSIGTPGQDFYLLFDTGSSDTWVPHKGCDNSEGCVGKRFFDP
SSSSTFKETDYNLNITYGTGGANGIYFRDSITVGGATVKQQTLAYVDNVSGPTAEQSPDSELFLD
GIFGAAYPDNTAMEAEYGDTYNTVHVNLYKQGLISSPVFSVYMNTNDGGGQVVFGGVNNTLLGGD
IQYTDVLKSRGGYFFWDAPVTGVKIDGSDAVSFDGAQAFTIDTGTNFFIAPSSFAEKVVKAALPD
ATESQQGYTVPCSKYQDSKTTFSLVLQKSGSSSDTIDVSVPISKMLLPVDKSGETCMFIVLPDGG
NQFIVGNLFLRFFVNVYDFGKNRIGFAPLASGYEND
>1MSK-_
KKPRTPPVTLEAARDNDFAFDWQAYTPPVAHRLGVQEVEASIETLRNYIDWTPFFMTWSLAGKYP
RILEDEVVGVEAQRLFKDANDMLDKLSAEKTLNPRGVVGLFPANRVGDDIEIYRDETRTHVINVS
HHLRQQTEKTGFANYCLADFVAPKLSGKADYIGAFAVTGGLEEDALADAFEAQHDDYNKIMVKAL
ADRLAEAFAEYLHERVRKVYWGYAPNENLSNEELIRENYQGIRPAPGYPACPEHTEKATIWELLE
VEKHTGMKLTESFAMWPGASVSGWYFSHPDSKYYAVAQIQRDQVEDYARRKGMSVTEVERWLAPN
LGYDAD
>1MUP-_
CVHAEEASSTGRNFNVEKINGEWHTIILASDKREKIEDNGNFRLFLEQIHVLENSLVLKFHTVRD
EECSELSMVADKTEKAGEYSVTYDGFNTFTIPKTDYDNFLMAHLINEKDGETFQLMGLYGREPDL
SSDIKERFAQLCEEHGILRENIIDLSNANRCLQARE
>1NAR-_
PKPIFREYIGVKPNSTTLHDFPTEIINTETLEFHYILGFAIESYYESGKGTGTFEESWDVELFGP
EKVKNLKRRHPEVKVVISIGGRGVNTPFDPAEENVWVSNAKESLKLIIQKYSDDSGNLIDGIDIH
YEHIRSDEPFATLMGQLITELKKDDDLNINVVSIAPSENNSSHYQKLYNAKKDYINWVDYQFSNQ
QKPVSTDDAFVEIFKSLEKDYHPHKVLPGFSTDPLDTKHNKITRDIFIGGCTRLVQTFSLPGVFF
WNANDSVIPKRDGDKPFIVELTLQQLLAAR

>1NEU-_
IVVYTDREVYGAVGSQVTLHCSFWSSEWVSDDISFTWRYQPEGGRDAISIFHYAKGQPYIDEVGT
FKERIQWVGDPSWKDGSIVIHNLDYSDNGTFTCDVKNPPDIVGKTSQVTLYVFEKVPTR
>1NFP-_
MTKWNYGVFFLNFYHVGQQEPSLTMSNALETLRIIDEDTSIYDVVAFSEHHIDKSYNDETKLAPF
VSLGKQIHVLATSPETVVKAAKYGMPLLFKWDDSQQKRIELLNHYQAAAAKFNVDIANVRHRLML
FVNVNDNPTQAKAELSIYLEDYLSYTQAETSIDEIINSNAAGNFDTCLHHVAEMAQGLNNKVDFL
FCFESMKDQENKKSLMINFDKRVINYRKEHNLN
>1NIF-_
AAGAAPVDISTLPRVKVDLVKPPFVHAHDQVAKTGPRVVEFTMTIEEKKLVIDREGTEIHAMTFN
GSVPGPLMVVHENDYVELRLINPDTNTLLHNIDFHAATGALGGGALTQVNPGEETTLRFKATKPG
VFVYHCAPEGMVPWHVTSGMNGAIMVLPRDGLKDEKGQPLTYDKIYYVGEQDFYVPKDEAGNYKK
YETPGEAYEDAVKAMRTLTPTHIVFNGAVGALTGDHALTAAVGERVLVVHSQANRDTRPHLIGGH
GDYVWATGKFRNPPDLDQETWLIPGGTAGAAFYTFRQPGVYAYVNHNLIEAFELGAAGHFKVTGE
WNDDLMTSVVKPASM
>1NKR-_
MHEGVHRKPSLLAHPGPLVKSEETVILQCWSDVMFEHFLLHREGMFNDTLRLIGEHHDGVSKANF
SISRMTQDLAGTYRCYGSVTHSPYQVSAPSDPLDIVIIGLYEKPSLSAQPGPTVLAGENVTLSCS
SRSSYDMYHLSREGEAHERRLPAGPKVNGTFQADFPLGPATHGGTYRCFGSFHDSPYEWSKSSDP
LLVSVT
>1NLS-_
ADTIVAVELDTYPNTDIGDPSYPHIGIDIKSVRSKKTAKWNMQNGKVGTAHIIYNSVDKRLSAVV
SYPNADSATVSYDVDLDNVLPEWVRVGLSASTGLYKETNTILSWSFTSKLKSNSTHETNALHFMF
NQFSKDQKDLILQGDATTGTDGNLELTRVSSNGSPQGSSVGRALFYAPVHIWESSAVVASFEATF
TFLIKSPDSHPADGIAFFISNIDSSIPSGSTGRLLGLFPDAN
>1NNC-_
RDFNNLTKGLCTINSWHIYGKDNAVRIGEDSDVLVTREPYVSCDPDECRFYALSQGTTIRGKHSN
GTIHDRSQYRALISWPLSSPPTVYNSRVECIGWSSTSCHDGKTRMSICISGPNNNASAVIWYNRR
PVTEINTWARNILRTQESECVCHNGVCPVVFTDGSATGPAETRIYYFKEGKILKWEPLAGTAKHI
EECSCYGERAEITCTCRDNWQGSNRPVIRIDPVAMTHTSQYICSPVLTDNPRPNDPTVGKCNDPY
PGNNNNGVKGFSYLDGVNTWLGRTISIASRSGYEMLKVPNALTDDKSKPTQGQTIVLNTDWSGYS
GSFMDYWAEGECYRACFYVELIRGRPKEDKVWWTSNSIVSMCSSTEFLGQWDWPDGAKIEYFL
>1NPK-_
STNKVNKERTFLAVKPDGVARGLVGEIIARYEKKGFVLVGLKQLVPTKDLAESHYAEHKERPFFG
GLVSFITSGPVVAMVFEGKGVVASARLMIGVTNPLASAPGSIRGDFGVDVGRNIIHGSDSVESAN
REIALWFKPEELLTEVKPNPNLYE
>1OBR-_
DFPSYDSGYHNYNEMVNKINTVASNYPNIVKKFSIGKSYEGRELWAVKISDNVGTDENEPEVLYT
ALHHAREHLTVEMALYTLDLFTQNYNLDSRITNLVNNREIYIVFNINPDGGEYDISSGSYKSWRK
NRQPNSGSSYVGTDLNRNYGYKWGCCGGSSGSPSSETYRGRSAFSAPETAAMRDFINSRVVGGKQ
QIKTLITFHTYSELILYPYGYTYTDVPSDMTQDDFNVFKTMANTMAQTNGYTPQQASDLYITDGD
MTDWAYGQHKIFAFTFEMYPTSYNPGFYPPDEVIGRETSRNKEAVLYVAEKADCPYSVIGKSCST
K
>1OPS-_

SQSVVATQLIPMNTALTPAMMEGKVTNPIGIPFAEMSQLVGKQVNTPVAKGQTLMPNMVKTYAA
>1OPY-_
MNLPTAQEVQGLMARYIELVDVGDIEAIVQMYADDATVEDPFGQPPIHGREQIAAFYRQGLGGGK
VRACLTGPVRASHNGCGAMPFRVEMVWNGQPCALDVIDVMRFDEHGRIQTMQAYWSEVNLSVREP
Q
>1OSA-_
AEQLTEEQIAEFKEAFALFDKDGDGTITTKELGTVMRSLGQNPTEAELQDMINEVDADGNGTIDF
PEFLSLMARKMKEQDSEEELIEAFKVFDRDGNGLISAAELRHVMTNLGEKLTDDEVDEMIREADI
DGDGHINYEEFVRMMVSK
>1PDO-_
TIAIVIGTHGWAAEQLLKTAEMLLGEQENVGWIDFVPGENAETLIEKYNAQLAKLDTTKGVLFLV
DTWGGSPFNAASRIVVDKEHYEVIAGVNIPMLVETLMARDDDPSFDELVALAVETGREGVKALKA
KPFAG
>1PEA-_
MGSHQERPLIGLLFSETGVTADIERSQRYGALLAVEQLNREGGVGGRPIETLSQDPGGDPDRYRL
CAEDFIRNRGVRFLVGCYMSHTRKAVMPVVERADALLCYPTPYEGFEYSPNIVYGGPAPNQNSAP
LAAYLIRHYGERVVFIGSDYIYPRESNHVMRHLYRQHGGTVLEEIYIPLYPSDDDLQRAVERIYQ
ARADVVFSTVVGTGTAELYRAIARRYGDGRRPPIASLTTSEAEVAKMESDVAEGQVVVAPYFSSI
DTPASRAFVQACHGFFPENATITAWAEAAYWQTLLLGRAAQAAGNWRVEDVQRHLYDIDIDAPQG
PVRVERQNNHSRLSSRIAEIDARGVFQVRWQSPEPIRPDPYVVVHNLDDWSASMGGGPLP
>1PGS-_
APADNTVNIKTFDKVKNAFGDGLSQSAEGTFTFPADVTTVKTIKMFIKNECPNKTCDEWDRYANV
YVKNKTTGEWYEIGRFITPYWVGTEKLPRGLEIDVTDFKSLLSGNTELKIYTETWLAKGREYSVD
FDIVYGTPDYKYSAVVPVIQYNKSSIDGVPYGKAHTLGLKKNIQLPTNTEKAYLRTTISGWGHAK
PYDAGSRGCAEWCFRTHTIAINNANTFQHQLGALGCSANPINNQSPGNWTPDRAGWCPGMAVPTR
IDVLNNSLTGSTFSYEYKFQSWTNNGTNGDAFYAISSFVIAKSNTPISAPVVTN
>1PHD-_
TTETIQSNANLAPLPPHVPEHLVFDFDMYNPSNLSAGVQEAWAVLQESNVPDLVWTRCNGGHWIA
TRGQLIREAYEDYRHFSSECPFIPREAGEAYDFIPTSMDPPEQRQFRALANQVVGMPVVDKLENR
IQELACSLIESLRPQGQCNFTEDYAEPFPIRIFMLLAGLPEEDIPHLKYLTDQMTRPDGSMTFAE
AKEALYDYLIPIIEQRRQKPGTDAISIVANGQVNGRPITSDEAKRMCGLLLVGGLDTVVNFLSFS
MEFLAKSPEHRQELIERPERIPAACEELLRRFSLVADGRILTSDYEFHGVQLKKGDQILLPQMLS
GLDERENACPMHVDFSRQKVSHTTFGHGSHLCLGQHLARREIIVTLKEWLTRIPDFSIAPGAQIQ
HKSGIVSGVQALPLVWDPATTKAV
>1PHM-_
NECLGTIGPVTPLDASDFALDIRMPGVTPKESDTYFCMSMRLPVDEEAFVIDFKPRASMDTVHHM
LLFGCNMPSSTGSYWFCDEGTCTDKANILYAWARNAPPTRLPKGVGFRVGGETGSKYFVLQVHYG
DISAFRDNHKDCSGVSVHLTRVPQPLIAGMYLMMSVDTVIPPGEKVVNADISCQYKMYPMHVFAY
RVHTHHLGKVVSGYRVRNGQWTLIGRQNPQLPQAFYPVEHPVDVTFGDILAARCVFTGEGRTEAT
HIGGTSSDEMCNLYIMYYMEAKYALSFMTCTKNVAPDMFRTIPAEANIPI
>1PHR-_
AEQVTKSVLFVCLGNICRSPIAEAVFRKLVTDQNISDNWVIDSGAVSDWNVGRSPDPRAVSCLRN
HGINTAHKARQVTKEDFVTFDYILCMDESNLRDLNRKSNQVKNCRAKIELLGSYDPQKQLIIEDP
YYGNDADFETVYQQCVRCCRAFLEKVR

>1PHT-_
MSAEGYQYRALYDYKKEREEDIDLHLGDILTVNKGSLVALGFSDGQEARPEEIGWLNGYNETTGE
RGDFPGTYVEYIGRKKISPP
>1PMI-_
SSEKLFRIQCGYQNYDWGKIGSSSAVAQFVHNSDPSITIDETKPYAELWMGTHPSVPSKAIDLNN
QTLRDLVTAKPQEYLGESIITKFGSSKELPFLFKVLSIEKVLSIQAHPDKKLGAQLHAADPKNYP
DDNHKPEMAIAVTDFEGFCGFKPLDQLAKTLATVPELNEIIGQELVDEFISGIKLPAEVGSQDDV
NNRKLLQKVFGKLMNTDDDVIKQQTAKLLERTDREPQVFKDIDSRLPELIQRLNKQFPNDIGLFC
GCLLLNHVGLNKGEAMFLQAKDPHAYISGDIIECMAASDNVVRAGFTPKFKDVKNLVEMLTYSYE
SVEKQKMPLQEFPRSKGDAVKSVLYDPPIAEFSVLQTIFDKSKGGKQVIEGLNGPSIVIATNGKG
TIQITGDDSTKQKIDTGYVFFVAPGSSIELTADSANQDQDFTTYRAFVEA
>1PNE-_
XAGWNAYIDNLMADGTCQDAAIVGYKDSPSVWAAVPGKTFVNITPAEVGILVGKDRSSFFVNGLT
LGGQKCSVIRDSLLQDGEFTMDLRTKSTGGAPTFNITVTMTAKTLVLLMGKEGVHGGMINKKCYE
MASHLRRSQY
>1POA-_
NLYQFKNMIQCTVPSRSWWDFADYGCYCGRGGSGTPVDDLDRCCQVHDNCYNEAEKISGCWPYFK
TYSYECSQGTLTCKGGNNACAAAVCDCDRLAAICFAGAPYNDNDYNINLKARC
>1POC-_
IIYPGTLWCGHGNKSSGPNELGRFKHTDACCRTHDMCPDVMSAGESKHGLTNTASHTRLSCDCDD
KFYDCLKNSADTISSYFVGKMYFNLIDTKCYKLEHPVTGCGERTEGRCLHYTVDKSKPKVYQWFD
LRKY
>1PRN-_
EISLNGYGRFGLQYVEDRGVGLEDTIISSRLRINIVGTTETDQGVTFGAKLRMQWDDGDAFAGTA
GNAAQFWTSYNGVTVSVGNVDTAFDSVALTYDSEMGYEASSFGDAQSSFFAYNSKYDASGALDNY
NGIAVTYSISGVNLYLSYVDPDQTVDSSLVTEEFGIAADWSNDMISLAAAYTTDAGGIVDNDIAF
VGAAYKFNDAGTVGLNWYDNGLSTAGDQVTLYGNYAFGATTVRAYVSDIDRAGADTAYGIGADYQ
FAEGVKVSGSVQSGFANETVADVGVRFDF
>1PTF-_
MEKKEFHIVAETGIHARPATLLVQTASKFNSDINLEYKGKSVNLKSIMGVMSLGVGQGSDVTITV
DGADEAEGMAAIVETLQKEGLAE
>1PUC-_
SKSGVPRLLTASERERLEPFIDQIHYSPRYADDEYEYRHVMLPKAMLKAIPTDYFNPETGTLRIL
QEEEWRGLGITQSLGWEMYEVHVPEPHILLFKREKDYQMK
>1RB9-_
XMKKYVCTVCGYEYDPAEGDPDNGVKPGTSFDDLPADWVCPVCGAPKSEFEAA
>1REC-_
GNSKSGALSKEILEELQLNTKFTEEELSSWYQSFLKECPSGRITRQEFQTIYSKFFPEADPKAYA
QHVFRSFDANSDGTLDFKEYVIALHMTSAGKTNQKLEWAFSLYDVDGNGTISKNEVLEIVTAIFK
MISPEDTKHLPEDENTPEKRAEKIWGFFGKKDDDKLTEKEFIEGTLANKEILRLIQFEPQKVKEK
LKEKKL
>1RFS-_
FVPPGGGAGTGGTIAKDALGNDVIAAEWLKTHAPGDRTLTQGLKGDPTYLVVESDKTLATFGINA
VCTHLGCVVPFNAAENKFICPCHGSQYNNQGRVVRGPAPLSLALAHCDVDDGKVVFVPWTETDFR

TGEAPWWSA
>1RH4-_
XAALAQXKKEIAYLLAKXKAEILAALKKXKQEIAX
>1RIE-_
VLAMSKIEIKLSDIPEGKNMAFKWRGKPLFVRHRTKKEIDQEAAVEVSQLRDPQHDLERVKKPEW
VILIGVCTHLGCVPIANAGDFGGYYCPCHGSHYDASGRIRKGPAPLNLEVPSYEFTSDDMVIVG
>1RKD-_
MQNAGSLVVLGSINADHILNLQSFPTPGETVTGNHYQVAFGGKGANQAVAAGRSGANIAFIACTG
DDSIGESVRQQLATDNIDITPVSVIKGESTGVALIFVNGEGENVIGIHAGANAALSPALVEAQRE
RIANASALLMQLESPLESVMAAAKIAHQNKTIVALNPAPARELPDELLALVDIITPNETEAEKLT
GIRVENDEDAAKAAQVLHEKGIRTVLITLGSRGVWASVNGEGQRVPGFRVQAVDTIAAGDTFNGA
LITALLEEKPLPEAIRFAHAAAAIAVTRKGAQPSVPWREEIDAFLDRQR
>1RMG-_
QLSGSVGPLTSASTKGATKTCNILSYGAVADNSTDVGPAITSAWAACKSGGLVYIPSGNYALNTW
VTLTGGSATAIQLDGIIYRTGTASGNMIAVTDTTDFELFSSTSKGAVQGFGYVYHAEGTYGARIL
RLTDVTHFSVHDIILVDAPAFHFTMDTCSDGEVYNMAIRGGNEGGLDGIDVWGSNIWVHDVEVTN
KDECVTVKSPANNILVESIYCNWSGGCAMGSLGADTDVTDIVYRNVYTWSSNQMYMIKSNGGSGT
VSNVLLENFIGHGNAYSLDIDGYWSSMTAVAGDGVQLNNITVKNWKGTEANGATRPPIRVVCSDT
APCTDLTLEDIAIWTESGSSELYLCRSAYGSGYCLKDSSSHTSYTTTSTVTAAPSGYSATTMAAD
LATAFGLTASIPIPTIPTSFYPGLTPYSALAG
>1RSY-_
GGGILDSMVEKLGKLQYSLDYDFQNNQLLVGIIQAAELPALDMGGTSDPYVKVFLLPDKKKKFET
KVHRKTLNPVFNEQFTFKVPYSELGGKTLVMAVYDFDRFSKHDIIGEFKVPMNTVDFGHVTEEWR
DLQSA
>1RZL-_
ITCGQVNSAVGPCLTYARGGAGPSAACCSGVRSLKAAASTTADRRTACNCLKNAARGIKGLNAGN
AASIPSKCGVSVPYTISASIDCSRVS
>1SBP-_
KDIQLLNVSYDPTRELYEQYNKAFSAHWKQETGDNVVIDQSHGGSGKQATSVINGIEADTVTLAL
AYDVNAIAERGRIDKNWIKRLPDDSAPYTSTIVFLVRKGNPKQIHDWNDLIKPGVSVITPNPKSS
GGARWNYLAAWGYALHHNNNDQAKAEDFVKALFKNVEVLDSGARGSTNTFVERGIGDVLIAWENE
ALLATNELGKDKFEIVTPSESILAEPTVSVVDKVVEKKDTKAVAEAYLKYLYSPEGQEIAAKNFY
RPRDADVAKKYDDAFPKLKLFTIDEVFGGWAKAQKDHFADGGTFDQISKR
>1SEK-_
MAGETDLQKILRESNDQFTAQMFSEVVKANPGQNVVLSAFSVLPPLGQLALASVGESHDELLRAL
ALPNDNVTKDVFADLNRGVRAVKGVDLKMASKIYVAKGLELNDDFAAVSRDVFGSEVQNVDFVKS
VEAAGAINKWVEDQTNNRIKNLVDPDALDETTRSVLVNAIYFKGSWKDKFNKERTMDRDFHVSKD
KTIKVPTMIGKKDVRYADVPELDAKMIEMSYEGDQASMIIILPNQVDGITALEQKLKDPKALSRA
EERLYNTEVEIYLPKFKIETTTDLKEVLSNMNIKKLFTPGAARLENLLKTKESLYVDAAIQKAFI
EVNEEGAEAAAANAFKITTYSFHFVPKVEINKPFFFSLKYNRNSMFSGVCVQP
>1SFP-_
MDWLPRNTNCGGILKEESGVIATYYGPKTNCVWTIQMPPEYHVRVSIQYLQLNCNKESLEIIDGL
PGSPVLGKICEGSLMDYRSSGSIMTVKYIREPEHPASFYEVLYFQDPQA
>1SMD-_

XYSSNTQQGRTSIVHLFEWRWVDIALECERYLAPKGFGGVQVSPPNENVAIHNPFRPWWERYQPV
SYKLCTRSGNEDEFRNMVTRCNNVGVRIYVDAVINHMCGNAVSAGTSSTCGSYFNPGSRDFPAVP
YSGWDFNDGKCKTGSGDIENYNDATQVRDCRLSGLLDLALGKDYVRSKIAEYMNHLIDIGVAGFR
IDASKHMWPGDIKAILDKLHNLNSNWFPEGSKPFIYQEVIDLGGEPIKSSDYFGNGRVTEFKYGA
KLGTVIRKWNGEKMSYLKNWGEGWGFMPSDRALVFVDNHDNQRGHGAGGASILTFWDARLYKMAV
GFMLAHPYGFTRVMSSYRWPRYFENGKDVNDWVGPPNDNGVTKEVTINPDTTCGNDWVCEHRWRQ
IRNMVNFRNVVDGQPFTNWYDNGSNQVAFGRGNRGFIVFNNDDWTFSLTLQTGLPAGTYCDVISG
DKINGNCTGIKIYVSDDGKAHFSISNSAEDPFIAIHAESKL

>1SRA-_

PPCLDSELTEFPLRMRDWLKNVLVTLYERDEDNNLLTEKQKLRVKKIHENEKRLEAGDHPVELLA
RDFEKNYNMYIFPVHWQFGQLDQHPIDGYLSHTELAPLRAPLIPMEHCTTRFFETCDLDNDKYIA
LDEWAGCFGIKQKDIDKDLVI

>1SVY-_

SGFNHVKPTEYKPRLLHISGDKNAKVAEVPLATSSLNSGDCFLLDAGLTIYQFNGSKSSPQEKNK
AAEVARAIDAERKGLPKVEVFXETDSDIPAEFWKLLGGKGAIAAKHETA

>1TCA-_

LPSGSDPAFSQPKSVLDAGLTCQGASPSSVSKPILLVPGTGTTGPQSFDSNWIPLSTQLGYTPCW
ISPPPFMLNDTQVNTEYMVNAITALYAGSGNNKLPVLTWSQGGLVAQWGLTFFPSIRSKVDRLMA
FAPDYKGTVLAGPLDALAVSAPSVWQQTTGSALTTALRNAGGLTQIVPTTNLYSATDEIVQPQVS
NSPLDSSYLFNGKNVQAQAVCGPLFVIDHAGSLTSQFSYVVGRSALRSTTGQARSADYGITDCNP
LPANDLTPEQKVAAAALLAPAAAAIVAGPKQNCEPDLMPYARPFAVGKRTCSGIVTP

>1TDE-_

GTTKHSKLLILGSGPAGYTAAVYAARANLQPVLITGMEKGGQLTTTTEVENWPGDPNDLTGPLLM
ERMHEHATKFETEIIFDHINKVDLQNRPFRLNGDNGEYTCDALIIATGASARYLGLPSEEAFKGR
GVSACATCDGFFYRNQKVAVIGGGNTAVEEALYLSNIASEVHLIHRRDGFRAEKILIKRLMDKVE
NGNIILHTNRTLEEVTGDQMGVTGVRLRDTQNSDNIESLDVAGLFVAIGHSPNTAIFEGQLELEN
GYIKVQSGIHGNATQTSIPGVFAAGDVMDHIYRQAITSAGTGCMAALDAERYLDGL

>1TFE-_

AREGIIGHYIHHNQRVGVLVELNCETDFVARNELFQNLAKDLAMHIAMMNPRYVSAEEIPAEELE
KERQIYIQAALNEGKPQQIAEKIAEGRLKKYLEEVVLLEQPFVKDDKVKVKELIQQAIAKIGENI
VVRRFCRFELGAMMC

>1TMY-_

MGKRVLIVDDAAFMRMMLKDIITKAGYEVAGEATNGREAVEKYKELKPDIVTMDITMPEMNGIDA
IKEIMKIDPNAKIIVCSAMGQQAMVIEAIKAGAKDFIVKPFQPSRVVEALNKVSK

>1TON-_

IVGGYKCEKNSQPWQVAVINEYLCGGVLIDPSWVITAAHCYSNNYQVLLGRNNLFKDEPFAQRRL
VRQSFRHPDYIPLIVTNDTEQPVHDHSNDLMLLHLSEPADITGGVKVIDLPTKEPKVGSTCLASG
WGSTNPSEMVVSHDLQCVNIHLLSNEKCIETYKDNVTDVMLCAGEMEGGKDTCAGDSGGPLICDG
VLQGITSGGATPCAKPKTPAIYAKLIKFTSWIKKVMKENP

>1TUL-_

MASMSNGTPDIIVNAQINSEDENVLDFIIEDEYYLKKRGVGAHIIKVASSPQLRLLYKNAYSTVS
CGNYGVLCNLVQNGEYDLNAIMFNCAEIKLNKGQMLFQTKIWR

>1UOK-_

MEKQWWKESVVYQIYPRSFMDSNGDGIGDLRGIISKLDYLKELGIDVIWLSPVYESPNDDNGYDI

SDYCKIMNEFGTMEDWDELLHEMHERNMKLMMDLVVNHTSDEHNWFIESRKSKDNKYRDYYIWRP
GKEGKEPNNWGAAFSGSAWQYDEMTDEYYLHLFSKKQPDLNWDNEKVRQDVYEMMKFWLEKGIDG
FRMDVINFISKEEGLPTVETEEEGYVSGHKHFMNGPNIHKYLHEMNEEVLSHYDIMTVGEMPGVT
TEEAKLYTGEERKELQMVFQFEHMDLDSGEGGKWDVKPCSLLTLKENLTKWQKALEHTGWNSLYW
NNHDQPRVVSRFGNDGMYRIESAKMLATVLHMMKGTPYIYQGEEIGMTNVRFESIDEYRDIETLN
MYKEKVMERGEDIEKVMQSIYIKGRDNARTPMQWDDQNHAGFTTGEPWITVNPNYKEINVKQAIQ
NKDSIFYYYKKLIELRKNNEIVVYGSYDLILENNPSIFAYVRTYGVEKLLVIANFTAEECIFELP
EDISYSEVELLIHNYDVENGPIENITLRPYEAMVFKLK

>1USH-_

MKLLQRGVALALLTTFTLASETALAYEQDKTYKITVLHTNDHHGHFWRNEYGEYGLAAQKTLVDG
IRKEVAAEGGSVLLLSGGDINTGVPESDLQDAEPDFRGMNLVGYDAMAIGNHEFDNPLTVLRQQE
KWAKFPLLSANIYQKSTGERLFKPWALFKRQDLKIAVIGLTTDDTAKIGNPEYFTDIEFRKPADE
AKLVIQELQQTEKPDIIIAATHMGHYDNGEHGSNAPGDVEMARALPAGSLAMIVGGHSQDPVCMA
AENKKQVDYVPGTPCKPDQQNGIWIVQAHEWGKYVGRADFEFRNGEMKMVNYQLIPVNLKKKVTW
EDGKSERVLYTPEIAENQQMISLLSPFQNKGKAQLEVKIGETNGRLEGDRDKVRFVQTNMGRLIL
AAQMDRTGADFAVMSGGGIRDSIEAGDISYKNVLKVQPFGNVVVYADMTGKEVIDYLTAVAQMKP
DSGAYPQFANVSFVAKDGKLNDLKIKGEPVDPAKTYRMATLNFNATGGDGYPRLDNKPGYVNTGF
IDAEVLKAYIQKSSPLDVSVYEPKGEVSWQ

>1UTG-_

GICPRFAHVIENLLLGTPSSYETSLKEFEPDDTMKDAGMQMKKVLDSLPQTTRENIMKLTEKIVK
SPLCM

>1VLS-_

MNQQGFVISNELRQQQSELTSTWDLMLQTRINLSRSAARMMMDASNQQSSAKTDLLQNAKTTLAQ
AAAHYANFKNMTPLPAMAEASANVDEKYQRYQAALAELIQFLDNGNMDAYFAQPTQGMQNALGEA
LGNYARVSENLYRQT

Supplementary S3. The benchmark dataset $\mathbb{S}$ contains 1075 protein sequences, which are classified into subset $\mathbb{S}^+$ with 525 DNA-binding proteins (positive samples) and subset $\mathbb{S}^-$ with 550 non DNA-binding proteins (negative samples).

---

(1).525 DNA-binding proteins

>1AKHA
KKEKSPKGKSSISPQARAFLEEVFRRKQSLNSKEKEEVAKKCGITPLQVRVWFINKRMRSK
>1AOII
ATCAATATCCACCTGCAGATTCTACCAAAAGTGTATTTGGAAACTGCTCCATCAAAAGGCATGTT
CAGCTGAATTCAGCTGAACATGCCTTTTGATGGAGCAGTTTCCAAATACACTTTTGGTAGAATCT
GCAGGTGGATATTGAT
>1B6WA
MELPIAPIGRIIKDAGAERVSDDARITLAKILEEMGRDIASEAIKLARHAGRKTIKAEDIELAVR
RFKK
>1C1KA
MIKLRMPAGGERYIDGKSVYKLYLMIKQHMNGKYDVIKYNWCMRVSDAAYQKRRDKYFFQKLSEK
YKLKELALIFISNLVANQDAWIGDISDADALVFYREYIGRLKQIKFKFEEDIRNIYYFSKKVEVS
AFKEIFEYNPKVQSSYIFKLLQSNIISFETFILLDSFLNIIDKHDEQTDNLVWNNYSIKLKAYRK
ILNIDSQKAKNVFIETVKSCKY
>1C6VX
QQSKNSKFKNFRVYYREGRDQLWKGPGELLWKGEGAVLLKVGTDIKVVPRRKAKIIKDYGGGKEV
DSSSHMEDTGEAREVA
>1C6VD
IHGQVNSDLGTWQMDCTHLEGKIVIVAVHVASGFIEAEVIPQETGRQTALFLLKLAGRWPITHLH
TDNGANFASQEVKMVAWWAGIEHTFGVPYNPQSQGVVEAMNHHLKNQIDRIREQANSVETIVLMA
VHCMNHKRRGGIGDMTPAERLINMITTEQEIQFQ
>1CI4B
MTTSQKHRDFVAEPMGEKPVGSLAGIGEVLGKKLEERGFDKAYVVLGQFLVLKKDEDLFREWLKD
TCGANAKQSRDCFGCLREWCDAFL
>1D4UA
MEFDYVICEECGKEFMDSYLMDHFDLPTCDDCRDADDKHKLITKTEAKQEYLLKDCDLEKREPPL
KFIVKKNPHHSQWGDMKLYLKLQIVKRSLEVWGSQEALEEAKEVRQ
>1D8BA
ELNNLRMTYERLRELSLNLGNRMVPPVGNFMPDSILKKMAAILPMNDSAFATLGTVEDKYRRRFK
YFKATIADLSKKRSSE
>1DMLG
MTDSPGGVAPASPVEDASDASLGQPEEGAPCQVVLQGAELNGILQAFAPLRTSLLDSLLVMGDRG
ILIHNTIFGEQVFLPLEHSQFSRYRWRGPTAAFLSLVDQKRSLLSVFRANQYPDLRRVELAITGQ
APFRTLVQRIWTTTSDGEAVELASETLMKRELTSFVVLVPQGTPDVQLRLTRPQLTKVLNATGAD
SATPTTFELGVNGKFSVFTTSTCVTFAAREEGVSSSTSTQVQILSNALTKAGQAAANAKTVYGEN
THRTFSVVVDDCSMRAVLRRLQVGGGTLKFFLTTPVPSLCVTATGPNAVSAVFLLKPQK
>1EE8B
PELPEVETTRRRLRPLVLGQTLRQVVHRDPARYRNTALAEGRRILEVDRRGKFLLFALEGGVELV
AHLGMTGGFRLEPTPHTRAALVLEGRTLYFHDPRRFGRLFGVRRGDYREIPLLLRLGPEPLSEAF

AFPGFFRGLKESARPLKALLLDQRLAAGVGNIYADEALFRARLSPFRPARSLTEEEARRLYRALR
EVLAEAVELGGSTLSDQSYRQPDGLPGGFQTRHAVYGREGLPCPACGRPVERRVVAGRGTHFCPT
CQGEGP

>1EIJA

MRQQLEMQKKQIMMQILTPEARSRLANLRLTRPDFVEQIELQLIQLAQMGRVRSKITDEQLKELL
KRVAGKKREIKISRK

>1F1EA

MAVELPKAAIERIFRQGIGERRLSQDAKDTIYDFVPTMAEYVANAAKSVLDASGKKTLMEEHLKA
LADVLMVEGVEDYDGELFGRATVRRILKRAGIERASSDAVDLYNKLICRATEELGEKAAEYADED
GRKTVQGEDVEKAITYSMPKGGEL

>1F2RI

MELSRGASAPDPDDVRPLKPCLLRRNHSRDQHGVAASSLEELRSKACELLAIDKSLTPITLVLAE
DGTIVDDDDYFLCLPSNTKFVALACNEKWTYNDSD

>1F6VA

GSRIAKRTAINKTKKADVKAIADAWQINGEKELELLQQIAQKPGALRILNHSLRLAAMTAHGKGE
RVNEDYLRQAFRELDLDVDISTLLRN

>1G5HA

WLSGYAGPADGTQQPDAPEHAVAREALVDLCRRRHFLSGTPQQLSTAALLSGCHARFGPLGVELR
KNLASQWWSSMVVFREQVFAVDSLHQEPGSSQPRDSAFRLVSPESIREILQDREPSKEQLVAFLE
NLLKTSGKLRATLLHGALEHYVNCLDLVNRKLPFGLAQIGVCFHPVSNSNQTPSSVTRVGEKTEA
SLVWFTPTRTSSQWLDFWLRHRLLWWRKFAMSPSNFSSADCQDELGRKGSKLYYSFPWGKEPIET
LWNLGDQELLHTYPGNVSTIQGRDGRKNVVPCVLSVSGDVDLGTLAYLYDSFQLAENSFARKKSL
QRKVLKLHPCLAPIKVALDVGKGPTVELRQVCQGLLNELLENGISVWPGYSETVHSSLEQLHSKY
DEMSVLFSVLVTETTLENGLIQLRSRDTTMKEMMHISKLRDFLVKYLASASNVAAALDHHHHHH

>1GDTB

MRLFGYARVSTSQQSLDIQVRALKDAGVKANRIFTDKASGSSSDRKGLDLLRMKVEEGDVILVKK
LDRLGRDTADMIQLIKEFDAQGVSIRFIDDGISTDGEMGKMVVTILSAVAQAERQRILERTNEGR
QEAMAKGVVFGRKRKIDRDAVLNMWQQGLGASHISKTMNIARSTVYKVINESN

>1HCRA

GRPRAINKHEQEQISRLLEKGHPRQQLAIIFGIGVSTLYRYFPASSIKKRMN

>1HKQA

MVDNKVTQSNKLIESSHTLTLNEKRLVLCAASLIDSRKPLPKDGYLTIRADTFAEVFGIDVKHAY
AALDDAATKLFNRDIRRYVKGKVVERMRWVFHVKYREGQGCVELGFSPTIIPHLTMLHKEFTSYQ
LK

>1HLVA

MGPKRRQLTFREKSRIIQEVEENPDLRKGEIARRFNIPPSTLSTILKNKRAILASERKYGVASTC
RKTNKLSPYDKLEGLLIAWFQQIRAAGLPVKGIILKEKALRIAEELGMDDFTASNGWLDRFRRRR
S

>1HQ3H

MSGRGKGGKGLGKGGAKRHRKVLRDNIQGITKPAIRRLARRGGVKRISGLIYEETRGVLKVFLEN
VIRDAVTYTEHAKRKTVTAMDVVYALKRQGRTLYGFGG

>1I11A

GSPHIKRPMNAFMVWAKDERRKILQAFPDMHNSNISKILGSRWKAMTNLEKQPYYEEQARLSKQH
LEKYPDYKYKPRPKRT

>1IGNB

GALPSHNKASFTDEEDEFILDVVRKNPTRRTTHTLYDEISHYVPNHTGNSIRHRFRVYLSKRLEY
VYEVDKFGKLVRDDDGNLIKTKVLPPSIKRKFSADEDYTLAIAVKKQFYRDLFQIDPDTGRSLIT
DEDTPTAIARRNMTMDPNHVPGSEPNFAAYRTQSRRGPIAREFFKHFAEEHAAHTENAWRDRFRK
FLLAYGIDDYISYYEAEKAQNREPEPMKNLTNRPKRPGVPTPGNYNSAAKR

>1IN4A

MSEFLTPERTVYDSGVQFLRPKSLDEFIGQENVKKKLSLALEAAKMRGEVLDHVLLAGPPGLGKT
TLAHIIASELQTNIHVTSGPVLVKQGDMAAILTSLERGDVLFIDEIHRLNKAVEELLYSAIEDFQ
IDIMIGKGPSAKSIRIDIQPFTLVGATTRSGLLSSPLRSRFGIILELDFYTVKELKEIIKRAASL
MDVEIEDAAAEMIAKRSRGTPRIAIRLTKRVRDMLTVVKADRINTDIVLKTMEVLNIDDEGLDEF
DRKILKTIIEIYRGGPVGLNALAASLGVEADTLSEVYEPYLLQAGFLARTPRGRIVTEKAYKHLK
YEVPENRLF

>1IRZA

TAQKKPRVLWTHELHNKFLAAVDHLGVERAVPKKILDLMNVDKLTRENVASHLQKFRVALKKVS

>1IUFA

GIHMGKIKRRAITEHEKRALRHYFFQLQNRSGQQDLIEWFREKFGKDISQPSVSQILSSKYSYLD
NTVEKPWDVKRNRPPKYPLLEAALFEWQVQQGDDATLSGETIKRAAAILWHKIPEYQDQPVPNFS
NGWLEGFRKRHILH

>1IV6A

MTPEKHRARKRQAWLWEEDKNLRSGVRKYGEGNWSKILLHYKFNNRTSVMLKDRWRTMKKLKLIS
SDSED

>1IXCB

MEFRQLKYFIAVAEAGNMAAAAKRLHVSQPPITRQMQALEADLGVVLLERSHRGIELTAAGHAFL
EDARRILELAGRSGDRSRAAARGDVGELSVAYFGTPIYRSLPLLLRAFLTSTPTATVSLTHMTKD
EQVEGLLAGTIHVGFSRFFPRHPGIEIVNIAQEDLYLAVHRSQSGKFGKTCKLADLRAVELTLFP
RGGRPSFADEVIGLFKHAGIEPRIARVVEDATAALALTMAGAASSIVPASVAAIRWPDIAFARIV
GTRVKVPISCIFRKEKQPPILARFVEHVRRSAKD

>1IYMA

AMDDGVECAVCLAELEDGEEARFLPRCGHGFHAECVDMWLGSHSTCPLCRLTVVV

>1J2FB

GAMGSSLDNPTPFPNLGPSENPLKRLLVPGEEWEFEVTAFYRGRQVFQQTISCPEGLRLVGSEVG
DRTLPGWPVTLPDPGMSLTDRGVMSYVRHVLSCLGGGLALWRAGQWLWAQRLGHCHTYWAVSEEL
LPNSGHGPDGEVPKDKEGGVFDLGPFIVDLITFTEGSGRSPRYALWFCVGESWPQDQPWTKRLVM
VKVVPTCLRALVEMARVGGASSLENTVDLHISNSHPLSLTSDQYKAYLQDLVEGMDFQGPGES

>1JE5B

MAKKIFTSALGTAEPYAYIAKPDYGNEERGFGNPRGVYKVDLTIPNKDPRCQRMVDEIVKCHEEA
YAAAVEEYEANPPAVARGKKPLKPYEGDMPFFDNGDGTTTFKFKCYASFQDKKTKETKHINLVVV
DSKGKKMEDVPIIGGGSKLKVKYSLVPYKWNTAVGASVKLQLESVMLVELATFGGGEDDWADEVE
ENGYVASGSAK

>1JEQB

MVRSGNKAAVVLCMDVGFTMSNSIPGIESPFEQAKKVITMFVQRQVFAENKDEIALVLFGTDGTD
NPLSGGDQYQNITVHRHLMLPDFDLLEDIESKIQPGSQQADFLDALIVSMDVIQHETIGKKFEKR
HIEIFTDLSSRFSKSQLDIIIHSLKKCDISLQFFLPFSLGKEDGSGDRGDGPFRLGGHGPSFPLK
GITEQQKEGLEIVKMVMISLEGEDGLDEIYSFSESLRKLCVFKKIERHSIHWPCRLTIGSNLSIR

IAAYKSILQERVKKTWTVVDAKTLKKEDIQKETVYCLNDDDETEVLKEDIIQGFRYGSDIVPFSK
VDEEQMKYKSEGKCFSVLGFCKSSQVQRRFFMGNQVLKVFAARDDEAAAVALSSLIHALDDLDMV
AIVRYAYDKRANPQVGVAFPHIKHNYECLVYVQLPFMEDLRQYMFSSLKNSKKYAPTEAQLNAVD
ALIDSMSLAKKDEKTDTLEDLFPTTKIPNPRFQRLFQCLLHRALHPREPLPPIQQHIWNMLNPPA
EVTTKSQIPLSKIKTLFPLIEAKKKDQVTAQEIFQDNHEDGPTAK
>1JEQA
MSGWESYYKTEGDEEAEEEQEENLEASGDYKYSGRDSLIFLVDASKAMFESQSEDELTPFDMSIQ
CIQSVYISKIISSDRDLLAVVFYGTEKDKNSVNFKNIYVLQELDNPGAKRILELDQFKGQQGQKR
FQDMMGHGSDYSLSEVLWVCANLFSDVQFKMSHKRIMLFTNEDNPHGNDSAKASRARTKAGDLRD
TGIFLDLMHLKKPGGFDISLFYRDIISIAEDEDLRVHFEESSKLEDLLRKVRAKETRKRALSRLK
LKLNKDIVISVGIYNLVQKALKPPPIKLYRETNEPVKTKTRTFNTSTGGLLLPSDTKRSQIYGSR
QIILEKEETEELKRFDDPGLMLMGFKPLVLLKKHHYLRPSLFVYPEESLVIGSSTLFSALLIKCL
EKEVAALCRYTPRRNIPPYFVALVPQEEELDDQKIQVTPPGFQLVFLPFADDKRKMPFTEKIMAT
PEQVGKMKAIVEKLRFTYRSDSFENPVLQQHFRNLEALALDLMEPEQAVDLTLPKVEAMNKRLGS
LVDEFKELVYPPDYNPEGKVTKRKHDNEGSGSKRPKVEYSEEELKTHISKGTLGKFTVPMLKEAC
RAYGLKSGLKKQELLEALTKHFQD
>1JSPB
GSHMRKKIFKPEELRQALMPTLEALYRQDPESLPFRQPVDPQLLGIPDYFDIVKNPMDLSTIKRK
LDTGQYQEPWQYVDDVWLMFNNAWLYNRKTSRVYKFCSKLAEVFEQEIDPVMQSLG
>1K99A
MKKLKKHPDFPKKPLTPYFRFFMEKRAKYAKLHPEMSNLDLTKILSKKYKELPEKKKMKYIQDFQ
REKQEFERNLARFREDHPDLIQNAKKLEHHHHHH
>1KFTA
MGSSHHHHHHSSGLVPRGSHMNTSSLETIEGVGPKRRQMLLKYMGGLQGLRNASVEEIAKVPGIS
QGLAEKIFWSLKH
>1KIXA
MSTAAKQNRSTSRVSKKKTAAPKEGAAKKSDKGHKYEYVELAKASLTSAQPQHFYAVVIDATFPY
KTNQERYICSLKIVDPTLYLKQQKGAGDASDYATLVLYAKRFEDLPIIHRAGDIIRVHRATLRLY
NGQRQFNANVFYSSSWALFSTDKRSVTQEINNQDAVSDTTPFSFSSKHATIEKNEISILQNLRKW
ANQYFSSYSVISSDMYTALNKAQAQKGDFDVVAKILQVHELDEYTNELKLKDASGQVFYTLSLKL
KFPHVRTGEVVRIRSATYDETSTQKKVLILSHYSNIITFIQSSKLAKELRAKIQDDHSVEVASLK
KNVSLNAVVLTEVDKKHAALPSTSLQDLFHHADSDKELQAQDTFRTQFYVTKIEPSDVKEWVKGY
DRKTKKSSSLKGASGKGDNIFQVQFLVKDASTQLNNNTYRVLLYTQDGLGANFFNVKADNLHKNA
DARKKLEDSAELLTKFNSYVDAVVERRNGFYLIKDTKLIY
>1KKXA
MRGSGSHHHHHHGSNNKQYELFMKSLIENCKKRNMPLQSIPEIGNRKINLFYLYMLVQKFGGADQ
VTRTQQWSMVAQRLQISDYQQLESIYFRILLPYERHMISQEGIKETQAKRILQPSLIS
>1KN0A
MSGTEEAILGGRDSHPAAGGGSVLCFGQCQYTAEEYQAIQKALRQRLGPEYISSRMAGGGQKVCY
IEGHRVINLANEMFGYNGWAHSITQQNVDFVDLNNGKFYVGVCAFVRVQLKDGSYHEDVGYGVSE
GLKSKALSLEKARKEAVTDGLKRALRSFGNALGNCILDKDYLRSLNKLPRQLPLEVDLTKAKRQD
LEPSVEEARYNSCRPNM
>1KNUB
LNPESADLRALAKHLYDSYIKSFPLTKAKARAILTGKTTDKSPFVIYDMNSLMMGEDKIKFKHIT

PLQEQSKEVAIRIFQGCQFRSVEAVQEITEYAKSIPGFVNLDLNDQVTLLKYGVHEIIYTMLASL
MNKDGVLISEGQGFMTREFLKSLRKPFGDFMEPKFEFAVKFNALELDDSDLAIFIAVIILSGDRP
GLLNVKPIEDIQDNLLQALELQLKLNHPESSQLFAKLLQKMTDLRQIVTEHVQLLQVIKKTETDM
SLHPLLQEIYKDLY
>1KU9B
MIIMEEAKKLIIELFSELAKIHGLNKSVGAVYAILYLSDKPLTISDIMEELKISKGNVSMSLKKL
EELGFVRKVWIKGERKNYYEAVDGFSSIKDIAKRKHDLIAKTYEDLKKLEEKCNEEEKEFIKQKI
KGIERMKKISEKILEALNDLDN
>1KW4A
METKRVNGTDRPPISSWSVDDVSNFIRELPGCQDYVDDFIQQEIDGQALLRLKEKHLVNAMGMKL
GPALKIVAKVESIKEVRDHHHHHH
>1KZYD
ALEEQRGPLPLNKTLFLGYAFLLTMATTSDKLASRSKLPDGPTGSSEEEEEFLEIPPFNKQYTES
QLRAGAGYILEDFNEAQCNTAYQCLLIADQHCRTRKYFLCLASGIPCVSHVWVHDSCHANQLQNY
RNYLLPAGYSLEEQRILDWQPRENPFQNLKVLLVSDQQQNFLELWSEILMTGGAASVKQHHSSAH
NKDIALGVFDVVVTDPSCPASVLKCAEALQLPVVSQEWVIQCLIVGERIGFKQHPKYKHDYVSH
>1L3AD
MASMTGGQQMGRGSDYFEPQQQQQQQQQQPQGASTPKVFVGYSIYKGKAALTVEPRSPEFSPLDS
GAFKLSREGMVMLQFAPAAGVRQYDWSRKQVFSLSVTEIGSIISLGTKDSCEFFHDPNKGRSDEG
RVRKVLKVEPLPDGSGHFFNLSVQNKLINLDENIYIPVTKAEFAVLVSAFNFVMPYLLGWHTAVN
SFKPEDASRSNNANPRSGAELEWNLEHHHHHH
>1L8YA
MGKLPESPKRAEEIWQQSVIGDYLARFKNDRVKALKAMEMTWNNMEKKEKLMWIKKAAEDQKRYE
RELSEMRAPPAATNSSKKLEHHHHHH
>1MH3A
KIEEGKLVIWINGDKGYNGLAEVGKKFEKDTGIKVTVEHPDKLEEKFPQVAATGDGPDIIFWAHD
RFGGYAQSGLLAEITPDKAFQDKLYPFTWDAVRYNGKLIAYPIAVEALSLIYNKDLLPNPPKTWE
EIPALDKELKAKGKSALMFNLQEPYFTWPLIAADGGYAFKYENGKYDIKDVGVDNAGAKAGLTFL
VDLIKNKHMNADTDYSIAEAAFNKGETAMTINGPWAWSNIDTSKVNYGVTVLPTFKGQPSKPFVG
VLSAGINAASPNKELAKEFLENYLLTDEGLEAVNKDKPLGAVALKSYEEELAKDPRIAATMENAQ
KGEIMPNIPQMSAFWYAVRTAVINAASGRQTVDAALAAAQTAAAAAISPQARAFLEQVFRRKQSL
NSKEKEEVAKKCGITPLQVRVWFINKRMRSK
>1MOJA
MSTQKNARATAGEVEGSDALRMDADRAEQCVDALNADLANVYYVLYHQLKKHHWNVEGAEFRDLHL
FLGEAAETAEEVADELAERVQALGGVPHASPETLQAEASVDVEDEDVYDIRTSLANDMAIYGDII
EATREHTELAENLGDHATAHMLREGLIELEDDAHHIEHYLEDDTLVTQGALE
>1MP9B
YIIPDEIPYKAVVNIENIVATVTLDQTLDLYAMERSVPNVEYDPDQFPGLIFRLESPKITSLIFK
SGKMVVTGAKSTDELIKAVKRIIKTLKKYGMQLTGKPKIQIQNIVASANLHVIVNLDKAAFLLEN
NMYEPEQFPGLIYRMDEPRVVLLIFSSGKMVITGAKREDEVHKAVKKIFDKLVELDCVKPVEEEE
LEF
>1MSZA
MGSLNGGSPEGVESQDGVDHFRAMIVEFMASKKMQLEFPPSLNSHDRLRVHQIAEEHGLRHDSSG
EGKRRFITVSKRAGSHHHHHH

>1N1JB

GSHMEEIRNLTVKDFRVQELPLARIKKIMKLDEDVKMISAEAPVLFAKAAQIFITELTLRAWIHT
EDNKRRTLQRNDIAMAITKFDQFDFLIDIVPR

>1N1JA

SFREQDIYLPIANVARIMKNAIPQTGKIAKDAKECVQECVSEFISFITSEASERCHQEKRKTING
EDILFAMSTLGFDSYVEPLKLYLQKFRE

>1NGNA

ALSPPRRKSFKKWTPPRSPFNLVQEILFHDPWKLLIATIFLNRTSGKMAIPVLWEFLEKYPSAEV
ARAADWRDVSELLKPLGLYDLRAKTIIKFSDEYLTKQWRYPIELHGIGKYGNDSYRIFCVNEWKQ
VHPEDHKLNKYHDWLWENHEKLSLS

>1NK2P

ASDGLPNKKRKRRVLFTKAQTYELERRFRQQRYLSAPEREHLASLIRLTPTQVKIWFQNHRYKTK
RAQNEKGYEGHP

>1NZPA

MAQPSSQKATNHNLHITEKLEVLAKAYSVQGDKWRALGYAKAINALKSFHKPVTSYQEACSIPGI
GKRMAEKIIEILESGHLRKLDH

>1O57A

MKFRRSGRLVDLTNYLLTHPHELIPLTFFSERYESAKSSISEDLTIIKQTFEQQGIGTLLTVPGA
AGGVKYIPKMKQAEAEEFVQTLGQSLANPERILPGGYVYLTDILGKPSVLSKVGKLFASVFAERE
IDVVMTVATKGIPLAYAAASYLNVPVVIVRKDNKVTEGSTVSINYVSGSSNRIQTMSLAKRSMKT
GSNVLIIDDFMKAGGTINGMINLLDEFNANVAGIGVLVEAEGVDERLVDEYMSLLTLSTINMKEK
SIEIQNGNFLRFFKDNLLKNGETESHHHHHH

>1OQJB

GAMEDMEIAYPITCGESKAILLWKKFVCPGINVKCVKFNDQLISPKHFVHLAGKSTLKDWKRAIR
LGGIMLRKMMDSGQIDFYQHDKVCSNTCRSTK

>1OSVB

AELTVDQQTLLDYIMDSYSKQRMPQEITNKILKEEFSAEENFLILTEMATSHVQILVEFTKRLPG
FQTLDHEDQIALLKGSAVEAMFLRSAEIFNKKLPAGHADLLEERIRKSGISDEYITPMFSFYKSV
GELKMTQEEYALLTAIVILSPDRQYIKDREAVEKLQEPLLDVLQKLCKIYQPENPQHFACLLGRL
TELRTFNHHHAEMLMSWRVNDHKFTPLLCEIWDVQ

>1OY3D

VFGYVTEDGDTALHLAVIHQHEPFLDFLLGFSAGHEYLDLQNDLGQTALHLAAILGEASTVEKLY
AAGAGVLVAERGGHTALHLACRVRAHTCACVLLQPRPSHPRDASDTYLTQSQDCTPDTSHAPAAV
DSQPNPENEEEPRDEDWRLQLEAENYDGHTPLHVAVIHKDAEMVRLLRDAGADLNKPEPTCGRTP
LHLAVEAQAASVLELLLKAGADPTARMYGGRTPLGSALLRPNPILARLLRAHGAPEPEDGGDKLS
PCSSSGSDSDSDNRDEGDEYDD

>1OY3B

TAELKICRVNRNSGSCLGGDEIFLLCDKVQKEDIEVYFTGPGWEARGSFSQADVHRQVAIVFRTP
PYADPSLQAPVRVSMQLRRPSDRELSEPMEFQYLPDTDDRHRIEEKRKRTYETFKSIMKKSPFNG
PTEPRP

>1P1AA

GSHMQVTLKTLQQQTFKIDIDPEETVKALKEKIESEKGKDAFPVAGQKLIYAGKILNDDTALKEY
KIDEKNFVVVMVTKPKAVST

>1P4EC

SQFDILCKTPPKVLVRQFVERFERPSGEKIASCAAELTYLCWMITHNGTAIKRATFMSYNTIISN
SLSFDIVNKSLQFKYKTQKATILEASLKKLIPAWEFTIIPYNGQKHQSDITDIVSSLQLQFESSE
EADKGNSHSKKMLKALLSEGESIWEITEKILNSFEYTSRFTKTKTLYQFLFLATFINCGRFSDIK
NVDPKSFKLVQNKYLGVIIQCLVTETKTSVSRHIYFFSARGRIDPLVYLDEFLRNSEPVLKRVNR
TGNSSSNKQEYQLLKDNLVRSYNKALKKNAPYPIFAIKNGPKSHIGRHLMTSFLSMKGLTELTNV
VGNFSDKRASAVARTTYTHQITAIPDHYFALVSRYYAYDPISKEMIALKDETNPIEEWQHIEQLK
GSAEGSIRYPAWNGIISQEVLDYLSSYINRRIGHHHHHH

>1P4WA

MRGSHHHHHHGSYTPESVAKLLEKISAGGYGDKRLSPKESEVLRLFAEGFLVTEIAKKLNRSIKT
ISSQKKSAMMKLGVDNDIALLNYLSSVSMTPVDK

>1P92A

MKDLVDTTEMYLRTIYELEEEGVTPLRARIAERLEQSGPTVSQTVARMERDGLVVVASDRSLQMT
PTGRTLATAVMRKARLAERLLTDIIGLDINKVHDEACRWEHVMSDEVERRLVKVLKDVSRSPFGN
PIPGLDELGVGNSDAAAPGTRVIDAATSMPRKVRIVQINEIFQVETDQFTQLLDADIRVGSEVEI
VDRDGHITLSHNGKDVELLDDLAHTIRIEEL

>1PGZA

SKSESPKEPEQLRKLFIGGLSFETTDESLRSHFEQWGTLTDCVVMRDPNTKRSRGFGFVTYATVE
EVDAAMNARPHKVDGRVVEPKRAVSREDSQRPGAHLTVKKIFVGGIKEDTEEHHLRDYFEQYGKI
EVIEIMTDRGSGKKRGFAFVTFDDHDSVDKIVIQKYHTVNGHNCEVRKALSKQEMASASSSQRGR

>1PH1B

PQQQSAFKQLYTELFNNEGDFSKVSSNLKKPLKCYVKESYPHFLVTDGYFFVAPYFTKEAVNEFH
AKFPNVNIVDLTDKVIVINNWSLELRRVNSAEVFTSYANLEARLIVHSFKPNLQERLNPTRYPVN
LFRDDEFKTTIQHFRHTALQAAINKTVKGDNLVDISKVADAAGKKGKVDAGIVKASASKGDEFSD
FSFKEGNTATLKIADIFVQEKG

>1PL5S

SNTTEILTSVDVLGTHSQTGTQQSNMYTSTQKTELEIDNKDSVTECSKDMKEDGLSFVDIVLSKA
ASALDEKEKQLAVANEIIRSLSDEVMRNEIRITSLQGDLTFTKKCLENARSQISEKDAKINKLME
KDFQVNKEIKPY

>1POGA

RGSHMRRRKKRTSIETNIRVALEKSFLENQKPTSEEITMIADQLNMEKEVIRVWFCNRRQKEKRI
DI

>1PVEA

GSHMPLEFLRNQPQFQQMRQIIQQNPSLLPALLQQIGRENPQLLQQISQHQEHFIQMLNEPVQEA
GGQGGGG

>1Q1VA

DEPLIKKLKKPPTDEELKETIKKLLASANLEEVTMKQICKKVYENYPTYDLTERKDFIKTTVKEL
ISLEH

>1Q87B

PVNTKRSNGTKRVEFPTTKKSMCIGNSTPNEQETFRAKVDEIWFRLTQKTDGTVMRDFLIEKAAE
YFKQPEQPKQNAIEVISAIMAPQEEQTKSKADLYKFLAMFGPYETIMLKIASLLLISNNKGHWLT
FDPQAEKNANNQRDSISGWFDQNEPNCLILKTPTGIRKIWNKPLIEATGQYLMDENGEKYDSWDK
YFEMKPIETYLTAYPTFAPMHHHHHH

>1QZGB

GPGGEDVIDSLQLNELLNAGEYKIGELTFQSIRSSQELQKKNTIVNLFGIVKDFTPSRQSLHGTK

DWVTTVYLWDPTCDTSSIGLQIHLFSKQGNDLPVIKQVGQPLLLHQITLRSYRDRTQGLSKDQFR
YALWPDFSSNSKDTLCPQPMPRLMKTGDKEEQFALLLNKIWDEQTNKHKNGELLSTS
>1QZQB
MEEYMPTEHHHHHHENLYFQGTSGEGQDIWDMLDKGNPFQFYLTRVSGVKPKYNSGALHIKDILS
PLFGTLVSSAQFNYCFDVDWLVKQYPPEFRKKPILLVHGDKREAKAHLHAQAKPYENISLCQAKL
DIAFGTHHTKMMLLLYEEGLRVVIHTSNLIHADWHQKTQGIWLSPLYPRIADGTHKSGESPTHFK
ADLISYLMAYNAPSLKEWIDVIHKHDLSETNVYLIGSTPGRFQGSQKDNWGHFRLKKLLKDHASS
MPNAESWPVVGQFSSVGSLGADESKWLCSEFKESMLTLGKESKTPGKSSVPLYLIYPSVENVRTS
LEGYPAGGSLPYSIQTAEKQNWLHSYFHKWSAETSGRSNAMPHIKTYMRPSPDFSKIAWFLVTSA
NLSKAAWGALEKNGTQLMIRSYELGVLFLPSAFGLDSFKVKQKFFAGSQEPMATFPVPYDLPPEL
YGSKDRPWIWNIPYVKAPDTHGNMWVPS
>1R5KC
MDPMIKRSKKNSLALSLTADQMVSALLDAEPPILYSEYDPTRPFSEASMMGLLTNLADRELVHMI
NWAKRVPGFVDLTLHDQVHLLECAWLEILMIGLVWRSMEHPGKLLFAPNLLLDRNQGKCVEGMVE
IFDMLLATSSRFRMMNLQGEEFVCLKSIILLNSGVYTFLSSTLKSLEEKDHIHRVLDKITDTLIH
LMAKAGLTLQQQHQRLAQLLLILSHIRHMSNKGMEHLYSMKCKNVVPLYDLLLEMLDAHRLHAPT
S
>1RH6B
MYLTLQEWNARQRRPRSLETVRRWVRESRIFPPPVKDGREYLFHESAVKVDLNRP
>1RI7A
MGSSHHHHHHSSGLVPRGSHMRVPLDEIDKKIIKILQNDGKAPLREISKITGLAESTIHERIRKL
RESGVIKKFTAIIDPEALGYSMLAFILVKVKAGKYSEVASNLAKYPEIVEVYETTGDYDMVVKIR
TKNSEELNNFLDLIGSIPGVEGTHTMIVLKTHKETTELPIK
>1RIFB
MDIKVHFHDFSHVRIDCEESTFHELRDFFSFEADGYRFNPRFRYGNWDGRIRLLDYNRLLPFGLV
GQIKKFCDNFGYKAWIDPQINEKEELSRKDFDEWLSKLEIYSGNKRIEPHWYQKDAVFEGLVNRR
RILNLPTSAGRSLIQALLARYYLENYEGKILIIVPTTALTTQMADDFVDYRLFSHAMIKKIGGGA
SKDDKYKNDAPVVVGTWQTVVKQPKEWFSQFGMMMNDECHLATGKSISSIISGLNNCMFKFGLSG
SLRDGKANIMQYVGMFGEIFKP
>1RW2A
MHHHHHHKLKTEQGGAHFSVSSLAEGSVTSVGSVNPAENFRVLVKQKKASFEEASNQLINHIEQF
LDTNETPYFMKSIDCIRAFREEAIKFSEEQRFNNFLKALQEKVEIKQLNHFWEIVVQDGITLITK
EEASGSSVTAEEAKKFLAPKDK
>1S6MA
MLSHMVLTRQDIGRAASYYEDGADDYYAKDGDASEWQGKGAEELGLSGEVDSKRFRELLAGNIGE
GHRIMRSATRQDSKERIGLDLTFSAPKSVSLQALVAGDAEIIKAHDRAVARTLEQAEARAQARQK
IQGKTRIETTGNLVIGKFRHETSRERDPQLHTHAVILNMTKRSDGQWRALKNDEIVKATRYLGAV
YNAELAHELQKLGYQLRYGKDGNFDLAHIDRQQIEGFSKRTEQIAEWYAARGLDPNSVSLEQKQA
AKVLSRAKKTSVDREALRAEWQATAKELGIDFS
>1SD4B
MTNKQVEISMAEWDVMNIIWDKKSVSANEIVVEIQKYKEVSDKTIRTLITRLYKKEIIKRYKSEN
IYFYSSNIKEDDIKMKTAKTFLNKLYGGDMKSLVLNFAKNEELNNKEIEELRDILNDISKK
>1SE8A
MARGMNHVYLIGALARDPELRYTGNGMAVFEATVAGEDRVIGNDGRERNLPWYHRVSILGKPAEW

QAERNLKGGDAVVVEGTLEYRQWEAPEGGKRSAVNVKALRMEQLGTQPELIQDAGGGVRMSGAMN
EVLVLGNVTRDPEIRYTPAGDAVLSLSIAVNENYQDRQGQRQEKVHYIDATLWRDLAENMKELRK
GDPVMIMGRLVNEGWTDKDGNKRNSTRVEATRVEALARGAGNANSGYAAATPAAPRTQTASSAAR
PTSGGYQSQPSRAANTGSRSGGLDIDQGLDDFPPEEDDLPF
>1SFUB
MDLLSCTVNDAEIFSLVKKEVLSLNTNDYTTAISLSNRLKINKKKINQQLYKLQKEDTVKMVPSN
PPKWFKNYNC
>1SQ8A
MLMGERIRARRIQLGLNQAELAQKVGVDQQAIEQLENGKAKRPRFLPELARALGVAVDWLLNGA
>1T0FC
GSAIKVVKPSDWDSLPDTDLRYIYSQRQPEKTMHERLKGKGVIVDMASLFKQAG
>1T0FA
GSAMAKANSSFSEVQIARRIKEGRGQGHGKDYIPWLTVQEVPSSGRSHRIYSHKTGRVHHLLSDL
ELAVFLSLEWESSVLDIREQFPLLPSDTRQIAIDSGIKHPVIRGVDQVMSTDFLVDCKDGPFEQF
AIQVKPAAALQDERTLEKLELERRYWQQKQIPWFIFTDKEINPVVKENIEWLYSVKTEEVSAELL
AQLSPLAHILQEKGDENIINVCKQVDIAYDLELGKTLSEIRALTANGFIKFNIYKSFRANKCADL
CISQVVNMEELRYVAN
>1T23A
SNTRNFVLRDEDGNEHGVFTGKQPRQAALKAANRGSGTKANPDIIRLRERGTKKVHVFKAWKEIV
DAPKNRPAWMPEKISKPFVKKERIEKLE
>1U2WD
MKKKDTCEIFGYDEEKVNRIQGDLQTVDISGVSQILKAIADENRAKITYALCQDEELCVCDIANI
LGVTIANASHHLRTLYKQGVVNFRKEGKLALYSLGDEHIRQIMMIALAHKKEVKVNV
>1U3EM
MEWKDIKGYEGHYQVSNTGEVYSIKSGKTLKHQIPKDGYHRIGLFKGGKGKTFQVHRLVAIHFCE
GYEEGLVVDHKDGNKDNNLSTNLRWVTQKINVENQMSRGTLNVSKAQQIAKIKNQKPIIVISPDG
IEKEYPSTKCACEELGLTRGKVTDVLKGHRIHHKGYTFRYKLNG
>1U78A
MPRGSALSDTERAQLDVMKLLNVSLHEMSRKISRSRHCIRVYLKDPVSYGTSKRAPRRKALSVRD
ERNVIRAASNSCKTARDIRNELQLSASKRTILNVIKRSGVIVRQKLRPAPLLSADHKLKRLEFAK
NNMGTHHHHHH
>1U9NA
MGSSHHHHHHSSGLVPRGSHVTTSAASQASLPRGRRTARPSGDDRELAILATAENLLEDRPLADI
SVDDLAKGAGISRPTFYFYFPSKEAVLLTLLDRVVNQADMALQTLAENPADTDRENMWRTGINVF
FETFGSHKAVTRAGQAARATSVEVAELWSTFMQKWIAYTAAVIDAERDRGAAPRTLPAHELATAL
NLMNERTLFASFAGEQPSVPEARVLDTLVHIWVTSIYGENR
>1UB4C
GPHMIHSSVKRWGNSPAVRIPATLMQALNLNIDDEVKIDLVDGKLIIEPVRKEPVFTLAELVNDI
TPENLHENIDWGEPKDKEVW
>1UB4B
VSRYVPDMGDLIWVDFDPTKGSEQAGHRPAVVLSPFMYNNKTGMCLCVPCTTQSKGYPFEVVLSG
QERDGVALADQVKSIAWRARGATKKGTVAPEELQLIKAKINVLIG
>1UDVB
MTEKLNEIVVRKTKNVEDHVLDVIVLFNQGIDEVILKGTGREISKAVDVYNSLKDRLGDGVQLVN

VQTGSEVRDRRRISYILLRLKRVY

>1UFID

GSHMPVPSFGEAMAYFAMVKRYLTSFPIDDRVQSHILHLEHDLVHVTRKNHARQAGVRGLGHQS

>1UKLF

RSSINDKIIELKDLVMGTDAKMHKSGVLRKAIDYIKYLQQVNHKLRQENMVLKLANQKNKL

>1UKLB

MELITILEKTVSPDRLELEAAQKFLERAAVENLPTFLVELSRVLANPGNSQVARVAAGLQIKNSL
TSKDPDIKAQYQQRWLAIDANARREVKNYVLQTLGTETYRPSSASQCVAGIACAEIPVSQWPELI
PQLVANVTNPNSTEHMKESTLEAIGYICQDIDPEQLQDKSNEILTAIIQGMRKEEPSNNVKLAAT
NALLNSLEFTKANFDKESERHFIMQVVCEATQCPDTRVRVAALQNLVKIMSLYYQYMETYMGPAL
FAITIEAMKSDIDEVALQGIEFWSNVCDEEMDLAIEASEAAEQGRPPEHTSKFYAKGALQYLVPI
LTQTLTKQDENDDDDDWNPCKAAGVCLMLLSTCCEDDIVPHVLPFIKEHIKNPDWRYRDAAVMAF
GSILEGPEPNQLKPLVIQAMPTLIELMKDPSVVVRDTTAWTVGRICELLPEAAINDVYLAPLLQC
LIEGLSAEPRVASNVCWAFSSLAEAAYEAADVADDQEEPATYCLSSSFELIVQKLLETTDRPDGH
QNNLRSSAYESLMEIVKNSAKDCYPAVQKTTLVIMERLQQVLQMESHIQSTSDRIQFNDLQSLLC
ATLQNVLRKVQHQDALQISDVVMASLLRMFQSTAGSGGVQEDALMAVSTLVEVLGGEFLKYMEAF
KPFLGIGLKNYAEYQVCLAAVGLVGDLCRALQSNILPFCDEVMQLLLENLGNENVHRSVKPQILS
VFGDIALAIGGEFKKYLEVVLNTLQQASQAQVDKSDFDMVDYLNELRESCLEAYTGIVQGLKGDQ
ENVHPDVMLVQPRVEFILSFIDHIAGDEDHTDGVVACAAGLIGDLCTAFGKDVLKLVEARPMIHE
LLTEGRRSKTNKAKTLATWATKELRKLKNQA

>1UL1Z

GIQGLAKLIADVAPSAIRENDIKSYFGRKVAIDASMSIYQFLIAVRQGGDVLQNEEGETTSHLMG
MFYRTIRMMENGIKPVYVFDGKPPQLKSGELAKRSERRAEAEKQLQQAQAAGAEQEVEKFTKRLV
KVTKQHNDECKHLLSLMGIPYLDAPSEAEASCAALVKAGKVYAAATEDMDCLTFGSPVLMRHLTA
SEAKKLPIQEFHLSRILQELGLNQEQFVDLCILLGSDYCESIRGIGPKRAVDLIQKHKSIEEIVR
RLDPNKYPVPENWLHKEAHQLFLEPEVLDPESVELKWSEPNEEELIKFMCGEKQFSEERIRSGVK
RLSKSRQGSTQGRLDDFFKVTGSLSSAKRKEPEPKGSTKKKAKTGAAGKFKRGK

>1UL4A

GSSGSSGLRLCQVDRCTADMKEAKLYHRRHKVCEVHAKASSVFLSGLNQRFCQQCSRFHDLQEFD
EAKRSCRRRLAGHNERRRKSSGESGPSSG

>1ULYA

MAKKVKVITDPEVIKVMLEDTRRKILKLLRNKEMTISQLSEILGKTPQTIYHHIEKLKEAGLVEV
KRTEMKGNLVEKYYGRTADVFYINLYLGDEELRYIARSRLKTKIDIFKRLGYQFEENELLNIMDR
MSQKEFDATVRISKYIEEKEDALKDFSNEDIIHAIEWLSTAELARDEEYLELLKRLGSILKR

>1USTA

KKEEASSKSYRELIIEGLTALKERKGSSRPALKKFIKENYPIVGSASNFDLYFNNAIKKGVEAGD
FEQPKGPAGAVKLAKKKSPEVKKEKEVS

>1UVHD

MTSFTIPGLSDKKASDVADLLQKQLSTYNDLHLTLKHVHWNVVGPNFIGVHEMIDPQVELVRGYA
DEVAERIATLGKSPKGTPGAIIKDRTWDDYSVERDTVQAHLAALDLVYNGVIEDTRKSIEKLEDL
DLVSQDLLIAHAGELEKFQWFVRAHLESAGGQLTHEGQSTEKGAADKARRKSA

>1V63A

GSSGSSGPKKPPMNGYQKFSQELLSNGELNHLPLKERMVEIGSRWQRISQSQKEHYKKLAEEQQR
QYKVHLDLWVKSLSPQDRAAYKEYISNKRKSGPSSG

>1VJFA
MGSDKIHHHHHHMKTRADLFAFFDAHGVDHKTLDHPPVFRVEEGLEIKAAMPGGHTKNLFLKDAK
GQLWLISALGETTIDLKKLHHVIGSGRLSFGPQEMMLETLGVTPGSVTAFGLINDTEKRVRFVLD
KALADSDPVNFHPLKNDATTAVSQAGLRRFLAALGVEPMIVDFAAMEVVG
>1WEOA
GSSGSSGPKPLKNLDGQFCEICGDQIGLTVEGDLFVACNECGFPACRPCYEYERREGTQNCPQCK
TRYKRLRGSPRVEGDEDEEDIDSGPSSG
>1WEPA
GSSGSSGMALVPVYCLCRQPYNVNHFMIECGLCQDWFHGSCVGIEEENAVDIDIYHCPDCEAVFG
PSIMKNWHSGPSSG
>1WEUA
GSSGSSGSPEYGMPSVTFGSVHPSDVLDMPVDPNEPTYCLCHQVSYGEMIGCDNPDCSIEWFHFA
CVGLTTKPRGKWFCPRCSQESGPSSG
>1WEWA
GSSGSSGEDPFQPEIKVRCVCGNSLETDSMIQCEDPRCHVWQHVGCVILPDKPMDGNPPLPESFY
CEICRLTSGPSSG
>1WG2A
GSSGSSGPSRPVRPNNRCFSCNKKVGVMGFKCKCGSTFCGSHRYPEKHECSFDFKEVGSGPSSG
>1WG6A
GSSGSSGLKGEPDCYALSLESSEQLTLEIPLNDSGSAGLGVSLKGNKSRETGTDLGIFIKSIIHG
GAAFKDGRLRMNDQLIAVNGETLLGKSNHEAMETLRRSMSMEGNIRGMIQLVILRRSGPSSG
>1WH5A
GSSGSSGSSAEAGGGIRKRHRTKFTAEQKERMLALAERIGWRIQRQDDEVIQRFCQETGVPRQVL
KVWLHNNKHSGPSSG
>1WI3A
GSSGSSGPRSRTKISLEALGILQSFIHDVGLYPDQEAIHTLSAQLDLPKHTIIKFFQNQRYHVKH
SGPSSG
>1WIJA
GSSGSSGSQFVLQDLQDATLGSLLSSLMQHCDPPQRKYPLEKGTPPPWWPTGNEEWWVKLGLPKS
QSPPYRKPHDLKKMWKVGVLTAVINHMLPDIAKIKRHVRQSKCLQDKMTAKESAIWLAVLNQEES
LIQQSGPSSG
>1WJ2A
GSSGSSGVQTTSEVDLLDDGYRWRKYGQKVVKGNPYPRSYYKCTTPGCGVRKHVERAATDPKAVV
TTYEGKHNHDLPA
>1WJVA
GSSGSSGMVFFTCNACGESVKKIQVEKHVSNCRNCECLSCIDCGKDFWGDDYKSHVKCISEGQKY
GGKGYEAKSGPSSG
>1WPKA
MKKATCLTDDQRWQSVLARDPNADGEFVFAVRTTGIFCRPSCRARHALRENVSFYANASEALAAG
FRPCKRCQPEKANAQQHRLDKITHACRLLEQETPVTLEALADQVAMSPFHLHRLFKATTGMTPKA
WQQAWRARRLRESLAK
>1X3CA
GSSGSSGRKKPVSQSLEFPTRYSPYRPYRCVHQGCFAAFTIQQNLILHYQAVHKSDLPAFSAEVE
EESGPSSG

>1X51A

GSSGSSGPRKASRKPPREESSATCVLEQPGALGAQILLVQRPNSGLLAGLWEFPSVTWEPSEQLQ
RKALLQELQRWAGPLPATHLRHLGEVVHTFSHIKLTYQVYGLALEGQTPVTTVPPGARWLTQEEF
HTAAVSTAMKKVFRVYQGQSGPSSG

>1X57A

GSSGSSGDRVTLEVGKVIQQGRQSKGLTQKDLATKINEKPQVIADYESGRAIPNNQVLGKIERAI
GLKLRGKDIGKPIEKGPRAKSGPSSG

>1X6FA

GSSGSSGLKRDFIILGNGPRLQNSTYQCKHCDSKLQSTAELTSHLNIHNEEFQKRAKRQERRKQL
LSKQKYADGAFADFKQESGPSSG

>1XCBA

MKVPEAAISRLITYLRILEELEAQGVHRTSSEQLGELAQVTAFQVRKDLSYFGSYGTRGVGYTVP
VLKRELRHILGLNRKWGLCIVGMGRLGSALADYPGFGESFELRGFFDVDPEKVGRPVRGGVIEHV
DLLPQRVPGRIEIALLTVPREAAQKAADLLVAAGIKGILNFAPVVLEVPKEVAVENVDFLAGLTR
LSFAILNPKWREEMMG

>1XD7A

MSLINSRLAVAIHILSLISMDEKTSSEIIADSVNTNPVVVRRMISLLKKADILTSRAGVPGASLK
KDPADISLLEVYRAVQKQEELFAVHENPNPKCPVGKKIQNALDETFESVQRAMENELASKSLKDV
MNHLFEGGSHHHHHH

>1XNAA

MPEIRLRHVVSCSSQDSTHCAENLLKADTYRKWRAAKAGEKTISVVLQLEKEEQIHSVDIGNDGS
AFVEVLVGSSAGGAGEQDYEVLLVTSSFMSPSESRSGSNPNRVRMFGPDKLVRAAAEKRWDRVKI
VCSQPYSKDSPFGLSFVRFHSPPDKDEAEAPSQKVTVTKLGQFRVKEEEESAN

>1XP8A

GSHMSKDATKEISAPTDAKERSKAIETAMSQIEKAFGKGSIMKLGAESKLDVQVVSTGSLSLDLA
LGVGGIPRGRITEIYGPESGGKTTLALAIVAQAQKAGGTCAFIDAEHALDPVYARALGVNTDELL
VSQPDNGEQALEIMELLVRSGAIDVVVVDSVAALTPRAEIEGDMGDSLPGLQARLMSQALRKLTA
ILSKTGTAAIFINQVREKIGVMYGNPETTTGGRALKFYASVRLDVRKIGQPTKVGNDAVANTVKI
KTVKNKVAAPFKEVELALVYGKGFDQLSDLVGLAADMDIIKKAGSFYSYGDERIGQGKEKTIAYI
AERPEMEQEIRDRVMAAIRAGNAGEAPALAPAPAAPEAAEA

>1XV9B

PVQLSKEQEELIRTLLGAHTRHMGTMFEQFVQFRPPAHLFIHHQPLPTLAPVLPLVTHFADINTF
MVLQVIKFTKDLPVFRSLPIEDQISLLKGAAVEICHIVLNTTFCLQTQNFLCGPLRYTIEDGARV
GFQVEFLELLFHFHGTLRKLQLQEPEYVLLAAMALFSPDRPGVTQRDEIDQLQEEMALTLQSYIK
GQQRRPRDRFLYAKLLGLLAELRSINEAYGYQIQHIQGLSAMMPLLQEICS

>1XWRD

MVRANKRNEALRIESALLNKIAMLGTEKTAEAVGVDKSQISRWKRDWIPKFSMLLAVLEWGVVDD
DMARLARQVAAILTNKKRPAATERSEQIQMEF

>1Y6UA

AGHMKQTDIPIWERYTLTIEEASKYFRIGENKLRRLAEENKNANWLIMNGNRIQIKRKQFEKIID
TLDAI

>1YD6A

MNERLKEKLAVLPEQPGCYLMKDKHGTVIYVGKAKSLKERVRSYFTGTHDGKTQRLVEEIADFEY
IVTSSNAEALILEMNLIKKHDPKYNVMLKDDKSY

>1YDXA
MGHHHHHHHHHHSSGHIDDDDKHMTPKLKLNNNINWTKRTIDSLFDLKKGEMLEKELITPEGKYE
YFNGGVKNSGRTDKFNTFKNTISVIVGGSCGYVRLADKNFFCGQSNCTLNLLDPLELDLKFAYYA
LKSQQERIEALAFGTTIQNIRISDLKELEIPFTSNKNEQHAIANTLSVFDERLENLASLIEINRK
LRDEYAHKLFSLDEAFLSHWKLEALQSQMHEITLGEIFNFKSGKYLKSEERLEEGKFPYYGAGID
NTGFVAEPNTEKDTISIISNGYSLGNIRYHEIPWFNGTGSIALEPMNNEIYVPFFYCALKYLQKD
IKERMKSDDSPFLSLKLAGEIKVPYVKSFQLQRKAGKIVFLLDQKLDQYKKELSSLTVIRDTLLK
KLFPDMTERTKSIKDY
>1YIOA
MTAKPTVFVVDDDMSVREGLRNLLRSAGFEVETFDCASTFLEHRRPEQHGCLVLDMRMPGMSGIE
LQEQLTAISDGIPIVFITAHGDIPMTVRAMKAGAIEFLPKPFEEQALLDAIEQGLQLNAERRQAR
ETQDQLEQLFSSLTGREQQVLQLTIRGLMNKQIAGELGIAEVTVKVHRHNIMQKLNVRSLANLVH
LVEKYESFERGVS
>1YJMC
MSQLGSRGRLWLQSPTGGPPPIFLPSDGQALVLGRGPLTQVTDRKCSRNQVELIADPESRTVAVK
QLGVNPSTVGVHELKPGLSGSLSLGDVLYLVNGLYPLTLRWEELS
>1YSEA
GSHVSRSMNKPLEQQVSTNTEVSSEIYQWVRDELKRAGISQAVFARVAFNRTQGLLSEILRKEED
PKTASQSLLVNLRAMQNFLQLPEAERDRIYQDERERSLNAASAMGPAPLISTPPSRPPQVKTATI
ATERNGKPENN
>1YUAA
MNGEVAPPKEDPVPLPELPCEKSDAYFVLRDGAAGVFLAANTFPKSRETRAPLVEELYRFRDRLP
EKLRYLADAPQQDPEGNKTMVRFSRKTKQQYVSSEKDGKATGWSAFYVDGKWVEGKK
>1YUIA
PKAKRAKHPPGTEKPRSRSQSEQPATCPICYAVIRQSRNLRRHLELRHFAKPGV
>1Z1BB
MGRRRSHERRDLPPNLYIRNNGYYCYRDPRTGKEFGLGRDRRIAITEAIQANIELFSGHKHKPLT
ARINSDNSVTLHSWLDRYEKILASRGIKQKTLINYMSKIKAIRRGLPDAPLEDITTKEIAAMLNG
YIDEGKAASAKLIRSTLSDAFREAIAEGHITTNHVAATRAAKSKVRRSRLTADEYLKIYQAAESS
PCWLRLAMELAVVTGQRVGDLCEMKWSDIVDGYLYVEQSKTGVKIAIPTALHIDALGISMKETLD
KCKEILGGETIIASTRREPLSSGTVSRYFMRARKASGLSFEGDPPTFHELRSLSARLYEKQISDK
FAQHLLGHKSDTMASQYRDDRGREWDKIEIK
>1Z4HA
MQHELQPDSLVDLKFIMADTGFGKTFIYDRIKSGDLPKAKVIHGRARWLYRDHCEFKNKLLSRAN
G
>1Z91A
MENKFDHMKLENQLSFLLYASSREMTKQYKPLLDKLNITYPQYLALLLLWEHETLTVKKMGEQLY
LDSGTLTPMLKRMEQQGLITRKRSEEDERSVLISLTEDGALLKEKAVDIPGTILGLSKQSGEDLK
QLKSALYTLLETLHQKN
>1ZAEB
HMDKTVNLSACEVAVLDLYEQSNIRIPSDIIEDLVNQRLQSEQEVLNYIETQRTYWKLENQKKLY
RGSLK
>1ZI0B
TQEDVVVTLSHQGYVKYQPLSEYEAQRRGGKGKSAARIKEEDFIDRLLVANTHDHILCFSSRGRV

YSMKVYQLPEATRGARGRPIVNLLPLEQDERITAILPVTEFEEGVKVFMATANGTVKKTVLTEFN
RLRTAGKVAIKLVDGDELIGVDLTSGEDEVMLFSAEGKVVRFKESSVRAMGCNTTGVRGIRLGEG
DKVVSLIVPRGDGAILTATQNGYGKRTAVAEYPTKSRATKGVISIKVTERNGLVVGAVQVDDCDQ
IMMITDAGTLVRTRVSEISIVGRNTQGVILIRTAEDENVVGLQRVAE
>1ZP7B
MIRYPNGKTFQPKHSVSSQNSQKRAPSYSNRGMTLEDDLNETNKYYLTNQIAVIHKKPTPVQIVN
VHYPKRSAAVIKEAYFKQSSTTDYNGIYKGRYIDFEAKETKNKTSFPLQNFHDHQIEHMKQVKAQ
DGICFVIISAFDQVYFLEADKLFYFWDRKEKNGRKSIRKDELEETAYPISLGYAPRIDYISIIEQ
LYFSPSSGAKG
>1ZRJA
GSSGSSGMDVRRLKVNELREELQRRGLDTRGLKAELAERLQAALSGPSSG
>1ZS3A
MITKLMIDEKYAKELDKAEIDHHKPTAGAMLGHVLSNLFIENIRLTQAGIYAKSPVKCEYLREIA
QREVEYFFKISDLLLDENEIVPSTTEEFLKYHKFITEDPKAKYWTDEDLLESFIVDFQAQNMFIT
RAIKLANKEEKFALAAGVVELYGYNLQVIRNLAGDLGKSVADFHDEDEDNDN
>1ZZKA
GAMGPIITTQVTIPKDLAGSIIGKGGQRIKQIRHESGASIKIDEPLEGSEDRIITITGTQDQIQN
AQYLLQNSVKQYSGKFF
>2A1IA
MGSSHHHHHHSQDPAKSNSIIVSPRQRGNPVLKFVRNVPWEFGDVIPDYVLGQSTCALFLSLRYH
NLHPDYIHGRLQSLGKNFALRVLLVQVDVKDPQQALKELAKMCILADCTLILAWSPEEAGRYLET
YKAYEQKPADLLMEKL
>2A1JB
MGSSHHHHHHSQDPADLLMEKLEQDFVSRVTECLTTVKSVNKTDSQTLLTTFGSLEQLIAASRED
LALCPGLGPQKARRLFDVLHEPFLKV
>2A1JA
MPQDFLLKMPGVNAKNCRSLMHHVKNIAELAALSQDELTSILGNAANAKQLYDFIHTSFAEVV
>2ADLB
MKQRITVTVDSDSYQLLKAYDVNISGLVSTTMQNEARRLRAERWKVENQEGMVEVARFIEMNGSF
ADENKDW
>2AIFA
GSSQNEASEDTGFNPKAFPLASPDLNNKIINLVQQACNYKQLRKGANEATKALNRGIAEIVLLAA
DAEPLEILLHLPLVCEDKNTPYVFVRSKVALGRACGVSRPVIAAAITSKDGSSLSSQITELKDQI
EQILV
>2ALCA
GSMADTRRRQNHSCDPCRKGKRRCDAPENRNEANENGWVSCSNCKRWNKDCTFNWLSSQRSKNSS
>2AN7B
MSRLTIDMTDQQHQSLKALAALQGKTIKQYALERLFPGDADADQAWQELKTMLGNRINDGLAGKV
STKSVGEILDEELSGDRA
>2AQLB
MNRVEVKVKIPEELKPWLVDDWDLITRQKQLFYLPAKKNVDSILEDYANYKKSRGNTDNKEYAVN
EVVAGIKEYFNVMLGTQLLYKFERPQYAEILADHPDAPMSQVYGAPHLLRLFVRIGAMLAYTPLD
EKSLALLLNYLHDFLKYLAKNSATLFSASDYEVAPPEYHRKAV
>2ATQB

MKGFSSEDKGEWKLKLDASGNGQAVIRFLPAKTDDALPFAILVNHGFKKNGKWYIETCSSTHGDY
DSCPVCQYISKNDLYNTNKTEYSQLKRKTSYWANILVVKDPQAPDNEGKVFKYRFGKKIWDKINA
MIAVDTEMGETPVDVTCPWEGANFVLKVKQVSGFSNYDESKFLNQSAIPNIDDESFQKELFEQMV
DLSEMTSKDKFKSFEELNTKFNQVLGTAALGGAAAAAAS
>2ATQA
MKEFYLTVEQIGDSIFERYIDSNGRERTREVEYKPSLFAHCPESQATKYFDIYGKPCTRKLFANM
RDASQWIKRMEDIGLEALGMDDFKLAYLSDTYNYEIKYDHTKIRVANFDIEVTSPDGFPEPSQAK
HPIDAITHYDSIDDRFYVFDLLNSPYGNVEEWSIEIAAKLQEQGGDEVPSEIIDKIIYMPFDNEK
ELLMEYLNFWQQKTPVILTGWNVESFAIPYVYNRIKNIFGESTAKRLSPHRKTRVKVIENMYGSR
EIITLFGISVLDYIDLYKKFSFTNQPSYSLDYISEFELNVGKLKYDGPISKLRESNHQRYISYNI
IAVYRVLQIDAKRQFINLSLDMGYYAKIQIQSVFSPIKTWDAIIFNSLKEQNKVIPQGRSHPVQP
YPGAFVKEPIPNRYKYVMSFDLTSLYPSIIRQVNISPETIAGTFKVAPLHDYINAVAERPSDVYS
CSPNGMMYYKDRDGVVPTEITKVFNQRKEHKGYMLAAQRNGEIIKEALHNPNLSVDEPLDVDYRF
DFSDEIKEKIKKLSAKSLNEMLFRAQRTEVAGMTAQINRKLLINSLYGALGNVWFRYYDLRNATA
ITTFGQMALQWIERKVNEYLNEVCGTEGEAFVLYGDTDSIYVSADKIIDKVGESKFRDTNHWVDF
LDKFARERMEPAIDRGFREMCEYMNNKQHLMFMDREAIAGPPLGSKGIGGFWTGKKRYALNVWDM
EGTRYAEPKLKIMGLETQKSSTPKAVQKALKECIRRMLQEGEESLQEYFKEFEKEFRQLNYISIA
SVSSANNIAKYDVGGFPGPKCPFHIRGILTYNRAIKGNIDAPQVVEGEKVYVLPLREGNPFGDKC
IAWPSGTEITDLIKDDVLHWMDYTVLLEKTFIKPLEGFTSAAKLDYEKKASLFDMFDF
>2AXLA
MDDSEDTSWDFGPQAFKLLSAVDILGEKFGIGLPILFLRGSNSQRLADQYRRHSLFGTGKDQTES
WWKAFSRQLITEGFLVEVSRYNKFMKICALTKKGRNWLHKANTESQSLILQANEELCPKKLLLPS
SKTVSSGTKEHCYN
>2AY0A
MGTTTMGVMLDDATRERIKSAATRIDRTPHWLIKQAIFSYLEQLENSDTLPEHHHHHH
>2AY0B
MGTTTMGVMLDDATRERIKSAATRIDRTPHWLIKQAIFSYLEQLENSDTLPEHHHHHH
>2AY0C
MGTTTMGVMLDDATRERIKSAATRIDRTPHWLIKQAIFSYLEQLENSDTLPEHHHHHH
>2B0LC
GSSHHHHHHMSKAVVQMAISSLSYSELEAIEHIFEELDGNEGLLVASKIADRVGITRSVIVNALR
KLESAGVIESRSLGMKGTYIKVLNNKFLIELENLKSH
>2BA3B
SDSAVRKKSEVRQKTVVRTLRFSPVEDETIRKKAEDSGLTVSAYIRNAALN
>2BDEA
MDTHKVFVNRIINMRKIKLIGLDMDHTLIRYNSKNFESLVYDLVKERLAESFHYPEEIKKFKFNF
DDAIRGLVIDSKNGNILKLSRYGAIRLSYHGTKQISFSDQKKIYRSIYVDLGDPNYMAIDTSFSI
AFCILYGQLVDLKDTNPDKMPSYQAIAQDVQYCVDKVHSDGTLKNIIIKNLKKYVIREKEVVEGL
KHFIRYGKKIFILTNSEYSYSKLLLDYALSPFLDKGEHWQGLFEFVITLANKPRFFYDNLRFLSV
NPENGTMTNVHGPIVPGVYQGGNAKKFTEDLGVGGDEILYIGDHIYGDILRLKKDCNWRTALVVE
ELGEEIASQIRALPIEKKIGEAMAIKKELEQKYVDLCTRSIDESSQQYDQEIHDLQLQISTVDLQ
ISRLLQEQNSFYNPKWERVFRAGAEESYFAYQVDRFACIYMEKLSDLLEHSPMTYFRANRRLLAH
DIDIAAALEHHHHHH
>2BNZA

MAKKDIMGDKTVRVRADLHHIIKIETAKNGGNVKEVMDQALEEYIRKYLPDKL
>2COBA
GSSGSSGRGRYRQYNSEILEEAISVVMSGKMSVSKAQSIYGIPHSTLEYKVKERLGTLKNPPKKK
MKLMR
>2CXYA
GSSGSSGEKITKVYELGNEPERKLWVDRYLTFMEERGSPVSSLPAVGKKPLDLFRLYVCVKEIGG
LAQVNKNKKWRELATNLNVGTSSSAASSLKKQYIQYLFAFECKIERGEEPPPEVFSTGDT
>2D7LA
GSSGSSGRPKTGFQMWLEENRSNILSDNPDFSDEADIIKEGMIRFRVLSTEERKVWANKAKGETA
SEGTEAKKRKSGPSSG
>2D8MA
GSSGSSGEPRRPRAGPEELGKILQGVVVVLSGFQNPFRSELRDKALELGAKYRPDWTRDSTHLIC
AFANTPKYSQVLGLGGRIVRKEWVLDCHRMRRRLPSQRYLMAGPGSSSEEDEASHSGGSGPSSG
>2D9HA
GSSGSSGLQCEICGFTCRQKASLNWHQRKHAETVAALRFPCEFCGKRFEKPDSVAAHRSKSHPAL
LLAPQESSGPSSG
>2DA6A
GSSGSSGRNRFKWGPASQQILYQAYDRQKNPSKEEREALVEECNRAECLQRGVSPSKAHGLGSNL
VTEVRVYNWFANRRKEEAFRQKLAMDAYSSNSGPSSG
>2DGZA
GSSGSSGSSQPVISAQEQETQIVLYGKLVEARQKHANKMDVPPAILATNKILVDMAKMRPTTVEN
VKRIDGVSEGKAAMLAPLWEVIKHFCQTNSVQTDLFSSTKPQSGPSSG
>2DIGA
GSSGSSGMPSRKFADGEVVRGRWPGSSLYYEVEILSHDSTSQLYTVKYKDGTELELKENDIKSGP
SSG
>2DINA
GSSGSSGKKTEWSREEEEKLLHLAKLMPTQWRTIAPIIGRTAAQCLEHYEFLLDKAAQRDSGPSS
G
>2DMPA
GSSGSSGAYPDFAPQKFKEKTQGQVKILEDSFLKSSFPTQAELDRLRVETKLSRREIDSWFSERR
KLRDSMEQAVLDSMGSGKSGPSSG
>2DMQA
GSSGSSGKRMRTSFKHHQLRTMKSYFAINHNPDAKDLKQLAQKTGLTKRVLQVWFQNARAKFRRN
LLRQENGGVSGPSSG
>2DPDB
MKEEKRSSTGFLVKQRAFLKLYMITMTEQERLYGLKLLEVLRSEFKEIGFKPNHTEVYRSLHELL
DDGILKQIKVKKEGAKLQEVVLYQFKDYEAAKLYKKQLKVELDRSKKLIEKALSDNF
>2DQBA
MRFSREALLELEASRLAPYAQKARDTRGRAHPEPESLYRTPYQKDRDRILHTTAFRRLEYKTQVL
PGWAGDYYRTRLTHTLEVAQVSRSIARALGLNEDLTEAIALSHDLGHPPFGHTGEHVLNALMQDH
GGFEHNAQALRILTHLEVRYPGFRGLNLTYEVLEGIATHEAAYSPGFKPLYEGQGTLEAQVVDLS
DAIAYAAHDLDDGFRAGLLHPEELKEVELLQALALEEGLDLLRLPELDRRVLVRQLLGYFITAAI
EATHRRVEEAGVQSAEAVRRHPSRLAALGEEAEKALKALKAFLMERFYRHPEVLRERRKAEAVLE
GLFAAYTRYPELLPREVQAKIPEEGLERAVCDYIAGMTDRFALEAYRRLSP

>2E5RA
GSSGSSGVFHPVECSYCHSESMMGFRYRCQQCHNYQLCQDCFWRGHAGGSHSNQHQMKEYTSW
>2E6RA
GSSGSSGHSSAQFIDSYICQVCSRGDEDDKLLFCDGCDDNYHIFCLLPPLPEIPRGIWRCPKCIL
AECKQPPEAFGFEQATQEYSLSGPSSG
>2EBIA
KKRAETWVQDETRSLIMFRRGMDGLFNTSKSNKHLWEQISSKMREKGFDRSPDMCTDKWRNLLKE
FKKAKHHDRGNGSAKMSYYKE
>2ELHA
GSSGSSGMNIRMGTKGKRPLRSLTPRDKIHAIQRIHDGESKASVARDIGVPESTLRGWCKNEDKL
RFMSRQSATDNLCADALGDKMD
>2EO0B
MYIVNSNKSRGSSVERYIVSRLRDKGFAVIRAPASGSKRKDHVPDIIALKSGVIILIEVKSRKNG
QKIYIEKEQAEGIREFAKRSGGELFLGVKLPKMLRFIKFDMLRQTEGGNYAIDLETVEKGMELED
LVRYVESKISRTLDSFL
>2EWTA
MSSEYAKQLGAKLRAIRTQQGLSLHGVEEKSQGRWKAVVVGSYERGDRAVTVQRLAELADFYGVP
VQELLP
>2F2EB
MVKRTSHKQASCPVARPLDVIGDGWSMLIVRDAFEGLTRFGEFQKSLGLAKNILAARLRNLVEHG
VMVAVPAESGSHQEYRLTDKGRALFPLLVAIRQWGEDYFFAPDESHVRLVERDSGQPVPRLQVRA
GDGSPLAAEDTRVSRD
>2F7NA
MTKKSTKSEAASKTKKSGVPETGAQGVRAGGADHADAAHLGTVNNALVNHHYLEEKEFQTVAETL
QRNLATTISLYLKFKKYHWDIRGRFFRDLHLAYDEFIAEIFPSIDEQAERLVALGGSPLAAPADL
ARYSTVQVPQETVRDARTQVADLVQDLSRVGKGYRDDSQACDEANDPVTADMYNGYAATIDKIRW
MLQAIMDDERLD
>2FC7A
GSSGSSGQQMQAESGFVQHVGFKCDNCGIEPIQGVRWHCQDCPPEMSLDFCDSCSDCLHETDIHK
EDHQLEPIYRSSGPSSG
>2FE3B
MAAHELKEALETLKETGVRITPQRHAILEYLVNSMAHPTADDIYKALEGKFPNMSVATVYNNLRV
FRESGLVKELTYGDASSRFDFVTSDHYHAICENCGKIVDFHYPGLDEVEQLAAHVTGFKVSHHRL
EIYGVCQECSKKENH
>2FMYA
ATQMRLTDTNLLEVLNSEEYSGVLKEFREQRYSKKAILYTPNTERNLVFLVKSGRVRVYLAYEDK
EFTLAILEAGDIFCTHTRAFIQAMEDTTILYTDIRNFQNIVVEFPAFSLNMVKVLGDLLKNSLTI
INGLVFKDARLRLAEFLVQAAMDTGLKVPQGIKLELGLNTEEIALMLGTTRQTVSVLLNDFKKMG
ILERVNQRTLLLKDLQKLKEFSSGV
>2FPHX
GIYQHFSIEDRPFLDKGMEWIKKVEDSYAPFLTPFINPHQEKLLKILAKTYGLACSSSGEFVSSE
YVRVLLYPDYFQPEFSDFEISLQEIVYSNKFEYLTHAKILGTVINQLGIERKLFGDILVDEERAQ
IMINQQFLLLFQDGLKKIGRIPVSLEERPFTEKID
>2FU4B

MTDNNTALKKAGLKVTLPRLKILEVLQEPDNHHVSAEDLYKRLIDMGEEIGLATVYRVLNQFDDA
GIVTRHNFEGGKSVFELT
>2FWRA
MGSSHHHHHHSSGLVPRGSHMQMIAEIYYERGTIVVKGDAHVPHAKFDSRSGTYRALAFRYRDII
EYFESNGIEFVDNAADPIPTPYFDAEISLRDYQEKALERWLVDKRGCIVLPTGSGKTHVAMAAIN
ELSTPTLIVVPTLALAEQWKERLGIFGEEYVGEFSGRIKELKPLTVSTYDSAYVNAEKLGNRFML
LIFDEVHHLPAESYVQIAQMSIAPFRLGLTATFEREDGRHEILKEVVGGKVFELFPDSLAGKHLA
KYTIKRIFVPLAEDERVEYEKREKVYKQFLRARGITLRRAEDFNKIVMASGYDERAYEALRAWEE
ARRIAFNSKNKIRKLREILERHRKDKIIIFTRHNELVYRISKVFLIPAITHRTSREEREEILEGF
RTGRFRAIVSSQVLDEGIDVPDANVGVIMSGSGSAREYIQRLGRILRPSKGKKEAVLYELISRGT
GEVNTARRRKNAAKGAA
>2G9WB
MAKLTRLGDLERAVMDHLWSRTEPQTVRQVHEALSARRDLAYTTVMAVLQRLAKKNLVLQIRDDR
AHRYAPVHGRDELVAGLMVDALAQAEDSGSRQAALVHFVERVGADEADALRRALAELEAGHGNRP
PAGAATET
>2GFUA
KAKNLNGGLRRSVAPAAPTSSDFSPGDLVWAKMEGYPWWPSLVYNHPFDGTFIREKGKSVRVHVQ
FFDDSPTRGWVSKRLLKPYTGSKSKEAQKGGHFYSAKPEILRAMQRADEALNKDKIKRLELAVSD
EPSE
>2GMGA
AHHHHHHGSATRREKIIELLLEGDYSPSELARILDMRGKGSKKVILEDLKVISKIAKREGMVLLI
KPAQCRKCGFVFKAEINIPSRCPKCKSEWIEEPRFKLERK
>2H6BB
MSVEGLGKDFCGAIIPDNFFPIEKLRNYTQMGLIRDFAKGSAVIMPGEEITSMIFLVEGKIKLDI
IFEDGSEKLLYYAGGNSLIGKLYPTGNNIYATAMEPTRTCWFSEKSLRTVFRTDEDMIFEIFKNY
LTKVAYYARQVAEMNTYNPTIRILRLFYELCSSQGKRVGDTYEITMPLSQKSIGEITGVHHVTVS
RVLACLKRENILDKKKNKIIVYNLGELKHLSEQTSYYSDPNSSSVDKLAAALDHH
>2H9UA
MACEGAPEVRIGRKPVMNYVLAILTTLMEQGTNQVVVKARGRNINRAVDAVEIVRKRFAKNIEIK
DIKIDSQEIEVQTPEGQTRTRRVSSIEICLEKAGESA
>2HGVA
GSSHHHHHHMALLQKTRIINSMLQAAAGKPVNFKEMAETLRDVIDSNIFVVSRRGKLLGYSINQQ
IENDRMKKMLEDRQFPEEYTKNLFNVPETSSNLDINSEYTAFPVENRDLFQAGLTTIVPIIGGGE
RLGTLILSRLQDQFNDDDLILAEYGATVVGMEIL
>2HKVA
GMTDWQQALDRHVGVGVRTTRDLIRLIQPEDWDKRPISGKRSVYEVAVHLAVLLEADLRIATGAT
ADEMAQFYAVPVLPEQLVDRLDQSWQYYQDRLMADFSTETTYWGVTDSTTGWLLEAAVHLYHHRS
QLLDYLNLLGYDIKLDLFE
>2HQLF
GGGGGGMLNRVFLEGEIESSCWSVKKTGFLVTIKQMRFFGERLFTDYYVIYANGQLAYELEKHTK
KYKTISIEGILRTYLERKSEIWKTTIEIVKIFNPKNEIVIDYKEI
>2HUEA
PLGSPNSSIVSLLGIKVLNNPAKFTDPYEFEITFECLESLKHDLEWKLTYVGSSRSLDHDQELDS
ILVGPVPVGVNKFVFSADPPSAELIPASELVSVTVILLSCSYDGREFVRVGYYVNNEYDEEELRE

NPPAKVQVDHIVRNILAEKPRVTRFNIVWDNENEGDLYPPEQPGV
>2I13B
ISEFGSSSSVAQAALEPGEKPYACPECGKSFSRSDHLAEHQRTHTGEKPYKCPECGKSFSDKKDL
TRHQRTHTGEKPYKCPECGKSFSQRANLRAHQRTHTGEKPYACPECGKSFSQLAHLRAHQRTHTG
EKPYKCPECGKSFSREDNLHTHQRTHTGEKPYKCPECGKSFSRRDALNVHQRTHTGKKTS
>2IJGX
MNDHIHRVPALTEEEIDSVAIKTFERYALPSSSSVKRKGKGVTILWFRNDLRVLDNDALYKAWSS
SDTILPVYCLDPRLFHTTHFFNFPKTGALRGGFLMECLVDLRKNLMKRGLNLLIRSGKPEEILPS
LAKDFGARTVFAHKETCSEEVDVERLVNQGLKRVGNSTKLELIWGSTMYHKDDLPFDVFDLPDVY
TQFRKSVEAKCSIRSSTRIPLSLGPTPSVDDWGDVPTLEKLGVEPQEVTRGMRFVGGESAGVGRV
FEYFWKKDLLKVYKETRNGMLGPDYSTKFSPWLAFGCISPRFIYEEVQRYEKERVANNSTYWVLF
ELIWRDYFRFLSIKCGNSLFHLGGPRNVQGKWSQDQKLFESWRDAKTGYPLIDANMKELSTTGFM
SNRGRQIVCSFLVRDMGLDWRMGAEWFETCLLDYDPCSNYGNWTYGAGVGNDPREDRYFSIPKQA
QNYDPEGEYVAFWLQQLRRLPKEKRHWPGRLMYMDTVVPLKHGNGPMAGGSKSGGGFRGSHSGRR
SRHNGP
>2INGX
GPRMSMVVSGLTPEEFMLVYKFARKHHITLTNLITEETTHVVMKTDAEFVCERTLKYFLGIAGGK
WVVSYFWVTQSIKERKMLNEHDFEVRGDVVNGRNHQGPKRARESQDRKIFRGLEICCYGPFTNKP
TDQLEWMVQLCGASVVKELSSFTLGTGVHPIVVVQPDAWTEDNGFHAIGQMCEAPVVTREWVLDS
VALYQCQELDTYLIPQIP
>2IO4B
MMKAKVIDAVSFSYILRTVGDFLSEANFIVTKEGIRVSGIDPSRVVFLDIFLPSSYFEGFEVSQE
KEIIGFKLEDVNDILKRVLKDDTLILSSNESKLTLTFDGEFTRSFELPLIQVESTQPPSVNLEFP
FKAQLLTITFADIIDELSDLGEVLNIHSKENKLYFEVIGDLSTAKVELSTDNGTLLEASGADVSS
SYGMEYVANTTKMRRASDSMELYFGSQIPLKLRFKLPQEGYGDFYIAPRAD
>2IO4A
MVKIVYPNAKDFFSFINSITNVTDSIILNFTEDGIFSRHLTEDKVLMAIMRIPKDVLSEYSIDSP
TSVKLDVSSVKKILSKASSKKATIELTETDSGLKIIIRDEKSGAKSTIYIKAEKGQVEQLTEPKV
NLAVNFTTDESVLNVIAADVTLVGEEMRISTEEDKIKIEAGEEGKRYVAFLMKDKPLKELSIDTS
ASSSYSAEMFKDAVKGLRGFSAPTMVSFGENLPMKIDVEAVSGGHMIFWIAPRL
>2JD3B
MDDERKRKKYTLYLHPEKAADFQTLEAIESVPRSERGELFRNAFISGMALHQLDPRLPVLLTAIL
SEEFSADQVVTLLSQTTGWKPSQADIRAVLTELGASQSVEKMPPSATDSVQEAMNDVRLKMKKLF
>2JMPA
MGGGGGGMEQFNAFKSLLKKHYEKTIGFHDKYIKDINRFVFKNNVLLILLENEFARNSLNDNSEI
IHLAESLYEGIKSVNFVNEQDFFFNLAKLEENSRDTLYQNSG
>2JPCA
LRERQVLKLIDEGYTNHGISEKLHISIKTVETHRMNMMRKLQVHKVTELLNCARRMRLIEY
>2JR1A
FNVKQKSEITALVKEVTPPRKAPSKAKREAPIKYWLPHSGATWSGRGKIPKPFEAWIGTAAYTAW
KAKHPDEKFPAFPG
>2JTMA
MSSGKKPVKVKTPAGKEAELVPEKVWALAPKGRKGVKIGLFKDPETGKYFRHKLPDDYPI
>2JULA

MQRTKEAVKASDGNLLGDPGRIPLSKRESIKWQRPRFTRQALMRCCLIKWILSSAAPQGSDSSDS
ELELSTVRHQPEGLDQLQAQTKFTKKELQSLYRGFKNECPTGLVDEDTFKLIYSQFFPQGDATTY
AHFLFNAFDADGNGAIHFEDFVVGLSILLRGTVHEKLKWAFNLYDINKDGCITKEEMLAIMKSIY
DMMGRHTYPILREDAPLEHVERFFQKMDRNQDGVVTIDEFLETCQKDENIMNSMQLFENVI
>2JX3A
FTIAQGKGQKLCEIERIHFFLSKKKTDELRNLHKLLYNRPGTVSSLKKNVGQFSGFPFEKGSVQY
KKKEEMLKKFRNAMLKSICEVLDLERSGVNSELVKRILNFLMHPKPSGKPLPKSKKTCSKGSKKE
R
>2K4BA
MSYYHHHHHHDYDIPTTENLYFQGAMNEVEFNVSNAELIVMRVIWSLGEARVDEIYAQIPQELEW
SLATVKTLLGRLVKKEMLSTEKEGRKFVYRPLME
>2K5VA
MNYKISELMPNLSGTINAEVVAAYPKKEFSRKDGTKGQLKSLFLKDDTGSIRGTLWNELADFEVK
KGDIAEVSGYVKQGYSGLEISVDNIGIIEKSLEHHHHHH
>2K6GA
KRTNYQAYRSYLNREGPKALGSKEIPKGAENCLEGLIFVITGVLESIERDEAKSLIERYGGKVTG
NVSKKTNYLVMGRDSGQSKSDKAAALGTKIIDEDGLLNLIRNLE
>2K75A
SDLVKIRDVSLSTPYVSVIGKITGIHKKEYESDGTTKSVYQGYIEDDTARIRISSFGKQLQDSDV
VRIDNARVAQFNGYLSLSVGDSSRIESVNVNIPLEHHHHHH
>2K86A
GSSSRRNAWGNLSYADLITRAIESSPDKRLTLSQIYEWMVRCVPYFKDKGDSNSSAGWKNSIRHN
LSLHSRFMRVQNEGTGKSSWWIINPDGGKSGKAPRRRA
>2K9IB
GRPYKLLNGIKLGVYIPQEWHDRLMEIAKEKNLTLSDVCRLAIKEYLDNHDKQKK
>2K9NA
KVKFTEEEDLKLQQLVMRYGAKDWIRISQLMITRNPRQCRERWNNYINPALRTDPWSPEEDMLLD
QKYAEYGPKWNKISKFLKNRSDNNIRNRWMMIARHRAKHQKS
>2KEBA
MGSSHHHHHHGSSLEVLFQGPGSMSASAQQLAEELQIFGLDCEEALIEKLVELCVQYGQNEEGMV
GELIAFCTSTHKVGLTSEILNSFEHEFLSKRLSKAR
>2KHQA
MITFADYFYQWYEVNKLPHVSESTKRHYESAYKHIKDHFRHKLLKDIKRTEYQKFLNEYGLTHSY
ETIRKLNSYIRNAFDDAIHEGYVIKNPTYKAELHASVLEHHHHHH
>2KI2A
MRNIYVGNLVYSATSEQVKELFSQFGKVFNVKLIYDRETKKPKGFGFVEMQEESVSEAIAKLDNT
DFMGRTIRVTEANPKKSLEHHHHHH
>2KIWA
TFKQVADDWLKQYANDVKVSSVRAREKAIQHAIERFNTKPIQTIKKHDYQRFVDDISAQYSKNYV
DSIVASTNMIFKYAYDTRLIKAMPSEGIKRPKKKVSVELEHHHHHH
>2KJ8A
SSNNNSFSAIYKEWYEHKKQVWSVGYATELAKMFDDDILPIIGGLEIQDIEPMQLLEVIRRFEDR
GAMERANKARRRCGEVFRYAIVTGRAKYNPAPDLADAMKGYRKKNLEHHHHHH
>2KKOB

MAGQSDRKAALLDQVARVGKALANGRRLQILDLLAQGERAVEAIATATGMNLTTASANLQALKSG
GLVEARREGTRQYYRIAGEDVARLFALVQVVADEHLEHHHHHH
>2KKPA
MIEPSKITVEQWLNRWLTDYAKPHLRQSTWESYETVLRLHVIPTLGSIPLKKLQPADIQRLYASK
LESGLSPTRVRYIHVVLHEAMSQARESGLLLQNPTEAAKPPRHPLEHHHHHH
>2KKVA
MENSGAYTFETIAREWHESNKRWSEDHRSRVLRYLELYIFPHIGSSDIRQLKTSHLLAPIKEVDT
SGKHDVAQRLQQRVTAIMRYAVQNDYIDSNPASDMAGALSTTKARHYPLEHHHHHH
>2KNGA
SGSGRGRGAIDREQSAAIREWARRNGHNVSTRGRIPADVIDAYHAATLEHHHHHH
>2KW3C
GHGTGSFGDRPARPTLLEQVLNQKRLSLLRSPEVVQFLQKQQQLLNQQVLEQRQQQFPGTSM
>2KW3B
GAGEPTTLLQRLRGTISKAVQNKVEGILQDVQKFSDNDKLYLYLQLPSGPTTGDKSSEPSTLSNE
EYM
>2KWQA
GPMGMQSIREQSCRVVTCKTCKYTHFKPKETCVSENHDFHWHNGVKRFFKCPCGNRTISLDRLPK
KHCSTCGLFKWERVGMLKEKTGPKLGG
>2L1PA
MGHHHHHHSHMLPPEQWSHTTVRNALKDLLKDMNQSSLAKECPLSQSMISSIVNSTYYANVSAAK
CQEFGRWYKHFKKTKDMM
>2L3NA
SVSILRSSVNHREVDEAIDNILRYTNSTEQQFLEAMESTGGRVRIAIAKLLSKQTSGGSGGSKLG
GSGGSRKDLSVKGMLYDSDSQQILNRLRERVSGSTAQSA
>2L49B
MSNTISEKIVLMRKSEYLSRQQLADLTGVPYGTLSYYESGRSTPPTDVMMNILQTPQFTKYTLWF
MTNQIAPESGQIAPALAHFGQNETTSPHSGQKTG
>2L92A
MSTVPKYRDPATGKTWSGRGRQPAWLGNDPAAFLIQPDLPAILEHHHHHH
>2L93A
AARPAKYSYVDENGETKTWTGQGRTPAVIKKAMEEQGKQLEDFLIKELEHHHHHH
>2LFHB
MGHHHHHHSHMGGGKGPAAEEPLSLLDDMNHCYSRLRELVPGVPRGTQLSQVEILQRVIDYILDL
QVV
>2LJ6A
MPSSKPLAEYARKRDFRQTPEPSGRKPRKDSTGLLRYCVQKHDASRLHYDFRLELDGTLKSWAVP
KGPCLDPAVKRLAVQVEDHPLDYADFEGSIPQGHYGAGDVIVWDRGAWTPLDDPREGLEKGHLSF
ALDGEKLSGRWHLIRTNLRGKQSQWFLVKAKDGEARSLDRFDVLKER
>2LLHA
GSHMQESFKKQEKTPKTPKGPSSVEDIKAKMQASIEKGGSLPKVEAKFINYVKNCFRMTDQEAIQ
DLWQWRKSL
>2LSSA
MATNIVGKVKWYNSTKNFGFIEQDNGGKDVFVHKSAVDAAGLHSLEEGQDVIFDLEEKQGKAYAV
NLRIK

>2LTTB
MADKLKFEIIEELIVLSENAKGWRKELNRVSWNDAEPKYDIRTWSPDHEKMGKGITLSEEEFGVL
LKELGNKLEHHHHHH
>2LUAA
SPPKPKCRCGISGSSNTLTTCRNSRCPCYKSYNSCAGCHCVGCKNPHKEDYV
>2LUYA
HMGKNDNDALIMCMRCRKVKGIDSYSKTQWSKTFTFVRGRTVSVSDPKVICRTCQPKQHDSIWCT
ACQQTKGINEFSKAQRHVLDPRCQICVHSQRN
>2LW1A
GSHMKAETVKRSSSKLSYKLQRELEQLPQLLEDLEAKLEALQTQVADASFFSQPHEQTQKVLADM
AAAEQELEQAFERWEYLEALKNGG
>2LYJB
MIINNLKLIREKKKISQSELAALLEVSRQTINGIEKNKYNPSLQLALKIAYYLNTPLEDIFQWQP
E
>2M14A
PSHSGAAIFEKVSGIIAINEDVSPAELTWRSTDGDKVHTVVLSTIDKLQATPASSEKMMLRLIGK
VDESKKRKDNEGNEVVPKPQRHMFSFNNRTVMDNIKMTLQQIISRYKDADGNSS
>2M8EA
ASMGKSKEISQDLRKKIVDLHKSGSSLGAISKRLKVPRSSVQTIVRKYKHHGTTQHH
>2MA1A
HDAPLFEALRAWRLQKAKELSLPPYTIFHDATLKTIAELRPGSHATLGTVSGVGGRKLAAYGDEV
LQVVRDSSGG
>2MAMA
ADEPAYLTVGTDVSAKYRGAFCEAKIKTVKRLVKVKVLLKQDNTTQLVQDDQVKGPLRVGAIVET
RTSDGSFQEAIISKLTDASWYTVVFDDGDERTLRRTSLCLKGERHFAESETLD
>2MH3B
MKPKTASEHRKSSKPIMEKRRRARINESLSQLKTLILDALKKDSSRHSKLEKADILEMTVKHLRN
LQRAQ
>2NMUA
MAGDPNSMTVSHHNASTARFYALRLLPGQEVFSQLHAFVQQNQLRAAWIAGCTGSLTDVALRYAG
QEATTSLTGTFEVISLNGTLELTGEHLHLAVSDPYGVMLGGHMMPGCTVRTTLELVIGELPALTF
SRQPCAISGYDELHISSRLEHHHHHH
>2NOGB
MVSEPKVPKAPRPPKQPNVQDFQFFPPRLFELLEKEILYYRKTIGYKVPRNPDLPNSAQVQKEEQ
LKIDEAEPLNDEELEEKEKLLTQGFTNWNKRDFNQFIKANEKWGRDDIENIAREVEGKTPEEVIE
YSAVFWERCNELQDIEKTMAQIERGEARIQRRISILEHHHHHH
>2NP2B
MSFSRRPKVTKSDIVDQIALNIKNNNLKLEKKYIRLVIDAFFEELKSNLCSNNVIEFRSFGTFEV
RKRKGRLNARNPQTGEYVKVLDHHVAYFRPGKDLKERVWGIKG
>2O3CC
GSHMEAPILYEDPPEKLTSKDGRAANMKITSWNVDGLRAWVKKNGLDWVRKEDPDILCLQETKCA
EKALPADITAMPEYPHKYWAGSEDKEGYSGVAMLCKTEPLNVTYGIGKEEHDKEGRVITAEFPDF
FLVTAYVPNASRGLVRLDYRKTWDVDFRAYLCGLDARKPLVLCGDLNVAHQEIDLKNPKGNRKNA
GFTPEEREGFTQLLEAGFTDSFRELYPDQAYAYTFWTYMMNARSKNVGWRLDYFVLSSALLPGLC

DSKIRNTAMGSDHCPITLFLAV
>2O8BB
MGSAPQNSESQAHVSGGGDDSSRPTVWYHETLEWLKEEKRRDEHRRRPDHPDFDASTLYVPEDFL
NSCTPGMRKWWQIKSQNFDLVICYKVGKFYELYHMDALIGVSELGLVFMKGNWAHSGFPEIAFGR
YSDSLVQKGYKVARVEQTETPEMMEARCRKMAHISKYDRVVRREICRIITKGTQTYSVLEGDPSE
NYSKYLLSLKEKEEDSSGHTRAYGVCFVDTSLGKFFIGQFSDDRHCSRFRTLVAHYPPVQVLFEK
GNLSKETKTILKSSLSCSLQEGLIPGSQFWDASKTLRTLLEEEYFREKLSDGIGVMLPQVLKGMT
SESDSIGLTPGEKSELALSALGGCVFYLKKCLIDQELLSMANFEEYIPLDSDTVSTTRSGAIFTK
AYQRMVLDAVTLNNLEIFLNGTNGSTEGTLLERVDTCHTPFGKRLLKQWLCAPLCNHYAINDRLD
AIEDLMVVPDKISEVVELLKKLPDLERLLSKIHNVGSPLKSQNHPDSRAIMYEETTYSKKKIIDF
LSALEGFKVMCKIIGIMEEVADGFKSKILKQVISLQTKNPEGRFPDLTVELNRWDTAFDHEKARK
TGLITPKAGFDSDYDQALADIRENEQSLLEYLEKQRNRIGCRTIVYWGIGRNRYQLEIPENFTTR
NLPEEYELKSTKKGCKRYWTKTIEKKLANLINAEERRDVSLKDCMRRLFYNFDKNYKDWQSAVEC
IAVLDVLLCLANYSRGGDGPMCRPVILLPEDTPPFLELKGSRHPCITKTFFGDDFIPNDILIGCE
EEEQENGKAYCVLVTGPNMGGKSTLMRQAGLLAVMAQMGCYVPAEVCRLTPIDRVFTRLGASDRI
MSGESTFFVELSETASILMHATAHSLVLVDELGRGTATFDGTAIANAVVKELAETIKCRTLFSTH
YHSLVEDYSQNVAVRLGHMACMVENECEDPSQETITFLYKFIKGACPKSYGFNAARLANLPEEVI
QKGHRKAREFEKMNQSLRLFREVCLASERSTVDAEAVHKLLTLIKEL
>2O8BA
MAVQPKETLQLESAAEVGFVRFFQGMPEKPTTTVRLFDRGDFYTAHGEDALLAAREVFKTQGVIK
YMGPAGAKNLQSVVLSKMNFESFVKDLLLVRQYRVEVYKNRAGNKASKENDWYLAYKASPGNLSQ
FEDILFGNNDMSASIGVVGVKMSAVDGQRQVGVGYVDSIQRKLGLCEFPDNDQFSNLEALLIQIG
PKECVLPGGETAGDMGKLRQIIQRGGILITERKKADFSTKDIYQDLNRLLKGKKGEQMNSAVLPE
MENQVAVSSLSAVIKFLELLSDDSNFGQFELTTFDFSQYMKLDIAAVRALNLFQGSVEDTTGSQS
LAALLNKCKTPQGQRLVNQWIKQPLMDKNRIEERLNLVEAFVEDAELRQTLQEDLLRRFPDLNRL
AKKFQRQAANLQDCYRLYQGINQLPNVIQALEKHEGKHQKLLLAVFVTPLTDLRSDFSKFQEMIE
TTLDMDQVENHEFLVKPSFDPNLSELREIMNDLEKKMQSTLISAARDLGLDPGKQIKLDSSAQFG
YYFRVTCKEEKVLRNNKNFSTVDIQKNGVKFTNSKLTSLNEEYTKNKTEYEEAQDAIVKEIVNIS
SGYVEPMQTLNDVLAQLDAVVSFAHVSNGAPVPYVRPAILEKGQGRIILKASRHACVEVQDEIAF
IPNDVYFEKDKQMFHIITGPNMGGKSTYIRQTGVIVLMAQIGCFVPCESAEVSIVDCILARVGAG
DSQLKGVSTFMAEMLETASILRSATKDSLIIIDELGRGTSTYDGFGLAWAISEYIATKIGAFCMF
ATHFHELTALANQIPTVNNLHVTALTTEETLTMLYQVKKGVCDQSFGIHVAELANFPKHVIECAK
QKALELEEFQYIGESQGYDIMEPAAKKCYLEREQGEKIIQEFLSKVKQMPFTEMSEENITIKLKQ
LKAEVIAKNNSFVNEIISRIKVTT
>2O99A
GHMSRNLLAIVHPILRNLMEESGETVNMAVLDQSDHEAIIIDQVQCTHLMRMSAPIGGKLPMHAS
GAGKAFLAQLSEEQVTKLLHRKGLHAYTHATLVSPVHLKEDLAQTRKRGYSFDDEEHALGLRCLA
ACIFDEHREPFAAISISGPISRITDDRVTEFGAMVIKAAKEVTLAYGGMRGS
>2OBPB
GMSDPGNEQNGDGIDPAIVEVLLVLREAGIENGATPWSLPKIAKRAQLPMSVLRRVLTQLQAAGL
ADVSVEADGRGHASLTQEGAALAAQLFPDPF
>2OCJD
SSSVPSQKTYQGSYGFRLGFLHSGTAKSVTCTYSPALNKMFCQLAKTCPVQLWVDSTPPPGTRVR
AMAIYKQSQHMTEVVRRCPHHERCSDSDGLAPPQHLIRVEGNLRVEYLDDRNTFRHSVVVPYEPP

EVGSDCTTIHYNYMCNSSCMGGMNRRPILTIITLEDSSGNLLGRNSFEVRVCACPGRDRRTEEEN
LRKKGEPHHELPPGSTKRALPNNT
>2OD5A
GMTGAVETESMKTVRIREKIKKFLGDRPRNTAEILEHINSTMRHGTTSQQLGNVLSKDKDIVKVG
YIKRSGILSGGYDICEWATRNWVAEHCPEWTEGQPIILNEEGDFTLGPLPE
>2OKFA
GMSARDVFHEVVKTALKKDGWQITDDPLTISVGGVNLSIDLAAQKLIAAERQGQKIAVEVKSFLK
QSSAISEFHTALGQFINYRGALRKVEPDRVLYLAVPLTTYKTFFQLDFPKEIIIENQVKMLVYDV
EQEVIFQWIN
>2OWYB
MWFRNLLVYRLTQDLQLDADSLEKALGEKSARPCASQELTTYGFTAPFGKGPDAPLVHVSQDFFL
ISARKEERILPGSVVRDALKEKVDEIEAQQMRKVYKKERDQLKDEIVQTLLPRAFIRRSSTFAAI
APSLGLILVDSASAKKAEDLLSTLREALGSLPVRPLSVKVAPTATLTDWVKTQEAAGDFHVLDEC
ELRDTHEDGGVVRCKRQDLTSEEIQLHLTAGKLVTQLSLAWSDKLSFVLDDKLAVKRLRFEDLLQ
EQAEKDGGEDALGQLDASFTLMMLTFAEFLPALFEALGGEEIPQGV
>2OZ9R
AQQSPYSAAMAEQRHQEWLRFVDLLKNAYQNDLHLPLLNLMLTPDEREALGTRVRIVEELLRGEM
SQRELKNELGAGIATITRGSNSLKAAPVELRQWLEEVLLKSD
>2OZEA
MIQYYYTKKEWGVVMEKEELKILEELRRILSNKNEAIVILNNYFKGGVGKSKLSTMFAYLTDKLN
LKVLMIDKDLQATLTKDLAKTFKVELPRVNFYEGLKNGNLASSIVHLTDNLDLIPGTFDLMLLPK
LTRSWTFENESRLLATLLAPLKSDYDLIIIDTVPTPSVYTNNAIVASDYVMIPLQAEEESTNNIQ
NYISYLIDLQEQFNPGLDMIGFVPYLVDTDSATIKSNLEELYKQHKEDNLVFQNIIKRSNKVSTW
SKNGITEHKGYDKKVLSMYKNVFFEMLERIIQLENEKE
>2P2UB
SLSRRKPNPVIVADIRQAEGALAEIATIDRKVGEIEAQMNEAIDAAKARASQKSAPLLARRKELE
DGVATFATLNKTEMFKDRKSLDLGFGTIGFRLSTQIVQMSKITKDMTLERLRQFGISEGIRIKED
VNKEAMQGWPDERLEMVGLKRRTTDAFYIEINREEVADTAA
>2P5KA
MNKGQRHIKIREIITSNEIETQDELVDMLKQDGYKVTQATVSRDIKELHLVKVPTNNGSYKYSL
>2P5MC
MQRFNPLSKLKRALMDAFVKIDSASHMIVLKTMPGNAQAIGALMDNLDWDEMMGTICGDDTILII
CRTPEDTEGVKNRLLELL
>2P6RA
MKVEELAESISSYAVGILKEEGIEELFPPQAEAVEKVFSGKNLLLAMPTAAGKTLLAEMAMVREA
IKGGKSLYVVPLRALAGEKYESFKKWEKIGLRIGISTGDYESRDEHLGDCDIIVTTSEKADSLIR
NRASWIKAVSCLVVDEIHLLDSEKRGATLEILVTKMRRMNKALRVIGLSATAPNVTEIAEWLDAD
YYVSDWRPVPLVEGVLCEGTLELFDGAFSTSRRVKFEELVEECVAENGGVLVFESTRRGAEKTAV
KLSAITAKYVENEGLEKAILEENEGEMSRKLAECVRKGAAFHHAGLLNGQRRVVEDAFRRGNIKV
VVATPTLAAGVNLPARRVIVRSLYRFDGYSKRIKVSEYKQMAGRAGRPGMDERGEAIIIVGKRDR
EIAVKRYIFGEPERITSKLGVETHLRFHSLSIICDGYAKTLEELEDFFADTFFFKQNEISLSYEL
ERVVRQLENWGMVVEAAHLAPTKLGSLVSRLYIDPLTGFIFHDVLSRMELSDIGALHLICRTPDM
ERLTVRKTDSWVEEEAFRLRKELSYYPSDFSVEYDWFLSEVKTALCLKDWIEEKDEDEICAKYGI
APGDLRRIVETAEWLSNAMNRIAEEVGNTSVSGLTERIKHGVKEELLELVRIRHIGRVRARKLYN

AGIRNAEDIVRHREKVASLIGRGIAERVVEGISVKSLNPESAAALEHHHHHH
>2PG4A
GMDDETLRLQFGHLIRILPTLLEFEKKGYEPSLAEIVKASGVSEKTFFMGLKDRLIRAGLVKEET
LSYRVKTLKLTEKGRRLAECLEKCRDVLGS
>2PI2E
MGHHHHHHHHHHSSGHIEGRHMVDMMDLPRSRINAGMLAQFIDKPVCFVGRLEKIHPTGKMFILS
DGEGKNGTIELMEPLDEEISGIVEVVGRVTAKATILCTSYVQFKEDSHPFDLGLYNEAVKIIHDF
PQFYPLGIVQHD
>2PI2A
MWNSGFESYGSSSYGGAGGYTQSPGGFGSPAPSQAEKKSRARAQHIVPCTISQLLSATLVDEVFR
IGNVEISQVTIVGIIRHAEKAPTNIVYKIDDMTAAPMDVRQWVDTDDTSSENTVVPPETYVKVAG
HLRSFQNKKSLVAFKIMPLEDMNEFTTHILEVINAHMVLSKANSQPSAGRAPISNPGMSEAGNFG
GNSFMPANGLTVAQNQVLNLIKACPRPEGLNFQDLKNQLKHMSVSSIKQAVDFLSNEGHIYSTVD
DDHFKSTDAE
>2PKHH
SNAARGHRHTCKVMVLKEEAAGSERALALDMREGQRVFHSLIVHFENDIPVQIEDRFVNAQVAPD
YLKQDFTLQTPYAYLSQVAPLTEGEHVVEAILAEADECKLLQIDAGEPCLLIRRRTWSGRQPVTA
ARLIHPGSRHRLEGRFTK
>2PNHB
GSHMTNRLVLSGTVCRAPLRKVSPSGIPHCQFVLEHRSVQEAAGFHRQAWCQMPVIVSGHENQAI
THSITVGSRITVQGFISCHKAKNGLSKMVLHAEQIELIDSGD
>2Q2KB
MGSSHHHHHHSSGLVPGSHMDKKETKHLLKIKKEDYPQIFDFLENVPRGTKTAHIREALRRYIEE
IGENP
>2Q79A
TTPIVHLKGDANTLKCLRYRFKKHCTLYTAVSSTWHWTGHNVKHKSAIVTLTYDSEWQRDQFLSQ
VKIPKTITVSTGFMSIGGGTGGGSGGGS
>2QLCA
MNLKVKGARDVFEYMKGRIPDETKEHLFVLFLSTKNQILRHETITIGTLTASLIHPREIFKAAIR
ESAHSIILVHNHPSGDVQPSNADKQVTSILKKAGDLLQIELLDHVIVGNNDWFSFRDHALL
>2QPYA
CQPIFLNVLEAIEPGVVCAGHDNNQPDSFAALLSSLNELGERQLVHVVKWAKALPGFRNLHVDDQ
MAVIQYSWMGLMVFAMGWRSFTNVNSRMLYFAPDLVFNEYRMHKSRMYSQCVRMRHLSQEFGWLQ
ITPQEFLCMKALLLFSIIPVDGLKNQKFFDELRMNYIKELDRIIACKRKNPTSCSRRFYQLTKLL
DSVQPIARELHQFTFDLLIKSHMVSVDFPEMMAEIISVQVPKILSGKVKPIYFHTQ
>2QSFX
GSGNASSGALGTTGGATDAAQGGPPGSIGLTVEDLLSLRQVVSGNPEALAPLLENISARYPQLRE
HIMANPEVFVSMLLEAVGDNMQDVMEGADDMVEGEDIEVTGEAAAAGLGQGEGEGSFQVDYTPED
DQAISRLCELGFERDLVIQVYFACDKNEEAAANILFSDHAD
>2QSHA
GSSRAMGNEVAGVEDISVEIKPSSKRNSDARRTSRNVCSNEERKRRKYFHMLYLVCLMVHGFIRN
EWINSKRLSRKLSNLVPEKVFELLHPQKDEELPLRSTRKLLDGLKKCMELWQKHWKITKKYDNEG
LYMRTWKEIEMSANNKRKFKTLKRSDFLRAVSKGHGDPDISVQGFVAMLRACNVNARLIMSCQPP
DFTNMKIDTSLNGNNAYKDMVKYPIFWCEVWDKFSKKWITVDPVNLKTIEQVRLHSKLAPKGVAC

CERNMLRYVIAYDRKYGCRDVTRRYAQWMNSKVRKRRITKDDFGEKWFRKVITALHHRKRTKIDD
YEDQYFFQRDESEGIPDSVQDLKNHPYYVLEQDIKQTQIVKPGCKECGYLKVHGKVGKVLKVYAK
RDIADLKSARQWYMNGRILKTGSRCKKVIKRTVGRPKGEAEEEDERLYSFEDTELYIPPLASASG
EITKNTFGNIEVFAPTMIPGNCCLVENPVAIKAARFLGVEFAPAVTSFKFERGSTVKPVLSGIVV
AKWLREAIETAIDGIEFI
>2QUQA
KLITASSSKEYLPDLLLFWQNYEYWITNIGLYKTKQRDLTRTPANLDTDTEECMFWMNYLQKDQS
FQLMNFAMENLGALYFGSIGDISELYLRVEQYWDRRADKNHSVDGKYWDALIWSVFTMCIYYMPV
EKLAEIFSVYPLHEYLGSNKRLNWEDGMQLVMCQNFARCSLFQLKQCDFMAHPDIRLVQAYLILA
TTTFPYDEPLLANSLLTQCIHTFKNFHVDDFRPLLNDDPVESIAKVTLGRIFYRLCGCDYLQSGP
RKPIALHTEVSSLLQHAAYLQDLPNVDVYREENSTEVLYWKIISLDRDLDQYLNKSSKPPLKTLD
AIRRELDIFQYKVDSLEEDFRSNNSRFQKFIALFQISTVSWKLFKMYLIYYDTADSLLKVIHYSK
VIISLIVNNFHAKSEFFNRHPMVMQTITRVVSFISFYQIFVESAAVKQLLVDLTELTANLPTIFG
SKLDKLVYLTERLSKLKLLWDKVQLLDSGDSFYHPVFKILQNDIKIIELKNDEMFSLIKGLGSLV
PLNKLRQESLLEEEDENNTEPSDFRTIVEEFQSEYNISDILS
>2RH3A
IQVFLSARPPAPEVSKIYDNLILQYSPSKSLQMILRRALGDFENMLADGSFRAAPKSYPIPHTAF
EKSIIVQTSRMFPVSLIEAARNHFDPLGLETARAFGHKLATAALACFFAREKATNS
>2ROHA
GSPFADPNSLALANVPLSRSKRPDFGQRRIRRPFTVAEVELLVEAVEHLGTGRWRDVKFRAFENV
HHRTYVDLKDKWKTLVHTASIAPQQRRGAPVPQELLDRVLAAQAYWSVDSSGRIVTL
>2RRDA
GIPEFKQKALVAKVSQREEMVKKCLGELTEVCKSLGKVFGVHYFNIFNTVTLKKLAESLSSDPEV
LLQIDGVTEDKLEKYGAEVISVLQKYSEWTSPAEDS
>2RT6A
MKTALLLEKLEGQLATLRQRCAPVSQFATLSARFDRHLFQTRATTLQACLDEAGDNLAALRHAVE
QQQLPQVAWLAEHLAAQLEAIAREASAWSLREW
>2VL6C
MEIPSKQIDYRDVFIEFLTTFKGNNNQNKYIERINELVAYRKKSLIIEFSDVLSFNENLAYEIIN
NTKIILPILEGALYDHILQLDPTYQRDIEKVHVRIVGIPRVIELRKIRSTDIGKLITIDGILVKV
TPVKERIYKATYKHIHPDCMQEFEWPEDEEMPEVLEMPTICPKCGKPGQFRLIPEKTKLIDWQKA
VIQERPEEVPSGQLPRQLEIILEDDLVDSARPGDRVKVTGILDIKQDSPVKRGSRAVFDIYMKVS
SIEVSQKV
>2WKCD
GTIITVTAQANEKNTRTVSTAKGDKKIISVPLFEKEKGSNVKVAYGSAFLPDFIQLGDTVTVSGR
VQAKESGEYVNYNFVFPTVEKVFITNDNSSQSQAKQDLFGGSEPIEVNSEDLPF
>2WP0D
MDTNNNIEKEILALVKQNPKVSLIEYENYFSQLKYNPNASKSDIAFFYAPNQVLCTTITAKYGAL
LKEILSQNKVGMHLAHSVDVRIEVAPKIQINAQSNINYKAIKTSVKD
>2WP0B
MKNFYDWIKEFIRDQGEFIAQQSGWLELERSSYAKLIAQTISHVLNGGSLLVSADSSRHWFLNYI
LSNLNPKDLKERPLLSVIDFNASSFYPKNDANLSLATIEMTYQNPMFWHVGKIENEGLKTILLSK
IPSFLWLFEELKEDCLLLKEHDSLLDYKLLQLFKLFENALFSVLYNKVTL
>2XE0B

NTKYNKEFLLYLAGFVDGDGSIIAQIKPRASNKFAHQLSLTFAVTQKTQRRWFLDKLVDEIGVGY
VYDSGSVSDYRLSEIKPLHNFLTQLQPFLKLKQKQANLVLAIIEQLPSAKASPDAFLEVCTWVDQ
IAALNDSKTRATTSATVRAALD
>2XIWB
MRGSHHHHHHGSVKVKFLLNGEEKEVDTSKIRDVSRQGKNVKFLYNDNGKYGAGNVDEKDAPKEL
LDMLARAEREKKLN
>2XMAA
GSHMTYVILPLEMKKGRGYVYQLEYHLIWCVKYRHQVLVGEVADGLKDILRDIAAQNGLEVITME
VMPDHVHLLLSATPQQAIPDFVKALKGASARRMFVAYPQLKEKLWGGNLWNPSYCILTVSENTRA
QIQKYIESQHDKE
>2YPFA
VDLRTLAYSQQQQEKIKPKVRSTVAQHHEALVAHAFTHAHIVALSQHPAALATVAVKYQDMIAAL
PEATHEAIVAVAKQWSGARALEALLTVAGELRGPPLQLDTGQLLKIAKRGGVTAVEAVHAWRNAL
TGAPLNLTPEQVVAIASHDGGKQALETVQRLLPVLCQAHGLTPQQVVAIASNGGGKQALETVQRL
LPVLCQAHGLTPEQVVAIASNIGGKQALETVQALLPVLCQAHGLTPQQVVAIASNGGGKQALETV
QRLLPVLCQAHGLTPEQVVAIASNIGGKQALETVQALLPVLCQAHGLTPEQVVAIASNIGGKQAL
ETVQALLPVLCQAHGLTPEQVVAIASNIGGKQALETVQALLPVLCQAHGLTPEQVVAIASHDGGK
QALETVQRLLPVLCQAHGLTPEQVVAIASHDGGKQALETVQRLLPVLCQAHGLTPQQVVAIASNG
GGKQALETVQRLLPVLCQAHGLTPEQVVAIASNIGGKQALETVQALLPVLCQAHGLTPEQVVAIA
SNIGGKQALETVQALLPVLCQAHGLTPEQVVAIASHDGGKQALETVQRLLPVLCQAHGLTPEQVV
AIASHDGGKQALETVQRLLPVLCQAHGLTPEQVVAIASHDGGKQALETVQRLLPVLCQAHGLTPQ
QVVAIASNGGGKQALETVQRLLPVLCQAAGLTPEQVVAIASHDGGKQALETVQRLLPVLCQAHGL
TPQQVVAIASNGGGRPALESIVAQLSRPDPGSSAALEHHHHHH
>2YRQA
GSSGSSGMGKGDPKKPRGKMSSYAFFVQTCREEHKKKHPDASVNFSEFSKKCSERWKTMSAKEKG
KFEDMAKADKARYEREMKTYIPPKGETKKKFKDPNAPKRPPSAFFLFCSEYRPKIKGEHPGLSIG
DVAKKLGEMWNNTAADDKQPYEKKAAKLKEKYEKDIAAYRAKG
>2YVAB
MQERIKACFTESIQTQIAAAEALPDAISRAAMTLVQSLLNGNKILCCGNGTSAANAQHFAASMIN
RFETERPSLPAIALNTDNVVLTAIANDRLHDEVYAKQVRALGHAGDVLLAISTRGNSRDIVKAVE
AAVTRDMTIVALTGYDGGELAGLLGPQDVEIRIPSHRSARIQEMHMLTVNCLCDLIDNTLFPHQD
D
>2Z3XC
AKLLIPQAASAIEQMKLEIASEFGVQLGAETTSRANGSVGGEITKRLVRLAQQNMGGQFHGQQ
>2Z4RC
MKERILQEIKTRVNRKSWELWFSSFDVKSIEGNKVVFSVGNLFIKEWLEKKYYSVLSKAVKVVLG
NDATFEITYEAFEPHSSYSEPLVKKRAVLLTPLNPDYTFENFVVGPGNSFAYHAALEVAKHPGRY
NPLFIYGGVGLGKTHLLQSIGNYVVQNEPDLRVMYITSEKFLNDLVDSMKEGKLNEFREKYRKKV
DILLIDDVQFLIGKTGVQTELFHTFNELHDSGKQIVICSDREPQKLSEFQDRLVSRFQMGLVAKL
EPPDEETRKSIARKMLEIEHGELPEEVLNFVAENVDDNLRRLRGAIIKLLVYKETTGKEVDLKEA
ILLLKDFIKPNRVKAMDPIDELIEIVAKVTGVPREEILSNSRNVKALTARRIGMYVAKNYLKSSL
RTIAEKFNRSHPVVVDSVKKVKDSLLKGNKQLKALIDEVIGEISRRALSG
>2ZDSF
MPRNFTLFTGQWADLPLEEVCRLARDFGYDGLELACWGDHFEVDKALADPSYVDSRHQLLDKYGL

KCWAISNHLVGQAVCDAIIDERHEAILPARIWGDGDAEGVRQRAAAEIKDTARAAARLGVDTVIG
FTGSAIWHLVAMFPPAPESMIERGYQDFADRWNPILDVFDAEGVRFAHEVHPSEIAYDYWTTHRA
LEAVGHRPAFGLNFDPSHFVWQDLDPVGFLWDFRDRIYHVDCKEARKRLDGRNGRLGSHLPWGDP
RRGWDFVSAGHGDVPWEDVFRMLRSIDYQGPVSVEWEDAGMDRLQGAPEALTRLKAFDFEPPSAS
FDAAFNSLEHHHHHH
>2ZQEA
MREVKEVDLRGLTVAEALLEVDQALEEARALGLSTLRLLHGKGTGALRQAIREALRRDKRVESFA
DAPPGEGGHGVTVVALRP
>2ZTCD
MASMTGGQQMGRGSEFMIASVRGEVLEVALDHVVIEAAGVGYRVNATPATLATLRQGTEARLITA
MIVREDSMTLYGFPDGETRDLFLTLLSVSGVGPRLAMAALAVHDAPALRQVLADGNVAALTRVPG
IGKRGAERMVLELRDKVGVAATGGALSTNGHAVRSPVVEALVGLGFAAKQAEEATDTVLAANHDA
TTSSALRSALSLLGKAR
>3AL2A
GPLGSLKKQYIFQLSSLNPQERIDYCHLIEKLGGLVIEKQCFDPTCTHIVVGHPLRNEKYLASVA
AGKWVLHRSYLEACRTAGHFVQEEDYEWGSSSILDVLTGINVQQRRLALAAMRWRKKIQQRQESG
IVEGAFSGWKVILHVDQSREAGFKRLLQSGGAKVLPGHSVPLFKEATHLFSDLNKLKPDDSGVNI
AEAAAQNVYCLRTEYIADYLMQESPPHVENYCLPEAISFI
>3AQQD
MSSEPPPPPQPPTHQASVGLLDTPRSRERSPSPLRGNVVPSPLPTRRTRTFSATVRASQGPVYKG
VCKCFCRSKGHGFITPADGGPDIFLHISDVEGEYVPVEGDEVTYKMCSIPPKNEKLQAVEVVITH
LAPGTKHETWSGHVISS
>3ASKA
SLYKVNEYVDARDTNMGAWFEAQVVRVTRKAPSRPALEEDVIYHVKYDDYPENGVVQMNSRDVRA
RARTIIKWQDLEVGQVVMLNYNPDNPKERGFWYDAEISRKRETRTARELYANVVLGDDSLNDCRI
IFVDEVFKIERPGEGSPMVDNPMRRKSGPSCKHCKDDVNRLCRVCACHLCGGRQDPDKQLMCDEC
DMAFHIYCLDPPLSSVPSEDEWYCPECRNDA
>3AXJB
MPKNGGAGHRNTAPRKRQIPAAQLDEDSPIVQQFRIYSNELIMKHDRHERIVKLSRDITIESKRI
IFLLHSIDSRKQNKEKVLEEARQRLNKLIAVNFRAVALELRDQDVYQFRSSYSPGLQEFIQAYTY
MEYLCHEDAEGENETKSVSDWQAIQAVMQYVEESSQPKEEPTEGEDVQAIAQVESPKKFQFFVDP
TEYILGLSDLTGELMRRCINSLGSGDTDTCLDTCKALQHFYSGYISLNCQRARELWRKITTMKQS
VLKAENVCYNVKVRGGEAAKWGATFDQKPADEVDEGFY
>3B0CT
GSTREPEIASSLIKQIFSHYVKTPVTRDAYKIVEKCSERYFKQISSDLEAYSQHAGRKTVEMADV
ELLMRRQGLVTDKMPLHVLVERHLPLEYRKLLIPIAVSGNKVIPCK
>3BOSB
GMRSNRVTQHPPLQLSLPVHLPDDETFTSYYPAAGNDELIGALKSAASGDGVQAIYLWGPVKSGR
THLIHAACARANELERRSFYIPLGIHASISTALLEGLEQFDLICIDDVDAVAGHPLWEEAIFDLY
NRVAEQKRGSLIVSASASPMEAGFVLPDLVSRMHWGLTYQLQPMMDDEKLAALQRRAAMRGLQLP
EDVGRFLLNRMARDLRTLFDVLDRLDKASMVHQRKLTIPFVKEMLRL
>3BRFA
SGPLGSGDSVQSLTSDRMIDFLSNKEKYECVISIFHAKVAQKSYGNEKRFFCPPPCIYLIGQGWK
LKKDRVAQLYKTLKASAQKDAAIENDPIHEQQATELVAYIGIGSDTSERQQLDFSTGKVRHPGDQ

RQDPNIYDYCAAKTLYISDSDKRKYFDLNAQFFYGCGMEIGGFVSQRIKVISKPSKKKQSMKNTD
CKYLCIASGTKVALFNRLRSQTVSTRYLHVEGNAFHASSTKWGAFTIHLFDDERGLQETDNFAVR
DGFVYYGSVVKLVDSVTGIALPRLRIRKVDKQQVILDASCSEEPVSQLHKCAFQMIDNELVYLCL
SHDKIIQHQATAINEHRHQINDGAAWTIISTDKAEYRFFEAMGQVANPISPCPVVGSLEVDGHGE
ASRVELHGRDFKPNLKVWFGATPVETTFRSEESLHCSIPPVSQVRNEQTHWMFTNRTTGDVEVPI
SLVRDDGVVYSSGLTFSYKSLER
>3BS3A
SNAMSNNQQMMLNRIKVVLAEKQRTNRWLAEQMGKSENTISRWCSNKSQPSLDMLVKVAELLNVD
PRQLINGKIKI
>3BTPB
MVIIKLNANKNMPVLAVEKPQEIHKEELSDHHQSNGFTSLDLEMIELENFVLHCPLPEENLAG
>3BTPA
MDPKAEGNGENITETAAGNVETSDFVNLKRQKREGVNSTGMSEIDMTGSQETPEHNMHGSPTHTD
DLGPRLDADMLDSQSSHVSSSAQGNRSEVENELSNLFAKMALPGHDRRTDEYILVRQTGQDKFAG
TTKCNLDHLPTKAEFNASCRLYRDGVGNYYPPPLAFERIDLPEQLAAQLHNLEPREQSKQCFQYK
LEVWNRAHAEMGITGTDIFYQTDKNIKLDRNYKLRPEDRYIQTEKYGRREIQKRYEHQFQAGSLL
PDILIKTPQNDIHFSYRFAGDAYANKRFEEFERAIKTKYGSDTEIKLKSKSGIMHDSKYLESWER
GSADIRFAEFAGENRAHNKQFPAATVNMGRQPDGQGGMTRDRHVSVDYLLQNLPNSPWTQALKEG
KLWDRVQVLARDGNRYMSPSRLEYSDPEHFTQLMDQVGLPVSMGRQSHANSVKFEQFDRQAAVIV
ADGPNLREVPDLSPEKLQQLSQKDVLIADRNEKGQRTGTYTNVVEYERLMMKLPSDAAQLLAEPS
DRYSRAFVRPEPALPPISDSRRTYESRPRGPTVNSL
>3BU8B
GAGEARLEEAVNRWVLKFYFHEALRAFRGSRYGDFRQIRDIMQALLVRPLGKEHTVSRLLRVMQC
LSRIEEGENLDCSFDMEAELTPLESAINVLEMIKTEFTLTEAVVESSRKLVKEAAVIICIKNKEF
EKASKILKKHMSKDPTTQKLRNDLLNIIREKNLAHPVIQNFSYETFQQKMLRFLESHLDDAEPYL
LTMAKKALKSESAASSTGKEDKQPAPGPVEKPPREPARQL
>3C1DB
GPAYARLLDRAVRILAVRDHSEQELRRKLAAPIMGKNGPEEIDATAEDYERVIAWCHEHGYLDDS
RFVARFIASRSRKGYGPARIRQELNQKGISREATEKAMREADIDWAALARDQATRKYGEPLPTVF
SEKVKIQRFLLYRGYLMEDIQDIWRNFAD
>3C1YB
MGSSHHHHHHSSGLVPRGSHMGVKSLVPQELIEKIKLISPGTELRKALDDIINANFGALIFLVDD
PKKYEDVIQGGFWLDTDFSAEKLYELSKMDGAIVLSEDITKIYYANVHLVPDPTIPTGETGTRHR
TAERLAKQTGKVVIAVSRRRNIISLYYKNYKYVVNQVDFLISKVTQAISTLEKYKDNFNKLLSEL
EVLELENRVTLADVVRTLAKGFELLRIVEEIRPYIVELGEEGRLARMQLRELTEDVDDLLVLLIM
DYSSEEVEEETAQNILQDFITRREPSPISISRVLGYDVQQAAQLDDVLVSARGYRLLKTVARIPL
SIGYNVVRMFKTLDQISKASVEDLKKVEGIGEKRARAISESISSLKHRKTSE
>3CNBC
MSLNVKNDFSILIIEDDKEFADMLTQFLENLFPYAKIKIAYNPFDAGDLLHTVKPDVVMLDLMMV
GMDGFSICHRIKSTPATANIIVIAMTGALTDDNVSRIVALGAETCFGKPLNFTLLEKTIKQLVEQ
KKATSEGHHHHHH
>3D6WB
GKSVVTLKTTDGWIPVPFSKVMYLEAKDKKTYVNAEELTGTHKYSLQEFEYLLPKDSFIRCHRSF
IVNVNHIKAIYPDTHSTFLLSMDNGERVPVSQSYASYFRKLLGFGS

>3DPJA
SNAMVQAQTRDQIVAAADELFYRQGFAQTSFVDISAAVGISRGNFYYHFKTKDEILAEVIRLRLA
RTAQMLADWQGTGDSPRARIASFIDLMIMNRAKITRYGCPVGSLCTELSKLDHAAQGQANGLFTL
FRDWLQRQFAEAGCTTEAPALAMHLLARSQGAATLAQSFHDEGFLRSEVADMHRWLDNTLPMTT
>3EI4F
MHHHHHHRRLVPRGSGGRQKTSEIVLRPRNKRSRSPLELEPEAKKLCAKGSGPSRRCDSDCLWVG
LAGPQILPPCRSIVRTLHQHKLGRASWPSVQQGLQQSFLHTLDSYRILQKAAPFDRRATSLAWHP
THPSTVAVGSKGGDIMLWNFGIKDKPTFIKGIGAGGSITGLKFNPLNTNQFYASSMEGTTRLQDF
KGNILRVFASSDTINIWFCSLDVSASSRMVVTGDNVGNVILLNMDGKELWNLRMHKKKVTHVALN
PCCDWFLATASVDQTVKIWDLRQVRGKASFLYSLPHRHPVNAACFSPDGARLLTTDQKSEIRVYS
ASQWDCPLGLIPHPHRHFQHLTPIKAAWHPRYNLIVVGRYPDPNFKSCTPYELRTIDVFDGNSGK
MMCQLYDPESSGISSLNEFNPMGDTLASAMGYHILIWSQEEARTRK
>3EIVD
MAGETVITVVGNLVDDPELRFTPSGAAVAKFRVASTPRTFDRQTNEWKDGESLFLTCSVWRQAAE
NVAESLQRGMRVIVQGRLKQRSYEDREGVKRTVYELDVDEVGASLRSATAKVTKTSGQGRGGQGG
YGGGGGGQGGGGWGGGPGGGQQGGGAPADDPWATGGAPAGGQQGGGGQGGGGWGGGSGGGGGYSD
EPPF
>3EQXB
GMEWQAEQAYNHLPPLPLDSKLAELAETLPILKACIPARAALAELKQAGELLPNQGLLINLLPLL
EAQGSSEIENIVTTTDKLFQYAQEDSQADPMTKEALRYRTALYQCFTQLSNRPLCVTTALEICST
IKSVQMDVRKVPGTSLTNQATGEVIYTPPAGESVIRDLLSNWEAFLHNQDDVDPLIKMAMAHYQF
EAIHPFIDGNGRTGRVLNILYLIDQQLLSAPILYLSRYIVAHKQDYYRLLLNVTTQQEWQPWIIF
ILNAVEQTAKWTTHKIAAARELIAHTTEYVRQQLPKIYSHELVQVIFEQPYCRIQNLVESGLAKR
QTASVYLKQLCDIGVLEEVQSGKEKLFVHPKFVTLMTKDSNQFSRYAL
>3EUSB
QAMTKTLRTPEHVYLCQRLRQARLDAGLTQADLAERLDKPQSFVAKVETRERRLDVIEFAKWMAA
CEGLDVVSEIVATIAEGRAQA
>3EYIB
HMASPQFSQQREEDIYRFLKDNGPQRALVIAQALGMRTAKDVNRDLYRMKSRHLLDMDEQSKAWT
IYRWTIY
>3EZ2A
MSDSSQLHKVAQRANRMLNVLTEQVQLQKDELHANEFYQVYAKAALAKLPLLTRANVDYAVSEME
EKGYVFDKRPAGSSMKYAMSIQNIIDIYEHRGVPKYRDRYSEAYVIFISNLKGGVSKTVSTVSLA
HAMRAHPHLLMEDLRILVIDLDPQSSATMFLSHKHSIGIVNATSAQAMLQNVSREELLEEFIVPS
VVPGVDVMPASIDDAFIASDWRELCNEHLPGQNIHAVLKENVIDKLKSDYDFILVDSGPHLDAFL
KNALASANILFTPLPPATVDFHSSLKYVARLPELVKLISDEGCECQLATNIGFMSKLSNKADHKY
CHSLAKEVFGGDMLDVFLPRLDGFERCGESFDTVISANPATYVGSADALKNARIAAEDFAKAVFD
RIEFIRSN
>3F1ZA
GASKAFYSAGDKLFQPGDDAVASMQTYSVAQFLQPFTLNPAKASSDYLGKWVKVRGVIVDIRRKS
GIAGSYYFIVTMRDEQNKTDKRLTFNFGSHNSADVEALSNGSVATIVGQVHQVQDSTIPTLQNPK
VVK
>3F6CB
SLNAIIIDDHPLAIAAIRNLLIKNDIEILAELTEGGSAVQRVETLKPDIVIIDVDIPGVNGIQVL

ETLRKRQYSGIIIIVSAKNDHFYGKHCADAGANGFVSKKEGMNNIIAAIEAAKNGYCYFPFSLNR
FVGS
>3FDQB
MPKSEIRKLLQEIKKQVDNPGNSSTTEIKKMASEAGIDEQTAEEIYHLLTEFYQAVEEHGGIEKY
MHSNISWLKIELELLSACYQIAILEDMKVLDISEMLSLNDLRIFPKTPSQLQNTYYKLKKELIQV
EDIPKNKPGRKRKTQKNTKKEKTNIFGKVVPALEHHHHHH
>3FDQA
MPKSEIRKLLQEIKKQVDNPGNSSTTEIKKMASEAGIDEQTAEEIYHLLTEFYQAVEEHGGIEKY
MHSNISWLKIELELLSACYQIAILEDMKVLDISEMLSLNDLRIFPKTPSQLQNTYYKLKKELIQV
EDIPKNKPGRKRKTQKNTKKEKTNIFGKVVPALEHHHHHH
>3FHWB
MNTLELSARVLECGAMRHTPAGLPALELLLVHESEVVEAGHPRRVELTISAVALGDLALLLADTP
LGTEMQVQGFLAPARKDSVKVKLHLQQARRIAGSMGRDPLVGLEHHHHHH
>3FRQA
GMPRPKLKSDDEVLEAATVVLKRCGPIEFTLSGVAKEVGLSRAALIQRFTNRDTLLVRMMERGVE
QVRHYLNAIPIGAGPQGLWEFLQVLVRSMNTRNDFSVNYLISWYELQVPELRTLAIQRNRAVVEG
IRKRLPPGAPAAAELLLHSVIAGATMQWAVDPDGELADHVLAQIAAILCLMFPEHDDFQLLQAHA
>3FYMA
MKTVGEALKGRRERLGMTLTELEQRTGIKREMLVHIENNEFDQLPNKNYSEGFIRKYASVVNIEP
NQLIQAHQDEIPSNQAEWDEVITVFNNNKDLDYKSKSKEPIQLLVIMGITVLITLLLWIMLVLIF
>3G1CA
MNNKLKTQAVEQLFQAILSLKDLDEAYDFFEDVCTINEILSLSQRFEVAKMLREHRTYLDIAEKT
GASTATISRVNRSLNYGNDGYDRVFERLGMLEKESEDNK
>3GIOB
MGSSHHHHHHSSGLVPRGSHMPNTSQRNSFLQDVPYWMLQNRSEYITQGVDSSHIVDGKKTEEIE
KIATKRATIRVAQNIVHKLKEAYLSKTNRIKQKITNEMFIQMTQPIYDSLMNVDRLGIYINPNNE
EVFALVRARGFDKDALSEGLHKMSLDNQAVSILVAKVEEIFKDSVNYGDVKVPIAM
>3GN5B
GHMKCPVCHQGEMVSGIKDIPYTFRGRKTVLKGIHGLYCVHCEESIMNKEESDAFMAQVKAFRAS
VNAETVAPEFIVKVRKKLSLTQKEASEIFGGGVNAFSRYEKGNAQPHPSTIKLLRVLDKHPELLN
EIR
>3GVAB
MRMDEFYTKVYDAVCEIPYGKVSTYGEIARYVGMPSYARQVGQAMKHLHPETHVPWHRVINSRGT
ISKRDISAGEQRQKDRLEEEGVEIYQTSLGEYKLNLPEYMWKPGSHHHHHH
>3GXQB
ENSVFFGKKKKVSLHLLVDPDMKDEIIKYAQEKDFDNVSQAGREILKKGLEQIA
>3GYDB
MGSDKIHHHHHHENLYFQGMYPDLVHLGGADKYFEEILEIVNKIKLFGDFSNEEVRYLCSYMQCY
AAPRDCQLLTEGDPGDYLLLILTGEVNVIKDIPNKGIQTIAKVGAGAIIGEMSMIDGMPRSASCV
ASLPTDFAVLSRDALYQLLANMPKLGNKVLIRLLQLLTARFRESYDRILPKTLGELI
>3GZ5B
GSHMTEAEYLANYDPKAFKAQLLTVDAVLFTYHDQQLKVLLVQRSNHPFLGLWGLPGGFIDETCD
ESLEQTVLRKLAEKTAVVPPYIEQLCTVGNNSRDARGWSVTVCYTALMSYQACQIQIASVSDVKW
WPLADVLQMPLAFDHLQLIEQARERLTQKALYSLVPGFALSEPFTLPELQHVHEVLLGKPIQGKS

FRRRVEQADLLIDTGLKRTERGRPANLYCLKPDTASYRFLRNLEC
>3H4LB
GSMTQIHQINDIDVHRITSGQVITDLTTAVKELVDNSIDANANQIEIIFKDYGLESIECSDNGDG
IDPSNYEFLALKHYTSKIAKFQDVAKVQTLGFRGEALSSLCGIAKLSVITTTSPPKADKLEYDMV
GHITSKTTTSRNKGTTVLVSQLFHNLPVRQKEFSKTFKRQFTKCLTVIQGYAIINAAIKFSVWNI
TPKGKKNLILSTMRNSSMRKNISSVFGAGGMRGLEEVDLVLDLNPFKNRMLGKYTDDPDFLDLDY
KIRVKGYISQNSFGCGRNSKDRQFIYVNKRPVEYSTLLKCCNEVYKTFNNVQFPAVFLNLELPMS
LIDVNVTPDKRVILLHNERAVIDIFKTTLSDYYNRQELALPK
>3HI2D
GSHMEKRTPHTRLSQVKKLVNAGQVRTTRSALLNADELGLDFDGMCNVIIGLSESDFYKSMTTYS
DHTIWQDVYRPRLVTGQVYLKITVIHDVLIVSFKEK
>3HOSA
MSSFVPNKEQTRTVLIFCFHLKKTAAESHRMLVEAFGEQVPTVKTCERWFQRFKSGDFDVDDKEH
GKPPKRYEDAELQALLDEDDAQTQKQLAEQLEVSQQAVSNRLREMGKIQKVGRWVPHELNERQME
RRKNTCEILLSRYKRKSFLHRIVTGDEKWIFFVNPKRKKSYVDPGQPATSTARPNRFGKKTMLCV
WWDQSGVIYYELLKPGETVNAARYQQQLINLNRALQRKRPEYQKRQHRVIFLHDNAPSHTARAVR
DTLETLNWEVLPHAAYSPDLAPSDYHLFASMGHALAEQRFDSYESVKKWLDEWFAAKDDEFYWRG
IHKLPERWEKCVASDGKYFE
>3HTAD
MGSSHHHHHHSSGLVPRGSHMPRRHDPERRQRIIDAAIRVVGQKGIAGLSHRTVAAEADVPLGST
TYHFATLDDLMVAALRQANEGFARVVAAHPALSDPEADLSGELARVLGEWLGGDRTGVELEYELY
LAALRRPALRPVAAEWAEGVGALLAARTDPTTARALVAVLDGICLQVLLTDTPYDEEYAREVLTR
LIPVPATRDGRGPGSHPPATAG
>3I54D
MGSSHHHHHHSSGLGGTENLYFQSHMDEILARAGIFQGVEPSAIAALTKQLQPVDFPRGHTVFAE
GEPGDRLYIIISGKVKIGRRAPDGRENLLTIMGPSDMFGELSIFDPGPRTSSATTITEVRAVSMD
RDALRSWIADRPEISEQLLRVLARRLRRTNNNLADLIFTDVPGRVAKQLLQLAQRFGTQEGGALR
VTHDLTQEEIAQLVGASRETVNKALADFAHRGWIRLEGKSVLISDSERLARRAR
>3II2A
GSEMSVVEYEVVSKNLTSKMSHELLFSVKKRWFVKPFRHDRQLGKLHYKLLPGNYIKFGLYVLKN
QDYARFEIAWVHVDKDGKIEERTVYSIETYWHIFIDIENDLNCPYVLAKFIEMRPEFHKTAWVEE
SNYSIAEDDIQMVESIKRYLERKIASD
>3II6X
GAMGSKISNIFEDVEFCVMSGTDSQPKPDLENRIAEFGGYIVQNPGPDTYCVIAGSENIRVKNII
LSNKHDVVKPAWLLECFKTKSFVPWQPRFMIHMCPSTKEHFAREYDCYGDSYFIDTDLNQLKEVF
SGIKNSNEQTPEEMASLIADLEYRYSWDCSPLSMFRRHTVYLDSYAVINDLSTKNEGTRLAIKAL
ELRFHGAKVVSCLAEGVSHVIIGEDHSRVADFKAFRRTFKRKFKILKESWVTDSIDKCELQEENQ
YLI
>3II6A
MERKISRIHLVSEPSITHFLQVSWEKTLESGFVITLTDGHSAWTGTVSESEISQEADDMEMEKGK
YVGELRKALLSGAGPADVYTFNFSKESCYFFFEKNLKDVSFRLGSFNLEKVENPAEVIRELICYC
LDTTAENQAKNEHLQKENERLLRDWNDVQGRFEKCVSAKEALETDLYKRFILVLNEKKTKIRSLH
NKLLNAAQ
>3IO5B

GSHMDVVRTKIPMMNIALSGEITGGMQSGLLILAGPSKSFKSNFGLTMVSSYMRQYPDAVCLFYD
SEFGITPAYLRSMGVDPERVIHTPVQSLEQLRIDMVNQLDAIERGEKVVVFIDSLGNLASKKETE
DALNEKVVSDMTRAKTMKSLFRIVTPYFSTKNIPCIAINHTYETQEMFSKTVMGGGTGPMYSADT
VFIIGKRQIKDGSDLQGYQFVLNVEKSRTVKEKSKFFIDVKFDGGIDPYSGLLDMALELGFVVKP
KNGWYAREFLDEETGEMIREEKSWRAKDTNCTTFWGPLFKHQPFRDAIKRAYQLGAIDSNEIVEA
EVDELINS
>3IV5B
MFEQRVNSDVLTVSTVNSQDQVTQKPLRDSVKQALKNYFAQLNGQDVNDLYELVLAEVEQPLLDM
VMQYTRGNQTRAALMMGINRGTLRKKLKKYGMN
>3IVPD
MRKKEDKYDFRALGLAIKEARKKQGLTREQVGAMIEIDPRYLTNIENKGQHPSLQVLYDLVSLLN
VSVDEFFLPASSQVKSTKRRQLENKIDNFTDADLVIMESVADGIVKSKEVGEMAGENLYFQ
>3JU0A
MSLTDSKVKNAKSLEKEYKLTDGFGMHLLVHPNGSKYWRLSYRFEKKQRLLALGVYPAVSLADAR
QRRDEAKKLLAAGIDPSAKKQADNKTIQEKRNNTRLEHHHHHH
>3K2AB
GSGIFPKVATNIMRAWLFQHLTHPYPSEEQKKQLAQDTGLTILQVNNWFINARRRIVQPMIDQSN
RA
>3K4XA
GPHMASMLEAKFEEASLFKRIIDGFKDCVQLVNFQCKEDGIIAQAVDDSRVLLVSLEIGVEAFQE
YRCDHPVTLGMDLTSLSKILRCGNNTDTLTLIADNTPDSIILLFEDTKKDRIAEYSLKLMDIDAD
FLKIEELQYDSTLSLPSSEFSKIVRDLSQLSDSINIMITKETIKFVADGDIGSGSVIIKPFVDME
HPETSIKLEMDQPVDLTFGAKYLLDIIKGSSLSDRVGIRLSSEAPALFQFDLKSGFLQFFLAPKF
NDEGSNSQSNGSGALEAKFEEASLFKRIIDGFKDCVQLVNFQCKEDGIIAQAVDDSRVLLVSLEI
GVEAFQEYRCDHPVTLGMDLTSLSKILRCGNNTDTLTLIADNTPDSIILLFEDTKKDRIAEYSLK
LMDIDADFLKIEELQYDSTLSLPSSEFSKIVRDLSQLSDSINIMITKETIKFVADGDIGSGSVII
KPFVDMEHPETSIKLEMDQPVDLTFGAKYLLDIIKGSSLSDRVGIRLSSEAPALFQFDLKSGFLQ
FFLAPKFNDEGSNSQASNSGALEAKFEEASLFKRIIDGFKDCVQLVNFQCKEDGIIAQAVDDSRV
LLVSLEIGVEAFQEYRCDHPVTLGMDLTSLSKILRCGNNTDTLTLIADNTPDSIILLFEDTKKDR
IAEYSLKLMDIDADFLKIEELQYDSTLSLPSSEFSKIVRDLSQLSDSINIMITKETIKFVADGDI
GSGSVIIKPFVDMEHPETSIKLEMDQPVDLTFGAKYLLDIIKGSSLSDRVGIRLSSEAPALFQFD
LKSGFLQFFLAPKFNDEE
>3KDEC
MKYCKFCCKAVTGVKLIHVPKCAIKRKLWEQSLGCSLGENSQICDTHFNDSQWKAAPAKGQTFKR
RRLNADAVPSKV
>3KHKB
MSLDIEQQFLNDLDNQLWRAADKLRSNLDAANYKHVVLGLIFLKYVSDAFEERQQELTELFQKDD
DDNIYYLPREDYDSDEAYQQAIAEELEIGDYYTEKNVFWVPKTARWNKLRDVITLPTGSVIWQDE
QGEDVKLRSVSWLIDNAFDDIEKANPKLKGILNRISQYQLDADKLIGLINEFSLTSFNNPEYNGE
KLNLKSKDILGHVYEYFLGQFALAEGKQGGQYYTPKSIVTLIVEMLEPYKGRVYDPAMGSGGFFV
SSDKFIEKHANVKHYNASEQKKQISVYGQESNPTTWKLAAMNMVIRGIDFNFGKKNADSFLDDQH
PDLRADFVMTNPPFNMKDWWHEKLADDPRWTINTNGEKRILTPPTGNANFAWMLHMLYHLAPTGS
MALLLANGSMSSNTNNEGEIRKTLVEQDLVECMVALPGQLFTNTQIPACIWFLTKDKNAKNGKRD
RRGQVLFIDARKLGYMKDRVLRDFKDEDIQKLADTFHNWQQEWSEENNQAGFCFSADLALIRKND

FVLTPGRYVGAEAEEDEGHHHHHH
>3KJOA
MSLVPATNYIYTPLNQLKGGTIVNVYGVVKFFKPPYLSKGTDYCSVVTIVDQTNVKLTCLLFSGN
YEALPIIYKNGDIVRFHRLKIQVYKKETQGITSSGFASLTFEGTLGAPIIPRTSSKYFNFTTEDH
KMVEALRVWASTHMSPSWTLLKLCDVQPMQYFDLTCQLLGKAEVDGASFLLKVWDGTRTPFPSWR
VLIQDLVLEGDLSHIHRLQNLTIDILVYDNHVHVARSLKVGSFLRIYSLHTKLQSMNSENQTMLS
LEFHLHGGTSYGRGIRVLPESNSDVDQLKKDLESANLTA
>3KOJB
MGHHHHHHSHMNSCILQATVVEAPQLRYAQDNQTPVAEMVVQFPGLSSKDAPARLKVVGWGAVAQ
ELQDRCRLNDEVVLEGRLRINSLLKPDGNREKQTELTVTRVHH
>3LDAA
MSQVQEQHISESQLQYGNGSLMSTVPADLSQSVVDGNGNGSSEDIEATNGSGDGGGLQEQAEAQG
EMEDEAYDEAALGSFVPIEKLQVNGITMADVKKLRESGLHTAEAVAYAPRKDLLEIKGISEAKAD
KLLNEAARLVPMGFVTAADFHMRRSELICLTTGSKNLDTLLGGGVETGSITELFGEFRTGKSQLC
HTLAVTCQIPLDIGGGEGKCLYIDTEGTFRPVRLVSIAQRFGLDPDDALNNVAYARAYNADHQLR
LLDAAAQMMSESRFSLIVVDSVMALYRTDFSGRGELSARQMHLAKFMRALQRLADQFGVAVVVTN
QVVAQVDGGMAFNPDPKKPIGGNIMAYSSTTRLGFKKGKGCQRLCKVVDSPCLPEAECVFAIYED
GVGDPREEDE
>3LHKD
SNAKIIGYARVSFNAQKDDLERQIQLIKSYAEENGWDIQILKDIGSGLNEKRKNYKKLLKMVMNR
KVEKVIIAYPDRLTRFGFETLKEFFKSYGTEIVIINKKHKTPQEELVEDLITIVSHFAGKLYGMH
SHKYKKLTKTVKEIVREEDAKEKE
>3M03C
MSNIGIRDLAVQFSCIEAVNMASKILKSYESSLPQTQQVDLDLSRPLFTSAALLSACKILKLKVD
KNKMVATSGVKKAIFDRLCKQLEKIGQQVD
>3M8EB
MGSSHHHHHHSSGLVPRGSHMNRDHFYTLNIAEIAERIGNDDCAYQVLMAFINENGEAQMLNKTA
VAEMIQLSKPTVFATVNSFYCAGYIDETRVGRSKIYTLSDLGVEIVECFKQKAMEMRNL
>3MAJA
GHMDVGERSSDQGTTVLTEAQRIDWMRLIRAENVGPRTFRSLINHFGSARAALERLPELARRGGA
ARAGRIPSEDEARREIEAGRRIGVELVAPGETGYPTRLATIDDAPPLLGVHALPEALAVMARPMI
AIVGSRNASGAGLKFAGQLAADLGAAGFVVISGLARGIDQAAHRASLSSGTVAVLAGGHDKIYPA
EHEDLLLDIIQTRGAAISEMPLGHVPRGKDFPRRNRLISGASVGVAVIEAAYRSGSLITARRAAD
QGREVFAVPGSPLDPRAAGTNDLIKQGATLITSASDIVEAVASILERPIELPGREPEHAPPEGEP
DTGDRTRILALLGPSPVGIDDLIRLSGISPAVVRTILLELELAGRLERHGGSLVSLS
>3MKYB
MGSSHHHHHHSSGLVPRGSHYRPTSAYERGQRYASRLQNEFAGNISALADAENISRKIITRCINT
AKLPKSVVALFSHPGELSARSGDALQKAFTDKEELLKQQASNLHEQKKAGVIFEADEVITLLTSV
LKTSSASRTSLSSRHQFAPGATVLYKGDKMVLNLDRSRVPTECIEKIEAILKELEKPAP
>3MU6D
GRKKIQITRIMDERNRQVTFTKRKFGLMKKAYELSVLCDCEIALIIFNSSNKLFQYASTDMDKVL
LKYTAY
>3MUJB
SMEATPCIKAISPSEGWTTGGATVIIIGDNFFDGLQVVFGTMLVWSELITPHAIRVQTPPRHIPG

VVEVTLSYKSKQFCKGAPGRFVYTALNEPTIDYGFQRLQKVIPRHPGDPERLPKEVLLKRAADLV
EALYGMPH
>3N4PA
MGHHHHHHDYDIPTTENLYFQGGGTNKISQNTVLITDQSREEFDILRYSTLNTNAYDYFGKTLYV
YLDPAFTTNRKASGTGVAAVGAYRHQFLIYGLEHFFLRDLSESSEVAIAECAAHMIISVLSLHPY
LDELRIAVEGNTNQAAAVRIACLIRQSVQSSTLIRVLFYHTPDQNHIEQPFYLMGRDKALAVEQF
ISRFNSGYIKASQELVSYTIKLSHDPIEYLLEQIQNLHRVTLAEGTTARYSAKRQNRISDDLIIA
VIMATYLCDDIHAIRFRVS
>3NCTD
MKTELTLNVLQTMNAQEYEDIRAAGSDERRELTHAVMRELDAPDNWTMNGEYGSEFGGFFPVQVR
FTPAHERFHLALCSPGDVSQVWVLVLVNAGGEPFAVVQVQRRFASEAVSHSLALAASLDTQGYSV
NDIIHILMAEGGQV
>3NFHB
MTDSAIDIVDSVRTASKDLPTRAQLDEITSNDRPTPLANIDATDVEQIYPIESIIPKKELQFIRV
SSILKEADKEKKLELFPYQNNSKYVAKKLDSLTQPSQMTKLQLLYYLSLLLGVYENRRVNNKTKL
LERLNSPPEILVDGILSRFTVIKPGQFGRSKDRSYFIDPQNEDKILCYILAIIMHLDNFIVEITP
LAHELNLKPSKVVSLFRVLGAIVKGATVAQAEAFGIPKSTAASYKIATMKV
>3NO7A
GPEASARSEVKMTVTVGEERRARLRTAYTLTHLQEGHRTFSGFIAAALDAEVQRLEQRYNEGRRF
ENAERGVTRGRPLGS
>3NQUA
MGPRRRSRKPEAPRRRSPSPTPTPGPSRRGPSLGASSHQHSRRRQGWLKEIRKLQKSTHLLIRKL
PFSRLAREICVKFTRGVDFNWQAQALLALQEAAEAFLVHLFEDAYLLTLHAGRVTLFPKDVQLAR
RIRGLEEGLG
>3NR7B
GSHMSEALKILNNIRTLRAQARESTLETLEEMLEKLEVVVNERREEESAAAAEVEERTRKLQQYR
EMLIADGIDPNELLNSMAAAK
>3NXCA
MPPGKCLFSGVFCNMAEKQTAKRNRREEILQSLALMLESSDGSQRITTAKLAASVGVSEAALYRH
FPSKTRMFDSLIEFIEDSLITRINLILKDEKDTTARLRLIVLLLLGFGERNPGLTRILTGHALMF
EQDRLQGRINQLFERIEAQLRQVLREKRMREGEGYATDETLLASQILAFCEGMLSRFVRSEFKYR
PTDDFDARWPLIAAQLQ
>3O27B
MRPGIRKLVVLNPRAYKGGSGHTTFYLLIPKDIAEALDIKPDDTFILNMEQKDGDIVLSYKRVKE
LKI
>3OA6A
MKKHHHHHHMSASEGMKFKFHSGEKVLCFEPDPTKARVLYDAKIVDVIVGKDEKGRKIPEYLIHF
NGWNRSWDRWAAEDHVLRDTDENRRLQRKLARKAVARLRSTGRKK
>3OD8H
MGSSHHHHHHSSGLVPRGSHMAESSDKLYRVEYAKSGRASCKKCSESIPKDSLRMAIMVQSPMFD
GKVPHWYHFSCFWKVGHSIRHPDVEVDGFSELRWDDQQKVKKTAEAGGVTG
>3OKGA
MSHHHHHHSMDIEFMTEGPYKLPPGWRWVRLGEVCLPTERRDPTKNPSTYFVYVDISAIDSTVGK
IVSPKEILGQHAPSRARKVIRSGDVIFATTRPYLKNIALVPPDLDGQICSTGFCVIRANREFAEP

EFLFHLCRSDFITNQLTASKMRGTSYPAVTDNDVYNTLIPLPPLEEQRRIVAKVEALMERVREVR
RLRAEAQKDTELLMQTALAEVFPHPGADLPPGWRWVRLGEVCDIIMGQSPPSSTYNFEGNGLPFF
QGKADFGDLHPTPRIWCSAPQKVARPGDVLISVRAPVGSTNVANLACCIGRGLAALRPRDSLERF
WLLYYLHYLEPELSKMGAGSTFNAITKKDLQNVFIPLPPLEEQRRIVAYLDQIQQQVAALKRAQA
ETEAELKRLEQAILDKAFRGDL

>3OLCX

MSRNDKEPFFVKFLKSSDNSKCFFKALESIKEFQSEEYLQIITEEEALKIKENDRSLYICDPFSG
VVFDHLKKLGCRIVGPQVVIFCMHHQRCVPRAEHPVYNMVMSDVTISCTSLEKEKREEVHKYVQM
MGGRVYRDLNVSVTHLIAGEVGSKKYLVAANLKKPILLPSWIKTLWEKSQEKKITRYTDINMEDF
KCPIFLGCIICVTGLCGLDRKEVQQLTVKHGGQYMGQLKMNECTHLIVQEPKGQKYECAKRWNVH
CVTTQWFFDSIEKGFCQDESIYKTEPRPEALEHHHHHH

>3ON0D

MPKIQTYVNNNVYEQITDLVTIRKQEGIEEASLSNVSSMLLELGLRVYMIQQEKREGGFNQMEYN
KLMLENVSRVRAMCTEILKMSVLNQESIASGNFDYAVIKPAIDKFAREQVSIFFPDDEDDQE

>3P7NB

MRGSHHHHHHGMASMTGGQQMGRDLYDDDDKDHPFTMGQDRPIDGSGAPGADDTRVEVQPPAQWV
LDLIEASPIASVVSDPRLADNPLIAINQAFTDLTGYSEEECVGRNCRFLAGSGTEPWLTDKIRQG
VREHKPVLVEILNYKKDGTPFRNAVLVAPIYDDDDELLYFLGSQVEVDDDQPNMGMARRERAAEM
LKTLSPRQLEVTTLVASGLRNKEVAARLGLSEKTVKMHRGLVMEKLNLKTSADLVRIAVEAGI

>3P83F

GPLGSPEFPGRLMKAGIDEAGKGCVIGPLVVAGVACSDEDRLRKLGVKDSKKLSQGRREELAEEI
RKICRTEVLKVSPENLDERMAAKTINEILKECYAEIILRLKPEIAYVDSPDVIPERLSRELEEIT
GLRVVAEHKADEKYPLVAAASIIAKVEREREIERLKEKFGDFGSGYASDPRTREVLKEWIASGRI
PSCVRMRWKTVSNLRQKTLDDF

>3P83C

MIDVIMTGELLKTVTRAIVALVSEARIHFLEKGLHSRAVDPANVAMVIVDIPKDSFEVYNIDEEK
TIGVDMDRIFDISKSISTKDLVELIVEDESTLKVKFGSVEYKVALIDPSAIRKEPRIPELELPAK
IVMDAGEFKKAIAAADKISDQVIFRSDKEGFRIEAKGDVDSIVFHMTETELIEFNGGEARSMFSV
DYLKEFCKVAGSGDLLTIHLGTNYPVRLVFELVGGRAKVEYILAPRIESE

>3P9AA

MAAPKGNRFWEARSSHGRNPKFESPEALWAACCEYFEWVEANPLWEMKAFSYQGEVIQEPIAKMR
AMTITGLTLFIDVTLETWRTYRLREDLSEVVTRAEQVIYDQKFSGAAADLLNANIIARDLGLKEQ
SQVEDVTPDKGDRDKRRSRIKELFNRGTGRDS

>3PGGB

SYKVNYMSETPANKSQGGSNQKGGNIILPLALIDKCIGNRIYVVMKGDKEFSGVLRGFDEYVNMV
LDDVQEYGFKADEEDISGGNKKLKRVMVNRLETILLSGNNVAMLVPGGDPDSFNFS

>3POVA

GAMEATPTPADLFSEDYLVDTLDGLTVDDQQAVLASLSFSKFLKHAKVRDWCAQAKIQPSMPALR
MAYNYFLFSKVGEFIGSEDVCNFFVDRVFGGVRLLDVASVYAACSQMNAHQRHHICCLVERATSS
QSLNPVWDALRDGIISSSKFHWAVKQQNTSKKIFSPWPITNNHFVAGPLAFGLRCEEVVKTLLAT
LLHPDETNCLDYGFMQSPQNGIFGVSLDFAANVKTDTEGRLQFDPNCKVYEIKCRFKYTFAKMEC
DPIYAAYQRLYEAPGKLALKDFFYSISKPAVEYVGLGKLPSESDYLVAYDQEWEACPRKKRKLTP
LHNLIRECILHNSTTESDVYVLTDPQDTRGQISIKARFKANLFVNVRHSYFYQVLLQSSIVEEYI
GLDSGIPRLGSPKYYIATGFFRKRGYQDPVNCTIGGDALDPHVEIPTLLIVTPVYFPRGAKHRLL

HQAANFWSRSAKDTFPYIKWDFSYLSANVPHSP
>3PVPB
GPHMISAATIMAATAEYFDTTVEELRGPGKTRALAQSRQIAMYLCRELTDLSLPKIGQAFGRDHT
TVMYAQRKILSEMAERREVFDHVKELTTRIRQRSKR
>3Q8DB
MEGWQRAFVLHSRPWSETSLMLDVFTEESGRVRLVAKGARSKRSTLKGALQPFTPLLLRFGGRGE
VKTLRSAEAVSLALPLSGITLYSGLYINELLSRVLEYETRFSELFFDYLHCIQSLAGVTGTPEPA
LRRFELALLGHLGYGVNFTHCAGSGEPVDDTMTYRYREEKGFIASVVIDNKTFTGRQLKALNARE
FPDADTLRAAKRFTRMALKPYLGGKPLKSRELFRQFMPKRTVKTHYE
>3QMBA
MHHHHHHSSRENLYFQGQIKRSARMCGECEACRRTEDCGHCDFCRDMKKFGGPNKIRQKCRLRQC
QLRARESYKYFPSS
>3QO2A
HMGEDVFEVEKILDMKTEGGKVLYKVRWKGYTSDDDTWEPEIHLEDCKEVLLEFRKKIAENKAK
>3QODB
SNAMSNDVDLIKRLGPSAMDQIMLYLAFSAMRTSGHRHGAFLDAAATAAKCAIYMTYLEQGQNLR
MTGHLHHLEPKRVKAIVEEVRQALTEGKLLKMLGSQEPRYLIQFPYVWMEKYPWRPGRSRIPGTS
LTSEEKRQIEQKLPSNLPDAHLITSFEFLELIEFLHKRSQEDLPKEHQMPLSEALAEHIKRRLLY
SGTVTRIDSPWGMPFYALTRPFYAPADDQERTYIMVEDTARFFRMMRDWAEKRPNTMRVLEELDI
LPEKMQQAKDELDEIIRAWADKYHQDDGVPVVLQMVFGKKED
>3QRFN
SSVPLEWPLSSQSGSYELRIEVQPKPHHRAHYETEGSRGAVKAPTGGHPVVQLHGYMENKPLGLQ
IFIGTADERILKPHAFYQVHRITGKTVTTTSYEKIVGNTKVLEIPLEPKNNMRATIDCAGILKLR
NADIELRKGETDIGRKNTRVRLVFRVHIPESSGRIVSLQTASNPIECSQRSAHELPMVERQDTDS
CLVYGGQQMILTGQNFTSESKVVFTEKTTDGQQIWEMEATVAAAAAAPNMLFVEIPEYRNKHIRT
PVKVNFYVINGKRKRSQPQHFTYHPV
>3QU3A
GSHMAEVRGVQRVLFGDWLLGEVSSGQYEGLQWLNEARTVFRVPWKHFGRRDLDEEDAQIFKAWA
VARGRWPPSGVNLPPPEAEAAERRERRGWKTNFRCALHSTGRFILRQDNSGDPVDPHKVYELSRE
LGSTVGP
>3QVGD
MQEPPDLPVPELPDFFEGKHFFLYGEFPGDERRRLIRYVTAFNGELEDRMNERVQFVITAQEWDP
NFEEALMENPSLAFVRPRWIYSCNEKQKLLPHQLYGVVPQA
>3QVGC
SNSADETLCQTKVLLDIFTGVRLYLPPSTPDFSRLRRYFVAFDGDLVQEFDMTSATHVLGSRDKN
PAAQQVSPEWIWACIRKRRLVAPS
>3R0JB
GTHMRKGVDLVTAGTPGENTTPEARVLVVDDEANIVELLSVSLKFQGFEVYTATNGAQALDRARE
TRPDAVILDVMMPGMDGFGVLRRLRADGIDAPALFLTARDSLQDKIAGLTLGGDDYVTKPFSLEE
VVARLRVILRRAGKGNKEPRNVRLTFADIELDEETHEVWKAGQPVSLSPTEFTLLRYFVINAGTV
LSKPKILDHVWRYDFGGDVNVVESYVSYLRRKIDTGEKRLLHTLRGVGYVLREPR
>3R4KA
GMGTVSKALTLLTYFNHGRLEIGLSDLTRLSGMNKATVYRLMSELQEAGFVEQVEGARSYRLGPQ
VLRLAALREASVPILSASRRVLRELSEDTGETTHLSLLQGEQLASLSHAYSSRNATKVMMEDAEV

LTFHGTASGLAVLAYSEPSFVDAVLAAPLTARTPQTQTDPAAIRAEIAEVRRTGLAQSIGGFEAE
VHSHAVPIFGPDRAVLGALAVAAPTSRMTPDQKRTIPPALRAAGLSLTERIGGACPPEFPTDIAA
>3RCOA
ENLYFQGMLEGDLVSKMLRAVLQSHKNGVALPRLQGEYRSLTGDWIPFKQLGFPTLEAYLRSVPA
VVRIETSRSGEITCYAMACTETAR
>3RH2A
GMKTRDKIIQASLELFNEHGERTITTNHIAAHLDISPGNLYYHFRNKEDIIRCIFDQYEQHLLLG
FKPYADQKVDLELLMSYFDAMFYTMWQFRFMYANLADILARDDTLKARYLKVQQAVLEQSIAVLN
QLKKDGILQIEDERIADLADTIKMIIGFWISYKLTQSSIATISKASLYEGLLRVLMIFKAYSTPD
SLANFDRLEQHFRSQSN
>3RLOA
GSVDDSAQSDLKEVMVLNATESFVYEPKEQKKMFHATVATENEVFRVKVFNIDLKEKFTPKKIIA
IANYVCRNGFLEVYPFTLVADVNADRNMEIPKGLIRSASVTPKINQLCSQTKGSFVNGVFEVHKK
NVRGEFTYYEIQDNTGKMEVVVHGRLTTINCEEGDKLKLTCFELAPKSGNTGELRSVIHSHIKVI
KTRKNAAAS
>3S4WB
SHNSHEVEENGSVFVKLLKASGLTLKTGENQNQLGVDQVIFQRKLFQALRKHPAYPKVIEEFVNG
LESYTEDSESLRNCLLSCERLQDEEASMGTFYSKSLIKLLLGIDILQPAIIKMLFEKVPQFLFES
ENRDGINMARLIINQLKWLDRIVDGKDLTAQMMQLISVAPVNLQHDFITSLPEILGDSQHANVGK
ELGELLVQNTSLTVPILDVFSSLRLDPNFLSKIRQLVMGKLSSVRLEDFPVIVKFLLHSVTDTTS
LEVIAELRENLNVQQFILPSRIQASQSKLKSKGLASSSGNQENSDKDCIVLVFDVIKSAIRYEKT
ISEAWFKAIERIESAAEHKSLDVVMLLIIYSTSTQTKKGVEKLLRNKIQSDCIQEQLLDSAFSTH
YLVLKDICPSILLLAQTLFHSQDQRIILFGSLLYKYAFKFFDTYCQQEVVGALVTHVCSGTEAEV
DTALDVLLELIVLNASAMRLNAAFVKGILDYLENMSPQQIRKIFCILSTLAFSQQPGTSNHIQDD
MHLVIRKQLSSTVFKYKLIGIIGAVTMAGIMAEDRSVPSNSSQRSANVSSEQRTQVTSLLQLVHS
CTEHSPWASSLYYDEFANLIQERKLAPKTLEWVGQTIFNDFQDAFVVDFCAAPEGDFPFPVKALY
GLEEYSTQDGIVINLLPLFYQECAKDASRATSQESSQRSMSSLCLASHFRLLRLCVARQHDGNLD
EIDGLLDCPLFLPDLEPGEKLESMSAKDRSLMCSLTFLTFNWFREVVNAFCQQTSPEMKGKVLSR
LKDLVELQGILEKYLAVIPDYVPPFASVDLDTLDMMPRKTFVSLQNYRAFFRELDIEVFSILHSG
LVTKFILDTEMHTEATEVVQLGPAELLFLLEDLSQKLENMLTAPFAKRICCFKNKGRQNIGFSHL
HQRSVQDIVHCVVQLLTPMCNHLENIHNFFQCLGAEHLSADDKARATAQEQHTMACCYQKLLQVL
HALFAWKGFTHQSKHRLLHSALEVLSNRLKQMEQDQPLEELVSQSFSYLQNFHHSVPSFQCGLYL
LRLLMALLEKSAVPNQKKEKLASLAKQLLCRAWPHGEKEKNPTFNDHLHDVLYIYLEHTDNVLKA
IEEITGVGVPELVSAPKDAASSTFPTLTRHTFVIFFRVMMAELEKTVKGLQAGTAADSQQVHEEK
LLYWNMAVRDFSILLNLMKVFDSYPVLHVCLKYGRRFVEAFLKQCMPLLDFSFRKHREDVLSLLQ
TLQLNTRLLHHLCGHSKIRQDTRLTKHVPLLKKSLELLVCRVKAMLVLNNCREAFWLGTLKNRDL
QGEEIISQDPSSSESNAEDSEDG
>3S4WA
MDLKILSLATDKTTDKLQEFLQTLKDDDLASLLQNQAVKGRAVGTLLRAVLKGSPCSEEDGALRR
YKIYSCCIQLVESGDLQQDVASEIIGLLMLEVHHFPGPLLVDLASDFVGAVREDRLVNGKSLELL
PIILTALATKKEVLACGKGDLNGEEYKRQLIDTLCSVRWPQRYMIQLTSVFKDVCLTPEEMNLVV
AKVLTMFSKLNLQEIPPLVYQLLVLSSKGSRRSVLDGIIAFFRELDKQHREEQSSDELSELITAP
ADELYHVEGTVILHIVFAIKLDCELGRELLKHLKAGQQGDPSKCLCPFSIALLLSLTRIQRFEEQ
VFDLLKTSVVKSFKDLQLLQGSKFLQTLVPQRTCVSTMILEVVRNSVHSWDHVTQGLIEFGFILM

DSYGPKKILDGKAVEIGTSLSKMTNQHACKLGANILLETFKIHEMIRQEILEQVLNRVVTRTSSP
INHFLDLFSDIIMYAPLILQNCSKVTETFDYLTFLPLQTVQGLLKAVQPLLKISMSMRDSLILVL
RKAMFASQLDARKSAVAGFLLLLKNFKVLGSLPSSQCTQSIGVTQVRVDVHSRYSAVANETFCLE
IIDSLKRSLGQQADIRLMLYDGFYDVLRRNSQLASSIMQTLFSQLKQFYEPEPDLLPPLKLGACV
LTQGSQIFLQEPLDHLLSCIQHCLAWYKSRVVPLQQGDEGEEEEEELYSELDDMLESITVRMIKS
ELEDFELDKSADFSQNTNVGIKNNICACLIMGVCEVLMEYNFSISNFSKSKFEEILSLFTCYKKF
SDILSEKAGKGKAKMTSKVSDSLLSLKFVSDLLTALFRDSIQSHEESLSVLRSSGEFMHYAVNVT
LQKIQQLIRTGHVSGPDGQNPDKIFQNLCDITRVLLWRYTSIPTSVEESGKKEKGKSISLLCLEG
LQKTFSVVLQFYQPKVQQFLQALDVMGTEEEEAGVTVTQRASFQIRQFQRSLLNLLSSEEDDFNS
KEALLLIAVLSTLSRLLEPTSPQFVQMLSWTSKICKEYSQEDASFCKSLMNLFFSLHVLYKSPVT
LLRDLSQDIHGQLGDIDQDVEIEKTDHFAVVNLRTAAPTVCLLVLSQAEKVLEEVDWLIAKIKGS
ANQETLSDKVTPEDASSQAVPPTLLIEKAIVMQLGTLVTFFHELVQTALPSGSCVDTLLKGLSKI
YSTLTAFVKYYLQVCQSSRGIPNTVEKLVKLSGSHLTPVCYSFISYVQNKSSDAPKCSEKEKAAV
STTMAKVLRETKPIPNLVFAIEQYEKFLIQLSKKSKVNLMQHMKLSTSRDFKIKGSVLDMVLRED
EEHHHHHH

>3S5RB

GMQATMTDKNTRELLLDAATTLFAEQGIAATTMAEIAASVGVNPAMIHYYFKTRDSLLDTIIEER
IGRIIDMIWEPVTGEEDDPLIMVRDLVNRIVNTCETMLWLPSLWIREIVNEGGALREKMLNNIPI
DKMNKFSAKIAEGQKQGVINSGIDSRLLIGSIIGLTMLPLATAKLRDQIPTMKGLSSEDIVCHVT
ALLFTGLTNPSNSDDIKKQRT

>3SIAA

MQPPVANFCLWNLQPIQGSWMGAACIYQMPPSVRNTWWFPLLNTIPLDQYTRIYQWFMGVDRDRS
GTLEINELMMGQFPGGIRLSPQTALRMMRIFDTDFNGHISFYEFMAMYKFMELAYNLFVMNARAR
SGTLEPHEILPALQQLGFYINQRTSLLLHRLFARGMAFCDLNCWIAICAFAAQTRSAYQMIFMNP
YYGPMKPFNPMEFGKFLDVVTSLLE

>3SQIA

MSKLDSLLKELPTRTAHLYRSIWHKYTEWLKTMPDLTGADLKLFLSQKYIVKYIASHDDIAKDPL
PTCDAMIWFSRALDIENNDVLVLQQRLYGLVKLLEFDYSNVIAILQKISINLWNPSTDSLQSKHF
KTCQDKLKLLLDFQWKFNTNVSFEDRTTVSLKDLQCILDDENGKCGLAHSSKPNFVLVPNFQSPF
TCPIFTMAVYYYLRFHGVKKYYKGDGYQILSQLEHIPIIRGKSLDQYPRELTLGNWYPTIFKYCQ
LPYTKKHWFQVNQEWPQFPDFSDSSENTSTLAESDSENTIGIPDFYIEKMNRTKLQPCPQVHVHL
FPTDLPPDIQAVFDLLNSVLVTSLPLLYRVFPTHDIFLDPSLKTPQNIAFLTGTLPLDIESQEHL
LAQLIDKTGTVSELVPNPVKIDQNEHTLTPIGTSLSQTDIPMLDQLKTELQKLIQLQTSTGFSQL
ITVLLEIFQRLDFKKSNKQFVIDLLQSCRKDMRNKLMDPCSLSTNFADELSDDENEKGNKTGAIY
DPETDNGNEESVSD

>3SSCB

MESIQPWIEKFIKQAQQRSQSTKDYPTSYRNLRVKLSFGYGNFTSIPWFAFLGEGQEASNGIYP
VILYYKDFDELVLAYGISDTNEPHAQWQFSSDIPKTIAEYFQATSGVYPKKYGQSYYACSQKVSQ
GIDYTRFASMLDNIINDYKLIFNSGKSVIPPLEGHHHHHH

>3TB6B

GIDPFTSAKSALHSNKTIGVLTTYISDYIFPSIIRGIESYLSEQGYSMLLTSTNNNPDNERRGLE
NLLSQHIDGLIVEPTKSALQTPNIGYYLNLEKNGIPFAMINASYAELAAPSFTLDDVKGGMMAAE
HLLSLGHTHMMGIFKADDTQGVKRMNGFIQAHRERELFPSPDMIVTFTTEEKESKLLEKVKATLE
KNSKHMPTAILCYNDEIALKVIDMLREMDLKVPEDMSIVGYDDSHFAQISEVKLTSVKHPKSVLG

KAAAKYVIDCLEHKKPKQEDVIFEPELIIRQSARKLNE

>3TEDA

GPDMDSIGESEVRALYKAILKFGNLKEILDELIADGTLPVKSFEKYGETYDEMMEAAKDCVHEEE
KNRKEILEKLEKHATAYRAKLKSGEIKAENQPKDNPLTRLSLKKREKKAVLFNFKGVKSLNAESL
LSRVEDLKYLKNLINSNYKDDPLKFSLGNNTPKPVQNWSSNWTKEEDEKLLIGVFKYGYGSWTQI
RDDPFLGITDKIFLNEVHNPVAKKSASSSDTTPTPSKKGKGITGSSKKVPGAIHLGRRVDYLLSF
LRGGLNTKSPS

>3TEKB

MGEELREEERGEVRSELITKGEKKLVLIRWNTGKTSAGRLFGRYGPGGRPEFFKLLFGAVAGSLR
EQFGPDGENIFNRIRDSEKFRETSRELFDGLKKWFFEEAVPRYNLERGDIFMISTELVLDPDTGE
LLWNRDKTQLIYWIRSDR

>3THOB

MKILHTSDWHLGVTSWTSSRPVDRREELKKALDKVVEEAEKREVDLILLTGDLLHSRNNPSVVAL
HDLLDYLKRMMRTAPVVVLPGNQDWKGLKLFGNFVTSISSDITFVMSFEPVDVEAKRGQKVRILP
FPYPDESEALRKNEGDFRFFLESRLNKLYEEALKKEDFAIFMGHFTVEGLAGYAGIEQGREIIIN
RALIPSVVDYAALGHIHSFREIQKQPLTIYPGSLIRIDFGEEADEKGAVFVELKRGEPPRYERID
ASPLPLKTLYYKKIDTSALKSIRDSCRNFPGYVRVVYEEDSGILPDLMGEIDNLVKIERKSRREI
EEVLRESPEEFKEELDKLDYFELFKEYLKKREENHEKLLKILDELLDEVKKSEA

>3THOA

HHHHHHSSGENLYFQGHMRPERLTVRNFLGLKNVDIEFQSGITVVEGPNGAGKSSLFEAISFALF
GNGIRYPNSYDYVNRNAVDGTARLVFQFERGGKRYEIIREINALQRKHNAKLSEILENGKKAAIA
AKPTSVKQEVEKILGIEHRTFIRTVFLPQGEIDKLLISPPSEITEIISDVFQSKETLEKLEKLLK
EKMKKLENEISSGGAGGAGGSLEKKLKEMSDEYNNLDLLRKYLFDKSNFSRYFTGRVLEAVLKRT
KAYLDILTNGRFDIDFDDEKGGFIIKDWGIERPARGLSGGERALISISLAMSLAEVASGRLDAFF
IDEGFSSLCTENKEKIASVLKELERLNKVIVFITCDREFSEAFDRKLRITGGVVVNE

>3THWD

CCTCTATCTGAAGCCGATCGATGAAGCATCGATCGCACAGCTTCAGATAGAGG

>3TOCB

GSFTMNLNFSLLDEPIPLRGGTILVLEDVCVFSKIVQYCYQYEEDSELKFFDHKMKTIKESEIML
VTDILGFDVNSSTILKLIHADLESQFNEKPEVKSMIDKLVATITELIVFECLENELDLEYDEITI
LELIKSLGVKVETQSDTIFEKCLEILQIFKYLTKKKLLIFVNSGAFLTKDEVASLQEYISLTNLT
VLFLEPRELYDFPQYILDEDYFLITKNMV

>3TRBB

SNAMAANRMRPIHPGEILAEELGFLDKMSANQLAKHLAIPTNRVTAILNGARSITADTALRLAKF
FGTTPEFWLNLQDAYDIKMALKKSGKKIEKEVTPYDQAA

>3TUOD

GPGSGTMLPVFCVVEHYENAIEYDCKEEHAEFVLVRKDMLFNQLIEMALLSLGYSHSSAAQAKGL
IQVGKWNPVPLSYVTDAPDATVADMLQDVYHVVTLKIQLH

>3U50C

QRIYSSIEEIIQQAQASEIGQKKEFYVYGNLVSIQMKNKLYYYRCTCQGKSVLKYHGDSFFCESC
QQFINPQVHLMLRAFVQDSTGTIPVMIFDQQSSQLINQIDPSIHVQEAGQYVKNCIENGQEEIIR
QLFSKLDFARFIFEIQFENKEFNNEQEIAYKVLKIEKENIKE

>3U58A

GSLSDQLSKQTLLISQLQVGKNRFSFKFEGRVVYKSSTFQNQQDSKYFFITAQDANNQEINLSFW

QKVDQSYQTLKVGQYYYFIGGEVKQFKNNLELKFKFGDYQIIPKETLGGSGGSTLLISEVLKTSK
QYLSVLAQVVDIQSSDKNIRLKICDNSCNQELKVVIFPDLCYEWRDKFSINKWYYFNEFVRQIYN
DEVQLKNNIHSSIKESDD
>3U5ZF
MKLSKDTTALLKNFATINSGIMLKSGQFIMTRAVNGTTYAEANISDVIDFDVAIYDLNGFLGILS
LVNDDAEISQSEDGNIKIADARSTIFWPAADPSTVVAPNKPIPFPVASAVTEIKAEDLQQLLRVS
RGLQIDTIAITVKEGKIVINGFNKVEDSALTRVKYSLTLGDYDGENTFNFIINMANMKMQPGNYK
LLLWAKGKQGAAKFEGEHANYVVALEADSTHDF
>3U5ZA
SLFKDDIQLNEHQVAWYSKDWTAVQSAADSFKEKAENEFFEIIGAINNKTKCSIAQKDYSKFMVE
NALSQFPECMPAVYAMNLIGSGLSDEAHFNYLMAAVPRGKRYGKWAKLVEDSTEVLIIKLLAKRY
QVNTNDAINYKSILTKNGKLPLVLKELKGLVTDDFLKEVTKNVKEQKQLKKLALEWGLEHHHHHH
HHHH
>3U5ZM
GPGGSMITVNEKEHILEQKYRPSTIDECILPAFDKETFKSITSKGKIPHIILHSPSPGTGKTTVA
KALCHDVNADMMFVNGSDCKIDFVRGPLTNFASAASFDGRQKVIVIDEFDRSGLAESQRHLRSFM
EAYSSNCSIIITANNIDGIIKPLQSRCRVITFGQPTDEDKIEMMKQMIRRLTEICKHEGIAIADM
KVVAALVKKNFPDFRKTIGELDSYSSKGVLDAGILSLVTNDRGAIDDVLESLKNKDVKQLRALAP
KYAADYSWFVGKLAEEIYSRVTPQSIIRMYEIVGENNQYHGIAANTELHLAYLFIQLACEMQWK
>3ULJA
GSDPQVLRGSGHCKWFNVRMGFGFISMTSREGSPLENPVDVFVHQSKLYMEGFRSLKEGEPVEFT
FKKSSKGFESLRVTGPGGNPCLGNE
>3ULXA
MGMRRERDAEAELNLPPGFRFHPTDDELVEHYLCRKAAGQRLPVPIIAEVDLYKFDPWDLPERAL
FGAREWYFFTPRDRKYPNGSRPNRAAGNGYWKATGADKPVAPRGRTLGIKKALVFYAGKAPRGVK
TDWIMHEYRLADAGRAAAGAKKGSLRLDDWVLCRLYNKKNEWEK
>3UWXB
MGSSHHHHHHSSGLVPRGSHMGPKKVEGRFQLVSPYEPQGDQPQAIAKLVDGLRLGVKHQTLLGA
TGTGKTFTISNVIAQVNKPTLVIAHNKTLAGQLHSELKEFFPHNAVEYFVSYYDYYQPEAYVPQT
DTYIEKDAKINDEIDKLRHSATSALFERRDVIIVASVSCIYGLGSPEEYRELVVSLRVGMEIERN
ALLRRLVDIQYDRNDIDFRRGTFRVRGDVVEIFPASRDEHCIRVEFFGDEIERIREVDALTGEVL
GEREHVAIFPASHFVTREEKMRLAIQNIEQELEERLAELRAQGKLLEAQRLEQRTRYDLEMMREM
GFCSGIENYSRHLALRPPGSTPYTLLDYFPDDFLIIVDESHVTLPQLRGMYNGDRARKQVLVDHG
FRLPSALDNRPLTFEEFEQKINQIIYISATPGPYELEHSPGVVEQIIRPTGLLDPTIDVRPTKGQ
IDDLIGEIHERVERNERTLVTTLTKKMAEDLTDYLKEAGIKVAYLHSEIKTLERIEIIRDLRLGK
YDVLVGINLLREGLDIPEVSLVAILDADKEGFLRSERSLIQTIGRAARNANGHVIMYADTITKSM
EIAIQETKRRRAIQEEYNRKHGIVPRTVKKEIRDVIRATYAAEETEMYEAKPAAAMTKQEREELI
RKLEAEMKEAAKALDFERAAQLRDIIFELKAEG
>3V20B
MTTNLTNSNCVEEYKENGKTKIRIKPFNALIELYHHQTPTGSIKENLDKLENYVKDVVKAKGLAI
PTSGAFSNTRGTWFEVMIAIQSWNYRVKRELNDYLIIKMPNVKTFDFRKIFDNETREKLHQLEKS
LLTHKQQVRLITSNPDLLIIRQKDLIKSEYNLPINKLTHENIDVALTLFKDIEGKCKWDSLVAGV
GLKTSLRPDRRLQLVHEGNILKSLFAHLKMAYWNPKAEFKYYGASSEPVSKADDDALQTAATHTI
VNVNSTPERAVDDIFSLTSFEDIDKMLDQIIKK

>3V4GA

MHHHHHHSSGVDLGTENLYFQSNAMRPSEKQDNLVRAFKALLKEERFGSQGEIVEALKQEGFENI
NQSKVSRMLTKFGAVRTRNAKMEMVYCLPTELGVPTVSSSLRELVLDVDHNQALVVIHTGPGAAQ
LIARMLDSLGKSEGILGVVAGDDTIFITPTLTITTEQLFKSVCELFEYAG

>3V60A

GSHMRPETHINLKVSDGSSEIFFKIKKTTPLRRLMEAFAKRQGKEMDSLRFLYDGIRIQADQTPE
DLDMEDNDIIEAHREQIGG

>3V62C

SHNPDDTTVDNRPIISNAKFLADAAMKKTQKFSKKVKNEPASSQMDIFSQLSRAKKKSKLNNGEI
IVID

>3V68A

MIERILEFTAKHEEWIVGENVEDFTNENIAMFLSRVSNTVSSKIPGYLGEKIDVNGLLSIKIEGS
LEEKLKALISPKVSRQIGRLVMEDDKKLKKLLVEVAKAVLTREILKNELPIEFPGGKIEGLKIQP
RYEEDHINFTARYGSWIVVKRMIIDEKTPLLDIARLLASINETAVNKIKDFADVDDKKIVEYFGG
FKKVKKEEEIKEIVQLFREFKGNEFEVRYAAREMLSKLGLKVDVPSKNLEKYLEKAG

>3V72A

MSKRKAPQETLNGGITDMLVELANFEKNVSQAIHKYNAYRKAASVIAKYPHKIKSGAEAKKLPGV
GTKIAEKIDEFLATGKLRKLEKIRQDDTSSSINFLTRVTGIGPSAARKLVDEGIKTLEDLRKNED
KLNHHQRIGLKYFEDFEKRIPREEMLQMQDIVLNEVKKLDPEYIATVCGSFRRGAESSGDMDVLL
THPNFTSESSKQPKLLHRVVEQLQKVRFITDTLSKGETKFMGVCQLPSENDENEYPHRRIDIRLI
PKDQYYCGVLYFTGSDIFNKNMRAHALEKGFTINKYTIRPLGVTGVAGEPLPVDSEQDIFDYIQW
RYREPKDRSE

>3V9RD

MLSKEALIKILSQNEGGNDMKIADEVVPMIQKYLDIFIDEAVLRSLQSHKDINGERGDKSPLELS
HQDLERIVGLLLMDMLEHHHHHH

>3V9RC

MNDDEDRAQLKARLWIRVEERLQQVLSSEDIKYTPRFINSLLELAYLQLGEMGSDLQAFARHAGR
GVVNKSDLMLYLRKQPDLQERVTQE

>3VEAA

MKYQQLENLESGWKWAYLVKKHREGEAITRHIENSAAQDAVEQLMKLENEPVKVQEWIDAHMNVN
LATRMKQTIRARRKRHFNAEHQHTRKKSIDLEFLVWQRLAVLARRRGNTLSDTVVQLIEDAERKE
KYASQMSSLKQDLKDILDKEV

>3VH5W

GYRRTVPRGTLRKIIKKHKPHLRLAANTDLLVHLSFLLFLHRLAEEARTNAFENKSKIIKPEHTI
AAAKVILKKSRG

>3VH5A

GSEAAGGEQRELLIQRLRAAVHYTTGALAQDVAEDKGVLFSKQTVAAISEITFRQAENFARDLEM
FARHAKRSTITSEDVKLLARRSNSLLKYITQKSDELASSNMEQKEKKKKKSSAAKGRKTEENETP
VTESEDSNMA

>3VIBA

MRKTKTEALKTKEHLMLAALETFYRKGIARTSLNEIAQAAGVTRDALYWHFKNKEDLFDALFQRI
CDDIENCIAQDAADAEGGSWTVFRHTLLHFFERLQSNDIHYKFHNILFLKCEHTEQNAAVIAIAR
KHQAIWREKITAVLTEAVENQDLADDLDKETAVIFIKSTLDGLIWRWFSSGESFDLGKTAPRIIG
IMMDNLENHPCLRRK

>3VK0C
MMGNKLTLPAELPDEQDLRAVLAYNMRLFRVNKGWSQEELARQCGLDRTYVSAVERKRWNIALSN
IEKMAAALGVAAYQLLLPPQERLKLMTNSADTRQMPSESGILEHHHHHH
>3VPRD
VTTTRDRILEEAAKLFTEKGYEATSVQDLAQALGLSKAALYHHFGSKEEILYEISLLALKGLVAA
GEKALEVADPKEALRRFMEAHARYFEENYPFFVTMLQGIKSLSPENRLKTIALRDRHEENLRAIL
RRGVEQGVFREVDVALAGRAVLSMLNWMIRWFRPDGPMRAEEVARAYHDLILRGLERGSA
>3VW4A
MGHHHHHHRNYHLFEKVRKWAYRAIRQGWPVFSQWLDAVIQRVEMYNASLPVPLSPAECRAIGKS
IAKYTHRKFSPEGFSAVQAARGRKGGTKSKRAAVPTSARSLKPWEALGISRATYYRKLKCDPD
>3VZHA
GHMYRSRDFYVRVSGQRALFTNPATKGGSERSSYSVPTRQALNGIVDAIYYKPTFTNIVTEVKVI
NQIQTELQGVRALLHDYSADLSYVSYLSDVVYLIKFHFVWNEDRKDLNSDRLPAKHEAIMERSIR
KGGRRDVFLGTRECLGLVDDISQEEYETTVSYYNGVNIDLGIMFHSFAYPKDKKTPLKSYFTKTV
MKNGVITFKAQSECDIVNTLSSYAFKAPEEIKSVNDECMEYDAMEKGEN
>3W03B
SGMEELEQGLLMQPWAWLQLAENSLLAKVFITKQGYALLVSDLQQVWHEQVDTSVVSQRAKELNK
RLTAPPAAFLCHLDNLLRPLLKDAAHPSEATFSCDCVADALILRVRSELSGLPFYWNFHCMLASP
SLVSQHLIRPLMGMSLALQCQVRELATLLHMKDLEIQDYQESGATLIRDRLKTEPFEENSFLEQF
MIEKLPEACSIGDGKPFVMNLQDLYMAVTTQEVQVGQKHQ
>3W3WB
PRRRTVGMKSSQGNVPTGNKQSVGKSAKISKPLHIKTSAYQKQYKINLETKARPSAGDEDSAHPD
KNKE
>3W3WA
MSALPEEVNRTLLQIVQAFASPDNQIRSVAEKALSEEWITENNIEYLLTFLAEQAAFSQDTTVAA
LSAVLFRKLALKAPITHIRKEVLAQIRSSLLKGFLSERADSIRHKLSDAIAECVQDDLPAWPELL
QALIESLKSGNPNFRESSFRILTTVPYLITAVDINSILPIFQSGFTDASDNVKIAAVTAFVGYFK
QLPKSEWSKLGILLPSLLNSLPRFLDDGKDDALASVFESLIELVELAPKLFKDMFDQIIQFTDMV
IKNKDLEPPARTTALELLTVFSENAPQMCKSNQNYGQTLVMVTLIMMTEVSIDDDDAAEWIESDD
TDDEEEVTYDHARQALDRVALKLGGEYLAAPLFQYLQQMITSTEWRERFAAMMALSSAAEGCADV
LIGEIPKILDMVIPLINDPHPRVQYGCCNVLGQISTDFSPFIQRTAHDRILPALISKLTSECTSR
VQTHAAAALVNFSEFASKDILEPYLDSLLTNLLVLLQSNKLYVQEQALTTIAFIAEAAKNKFIKY
YDTLMPLLLNVLKVNNKDNSVLKGKCMECATLIGFAVGKEKFHEHSQELISILVALQNSDIDEDD
ALRSYLEQSWSRICRILGDDFVPLLPIVIPPLLITAKATQDVGLIEEEEAANFQQYPDWDVVQVQ
GKHIAIHTSVLDDKVSAMELLQSYATLLRGQFAVYVKEVMEEIALPSLDFYLHDGVRAAGATLIP
ILLSCLLAATGTQNEELVLLWHKASSKLIGGLMSEPMPEITQVYHNSLVNGIKVMGDNCLSEDQL
AAFTKGVSANLTDTYERMQDRHGDGDEYNENIDEEEDFTDEDLLDEINKSIAAVLKTTNGHYLKN
LENIWPMINTFLLDNEPILVIFALVVIGDLIQYGGEQTASMKNAFIPKVTECLISPDARIRQAAS
YIIGVCAQYAPSTYADVCIPTLDTLVQIVDFPGSKLEENRSSTENASAAIAKILYAYNSNIPNVD
TYTANWFKTLPTITDKEAASFNYQFLSQLIENNSPIVCAQSNISAVVDSVIQALNERSLTEREGQ
TVISSVKKLLGFLPSSDAMAIFNRYPADIMEKVHKWFA
>3WE2B
GPLGSKTKDYKTRDVTDDVKSIVRFVQEHSSSQGMRNIKHVGPSGRFTMNMLVDIFLGSKSAKIQ
SGIFGKGSAYSRHNAERLFKKLILDKILDEDLYINANDQAIAYVMLGNKAQTVLNGNLKVDFMET

ENSSSVKKQKALVAKVS
>3ZLJD
PNAAATQVDGTQMSLLSVPEETSPAVEALENLDPRSLTPRQALEWIYRLKSLV
>3ZQJA
MGHHHHHHHHHHSSGHIEGRHMADRLIVKGAREHNLRSVDLDLPRDALIVFTGLSGSGKSSLAFD
TIFAEGQRRYVESLSAYARQFLGQMDKPDVDFIEGLSPAVSIDQKSTNRNPRSTVGTITEVYDYL
RLLYARAGTPHCPTCGERVARQTPQQIVDQVLAMPEGTRFLVLAPVVRTRKGEFADLFDKLNAQG
YSRVRVDGVVHPLTDPPKLKKQEKHDIEVVVDRLTVKAAAKRRLTDSVETALNLADGIVVLEFVD
HELGAPHREQRFSEKLACPNGHALAVDDLEPRSFSFNSPYGACPECSGLGIRKEVDPELVVPDPD
RTLAQGAVAPWSNGHTAEYFTRMMAGLGEALGFDVDTPWRKLPAKARKAILEGADEQVHVRYRNR
YGRTRSYYADFEGVLAFLQRKMSQTESEQMKERYEGFMRDVPCPVCAGTRLKPEILAVTLAGESK
GEHGAKSIAEVCELSIADCADFLNALTLGPREQAIAGQVLKEIRSRLGFLLDVGLEYLSLSRAAA
TLSGGEAQRIRLATQIGSGLVGVLYVLDEPSIGLHQRDNRRLIETLTRLRDLGNTLIVVEHDEDT
IEHADWIVDIGPGAGEHGGRIVHSGPYDELLRNKDSITGAYLSGRESIEIPAIRRSVDPRRQLTV
VGAREHNLRGIDVSFPLGVLTSVTGVSGSGKSTLVNDILAAVLANRLNGARQVPGRHTRVTGLDY
LDKLVRVDQSPIGRTPRSNPATYTGVFDKIRTLFAATTEAKVRGYQPGRFSFNVKGGRCEACTGD
GTIKIEMNFLPDVYVPCEVCQGARYNRETLEVHYKGKTVSEVLDMSIEEAAEFFEPIAGVHRYLR
TLVDVGLGYVRLGQPAPTLSGGEAQRVKLASELQKRSTGRTVYILDEPTTGLHFDDIRKLLNVIN
GLVDKGNTVIVIEHNLDVIKTSDWIIDLGPEGGAGGGTVVAQGTPEDVAAVPASYTGKFLAEVVG
GGASAATSRSNRRRNVSA
>4A0AA
MHHHHHHVDENLYFQGGGRMSYNYVVTAQKPTAVNGCVTGHFTSAEDLNLLIAKNTRLEIYVVTA
EGLRPVKEVGMYGKIAVMELFRPKGESKDLLFILTAKYNACILEYKQSGESIDIITRAHGNVQDR
IGRPSETGIIGIIDPECRMIGLRLYDGLFKVIPLDRDNKELKAFNIRLEELHVIDVKFLYGCQAP
TICFVYQDPQGRHVKTYEVSLREKEFNKGPWKQENVEAEASMVIAVPKPFGGAIIIGQESITYHN
GDKYLAIAPPIIKQSTIVCHNRVDPNGSRYLLGDMEGRLFMLLLEKEEQMDGTVTLKDLRVELLG
ETSIAECLTYLDNGVVFVGSRLGDSQLVKLNVDSNEQGSYVVAMETFTNLGPIVDMCVVDLERQG
QGQLVTCSGAFKEGSLRIIRNGIGIHEHASIDLPGIKGLWPLRSDPNRETDDTLVLSFVGQTRVL
MLNGEEVEETELMGFVDDQQTFFCGNVAHQQLIQITSASVRLVSQEPKALVSEWKEPQAKNISVA
SCNSSQVVVAVGRALYYLQIHPQELRQISHTEMEHEVACLDITPLGDSNGLSPLCAIGLWTDISA
RILKLPSFELLHKEMLGGEIIPRSILMTTFESSHYLLCALGDGALFYFGLNIETGLLSDRKKVTL
GTQPTVLRTFRSLSTTNVFACSDRPTVIYSSNHKLVFSNVNLKEVNYMCPLNSDGYPDSLALANN
STLTIGTIDEIQKLHIRTVPLYESPRKICYQEVSQCFGVLSSRIEVQDTSGGTTALRPSASTQAL
SSSVSSSKLFSSSTAPHETSFGEEVEVHNLLIIDQHTFEVLHAHQFLQNEYALSLVSCKLGKDPN
TYFIVGTAMVYPEEAEPKQGRIVVFQYSDGKLQTVAEKEVKGAVYSMVEFNGKLLASINSTVRLY
EWTTEKELRTECNHYNNIMALYLKTKGDFILVGDLMRSVLLLAYKPMEGNFEEIARDFNPNWMSA
VEILDDDNFLGAENAFNLFVCQKDSAATTDEERQHLQEVGLFHLGEFVNVFCHGSLVMQNLGETS
TPTQGSVLFGTVNGMIGLVTSLSESWYNLLLDMQNRLNKVIKSVGKIEHSFWRSFHTERKTEPAT
GFIDGDLIESFLDISRPKMQEVVANLQYDDGSGMKREATADDLIKVVEELTRIH
>4A11B
MLGFLSARQTGLEDPLRLRRAESTRRVLGLELNKDRDVERIHGGGINTLDIEPVEGRYMLSGGSD
GVIVLYDLENSSRQSYYTCKAVCSIGRDHPDVHRYSVETVQWYPHDTGMFTSSSFDKTLKVWDTN
TLQTADVFNFEETVYSHHMSPVSTKHCLVAVGTRGPKVQLCDLKSGSCSHILQGHRQEILAVSWS
PRYDYILATASADSRVKLWDVRRASGCLITLDQHNGKKSQAVESANTAHNGKVNGLCFTSDGLHL

LTVGTDNRMRLWNSSNGENTLVNYGKVCNNSKKGLKFTVSCGCSSEFVFVPYGSTIAVYTVYSGE
QITMLKGHYKTVDCCVFQSNFQELYSGSRDCNILAWVPSLYEPVPDDDETTTKSQLNPAFEDAWS
SSDEEGGTSAWSHPQFEK
>4ACOA
MRSSILFLLKLMKIMDVQQQQEAMSSEDRFQELVDSLKPRTAHQYKTYYTKYIQWCQLNQIIPTP
EDNSVNSVPYKDLPISAELIHWFLLDTLITDDKPGEKREETEDLDEEEENSFKIATLKKIIGSLN
FLSKLCKVHENPNANIDTKYLESVTKLHTHWIDSQKAITTNETNNTNTQVLCPPLLKVSLNLWNP
ETNHLSEKFFKTCSEKLRFLVDFQLRSYLNLSFEERSKIRFGSLKLGKRDRDAIIYHKVTHSAEK
KDTPGHHQLLALLPQDCPFICPQTTLAAYLYLRFYGIPSVSKGDGFPNLNADENGSLLQDIPILR
GKSLTTYPREETFSNYYTTVFRYCHLPYKRREYFNKCNLVYPTWDEDTFRTFFNEENHGNWLEQP
EAFAFPDKIPFDFKKIMNFKSPYTSYSTNAKKDPFPPPKDLLVQIFPEIDEYKRHDYEGLSQNSR
DFLDLMEVLRERFLSNLPWIYKFFPNHDIFQDPIFGNSDFQSYFNDKTIHSKGSPILSFDILPGF
NKIYKNKTNFYSLLIERPSQLTFASSHNPDTHPTQKQESEGPLQMSQLDTTQLNELLKQQSFEYV
QFQTLSNFQILLSVFNKIFEKLEMKKSSRGYILHQLNLFKITLDERIKKSKIDDADKFIRDNQPI
KKEENIVNEDGPNTSRRTKRPKQIRLLSIADSSDESSTEDSNVFKKDGESIEDGAYGENEDENDS
EMQEQLKSMINELINSKISTFLRDQMDQFELKINALLDKILEEKVTRIIEQKLGSHTGKFSTLKR
PQLYMTEEHNVGFDMEVPKKLRTSGKYAETVKDNDDHQAMSTTASPSPEQDQEAKSYTDEQEFML
DKSIDSIEGIILEWFTPNAKYANQCVHSMNKSGNKSWRANCEALYKERKSIVEFYIYLVNHESLD
RYKAVDICEKLRDQNEGSFSRLAKFLRKWRHDHQNSFDGLLVYLSN
>4AD8A
GIDPFTMTRKARTPKAAPVPEAVAVVEPPPPDAAPTGPRLSRLEIRNLATITQLELELGGGFCAF
TGETGAGKSIIVDALGLLLGGRANHDLIRSGEKELLVTGFWGDGDESEADSASRRLSSAGRGAAR
LSGEVVSVRELQEWAQGRLTIHWQHSAVSLLSPANQRGLLDRRVTKEAQAYAAAHAAWREAVSRL
ERLQASQRERARQIDLLAFQVQEISEVSPDPGEEEGLNTELSRLSNLHESSKHPTSLVPRGSGSA
ADPEALDRVEARLSALSKLKNKYGPTLEDVVEFGAQAAEELAGLEEDERDAGSLQADVDALHAEL
LKVGQALDAAREREAPLVDSLLAVIRELGMPHARMEFALSALAEPAAYGLSDVLLRFSANPGEE
LGPLSDVASGGELSRVMLAVSTVLGADTPSVVFDEVDAGIGGAAAIAVAEQLSRLADTRQVLVVT
HLAQIAARAHHHYKVEKQVEDGRTVSHVRLLTGDERLEEIARMLSGNTSEAALEHARELLAG
>4ATKB
ANIKRELTACIFPTESEARALAKERQKKDNHNLIERRRRFNINDRIKELGTLIPKSNDPDMRWNK
GTILKASVDYIRKLQREQQRAKDLENRQKKLEHANRHLLLRVQELEMQARAHG
>4BHXB
GHMTDSEFFHQRFRNLIYVEFVGPRKTLIKLRNLCLDWLQPETRTKEEIIELLVLEQYLTIIPEK
LKPWVRAKKPENCEKLVTLLENYKEMYQP
>4BJ1A
GGGRVDHVFYQKFKSMALQELGTNYLSISYVPSLSKFLSKNLRSMKNCIVFFDKVEHIHQYAGID
RAVSETLSLVDINVVIIEMNDYLMKEGIQSSKSKECIESMGQASYSGQLDFEASEKPSNHTSDLM
MMVMRKINNDESIDHIVYFKFEQLDKLSTSTIIEPSKLTEFINVLSVLEKSNNIAFKVLIYSNNV
SISSLLSTSLKKKLNTKYTVFEMPILTCAQEQEYLKKMIKFTFDSGSKLLQSYNSLVTCQLNNKE
SNLAIFFEFLKVFPHPFTYLFNAYTEIIVQSRTFDELLDKIRNRLTIKNYPHSAYNFKK
>4BJIA
GAMGKGQCRVWIITTNMGVESVPTCRHSKLGEPSKTIQEVIEALKPLFEKRPVWTRRALLNHLDP
SYTHYLKFALPYLSYLWTSGPFRDTYTRFGYDPRKDSNAAAYQALFFKLKLNGKHKGTKTHVFDG
KTLFPTNRVYQVCDIVDPTIAPLLKDTQLRSECHRDTGWYRSGRYYKVRDLMREKLFALIEGEMP

SEVAVNMILNAEEVEESDRY
>4BJXA
GSSHHHHHHSSSMNPPPPETSNPNKPKRQTNQLQYLLRVVLKTLWKHQFAWPFQQPVDAVKLNLP
DYYKIIKTPMDMGTIKKRLENNYYWNAQECIQDFNTMFTNCYIYNKPGDDIVLMAEALEKLFLQK
INELPTEEQELVVTIPNSHKKGA
>4DAPA
MEFSPPLQRATLIQRYKRFLADVITPDGRELTLHCPNTGAMTGCATPGDTVWYSTSDNTKRKYPH
TWELTQSQSGAFICVNTLWANRLTKEAILNESISELSGYSSLKSEVKYGAERSRIDFMLQADSRP
DCYIEVKSVTLAENEQGYFPDAVTERGQKHLRELMSVAAEGQRAVIFFAVLHSAITRFSPARHID
EKYAQLLSEAQQRGVEILAYKAEISAEGMALKKSLPVTL
>4DFCC
GPHMASALVMKKGQRLSRDALRTQLDSAGYRHVDQVMEHGEYATRGALLDLFPMGSELPYRLDFF
DDEIDSLRVFDVDSQRTLEEVEAINLLP
>4DG7H
MGSSHHHHHHSSGLVPRGSHMSNFVNLDIFSNYQKYIDNEQEVRENIRIVVREIEHLSKEAQIKL
QIIHSDLSQISAACGLARKQVELCAQKYQKLAELVPAGQYYRYSDHWTFITQRLIFIIALVIYLE
AGFLVTRETVAEMLGLKISQSEGFHLDVEDYLLGILQLASELSRFATNSVTMGDYERPLNISHFI
GDLNTGFRLLNLKNDGLRKRFDALKYDVKKIEEVVYDVSIRGLSSKEKDQQEEPAVPATE

>4DHXF
MVVSKMNKDAQMRAAINQKLIETGERERLKELLRAKLIECGWKDQLKAHCKEVIKEKGLEHVTVD
DLVAEITPKGRALVPDSVKKELLQRIRTFLAQHASL
>4DHXD
GSLVLSELSQGLAVELMERVMMEFVRETCSQELKNAVETDQRVRVARCCEDVCAHLVDLFLVEEI
FQTAKETLQE
>4DKYB
MNKAELIDVLTQKLGSDRRQATAAVENVVDTIVRAVHKGDSVTITGFGVFEQRRRAARVARNPRT
GETVKVKPTSVPAFRPGAQFKAVVSGAQRLPAEGPHHHHHH
>4DRAE
GSHMEGAGAGSGFRKELVSRLLHLHFKDDKTKVSGDALQLMVELLKVFVVEAAVRGVRQAQAEDA
LRVDVDQLEKVLPQLLLDF
>4DRBI
GSIFSYRDGMRQSSLKKDWFLSEEEFKLWNRLYRLRDSDEIKEITLPQVQFSSLQNEENKPAQES
TTGIHQLSLSEWRLWQDHPLPTHQVDHSDRCRHFIGLMQMIEGMRHEEGECSYELEVESYLQMED
VTSTFIAPRNE
>4DWPA
MGSSHHHHHHSSGLVPRGSHMLAAKRKTKTPVLVERIDQFVGQIKEAMKSDDASRNRKIRDLWDA
EVRYHFDNGRTEKTLELYIMKYRNALKAEFGPKSTPLAICNMKKLRERLNTYIARGDYPKTGVAT
SIVEKIERAEFNTAGRKPTVLLRIADFIAAMNGMDAKQDMQALWDAEIAIMNGRAQTTIISYITK
YRNAIREAFGDDHPMLKIATGDAAMYDEARRVKMEKIANKHGALITFENYRQVLKICEDCLKSSD
PLMIGIGLIGMTGRRPYEVFTQAEFSPAPYGKGVSKWSILFNGQAKTKQGEGTKFGITYEIPVLT
RSETVLAAYKRLRESGQGKLWHGMSIDDFSSETRLLLRDTVFNLFEDVWPKEELPKPYGLRHLYA
EVAYHNFAPPHVTKNSYFAAILGHNNNDLETSLSYMTYTLPEDRDNALARLKRTNERTLQQMATI
APVSRKG

>4E1RB
MSYYHHHHHHDYDIPTTENLYFQGAMAKKVTVTLVDDFDGSGAADETVEFGLDGVTYEIDLSTKN
ATKLRGDLKQWVAAGRRVGGR
>4E2IL
KQVSWKLVTEYAMETKCDDVLLLLGMYLEFQYSFEMCLKCIKKEQPSHYKYHEKHYANAAIFADS
KNQKTICQQAVDTVLAKKRVDSLQLTREQMLTNRFNDLLDRMDIMFGSTGSADIEEWMAGVAWLH
CLLPKMDSVVYDFLKCMVYNIPKKRYWLFKGPIDSGKTTLAAALLELCGGKALNVNLPLDRLNFE
LGVAIDQFLVVFEDVKGTGGESRDLPSGQGINNLDNLRDYLDGSVKVNLEKKHLNKRTQIFPPGI
VTMNEYSVPKTLQARFVKQIDFRPKDYLKHCLERSEFLLEKRIIQSGIALLLMLIWYRPVAEFAQ
SIQSRIVEWKERLDKEFSLSVYQKMKFNVAMGIGVLD
>4EOGA
MGMRVLVTTWGNPFQWEPITYEYRGIKVKSRNTLPILVKTLEPERILILVADTMANYYDSGKNKP
EIEEKSFSSYSEVVEDTKERILWHIKEEVIEELREEDPELAKKIENMLKDERITIEVLPGVGVFG
NITVEGEMLDFYYYATYKLAEWLPVQNNLEVYLDLTHGINFMPTFTYRALRNLLGLLAYLYNVKF
EIVNSEPYPLGVSQEIREDTILHIREIGEGVVRPRPQYSPVEGKLYWNAFISSVANGFPLVFASF
YPNIRDVEDYLNKKLEEFLVGIEVGEREDGKPYVKREKALDRSFKNASKLYYALRVFNTKFQNYP
KKEVPIEEIMEISKIFESLPRIGIILERQVEWLRNLVYGRLWYENGEQKIKKGLLEIIKDKKDKR
KEAEALKKGKTISLAEAAKLTRIFSPSGERIETIESPNVVRNFIAHSGFEYNIVYVKYDRLSDRL
YFFYKDKEKAANLAYEALLYRGEKE
>4EQ6B
MGSSHHHHHHSHGSMEVLKNIRIYPLSNFITSTKNYINLPNELRNLISEEQESKLGFLHIIESDF
KPSVALQKLVNCTTGDEKILIIDIVSIWSQQKQRQHGAIYMNSLSCINITGLIVFLELLYDSPMD
ALRRCQVDNFNFQLRGIVIDNLSFLNFESDKNYDVINLSKFEKLFKILRKLREFLGCWIITKSFP
TDFYNGIENTLVDKWSIKRKSGVTLYPTKLPDSYMKGMDLIIYREVVDGRPQYRRIAALEE
>4EQ6A
MMEYEDLELITIWPSPTKNKLCQFIKQNLSKEHVVTQLFFIDATSSFPLSQFQKLVPPTLPENVR
IYENIRINTCLDLEELSAITVKLLQILSMNKINAQRGTEDAVTEPLKIILYINGLEVMFRNSQFK
SSPQRSHELLRDTLLKLRVMGNDENENASIRTLLEFPKEQLLDYYLKKNNNTRTSSVRSKRRRIK
NGDSLAEYIWKYYADSLFE
>4ER8A
MSEYRRYYIKGGTWFFTVNLRNRRSQLLTTQYQMLRHAIIKVKRDRPFEINAWVVLPEHMHCIWT
LPEGDDDFSSRWREIKKQFTHACGLKNIWQPRFWEHAIRNTKDYRHHVDYIYINPVKHGWVKQVS
DWPFSTFHRDVARGLYPIDWAGDVTDFSAGERIIS
>4EXWE
GIDPFTMLHIEFITDLGAKVTVDVESADKLLDVQRQYGRLGWTSGEVPVGGYQFPLENEPDFDWS
LIGARKWTNPEGEEMILHRGHAYRRRELEAVDSRKMKLPAAVKYSRGAKNTDPEHVREKADGEFE
YVTLAIFRGGKRQERYAVPGSNRPQAGAPARSAATRAQGARPGAVAVQDEETPF
>4F6MA
ANKRMKVKHDDHYELIVDGRVYYICIVCKRSYVCLTSLRRHFNIHSWEKKYPCRYCEKVFPLAEY
RTKHEIHHTGERRYQCLACGKSFINYQFMSSHIKSVHSQDPSGDSKLYRLHPCRSLQIRQYAYLS
DRS
>4FB3E
MHHHHHHSDFPSSLTGYLSHAIYSNKTFPAFLVYSTKEKCKQLYDTIGKFRPEFKCLVHYEEGGM
LFFLTMTKHRVSAVKNYCSKLCSVSFLMCKAVTKPMECYQVVTAAPFQLITENKPGLHQFEFTDE

PEEQKAVDGSHHHHHH
>4FCYC
GTTTTCGCATTTATCGTGAAACGCTTTCGCGTTTTTCGTGCGCCGCTTCATCTGATGTGTTGTTG
ACG
>4FCYB
IARPTLEAHDYDREALWSKWDNASDSQRRLAEKWLPAVQAADEMLNQGISTKTAFATVAGHYQVS
ASTLRDKYYQVQKFAKPDWAAALVDGRGASRRNVHKSEFDEDAWQFLIADYLRPEKPAFRKCYER
LELAAREHGWSIPSRATAFRRIQQLDEAMVVACREGEHALMHLIPAQQRTVEHLDAMQWINGDGY
LHNVFVRWFNGDVIRPKTWFWQDVKTRKILGWRCDVSENIDSIRLSFMDVVTRYGIPEDFHITID
NTRGAANKWLTGGAPNRYRFKVKEDDPKGLFLLMGAKMHWTSVVAGKGWGQAKPVERAFGVGGLE
EYVDKHPALAGAYTGPNPQAKPDNYGDRAVDAELFLKTLAEGVAMFNARTGRETEMCGGKLSFDD
VFEREYARTIVRKPTEEQKRMLLLPAEAVNVSRKGEFTLKVGGSLKGAKNVYYNLALMLAGVKKV
VVRFDPQQLHSTVYCYTLDGRFICEAECLAPVAFNDAAAGREYRRRQKQLKSATKAAIKAQKQMD
ALEVAELLP
>4FE7A
MGSSHHHHHHSSGLVPRGSHMFTKRHRITLLFNANKAYDRQVVEGVGEYLQASQSEWDIFIEEDF
RARIDKIKDWLGDGVIADFDDKQIEQALADVDVPIVGVGGSYHLAESYPPVHYIATDNYALVESA
FLHLKEKGVNRFAFYGLPESSGKRWATEREYAFRQLVAEEKYRGVVYQGLETAPENWQHAQNRLA
DWLQTLPPQTGIIAVTDARARHILQVCEHLHIPVPEKLCVIGIDNEELTRYLSRVALSSVAQGAR
QMGYQAAKLLHRLLDKEEMPLQRILVPPVRVIERRSTDYRSLTDPAVIQAMHYIRNHACKGIKVD
QVLDAVGISRSNLEKRFKEEVGETIHAMIHAEKLEKARSLLISTTLSINEISQMCGYPSLQYFYS
VFKKAYDTTPKEYRDVNSEVML
>4FJOC
MTTLTRQDLNFGQVVADVLSEFLEVAVHLILYVREVYPVGIFQKRKKYNVPVQMSCHPELNQYIQ
DTLHCVKPLLEKNDVEKVVVVILDKEHRPVEKFVFEITQPPLLSINSDSLLSHVEQLLAAFILKI
SVCDAVLDHNPPGCTFTVLVHTREAATRNMEKIQVIKDFPWILADEQDVHMHDPRLIPLKTMTSD
ILKMQLYVEERAHKN
>4FJOA
AAPNLAGAVEFSDVKTLLKEWITTISDPMEEDILQVVRYCTDLIEEKDLEKLDLVIKYMKRLMQQ
SVESVWNMAFDFILDNVQVVLQQTYGSTLKVT
>4FW2B
PLREAKDLHTALHIGPRALSKASNISMQQAREVVQTCPHCNSAPALEAGVNPRGLGPLQIWQTDF
TLEPRMAPRSWLAVTVDTASSAIVVTQHGRVTSVAVQHHWATAIAVLGRPKAIKTDNGSCFTSKS
TREWLARWGIAHTTGIPGNSQGQAMVERANRLLKDKIRVLAEGDGFMKRIPTSKQGELLAKAMYA
LNHKERGENTKTPIQKHWRPTVLTEGPPVKIRIETGEWEKGWNVLVWGRGYAAVKNRDTDKVIWV
PSRKVKPDIT
>4G12B
MTASAPDGRPGQPEATNRRSQLKSDRRFQLLAAAERLFAERGFLAVRLEDIGAAAGVSGPAIYRH
FPNKESLLVELLVGVSARLLAGARDVTTRSANLAAALDGLIEFHLDFALGEADLIRIQDRDLAHL
PAVAERQVRKAQRQYVEVWVGVLRELNPGLAEADARLMAHAVFGLLNSTPHSMKAADSKPARTVR
ARAVLRAMTVAALSAADRCL
>4G4KB
MDNSVETIELKRGSNSVYVQYDDIMFFESSTKSHRLIAHLDNRQIEFYGNLKELSQLDDRFFRCH
NSFVVNRHNIESIDSKERIVYFKNKEHCYASVRNVKKI

>4G6DB
MKLKILDKDNATLNVFHRNKEHKTIDNVPTANLVDWYPLSNAYEYKLSRNGEYLELKRLRSTLPS
SYGLDDNNQDIIRDNNHRCKIGYWYNPAVRKDNLKIIEKAKQYGLPIITEEYDANTVEQGFRDIG
VIFQSLKTIVVTRYLEGKTEEELRIFNMKSEESQLNEALKESDFSVDLTYSDLGQIYNMLLLMKK
ISK
>4G6DA
MKEQLEDVLDTLTDREENVLRLRFGLDDGRTRTLEEVGKVFGVTRERIRQIEAKALRKLRHPSRS
KRLKDFMD
>4GNXL
TTTTTTTTTTTTTTTTTTTTTTTTTTTTTTTTTTTTTTTTTTTTTTTTTTTTTTTTT
>4GNXK
TTTTTTTTTTTTTTTTTTTTTTTTTTTTTTTTTTTTTTTTTTTTTTTTTTTTTTTTT
>4GNXZ
MPIYPIEGLSPYQNRWTIKARVTSKSDIRHWSNQRGEGKLFSVNLLDDSGEIKATGFNDAVDRFY
PLLQENHVYLISKARVNIAKKQFSNLQNEYEITFENSTEIEECTDATDVPEVKYEFVRINELESV
EANQQCDVIGILDSYGELSEIVSKASQRPVQKRELTLVDQGNRSVKLTLWGKTAETFPTNAGVDE
KPVLAFKGVKVGDFGGRSLSMFSSSTMLINPDITESHVLRGWYDNDGAHAQFQPYTNGGVGGGAM
GGGGAGANMAERRTIVQVKDENLGMSEKPDYFNVRATVVYIKQENLYYTACASEGCNKKVNLDHE
NNWRCEKCDRSYATPEYRYILSTNVADATGQMWLSGFNEDATQLIGMSAGELHKLREESESEFSA
ALHRAANRMYMFNCRAKMDTFNDTARVRYTISRAAPVDFAKAGMELVDAIRAYM
>4GS3A
SNAMAGNFLENNTVTLVGKVFTPLEFSHELYGEKFFNFILEVPRLSETKDYLPITISNRLFEGMN
LEVGTRVKIEGQLRSYNRKSPEEGKNKLILTVFARDISVVPE
>4H79A
GSMTTTETPKTISLTWVGTFVDQRVREIQEGYRLDNPRAVATLARLRRGAGKEIGDTPDLWGLIL
DDRFYADAPPLKEKDMEVAENSAHIALTLYAIHQQSRRDDRMHQRGWGLGEAVRRLMPSSEIDEP
LRKRFVQVGHAVTYKALAQRLREIVTLLRRDAIPLDYGLLADQLYQFRTPQGAQRVRTAWGRGFH
AYRPKTTQNPDSTTTTEKDNS
>4H7AB
MSRGHHHHHHGSMSPGERFLDWLKRLQGQKAWTAARAAFRRSLAFPPGAYPRAMPYVEPFLAKGD
WRQEEREAHYLVAALYALKDGDHQVGRTLARALWEKAQGSASVEKRFLALLEADRDQIAFRLRQA
VALVEGGIDFARLLDDLLRWFSPERHVQARWAREYYGA
>4H9SE
GSRRQIQRLEQLLALYVAEIRRLQEKELDLSELDDPDSAYLQEARLKRKLIRLFGRLCELKDCSS
LTGRVIEQRIPYRGTRYPEVNRRIERLINKPGPDTFPDYGDVLRAVEKAAARHSLGLPRQQLQLM
AQDAFRDVGIRLQERRHLDLIYNFGCHLTDDYRPGVDPALSDPVLARRLRENRSLAMSRLDEVIS
KYAMLQDKSEEGERKKRRARL
>4HD0B
HHHHHHMKFAHLADIHLGYEQFHKPQREEEFAEAFKNALEIAVQENVDFILIAGDLFHSSRPSPG
TLKKAIALLQIPKEHSIPVFAIEGNHDRTQRGPSVLNLLEDFGLVYVIGMRKEKVENEYLTSERL
GNGEYLVKGVYKDLEIHGMKYMSSAWFEANKEILKRLFRPTDNAILMLHQGVREVSEARGEDYFE
IGLGDLPEGYLYYARGHIHKRYETSYSGSPVVYPGSLERWDFGDYEVRYEWDGIKFKERYGVNKG
FYIVEDFKPRFVEIKVRPFIDVKIKGSEEEIRKAIKRLIPLIPKNAYVRLNIGWRKPFDLTEIKE
LLNVEYLKIDTWRI

>4HIDA
MSDSFSLLSQITPHQRCSFYAQVIKTWYSDKNFTLYVTDYTENELFFPMSPYTSSSRWRGPFGRF
SIRCILWDEHDFYCRNYIKEGDYVVMKNVRTKIDHLGYLECILHGDSAKRYNMSIEKVDSEEPEL
NEIKSRKRLYVQN
>4HLXD
MHHHHHHSSGVDLGTENLYFQSMGKASIKDWIVCQVNSGKFPGVEWEDEERTRFRIPVTPLADPC
FEWRRDGELGVVYIRERGNMPVDASFKGTRGRRRMLAALRRTRGLQEIGKGISQDGHHFLVFRVR
KP
>4HOBA
GSHMASNPISEEMNLKILAYLGTKQGAKAVHIAQSLGAQRSEVNRHLYRMSEDGRVRKHPQHPVW
YLPA
>4HP1C
MHHHHHHSSGRENLYFQGSNKKRKRCGVCVPCLRKEPCGACYNCVNRSTSHQICKMRKCEQLKKK
RVVPMKG
>4HTOA
MAASQTSQTVASHVPFADLCSTLERIQKSKGRAEKIRHFREFLDSWRKFHDALHKNHKDVTDSFY
PAMRLILPQLERERMAYGIKETMLAKLYIELLNLPRDGKDALKLLNYRTPTGTHGDAGDFAMIAY
FVLKPRCLQKGSLTIQQVNDLLDSIASNNSAKRKDLIKKSLLQLITQSSALEQKWLIRMIIKDLK
LGVSQQTIFSVFHNDAAELHNVTTDLEKVCRQLHDPSVGLSDISI
>4HW0C
MQLERRKRGTMEIMFDILRNCEPKCGITRVIYGAGINYVVAQKYLDQLVKVGALNIKTENDRKIY
EITEKGKLLRTHIEEFIKIRENLYSAKEKVSELLRTDSE
>4I1KB
MRGSHHHHHHGSRSKFYESASARKRTVTAEERERAINAAKTFEPTNPFFRVVLRPSYLYRGCIMY
LPSGFAEKYLSGISGFIKVQLAEKQWPVRCLYKAGRAKFSQGWYEFTLENNLGEGDVCVFELLRT
RDFVLKVTAFRVNEYV
>4I99D
KKVEIDEEIFVIDDFRVDIEKYVEELYKVVKKIYEKTGTPIKFWDLVPDVEPKIIARTFLYLLFL
ENMGRVEIIQEEPFGEILVVPM
>4I99B
MPYIEKLELKGFKSYGNKKVVIPFSKGFTAIVGANGSGKSNIGDAILFVLGGLSAKAMRASRISD
LIFAGSKNEPPAKYAEVAIYFNNEDRGFPIDEDEVVIRRRVYPDGRSSYWLNGRRATRSEILDIL
TAAMISPDGYNIVLQGDITKFIKMSPLERRLLIDDISGIAEYDSKKEKALEEEKEKKNVFMRTFE
AISRNFSEIFAKLSPGGSARLILENPEDPFSGGLEIEAKPAGKDVKRIEAMSGGEKALTALAFVF
AIQKFKPAPFYLFDEIDAHLDDANVKRVADLIKESSKESQFIVITLRDVMMANADKIIGVSMRDG
VSKVVSLSLEKAMKILEEIRKKQGWEHGN
>4ICGD
GSHMSDKPLTKTDYLMRLRRCQTIDTLERVIEKNKYELSDNELAVFYSAADHRLAELTMNKLYDK
IPSSVWKFIR
>4IDUB
MSSQFIFEDVPQRNAATFNPEVGYVAFIGKYGQQLNFGVARVFFLNQKKAKMVLHKTAQPSVDLT
FGGVKFTVVNNHFPQYVSNPVPDNAITLHRMSGYLARWIADTCKASVLKLAEASAQIVMPLAEVK
GCTWADGYTMYLGFAPGAEMFLDAFDFYPLVIEMHRVLKDNMDVNFMKKVLRQRYGTMTAEEWMT
QKITEIKAAFNSVGQLAWAKSGFSPAARTFLQQFGINI

>4IJHA
GSHMVGQLSRGAIAAIMQKGDTNIKPILQVINIRPITTGNSPPRYRLLMSDGLNTLSSFMLATQL
NPLVEEEQLSSNCVCQIHRFIVNTLKDGRRVVILMELEVLKSAEAVGVKIGNPVPYNE
>4IRHA
GAMVPKTEDQRPQLDPYQILGPTSSRLANPGSGQIQLWQFLLELLSDSSNSSCITWEGTNGEFKM
TDPDEVARRWGERKSKPNMNYDKLSRALRYYYDKNIMTKVHGKRYAYKFDFHGIAQALQPHPPE
>4IX7B
DNVMVSIGPNNTCVPASVFENINWSVCSLATRKLLVTIFDRETLATHSVTGKPSPAFKDQDKPLK
RMLDPGKIQDIIFAVTHKCNASEKEVRNAITTKCADENKMMKIQNVKRRS
>4JJNJ
ATCGGATGTATATATCTGACACGTGCCTGGAGACTAGGGAGTAATCCCCTTGGCGGTTAAAACGC
GGGGGACAGCGCGTACGTGCGTTTAAGCGGTGCTAGAGCTGTCTACGACCAATTGAGCGGCCTCG
GCACCGGGATTCTCGAT
>4JJNI
ATCGAGAATCCCGGTGCCGAGGCCGCTCAATTGGTCGTAGACAGCTCTAGCACCGCTTAAACGCA
CGTACGCGCTGTCCCCCGCGTTTTAACCGCCAAGGGGATTACTCCCTAGTCTCCAGGCACGTGTC
AGATATATACATCCGAT
>4JJNL
SAKTLKDLDGWQVIITDDQGRVIDDNNRRRSRKRGGENVFLKRISDGLSFGKGESVIFNDNVTET
YSVYLIHEIRLNTLNNVVEIWVFSYLRWFELKPKLYYEQFRPDLIKEDHPLEFYKDKFFNEVNKS
ELYLTAELSEIWLKDFIAVGQILPESQWNDSSIDKIEDRDFLVRYACEPTAEKFVPIDIFQIIRR
VKEMEPKQSNEYLKRVSVPVSGQKTNRQVMHKMGVERSSKRLAKKPSMKKIKIEPSADDDVNNGN
IPSQRGTSTTHGSISPQEESVSPNISSASPSALTSPTDSSKILQKRSISKELIVSEEIPINSSEQ
ESDYEPNNETSVLSSKPGSKPEKTSTELVDGRENFVYANNPEVSDDGGLEEETDEVS
>4JJNH
SSAAEKKPASKAPAEKKPAAKKTSTSVDGKKRSKVRKETYSSYIYKVLKQTHPDTGISQKSMSIL
NSFVNDIFERIATEASKLAAYNKKSTISAREIQTAVRLILPGELAKHAVSEGTRAVTKYSSSTQA
>4JJNG
SGGKGGKAGSAAKASQSRSAKAGLTFPVGRVHRLLRRGNYAQRIGSGAPVYLTAVLEYLAAEILE
LAGNAARDNKKTRIIPRHLQLAIRNDDELNKLLGNVTIAQGGVLPNIHQNLLPKKSAKTAKASQE
L
>4JLXA
GAMGAWKLQTVLEKVRLSRHEISEAAEVVNWVVEHLLRRLQGGESEFKGVALLRTGSYYERVKIS
APNEFDVMFKLEVPRIQLEEYCNSGAHYFVKFKRNPGGNPLEQFLEKEILSASKMLSKFRKIIKE
EIKNIEDTGVTVERKRRGSPAVTLLISKPKEISVDIILALESKSSWPASTQKGLPISQWLGAKVK
NNLKRQPFYLVPKHAKEGSGFQEETWRLSFSHIEKDILKNHGQSKTCCEIDGVKCCRKECLKLMK
YLLEQLKKKFGNRRELAKFCSYHVKTAFFHVCTQDPHDNQWHLKNLECCFDNCVAYFLQCLKTEQ
LANYFIPGVNLFSRDLIDKPSKEFLSKQIEYERNNGFPVFW
>4JOIC
MLPKPGTYYLPWEVSAGQVPDGSTLRTFGRLCLYDMIQSRVTLMAQHGSDQHQVLVCTKLVEPFH
AQVGSLYIVLGELQHQQDRGSVVKARVLTCVEGMNLPLLEQAIREQRLYKQERGGSQ
>4JOIB
LDPVFLAFAKLYIRDILDMKESRQVPGVFLYNGHPIKQVDVLGTVIGVRERDAFYSYGVDDSTGV
INCICWKKLNTESVSAAPSAARELSLTSQLKKLQETIEQKTKIEIGDTIRVRGSIRTYREEREIH

ATTYYKVDDPVWNIQIARMLELPTIYRKVYDQPFHS
>4JOLD
SEEMIDHRLTDREWAEEWKHLDHLLNCIMDMVEKTRRSLTVLRRCQEADREELNYWIRRYSDAE
>4JQFA
AEALSNPGALDLPSLTSLLSEKAKEFLMENRVQSFYQQELEMVESLLSLANQPVIHSASSDQVNF
KKDTTSKAIHSIFKNAIQLLQEKGLVFQKDDGFDNLYYVTREDKDLHRKIHRIIQQDCQKPNHME
KGCHFLHILACARLSIRPGLSEAVLQQVLELLEDQSDIVSTMEHYYTAF
>4JW3D
MRGSHHHHHHTDPEKVEMYIKNLQDDSYFVRRAAAAALGKIGDERAVEPLIKALKDEDRFVRSSA
AYALGEIGDERAVEPLIKALKDEDWFVRRAAAVALGEIGGERVRAAMEKLAETGTGFARKVAVNY
LETHKSLIS
>4JW3B
AAPTATVTPSSGLSDGTVVKVAGAGLQAGTAYWVAQWARVDTGVWAYNPADNSSVTADANGSAST
SLTVRRSFEGFLFDGTRWGTVDCTTAACQVGLSDAAGNGPEGVAISFAAHHHHHH
>4K2JA
SHPRYQQPPVPYRQIDDCPAKARPQHIFYRRFLGKDGRRDPKCQWKFAVIFWGNDPYGLKKLSQA
FQFGGVKAGPVSCLPHPGPDQSPITYCVYVYCQNKDTSKKVQMARLAWEASHPLAGNLQSSIVKF
KKPLPLTQPG
>4K74B
HHHHHHMKFTVEREHLLKPLQQVSGPLGGRPTLPILGNLLLQVADGTLSLTGTDLEMEMVARVAL
VQPHEPGATTVPARKFFDICRGLPEGAEIAVQLEGERMLVRSGRSRFSLSTLPAADFPNLDDWQS
EVEFTLPQATMKRLIEATQFSMAHQDVRYYLNGMLFETEGEELRTVATDGHRLAVCSMPIGQSLP
SHSVIVPRKGVIELMRMLDGGDNPLRVQIGSNNIRAHVGDFIFTSKLVDGRFPDYRRVLPKNPDK
HLEAGCDLLKQAFARAAILSNEKFRGVRLYVSENQLKITANNPEQEEAEEILDVTYSGAEMEIGF
NVSYVLDVLNALKCENVRMMLTDSVSSVQIEDAASQSAAYVVMPMRL
>4KPYA
MNHLGKTEVFLNRFALRPLNPEELRPWRLEVVLDPPPGREEVYPLLAQVARRAGGVTVRMGDGLA
SWSPPEVLVLEGTLARMGQTYAYRLYPKGRRPLDPKDPGERSVLSALARRLLQERLRRLEGVWVE
GLAVYRREHARGPGWRVLGGAVLDLWVSDSGAFLLEVDPAYRILCEMSLEAWLAQGHPLPKRVRN
AYDRRTWELLRLGEEDPKELPLPGGLSLLDYHASKGRLQGREGGRVAWVADPKDPRKPIPHLTGL
LVPVLTLEDLHEEEGSLALSLPWEERRRRTREIASWIGRRLGLGTPEAVRAQAYRLSIPKLMGRR
AVSKPADALRVGFYRAQETALALLRLDGAQGWPEFLRRALLRAFGASGASLRLHTLHAHPSQGLA
FREALRKAKEEGVQAVLVLTPPMAWEDRNRLKALLLREGLPSQILNVPLREEERHRWENALLGLL
AKAGLQVVALSGAYPAELAVGFDAGGRESFRFGGAACAVGGDGGHLLWTLPEAQAGERIPQEVVW
DLLEETLWAFRRKAGRLPSRVLLLRDGRVPQDEFALALEALAREGIAYDLVSVRKSGGGRVYPVQ
GRLADGLYVPLEDKTFLLLTVHRDFRGTPRPLKLVHEAGDTPLEALAHQIFHLTRLYPASGFAFP
RLPAPLHLADRLVKEVGRLGIRHLKEVDREKLFFV
>4LDUA
MMASLSCVEDKMKTSCLVNGGGTITTTTSQSTLLEEMKLLKDQSGTRKPVINSELWHACAGPLVC
LPQVGSLVYYFSQGHSEQVAVSTRRSATTQVPNYPNLPSQLMCQVHNVTLHADKDSDEIYAQMSL
QPVHSERDVFPVPDFGMLRGSKHPTEFFCKTLTASDTSTHGGFSVPRRAAEKLFPPLDYSAQPPT
QELVVRDLHENTWTFRHIYRGQPKRHLLTTGWSLFVGSKRLRAGDSVLFIRDEKSQLMVGVRRAN
RQQTALPSSVLSADSMHIGVLAAAAHATANRTPFLIFYNPRACPAEFVIPLAKYRKAICGSQLSV
GMRFGMMFETEDSGKRRYMGTIVGISDLDPLRWPGSKWRNLQVEWDEPGCNDKPTRVSPWDIETP

NSYSQSM
>4LG8A
MHHHHHHSSGRENLYFQGTPEIIQKLQDKATVLTTERKERGKTVPEELVKPEELSKYRQVASHVG
LHSASIPGILALDLCPSDTNKILTGGADKNVVVFDKSSEQILATLKGHTKKVTSVVFHPSQDLVF
SASPDATIRIWSVPNASCVQVVRAHESAVTGLSLHATGDYLLSSSDDQYWAFSDIQTGRVLTKVT
DETSGCSLTCAQFHPDGLIFGTGTMDSQIKIWDLKERTNVANFPGHSGPITSIAFSENGYYLATA
ADDSSVKLWDLRKLKNFKTLQLDNNFEVKSLIFDQSGTYLALGGTDVQIYICKQWTEILHFTEHS
GLTTGVAFGHHAKFIASTGMDRSLKFYSL
>4LJKA
MKSHFQYSTLENIPKAFDILKDPPKKLYCVGDTKLLDTPLKVAIIGTRRPTPYSKQHTITLAREL
AKNGAVIVSGGALGVDIIAQENALPKTIMLSPCSLDFIYPTNNHKVIQEIAQNGLILSEYEKDFM
PIKGSFLARNRLVIALSDVVIIPQADLKSGSMSSARLAQKYQKPLFVLPQRLNESDGTNELLEKG
QAQGIFNIQNFINTLLKDYHLKEMPELEHHHHHH
>4LRVL
SMLPNRMALSRQTEDQLKKLKGYTGITPNIAARLAFFRSVESEFRYSPERDSKKLDGTLVLDKIT
WLGETLQATELVLKMLYPQLEQKALIKAWAAHVEDGIAALRNHK
>4M6WB
MGHIVANEKWRGSQLAQEMQGKIKLIFEDGLTPDFYLSNRCCILYVTEADLVAGNGYRKRLVRVR
NSNNLKGIVVVEKTRMSEQYFPALQKFTVLDLGMVLLPVASQMEASCLVIQLVQEQTKEPSKNPL
LGKKRALLLSEPSLLRTVQQIPGVGKVKAPLLLQKFPSIQQLSNASIGELEQVVGQAVAQQIHAF
FTQPRLEHHHHHH
>4M6WA
MGQEGKGTCILVGGHEITSGLEVISSLRAIHGLQVEVCPLNGCDYIVSNRMVVERRSQSEMLNSV
NKNKFIEQIQHLQSMFERICVIVEKDREKTGDTSRMFRRTKSYDSLLTTLIGAGIRILFSSCQEE
TADLLKELSLVEQRKNVGIHVPTVVNSNKSEALQFYLSIPNISYITALNMCHQFSSVKRMANSSL
QEISMYAQVTHQKAEEIYRYIHYVFD
>4MZ9D
MASRGVNKVILVGNLGQDPEVRYMPNGGAVANITLATSESWRDKATGEMKEQTEWHRVVLFGKLA
EVASEYLRKGSQVYIEGQLRTRKWTDQSGQDRYTTEVVVNVGGTMQMLGGRQGGGAPAGGNIGGG
QPQGGWGQPQQPQGGNQFSGGAQSRPQQSAPAAPSNEPPMDFDDDIPF
>4N0UE
GGPSVFLFPPKPKDTLYITREPEVTCVVVDVSHEDPEVKFNWYVDGVEVHNAKTKPREEQYNSTY
RVVSVLTVLHQDWLNGKEYKCKVSNKALPAPIEKTISKAKGQPREPQVYTLPPSRDELTKNQVSL
TCLVKGFYPSDIAVEWESNGQPENNYKTTPPVLDSDGSFFLYSKLTVDKSRWQQGNVFSCSVMHE
ALHNHYTQKSLSLS
>4N0UD
HKSEVAHRFKDLGEENFKALVLIAFAQYLQQCPFEDHVKLVNEVTEFAKTCVADESAENCDKSLH
TLFGDKLCTVATLRETYGEMADCCAKQEPERNECFLQHKDDNPNLPRLVRPEVDVMCTAFHDNEE
TFLKKYLYEIARRHPYFYAPELLFFAKRYKAAFTECCQAADKAACLLPKLDELRDEGKASSAKQR
LKCASLQKFGERAFKAWAVARLSQRFPKAEFAEVSKLVTDLTKVHTECCHGDLLECADDRADLAK
YICENQDSISSKLKECCEKPLLEKSHCIAEVENDEMPADLPSLAADFVESKDVCKNYAEAKDVFL
GMFLYEYARRHPDYSVVLLLRLAKTYETTLEKCCAAADPHECYAKVFDEFKPLVEEPQNLIKQNC
ELFEQLGEYKFQNALLVRYTKKVPQVSTPTLVEVSRNLGKVGSKCCKHPEAKRMPCAEDYLSVVL
NQLCVLHEKTPVSDRVTKCCTESLVNRRPCFSALEVDETYVPKEFNAETFTFHADICTLSEKERQ

IKKQTALVELVKHKPKATKEQLKAVMDDFAAFVEKCCKADDKETCFAEEGKKLVAASQAALGL
>4N0UB
IQRTPKIQVYSRHPAENGKSNFLNCYVSGFHPSDIEVDLLKNGERIEKVEHSDLSFSKDWSFYLL
YYTEFTPTEKDEYACRVNHVTLSQPKIVKWDRDM
>4N0UA
HLSLLYHLTAVSSPAPGTPAFWVSGWLGPQQYLSYNSLRGEAEPCGAWVWENQVSWYWEKETTDL
RIKEKLFLEAFKALGGKGPYTLQGLLGCELGPDNTSVPTAKFALNGEEFMNFDLKQGTWGGDWPE
ALAISQRWQQQDKAANKELTFLLFSCPHRLREHLERGRGNLEWKEPPSMRLKARPSSPGFSVLTC
SAFSFYPPELQLRFLRNGLAAGTGQGDFGPNSDGSFHASSSLTVKSGDEHHYCCIVQHAGLAQPL
RVEL
>4N6QA
MSAANYPDPSLPRPSTSDDFELIVRQNPNRARVAGGKEKERKPVDPPPIVQIRVREEGTYLAQHY
LQSPYFFMSCSLYDAQEDAPASIPPSTALTGTLVSSLHRLKDVDNTDGGFFVWGDLSIKVEGDFR
LKFSLFEMRKTDVVFLKSIVSERFTVSPPKSFPGMAESTFLSRSFADQGVKLRIRKEPRTSAWSH
PQFEK
>4N6RB
MYAVEDRAHSGHHPPPLSMDRIPPPSTMYPSSAGPSAMVSPAGQPEPESLSTVHDGRIWSLQVVQ
QPIRARMCGFGDKDRRPITPPPCIRLIVKDAQTQKEVDINSLDSSFYVVMADLWNADGTHEVNLV
KHSATSPSISTAMSSSYPPPPHPTSSDYPASYQTNPYGQPVGQPVGQPVGYAGVGNYYGGSTQLQ
YQNAYPNPQAQYYQPMYGGMAQPQMPAAQPVTPGPGGMFTRNLIGCLSASAYRLYDTEDKIGVWF
VLQDLSVRTEGIFRLKFSFVNVGKSVSDLPQSDIAEVINKGTAPILASTFSEPFQVFSAKKFPGV
IESTPLSKVFANQGIKIPIRKDGVKGQGSRGRHSDEDDGLDNEYSAHHHHHH
>4NDFA
GSHMGHWSQGLKISMQDPKMQVYKDEQVVVIKDKYPKARYHWLVLPWTSISSLKAVAREHLELLK
HMHTVGEKVIVDFAGSSKLRFRLGYHAIPSMSHVHLHVISQDFDSPCLKNKKHWNSFNTEYFLES
QAVIEMVQEAGRVTVRDGMPELLKLPLRCHECQQLLPSIPQLKEHLRKHWTQ
>4NJXD
MSHHHHHHSMAAAVVLAAGLRAARRAVAATGVRGGQVRGAAGVTDGNEVAKAQQATPGGAAPTIF
SRILDKSLPADILYEDQQCLVFRDVAPQAPVHFLVIPKKPIPRISQAEEEDQQLLGHLLLVAKQT
AKAEGLGDGYRLVINDGKLGAQSVYHLHIHVLGGRQLQWPPG
>4NL4H
HHHHHHSSGLVPRGSHMSVAHVALPVPLPRTFDYLLPEGMAVKAGCRVRVPFGKQERIGIVAAVS
ERSELPLDELKPVAEALDDEPVFSTTVWRLLMWAAEYYHHPIGDVLFHALPVMLRQGKPASATPL
WYWFATEQGQVVDLNGLKRSRKQQQALAALRQGKIWRHQVGELEFNEAALQALRGKGLAELACEA
PALTDWRSAYSVAGERLRLNTEQATAVGAIHSAADRFSAWLLAGITGSGKTEVYLSVLENVLAQG
RQALVMVPEIGLTPQTIARFRQRFNAPVEVLHSGLNDSERLSAWLKAKNGEAAIVIGTRSSLFTP
FKDLGVIVIDEEHDSSYKQQEGWRYHARDLAVWRAHSEQIPIILGSATPALETLHNVRQGKYRQL
TLSKRAGNARPAQQHVLDLKGQPLQAGLSPALISRMRQHLQADNQVILFLNRRGFAPALLCHDCG
WIAECPRCDSYYTLHQAQHHLRCHHCDSQRPIPRQCPSCGSTHLVPVGIGTEQLEQALAPLFPEV
PISRIDRDTTSRKGALEEHLAAVHRGGARILIGTQMLAKGHHFPDVTLVSLLDVDGALFSADFRS
AERFAQLYTQVSGRAGRAGKQGEVILQTHHPEHPLLQTLLYKGYDAFAEQALAERQTMQLPPWTS
HVLIRAEDHNNQQAPLFLQQLRNLLQASPLADEKLWVLGPVPALAPKRGGRWRWQILLQHPSRVR
LQHIVSGTLALINTLPEARKVKWVLDVDPIEG
>4NQWB

MTEHTDFELLELATPYALNAVSDDERADIDRRVAAAPSPVAAAFNDEVRAVRETMAVVSAATTAE
PPAHLRTAILDATKP

(2).550 non DNA-binding proteins

>1RQWA
ATFEIVNRCSYTVWAAASKGDAALDAGGRQLNSGESWTINVEPGTKGGKIWARTDCYFDDSGSGI
CKTGDCGGLLRCKRFGRPPTTLAEFSLNQYGKDYIDISNIKGFNVPMDFSPTTRGCRGVRCAADI
VGQCPAKLKAPGGGCNDACTVFQTSEYCCTTGKCGPTEYSRFFKRLCPDAFSYVLDKPTTVTCPG
SSNYRVTFCPTA
>1H2GB
SNMWVIGKSKAQDAKAIMVNGPQFGWYAPAYTYGIGLHGAGYDVTGNTPFAYPGLVFGHNGVISW
GSTAGLGDDVDIFAERLSAEKPGYYLHNGKWVKMLSREETITVKNGQAETFTVWRTVHGNILQTD
QTTQTAYAKSRAWDGKEVASLLAWTHQMKAKNWQEWTQQAAKQALTINWYYADVNGNIGYVHTGA
YPDRQSGHDPRLPVPGTGKWDWKGLLPFEMNPKVYNPQSGYIANWNNSPQKDYPASDLFAFLWGG
ADRVTEIDRLLEQKPRLTADQAWDVIRQTSRQDLNLRLFLPTLQAATSGLTQSDPRRQLVETLTR
WDGINLLNDDGKTWQQPGSAILNVWLTSMLKRTVVAAVPMPFDKWYSASGYETTQDGPTGSLNIS
VGAKILYEAVQGDKSPIPQAVDLFAGKPQQEVVLAALEDTWETLSKRYGNNVSNWKTPAMALTFR
ANNFFGVPQAAAEETRHQAEYQNRGTENDMIVFSPTTSDRPVLAWDVVAPGQSGFIAPDGTVDKH
YEDQLKMYENFGRKSLWLTKQDVEAHKESQEVLHVQR
>1A12A
RRSPPADAIPKSKKVKVSHRSHSTEPGLVLTLGQGDVGQLGLGENVMERKKPALVSIPEDVVQAE
AGGMHTVCLSKSGQVYSFGCNDEGALGRDTSVEGSEMVPGKVELQEKVVQVSAGDSHTAALTDDG
RVFLWGSFRDNNGVIGLLEPMKKSMVPVQVQLDVPVVKVASGNDHLVMLTADGDLYTLGCGEQGQ
LGRVPELFANRGGRQGLERLLVPKCVMLKSRGSRGHVRFQDAFCGAYFTFAISHEGHVYGFGLSN
YHQLGTPGTESCFIPQNLTSFKNSTKSWVGFSGGQHHTVCMDSEGKAYSLGRAEYGRLGLGEGAE
EKSIPTLISRLPAVSSVACGASVGYAVTKDGRVFAWGMGTNYQLGTGQDEDAWSPVEMMGKQLEN
RVVLSVSSGGQHTVLLVKDKEQS
>3INGA
GMKEIRIILMGTGNVGLNVLRIIDASNRRRSAFSIKVVGVSDSRSYASGRNLDISSIISNKEKTG
RISDRAFSGPEDLMGEAADLLVDCTPASRDGVREYSLYRMAFESGMNVVTANKSGLANKWHDIMD
SANQNSKYIRYEATVAGGVPLFSVLDYSILPSKVKRFRGIVSSTINYVIRNMANGRSLRDVVDDA
IKKGIAESNPQDDLNGLDAARKSVILVNHIFGTEYTLNDVEYSGVDERSYNANDRLVTEVYVDDR
RPVAVSRIISLNKDDFLMSIGMDGLGYQIETDSNGTVNVSDIYDGPYETAGAVVNDILLLSKVQK
>2RAUA
GMYEEWKIVKREAPILGNDQLIENIWKMKREDSPYDIISLHKVNLIGGGNDAVLILPGTWSSGEQ
L23VTISWNGVHYTIPDYRKSIVLYLARNGFNVYTIDYRTHYVPPFLKDRQLSFTANWGWSTWIS
DIKEVVSFIKRDSGQERIYLAGESFGGIAALNYSSLYWKNDIKGLILLDGGPTKHGIRPKFYTPE
VNSIEEMEAKGIYVIPSRGGPNNPIWSYALANPDMPSPDPKYKSISDFLMDSLYVTGSANPYDYP
YSKKEDMFPILASFDPYWPYRLSLERDLKFDYEGILVPTIAFVSERFGIQIFDSKILPSNSEIIL
LKGYGHLDVYTGENSEKDVNSVVLKWLSQQR
>1UAIA
AEPCDYPAQQLDLTDWKVTLPIGSSGKPSEIEQPALDTFATAPWFQVNAKCTGVQFRAAVNGVTT
SGSGYPRSELREMTDGGEEKASWSATSGTHTMVFREAFNHLPEVKPHLVGAQIHDGDDDVTVFRL
EGTSLYITKGDDTHHKLVTSDYKLNTVFEGKFVVSGGKIKVYYNGVLQTTISHTSSGNYFKAGAY

TQANCSNSSPCSSSNYGQVSLYKLQVTHS

>3H5QA

SNAMRMIDIIEKKRDGHTLTTEEINFFIGGYVKGDIPDYQASSLAMAIYFQDMNDDERVALTMAM
VNSGDMIDLSDIKGVKVDKHSTGGVGDTTTLVLAPLVAAVDVPVAKMSGRGLGHTGGTIDKLEAI
DGFHVEIDEATFVKLVNENKVAVVGQSGNLTPADKKLYALRDVTGTVNSIPLIASSIMSKKIAAG
ADAIVLDVKTGSGAFMKTLEDAEALAHAMVRIGNNVGRNTMAIISDMNQPLGRAIGNALELQEAI
DTLKGQGPKDLTELVLTLGSQMVVLANKAETLEEARALLIEAINSGAALEKFKTFIKNQGGDETV
IDHPERLPQAQYQIEYKAKKSGYVTELVSNDIGVASMMLGAGRLTKEDDIDLAVGIVLNKKIGDK
VEEGESLLTIHSNRQDVDDVVKKLDSSITIADHVVSPTLIHKIITE

>3N20A

MSMLKREDWYDLTRTTNWTPKYVTENELFPEEMSGARGISMEAWEKYDEPYKITYPEYVSIQREK
DSGAYSIKAALERDGFVDRADPGWVSTMQLHFGAIALEEYAASTAEARMARFAKAPGNRNMATFG
MMDENRHGQIQLYFPYANVKRSRKWDWAHKAIHTNEWAAIAARSFFDDMMMTRDSVAVSIMLTFA
FETGFVNMQFLGLAADAAEAGDHTFASLISSIQTDESRHAQQGGPSLKILVENGKKDEAQQMVDV
AIWRSWKLFSVLTGPIMDYYTPLESRNQSFKEFMLEWIVAQFERQLLDLGLDKPWYWDQFMQDLD
ETHHGMHLGVWYWRPTVWWDPAAGVSPEEREWLEEKYPGWNDTWGQCWDVITDNLVNGKPELTVP
ETLPTICNMCNLPIAHTPGNKWNVKDYQLEYEGRLYHFGSEADRWCFQIDPERYENHTNLVDRFL
KGEIQPADLAGALMYMSLEPGVMGDDAHDYEWVKAYQKKTNAA

>3N2BA

MHHHHHHSSGVDLGTENLYFQSNAMDYFNYQEDGQLWAEQVPLADLANQYGTPLYVYSRATLERH
WHAFDKSVGDYPHLICYAVKANSNLGVLNTLARLGSGFDIVSVGELERVLAAGGDPSKVVFSGVG
KTEAEMKRALQLKIKCFNVESEPELQRLNKVAGELGVKAPISLRINPDVDAKTHPYISTGLRDNK
FGITFDRAAQVYRLAHSLPNLDVHGIDCHIGSQLTALAPFIDATDRLLALIDSLKAEGIHIRHLD
VGGGLGVVYRDELPPQPSEYAKALLDRLERHRDLELIFEPGRAIAANAGVLVTKVEFLKHTEHKN
FAIIDAAMNDLIRPALYQAWQDIIPLRPRQGEAQTYDLVGPVCETSDFLGKDRDLVLQEGDLLAV
RSSGAYGFTMSSNYNTRPRVAEVMVDGNKTYLVRQREELSSLWALESVLPE

>1P0WA

TIKEMPQPKTFGELKNLPLLNTDKPVQALMKIADELGEIFKFEAPGRVTRYLSSQRLIKEACDES
RFDKNLSQALKFVRDFAGDGLFTSWTHEKNWKKAHNILLPSFSQQAMKGYHAMMVDIAVQLVQKW
ERLNADEHIEVPEDMTRLTLDTIGLCGFNYRFNSFYRDQPHPFITSMVRALDEAMNKLQRANPDD
PAYDENKRQFQEDIKVMNDLVDKIIADRKASGEQSDDLLTHMLNGKDPETGEPLDDENIRYQIIT
FLIAGHETTSGLLSFALYFLVKNPHVLQKAAEEAARVLVDPVPSYKQVKQLKYVGMVLNEALRLW
PTAPAFSLYAKEDTVLGGEYPLEKGDELMVLIPQLHRDKTIWGDDVEEFRPERFENPSAIPQHAF
KPWGNGQRACIGQQFALHEATLVLGMMLKHFDFEDHTNYELDIKETLTLKPEGFVVKAKSKKIPL

>1UJ0A

AGHMARRVRALYDFEAVEDNELTFKHGELITVLDDSDANWWQGENHRGTGLFPSNFVTTDLS

>1UJMA

AKIDNAVLPEGSLVLVTGANGFVASHVVEQLLEHGYKVRGTARSASKLANLQKRWDAKYPGRFET
AVVEDMLKQGAYDEVIKGAAGVAHIASVVSFSNKYDEVVTPAIGGTLNALRAAAATPSVKRFVLT
SSTVSALIPKPNVEGIYLDEKSWNLESIDKAKTLPESDPQKSLWVYAASKTEAELAAWKFMDENK
PHFTLNAVLPNYTIGTIFDPETQSGSTSGWMMSLFNGEVSPALALMPPQYYVSAVDIGLLHLGCL
VLPQIERRRVYGTAGTFDWNTVLATFRKLYPSKTFPADFPDQGQDLSKFDTAPSLEILKSLGRPG
WRSIEESIKDLVGSETA

>1PAHA

TVPWFPRTIQELDRFANQILSYGAELDADHPGFKDPVYRARRKQFADIAYNYRHGQPIPRVEYME
EEKKTWGTVFKTLKSLYKTHACYEYNHIFPLLEKYCGFHEDNIPQLEDVSQFLQTCTGFRLRPVA
GLLSSRDFLGGLAFRVFHCTQYIRHGSKPMYTPEPDICHELLGHVPLFSDRSFAQFSQEIGLASL
GAPDEYIEKLATIYWFTVEFGLCKQGDSIKAYGAGLLSSFGELQYCLSEKPKLLPLELEKTAIQN
YTVTEFQPLYYVAESFNDAKEKVRNFAATIPRPFSVRYDPYTQRIEVL
>1XKWA
ESTSATQPPGVTTLGKVPLKPRELPQSASVIDHERLEQQNLFSLDEAMQQATGVTVQPFQLLTTA
YYVRGFKVDSFELDGVPALLGNTASSPQDMAIYERVEILRGSNGLLHGTGNPAATVNLVRKRPQR
EFAASTTLSAGRWDRYRAEVDVGGPLSASGNVRGRAVAAYEDRDYFYDVADQGTRLLYGVTEFDL
SPDTLLTVGAQYQHIDSITNMAGVPMAKDGSNLGLSRDTYLDVDWDRFKWDTYRAFGSLEQQLGG
GWKGKVSAEYQEADSRLRYAGSFGAIDPQTGDGGQLMGAAYKFKSIQRSLDANLNGPVRLFGLTH
ELLGGVTYAQGETRQDTARFLNLPNTPVNVYRWDPHGVPRPQIGQYTSPGTTTTTQKGLYALGRI
KLAEPLTLVVGGRESWWDQDTPATRFKPGRQFTPYGGLIWDFARDWSWYVSYAEVYQPQADRQTW
NSEPLSPVEGKTYETGIKGELADGRLNLSLAAFRIDLENNPQEDPDHPGPPNNPFYISGGKVRSQ
GFELEGTGYLTPYWSLSAGYTYTSTEYLKDSQNDSGTRYSTFTPRHLLRLWSNYDLPWQDRRWSV
GGGLQAQSDYSVDYRGVSMRQGGYALVNMRLGYKIDEHWTAAVNVNNLFDRTYYQSLSNPNWNNR
YGEPRSFNVSLRGAF
>2EX4A
MGSSHHHHHHSSGLVPRGSTSEVIEDEKQFYSKAKTYWKQIPPTVDGMLGGYGHISSIDINSSRK
FLQRFLREGPNKTGTSCALDCGAGIGRITKRLLLPLFREVDMVDITEDFLVQAKTYLGEEGKRVR
NYFCCGLQDFTPEPDSYDVIWIQWVIGHLTDQHLAEFLRRCKGSLRPNGIIVIKDNMAQEGVILD
DVDSSVCRDLDVVRRIICSAGLSLLAEERQENLPDEIYHVYSFALR
>1ZDQA
ATAAEIAALPRQKVELVDPPFVHAHSQVAEGGPKVVEFTMVIEEKKIVIDDAGTEVHAMAFNGTV
PGPLMVVHQDDYLELTLINPETNTLMHNIDFHAATGALGGGGLTEINPGEKTILRFKATKPGVFV
YHCAPPGMVPWHVVSGGNGAIMVLPREGLHDGKGKALTYDKIYYVGEQDFYVPRDENGKYKKYEA
PGDAYEDTVKVMRTLTPTHVVFNGAVGALTGDKAMTAAVGEKVLIVHSQANRDTRPHLIGGHGDY
VWATGKFNTPPDVDQETWFIPGGAAGAAFYTFQQPGIYAYVNHNLIEAFELGAAAHFKVTGEWND
DLMTSVLAPSG
>3ME7A
MSLGTYVPGDITLVDSYGNEFQLKNLKGKPIILSPIYTHCRAACPLITKSLLKVIPKLGTPGKDF
WVITFTFDPKDTLEDIKRFQKEYGIDGKGWKVVKAKTSEDLFKLLDAIDFRFMTAGNDFIHPNVV
VVLSPELQIKDYIYGVNYNYLEFVNALRLARGEGHHHHHH
>2NT3A
GSHMSKKILIVESDTALSATLRSALEGRGFTVDETTDGKGSVEQIRRDRPDLVVLAVDLSAGQNG
YLICGKLKKDDDLKNVPIVIIGNPDGFAQHRKLKAHADEAVAKPVDADQLVERAGALIGFPE
>1RFXA
SSMPLCPIDEAIDKKIKQDFNSLFPNAIKNIGLNCWTVSSRGKLASCPEGTAVLSCSCGSACGSW
DIREEKVCHCQCARIDWTAARCCKLQVAS
>1C7JA
MTHQIVTTQYGKVKGTTENGVHKWKGIPYAKPPVGQWRFKAPEPPEVWEDVLDATAYGPVCPQPS
DLLSLSYTELPRQSEDCLYVNVFAPDTPSQNLPVMVWIHGGAFYLGAGSEPLYDGSKLAAQGEVI
VVTLNYRLGPFGFMHLSSFDEAYSDNLGLLDQAAALKWVRENISAFGGDPDNVTVFGESAGGMSI
AALLAMPAAKGLFQKAIMESGASRTMTKEQAASTAAAFLQVLGINESQLDRLHTVAAEDLLKAAD

QLRIAEKENIFQLFFQPALDPKTLPEEPEKSIAEGAASGIPLLIGTTRDEGYLFFTSDSDVRSQE
TLDAALEYSLGKPLAEKAADLYPRSLESQIHMVTDLLFWRPAVAFASAQSHYAPVWMYRFDWHPE
KPPYNKAFHALELPFVFGNLDGLERMAKAEITDEVKQLSHTIQSAWITFAKTGNPSTEAVNWPAY
HEETRETVILDSEITIENDPESEKRQKLFPSKGE
>1H0HB
SKGFFVDTTRCTACRGCQVACKQWHGNPATPTENTGFHQNPPDFNFHTYKLVRMHEQEIDGRIDW
LFFPDQCRHCIAPPCKATADMEDESAIIHDDATGCVLFTPKTKDLEDYESVISACPYDVPRKVAE
SNQMAKCDMCIDRITNGLRPACVTSCPTGAMNFGDLSEMEAMASARLAEIKAAYSDAKLCDPDDV
RVIFLTAHNPKLYHEYAVA
>1ZE3D
DLYFNPRFLADDPQAVADLSRFENGQELPPGTYRVDIYLNNGYMATRDVTFNTGDSEQGIVPCLT
RAQLASMGLNTASVAGMNLLADDACVPLTTMVQDATAHLDVGQQRLNLTIPQAFMSNRAR
>1UHAA
APECGERASGKRCPNGKCCSQWGYCGTTDNYCGQGCQSQCDYWRCGRDFGGRLCEEDMCCSKYGW
CGYSDDHCEDGCQSQCD
>1J8MF
SKLLDNLRDTVRKFLTGSSSYDKAVEDFIKELQKSLISADVNVKLVFSLTNKIKERLKNEKPPTY
IERREWFIKIVYDELSNLFGGDKEPKVIPDKIPYVIMLVGVQGTGKTTTAGKLAYFYKKKGFKVG
LVGADVYRPAALEQLQQLGQQIGVPVYGEPGEKDVVGIAKRGVEKFLSEKMEIIIVDTAGRHGYG
EEAALLEEMKNIYEAIKPDEVTLVIDASIGQKAYDLASKFNQASKIGTIIITKMDGTAKGGGALS
AVAATGATIKFIGTGEKIDELEVFNPRRFVARLHHHH
>3K8GA
GSGAWKASVDPLGVVGSGADVYLYFPVAGNENLISRIIENHESKADIKKIVDRTTAVYGAFFARS
KEFRLFGSGSYPYAFTNLIFSRSDGWASTKTEHGITYYESEHTDVSIPAPHFSCVIFGSSKRERM
SKMLSRLVNPDRPQLPPRFEKECTSEGTSQTVALYIKNGGHFITKLLNFPQLNLPLGAMELYLTA
RRNEYLYTLSLQLGNAKINFPIQFLISRVLNAHIHVEGDRLIIEDGTISAERLASVISSLYSKKG
SS
>2B1LA
MQFYQADVLTQGKPVLLNVWATWCPTCRAEHQYLNQLSAQGIRVVGMNYKDDRQKAISWLKELGN
PYALSLFDGDGMLGLDLGVYGAPETFLIDGNGIIRYRHAGDLNPRVWEEEIKPLWEKYSKEAAQ
>2ZZ3A
GSHMRSRRVDVMDVMNRLILAMDLMNRDDALRVTGEVREYIDTVKIGYPLVLSEGMDIIAEFRKR
FGCRIIAAFKVADIPETNEKICRATFKAGADAIIVHGFPGADSVRACLNVAEEMGREVFLLTEMS
HPGAEMFIQGAADEIARMGVDLGVKNYVGPSTRPERLSRLREIIGQDSFLISPGVGAQGGDPGET
LRFADAIIVGRSIYLADNPAAAAAGIIESIKDLRIPEDPAANKARKEAELAAATAEQ
>3GY1A
MSLEPTIITDVLCYITKPDRHNLVVVKVETNKGIYGLGCATFQQRPKAVSLVVSEYLKPILIGRD
ANNIEDLWQMMMVNSYWRNGPILNNAISGVDMALWDIKGKLANMPLYQLFGGKSRDAIAAYTHAV
ADNLEDLYTEIDEIRKKGYQHIRCQLGFYGGNSSEFHTTDNPTQGSYFDQDEYMRTTVSMFSSLR
EKYGYKFHILHDVHERLFPNQAVQFAKDVEKYKPYFIEDILPPDQNEWLGQIRSQTSTPLATGEL
FNNPMEWKSLIANRQVDFIRCHVSQIGGITPALKLGSLCAAFGVRIAWHTPSDITPIGVAVNIHL
NINLHNAAIQENIEINDNTRCVFSGIPEAKNGFFYPIESPGIGVDIDENEIIKYPVEYRPHEWTQ
SRIPDGTIVTEGHHHHHH
>1XMZA

MRGSHHHHHHGSASFLKKTMPFKTTIEGTVNGHYFKCTGKGEGNPFEGTQEMKIEVIEGGPLPFA
FHILSTSCXSKTFIKYVSGIPDYFKQSFPEGFTWERTTTYEDGGFLTAHQDTSLDGDCLVYKVKI
LGNNFPADGPVMQNKAGRWEPGTEIVYEVDGVLRGQSLMALKCPGGRHLTCHLHTTYRSKKPASA
LKMPGFHFEDHRIEIMEEVEKGKCYKQYEAAVGRYCDAAPSKLGHN
>2P35A
QGHMAWSAQQYLKFEDERTRPARDLLAQVPLERVLNGYDLGCGPGNSTELLTDRYGVNVITGIDS
DDDMLEKAADRLPNTNFGKADLATWKPAQKADLLYANAVFQWVPDHLAVLSQLMDQLESGGVLAV
QMPDNLQEPTHIAMHETADGGPWKDAFSGGGLRRKPLPPPSDYFNALSPKSSRVDVWHTVYNHPM
KDADSIVEWVKGTGLRPYLAAAGEENREAFLADYTRRIAAAYPPMADGRLLLRFPRLFVVAVKK
>2P3JA
VLDGPYQPTTFNPPVDYWMLLAPTAAGVVVEGTNNTDAWLATILVEPNVTSETRSYTLFGTQEQI
TIANASQTQWKFIDVVKTTQNGSYSQYGPLQSTPKLYAVMKHNGKIYTYNGETPNVTTKYYSTTN
YDSVNMTAFCDFYIIPREEESTCTEYINNGL
>2OFXA
QGHMATNVTYQAHHVSRNKRGQVVGTRGGFRGCTVWLTGLSGAGKTTVSMALEEYLVCHGIPCYT
LDGDNIRQGLNKNLGFSPEDREENVRRIAEVAKLFADAGLVCITSFISPYTQDRNNARQIHEGAS
LPFFEVFVDAPLHVCEQRDVKGLYKKARAGEIKGFTGIDSEYEKPEAPELVLKTDSCDVNDCVQQ
VVELLQERDIVP
>2QSIA
GHMSGSLARAAARPNAPTLVDEATVDDFIAHSGKIVVLFFRGDAVRFPEAADLAVVLPELINAFP
GRLVAAEVAAEAERGLMARFGVAVCPSLAVVQPERTLGVIAKIQDWSSYLAQIGAMLAEVDQPGE
AELQSGS
>2J9FA
SSLDDKPQFPGASAEFIDKLEFIQPNVISGIPIYRVMDRQGQIINPSEDPHLPKEKVLKLYKSMT
LLNTMDRILYESQRQGRISFYMTNYGEEGTHVGSAAALDNTDLVFGQYREAGVLMYRDYPLELFM
AQCYGNISDLGKGRQMPVHYGCKERHFVTISSPLATQIPQAVGAAYAAKRANANRVVICYFGEGA
ASEGDAHAGFNFAATLECPIIFFCRNNGYAISTPTSEQYRGDGIAARGPGYGIMSIRVDGNDVFA
VYNATKEARRRAVAENQPFLIEAMTYRIGHHSTSDDSSAYRPVDEVNYWDKQDHPISRLRHYLLS
QGWWDEEQEKAWRKQSRRKVMEAFEQAERKPKPNPNLLFSDVYQEMPAQLRKQQESLARHLQTYG
EHYPLDHFDK
>3BB0A
MGSVTPIPLPKIDEPEEYNTNYILFWNHVGLELNRVTHTVGGPLTGPPLSARALGMLHLAIHDAY
FSICPPTDFTTFLSPDTENAAYRLPSPNGANDARQAVAGAALKMLSSLYMKPVEQPNPNPGANIS
DNAYAQLGLVLDRSVLEAPGGVDRESASFMFGEDVADVFFALLNDPRGASQEGYHPTPGRYKFDD
EPTHPVVLIPVDPNNPNGPKMPFRQYHAPFYGKTTKRFATQSEHFLADPPGLRSNADETAEYDDA
VRVAIAMGGAQALNSTKRSPWQTAQGLYWAYDGSNLIGTPPRFYNQIVRRIAVTYKKEEDLANSE
VNNADFARLFALVDVACTDAGIFSWKEKWEFEFWRPLSGVRDDGRPDHGDPFWLTLGAPATNTND
IPFKPPFPAYPSGHATFGGAVFQMVRRYYNGRVGTWKDDEPDNIAIDMMISEELNGVNRDLRQPY
DPTAPIEDQPGIVRTRIVRHFDSAWELMFENAISRIFLGVHWRFDAAAARDILIPTTTKDVYAVD
NNGATVFQNVEDIRYTTRGTREDREGLFPIGGVPLGIEIADEIFNNGLKPTPPEIQPMPQETPVQ
KPVGQQPVKGMWEEEQAPVVKEAP
>3H9CA
TQVAKKILVTCALPYANGSIHLGHMLEHIQADVWVRYQRMRGHEVNFICADDAHGTPIMLKAQQL
GITPEQMIGEMSQEHQTDFAGFNISYDNYHSTHSEENRQLSELIYSRLKENGFIKNRTISQLYDP

EKGMFLPDRFVKGTCPKCKSPDQYGDNCEVCGATYSPTELIEPKSVVSGATPVMRDSEHFFFDLP
SFSEMLQAWTRSGALQEQVANKMQEWFESGLQQWDISRDAPYFGFEIPNAPGKYFYVWLDAPIGY
MGSFKNLCDKRGDSVSFDEYWKKDSTAELYHFIGKDIVYFHSLFWPAMLEGSNFRKPSNLFVHGY
VTVNGAKMSKSRGTFIKASTWLNHFDADSLRYYYTAKLSSRIDDIDLNLEDFVQRVNADIVNKVV
NLASRNAGFINKRFDGVLASELADPQLYKTFTDAAEVIGEAWESREFGKAVREIMALADLANRYV
DEQAPWVVAKQEGRDADLQAICSMGINLFRVLMTYLKPVLPKLTERAEAFLNTELTWDGIQQPLL
GHKVNPFKALYNRIDMRQVEALVEASK
>2FHLA
ARKCSLTGKWTNNLGSIMTIRAVNSRGEFTGTYLTAVADNPGNITLSPLLGIQHKRASQPTFGFT
VHWNFSESTTVFTGQCFIDRNGKEVLKTMWLLRSSVNDISYDWKATRVGYNNFTRLS
>2PRVA
GMIYSKVENFINENKQNAIFTEGASHENIGRIEENLQCDLPNSYKWFLEKYGAGGLFGVLVLGYN
FDHASVVNRTNEYKEHYGLTDGLVVIEDVDYFAYCLDTNKMKDGECPVVEWDRVIGYQDTVADSF
IEFFYNKIQEAKDDWDEDEDWDD
>1JLJA
MATEGMILTNHDHQIRVGVLTVSDSCFRNLAEDRSGINLKDLVQDPSLLGGTISAYKIVPDEIEE
IKETLIDWCDEKELNLILTTGGTGFAPRDVTPEATKEVIEREAPGMALAMLMGSLNVTPLGMLSR
PVCGIRGKTLIINLPGSKKGSQECFQFILPALPHAIDLLRDAIVKVKEVHDRSHHHHHH
>1XFIA
SESDSEMVPFPQLPMPIENNYRACTIPYRFPSDDPKKATPNEISWINVFANSIPSFKKRAESDIT
VPDAPARAEKFAERYAGILEDLKKDPESHGGPPDGILLCRLREQVLRELGFRDIFKKVKDEENAK
AISLFPQVVSLSDAIEDDGKRLENLVRGIFAGNIFDLGSAQLAEVFSRDGMSFLASCQNLVPRPW
VIDDLENFQAKWINKSWKKAVIFVDNSGADIILGILPFARELLRRGAQVVLAANELPSINDITCT
ELTEILSQLKDENGQLLGVDTSKLLIANSGNDLPVIDLSRVSQELAYLSSDADLVIVEGMGRGIE
TNLYAQFKCDSLKIGMVKHLEVAEFLGGRLYDCVFKFNEVQS
>1KZ1A
MFSGIKGPNPSDLKGPELRILIVHARGNLQAIEPLVKGAVETMIEKHDVKLENIDIESVPGSWEL
PQGIRASIARNTYDAVIGIGVLIKGSTMHFEYISEAVVHGLMRVGLDSGVPVILGLLTVLNEEQA
LYRAGLNGGHNHGNDWGSAAVEMGLKALY
>2WL5A
STPSIVIASAARTAVGSFNGAFANTPAHELGATVISAVLERAGVAAGEVNEVILGQVLPAGEGQN
PARQAAMKAGVPQEATAWGMNQLCGSGLRAVALGMQQIATGDASIIVAGGMESMSMAPHCAHLRG
GVKMGDFKMIDTMIKDGLTDAFYGYHMGTTAENVAKQWQLSRDEQDAFAVASQNKAEAAQKDGRF
KDEIVPFIVKGRKGDITVDADEYIRHGATLDSMAKLRPAFDKEGTVTAGNASGLNDGAAAALLMS
EAEASRRGIQPLGRIVSWATVGVDPKVMGTGPIPASRKALERAGWKIGDLDLVEANEAFAAQACA
VNKDLGWDPSIVNVNGGAIAIGNPIGASGARILNTLLFEMKRRGARKGLATLCIGGGMGVAMCIE
SL
>1E2XA
FADRMVIKAQSPAGFAEEYIIESIWNNRFPPGTILPAERELSELIGVTRTTLREVLQRLARDGWL
TIQHGKPTKVNNFWETSGLNILETLARLDHESVPQLIDNLLSVRTNISTIFIRTAFRQHPDKAQE
VLATANEVADHADAFAELDYNIFRGLAFASGNPIYGLILNGMKGLYTRIGRHYFANPEARSLALG
FYHKLSALCSEGAHDQVYETVRRYGHESGEIWHRMQKNLPGDLAIQGR
>2O7IA
MQVSLPREDTVYIGGALWGPATTWNLYAPQSTWGTDQFMYLPAFQYDLGRDAWIPVIAERYEFVD

DKTLRIYIRPEARWSDGVPITADDFVYALELTKELGIGPGGGWDTYIEYVKAVDTKVVEFKAKEE
NLNYFQFLSYSLGAQPMPKHVYERIRAQMNIKDWINDKPEEQVVSGPYKLYYYDPNIVVYQRVDD
WWGKDIFGLPRPKYLAHVIYKDNPSASLAFERGDIDWNGLFIPSVWELWEKKGLPVGTWYKKEPY
FIPDGVGFVYVNNTKPGLSDPAVRKAIAYAIPYNEMLKKAYFGYGSQAHPSMVIDLFEPYKQYID
YELAKKTFGTEDGRIPFDLDMANKILDEAGYKKGPDGVRVGPDGTKLGPYTISVPYGWTDWMMMC
EMIAKNLRSIGIDVKTEFPDFSVWADRMTKGTFDLIISWSVGPSFDHPFNIYRFVLDKRLSKPVG
EVTWAGDWERYDNDEVVELLDKAVSTLDPEVRKQAYFRIQQIIYRDMPSIPAFYTAHWYEYSTKY
WINWPSEDNPAWFRPSPWHADAWPTLFIISKKSDPQPVPSWLGTVDEGGIEIPTAKIFEDLQKAT
MHHHHHH

>1YU0A

MSTAVQFRGGTTAQHATFTGAAREITVDTDKNTVVVHDGATAGGFPLARHDLVKTAFIKADKSAV
AFTRTGNATASIKAGTIVEVNGKLVQFTADTAITMPALTAGTDYAIYVCDDGTVRADSNFSAPTG
YTSTTARKVGGFHYAPGSNAAAQAGGNTTAQINEYSLWDIKFRPAALDPRGMTLVAGAFWADIYL
LGVNHLTDGTSKYNVTIADGSASPKKSTKFGGDGSAAYSDGAWYNFAEVMTHHGKRLPNYNEFQA
LAFGTTEATSSGGTDVPTTGVNGTGATSAWNIFTSKWGVVQASGCLWTWGNEFGGVNGASEYTAN
TGGRGSVYAQPAAALFGGAWNGTSLSGSRAALWYSGPSFSFAFFGARGVCDHLILE

>1YUKA

QECTKFKVSSCRECIESGPGCTWCQKLNFTGPGDPDSIRCDTRPQLLMRGCAADDIMDPTSLAET
QEDHNGGQKQLSPQKVTLYLRPGQAAAFNVTFRRAKGY

>2QNDA

ASRFHEQFIVREDLMGLAIGTHGANIQQARKVPGVTAIDLDEDTCTFHIYGEDQDAVKKARSFLE
FAEDVIQVPRNLVGKVIGKNGKLIQEIVDKSGVVRVRIEAENEKNVPQEEGMVPFVFVGTKDSIA
NATVLLDYHLNYLK

>3EOFA

GMMDTVKNRRTIRKYQQKDITPDLLNDLLETSFRASTMGGMQLYSVVVTRDAEKKEILSPAHFNQ
PMVKEAPVVLTFCADFRRFCKYCQERNAVPGYGNLMSFLNAAMDTLLVAQTFCTLAEEAGLGICY
LGTTTYNPQMIIDALHLPELVFPITTVTVGYPAESPKQVDRLPIEGIIHEESYHDYTAEDINRLY
AYKESLPENKLFIEENQKETLPQVFTDVRYTKKDNEFMSENLLKVLRRQGFMD

>2GRRA

GSHMSGIALSRLAQERKAWRKDHPFGFVAVPTKNPDGTMNLMNWECAIPGKKGTPWEGGLFKLRM
LFKDDYPSSPPKCKFEPPLFHPNVYPSGTVCLSILEEDKDWRPAITIKQILLGIQELLNEPNIQS
PAQAEAYTIYCQNRVEYEKRVRAQAKKFAPS

>3EJVA

MGSDKIHHHHHHENLYFQGMTMADETIILNVLGQYTRAHDRRDPDAMAALFAPEATIEIVDAVGG
ASRSISRLEGRDAIRVAVRQMMAPHGYRAWSQNVVNAPIIVIEGDHAVLDAQFMVFSILAAEVPD
GGWPTGTFGAQGRIVPIEAGQYRLTLRTVADGWVISAMRIEHRLPMAFG

>3GWBA

ELDGKAPSHRNLNVQTWSTAEGAKVLFVEARELPMFDLRLIFAAGSSQDGNAPGVALLTNAMLNE
GVAGKDVGAIAQGFEGLGADFGNGAYKDMAVASLRSLSAVDKREPALKLFAEVVGKPTFPADSLA
RIKNQMLAGFEYQKQNPGKLASLELMKRLYGTHPYAHASDGDAKSIPPITLAQLKAFHAKAYAAG
NVVIALVGDLSRSDAEAIAAQVSAALPKGPALAKIEQPAEPKASIGHIEFPSSQTSLMLAQLGID
RDDPDYAAVSLGNQILGGGGFGTRLMSEVREKRGLTYGVYSGFTPMQARGPFMINLQTRAEMSEG
TLKLVQDVFAEYLKNGPTQKELDDAKRELAGSFPLSTASNADIVGQLGAMGFYNLPLSYLEDFMR
QSQELTVEQVKAAMNKHLNVDKMVIVSAGPTVAQKPLEHHHHHH

>1GWTA
MQLTPTFYDNSCPNVSNIVRDTIVNELRSDPRIAASILRLHFHDCFVNGCDASILLDNTTSFRTE
KDAFGNANSARGFPVIDRMKAAVESACPRTVSCADLLTIAAQQSVTLAGGPSWRVPLGRRDSLQA
FLDLANANLPAPFFTLPQLKDSFRNVGLNRSSDLVALSGGHTFGKNQCRFIMDRLYNFSNTGLPD
PTLNTTYLQTLRGLCPLNGNLSALVDMDLRTPTIFDNKYYVNLEEQKGLIQSDQELFSSPNATDT
IPLVRSFANSTQTFFNAFVEAMDRMGNITPLTGTQGQIRLNCRVVNSNS
>1E39A
ADNLAEFHVQNQECDSCHTPDGELSNDSLTYENTQCVSCHGTLAEVAETTKHEHYNAHASHFPGE
VACTSCHSAHEKSMVYCDSCHSFDFNMPYAKKWLRDEPTIAELAKDKSERQAALASAPHDTVDVV
VVGSGGAGFSAAISATDSGAKVILIEKEPVIGGNAKLAAGGMNAAWTDQQKAKKITDSPELMFED
TMKGGQNINDPALVKVLSSHSKDSVDWMTAMGADLTDVGMMGGASVNRAHRPTGGAGVGAHVVQV
LYDNAVKRNIDLRMNTRGIEVLKDDKGTVKGILVKGMYKGYYWVKADAVILATGGFAKNNERVAK
LDPSLKGFISTNQPGAVGDGLDVAENAGGALKDMQYIQAAPTLSVKGGVMVTEAVRGNGAILVNR
EGKRFVNEITTRDKASAAILAQTGKSAYLIFDDSVRKSLSKIDKYIGLGVAPTADSLVKLGKMEG
IDGKALTETVARYNSLVSSGKDTDFERPNLPRALNEGNYYAIEVTPGVHHTMGGVMIDTKAEVMN
AKKQVIPGLYGAGEVTGGVHGANRLGGNAISDIITFGRLAGEEAAKYSKKN
>3E3UA
MAVVPIRIVGDPVLHTATTPVTVAADGSLPADLAQLIATMYDTMDAANGVGLAANQIGCSLRLFV
YDCAADRAMTARRRGVVINPVLETSEIPETMPDPDTDDEGCLSVPGESFPTGRAKWARVTGLDAD
GSPVSIEGTGLFARMLQHETGHLDGFLYLDRLIGRYARNAKRAVKSHGWGVPGLSWLPGEDPDPF
GH
>1E30A
GTLDTTWKEATLPQVKAMLEKDTGKVSGDTVTYSGKTVHVVAAAVLPGFPFPSFEVHDKKNPTLE
IPAGATVDVTFINTNKGFGHSFDITKKGPPYAVMPVIDPIVAGTGFSPVPKDGKFGYTNFTWHPT
AGTYYYVCQIPGHAATGQFGKIVVK
>3K2CA
MAHHHHHHMGTLEAQTQGPGSMAKEASGNVYFDVYANEESLGRIVMKLEDDIVPKTAKNFRTLCE
RPKGEGYKGSTFHRIIPGFMVQGGDYTAHNGTGGRSIYGEKFPDENFELKHTKEGILSMANCGAH
TNGSQFFITLGKTQWLDEKHVVFGEVVEGMDVVHKIAKYGSESGQVKKGYRIEIRDCGVLGSN
>2R1BA
GSSLRGGHAGTTYIFSKGGGQITYKWPPNDRPSTRADRLAIGFSTVQKEAVLVRVDSSSGLGDYL
ELHIHQGKIGVKFNVGTDDIAIEESNAIINDGKYHVVRFTRSGGNATLQVDSWPVIERYPAGNND
NERLAIARQRIPYRLGRVVDEWLLDKGRQLTIFNSQATIIIGGKEQGQPFQGQLSGLYYNGLKVL
NMAAENDANIAIVGNVRLVGEVPSS
>1I9YA
YDPIHEYVNHELRKRENEFSEHKNVKIFVASYNLNGCSATTKLENWLFPENTPLADIYVVGFQEI
VQLTPQQVISADPAKRREWESCVKRLLNGKCTSGPGYVQLRSGQLVGTALMIFCKESCLPSIKNV
EGTVKKTGLGGVSGNKGAVAIRFDYEDTGLCFITSHLAAGYTNYDERDHDYRTIASGLRFRRGRS
IFNHDYVVWFGDFNYRISLTYEEVVPCIAQGKLSYLFEYDQLNKQMLTGKVFPFFSELPITFPPT
YKFDIGTDIYDTSDKHRVPAWTDRILYRGELVPHSYQSVPLYYSDHRPIYATYEANIVKVDREKK
KILFEELYNQRKQEVRDASQTS
>1OAIA
PTLSPEQQEMLQAFSTQSGMNLEWSQKCLQDNNWDYTRSAQAFTHLKAKGEIPEVAFMK
>3C1JA

APAVADKADNAFMMICTALVLFMTIPGIALFYGGLIRGKNVLSMLTQVTVTFALVCILWVVYGYS
LAFGEGNNFFGNINWLMLKNIELTAVMGSIYQYIHVAFQGSAACITVGLIVGALAERIRFSAVLI
FVVVWLTLSYIPIAHMVWGGGLLASHGALDFAGGTVVHINAAIAGLVGAYLIGKRVGFGKEAFKP
HNLPMVFTGTAILYIGWFGANAGSAGTANEIAALAFVNTVVATAAAILGWIFGEWALRGKPSLLG
ACSGAIAGLVGVTPACGYIGVGGALIIGVVAGLAGLWGVTMLKRLLRVDDPCDVFGVHGVCGIVG
CIMTGIFAASSLGGVGFAEGVTMGHQLLVQLESIAITIVWSGVVAFIGYKLADLTVGLRVPEEQE
REGLDVNSHGENAYNADQAQQPAQADLEHHHHHH
>2C15A
MSFTPANRAYPYTRLRRNRRDDFSRRLVRENVLTVDDLILPVFVLDGVNQRESIPSMPGVERLSI
DQLLIEAEEWVALGIPALALFPVTPVEKKSLDAAEAYNPEGIAQRATRALRERFPELGIITDVAL
DPFTTHGQDGILDDDGYVLNDVSIDVLVRQALSHAEAGAQVVAPSDMMDGRIGAIREALESAGHT
NVRVMAYSAKYASAYYGPFRDAVGSASNLGKGNKATYQMDPANSDEALHEVAADLAEGADMVMVX
PGMPYLDIVRRVKDEFRAPTFVYQVSGEYAMHMGAIQNGWLAESVILESLTAFKRAGADGILTYF
AKQAAEQLRRGR
>1QL3A
ADPAAGEKVFGKCKACHKLDGNDGVGPHLNGVVGRTVAGVDGFNYSDPMKAHGGDWTPEALQEFL
TNPKAVVKGTKMAFAGLPKIEDRANLIAYLEGQQ
>1CHMA
QMPKTLRIRNGDKVRSTFSAQEYANRQARLRAHLAAENIDAAIFTSYHNINYYSDFLYCSFGRPY
ALVVTEDDVISISANIDGGQPWRRTVGTDNIVYTDWQRDNYFAAIQQALPKARRIGIEHDHLNLQ
NRDKLAARYPDAELVDVAAACMRMRMIKSAEEHVMIRHGARIADIGGAAVVEALGDQVPEYEVAL
HATQAMVRAIADTFEDVELMDTWTWFQSGINTDGAHNPVTTRKVNKGDILSLNCFPMIAGYYTAL
ERTLFLDHCSDDHLRLWQVNVEVHEAGLKLIKPGARCSDIARELNEIFLKHDVLQYRTFGYGHSF
GTLSHYYGREAGLELREDIDTVLEPGMVVSMEPMIMLPEGLPGAGGYREHDILIVNENGAENITK
FPYGPEKNIIR
>3CZVA
GSMSRLSWGYREHNGPIHWKEFFPIADGDQQSPIEIKTKEVKYDSSLRPLSIKYDPSSAKIISNS
GHSFNVDFDDTENKSVLRGGPLTGSYRLRQVHLHWGSADDHGSEHIVDGVSYAAELHVVHWNSDK
YPSFVEAAHEPDGLAVLGVFLQIGEPNSQLQKITDTLDSIKEKGKQTRFTNFDLLSLLPPSWDYW
TYPGSLTVPPLLESVTWIVLKQPINISSQQLAKFRSLLCTAEGEAAAFLVSNHRPPQPLKGRKVR
ASFH
>1CZYA
AMADLEQKVLEMEASTYDGVFIWKISDFPRKRQEAVAGRIPAIFSPAFYTSRYGYKMCLRIYLNG
DGTGRGTHLSLFFVVMKGPNDALLRWPFNQKVTLMLLDQNNREHVIDAFRPDVTSSSFQRPVNDM
NIASGCPLFCPVSKMEAKNSYVRDDAIFIKAIVDLTGL
>2V5IA
MVSVGDAAFRQEANKKFKYSVKLSDYSTLQDAVTDAVDGLLIDINYNFTDGESVDFXGKILTINC
KAKFIGDGALIFNNMGPGSVINQPFMESKTTPWVIFPWDADGKWITDAALVAATLKQSKIEGYQP
GVNDWVKFPGLEALLPQNVKDQHIAATLDIRSASRVEIRNAGGLMAAYLFRSCHHCKVIDSDSII
GGKDGIITFENLSGDWGLGNYVIGGRVHYGSGSGVQFLRNNGGESHNGGVIGVTSWRAGESGFKT
YQGSVGGGTARNYNLQFRDSVALSPVWDGFDLGSDPGMAPEPDRPGDLPVSEYPFHQLPNNHLVD
NILVMNSLGVGLGMDGSGGYVSNVTVQDCAGAGMLAHTYNRVFSNITVIDCNYLNFDSDQIIIIG
DCIVNGIRAAGIKPQPSNGLVISAPNSTISGLVGNVPPDKILVGNLLDPVLGQSRVIGFNSDTAE
LALRINKLSATLDSGALRSHLNGYAGSGSAWTELTALSGSTPNAVSLKVNRGDYKTTEIPISGTV

LPDEGVLDINTMSLYLDAGALWALIRLPDGSKTRMKLSV
>1YCLA
PSVESFELDHNAVVAPYVRHCGVHKVGTDGVVNKFDIRFCQPNKQAMKPDTIHTLEHLLAFTIRS
HAEKYDHFDIIDISPMGAQTGYYLVVSGEPTSAEIVDLLEDTMKEAVEITEIPAANEKQCGQAKL
HDLEGAKRLMRFWLSQDKEELLKVFG
>2G8FA
GSHMAKEEIIWESLSVDVGSQGNPGIVEYKGVDTKTGEVLFEREPIPIGTNNMGEFLAIVHGLRY
LKERNSRKPIYSDSQTAIKWVKDKKAKSTLVRNEETALIWKLVDEAEEWLNTHTYETPILKWQTD
KWGAIKADYGRK
>1G8KB
RTTLAYPATAVSVAKNLAANEPVSFTYPDTSSPCVAVKLGAPVPGGVGPDDDIVAYSVLCTHMGC
PTSYDSSSKTFSCPCHFTEFDAEKAGQMICGEATADLPRVLLRYDAASDALTAVGVDGLIYGRQA
NVI
>2NS9A
SLRLHAGVWGLKVRYEGSFEVSKTPEEVFEFLTDPKRFSRAFPGFKSVEVEDGSFTIELRLSLGP
LRGDARVRASFEDLEKPSKATVKGSGRGAGSTLDFTLRFAVEPSGGGSRVSWVFEGNVGGLAASM
GGRVLDSLARRMINDVISGVKRELGEA
>2V9MA
MQNITQSWFVQGMIKATTDAWLKGWDERNGGNLTLRLDDADIAPYHDNFHQQPRYIPLSQPMPLL
ANTPFIVTGSGKFFRNVQLDPMANLGIVKVDSDGAGYHILWGLFNEAVPTSELPAHFLSHCERIK
ATNGKDRVIMHCHATNLIALTYVLENDTAVFTRQLWEGSTECLVVFPDGVGILPWMVPGTDAIGQ
ATAQEMQKHSLVLWPFHGVFGSGPTLDETFGLIDTAEKSAQVLVKVYSMGGMKQTISREELIALG
KRFGVTPLASALAL
>2QIQA
AGFRKMAFPSGKVEGCMVQVTCGTTTLNGLWLDDTVYCPRHVICTAEDMLNPNYEDLLIRKSNHS
FLVQAGNVQLRVIGHSMQNCLLRLKVDTSNPKTPKYKFVRIQPGQTFSVLACYNGSPSGVYQCAM
RPNHTIKGSFLNGSCGSVGFNIDYDCVSFCYMHHMELPTGVHAGTDLEGKFYGPFVDRQTAQAAG
TDTTITLNVLAWLYAAVINGDRWFLNRFTTTLNDFNLVAMKYNYEPLTQDHVDILGPLSAQTGIA
VLDMCAALKELLQNGMNGRTILGSTILEDEFTPFDVVRQCS
>2Z25A
TAPSQVLKIRRPDDWHLHLRDGDMLKTVVPYTSEIYGRAIVMPNLAPPVTTVEAAVAYRQRILDA
VPAGHDFTPLMTCYLTDSLDPNELERGFNEGVFTAAKLYPANATVNSSHGVTSVDAIMPVLERME
KIGMPLLVHGEVTHADIDIFDREARFIESVMEPLRQRLTALKVVFEHITTKDAADYVRDGNERLA
ATITPQHLMFNRNHMLVGGVRPHLYCLPILKRNIHQQALRELVASGFNRVFLGTDSAPHARHRKE
SSCGCAGCFNAPTALGSYATVFEEMNALQHFEAFCSVNGPQFYGLPVNDTFIELVREEQQVAESI
ALTDDTLVPFLAGETVRWSVKQ
>3NZNA
SNAVNLFGQKDRGNHVSGVDRGKVIMYGLSTCVWCKKTKKLLTDLGVDFDYVYVDRLEGKEEEEA
VEEVRRFNPSVSFPTTIINDEKAIVGFKEKEIRESLGF
>1YPQA
RVANCSAPCPQDWIWHGENCYLFSSGSFNWEKSQEKCLSLDAKLLKINSTADLDFIQQAISYSSF
PFWMGLSRRNPSYPWLWEDGSPLMPHLFRVRGAVSQTYPSGTCAYIQRGAVYAENCILAAFSICQ
KKANL
>3ENUA

TIEVPVLTFVPVQVSAELENRGCWVKFFDKKNFQGDSLFLSGPATLPRLIGPFGYDWENKVRSVK
VGPRANLTIFDNHNYRDEDKFLDAGANVANLSKEMGFFDNFRSMVLNCI
>3APAA
GSARSSSYSGEYGSGGGKRFSHSGNQLDGPITALRVRVNTYYIVGLQVRYGKVWSDYVGGRNGDL
EEIFLHPGESVIQVSGKYKWYLKKLVFVTDKGRYLSFGKDSGTSFNAVPLHPNTVLRFISGRSGS
LIDAIGLHWDV
>2Q5XA
GIILTKVGYYTIPSMDDLAKITNEKGECIVSDFTIGRKGYGSIYFEGDVNLTNLNLDDIVHIRRK
EVVVYLDDNQKPPVGEGLNRKAEVTLDGVWPTDKTSRCLIKSPDRLADINYEGRLEAVSRKQGAQ
FKEYRPETGSWVFKVSHFAKYGLQD
>2Q66A
KVFGITGPVSTVGATAAENKLNDSLIQELKKEGSFETEQETANRVQVLKILQELAQRFVYEVSKK
KNMSDGMARDAGGKIFTYGSYRLGVHGPGSDIDTLVVVPKHVTREDFFTVFDSLLRERKELDEIA
PVPDAFVPIIKIKFSGISIALICARLDQPQVPLSLTLSDKNLLRNLDEKDLRALNGTRVTDEILE
LVPKPNVFRIALRAIKLWAQRRAVYANIFGFPGGVAWAMLVARICQLYPNACSAVILNRFFIILS
EWNWPQPVILKPIEDGPLQVRVWNPKIYAQDRSHRMPVITPAYPSMCATHNITESTKKVILQEFV
RGVQITNDIFSNKKSWANLFEKNDFFFRYKFYLEITAYTRGSDEQHLKWSGLVESKVRLLVMKLE
VLAGIKIAHPFTKPFESSYCCPTEDDYEMIQDKYGSHKTETALNALKLVTDENKEEESIKDAPKA
YLSTMYIGLDFNIENKKEKVDIHIPCTEFVNLCRSFNEDYGDHKVFNLALRFVKGYDLPDEVFDE
NEKRP
>1DQEA
SQEVMKNLSLNFGKALDECKKEMTLTDAINEDFYNFWKEGYEIKNRETGCAIMCLSTKLNMLDPE
GNLHHGNAMEFAKKHGADETMAQQLIDIVHGCEKSTPANDDKCIWTLGVATCFKAEIHKLNWAPS
MDVAVGE
>1PC5A
MAFVVTDNCIKCKYTDCVEVCPVDCFYEGPNFLVIHPDECIDCALCEPECGAQAIFSEDEVPEDM
QEFIQLNAELAEVWPNITEKKDPLPDAEDWDGVKGKLQHLER
>2FW6A
MMSETAPLPSASSALEDKAASAPVVGIIMGSQSDWETMRHADALLTELEIPHETLIVSANRTPDR
LADYARTAAERGLNVIIAGAGGAAHLPGMCAAWTRLPVLGVPVESRALKGMDSLLSIVQMPGGVP
VGTLAIGASGAKNAALLAASILALYNPALAARLETWRALQTASVPNSPITEDK
>2QQRA
GHMQSITAGQKVISKHKNGRFYQCEVVRLTTETFYEVNFDDGSFSDNLYPEDIVSQDCLQFGPPA
EGEVVQVRWTDGQVYGAKFVASHPIQMYQVEFEDGSQLVVKRDDVYTLDEELP
>1BF6A
SFDPTGYTLAHEHLHIDLSGFKNNVDCRLDQYAFICQEMNDLMTRGVRNVIEMTNRYMGRNAQFM
LDVMRETGINVVACTGYYQDAFFPEHVATRSVQELAQEMVDEIEQGIDGTELKAGIIAEIGTSEG
KITPLEEKVFIAAALAHNQTGRPISTHTSFSTMGLEQLALLQAHGVDLSRVTVGHCDLKDNLDNI
LKMIDLGAYVQFDTIGKNSYYPDEKRIAMLHALRDRGLLNRVMLSMDITRRSHLKANGGYGYDYL
LTTFIPQLRQSGFSQADVDVMLRENPSQFFQ
>3AHYA
MHHHHHHMLPKDFQWGFATAAYQIEGAVDQDGRGPSIWDTFCAQPGKIADGSSGVTACDSYNRTA
EDIALLKSLGAKSYRFSISWSRIIPEGGRGDAVNQAGIDHYVKFVDDLLDAGITPFITLFHWDLP
EGLHQRYGGLLNRTEFPLDFENYARVMFRALPKVRNWITFNEPLCSAIPGYGSGTFAPGRQSTSE

PWTVGHNILVAHGRAVKAYRDDFKPASGDGQIGIVLNGDFTYPWDAADPADKEAAERRLEFFTAW
FADPIYLGDYPASMRKQLGDRLPTFTPEERALVHGSNDFYGMNHYTSNYIRHRSSPASADDTVGN
VDVLFTNKQGNCIGPETQSPWLRPCAAGFRDFLVWISKRYGYPPIYVTENGTSIKGESDLPKEKI
LEDDFRVKYYNEYIRAMVTAVELDGVNVKGYFAWSLMDNFEWADGYVTRFGVTYVDYENGQKRFP
KKSAKSLKPLFDELIAAA
>2AHEA
MALSMPLNGLKEEDKEPLIELFVKAGSDGESIGNCPFSQRLFMILWLKGVVFSVTTVDLKRKPAD
LQNLAPGTHPPFITFNSEVKTDVNKIEEFLEEVLCPPKYLKLSPKHPESNTAGMDIFAKFSAYIK
NSRPEANEALERGLLKTLQKLDEYLNSPLPDEIDENSMEDIKFSTRKFLDGNEMTLADCNLLPKL
HIVKVVAKKYRNFDIPKEMTGIWRYLTNAYSRDEFTNTCPSDKEVEIAYSDVAKRLPSKVPKGEF
QHTGGRY
>1OX3A
ADIVLNDLPFVDGPPAEGQSRISWIKNGEEILGADTQYGSEGSMNRPTVSVLRNVEVLDKNIGIL
KTSLETANSDIKTIQEAGYIPEAPRDGQAYVRKDGEWVLLSTFL
>1QZ0A
MRERPHTSGHHGAGEARATAPSTVSPYGPEARAELSSRLTTLRNTLAPATNDPRYLQACGGEKLN
RFRDIQCRRQTAVRADLNANYIQVGNTRTIACQYPLQSQLESHFRMLAENRTPVLAVLASSSEIA
NQRFGMPDYFRQSGTYGSITVESKMTQQVGLGDGIMADMYTLTIREAGQKTISVPVVHVGNWPDQ
TAVSSEVTKALASLVDQTAETKRNMYESKGSSAVADDSKLRPVIHCRAGVGRTAQLIGAMCMNDS
RNSQLSVEDMVSQMRVQRNGIMVQKDEQLDVLIKLAEGQGRPLLNS
>1LT4A
NGDRLYRADSRPPDEIKRSGGLMPRGHNEYFDRGTQMNINLYDHARGTQTGFVRYDDGYVSTKLS
LRSAHLAGQSILSGYSTYYIYVIATAPNMFNVNDVLGVYSPHPYEQEVSALGGIPYSQIYGWYRV
NFGVIDERLHRNREYRDRYYRNLNIAPAEDGYRLAGFPPDHQAWREEPWIHHAPQGCGNSSNSSR
TITRTITGDTCNEETQNLSTIYLREYQSKVKRQIFSDYQSEVDIYNRIRDEL
>1OCBA
YNGNPFEGVQLWANNYYRSEVHTLAIPQITDPALRAAASAVAEVPSFQWLDRNVTVDTLLVQTLS
EIREANQAGANPQYAAQIVVYDLPDRDCAAAASNGEWAIANNGVNNYKAYINRIREILISFSDVR
TILVIEPDSLANMVTNMNVPKCSGAASTYRELTIYALKQLDLPHVAMYMDAGHAGWLGWPANIQP
AAELFAKIYEDAGKPRAVRGLATNVANYNAWSVSSPPPYTSPNPNYDEKHYIEAFRPLLEARGFP
AQFIVDQGRSGKQPTGQKEWGHWCNAIGTGFGMRPTANTGHQYVDAFVWVKPGGECDGTSDTTAA
RYDYHCGLEDALKPAPEAGQWFNEYFIQLLRNANPPF
>3OCCA
MATPHINAEMGDFADVVLMPGDPLRAKFIAETFLQDVREVNNVRGMLGFTGTYKGRKISVMGHGM
GIPSCSIYAKELITDFGVKKIIRVGSCGAVRTDVKLRDVVIGMGACTDSKVNRMRFKDHDYAAIA
DFEMTRNAVDAAKAKGVNVRVGNLFSADLFYTPDPQMFDVMEKYGILGVEMEAAGIYGVAAEFGA
KALTICTVSDHIRTGEQTTAAERQTTFNDMIEIALESVLLGDNA
>1ZCJA
ASGQAKALQYAFFAEKSANKWSTPSGASWKTASAQPVSSVGVLGLGTMGRGIAISFARVGISVVA
VESDPKQLDAAKKIITFTLEKEASRAHQNGQASAKPKLRFSSSTKELSTVDLVVEAVFEDMNLKK
KVFAELSALCKPGAFLCTNTSALNVDDIASSTDRPQLVIGTHFFSPAHVMRLLEVIPSRYSSPTT
IATVMSLSKKIGKIGVVVGNCYGFVGNRMLAPYYNQGFFLLEEGSKPEDVDGVLEEFGFKMGPFR
VSDLAGLDVGWKIRKGQGLTGPSLPPGTPVRKRGNSRYSPLGDMLCEAGRFGQKTGKGWYQYDKP
LGRIHKPDPWLSTFLSQYREVHHIEQRTISKEEILERCLYSLINEAFRILEEGMAARPEHIDVIY

LHGYGWPRHKGGPMFYAASVGLPTVLEKLQKYYRQNPDIPQLEPSDYLRRLVAQGSPPLKEWQSL
AGPHGSKL
>1LVMA
GHHHHHHHGESLFKGPRDYNPISSTICHLTNESDGHTTSLYGIGFGPFIITNKHLFRRNNGTLLV
QSLHGVFKVKNTTTLQQHLIDGRDMIIIRMPKDFPPFPQKLKFREPQREERICLVTTNFQTKSMS
SMVSDTSCTFPSSDGIFWKHWIQTKDGQCGSPLVSTRDGFIVGIHSASNFTNTNNYFTSVPKNFM
ELLTNQEAQQWVSGWRLNADSVLWGGHKVFMDKP
>1E0WA
AESTLGAAAAQSGRYFGTAIASGRLSDSTYTSIAGREFNMVTAENEMKIDATEPQRGQFNFSSAD
RVYNWAVQNGKQVRGHTLAWHSQQPGWMQSLSGSALRQAMIDHINGVMAHYKGKIVQWDVVNEAF
ADGSSGARRDSNLQRSGNDWIEVAFRTARAADPSAKLCYNDYNVENWTWAKTQAMYNMVRDFKQR
GVPIDCVGFQSHFNSGSPYNSNFRTTLQNFAALGVDVAITELDIQGAPASTYANVTNDCLAVSRC
LGITVWGVRDSDSWRSEQTPLLFNNDGSKKAAYTAVLDALNGGDSSEPPADGG
>3EMHA
GPLGSPEFQSKPTPVKPNYALKFTLAGHTKAVSSVKFSPNGEWLASSSADKLIKIWGAYDGKFEK
TISGHKLGISDVAWSSDSNLLVSASDDKTLKIWDVSSGKCLKTLKGHSNYVFCCNFNPQSNLIVS
GSFDESVRIWDVKTGKCLKTLPAHSDPVSAVHFNRDGSLIVSSSYDGLCRIWDTASGQCLKTLID
DDNPPVSFVKFSPNGKYILAATLDNTLKLWDYSKGKCLKTYTGHKNEKYCIFANFSVTGGKWIVS
GSEDNLVYIWNLQTKEIVQKLQGHTDVVISTACHPTENIIASAALENDKTIKLWKSDC
>3G5JA
SNAMSVIKIEKALKLDKVIFVDVRTEGEYEEDHILNAINMPLFKNNEHNEVGTIYKMQGKHEAIQ
KGFDYVSYKLKDIYLQAAELALNYDNIVIYCARGGMRSGSIVNLLSSLGVNVYQLEGGYKAYRNF
VLEY
>3B7AA
MTMEQFLTSLDMIRSGCAPKFKLKTEDLDRLRVGDFNFPPSQDLMCYTKCVALMAGTVNKKGEFN
APKALAQLPHLVPPEMMEMSRKSVEACRDTHKQFKESCERVYQTAKCFSENADGQFMWP
>3B7CA
GMPTDDIVQLLKGQEEAWNRGDLDAYMQGYWQNEQLMLISNGKFRNGWDETLAAYKKNYPDKESL
GELKFTIKEIKMLSNYAAMVVGRWDLKRLKDTPTGVFTLLVEKIDDRWVITMDHSSD
>2AD6A
DADLDKQVNTAGAWPIATGGYYSQHNSPLAQINKSNVKNVKAAWSFSTGVLNGHEGAPLVIGDMM
YVHSAFPNNTYALNLNDPGKIVWQHKPKQDASTKAVMCCDVVDRGLAYGAGQIVKKQANGHLLAL
DAKTGKINWEVEVCDPKVGSTLTQAPFVAKDTVLMGCSGAELGVRGAVNAFDLKTGELKWRAFAT
GSDDSVRLAKDFNSANPHYGQFGLGTKTWEGDAWKIGGGTNWGWYAYDPKLNLFYYGSGNPAPWN
ETMRPGDNKWTMTIWGRDLDTGMAKWGYQKTPHDEWDFAGVNQMVLTDQPVNGKMTPLLSHIDRN
GILYTLNRENGNLIVAEKVDPAVNVFKKVDLKTGTPVRDPEFATRMDHKGTNICPSAMGFHNQGV
DSYDPESRTLYAGLNHICMDWEPFMLPYRAGQFFVGATLAMYPGPNGPTKKEMGQIRAFDLTTGK
AKWTKWEKFAAWGGTLYTKGGLVWYATLDGYLKALDNKDGKELWNFKMPSGGIGSPMTYSFKGKQ
YIGSMYGVGGWPGVGLVFDLTDPSAGLGAVGAFRELQNHTQMGGGLMVFSL
>1WSRA
AQEVLRRTPLYDFHLAHGGKMVAFAGWSLPVQYRDSHTDSHLHTRQHCSLFDVSHMLQTKILGSD
RVKLMESLVVGDIAELRPNQGTLSLFTNEAGGILDDLIVTNTSEGHLYVVSNAGCWEKDLALMQD
KVRELQNQGRDVGLEVLDNALLALQGPTAAQVLQAGVADDLRKLPFMTSAVMEVFGVSGCRVTRC
GYTGEDGVEISVPVAGAVHLATAILKNPEVKLAGLAARDSLRLEAGLCLYGNDIDEHTTPVEGSL

SWTLGKRRRAAMDFPGAKVIVPQLKGRVQRRRVGLMCEGAPMRAHSPILNMEGTKIGTVTSGCPS
PSLKKNVAMGYVPCEYSRPGTMLLVEVRRKQQMAVVSKMPFVPTNYYTLK
>1BCH1
AIEVKLANMEAEINTLKSKLELTNKLHAFSMGKKSGKKFFVTNHERMPFSKVKALCSELRGTVAI
PRNAEENKAIQEVAKTSAFLGITDEVTEGQFMYVTGGRLTYSNWKKDQPDDWYGHGLGGGEDCVH
IVDNGLWNDISCQASHTAVCEFPA
>1DXKA
SQKVEKTVIKNETGTISISQLNKNVWVHTELGSFNGEAVPSNGLVLNTSKGLVLVDSSWDDKLTK
ELIEMVEKKFQKRVTDVIITHAHADRIGGIKTLKERGIKAHSTALTAELAKKNGYEEPLGDLQTV
TNLKFGNMKVETFYPGKGHTEDNIVVWLPQYNILVGGSLVKSTSAKDLGNVADAYVNEWSTSIEN
VLKRYRNINAVVPGHGEVGDKGLLLHTLDLLK
>3H8TA
DEPNQPSTPEAVTKTVTIDASKYETWQYFSFSKGEVVNVTDYKNDLNWDMALHRYDVRLNCGESG
KGKGGAVFSGKTEMDQATTVPTDGYTVDVLGRITVKYEMGPDGHQMEYEEQGFSEVITGKKNAQG
FASGGWLEFSHGPAGPTYKLSKRVFFVRGADGNIAKVQFTDYQDAELKKGVITFTYTYPVK
>3M7OA
MNGVAAALLVWILTSPSSSDHGSENGWPKHTACNSGGLEVVYQSCDPLQDFGLSIDQCSKQIQSN
LNIRFGIILRQDIRKLFLDITLMAKGSSILNYSYPLCEEDQPKFSFCGRRKGEQIYYAGPVNNPG
LDVPQGEYQLLLELYNENRATVACANATVTSS
>3OYYA
MASMKTAQEFRAGQVANINGAPWVIQKAEFNKSGRNAAVVKMKLKNLLTGAGTETVFKADDKLEP
IILDRKEVTYSYFADPLYVFMDSEFNQYEIEKDDLEGVLTFIEDGMTDICEAVFYNDKVISVELP
TTIVRQIAYTEPAVRGDTSGKVMKTARLNNGAELQVSAFCEIGDSIEIDTRTGEYKSRVKA
>1FAZA
APADKPQVLASFTQTSASSQNAWLAANRNQSAWAAYEFDWSTDLCTQAPDNPFGFPFNTACARHD
FGYRNYKAAGSFDANKSRIDSAFYEDMKRVCTGYTGEKNTACNSTAWTYYQAVKIFG
>1ODZA
MRADVKPVTVKLVDSQATMETRSLFAFMQEQRRHSIMFGHQHETTQGLTITRTDGTQSDTFNAVG
DFAAVYGWDTLSIVAPKAEGDIVAQVKKAYARGGIITVSSHFDNPKTDTQKGVWPVGTSWDQTPA
VVDSLPGGAYNPVLNGYLDQVAEWANNLKDEQGRLIPVIFRLYHENTGSWFWWGDKQSTPEQYKQ
LFRYSVEYLRDVKGVRNFLYAYSPNNFWDVTEANYLERYPGDEWVDVLGFDTYGPVADNADWFRN
VVANAALVARMAEARGKIPVISGIGIRAPDIEAGLYDNQWYRKLISGLKADPDAREIAFLLVWRN
APQGVPGPNGTQVPHYWVPANRPENINNGTLEDFQAFYADEFTAFNRDIEQVYQRPTLIVK
>1PF3A
AERPTLPIPDLLTTDARNRIQLTIGAGQSTFGGKTATTWGYNGNLLGPAVKLQRGKAVTVDIYNQ
LTEETTLHWHGLEVPGEVDGGPQGIIPPGGKRSVTLNVDQPAATCWFHPHQHGKTGRQVAMGLAG
LVVIEDDEILKLMLPKQWGIDDVPVIVQDKKFSADGQIDYQLDVMTAAVGWFGDTLLTNGAIYPQ
HAAPRGWLRLRLLNGCNARSLNFATSDNRPLYVIASDGGLLPEPVKVSELPVLMGERFEVLVEVN
DNKPFDLVTLPVSQMGMAIAPFDKPHPVMRIQPIAISASGALPDTLSSLPALPSLEGLTVRKLQL
SMDPMLDMMGMQMLMEKYGDQAMAGMDHSQMMGHMGHGNMNHMNHGGKFDFHHANKINGQAFDMN
KPMFAAAKGQYERWVISGVGDMLLHPFHIHGTQFRILSENGKPPAAHRAGWKDTVKVEGNVSEVL
VKFNHDAPKEHAYMAHCHLLEHEDTGMMLGFTVSAWSHPNFEK
>1UWLA
MTDNNNYRDVEIRAPRGNKLTAKSWLTEAPLRMLMNNLDPQVAENPKELVVYGGIGRAARNWECY

DKIVETLTRLEDDETLLVQSGKPVGVFKTHSNAPRVLIANSNLVPHWANWEHFNELDAKGLAMYG
QMTAGSWIYIGSQGIVQGTYETFVEAGRQHYGGSLKGKWVLTAGLGGMGGAQPLAATLAGACSLN
IESQQSRIDFRLETRYVDEQATDLDDALVRIAKYTAEGKAISIALHGNAAEILPELVKRGVRPDM
VTDQTSAHDPLNGYLPAGWTWEQYRDRAQTEPAAVVKAAKQSMAVHVQAMLDFQKQGVPTFDYGN
NIRQMAKEEGVANAFDFPGFVPAYIRPLFCRGVGPFRWAALSGEAEDIYKTDAKVKELIPDDAHL
HRWLDMARERISFQGLPARICWVGLGLRAKLGLAFNEMVRSGELSAPVVIGRDHLDSGSVSSPNR
ETEAMRDGSDAVSDWPLLNALLNTAGGATWVSLHHGGGVGMGFSQHSGMVIVCDGTDEAAERIAR
VLTNDPGTGVMRHADAGYDIAIDCAKEQGLDLPMITG
>3MCXA
GDWLDLNTTSSVETGQAIVTLDDAQIALNGIYRLASGHSYYGDNYWYYGDCRAADVQARITKGDG
KRVSPYYEYNVLASDNLNIVLPWNTVYKVIRQTNNLIQKIESGSIQSSDTKELNRIKSEALVMRG
LSLFNLTRLFGMPYTNDKGASLGVPIETSPSDPTHKPSRSTVAQCYEQVVSDMSNALSGLRQETS
NGYINYWAAQALLSRVYLNMGEYQKAYDAATDVIKNNGGRYQLYSYEEYPNVWGQDFQSESLFEL
YITLSEPSGGTGGEGAPMVYANEATVDWNNLILSEDFLNLLNEDPKDVRHCLTKESVIENNTGLP
AAAMHEKVYLAKFPGKTGDDPKTNNICIIRLSEVYLNAAEAGLKKGTDIEEAQGYLNDIISRRTT
DTSQQVSTETFTLDRILKERRKELVGEGEVFYDYLRNGLAIERKGSWHLETLKASNAQKIEATDL
RIALPIPQSEIDANPNIQQNPR
>1Z82A
MGSDKIHHHHHHMEMRFFVLGAGSWGTVFAQMLHENGEEVILWARRKEIVDLINVSHTSPYVEES
KITVRATNDLEEIKKEDILVIAIPVQYIREHLLRLPVKPSMVLNLSKGIEIKTGKRVSEIVEEIL
GCPYAVLSGPSHAEEVAKKLPTAVTLAGENSKELQKRISTEYFRVYTCEDVVGVEIAGALKNVIA
IAAGILDGFGGWDNAKAALETRGIYEIARFGMFFGADQKTFMGLAGIGDLMVTCNSRYSRNRRFG
ELIARGFNPLKLLESSNQVVEGAFTVKAVMKIAKENKIDMPISEEVYRVVYEGKPPLQSMRDLMR
RSLKDEFWAS
>3GZRA
GGEGTDAIQALIQAYFTAWNTNAPERFAEIFWPDGSWVNVVGMHWRGRDQIVFAHTAFLKTIFKD
CKQELVTIEARTIAPGSALAVVTLIQDAYVTPDGRQMPRAHDRLTLLAVEREGVWRFIHGHNTIV
NPDAANNDPVLRMKPA
>2A33A
MEIKGESMQKSKFRRICVFCGSSQGKKSSYQDAAVDLGNELVSRNIDLVYGGGSIGLMGLVSQAV
HDGGRHVIGIIPKTLMPRELTGETVGEVRAVADMHQRKAEMAKHSDAFIALPGGYGTLEELLEVI
TWAQLGIHDKPVGLLNVDGYYNSLLSFIDKAVEEGFISPTAREIIVSAPTAKELVKKLEEYAPCH
ERVATKLCWEMERIGYSSEE
>1DHIA
MISLIAALAVDRVIGMENAMPWNLPASLAWFKRNTLDKPVIMGRHTWESIGRPLPGRKNIILSSQ
PGTDDRVTWVKSVDEAIAACGDVPEIMVIGGGRVYEQFLPKAQKLYLTHIDAEVEGDTHFPDYEP
DDWESVFSEFHDADAQNSHSYCFEILERR
>3A8GA
MSVTIDHTTENAAPAQAPVSDRAWALFRALDGKGLVPDGYVEGWKKTFEEDFSPRRGAELVARAW
TDPEFRQLLLTDGTAAVAQYGYLGPQGEYIVAVEDTPTLKNVIVCSLCACTAWPILGLPPTWYKS
FEYRARVVREPRKVLSEMGTEIASDIEIRVYDTTAETRYMVLPQRPAGTEGWSQEQLQEIVTKDC
LIGVAIPQVPTV
>1VDWA
MSVKTWRKIAIDIIRDFDHNIMPLFGNPKASETISISPSGDETKVVDKVAENIIISKFKDLGVNV

VSEEIGRIDQGSDYTVVVDPLDGSYNFINGIPFFAVSVAIFHEKDPIYAFIYEPIVERLYEGIPG
KGSYLNGEKIKVRELAEKPSISFYTKGKGTKIIDKVKRTRTLGAIALELAYLARGALDAVVDIRN
YLRPTDIAAGVVIAREAGAIVKDLDGKDVEITFSATEKVNIIAANNEELLETILRSIEK
>2QKFA
MDIKINDITLGNNSPFVLFGGINVLESLDSTLQTCAHYVEVTRKLGIPYIFKASFDKANRSSIHS
YRGVGLEEGLKIFEKVKAEFGIPVITDVHEPHQCQPVAEVCDVIQLPAFLARQTDLVVAMAKTGN
VVNIKKPQFLSPSQMKNIVEKFHEAGNGKLILCERGSSFGYDNLVVDMLGFGVMKQTCGNLPVIF
DVTHSLQTRDAGSAASGGRRAQALDLALAGMATRLAGLFLESHPDPKLAKCDGPSALPLHLLEDF
LIRIKALDDLIKSQPILTIE
>2ZU1A
GPAFEFAVAMMKRNSSTVKTEYGEFTMLGIYDRWAVLPRHAKPGPTILMNDQEVGVLDAKELVDK
DGTNLELTLLKLNRNEKFRDIRGFLAKEEVEVNEAVLAINTSKFPNMYIPVGQVTEYGFLNLGGT
PTKRMLMYNFPTRAGQAGGVLMSTGKVLGIHVGGNGHQGFSAALLKHYFNDEQ
>2GJPA
HHMGTNGTMMQYFEWHLPNDGQHWNRLRDDASNLRNRGITAIWIPPAWKGTSQNDVGYGAYDLYD
LGEFNQKGTVRTKYGTRSQLESAIHALKNNGVQVYGDVVMNHKGGADATENVLAVEVNPNNRNQE
ISGDYTIEAWTKFDFPGRGNTYSDFKWRWYHFDGVDWDQSRQFQNRIYKFRGDGKAWDWEVDSEN
GNYDYLMYADVDMDHPEVVNELRRWGEWYTNTLNLDGFRIDAVKHIKYSFTRDWLTHVRNATGKE
MFAVAEFWKNDLGALENYLNKTNWNHSVFDVPLHYNLYNASNSGGNYDMAKLLNGTVVQKHPMHA
VTFVDNHDSQPGESLESFVQEWFKPLAYALILTREQGYPSVFYGDYYGIPTHSVPAMKAKIDPIL
EARQNFAYGTQHDYFDHHNIIGWTREGNTTHPNSGLATIMSDGPGGEKWMYVGQNKAGQVWHDIT
GNKPGTVTINADGWANFSVNGGSVSIWVKR
>3B5NC
GSIKFTKQSSVASTRNTLKMAQDAERAGMNTLGMLGHQSEQLNNVEGNLDLMKVQNKVADEKVAE
LKKLQ
>1HQGA
MSSKPKPIEIIGAPFSKGQPRGGVEKGPAALRKAGLVEKLKETEYNVRDHGDLAFVDVPNDSPFQ
IVKNPRSVGKANEQLAAVVAETQKNGTISVVLGGDHSMAIGSISGHARVHPDLCVIWVDAHTDIN
TPLTTSSGNLCGQPVAFLLKELKGKFPDVPGFSWVTPCISAKDIVYIGLRDVDPGEHYIIKTLGI
KYFSMTEVDKLGIGKVMEETFSYLLGRKKRPIHLSFDVDGLDPVFTPATGTPVVGGLSYREGLYI
TEEIYKTGLLSGLDIMEVNPTLGKTPEEVTRTVNTAVALTLSCFGTKREGNHKPETDYLKPPK
>2OQYA
MKITDLELHAVGIPRHTGFVNKHVIVKIHTDEGLTGIGEMSDFSHLPLYSVDLHDLKQGLLSILL
GQNPFDLMKINKELTDNFPETMYYYEKGSFIRNGIDNALHDLCAKYLDISVSDFLGGRVKEKIKV
CYPIFRHRFSEEVESNLDVVRQKLEQGFDVFRLYVGKNLDADEEFLSRVKEEFGSRVRIKSYDFS
HLLNWKDAHRAIKRLTKYDLGLEMIESPAPRNDFDGLYQLRLKTDYPISEHVWSFKQQQEMIKKD
AIDIFNISPVFIGGLTSAKKAAYAAEVASKDVVLGTTQELSVGTAAMAHLGCSLTNINHTSDPTG
PELYVGDVVKNRVTYKDGYLYAPDRSVKGLGIELDESLLAKYQVPDLSWDNVTVHQLQDRTADTK
S
>2OQAA
DVSFSLSGSSSTSYSKFIGALRKALPSNGTVYNITLLLSSASGASRYTLMKLSNYDGKAITVAID
VTNVYIMGYLVNSTSYFFNESDAKLASQYVFAGSTIVTLPYSGNYEKLQTAAGKIREKIPLGFPA
LDSAITTLFHYDSTAAAAAFLVIIQTTAESSRFKYIEGQIIMRISKNGVPSLATISLENEWSALS
KQIQLAQTNNGTFKTPVVIMDAGGQRVEIGNVGSKVVTKNIQLLLN

>3QD5A
GPGSMAATPLPPLRLAIACDDAGVSYKEALKAHLSDNPLVSSITDVGVTSTTDKTAYPHVAIQAA
QLIKDGKVDRALMICGTGLGVAISANKVPGIRAVTAHDTFSVERAILSNDAQVLCFGQRVIGIEL
AKRLAGEWLTYRFDQKSASAQKVQAISDYEKKFVEVN
>3D1RA
GMRRELAIEFSRVTESAALAGYKWLGRGDKNTADGAAVNAMRIMLNQVNIDGTIVIGEGEIAEAP
MLYIGEKVGTGRGDAVDIAVDPIEGTRMTAMGQANALAVLAVGDKGCFLNAPDMYMEKLIVGPGA
KGTIDLNLPLADNLRNVAAALGKPLSELTVTILAKPRHDAVIAEMQQLGVRVFAIPDGDVAASIL
TCMPDSEVDVLYGIGGAPEGVVSAAVIRALDGDMNGRLLARHDVKGDNEENRRIGEQELARCKAM
GIEAGKVLRLGDMARSDNVIFSATGITKGDLLEGISRKGNIATTETLLIRGKSRTIRRIQSIHYL
DRKDPEMQVHIL
>1RWIB
RPSWSPTQASGQTVLPFTGIDFRLSPSGVAVDSAGNVYVTSEGMYGRVVKLATGSTGTTVLPFNG
LYQPQGLAVDGAGTVYVTDFNNRVVTLAAGSNNQTVLPFDGLNYPEGLAVDTQGAVYVADRGNNR
VVKLAAGSKTQTVLPFTGLNDPDGVAVDNSGNVYVTDTDNNRVVKLEAESNNQVVLPFTDITAPW
GIAVDEAGTVYVTEHNTNQVVKLLAGSTTSTVLPFTGLNTPLAVAVDSDRTVYVADRGNDRVVKL
TSLEHHHHHH
>3NXDC
GSHMGGVEVLEVKTGVDSITEVECFLTPEMGDPDEHLRGFSKSISISDTFESDSPNRDMLPCYSV
ARIPLPNLNEDLTCGNILMWEAVTLKTEVIGVTSLMNVHSNGQATHDNGAGKPVQGTSFHFFSVG
GEALELQGVLFNYRTKYPDGTIFPKNATVQSQVMNTEHKAYLDKNKAYPVECWVPDPTRNENTRY
FGTLTGGENVPPVLHITNTATTVLLDEFGVGPLCKGDNLYLSAVDVCGMFTNRSGSQQWRGLSRY
FKVQLRKRRVKN
>1TEJB
NSVNPCCDPQTCKPIEGKHCISGPCCENCYFLRSGTICQRARGDGNNDYCTGITPDCPRNRYNV
>3EWDA
MNILQEPIDFLKKEELKNIDLSQMSKKERYKIWKRIPKCELHCHLDLCFSADFFVSCIRKYNLQP
NLSDEEVLDYYLFAKGGKSLGEFVEKAIKVADIFHDYEVIEDLAKHAVFNKYKEGVVLMEFRYSP
TFVAFKYNLDIELIHQAIVKGIKEVVELLDHKIHVALMCIGTGHEAANIKASADFCLKHKADFVG
FDHGGHEVDLKEYKEIFDYVRESGVPLSVHAGEDVTLPNLNTLYSAIQVLKVERIGHGIRVAESQ
ELIDMVKEKNILLEVCPISNVLLKNAKSMDTHPIRQLYDAGVKVSVNSDDPGMFLTNINDDYEEL
YTHLNFTLEDFMKMNEWALEKSFMDSNIKDKIKNLYFKGEFEAYV
>1EW4A
MNDSEFHRLADQLWLTIEERLDDWDGDSDIDCEINGGVLTITFENGSKIIINRQEPLHQVWLATK
QGGYHFDLKGDEWICDRSGETFWDLLEQAATQQAGETVSFR
>1H6WA
LSYPNATESVYGLTRYSTNDEAIAGVNNESSITPAKFTVALNNVFETRVSTESSNGVIKISSLPQ
ALAGADDTTAMTPLKTQQLAVKLIAQIAPSKNAATESEQGVIQLATVAQARQGTLREGYAISPYT
FMNSTATEEYKGVIKLGTQSEVNSNNASVAVTGATLNGRGSTTSMRGVVKLTTTAGSQSGGDASS
ALAWNADVIHQRGGQTINGTLRINNTLTIASGGANITGTVNMTGGYIQGKRVVTQNEIDRTIPVG
AIMMWAADSLPSDAWRFCHGGTVSASDCPLYASRIGTRYGGSSSNPGLPDMR
>2CB9A
MARSQLSAAGEQHVIQLNQQGGKNLFCFPPISGFGIYFKDLALQLNHKAAVYGFHFIEEDSRIEQ
YVSRITEIQPEGPYVLLGYSAGGNLAFEVVQAMEQKGLEVSDFIIVDAYKKDQSITADTENDDSA

AYLPEAVRETVMQKKRCYQEYWAQLINEGRIKSNIHFIEAGIQTETSGAMVLQKWQDAAEEGYAE
YTGYGAHKDMLEGEFAEKNANIILNILDKINSDQKVLPNKHGSHHHHHH
>1GXJA
EKEMIERDMREYRGFSRAVRAVFEEKERFPGLVDVVSNLIEVDEKYSLAVSVLLGGTAQNIVVRN
VDTAKAIVEFLKQNEAGRVTILPLDLIDGSFNRISGLENERGFVGYAVDLVKFPSDLEVLGGFLF
GNSVVVETLDDAIRMKKKYRLNTRIATLDGELISGRGAITGGREERSSNVFERRIK
>1VLCA
MGSDKIHHHHHHMKIAVLPGDGIGPEVVREALKVLEVVEKKTGKTFEKVFGHIGGDAIDRFGEPL
PEETKKICLEADAIFLGSVGGPKWDDLPPEKRPEIGGLLALRKMLNLYANIRPIKVYRSLVHVSP
LKEKVIGSGVDLVTVRELSYGVYYGQPRGLDEEKGFDTMIYDRKTVERIARTAFEIAKNRRKKVT
SVDKANVLYSSMLWRKVVNEVAREYPDVELTHIYVDNAAMQLILKPSQFDVILTTNMFGDILSDE
SAALPGSLGLLPSASFGDKNLYEPAGGSAPDIAGKNIANPIAQILSLAMMLEHSFGMVEEARKIE
RAVELVIEEGYRTRDIAEDPEKAVSTSQMGDLICKKLEEIW
>2VLBA
GQMQQASTPTIGMIVPPAAGLVPADGARLYPDLPFIASGLGLGSVTPEGYDAVIESVVDHARRLQ
KQGAAVVSLMGTSLSFYRGAAFNAALTVAMREATGLPCTTMSTAVLNGLRALGVRRVALATAYID
DVNERLAAFLAEESLVPTGCRSLGITGVEAMARVDTATLVDLCVRAFEAAPDSDGILLSCGGLLT
LDAIPEVERRLGVPVVSSSPAGFWDAVRLAGGGAKARPGYGRLFDESGGSHHHHHH
>2VLPB
MESKRNKPGKATGKGKPVGDKWLDDAGKDSGAPIPDRIADKLRDKEFKSFDDFAKAVWEEVSKDP
ELSKNLNPSNKSSVSKGYSPFTPKNQQVGGRKVYELHHDKPISQGGEVYDMDNIRVTTPKRHIDI
HRGK
>2Y88A
MPLILLPAVNVVEGRAVRLVQGKAGSQTEYGSAVDAALGWQRDGAEWIHLVDLDAAFGRGSNHEL
LAEVVGKLDVQVELSGGIRDDESLAAALATGCARVNVGTAALENPQWCARVIGEHGDQVAVGLDV
QIIDGEHRLRGRGWETDGGDLWDVLERLDSEGCSRFVVTDITKDGTLGGPNLDLLAGVADRTDAP
VIASGGVSSLDDLRAIATLTHRGVEGAIVGKALYARRFTLPQALAAVRD
>3DV9A
SNAMFKEAINNYLHTHGYESIDLKAVLFDMDGVLFDSMPNHAESWHKIMKRFGFGLSREEAYMHE
GRTGASTINIVSRRERGHDATEEEIKAIYQAKTEEFNKCPKAERMPGALEVLTKIKSEGLTPMVV
TGSGQTSLLDRLNHNFPGIFQANLMVTAFDVKYGKPNPEPYLMALKKGGFKPNEALVIENAPLGV
QAGVAAGIFTIAVNTGPLHDNVLLNEGANLLFHSMPDFNKNWETLQSALKQD
>2QRJA
MGHHHHHHHHHHSSGHIEGRHMAAVTLHLRAETKPLEARAALTPTTVKKLIAKGFKIYVEDSPQS
TFNINEYRQAGAIIVPAGSWKTAPRDRIIIGLKEMPETDTFPLVHEHIQFAHCYKDQAGWQNVLM
RFIKGHGTLYDLEFLENDQGRRVAAFGFYAGFAGAALGVRDWAFKQTHSDDEDLPAVSPYPNEKA
LVKDVTKDYKEALATGARKPTVLIIGALGRCGSGAIDLLHKVGIPDANILKWDIKETSRGGPFDE
IPQADIFINCIYLSKPIAPFTNMEKLNNPNRRLRTVVDVSADTTNPHNPIPIYTVATVFNKPTVL
VPTTAGPKLSVISIDHLPSLLPREASEFFSHDLLPSLELLPQRKTAPVWVRAKKLFDRHCARVKR
SSRL
>2O6XA
NDDLWHQWKRMYNKEYNGADDQHRRNIWEKNVKHIQEHNLRHDLGLVTYTLGLNQFTDMTFEEFK
AKYLTEMSRASDILSHGVPYEANNRAVPDKIDWRESGYVTEVKDQGNCGSGWAFSTTGTMEGQYM
KNERTSISFSEQQLVDCSRPWGNNGCGGGLMENAYQYLKQFGLETESSYPYTAVEGQCRYNKQLG

VAKVTGFYTVHSGSEVELKNLVGAEGPAAVAVDVESDFMMYRSGIYQSQTCSPLRVNHAVLAVGY
GTQGGTDYWIVKNSWGLSWGERGYIRMVRNRGNMCGIASLASLPMVARFP
>1GQGA
DTSSLIVEDAPDHVRPYVIRHYSHARAVTVDTQLYRFYVTGPSSGYAFTLMGTNAPHSDALGVLP
HIHQKHYENFYCNKGSFQLWAQSGNETQQTRVLSSGDYGSVPRNVTHTFQIQDPDTEMTGVIVPG
GFEDLFYYLGTNATDTTHTPYIPSSSDSSSTTGPDSSTISTLQSFDVYAELSFTPRTDTVNGTAP
ANTVWHTGANALASTAGDPYFIANGWGPKYLNSQYGYQIVAPFVTATQAQDTNYTLSTISMSTTP
STVTVPTWSFPGACAFQVQEGRVVVQIGDYAATELGSGDVAFIPGGVEFKYYSEAYFSKVLFVSS
GSDGLDQNLVNGGEEWSSVSFPADW
>3MSUA
SNAMEVMLMSKYATLKYADKNIEIELPVYSPSLGNDCIDVSSLVKHGIFTYDPGFMSTAACESKI
TYIDGGKGVLLHRGYPIEEWTQKSNYRTLCYALIYGELPTDEQVKSFRQEIINKMPVCEHVKAAI
AAMPQHTHPMSSLIAGVNVLAAEHIHNGQKESQDEVAKNIVAKIATIAAMAYRHNHGKKFLEPKM
EYGYAENFLYMMFADDESYKPDELHIKAMDTIFMLHADHEQNASTSTVRLSGSTGNSPYAAIIAG
ITALWGPAHGGANEAVLKMLSEIGSTENIDKYIAKAKDKDDPFRLMGFGHRVYKNTDPRATAMKK
NCEEILAKLGHSDNPLLTVAKKLEEIALQDEFFIERKLFSNVDFYSGIILKAMGIPEDMFTAIFA
LARTSGWISQWIEMVNDPAQKIGRPRQLYTGATNRNF
>1KTBA
LENGLARTPPMGWLAWERFRCNVNCREDPRQCISEMLFMEMADRIAEDGWRELGYKYINIDDCWA
AKQRDAEGRLVPDPERFPRGIKALADYVHARGLKLGIYGDLGRLTCGGYPGTTLDRVEQDAQTFA
EWGVDMLKLDGCYSSGKEQAQGYPQMARALNATGRPIVYSCSWPAYQGGLPPKVNYTLLGEICNL
WRNYDDIQDSWDSVLSIVDWFFTNQDVLQPFAGPGHWNDPDMLIIGNFGLSYEQSRSQMALWTIM
AAPLLMSTDLRTISPSAKKILQNRLMIQINQDPLGIQGRRIIKEGSHIEVFLRPLSQAASALVFF
SRRTDMPFRYTTSLAKLGFPMGAAYEVQDVYSGKIISGLKTGDNFTVIINPSGVVMWYLCPKALL
IQQQAPGGPSRLPLL
>2WGVA
MGSITENTSWNKEFSAEAVNGVFVLCKSSSKSCATNDLARASKEYLPASTFKIPNAIIGLETGVI
KNEHQVFKWDGKPRAMKQWERDLTLRGAIQVSATPVFQQIAREVGEVRMQKYLKKFSYGNQNISG
GIDKFWLEGQLRISAVNQVEFLESLYLNKLSASKENQLIVKEALVTEAAPEYLVHSKTGFSGVGT
ESNPGVAWWVGWVEKETEVYFFAFNMDIDNESKLPLRKSIPTKIMESEGIIGG
>3GBXA
SNAMLKREMNIADYDAELWQAMEQEKVRQEEHIELIASENYTSPRVMQAQGSQLTNKYAEGYPGK
RYYGGCEYVDVVEQLAIDRAKELFGADYANVQPHSGSQANFAVYTALLQPGDTVLGMNLAQGGHL
THGSPVNFSGKLYNIVPYGIDESGKIDYDEMAKLAKEHKPKMIIGGFSAYSGVVDWAKMREIADS
IGAYLFVDMAHVAGLIAAGVYPNPVPHAHVVTTTTHKTLAGPRGGLILAKGGDEELYKKLNSAVF
PSAQGGPLMHVIAGKAVALKEAMEPEFKVYQQQVAKNAKAMVEVFLNRGYKVVSGGTENHLFLLD
LVDKNLTGKEADAALGRANITVNKNSVPNDPKSPFVTSGIRIGSPAVTRRGFKEAEVKELAGWMC
DVLDNINDEATIERVKAKVLDICARFPVYA
>1E6WA
AAAVRSVKGLVAVITGGASGLGLSTAKRLVGQGATAVLLDVPNSEGETEAKKLGGNCIFAPANVT
SEKEVQAALTLAKEKFGRIDVAVNCAGIAVAIKTYHEKKNQVHTLEDFQRVINVNLIGTFNVIRL
VAGVMGQNEPDQGGQRGVIINTASVAAFEGQVGQAAYSASKGGIVGMTLPIARDLAPIGIRVVTI
APGLFATPLLTTLPDKVRNFLASQVPFPSRLGDPAEYAHLVQMVIENPFLNGEVIRLDGAIRMQP
>1E6FA

MKSNEHDDCQVTNPSTGHLFDLSSLSGRAGFTAAYSEKGLVYMSICGENENCPPGVGACFGQTRI
SVGKANKRLRYVDQVLQLVYKDGSPCPSKSGLSYKSVISFVCRPEAGPTNRPMLISLDKQTCTLF
FSWHTPLACEQAT

>3A75B

TTHFTVADRWGNVVSYTTTIEQLFGTGIMVPDYGVILNNELTDFDAIPGGANEVQPNKRPLSSMT
PTILFKDDKPVLTVGSPGGATIISSVLQTILYHIEYGMELKAAVEEPRIYTNSMSSYRYEDGVPK
DVLSKLNGMGHKFGTSPVDIGNVQSISIDHENGTFKGVADSSRNGAAIGINLKRK

>3FIRA

MKKIITLFGACALAFSMANADVNLYGPGGPHTALKDIANKYSEKTGVKVNVNFGPQATWFEKAKK
DADILFGASDQSALAIASDFGKDFNVSKIKPLYFREAIILTQKGNPLKIKGLKDLANKKVRIVVP
EGAGESNTSGTGVWEDMIGRTQDIKTIQNFRNNIVAFVPNSGSARKLFAQDQADAWITWIDWSKS
NPDIGTAVAIEKDLVVYRTFNVIAKEGASKETQDFIAYLSSKEAKEIFKKYGWREH

>2IAPA

IPVIEPLFTKVTEDIPGAQGPVFDKNGDFYIVAPEVEVNGKPAGEILRIDLKTGKKTVICKPEVN
GYGGIPAGCQCDRDANQLFVADMRLGLLVVQTDGTFEEIAKKDSEGRRMQGCNDCAFDYEGNLWI
TAPAGEVAPADYTRSMQEKFGSIYCFTTDGQMIQVDTAFQFPNGIAVRHMNDGRPYQLIVAETPT
KKLWSYDIKGPAKIENKKVWGHIPGTHEGGADGMDFDEDNNLLVANWGSSHIEVFGPDGGQPKMR
IRCPFEKPSNLHFKPQTKTIFVTEHENNAVWKFEWQRNGKKQYCETLKFGIF

>1Q74A

MSETPRLLFVHAHPDDESLSNGATIAHYTSRGAQVHVVTCTLGEEGEVIGDRWAQLTADHADQLG
GYRIGELTAALRALGVSAPIYLGGAGRWRDSGMAGTDQRSQRRFVDADPRQTVGALVAIIRELRP
HVVVTYDPNGGYGHPDHVHTHTVTTAAVAAAGVGSGTADHPGDPWTVPKFYWTVLGLSALISGAR
ALVPDDLRPEWVLPRADEIAFGYSDDGIDAVVEADEQARAAKVAALAAHATQVVVGPTGRAAALS
NNLALPILADEHYVLAGGSAGARDERGWETDLLAGLGFTASGT

>2CWLA

MFLRIDRLQIELPMPKEQDPNAAAAVQALLGGRFGEMSTLMNYMYQSFNFRGKKALKPYYDLIAN
IATEELGHIELVAATINSLLAKNPGKDLEEGVDPESAPLGFAKDVRNAAHFIAGGANSLVMGAMG
EHWNGEYVFTSGNLILDLLHNFFLEVAARTHKLRVYEMTDNPVAREMIGYLLVRGGVHAAAYGKA
LESLTGVEMTKMLPIPKIDNSKIPEAKKYMDLGFHRNLYRFSPEDYRDLGLIWKGASPEDGTEVV
VVDGPPTGGPVFDAGHDAAEFAPEFHPGELYEIAKKLYEKAK

>3NK6A

SEFMTEPAIITNASDPAVQRIIDVTKHSRASIKTTLIEDTEPLMECIRAGVQFIEVYGSSGTPLD
PALLDLCRQREIPVRLIDVSIVNQLFKAERKAKVFGIARVPRPARLADIAERGGDVVVLDGVKIV
GNIGAIVRTSLALGAAGIVLVDSDLATIADRRLLRASRGYVFSLPVVLADREEAVSFLRDNDIAL
MVLDTDGDLGVKDLGDRADRMALVFGSEKGGPSGLFQEASAGTVSIPMLSSTESLNVSVSVGIAL
HERSARNFAVRRAAAQA

>1V3IA

ATSDSNMLLNYVPVYVMLPLGVVNVDNVFEDPDGLKEQLLQLRAAGVDGVMVDVWWGIIELKGPK
QYDWRAYRSLLQLVQECGLTLQAIMSFHQCGGNVGDIVNIPIPQWVLDIGESNHDIFYTNRSGTR
NKEYLTVGVDNEPIFHGRTAIEIYSDYMKSFRENMSDFLESGLIIDIEVGLGPAGELRYPSYPQS
QGWEFPGIGEFQCYDKYLKADFKAAVARAGHPEWELPDDAGKYNDVPESTGFFKSNGTYVTEKGK
FFLTWYSNKLLNHGDQILDEANKAFLGCKVKLAIKVSGIHWWYKVENHAAELTAGYYNLNDRDGY
RPIARMLSRHHAILNFTCLEMRDSEQPSDAKSGPQELVQQVLSGGWREDIRVAGQNALPRYDATA
YNQIILNARPQGVNNNGPPKLSMFGVTYLRLSDDLLQKSNFNIFKKFVLKMHADQDYCANPQKYN

HAITPLKPSAPKIPIEVLLEATKPTLPFPWLPETDMKVDG
>2W87A
ALLLQEAQAGFCRVDGTIDNNHTGFTGSGFANTNNAQGAAVVWAIDATSSGRRTLTIRYANGGTA
NRNGSLVINGGSNGNYTVSLPTTGAWTTWQTATIDVDLVQGNNIVQLSATTAEGLPNIDSLSVVG
GTVRAGNCG
>2W8TA
TEAAAQPHALPADAPDIAPERDLLSKFDGLIAERQKLLDSGVTDPFAIVMEQVKSPTEAVIRGKD
TILLGTYNYMGMTFDPDVIAAGKEALEKFGSGTCGSRMLNGTFHDHMEVEQALRDFYGTTGAIVF
STGYMANLGIISTLAGKGEYVILDADSHASIYDGCQQGNAEIVRFRHNSVEDLDKRLGRLPKEPA
KLVVLEGVYSMLGDIAPLKEMVAVAKKHGAMVLVDEAHSMGFFGPNGRGVYEAQGLEGQIDFVVG
TFSKSVGTVGGFVVSNHPKFEAVRLACRPYIFTASLPPSVVATATTSIRKLMTAHEKRERLWSNA
RALHGGLKAMGFRLGTETCDSAIVAVMLEDQEQAAMMWQALLDGGLYVNMARPPATPAGTFLLRC
SICAEHTPAQIQTVLGMFQAAGRAVGVIGLEHHHHHH
>1Y0BA
SNAMEALKRKIEEEGVVLSDQVLKVDSFLNHQIDPLLMQRIGDEFASRFAKDGITKIVTIESSGI
APAVMTGLKLGVPVVFARKHKSLTLTDNLLTASVYSFTKQTESQIAVSGTHLSDQDHVLIIDDFL
ANGQAAHGLVSIVKQAGASIAGIGIVIEKSFQPGRDELVKLGYRVESLARIQSLEEGKVSFVQEV
HS
>3K3KA
MGSSHHHHHHSSGLVPRGSHMPSELTPEERSELKNSIAEFHTYQLDPGSCSSLHAQRIHAPPELV
WSIVRRFDKPQTYKHFIKSCSVEQNFEMRVGCTRDVIVISGLPANTSTERLDILDDERRVTGFSI
IGGEHRLTNYKSVTTVHRFEKENRIWTVVLESYVVDMPEGNSEDDTRMFADTVVKLNLQKLATVA
EAMARNSGDGSGSQVT
>3LIDA
MEKYQALLANNVENTAKEALHQLAYTGREYNNIQDQIETISDLLGHSQSLYDYLREPSKANLTIL
ENMWSSVARNQKLYKQIRFLDTSGTEKVRIKYDFKTSIAGPSLILRDKSAREYFKYAQSLDNEQI
SAWGIELERDKGELVYPLSPSLRILMPISVNDVRQGYLVLNVDIEYLSSLLNYSPVRDFHIELVK
HKGFYIASPDESRLYGDIIPERSQFNFSNMYPDIWPRVVSEQAGYSYSGEHLIAFSSIKFVSNEP
LHLIIDLSNEQLSKRATRDINDLIQESLEHHHHHH
>1PGTA
MPPYTVVYFPVRGRCAALRMLLADQGQSWKEEVVTVETWQEGSLKASCLYGQLPKFQDGDLTLYQ
SNTILRHLGRTLGLYGKDQQEAALVDMVNDGVEDLRCKYVSLIYTNYEAGKDDYVKALPGQLKPF
ETLLSQNQGGKTFIVGDQISFADYNLLDLLLIHEVLAPGCLDAFPLLSAYVGRLSARPKLKAFLA
SPEYVNLPINGNGKQ
>1ZLHB
NECVSKGFGCLPQSDCPQEARLSYGGCSTVCCDLSKLTGCKGKGGECNPLDRQCKELQAESASCG
KGQKCCVWLH
>3CIFA
MGSSHHHHHHSSGLVPRGSHMTATLGINGFGRIGRLVLRACMERNDITVVAINDPFMDVEYMAYL
LKYDSVHGNFNGTVEVSGKDLCINGKVVKVFQAKDPAEIPWGASGAQIVCESTGVFTTEEKASLH
LKGGAKKVIISAPPKDNVPMYVMGVNNTEYDPSKFNVISNASSTTNCLAPLAKIINDKFGIVEGL
MTTVHSLTANQLTVDGPSKGGKDWRAGRCAGNNIIPASTGAAKAVGKVIPALNGKLTGMAIRVPT
PDVSVVDLTCKLAKPASIEEIYQAVKEASNGPMKGIMGYTSDDVVSTDFIGCKYSSILDKNACIA
LNDSFVKLISWYDNESGYSNRLVDLAVYVASRGL

>2JINA
SMRVDYLVTEEEINLTRGPSGLGFNIVGGTDQQYVSNDSGIYVSRIKENGAAALDGRLQEGDKIL
SVNGQDLKNLLHQDAVDLFRNAGYAVSLRVQHRESSI
>2RK6A
GSHMASKRALVILAKGAEEMETVIPVDVMRRAGIKVTVAGLAGKDPVQCSRDVVICPDASLEDAK
KEGPYDVVVLPGGNLGAQNLSESAAVKEILKEQENRKGLIAAICAGPTALLAHEIGFGSKVTTHP
LAKDKMMNGGHYTYSENRVEKDGLILTSRGPGTSFKFALAIVEALNGKEVAAQVKAPLVLKD
>2GAIA
MAKKVKKYIVVESPAKAKTIKSILGNEYEVFASMGHIIDLPKSKFGVDLEKDFEPEFAVIKGKEK
VVEKLKDLAKKGELLIASDMDREGEAIAWHIARVTNTLGRKNRIVFSEITPRVIREAVKNPREID
MKKVRAQLARRILDRIVGYSLSPVLWRNFKSNLSAGRVQSATLKLVCDREREILRFVPKKYHRIT
VNFDGLTAEIDVKEKKFFDAETLKEIQSIDELVVEEKKVSVKKFAPPEPFKTSTLQQEAYSKLGF
SVSKTMMIAQQLYEGVETKDGHIAFITYMRTDSTRVSDYAKEEARNLITEVFGEEYVGSKRERRK
SNAKIQDAHEAIRPTNVFMTPEEAGKYLNSDQKKLYELIWKRFLASQMKPSQYEETRFVLRTKDG
KYRFKGTVLKKIFDGYEKVWKTERNTGEFPFEEGESVKPVVVKIEEQETKPKPRYTEGSLVKEME
RLGIGRPSTYASTIKLLLNRGYIKKIRGYLYPTIVGSVVMDYLEKKYSDVVSVSFTAEMEKDLDE
VEQGKKTDKIVLREFYESFSSVFDRNDRIVVDFPTNQKCSCGKEMRLSFGKYGFYLKCECGKTRS
VKNDEIAVIDDGKIFLGRKDSESGSPDGRSVEGKGNLSEKRRKGKKGS
>2GAKA
PEFFSVRHLELAGDDPYSNVNCTKILQGDPEEIQKVKLEILTVQFKKRPRWTPHDYINMTRDCAS
FIRTRKYIVEPLTKEEVGFPIAYSIVVHHKIEMLDRLLRAIYMPQNFYCIHVDRKAEESFLAAVQ
GIASCFDNVFVASQLESVVYASWTRVKADLNCMKDLYRMNANWKYLINLCGMDFPIKTNLEIVRK
LKCSTGENNLETEKMPPNKEERWKKRYAVVDGKLTNTGIVKAPPPLKTPLFSGSAYFVVTREYVG
YVLENENIQKLMEWAQDTYSPDEFLWATIQRIPEVPGSFPSSNKYDLSDMNAIARFVKWQYFEGD
VSNGAPYPPCSGVHVRSVCVFGAGDLSWMLRQHHLFANKFDMDVDPFAIQCLDEHLRRKALENLE
H
>2RLCA
CTGLALETKDGLHLFGRNMDIEYSFNQSIIFIPRNFKCVNKSNKKELTTKYAVLGMGTIFDDYPT
FADGMNEKGLGCAGLNFPVYVSYSKEDIEGKTNIPVYNFLLWVLANFSSVEEVKEALKNANIVDI
PISENIPNTTLHWMISDITGKSIVVEQTKEKLNVFDNNIGVLTNSPTFDWHVANLNQYVGLRYNQ
VPEFKLGDQSLTALGQGTGLVGLPGDFTPASRFIRVAFLRDAMIKNDKDSIDLIEFFHILNNVAM
VRGSTRTVEEKSDLTQYTSCMCLEKGIYYYNTYENNQINAIDMNKENLDGNEIKTYKYNKTLSIN
HVN
>3DR3A
SNAMLNTLIVGASGYAGAELVTYVNRHPHMNITALTVSAQSNDAGKLISDLHPQLKGIVELPLQP
MSDISEFSPGVDVVFLATAHEVSHDLAPQFLEAGCVVFDLSGAFRVNDATFYEKYYGFTHQYPEL
LEQAAYGLAEWCGNKLKEANLIAVPGCYPTAAQLALKPLIDADLLDLNQWPVINATSGVSGAGRK
AAISNSFCEVSLQPYGVFTHRHQPEIATHLGADVIFTPHLGNFPRGILETITCRLKSGVTQAQVA
QALQQAYAHKPLVRLYDKGVPALKNVVGLPFCDIGFAVQGEHLIIVATEDNLLKGAAAQAVQCAN
IRFGYAETQSLI
>2CN3A
ISSQAVTSVPYKWDNVVIGGGGGFMPGIVFNETEKDLIYARAAIGGAYRWDPSTETWIPLLDHFQ
MDEYSYYGVESIATDPVDPNRVYIVAGMYTNDWLPNMGAILRSTDRGETWEKTILPFKMGGNMPG
RSMGERLAIDPNDNRILYLGTRCGNGLWRSTDYGVTWSKVESFPNPGTYIYDPNFDYTKDIIGVV

WVVFDKSSSTPGNPTKTIYVGVADKNESIYRSTDGGVTWKAVPGQPKGLLPHHGVLASNGMLYIT
YGDTCGPYDGNGKGQVWKFNTRTGEWIDITPIPYSSSDNRFCFAGLAVDRQNPDIIMVTSMNAWW
PDEYIFRSTDGGATWKNIWEWGMYPERILHYEIDISAAPWLDWGTEKQLPEINPKLGWMIGDIEI
DPFNSDRMMYVTGATIYGCDNLTDWDRGGKVKIEVKATGIEECAVLDLVSPPEGAPLVSAVGDLV
GFVHDDLKVGPKKMHVPSYSSGTGIDYAELVPNFMALVAKADLYDVKKISFSYDGGRNWFQPPNE
APNSVGGGSVAVAADAKSVIWTPENASPAVTTDNGNSWKVCTNLGMGAVVASDRVNGKKFYAFYN
GKFYISTDGGLTFTDTKAPQLPKSVNKIKAVPGKEGHVWLAAREGGLWRSTDGGYTFEKLSNVDT
AHVVGFGKAAPGQDYMAIYITGKIDNVLGFFRSDDAGKTWVRINDDEHGYGAVDTAITGDPRVYG
RVYIATNGRGIVYGEPASDEPV
>1XA3A
MHHHHHHGSTSLYKKAGSETLYIQGDHLPMPKFGPLAGLRVVFSGIEIAGPFAGQMFAEWGAEVI
WIENVAWADTIRVQPNYPQLSRRNLHALSLNIFKDEGREAFLKLMETTDIFIEASKGPAFARRGI
TDEVLWQHNPKLVIAHLSGFGQYGTEEYTNLPAYNTIAQAFSGYLIQNGDVDQPMPAFPYTADYF
SGLTATTAALAALHKVRETGKGESIDIAMYEVMLRMGQYFMMDYFNGGEMCPRMSKGKDPYYAGC
GLYKCADGYIVMELVGITQIEECFKDIGLAHLLGTPEIPEGTQLIHRIECPYGPLVEEKLDAWLA
THTIAEVKERFAELNIACAKVLTVPELESNPQYVARESITQWQTMDGRTCKGPNIMPKFKNNPGQ
IWRGMPSHGMDTAAILKNIGYSENDIQELVSKGLAKVEDSTHHHHHH
>1GPIA
QQAGTNTAENHPQLQSQQCTTSGGCKPLSTKVVLDSNWRWVHSTSGYTNCYTGNEWDTSLCPDGK
TCAANCALDGADYSGTYGITSTGTALTLKFVTGSNVGSRVYLMADDTHYQLLKLLNQEFTFDVDM
SNLPCGLNGALYLSAMDADGGMSKYPGNKAGAKYGTGYCDSQCPKDIKFINGEANVGNWTETGSN
TGTGSYGTCCSEMDIWEANNDAAAFTPHPCTTTGQTRCSGDDCARNTGLCDGDGCDFNSFRMGDK
TFLGKGMTVDTSKPFTVVTQFLTNDNTSTGTLSEIRRIYIQNGKVIQNSVANIPGVDPVNSITDN
FCAQQKTAFGDTNWFAQKGGLKQMGEALGNGMVLALSIWDDHAANMLWLDSDYPTDKDPSAPGVA
RGTCATTSGVPSDVESQVPNSQVVFSNIKFGDIGSTFSGTS
>1CMNA
ASGAPSFPFSRASGPEPPAEFAKLRATNPVSQVKLFDGSLAWLVTKHKDVCFVATSEKLSKVRTR
QGFPELSASGKQAAKAKPTFVDMDPPEHMHQRSMVEPTFTPEAVKNLQPYIQRTVDDLLEQMKQK
GCANGPVDLVKEFALPVPSYIIYTLLGVPFNDLEYLTQQNAIRTNGSSTAREASAANQELLDYLA
ILVEQRLVEPKDDIISKLCTEQVKPGNIDKSDAVQIAFLLLVAGNATMVNMIALGVATLAQHPDQ
LAQLKANPSLAPQFVEELCRYHTAVALAIKRTAKEDVMIGDKLVRANEGIIASNQSANRDEEVFE
NPDEFNMNRKWPPQDPLGFGFGDHRCIAEHLAKAELTTVFSTLYQKFPDLKVAVPLGKINYTPLN
RDVGIVDLPVIF
>1LRIA
TACTASQQTAAYKTLVSILSDASFNQCSTDSGYSMLTAKALPTTAQYKLMCASTACNTMIKKIVT
LNPPNCDLTVPTSGLVLNVYSYANGFSNKCSSL
>2QECA
GMSPTVLPATQADFPKIVDVLVEAFANDPTFLRWIPQPDPGSAKLRALFELQIEKQYAVAGNIDV
ARDSEGEIVGVALWDRPDGNHSAKDQAAMLPRLVSIFGIKAAQVAWTDLSSARFHPKFPHWYLYT
VATSSSARGTGVGSALLNHGIARAGDEAIYLEATSTRAAQLYNRLGFVPLGYIPSDDDGTPELAM
WKPPAMPTV
>1VAJA
MVFKIKDEWGEFLVRLARRAIEEYLKTGKEIEPPKDTPPELWEKMGVFVTLNRYNVPPQTALRGC
IGFPTPIYPLVEATIKAAIYSAVDDPRFPPVKLEEMDNLVVEVSVLTPPELIEGPPEERPRKIKV

GRDGLIVEKGIYSGLLLPQVPVEWGWDEEEFLAETCWKAGLPPDCWLDEDTKVYKFTAEIFEEEY
PRGPIKRKPLVLEHHHHHH
>1D7EA
VAFGSEDIENTLAKMDDGQLDGLAFGAIQLDGDGNILQYNAAEGDITGRDPKQVIGKNFFKDVAP
CTDSPEFYGKFKEGVASGNLNTMFEYTFDYQMTPTKVKVHMKKALSGDSYWVFVKRV
>1YNVX
DVSGTVCLSALPPEATDTLNLIASDGPFPYSQDGVVFQNRESVLPTQSYGYYHEYTVITPGARTR
GTRRIITGEATQEDYYTGDHYATFSLIDKTC
>1XH9A
GNAAAAKKGSEQESVKEFLAKAKEDFLKKWENPAQNTAHLDQFERIKTLGTGSFGRVMLVKHMET
GNHYAMKILDKQKVVKLKEIEHTLNEKRILQAVNFPFLVKLEFSFKDNSNLYMVMEYAPGGEMFS
HLRRIGRFSEPHARFYAAQIVLTFEYLHSLDLIYRDLKPENLMIDQQGYIKVTDFGLAKRVKGRT
WTLCGTPEYLAPEIILSKGYNKAVDWWALGVLIYEMAAGYPPFFADQPIQIYEKIVSGKVRFPSH
FSSDLKDLLRNLLQVDLTKRFGNLKNGVNDIKNHKWFATTDWIAIYQRKVEAPFIPKFKGPGDTS
NFDDYEEEEIRVSINEKCGKEFSEF
>3OIRA
SNADGLEGMDDPDATSKKVVPLGVEIYEINGPFFFGVADRLKGVLDVIEETPKVFILRMRRVPVI
DATGMHALWEFQESCEKRGTILLLSGVSDRLYGALNRFGFIEALGEERVFDHIDKALAYAKLLVE
TAEER
>2V4CA
ADYDLKFGMNAGTSSNEYKAAEMFAKEVKEKSQGKIEISLYPSSQLGDDRAMLKQLKDGSLDFTF
AESARFQLFYPEAAVFALPYVISNYNVAQKALFDTEFGKDLIKKMDKDLGVTLLSQAYNGTRQTT
SNRAINSIADMKGLKLRVPNAATNLAYAKYVGASPTPMAFSEVYLALQTNAVDGQENPLAAVQAQ
KFYEVQKFLAMTNHILNDQLYLVSNETYKELPEDLQKVVKDAAENAAKYHTKLFVDGEKDLVTFF
EKQGVKITHPDLVPFKESMKPYYAEFVKQTGQKGESALKQIEAINPHHH
>1JNRA
MVYYPKKYELYKADEVPTEVVETDILIIGGGFSGCGAAYEAAYWAKLGGLKVTLVEKAAVERSGA
VAQGLSAINTYIDLTGRSERQNTLEDYVRYVTLDMMGLAREDLVADYARHVDGTVHLFEKWGLPI
WKTPDGKYVREGQWQIMIHGESYKPIIAEAAKMAVGEENIYERVFIFELLKDNNDPNAVAGAVGF
SVREPKFYVFKAKAVILATGGATLLFRPRSTGEAAGRTWYAIFDTGSGYYMGLKAGAMLTQFEHR
FIPFRFKDGYGPVGAWFLFFKCKAKNAYGEEYIKTRAAELEKYKPYGAAQPIPTPLRNHQVMLEI
MDGNQPIYMHTEEALAELAGGDKKKLKHIYEEAFEDFLDMTVSQALLWACQNIDPQEQPSEAAPA
EPYIMGSHSGEAGFWVCGPEDLMPEEYAKLFPLKYNRMTTVKGLFAIGDCAGANPHKFSSGSFTE
GRIAAKAAVRFILEQKPNPEIDDAVVEELKKKAYAPMERFMQYKDLSTADDVNPEYILPWQGLVR
LQKIMDEYAAGIATIYKTNEKMLQRALELLAFLKEDLEKLAARDLHELMRAWELVHRVWTAEAHV
RHMLFRKETRWPGYYYRTDYPELNDEEWKCFVCSKYDAEKDEWTFEKVPYVQVIEWSF
>2QM0A
SNAMNTTVEKQQIITSNTEQWKMYSKLEGKEYQIHISKPKQPAPDSGYPVIYVLDGNAFFQTFHE
AVKIQSVRAEKTGVSPAIIVGVGYPIEGAFSGEERCYDFTPSVISKDAPLKPDGKPWPKTGGAHN
FFTFIEEELKPQIEKNFEIDKGKQTLFGHXLGGLFALHILFTNLNAFQNYFISSPSIWWNNKSVL
EKEENLIIELNNAKFETGVFLTVGSLEREHMVVGANELSERLLQVNHDKLKFKFYEAEGENHASV
VPTSLSKGLRFISYV
>2QM6A
MGSSHHHHHHSSGLVPRGSHMASAASYPPIKNTKVGLALSSHPLASEIGQKVLEEGGNAIDAAVA

IGFALAVVHPAAGNIGGGGFAVIHLANGENVALDFREKAPLKATKNMFLDKQGNVVPKLSEDGYL
AAGVPGTVAGMEAMLKKYGTKKLSQLIDPAIKLAENGYAISQRQAETLKEARERFLKYSSSKKYF
FKKGHLDYQEGDLFVQKDLAKTLNQIKTLGAKGFYQGQVAELIEKDMKKNGGIITKEDLASYNVK
WRKPVVGSYRGYKIISMSPPSSGGTHLIQILNVMENADLSALGYGASKNIHIAAEAMRQAYADRS
VYMGDADFVSVPVDKLINKAYAKKIFDTIQPDTVTPSSQIKPGMGQLHEGSN
>3GHJA
MGSSHHHHHHSSGRENLYFQGVPMNIKGLFEVAVKVKNLEKSSQFYTEILGFEAGLLDSARRWNF
LWVSGRAGMVVLQEEKENWQQQHFSFRVEKSEIEPLKKALESKGVSVHGPVNQEWMQAVSLYFAD
PNGHALEFTAL
>2Z58B
NTIRVIVSVDKAKFNPHEVLGIGGHIVYQFKLIPAVVVDVPANAVGKLKKMPWVEKVEFDHQAVL
L
>1CUHA
LPTSNPAQELEARQLGRTTRDDLINGNSASCADVIFIYARGSTETGNLGTLGPSIASNLESAFGK
DGVWIQGVGGAYRATLGDNALPRGTSSAAIREMLGLFQQANTKCPDATLIAGGYSQGAALAAASI
EDLDSAIRDKIAGTVLFGYTKNLQNRGRIPNYPADRTKVFCNTGDLVCTGSLIVAAPHLAYGPDA
EGPAPEFLIEKVRAVRGSA
>1OJQA
AETKNFTDLVEATKWGNSLIKSAKYSSKDKMAIYNYTKNSSPINTPLRSANGDVNKLSENIQEQV
RQLDSTISKSVTPDSVYVYRLLNLDYLSSITGFTREDLHMLQQTNNGQYNEALVSKLNNLMNSRI
YRENGYSSTQLVSGAALAGRPIELKLELPKGTKAAYIDSKELTAYPGQQEVLLPRGTEYAVGSVK
LSDNKRKIIITAVVFKK
>1NIJA
MNPIAVTLLTGFLGAGKTTLLRHILNEQHGYKIAVIENEFGEVSVDDQLIGDRATQIKTLTNGCI
CCSRSNELEDALLDLLDNLDKGNIQFDRLVIECTGMADPGPIIQTFFSHEVLCQRYLLDGVIALV
DAVHADEQMNQFTIAQSQVGYADRILLTKTDVAGEAEKLHERLARINARAPVYTVTHGDIDLGLL
FNTNGFMLEENVVSTKPRFHFIADKQNDISSIVVELDYPVDISEVSRVMENLLLESADKLLRYKG
MLWIDGEPNRLLFQGVQRLYSADWDRPWGDEKPHSTMVFIGIQLPEEEIRAAFAGLRK
>2O2GA
GMDRTLTHQPQEYAVSVSVGEVKLKGNLVIPNGATGIVLFAHGSGSSRYSPRNRYVAEVLQQAGL
ATLLIDLLTQEEEEIDLRTRHLRFDIGLLASRLVGATDWLTHNPDTQHLKVGYFGASTGGGAALV
AAAERPETVQAVVSRGGRPDLAPSALPHVKAPTLLIVGGYDLPVIAMNEDALEQLQTSKRLVIIP
RASHLFEEPGALTAVAQLASEWFMHYLR
>1O2DA
MGSDKIHHHHHHVWEFYMPTDVFFGEKILEKRGNIIDLLGKRALVVTGKSSSKKNGSLDDLKKLL
DETEISYEIFDEVEENPSFDNVMKAVERYRNDSFDFVVGLGGGSPMDFAKAVAVLLKEKDLSVED
LYDREKVKHWLPVVEIPTTAGTGSEVTPYSILTDPEGNKRGCTLMFPVYAFLDPRYTYSMSDELT
LSTGVDALSHAVEGYLSRKSTPPSDALAIEAMKIIHRNLPKAIEGNREARKKMFVASCLAGMVIA
QTGTTLAHALGYPLTTEKGIKHGKATGMVLPFVMEVMKEEIPEKVDTVNHIFGGSLLKFLKELGL
YEKVAVSSEELEKWVEKGSRAKHLKNTPGTFTPEKIRNIYREALGV
>3MR1A
GPGSMIKIHTEKDFIKMRAAGKLAAETLDFITDHVKPNVTTNSLNDLCHNFITSHNAIPAPLNYK
GFPKSICTSINHVVCHGIPNDKPLKNGDIVNIDVTVILDGWYGDTSRMYYVGDVAIKPKRLIQVT
YDAMMKGIEVVRPGAKLGDIGYAIQSYAEKHNYSVVRDYTGHGIGRVFHDKPSILNYGRNGTGLT

LKEGMFFTVEPMINAGNYDTILSKLDGWTVTTRDKSLSAQFEHTIGVTKDGFEIFTL
>3NJCA
MGHHHHHHSHMKSKFEASIDNLKEIEMNAYAYELIREIVLPDMLGQDYSSMMYWAGKHLARKFPL
ESWEEFPAFFEEAGWGTLTNVSAKKQELEFELEGPIISNRLKHQKEPCFQLEAGFIAEQIQLMND
QIAESYEQVKKRADKVVLTVKWDMKDPV
>3R0NA
MQDVRVQVLPEVRGQLGGTVELPCHLLPPVPGLYISLVTWQRPDAPANHQNVAAFHPKMGPSFPS
PKPGSERLSFVSAKQSTGQDTEAELQDATLALHGLTVEDEGNYTCEFATFPKGSVRGMTWLRV
>1FPZA
MKPPSSIQTSEFDSSDEEPIEDEQTPIHISWLSLSRVNCSQFLGLCALPGCKFKDVRRNVQKDTE
ELKSCGIQDIFVFCTRGELSKYRVPNLLDLYQQCGIITHHHPIADGGTPDIASCCEIMEELTTCL
KNYRKTLIHSYGGLGRSCLVAACLLLYLSDTISPEQAIDSLRDLRGSGAIQTIKQYNYLHEFRDK
LAAHLSSRDSQSRSVSR
>3LX4A
MGSSHHHHHHSQDPNSAAPAAEAPLSHVQQALAELAKPKDDPTRKHVCVQVAPAVRVAIAETLGL
APGATTPKQLAEGLRRLGFDEVFDTLFGADLTIMEEGSELLHRLTEHLEAHPHSDEPLPMFTSCC
PGWIAMLEKSYPDLIPYVSSCKSPQMMLAAMVKSYLAEKKGIAPKDMVMVSIMPCTRKQSEADRD
WFCVDADPTLRQLDHVITTVELGNIFKERGINLAELPEGEWDNPMGVGSGAGVLFGTTGGVMEAA
LRTAYELFTGTPLPRLSLSEVRGMDGIKETNITMVPAPGSKFEELLKHRAAARAEAAAHGTPGPL
AWDGGAGFTSEDGRGGITLRVAVANGLGNAKKLITKMQAGEAKYDFVEIMACPAGCVGGGGQPRS
TDKAITQKRQAALYNLDEKSTLRRSHENPSIRELYDTYLGEPLGHKAHELLHTHYVAGGVEEKDE
KK
>1SCTB
SKVAELANAVVSNADQKDLLRMSWGVLSVDMEGTGLMLMANLFKTSPSAKGKFARLGDVSAGKDN
SKLRGHSITLMYALQNFVDALDDVERLKCVVEKFAVNHINRQISADEFGEIVGPLRQTLKARMGN
YFDEDTVAAWASLVAVVQASL
>3M1MA
SSNFSSERIRYAKWFLEHGFNIIPIDPESKKPVLKEWQKYSHEMPSDEEKQRFLKMIEEGYNYAI
PGGQKGLVILDFESKEKLKAWIGESALEELCRKTLCTNTVHGGIHIYVLSNDIPPHKINPLFEEN
GKGIIDLQSYNSYVLGLGSCVNHLHCTTDKCPWKEQNYTTCYTLYNELKEISKVDLKSLLRFLAE
KGKRLGITLSKTAKEWLEGKKEEEDTVVEFEELRKELVKRDSGKPVEKIKEEICTKSPPKLIKEI
ICENKTYADVNIDRSRGDWHVILYLMKHGVTDPDKILELLPRDSKAKENEKWNTQKYFVITLSKA
WSVVKKYLEA
>3M12A
STHFDVIVVGAGSMGMAAGYQLAKQGVKTLLVDAFDPPHTNGSHHGDTRIIRHAYGEGREYVPLA
LRSQELWYELEKETHHKIFTKTGVLVFGPKGESAFVAETMEAAKEHSLTVDLLEGDEINKRWPGI
TVPENYNAIFEPNSGVLFSENCIRAYRELAEARGAKVLTHTRVEDFDISPDSVKIETANGSYTAD
KLIVSMGAWNSKLLSKLNLDIPLQPYRQVVGFFESDESKYSNDIDFPGFMVEVPNGIYYGFPSFG
GCGLRLGYHTFGQKIDPDTINREFGVYPEDESNLRAFLEEYMPGANGELKRGAVCMYTKTLDEHF
IIDLHPEHSNVVIAAGFSGHGFKFSSGVGEVLSQLALTGKTEHDISIFSINRPALKESLQKTTI
>3BDZA
TSLFTTADHYHTPLGPDGTPHAFFEALRDEAETTPIGWSEAYGGHWVVAGYKEIQAVIQNTKAFS
NKGVTFPRYETGEFELMMAGQDDPVHKKYRQLVAKPFSPEATDLFTEQLRQSTNDLIDARIELGE
GDAATWLANEIPARLTAILLGLPPEDGDTYRRWVWAITHVENPEEGAEIFAELVAHARTLIAERR

TNPGNDIMSRVIMSKIDGESLSEDDLIGFFTILLLGGIDATARFLSSVFWRLAWDIELRRRLIAH
PELIPNAVDELLRFYGPAMVGRLVTQEVTVGDITMKPGQTAMLWFPIASRDRSAFDSPDNIVIER
TPNRHLSLGHGIHRCLGAHLIRVEARVAITEFLKRIPEFSLDPNKECEWLMGQVAGMLHVPIIFP
KGKRLSE
>1FY3A
IVGGRKARPRQFPFLASIQNQGRHFCGGALIHARFVMTAASCFQSQNPGVSTVVLGAYDLRRRER
QSRQTFSISSMSENGYDPQQNLNDLMLLQLDREANLTSSVTILPLPLQNATVEAGTRCQVAGWGS
QRSGGRLSRFPRFVNVTVTPEDQCRPNNVCTGVLTRRGGICNGDQGTPLVCEGLAHGVASFSLGP
CGRGPDFFTRVALFRDWIDGVLNNPGPGPA
>2VR3A
MRGSHHHHHHGSGTDITNQLTNVTVGIDSGTTVYPHQAGYVKLNYGFSVPNSAVKGDTFKITVPK
ELNLNGVTSTAKVPPIMAGDQVLANGVIDSDGNVIYTFTDYVNTKCDVKATLTMPAYIDPENVKK
TGNVTLATGIGSTTANKTVLVDYEKYGKFYNLSIKGTIDQIDKTNNTYRQTIYVNPSGDNVIAPV
LTGNLKPNTDSNALIDQQNTSIKVYKVDNAADLSESYFVNPENFEDVTNSVNITFPNPNQYKVEF
NTPDDQITTPYIVVVNGHIDPNSKGDLALRSTLYGYNSNIIWRSMSWDNEVAFNNGSGSGDGIDC
PVVP
>1R8ME
LEANEGSKTLQRNRKMAMGRKKFNMDPKKGIQFLVENELLQNTPEEIARFLYKGEGLNKTAIGDY
LGEREELNLAVLHAFVDLHEFTDLNLVQALRQFLWSFRLPGEAQKIDRMMEAFAQRYCLCNPGVF
QSTDTCYVLSYSVIMLNTDLHNPNVRDKMGLERFVAMNRGINEGGDLPEELLRNLYDSIRNEPFK
IPEDDGND
>1BYPA
AEVLLGSSDGGLAFVPSDLSIASGEKITFKNNAGFPHNDLFDKKEVPAGVDVTKISMPEEDLLNA
PGEEYSVTLTEKGTYKFYCAPHAGAGMVGKVTVN
>3D0KA
SNAMKPADLTNADRIALELGHAGRNAIPYLDDDRNADRPFTLNTYRPYGYTPDRPVVVVQHGVLR
NGADYRDFWIPAADRHKLLIVAPTFSDEIWPGVESYNNGRAFTAAGNPRHVDGWTYALVARVLAN
IRAAEIADCEQVYLFGHSAGGQFVHRLMSSQPHAPFHAVTAANPGWYTLPTFEHRFPEGLDGVGL
TEDHLARLLAYPMTILAGDQDIATDDPNLPSEPAALRQGPHRYARARHYYEAGQRAAAQRGLPFG
WQLQVVPGIGHDGQAMSQVCASLWFDGRMPDAAELARLAGSQSA
>1ZHVA
APRIKLKILNGSYGIARLSASEAIPAWADGGGFVSITRTDDELSIVCLIDRIPQDVRVDPGWSCF
KFQGPFAFDETGIVLSVISPLSTNGIGIFVVSTFDGDHLLVRSNDLEKTADLLANAGHSLLLEHH
HHHH
>3H3JA
MNKFKGNKVVLIGNGAVGSSYAFSLVNQSIVDELVIIDLDTEKVRGDVMDLKHATPYSPTTVRVK
AGEYSDCHDADLVVICAGARQKPGETRLDLVSKNLKIFKSIVGEVMASKFDGIFLVATNPVDILA
YATWKFSGLPKERVIGSGTILDSARFRLLLSEAFDVAPRSVDAQIIGEHGDTELPVWSHANIAGQ
PLKTLLEQRPEGKAQIEQIFVQTRDAAYDIIQAKGATYYGVAMGLARITEAIFRNEDAVLTVSAL
LEGEYEEEDVYIGVPAVINRNGIRNVVEIPLNDEEQSKFAHSAKTLKDIMAEAEELK
>3H36A
SNAVELLQVDADLQAEIVGKYNADLQKAVQIEEKKASEIATEAVKEHVTAEYEERYAEHEEHDRI
MRDVAEILEQMEHAEVRRLITEDKVRPD
>1Z3EB

MEKEKVLEMTIEELDLSVRSYNCLKRAGINTVQELANKTEEDMMKVRNLGRKSLEEVKAKLEELG
LGLRKDDG
>3KFFA
EEATSKGQNLNVEKINGEWFSILLASDKREKIEEHGSMRVFVEHIHVLENSLAFKFHTVIDGECS
EIFLVADKTEKAGEYSVMYDGFNTFTILKTDYDNYIMFHLINEKDGKTFQLMELYGRKADLNSDI
KEKFVKLCEEHGIIKENIIDLTKTNRCLKARE
>2RB8A
MRLDAPSQIEVKDVTDTTALITWMPPSQPVDGFELTYGIKDVPGDRTTIDLTEDENQYSIGNLKP
DTEYEVSLISRRGDMSSNPAKETFTTGLAAALEHHHHHH
>2RBDA
GMGILSGNPQDEPLHYGEVFSTWTYLSTNNGLINGYRSFINHTGDEDLKNLIDEAIQAMQDENHQ
LEELLRSNGVGLPPAPPDRPAARLDDIPVGARFNDPEISATISMDVAKGLVTCSQIIGQSIREDV
ALMFSQFHMAKVQFGGKMLKLNKNKGWLIPPPLHSDRPIKE
>2W1VA
MSTFRLALIQLQVSSIKSDNLTRACSLVREAAKQGANIVSLPECFNSPYGTTYFPDYAEKIPGES
TQKLSEVAKESSIYLIGGSIPEEDAGKLYNTCSVFGPDGSLLVKHRKIHLFDIDVPGKITFQESK
TLSPGDSFSTFDTPYCKVGLGICYDMRFAELAQIYAQRGCQLLVYPGAFNLTTGPAHWELLQRAR
AVDNQVYVATASPARDDKASYVAWGHSTVVDPWGQVLTKAGTEETILYSDIDLKKLAEIRQQIPI
LKQKRADLYTVESKKP
>3OBIA
GMPHHQYVLTLSCPDRAGIVSAVSTFLFENGQNILDAQQYNDTESGHFFMRVVFNAAAKVIPLAS
LRTGFGVIAAKFTMGWHMRDRETRRKVMLLVSQSDHCLADILYRWRVGDLHMIPTAIVSNHPRET
FSGFDFGDIPFYHFPVNKDTRRQQEAAITALIAQTHTDLVVLARYMQILSDEMSARLAGRCINIH
HSFLPGFKGAKPYHQAFDRGVKLIGATAHYVTSALDEGPIIDQDVERISHRDTPADLVRKGRDIE
RRVLSRALHYHLDDRVILNGRKTVVFTD
>2BZUA
RIAISNSNRTRSVPSLTTIWSISPTPNCSIYETQDANLFLCLTKNGAHVLGTITIKGLKGALREM
HDNALSLKLPFDNQGNLLNCALESSTWRYQETNAVASNALTFMPNSTVYPRNKTAHPGNMLIQIS
PNITFSVVYNEINSGYAFTFKWSAEPGKPFHPPTAVFCYITEQGSHHHHHH
>1Q16A
MSKFLDRFRYFKQKGETFADGHGQLLNTNRDWEDGYRQRWQHDKIVRSTHGVNCTGSCSWKIYVK
NGLVTWETQQTDYPRTRPDLPNHEPRGCPRGASYSWYLYSANRLKYPMMRKRLMKMWREAKALHS
DPVEAWASIIEDADKAKSFKQARGRGGFVRSSWQEVNELIAASNVYTIKNYGPDRVAGFSPIPAM
SMVSYASGARYLSLIGGTCLSFYDWYCDLPPASPQTWGEQTDVPESADWYNSSYIIAWGSNVPQT
RTPDAHFFTEVRYKGTKTVAVTPDYAEIAKLCDLWLAPKQGTDAAMALAMGHVMLREFHLDNPSQ
YFTDYVRRYTDMPMLVMLEERDGYYAAGRMLRAADLVDALGQENNPEWKTVAFNTNGEMVAPNGS
IGFRWGEKGKWNLEQRDGKTGEETELQLSLLGSQDEIAEVGFPYFGGDGTEHFNKVELENVLLHK
LPVKRLQLADGSTALVTTVYDLTLANYGLERGLNDVNCATSYDDVKAYTPAWAEQITGVSRSQII
RIAREFADNADKTHGRSMIIVGAGLNHWYHLDMNYRGLINMLIFCGCVGQSGGGWAHYVGQEKLR
PQTGWQPLAFALDWQRPARHMNSTSYFYNHSSQWRYETVTAEELLSPMADKSRYTGHLIDFNVRA
ERMGWLPSAPQLGTNPLTIAGEAEKAGMNPVDYTVKSLKEGSIRFAAEQPENGKNHPRNLFIWRS
NLLGSSGKGHEFMLKYLLGTEHGIQGKDLGQQGGVKPEEVDWQDNGLEGKLDLVVTLDFRLSSTC
LYSDIILPTATWYEKDDMNTSDMHPFIHPLSAAVDPAWEAKSDWEIYKAIAKKFSEVCVGHLGKE
TDIVTLPIQHDSAAELAQPLDVKDWKKGECDLIPGKTAPHIMVVERDYPATYERFTSIGPLMEKI

GNGGKGIAWNTQSEMDLLRKLNYTKAEGPAKGQPMLNTAIDAAEMILTLAPETNGQVAVKAWAAL
SEFTGRDHTHLALNKEDEKIRFRDIQAQPRKIISSPTWSGLEDEHVSYNAGYTNVHELIPWRTLS
GRQQLYQDHQWMRDFGESLLVYRPPIDTRSVKEVIGQKSNGNQEKALNFLTPHQKWGIHSTYSDN
LLMLTLGRGGPVVWLSEADAKDLGIADNDWIEVFNSNGALTARAVVSQRVPAGMTMMYHAQERIV
NLPGSEITQQRGGIHNSVTRITPKPTHMIGGYAHLAYGFNYYGTVGSNRDEFVVVRKMKNIDWLD
GEGNDQVQESVK
>3CX5C
MAFRKSNVYLSLVNSYIIDSPQPSSINYWWNMGSLLGLCLVIQIVTGIFMAMHYSSNIELAFSSV
EHIMRDVHNGYILRYLHANGASFFFMVMFMHMAKGLYYGSYRSPRVTLWNVGVIIFILTIATAFL
GYCCVYGQMSHWGATVITNLFSAIPFVGNDIVSWLWGGFSVSNPTIQRFFALHYLVPFIIAAMVI
MHLMALHIHGSSNPLGITGNLDRIPMHSYFIFKDLVTVFLFMLILALFVFYSPNTLGHPDNYIPG
NPLVTPASIVPEWYLLPFYAILRSIPDKLLGVITMFAAILVLLVLPFTDRSVVRGNTFKVLSKFF
FFIFVFNFVLLGQIGACHVEVPYVLMGQIATFIYFAYFLIIVPVISTIENVLFYIGRVNK
>3I7QA
MFTGSIVAIVTPMDEKGNVCRASLKKLIDYHVASGTSAIVSVGTTGESATLNHDEHADVVMMTLD
LADGRIPVIAGTGANATAEAISLTQRFNDSGIVGCLTVTPYYNRPSQEGLYQHFKAIAEHTDLPQ
ILYNVPSRTGCDLLPETVGRLAKVKNIIGIREATGNLTRVNQIKELVSDDFVLLSGDDASALDFM
QLGGHGVISVTANVAARDMAQMCKLAAEGHFAEARVINQRLMPLHNKLFVEPNPIPVKWACKELG
LVATDTLRLPMTPITDSGRETVRAALKHAGLL
>3P1GA
GSILAETHGTRPDLTDQPIPDADYTWYTDGSSFLQEGQRRAGAAVTTETEVIWARALPAGTSAQR
AELIALTQALKMAEGKKLNVYTDSRYAFATAHVHSEGREIKNKNEILALLKALFLPKRLSIIHCP
GHQKGNSAEARGNRMADQAAREAAMKAVLETSTLL
>3EA6A
QGDIGIDNLRNFYTKKDFVDLKDVKDNDTPIANQLQFSNESYDLISESKDFNKFSNFKGKKLDVF
GISYNGQCNTKYIYGGVTATNEYLDKSRNIPINIWINGNHKTISTNKVSTNKKFVTAQEIDVKLR
KYLQEEYNIYGHNGTKKGEEYGHKSKFYSGFNIGKVTFHLNNNDTFSYDLFYTGDDGLPKSFLKI
YEDNKTVESEKFHLDVDISYKETI
>1EZ6A
ATSTKKLHKEPATLIKAIDGDTVKLMYKGQPMVFRLLLVDIPETKHPKKGVEKYGPEAAAFTKKM
VENAKKIEVEFDKGQRTDKYGRGLAYIYADGKMVNEALVRQGLAKVAYVYKGNNTHEQLLRKAEA
QAKKEKLNIWSEDNADSGQ
>3GXBA
MVLDVAFVLEGSDKIGEADFNRSKEFMEEVIQRMDVGQDSIHVTVLQYSYMVTVEYPFSEAQSKG
DILQRVREIRYQGGNRTNTGLALRYLSDHSFLVSQGDREQAPNLVYMVTGNPASDEIKRLPGDIQ
VVPIGVGPNANVQELERIGWPNAPILIQDFETLPREAPDLVLQRCCSPHHHHHH
>1LG7A
VDEMDTHDPHQLRYEKFFFTVKMTVRSNRPFRTYSDVAAAVSHWDHMYIGMAGKRPFYKILAFLG
SSNLKATPAVLADQGQPEYHAHCEGRAYLPHRMGKTPPMLNVPEHFRRPFNIGLYKGTVELTMTI
YDDESLEAAPMIWDHFNSSKFSDFREKALMFGLIVEKKASGAWVLDSVSHFK
>2Y0GA
MAHHHHHHGHHHQLVSKGEELFTGVVPILVELDGDVNGHKFSVSGEGEGDATYGKLTLKFICTTG
KLPVPWPTLVTTLXVQCFSRYPDHMKQHDFFKSAMPEGYVQERTIFFKDDGNYKTRAEVKFEGDT
LVNRIELKGIDFKEDGNILGHKLEYNYNSHNVYIMADKQKNGIKVNFKIRHNIEDGSVQLADHYQ

QNTPIGDGPVLLPDNHYLSTQSALSKDPNEKRDHMVLLEFVTAAGITLGMDELYK
>1K3UB
TTLLNPYFGEFGGMYVPQILMPALNQLEEAFVSAQKDPEFQAQFADLLKNYAGRPTALTKCQNIT
AGTRTTLYLKREDLLHGGAHKTNQVLGQALLAKRMGKSEIIAETGAGQHGVASALASALLGLKCR
IYMGAKDVERQSPNVFRMRLMGAEVIPVHSGSATLKDACNEALRDWSGSYETAHYMLGTAAGPHP
YPTIVREFQRMIGEETKAQILDKEGRLPDAVIACVGGGSNAIGMFADFINDTSVGLIGVEPGGHG
IETGEHGAPLKHGRVGIYFGMKAPMMQTADGQIEESYSISAGLDFPSVGPQHAYLNSIGRADYVS
ITDDEALEAFKTLCRHEGIIPALESSHALAHALKMMREQPEKEQLLVVNLSGRGDKDIFTVHDIL
KARGEI
>3NYSA
MIEFIDLKNQQARIKDKIDAGIQRVLRHGQYILGPEVTELEDRLADFVGAKYCISCANGTDALQI
VQMALGVGPGDEVITPGFTYVATAETVALLGAKPVYVDIDPRTYNLDPQLLEAAITPRTKAIIPV
SLYGQCADFDAINAIASKYGIPVIEDAAQSFGASYKGKRSCNLSTVACTSFFPSAPLGCYGDGGA
IFTNDDELATAIRQIARHGQDRRYHHIRVGVNSRLDTLQAAILLPKLEIFEEEIALRQKVAAEYD
LSLKQVGIGTPFIEVNNISVYAQYTVRMDNRESVQASLKAAGVPTAVHYPIPLNKQPAVADEKAK
LPVGDKAATQVMSLPMHPYLDTASIKIICAALTNLEHHHHHH
>2A6ZA
GSDASKLSSDYSLPDLINTRKVPNNWQTGEQASLEEGRIVLTSNQNSKGSLWLKQGFDLKDSFTM
EWTFRSVGYSGQTDGGISFWFVQDSNIPRDKQLYNGPVNYDGLQLLVDNNGPLGPTLRGQLNDGQ
KPVDKTKIYDQSFASCLMGYQDSSVPSTIRVTYDLEDDNLLKVQVDNKVCFQTRKVRFPSGSYRI
GVTAQNGAVNNNAESFEIFKMQFFNGV
>1G72B
YDGQNCKEPGNCWENKPGYPEKIAGSKYDPKHDPVELNKQEESIKAMDARNAKRIANAKSSGNFV
FDVK
>1A8P
SNLNVERVLSVHHWNDTLFSFKTTRNPSLRFENGQFVMIGLEVDGRPLMRAYSIASPNYEEHLEF
FSIKVQNGPLTSRLQHLKEGDELMVSRKPTGTLVTSDLLPGKHLYMLSTGTGLAPFMSLIQDPEV
YERFEKVVLIHGVRQVNELAYQQFITEHLPQSEYFGEAVKEKLIYYPTVTRESFHNQGRLTDLMR
SGKLFEDIGLPPINPQDDRAMICGSPSMLDESCEVLDGFGLKISPRMGEPGDYLIERAFVEK
>1A8Y
GLDFPEYDGVDRVINVNAKNYKNVFKKYEVLALLYHEPPEDDKASQRQFEMEELILELAAQVLED
KGVGFGLVDSEKDAAVAKKLGLTEEDSIYVFKEDEVIEYDGEFSADTLVEFLLDVLEDPVELIEG
ERELQAFENIEDEIKLIGYFKNKDSEHYKAFKEAAEEFHPYIPFFATFDSKVAKKLTLKLNEIDF
YEAFMEEPVTIPDKPNSEEEIVNFVEEHRRSTLRKLKPESMYETWEDDMDGIHIVAFAEEADPDG
YEFLEILKSVAQDNTDNPDLSIIWIDPDDFPLLVPYWEKTFDIDLSAPQIGVVNVTDADSVWMEP
SAEELEDWLEDVL
>1ABE
NLKLGFLVKQPEEPWFQTEWKFADKAGKDLGFEVIKIAVPDGEKTLNAIDSLAASGAKGFVICTP
DPKLGSAIVAKARGYDMKVIAVDDQFVNAKGKPMDTVPLVMMAATKIGERQGQELYKEMQKRGWD
VKESAVMAITANELDTARRRTTGSMDALKAAGFPEKQIYQVPTKSNDIPGAFDAANSMLVQHPEV
KHWLIVGMNDSTVLGGVRATEGQGFKAADIIGIGINGVDAVSELSKAQATGFYGSLLPSPDVHGY
KSSEMLYNWVAKDVEPPKFTEVTDVVLITRDNFKEELEKKGLGGK
>1AIR
ATDTGGYAATAGGNVTGAVSKTATSMQDIVNIIDAARLDANGKKVKGGAYPLVITYTGNEDSLIN

AAAANICGQWSKDPRGVEIKEFTKGITIIGANGSSANFGIWIKKSSDVVVQNMRIGYLPGGAKDG
DMIRVDDSPNVWVDHNELFAANHECDGTPDNDTTFESAVDIKGASNTVTVSYNYIHGVKKVGLDG
SSSSDTGRNITYHHNYYNDVNARLPLQRGGLVHAYNNLYTNITGSGLNVRQNGQALIENNWFEKA
INPVTSRYDGKNFGTWVLKGNNITKPADFSTYSITWTADTKPYVNADSWTSTGTFPTVAYNYSPV
SAQCVKDKLPGYAGVGKNLATLTSTAC
>1AL3
TWPDKGSLYVATTHTQARYALPGVIKGFIERYPRVSLHMHQGSPTQIAEAVSKGNADFAIATEAL
HLYDDLVMLPCYHWNRSIVVTPEHPLATKGSVSIEELAQYPLVTYTFGFTGRSELDTAFNRAGLT
PRIVFTATDADVIKTYVRLGLGVGVIASMAVDPVSDPDLVKLDANGIFSHSTTKIGFRRSTFLRS
YMYDFIQRFAPHLTRDVVDTAVALRSNEDIEAMFKDIKLPEK
>1ALHA
MPVLENRAAQGDITAPGGARRLTGDQTAALRDSLSDKPAKNIILLIGDGMGDSEITAARNYAEGA
GGFFKGIDALPLTGQYTHYALNKKTGKPDYVTDSAASATAWSTGVKTYNGALGVDIHEKDHPTIL
EMAKAAGLATGNVSTAELQDATPAALVAHVTSRKCYGPSATSEKCPGNALEKGGKGSITEQLLNA
RADVTLGGGAKTFAETATAGEWQGKTLREQAEARGYQLVSDAASLNSVTEANQQKPLLGLFADGN
MPVRWLGPKATYHGNIDKPAVTCTPNPQRNDSVPTLAQMTDKAIELLSKNEKGFFLQVEGASIDK
QDHAANPCGQIGETVDLDEAVQRALEFAKKEGNTLVIVTANHAHASQIVAPDTKAPGLTQALNTK
DGAVMVMSYGNSEEDSQEHTGSQLRIAAYGPHAANVVGLTDQTDLFYTMKAALGLK
>1AMF
GKITVFAAASLTNAMQDIATQFKKEKGVDVVSSFASSSTLARQIEAGAPADLFISADQKWMDYAV
DKKAIDTATRQTLLGNSLVVVAPKASVQKDFTIDSKTNWTSLLNGGRLAVGDPEHVPAGIYAKEA
LQKLGAWDTLSPKLAPAEDVRGALALVERNEAPLGIVYGSDAVASKGVKVVATFPEDSHKKVEYP
VAVVEGHNNATVKAFYDYLKGPQAAEIFKRYGFTIK
>1AMK
SAKPQPIAAANWKCNGTTASIEKLVQVFNEHTISHDVQCVVAPTFVHIPLVQAKLRNPKYVISAE
NAIAKSGAFTGEVSMPILKDIGVHWVILGHSERRTYYGETDEIVAQKVSEACKQGFMVIACIGET
LQQREANQTAKVVLSQTSAIAAKLTKDAWNQVVLAYEPVWAIGTGKVATPEQAQEVHLLLRKWVS
ENIGTDVAAKLRILYGGSVNAANAATLYAKPDINGFLVGGASLKPEFRDIIDATR
>1AMX
TSSVFYYKTGDMLPEDTTHVRWFLNINNEKSYVSKDITIKDQIQGGQQLDLSTLNINVTGTHSNY
YSGQSAITDFEKAFPGSKITVDNTKNTIDVTIPQGYGSYNSFSINYKTKITNEQQKEFVNNSQAW
YQEHGKEEVNGKSFNHTVHN
>1ARB
GVSGSCNIDVVCPEGDGRRDIIRAVGAYSKSGTLACTGSLVNNTANDRKMYFLTAHHCGMGTAST
AASIVVYWNYQNSTCRAPNTPASGANGDGSMSQTQSGSTVKATYATSDFTLLELNNAANPAFNLF
WAGWDRRDQNYPGAIAIHHPNVAEKRISNSTSPTSFVAWGGGAGTTHLNVQWQPSGGVTEPGSSG
SPIYSPEKRVLGQLHGGPSSCSATGTNRSDQYGRVFTSWTGGGAAASRLSDWLDPASTGAQFIDG
LDS
>1ARU
SVTCPGGQSTSNSQCCVWFDVLDDLQTNFYQGSKCESPVRKILRIVFHDAIGFSPALTAAGQFGG
GGADGSIIAHSNIELAFPANGGLTDTIEALRAVGINHGVSFGDLIQFATAVGMSNCPGSPRLEFL
TGRSNSSQPSPPSLIPGPGNTVTAILDRMGDAGFSPDEVVDLLAAHSLASQEGLNSAIFRSPLDS
TPQVFDTQFYIETLLKGTTQPGPSLGFAEELSPFPGEFRMRSDALLARDSRTACRWQSMTSSNEV
MGQRYRAAMAKMSVLGFDRNALTDCSDVIPSAVSNNAAPVIPGGLTVDDIEVSCPSEPFPEIATA

SGPLPSLAPAP
>1AT0
CFTPESTALLESGVRKPLGELSIGDRVLSTANGQAVYSEVILFDRNLEQQNFVQLHTDGGAVLTV
TPAHLVSVWQPESQKLTFVFADRIEEKNQVLVRDVETGELRPQRVVKVGSVRSKGVVAPLTREGT
IVVNSVAASCYA
>1AV4
ASPFRLASAGEISEVQGILRTAGLLGPEKRIAYLGVLDPARGAGSEAEDRRFRVFIHDVSGARPQ
EVTVSVTNGTVISAVELDTAATGELPVLEEEFEVVEQLLATDERWLKALAARNLDVSKVRVAPLS
AGVFEYAEERGRRILRGLAFVQDFPEDSAWAHPVDGLVAYVDVVSKEVTRVIDTGVFPVPAEHGN
YTDPELTGPLRTTQKPISITQPEGPSFTVTGGNHIEWEKWSLDVGFDVREGVVLHNIAFRDGDRL
RPIINRASIAEMVVPYGDPSPIRSWQNYFDTGEYLVGQYANSLELGCDCLGDITYLSPVISDAFG
NPREIRNGICMHEEDWGILAKHSDLWSGINYTRRNRRMVISFFTTIGNDYGFYWYLYLDGTIEFE
AKATGVVFTSAFPEGGSDNISQLAPGLGAPFHQHIFSARLDMAIDGFTNRVEEEDVVRQTMGPGN
ERGNAFSRKRTVLTRESEAVREADARTGRTWIISNPESKNRLNEPVGYKLHAHNQPTLLADPGSS
IARRAAFATKDLWVTRYADDERYPTGDFVNQHSGGAGLPSYIAQDRDIDGQDIVVWHTFGLTHFP
RVEDWPIMPVDTVGFKLRPEGFFDRSPVLDVPAN
>1AYL
MRVNNGLTPQELEAYGISDVHDIVYNPSYDLLYQEELDPSLTGYERGVLTNLGAVAVDTGIFTGR
SPKDKYIVRDDTTRDTFWWADKGKGKNDNKPLSPETWQHLKGLVTRQLSGKRLFVVDAFCGANPD
TRLSVRFITEVAWQAHFVKNMFIRPSDEELAGFKPDFIVMNGAKCTNPQWKEQGLNSENFVAFNL
TERMQLIGGTWYGGEMKKGMFSMMNYLLPLKGIASMHCSANVGEKGDVAVFFGLSGTGKTTLSTD
PKRRLIGDDEHGWDDDGVFNFEGGCYAKTIKLSKEAEPEIYNAIRRDALLENVTVREDGTIDFDD
GSKTENTRVSYPIYHIDNIVKPVSKAGHATKVIFLTADAFGVLPPVSRLTADQTQYHFLSGFTAK
LAPTPTFSACFGAAFLSLHPTQYAEVLVKRMQAAGAQAYLVNTGWNGTGKRISIKDTRAIIDAIL
NGSLDNAETFTLPMFNLAIPTELPGVDTKILDPRNTYASPEQWQEKAETLAKLFIDNFDKYTDTP
AGAALVAAGPKL
>1B51A
ADVPAGVQLADKQTLVRNNGSEVQSLDPHKIEGVPESNVSRDLFEGLLISDVEGHPSPGVAEKWE
NKDFKVWTFHLRENAKWSDGTPVTAHDFVYSWQRLADPNTASPYASYLQYGHIANIDDIIAGKKP
ATDLGVKALDDHTFEVTLSEPVPYFYKLLVHPSVSPVPKSAVEKFGDKWTQPANIVTNGAYKLKN
WVVNERIVLERNPQYWDNAKTVINQVTYLPISSEVTDVNRYRSGEIDMTYNNMPIELFQKLKKEI
PNEVRVDPYLCTYYYEINNQKAPFNDVRVRTALKLALDRDIIVNKVKNQGDLPAYSYTPPYTDGA
KLVEPEWFKWSQQKRNEEAKKLLAEAGFTADKPLTFDLLYNTSDLHKKLAIAVASIWKKNLGVNV
NLENQEWKTFLDTRHQGTFDVARAGWCADYNEPTSFLNTMLSDSSNNTAHYKSPAFDKLIADTLK
VADDTQRSELYAKAEQQLDKDSAIVPVYYYVNARLVKPWVGGYTGKDPLDNIYVKNLYIIKH
>1B6A
KVQTDPPSVPICDLYPNGVFPKGQECEYPEEKKALDQASEEIWNDFREAAEAHRQVRKYVMSWIK
PGMTMIEICEKLEDCSRKLIKENGLNAGLAFPTGCSLNNCAAHYTPNAGDTTVLQYDDICKIDFG
THISGRIIDCAFTVTFNPKYDTLLKAVKDATNTGIKCAGIDVRLCDVGEAIQEVMESYEVEIDGK
TYQVKPIRNLNGHSIGQYRIHAGKTVPIVKGGEATRMEEGEVYAIETFGSTGKGVVHDDMECSHY
MKNFDVGHVPIRLPRTKHLLNVINENFGTLAFCRRWLDRLGESKYLMALKNLCDLGIVDPYPPLC
DIKGSYTAQFEHTILLRPTCKEVVSRGDDY
>1BB9
TTGRLDLPPGFMFKVQAQHDYTATDTDELQLKAGDVVLVIPFQNPEEQDEGWLMGVKESDWNQHK

ELEKCRGVFPENFTERVQ
>1BDB
MKLKGEAVLITGGASGLGRALVDRFVAEGAKVAVLDKSAERLAELETDHGDNVLGIVGDVRSLED
QKQAASRCVARFGKIDTLIPNAGIWDYSTALVDLPEESLDAAFDEVFHINVKGYIHAVKACLPAL
VASRGNVIFTISNAGFYPNGGGPLYTAAKHAIVGLVRELAFELAPYVRVNGVGSGGINSDLRGPS
SLGPLADMLKSVLPIGRMPEVEEYTGAYVFFATRGDAAPATGALLNYDGGLGVRGFFSGAGGNDL
LEQLNIH
>1BFD
ASVHGTTYELLRRQGIDTVFGNPGSNELPFLKDFPEDFRYILALQEACVVGIADGYAQASRKPAF
INLHSAAGTGNAMGALSNAWNSHSPLIVTAGQQTRAMIGVEALLTNVDAANLPRPLVKWSYEPAS
AAEVPHAMSRAIHMASMAPQGPVYLSVPYDDWDKDADPQSHHLFDRHVSSSVRLNDQDLDILVKA
LNSASNPAIVLGPDVDAANANADCVMLAERLKAPVWVAPSAPRCPFPTRHPCFRGLMPAGIAAIS
QLLEGHDVVLVIGAPVFRYHQYDPGQYLKPGTRLISVTCDPLEAARAPMGDAIVADIGAMASALA
NLVEESSRQLPTAAPEPAKVDQDAGRLHPETVFDTLNDMAPENAIYLNESTSTTAQMWQRLNMRN
PGSYYFCAAGGLGFALPAAIGVQLAEPERQVIAVIGDGSANYSISALWTAAQYNIPTIFVIMNNG
TYGALRWFAGVLEAENVPGLDVPGIDFRALAKGYGVQALKADNLEQLKGSLQEALSAKGPVLIEV
STV
>1BG2
DLAECNIKVMCRFRPLNESEVNRGDKYIAKFQGEDTVVIASKPYAFDRVFQSSTSQEQVYNDCAK
KIVKDVLEGYNGTIFAYGQTSSGKTHTMEGKLHDPEGMGIIPRIVQDIFNYIYSMDENLEFHIKV
SYFEIYLDKIRDLLDVSKTNLSVHEDKNRVPYVKGCTERFVCSPDEVMDTIDEGKSNRHVAVTNM
NEHSSRSHSIFLINVKQENTQTEQKLSGKLYLVDLAGSEKVSKTGAEGAVLDEAKNINKSLSALG
NVISALAEGSTYVPYRDSKMTRILQDSLGGNCRTTIVICCSPSSYNESETKSTLLFGQRAKTI
>1BG6
SKTYAVLGLGNGGHAFAAYLALKGQSVLAWDIDAQRIKEIQDRGAIIAEGPGLAGTAHPDLLTSD
IGLAVKDADVILIVVPAIHHASIAANIASYISEGQLIILNPGATGGALEFRKILRENGAPEVTIG
ETSSMLFTCRSERPGQVTVNAIKGAMDFACLPAAKAGWALEQIGSVLPQYVAVENVLHTSLTNVN
AVMHPLPTLLNAARCESGTPFQYYLEGITPSVGSLAEKVDAERIAIAKAFDLNVPSVCEWYPATI
YEAVQGNPAYRGIAGPINLNTRYFFEDVSTGLVPLSELGRAVNVPTPLIDAVLDLISSLIDTDFR
KEGRTLEKLGLSGLTAAGIRSAVE
>1CHD
LLSSEKLIAIGASTGGTEAIRHVLQPLPLSSPAVIITQHMPPGFTRSFAERLNKLCQISVKEAED
GERVLPGHAYIAPGDKHMELARSGANYQIKIHDGPPVNRHRPSVDVLFHSVAKHAGRNAVGVILT
GMGNDGAAGMLAMYQAGAWTIAQNEASCVVFGMPREAINMGGVSEVVDLSQVSQQMLAKISAGQA
IRI
>1CIY
YTPIDISLSLTQFLLSEFVPGAGFVLGLVDIIWGIFGPSQWDAFLVQIEQLINQRIEEFARNQAI
SRLEGLSNLYQIYAESFREWEADPTNPALREEMRIQFNDMNSALTTAIPLLAVQNYQVPLLSVYV
QAANLHLSVLRDVSVFGQRWGFDAATINSRYNDLTRLIGNYTDYAVRWYNTGLERVWGPDSRDWV
RYNQFRRELTLTVLDIVALFSNYDSRRYPIRTVSQLTREIYTNPVLENFDGSFRGMAQRIEQNIR
QPHLMDILNSITIYTDVHRGFNYWSGHQITASPVGFSGPEFAFPLFGNAGNAAPPVLVSLTGLGI
FRTLSSPLYRRIILGSGPNNQELFVLDGTEFSFASLTTNLPSTIYRQRGTVDSLDVIPPQDNSVP
PRAGFSHRLSHVTMLSQAAGAVYTLRAPTFSWQHRSAEFNNIIPSSQITQIPLTKSTNLGSGTSV
VKGPGFTGGDILRRTSPGQISTLRVNITAPLSQRYRVRIRYASTTNLQFHTSIDGRPINQGNFSA

TMSSGSNLQSGSFRTVGFTTPFNFSNGSSVFTLSAHVFNSGNEVYIDRIEFVPAEVT
>1CLC
IETKVSAAKITENYQFDSRIRLNSIGFIPNHSKKATIAANCSTFYVVKEDGTIVYTGTATSMFDN
DTKETVYIADFSSVNEEGTYYLAVPGVGKSVNFKIAMNVYEDAFKTAMLGMYLLRCGTSVSATYN
GIHYSHGPCHTNDAYLDYINGQHTKKDSTKGWHDAGDYNKYVVNAGITVGSMFLAWEHFKDQLEP
VALEIPEKNNSIPDFLDELKYEIDWILTMQYPDGSGRVAHKVSTRNFGGFIMPENEHDERFFVPW
SSAATADFVAMTAMAARIFRPYDPQYAEKCINAAKVSYEFLKNNPANVFANQSGFSTGEYATVSD
ADDRLWAAAEMWETLGDEEYLRDFENRAAQFSKKIEADFDWDNVANLGMFTYLLSERPGKNPALV
QSIKDSLLSTADSIVRTSQNHGYGRTLGTTYYWGCNGTVVRQTMILQVANKISPNNDYVNAALDA
ISHVFGRNYYNRSYVTGLGINPPMNPHDRRSGADGIWEPWPGYLVGGGWPGPKDWVDIQDSYQTN
EIAINWNAALIYALAGFVNYN
>1CNV
DISSTEIAVYWGQREDGLLRDTCKTNNYKIVFISFLDKFGCEIRKPELELEGVCGPSVGNPCSFL
ESQIKECQRMGVKVFLALGGPKGTYSACSADYAKDLAEYLHTYFLSERREGPLGKVALDGIHFDI
QKPVDELNWDNLLEELYQIKDVYQSTFLLSAAPGCLSPDEYLDNAIQTRHFDYIFVRFYNDRSCQ
YSTGNIQRIRNAWLSWTKSVYPRDKNLFLELPASQATAPGGGYIPPSALIGQVLPYLPDLQTRYA
GIALWNRQADKETGYSTNIIRYL
>1COT
DGDAAKGEKEFNKCKACHMIQAPDGTDIIKGGKTGPNLYGVVGRKIASEEGFKYGEGILEVAEKN
PDLTWTEADLIEYVTDPKPWLVKMTDDKGAKTKMTFKMGKNQADVVAFLAQNSPDA
>1CPO
EPGSGIGYPYDNNTLPYVAPGPTDSRAPCPALNALANHGYIPHDGRAISRETLQNAFLNHMGIAN
SVIELALTNAFVVCEYVTGSDCGDSLVNLTLLAEPHAFEHDHSFSRKDYKQGVANSNDFIDNRNF
DAETFQTSLDVVAGKTHFDYADMNEIRLQRESLSNELDFPGWFTESKPIQNVESGFIFALVSDFN
LPDNDENPLVRIDWWKYWFTNESFPYHLGWHPPSPAREIEFVTSASSAVLAASVTSTPSSLPSGA
IGPGAEAVPLSFASTMTPFLLATNAPYYAQDPTLGPND
>1CV8
NEQYVNKLENFKIRETQGNNGWCAGYTMSALLNATYNTNKYHAEAVMRFLHPNLQGQQFQFTGLT
PREMIYFGQTQGRSPQLLNRMTTYNEVDNLTKNNKGIAILGSRVESRNGMHAGHAMAVVGNAKLN
NGQEVIIIWNPWDNGFMTQDAKNNVIPVSNGDHYQWYSSIYGY
>1CVL
ADTYAATRYPVILVHGLAGTDKFANVVDYWYGIQSDLQSHGAKVYVANLSGFQSDDGPNGRGEQL
LAYVKQVLAATGATKVNLIGHSQGGLTSRYVAAVAPQLVASVTTIGTPHRGSEFADFVQDVLKTD
PTGLSSTVIAAFVNVFGTLVSSSHNTDQDALAALRTLTTAQTATYNRNFPSAGLGAPGSCQTGAA
TETVGGSQHLLYSWGGTAIQPTSTVTGATDTSTGTLDVANVTDPSTLALLATGAVMINRASGQND
GLVSRCSSLFGQVISTSYHWNHLDEINQLLGVRGANAEDPVAVIRTHVNRLKLQGV
>1CYO
SKAVKYYTLEEIQKHNNSKSTWLILHYKVYDLTKFLEEHPGGEEVLREQAGGDATENFEDVGHST
DARELSKTFIIGELHPDDRSKIT
>4FHZA
MHHHHHHSSGLVPRGSGMKETAAAKFERQHMDSPDLGTDDDDKAMADIMTRKLTFGRRGAAPGEA
TSLVVFLHGYGADGADLLGLAEPLAPHLPGTAFVAPDAPEPCRANGFGFQWFPIPWLDGSSETAA
AEGMAAAARDLDAFLDERLAEEGLPPEALALVGFSQGTMMALHVAPRRAEEIAGIVGFSGRLLAP
ERLAEEARSKPPVLLVHGDADPVVPFADMSLAGEALAEAGFTTYGHVMKGTGHGIAPDGLSVALA

FLKERLPDACGRTRAPPPPPLRSGC
>4FHRA
GTKFSKEQLRTFQMIHENFGRALSTYLSGRLRTFVDVEISIDQLTYEEFIRSVMIPSFIVIFTGD
VFEGSAIFEMRLDLFYTMLDIIMGGPGENPPNRPPTEIETSIMRKEVTNMLTLLAQAWSDFQYFI
PSIENVETNPQFVQIVPPNEIVLLVTASVSWGEFTSFINVCWPFSLLEPLLEKLSDR
>4FGZA
MTLIENLNSDKTFLENNQYTDEGVKVYEFIFGENYISSGGLEATKKILSDIELNENSKVLDIGSG
LGGGCMYINEKYGAHTHGIDICSNIVNMANERVSGNNKIIFEANDILTKEFPENNFDLIYSRDAI
LHLSLENKNKLFQKCYKWLKPTGTLLITDYCATEKENWDDEFKEYVKQRKYTLITVEEYADILTA
CNFKNVVSKDLSDYWNQLLEVEHKYLHENKEEFLKLFSEKKFISLDDGWSRKIKDSKRKMQRWGY
FKATKN
>4FFXA
GSHMAAGGDHGSPDSYRSPLASRYASPEMCFVFSDRYKFRTWRQLWLWLAEAEQTLGLPITDEQI
REMKSNLENIDFKMAAEEEKRLRHDVMAHVHTFGHCCPKAAGIIHLGATSCYVGDNTDLIILRNA
LDLLLPKLARVISRLADFAKERASLPTLGFTHFQPAQLTTVGKRCCLWIQDLCMDLQNLKRVRDD
LRFRGVKGTTGTQASFLQLFEGDDHKVEQLDKMVTEKAGFKRAFIITGQTYTRKVDIEVLSVLAS
LGASVHKICTDIRLLANLKEMEEPFEKQQIGSSAMPYKRNPMRSERCCSLARHLMTLVMDPLQTA
SVQWFERTLDDSANRRICLAEAFLTADTILNTLQNISEGLVVYPKVIERRIRQELPFMATENIIM
AMVKAGGSRQDCHEKIRVLSQQAASVVKQEGGDNDLIERIQVDAYFSPIHSQLDHLLDPSSFTGR
ASQQVQRFLEEEVYPLLKPYESVMKVKAELCL
>4AVXA
SMGRGSGTFERLLDKATSQLLLETDWESILQICDLIRQGDTQAKYAVNSIKKKVNDKNPHVALYA
LEVMESVVKNCGQTVHDEVANKQTMEELKDLLKRQVEVNVRNKILYLIQAWAHAFRNEPKYKVVQ
DTYQIMKVEGHVFPEFKESDAMFAAERAPDWVDAEECHRCRVQFGVMTRKHHCRACGQIFCGKCS
SKYSTIPKFGIEKEVRVCEPCYEQLNRKAEG
>4FEIA
QGGPWTPAADWRDAGTHLDLLLDVPGVDAGTLALAEDGGQLTVSGERPGTEHLLRSERPSGRFVR
ELAFPEPVRPASGVASLAGGVLTVRFEKLRPTIDVTA
>4FF1A
MGGSHHHHHHRSESTVTEELKEGIDAVYPSLVGTADSKAEGIKNYFKLSFTLPEEQKSRTVGSEA
PLKDVAQALSSRARYELFTEKETANPAFNGEVIKRYKELMEHGEGIADILRSRLAKFLNTKDVGK
RFAQGTEANRWVGGKLLNIVEQDGDTFKYNEQLLQTAVLAGLQWRLTATSNTAIKDAKDVAAITG
IDQALLPEGLVEQFDTGMTLTEAVSSLAQKIESYWGLSRNPNAPLGYTKGIPTAMAAEILAAFVE
STDVVENIVDMSEIDPDNKKTIGLYTITELDSFDPINSFPTAIEEAVLVNPTEKMFFGDDIPPVA
NTQLRNPAVRNTPEQKAALKAEQATEFYVHTPMVQFYETLGKDRILELMGAGTLNKELLNDNHAK
SLEGKNRSVEDSYNQLFSVIEQVRAQSEDISTVPIHYAYNMTRVGRMQMLGKYNPQSAKLVREAI
LPTKATLDLSNQNNEDFSAFQLGLAQALDIKVHTMTREVMSDELTKLLEGNLKPAIDMMVEFNTT
GSLPENAVDVLNTALGDRKSFVALMALMEYSRYLVAEDKSAFVTPLYVEADGVTNGPINAMMLMT
GGLFTPDWIRNIAKGGLFIGSPNKTMNEHRSTADNNDLYQASTNALMESLGKLRSNYASNMPIQS
QIDSLLSLMDLFLPDINLGENGALELKRGIAKNPLTITIYGSGARGIAGKLVSSVTDAIYERMSD
VLKARAKDPNISAAMAMFGKQAASEAHAEELLARFLKDMETLTSTVPVKRKGVLELQSTGTGAKG
KINPKTYTIKGEQLKALQENMLHFFVEPLRNGITQTVGESLVYSTEQLQKATQIQSVVLEDMFKQ
RVQEKLAEKAKDPTWKKGDFLTQKELNDIQASLNNLAPMIETGSQTFYIAGSENAEVANQVLATN
LDDRMRVPMSIYAPAQAGVAGIPFMTIGTGDGMMMQTLSTMKGAPKNTLKIFDGMNIGLNDITDA

SRKANEAVYTSWQGNPIKNVYESYAKFMKNVDFSKLSPEALEAIGKSALEYDQRENATVDDIANA
ASLIERNLRNIALGVDIRHKVLDKVNLSIDQMAAVGAPYQNNGKIDLSNMTPEQQADELNKLFRE
ELEARKQKVAKAR
>4FE1A
MTISPPEREPKVRVVVDNDPVPTSFEKWAKPGHFDRTLARGPQTTTWIWNLHALAHDFDTHTSDL
EDISRKIFSAHFGHLAVVFIWLSGMYFHGAKFSNYEAWLADPTGIKPSAQVVWPIVGQGILNGDV
GGGFHGIQITSGLFQLWRASGITNEFQLYCTAIGGLVMAGLMLFAGWFHYHKRAPKLEWFQNVES
MLNHHLAGLLGLGSLAWAGHQIHVSLPINKLLDAGVAAKDIPLPHEFILNPSLMAELYPKVDWGF
FSGVIPFFTFNWAAYSDFLTFNGGLNPVTGGLWLSDTAHHHLAIAVLFIIAGHMYRTNWGIGHSL
KEILEAHKGPFTGAGHKGLYEVLTTSWHAQLAINLAMMGSLSIIVAQHMYAMPPYPYLATDYPTQ
LSLFTHHMWIGGFLVVGGAAHGAIFMVRDYDPAMNQNNVLDRVLRHRDAIISHLNWVCIFLGFHS
FGLYVHNDTMRAFGRPQDMFSDTGIQLQPVFAQWVQNLHTLAPGGTAPNAAATASVAFGGDVVAV
GGKVAMMPIVLGTADFMVHHIHAFTIHVTVLILLKGVLFARSSRLIPDKANLGFRFPCDGPGRGG
TCQVSGWDHVFLGLFWMYNCISVVIFHFSWKMQSDVWGTVAPDGTVSHITGGNFAQSAITINGWL
RDFLWAQASQVIGSYGSALSAYGLLFLGAHFIWAFSLMFLFSGRGYWQELIESIVWAHNKLKVAP
AIQPRALSIIQGRAVGVAHYLLGGIATTWAFFLARIISVG
>4AVPA
SMGPTSQRRGSLQLWQFLVALLDDPSNSHFIAWTGRGMEFKLIEPEEVARRWGIQKNRPAMNYDK
LSRSLRYYYEKGIMQKVAGERYVYKFVCDPEALFSMAFSDN
>4FD5A
MLDSKLNNIRFETISSKYYDDVIEHLRQTFFADEPLNKAVNLTRPGQGHPLLEQHSLSTLKDNVS
IMAISNDGDIAGVALNGILYGNTDIEKSREKLNEIQDESFKKIFKLLYEQNLKINLFKQFDVDKI
FEIRILSVDSRFRGKGLAKKLIEKSEELALDRGFQVMKTDATGAFSQRVVSSLGFITKCEINYTD
YLDENGEQIFVVDPPHEKLKIMCKVIN
>4FCIA
MSAKSRTIGIIGAPFSKGQPRGGVEEGPTVLRKAGLLEKLKEQECDVKDYGDLPFADIPNDSPFQ
IVKNPRSVGKASEQLAGKVAEVKKNGRISLVLGGDHSLAIGSISGHARVHPDLGVIWVDAHTDIN
TPLTTTSGNLHGQPVSFLLKELKGKIPDVPGFSWVTPCISAKDIVYIGLRDVDPGEHYILKTLGI
KYFSMTEVDRLGIGKVMEETLSYLLGRKKRPIHLSFDVDGLDPSFTPATGTPVVGGLTYREGLYI
TEEIYKTGLLSGLDIMEVNPSLGKTPEEVTRTVNTAVAITLACFGLAREGNHKPIDYLNPPK
>4F9KA
MSGLNDIFEAQKIEWHEHHHHHHENLYFQSHMEDESLKGCELYVQLHGIQQVLKDCIVHLCISKP
ERPMKFLREHFEKLEKEENRQILARQKSNS
>4F99B
GPGTRTGRLKKPFVKVEDMSQLYRPFYLQLTNMPFINYSIQKPCSPFDVDKPSSMQKQTQVKLRI
QTDGDKYGGTSIQLQLKEKKKKGYCECCLQKYEDLETHLLSEQHRNFAQSNQYQVVDDIVSKLVF
DFVEYEKDTPKKKR
>4F8CA
MAHHHHHHSSGLEVLFQGPPVSHSINNPSIQHVQDFATLSARSLRANVLLNSDDHSVPIHAKNPS
ELLEAIDNNISQTAQDWGVSIQEVEVILGSSKRIIEPVAGVTANTIMKLFLDNDIFSYSFEKGQS
LSLSQLQERLASLPAHKNFILRVNDGGLGHAYVIDFPATTNPSRDAFLYQSDLGEGVTREVRFED
WMTQKASHPISLDDINTHFIGIAQDQIDLAHIAKLFDVDGNVKMLRADHLISHKTSEFNFQLFEY
DLKNLENNMSIIKTH
>4F84A

MGSSHHHHHHSSGLVPRGSHMAAASAPVPGPGGASSTARGRIPAPATPYQEDIARYWNNEARPVN
LRLGDVDGLYHHHYGIGAVDHAALGDPGDGGYEARLIAELHRLESAQAEFLLDHLGPVGPGDTLV
DAGCGRGGSMVMAHQRFGCKVEGVTLSAAQAEFGNRRARELGIDDHVRSRVCNMLDTPFEKGTVA
ASWNNESSMYVDLHDVFAEHSRFLRVGGRYVTVTGCWNPRYGQPSKWVSQINAHFECNIHSRREY
LRAMADNRLVPQTVVDLTPETLPYWELRATSSLVTGIEEAFIESYRDGSFQYVLIAADRV
>4F6OA
EQAKAQLSNGYNNPNVNASNMYGPPQNMSLPPPQTQTIQGTDQPYQYSQCTGRRKALIIGINYIG
SKNQLRGCINDAHNIFNFLTNGYGYSSDDIVILTDDQNDLVRVPTRANMIRAMQWLVKDAQPNDS
LFLHYSGHGGQTEDLDGDEEDGMDDVIYPVDFETQGPIIDDEMHDIMVKPLQQGVRLTALFDSCH
SGTVLDLPYTYSTKGIIKEPNIWKDVGQDGLQAAISYATGNRAALIGSLGSIFKTVKGGMGNNVD
RERVRQIKFSAADVVMLSGSKDNQTSADAVEDGQNTGAMSHAFIKVMTLQPQQSYLSLLQNMRKE
LAGKYSQKPQLSSSHPIDVNLQFIM
>2LT5A
RPCKYKLKKSTNKFCVTCENQAPVHFVGVGSCGSGGSGIFLETSLSAGSDWLTFQKKHITNTRDV
DCDNIMSTNLFHCKDKNTFIYSRPEPVKAICKGIIASKNVLTTSEFYLSDCNVTS
>4F52B
GSMDVDTPSGTNSGAGKKRFEVKKWNAVALWAWDIVVDNCAICRNHIMDLCIECQANQASATSEE
CTVAWGVCNHAFHFHCISRWLKTRQVCPLDNREWEFQKYGH
>4F52E
GSMAVEELQSIIKRCQILEEQDFKEEDFGLFQLAGQRCIEEGHTDQLLEIIQNEKNKVIIKNMGW
NLVGPVVRCLLCKDKEDSKRKVYFLIFDLLVKLCNPKELLLGLLELIEEPSGKQISQSILLLLQP
LQTVIQKLHNKAYSIGLALSTLWNQLSLLPVPYSKEQIQMDDYGLCQCCKALIEFTKPFVEEVID
NKENSLENEKLKDELLKFCFKSLKCPLLTAQFFEQSEEGGNDPFRYFASEIIGFLSAIGHPFPKM
IFNHGRKKRTWNYLEFEEEENKQLADSMASLAYLVFVQGIHIDQLPMVLSPLYLLQFNMGHIEVF
LQRTEESVISKGLELLENSLLRIEDNSLLYQYLEIKSFLTVPQGLVKVMTLCPIETLRKKSLAML
QLYINKLDSQGKYTLFRCLLNTSNHSGVEAFIIQNIKNQIDMSLKRTRNNKWFTGPQLISLLDLV
LFLPEGAETDLLQNSDRIMASLNLLRYLVIKDNENDNQTGLWTELGNIENNFLKPLHIGLNMSKA
HYEAEIKNSQEAQKSKDLCSITVSGEEIPNMPPEMQLKVLHSALFTFDLIESVLARVEELIEIKT
KSTSEENIGIK
>4F3WA
GPGSMPDIDWKQLRDKATQVAAGAYAPYSRFPVGAAALVDDGRVVTGCNVENVSYGLALCAECGV
VCALHATGGGRLVALACVDGRGAPLMPCGRCRQLLFEHGGPELLVDHLAGPRRLGDLLPEPFHAD
LTGEP
>4F2ME
YPYDVPDYAGAQPARSPGLVPRGSRTANLNNGFYPVSSSEVGLVNKSVVLLPSFYTHTIVNITID
LGMKRSGYGQPIASTLSNITLPMQDNNTDVYCIRSDQFSVYVHSTCKSSLWDNIFKRNCTDVLDA
TAVIKTGTCPFSFDKLNNYLTFNKFCLSLSPVGANCKFDVAARTRTNEQVVRSLYVIYEEGDNIV
LVPRGSDYKDDDDK
>4F2ZA
MSSQVEHPAGGYKKLFETVEELSSPLTAHVTGRIPLWLTGSLLRCGPGLFEVGSEPFYHLFDGQA
LLHKFDFKEGHVTYHRRFIRTDAYVRAMTEKRIVITEFGTCAFPDPCKNIFSRFFSYFRGVEVTD
NALVNIYPVGEDYYACTETNFITKVNPETLETIKQVDLCNYVSVNGATAHPHIENDGTVYNIGNC
FGKNFSIAYNIVKIPPLQADKEDPISKSEIVVQFPCSDRFKPSYVHSFGLTPNYIVFVETPVKIN
LFKFLSSWSLWGANYMDCFESNETMGVWLHIADKKRKKYINNKYRTSPFNLFHHINTYEDHEFLI

VDLCCWKGFEFVYNYLYLANLRENWEEVKKNARKAPQPEVRRYVLPLNIDKADTGKNLVTLPNTT
ATAILCSDETIWLEPEVLFSGPRQAFEFPQINYQKYGGKPYTYAYGLGLNHFVPDRLCKLNVKTK
ETWVWQEPDSYPSEPIFVSHPDALEEDDGVVLSVVVSPGAGQKPAYLLILNAKDLSEVARAEVEI
NIPVTFHGLFKKS

>4F0VA

MGSSHHHHHHSSGENLYFEGSHMASMTGGQQMGRMDSLDQCIVNACKNSWDKSYLAGTPNKDNCS
GFVQSVAAELGVPMPRGNANAMVDGLEQSWTKLASGAEAAQKAAQGFLVIAGLKGRTYGHVAVVI
SGPLYRQKYPMCWCGSIAGAVGQSQGLKSVGQVWNRTDRDRLNYYVYSLASCSLPRAS

>4F02A

GPLGSMNPSAPSYPMASLYVGDLHPDVTEAMLYEKFSPAGPILSIRVCRDMITRRSLGYAYVNFQ
QPADAERALDTMNFDVIKGKPVRIMWSQRDPSLRKSGVGNIFIKNLDKSIDNKALYDTFSAFGNI
LSCKVVCDENGSKGYGFVHFETQEAAERAIEKMNGMLLNDRKVFVGRFKSRKEREAELGARAKEF
YPYDVPDYAGSSGRIVTD

>4ASUH

AEAAAAQAPAAGPGQMSFTFASPTQVFFNSANVRQVDVPTQTGAFGILAAHVPTLQVLRPGLVVV
HAEDGTTSKYFVSSGSVTVNADSSVQLLAEEAVTLDMLDLGAAKANLEKAQSELLGAADEATRAE
IQIRIEANEALVKALE

>4EY0A

HSNEKWFHGKLGAGRDGRHIAERLLTEYCIETGAPDGSFLVRESETFVGDYTLSFWRNGKVQHCR
IHSRQDAGTPKFFLTDNLVFDSLYDLITHYQQVPLRCNEFEMRLSEPVPQTNAHESKEWYHASLT
RAQAEHMLMRVPRDGAFLVRKRNEPNSYAISFRAEGKIKHCRVQQEGQTVMLGNSEFDSLVDLIS
YYEKHPLYRKMKLRYPINEEALEKIGTAEPDFGALFEGRNPGFYVEANPMP

>4ARZB

MSLEATDSKAMVLLMGVRRCGKSSICKVVFHNMQPLDTLYLESTSNPSLEHFSTLIDLAVMELPG
QLNYFEPSYDSERLFKSVGALVYVIDSQDEYINAITNLAMIIEYAYKVNPSINIEVLIHKVDGLS
EDFKVDAQRDIMQRTGEELLELGLDGVQVSFYLTSIFDHSIYEAFSRIVQKLIPELSFLENMLDN
LIQHSKIEKAFLFDVNSKIYVSTDSNPVDIQMYEVCSEFIDVTIDLFDLYKAPVLRNSQKSSDKD
NVINPRNELQNVSQLANGVIIYLRQMIRGLALVAIIRPNGTDMESCLTVADYNIDIFKKGLEDIW
ANARASQAKNSIEDDV

>4EVWA

MIVIPMAGMSSRFFKAGYTQPKYMLEAHGQTLFEHSVNSFAAYFASTPFLFIVRNVYDTAVFVRE
KATQLGIKQFYIAELHTETRGQAETVTLGLEELAKQGVDYQGSITVFNIDTFRPNFVFPDISQHS
DGYLEVFQGGGDNWSFAKPEHAGSTKVIQTAEKNPISDLCSTGLYHFNRKEDYLEAYREYVARPS
QEWERGELYIAPLYNELIQKGLNIHYHLIARHEVIFCGVPDEYTDFLRQPQPLEHHHHHH

>4EVFA

MPKVTDIANELKQAIDAKDEVQIAFIASEYSAESREKIAKAYVASYGKELPDDIKKALKGGSEES
LLMDLFSDRHEVRAQHIRDALSGRNDHMAFFDTVILCTPEDWHETVAAYTRMFKKPLVEDFMKDV
GRKEDWCLLMEKWMAHERVSRPGSPEDEAQRLDQAFDQKNTAYLIDFFGTVPSAEYRPIAEAFKA
QNGKSIEQAIATIYTKTDYYTFYCAHFALLGMHRLAAYLINCACNDKGDEKRMRRITGMMVDKCL
GAKHAYKIYGDMGTDIERCFDKRMAPILRTLWRVK

>4EUWA

MAHHHHHHVDDDDKMSENLYFQSSKNKPHVKRPMNAFMVWAQAARRKLADQYPHLHNAELSKTLG
KLWRLLNESEKRPFVEEAERLRVQHKKDHPDYKYQPRRRKS

>4ETYA
SMQEGSLPDITIFPNSSLMISQGTFVTVVCSYSDKHDLYNMVRLEKDGSTFMEKSTEPYKTEDEF
EIGPVNETITGHYSCIYSKGITWSERSKTLELKVIKENVIQTPAPGPTSEHLG
>4ETPB
GASEIAALEKEIAALEKEIAALEKEISKQEKFYNDTYNTVCKELLRSRRLENSIIEQKGTMRVYA
YVMEQNLPENLLFDYENGVITQGLSEHVYKFNRVIPHLKVSEDCFFTQEYSVYHDMALNQKKNFN
LISLSTTPHGSLRESLIKFLAEKDTIYQKQYVITLQFVFLSDDEFSQDMLLDYSHNDKDSIKLKF
EKHSISLDSKLVIIENGLEDLPLNFSADEHPNLPHSGMGIIKVQFFPRDSKSDGNNDPVPVDFYF
IELNNLKSIEQFDKSIFKKESAETPIALVLKKLISDTKSFFLLNLNDSKNVNKLLTISEEVQTQL
AKRKKKLT
>4ESVA
MSELFSERIPPQSIEAEQAVLGAVFLDPAALVPASEILIPEDFYRAAHQKIFHAMLRVADRGEPV
DLVTVTAELAASEQLEEIGGVSYLSELADAVPTAANVEYYARIVEEKSVLRRLIRTATSIAQDGY
TREDEIDVLLDEADRKIMEVSQRKHSGAFKNIKDILVQTYDNIEMLHNRDGEITGIPTGFTELDR
MTSGFQRSDLIIVAARPSVGKTAFALNIAQNVATKTNENVAIFSLEMSAQQLVMRMLCAEGNINA
QNLRTGKLTPEDWGKLTMAMGSLSNAGIYIDDTPSIRVSDIRAKCRRLKQESGLGMIVIDYLQLI
QGSGRSKENRQQEVSEISRSLKALARELEVPVIALSQLSRSVEQRQDKRPMMSDIRESGSIEQDA
DIVAFLYRDDYYNKDSENKNIIEIIIAKQRNGPVGTVQLAFIKEYNKFVNLERRFDEAQIPPGA
>4ESEA
SNAMSKVLVLKSSILATSSQSNQLADFFVEQWQAAHAGDQITVRDLAAQPIPVLDGELVGALRPS
GTALTPRQQEALALSDELIAELQANDVIVIAAPMYNFNIPTQLKNYFDMIARAGVTFRYTEKGPE
GLVTGKRAIILTSRGGIHKDTPTDLVVPYLRLFLGFIGITDVEFVFAEGIAYGPEVATKAQADAK
TLLAQVVAA
>2LS8A
MVCPIDWRAFQSNCYFPLTDNKTWAESERNCSGMGAHLMTISTEAEQNFIIQFLDRRLSYFLGLR
DENAKGQWRWVDQTPFNPRRVFWHKNEPDNSQGENCVVLVYNQDKWAWNDVPCNFEASRICKIPG
TTLNAENLYFQSHHHHHHWSHPQFEK
>4ERRA
MGQIFTVQELKERAKVFAKPIGASYQGILDQLDLVHQAKGRDQIAASFELNKKINDYIAEHPTSG
RNQALTQLKEQVTSALGLEHHHHHH
>4EPAA
GAMGQTSQQDESTLVVTASKQSSRSASANNVSSTVVSAPELSDAGVTASDKLPRVLPGLNIENSG
NMLFSTISLRGVSSAQDFYNPAVTLYVDGVPQLSTNTIQALTDVQSVELLRGPQGTLYGKSAQGG
IINIVTQQPDSTPRGYIEGGVSSRDSYRSKFNLSGPIQDGLLYGSVTLLRQVDDGDMINPATGSD
DLGGTRASIGNVKLRLAPDDQPWEMGFAASRECTRATQDAYVGWNDIKGRKLSISDGSPDPYMRR
CTDSQTLSGKYTTDDWVFNLISAWQQQHYSRTFPSGSLIVNMPQRWNQDVQELRAATLGDARTVD
MVFGLYRQNTREKLNSAYDMPTMPYLSSTGYTTAETLAAYSDLTWHLTDRFDIGGGVRFSHDKSS
TQYHGSMLGNPFGDQGKSNDDQVLGQLSAGYMLTDDWRVYTRVAQGYKPSGYNIVPTAGLDAKPF
VAEKSINYELGTRYETADVTLQAATFYTHTKDMQLYSGPVRMQTLSNAGKADATGVELEAKWRFA
PGWSWDINGNVIRSEFTNDSELYHGNRVPFVPRYGAGSSVNGVIDTRYGALMPRLAVNLVGPHYF
DGDNQLRQGTYATLDSSLGWQATERMNISVYVDNLFDRRYRTYGYMNGSSAVAQVNMGRTVGINT
RIDFF
>4EPCA
GSTTSTKPSQPSKPSGGTNNKLTVSANRGVAQIKPTNNGLYTTVYDSKGHKTDQVQKTLSVTKTA

TLGNNKFYLVEDYNSGKKYGWVKQGDVVYNTAKAPVKVNQTYNVKAGSTLYTVPWGTPKQVASKV
SGTGNQTFKATKQQQIDKATYLYGTVNGKSGWISKYYLTTASKPSNPTKPSTNNQLTVTNNSGVA
QINAKNSGLYTTVYDTKGKTTNQIQRTLSVTKAATLGDKKFYLVGDYNTGTNYGWVKQDEVIYNT
AKSPVKINQTYNVKPGVKLHTVPWGTYNQVAGTVSGKGDQTFKATKQQQIDKATYLYGTVNGKSG
WISKYYLTA
>4EOZA
GSNMVKVPECRLADELGGLWENSRFTDCCLCVAGQEFQAHKAILAARSPVFSAMFEHEMEESKKN
RVEINDVEPEVFKEMMCFIYTGKAPNLDKMADDLLAAADKYALERLKVMCEDALCSNLSVENAAE
ILILADLHSADQLKT
>4EODA
MAVTDLSLTNSSLMPTLNPMIQQLALAIAASWQSLPLKPYQLPEDLGYVEGRLEGEKLVIENRCY
QTPQFRKMELELAKVGKGLDILHCVMFPEPLYGLPLFGCDIVAGPGGVSAAIADLSPTQSDRQLP
AAYQKSLAELGQPEFEQQRELPPWGEIFSEYCLFIRPSNVTEEERFVQRVVDFLQIHCHQSIVAE
PLSEAQTLEHRQGQIHYCQQQQKNDKTRRVLEKAFGEAWAERYMSQVLFDVIQ
>4EO1A
PSEQTPEEICEAKPPIDGVFNNVFKGDEGGFYINYNGCEYEATGVTVCQNDGTVCSSSAWKPTGY
VPESG
>4EN6B
QTILPYPNGLYVINKGDGYMRTNDKDLIGTLLIESSTSGSIIQPRLRNTTRPLFNTSNPTIFSQE
YTEARLNDAFNIQLFNTSTTLFKFVEEAPTNKNISMKVYNTYEKYELINYQNGNIDDKAEYYLPS
LGKCEVSDAPSPQAPVVETPVDQDGFIQTGPNENIIVGVINPSENIEEISTPIPDDYTYNIPTSI
QNNACYVLFKVNTTGVYKITTKNNLPPLIIYEAIGSSNRNMNSNNLSNDNIKAIKYITGLNRSDA
KSYLIVSLFKDKNYYIRIPQISSSTTSQLIFKRELGNISDLADSTVNILDNLNTSGTHYYTRQSP
DVGNYISYQLTIPGDFNNIASSIFSFRTRNNQGIGTLYRLTESINGYNLITINNYSDLLNNVEPI
SLLNGATYIFRVKVTELNNYNIIFDAYRNS
>4EMOA
GSMAPPAGGAAAAASDLGSAAVLMAVHAAVRPLGAGPDAEAQLRRLQLSADPERPGRFRLELLGA
GPGAVNLEWPLESVSYTIRGPTQHELQPPPGGPGTLSMHFLNPQEAQRWAVLVRGATVEGQNGS
>4AQ1A
MASFTDVAPQYKDAIDFLVSTGATKGKTETKFGVYDEITRLDAAVILARVLKLDVGNAKDAGFTD
VPKDRAKYVNALVEAGVLNGKAPGKFGAYDPLTRVEMAKIIANAHKLKADDVKLPFTDVNDTWAP
YVKALYKYEVTKGKTPTSFGAYQNITRGDFAQFVYRAVNINAVPEIVEVTAVNSTTVKVTFNTQI
ADVDFTNFAIDNGLTVTKATLSRDKKSVEVVVNKPFTRNQEYTITATGIKNLKGETAKELTGKFV
WSVQDAVTVALNNSSLKVGEESGLTVKDQDGKDVVGAKVELTSSNTNIVVVSSGEVSVSAAKVTA
VKPGTADVTAKVTLPDGVVLTNTFKVTVTEVPVQVQNQGFTLVDNLSNAPQNTVAFNKAEKVTSM
FAGETKTVAMYDTKNGDPETKPVDFKDATVRSLNPIIATAAINGSELLVTANAGQSGKASFEVTF
KDNTKRTFTVDVKKEPVLQDIKVDATSVKLSDEAVGGGEVEGVNQKTIKVSAVDQYGKEIKFGTK
GKVTVTTNTEGLVIKNVNSDNTIDFDSGNSATDQFVVVATKDKIVNGKVEVKYFKNASDTTPTST
KTITVNVVNVKADATPVGLDIVAPSEIDVNAPNTASTADVDFINFESVEIYTLDSNGNRLKKVTP
TATTLVGTNDYVEVNGNVLQFKGNDELTLLTSSSTVNVDVTADGITKRIPVKYINSASVPASATV
ATSPVTVKLNSSDNDLTFEELIFGVIDPTQLVKDEDINEFIAVSKAAKNDGYLYNKPLVTVKDAS
GEVIPTGANVYGLNHDATNGNIWFDEEQAGLAKKFSDVHFDVDFSLANVVKTGSGTVSSSPSLSD
AIQLTNSGDAVSFTLVIKSIYVKGADKDDNNLLAAPVSVNVTVTKGS
>4EMZB

MGGKWSKSSVIGWPAVRERMRRAEPAADGVGAVSRDLEKHGAITSSNTAANNAACAWLEAQEEEE
VGFPVTPQVPLRPMTYKAAVDLSHFLKEKGGLEGLIHSQRRQDILDLWIYHTQGYFPDWQNYTPG
PGVRYPLTFGWCYKLVPVEPDKVEEANKGENTSLLHPVSLHGMDDPEREVLEWRFDSRLAFHHVA
RELHPEYFKNC
>4EMKB
MDSSPNEFLNKVIGKKVLIRLSSGVDYKGILSCLDGYMNLALERTEEYVNGKKTNVYGDAFIRGN
NVLYVSALDD
>4EMCC
QKKRFLPQSVLIKREDEIAFDDFHLDARKVLNDLSATSENPFSSSPNTKKIKSKGKTLEVVPKKK
NKKII
>4ELLA
GEFNTIQQLMMILNSASDQPSENLISYFNNCTVNPKESILKRVKDIGYIFKEKFAKAVGQGCVEI
GSQRYKLGVRLYYRVMESMLKSEEERLSIQNFSKLLNDNIFHMSLLACALEVVMATYSRSTSQNL
DSGTDLSFPWILNVLNLKAFDFYKVIESFIKAEGNLTREMIKHLERCEHRIMESFAWLSDSPLFD
LIKQSKDREGPTDHLESACPLNLPLQNNHTAADMYLEPVRAPKKKGSTTRVNSTANAETQATSAF
QTQKPLKSTSLSLFYKKVYRLAYLRLNTLCERLLSEHPELEHIIWTLFQHTLQNEYELMRDRHLD
QIMMCSMYGICKVKNIDLKFKIIVTAYKDLPHAVQETFKRVLIKEEEYDSIIVFYNSVFMQRLKT
NILQYASTRPPTLAPIPHIPR
>3VRCA
ADLSPEEQIETRQAGYAFMAWNMGKIKANLEGEYNADQVRAAANVVAAIANSGMGALYGPGTDKN
VGAVKTRAKPELFQNLEDVGKLARDLGTAANALAAAAATGEANAVKSAFADVGAACKACHQKYRA
D
>3VR8A
MLRAVRALICRIGARRTLSVSSSRLDVSTSNIAQYKVIDHAYDVVIIGAGGAGLRAAMGLGEAGF
KTAVVTKMFPTRSHTTAAQGGINAALGSMNPDDWKWHFYDTAKGSDWLGDQNAMHYLTRNAVEAV
TELENFGMPFSRTPEGKIYQRSFGGQSNNYGKGGVAKRTCCVADRTGHSMLHTLYGNSLRCHCTF
FIEYFALDLLMDKGRCVGVIALCLEDGTIHRFRSKRTIVATGGYGRAYFSCTTAHMNTGDGTALA
TRAGIALEDLEFIQFHPTGIYGVGCLITEGSRGEGGFLVNSEGERFMERYAPKAKDLASRDVVSR
AETIEIMEGRGVGPEKDHIYLQLHHLPAEQLHQRLPGISETAKIFAGVDVTKEPIPVIPTVHYNM
GGIPTNYKAQVIKYTKEGGDKIVPGLYACGECACHSVHGANRLGANSLLDAVVFGRACSINIKEE
LKPDEKIPELPEGAGEESIANLDAVRYANGDVPTAELRLTMQKTMQKHAGVFRRGDILAEGVKKM
MDLSKELKRLKTTDRSLIWNSDLTESLELQNLMLNATQTIVAAENRKESRGAHARDDFPKREDEY
DYSKPIEGQTKRPFEKHWRKHTLTKQDPRTGHITLDYRPVIDKTLDPAEVDWIPPIIRSY
>4EJOA
SNAMAYDDIVSSMVLELRRGTLVMLVLSQLREPAYGYALVKSLADHGIPIEANTLYPLMRRLESQ
GLLASEWDNGGSKPRKYYRTTDEGLRVLREVEAQWHVLCDGVGKLLETNGEDREHAER
>4EJ7A
MGSSHHHHHHSSGRENLYFQGMSHIQRETSCSRPRLNSNLDADLYGYRWARDNVGQSGATIYRLY
GKPNAPELFLKHGKGSVANDVTDEMVRLNWLTAFMPLPTIKHFIRTPDDAWLLTTAIPGKTAFQV
LEEYPDSGENIVDALAVFLRRLHSIPVCNCPFNSDRVFRLAQAQSRMNNGLVDASDFDDERNGWP
VEQVWKEMHKLLPFSPDSVVTHGDFSLDNLIFDEGKLIGCIDVGRVGIADRYQDLAILWNCLGEF
SPSLQKRLFQKYGIDNPDMNKLQFHLMLDEFF
>4EIYA
MKTIIALSYIFCLVFADYKDDDDGAPPIMGSSVYITVELAIAVLAILGNVLVCWAVWLNSNLQNV

TNYFVVSLAAADIAVGVLAIPFAITISTGFCAACHGCLFIACFVLVLTQSSIFSLLAIAIDRYIA
IRIPLRYNGLVTGTRAKGIIAICWVLSFAIGLTPMLGWNNCGQPKEGKNHSQGCGEGQVACLFED
VVPMNYMVYFNFFACVLVPLLLMLGVYLRIFLAARRQLADLEDNWETLNDNLKVIEKADNAAQVK
DALTKMRAAALDAQKATPPKLEDKSPDSPEMKDFRHGFDILVGQIDDALKLANEGKVKEAQAAAE
QLKTTRNAYIQKYLERARSTLQKEVHAAKSLAIIVGLFALCWLPLHIINCFTFFCPDCSHAPLWL
MYLAIVLSHTNSVVNPFIYAYRIREFRQTFRKIIRSHVLRQQEPFKAHHHHHHHHHH
>2LRKA
AEELEEVVMGLIINSGQARSLAYAALKQAKQGDFAAAKAMMDQSRMALNEAHLVQTKLIEGDAGE
GKMKVSLVLVEAQLHLMTSMLARELITELIELHEKLKA
>2LRKD
MFQQEVTITAPNGLHTRPAAQFVKEAKGFTSEITVTSNGKSASAKSLFKLQTLGLTQGTVVTISA
EGEDEQKAVEHLVKLMAELE
>4EIVA
MHHHHHHENLYFQGGTIYKQFTSRTLLNFFEVAALTDGETNESVAAVCKIAAKDPAIVGVSVRPA
FVRFIRQELVKSAPEVAGIKVCAAVNFPEGTGTPDTVSLEAVGALKDGADEIECLIDWRRMNENV
ADGESRIRLLVSEVKKVVGPKTLKVVLSGGELQGGDIISRAAVAALEGGADFLQTSSGLGATHAT
MFTVHLISIALREYMVRENERIRVEGINREGAAVRCIGIKIEVGDVHMAETADFLMQMIFENGPR
SIVRDKFRVGGGFNLLKELRDCYESWDSVGVSPDTSP
>4APMA
GSAMGSTPKDIWGRYMAKFDLAKSHGSGIYVDLGGTERVGATQHRMPTGKCPVMGKVINLGNNAD
FLNRISAENPQDRGLAFPDTAVAVTRNSNARNRAAAEKTEIILSPVSAADLVRWGYDGNDVANCA
EYAGNIIPASDTATKYRYPFVYDAKEEMCHILFTPMQYNRGSRYCDNDGSQDEGTSSLLCMEPMK
SGIDAHLYYGSSRVDKKWEENCPMYPVKDAIFGRGANGSCVAIESAFEEFTRDAEECSALMFENA
AADLEIDEEADNFDELKTLSDGLRNIKASKIAQALFSPIAKAGTSAKNSKGVGMNWANYDSNTGL
CRVIEETPNCLIIDAGSFAMTAVGSPLEQDAVPFPCDIVTNGYIEPRPRSRHRNTTPIFEVTTAL
SREALKCSKYVHEKYSESCGTYYYCSEEKPSSWAFWRNLDAAALVPR
>2LRJA
GSSISHSGNLYTAGQCTWYVYDKVGGEIGSTWGNANNWAAAAQGAGFTVNHTPSKGAILQSSEGP
FGHVAYVESVNSDGSVTISEMNYSGGPFSVSSRTISASEAGNYNYIHI
>4EFOA
GPLGSTSDILHRMVIHVFSLQQMTAHKIYIHSYNTATIFHELVYKQTKIISSNQELIYEGRRLVL
EPGRLAQHFPKTTEENPIFVVSLERPHRD
>4EFZA
GPGSMTVEGFFDPATCTISYLLFDSGSGECALIDSVLDYDPKSGRTRTASADQLIARVAALGARV
RWLLETHVHADHLSAAPYLKTRVGGEIAIGRHVTRVQDVFGKLFNAGPAFAHDGSQFDRLLDDGD
TLALGALSIRAMHTPGHTPACMTYVVTEAHAAHDARDAAAFVGDTLFMPDYGTARCDFPGGDARS
LYRSIRKVLSLPPATRLYMCHDYQPNGRAIQYASTVADELRENVHIREGVTEDDFVAMRTARDAT
LDMPVLMLPSVQVNMRAGRLPEPEDNGVRYLKIPLDAI
>4AOWA
MHHHHHHSSGVDLGTENLYFQSMMTEQMTLRGTLKGHNGWVTQIATTPQFPDMILSASRDKTIIM
WKLTRDETNYGIPQRALRGHSHFVSDVVISSDGQFALSGSWDGTLRLWDLTTGTTTRRFVGHTKD
VLSVAFSSDNRQIVSGSRDKTIKLWNTLGVCKYTVQDESHSEWVSCVRFSPNSSNPIIVSCGWDK
LVKVWNLANCKLKTNHIGHTGYLNTVTVSPDGSLCASGGKDGQAMLWDLNEGKHLYTLDGGDIIN
ALCFSPNRYWLCAATGPSIKIWDLEGKIIVDELKQEVISTSSKAEPPQCTSLAWSADGQTLFAGY

TDNLVRVWQVTIGTR
>4EFIA
MSSPDFSAGRELRTQGARIAGVVSCVPSKQVDNDYFVERFDASAVRDVVKMIGVNRRRWADAQTS
AGDLCRKAGEKLLAGLGWQADSIDALIFVSQTPNYRLPATAFVLQAELDLPASCLALDINLGCSG
YPQALWLGMNLIQTGAAKRVLLAVGDTISKMIDPTDRSTSLLFGDAGTMTALETSNGDAAAHFII
GADGKGARNLIVPSGGFKPYDAAADERMAGKSPECLFMDGGEIFNFTLNAVPKLVSRTLDIAGRD
KDSYDAFLFHQANLFMLKHLAKKAGLPAERVPVNIGEYGNTSCASIPLLITTELKDRLKEETLQL
GMFGFGVGYSWASAALAVGPLNIVDTIET
>4EEEA
MECVKTRSVNIHVPVKETSKVVLECRGDSYFRHFSYVYWIIGKNKTVDQLPPNSGYRERIYLFKK
PHRCENRPRADLILTNITDEMRNEKLTCVLIDPKDPLKESVILSKIWNCVYKI
>2LRDA
AMGKCSVLKKVACAAAIAGAVAACGGIDLPCVLAALKAAEGCASCFCEDHCHGVCKDLHLC
>4EEIA
MIKRYDVAEISKIWADENKYAKMLEVELAILEALEDRMVPKGTAAEIRARAQIRPERVDEIEKVT
KHDIIAFCTSIAEQFTAETGKFFHFGVTSSDIIDSALSLQIRDSMSYVIKDLEALCDSLLTKAEE
TKEIITMGRSHGMFAEPMSFGQKFLGAYVEFKRRLKDLKDFQKDGLTVQFSGAVGNYCILTTEDE
KKAADILGLPVEEVSTQVIPRDRIAKLISIHGLIASAIERLAVEIRHLHRSDVFEVYEGFSKGQK
GSSTMPHKKNPISTENLTGMARMLRSHVSIALENCVLWHERDISHSSAERFYLPDNFGIMVYALR
RMKNTIDNLVVQRDIIEDRVRSTSAYLSSFYLHFLVANTPFMREDCYKIVQQVAFDLKQGESFSK
KLQKVMHDEHNIILDIPEMDFEGIKKTYLKEIDHVFDRSVKARGENLY
>4EDFA
MFEIKKICCIGAGYVGGPTCSVIAHMCPEIRVTVVDVNESRINAWNSPTLPIYEPGLKEVVESCR
GKNLFFSTNIDDAIKEADLVFISVNTPTETYGMGKGRAADLKYIEACARRIVQNSNGYKIVTEKS
TVPVRAAESIRRIFDANTKPNLNLQVLSNPEFLAEGTAIKDLKNPDRVLIGGDETPEGQRAVQAL
CAVYEHWVPREKILTTNTWSSELSKLAANAFLAQRISSINSISALCEATGADVEEVATAIGMDQR
IGNKFLKASVGFGGSCFQKDVLNLVYLCEALNLPEVARYWQQVIDMNDYQRRRFASRIIDSLFNT
VTDKKIAILGFAFKKDTGDTRESSSIYISKYLMDEGAHLHIYDPKVPREQIVVDLSHPGVSEDDQ
VSRLVTISKDPYEACDGAHAVVICTEWDMFKELDYERIHKKMLKPAFIFDGRRVLDGLHNELQTI
GFQIETIGKKVSSKRIPYAPSGEIPKFSLQDPPNKKPKV
>3VQKA
MYYLGKELQKRSEELSRGFYELVYPPVDMYEEGGYLVVVADLAGFNKEKIKARVSGQNELIIEAE
REITEPGVKYLTQRPKYVRKVIRLPYNVAKDAEISGKYENGVLTIRIPIAGTSVIKIE
>4EBBA
PDPGFQERFFQQRLDHFNFERFGNKTFPQRFLVSDRFWVRGEGPIFFYTGNEGDVWAFANNSAFV
AELAAERGALLVFAEHRYYGKSLPFGAQSTQRGHTELLTVEQALADFAELLRALRRDLGAQDAPA
IAFGGSYGGMLSAYLRMKYPHLVAGALAASAPVLAVAGLGDSNQFFRDVTADFEGQSPKCTQGVR
EAFRQIKDLFLQGAYDTVRWEFGTCQPLSDEKDLTQLFMFARNAFTVLAMMDYPYPTDFLGPLPA
NPVKVGCDRLLSEAQRITGLRALAGLVYNASGSEHCYDIYRLYHSCADPTGCGTGPDARAWDYQA
CTEINLTFASNNVTDMFPDLPFTDELRQRYCLDTWGVWPRPDWLLTSFWGGDLRAASNIIFSNGN
LDPWAGGGIRRNLSASVIAVTIQGGAHHLDLRASHPEDPASVVEARKLEATIIGEWVKAARREQQ
PALRGGPRLSLENLYFQ
>4EAZA
DPNSMRSIASSKLWMLEFSAFLERQQDPDTYNKHLFVHISQSSPSYSDPYLETVDIRQIYDKFPE

KKGGLKELFERGPSNAFFLVKFWADLNTNIDDEGSAFYGVSSQYESPENMIITCSTKVCSFGKQV
VEKVETEYARYENGHYLYRIHRSPLCEYMINFIHKLKHLPEKYMMNSVLENFTILQVVTNRDTQE
TLLCIAYVFEVSASEHGAQHHIYRLVKE
>4EAGC
MESVAAESAPAPENEHSQETPESNSSVYTTFMKSHRCYDLIPTSSKLVVFDTSLQVKKAFFALVT
NGVRAAPLWDSKKQSFVGMLTITDFINILHRYYKSALVQIYELEEHKIETWREVYLQDSFKPLVC
ISPNASLFDAVSSLIRNKIHRLPVIDPESGNTLYILTHKRILKFLKLFITEFPKPEFMSKSLEEL
QIGTYANIAMVRTTTPVYVALGIFVQHRVSALPVVDEKGRVVDIYSKFDVINLAAEKTYNNLDVS
VTKALQHRSHYFEGVLKCYLHETLEAIINRLVEAEVHRLVVVDEHDVVKGIVSLSDILQALVLTG
GEKKP
>4E8JA
MKNNNVTEKELFYILDLFEHMKVTYWLDGGWGVDVLTGKQQREHRDIDIDFDAQHTQKVIQKLED
IGYKIEVHWMPSRMELKHEEYGYLDIHPINLNDDGSITQANPEGGNYVFQNDWFSETNYKDRKIP
CISKEAQLLFHSGYDLTETDHFDIKNLKSIT
>4E88A
MGGRALRVLVDMDGVLADVEGGLLRKFRARFPDQPFIALEDRRGYKACEQYGRLRPGLSEKARSI
AESKNFFFELEPLPGAVEAVKEMASLQNTDVFICTSPHKMFKYCPYEKYAWVEKYFGPDFLEQIV
LTRDKTVVSADLLIDDRPDITGAEPTPSWEHVLFTACHNQHLQLQPPRRRLHSWADDWKAILDSK
RPCGSLEHHHHHH
>4AN6A
DYTVHDTDGKPVLNNAGQYYILPAKQGKGGGLGLSNDDDGNCPLTVSQTPIDLPIGLPVRFSSRA
RISHITTALSLNIEFTIAPACAPKPARWRIFNEQSSEKGYTPVKISDDFSSAAPFQIKKFEEDYK
LVYCSKSESGERKCVDLGIKIDDEKNRRLVLKEGDPFKVKFKKVDEESSEEWSIV
>4E4TA
MAHHHHHHMGTLEAQTQGPGSMTATPDSVSPILPGAWLGMVGGGQLGRMFCFAAQSMGYRVAVLD
PDPASPAGAVADRHLRAAYDDEAALAELAGLCEAVSTEFENVPAASLDFLARTTFVAPAGRCVAV
AQDRIAEKRFIEASGVPVAPHVVIESAAALAALDDAALDAVLPGILKTARLGYDGKGQVRVSTAR
EARDAHAALGGVPCVLEKRLPLKYEVSALIARGADGRSAAFPLAQNVHHNGILALTIVPAPAADT
ARVEEAQQAAVRIADTLGYVGVLCVEFFVLEDGSFVANEMAPRPHNSGHYTVDACATSQFEQQVR
AMTRMPLGNPRQHSPAAMLNILGDVWFPNGAAAGAVTPPWDTVAAMPAAHLHLYGKEEARVGRKM
GHVNFTAEMRDDAVAAATACAQLLRVPLD
>4E51A
MAHHHHHHMGTLEAQTQGPGSMTEQKRKLEKLTGVKGMNDILPQDAGLWEFFEATVKSLLRAYGY
QNIRTPIVEHTPLFTRGIGEVTDIVEKEMYSFVDALNGENLTLRPENTAAVVRAAIEHNMLYDGP
KRLWYIGPMFRHERPQRGRYRQFHQVGVEALGFAGPDADAEIVMMCQRLWEDLGLTGIKLEINSL
GLAEERAAHRVELIKYLEQHADKLDDDAQRRLYTNPLRVLDTKNPALQEIVRNAPKLIDFLGDVS
RAHFEGLQRLLKANNVPFTINPRLVRGLDYYNLTVFEWVTDKLGAQGTVAAGGRYDPLIEQLGGK
PTAACGWAMGIERILELLKEEHLVPEQEGVDVYVVHQGDAAREQAFIVAERLRDTGLDVILHCSA
DGAGASFKSQMKRADASGAAFAVIFGEDEVTNGTASVKPLRGTGDDGEKSVQQSVPVESLTEFLI
NAMVATAEDGDD
>2LQOA
MVTAALTIYTTSWCGYCLRLKTALTANRIAYDEVDIEHNRAAAEFVGSVNGGNRTVPTVKFADGS
TLTNPSADEVKAKLVKIAGLEHHHHHH
>4E1YA

GSHPFDQAVVKDPTASYVDVKARRTFLQSGQLDDRLKAALPKEYDCTTEATPNPQQGEMVIPRRY
LSGNHGPVNPDYEPVVTLYRDFEKISATLGNLYVATGKPVYATCLLNMLDKWAKADALLNYDPKS
QSWYQVEWSAATAAFALSTMMAEPNVDTAQRERVVKWLNRVARHQTSFPGGDTSCCNNASYWRGQ
EATIIGVISKDDELFRWGLGRYVQAMGLINEDGSFVHEMTRHEQSLHYQNYAMLPLTMIAETASR
QGIDLYAYKENGRDIHSARKFVFAAVKNPDLIKKYASEPQDTRAFKPGRGDLNWIEYQRARFGFA
DELGFMTVPIFDPRTGGSGTLLAYKPQG

>4E1JA

MHHHHHHSSGVDLGTENLYFQSMMGGYILAIDQGTTSTRAIVFDGNQKIAGVGQKEFKQHFPKSG
WVEHDPEEIWQTVVSTVKEAIEKSGITANDIAAIGITNQRETVVVWDRETGKPIHNAIVWQDRRT
AAFCDKLKKKGLEKTFVKKTGLLLDPYFSGTKLNWLLSNVKGAQVRAAKGELCFGTIDTFLIWRL
TGGECFCTDATNASRTLLYNIAENAWDDELTEVLRVPKEMLPEVKDCAADFGVTDPSLFGAAIPI
LGVAGDQQAATIGQACFKPGMLKSTYGTGCFALLNTGKDMVRSKNRLLTTIAYRLDGETTYALEG
SIFVAGAAVQWLRDGLKVIKAAPDTGSLAESADPSQEVYLVPAFTGLGAPHWDPDARGAIFGMTR
NTGPAEFARAALEAVCYQTRDLLEAMHKDWRRNGNDTVLRVDGGMVASDWTMQRLSDLLDAPVDR
PVILETTALGVAWLAGSRAGVWPNQEAFAKSWARDRRFEPHMDEATRKVKLKGWRSAVKRTLIAA

>4E0VA

MNVFFMFSKPGKLADDRNPLEECFRETDYEEFLEIAKNGLSTTSNPKRVVIVGAGMSGLSAAYVL
ANAGHQVTVLEASERAGGQVKTYRNEKEGWYANLGPMRLPEKHRIVREYIRKFGLQLNEFSQENE
NAWYFIKNIRKRVGEVNKDPGVLDYPVKPSEVGKSAGQLYEESLQKAVEELRRTNCSYMLNKYDT
YSTKEYLLKEGNLSPGAVDMIGDLLNEDSGYYVSFIESLKHDDIFAYEKRFDEIVGGMDKLPTSM
YQAIQEKVHLNARVIKIQQDVKEVTVTYQTSEKETLSVTADYVIVCTTSRAARRIKFEPPLPPKK
AHALRSVHYRSGTKIFLTCTKKFWEDDGIHGGKSTTDLPSRFIYYPNHNFPNGVGVIIAYGIGDD
ANYFEALDFEDCGDIVINDLSLIHQLPKEEIQAICRPSMIQRWSLDKYAMGGITTFTPYQFQHFS
EALTAPVDRIYFAGEYTAQAHGWIASTIKSGPEGLDVNRASE

>4E0IA

MKAIDKMTDNPPQEGLSGRKIIYDEDGKPSRSCNTLLDFQYVTGKISNGLKNLSSNGKLAGTGAL
TGEASELMPGSRTYRKVDPPDVEQLGRSSWTLLHSVAASYPAQPTDQQKGEMKQFLNIFSHIYPC
NWSAKDFEKYIRENAPQVESREELGRWMCEAHNKVNKKLRKPKFDCNFWEKRWKDGWDE

>4DYLA

SMGFSSELCSPQGHGVLQQMQEAELRLLEGMRKWMAQRVKSDREYAGLLHHMSLQDSGGQSRAIS
PDSPISQSWAEITSQTEGLSRLLRQHAEDLNSGPLSKLSLLIRERQQLRKTYSEQWQQLQQELTK
THSQDIEKLKSQYRALARDSAQAKRKYQEASKDKDRDKAKDKYVRSLWKLFAHHNRYVLGVRAAQ
LHHQHHHQLLLPGLLRSLQDLHEEMACILKEILQEYLEISSLVQDEVVAIHREMAAAAARIQPEA
EYQGFLRQYGSAPDVPPCVTFDESLLEEGEPLEPGELQLNELTVESVQHTLTSVTDELAVATEMV
FRRQEMVTQLQQELRNEEENTHPRERVQLLGKRQVLQEALQGLQVALCSQAKLQAQQELLQTKLE
HLGPGEPPPVLLLQDD

>4DXRA

GPGGSGGVTEEQVHHIVKQALQRYSEDRIGLADYALESGGASVISTRCSETYETKTALLSLFGIP
LWYHSQSPRVILQPDVHPGNCWAFQGPQGFAVVRLSARIRPTAVTLEHVPKALSPNSTISSAPKD
FAIFGFDEDLQQEGTLLGKFTYDQDGEPIQTFHFQAPTMATYQVVELRILTNWGHPEYTCIYRFR
VHGEPAH

>3VP7A

INIFNATFKISHSGPFATINGLRLGSIPESVVPWKEINAALGQLILLLATINKNLKINLVDYELQ
PMGSFSKIKKRMVNSVEYNNSTTNAPGDWLILPVYYDENFNLGRIFRKETKFDKSLETTLEIISE

ITRQLSTIASSYSSQTLTTSQDESSMNNANDVENSTSILELPYIMNKDKINGLSVKLHGSSPNLE
WTTAMKFLLTNVKWLLAFSSNLLSK
>4DXDA
MHHHHHHLEFEQGFNHLATLKVIGVGGGGNNAVNRMIDHGMNNVEFIAINTDGQALNLSKAESKI
QIGEKLTRGLGAGANPEIGKKAAEESREQIEDAIQGADMVFVTSGMGGGTGTGAAPVVAKIAKEM
GALTVGVVTRPFSFEGRKRQTQAAAGVEAMKAAVDTLIVIPNDRLLDIVDKSTPMMEAFKEADNV
LRQGVQGISDLIAVSGEVNLDFADVKTIMSNQGSALMGIGVSSGENRAVEAAKKAISSPLLETSI
VGAQGVLMNITGGESLSLFEAQEAADIVQDAADEDVNMIFGTVINPELQDEIVVTVIATGFDDKP
TSHGRKSGSTGFGTSVNTSSNATSKDESFTSNSSNAQATDSVSERTHTTKEDDIPSFIRNREERR
SRRTRR
>4DX1A
MFNRPILFDIVSRGSTADLDGLLPFLLTHKKRLTDEEFREPSTGKTCLPKALLNLSNGRNDTIPV
LLDIAERTGNMREFINSPFRDIYYRGQTALHIAIERRCKHYVELLVAQGADVHAQARGRFFQPKD
EGGYFYFGELPLSLAACTNQPHIVNYLTENPHKKADMRRQDSRGNTVLHALVAIADNTRENTKFV
TKMYDLLLLKCARLFPDSNLEAVLNNDGLSPLMMAAKTGKIGIFQHIIRREVTDEAAAHHHHHH
>4AKKA
MRGSHHHHHHTDPHASSVPGRGSIEGRMNNMAGNTPEVVDWFARARRLQKQQLHQLAQQGTLAGQ
ISALVHMLQCERGASNIWLCSGGRLYAAECRAGAALVDEQLTRFYAALEPARDAASSALCWRIAC
AVWYLPQLAALRKRVRDREIAAEEATGQFSRIIRHLLNIVPQLNDSIDDPQIAGRMVALYSFMQG
KELAGQERALGALGFARGQFSDELRQQLVDRIDGQQPCFDSFQALAQPPQTALFAEQCQASLEIE
QLRRVACTRQPPADEGETALRWFCAQTQRLEQLRGVEELLIVDLLNAADALLEGEEPEAQLPPAD
WQEDSIALRLDKQLLPLVRQQAHELQQLSGQLASLKDALEERKLIEKAKSVLMTYQGMQEEQAWQ
ALRKMAMDKNQRMVEIARALLTVKALWRVTPKE
>4DW0A
GSSKKVGTLNRFTQALVIAYVIGYVFVYNKGYQDTDTVLSSVTTKVKGIALTKTSELGERIWDVA
DYIIPPQEDGSFFVLTNMIITTNQTQSKCAENPTPASTCTSHRDCKRGFNDARGDGVRTGRCVSY
SASVKTCEVLSWCPLEKIVDPPNPPLLADAERFTVLIKNNIRYPKFNFNKRNILPNINSSYLTHC
VFSRKTDPDCPIFRLGDIVGEAEEDFQIMAVRGGVMGVQIRWDCDLDMPQSWCVPRYTFRRLDNK
DPDNNVAPGYNFRFAKYYKNSDGTETRTLIKGYGIRFDVMVFGQAGKFNIIPTLLNIGAGLALLG
LVNVICDWIVLTFMK
>4DT4A
MGSSHHHHHHSSGLVPRGSHMSESVQSNSAVLVHFTLKLDDGTTAESTRNNGKPALFRLGDASLS
EGLEQHLLGLKVGDKTTFSLEPDAAFGVPSPDLIQYFSRREFMDAGEPEIGAIMLFTAMDGSEMP
GVIREINGDSITVDFNHPLAGQTVHFDIEVLEIDPALEA
>2LPIA
GSGNSHTTPWTNPGLAENFMNSFMQGLSSMPGFTASQLDDMSTIAQSMVQSIQSLAAQGRTSPNK
LQALNMRFASSMAEIAASEEGGGSLSTKTSSIASAMSNAFLQTTGVVNQPFINEITQLVSMFAQA
GMNDVSA
>4DPPA
MHHHHHHGLPIPNPLLGLDSTENLYFQGIDPFTAAVVPNFHLPMRSLEVKNRTNTDDIKALRVIT
AIKTPYLPDGRFDLEAYDDLVNIQIQNGAEGVIVGGTTGEGQLMSWDEHIMLIGHTVNCFGGSIK
VIGNTGSNSTREAIHATEQGFAVGMHAALHINPYYGKTSIEGLIAHFQSVLHMGPTIIYNVPGRT
GQDIPPRAIFKLSQNPNLAGVKECVGNKRVEEYTENGVVVWSGNDDECHDSRWDYGATGVISVTS
NLVPGLMRKLMFEGRNSSLNSKLLPLMAWLFHEPNPIGINTALAQLGVSRPVFRLPYVPLPLSKR

LEFVKLVKEIGREHFVGEKDVQALDDDDFILIGRY
>4DOTA
MRAPIPEPKPGDLIEIFRPFYRHWAIYVGDGYVVHLAPPSEVAGAGAASVMSALTDKAIVKKELL
YDVAGSDKYQVNNKHDDKYSPLPCSKIIQRAEELVGQEVLYKLTSENCEHFVNELRYGVARSDQV
RDLEHHHHHH
>4DOJA
LENPTNLEGKLADAEEEIILEGEDTQASLNWSVIVPALVIVLATVVWGIGFKDSFTNFASSALSA
VVDNLGWAFILFGTVFVFFIVVIAASKFGTIRLGRIDEAPEFRTVSWISMMFAAGMGIDLMFYGT
TEPLTFYRNGVPGHDEHNVGVAMSTTMFHWTLHPWAIYAIVGLAIAYSTFRVGRKQLLSSAFVPL
IGEKGAEGWLGKLIDILAIIATVFGTACSLGLGALQIGAGLSAANIIEDPSDWTIVGIVSVLTLA
FIFSAISGVGKGIQYLSNANMVLAALLAIFVFVVGPTVSILNLLPGSIGNYLSNFFQMAGRTAMS
ADGTAGEWLGSWTIFYWAWWISWSPFVGMFLARISRGRSIREFILGVLLVPAGVSTVWFSIFGGT
AIVFEQNGESIWGDGAAEEQLFGLLHALPGGQIMGIIAMILLGTFFITSADSASTVMGTMSQHGQ
LEANKWVTAAWGVATAAIGLTLLLSGGDNALSNLQNVTIVAATPFLFVVIGLMFALVKDLSNDVI
YLEYREQQRFNARLARERRVHNEHRKRELAAKRRRERKASGAGKRR
>4AIEA
MASASWWKNAVVYQVYPKSFQDSNGDGIGDLQGIISRLDYLEKLGIDAIWLSPVYQSPGVDNGYD
ISDYEAIDPQYGTMADMDELISKAKEHHIKIVMDLVVNHTSDQHKWFVEAKKGKDNQYRDYYIWR
DPVDEHEPNDLKSAFSGSAWKYDERSGQYYLHFFADQQPDLNWQNTELRQKIYNMMNFWLDKGIG
GFRMDVIELIGKDPDKNIRENGPMLHPYLQEMNKATFGKRDVMTVGETWNATPKIAEEYSDPDRH
ELSMVFQFENQSLDQQPGKEKWDLKPLDLGELKKVLVKWQTKIDFDHAWNSLFWENHDIPRVISR
WGNDQEYRVQCAKMFAIILHMMHGTPYIFNGEEIGMTNCPVKNIDEVEDIESINMYNERLAEGYD
EEELIHAINVKGRDNARRPMQWNDEKNAGFSEVDPWLSVNPNYKDINVENALADPNSIFYTYQKL
IKLRHENPIVVDGDFSLVSNTQDAVLAYYRILNDKKWLVVANLSNEEQNFVSNDQIETILSNYPE
RNNVQNITLKPYEAFISKVIELEHHHHHH
>4DMUB
APDCSQPLDVILLLDGSSSFPASYFDEMKSFAKAFISKANIGPRLTQVSVLQYGSITTIDVPWNV
VPEKAHLLSLVDVMQREGGPSQIGDALGFAVRYLTSEMHGARPGASKAVVILVTDVSVDSVDAAA
DAARSNRVTVFPIGIGDRYDAAQLRILAGPAGDSNVVKLQRIEDLPTMVTLGNSFLHKLCSG
>4DM3A
MSGADRSPNAGAAPDSAPGQAAVASAYQRFEPRAYLRNNYAPPRGDLCNPNGVGPWKLRCLAQTF
ATGEVSGRTLIDIGSGPTVYQLLSACSHFEDITMTDFLEVNRQELGRWLQEEPGAFNWSMYSQHA
CLIEGKGECWQDKERQLRARVKRVLPIDVHQPQPLGAGSPAPLPADALVSAFCLEAVSPDLASFQ
RALDHITTLLRPGGHLLLIGALEESWYLAGEARLTVVPVSEEEVREALVRSGYKVRDLRTYIMPA
HLQTGVDDVKGVFFAWAQKVGLEHHHHHH
>4DLFA
MGALRIDSHQHFWRYRAADYPWIGAGMGVLARDYLPDALHPLMHAQALGASIAVQARAGRDETAF
LLELACDEARIAAVVGWEDLRAPQLAERVAEWRGTKLRGFRHQLQDEADVRAFVDDADFARGVAW
LQANDYVYDVLVFERQLPDVQAFCARHDAHWLVLDHAGKPALAEFDRDDTALARWRAALRELAAL
PHVVCKLSGLVTEADWRRGLRASDLRHIEQCLDAALDAFGPQRLMFGSDWPVCLLAASYDEVASL
VERWAESRLSAAERSALWGGTAARCYALPEPADARLAENLYFQ
>4DJSA
LATRAIPELTKLLNDEDQVVVNKAAVMVHQLSKKEASRHAIMRSPQMVSAIVRTMQNTNDVETAR
CTAGTLHNLSHHREGLLAIFKSGGIPALVKMLGSPVDSVLFYAITTLHNLLLHQEGAKMAVRLAG

GLQKMVALLNKTNVKFLAITTDCLQILAYGNQESKLIIILASGGPQALVNIMRTYTYEKLLWTTSR
VLKVLSVCSSNKPAIVEAGGMQALGLHLTDPSQRLVQNCLWTLRNLSDAATKQEGMEGLLGTLVQ
LLGSDDINVVTCAAGILSNLTCNNYKNKMMVCQVGGIEALVRTVLRAGDREDITEPAICALRHLT
SRHQEAEMAQNAVRLHYGLPVVVKLLHPPSHWPLIKATVGLIRNLALCPANHAPLREQGAIPRLV
QLLVRAHQDTQRRTSMGGTQQQFVEGVRMEEIVEGCTGALHILARDVHNRIVIRGLNTIPLFVQL
LYSPIENIQRVAAGVLCELAQDKEAAEAIEAEGATAPLTELLHSRNEGVATYAAAVLFRMSED
>4DJTA
GPGSMERRELTYKICLIGDGGVGKTTYINRVLDGRFEKNYNATVGAVNHPVTFLDDQGNVIKFNV
WDTAGQEKKAVLKDVYYIGASGAILFFDVTSRITCQNLARWVKEFQAVVGNEAPIVVCANKIDIK
NRQKISKKLVMEVLKGKNYEYFEISAKTAHNFGLPFLHLARIFTGRPDLIFVSNVNLEPTEVNYD
YHSPEESKYIDYMEQASKMAPEE
>4DJBA
MGSSHHHHHHSQDPMIRCLRLKVEGALEQIFTMAGLNIRDLLRDILRRWRDENYLGMVEGAGMFI
EEIHPEGFSLYVHLDVRAVSLLEAIVQHLTEAIISSLAVEFDHATGGERVHLIDLHFEVLDNLLE
>4DIXA
GPSSSKSEENISLVYEIDGTEALGSCLRVRPCSNDAPDLSKCTIQWYRSSSDGSKKELISGATKS
VYAPEPFDVGRVLHADIIYDGHSLSLSTVGKIDPAAGLGSYVEALVRKHDVDFNVVVTQMSGEDH
TSESIHLFHVGKMRIKLCKGKTVIAKEYYSSAMQLCGVRGGGNAAAQALYWQAKKGVSFVIAFES
ERERNAAIMLARRFACDCNVTLAGPEDRTETGQSP
>4DIPA
YFQSMGALIPEPEVKIEVLQKPFICHRKTKGGDLMLVHYEGYLEKDGSLFHSTHKHNNGQPIWFT
LGILEALKGWDQGLKGMCVGEKRKLIIPPALGYGKEGKGKIPPESTLIFNIDLLEIRNGP
>4DIQA
LRRRYTMASGPQVDNTGGEPAWDSPLRRVLAELNRIPSSRRRAARLFEWLIAPMPPDHFYRRLWE
REAVLVRRQDHTYYQGLFSTADLDSMLRNEEVQFGQHLDAARYINGRRETLNPPGRALPAAAWSL
YQAGCSLRLLCPQAFSTTVWQFLAVLQEQFGSMAGSNVYLTPPNSQGFAPHYDDIEAFVLQLEGR
KLWRVYRPRAPTEELALTSSPNFSQDDLGEPVLQTVLEPGDLLYFPRGFIHQAECQDGVHSLHLT
LSTYQRNTWGDFLEAILPLAVQAAMEENVEFRRGLPRDFMDYMGAQHSDSKDPRRTAFMEKVRVL
VARLGHFAPVDAVADQRAKDFIHDSLPPVLTDRERALSVYGLPIRWEAGEPVNVGAQLTTETEVH
MLQDGIARLVGEGGHLFLYYTVENSRVYHLEEPKCLEIYPQQADAMELLLGSYPEFVRVGDLPCD
SVEDQLSLATTLYDKGLLLTKMPLALNAENLYFQ
>2LOYA
MLIYKDIFTDDELSSDSFPMKLVDDLVYEFKGKHVVRKEGEIVLAGSNPSAEEGAEDDGSDEHVE
RGIDIVLNHKLVEMNCYEDASMFKAYIKKFMKNVIDHMEKNNRDKADVDAFKKKIQGWVVSLLAK
DRFKNLAFFIGERAAEGAENGQVAIIEYRDVDGTEVPTLMLVKEAIIEEKCLEHHHHHH
>2LORA
MVNLGLSRVDDAVAAKHPGLGEYAACQSHAFMKGVFTFVTGTGMAFGLQMFIQRKFPYPLQWSLL
VAVVAGSVVSYGVTRVESEKCNNLWLFLETGQLPKDRSTDQRS
>2LONA
MSANRRWWVPPDDEDCVSEKLLRKTRESPLVPIGLGGCLVVAAYRIYRLRSRGSTKMSIHLIHTR
VAAQACAVGAIMLGAVYTMYSDYVKRMAQDAGEK
>4DG8A
GHMDSFFRKKAIVRMSQNSLLDLYAHPTVVARFSEMAALHPHREAIRDRFGSVDYRQLLDSAEQL
SDYLLEHYPQPGVCLGVYGEYSRESITCLLAILLSGHHYLYIDLKQPAAWNAELCRQVDCRLILD

CSTTPTPANGLPCVPVRHLPAAPASVARPCFAADQIAYINFSSGTTGRPKAIACTHAGITRLCLG
QSFLAFAPQMRFLVNSPLSFDAATLEIWGALLNGGCCVLNDLGPLDPGVLRQLIGERGADSAWLT
ASLFNTLVDLDPDCLGGLRQLLTGGDILSVPHVRRALLRHPRLHLVNGYGPTENTTFTCCHVVTD
DDLEEDDIPIGKAIAGTAVLLLDEHGQEIAEPDRAGEIVAFGAGLAQGYRNDAARTRASFVELPY
RGRLLRAYRTGDRARYDEQGRLRFIGRGDGQVKLNGYRLDLPALEQRFRRQPGILDCALLVRERN
GVKQLLCAWTGKADASPQALLRQLPTWQRPHACVRVEALPLTAHGKLDRAALLRRLEEPLERCAS
ALDPDQRGCAQLWSELLGCEVGAADQDFFLCGGNSLLALQLVALCQSAGAGANLGLADLQANSRL
DQFSRLLRSHGLAPERLLERAATPEQPLVLSRSAA
>2LOEA
EKVKGCDFTTSESTIFSKGYSINEISNKSSNNQQDIVCTVKAHANDLIGFKCPSNYSVEPHDCFV
SAFNLSGKNENLENKLKLTNIIMDHYNNTFYSRLPSLISDNWKFFCVCSKDNEKKLVFTVEA
>4DEPB
EPLEADKCKEREEKIILVSSANEIDVRPCPLNPNEHKGTITWYKDDSKTPVSTEQASRIHQHKEK
LWFVPAKVEDSGHYYCVVRNSSYCLRIKISAKFVENEPNLCYNAQAIFKQKLPVAGDGGLVCPYM
EFFKNENNELPKLQWYKDCKPLLLDNIHFSGVKDRLIVMNVAEKHRGNYTCHASYTYLGKQYPIT
RVIEFITLEENKPTRPVIVSPANETMEVDLGSQIQLICNVTGQLSDIAYWKWNGSVIDEDDPVLG
EDYYSVENPANKRRSTLITVLNISEIESRFYKHPFTCFAKNTHGIDAAYIQLIYPVTNFQK
>2LOBA
HHHHHHHHHHSSGHIEGRHMENLYFQGIRKVLLLKEDHEGLGISITGGKEHGVPILISEIHPGQP
ADRCGGLHVGDAILAVNGVNLRDTKHKEAVTILSQQRGEIEFEVVYV
>4AFIA
GSPFYIKSSPSPQKRYQDTPGVEHIPVVQIDLSVPLKVPGLPMSDQYVKLEEAMAILFAVVARGT
TILAKHAWCGGNFLEVTEQILAKIPSENNKLTYSHGNYLFHYICQDRIVYLCITDDDFERSRAFS
FLNEVKKRFQTTYGSRAQTALPYAMNSEFSSVLAAQLKHHSEN
>4DCNC
GSRTVDLELELQIELLRETKRKYESVLQLGRALTAHLYSLLQTQHALGDAFADLSQKSPELQEEF
GYNAETQKLLCKNGETLLGAVNFFVSSINTLVTKTMEDTLMTVKQYEAARLEYDAYRTDLEELSL
GPRDAGTRGRLESAQATFQAHRDKYEKLRGDVAIKLKFLEENKIKVMHKQLLLFHNAVSAYFAGN
QKQ
>3VO1A
MVSTTETAEAEPVKKLEKVSKKQEEGLVTNKYKPKEPYVGRCLLNTRITGDQAPGETWHMVFSTE
GEVPYREGQSIGVIADGEDKNGKPHKLRLYSIASSALGDFGDSKTVSLCVKRLVYTNDQGEVVKG
VCSNFLCDLKPGAEVKITGPVGKEMLMPKDPNATIIMLATGTGIAPFRSFLWKMFFEEHEDYKYT
GLAWLFLGVPTSDTLLYKEELEKMKEMAPDNFRLDFAVSREQTNAAGEKMYIQTRMAEYKEELWE
LLKKDNTYVYMCGLKGMEKGIDDIMLDLAAKDGINWLDYKKQLKKSEQWNVEVY
>4DCXA
AAPDEITTAWPVNVGPLNPHLYTPNQMFAQSMVYEPLVKYQADGSVIPWLAKSWTHSEDGKTWTF
TLRDDVKFSNGEPFDAEAAAENFRAVLDNRQRHAWLELANQIVDVKALSKTELQITLKSAYYPFL
QELALPRPFRFIAPSQFKNHETMNGIKAPIGTGPWILQESKLNQYDVFVRNENYWGEKPAIKKIT
FNVIPDPTTRAVAFETGDIDLLYGNEGLLPLDTFARFSQNPAYHTQLSQPIETVMLALNTAKAPT
NELAVREALNYAVNKKSLIDNALYGTQQVADTLFAPSVPYANLGLKPSQYDPQKAKALLEKAGWT
LPAGKDIREKNGQPLRIELSFIGTDALSKSMAEIIQADMRQIGADVSLIGEEESSIYARQRDGRF
GMIFHRTWGAPYDPHAFLSSMRVPSHADFQAQQGLADKPLIDKEIGEVLATHDETQRQALYRDIL
TRLHDEAVYLPISYISMMVVSKPELGNIPYAPIATEIPFEQIKPVKP

>4DCKC
MALLRKSYSEPQLKGIVTKLYSRQGYHLQLQADGTIDGTKDEDSTYTLFNLIPVGLRVVAIQGVQ
TKLYLAMNSEGYLYTSELFTPECKFKESVFENYYVTYSSMIYRQQQSGRGWYLGLNKEGEIMKGN
HVKKNKPAAHFLPKPLKVAMYKEPSLHDLTEFSRSGSGTPTKSRSVSGVLNGGKSMSHNEST
>4DBLC
MSIVMQLQDVAESTRLGPLSGEVRAGEILHLVGPNGAGKSTLLARMAGMTSGKGSIQFAGQPLEA
WSATKLALHRAYLSQQQTPPFATPVWHYLTLHQHDKTRTELLNDVAGALALDDKLGRSTNQLSGG
EWQRVRLAAVVLQITPQANPAGQLLLLDQPMNSLDVAQQSALDKILSALSQQGLAIVMSSHDLNH
TLRHAHRAWLLKGGKMLASGRREEVLTPPNLAQAYGMNFRRLDIEGHRMLISTI
>4D97A
MRGSHHHHHHGMASMPLHHLTRFPRLEFIGAPTPLEYLPRLSDYLGREIYIKRDDVTPIAMGGNK
LRKLEFLVADALREGADTLITAGAIQSNHVRQTAAVAAKLGLHCVALLENPIGTTAENYLTNGNR
LLLDLFNTQIEMCDALTDPDAQLQTLATRIEAQGFRPYVIPVGGSSALGAMGYVESALEIAQQCE
EVVGLSSVVVASGSAGTHAGLAVGLEHLMPDVELIGVTVSRSVAEQKPKVIALQQAIAGQLALTA
TADIHLWDDYFAPGYGVPNDAGMEAVKLLASLEGVLLDPVYTGKAMAGLIDGISQKRFNDDGPIL
FIHTGGAPALFAYHPHVTYPE
>4D8QH
MSLRLPQNPNAGLFKQGYNSYSNADGQIIKSIAAIRELHQMCLTSMGPCGRNKIIVNHLGKIIIT
NDAATMLRELDIVHPAVKVLVMATEQQKIDMGDGTNLVMILAGELLNVSEKLISMGLSAVEIIQG
YNMARKFTLKELDEMVVGEITDKNDKNELLKMIKPVISSKKYGSEDILSELVSEAVSHVLPVAQQ
AGEIPYFNVDSIRVVKIMGGSLSNSTVIKGMVFNREPEGHVKSLSEDKKHKVAVFTCPLDIANTE
TKGTVLLHNAQEMLDFSKGEEKQIDAMMKEIADMGVECIVAGAGVGELALHYLNRYGILVLKVPS
KFELRRLCRVCGATPLPRLGAPTPEELGLVETVKTMEIGGDRVTVFKQEQGEISRTSTIILRGAT
QNNLDDIERAIDDGVAAVKGLMKPSGGKLLPGAGATEIELISRITKYGERTPGLLQLAIKQFAVA
FEVVPRTLAETAGLDVNEVLPNLYAAHNVTEPGAVKTDHLYKGVDIDGESDEGVKDIREENIYDM
LATKKFAINVATEAATTVLSIDQIIMAKKAGGPRAPQGPRPGNWDQED
>4D87A
MSNKYRVRKNVLHLTDTEKRDFVRTVLILKEKGIYDRYIAWHGAAGKFHTPPGSDRNAAHMSSAF
LPWHREYLLRFERDLQSINPEVTLPYWEWETDAQMQDPSQSQIWSADFMGGNGNPIKDFIVDTGP
FAAGRWTTIDEQGNPSGGLKRNFGATKEAPTLPTRDDVLNALKITQYDTPPWDMTSQNSFRNQLE
GFINGPQLHNRVHRWVGGQMGVVPTAPNDPVFFLHHANVDRIWAVWQIIHRNQNYQPMKNGPFGQ
NFRDPMYPWNTTPEDVMNHRKLGYVYDIELRKSKRSSHHHHHH
>4D8KA
GGSNPPASPLQDNLVIALHSYEPSHDGDLGFEKGEQLRILEQSGEWWKAQSLTTGQEGFIPFNFV
AKANSLEPEPWFFKNLSRKDAERQLLAPGNTHGSFLIRESESTAGSFSLSVRDFDQNQGEVVKHY
KIRNLDNGGFYISPRITFPGLHELVRHYTNASDGLCTRLSRPCQT
>3VF0B
HMLDPEEIRKRLEHTERQFRNRRKILIRGLPGDVTNQEVHDLLSDYELKYCFVDKYKGTAFVTLL
NGEQAEAAINAFHQSRLRERELSVQLQPTDALLCVANLPPSLTQQQFEELVRPFGSLERCFLVYS
ERTGQSKGYGFAEYMKKDSAARAKSDLLGKPLGPRTLYVHWTDAGQLTPALLHSRCLCVDRLPPG
FNDVDALCRALSAVHSPTFCQLACGQDGQLKGFAVLEYETAEMAEEAQQQADGLSLGGSHLRVSF
CAPGPPGRSMLAALIAAQATALNRG
>3VFDA
ESGAVPKRKDPLTHTSNSLPRSKTVMKTGSAGLSGHHRAPSYSGLSMVSGVKQGSGPAPTTHKGT

PKTNRTNKPSTPTTATRKKKDLKNFRNVDSNLANLIMNEIVDNGTAVKFDDIAGQDLAKQALQEI
VILPSLRPELFTGLRAPARGLLLFGPPGNGKTMLAKAVAAESNATFFNISAASLTSKYVGEGEKL
VRALFAVARELQPSIIFIDQVDSLLCERREGEHDASRRLKTEFLIEFDGVQSAGDDRVLVMGATN
RPQELDEAVLRRFIKRVYVSLPNEETRLLLLKNLLCKQGSPLTQKELAQLARMTDGYSGSDLTAL
AKDAALGPIRELKPEQVKNMSASEMRNIRLSDFTESLKKIKRSVSPQTLEAYIRWNKDFGDTTV
>2LO0A
SVDVAVSAGAGERASAEQKESYEPPKPAVGPSGESVVATEAFWDDLQGFLEQRLKDYDEANKLRV
LFKEAWRSSF
>3VE0I
YPYDVPDYAIEGRGARSMPLGVVTNSTLEVTEIDQLVCKDHLASTDQLKSVGLNLEGSGVSTDIP
SATKRWGFRSGVPPKVVSYEAGEWAENCYNLEIKKPDGSECLPPPPDGVRGFPRCRYVHKAQGTG
PCPGDYAFHKDGAFFLYDRLASTVIYRGVNFAEGVIAFLILAKPKETFLQSPPIREAVNYTENTS
SYYATSYLEYEIENFGAQHSTTLFKINNNTFVLLDRPHTPQFLFQLNDTIHLHQQLSNTTGKLIW
TLDANINADIGEWAFWENKKNLSEQLRGEELSFETLSL
>4AE2A
ETGHHHHHHSADEPMDFKINTDEIMTSLKSVNGQIESLISPDGSRKNPARNCRDLKFCHPELKSG
EYWVDPNQGCKLDAIKVFCNMETGETCISANPLNVPRKHWWTDSSAEKKHVWFGESMDGGFQFSY
GNPELPEDVLDVQLAFLRLLSSRASQQITYHCKNSIAYMDQASGNVKKALKLMGSNEGEFKAEGN
SKFTYTVLEDGCTKHTGEWSKTVFEYRTRKAVRLPIVDIAPYDIGGPDQEFGVDVGPVCFL
>3VDIA
MALFGTKDTTTAHSDYEIILEGGSSSWGQIKGRAKVNVPAALPLLPADCNIKIEAKPLDAQKGVV
RFTSQIESIVDSTKNKLVVEVDIANETKDRRIAVGEGEVSVGDFSHKFSFEGSVVNMYYYRSDAV
RRNVPNPVYMQGRQFHDIMMKVPLDNKDLIETWEGFQQSISGGGVNFGDWIREFWFIGPAYTAIN
EGGQRISPIQVNNFGVESGEKGPVGVSRWKFSHAGSGIVDSISRWAELFPVEQLNKPASIEGGFR
SDSQGIEVKVDGNLPGVSRDAGGGLRRILNHPLIPLVHHGMVGKFNDFTVDTQLKVVLPKGYKIR
YAAPQFRSQNLEEYRWSGGAYARWVEHVCKGGTGQFEVLYAQ
>3VDJA
YVEFEPSDKHIKEYLNKIQNSLSTEWSPCSVTCGNGIQVRIKPGSANKPKDELDYANDIEKKICK
MEKCPHHHHHHA
>4ADZA
GSHMTTTEAGASAPSPAVDGAVNQTARQAEADGTDIVTDHDRGVHGYHKQKAEHLKRLRRIEGQI
RGLQRMVDEDVYCIDILTQVSASTKALQSFALQLLEEHLRHCVADAALKGGTEIDAKVEEATKAI
GRLLRT
>3VBAA
MRSIIKGRVWKFGNNVDTDAILPARYLVYTKPEELAQFVMTGADPDFPKKVKPGDIIVGGKNFGC
GSSREHAPLGLKGAGISCVIAESFARIFYRNAINVGLPLIECKGISEKVNEGDELEVNLETGEIK
NLTTGEVLKGQKLPEFMMEILEAGGLMPYLKKKMAESQLEHHHHHH
>3V9OA
GPGSMTGTTMFAALLHPRLADCRRLYLRNHEVYMNIGAFEHEKRGEQRVVINVDLFVPLALTTPV
EDKLREVVDYDLMKQSVAQCVARGHIHLQETLCDAIAASLLAHDAVRAVRVSTEKPDAYPDCDAV
GVEVFRIKDEERA
>3V9BA
IPRFGVKTEQEDVLAKELEDVNKWGLHVFRIAELSGNRPLTVIMHTIFQERDLLKTFKIPVDTLI
TYLMTLEDHYHADVAYHNNIHAADVVQSTHVLLSTPALEAVFTDLEILAAIFASAIHDVDHPGVS

NQFLINTNSELALMYNDSSVLENHHLAVGFKLLQEENCDIFQNLTKKQRQSLRKMVIDIVLATDM
SKHMNLLADLKTMVETKKVTSSGVLLLDNYSDRIQVLQNMVHCADLSNPTKPLQLYRQWTDRIME
EFFRQGDRERERGMEISPMCDKHNASVEKSQVGFIDYIVHPLWETWADLVHPDAQDILDTLEDNR
EWYQSTIPQSPSPAPDDPEEGRQGQTEKFQFELTL
>2LNAA
MGHHHHHHSHMKRSGREITWKDFVNNYLSKGVVDRLEVVNKRFVRVTFTPGKTPVDGQYVWFNIG
SVDTFERNLETLQQELGIEGENRVPVVYIAESDG
>3V65B
TGEENCNVNNGGCAQKCQMIRGAVQCTCHTGYRLTEDGRTCQDVNECAEEGYCSQGCTNSEGAFQ
CWCEAGYELRPDRRSCKALGPEPVLLFANRIDIRQVLPHRSEYTLLLNNLENAIALDFHHRRELV
FWSDVTLDRILRANLNGSNVEEVVSTGLESPGGLAVDWVHDKLYWTDSGTSRIEVANLDGAHRKV
LLWQSLEKPRAIALHPMEGTIYWTDWGNTPRIEASSMDGSGRRIIADTHLFWPNGLTIDYAGRRM
YWVDAKHHVIERANLDGSHRKAVISQGLPHPFAITVFEDSLYWTDWHTKSINSANKFTGKNQEII
RNKLHFPMDIHTLHPQRQPAGKNRCGDNNGGCTHLCLPSGQNYTCACPTGFRKINSHACAQ
>3V53A
MGHHHHHHMKRKHIKSLIEKIPTAKPELFAYPLDWSIVDSILMERRIRPWINKKIIEYIGEEEAT
LVDFVCSKVMAHSSPQSILDDVAMVLDEEAEVFIVKMWRLLIYETEAKKIGLVK
>3V57B
MLDAFSRVVVNSDAKAAYVGGSDLQALKSFIADGNKRLDAVNSIVSNASCMVSDAVSGMICENPG
LISPGGNCYTNRRMAACLRDGEIILRYVSYALLAGDASVLEDRCLNGLKETYIALGVPTNSSIRA
VSIMKAQAVAFITNTATERKMSFAAGDCTSLASEVASYFDRVGAAIS
>3V43A
HMEPIPICSFCLGTKEQNREKKPEELISCADCGNSGHPSCLKFSPELTVRVKALRWQCIECKTCS
SCRDQGKNADNMLFCDSCDRGFHMECCDPPLTRMPKGMWICQICRPR
>4AC5C
CFEPPPATTTQTGFRGLSMGEVLHPATVKAKKERDAQYPPALAAVKAEGPPVSQVYKNVKVLGNL
TEAEFLRTMTAITEWVSPQEGCTYCHDENNLASEAKYPYVVARRMLEMTRAINTNWTQHVAQTGV
TCYTCHRGTPLPPYVRYLEPTLPLNNRETPTHVERVETRSGYVVRLAKYTAYSALNYDPFTMFLA
NDKRQVRVVPQTALPLVGVSRGKERRPLSDAYATFALMMSISDSLGTNCTFCHNAQTFESWGKKS
TPQRAIAWWGIRMVRDLNMNYLAPLNASLPASRLGRQGEAPQADCRTCHQGVTKPLFGASRLKDY
PELGPIKAAAK
>3V48A
GHMKLSLSPPPYADAPVVVLISGLGGSGSYWLPQLAVLEQEYQVVCYDQRGTGNNPDTLAEDYSI
AQMAAELHQALVAAGIEHYAVVGHALGALVGMQLALDYPASVTVLISVNGWLRINAHTRRCFQVR
ERLLYSGGAQAWVEAQPLFLYPADWMAARAPRLEAEDALALAHFQGKNNLLRRLNALKRADFSHH
ADRIRCPVQIICASDDLLVPTACSSELHAALPDSQKMVMPYGGHACNVTDPETFNALLLNGLASL
LHHREAAL
>3V2AR
MQSKVLLAVALWLCVETRAASVGLPSVSLDLPRLSIQKDILTIKANTTLQITCRGQRDLDWLWPN
NQSGSEQRVEVTECSDGLFCKTLTIPKVIGNDTGAYKCFYRETDLASVIYVYVQDYRSPFIASVS
DQHGVVYITENKNKTVVIPCLGSISNLNVSLCARYPEKRFVPDGNRISWDSKKGFTIPSYMISYA
GMVFCEAKINDESYQSIMYIVVVVGYRIYDVVLSPSHGIELSVGEKLVLNCTARTELNVGIDFNW
EYPSSKHQHKKLVNRDLKTQSGSEMKKFLSTLTIDGVTRSDQGLYTCAASSGLMTKKNSTFVRVH
EKPFVAFGSGMESLVEATVGERVRIPAKYLGYPPPEIKWYKNGIPLESNHTIKAGHVLTIMEVSE

RDTGNYTVILTNPISKEKQSHVVSLVVYVPPQIGEKSLISPVDSYQYGTTQTLTCTVYAIPPPHH
IHWYWQLEEECANEPSQAVSVTNPYPCEEWRSVEDFQGGNKIEVNKNQFALIEGKNKTVSTLVIQ
AANVSALYKCEAVNKVGRGERVISFHVTRGPEITLQPDMQPTEQESVSLWCTADRSTFENLTWYK
LGPQPLPIHVGELPTPVCKNLDTLWKLNATMFSNSTNDILIMELKNASLQDQGDYVCLAQDRKTK
KRHCVVRQLTVLERVAPTITGNLENQTTSIGESIEVSCTASGNPPPQIMWFKDNETLVEDSGIVL
KDGNRNLTIRRVRKEDEGLYTCQACSVLGCAKVEAFFIIEGAQEKTNLERTHHHHHH
>3J16C
MKLNISYPVNGSQKTFEIDDEHRIRVFFDKRIGQEVDGEAVGDEFKGYVFKISGGNDKQGFPMKQ
GVLLPTRIKLLLTKNVSCYRPRRDGERKRKSVRGAIVGPDLAVLALVIVKKGEQELEGLTDTTVP
KRLGPKRANNIRKFFGLSKEDDVRDFVIRREVTKGEKTYTKAPKIQRLVTPQRLQRKRHQRALKV
RNAQAQREAAAEYAQLLAKRLSERKAEKAEIRKRRASSLKA
>3VMGA
MANVDEAILKRVKGWAPYVDAKLGFRNHWYPVMFSKEINEGEPKTLKLLGENLLVNRIDGKLYCL
KDRCLHRGVQLSVKVECKTKSTITCWYHAWTYRWEDGVLCDILTNPTSAQIGRQKLKTYPVQEAK
GCVFIYLGDGDPPPLARDTPPNFLDDDMEILGKNQIIKSNWRLAVENGFDPSHIYIHKDSILVKD
NDLALPLGFAPGGDRKQQTRVVDDDVVGRKGVYDLIGEHGVPVFEGTIGGEVVREGAYGEKIVAN
DISIWLPGVLKVNPFPNPDMMQFEWYVPIDENTHYYFQTLGKPCANDEERKKYEQEFESKWKPMA
LEGFNNDDIWAREAMVDFYADDKGWVNEILFESDEAIVAWRKLASEHNQGIQTQAHVSGLEHHHH
HH
>3V33A
GGGTPKAPNLEPPLPEEEKEGSDLRPVVIDGSNVAMSHGNKEVFSCRGILLAVNWFLERGHTDIT
VFVPSWRKEQPRPDVPITDQHILRELEKKKILVFTPSRRVGGKRVVCYDDRFIVKLAYESDGIVV
SNDTYRDLQGERQEWKRFIEERLLMYSFVNDKFMPPDDPLGRHGPSLDNFLRKKPLTLEHRKQPC
PYGRKCTYGIKCRFFHPERPSCPQRSVA
>3V22V
MKRQKRDRLERAHQRGYQAGIAGRSKEMCPYQTLNQRSQWLGGWREAMADRVVMAHHHHHH
>4ABRL
MVALPTINQLVRKGREKVRKKSKVPALKGAPFRRGVCTVVRTVTPKKPNSALRKVAKVRLTSGYE
VTAYIPGEGHNLQEHSVVLIRGGRVKDLPGVRYHIVRGVYDAAGVKDRKKSRSKYGTKKPKEAAK
TAAKK
>3VM6A
MNHKVHHHHHHIEGRHMAVVKEVLEIAEKIKNMEIRGAGKIARSAAYALQLQAEKSKATNVDEFW
KEMKQAAKILFETRPTAVSLPNALRYVMHRGKIAYSSGADLEQLRFVIINAAKEFIHNSEKALER
IGEFGAKRIEDGDVIMTHSHSKAAISVMKTAWEQGKDIKVIVTETRPKWQGKITAKELASYGIPV
IYVVDSAARHYMKMTDKVVMGADSITVNGAVINKIGTALIALTAKEHRVWTMIAAETYKFHPETM
LGQLVEIEMRDPTEVIPEDELKTWPKNIEVWNPAFDVTPPEYVDVIITERGIIPPYAAIDILREE
FGWALKYTEPWED
>3J0TL
MKTFTAKPETVKRDWYVVDATGKTLGRLATELARRLRGKHKAEYTPHVDTGDYIIVLNADKVAVT
GNKRTDKVYYHHTGHIGGIKQATFEEMIARRPERVIEIAVKGMLPKGPLGRAMFRKLKVYAGNEH
NHAAQQPQVLDI
>3UXQD
MAVKKFKPYTPSRRFMTVADFSEITKTEPEKSLVKPLKKTGGRNNQGRITVRFRGGGHKRLYRII
DFKRWDKVGIPAKVAAIEYDPNRSARIALLHYVDGEKRYIIAPDGLQVGQQVVAGPDAPIQVGNA

LPLRFIPVGTVVHAVELEPKKGAKLARAAGTSAQIQGREGDYVILRLPSGELRKVHGECYATVGA
VGNADHKNIVLGKAGRSRWLGRRPHVRGAAMNPVDHPHGGGEGRAPRGRPPASPWGWQTKGLKTR
KRRKPSSRFIIARRKK
>3UX2A
SNARIMEEKALEVYDLIRTIRDPEKPNTLEELEVVSESCVEVQEINEEEYLVIIRFTPTVPHCSL
ATLIGLCLRVKLQRCLPFKHKLEIYISEGTHSTEEDINKQINDKERVAAAMENPNLREIVEQCVL
>3UW8A
MAETPNSDMSGATGGRSKRPKSNQDWWPSKLNLEILDQNARDVGPVEDDFDYAEEFQKLDLEAVK
SDLEELMTSSQDWWPADYGHYGPLFIRMAWHSAGTYRTADGRGGAAGGRQRFAPINSWPDNANLD
KARRLLLPIKQKYGQKISWADLMILAGNVAIESMGFKTFGYAGGREDAFEEDKAVNWGPEDEFET
QERFDEPGEIQEGLGASVMGLIYVNPEGPDGNPDPEASAKNIRQTFDRMAMNDKETAALIAGGHT
FGKVHGADDPEENLGPEPEAAPIEQQGLGWQNKNGNSKGGEMITTGIEGPWTQSPTEWDMGYINN
LLDYEWEPEKGPGGAWQWAPKSEELKNSVPDAHDPDEKQTPMMLTTDIALKRDPDYREVMETFQE
NPMEFGMNFAKAWYKLTHRDMGPPERFLGPEVPDEEMIWQDPLPDADYDLIGDEEIAELKEEILD
SDLSVSQLVKTAWASASTYRDSDKRGGANGARLRLEPQKNWEVNEPEQLETVLGTLENIQTEFND
SRSDGTQVSLADLIVLGGNAAVEQAAANAGYDVEIPFEPGRVDAGPEHTDAPSFDALKPKVDGVR
NYIQDDITRPAEEVLVDNADLLNLTASELTALIGGMRSIGANYQDTDLGVFTDEPETLTNDFFVN
LLDMGTEWEPAADSEHRYKGLDRDTGEVKWEATRIDLIFGSNDRLRAISEVYGSADAEKKLVHDF
VDTWSKVMKLDRFDLEHHHHHH
>3UW2A
MAHHHHHHMGTLEAQTQGPGSMISQSIFKAYDIRGVIGKTLDADVARSIGRAFGSEVRAQGGDAV
VVARDGRLSGPELVGALADGLRAAGVDVVDVGMVPTPVGYFAASVPLALSGGERRVDSCIVVTGS
HNPPDYNGFKMVLRGAAIYGDQIQGLYKRIVDARFETGSGSYEQYDVADQYVERIVGDIKLTRPL
KLVVDAGNGVAGPLATRLFKALGCELVELFTDIDGNFPNHHPDPAHPENLQDVIAKLKATDAEIG
FAFDGDGDRLGVVTKDGQIIYPDRQLMLFAEEVLSRNPGAQIIYDVKCTRNLARWVREKGGEPLM
WKTGHSLVKAKLRETGAPLAGEMSGHVFFKDRWYGFDDGLYTGARLLEILARVADPSALLNGLPN
AVSTPELQLKLEEGENVKLIDKLRADAKFDGADEVVTIDGLRVEYPDGFGLARSSNTTPVVVLRF
EATSDAALARIQDDFRRALKAAKPGANLPF
>3UV1A
DPIHYDKITEEINKAIDDAIAAIEQSETIDPMKVPDHADKFERHVGILDFKGELAMRNIEARGLK
QMKRQGDANVKGEEGIVKAHLLIGVHDDIVSMEYDLAYKLGDLHPTTHVISDIQDFVVALSLEIP
DEGNITMTSFEVRQFANVVNHIGGLSILDPIFGVLSDVLTAIFQDTVRKEMTKVLAPAFKRELEK
N
>4A9WA
MDSVDVVVIGGGQSGLSAGYFLRRSGLSYVILDAEASPGGAWQHAWHSLHLFSPAGWSSIPGWPM
PASQGPYPARAEVLAYLAQYEQKYALPVLRPIRVQRVSHFGERLRVVARDGRQWLARAVISATGT
WGEAYTPEYQGLESFAGIQLHSAHYSTPAPFAGMRVAIIGGGNSGAQILAEVSTVAETTWITQHE
PAFLADDVDGRVLFERATERWKAQQEGREPDLPPGGFGDIVMVPPVLDARARGVLAAVPPPARFS
PTGMQWADGTERAFDAVIWCTGFRPALSHLKGLDLVTPQGQVEVDGSGLRALAVPSVWLLGYGDW
NGMASATLIGVTRYAREAVRQVTAYCADHQDR
>3UV2A
SMQCQSTEDAMTVLTPLTEKDYEGLKRVLRSLQAHKMAWPFLEPVDPNDAPDYYGVIKEPMDLAT
MEERVQRRYYEKLTEFVADMTKIFDNCRYYNPSDSPFYQCAEVLESFFVQKLKGFKASRSH
>3UUMA

DMDLDSYQIALEEVLTWLLSAEDTFQEQDDISDDVEDVKEQFATHETFMMELSAHQSSVGSVLQA
GNQLMTQGTLSDEEEFEIQEQMTLLNARWEALRVESMERQSRLHDALMELQKKQLQQL
>3UTNX
MGSSHHHHHHSSGLVPRGSHMASMPLFDLISPKAFVKLVASEKVHRIVPVDATWYLPSWKLDNKV
DFLTKPRIPNSIFFDIDAISDKKSPYPHMFPTKKVFDDAMSNLGVQKDDILVVYDRVGNFSSPRC
AWTLGVMGHPKVYLLNNFNQYREFKYPLDSSKVAAFSPYPKSHYESSESFQDKEIVDYEEMFQLV
KSGELAKKFNAFDARSLGRFEGTEPEPRSDIPSGHIPGTQPLPYGSLLDPETKTYPEAGEAIHAT
LEKALKDFHCTLDPSKPTICSCGTGVSGVIIKTALELAGVPNVRLYDGSWTEWVLKSGPEWIAEN
RD
>4A9AA
MHHHHHHMSTTVEKIKAIEDEMARTQKNKATSFHLGQLKAKLAKLRRELLTSASSGSGGGAGIGF
DVARTGVASVGFVGFPSVGKSTLLSKLTGTESEAAEYEFTTLVTVPGVIRYKGAKIQMLDLPGII
DGAKDGRGRGKQVIAVARTCNLLFIILDVNKPLHHKQIIEKELEGVGIRLNKTPPDILIKKKEKG
GISITNTVPLTHLGNDEIRAVMSEYRINSAEIAFRCDATVDDLIDVLEASSRRYMPAIYVLNKID
SLSIEELELLYRIPNAVPISSGQDWNLDELLQVMWDRLNLVRIYTKPKGQIPDFTDPVVLRSDRC
SVKDFCNQIHKSLVDDFRNALVYGSSVKHQPQYVGLSHILEDEDVVTILKK
>4A91A
MTDTQYIGRFAPSPSGELHFGSLIAALGSYLQARARQGRWLVRIEDIDPPREVPGAAETILRQLE
HYGLHWDGDVLWQSQRHDAYREALAWLHEQGLSYYCTCTRARIQSIGGIYDGHCRVLHHGPDNAA
VRIRQQHPVTQFTDQLRGIIHADEKLAREDFIIHRRDGLFAYNLAVVVDDHFQGVTEIVRGADLI
EPTVRQISLYQLFGWKVPDYIHLPLALNPQGAKLSKQNHAPALPKGDPRPVLIAALQFLGQQAEA
HWQDFSVEQILQSAVKNWRLTAVPESAIVNSTFSNASC
>3UR1C
SQIGETLENIRSIEKLIQNIMRIARETNILALNATIEAARAGEAGKGFMIVANEVQNLSNETNEV
TKQIVEKAREILESSQRSLE
>4A8JC
MHHHHHHMGSVQRQDLVLFSDQSVLPAHFFQDSNSHNLFFITHQSCTQPLWMINALVETHVLGSP
SSLNESSSSMLPSSTRSHAVLASFIHEQNYFTNSLNKLKIPSNNYNVLDFLSDFIVNNIHNKPRD
KILSDVLAKFSAAIQNNPTDTIVIIEQPELLLSLVSGLTCSELNNKFITPLLRQCKVLIIVSNSD
IFNIDEYDASVHSSNLQNFYKSSFIKSMINLNLNPLKTGFAKDVTGSLHVCRGGAPIATSNTSLH
VVENEYLYLNEKESTKLFYR
>3UR1B
KEFEVLSFEIDEQALAFDVDNIEMVIEKSDITPVPKSRHFVEGVINLRGRIIPVVNLAKILGISF
DEQKMKSIIVARTKDVEVGFLVDRVLGVLRITENQLDLTNVSDKFGKKSKGLVKTDGRLIIYLDI
DKIIEEITV
>4A8JB
MASSSHNPVILLKRILSLTESSPFILCLDSIAQTSYKLIQEFVHQSKSKGNEYPIVYISFETVNK
PSYCTQFIDATQMDFVHLVKQIISYLPAATATQAKKHMVIIDSLNYISTEYITRFLSEIASPHCT
MVATYHKDIKDENRTVIPDWNNNYPDKLTLLQFMATTIVDIDVVLTGTLDTEEVSELLNEFRIPR
GLNNDIFQLRLVNKRKSGRSLEYDFIVNSNTHEYELLSTTKQEEESSSNGLETPEMLQGLTTFNL
GTSNKQKLAK
>3UPIA
SMSYTWTGALITPCAAEESKLPINPLSNSLLRHHNMVYATTSRSASLRQKKVTFDRLQVLDDHYR
DVLKEMKAKASTVKAKLLSIEEACKLTPPHSAKSKFGYGAKDVRNLSSRAVNHIRSVWEDLLEDT

ETPIDTTIMAKSEVFCVQPEKGGRKPARLIVFPDLGVRVCEKMALYDVVSTLPQAVMGSSYGFQY
SPKQRVEFLVNTWKSKKCPMGFSYDTRCFDSTVTESDIRVEESIYQCCDLAPEARQAIRSLTERL
YIGGPLTNSKGQNCGYRRCRASGVLTTSCGNTLTCYLKATAACRAAKLQDCTMLVNGDDLVVICE
SAGTQEDAAALRAFTEAMTRYSAPPGDPPQPEYDLELITSCSSNVSVAHDASGKRVYYLTRDPTT
PLARAAWETARHTPINSWLGNIIMYAPTLWARMILMTHFFSILLAQEQLGKALDCQIYGACYSIE
PLDLPQIIERLHGLSAFTLHSYSPGEINRVASCLRKLGVPPLRTWRHRARSVRAKLLSQGGRAAI
CGRYLFNWAVRTKLKLTPIPAASQLDLSGWFVAGYSGGDIYHSLSRARPRENLYFQGLEHHHHHH
>3UOQG
MPRRRVIGQRKILPDPKFGSELLAKFVNILMVDGKKSTAESIVYSALETLAQRSGKSELEAFEVA
LENVRPTVEVKSRRVGGSTYQVPVEVRPVRRNALAMRWIVEAARKRGDKSMALRLANELSDAAEN
KGTAVKKREDVHRMAEANKAFAHYRWLSLRSFSHQAGASSKQPALGYLN
>3VKFA
SQKLDDVDPLVTTNFGKIRGIKKELNNEILGPVIQFLGVPYAAPPTGEHRFQPPEPPSPWSDIRN
ATQFAPVCPQNIIDGRLPEVMLPVWFTNNLDVVSSYVQDQSEDCLYLNIYVPTEDVKRISKECAR
KPGKKICRKGDIRDSGGPKPVMVYIHGGSYMEGTGNLYDGSVLASYGNVIVITVNYRLGVLGFLS
TGDQAAKGNYGLLDLIQALRWTSENIGFFGGDPLRITVFGSGAGGSCVNLLTLSHYSEGLFQRAI
AQSGTALSSWAVSFQPAKYARILATKVGCNVSDTVELVECLQKKPYKELVDQDVQPARYHIAFGP
VIDGDVIPDDPQILMEQGEFLNYDIMLGVNQGEGLKFVENIVDSDDGVSASDFDFAVSNFVDNLY
GYPEGKDVLRETIKFMYTDWADRHNPETRRKTLLALFTDHQWVAPAVATADLHSNFGSPTYFYAF
YHHCQTDQVPAWADAAHGDEVPYVLGIPMIGPTELFPCNFSKNDVMLSAVVMTYWTNFAKTGDPN
QPVPQDTKFIHTKPNRFEEVAWTRYSQKDQLYLHIGLKPRVKEHYRANKVNLWLELVPHLHNLND
>4A7FB
ELDRAQERLATALQKLEEAEKAADESERGMKVIESRAQKDEEKMEIQEIQLKEAKHIAEDADRKY
EEVARKLVIIESDLERAEERAELSEGKCAELEEELKTVTNNLKSLEAQAEKYSQKEDKYEEEIKV
LSDKLK
>4A7KA
MYSKVFLKPHCEPEQPAALPLFQPQLVQGGRPDGYWVEAFPFRSDSSKCPNIIGYGLGTYDMKSD
IQMLVNPYATTNNQSSSWTPVPLAKLDFPVAMHYADITKNGFNDVIITDQYGSSMDDIWAYGGRV
SWLENPGELRDNWTMRTIGHSPGMHRLKAGHFTRTDRVQVVAVPIVVASSDLTTPADVIIFTAPD
DPRSEQLWQRDVVGTRHLVHEVAIVPAAETDGEMRFDQIILAGRDGVDCLWYDGARWQRHLVGTG
LPEERGDPYWGAGSAAVGRVGDDYAGYICSAEAFHGNTVSVYTKPAGSPTGIVRAEWTRHVLDVF
GPLNGKHTGSIHQVVCADIDGDGEDEFLVAMMGADPPDFQRTGVWCYKLVDRTNMKFSKTKVSSV
SAGRIATANFHSQGSEVDIATISYSVPGYFESPNPSINVFLSTGILAERLDEEVMLRVVRAGSTR
FKTEMEFLDVAGKKLTLVVLPPFARLDVERNVSGVKVMAGTVCWADENGKHERVPATRPFGCESM
IVSADYLESGEEGAILVLYKPSSTSGRPPFRSMDELVAHNLFPAYVPDSVRAMKFPWVRCADRPW
AHGRFKDLDFFNLIGFHVNFADDSAAVLAHVQLWTAGIGVSAGFHNHVEASFCEIHACIANGTGR
GGMRWATVPDANFNPDSPNLEDTELIVVPDMHEHGPLWRTRPDGHPLLRMNDTIDYPWHAWLAGA
GNPSPQAFDVWVAFEFPGFETFSTPPPPRVLEPGRYAIRFGDPHQTASLALQKNDATDGTPVLAL
LDLDGGPSPQAWNISHVPGTDMYEIAHAKTGSLVCARWPPVKNQRVAGTHSPAAMGLTSRWAVTK
NTKGQITFRLPEAPDHGPLFLSVSAIRHQQEADAIPVIVQGDSIELSAWSLVPAN
>3UMFA
MGSSHHHHHHSSGLVPRGSHMTDQKLAKAKVIFVLGGPGSGKGTQCEKLVQKFHFNHLSSGDLLR
AEVQSGSPKGKELKAMMERGELVPLEVVLALLKEAMIKLVDKNCHFLIDGYPRELDQGIKFEKEV
CPCLCVINFDVSEEVMRKRLLKRAETSNRVDDNEETIVKRFRTFNELTKPVIEHYKQQNKVITID

ASGTVDAIFDKVNHELQKFGVK
>3ULRB
GPLGSSDLGITAIALYDYQAAGDDEISFDPDDIITNIEMIDDGWWRGVCKGRYGLFPANYVELRQ
>2LLIA
KEAAPKCNNCSQRGHLKKDCPHIICSYCGATDDHYSRHCPKAIQCSKCDEVGHYRSQCPHKWKKV
QCTLCKSKKHSKERCPSIWRAYILVDDNEKAKPKVLPFHTIYCYNCGGKGHFGDDCKEK
>4A6SA
AWKGEVLANNEAGQVTSIIYNPGDVITIVAAGWASYGPTQKWGPQGDREHPDQGLICHDAFCGAL
VMKIGNSGTIPVNTGLFRWVAPNNVQGAITLIYNDVPGTYGNNSGSFSVNIGKDQS
>2LLFA
PRLFECSNKTGRFLATEIVDFTQDDLDENDVYLLDTWDQIFFWIGKGANESEKEAAAETAQEYLR
SHPGSRDLDTPIIVVKQGFEPPTFTGWFMAWDPLCWSDRKSY
>3UILA
EDPPACGSIVPRREWRALASECRERLTRPVRYVVVSHTAGSHCDTPASCAQQAQNVQSYHVRNLG
WCDVGYNFLIGEDGLVYEGRGWNIKGAHAGPTWNPISIGISFMGNYMNRVPPPRALRAAQNLLAC
GVALGALRSNYEVKGHRDVQPTLSPGDRLYEIIQTWSHYRA
>3UGJA
GLVPRGSHMMEILRGSPALSAFRINKLLARFQAANLQVHNIYAEYVHFADLNAPLNDSEQAQLTR
LLQYGPALSSHTPAGKLLLVTPRPGTISPWSSKATDIAHNCGLQQVDRLERGVAYYIEASTLTAE
QWRQVAAELHDRMMETVFSSLTDAEKLFIHHQPAPVSSVDLLGEGRQALIDANLRLGLALAEDEI
DYLQEAFTKLGRNPNDIELYMFAQANSEHCRHKIFNADWIIDGKPQPKSLFKMIKNTFETTPDYV
LSAYKDNAAVMEGSAVGRYFADHNTGRYDFHQEPAHILMKVETHNHPTAISPWPGAATGSGGEIR
DEGATGRGAKPKAGLVGFSVSNLRIPGFEQPWEEDFGKPERIVTALDIMTEGPLGGAAFNNEFGR
PALTGYFRTYEEKVNSHNGEELRGYHKPIMLAGGIGNIRADHVQKGEIVVGAKLIVLGGPAMNIG
LGGGAASSMASGQSDADLDFASVQRDNPEMERRCQEVIDRCWQLGDANPILFIHDVGAGGLSNAM
PELVSDGGRGGKFELRDILSDEPGMSPLEIWCNESQERYVLAVAADQLPLFDELCKRERAPYAVI
GDATEEQHLSLHDNHFDNQPIDLPLDVLLGKTPKMTRDVQTLKAKGDALNRADITIADAVKRVLH
LPTVAEKTFLVTIGDRTVTGMVARDQMVGPWQVPVADCAVTTASLDSYYGEAMSIGERAPVALLD
FAASARLAVGEALTNIAATQIGDIKRIKLSANWMAAAGHPGEDAGLYDAVKAVGEELCPQLGLTI
PVGKDSMSMKTRWQEGNEQREMTSPLSLVISAFARVEDVRHTLTPQLSTEDNALLLIDLGKGHNA
LGATALAQVYRQLGDKPADVRDVAQLKGFYDAMQALVAARKLLAWHDRSDGGLLVTLAEMAFAGH
CGVQVDIAALGDDHLAALFNEELGGVIQVRAEDRDAVEALLAQYGLADCVHYLGQALAGDRFVIT
ANDQTVFSESRTTLRVWWAETTWQMQRLRDNPQCADQEHEAKANDTDPGLNVKLSFDINEDIAAP
YIATGARPKVAVLREQGVNSHVEMAAAFHRAGFDAIDVHMSDLLGGRIGLGNFHALVACGGFSYG
DVLGAGEGWAKSILFNHRVRDEFETFFHRPQTLALGVCNGCQMMSNLRELIPGSELWPRFVRNHS
DRFEARFSLVEVTQSPSLLLQGMVGSQMPIAVSHGEGRVEVRDDAHLAALESKGLVALRYVDNFG
KVTETYPANPNGSPNGITAVTTENGRVTIMMPHPERVFRTVANSWHPENWGEDSPWMRIFRNARK
QLG
>3UGQA
MGSSHHHHHHSSGLVPRGSHMASATKNASSATPATMTSMVSQRQDLFMTDPLSPGSMFFLPNGAK
IFNKLIEFMKLQQKFKFGFNEVVTPLIYKKTLWEKSGHWENYADDMFKVETTDEEKEEYGLKPMN
CPGHCLIFGKKDRSYNELPLRFSDFSPLHRNEASGALSGLTRLRKFHQDDGHIFCTPSQVKSEIF
NSLKLIDIVYNKIFPFVKGGSGAESNYFINFSTRPDHFIGDLKVWNHAEQVLKEILEESGKPWKL
NPGDGAFYGPKLDIMVTDHLRKTHQVATIQLDFQLPERFDLKFKDQDNSYKRPIMIHRATFGSIE

RFMALLIDSNEGRWPFWLNPYQAVIIPVNTKNVQQLDMCTALQKKLRNELEADDMEPVPLNDWHF
NVDLDIRNEPVGYRIKSAILKNYSYLIIVGDEEVQLQKYNIRERDNRKSFEKLTMSQIWEKFIEL
EKNYK
>4A69C
GAMRQLAVIPPMLYDADQQRIKFINMNGLMADPMKVYKDRQVMNMWSEQEKETFREKFMQHPKNF
GLIASFLERKTVAECVLYYYLTKKNENYK
>4A6DA
MGSSEDQAYRLLNDYANGFMVSQVLFAACELGVFDLLAEAPGPLDVAAVAAGVRASAHGTELLLD
ICVSLKLLKVETRGGKAFYRNTELSSDYLTTVSPTSQCSMLKYMGRTSYRCWGHLADAVREGRNQ
YLETFGVPAEELFTAIYRSEGERLQFMQALQEVWSVNGRSVLTAFDLSVFPLMCDLGGGAGALAK
ECMSLYPGCKITVFDIPEVVWTAKQHFSFQEEEQIDFQEGDFFKDPLPEADLYILARVLHDWADG
KCSHLLERIYHTCKPGGGILVIESLLDEDRRGPLLTQLYSLNMLVQTEGQERTPTHYHMLLSSAG
FRDFQFKKTGAIYDAILARKGTHHHHHH
>3UEZE
GSHMEEVSEYCSHMIGSGHLQSLQRLIDSQMETSCQITFEFVDQEQLKDPVCYLKKAFLLVQDIM
EDTMRFRDNTPNAIAIVQLQELSLRLKSCFTKDYEEHDKACVRTFYETPLQLLEKVKNVFNETKN
LLDKDWNIFSKNCNNSFAECSSQ
>4A5VA
SSEPAKLDLSCVHSDNKGSRAPTIGEPVPDVSLEQCAAQCKAVDGCTHFTYNDDSKMCHVKEGKP
DLYDLTGGKTASRSCDRSCFEQHVSYEGAPDVMTAMVTSQSADCQAACAADPSCEIFTYNEHDQK
CTFKGRGFSAFKERGVLGVTSGPKQFCDEGG
>3UBBA
ERAGPVTWVMMIACVVVFIAMQILGDQEVMLWLAWPFDPTLKFEFWRYFTHALMHFSLMHILFNL
LWWWYLGGAVEKRLGSGKLIVITLISALLSGYVQQKFSGPWFGGLSGVVYALMGYVWLRGERDPQ
SGIYLQRGLIIFALIWIVAGWFDLFGMSMANGAHIAGLAVGLAMAFVDSLNA
>3UBRA
SDKTEPRNEVYKDKFKNQYNSWHDTAKSEELVDALEQDPNMVILWAGYAFAKDYKAPRGHMYAVT
DVRNTLRTGAPKNAEDGPLPMACWSCKSPDVPRLIEEQGEDGYFKGKWAKGGPEVTNTIGCSDCH
EKGSPKLRISRPYVDRALDAIGTPFSKASKQDKESMVCAQCHVEYYFEKKEDKKGFVKFPWDMGV
TVDQMEVYYDGIEFSDWTHALSKTPMLKAQHPEYETWKMGIHGKNNVSCVDCHMPKVTSPEGKKF
TDHKVGNPFDRFEETCATCHSQTKEFLVGVTNERKAKVKEMKLKAEEQLVKAHFEAAKAWELGAT
EAEMKPILTDIRHAQWRWDLAIASHGVAAHAPEEALRVLGTSVNKAADARVKLAQLLAKKGLTDP
VAIPDISTKAKAQAVLGMDMEKMNAEKEAFKKDMLPKWDAEAKKREATY
>4A53A
GAMGMSVADFYGSNVEVLLNNDSKARGVITNFDSSNSILQLRLANDSTKSIVTKDIKDLRILPKN
EIMPKNGTKSPSTNSTKLKSAETYSSKNKWSMDCDEEFDFAANLEKFDKKQVFAEFREKD
>3UA0A
MGHHHHHHMRVKTFVILCCALQYVAYTNANINDFDEDYFGSDVTVQSSNTTDEIIRDASGAVIEE
QITTKKMQRKNKNHGILGKNEKMIKTFVITTDSDGNESIVEEDVLMKTLSDGTVAQSYVAADAGA
YSQS
>3U9GA
GPLGMADPGVCCFITKILCAHGGRMTLEELLGEIRLPEAQLYELLETAGPDRFVLLETGGQAGIT
RSVVATTRARVCRRKYCQRPCDSLHLCKLNLLGRCHYAQSQRNLCKYSHDVLSEQNFQILKNHEL
SGLNQEELACLLVQSDPFFLPEICKSYKGEGRKQTCGQPQPCERLHICEHFTRGNCSYLNCLRSH

NLMDRKVLTIMREHGLSPDVVQNIQDICNNKHAR
>3U88C
SMDSRLQRIHAEIKNSLKIDNLDVNRCIEALDELASLQVTMQQAQKHTEMITTLKKIRRFKVSQV
IMEKSTMLYNKFKNMFLVGEGDSV
>3U6WA
GAMTTSESPDAYTESFGAHTIVKPAGPPRVGQPSWNPQRASSMPVNRYRPFAEEVEPIRLRNRTW
PDRVIDRAPLWCAVDLRDGNQALIDPMSPARKRRMFDLLVRMGYKEIEVGFPSASQTDFDFVREI
IEQGAIPDDVTIQVLTQCRPELIERTFQACSGAPRAIVHFYNSTSILQRRVVFRANRAEVQAIAT
DGARKCVEQAAKYPGTQWRFEYSPESYTGTELEYAKQVCDAVGEVIAPTPERPIIFNLPATVEMT
TPNVYADSIEWMSRNLANRESVILSLHPHNDRGTAVAAAELGFAAGADRIEGCLFGNGERTGNVC
LVTLGLNLFSRGVDPQIDFSNIDEIRRTVEYCNQLPVHERHPYGGDLVYTAFSGSHQDAINKGLD
AMKLDADAADCDVDDMLWQVPYLPIDPRDVGRTYEAV
>3U43A
MELKHSISDYTEAEFLEFVKKICRAEGATEEDDNKLVREFERLTEHPDGSDLIYYPRDDREDSPE
GIVKEIKEWRAANGKSGFKQGLEHHHHHH
>3VIQB
MEKSQLESRVHLLEQQKEQLESSLQDALAKLKNRDAKQTVQKHIDLLHTYNEIRDIALGMIGKVA
EHEKCTSVELFDRFGVNGSE
>3U3EA
MVLSEGEWQLVLHVWAKVEADVAGHGQDILIRLFKSHPETLEKFDRFKHLKTEAEMKASEDLKKH
GVTVLTALGAILKKKGHHEAELKPLAQSHATKHKIPIKYLEFISEAIIHVLHSRHPGDFGADAQG
AMNKALELFRKDIAAKYKELGYQG
>3U2GA
MGTYEIRGQVASGFGDQSWDASSFAGFYYDIDDNVSTETLTVSDLDGNVIPEGGLVYTTTIADVD
FEYYNPDAGWDQYPVMGFFAEEYIPINPDKADKIAKLVLDSDDKYTIRTGEMLDLGEGYAIEAKQ
VDVDGEKVWLEFTKDGEFVDDEIISVSTADDEANTWDVELDDIEDEDDVVVLKVHVNQVFQGAVD
SIAQIEGLWLIDYANAMTIESDDEFGNLDDVSIDGDTLKISNEDTFTLTRDSEEEIGEGMYFMIA
DTSSSDLRYYPYVEKTIGLEHHHHHH
>3U22A
GACDGILEGIYDSPAASDSNELGFIRTDPSTHSGTIYIDATDYRRWTFIDFHTQKVDSVNVTDSE
QKEPEEWDIAVHRYDVKTNAGAVLETGFTGFSALRNADAMPEGAYVEDVWTTAKIAIDMSGMMDG
NIVYMESYYNEELSKWLNVDKSNMPPTYTLSNKVYMVKLKDGTYAAVRLTNYMNASGVKGFMTID
YIYPFEL
>3U0CA
GSQAANDAANKLFSLTIADLTANQNINTTNAHSTSNILIPELKAPKSLNASSQLTLLIGNLIQIL
GEKSLTALTNKITAWKSQQQARQQKNLEFSDKINTLLSETEGLTRDYEKQINKLKNADSKIKDLE
NKINQIQTRLSELDPESPEKKKLSREEIQLTIKKDAAVKDRTLIEQKTLSIHSKLTDKSMQLEKE
IDSFSA
>3U0OA
MSENSIRLTQYSHGAGCGCKISPKVLETILHSEQAKFVDPNLLVGNETRDDAAVYDLGNGTSVIS
TTDFFMPIVDNPFDFGRIAATNAISDIFAMGGKPIMAIAILGWPINKLSPEIAREVTEGGRYACR
QAGIALAGGHSIDAPEPIFGLAVTGIVPTERVKKNSTAQAGCKLFLTKPLGIGVLTTAEKKSLLK
PEHQGLATEVMCRMNIAGASFANIEGVKAMTDVTGFGLLGHLSEMCQGAGVQARVDYEAIPKLPG
VEEYIKLGAVPGGTERNFASYGHLMGEMPREVRDLLCDPQTSGGLLLAVMPEAENEVKATAAEFG

IELTAIGELVPARGGRAMVEIR
>4A2NB
MNENLWKICFIVMFIIWVFVRKVYGTRAMKNKSKKKVRPNFEKSLVFLNFIGMVFLPLTAVFSSY
LDSFNINLPDSIRLFALIVTFLNIGLFTKIHKDLGNNWSAILEIKDGHKLVKEGIYKNIRHPMYA
HLWLWVITQGIILSNWVVLIFGIVAWAILYFIRVPKEEELLIEEFGDEYIEYMGKTGRLFPKVV
>3VIAA
GSMAFVKSGWLLRQSTILKRWKKNWFDLWSDGHLIYYDDQTRQNIEDKVHMPMDCINIRTGQECR
DTQPPDGKSKDCMLQIVCRDGKTISLCAESTDDCLAWKFTLQDSRTN
>4A2AA
MIDLSKTVFYTSIDIGSRYIKGLVLGKRDQEWEALAFSSVKSRGLDEGEIKDAIAFKESVNTLLK
ELEEQLQKSLRSDFVISFSSVSFEREDTVIERDFGEEKRSITLDILSEMQSEALEKLKENGKTPL
HIFSKRYLLDDERIVFNPLDMKASKIAIEYTSIVVPLKVYEMFYNFLQDTVKSPFQLKSSLVSTA
EGVLTTPEKDRGVVVVNLGYNFTGLIAYKNGVPIKISYVPVGMKHVIKDVSAVLDTSFEESERLI
ITHGNAVYNDLKEEEIQYRGLDGNTIKTTTAKKLSVIIHARLREIMSKSKKFFREVEAKIVEEGE
IGIPGGVVLTGGGAKIPRINELATEVFKSPVRTGCYANSDRPSIINADEVANDPSFAAAFGNVFA
VSENPYEETPVKSENPLKKIFRLFKELME
>4A25A
TTIHDVQTTGLTQDAVTGFDASSRLNAGLQEVLVDLTALHLQGKQAHWNIVGENWRDLHLQLDTL
VEAARGFSDDVAERMRAVGGVPDARPQTVAASRIGDVGPDEIDTRACVEAIVALVRHTVDTIRRV
HDPIDAEDPASADLLHAITLELEKQAWMIGSENRSPRRR
>3TWLA
MPELPEVEAARRAIEENCLGKKIKRVIIADDNKVIHGISPSDFQTSILGKTIISARRKGKNLWLE
LDSPPFPSFQFGMAGAIYIKGVAVTKYKRSAVKDSEEWPSKYSKFFVELDDGLELSFTDKRRFAK
VRLLANPTSVSPISELGPDALLEPMTVDEFAESLAKKKITIKPLLLDQGYISGIGNWIADEVLYQ
ARIHPLQTASSLSKEQCEALHTSIKEVIEKAVEVDADSSQFPSNWIFHNREKKPGKAFVDGKKID
FITAGGRTTAYVPELQKLYGKDAEKAAKVRPAKRGVKPKEDDGDHHHHHH
>3TVLA
MAQGLIEVERKFLPGPGTEERLQELGGTLEYRVTFRDTYYDTPELSLMQADHWLRRREDSGWELK
CPGAAGVLGPHTEYKELTAEPTIVAQLCKVLRADGLGAGDVAAVLGPLGLQEVASFVTKRSAWKL
VLLGADEEEPQLRVDLDTADFGYAVGEVEALVHEEAEVPTALEKIHRLSSMLGVPAQETAPAKLI
VYLQRFRPQDYQRLLEVNSSRERPQETEDPDHCLG
>3TV0A
GSSSMAEKTQKSVKIAPGAVVCVESEIRGDVTIGPRTVIHPKARIIAEAGPIVIGEGNLIEEQAL
IINAYPDNITPDTEDPEPKPMIIGTNNVFEVGCYSQAMKMGDNNVIESKAYVGRNVILTSGCIIG
ACCNLNTFEVIPENTVIYGADCLRRVQTERPQPQTLQLDFLMKILPNYHHLKKTMKGSSTPVKN
>3TURA
RRYTLNATALGLGGAATRQLTFQTSSPAHLTMPYVMPGDGEVVGVGEPVAIRFDENIADRGAAEK
AIKITTNPPVEGAFYWLNNREVRWRPEHFWKPGTAVDVAVNTYGVDLGEGMFGEDNVQTHFTIGD
EVIATADDNTKILTVRVNGEVVKSMPTSMGKDSTPTANGIYIVGSRYKHIIMDSSTYGVPVNSPN
GYRTDVDWATQISYSGVFVHSAPWSVGAQGHTNTSHGCLNVSPSNAQWFYDHVKRGDIVEVVNTV
GGTLPGIDGLGDWNIPWDQWRAGNAKA
>3TUNA
GPGSMKVEKVFFVTSPIYYVNAAPHIGHVYSTLITDVIGRYHRVKGERVFALTGTDEHGQKVAEA
AKQKQVSPYDFTTAVAGEFKKCFEQMDYSIDYFIRTTNEQHKAVVKELWTKLEQKGDIYLGRYEG

WYSISDESFLTPQNITDGVDKDGNPCKVSLESGHVVTWVSEENYMFRLSAFRERLLEWYHANPGC
IVPEFRRREVIRAVEKGLPDLSVSRARATLHNWAIPVPGNPDHCVYVWLDALTNYLTGSRLRVDE
SGKEVSLVDDFNELERFPADVHVIGKDILKFHAIYWPAFLLSAGLPLPKKIVAHGWWTKDRKKIS
KSLGNVFDPVEKAEEFGYDALKYFLLRESGFSDDGDYSDKNMIARLNGELADTLGNLVMRCTSAK
INVNGEWPSPAAYTEEDESLIQLIKDLPGTADHYYLIPDIQKAIIAVFDVLRAINAYVTDMAPWK
LVKTDPERLRTVLYITLEGVRVTTLLLSPILPRKSVVIFDMLGVPEVHRKGIENFEFGAVPPGTR
LGPAVEGEVLFSKRSTENTKST
>3TU5A
MCDEDETTALVCDNGSGLVKAGFAGDDAPRAVFPSIVGRPRHQGVMVGMGQKDSYVGDEAQSKRG
ILTLKYPIEHGIITNWDDMEKIWHHTFYNELRVAPEEHPTLLTEAPLNPKANREKMTQIMFETFN
VPAMYVAIQAVLSLYASGRTTGIVLDSGDGVTHNVPIYEGYALPHAIMRLDLAGRDLTDYLMKIL
TERGYSFVTTAEREIVRDIKEKLCYVALDFENEMATAASSSSLEKSYELPDGQVITIGNERFRCP
ETLFQPSFIGMESAGIHETTYNSIMKCDIDIRKDLYANNVMSGGTTMYPGIADRMQKEITALAPS
TMKIKIIAPPERKYSVWIGGSILASLSTFQQMWITKQEYDEAGPSIVHRKCF
>3TU3A
MHHHHHHSSGVDLGTENLYFQSNAMIDTWLAQWGLRLPSSNDATLRLQPAEGPELVMERLEGGWL
FVVELGLVPSGLPLGVILQLLQVNSPFSSLAPVKLAADDAGRLVLWAEARDGVDDVDALNRLHDR
LREGHSRLVPLLEPTGELVPAQIQTSALVFV
>3TT1A
MEVKREHWATRLGLILAMAGNAVGLGNFLRFPVQAAENGGGAFMIPYIIAFLLVGIPLMWIEWAM
GRYGGAQGHGTTPAIFYLLWRNRFAKILGVFGLWIPLVVAIYFVYIESWTLGFAIKFLVGLVPEP
PPNATDPDSILRPFKEFLYSYIGVPKGDEPILKPSLFAYIVFLITMFINVSILIRGISKGIERFA
KIAMPTLFILAVFLVIRVFLLETPNGTAADGLNFLWTPDFEKLKDPGVWIAAVGQIFFTLSLGFG
AIITYASYVRKDQDIVLSGLTAATLNEAAEVILGGSISIPAAVAFFGVANAVAIAKAGAFNLGFI
TLPAIFSQTAGGTFLGFLWFFLLFFAGLTSSIAIMQPMIAFLEDELKLSRKHAVLWTAAIVFFSA
HLVMFLNKSLDEMDFWAGTIGVVFFGLTELIIFFWIFGADKAWEEINRGGIIKVPRIYYYVMRYI
TPAFLAVLLVVWAREYIPKIMEETHWTVWITRFYIIGLFLFLTFLVFLAERRRNHESAGTLVPR
>4A0XA
MRGETLKLKKDKRREAIRQQIDSNPFITDHELSDLFQVSIQTIRLDRTYLNIPELRKRIKLVAEK
NYDQISSIEEQEFIGDLIQVNPNVKAQSILDITSDSVFHKTGIARGHVLFAQANSLCVALIKQPT
VLTHESSIQFIEKVKLNDTVRAEARVVNQTAKHYYVEVKSYVKHTLVFKGNFKMFYDKRG
>3TS9A
GHMDTRENPFKEKLLEIMASIQTYCQKSPMSDFGTQHYEQWAIQMEKKAAKDGNRKDRVCAEHLR
KYNEALQINDTIRMIDAYSHLETFYTDEKEKKFAVLNDSKKSLKLDETDEFLMNLFFDNKKMLKK
LAENPKYE
>3VHXB
GSLLFQPDQNAPPIRLRHRRSRSAGDRWVDHKPASNMQTETVMQPHVPHAITVSVANEKALAKCE
KYMLTHQELASDGEIETKLIKGDIYKTRGGGQSVQFTDIETLKQESPNGSRKRRS
>3TRTA
GGSKPDCTAAMRDVRQQYESVAAKNLQEAEEWYKSKFADLSEAANRNNDALRQAKQESTEYRRQV
QSLTMEVDALKG
>4A0EA
GGGSWVCRFYQGKHRGVEVELPHGRCVFGSDPLQSDIVLSDSEIAPVHLVLMVDEEGIRLTDSAE
PLLQEGLPVPLGTLLRAGSCLEVGFLLWTFVAVGQPLPETLQVPTQRKEPTDRLPRSR

>4A03A
TMAHHHHHHVTNSTDGRADGRLRVVVLGSTGSIGTQALQVIADNPDRFEVVGLAAGGAHLDTLLR
QRAQTGVTNIAVADEHAAQRVGDIPYHGSDAATRLVEQTEADVVLNALVGALGLRPTLAALKTGA
RLALANKESLVAGGSLVLRAARPGQIVPVDSEHSALAQCLRGGTPDEVAKLVLTASGGPFRGWSA
ADLEHVTPEQAGAHPTWSMGPMNTLNSASLVNKGLEVIETHLLFGIPYDRIDVVVHPQSIIHSMV
TFIDGSTIAQASPPDMKLPISLALGWPRRVSGAAAACDFHTASSWEFEPLDTDVFPAVELARQAG
VAGGCMTAVYNAANEEAAAAFLAGRIGFPAIVGIIADVLHAADQWAVEPATVDDVLDAQRWARER
AQRAVSGM
>3TO8A
MGHHHHHHDSPVLMVYGLDQSKMNCDRVFNVFCLYGNVEKVKFMKSKPGAAMVEMADGYAVDRAI
THLNNNFMFGQKLNVCVSKQPAIMPGQSYGLEDGSCSYKDFSESRNNRFSTPEQAAKNRIQHPSN
VLHFFNAPLEVTEENFFEICDELGVKRPSSVKVFSGKSERSSSGLLEWESKSDALETLGFLNHYQ
MKNPNGPYPYTLKLCFSTAQHAS
>3TNXA
MHHHHHHSSGLVPRGSGMKETAAAKFERQHMDSPDLGTDDDDKMDFSIVGYSQNDLTSTERLIQL
FESWMLKHNKIYKNIDEKIYRFEIFKDNLKYIDETNKKNNSYWLGLNVFADMSNDEFKEKYTGSI
AGNYTTTELSYEEVLNDGDVNIPEYVDWRQKGAVTPVKNQGSCGSAWAFSAVSTIESIIKIRTGN
LNEYSEQELLDCDRRSYGCNGGYPWSALQLVAQYGIHYRNTYPYEGVQRYCRSREKGPYAAKTDG
VRQVQPYNEGALLYSIANQPVSVVLEAAGKDFQLYRGGIFVGPCGNKVDHAVAAVGYGPNYILIR
NSWGTGWGENGYIRIKRGTGNSYGVCGLYTSSFYPVKN
>3ZZOA
ECCTSRELVEFKMDRGDCEAVRAIENYPNGCEVTICADGVAQLGAYCGQGPCNIFGCNCDGGCLS
GDWSQEFVRRNQQYGIQIIKVTRLPFWRPL
>3TMUA
MRSLLILVLCFLPLAALGKVFGRCELAAAMKRHGLDNYRGYSLGNWVCAAKFESNFNTQATNRNT
DGSTDYGILQINSRWWCNDGRTPGSRNLCNIPCSALLSSDITASVNCAKKIVSDGNGMNAWVAWR
NRCKGTDVQAWIRGCRL
>3TKLB
GPLGSTSSTSQADKEIQKMLDEYEQAIKRAQENIKKGEELEKKLDKLERQGKDLEDKYKTYEENL
EGFEKLLTDSEELSLSEINEKMKAFSKDSEKLTQLMEKHKGDEKTVQSLQREHHDIKAKLANLQV
LHDAHTGKKSYVNEKGNPVSSLKDAHLAINKDQEVVEHKGQFYLLQKGQWDAIKNDPAALEKAQK
DYSQSKHDLATIKMEALIHKLSLEMEKQLETINDLIMSTDPKENEEATKLLHKHNGLNLKLANLQ
DMLAVHR
>3TIKA
KGKLPPVYPVTVPILGHIIQFGKSPLGFMQECKRQLKSGIFTINIVGKRVTIVGDPHEHSRFFLP
RNEVLSPREVYSFMVPVFGEGVAYAAPYPRMREQLNFLAEELTIAKFQNFVPAIQHEVRKFMAAN
WDKDEGEINLLEDCSTMIINTACQCLFGEDLRKRLDARRFAQLLAKMESSLIPAAVFLPILLKLP
LPQSARCHEARTELQKILSEIIIARKEEEVNKDSSTSDLLSGLLSAVYRDGTPMSLHEVCGMIVA
AMFAGQHTSSITTTWSMLHLMHPANVKHLEALRKEIEEFPAQLNYNNVMDEMPFAERCARESIRR
DPPLLMLMRKVMADVKVGSYVVPKGDIIACSPLLSHHDEEAFPEPRRWDPERDEKVEGAFIGFGA
GVHKCIGQKFGLLQVKTILATAFRSYDFQLLRDEVPDPDYHTMVVGPTASQCRVKYIRRKAAAA
>3TH0A
YDPDQYSIEADKKFKYSVKLSDYPTLQDAASAAVDGLLIDRDYNFYGGETVDFGGKVLTIECKAK
FIGDGNLIFTKLGKGSRIAGVFMESTTTPWVIKPWTDDNQWLTDAAAVVATLKQSKTDGYQPTVS

DYVKFPGIETLLPPNAKGQNITSTLEIRECIGVEVHRASGLMAGFLFRGCHFCKMVDANNPSGGK
DGIITFENLSGDWGKGNYVIGGRTSYGSVSSAQFLRNNGGFERDGGVIGFTSYRAGESGVKTWQG
TVGSTTSRNYNLQFRDSVVIYPVWDGFDLGADTDMNPELDRPGDYPITQYPLHQLPLNHLIDNLL
VRGALGVGFGMDGKGMYVSNITVEDCAGSGAYLLTHESVFTNIAIIDTNTKDFQANQIYISGACR
VNGLRLIGIRSTDGQSLTIDAPNSTVSGITGMVDPSRINVANLAEEGLGNIRANSFGYDSAAIKL
RIHKLSKTLDSGALYSHINGGAGSGSAYTQLTAISGSTPDAVSLKVNHKDCRGAEIPFVPDIASD
DFIKDSSCFLPYWENNSTSLKALVKKPNGELVRLTLATL

>3TGUB

SLKVAPKVAVSAAAERVKLCPGAEDLEITKLPNGLIIASLENFSPASRIGVFIKAGSRYETTANL
GTAHLLRLASPLTTKGASSFRITRGIEAVGGSLSVYSTREKMTYCVECLRDHVDTVMEYLLNVTT
APEFRPWEVTDLQPQLKVDKAVAFQSPQVGVLENLHAAAYKTALANPLYCPDYRIGKITSEQLHH
FVQNNFTSARMALVGIGVKHSDLKQVAEQFLNIRSGAGTSSAKATYWGGEIREQNGHSLVHAAVV
TEGAAVGSAEANAFSVLQHVLGAGPLIKRGSSVTSKLYQGVAKATTQPFDASAFNVNYSDSGLFG
FYTISQAAHAGEVIRAAMNQLKAAAQGGVTEEDVTKAKNQLKATYLMSVETAQGLLNEIGSEALL
SGTHTAPSVVAQKIDSVTSADVVNAAKKFVSGKKSMAASGDLGSTPFLDEL

>3ZXUB

MDFTSSSGVLDSERNTGSNDSDEPSSHSDVIETEELKLIKLQEHKNNLLRQRSELLDQLSQTRVV
EPRSVQLDDKLLLKLLRRNDNAVSDSSQSSNNPLPRVLPSLNIEQRKKYLDITLNDVTVTCEKDM
ILLRKGSFTASFRIAVENESIRSMAIDLNAFEVELQPIIQYAEDTQNVNVAMMAVVQFLRIKELH
EQMISKIVEASKFIRASNNTITLNDLEVSFHCYWNLPSPYPETLILTNKVQKILDFLIYQYGIQL
GVIKYGSTII

>2LHRA

SDDYVDEETYNLQKLLAPYHKAKTLERQVYELEKLQEKLPEKYKAEYKKKLDQTRVELADQVKSA
VTEFENVTPTNDQ

>3TEEA

QDINAQLTTWFSQRLAGFSDEVVVTLRSSPNLLPSCEQPAFSMTGSAKLWGNVNVVARCANEKRY
LQVNVQATGNYVAVAAPIARGGKLTPANVTLKRGRLDQLPPRTVLDIRQIQDAVSLRDLAPGQPV
QLTMIRQAWRVKAGQRVQVIANGEGFSVNAEGQAMNNAAVAQNARVRMTSGQIVSGTVDSDGNIL
INLDPNSSSVDKLAAALEHHHHHH

>3TDOA

MGRAHKETLDKLTNAAINKINLLNTSKVKYLVSSAFAGLYVGIGILLIFTIGGLLTDAGSPMTKI
VMGLSFAIALSLVIMTGTELFTGNNMVMSAGMLNKGVSIKDTSKIWAYSWVGNLIGALVLGIIFV
GTGLVDKGPVAEFFANTAASKASMPFTALFFRGILCNILVCVSVLCSFRTNSDTAKIIMIFLCLF
AFITSGFEHSVANMTIYSVSLFSPTISTVTIGGAIYNLVAVTLGNIVGGALFMGLGTYILGKEKL
NAAAENLY

>3TCJA

MSQFTLYKNKDKSSAKTYPYFVDVQSDLLDNLNTRLVIPLTPIELLDKKAPSHLCPTIHIDEGDF
IMLTQQMTSVPVKILSEPVNELSTFRNEIIAAIDFLITGI

>3ZXBA

FTCPECRPELCGDPGYCEYGTTKDACDCCPVCFQGPGGYCGGPEDVFGICADGFACVPLVGERDS
QDPEIVGTCVKIP

>3VGBA

TFAYKIDGNEVIFTLWAPYQKSVKLKVLEKGLYEMERDEKGYFTITLNNVKVRDRYKYVLDDASE
IPDPASRYQPEGVHGPSQIIQESKEFNNETFLKKEDLIIYEIHVGTFTPEGTFEGVIRKLDYLKD

LGITAIEIMPIAQFPGKRDWGYDGVYLYAVQNSYGGPEGFRKLVDEAHKKGLGVILDVVYNHVGP
EGNYMVKLGPYFSQKYKTPWGLTFNFDDAESDEVRKFILENVEYWIKEYNVDGFRLDAVHAIIDT
SPKHILEEIADVVHKYNRIVIAESDLNDPRVVNPKEKCGYNIDAQWVDDFHHSIHAYLTGERQGY
YTDFGNLDDIVKSYKDVFVYDGKYSNFRRKTHGEPVGELDGCNFVVYIQNHDQVGNRGKGERIIK
LVDRESYKIAAALYLLSPYIPMIFMGEEYGEENPFYFFSDFSDSKLIQGVREGRKKENGQDTDPQ
DESTFNASKLSWKIDEEIFSFYKILIKMRKELSIACDRRVNVVNGENWLIIKGREYFSLYVFSKS
SIEVKYSGTLLLSSNNSFPQHIEEGKYEFDKGFALYKL
>3ZX8A
MENDPRVRKFASDGAQWAIKWQKKGWSTLTSRQKQTARAAMGIKLSPVAQPVQKVTRLSAPVALA
YREVSTQPRVSTARDGITRSGSELITTLKKNTDTEPKYTTAVLNPSEPGTFNQLIKEAAQYEKYR
FTSLRFRYSPMSPSTTGGKVALAFDRDAAKPPPNDLASLYNIEGCVSSVPWTGFILTVPTDSTDR
FVADGISDPKLVDFGKLIMATYGQGAAQLGEVRVEYTVQLKNRTGSTSAQIGDFAGVKDGPRLVS
WSKTKGTAGWEHDCHFLGTGNFSLTLFYEKAPVSGLENADASDFSVLGEAAAGSVQWAGVKVAER
GQGVKMVTTEEQPKGKWQALRI
>3TBLA
MFEARLVQGSILKKVLEALKDLINEACWDISSSGVNLQSMDSSHVSLVQLTLRSEGFDTYRCDRN
LAMGVNLTSMSKILKCAGNEDIITLRAEDNADTLALVFEAPNQEKVSDYEMKLMDLDVEQLGIPE
QEYSCVVKMPSGEFARICRDLSHIGDAVVISCAKDGVKFSASGELGNGNIKLSQTSNVDKEEEAV
TIEMNEPVQLTFALRYLNFFTKATPLSSTVTLSMSADVPLVVEYKIADMGHLKYYLAPKIEDEEG
S
>3TAYA
GSLLDGPYQPTTFNPPTSYWILLAPTVEGVVIQGTNNIDRWLATILIEPNVQTTNRIYNLFGQQV
TLSVENTSQTQWKFIDVSKTTPTGNYTQHGSLFSTPKLYAVMKFSGRIYTYNGTTPNATTGYYST
TNYDTVNMTSFCDFYIIPRNQEEKCTEYINHGL
>3ZWSA
MATGDERFYAEHLMPTLQGLLDPESAHRLAVRFTSLGLLPRARFQDSDMLEVRVLGHKFRNPVGI
AAGFDKHGEAVDGLYKMGFGFVEIGSVTPKPQEGNPRPRVFRLPEDQAVINRYGFNSHGLSVVEH
RLRARQQKQAKLTEDGLPLGVNLGKNKTSVDAAEDYAEGVRVLGPLADYLVVNVSSPNTAGLRSL
QGKAELRRLLTKVLQERDGLRRVHRPAVLVKIAPDLTSQDKEDIASVVKELGIDGLIVTNTTVSR
PAGLQGALRSETGGLSGKPLRDLSTQTIREMYALTQGRVPIIGVGGVSSGQDALEKIRAGASLVQ
LYTALTFWGPPVVGKVKRELEALLKEQGFGGVTDAIGADHRR
>3T60A
MHLKIVCLSDEVREMYKNHKTHHEGDSGLDLFIVKDEVLKPKSTTFVKLGIKAIALQYKSNYYYK
CEKSENKKKDDDKSNIVNTSFLLFPRSSISKTPLRLANSIGLIDAGYRGEIIAALDNTSDQEYHI
KKNDKLVQLVSFTGEPLSFELVEELDETSRGEGGFGSTSNNKYLEHHHHHH
>3T5VA
GSPLPSDVRPPHILVKTLDYIVDNLLTTLPESEGFLWDRMRSIRQDFTYQNYSGPEAVDCNERIV
RIHLLILHIMVKSNVEFSLQQELEQLHKSLITLSEIYDDVRSSGGTCPNEAEFRAYALLSKIRDP
QYDENIQRLPKHIFQDKLVQMALCFRRVISNSAYTERGFVKTENCLNFYARFFQLMQSPSLPLLM
GFFLQMHLTDIRFYALRALSHTLNKKHKPIPFIYLENMLLFNNRQEIIEFCNYYSIEIINGDAAD
LKTLQHYSHKLSETQPLKKTYLTCLERRLQKTTYKGLINGGEDNLASSVYVKDPKK
>3T63M
PAQDNSRFVIRDRNWHPKALTPDYKTSIARSPRQALVSIPQSISETTGPNFSHLGFGAHDHDLLL
NFNNGGLPIGERIIVAGRVVDQYGKPVPNTLVEMWQANAGGRYRHKNDRYLAPLDPNFGGVGRCL

TDSDGYYSFRTIKPGPAPWRNGPNDWRPAHIYFGISGPSIATKLITQLYFEGDPLIPMCPIVKSI
ANPEAVQQLIAKLDMNNANPMDCLAYRFDIVLRGQRKTHFENC
>3T6BA
MADTQYILPNDIGVSSLDCREAFRLLSPTERLYAYHLSRAAWYGGLAVLLQTSPEAPYIYALLSR
LFRAQDPDQLRQHALAEGLTEEEYQAFLVYAAGVYSNMGNYKSFGDTKFVPNLPKEKLERVILGS
EAAQQHPEEVRGLWQTCGELMFSLEPRLRHLGLGKEGITTYFSGNCTMEDAKLAQDFLDSQNLSA
YNTRLFKEVDGEGKPYYEVRLASVLGSEPSLDSEVTSKLKSYEFRGSPFQVTRGDYAPILQKVVE
QLEKAKAYAANSHQGQMLAQYIESFTQGSIEAHKRGSRFWIQDKGPIVESYIGFIESYRDPFGSR
GEFEGFVAVVNKAMSAKFERLVASAEQLLKELPWPPTFEKDKFLTPDFTSLDVLTFAGSGIPAGI
NIPNYDDLRQTEGFKNVSLGNVLAVAYATQREKLTFLEEDDKDLYILWKGPSFDVQVGLHALLGH
GSGKLFVQDEKGAFNFDQETVINPETGEQIQSWYRSGETWDSKFSTIASSYEECRAESVGLYLCL
HPQVLEIFGFEGADAEDVIYVNWLNMVRAGLLALEFYTPEAFNWRQAHMQARFVILRVLLEAGEG
LVTITPTTGSDGRPDARVRLDRSKIRSVGKPALERFLRRLQVLKSTGDVAGGRALYEGYATVTDA
PPECFLTLRDTVLLRKESRKLIVQPNTRLEGSDVQLLEYEASAAGLIRSFSERFPEDGPELEEIL
TQLATADARFW
>3T5AA
MGSSHHHHHHSSGLVPRGSHMSVRSLPAALRACARLQPHDPAFTFMDYEQDWDGVAITLTWSQLY
RRTLNVAQELSRCGSTGDRVVISAPQGLEYVVAFLGALQAGRIAVPLSVPQGGVTDERSDSVLSD
SSPVAILTTSSAVDDVVQHVARRPGESPPSIIEVDLLDLDAPNGYTFKEDEYPSTAYLQYTSGST
RTPAGVVMSHQNVRVNFEQLMSGYFADTDGIPPPNSALVSWLPFYHDMGLVIGICAPILGGYPAV
LTSPVSFLQRPARWMHLMASDFHAFSAAPNFAFELAARRTTDDDMAGRDLGNILTILSGSERVQA
ATIKRFADRFARFNLQERVIRPSYWLAEATVYYVATSKPGQPPETVDFDTESLSAGHAKPCAGGGA
TSLISYMLPRSPIVRIVDSDTCIECPDGTVGEIWVHGDNVANGYWQKPDESERTFGGKIVTPSPG
TPEGPWLRTGDSGFVTDGKMFIIGR
>2LGDA
GSMALPIIVKWGGQEYSVTTLSEDDTVLDLKQFLKTLTGVLPERQKLLGLKVKGKPAENDVKLGA
LKLKPNTKIMMMGTREES
>3T1HR
MSTKNAKPKKEAQRRPSRKAKVKATLGEFDLRDYRNVEVLKRFLSETGKILPRRRTGLSGKEQRI
LAKTIKRARILGLLPFTEKLVRK
>3T1HQ
MPKKVLTGVVVSDKMQKTVTVLVERQFPHPLYGKVIKRSKKYLAHDPEEKYKLGDVVEIIESRPI
SKRKRFRVLRLVESGRMDLVEKYLIRRQNYQSLSKRGGKA
>3ZUIA
DSESDCTGSEPVDAFQAFSEGKEAYVLVRSTDPKARDCLKGEPAGEKQDNTLPVMMTFKNGTDWA
STDWTFTLDGAKVTATLGNLTQNREVVYDSQSHHCHVDKVEKEVPDYEMWMLDAGGLEVEVECCR
QKLEELASGRNQMYPHLKDC
>3SX6A
MRGSAHVVILGAGTGGMPAAYEMKEALGSGHEVTLISANDYFQFVPSNPWVGVGWKERDDIAFPI
RHYVERKGIHFIAQSAEQIDAEAQNITLADGNTVHYDYLMIATGPKLAFENVPGSDPHEGPVQSI
CTVDHAERAFAEYQALLREPGPIVIGAMAGASCFGPAYEYAMIVASDLKKRGMRDKIPSFTFITS
EPYIGHLGIQGVGDSKGILTKGLKEEGIEAYTNCKVTKVEDNKMYVTQVDEKGETIKEMVLPVKF
GMMIPAFKGVPAVAGVEGLCNPGGFVLVDEHQRSKKYANIFAAGIAIAIPPVETTPVPTGAPKTG
YMIESMVSAAVHNIKADLEGRKGEQTMGTWNAVAFADMGDRGAAFIALPQLKPRKVDVFAYGRWV

HLAKVAFEKYFIRKMKMGVSEPFYEKVLFKMMGITRLKEEDTHRKAS
>3SWMA
HHHHHHMGIQETDPLTQLSLPPGFRFYPTDEELMVQYLCRKAAGYDFSLQLIAEIDLYKFDPWVL
PNKALFGEKEWYFFSPRDRKYPNGSRPNRVAGSGYWKATGTDKIISTEGQRVGIKKALVFYIGKA
PKGTKTNWIMHEYRLIEPSRRNGSTKLDDWVLCRIYKKQSSAQK
>3STTA
GSMEKSMSPFVKKHFVLVHTAFHGAWCWYKIVALMRSSGHNVTALDLGASGINPKQALQIPNFSD
YLSPLMEFMASLPANEKIILVGHALGGLAISKAMETFPEKISVAVFLSGLMPGPNIDATTVCTKA
GSAVLGQLDNCVTYENGPTNPPTTLIAGPKFLATNVYHLSPIEDLALATALVRPLYLYLAEDISK
EVVLSSKRYGSVKRVFIVATENDALKKEFLKLMIEKNPPDEVKEIEGSDHVTMMSKPQQLFTTLL
SIANKYK
>3SS3A
GSHMVAAGDNKIKQGLLPSLEDLLFYTIAEGQEKIPVHKFITALKSTGLRTSDPRLKECMDMLRL
TLQTTSDGVMLDKDLFKKCVQSNIVLLTQAFRRKFVIPDFMSFTSHIDELYESAKKQSGGKVADY
IPQLAKFSPDLWGVSVCTVDGQRHSIGDTKVPFCLQSCVKPLKYAIAVNDLGTEYVHRYVGKEPS
GLRFNKLFLNEDDKPHNPMVNAGAIVVTSLIKQGVNNAEKFDYVMQFLNKMAGNEYVGFSNATFQ
SERESGDRNFAIGYYLKEKKCFPEGTDMVGILDFYFQLCSIEVTCESASVMAATLANGGFCPITG
ERVLSPEAVRNTLSLMHSCGMYDFSGQFAFHVGLPAKSGVAGGILLVVPNVMGMMCWSPPLDKMG
NSVKGIHFCHDLVSLCNFHNYDNLRHFAKKLDPRREGGDQRHSFGPLDYESLQQELALKDTVWKK
VSPESSDDTSTTVVYRMESLGERS
>3SQRA
MKYFTVFTALTALFAQASASAIPAVRSTLTPRQNTTASCANSATSRSCWGEYSIDTNWYDVTPTG
VTREYWLSVENSTITPDGYTRSAMTFNGTVPGPAIIADWGDNLIIHVTNNLEHNGTSIHWHGIRQ
LGSLEYDGVPGVTQCPIAPGDTLTYKFQVTQYGTTWYHSHFSLQYGDGLFGPLIINGPATADYDE
DVGVIFLQDWAHESVFEIWDTARLGAPPALENTLMNGTNTFDCSASTDPNCVGGGKKFELTFVEG
TKYRLRLINVGIDSHFEFAIDNHTLTVIANDLVPIVPYTTDTLLIGIGQRYDVIVEANAAADNYW
IRGNWGTTCSTNNEAANATGILRYDSSSIANPTSVGTTPRGTCEDEPVASLVPHLALDVGGYSLV
DEQVSSAFTNYFTWTINSSSLLLDWSSPTTLKIFNNETIFPTEYNVVALEQTNANEEWVVYVIED
LTGFGIWHPIHLHGHDFFIVAQETDVFNSDESPAKFNLVNPPRRDVAALPGNGYLAIAFKLDNPG
SWLLHCHIAWHASEGLAMQFVESQSSIAVKMTDTAIFEDTCANWNAYTPTQLFAEDDSGI
>3SO6A
MEGMVFSLKYLGMTLVERPKGEELSAAAVKRIVATAKASGKKLQKVTLKVSPRGIILTDSLTSQL
IENVSIYRISYCTADKMHDKVFAYIAQSQQNESLECHAFLCTKRKVAQAVTLTVAQAFKVAFEFW
QVSLVPR
>3SMHA
SRNNPFYFPSRRFSTRYGNQNGRIRVLQRFDQRSRQFQNLQNHRIVQIEAKPNTLVLPKHADADN
ILVIQQGQATVTVANGNNRKSFNLDEGHALRIPSGFISYILNRHDNQNLRVAKISMPVNTPGQFE
DFFPASSRDQSSYLQGFSRNTLEAAFNAEFNEIRRVLLEENAGGEQEERGQRRWSTRSSENNEGV
IVKVSKEHVEELTKHAKSVSKKGSEEEGDITNPINLREGEPDLSNNFGKLFEVKPDKKNPQLQDL
DMMLTCVEIKEGALVLPHFNSKAMVIVVVNKGTGNLELVAVRKEQQQRGRREEEEDEDEEEEGSN
REVRRYTARLKEGDVFIMPAAHPVAINASSELHLLGFGINAENNHRIFLAGDKDNVIDQIEKQAK
DLAFPGSGEQVEKLIKNQKESHFVSARP
>3B18A
MSHTDLTPCTRVLASSGTVPIAEELLARVLEPYSCKGCRYLIDAQYSATEDSVLAYGNFTIGESA

YIRSTGHFNAVELILCFNQLAYSAFAPAVLNEEIRVLRGWSIDDYCQHQLSSMLIRKASSRFRKP
LNPQKFSARLLCRDLQVIERTWRYLKVPCVIEFWDENGGAASGEIELAALNIP
>3ZSCA
SLNDKPVGFASVPTADLPEGTVGGLGGEIVFVRTAEELEKYTTAEGKYVIVVDGTIVFEPKREIK
VLSDKTIVGINDAKIVGGGLVIKDAQNVIIRNIHFEGFYMEDDPRGKKYDFDYINVENSHHIWID
HITFVNGNDGAVDIKKYSNYITVSWNKFVDHDKVSLVGSSDKEDPEQAGQAYKVTYHHNYFKNLI
QRMPRIRFGMAHVFNNFYSMGLRTGVSGNVFPIYGVASAMGAKVHVEGNYFMGYGAVMAEAGIAF
LPTRIMGPVEGYLTLGEGDAKNEFYYCKEPEVRPVEEGKPALDPREYYDYTLDPVQDVPKIVVDG
AGAGKLVFEELNTAQ
>2LENA
MQLKPMEINPEMLNKVLYRLGVAGQWRFVDVLGLEEESLGSVPAPACALLLLFPLTAQHENFRKK
QIEELKGQEVSPKVYFMKQTIGNSCGTIGLIHAVANNQDKLGFEDGSVLKQFLSETEKMSPEDRA
KCFEKNEAIQAAHDAVAQEGQCRVDDKVNFHFILFNNVDGHLYELDGRMPFPVNHGASSEDTLLK
DAAKVCREFTEREQGEVRFSAVALCKAALEHHHHHH
>2LELA
VDMSNVVKTYDLQDGSKVHVFKDGKMGMENKFGKSMNMPEGKVMETRDGTKIIMKGNEIFRLDEA
LRKGHSEGG
>3SGZA
PLVCLADFKAHAQKQLSKTSWDFIEGEADDGITYSENIAAFKRIRLRPRYLRDMSKVDTRTTIQG
QEISAPICISPTAFHSIAWPDGEKSTARAAQEANICYVISSYASYSLEDIVAAAPEGFRWFQLYM
KSDWDFNKQMVQRAEALGFKALVITIDTPVLGNRRRDKRNQLNLEANILKAALRALKEEKPTQSV
PVLFPKASFCWNDLSLLQSITRLPIILKGILTKEDAELAMKHNVQGIVVSNHGGRQLDEVSASID
ALREVVAAVKGKIEVYMDGGVRTGTDVLKALALGARCIFLGRPILWGLACKGEDGVKEVLDILTA
ELHRCMTLSGCQSVAEISPDLIQFSRL
>3SGFY
MAAKIRRDDEVIVLTGKDKGKRGKVKNVLSSGKVIVEGINLVKKHQKPVPALNQPGGIVEKEAAI
QVSNVAIFNAATGKADRVGFRFEDGKKVRFFKSNSETIK
>3ZQDA
GRKLLTYQVKQGDTLNSIAADFRISTAALLQANPSLQAGLTAGQSIVIPGLPDPYTIPYHIAVSI
GAKTLTLSLNNRVMKTYPIAVGKILTQTPTGEFYIINRQRNPGGPFGAYWLSLSKQHYGIHGTNN
PASIGKAVSKGCIRMHNKDVIELASIVPNGTRVTINRGSHHHHHH
>3SCIE
RVVPSGDVVRFPNITNLCPFGEVFNATKFPSVYAWERKKISNCVADYSVLYNSTFFSTFKCYGVS
ATKLNDLCFSNVYADSFVVKGDDVRQIAPGQTGVIADYNYKLPDDFMGCVLAWNTRNIDATSTGN
YNYKYRFLRHGKLRPFERDISNVPFSPDGKPCTPPAFNCYWPLNDYGFYTTTGIGYQPYRVVVLS
FELLNAPATVCGPKLSTDLIKNQCVNFHHHHHH
>2YMAA
GSIGSNSIDLITKYEPIFLGSGIYFLRPFNTDERDKLMVTDNAMSNWDEITETYYQKFGNAINKM
LSLRLVSLPNGHILQPGDSCVWLAEVVDMKDRFQTTLSLNILNSQRAEIFFNKTFTFNEDNGNFL
SYKIGDHGESTELGQITHSNKADINTAEIRS
>3SBSA
MNTVPFTSAPIEVTIGIDQYSFNVKENQPFHGIKDIPIGHVHVIHFQHADNSSMRYGYWFDCRMG
NFYIQYDPKDGLYKMMEERDGAKFENIVHNFKERQMMVSYPKIDEDDTWYNLTEFVQMDKIRKIV
RKDENQFSYVDSSMTTVQENELLKSSLQKAGSKMEAKNEDDPAHSLNYTVINFKSREAIRPGHEM

EDFLDKSYYLNTVMLQGIFKNSSNYFGELQFAFLNAMFFGNYGSSLQWHAMIELICSSATVPKHM
LDKLDEILYYQIKTLPEQYSDILLNERVWNICLYSSFQKNSLHNTEKIMENKYPELLGKDNEDDA
LIYGISDEERDDEDDEHNPTIVGGLYYQRPLEHHHHHH
>2YJGA
MANIEIPYGKSKLAFDLPDERIQGILRSKAGSYKVNMSEEDIVKRALENPIGTKRLQDLAEGKKN
IVIITSDHTRPVPSRITLPLLLDEIRKKNKSANVKILIATGFHRGTTLQEMKAKFGEDLVENEQF
VVHDSRNSENMELIGTLPSGGKLEINKLAVEADLLVAEGFIEPHFFAGFSGGRKSILPGIASVQC
ILANHCSEFIKNPYARTGVLENNPIHRDMIYAAKKANLAFILNVVIDSSHKIVNAFAGHSEKAHL
KGCEFVSEIATVNAKPADIVITSNGGYPLDQNIYQSVKGMTAGEAACKDGGVIIIAAECADGHGG
EGFYRWFKESKDPQDVMNKILSRGRDETLPDQWEAQILARILINHKVIMVTDSKNYEYVKDMFMT
PAKDLGEALKIAESIVNNDSKINVIPDGVSVIVREKASWSHPQFEK
>3S4EA
ASQVGVIKPWLLLGSQDAAHDLDTLKKNKVTHILNVAYGVENAFLSDFTYKSISILDLPETNILS
YFPECFEFIEEAKRKDGVVLVHSNAGVSRAAAIVIGFLMNSEQTSFTSAFSLVKNARPSICPNSG
FMEQLRTYQEGKES
>3S44A
MKTITLYLDPASLPALNQLMDFTQNNEDKTHPRIFGLSRFKIPDNIITQYQNIHFVELKDNRPTE
ALFTILDQYPGNIELNIHLNIAHSVQLIRPILAYRFKHLDRVSIQQLNLYDDGSDEYVDLEKEEN
KDISAEIKQAEKQLSHYLLTGKIKFDNPTIARYVWQSAFPVKYHFLSTDYFEKAEFLQPLKEYLA
ENYQKMDWTAYQQLTPEQQAFYLTLVGFNDEVKQSLEVQQAKFIFTGTTTWEGNTDVREYYAQQQ
LNLLNHFTQAEGDLFIGDHYKIYFKGHPRGGEINDYILNNAKNITNIPANISFEVLMMTGLLPDK
VGGVASSLYFSLPKEKISHIIFTSNKQVKSKEDALNNPYVKVMRRLGIIDESQVIFWDSLKQLGG
GLEHHHHHH
>3S2SA
MGSSHHHHHHSSGLVPRGSHMASMTGGQQMGRGSMSKALISIDYTYDFVADDGKLTAGKPAQAIS
KAIAQVTQKAYDNGDYIFFTIDGHDEGDDFHPETKLFPPHNIKGTSGRDLYGALADFYQKHENDK
RVFWMDKRHYSAFSGTDLDIRLRERRVDTVVLTGVLTDICVLHTAIDAYNLGYQIEVVQSAVASL
SQENHQFALNHLQNVLGATIIE
>3S2QA
GSHMRKQQRMVVVRAEGGGGINPEIRKNEDKVVDSVVVTELSKNITPYCRCWRSGTFPLCDGSHV
KHNKANGDNVGPLLLKKQ
>3S0PA
ATKKAVAVLKGNSNVEGVVTLSQDDDGPTTVNVRITGLAPGLHGFHLHEYGDTTNGCMSTGAHFN
PNKLTHGAPGDEIRHAGDLGNIVANADGVAEVTLVDNQIPLTGPNSVVGRALVVHELEDDLGKGG
HELSLTTGNAGGRLACGVVGLTPI
>3AYQA
FAGGTVSQRCLSCICKMESGCRNVGCKMDMGSLSCGYFQIKEAYWIDCGRPGSSWKSCAASSYCA
SLCVQNYMKRYAKWAGCPLRCEGFAREHNGGPRGCKKGSTIGYWNRLQKISGCHGVQ
>3RZIA
MNWTVDIPIDQLPSLPPLPTDLRTRLDAALAKPAAQQPTWPADQALAMRTVLESVPPVTVPSEIV
RLQEQLAQVAKGEAFLLQGGDCAETFMDNTEPHIRGNVRALLQMAVVLTYGASMPVVKVARIAGQ
YAKPRSADIDALGLRSYRGDMINGFAPDAAAREHDPSRLVRAYANASAAMNLVRALTSSGLASLH
LVHDWNREFVRTSPAGARYEALATEIDRGLRFMSACGVADRNLQTAEIYASHEALVLDYERAMLR
LSDGDDGEPQLFDLSAHTVWIGERTRQIDGAHIAFAQVIANPVGVKLGPNMTPELAVEYVERLDP

HNKPGRLTLVSRMGNHKVRDLLPPIVEKVQATGHQVIWQCDPMHGNTHESSTGFKTRHFDRIVDE
VQGFFEVHRALGTHPGGIHVEITGENVTECLGGAQDISETDLAGRYETACDPRLNTQQSLELAFL
VAEMLRD
>3RQOA
RAPAPATPHAPDHSPAPNSPTLTRPPEGPKFPRVKNWELGSITYDTLCAQSQQDGPCTPRRCLGS
LVLPRKLQTRPSPGPPPAEQLLSQARDFINQYYSSIKRSGSQAHEERLQEVEAEVASTGTYHLRE
SELVFGAKQAWRNAPRCVGRIQWGKLQVFDARDCSSAQEMFTYICNHIKYATNRGNLRSAITVFP
QRAPGRGDFRIWNSQLVRYAGYRQQDGSVRGDPANVEITELCIQHGWTPGNGRFDVLPLLLQAPD
EAPELFVLPPELVLEVPLEHPTLEWFAALGLRWYALPAVSNMLLEIGGLEFSAAPFSGWYMSTEI
GTRNLCDPHRYNILEDVAVCMDLDTRTTSSLWKDKAAVEINLAVLHSFQLAKVTIVDHHAATVSF
MKHLDNEQKARGGCPADWAWIVPPISGSLTPVFHQEMVNYILSPAFRYQPDPW
>3RO9A
KVPVVGIVAALLPEMGIGFQGNLPWRLAKEMKYFREVTTLTNDNSKQNVVIMGRKTWESIPQKFR
PLPKRINVVVSRSFDGELRKVEDGIYHSNSLRNCLTALQSSLANENKIERIYIIGGGEIYRQSMD
LADHWLITKIMPLPETTIPQMDTFLQKQELEQRFYDNSDKLVDFLPSSIQLEGRLTSQEWNGELV
KGLPVQEKGYQFYFTLYTKKLEHHHHHHHH
>3RMJA
GIDPFTMTQTNRVIIFDTTLRDGEQSPGAAMTKEEKIRVARQLEKLGVDIIEAGFAAASPGDFEA
VNAIAKTITKSTVCSLSRAIERDIRQAGEAVAPAPKKRIHTFIATSPIHMEYKLKMKPKQVIEAA
VKAVKIAREYTDDVEFSCEDALRSEIDFLAEICGAVIEAGATTINIPDTVGYSIPYKTEEFFREL
IAKTPNGGKVVWSAHCHNDLGLAVANSLAALKGGARQVECTVNGLGERAGNASVEEIVMALKVRH
DLFGLETGIDTTQIVPSSKLVSTITGYPVQPNKAIVGANAFSHESGIHQDGVLKHRETYEIMSAE
SVGWATNRLSLGKLSGRNAFKTKLADLGIELESEEALNAAFARFK
>3AXXA
MEGNTILKIVLICTILAGLFGQVVPVYAENTTYQTPTGIYYEVRGDTIYMINVTSGEETPIHLFG
VNWFGFETPNHVVHGLWKRNWEDMLLQIKSLGFNAIRLPFCTESVKPGTQPIGIDYSKNPDLRGL
DSLQIMEKIIKKAGDLGIFVLLDYHRIGCTHIEPLWYTEDFSEEDFINTWIEVAKRFGKYWNVIG
ADLKNEPHSVTSPPAAYTDGTGATWGMGNPATDWNLAAERIGKAILKVAPHWLIFVEGTQFTNPK
TDSSYKWGYNAWWGGNLMAVKDYPVNLPRNKLVYSPHVYGPDVYNQPYFGPAKGFPDNLPDIWYH
HFGYVKLELGYSVVIGEFGGKYGHGGDPRDVIWQNKLVDWMIENKFCDFFYWSWNPDSGDTGGIL
QDDWTTIWEDKYNNLKRLMDSCSKSSSSTQSVIRSTTPTKSNTSKKICGPAILIIILAVFSLLLRR
APR
>3RKLA
MSKITINIKDNTIEYGHKEFVLSNLQEDIKNLAEIVYQLAKLIEKLSQYEEEVDTELYNLLHEYA
IYLAGATSMFIDSENKHHHHHH
>3RIKA
ARPCIPKSFGYSSVVCVCNATYCDSFDPPTFPALGTFSRYESTRSGRRMELSMGPIQANHTGTGL
LLTLQPEQKFQKVKGFGGAMTDAAALNILALSPPAQNLLLKSYFSEEGIGYNIIRVPMASCDFSI
RTYTYADTPDDFQLHNFSLPEEDTKLKIPLIHRALQLAQRPVSLLASPWTSPTWLKTNGAVNGKG
SLKGQPGDIYHQTWARYFVKFLDAYAEHKLQFWAVTAENEPSAGLLSGYPFQCLGFTPEHQRDFI
ARDLGPTLANSTHHNVRLLMLDDQRLLLPHWAKVVLTDPEAAKYVHGIAVHWYLDFLAPAKATLG
ETHRLFPNTMLFASEACVGSKFWEQSVRLGSWDRGMQYSHSIITNLLYHVVGWTDWNLALNPEGG
PNWVRNFVDSPIIVDITKDTFYKQPMFYHLGHFSKFIPEGSQRVGLVASQKNDLDAVALMHPDGS
AVVVVLNRSSKDVPLTIKDPAVGFLETISPGYSIHTYLWHRQ

>3RFYA
GSPGISGGGGGILLVANPVIPDVSVLISGPPIKDPEALLRYALPIDNKAIREVQKPLEDITDSLK
IAGVKALDSVERNVRQASRTLQQGKSIIVAGFAESKKDHGNEMIEKLEAGMQDMLKIVEDRKRDA
VAPKQKEILKYVGGIEEDMVDGFPYEVPEEYRNMPLLKGRASVDMKVKIKDNPNIEDCVFRIVLD
GYNAPVTAGNFVDLVERHFYDGMEIQRSDGFVVQTGDPEGPAEGFIDPSTEKTRTVPLEIMVTGE
KTPFYGSTLEELGLYKAQVVIPFNAFGTMAMAREEFENDSGSSQVFWLLKESELTPSNSNILDGR
YAVFGYVTDNEDFLADLKVGDVIESIQVVSGLENLANPSYKIAG
>2YFKA
METFKEYIEKLDKLEFEKMYENDFFLTWEKTRDELEAVFTVADTLRYLRENNISTKIFDSGLGIS
LFRDNSTRTRFSFASACNLLGLEVQDLDEGKSQISHGETVRETANMISFMADIIGIRDDMYIGKG
NAYMHEVSESVQEGYKDGVLEQRPTLVNLQCDIDHPTQAMADALHLIHEFGGIENLKGKKVAMTW
AYSPSYGKPLSVPQGIVGLMTRLGMDVVLAHPEGYEIMPEVEEVAKKNAAEFGGNFTKTNSMAEA
FKDADVVYPKSWAPFAAMEKRTELYGNGDQAGIDQLEQELLSQNKKHKDWECTEELMKTTKDGKA
LYMHCLPADITGVSCEEGEVEASVFDRYRVELYKEASYKPYVIAAMIFLSKVKNPQKTLTDLADK
ATPREVKDPNSSSVDKLAAALEHHHHHH
>2LBTA
GQAPPGPPASGPCADLQSAINAVTGGPIAFGNDGASLIPAAYEILNRVADKLKACPDARVTINGY
TDNTGSEGINIPLSAQRAKIVADYLVARGVAGDHIATVGLGSVNPIASNATPEGRAKNRRVEIVV
NHHHHHH
>3AXDA
MVSMKDFSGAELYTLEEYQYGKFEARMKMAAASGTVSSMFLYQNGSEIADGRPWVEVDIEVLGKS
PGSFQSNIITGKAGAQKTSEKHHAVSPAADQAFHTYGLEWTPNYVRWTVDGQEVRKTEGGQVSNL
TGTQGLRFNLWSSESAAWVGQFDESKLPLFQFINWVKVYKYTPGQGEGGSDFTLDWTDNFDTFDG
SRWGKGDYTFDGNRVDLTDKNIYSRDGMLILALTRKGQESFNGQVPRDDEPAPL
>2YEVB
MQRSFAALGLWGLSLAQEAHRVAITHPGGSFNQEVAFLFPWVYFFSFLIFLVVAGSLAYVTWKFR
ARPEDQEEPPQIHGNDRLEVVWTLIPLAIVFVLFGLTAKALIQVNRPIPGAMKVEVTGYQFWWDF
HYPELGLRNSNELVLPAGVPVELEITSKDVIHSFWVPGLAGKRDAIPGQTTRISFEPKEPGLYYG
FCAELCGASHARMLFRVVVLPKEEFDRFVEAAKASPAPVADERGQQVFQQNCAACHGVARSMPPA
VIGPELGLWGNRTSLGAGIVENTPENLKAWIRDPAGMKPGVKMPGFPQLSEEDLDALVRYLEGLK
VEGFDFGALPKF
>2YEVA
MAITAKPKAGVWAVLWDLLTTVDHKKIGLMYTATAFFAFALAGVFSLLIRTQLAVPNNQFLTGEQ
YNQILTLHGATMLFFFIIQAGLTGFGNFVVPLMLGARDVALPRVNAFSYWAFLGAIVLALMSYFF
PGGAPSVGWTFYYPFSAQSESGVDFYLAAILLLGFSSLLGNANFVATIYNLRAQGMSLWKMPIYV
WSVFAASVLNLFSLAGLTAATLLVLLERKIGLSWFNPAVGGDPVLFQQFFWFYSHPTVYVMLLPY
LGILAEVASTFARKPLFGYRQMVWAQMGIVVLGTMVWAHHMFTVGESTLFQIAFAFFTALIAVPT
GVKLFNIIGTLWGGKLQMKTPLYWVLGFIFNFLLGGITGVMLSMTPLDYQFHDSYFVVAHFHNVL
MAGSGFGAFAGLYYWWPKMTGRMYDERLGRLHFWLFLVGYLLTFLPQYALGYLGMPRRYYTYNAD
IAGWPELNLLSTIGAYILGLGGLVWIYTMWKSLRSGPKAPDNPWGGYTLEWLTASPPKAHNFDVK
LPTEFPSERPLYDWKKKGVELKPEDPAHIHLPNSSFWPFYSAATLFAFFVAVAALPVPNVWMWVF
LALFAYGLVRWALEDEYSHPVEHHTVTGKSNAWMGMAWFIVSEVGLFAILIAGYLYLRLSGAATP
PEERPALWLALLNTFLLVSSSFTVHFAHHDLRRGRFNPFRFGLLVTIILGVLFFLVQSWEFYQFY
HHSSWQENLWTAAFFTIVGLHGLHVVIGGFGLILAYLQALRGKITLHNHGTLEAASMYWHLVDAV

WLVIVTIFYVW
>3RBUA
RSGLNDIFEAQKIEWHEGSGSGSENLYFQGRSKSSNEATNITPKHNMKAFLDELKAENIKKFLYN
FTQIPHLAGTEQNFQLAKQIQSQWKEFGLDSVELAHYDVLLSYPNKTHPNYISIINEDGNEIFNT
SLFEPPPPGYENVSDIVPPFSAFSPQGMPEGDLVYVNYARTEDFFKLERDMKINCSGKIVIARYG
KVFRGNKVKNAQLAGAKGVILYSDPADYFAPGVKSYPDGWNLPGGGVQRGNILNLNGAGDPLTPG
YPANEYAYRRGIAEAVGLPSIPVHPIGYYDAQKLLEKMGGSAPPDSSWRGSLKVPYNVGPGFTGN
FSTQKVKMHIHSTNEVTRIYNVIGTLRGAVEPDRYVILGGHRDSWVFGGIDPQSGAAVVHEIVRS
FGTLKKEGWRPRRTILFASWDAEEFGLLGSTEWAEENSRLLQERGVAYINADSSIEGNYTLRVDC
TPLMYSLVHNLTKELKSPDEGFEGKSLYESWTKKSPSPEFSGMPRISKLGSGNDFEVFFQRLGIA
SGRARYTKNWETNKFSGYPLYHSVYETYELVEKFYDPMFKYHLTVAQVRGGMVFELANSIVLPFD
CRDYAVVLRKYADKIYSISMKHPQEMKTYSVSFDSLFSAVKNFTEIASKFSERLQDFDKSNPIVL
RMMNDQLMFLERAFIDPLGLPDRPFYRHVIYAPSSHNKYAGESFPGIYDALFDIESKVDPSKAWG
EVKRQIYVAAFTVQAAAETLSEVA
>2YDQA
GSVGPKTGEENQVLVPNLNPTPENLEVVGDGFKITSSINLVGEEEADENAVNALREFLTANNIEI
NSENDPNSTTLIIGEVDDDIPELDEALNGTTAENLKEEGYALVSNDGKIAIEGKDGDGTFYGVQT
FKQLVKESNIPEVNITDYPTVSARGIVEGFYGTPWTHQDRLDQIKFYGENKLNTYIYAPKDDPYH
REKWREPYPESEMQRMQELINASAENKVDFVFGISPGIDIRFDGDAGEEDFNHLITKAESLYDMG
VRSFAIYWDNIQDKSAAKHAQVLNRFNEEFVKAKGDVKPLITVPTEYDTGAMVSNGQPRAYTRIF
AETVDPSIEVMWTGPGVVTNEIPLSDAQLISGIYDRNMAVWWNYPVTDYFKGKLALGPMHGLDKG
LNQYVDFFTVNPMEHAELSKISIHTAADYSWNMDNYDYDKAWNRAIDMLYGDLAEDMKVFANHST
RMDNKTWAKSGREDAPELRAKMDELWNKLSSKEDASALIEELYGEFARMEEACNNLKANLPEVAL
EECSRQLDELITLAQGDKASLDMIVAQLNEDTEAYESAKEIAQNKLNTALSSFAVISEKVAQSFI
QEALS
>3R6TA
MGDTKEQRILRYVQQNAKPGDPQSVLEAIDTYCTQKEWAMNVGDAKGQIMDAVIREYSPSLVLEL
GAYCGYSAVRMARLLQPGARLLTMEINPDCAAITQQMLNFAGLQDKVTILNGASQDLIPQLKKKY
DVDTLDMVFLDHWKDRYLPDTLLLEKCGLLRKGTVLLADNVIVPGTPDFLAYVRGSSSFECTHYS
SYLEYMKVVDGLEKAIYQGPSSPDKS
>3R3JA
LHNYGYTSTKSVDNQIEELREKVVSKNKNEPEFLQAFEEVLSCLKPVFKKDNVYIGVLENIAEPE
RVIQFRVPWINDKGEHKMNRGFRVQYNSVLGPYKGGLRFHPAVNLSVIKFLGFEQIFKNSLTTLP
MGGGKGGSDFDPKGKSENEILKFCQSFMTNLFRYIGPNTDVPAGDIGVGGREIGYLFGQYKKLKN
SFEGVLTGKNIKWGGSNIRAEATGYGVVYFAENVLKDLNDNLENKKCLVSGSGNVAQYLVEKLIE
KGAIVLTMSDSNGYILEPNGFTKEQLNYIMDIKNNQRLRLKEYLKYSKTAKYFENQKPWNIPCDI
AFPCATQNEINENDADLFIQNKCKMIVEGANMPTHIKALHKLKQNNIILCPSKAANAGGVAVSGL
EMSQNSMRLQWTHQETDMKLQNIMKSIYEQCHNTSKIYLNESDLVAGANIAGFLKVADSFLEQGG
L
>3R18A
APSYPEYTREEVGRHRSPEERVWVTHGTDVFDVTDFVELHPGGPDKILLAAGGALEPFWALYAVH
GEPHVLELLQQYKVGELSPDEAPAAPDAQDPFAGDPPRHPGLRVNSQKPFNAEPPAELLAERFLT
PNELFFTRNHLPVPAVEPSSYRLRVDGPGGGTLSLSLAELRSRFPKHEVTATLQCAGNRRSEMSR
VRPVKGLPWDIGAISTARWGGARLRDVLLHAGFPEELQGEWHVCFEGLDADPGGAPYGASIPYGR

ALSPAADVLLAYEMNGTELPRDHGFPVRVVVPGVVGARSVKWLRRVAVSPDESPSHWQQNDNKGF
SPCVDWDTVDYRTAPAIQELPVQSAVTQPRPGAAVPPGELTVKGYAWSGGGREVVRVDVSLDGGR
TWKVARLMGDKAPPGRAWAWALWELTVPVEAGTELEIVCKAVDSSYNVQPDSVAPIWNLMGVLST
AWHRVRVSVQD

>3QVSA

MKVWLVGAYGIVSTTAMVGARAIERGIAPKIGLVSELPHFEGIEKYAPFSFEFGGHEIRLLSNAY
EAAKEHWELNRHFDREILEAVKSDLEGIVARKGTALNCGSGIKELGDIKTLEGEGLSLAEMVSRI
EEDIKSFADDETVVINVASTEPLPNYSEEYHGSLEGFERMIDEDRKEYASASMLYAYAALKLGLP
YANFTPSPGSAIPALKELAEKKGVPHAGNDGKTGETLVKTTLAPMFAYRNMEVVGWMSYNILGDY
DGKVLSARDNKESKVLSKDKVLEKMLGYSPYSITEIQYFPSLVDNKTAFDFVHFKGFLGKLMKFY
FIWDAIDAIVAAPLILDIARFLLFAKKKGVKGVVKEMAFFFKSPMDTNVINTHEQFVVLKEWYSN
LK

>3QSYA

MAWPKVQPEVNIGVVGHVDHGKTTLVQAITGIWTSKHSEELKRGMTIKLGYAETNIGVCESCKKP
EAYVTEPSCKSCGSDDEPKFLRRISFIDAPGHEVLMATMLSGAALMDGAILVVAANEPFPQPQTR
EHFVALGIIGVKNLIIVQNKVDVVSKEEALSQYRQIKQFTKGTWAENVPIIPVSALHKINIDSLI
EGIEEYIKTPYRDLSQKPVMLVIRSFDVNKPGTQFNELKGGVIGGSIIQGLFKVDQEIKVLPGLR
VEKQGKVSYEPIFTKISSIRFGDEEFKEAKPGGLVAIGTYLDPSLTKADNLLGSIITLADAEVPV
LWNIRIKYNLLERVVGAKEMLKVDPIRAKETLMLSVGSSTTLGIVTSVKKDEIEVELRRPVAVWS
NNIRTVISRQIAGRWRMIGWGLVEI

>3AUKA

MAASRANDAPIVLLHGFTGWGREEMFGFKYWGGVRGDIEQWLNDNGYRTYTLAVGPLSSNWDRAC
EAYAQLVGGTVDYGAAHAAKHGHARFGRTYLGLLPELKRGGRIHIIAHSQGGQTARMLVSLLENG
SQEEREYAKAHNVSLSPLFEGGHHFVLSVTTIATPHDGTTLVNMVDFTDRFFDLQKAVLEAAAVA
SNVPYTSQVYDFKLDQWGLRRQPGESFDHYFERLKRSPVWTSTDTARYDLSVSGAEKLNQWVQAS
PNTYYLSFATERTYRGALTGNYYPELGMNAFSAVVCAPFLGSYRNPTLGIDDRWLENDGIVNTVS
MNGPKRGSSDRIVPYDGALKKGVWNDMGTYNVDHLEIIGVDPNPSFDIRAFYLRLAEQLASLQP

>3QMXA

MRGSHHHHHHGSAVSAKIEIYTWSTCPFCMRALALLKRKGVEFQEYCIDGDNEAREAMAARANGK
RSLPQIFIDDQHIGGCDDIYALDGAGKLDPLLHS

>3QLIA

HHHHHHSSGLVPRGSMDIRALYDEKLTTPEEAVSSIASGSHLSMGMFAAEPPALLKALADRATRG
DIGDLRVYYFETAKIAGDTILRYELNNRIKPYSMFVTAVERALIRRGIEDGGRKVVNYVPSNFHQ
APRLLAEEIGIDTFMHTVSPMDCHGYFSLGVGNDYSSRIARSARRFIVEVNRYMPRVQGEAAAIH
ISEVDAIVENHVPLIEMPVRSAIPEYTSISHIIADLVPDGACLQMGVGALPNLVCGVLKDRNDLG
IHTEVLNPGLVDLIRRGVVTNQRKTLDRGRSVFTFAMGQQEMYEYLNDHPAIFSRPVDYVNDPHI
IAQNDNVVSINATLQIDLTGACNSEHMLGHQYSASGGQLDFVRGAYASKGGRSIIATPSTAAKGT
VSRIIPRIDGPVTTPRIDTHYIVTEFGAVNLKGLSSTERALRIIELAHPDFRDELTQAAKKMHLI

>2L97A

MEGLGFAIPANDAINIIEQLEKNGKVTRPALGIQMVNLSNVSTSDIRRLNIPSNVTSGVIVRSVQ
SNMPANGHLEKYDVITKVDDKEIASSTDLQSALYNHSIGDTIKITYYRNGKEETTSIKLNKLEHH
HHHH

>3QKGA

GPVPTPPDNIQVQENFNISRIYGKWYNLAIGSTSPWLKKIMDRMTVSTLVLGEGATEAEISMTST

RWRKGVCEETSGAYEKTDTDGKFLYHKSKWNITMESYVVHTNYDEYAIFLTKKFSRHHGPTITAK
LYGRAPQLRETLLQDFRVVAQGVGIPEDSIFTMADRGECVPGEQEPEPILIPRSAWSHPQFEK
>3QJJA
HHHHMRIEVKLLPLKDNPILPFNYNYEVYSQILEKVNSIEPTIAKLLSSPHGFWTFSRIIVRKRK
ILPDKGIEILSDDVSLYISSSNEDIIRAIAEAVEKSPEFKIGELSFLVGDIKAIKVKELGKENVF
STLSPIVVRTVKFEGNKLRHWDLYPHDELFMDRLRKVMILRYSEVMGETPKDRDFTIEVLKFKPT
RLMVGSSYIRGSLMVFRYAGSEEIARFGYENGFGEKTGLGFGMVKLIE
>2Y6YA
GAMTIGRAKVYATLSKIFYHLFYDEAIPKDCREIIEKFGEIDFNLRSVLVRELRGSVLIKDMPQS
LAEVYESVMKDFYERYGFQASELHADHIAVELAFMSKLVEREISLAQQMKEEELYKIRAAQHRFI
KAHLQPLVKNLPSAPLLNFVRDFVREDAKYLYSSLVGEKNEGADNN
>2L8LA
CLAEGTRIFDPVTGTTHRIEDVVDGRKPIHVVAAAKDGTLHARPVVSWFDQGTRDVIGLRIAGGA
ILWATPDHKVLTEYGWRAAGELRKGDRVAVRDVETGELRYSVIREVLPTRRARTFDLEVEELHTL
VAEGVVVHN
>3QDDA
HMPEETQTQDQPMEEEEVETFAFQAEIAQLMSLIINTFYSNKEIFLRELISNSSDALDKIRYESL
TDPSKLDSGKELHINLIPNKQDRTLTIVDTGIGMTKADLINNLGTIAKSGTKAFMEALQAGADIS
MIGQFGVGFYSAYLVAEKVTVITKHNDDEQYAWESSAGGSFTVRTDTGEPMGRGTKVILHLKEDQ
TEYLEERRIKEIVKKHSQFIGYPITLFVEKERDKEVSDDEAE
>3QBDA
HHHHHHMTDIGAPVTVQVAVDPPYPVVIGTGLLDELEDLLADRHKVAVVHQPGLAETAEEIRKRL
AGKGVDAHRIEIPDAEAGKDLPVVGFIWEVLGRIGIGRKDALVSLGGGAATDVAGFAAATWLRGV
SIVHLPTTLLGMVDAAVGGKTGINTDAGKNLVGAFHQPLAVLVDLATLQTLPRDEMICGMAEVVK
AGFIADPVILDLIEADPQAALDPAGDVLPELIRRAITVKAEVVAADEKESELREILNYGHTLGHA
IERRERYRWRHGAAVSVGLVFAAELARLAGRLDDATAQRHRTILSSLGLPVSYDPDALPQLLEIM
AGDKKTRAGVLRFVVLDGLAKPGRMVGPDPGLLVTAYAGVCAP
>3QB4A
MKRQGKRPSKNLKARCSRKALHVNFKDMGWDDWIIAPLEYEAFHCEGLCEFPLASHLEPTNHAVI
QTLMNSMDPESTPPTCCVPTRLSPISILFIDSANNVVYKQYEDMVVESCGCR
>2L8BA
TSGIHVLDELSVRALSRDIMKQNRVTVHPEKSVPRTAGYSDAVSVLAQDRPSLAIVSGQGGAAGQ
RERVAELVMMAREQGREVQIIAADRRSQMNMKQDERLSGELITGRRQLLEGMAFTPGSTVIVDQG
EKLSLKETLTLLDGAARHNVQVLITDSGQRTGTGSALMAMKDAGVNTYRWQGGEQRPAT

Supplementary S4. The seven physicochemical properties used in pseudo amino acid composition. See the text of the paper for more information.

ID[a]	NOZY710101	HOPT810101	TSAJ990101	CHOC760101	PRAM820101	GEIM800101	FINA770101
A	0.5	-0.5	89.3	115	0.305	1.29	1.08
R	0	3	190.3	225	0.227	1	1.05
N	0	0.2	122.4	160	0.322	0.81	0.85
D	0	3	114.4	150	0.335	1.1	0.85
C	0	-1	102.5	135	0.339	0.79	0.95
Q	0	0.2	146.9	180	0.306	1.07	0.95
E	0	3	138.8	190	0.282	1.49	1.15
G	0	0	63.8	75	0.352	0.63	0.55
H	0.5	-0.5	157.5	195	0.215	1.33	1
I	1.8	-1.8	163	175	0.278	1.05	1.05
L	1.8	-1.8	163.1	170	0.262	1.31	1.25
K	0	3	165.1	200	0.391	1.33	1.15
M	1.3	-1.3	165.8	185	0.28	1.54	1.15
F	2.5	-2.5	190.8	210	0.195	1.13	1.1
P	0	0	121.6	145	0.346	0.63	0.71
S	0	0.3	94.2	115	0.326	0.78	0.75
T	0.4	-0.4	119.6	140	0.251	0.77	0.75
W	3.4	-3.4	226.4	255	0.291	1.18	1.1
Y	2.3	-2.3	194.6	230	0.293	0.71	1.1
V	1.5	-1.5	138.2	155	0.291	0.81	0.95

[a] ID represents the ID of the physicochemical property in the AAindex database.
